# Identification of RAG-like transposons in protostomes suggests their ancient bilaterian origin

**DOI:** 10.1186/s13100-020-00214-y

**Published:** 2020-05-06

**Authors:** Eliza C. Martin, Célia Vicari, Louis Tsakou-Ngouafo, Pierre Pontarotti, Andrei J. Petrescu, David G. Schatz

**Affiliations:** 1 Department of Bioinformatics and Structural Biochemistry, Institute of Biochemistry of the Romanian Academy, Splaiul Independentei 296, 060031 Bucharest, Romania; 2 Evolutionary biology team, Aix Marseille Université IRD, APHM, MEPHI, IHU Méditerranée Infection, Marseille, France; 3 SNC5039 CNRS, 19-21 Boulevard Jean Moulin, 13005 Marseille, France; 4 Department of Immunobiology, Yale School of Medicine, 300 Cedar Street, Box 208011, New Haven, CT 06520-8011 USA

**Keywords:** Recombination activating genes, RAG, Evolution, Transposon, Adaptive immunity, Transposon molecular domestication

## Abstract

**Background:**

V(D) J recombination is essential for adaptive immunity in jawed vertebrates and is initiated by the RAG1-RAG2 endonuclease. The *RAG1* and *RAG2* genes are thought to have evolved from a *RAGL* (RAG-like) transposon containing convergently-oriented *RAG1-like* (*RAG1L*) and *RAG2-like (RAG2L)* genes. Elements resembling this presumptive evolutionary precursor have thus far only been detected convincingly in deuterostomes, leading to the model that the *RAGL* transposon first appeared in an early deuterostome.

**Results:**

We have identified numerous *RAGL* transposons in the genomes of protostomes, including oysters and mussels (phylum Mollusca) and a ribbon worm (phylum Nemertea), and in the genomes of several cnidarians. Phylogenetic analyses are consistent with vertical evolution of *RAGL* transposons within the Bilateria clade and with its presence in the bilaterian ancestor. Many of the *RAGL* transposons identified in protostomes are intact elements containing convergently oriented *RAG1L* and *RAG2L* genes flanked by terminal inverted repeats (TIRs) and target site duplications with striking similarities with the corresponding elements in deuterostomes. In addition, protostome genomes contain numerous intact *RAG1L-RAG2L* adjacent gene pairs that lack detectable flanking TIRs. Domains and critical active site and structural amino acids needed for endonuclease and transposase activity are present and conserved in many of the predicted RAG1L and RAG2L proteins encoded in protostome genomes.

**Conclusions:**

Active *RAGL* transposons were present in multiple protostome lineages and many were likely transmitted vertically during protostome evolution. It appears that *RAGL* transposons were broadly active during bilaterian evolution, undergoing multiple duplication and loss/fossilization events, with the *RAGL* genes that persist in present day protostomes perhaps constituting both active *RAGL* transposons and domesticated *RAGL* genes. Our findings raise the possibility that the *RAGL* transposon arose earlier in evolution than previously thought, either in an early bilaterian or prior to the divergence of bilaterians and non-bilaterians, and alter our understanding of the evolutionary history of this important group of transposons.

## Background

The powerful adaptive immune systems found in vertebrates rely on highly diverse antigen receptors encoded by genes that are non-functional in the germline and assembled by recombination during lymphocyte development [1, 2]. In jawed vertebrates, this assembly reaction is known as V(D) J recombination and operates on arrays of V, D, and J gene segments of immunoglobulin and T-cell receptor loci [3]. V(D) J recombination is initiated by the RAG1/RAG2 endonuclease (RAG), which cleaves DNA at a conserved recombination signal sequence (RSS) that flanks each gene segment and consists of heptamer and nonamer elements separated by a 12 or 23 bp spacer [4]. RAG1 is a multi-domain protein that makes extensive DNA contacts and cleaves DNA using an RNaseH-domain DDE active site similar to that found in many DNA transposases and retroviral integrases [5, 6]. RAG2 assists RAG1 in DNA binding and cleavage and is composed of a kelch-type 6-bladed beta propeller connected to a plant homeodomain (PHD) finger by an acidic “hinge” region [4–6]. High resolution structures of the RAG heterotetramer from mouse and zebrafish, either alone or in complex with the RSS, have provided extensive mechanistic insights into the DNA binding and cleavage steps of the recombination reaction [5–8].

**Fig. 1:**
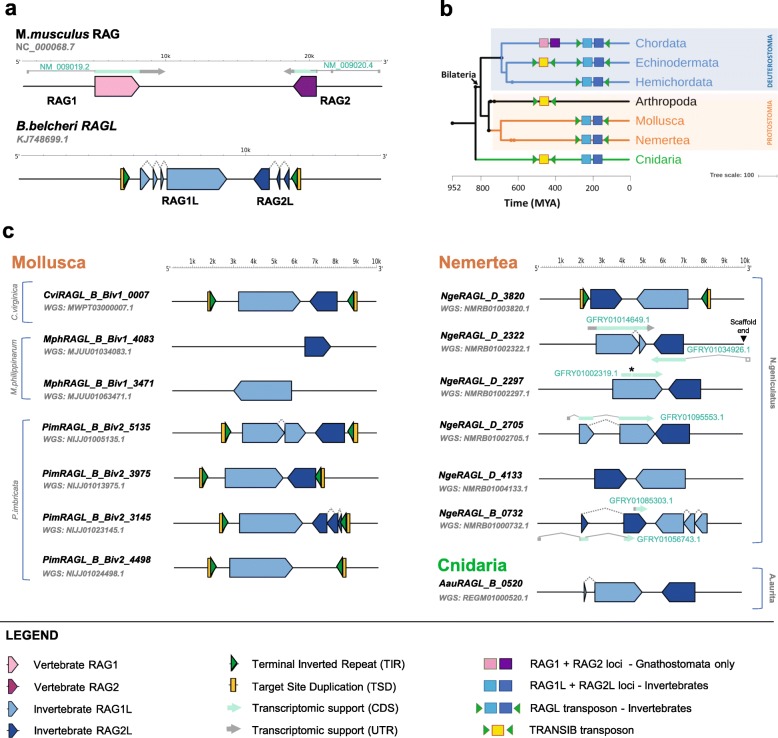
Genomic organization and phylogenetic tree of *RAG* and *RAGL* transposons. **a** Genomic organization of the mouse *RAG* locus and the amphioxus *RAGL* (*ProtoRAG*) transposon. The legend for panels (**a**), (**b**), and (**c**) is provided at the bottom of panel (c). **b** Tree depicting phyla within which *RAG* loci or *RAGL* or *Transib* transposons have been identified. Blue, orange and green shading indicate Deuterostomia, Protostomia and Cnidaria, respectively. Branches lacking evidence for RAG/RAGL sequences were omitted. With the exception of Cnidaria, all phyla with identified *RAGL* sequences contain at least one complete *RAGL* transposon with the configuration TSD-TIR5′-RAG1L-RAG2L-TIR3′-TSD. Prior to this study, potentially active *RAG1-RAG2* gene pairs and *RAGL* transposons had been reported only in deuterostomes [9], while outside this phylum, only as a single deteriorated *RAG1L-RAG2L* locus (in *C. gigas*) was previously reported [10]. **c** Genomic organization of the most complete *RAGL* copies detected in Mollusca, Nemertea, and Cnidaria. Predicted RAG1L/RAG2L coding regions, TIRs, and TSDs are depicted, using symbols explained in the legend at bottom. Supporting transcriptomic data are indicated along with corresponding TSA entry (Additional file 8: File S1 and Additional file 9: S2). Green and gray arrows indicate transcripts corresponding to coding and untranslated regions, respectively. Unmapped regions of transcripts are shown unfilled while the black star in GFRY01002319.1 indicates a frameshift caused by an 8 bp deletion

The RAG recombination machinery is present only in jawed vertebrates. For decades, this raised debate regarding its evolutionary origins. Early observations linked RAG to cut-and-paste DNA transposases, revealing that *RAG1* and *RAG2* invariably exist as a closely-juxtaposed, convergently transcribed gene pair (Fig. 1a), that RAG performs DNA cleavage by a nick-hairpin mechanism similar to that used by transposases, and that RAG possesses transposase activity in cell-free reactions [11]. Subsequently, sequence similarity was noted between RAG1’s essential core region and *Transib* transposases, and between RSSs and *Transib* terminal inverted repeats (TIRs) [12]. Functional [13, 14] and structural [15] studies have provided evidence that RAG1 and Transib transposase share a common ancestor [12, 16]. *Transib* elements, however, only contain a single gene encoding a protein similar to RAG1 and hence did not fully explain the origin of *RAG1-RAG2* gene pairs.

Since the discovery of *Transib*, multiple pairs of convergently oriented *RAG1-like* (*RAG1L*) and *RAG2-like* (*RAG2L*) genes have been discovered in invertebrate deuterostomes including echinoderms (purple sea urchin, bat star, and green sea urchin) [10, 17], cephalochordates (amphioxus) [18, 19], and hemichordates (acorn worm) [9]. TIRs flanking these gene pairs, when present, have sequence similarity to the RSS heptamer and the *Transib* TIR, and target site duplications (TSDs) are almost always 5 bp in length [9, 19], as is the case for transposition mediated by RAG and Transib [12, 13, 20, 21]. We refer to invertebrate RAG-like proteins as RAG1L and RAG2L, and transposons containing *RAG1L*-*RAG2L* gene pairs as *RAGL* transposons. Notably, the BbeRAG1L and BbeRAG2L proteins, encoded by the amphioxus *ProtoRAG* transposon of *Branchiostoma belcheri* (Fig. 1a), possess TIR-dependent endonuclease and transposase activities similar to those of RAG [19]. Furthermore, despite limited amino acid sequence identity, the BbeRAG1L/2 L and RAG1/2 complexes exhibit striking structural similarities [22]. Together, these findings provide compelling evidence for the hypothesis that the *RAG1*-*RAG2* gene pairs and RSSs of jawed vertebrates evolved from the transposase genes and TIRs, respectively, of a *RAGL* transposon [23].

Outside the Deuterostomia superphylum, there has been virtually no evidence for *RAG1L-RAG2L* gene pairs or for any gene encoding the combination of a kelch domain and PHD finger that uniquely identifies RAG2/RAG2L proteins [17]. *RAG1L* genes, in the context of *Transib* transposons, are widespread, having been found in protostomes, cnidarians, fungi, and echinoderms [12]. Nevertheless, there were hints that *RAG1-RAG2* gene pairs might not be limited to deuterostomes. Solitary *RAG1L* loci that resembled deuterostome *RAG1L* more than arthropod *Transib* transposase were described in cnidarians [12], and intriguingly, the deteriorated remnants of a *RAG1L-RAG2L* gene pair were reported in the protostome *Crassostrea gigas*, the pacific oyster [10]*.*

Here, we report the identification of numerous *RAG1L*-*RAG2L* gene pairs in the protostome clade, both in mollusks and in phylum Nemertea. Many of these elements exhibit all of the expected components of a RAGL transposon: convergent *RAG1L*-*RAG2L* genes flanked by TIRs that resemble the RSS heptamer, which in turn are flanked by 5 bp TSDs. Many of the identified *RAGL* coding sequences can be translated into full length RAG1L and RAG2L proteins in which key functional domains and amino acids are present, and sequence analysis reveals a new RAG-like transposon family in the nemertean *Notospermus geniculatus*. Phylogenetic analyses suggest that in protostomes, as in deuterostomes [9], the *RAGL* transposon evolved primarily in a vertical manner. We propose that the *RAGL* transposon arose very early in animal evolution, in a bilaterian if not earlier, and that subsequently, through duplication and vertical transmission, this transposon gave rise to several transposon families in protostomes and deuterostomes, with some extant elements retaining the potential to be active transposons.

## Results

### Distribution of *RAG1L*-*RAG2L* gene pairs across phyla

To search for additional *RAGL* transposons in disparate phyla, RAG1L and RAG2L amino acid sequences from invertebrate deuterostomes [9, 17, 19] were used to scan recently updated databases including Whole-Genome Shotgun Contigs (WGS), High Throughput Genomic Sequences (HTGS) and Transcriptomic Shotgun Assembly (TSA). Scans were performed on all metazoan invertebrate and jawless vertebrate projects available before February 2019. RAG1L proteins, possibly because of their direct role in catalysis, exhibit greater evolutionary conservation than RAG2L proteins, and detecting evolutionarily-distant RAG2L homologues using existing genome-wide blast techniques is challenging. For example, using tblastn [24, 25] and mouse or human RAG2 as query does not detect RAG2L from amphioxus WGS data (data not shown), and standard blast searches failed to identify RAG2L sequences in the purple sea urchin genome [17]. We overcame these difficulties using an iterative blast search approach (see Methods), which allowed the identification of new *RAG1L*-*RAG2L* gene pairs in protostomes, in both the Mollusca and Nemertea phyla, and in cnidarians (Fig. 1b, c). Notably, most *RAG2L* sequences identified were found to reside adjacent to *RAG1L* sequences, and in such gene pairs, the two genes invariably resided in transcriptionally convergent (tail-to-tail) configuration, an organization characteristic of *RAGL* transposons and jawed-vertebrate *RAG* loci (Fig. 1a).

In Mollusca, *RAG1L*-*RAG2L* gene pairs were found in class Bivalvia, subclass Pteriomorphia, in oysters (eastern oyster (*Crassostrea virginica*), pacific oyster (*Crassostrea gigas* - as previously reported by Kapitonov and Koonin [10]) and sydney rock oyster (*Saccostrea glomerata*)), mussels (Philippine horse mussel (*Modiolus philippinarum*) and the deep-sea mussel (*Bathymodiolus platifrons*)) and in Pterioida (akoya pearl oyster (*Pinctada imbricata*)) (Fig. 1c and Additional file 1: Figure S1). In Nemertea, numerous *RAG1L*-*RAG2L* gene pairs were detected in the ribbon worm *Notospermus geniculatus*, the only species with DNA or mRNA sequence data reported from this phyla. In *N. geniculatus*, several of the *RAGL* genes identified were supported by mRNA transcriptomic data (Fig. 1c and Additional file 1: Figure S1).

We also identified new *RAG1L*-*RAG2L* gene pairs in recently-sequenced invertebrate deuterostome genomes from the amphioxus *Branchiostoma lanceolatum* and the sea urchin *Hemicentrotus pulcherrimus*; these new elements are quite similar in sequence (> 60% protein sequence identity within the RAG1L core region) to those previously identified in amphioxus [19] and purple sea urchin [17], respectively (Additional file 1: Figure S1, and see below). Application of our search strategy to available sequence data from agnathans (jawless vertebrates) failed to identify *RAG1L* or *RAG2L* sequences.

Finally, WGS scans detected *RAG1L*-*RAG2L* gene pairs outside the Bilaterian clade in three species in the phylum Cnidaria: anthozoan stony coral *Porites rus*, mountainous star coral *Orbicella faveolata*, and moon jellyfish *Aurelia aurita* from the jellyfish Scyphozoan group (Fig. 1c and Additional file 1: Figure S1). Only a single *RAG1L*-*RAG2L* gene pair was detected in each species of coral, while in the moon jellyfish, one intact and two degenerate pairs were detected. Given the current low quality of the WGS data from these species, we did not attempt further analyses and interpret these findings cautiously. The intact *RAG1L*-*RAG2L* element from *A. aurita* is predicted to encode RAG1L and RAG2L proteins with striking conservation of functionally-important domains and sequence features (see below).

These findings indicate that *RAG1L*-*RAG2L* gene pairs are present not only in deuterostomes, but in protostomes and potentially non-bilaterians as well (Fig. 1 and Additional file 1: Figure S1).

### Identification of TIRs and TSDs flanking protostome *RAGL* transposons

TIRs define the boundaries of a transposable element and serve as sites that direct the binding and cleavage of transposase during transposition [26]. We searched for TIRs flanking *RAG1L*-*RAG2L* gene pairs using a homology variation-based approach that was based on the expectation that copies of a transposable element inserted in different sites in the genome should share a higher degree of homology within the elements than in their flanking regions (see Methods). We also required the presence of TSDs, short direct repeats flanking the 5′-TIR and 3′-TIR that arise as a consequence of staggered attack of the transposon ends on target DNA during transposition [26]. While many of the new *RAG1L*-*RAG2L* gene pairs identified failed to satisfy our stringent TIR criteria, multiple elements with TIRs and TSDs were found in three protostome species: eastern oyster (*C. virginica*), akoya pearl oyster (*P. imbricata*), and ribbon worm (*N. geniculatus*) (Fig. 1 and Additional file 1: Figure S1).

**Fig. 2:**
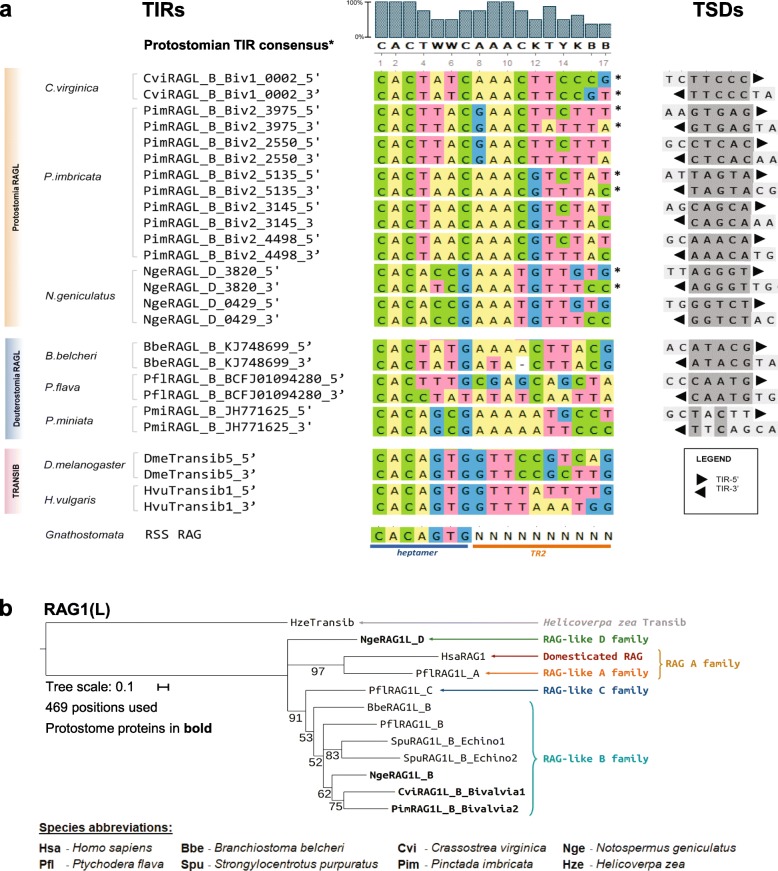
TIRs, TSDs, and phylogenetic tree (**a**) TIRs and TSDs of protostome and deuterostome *RAGL* transposons. Sequences of 5’-TIR and 3’-TIR (reverse complement) pairs are aligned with Transib TIRs and the consensus RSS heptamer. Protostome consensus TIR, shown at top, was generated using only nonredundant TIR sequences (asterisk). Most intact TIR sequence pairs detected in protostomes are flanked by 5 bp TSDs, displayed at right, with TIRs indicated black triangles and identities indicated with dark gray shading. **b** Phylogenetic trees of RAG1 and RAG1L. Phylogenetic trees were built using the Maximum Likelihood as described in Methods using MEGA X [27] and WAG with Freqs. (+) correction model [28]. The bootstrap numbers next to branches are the percentage of branches in which the associated proteins clustered together. Branches with a bootstrap value below 50% were collapsed together. Branch lengths represent the number of substitutions per site, with positions with gaps or missing data being ignored. The protein sequences used for these analyses were chosen as representative of the monophyletic group to which they belong. Bold indicates proteins from protostomes

While within a species TIRs contain extended regions of sequence similarity, between species the sequence similarity is largely confined to the outside termini of the TIRs, in a region of about 13 to 15 bp (Fig. 2a, Additional file 2: Figure S2a). The first 3 bp of protostome TIRs are 5′-CAC, matching the invariant and functionally critical first 3 bp of the RSS and the TIRs of *Transib* and deuterostome RAG transposons including *ProtoRAG*. The protostome TIR consensus, CACTWMCAAACKTYKBB, also includes a highly conserved AAA sequence at positions 8–10 that aligns with an A-rich region in deuterostome *RAGL* transposon TIRs [9, 19] (Fig. 2a). The TSDs found flanking protostome *RAGL* transposons are invariably 5 bp in length (Fig. 2a), similar to the predominant length of TSDs of *ProtoRAG*, *Transib*, and RAG [9, 10, 12, 19–21].

In addition to complete *RAGL* transposons with the structure TSD-5’TIR-*RAG1L*-*RAG2L*-3’TIR-TSD, numerous incomplete *RAGL* transposons were identified which lack one or both TIRs (and hence TSDs) and/or contain a solitary *RAG1L* or *RAG2L* gene (Fig. 1c and Additional file 1: Figure S1). We also detected a small number of 5′-3′ TIR pairs in which the intervening DNA lacked an intact *RAG1L* or *RAG2L* gene, structures which are known as non-autonomous transposable elements [29]. Such non-autonomous *RAGL* elements, often flanked by TSDs, were found in the oyster *P. imbricata* and in several deuterostomes (Additional file 2: Figure S2b). The occurrence of complete and incomplete *RAGL* transposon configurations indicates the existence of potentially active, fossilized, and possibly domesticated *RAGL* transposon copies.

Curiously, for the mussel *M. philippinarum*, WGS data contain a single *RAG1L*-*RAG2L* gene pair in which the genes are incomplete and contain stop codons, as well as unpaired *RAG1L* and *RAG2L* loci that have the potential to encode full length, intact protein products (Fig. 1c and Additional file 1: Figure S1).

### Phylogenetic analysis of protostome RAGL sequences suggests vertical transmission

**Fig. 3:**
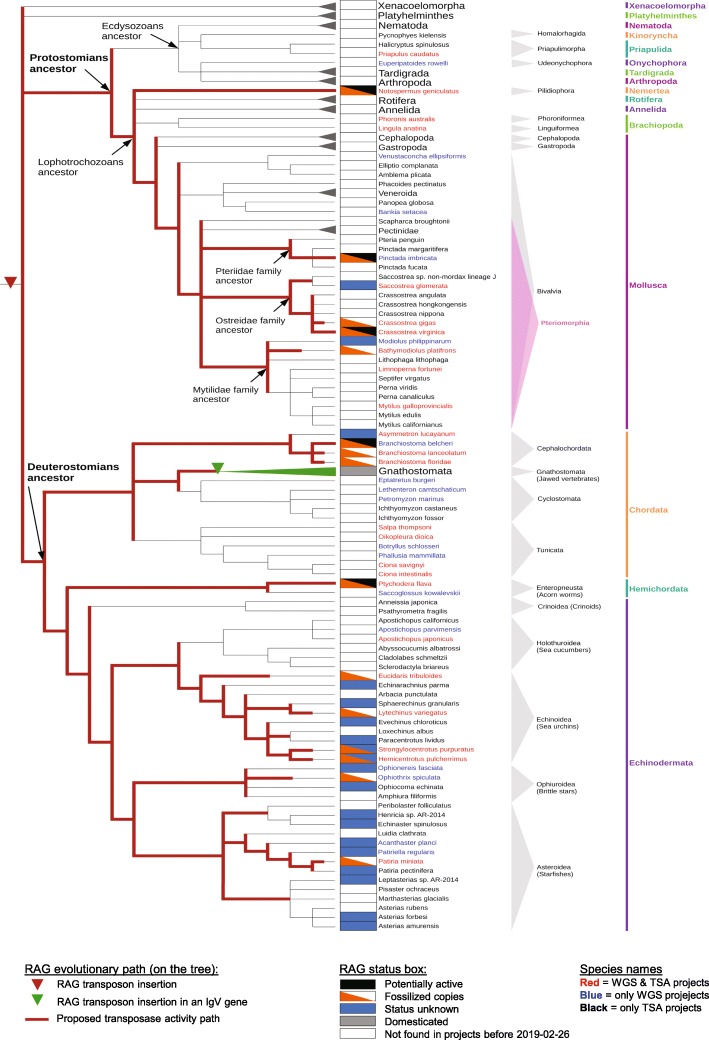
Bilaterian evolutionary tree and RAG evolutionary history. Tree was built with species for which there are at least one WGS and/or TSA project in the NCBI database. The species in which there are no RAG-like sequences found were regrouped into larger groups (Xenacoelomorpha, Platyhelminthes, Nematoda, Tardigrada, Arthropoda phyla), and the Gnathostome species were grouped as well, as RAG is domesticated in all of these species. Several species contain RAG1L-RAG2L copies with different statuses (e.g., one copy is potentially active and another is fossilized), and in such cases, the species are annotated as having more than one status within the status box. Red lines indicate branches in which *RAGL* transposon activity might have been present

Phylogenetic analysis of RAG1L sequences is more informative than that of RAG2L sequences because of RAG1L’s greater sequence conservation between lineages [11]. We constructed phylogenetic trees of RAG1L protein sequences using several different algorithms (Fig. 2b and Additional file 3: Figure S3, Additional file 4: Figure S4), which consistently yielded a tree structure similar to that of species phylogeny (Fig. 3). This finding suggests that the *RAGL* transposon evolved primarily by vertical transmission in deuterostomes and protostomes, with many duplication and loss events within clades, consistent with our previous analysis of deuterostome RAG1L sequences [9]. While our results are fully consistent with the hypothesis that the *RAGL* transposon was present in the bilaterian ancestor, we cannot rule out alternative scenarios that include horizontal gene transfer events. As expected, the phylogenetic analysis of RAG2L protein sequences was largely uninformative, with the only branch with > 50% bootstrap support being consistent with the observations deduced from the RAG1L proteins tree (Additional file 3: Figure S3e).

Our prior phylogenetic analyses indicate the existence of several families of RAGL proteins in deuterostomes [9]. The RAGL_A family, in the hemichordate *P. flava*, is the closest relative of vertebrate RAG. RAGL_C family members have also only been identified in *P. flava*. The RAGL_B family appears to be more widespread, with members identified in cephalochordates (including *ProtoRAG*), in several echinoderm lineages, and in *P. flava* [9]. Inclusion of protostome RAGL sequences in our analysis revealed four families: the RAG_A family composed of RAG in jawed vertebrates (*Gnathostomata*) and the RAGL_A family in *P. flava*; the RAGL_B family in all clades except jawed vertebrates; the RAGL_C family in *P. flava*; and the RAGL_D family in the nemertean *N. geniculatus* (Fig. 2b). Because of the relative lack of data, little can be concluded about families A, C, and D except that the RAG_A family was probably present in the deuterostomian ancestor. The most widespread and conserved family is RAGL_B, which is present in numerous copies in every species examined thus far except for jawed vertebrates. This distribution is consistent with a *RAG1L_B-RAG2L_B* gene pair being present in the bilaterian ancestor and its subsequent loss in jawed vertebrates.

RAG1L sequences from the class Pteriomorphia of bivalves, which includes *P. imbricata*, form a monophyletic group belonging to the RAGL_B family (Fig. 2b and Additional file 3: Figure S3c). Overall, the RAG1L phylogeny corresponds to the consensus species phylogeny (Fig. 3) except for some RAG1L sequences from the pearl oyster *P. imbricata*. The findings suggest that a duplication occurred in the Bivalvia clade, which led to the RAGL_B_Bivalvia1 and RAGL_B_Bivalvia2 subfamilies (Additional file 3: Figure S3c). Based on the RAG1L family tree, two distinct RAGL families were found in *N. geniculatus*, RAGL_B and RAGL_D (Figs. 1c, 2b). Interpretation of our findings (see Discussion) is limited by the availability of data in the databases (Fig. 3 and Additional file 6: Table S1). For example, we cannot be confident about the absence of *RAGL* sequences in some groups, as the number of sequenced species is not homogenous among clades.

### Protostomes encode intact and potentially functional RAG1L and RAG2L proteins

**Fig. 4:**
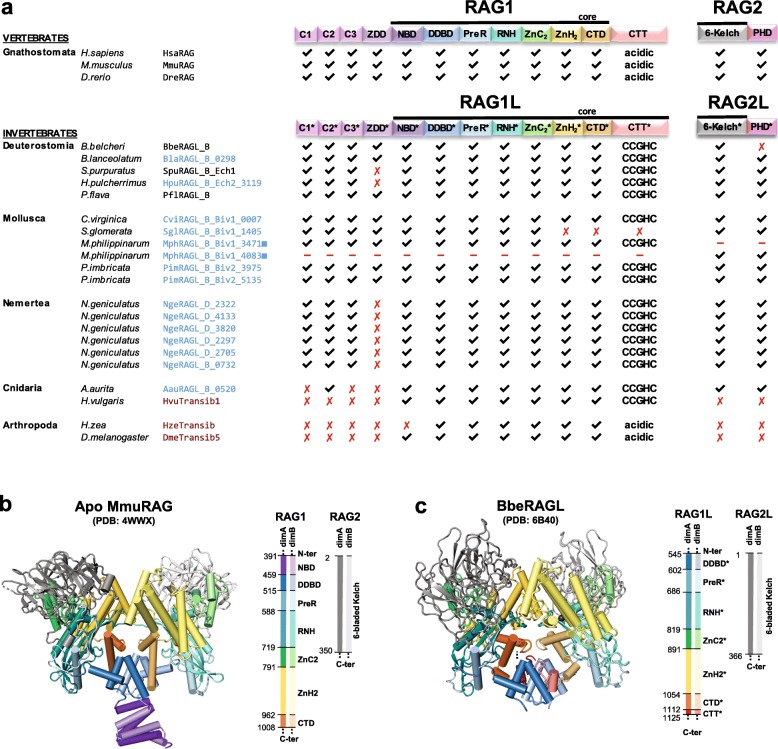
Protein domains found in protostome RAGL proteins. **a** Predicted domains present in the best preserved RAG1L and RAG2L proteins identified in this study (blue font) compared with RAG and RAGL protein sequences from deuterostomes (black font) and representative Transib transposase proteins (red font). All RAG1L and RAG2L proteins shown exist in tandem pairs with the exception of *M. philippinarum* RAG1L and RAG2L. Black lines, RAG1(L) and RAG2(L) core regions. Domains are not depicted to scale. RAG1L from *S. glomerata* is intact except for a premature stop codon in the ZnH2 domain. **b**, **c** Cartoon representations of the apo mouse RAG (i.e. in the absence of DNA) and *B. belcheri* RAGL (with DNA removed) tetramer structures, with domains colored as indicated and darker and lighter tones used to discriminate between subunits. Boundaries between domains are indicated with residue numbers. RAG1(L) domain abbreviations used: N-terminal zinc finger motifs, C1(*), C2(*), C3(*); ring zinc finger dimerization domain, ZDD(*); nonamer binding domain, NBD(*); dimerization and DNA binding domain, DDBD(*); pre-RNase H domain, PreR (*); catalytic RNase H domain, RNH(*); zinc finger ZnC2(*), zinc finger ZnH2(*),C-terminal domain, CTD(*); and C-terminal tail, CTT(*), that contains either the CCGHC motif of invertebrate RAG1L or acidic amino acids of vertebrate RAG1. RAG2(L) domain abbreviations used: 6-bladed kelch-type beta propeller domain, 6-Kelch(*); and plant homeodomain, PHD(*)

The *ProtoRAG*-encoded BbeRAG1L-BbeRAG2L proteins from the cephalochordate amphioxus together constitute an active endonuclease/transposase in vitro and in vivo and contain domains corresponding to all of the functionally essential “core” subdomains of jawed vertebrate RAG1 and RAG2 (Fig. 4) [19, 22]. These core subdomains are: the nonamer binding domain (NBD), the dimerization and DNA binding domain (DDBD), the Pre-RNase H domain (PreR), the catalytic RNase H domain (RNH), two zinc binding domains (ZnC2 and ZnH2) that together coordinate a zinc ion, and the C-terminal Domain (CTD) (Fig. 4). The RAG1 C-terminal tail (CTT) is not required for catalytic activity [4], while the corresponding domain from BbeRAG1L (CTT*; an asterisk specifies domains derived from RAG1L proteins) is critical for full activity and is part of the BbeRAG1L core region [19, 22]. Both RAG1 and BbeRAG1L contain zinc finger motifs C1/C1*, C2/C2*, and C3/C3*, as well as the ring zinc finger dimerization domain (ZDD/ZDD*) in their non-essential N-terminal regions (Fig. 4a). The RAG2 core domain is a 6-bladed β-propeller composed of 6 kelch repeats [5]; this region, but not the RAG2 C-terminal domain with its acidic hinge and plant homeodomain (PHD) finger, is found in BbeRAG2L (Fig. 4) [19].

**Fig. 5:**
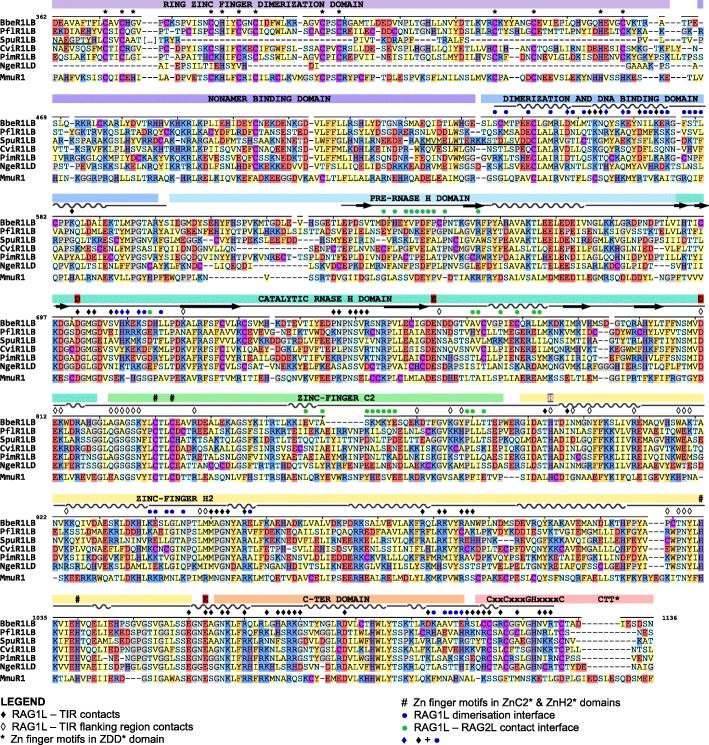
Alignment of RAG1L sequences from protostomes and deuterostomes. RAG1L sequences from 3 deuterostomes (the cephalochordate amphioxus (Bbe), echinoderm purple sea urchin (Spu), and hemichordate *P. flava* (Pfl)), 2 mollusk RAGL_B subfamilies (eastern oyster (Cvi) and pearly oyster (Pim)), and a nemertean *N. geniculatus* RAGL_D family representative (Nge) were aligned to mouse (Mmu) RAG1. Domains, sequence motifs, secondary structure assignment (helices - wavy lines; beta sheet - arrows, other - straight line), protein-protein and protein-DNA contact interactions (within 5 Å) displayed above the alignment derive from the BbeRAG1L cryo-EM structure (PDB: 6B40). Acidic catalytic residues, red; active site residue mouse H795, purple; zinc coordinating residues within ZDD (*) and ZnC2 and ZnH2 (#) are indicated above the sequences. Locations at which coding sequences span exon boundaries are underlined. Amino acid color code: hydrophobic aliphatic, yellow; hydrophobic aromatic, orange; positively charged, blue; negatively charged, red; neutral polar, light blue; glycine and prolines, grey; cysteine, purple; histidine, dark purple. Sequences displayed are BbeRAG1L_B (GenBank: KJ748699.1), PflRAG1L_B (TSA:GDGM01438088.1), SpuRAG1L_B_Ech1 (Uniprot: Q45ZT6), and CviRAG1L_B_Biv1_0007, PimRAG1L_B_Biv2_3145, and NgeRAG1L_D_2322 from this study (Additional file 7: Alignment S1a).

**Fig. 6:**
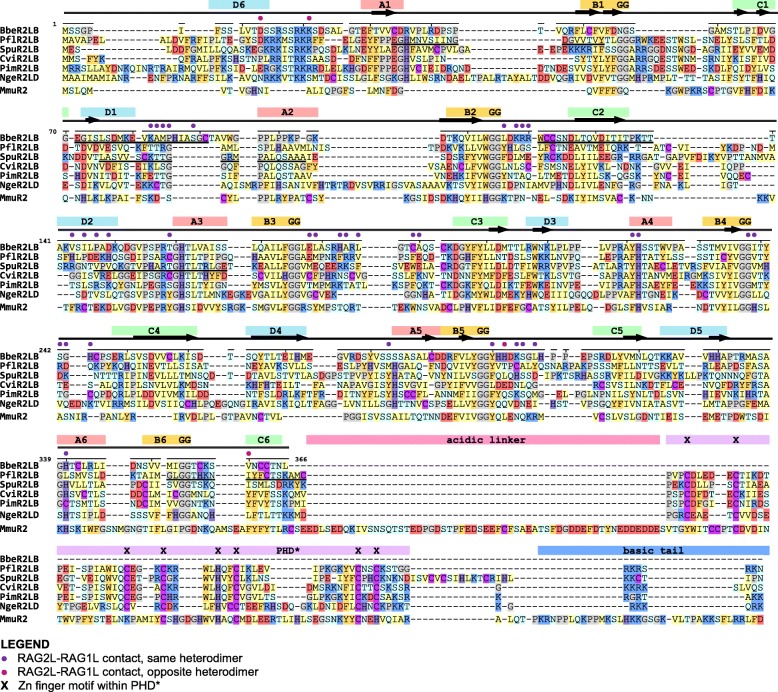
Alignment of RAG2L sequences from protostomes and deuterostomes. RAG2L sequences are aligned and displayed as in Fig. 5. Domains, beta sheet regions of each kelch-type blade, the GG motif, secondary structure (helixes - wavy lines; beta sheet - arrows, other - straight line), protein-protein interactions (5 Å threshold) displayed above the alignment derive from the BbeRAG2L cryo-EM structure (PDB: 6B40). Sequences displayed are: BbeRAG2L_B (GenBank: KJ748699.1), PflRAG2L_B (TSA:GDGM01438088.1), SpuRAG2L_B_Ech1 (Uniprot: Q45ZT5) and CviRAG2L_B_Biv1_0007, PimRAG2L_B_Biv2_5135, and NgeRAG1L_D_2322 from this study (Additional file 7: Alignment S1b). These RAG2L proteins are the transposon pairs of the RAG1L sequences displayed in Fig. 5 except that PimRAG2L_B_Biv2_5135 was used instead of PimRAG2L_B_Biv2_3145 due to merge uncertainties in the 3145 sequence; these two sequences are 98% identical on their counterpart RAG1L core. Species abbreviations as in Fig. 5

**Fig. 7:**
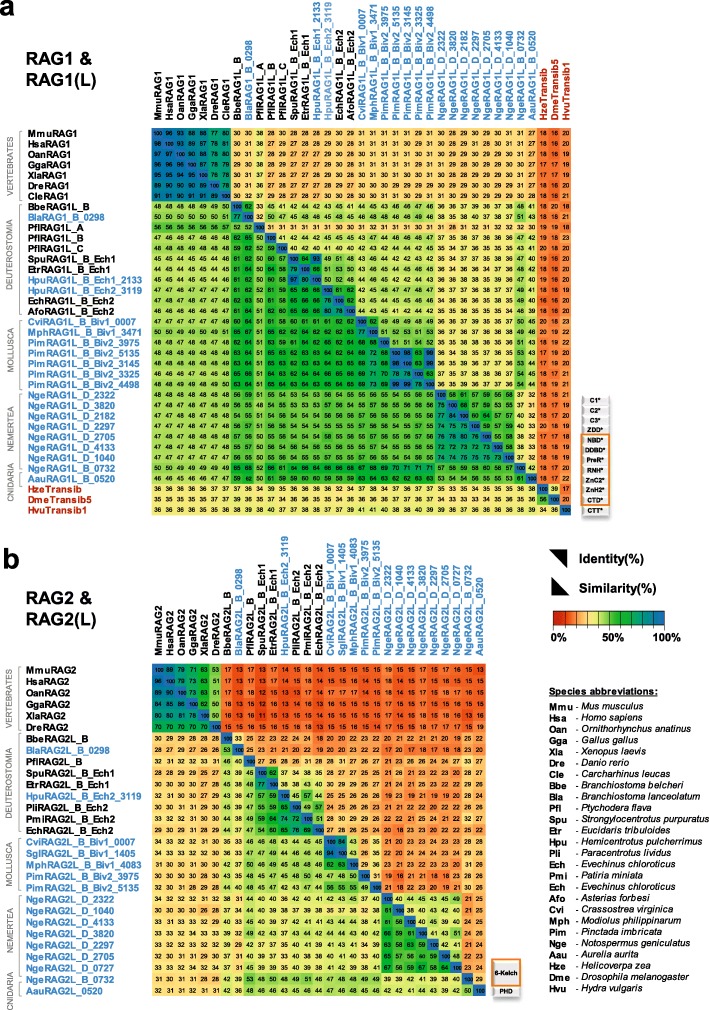
Identity and similarity matrices of the **a** RAG1L and **b** RAG2L core regions. Identity (upper right region) and similarity (lower left region) percentages were computed using the protein multiple sequence alignment shown in Additional file 7: Alignment S1a, b starting from the beginning of RAG1L NBD(*) until the end of CTD(*) and RAG2L kelch-type domain respectively, as described in Methods. Two sequences from Additional file 7: Alignment S1a (SglRAG1L_B_Biv1_1405 and NgeRAG1L_D_0727) were not included because they are incomplete in the core region interval

We analyzed the sequences of the predicted RAG1L and RAG2L proteins from protostomes to determine if these species, like amphioxus, had the potential to encode active RAGL complexes. Indeed, the phyla Mollusca and Nemertea each harbor multiple pairs of intact RAG1L-RAG2L open reading frames able to encode the kelch repeat domain of RAG2/BbeRAG2L and all of the essential core subdomains found in RAG1 and BbeRAG1L, including CTT* of BbeRAG1L (Fig. 4a; Figs. 5 and 6 show sequence alignments of selected RAG1L and RAG2L proteins, respectively, while alignments of all of the RAG1L and RAG2L protein sequences identified are shown in Additional file 7: Alignment S1a,b). In Mollusca, such pairs are observed in two species (*C. virginica* and *P. imbricata*), while the nemertean *N. geniculatus* harbors at least six different intact RAG1L-RAG2L protein pairs. Conservation of the core domains is also observed in the RAG1L-RAG2L pair identified in the cnidarian *A. aurita* (Figs. 4, 7 and Additional file 5: S5a,b).

Many protostome RAG1L proteins also exhibit substantial conservation with the RAG1 N-terminal non-core region. The three N-terminal zinc finger motifs are well conserved among most of the newly detected RAG1L homologs while ZDD* is readily identified in RAG1L sequences from Mollusca (Fig. 4a, Additional file 7: Alignment S1a). However, as in RAG1L proteins from echinoderms [9, 10, 17], ZDD* is absent from all RAG1L proteins from *N. geniculatus* and from RAG1L of *A. aurita* (Fig. 4a, Additional file 7: Alignment S1a). This suggests that this domain, which in RAG1 forms a tight dimer with E3 ubiquitin ligase activity [30], has undergone at least two independent loss events during RAG1L evolution.

With the exception of RAG2L from amphioxus [19], all invertebrate RAG2L proteins contain a C-terminal PHD finger (Figs. 4a, 6 and Additional file 5: S5b).

### Patterns of sequence conservation in RAG1L and RAG2L domains

We analyzed the patterns of conservation of key amino acid residues and domains of protostome RAG1L and RAG2L proteins to provide further insight into their potential functional properties and evolutionary relationships. Analysis of levels of sequence identity within the core region of RAG1L proteins reveals a broad correspondence with species phylogeny, with levels of identity highest between the RAG1L_B family sequences from protostomes and deuterostome invertebrates (Fig. 7a). And in general, core RAG1L sequences from invertebrates exhibit greater identity to one another than to core RAG1 sequences from jawed vertebrates, with the exception of the RAG1L_A family member from *P. flava*. Transib sequences diverge most strongly, exhibiting less than 22% sequence identity with the RAG1 and RAG1L proteins analyzed. Transib’s low sequence identity with RAG1/RAG1L and absence of elements corresponding to the RAG1 N-terminal non-core region allow one to distinguish between Transib and RAG1/RAG1L proteins. As was the case for RAG1/RAG1L, RAG2 and RAG2L core region sequences exhibit higher levels of identity within invertebrates than between jawed vertebrates and invertebrates (Fig. 7b). As expected, overall levels of sequence identity are lower for RAG2/RAG2L than for RAG1/RAG1L.

Numerous stretches of conservation are observed between protostome and deuterostome core RAG1L/RAG1 sequences beginning with the preR domain and extending to the CTD (Fig. 5 and Additional file 7: Alignment S1a). In addition, numerous functionally/structurally important residues are highly conserved in RAG1L sequences from protostomes. These include critical catalytic residues [4] and four zinc-coordinating residues from ZnC2 and ZnH2 that stabilize domain folding (Fig. 5, Additional file 5: Figure S5a and Additional file 7: Alignment S1a). The Cx_2_Cx_3_GHx_4_C motif that defines CTT* of BbeRAG1L is found in essentially all RAG1L sequences from protostomes and deuterostome invertebrates (Fig. 5 and Additional file 7: Alignment S1a). Numerous other potential zinc coordinating residues are also conserved in protostome RAG1L sequences including many in ZDD*, C1*, C2*, and C3* (Fig. 4a, and Additional file 7: Alignment S1a). Most protostome RAG1L proteins contain valine at the position equivalent to mouse RAG1 E649, and mutation of E649 to V or A increases the propensity of RAG to perform asynchronous, or “uncoupled” cleavage in vitro and in cells [22, 31] (Additional file 5: Figure S5a). Virtually all protostome RAG1L proteins contain a hydrophobic amino acid at the position equivalent to mouse RAG1 R848, a change that strongly activates the transposition activity of RAG in vitro and in cells [22] (Additional file 5: Figure S5a). Furthermore, invertebrate RAG2L proteins reported here and previously lack the acidic linker that exists between the RAG2 core and the PHD in jawed vertebrate RAG2 proteins (Fig. 6 and Additional file 7: Alignment S1b), and this acidic region has been shown to inhibit RAG-mediated transposition in cells [22]. These latter observations are consistent with the idea that protostome RAGL enzymes are, or evolved from, active transposases.

The core region of protostome RAG2L proteins preserves the structural features of a kelch-type domain including the GG motif that typifies the second β-strand of each kelch repeat (Fig. 6 and Additional file 7: Alignment S1b). The core region of protostome RAG2L is invariably followed by a cysteine-rich PHD, but the pattern of C and H zinc-coordinating residues found in protostome and deuterostome invertebrate PHDs (Cx_4-7_Cx_14-16_Cx_2-4_Cx_4_Hx_2_Cx_11-18_Cx_2_C) differs considerably from that seen in vertebrate RAG2 PHDs (CCx_2_Cx_22_Cx_5-6_Hx_2_Hx_2_Cx_19_Cx_2_H) (Additional file 5: Figure S5b and Additional file 7: Alignment S1b). The remarkable conservation of the C/H pattern in invertebrate RAG2L PHDs and its divergence from the pattern observed in its vertebrate counterparts suggest structural and functional differences that are as yet largely unexplored. The RAG2L PHD from the purple sea urchin *S. purpuratus* is capable of binding the tail of histone H3 when lysine 4 is methylated, although its preference for dimethylated lysine differs from the trimethylation preference of the mouse RAG2 PHD [32].

Together, these sequence analyses argue that many RAG1L-RAG2L protein pairs from protostomes have the potential to be active endonucleases with transposase activity.

### Analysis of protein-DNA and protein-protein interaction surfaces

**Fig. 8:**
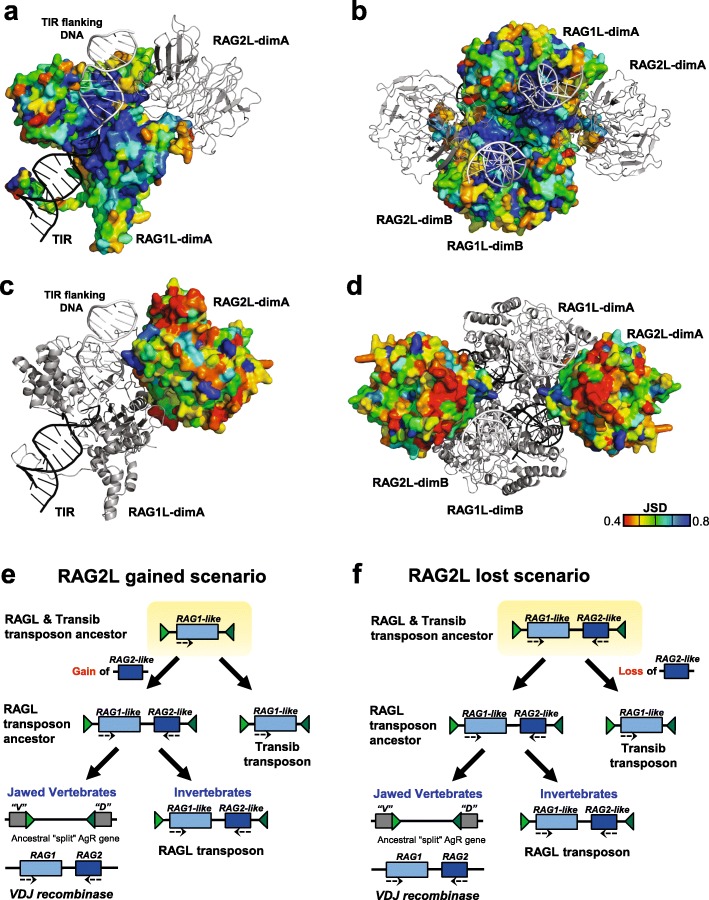
Sequence variability mapped onto BbeRAG1L/2 L cryo-EM structure (PDB: 6B40). **a**, **b**, **c**, **d** Surface representation of sequence variability of the protein-DNA and protein-protein contact interfaces of RAG1L (**a**, **b**) or RAG2L (**c**, **d**) in a lateral view of a RAG1L-RAG2L heterodimer (**a**, **c**) or a top view of the RAG1L-RAG2L tetramer (**b**, **d**). Jensen-Shannon divergence (JSD) conservation score is displayed using a rainbow color code as indicated with the scale bar, with blue and red indicating highly conserved and highly variable positions, respectively. RAG2L and RAG1L are shown in gray in (**a**, **b**) and (**c**, **d**), respectively, while TIR DNA and TIR flanking DNA are shown in black and white, respectively. **e**, **f** Alternative models for the evolutionary relationship between *Transib* and the *RAGL* transposon. In the current model [16] **(e)**, *Transib* was the ancestral element and the *RAGL* transposon was derived from *Transib* through acquisition of a *RAG2L* gene. In the alternative model **(f)**, the *RAGL* transposon was ancestral and the first *Transib* transposon arose from a *RAGL* transposon by loss of *RAG2L*

The availability of RAG1L-RAG2L sequences from protostomes provided an opportunity for a broad evolutionary examination of the conservation of interaction surfaces in the complexes formed by these proteins with each other and with DNA. This in silico analysis involved mapping representative RAG1L and RAG2L sequences from protostomes and deuterostomes (the six species whose sequences are shown in Figs. 5 and 6) onto the recently reported BbeRAG1L-BbeRAG2L three-dimensional structure, which closely resembles that of vertebrate RAG1-RAG2 [22]. This revealed strong conservation of the RAG1L DNA binding groove in regions that interact with both the TIR heptamer and the TIR heptamer-flanking region (Fig. 8a, b). This binding region contains numerous basic amino acid residues, creating a positively charged surface for DNA interaction [22]. This observation, combined with the high sequence conservation that surrounds the active site residues D701, E764, D811, H894 and E1063 in BbeRAG1L (Fig. 5, Additional file 5: Figure S5a and Additional file 7: Alignment S1a), suggests that protostome RAGL proteins have the potential to interact with and cleave DNA in a manner similar to that of RAG and BbeRAGL.

In contrast, the portions of RAG1L predicted to interact with RAG2L are less well conserved (Fig. 8a, b), and reciprocally, the portions of RAG2L predicted to interact with RAG1L also show high variability (Fig. 8c, d). Hence, the predicted RAG1L-RAG2L protein-protein interaction surfaces appear to have evolved more rapidly than the central DNA binding groove.

## Discussion

Over the last 15 years, multiple *RAG1L-RAG2L* gene pairs, some flanked by TIRs and TSDs, have been discovered in the genomes of invertebrate deuterostomes [9, 10, 17, 19], leading to the hypothesis that the *RAGL* transposon first arose in an early deuterostome [9, 16, 22]. Our finding of *RAG1L-RAG2L* gene pairs and potential *RAGL* transposons in protostomes calls this hypothesis into question. Phylogenetic analyses of RAG1L sequences suggest that *RAGL* transposon evolution has proceeded primarily through vertical transmission, supporting the possibility that the *RAGL* transposon arose in a bilaterian ancestor, if not earlier. Sequence and structural analyses argue that at least some of the RAG1L-RAG2L protein pairs from protostomes, and even one from a non-bilaterian, have the potential to be active endonucleases and transposases. Our findings have implications for our understanding of the evolutionary history of the RAGL transposon and for the process of transposon molecular domestication, an important contributor to genome and species evolution [33–35].

### Potentially active *RAGL* transposons in protostomes

Numerous findings described here support the conclusion that the *RAGL* transposon is present in the genomes of protostomes and has been active during protostome evolution: 1) Multiple elements with sequence similarity to known *RAG1*/*RAG1L* and *RAG2*/*RAG2L* genes of deuterostomes are present in protostome sequence databases; 2) protostome *RAG1L* and *RAG2L* genes often lie in close proximity in convergent transcriptional orientation; 3) many of these *RAG1L*-*RAG2L* gene pairs are flanked by TIRs and TSDs; 4) the TIRs resemble the sequence of the RSS heptamer and TIRs of deuterostome *RAGL* transposons; 5) protostome *RAGL* transposon TSDs are five bp in length; 6) predicted protostome RAG1L and RAG2L proteins often contain critical active site and structurally important amino acid residues and all of the domains required for activity by RAG or BbeRAGL; 7) conservation in protostome RAGL proteins extends to include non-essential but important regulatory domains at the N-terminus of RAG1/RAG1L and the C-terminus of RAG2/RAG2L of deuterostomes; 8) two different families (RAGL_B and RAGL_D), several RAGL_B subfamilies, and multiple degenerate copies of *RAG1L* and *RAG2L* sequences are detected in the genomes of protostomes, arguing for instances of transposon movement followed by inactivation.

The TIRs associated with protostome *RAGL* transposons exhibit two blocks of strong sequence conservation (Fig. 2a). The first is the perfectly conserved 5′-CAC sequence at the beginning of the heptamer, a region that is also rigidly conserved in jawed vertebrate RSSs. These residues are vital for cleavage [36], contributing to both a structural propensity for unwinding and sequence-specific protein-DNA contacts [7, 8]. The second conserved portion of the protostome TIR is an A-rich region from residues 8–10. Conservation of this sequence and the 13–15 bp length of the protostome TIR consensus are notable in light of the findings that only the first 16–17 bp of the *ProtoRAG* TIR are essential for cleavage and that CTT* of BbeRAG1L constitutes a novel DNA binding domain that interacts with TIR sequences that span this conserved AAA sequence [22]. CTT* from deuterostome RAG1L proteins contains a highly conserved Cx_2_Cx_3_GHx_4_C motif, and the same motif is strongly conserved in CTT* in RAG1L proteins from protostomes.

We therefore propose that protostome RAG1L-RAG2L protein complexes recognize a core TIR sequence of about 15 bp, with recognition of sequences flanking the heptamer mediated in part by CTT* [22]. If this is the case, then DNA recognition by invertebrate RAGL complexes is likely to follow distinct rules from those of their vertebrate RAG relatives: invertebrate RAGL will rely predominantly on the terminal ~ 15–17 bp of the TIR and CTT* and will be relatively insensitive to TIR asymmetry, whereas vertebrate RAG relies on a bipartite and asymmetric RSS, and in particular on a nonamer sequence separated from the heptamer by a spacer of 12 or 23 bp, with the nonamer recognized by a NBD on a flexible hinge [5–7]. Both protostome and deuterostome RAG1L proteins contain an NBD* domain with the potential to bind DNA—and in the case of BbeRAG1L, there is evidence that this region does indeed interact with more distal TIR sequences—but because of the presence of CTT*, the BbeRAG1L NBD* domain appears to serve a non-essential, auxiliary DNA binding function [22].

### Evolutionary history of the *RAGL* transposon in bilaterians

We have assembled existing information concerning the presence or absence of TIRs, TSDs, and open reading frames capable of encoding intact and potentially functional RAG1L and RAG2L proteins to predict the status of RAGL across protostome and deuterostome species, with status characterized as potentially active, fossilized, domesticated, not found, or unknown (Fig. 3). In some species, including several protostomes, both potentially active and fossilized *RAGL* elements are present. We emphasize that attribution of status is highly influenced by the availability (Additional file 6: Table S1) and quality of existing sequence data.

From this information and the assumption of vertical transmission, a working model for the evolutionary history of the *RAGL* transposon can be derived (red lines in Fig. 3). On this model, the *RAGL* transposon was present and active in the common bilaterian ancestor, remained active in both deuterostomes and protostomes, and might still be active in nemerteans, oysters and pearl oysters as well as some deuterostome invertebrate species [9]. We emphasize that while there is as yet no direct evidence for horizontal gene transfer of the *RAGL* transposon, such events cannot be ruled out, particularly between clades where the supporting sequence data remain sparse and the corresponding phylogenetic analyses provide lower levels of certainty. Our finding of several *RAG1L* and *RAG2L* sequences in cnidarians, including genes encoding a potentially active RAG1L-RAG2L protein pair, raises the possibility that the *RAGL* transposon arose prior to the origin of bilaterians, though again, horizontal gene transfer cannot be ruled out. Additional sequence data should allow testing of this idea and other predictions of the working model, providing a better understanding of the evolutionary events that led to the RAG recombinase.

Our findings suggest that four distinct RAGL protein families emerged during bilaterian evolution, two of which (RAGL_B and RAGL_D) are found in protostomes. The most widespread family, RAGL_B, is found in mollusks, nemerteans and many invertebrate deuterostomes, and is further divided into subfamilies, suggesting frequent duplication of the *RAGL* transposon in multiple clades. Many copies were subsequently lost while others were retained, some in fossilized form. This supports the idea that the *RAGL* transposon was broadly active during bilaterian evolution, giving rise to multiple families and subfamilies in some taxa.

The current model for *RAGL* transposon evolution posits that the first *RAGL* transposon was generated when a *Transib* transposon acquired a *RAG2-like* gene [16, 22]. This sequence of events, which places *Transib* prior to the *RAGL* transposon, is based on the widespread distribution of *Transib*, which is found even in fungi [12]. However, many uncertainties remain regarding early events in *RAGL*/*Transib* evolution, including uncertainties regarding the extent to which *Transib* was spread by horizontal transmission. These considerations, together with our data consistent with the hypothesis that the *RAGL* transposon arose earlier than previously thought, suggest that we consider the alternative possibility that the *RAGL* transposon arose prior to *Transib*. Hence, in addition to the current model that the *RAGL* transposon arose from *Transib* by gain of *RAG2L* (Fig. 8e) [16], we suggest that a different scenario also be considered in which *Transib* arose from a *RAGL* transposon by loss of *RAG2L* (Fig. 8f). Sequence data from additional eukaryotes will help test the plausibility of this second scenario.

### Transposon molecular domestication and the *RAGL* transposon

Transposon molecular domestication refers to a process in which transposon-derived sequences are co-opted by the host to perform new functions [37]. The repurposing of the components of a *RAGL* transposon for jawed vertebrate V(D) J recombination illustrates the large evolutionary impact this process can have. Our finding that several protostomes harbor potentially active *RAGL* transposons expands the range of species within which domestication of *RAGL* transposons could have occurred. A switch in biological function from transposase to sequence-specific endonuclease appears to be a common evolutionary event for multiple transposon families. In addition to the conversion of the RAGL transposase into the RAG recombinase, two such domestication events have been documented in the yeast *Kluyveromyces lactis*, where the Kat1 and α3 endonucleases, derived from hAT family and MULE family transposases, respectively, trigger mating type switching [38–40]. In ciliates, multiple endonucleases derived from PiggyBac family transposases mediate the programmed DNA rearrangements that remodel the somatic genome [41–43]. And PGBD5 and THAP9, factors derived from PiggyBac and *Drosophila* P-element family transposases, respectively, are active endonucleases expressed in humans whose domesticated function remains to be determined [44, 45]. Our findings identify multiple examples of intact *RAG1L* and *RAG2L* genes, either in pairs or in isolation, that appear to lack one or both flanking TIRs (Fig. 1c and Additional file 1: Figure S1a) and hence are unlikely to retain the ability to transpose. These *RAGL* genes, which are found in protostomes and in the moon jellyfish *A. aurita*, join the previously identified *RAG1L-RAG2L* gene pair from the purple sea urchin [17] as potentially domesticated derivatives of the *RAGL* transposon. Biochemical and structural analyses of the RAGL proteins encoded by these loci might shed light on their putative novel biological functions.

## Conclusion

The pivotal role played by a *RAGL* transposon in the evolution of the jawed vertebrate adaptive immune system represents a paradigmatic example of transposon molecular domestication. The findings reported here are consistent with a revised model for the evolutionary history of the *RAGL* transposon in which this transposon was present and active in the bilaterian ancestor. Our findings strongly suggest that *RAGL* transposons were transmitted vertically and in active form in multiple protostome lineages, as is also thought to be the case in deuterostomes. Our findings also argue that intact and potentially active *RAGL* transposons exist in the genomes of protostomes today, and similarly, that protostome genomes contain an assortment of intact *RAG1L-RAG2L* adjacent gene pairs that appear to lack flanking TIRs and are candidates for molecular domestication. Hence, the potential for *RAGL* transposons to have contributed novel gene functions during eukaryotic evolution is substantially broader than previously anticipated.

## Methods

### Genomic and Transcriptomic database screening

Detection of new RAG-like sequences was performed starting from a collection of previously reported RAG1-like and RAG2-like sequences from deuterostomian organisms *B. belcheri* [19], *S. purpuratus* [17], *P. flava* and *P. miniata* [9]. Queries were searched against all metazoan invertebrate and jawless vertebrate projects available before February 2019 from multiple databases such as Whole-Genome Shotgun Contigs (WGS), High Throughput Genomic Sequences (HTGS) and Transcriptomic Shotgun Assembly (TSA), using TBLASTN [24, 25], with a Blosum62, Blosum45 and PAM250 similarity matrices and a e-value threshold of 1e-08.

Regions containing RAG1L sequence signatures were further assessed for their potential to encode complete RAG1L proteins in the intron/exon context by analyzing the level and distribution of sequence similarity and the secondary structure profile match to RAG1L. The most complete newly detected RAG1L homologues were further used iteratively as queries in order to detect more divergent RAG1L sequences that were initially below the detection threshold or to allow for detection of RAG1L N-terminal non-core domain regions (which are more variable and hence harder to detect) in already detected RAG1L sequences.

In a second step, the WGS/TSA regions bordering *RAG1L* (within ~ 10 kb) were further explored to find potential *RAG2L* sequences. Here, special attention had to be drawn to sequence analysis. Because of the low levels of similarity among RAG2L proteins, retrieved hits often exhibited only partial coverage or were detected by only one or two RAG2L homologs. In such cases, a pool of translation product predictions was extracted and trimmed based on conservation of Kelch domain structural properties and its motif conservation.

The newly identified RAG1L and RAG2L sequences were further used independently as queries in an iterative manner to expand the detection threshold and find other more degraded copies within the same WGS projects or new hits in new WGS / TSA projects. In this step, the searches were performed in an unbiased fashion to identify not only RAG1L-RAG2L pairs, but also solitary loci. This resulted in only several solitary RAG1L or RAG2L that had the potential to be intact genes, sharing over 50% identity with their paralogues from a RAG1L-RAG2L tandem pair. These were also considered for further analysis as some of them might be translatable to protein even if they were isolated from the counterpart *RAGL* locus. Detailed information on the detected loci is presented in Additional file 8: File S1 and Additional file 9: File S2.

Prediction of protein translation products was performed starting from a FGENESH and FGENESH+ [46] and Augustus [47] pool of predicted products. The predicted protein sequences that have different exon composition were further trimmed based on mRNA sequence compatibility (when TSA entries were available) and based on the presence of highly conserved sequence motifs and subdomains that are found in full sized known deuterostomian homologs. Exon merging areas that are not covered in mRNA data are subjected to a higher degree of uncertainty and therefore are underlined where present.

### Phylogenetic analysis

Alignments were created with MEGA X [27] using ClustalW [48]. RAG trees (Fig. 2, Additional file 3: Figure S3) were built using MEGA X (Maximum Likelihood method, complete deletion, WAG with Freqs. (+) correction model [28], Gamma distribution with 5 categories, 1000 bootstrap replicates) and were confirmed with PhyML [49, 50] (Maximum Likelihood method) using the WAG substitution model, or AIC-based and BIC-based model selection (Additional File 4: Figure S4). The analyses were done first on RAG1L sequences because it is more conserved than RAG2L, and then the RAG2L sequences were analyzed to complement the results. To identify new RAGL sequence families, all RAG1L fragments longer than approx. 100 bp were aligned. Thereafter, we selected the areas of the alignment that were sufficiently conserved to identify the most significant positions (substitutions) with which to build the tree. This revealed the significant monophyletic groups with bootstrap values greater than 50, allowing us to define the representative sequences in each monophyletic group. The previously identified RAG/RAGL A, B and C families [9] represent different duplications of the *RAGL* transposon. Whenever a sequence did not significantly form a monophyletic group with a known family, we defined it as a new family, together with other sequences that form a monophyletic group with this sequence, as for example the *N. geniculatus* RAGL_D sequences. In contrast, if a sequence formed a monophyletic group significantly with an existing *RAGL* transposon family, we defined it as part of that family.

### Data availability & Bilateria tree

We established an overview of the species for whom sequence data was present in the WGS and TSA databases of NCBI on February 26, 2019 (Additional file 6: Table S1). While TSA projects are typically indicated as “TSA master”, some additional sequences marked as “Transcripts” are detected on a BLAST search against the TSA database (e.g., *Branchiostoma lanceolatum*), and some species that lack identifiable *RAGL* sequences might have been omitted inadvertently from Additional file 6: Table S1. From the available species in the NCBI database, a summary species tree was built using NCBI Taxonomy Common Tree [51, 52] and was edited with iTOL 4.3.2 [53]. The evolutionary tree timeline shown in Fig. 1b was obtained from TimeTree [54] and the tree editing was performed in online iTOL v4.3 [53].

### Detection of TIR and TSD sequences

The detection of TIRs is challenging due to their small size, the high incidence of short inverted repeats in DNA sequences, and the sequence drift expected to occur between the two TIRs after elements become domesticated. Moreover, previously reported TIR pairs in Deuterostomia [9] exhibit substantial variation between the 5′-TIR and 3′-TIR, with strong similarity only present in the vicinity of the terminal heptamer-like region. However, a significant drop in sequence identity is expected to occur at the tip of the TIR because the transposon cassette is expected to be similar in sequence to other transposon copies, while the flanking regions are expected to be divergent. We therefore designated sequences as TIRs only when they satisfied the following three stringent conditions: 1) a significant homology drop was detected on both sides of the *RAG1L*-*RAG2L* gene pair, 2) an inverted repeat, with greater than 50% identity between the two sequences, was present at the sites of the observed homology drops, and 3) a TSD was present flanking the terminal inverted repeats. The presence of TIR and TSD sequences was investigated in DNA regions where a significant drop in homology was detected using in a similar approach to that described previously [9]. Margins of 2–3 kb adjacent to *RAG1L* and *RAG2L* loci were compared between different cassette copies from the same or closely related organisms using the Needleman–Wunsch [55, 56] and Lalign [56, 57] pairwise alignment algorithms. In cases where a homology drop was detected at both ends flanking RAG1L and RAG2L loci, the homology boundaries were searched for inverted repeats. Furthermore, the presence of a 3–8 bp TSD adjacent to the identified inverted repeat was required and allowed us to discriminate between TIRs and a premature end of the transposon cassettes. The TIR pairs flanked by TSDs were then used to identify transposon margins containing solitary, unpaired TIRs using blastn [58, 59] and 150 bp TIR containing margins as queries of each WGS project data. Detailed information about the detected TIRs and TSDs are provided in Additional file 8: File S1 and Additional file 9: File S2.

### Sequence analysis and variability

Domains within each of the identified RAG1L and RAG2L pair were delineated using InterproScan [60], while RaptorX-property [61] was used to predict the local secondary structure. Multiple sequence alignments were performed using T-coffee in psicoffee mode using a Uniref50 database for homology searching [62, 63]. Due to the low homology between RAG2L sequences, the Kelch-type domains and the PHD domains were first aligned separately and subsequently merged into a single alignment.

Identity percentage matrices were computed excluding gaps, using Unipro Ugene v1.22.0 [64], as the ratio of identical amino acid pair counts over the length of the smallest sequences from the compared pair. Similarity percentages presented below the diagonal in the same figures were computed using an in-house implementation of the Ugene algorithm, but using for counts the matrix of all BLOSUM62 positive substitutions, as used in the blast-like methods. Ugene was also used to generate the graphics included in figures containing protein and nucleotide alignments, while AnnotationSketch [65] was used to generate genomic organization figures.

Given redundancies and the unbalanced distribution of RAGLs among the evolutionary branches, variability was computed only over a nonredundant set of 6 RAGL pairs sharing less than 50% protein sequence identity within the core RAG1L region. This set proves also to be representative for the evolutionary clades and consists of three deuterostome sequences: cephalochordata group (amphioxus), echinodermata group (sea urchin), hemichordata clade (*P. flava*) and three protostome sequences: one from each mollusk RAGL_B subfamily, and one nemertean *N. geniculatus* RAGL_D family representative. *P. flava* RAGL_A and RAGL_C were discarded given the low confidence protein prediction for their RAG2L counterparts.

Conservation Jensen-Shannon divergence (JSD) was used to compute similarity scores for each position in the alignment of the above six sequences and used to map the sequence variability of RAGL. JSD was calculated using the implementation of [66] based on Blosum62 background probabilities with a gap penalty of 1 and window = 0.

Relative entropy logo was generated using WebLogo [67] and PyMOL Molecular Graphics System, Version 2.2.3 Schrödinger, LLC was used to represent all protein structures.

## Supplementary information

**Additional file 1: Figure S1.** Genomic organization of *RAGL* and *RAGL* transposons identified in this study. **(a)** Genomic organization of *RAGL* copies identified in deuterostomes, mollusks, nemerteans and cnidarians. Only the most relevant RAG1L/RAG2L pairs are shown. The legend for panels (a) and (b) is provided at the bottom of panel (b). Loci that are likely to be pseudogenized are indicated with a white box. Supporting transcriptomic data are indicated along with corresponding TSA entry. Green and gray arrows indicate transcripts corresponding to coding and untranslated regions, respectively. Unmapped regions of transcripts are shown as unfilled rectangles outside of the gene track. **(b)** Genomic organization of incomplete and potentially pseudogenized RAG1L/RAG2L loci in cnidarians. Most of these regions either have stop codons or low sequence coverage and are therefore shown with vertical stripes. The *P. damisconis* locus is incomplete as it is located at the margin of the scaffold and might encode a complete protein. Assembly gaps near the detected loci are shown as black boxes.

**Additional file 2: Figure S2. (a)** Alignments of four groups of protostome RAGL transposon TIRs. Sequences of 5′-TIR and 3′-TIR (reverse complement) pairs are aligned from four groups: *C. virginica* (I), *P. imbricata* (II and III) and nemertean *N. geniculatus* (IV). Despite both being mollusks, the *C. virginica* and *P. imbricata* TIR sequences are very dissimilar both in sequence and length. None of the 4 groups contain an RSS nonamer-like region, however, the *C. virginica* 5′ and 3′ TIRs exhibit a length difference of 11 bp, reminiscent of the 11 bp difference in lengths of the spacers in the 12RSS and 23RSS that underlies the 12/23 rule of V(D) J recombination [3, 6]. Similarity color code: dark grey, fully conserved; light grey, partially conserved; white, no similarity. **(b)** Potential RAG-derived non-autonomous transposable elements in protostomes and deuterostomes. Summary of the potential RAG-derived non-autonomous elements identified in WGS database in organisms where the *RAGL* transposon was detected. The configuration of these elements, as well as TSD sequences (if present) are indicated. The e-values shown derive from blastn searches of the WGS project of each organism using previously detected *RAGL* TIR-containing margins (200 bp) as queries.

**Additional file 3: Figure S3.** Additional phylogenetic analyses. **(a-d)** Detailed phylogenetic trees of RAG1 and RAG1L protein sequences including several from: **(a)** Cephalochordata - indicated with blue shading., **(b)** Echinodermata - blue shading, **(c)** Mollusca - orange shading and **(d)** including one from cnidaria - green shading **(e)** RAG2/RAG2L phylogenetic trees. Trees were built using Maximum Likelihood and WAG substitution model as implemented in MEGA X [27] and are displayed as in Fig. 2b except that branches with bootstrap numbers below 50% were not collapsed together. Trees were built from a variable number of significant positions of their alignment: (a) 289, (b) 398, (c) 354, and (d) 469 respectively .

**Additional file 4: Figure S4.** Complementary RAG1L phylogenetic analyses using PhyML implementation [49, 50]. Trees are displayed as in Fig. 2b except that branches with bootstrap numbers below 50% were not collapsed together. Different substitution models were used as follows: **(a)** LG + G + I + F model selected via AIC minimization **(b)** LG + G + I model selected via BIC minimization **(c)** WAG substitution model.

**Additional file 5: Figure S5.** (a) Conservation of functional relevant amino acids in RAG1 and RAG1L. The alignments depict the extensive conservation of some of the most important and well characterized amino acids in RAG1/RAG1L proteins (numbers given for BbeRAG1L): from left to right, catalytic carboxylates (D701, E764, D818, and E1063), residues implicated in controlling coupled versus uncoupled cleavage (A1064 and V751), a residue that facilitates transposition (M949), a histidine component of the active site (H894), and zinc-coordinating residues (C830, C833, H1035 and H1040). Residue E649 in mouse RAG1 contributes to synchronous, or “coupled”, cleavage by RAG at two RSSs, in part through its ability to form a hydrogen bond with S963 [22, 31]. The BbeRAG1L/2 L complex (BbeRAGL) exhibits less propensity for coupled cleavage in part because E649 has been replaced with V751 [22]. Valine is highly conserved at this position in RAG1L proteins from protostomes, suggesting that DNA cleavage by these proteins, if it occurs, would more likely resemble the uncoupled cleavage activity of BbeRAGL. Mutation of the charged residue R848 in mouse RAG1 to the hydrophobic residue methionine, as is found in BbeRAG1L, strongly activates the transposition activity of RAG [22]. Virtually all invertebrate RAG1L proteins, including those from protostomes, have a hydrophobic amino acid, most often methionine, at this position. (b) RAG2/RAG2L PHD domain alignment. The pattern of conserved cysteine and histidine residues (marked with X) are different between invertebrate RAG2L (top) and jawed vertebrate RAG2 (bottom). Variability logo (top) shows relative entropy (bits) calculated on the invertebrate alignment group. Amino acid color code as in Fig. 5.

**Additional file 6: Table S1.** Genomic and transcriptomic data availability for bilaterian invertebrates. List of the bilaterian species for which there are Transcriptome Shotgun Assembly (TSA) and/or Whole Genome Shotgun (WGS) projects in the NCBI database as of February 26, 2019. Gnathostomata species are not listed. The taxonomic identifier is given in the NCBI taxid column and corresponds to that used in NCBI databases. The number of projects available is indicated. In the Number of TSA projects column, transcriptomic projects that were not marked as “TSA project” are indicated in parentheses.

**Additional file 7. **Alignment S1 Multiple sequence alignment of **(a)** RAG1/RAG1L and **(b)** RAG2/RAG2L predicted proteins. Domains, sequence motifs, secondary structure assignment, protein-protein and protein-DNA contact interactions (within 5 Å) displayed above the alignment derive from the BbeRAGL cryo-EM structure (PDB: 6B40). Additionally, for RAG1/RAG1L **(a)**, acidic catalytic residues, red; active site residue mouse H795, purple; zinc coordinating residues within ZDD (*) and ZnC2 and ZnH2 (#) are indicated above the sequences, while for RAG2/RAG2L **(b)** the beta sheet regions of each kelch-type blade and the GG motifs are shown above the alignment. Locations at which coding sequences span exon boundaries are underlined. Sequence descriptions including references to genomic, transcriptomic or protein databases are shown at the end of the alignment, along with a legend of the symbols used.

**Additional file 8: File S1.** Detailed presentation of the sequence information about the new *RAGL* loci identified in this study: detection relevant data (e-values and query-target alignments from TBLASTN), TIR and TSD detection information, predicted protein sequences, and additional relevant observations regarding some of the sequences.

**Additional file 9: File S2.** Identified *RAGL* loci mapped onto nucleotide sequence.

## Data Availability

The datasets analyzed during the current study are available in the GenBank repository, specifically in Whole-Genome Shotgun Contigs (WGS) and Transcriptomic Shotgun Assembly (TSA). Additional file 8: File S1 contains a detailed presentation of the sequence information about the new *RAGL* loci identified in this study: detection relevant data (e-values and the exact identified segments alignments from TBLASTN), TIR/TSD detection information, predicted protein sequences and additional relevant observations regarding some of the sequences, while Additional file 9: File S2 contains nucleotide sequence data.

## Abbreviations

RAG: Recombination Activating Gene
RAGL: RAG-like
TIR: Terminal inverted repeat
TSD: Target site duplication
RSS: recombination signal sequence
ZDD: ring zinc finger dimerization domain
NBD: nonamer binding domain
DDBD: dimerization and DNA binding domain
PreR: pre-RNase H domain
RNH: catalytic RNase H domain
ZnC2: zinc finger domain that contributes two cysteine residues
ZnH2: zinc finger domain that contributes two histidine residues
CTD: C-terminal domain
CTT: C-terminal tail
PHD: plant homeodomain
