# Supplementary material for: Identification of RAG-like transposons in protostomes suggests their ancient bilaterian origin
Source: Mob DNA. 2020 May 6;11:17. doi: 10.1186/s13100-020-00214-y (PMC7204232; doi:10.1186/s13100-020-00214-y)
Supplement: Supplementary file 1 — Additional file 1: Figure S1. Genomic organization of RAGL and RAGL transposons identified in this study. (a) Genomic organization of RAGL copies identified in deuterostomes, mollusks, nemerteans and cnidarians. Only the most relevant RAG1L/RAG2L pairs are shown. The legend for panels (a) and (b) is provided at the bottom of panel (b). Loci that are likely to be pseudogenized are indicated with a white box. Supporting transcriptomic data are indicated along with corresponding TSA entry. Green and gray arrows indicate transcripts corresponding to coding and untranslated regions, respectively. Unmapped regions of transcripts are shown as unfilled rectangles outside of the gene track. (b) Genomic organization of incomplete and potentially pseudogenized RAG1L/RAG2L loci in cnidarians. Most of these regions either have stop codons or low sequence coverage and are therefore shown with vertical stripes. The P. damisconis locus is incomplete as it is located at the margin of the scaffold and might encode a complete protein. Assembly gaps near the detected loci are shown as black boxes. [file 13100_2020_214_MOESM1_ESM.pdf]

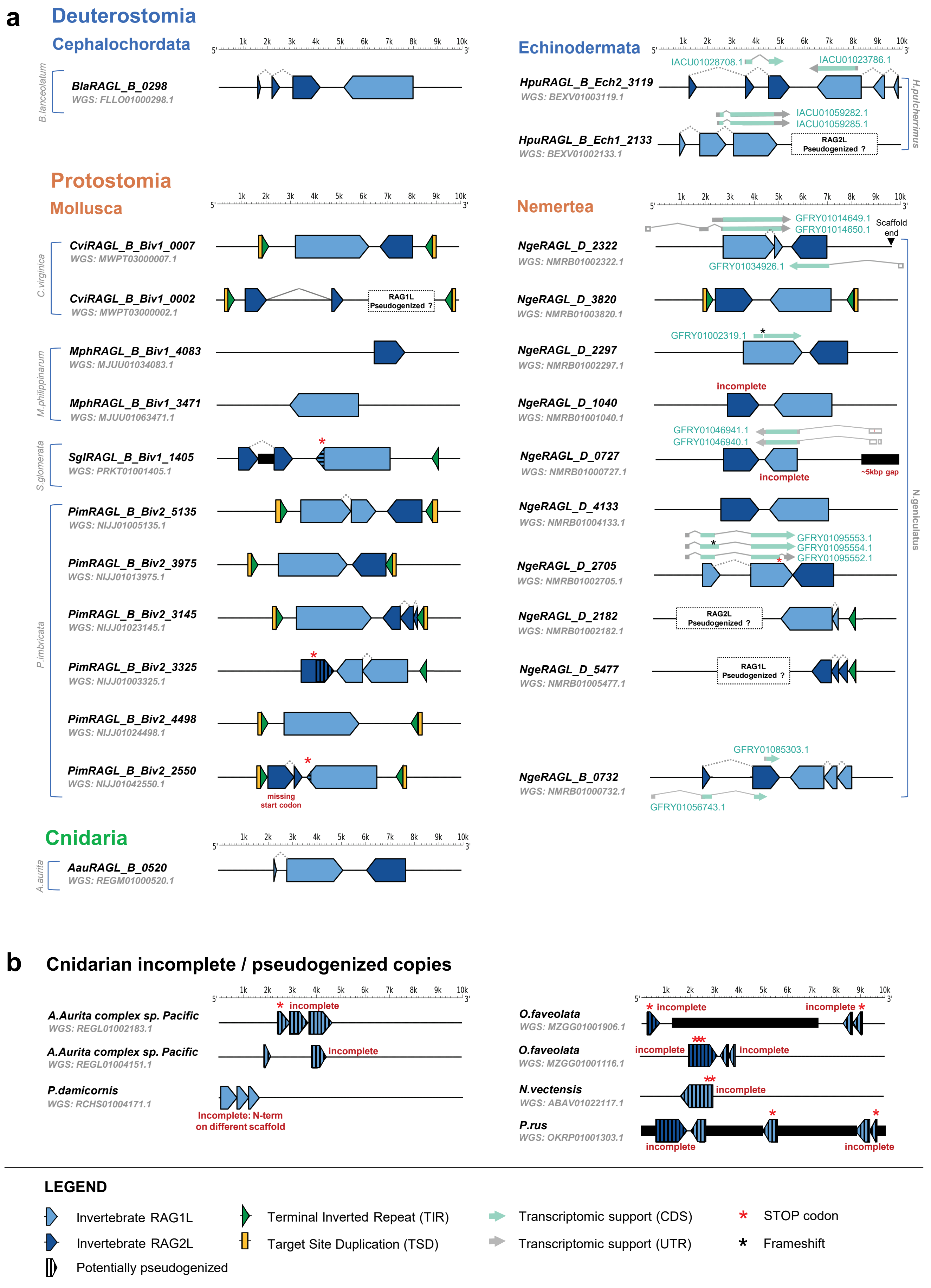

a
Deuterostomia
Cephalochordata
B.lanceolatum
BlaRAGL_B_0298
WGS: FLLO01000298.1
Echinodermata
IACU01028708.1
IACU01023786.1
HpuRAGL_B_Ech2_3119
WGS: BEXV01003119.1
IACU01059282.1
IACU01059285.1
HpuRAGL_B_Ech1_2133
WGS: BEXV01002133.1
RAG2L Pseudogenized ?
H.pulcherrimus
Protostomia
Mollusca
C.virginica
CviRAGL_B_Biv1_0007
WGS: MWPT03000007.1
CviRAGL_B_Biv1_0002
WGS: MWPT03000002.1
RAG1L Pseudogenized ?
M.philippinarum
MphRAGL_B_Biv1_4083
WGS: MJUU01034083.1
MphRAGL_B_Biv1_3471
WGS: MJUU01063471.1
S.glomerata
SglRAGL_B_Biv1_1405
WGS: PRKT01001405.1
P.imbricata
PimRAGL_B_Biv2_5135
WGS: NIJJ01005135.1
PimRAGL_B_Biv2_3975
WGS: NIJJ01013975.1
PimRAGL_B_Biv2_3145
WGS: NIJJ01023145.1
PimRAGL_B_Biv2_3325
WGS: NIJJ01003325.1
PimRAGL_B_Biv2_4498
WGS: NIJJ01024498.1
PimRAGL_B_Biv2_2550
WGS: NIJJ01042550.1
missing start codon
Nemertea
GFRY01014649.1
GFRY01014650.1
Scaffold end
NgeRAGL_D_2322
WGS: NMRB01002322.1
GFRY01034926.1
NgeRAGL_D_3820
WGS: NMRB01003820.1
GFRY01002319.1
NgeRAGL_D_2297
WGS: NMRB01002297.1
incomplete
NgeRAGL_D_1040
WGS: NMRB01001040.1
GFRY01046941.1
GFRY01046940.1
NgeRAGL_D_0727
WGS: NMRB01000727.1
incomplete
~5kbp gap
NgeRAGL_D_4133
WGS: NMRB01004133.1
GFRY01095553.1
GFRY01095554.1
GFRY01095552.1
NgeRAGL_D_2705
WGS: NMRB01002705.1
NgeRAGL_D_2182
WGS: NMRB01002182.1
RAG2L Pseudogenized ?
NgeRAGL_D_5477
WGS: NMRB01005477.1
RAG1L Pseudogenized ?
GFRY01085303.1
NgeRAGL_B_0732
WGS: NMRB01000732.1
GFRY01056743.1
N.geniculatus
Cnidaria
A.aurita
AauRAGL_B_0520
WGS: REGM01000520.1
1k 2k 3k 4k 5k 6k 7k 8k 9k 10k
5' 3'
b
Cnidarian incomplete / pseudogenized copies
A.Aurita complex sp. Pacific
WGS: REGL01002183.1
incomplete
A.Aurita complex sp. Pacific
WGS: REGL01004151.1
incomplete
P.damicornis
WGS: RCHS01004171.1
Incomplete: N-term on different scaffold
O.faveolata
WGS: MZGG01001906.1
incomplete
incomplete
O.faveolata
WGS: MZGG01001116.1
incomplete
incomplete
N.vectensis
WGS: ABAV01022117.1
incomplete
P.rus
WGS: OKRP01001303.1
incomplete
incomplete
LEGEND
Invertebrate RAG1L
Invertebrate RAG2L
Potentially pseudogenized
Terminal Inverted Repeat (TIR)
Target Site Duplication (TSD)
Transcriptomic support (CDS)
Transcriptomic support (UTR)
STOP codon
Frameshift
