# Supplementary material for: Identification of RAG-like transposons in protostomes suggests their ancient bilaterian origin
Source: Mob DNA. 2020 May 6;11:17. doi: 10.1186/s13100-020-00214-y (PMC7204232; doi:10.1186/s13100-020-00214-y)
Supplement: Supplementary file 3 — Additional file 3: Figure S3. Additional phylogenetic analyses. (a-d) Detailed phylogenetic trees of RAG1 and RAG1L protein sequences including several from: (a) Cephalochordata - indicated with blue shading., (b) Echinodermata - blue shading, (c) Mollusca - orange shading and (d) including one from cnidaria - green shading (e) RAG2/RAG2L phylogenetic trees. Trees were built using Maximum Likelihood and WAG substitution model as implemented in MEGA X [27] and are displayed as in Fig. 2b except that branches with bootstrap numbers below 50% were not collapsed together. Trees were built from a variable number of significant positions of their alignment: (a) 289, (b) 398, (c) 354, and (d) 469 respectively . [file 13100_2020_214_MOESM3_ESM.pdf]

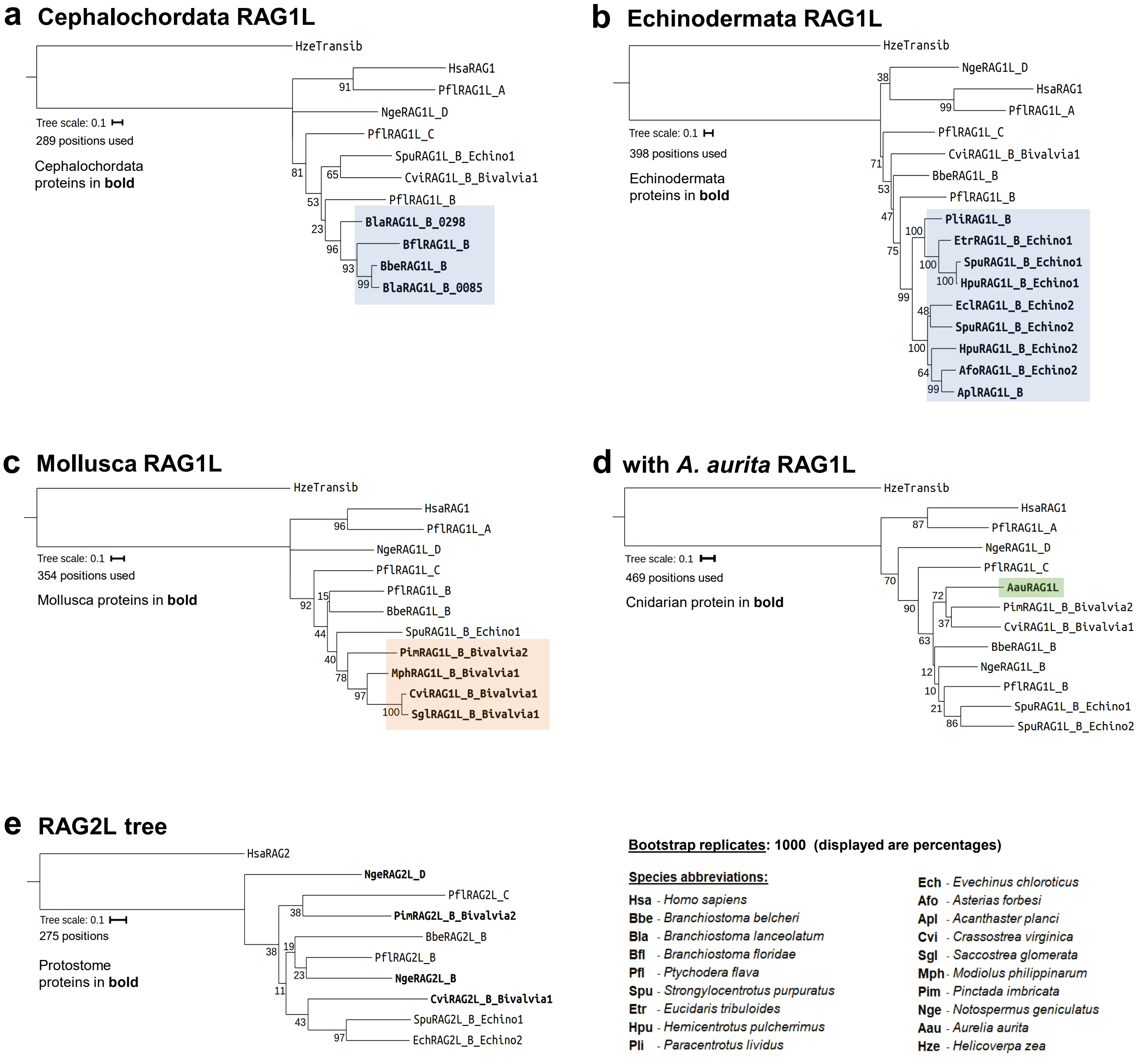

a Cephalochordata RAG1L
HzeTransib
HsaRAG1
PflRAG1L_A
NgeRAG1L_D
PflRAG1L_C
SpuRAG1L_B_Echino1
CviRAG1L_B_Bivalvia1
PflRAG1L_B
BlaRAG1L_B_0298
BflRAG1L_B
BbeRAG1L_B
BlaRAG1L_B_0085
91
81
65
53
23
96
93
99
Tree scale: 0.1
289 positions used
Cephalochordata proteins in bold
b Echinodermata RAG1L
HzeTransib
NgeRAG1L_D
HsaRAG1
PflRAG1L_A
PflRAG1L_C
CviRAG1L_B_Bivalvia1
BbeRAG1L_B
PflRAG1L_B
PliRAG1L_B
EtrRAG1L_B_Echino1
SpuRAG1L_B_Echino1
HpuRAG1L_B_Echino1
EclRAG1L_B_Echino2
SpuRAG1L_B_Echino2
HpuRAG1L_B_Echino2
AfoRAG1L_B_Echino2
AplRAG1L_B
38
99
71
53
47
100
75
100
100
99
48
100
64
99
Tree scale: 0.1
398 positions used
Echinodermata proteins in bold
c Mollusca RAG1L
HzeTransib
HsaRAG1
PflRAG1L_A
NgeRAG1L_D
PflRAG1L_C
PflRAG1L_B
BbeRAG1L_B
SpuRAG1L_B_Echino1
PimRAG1L_B_Bivalvia2
MphRAG1L_B_Bivalvia1
CviRAG1L_B_Bivalvia1
SglRAG1L_B_Bivalvia1
96
92
15
44
40
78
97
100
Tree scale: 0.1
354 positions used
Mollusca proteins in bold
d with A. aurita RAG1L
HzeTransib
HsaRAG1
PflRAG1L_A
NgeRAG1L_D
PflRAG1L_C
AauRAG1L
PimRAG1L_B_Bivalvia2
CviRAG1L_B_Bivalvia1
BbeRAG1L_B
NgeRAG1L_B
PflRAG1L_B
SpuRAG1L_B_Echino1
SpuRAG1L_B_Echino2
87
70
72
90
37
63
12
10
21
86
Tree scale: 0.1
469 positions used
Cnidarian protein in bold
e RAG2L tree
HsaRAG2
NgeRAG2L_D
PflRAG2L_C
PimRAG2L_B_Bivalvia2
BbeRAG2L_B
PflRAG2L_B
NgeRAG2L_B
CviRAG2L_B_Bivalvia1
SpuRAG2L_B_Echino1
EchRAG2L_B_Echino2
38
38
19
23
11
43
97
Tree scale: 0.1
275 positions
Protostome proteins in bold
Bootstrap replicates: 1000 (displayed are percentages)
Species abbreviations:
Hsa - Homo sapiens
Bbe - Branchiostoma belcheri
Bla - Branchiostoma lanceolatum
Bfl - Branchiostoma floridae
Pfl - Ptychodera flava
Spu - Strongylocentrotus purpuratus
Etr - Eucidaris tribuloides
Hpu - Hemicentrotus pulcherrimus
Pli - Paracentrotus lividus
Ech - Evechinus chloroticus
Afo - Asterias forbesi
Apl - Acanthaster planci
Cvi - Crassostrea virginica
Sgl - Saccostrea glomerata
Mph - Modiolus philippinarum
Pim - Pinctada imbricata
Nge - Notospermus geniculatus
Aau - Aurelia aurita
Hze - Helicoverpa zea
