# Supplementary material for: Identification of RAG-like transposons in protostomes suggests their ancient bilaterian origin
Source: Mob DNA. 2020 May 6;11:17. doi: 10.1186/s13100-020-00214-y (PMC7204232; doi:10.1186/s13100-020-00214-y)
Supplement: Supplementary file 4 — Additional file 4: Figure S4. Complementary RAG1L phylogenetic analyses using PhyML implementation [49, 50]. Trees are displayed as in Fig. 2b except that branches with bootstrap numbers below 50% were not collapsed together. Different substitution models were used as follows: (a) LG + G + I + F model selected via AIC minimization (b) LG + G + I model selected via BIC minimization (c) WAG substitution model. [file 13100_2020_214_MOESM4_ESM.pdf]

RAG1L trees - PhyML

a AIC : LG+G+I+F

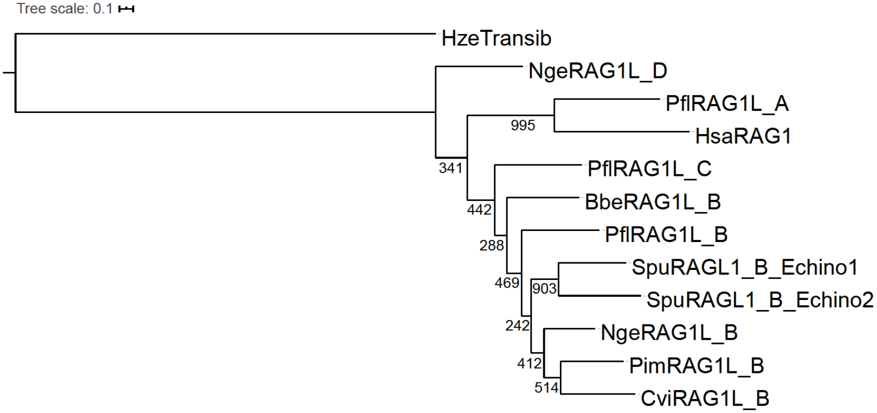

b BIC : LG+G+I

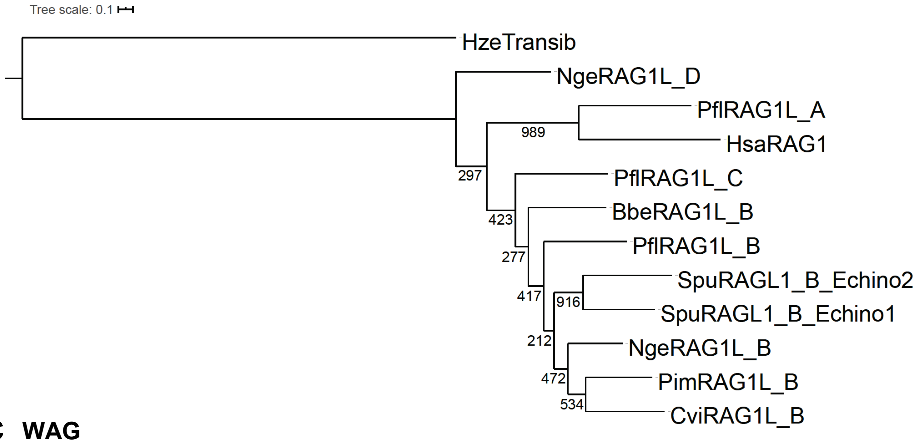

c WAG

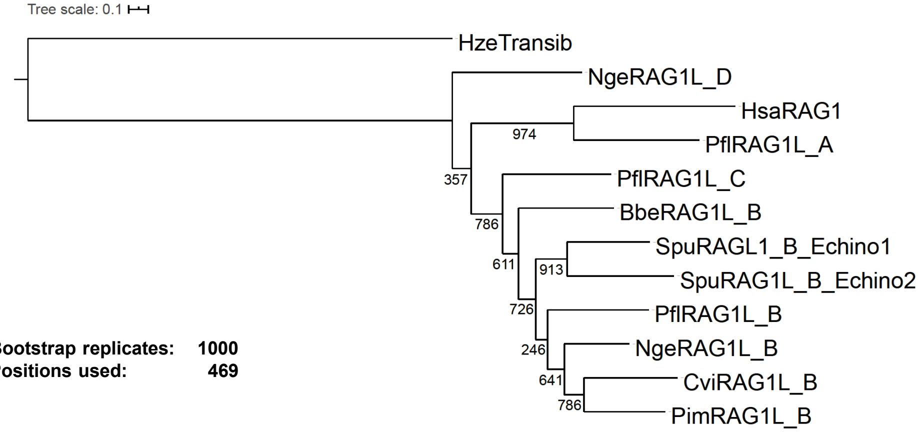

Bootstrap replicates: 1000
Positions used: 469
