# Supplementary material for: Identification of RAG-like transposons in protostomes suggests their ancient bilaterian origin
Source: Mob DNA. 2020 May 6;11:17. doi: 10.1186/s13100-020-00214-y (PMC7204232; doi:10.1186/s13100-020-00214-y)
Supplement: Supplementary file 5 — Additional file 5: Figure S5. (a) Conservation of functional relevant amino acids in RAG1 and RAG1L. The alignments depict the extensive conservation of some of the most important and well characterized amino acids in RAG1/RAG1L proteins (numbers given for BbeRAG1L): from left to right, catalytic carboxylates (D701, E764, D818, and E1063), residues implicated in controlling coupled versus uncoupled cleavage (A1064 and V751), a residue that facilitates transposition (M949), a histidine component of the active site (H894), and zinc-coordinating residues (C830, C833, H1035 and H1040). Residue E649 in mouse RAG1 contributes to synchronous, or “coupled”, cleavage by RAG at two RSSs, in part through its ability to form a hydrogen bond with S963 [22, 31]. The BbeRAG1L/2 L complex (BbeRAGL) exhibits less propensity for coupled cleavage in part because E649 has been replaced with V751 [22]. Valine is highly conserved at this position in RAG1L proteins from protostomes, suggesting that DNA cleavage by these proteins, if it occurs, would more likely resemble the uncoupled cleavage activity of BbeRAGL. Mutation of the charged residue R848 in mouse RAG1 to the hydrophobic residue methionine, as is found in BbeRAG1L, strongly activates the transposition activity of RAG [22]. Virtually all invertebrate RAG1L proteins, including those from protostomes, have a hydrophobic amino acid, most often methionine, at this position. (b) RAG2/RAG2L PHD domain alignment. The pattern of conserved cysteine and histidine residues (marked with X) are different between invertebrate RAG2L (top) and jawed vertebrate RAG2 (bottom). Variability logo (top) shows relative entropy (bits) calculated on the invertebrate alignment group. Amino acid color code as in Fig. 5. [file 13100_2020_214_MOESM5_ESM.pdf]

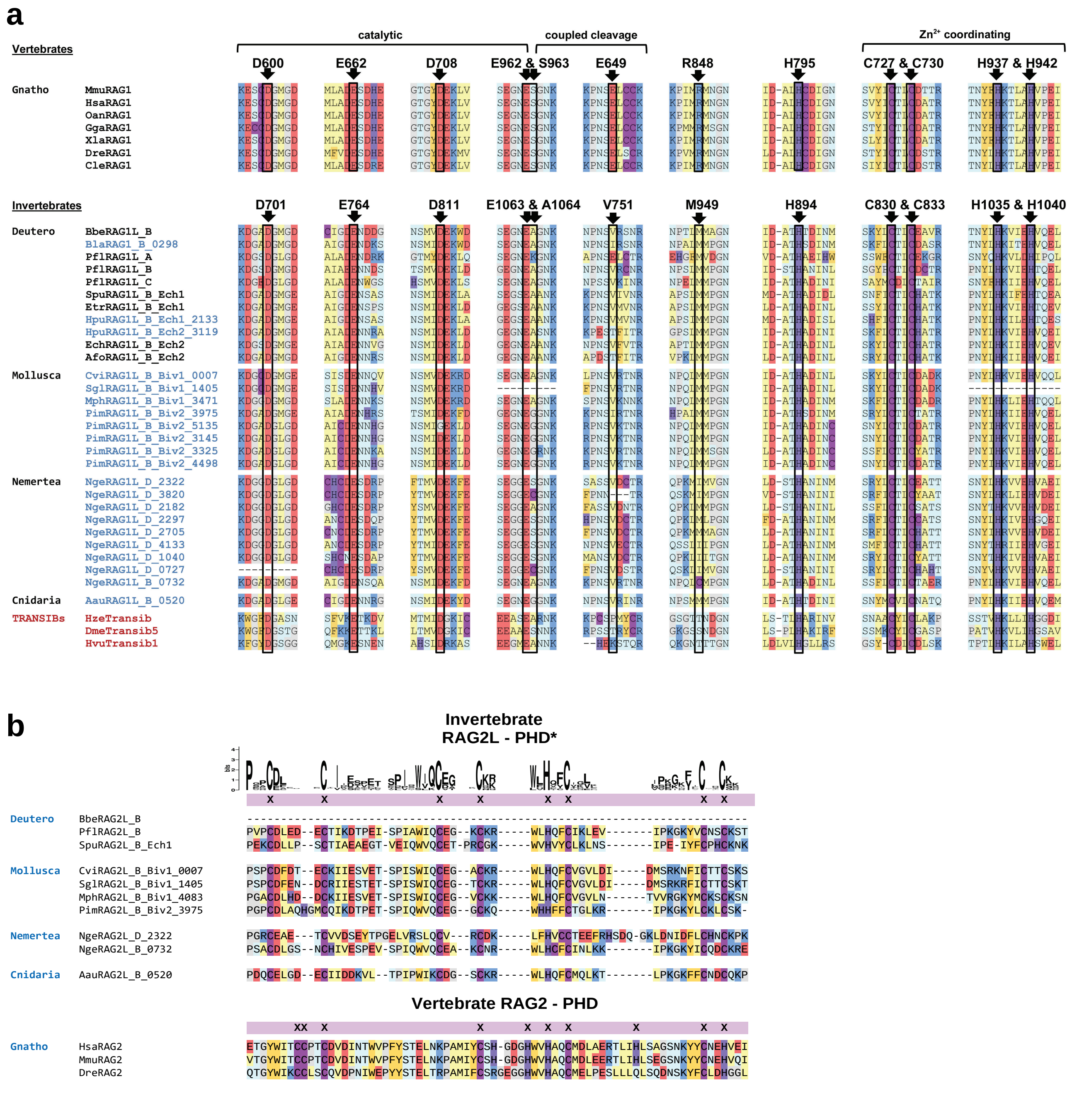

Species abbreviations:

Mmu - Mus musculus
Hsa - *Homo sapiens*
Oan - *Ornithorhynchus anatinus*
Gga - *Gallus gallus*
Xla - *Xenopus laevis*
Dre - Danio rerio
Cle - *Carcharhinus leucas*
Bbe - *Branchiostoma belcheri*
Bla - *Branchiostoma lanceolatum*
Pfl - *Ptychodera flava*
Spu - Strongylocentrotus purpuratus
Etr - *Eucidaris tribuloides*
Hpu - *Hemicentrotus pulcherrimus*
Ech - *Evechinus chloroticus*
Afo - *Asterias forbesi*
Cvi - Crassostrea virginica
Mph - *Modiolus philippinarum*
Pim - *Pinctada imbricata*
Nge - *Notospermus geniculatus*
Aau - *Aurelia aurita*
Hze - Helicoverpa zea
Dme - *Drosophila melanogaster*
Hvu - *Hydra vulgaris*
