# Supplementary material for: Identification of RAG-like transposons in protostomes suggests their ancient bilaterian origin
Source: Mob DNA. 2020 May 6;11:17. doi: 10.1186/s13100-020-00214-y (PMC7204232; doi:10.1186/s13100-020-00214-y)
Supplement: Supplementary file 6 — Additional file 6: Table S1. Genomic and transcriptomic data availability for bilaterian invertebrates. List of the bilaterian species for which there are Transcriptome Shotgun Assembly (TSA) and/or Whole Genome Shotgun (WGS) projects in the NCBI database as of February 26, 2019. Gnathostomata species are not listed. The taxonomic identifier is given in the NCBI taxid column and corresponds to that used in NCBI databases. The number of projects available is indicated. In the Number of TSA projects column, transcriptomic projects that were not marked as “TSA project” are indicated in parentheses. [file 13100_2020_214_MOESM6_ESM.pdf]

Table S1
Genomic and transcriptomic data availability for bilaterian invertebrates

Bilaterian WGS & TSA NCBI projects, before Feb 25th 2019

Clade	Species or subspecies	NCBI taxid	Number of WGS projects	Last WGS update	Number of TSA projects	Last TSA update
**CHORDATA**		**7711**				
Cephalochordata	Asymmetron lucayanum	223987	1	2016	2	2016
Cephalochordata	Branchiostoma belcheri	7741	3	2016	None	None
Cephalochordata	Branchiostoma floridae	7739	2	2014	3	2016
Cephalochordata	Branchiostoma lanceolatum	7740	1	2018	0 (Transcript: 1)	(2018)
Tunicata	Botryllus schlosseri	30301	1	2013	None	None
Tunicata	Ciona intestinalis	7719	2	2014	1	2015
Tunicata	Ciona savignyi	51511	1	2014	1	2018
Tunicata	Oikopleura dioica	34765	2	2010	1	2016
Tunicata	Phallusia mammillata	59560	1	2018	None	None
Tunicata	Salpa thompsoni	569448	1	2016	1	2016
Cyclostomata	Eptatretus burgeri	7764	1	2017	None	None
Cyclostomata	Ichtyomyzon castaneus	409034	None	None	1	2014
Cyclostomata	Ichtyomyzon fossor	245073	None	None	1	2014
Cyclostomata	Lethenteron camtschaticum	980415	1	2014	None	None
Cyclostomata	Petromyzon marinus	7757	2	2019	None	None
**Gnathostomata**		7776				
**ECHINODERMATA**		**7586**				
Asteroidea	Acanthaster planci	133434	3	2016	None	None
Asteroidea	Asterias forbesi	7603	None	None	1	2014
Asteroidea	Asterias rubens	7604	None	None	1	2014
Asteroidea	Asterias amurensis	7602	None	None	2	2015
Asteroidea	Echinaster spinulosus	1451296	None	None	1	2014
Asteroidea	Henricia sp. AR-2014	1462731	None	None	1	2014
Asteroidea	Leptasterias sp. AR-2014	1462732	None	None	1	2014
Asteroidea	Luidia clathrata	133437	None	None	1	2014
Asteroidea	Marthasterias glacialis	7609	None	None	1	2014
Asteroidea	Patiria miniata	46514	1	2014	2	2018
Asteroidea	Patiriella pectinifera	7594	None	None	3	2017
Asteroidea	Patiriella regularis	46519	1	2015	None	None
Asteroidea	Peribolaster folliculatus	478267	None	None	1	2016
Asteroidea	Pisaster ochraceus	7612	None	None	1	2014
Crinoidea	Anneissia japonica	1529436	None	None	1	2014
Crinoidea	Psathyrometra fragilis	707735	None	None	1	2016
Echinoidea	Arbacia punctulata	7641	None	None	1	2016
Echinoidea	Echinarachnius parma	869203	None	None	1	2014
Echinoidea	Eucidaris tribuloides	7632	1	2015	1	2014
Echinoidea	Evechinus chloroticus	137513	None	None	1	2014
Echinoidea	Hemicentrotus pulcherrimus	7650	1	2018	1	2018
Echinoidea	Loxechinus albus	240836	None	None	1	2018
Echinoidea	Lytechinus variegatus	7654	1	2015	1	2014
Echinoidea	Paracentrotus lividus	7656	None	None	3	2019
Echinoidea	Sphaerechinus granularis	39374	None	None	1	2014
Echinoidea	Strongylocentrotus purpuratus	7668	1	2015	1	2015
Holothuroidea	Abyssocucumis albatrossi	1777472	None	None	1	2016
Holothuroidea	Apostichopus californicus	2032702	None	None	1	2014
Holothuroidea	Apostichopus japonicus	307972	2	2017	8	2018
Holothuroidea	Apostichopus parvimensis	1902835	1	2015	None	None
Holothuroidea	Cladolabes schmeltzii	2033684	None	None	1	2018
Holothuroidea	Sclerodactyla briareus	7710	None	None	1	2014
Ophiuroidea	Amphiura filiformis	82378	None	None	1	2018
Ophiuroidea	Ophiocoma echinata	331088	None	None	1	2014
Ophiuroidea	Ophionereis fasciata	1401014	1	2015	None	None
Ophiuroidea	Ophiothrix spiculata	1266684	1	2015	None	None
**HEMICHORDATA**		**10219**				
Enteropneusta	Ptychodera flava	63121	1	2016	1	2015
Enteropneusta	Saccoglossus kowalevskii	10224	1	2013	None	None
**MOLLUSCA**		**6447**				
**Bivalvia**						
Heterodonta: Lucinoida	Phacoides pectinatus	244486	None	None	1	2018
Heterodonta: Myoida	Bankia setacea	693219	1	2016	None	None
Heterodonta: Myoida	Panopea globosa	1237092	None	None	1	2018
Heterodonta: Veneroida	Corbicula fluminea	45949	1	2016	1	2018
Heterodonta: Veneroida	Dreissena polymorpha	45954	1	2015	1	2017
Heterodonta: Veneroida	Eurhomalea rufa	1912411	None	None	1	2016
Heterodonta: Veneroida	Limecola balthica balthica	1697945	None	None	1	2017
Heterodonta: Veneroida	Paphia undulata	223148	None	None	1	2018
Heterodonta: Veneroida	Ruditapes decussatus	104385	None	None	1	2018
Heterodonta: Veneroida	Ruditapes philippinarum	129788	None	None	2	2018
Heterodonta: Veneroida	Sinonovacula constricta	98310	None	None	1	2013
Paleoheterodonta: Unionida	Amblema plicata	47525	None	None	1	2018
Paleoheterodonta: Unionida	Elliptio complanata	55832	None	None	1	2014
Paleoheterodonta: Unionida	Venustaconcha ellipsiformis	301928	1	2018	None	None

Clade	Species or subspecies	NCBI taxid	Number of WGS projects	Last WGS update	Number of TSA projects	Last TSA update
Pteriomorphia: Arcoida	Scapharca broughtonii	148819	None	None	1	2016
Pteriomorphia: Mytiloida	Bathymodiolus platifrons	220390	1	2017	1	2016
Pteriomorphia: Mytiloida	Limnoperna fortunei	356393	1	2018	1	2014
Pteriomorphia: Mytiloida	Lithophaga lithophaga	112135	None	None	1	2018
Pteriomorphia: Mytiloida	Modiolus philippinarum	310899	1	2017	None	None
Pteriomorphia: Mytiloida	Mytilus californianus	6549	None	None	3	2018
Pteriomorphia: Mytiloida	Mytilus edulis	6550	None	None	2	2018
Pteriomorphia: Mytiloida	Mytilus galloprovincialis	29158	2	2017	4	2018
Pteriomorphia: Mytiloida	Perna canaliculus	38949	None	None	1	2018
Pteriomorphia: Mytiloida	Perna viridis	73031	None	None	2	2018
Pteriomorphia: Mytiloida	Septifer virgatus	182745	None	None	1	2017
Pteriomorphia: Ostreoida	Crassostrea angulata	558553	None	None	1	2018
Pteriomorphia: Ostreoida	Crassostrea gigas	29159	1	2013	2	2018
Pteriomorphia: Ostreoida	Crassostrea hongkongensis	298176	None	None	1	2018
Pteriomorphia: Ostreoida	Crassostrea nippona	121615	None	None	1	2018
Pteriomorphia: Ostreoida	Crassostrea virginica	6565	1	2017	1	2018
Pteriomorphia: Ostreoida	Saccostrea glomerata	157728	1	2018	3	2018
Pteriomorphia: Ostreoida	Saccostrea sp. Non-mordax lineage J	1778413	None	None	1	2018
Pteriomorphia: Pectinoida	Adamussium colbecki	95546	None	None	1	2018
Pteriomorphia: Pectinoida	Mimachlamys varia	50417	None	None	1	2018
Pteriomorphia: Pectinoida	Mizuhopecten yessoensis	6573	1	2017	None	None
Pteriomorphia: Pectinoida	Nodipecten subnodosus	330909	None	None	3	2018
Pteriomorphia: Pectinoida	Pecten maximus	6579	None	None	2	2019
Pteriomorphia: Pectinoida	Placopecten magellanicus	6577	None	None	1	2015
Pteriomorphia: Pterioida	Pinctada fucata	50426	None	None	1	2016
Pteriomorphia: Pterioida	Pinctada imbricata	66713	1	2017	None	None
Pteriomorphia: Pterioida	Pinctada margaritifera	102329	None	None	1	2017
Pteriomorphia: Pterioida	Pinctada penguin	113549	None	None	1	2017
**Cephalopoda**						
Octopoda	Abdopus aculeatus	515833	None	None	1	2013
Octopoda	Hapalochlaena maculosa	61716	None	None	2	2016
Octopoda	Octopus bimaculoides	37653	1	2015	None	None
Octopoda	Octopus cyanea	34525	None	None	1	2013
Octopoda	Octopus kaurna	243731	None	None	2	2017
Octopoda	Octopus maya	623738	None	None	3	2018
Octopoda	Octopus vulgaris	6645	1	2019	1	2019
Octopoda	Pareledone turqueti	164545	None	None	1	2013
Sepiida	Sepia esculenta	31210	None	None	2	2018
Sepiida	Sepia latimanus	34528	None	None	1	2013
Sepiida	Sepia pharaonis	158019	None	None	2	2016
Sepiida	Sepiella maindroni	153280	None	None	1	2017
Sepiida	Sepioloidea lineolata	61742	None	None	1	2016
Sepiolida	Euprymna scolopes	6613	None	None	3	2017
Sepiolida	Euprymna tasmanica	70205	None	None	1	2017
Sepiolida	Idiosepius notoides	66914	None	None	1	2017
Teuthida	Chiroteuthis calyx	559536	None	None	1	2018
Teuthida	Dosidicus gigas	346249	None	None	2	2018
Teuthida	Loliolus noctiluca	70206	None	None	1	2013
Teuthida	Octopoteuthis deletron	1582096	None	None	1	2018
Teuthida	Pterygioteuthis hoylei	559549	None	None	1	2018
Teuthida	Sepioteuthis australis	61682	None	None	1	2013
Teuthida	Sepioteuthis lessoniana	34570	None	None	1	2014
Teuthida	Watasenia scintillans	6625	None	None	3	2016
Vampyromorpha	Vampyroteuthis infernalis	55288	None	None	1	2018
**Gastropoda**						
Caenogastropoda	Bithynia siamensis goniomphalos	479249	None	None	2	2018
Caenogastropoda	Cipangopaludina cathayensis	570432	None	None	1	2015
Caenogastropoda	Colubraria reticulata	604273	1	2016	None	None
Caenogastropoda	Conus consors	101297	1	2019	None	None
Caenogastropoda	Conus geographus	6491	None	None	1	2014
Caenogastropoda	Conus gloriamaris	37336	None	None	1	2017
Caenogastropoda	Conus lenavati	1519839	None	None	1	2015
Caenogastropoda	Conus lividus	89426	None	None	2	2014
Caenogastropoda	Conus sanguinolentus	97184	None	None	2	2014
Caenogastropoda	Conus sp. n. 1 NP-2014	1501980	None	None	2	2014
Caenogastropoda	Conus tribblei	101761	1	2016	2	2015
Caenogastropoda	Conus victoriae	319920	None	None	1	2015
Caenogastropoda	Crepidula atrasolea	136598	None	None	1	2017
Caenogastropoda	Crepidula navicella	1707815	None	None	1	2016
Caenogastropoda	Gemmula speciosa	439592	None	None	1	2014
Caenogastropoda	Littorina littorea	31216	None	None	1	2018
Caenogastropoda	Pomacea canaliculata	400727	1	2018	1	2015
Caenogastropoda	Potamopyrgus antipodarum	145637	None	None	2	2018
Caenogastropoda	Rapana venosa	55521	None	None	2	2018
Caenogastropoda	Semisulcospira coreana	364284	None	None	1	2018
Caenogastropoda	Turridrupa cerithina	1077925	None	None	1	2014
Caenogastropoda	Unedogemmula bisaya	746885	None	None	1	2014
Heterobranchia	Aplysia californica	6500	1	2014	9	2014
Heterobranchia	Arion vulgaris	1028688	None	None	1	2017
Heterobranchia	Bathyberthella antarctica	157136	None	None	1	2017
Heterobranchia	Biomphalaria glabrata	6526	1	2014	3	2015

Clade	Species or subspecies	NCBI taxid	Number of WGS projects	Last WGS update	Number of TSA projects	Last TSA update
Heterobranchia	Biomphalaria pfeifferi	112525	None	None	1	2018
Heterobranchia	Cepaea nemoralis	28835	None	None	1	2017
Heterobranchia	Clione limacina	71516	None	None	1	2016
Heterobranchia	Elysia chlorotica	188477	1	2019	None	None
Heterobranchia	Elysia cornigera	698696	None	None	1	2015
Heterobranchia	Elysia timida	154625	None	None	1	2015
Heterobranchia	Helix aspersa	6535	None	None	1	2017
Heterobranchia	Limacina antarctica	220648	None	None	1	2016
Heterobranchia	Limacina retroversa	1302792	None	None	1	2016
Heterobranchia	Lymnaea stagnalis	6523	1	2016	None	None
Heterobranchia	Melibe leonina	76178	None	None	1	2017
Heterobranchia	Phylliroe bucephala	1903125	None	None	1	2018
Heterobranchia	Radix auricularia	52793	1	2017	None	None
Patellogastropoda	Lottia gigantea	225164	2	2014	None	None
Vetigastropoda	Haliotis discus hannai	42344	None	None	2	2016
Vetigastropoda	Haliotis fulgens	6456	None	None	1	2018
Vetigastropoda	Haliotis laevigata	36097	None	None	1	2017
Vetigastropoda	Haliotis midae	36098	None	None	2	2016
Vetigastropoda	Haliotis rubra	36100	1	2018	None	None
Vetigastropoda	Haliotis rufescens	6454	1	2018	None	None
Vetigastropoda	Haliotis tuberculata	36103	None	None	1	2018
Vetigastropoda	Tegula atra	80343	None	None	1	2016
**PLATYHELMINTHES**		**6157**	91		43	
**ARTHROPODA**		**6656**	2249		1453	
**ONYCHOPHORA**		**27563**				
Udeonychophora	Euperipatoides rowelli	49087	1	2018	None	None
**TARDIGRADA**		**42241**				
Eutardigrada	Hypsibius dujardini	232323	3	2017	2	2017
Eutardigrada	Milnesium tardigradum	46460	None	None	1	2017
Eutardigrada	Paramacrobiotus richtersi	697321	None	None	1	2017
Eutardigrada	Ramazzottius varieornatus	947166	1	2016	None	None
Heterotardigrada	Echiniscus testudo	399800	None	None	1	2015
**NEMATODA**		**6231**	190		31	
**ANNELIDA**		**6340**				
Clitellata	Amynthas corticis	351238	2	2017	None	None
Clitellata	Elsenia fetida	6396	2	2019	None	None
Clitellata	Enchytraeus crypticus	913645	None	None	1	2014
Clitellata	Glossoscolex paulistus	1046353	None	None	1	2015
Clitellata	Haementeria vizottoi	1628691	None	None	1	2015
Clitellata	Helobdella robusta	6412	2	2014	None	None
Clitellata	Hirudo medicinalis	6421	None	None	1	2015
Clitellata	Hirudo verbana	311461	None	None	1	2018
Clitellata	Hormogaster elisae	452200	None	None	1	2015
Clitellata	Hormogaster samnitica	991110	None	None	1	2015
Clitellata	Lumbricus terrestris	6398	None	None	1	2014
Clitellata	Olavius algarvensis	188229	None	None	1	2017
Polychaeta	Capitella telela	283909	1	2014	None	None
Polychaeta	Glycera dibranchiata	6350	None	None	1	2014
Polychaeta	Glycera fallax	1446102	None	None	1	2014
Polychaeta	Glycera tridactyla	104710	None	None	2	2014
Polychaeta	Harmothoe imbricata	61848	None	None	1	2018
Polychaeta	Hydroides elegans	216498	1	2016	None	None
Polychaeta	Lamellibrachia satsuma	104711	None	None	1	2017
Polychaeta	Ophryotrocha diadema	169117	None	None	1	2016
Polychaeta	Perinereis aibuhitensis	126650	None	None	1	2016
Polychaeta	Platynereis dumerilii	6359	None	None	1	2015
Polychaeta	Pygospio elegans	51279	None	None	1	2017
Polychaeta	Spirobranchus lamarcki	2082999	None	None	1	2018
Polychaeta	Streblospio benedicti	95538	None	None	1	2015
**PRIAPULIDA**		**33467**				
Priapulimorpha	Halicryptus spinulosus	160677	None	None	1	2015
Priapulimorpha	Priapulus caudatus	37621	1	2015	1	2015
**NEMERTEA**		**6217**				
Pilidiophora	Notospermus geniculatus	416868	1	2017	1	2017
**ROTIFERA**		**10190**				
Bdelloidea	Adineta ricciae	249248	2	2018	None	None
Bdelloidea	Adineta vaga	104782	3	2018	None	None
Bdelloidea	Rotaria macrura	392029	2	2018	None	None
Bdelloidea	Rotaria magnacalcarata	392030	3	2018	1	2015
Bdelloidea	Rotaria socialis	392032	None	None	1	2015
Bdelloidea	Rotaria sordida	392033	None	None	1	2015
Bdelloidea	Rotaria tardigrada	392034	None	None	1	2015
Monogononta	Brachionus calyciflorus	104777	1	2018	2	2013
Monogononta	Brachionus koreanus	1199090	None	None	1	2016
Monogononta	Brachionus manjavacas	667381	None	None	2	2017
Monogononta	Brachionus plicatilis	10195	1	2018	1	2017
**BRACHIOPODA**		**7568**				
Linguliformea	Lingula anatina	7574	1	2018	1	2015
Phoroniformea	Phoronis australis	115415	1	2017	1	2017
**XENACOELOMORPHA**		**1312402**				
Acoelomorpha	Hofstenia miamia	442651	None	None	1	2017

Clade	Species or subspecies	NCBI taxid	Number of WGS projects	Last WGS update	Number of TSA projects	Last TSA update
Acoelomorpha	Isodiametra pulchra	504439	None	None	1	2017
Acoelomorpha	Symsagittifera roscoffensis	84072	None	None	1	2017
Xenoturbellida	Xenoturbella bocki	242395	None	None	1	2018
**KINORYNCHA**		**51516**				
Homalorhagida	Pycnophyes kielensis	51520	None	None	1	2015
