# Supplementary material for: Identification of RAG-like transposons in protostomes suggests their ancient bilaterian origin
Source: Mob DNA. 2020 May 6;11:17. doi: 10.1186/s13100-020-00214-y (PMC7204232; doi:10.1186/s13100-020-00214-y)
Supplement: Supplementary file 7 — Additional file 7. Alignment S1 Multiple sequence alignment of (a) RAG1/RAG1L and (b) RAG2/RAG2L predicted proteins. Domains, sequence motifs, secondary structure assignment, protein-protein and protein-DNA contact interactions (within 5 Å) displayed above the alignment derive from the BbeRAGL cryo-EM structure (PDB: 6B40). Additionally, for RAG1/RAG1L (a), acidic catalytic residues, red; active site residue mouse H795, purple; zinc coordinating residues within ZDD (*) and ZnC2 and ZnH2 (#) are indicated above the sequences, while for RAG2/RAG2L (b) the beta sheet regions of each kelch-type blade and the GG motifs are shown above the alignment. Locations at which coding sequences span exon boundaries are underlined. Sequence descriptions including references to genomic, transcriptomic or protein databases are shown at the end of the alignment, along with a legend of the symbols used. [file 13100_2020_214_MOESM7_ESM.pdf]

Alignment S1

(a) RAG1/RAG1L Multiple Sequence Alignment

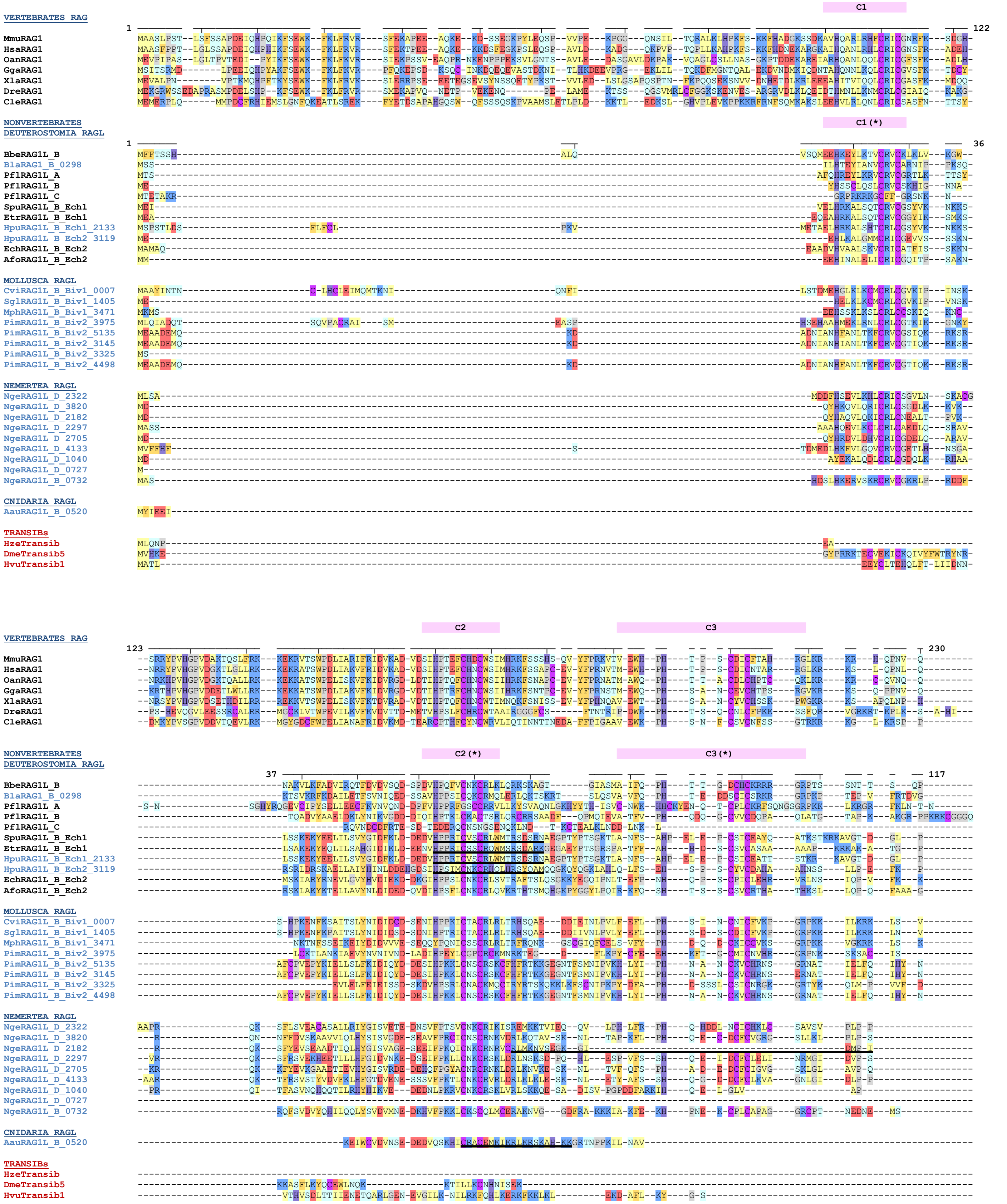

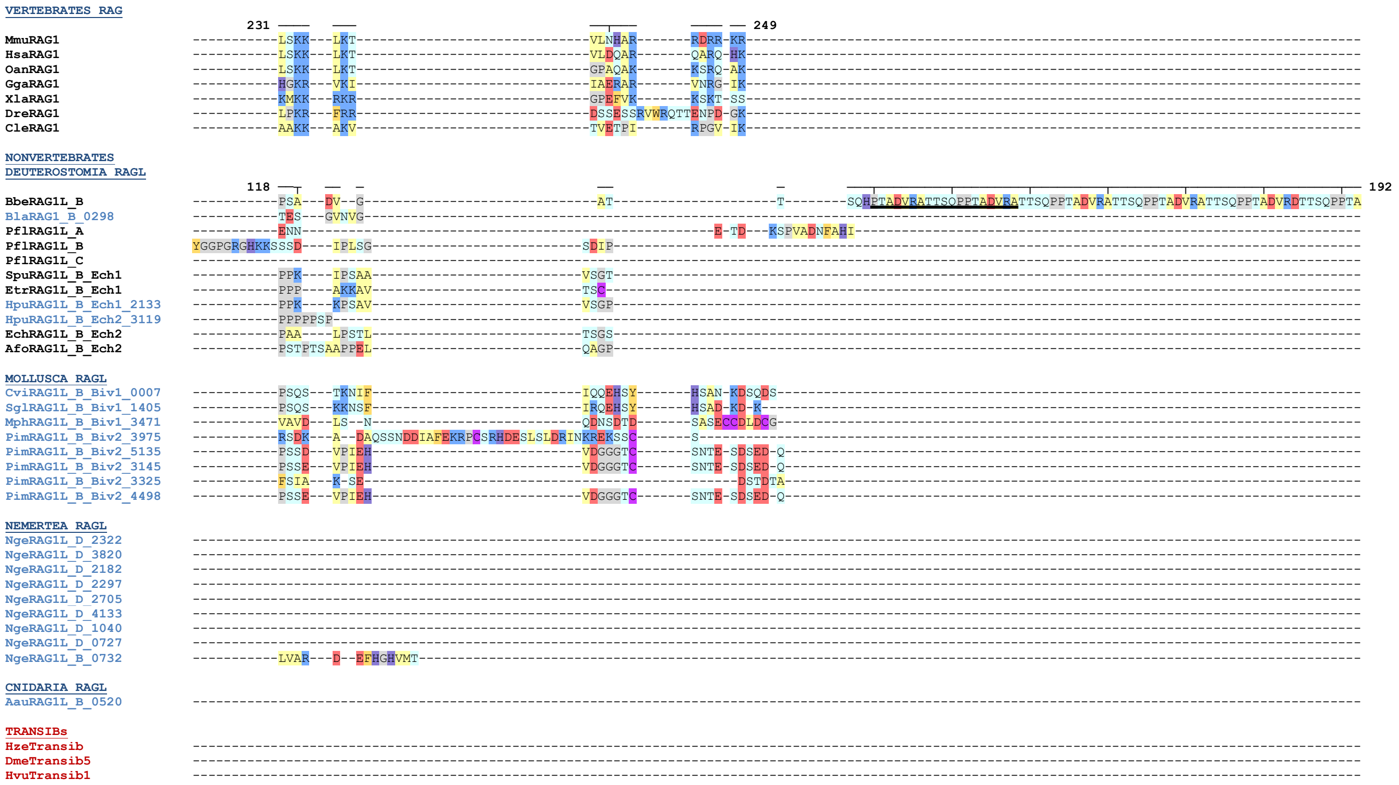

VERTEBRATES RAG
231
249
MmuRAG1
HsaRAG1
OanRAG1
GgaRAG1
XlaRAG1
DreRAG1
CleRAG1
NONVERTEBRATES
DEUTEROSTOMIA RAGL
118
192
BbeRAG1L_B
BlaRAG1_B_0298
PflRAG1L_A
PflRAG1L_B
PflRAG1L_C
SpuRAG1L_B_Ech1
EtrRAG1L_B_Ech1
HpuRAG1L_B_Ech1_2133
HpuRAG1L_B_Ech2_3119
EchRAG1L_B_Ech2
AfoRAG1L_B_Ech2
MOLLUSCA RAGL
CviRAG1L_B_Biv1_0007
SglRAG1L_B_Biv1_1405
MphRAG1L_B_Biv1_3471
PimRAG1L_B_Biv2_3975
PimRAG1L_B_Biv2_5135
PimRAG1L_B_Biv2_3145
PimRAG1L_B_Biv2_3325
PimRAG1L_B_Biv2_4498
NEMERTEA RAGL
NgeRAG1L_D_2322
NgeRAG1L_D_3820
NgeRAG1L_D_2182
NgeRAG1L_D_2297
NgeRAG1L_D_2705
NgeRAG1L_D_4133
NgeRAG1L_D_1040
NgeRAG1L_D_0727
NgeRAG1L_B_0732
CNIDARIA RAGL
AauRAG1L_B_0520
TRANSIBs
HzeTransib
DmeTransib5
HvuTransib1

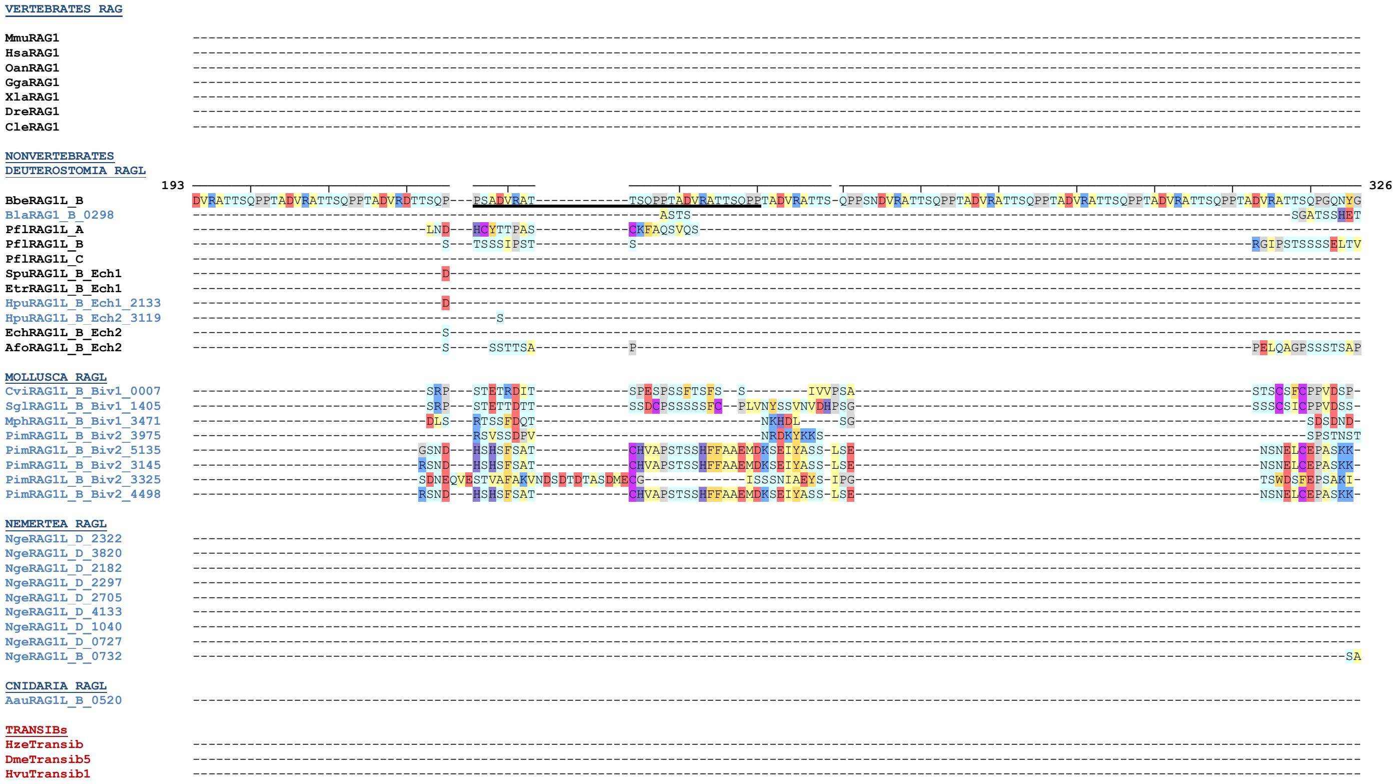

VERTEBRATES RAG
MmuRAG1
HsaRAG1
OanRAG1
GgaRAG1
XlaRAG1
DreRAG1
CleRAG1
NONVERTEBRATES
DEUTEROSTOMIA RAGL
193
326
BbeRAG1L_B
BlaRAG1_B_0298
PflRAG1L_A
PflRAG1L_B
PflRAG1L_C
SpuRAG1L_B_Ech1
EtrRAG1L_B_Ech1
HpuRAG1L_B_Ech1_2133
HpuRAG1L_B_Ech2_3119
EchRAG1L_B_Ech2
AfoRAG1L_B_Ech2
MOLLUSCA RAGL
CviRAG1L_B_Biv1_0007
SglRAG1L_B_Biv1_1405
MphRAG1L_B_Biv1_3471
PimRAG1L_B_Biv2_3975
PimRAG1L_B_Biv2_5135
PimRAG1L_B_Biv2_3145
PimRAG1L_B_Biv2_3325
PimRAG1L_B_Biv2_4498
NEMERTEA RAGL
NgeRAG1L_D_2322
NgeRAG1L_D_3820
NgeRAG1L_D_2182
NgeRAG1L_D_2297
NgeRAG1L_D_2705
NgeRAG1L_D_4133
NgeRAG1L_D_1040
NgeRAG1L_D_0727
NgeRAG1L_B_0732
CNIDARIA RAGL
AauRAG1L_B_0520
TRANSIBs
HzeTransib
DmeTransib5
HvuTransib1

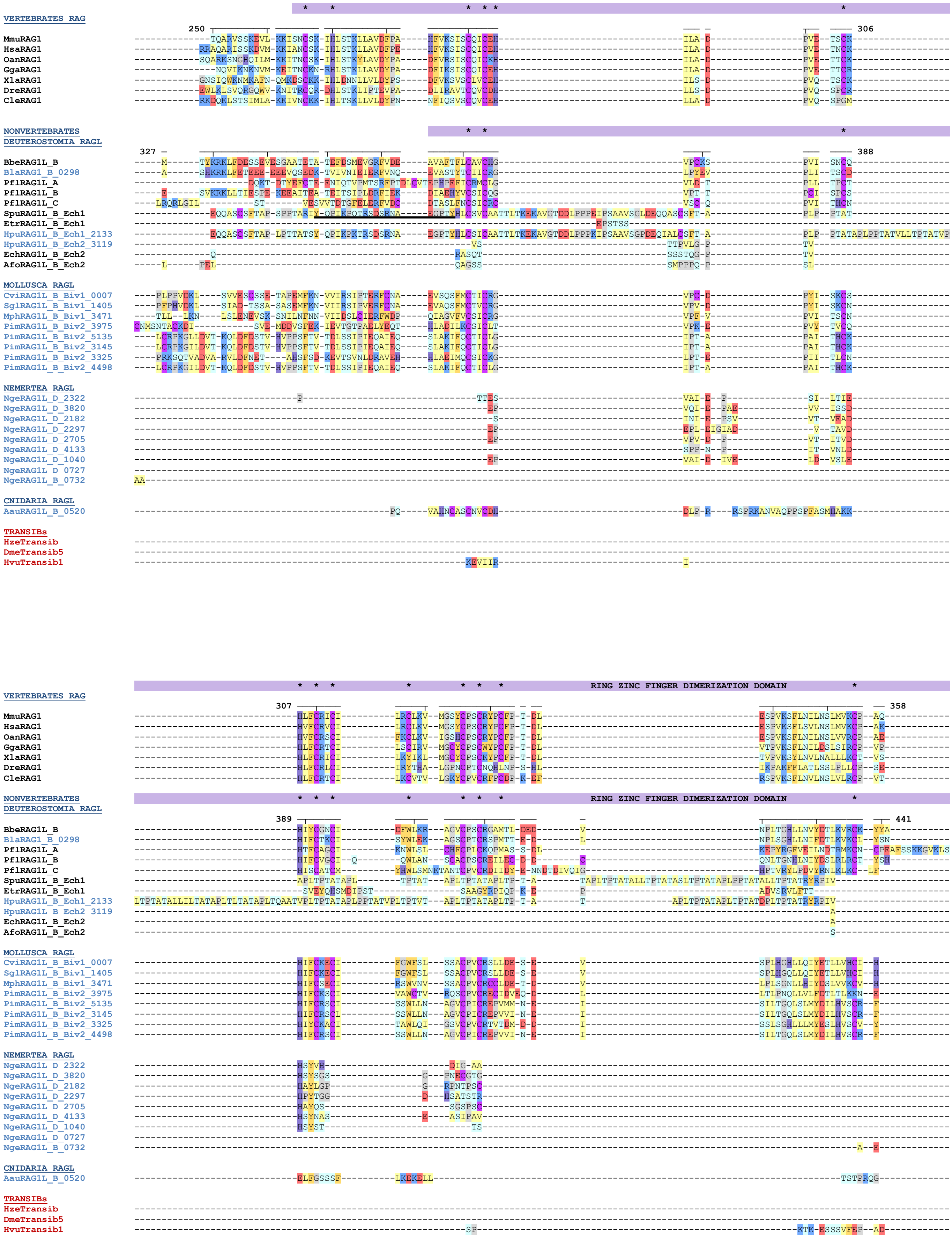

VERTEBRATES RAG
MmuRAG1
HsaRAG1
OanRAG1
GgaRAG1
XlaRAG1
DreRAG1
CleRAG1
NONVERTEBRATES DEUTEROSTOMIA RAGL
BbeRAG1L_B
BlaRAG1_B_0298
PflRAG1L_A
PflRAG1L_B
PflRAG1L_C
SpuRAG1L_B_Ech1
EtrRAG1L_B_Ech1
HpuRAG1L_B_Ech1_2133
HpuRAG1L_B_Ech2_3119
EchRAG1L_B_Ech2
AfoRAG1L_B_Ech2
MOLLUSCA RAGL
CviRAG1L_B_Biv1_0007
SglRAG1L_B_Biv1_1405
MphRAG1L_B_Biv1_3471
PimRAG1L_B_Biv2_3975
PimRAG1L_B_Biv2_5135
PimRAG1L_B_Biv2_3145
PimRAG1L_B_Biv2_3325
PimRAG1L_B_Biv2_4498
NEMERTEA RAGL
NgeRAG1L_D_2322
NgeRAG1L_D_3820
NgeRAG1L_D_2182
NgeRAG1L_D_2297
NgeRAG1L_D_2705
NgeRAG1L_D_4133
NgeRAG1L_D_1040
NgeRAG1L_D_0727
NgeRAG1L_B_0732
CNIDARIA RAGL
AauRAG1L_B_0520
TRANSIBs
HzeTransib
DmeTransib5
HvuTransib1
250
306
327
388
307
358
389
441
RING ZINC FINGER DIMERIZATION DOMAIN

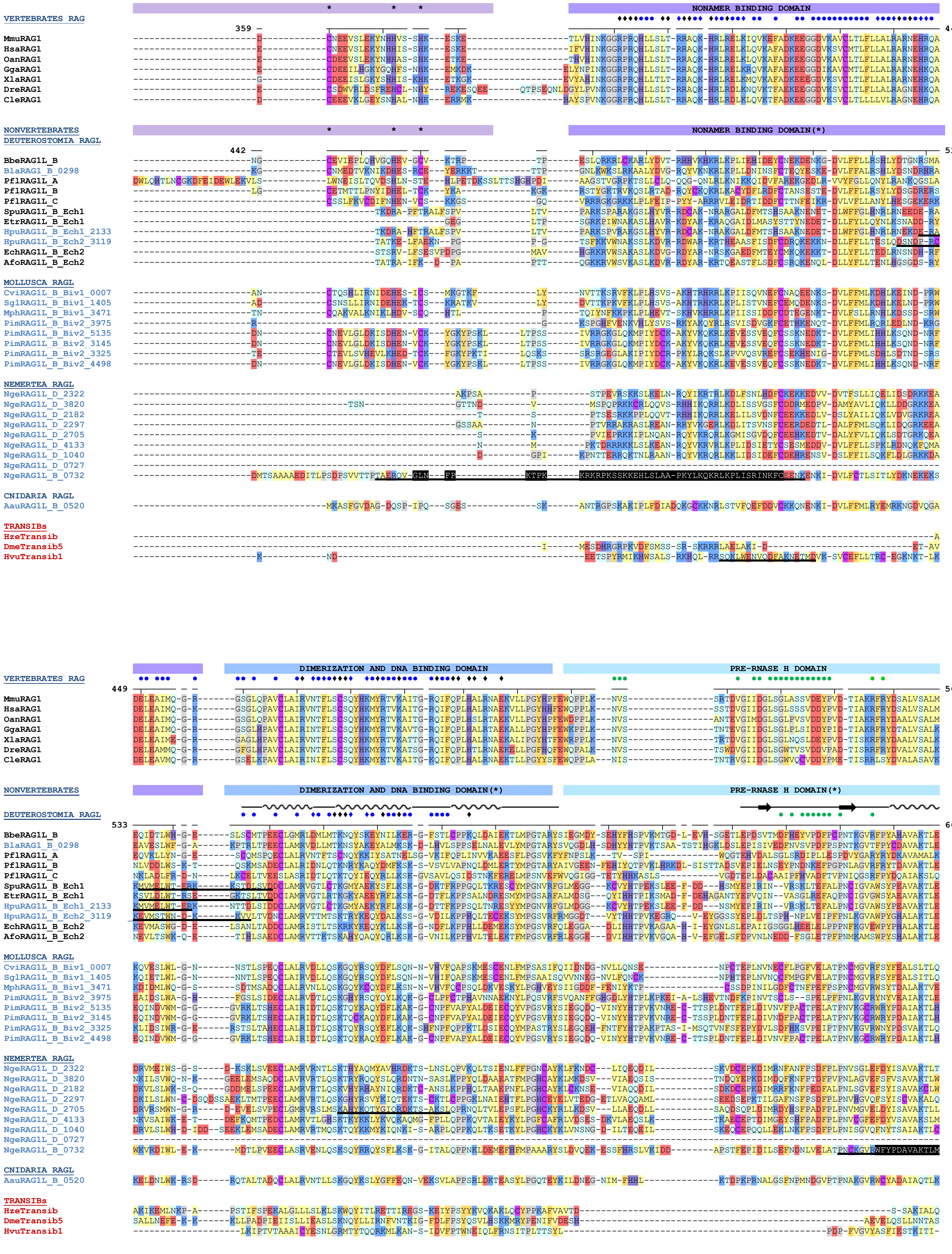

VERTEBRATES RAG
NONAMER BINDING DOMAIN
359
448
MmuRAG1
HsaRAG1
OanRAG1
GgaRAG1
XlaRAG1
DreRAG1
CleRAG1
NONVERTEBRATES
DEUTEROSTOMIA RAGL
NONAMER BINDING DOMAIN(*)
442
532
BbeRAG1L_B
BlaRAG1_B_0298
PflRAG1L_A
PflRAG1L_B
PflRAG1L_C
SpuRAG1L_B_Ech1
EtrRAG1L_B_Ech1
HpuRAG1L_B_Ech1_2133
HpuRAG1L_B_Ech2_3119
EchRAG1L_B_Ech2
AfoRAG1L_B_Ech2
MOLLUSCA RAGL
CviRAG1L_B_Biv1_0007
SglRAG1L_B_Biv1_1405
MphRAG1L_B_Biv1_3471
PimRAG1L_B_Biv2_3975
PimRAG1L_B_Biv2_5135
PimRAG1L_B_Biv2_3145
PimRAG1L_B_Biv2_3325
PimRAG1L_B_Biv2_4498
NEMERTEA RAGL
NgeRAG1L_D_2322
NgeRAG1L_D_3820
NgeRAG1L_D_2182
NgeRAG1L_D_2297
NgeRAG1L_D_2705
NgeRAG1L_D_4133
NgeRAG1L_D_1040
NgeRAG1L_D_0727
NgeRAG1L_B_0732
CNIDARIA RAGL
AauRAG1L_B_0520
TRANSIBs
HzeTransib
DmeTransib5
HvuTransib1
VERTEBRATES RAG
DIMERIZATION AND DNA BINDING DOMAIN
PRE-RNASE H DOMAIN
449
568
NONVERTEBRATES
DIMERIZATION AND DNA BINDING DOMAIN(*)
PRE-RNASE H DOMAIN(*)
DEUTEROSTOMIA RAGL
533
668

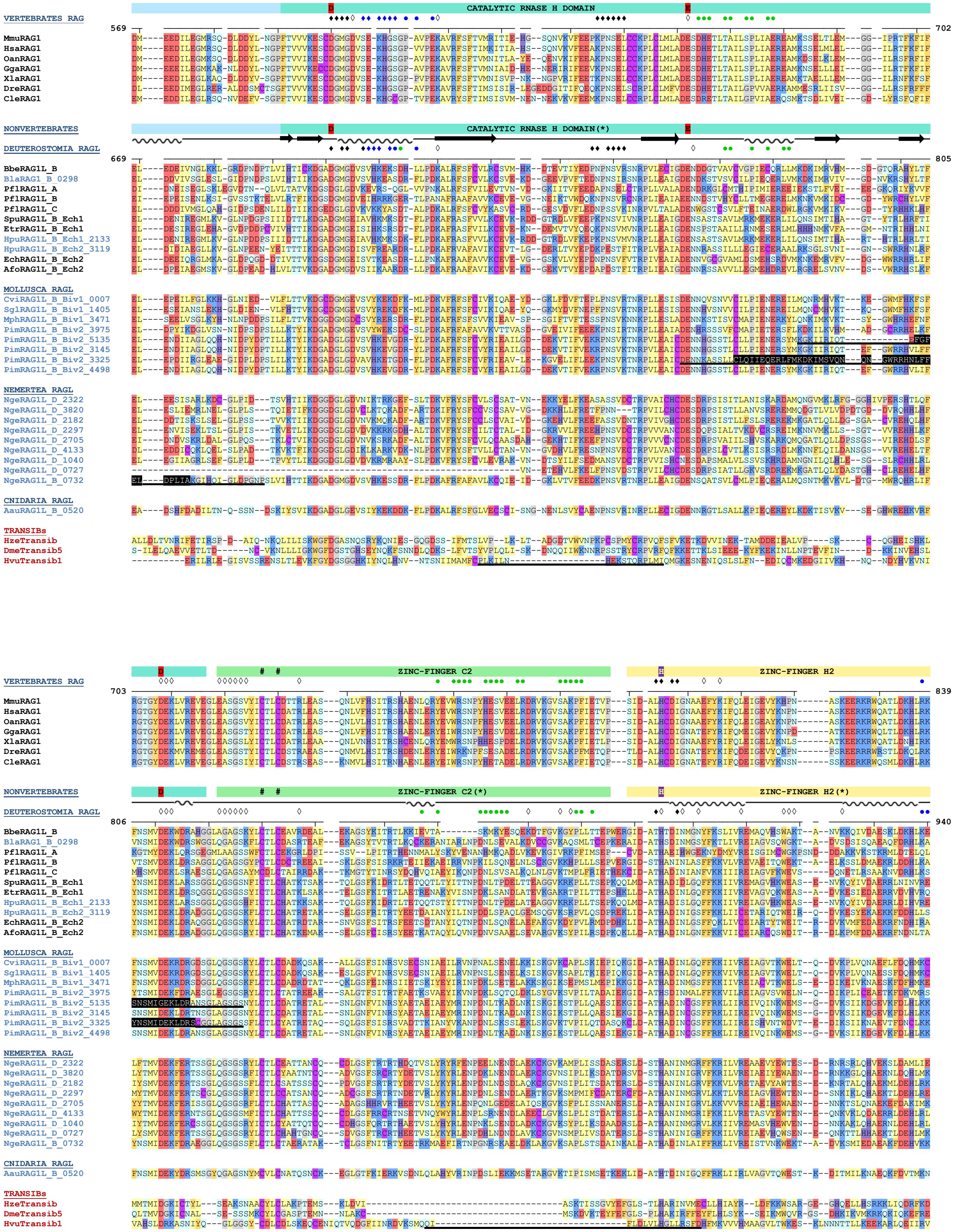

VERTEBRATES RAG
CATALYTIC RNASE H DOMAIN
569
702
MmuRAG1
HsaRAG1
OanRAG1
GgaRAG1
XlaRAG1
DreRAG1
CleRAG1
NONVERTEBRATES
CATALYTIC RNASE H DOMAIN(*)
DEUTEROSTOMIA RAGL
669
805
BbeRAG1L_B
BlaRAG1_B_0298
PflRAG1L_A
PflRAG1L_B
PflRAG1L_C
SpuRAG1L_B_Ech1
EtrRAG1L_B_Ech1
HpuRAG1L_B_Ech1_2133
HpuRAG1L_B_Ech2_3119
EchRAG1L_B_Ech2
AfoRAG1L_B_Ech2
MOLLUSCA RAGL
CviRAG1L_B_Biv1_0007
SglRAG1L_B_Biv1_1405
MphRAG1L_B_Biv1_3471
PimRAG1L_B_Biv2_3975
PimRAG1L_B_Biv2_5135
PimRAG1L_B_Biv2_3145
PimRAG1L_B_Biv2_3325
PimRAG1L_B_Biv2_4498
NEMERTEA RAGL
NgeRAG1L_D_2322
NgeRAG1L_D_3820
NgeRAG1L_D_2182
NgeRAG1L_D_2297
NgeRAG1L_D_2705
NgeRAG1L_D_4133
NgeRAG1L_D_1040
NgeRAG1L_D_0727
NgeRAG1L_B_0732
CNIDARIA RAGL
AauRAG1L_B_0520
TRANSIBs
HzeTransib
DmeTransib5
HvuTransib1
VERTEBRATES RAG
ZINC-FINGER C2
ZINC-FINGER H2
703
839
NONVERTEBRATES
ZINC-FINGER C2(*)
ZINC-FINGER H2(*)
DEUTEROSTOMIA RAGL
806
940

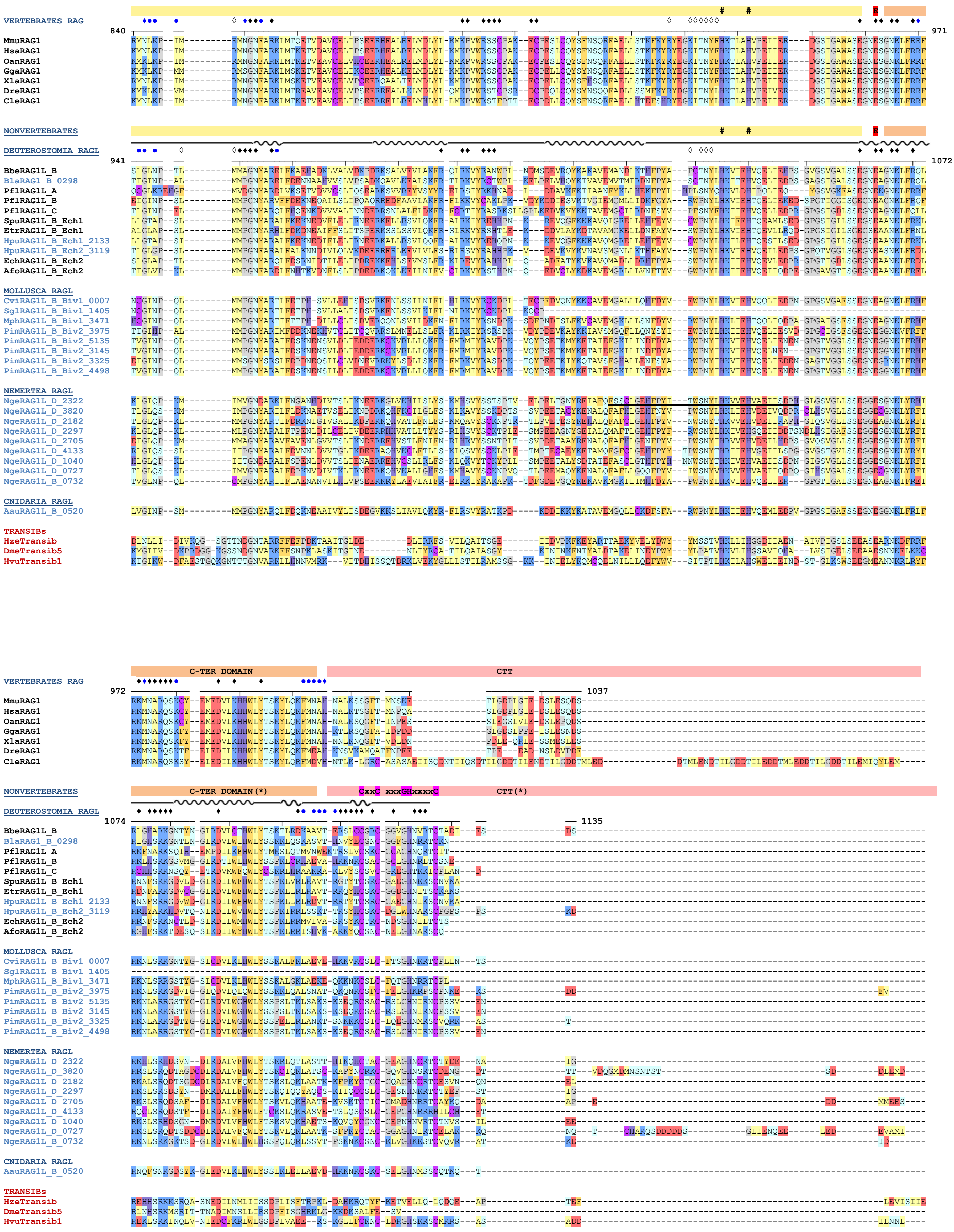

VERTEBRATES RAG
840
971
MmuRAG1
HsaRAG1
OanRAG1
GgaRAG1
XlaRAG1
DreRAG1
CleRAG1
NONVERTEBRATES
DEUTEROSTOMIA RAGL
941
1072
BbeRAG1L_B
BlaRAG1_B_0298
PflRAG1L_A
PflRAG1L_B
PflRAG1L_C
SpuRAG1L_B_Ech1
EtrRAG1L_B_Ech1
HpuRAG1L_B_Ech1_2133
HpuRAG1L_B_Ech2_3119
EchRAG1L_B_Ech2
AfoRAG1L_B_Ech2
MOLLUSCA RAGL
CviRAG1L_B_Biv1_0007
SglRAG1L_B_Biv1_1405
MphRAG1L_B_Biv1_3471
PimRAG1L_B_Biv2_3975
PimRAG1L_B_Biv2_5135
PimRAG1L_B_Biv2_3145
PimRAG1L_B_Biv2_3325
PimRAG1L_B_Biv2_4498
NEMERTEA RAGL
NgeRAG1L_D_2322
NgeRAG1L_D_3820
NgeRAG1L_D_2182
NgeRAG1L_D_2297
NgeRAG1L_D_2705
NgeRAG1L_D_4133
NgeRAG1L_D_1040
NgeRAG1L_D_0727
NgeRAG1L_B_0732
CNIDARIA RAGL
AauRAG1L_B_0520
TRANSIBs
HzeTransib
DmeTransib5
HvuTransib1
C-TER DOMAIN
CTT
VERTEBRATES RAG
972
1037
MmuRAG1
HsaRAG1
OanRAG1
GgaRAG1
XlaRAG1
DreRAG1
CleRAG1
NONVERTEBRATES
C-TER DOMAIN (*)
CxxC xxxGHxxxxC
CTT (*)
DEUTEROSTOMIA RAGL
1074
1135
BbeRAG1L_B
BlaRAG1_B_0298
PflRAG1L_A
PflRAG1L_B
PflRAG1L_C
SpuRAG1L_B_Ech1
EtrRAG1L_B_Ech1
HpuRAG1L_B_Ech1_2133
HpuRAG1L_B_Ech2_3119
EchRAG1L_B_Ech2
AfoRAG1L_B_Ech2
MOLLUSCA RAGL
CviRAG1L_B_Biv1_0007
SglRAG1L_B_Biv1_1405
MphRAG1L_B_Biv1_3471
PimRAG1L_B_Biv2_3975
PimRAG1L_B_Biv2_5135
PimRAG1L_B_Biv2_3145
PimRAG1L_B_Biv2_3325
PimRAG1L_B_Biv2_4498
NEMERTEA RAGL
NgeRAG1L_D_2322
NgeRAG1L_D_3820
NgeRAG1L_D_2182
NgeRAG1L_D_2297
NgeRAG1L_D_2705
NgeRAG1L_D_4133
NgeRAG1L_D_1040
NgeRAG1L_D_0727
NgeRAG1L_B_0732
CNIDARIA RAGL
AauRAG1L_B_0520
TRANSIBs
HzeTransib
DmeTransib5
HvuTransib1

```
                                          1040
VERTEBRATES RAG
MmuRAG1                  ---------MEF-------
HsaRAG1                  ---------MEF-------
OanRAG1                  ---------MEF-------
GgaRAG1                  ---------VEL-------
XlaRAG1                  ---------MEF-------
DreRAG1                  -------------------
CleRAG1                  ---------IQY-LK----

NONVERTEBRATES
DEUTEROSTOMIA RAGL
                                              1136
BbeRAG1L_B               ------------------N
BlaRAG1_B_0298           -------------------
PflRAG1L_A               -------------------
PflRAG1L_B               ------------------S
PflRAG1L_C               ------------------R
SpuRAG1L_B_Ech1          ------------------T
EtrRAG1L_B_Ech1          ------------------E
HpuRAG1L_B_Ech1_2133     ------------------T
HpuRAG1L_B_Ech2_3119     ------------------A
EchRAG1L_B_Ech2          ------------------K
AfoRAG1L_B_Ech2          ------------------R

MOLLUSCA RAGL
CviRAG1L_B_Biv1_0007     ------------------L
SglRAG1L_B_Biv1_1405     -------------------
MphRAG1L_B_Biv1_3471     ------------------L
PimRAG1L_B_Biv2_3975     ------------------E
PimRAG1L_B_Biv2_5135     ------------------V
PimRAG1L_B_Biv2_3145     ------------------V
PimRAG1L_B_Biv2_3325     ------------------H
PimRAG1L_B_Biv2_4498     ------------------V

NEMERTEA RAGL
NgeRAG1L_D_2322          -------------------
NgeRAG1L_D_3820          ---------MYG-DSDLSE
NgeRAG1L_D_2182          -------------------
NgeRAG1L_D_2297          -------------------
NgeRAG1L_D_2705          -------------------
NgeRAG1L_D_4133          -------------------
NgeRAG1L_D_1040          -------------------
NgeRAG1L_D_0727          -------------CVNC-M
NgeRAG1L_B_0732          ------------------K

CNIDARIA RAGL
AauRAG1L_B_0520          ------------------L

TRANSIBs
HzeTransib               PDSEVDSDGDEL-------
DmeTransib5              --YALLEE---G-------
HvuTransib1              -----------FVCK----
```

LEGEND

Sequence alignment markup (shown above the alignment)

♦ RAG1(L) – RSS/TIR contacts
◊ RAG1(L) – RSS/TIR flanking region contacts
● RAG1(L) – RAG2(L) contact interface
● RAG1(L) dimerisation interface
♦ ♦ + ●
Conserved Zn finger motifs in ZnC2(*) & ZnH2(*) domains
* Conserved Zn-finger motifs in ZDD(*) domain

Mapped contacts (within 5Å) and secondary structure were obtaiend from PDB structures: 5ZE1 (M.musculus) and 6B40 (B.belcheri).

Amino acid colorcode

Yellow - hydrophobic aliphatic;
Orange - hydrophobic aromatic;
Blue - positively charged;
Red - negatively charged;
Light blue - neutral polar
Grey - glycine and prolines
Purple - cysteine;
Dark purple - histidine.

Sites at which coding sequences are merged are indicated with underlining.
Regions that could be assigned to the coding region with lower confidence due to conflicting protein translation predictions are highlighted in black.
Sequences identified within this study are shown with a blue font.

RAG1L sequence descriptions

Taxonomic Group	Sequence	Organism	No. CDSs	Refferences
**Vertebrates RAG**	HsaRAG1	*Homo sapiens (Human)*	1 CDS	Uniprot: P15918
	MmuRAG1	*Mus musculus (Mouse)*	1 CDS	Uniprot: P15919
	OanRAG1	*Ornithorhynchus anatinus (Duckbill platypus)*	1 CDS	Uniprot: A7KS66
	GgaRAG1	*Gallus gallus (Chicken)*	1 CDS	Uniprot: P24271
	XlaRAG1	*Xenopus laevis (African clawed frog)*	1 CDS	Uniprot: Q91829
	DreRAG1	*Danio rerio (Zebrafish)*	1 CDS	Uniprot: O13033
	CleRAG1	*Carcharhinus leucas (Bull shark)*	1 CDS	Uniprot: Q90381
**Deuterostomia Invertebrates RAGL**	BbeRAG1L_B	*Branchiostoma belcheri (Amphioxus)*	4 CDS	NBCI: KJ748699.1 (Huang,et al , 2016)
	PflRAG1L_A	*Ptychodera flava (Acorn worm)*	-	TSA: GDGM01063948.1 (Morales Poole et al , 2017)
	PflRAG1L_B	*Ptychodera flava (Acorn worm)*	-	TSA: GDGM01438088.1 (Morales Poole et al , 2017)
	PflRAG1L_C	*Ptychodera flava (Acorn worm)*	1 CDS	WGS: BCFJ01036631.1 (Morales Poole et al , 2017)
	SpuRAG1L_B_Ech1	*Strongylocentrotus purpuratus (Purple sea urchin)*	4 CDS	Uniprot : Q45ZT6
	EtrRAG1L_B_Ech1	*Eucidaris tribuloides (Slate pencil urchin)*	3 CDS	WGS: JZLH010201023.1 (Morales Poole et al , 2017)
	EchRAG1L_B_Ech2	*Evechinus chloroticus (Kina sea urchin)*	-	TSA: GAPB01003278.1 (Morales Poole et al , 2017)
	AfoRAG1L_B_Ech2	*Asterias forbesi (Forbes sea star)*	-	TSA: GAUS01036390.1 (Morales Poole et al , 2017)
	BlaRAG1L_B_0298	*Branchiostoma lanceolatum (Amphioxus)*	1 CDS	WGS: FLLO01000298.1
	HpuRAG1L_B_Ech1_2133	*Hemicentrotus pulcherrimus (sea urchin)*	3 CDS	WGS: BEXV01002133.1
	HpuRAG1L_B_Ech2_3119	*Hemicentrotus pulcherrimus (sea urchin)*	3 CDS	WGS: BEXV01003119.1
**Protostomia Mollusca RAGL**	CviRAG1L_B_Biv1_0007	*Crassostrea virginica (Eastern oyster)*	1 CDS	WGS: MWPT03000007.1
	SglRAG1L_B_Biv1_1405	*Saccostrea glomerata (Sydney rock oyster)*	1 CDS	WGS: PRKT01001405.1
	MphRAG1L_B_Biv1_3471	*Modiolus philippinarum (Philippine horse mussel)*	1 CDS	WGS: MJUU01063471.1
	PimRAG1L_B_Biv2_3975	*Pinctada imbricata (Akoya pearl oyster)*	1 CDS	WGS: NIJJ01013975.1
	PimRAG1L_B_Biv2_4498	*Pinctada imbricata (Akoya pearl oyster)*	1 CDS	WGS: NIJJ01024498.1
	PimRAG1L_B_Biv2_3145	*Pinctada imbricata (Akoya pearl oyster)*	1 CDS	WGS: NIJJ01023145.1
	PimRAG1L_B_Biv2_3325	*Pinctada imbricata (Akoya pearl oyster)*	2 CDS	WGS: NIJJ01003325.1
	PimRAG1L_B_Biv2_5135	*Pinctada imbricata (Akoya pearl oyster)*	2 CDS	WGS: NIJJ01005135.1
**Protostomia Nemertea RAGL**	NgeRAG1L_D_2322	*Notospermus geniculatus (Ribbon worms)*	2 CDS	WGS: NMRB01002322.1
	NgeRAG1L_D_1040	*Notospermus geniculatus (Ribbon worms)*	1 CDS	WGS: NMRB01001040.1
	NgeRAG1L_D_4133	*Notospermus geniculatus (Ribbon worms)*	1 CDS	WGS: NMRB01004133.1
	NgeRAG1L_D_3820	*Notospermus geniculatus (Ribbon worms)*	1 CDS	WGS: NMRB01003820.1
	NgeRAG1L_D_2297	*Notospermus geniculatus (Ribbon worms)*	1 CDS	WGS: NMRB01002297.1
	NgeRAG1L_D_2182	*Notospermus geniculatus (Ribbon worms)*	2 CDS	WGS: NMRB01002182.1
	NgeRAG1L_D_2705	*Notospermus geniculatus (Ribbon worms)*	2 CDS	WGS: NMRB01002705.1
	NgeRAG1L_B_0732	*Notospermus geniculatus (Ribbon worms)*	3 CDS	WGS: NMRB01000732.1
	NgeRAG1L_D_0727	*Notospermus geniculatus (Ribbon worms)*	1 CDS	WGS: NMRB01000727.1
**Cnidaria RAGL**	AauRAG1L_0520	*Aurelia aurita (Moon jellyfish)*	2 CDS	WGS: REGM01000520.1
**TRANSIB**	HzeTransib	*Helicoverpa zea (Corn earworm moth)*	1 CDS	Uniprot: B0F0C5
	DmeTransib5	*Drosophila melanogaster (fruit fly)*	1 CDS	RepBase: Transib5 translation (Kapitonov et al 2005)
	HvuTransib1	*Hydra vulgaris (hydra)*	4 CDS	RepBase: Transib-1_HM translation (Kapitonov et al 2008)

(b) RAG2/RAG2L Multiple Sequence Alignment

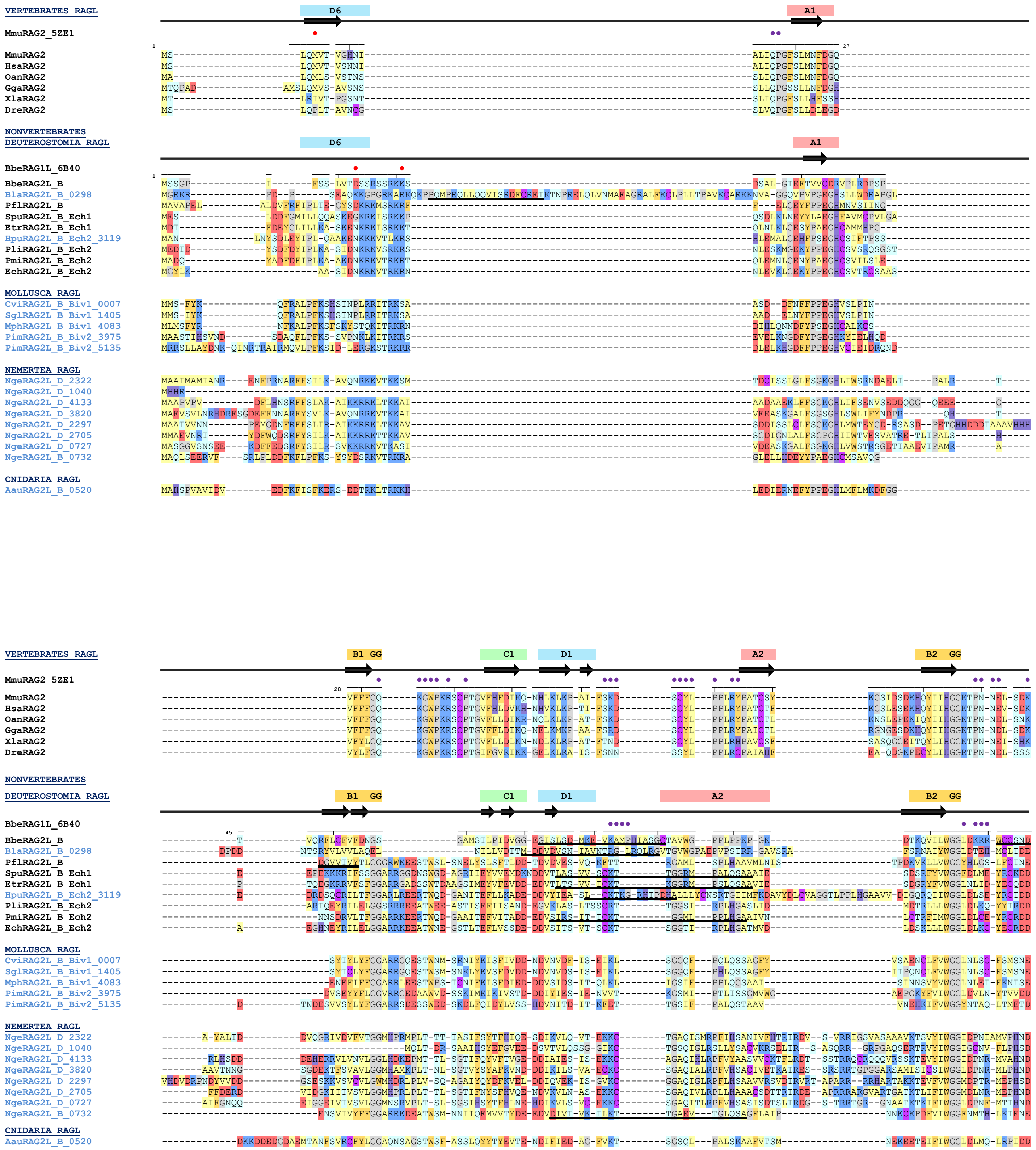

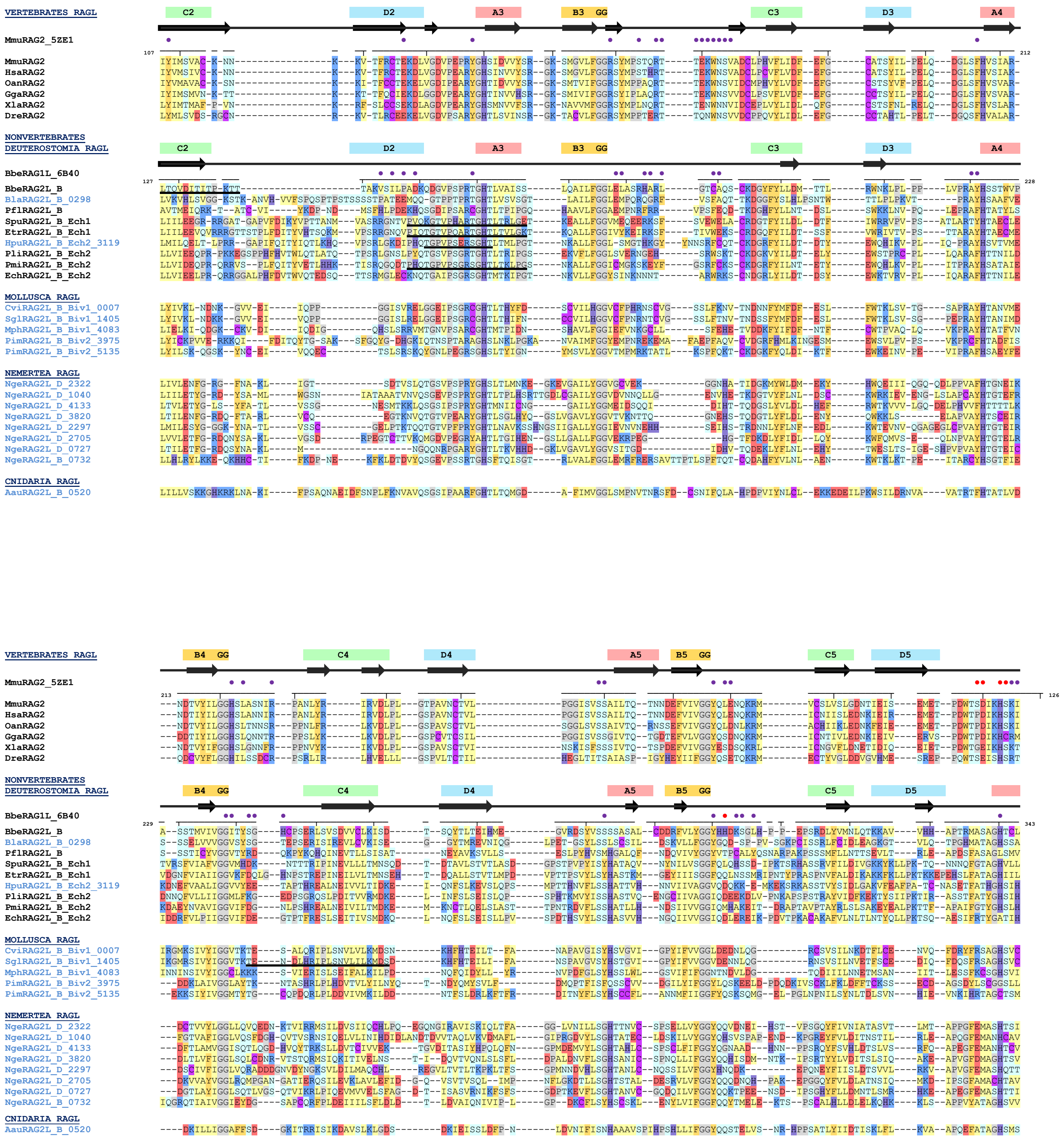

VERTEBRATES RAGL
C2
D2
A3
B3 GG
C3
D3
A4
MmuRAG2_5ZE1
107
212
MmuRAG2
HsaRAG2
OanRAG2
GgaRAG2
XlaRAG2
DreRAG2
NONVERTEBRATES
DEUTEROSTOMIA RAGL
BbeRAG1L_6B40
127
228
BbeRAG2L_B
BlaRAG2L_B_0298
PflRAG2L_B
SpuRAG2L_B_Ech1
EtrRAG2L_B_Ech1
HpuRAG2L_B_Ech2_3119
PliRAG2L_B_Ech2
PmiRAG2L_B_Ech2
EchRAG2L_B_Ech2
MOLLUSCA RAGL
CviRAG2L_B_Biv1_0007
SglRAG2L_B_Biv1_1405
MphRAG2L_B_Biv1_4083
PimRAG2L_B_Biv2_3975
PimRAG2L_B_Biv2_5135
NEMERTEA RAGL
NgeRAG2L_D_2322
NgeRAG2L_D_1040
NgeRAG2L_D_4133
NgeRAG2L_D_3820
NgeRAG2L_D_2297
NgeRAG2L_D_2705
NgeRAG2L_D_0727
NgeRAG2L_B_0732
CNIDARIA RAGL
AauRAG2L_B_0520
VERTEBRATES RAGL
B4 GG
C4
D4
A5
B5 GG
C5
D5
MmuRAG2_5ZE1
213
126
NONVERTEBRATES
DEUTEROSTOMIA RAGL
BbeRAG1L_6B40
229
343
MOLLUSCA RAGL
NEMERTEA RAGL
CNIDARIA RAGL

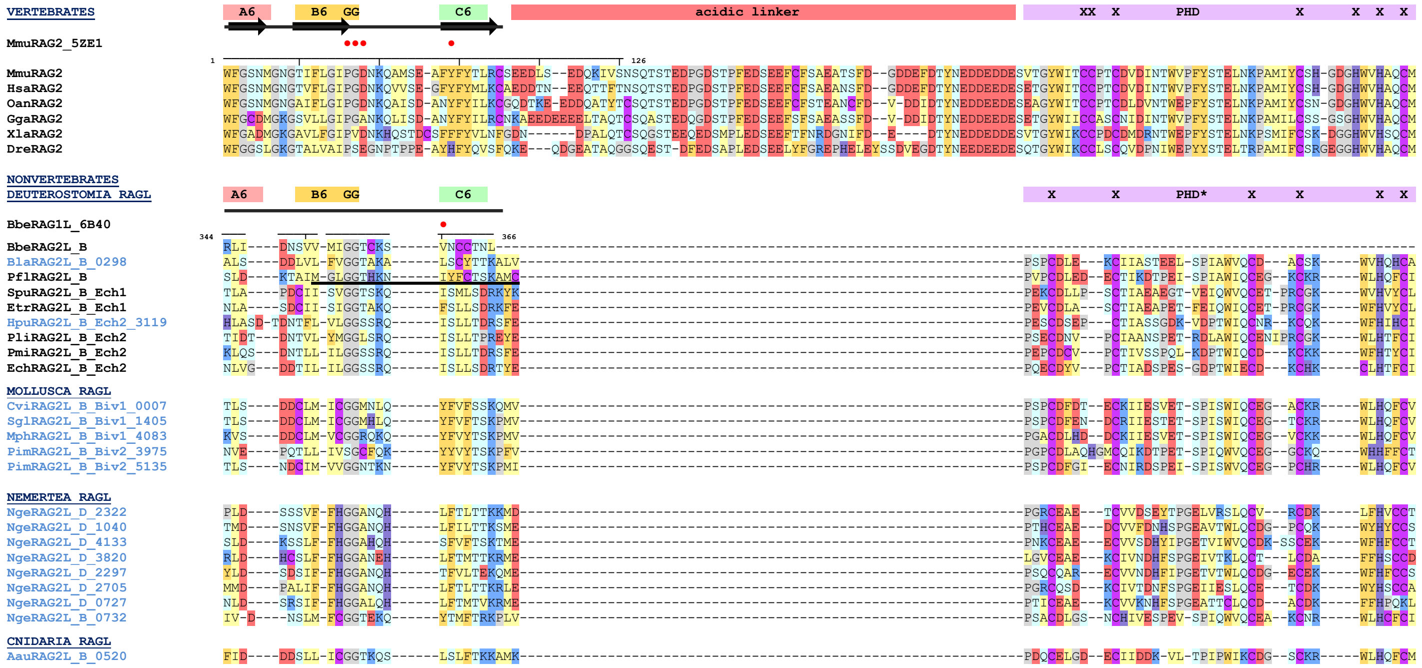
VERTEBRATES
A6
B6 GG
C6
acidic linker
XX X PHD X X X X
MmuRAG2_5ZE1
MmuRAG2
HsaRAG2
OanRAG2
GgaRAG2
XlaRAG2
DreRAG2
NONVERTEBRATES
DEUTEROSTOMIA RAGL
X X PHD* X X X X
BbeRAG1L_6B40
BbeRAG2L_B
BlaRAG2L_B_0298
PflRAG2L_B
SpuRAG2L_B_Ech1
EtrRAG2L_B_Ech1
HpuRAG2L_B_Ech2_3119
PliRAG2L_B_Ech2
PmiRAG2L_B_Ech2
EchRAG2L_B_Ech2
MOLLUSCA RAGL
CviRAG2L_B_Biv1_0007
SglRAG2L_B_Biv1_1405
MphRAG2L_B_Biv1_4083
PimRAG2L_B_Biv2_3975
PimRAG2L_B_Biv2_5135
NEMERTEA RAGL
NgeRAG2L_D_2322
NgeRAG2L_D_1040
NgeRAG2L_D_4133
NgeRAG2L_D_3820
NgeRAG2L_D_2297
NgeRAG2L_D_2705
NgeRAG2L_D_0727
NgeRAG2L_B_0732
CNIDARIA RAGL
AauRAG2L_B_0520

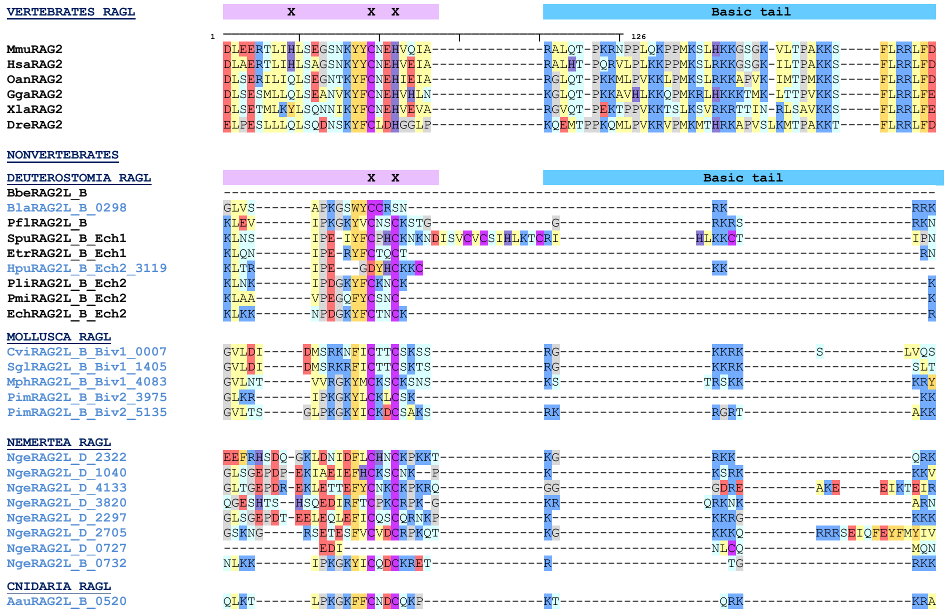
VERTEBRATES RAGL
X X X
Basic tail
MmuRAG2
HsaRAG2
OanRAG2
GgaRAG2
XlaRAG2
DreRAG2
NONVERTEBRATES
DEUTEROSTOMIA RAGL
BbeRAG2L_B
BlaRAG2L_B_0298
PflRAG2L_B
SpuRAG2L_B_Ech1
EtrRAG2L_B_Ech1
HpuRAG2L_B_Ech2_3119
PliRAG2L_B_Ech2
PmiRAG2L_B_Ech2
EchRAG2L_B_Ech2
MOLLUSCA RAGL
CviRAG2L_B_Biv1_0007
SglRAG2L_B_Biv1_1405
MphRAG2L_B_Biv1_4083
PimRAG2L_B_Biv2_3975
PimRAG2L_B_Biv2_5135
NEMERTEA RAGL
NgeRAG2L_D_2322
NgeRAG2L_D_1040
NgeRAG2L_D_4133
NgeRAG2L_D_3820
NgeRAG2L_D_2297
NgeRAG2L_D_2705
NgeRAG2L_D_0727
NgeRAG2L_B_0732
CNIDARIA RAGL
AauRAG2L_B_0520

LEGEND

Sequence alignment markup (shown above the alignment)

- ● RAG2(L)-RAG1(L) contact, same heterodimer
- ● RAG2(L)-RAG1(L) contact, opposite heterodimer
- **X** Zn finger motif within PHD

Mapped contacts (within 5Å) and secondary structure were obtaiend from PDB structures: 5ZE1 (M.musculus) and 6B40 (B.belcheri).

Amino acid colorcode

Yellow - hydrophobic aliphatic;
Orange - hydrophobic aromatic;
Blue - positively charged;
Red - negatively charged;
Light blue - neutral polar
Grey - glycine and prolines
Purple - cysteine;
Dark purple - histidine.

Sites at which coding sequences are merged are indicated with underlining
Sequences identified within this study are shown with a blue font.

RAG2L sequence descriptions

Taxonomic Group	Sequence	Organism	CDSs	Refferences
**Deuterostomia Vertebrates RAG**	HsaRAG2	*Homo sapiens (Human)*	1 CDS	Uniprot: P55895
	MmuRAG2	*Mus musculus (Mouse)*	1 CDS	Uniprot: P21784
	OanRAG2	*Ornithorhynchus anatinus (Duckbill platypus)*	1 CDS	Uniprot: F6S5J9
	GgaRAG2	*Gallus gallus (Chicken)*	1 CDS	Uniprot: P25022
	XlaRAG2	*Xenopus laevis (African clawed frog)*	1 CDS	Uniprot: Q91830
	DreRAG2	*Danio rerio (Zebrafish)*	1 CDS	Uniprot: O13034
**Deuterostomia Invertebrates RAGL**	BbeRAG2L_B	*Branchiostoma belcheri (Amphioxus)*	3 CDS	KJ748699.1 (Huang,et al , 2016)
	PflRAG2L_B	*Ptychodera flava (Acorn worm)*	3 CDS	WGS: BCFJ01052781.1 (Morales Poole et al , 2017)
	SpuRAG2L_B_Ech1	*Strongylocentrotus purpuratus (Purple sea urchin)*	3 CDS	Uniprot: Q45ZT5
	EtrRAG2L_B_Ech1	*Eucidaris tribuloides (Slate pencil urchin)*	3 CDS	WGS: JZLH010201023.1 (Morales Poole et al , 2017)
	PliRAG2L_B_Ech2	*Paracentrotus lividus (Common sea urchin)*	-	TSA: GFRN01302616.1 (Morales Poole et al , 2017)
	PmiRAG2L_B_Ech2	*Patiria miniata (Bat star)*	3 CDS	WGS: AKZP01064647.1 (Morales Poole et al , 2017)
	EchRAG2L_B_Ech2	*Evechinus chloroticus (Kina sea urchin)*	-	TSA: GAPB01022632.1 (Morales Poole et al , 2017)
	BlaRAG2L_B_0298	*Branchiostoma lanceolatum (Amphioxus)*	3 CDS	WGS: FLLO01000298.1
	HpuRAG2L_B_Ech2_3119	*Hemicentrotus pulcherrimus (sea urchin)*	3 CDS	WGS: BEXV01003119.1
**Protostomia Bivalvia RAGL**	CviRAG2L_B_Biv1_0007	*Crassostrea virginica (Eastern oyster)*	1 CDS	WGS: MWPT03000007.1
	SglRAG2L_B_Biv1_1405	*Saccostrea glomerata (Sydney rock oyster)*	2 CDS	WGS: PRKT01001405.1
	MphRAG2L_B_Biv1_4083	*Modiolus philippinarum (Philippine horse mussel)*	1 CDS	WGS: MJUU01034083.1
	PimRAG2L_B_Biv2_3975	*Pinctada imbricata (Akoya pearl oyster)*	1 CDS	WGS: NIJJ01013975.1
	PimRAG2L_B_Biv2_5135	*Pinctada imbricata (Akoya pearl oyster)*	1 CDS	WGS: NIJJ01005135.1
**Protostomia Nemertea RAGL**	NgeRAG2L_D_2322	*Notospermus geniculatus (Ribbon worms)*	1 CDS	WGS: NMRB01002322.1
	NgeRAG2L_D_1040	*Notospermus geniculatus (Ribbon worms)*	1 CDS	WGS: NMRB01001040.1
	NgeRAG2L_D_4133	*Notospermus geniculatus (Ribbon worms)*	1 CDS	WGS: NMRB01004133.1
	NgeRAG2L_D_3820	*Notospermus geniculatus (Ribbon worms)*	1 CDS	WGS: NMRB01003820.1
	NgeRAG2L_D_2297	*Notospermus geniculatus (Ribbon worms)*	1 CDS	WGS: NMRB01002297.1
	NgeRAG2L_D_2705	*Notospermus geniculatus (Ribbon worms)*	1 CDS	WGS: NMRB01002705.1
	NgeRAG2L_D_0727	*Notospermus geniculatus (Ribbon worms)*	1 CDS	WGS: NMRB01000732.1
	NgeRAG2L_B_0732	*Notospermus geniculatus (Ribbon worms)*	2 CDS	WGS: NMRB01000727.1
**Cnidaria RAGL**	AauRAG2L_B_0520	*Aurelia aurita (Moon jellyfish)*	1 CDS	WGS: REGM01000520.1
