# Supplementary material for: Identification of RAG-like transposons in protostomes suggests their ancient bilaterian origin
Source: Mob DNA. 2020 May 6;11:17. doi: 10.1186/s13100-020-00214-y (PMC7204232; doi:10.1186/s13100-020-00214-y)
Supplement: Supplementary file 8 — Additional file 8: File S1. Detailed presentation of the sequence information about the new RAGL loci identified in this study: detection relevant data (e-values and query-target alignments from TBLASTN), TIR and TSD detection information, predicted protein sequences, and additional relevant observations regarding some of the sequences. [file 13100_2020_214_MOESM8_ESM.pdf]

File S1

Detected RAG1/2 -like loci summary

PROTOSTOMIA - Mollusca……… 2
Crassostrea viriginica……… 2
Crassostrea gigas……… 16
Saccostrea glomerata……… 19
Modiolus philippinarum ……… 24
Bathymodiolus platifrons……… 28
Pinctada imbricata……… 30

PROTOSTOMIA - Nemertea ……… 59
Notospermus geniculatus……… 59

CNIDARIA……… 98
Aurelia aurita……… 98
Porites Rus……… 104
Orbicella faveolata……… 107
Nematostella vectensis……… 109
Pocillopora damicornis……… 110

DEUTEROSTOMIA ……… 112
Branchiostoma lancelatum……… 112
Hemicentrotus pulcherrimus……… 116

PROTOSTOMIA - MOLLUSCA

Crassostrea Virginica (Cvi)

(TaxID: 6565; Protostomia; Mollusca; Bivalvia; Ostreoida)

WGS: MWPT03000007.1

1. Summary

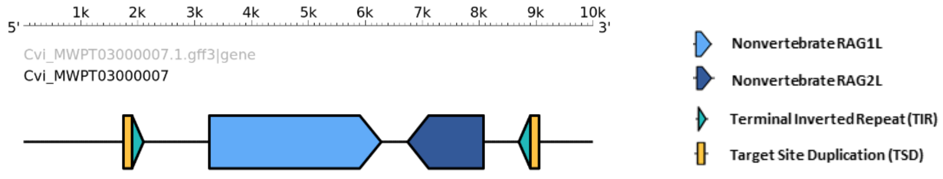

Detected configuration: TSD – TIR5 - RAG1L - RAG2L – TIR3 - TSD

Detected loci	Predicted CDS	start	end	strand	Observations
RAG1L	1	10207215	10210232	+	complete; no stop codons
RAG2L	1	10212035	10210701	-	complete; no stop codons

Predicted TIR	Distance from CDS (bp)	start	strand	TSD sequence	Assessment method
TIR5' (RAG1L side)	1334	10205881	+	5'-GGGCGcgg-TIR	Homology variation
TIR3' (RAG2L side)	688	10212723	-	TIR-GGGCGgcc-3'	Homology variation

```
CviTIR5'_0007                          5'----ATCAAACTTC-----------CCGTCTCCTGCTCAG 3'
                                             ||||||||||           |||||||||||| ||
CviTIR3'_0007 (reverse complement)     3'CACTATCAAACTTCCGTTATATAAGCCGTCTCCTGCTAAG 5'
```

Selected predicted protein product

CviRAG1L_B_Biv1_0007

```
MAAYINTNCLHCLEIMQMTKNIQNFILSTDMEHGLKLKCMCRLCGVKIPINSKSHPKENFKSAI
TSLYNIDIDCDSENIHPPKICTACRLRLTRHSQAEDDIEINLPVLFEFLPHSINCNICFVKPGR
PKKILKRKLSVPSQSTKNIFIQQEHSYHSANKDSQDSSRPSTETRDITSPESPSSFTSFSSIVV
PSASTSCSFCPPVDSPPLPPVDKLSVVESCSSETAPEMFKNVVIRSIPTERFCNAEVSQSFMCT
ICRGVPCDPYISKCSHIFCKECIFGWFSLSSACPVCRSLLDESEVSPLHGHLLQIYETLLVHCI
HANCTQSHLIRNIDEHESICSMKGTKFLYNVTTKSRVFKLPLHSVSAKHTRHRRLKPIISQVNE
FCNAQEENKSDVLFFMLKDHLKEINDPRWKQVESLWLGNNSTLSPEQCLALRVDLLQSKGQYRS
QYDFLSQNNVHVFQAPSKMESCENLFMPSASIFQIIDNDGNVLLQNSENPCTEPLNVNECFLPG
FVELATPNCMGVRFSYFEALSLTLQELEPEILFGLKKHGLNIEDVLFLTTVKDGCDGMGEVSVY
KEKDFKMLPDKVFRFSFCIVKIQAEYDGKLFDVFTEPLPNSVRTNRPLLESISDENNQVSNVVC
ILPIENEREILMQNRMHVKTKEGWMFHKFSFFNSMVDEKRDRGDSGLQGSGSKYLCTLCDADKQ
SAKALLGSFSINRSVSECSNIAEILRVNPNALSENELKKISKGVKCAPLSKIEPIQKGIDATHA
DINLGQFFKKIIVREIAGVTKWELTQDVKPLVQNAEFLFDQHMKCNCGINPQLMMPGNYARTLF
ETPHSVLLEHISDSVRKENLSSILNIFLHLRKVYRCKDPLTECPFDVQNYKKCAVEMGALLLQH
FDYVEWPNYLHKVIEHVQQLIEDPNGPGSVGAFSSEGNEAGNKLFRHFRKNLSRRGNTYGSLCD
VLKLHWLYSSKALFKLAEVEHKKVRCSLCFTSGHNKRTCPLLNTSL
```

Prediction performed with FGENESH (*C.elegans* dataset)

CviRAG2L_B_Biv1_0007

```
MMSFYKQFRALPFKSHSTNPLRRITRKSAASDDFNFFPPEGHVSLPINSYTYLYFGGARRGQES
TWNMSRNIYKISFIVDDNDVNVDFISEIKLSGGQFPQLQSSAGFYVSAENCLFVWGGLNLSCFS
MSNELYIVKLNDNKGVVEIIQPPGGISVRELGGEIPSGRCGHTLTHYFDSCVILHGGVCFPHRN
SCVGSSLFKNVTNDNNFYMFDFESLFWTKLSVTGSAPRAYHTANVMEIRGMKSIVYIGGVTKTE
SALQRIPLSNVLVLKMDSNKHFHTEILTFANAPAVGISYHSVGVIGPYIFVVGGLDEDNLQGRC
SVSILNKDTFLCENVQFDRYFRSAGHSVCTLSDDCLMICGGMNLQYFVFSSKQMVPSPCDFDTE
CKIIESVETSPISWIQCEGACKRWLHQFCVGVLDIDMSRKNFICTTCSKSSRGKKRKSLVQS
```

Prediction performed with FGENESH (*C.elegans* dataset)

2. RAGL Detection info

Method: TBLASTN
Database searched: Whole-genome shotgun contigs (WGS) + only in Mollusca group (taxid: 6447)

2.1. RAG1L

Query: PflRAG1L_B from P.flava (Morales Poole et al, 2017)
Subject: MWPT03000007.1 Length: 59691872 Number of Matches: 1

Score	Expect	Method	Identities	Positives	Gaps	Frame
632 bits(1630)	0.0	Compositional matrix adjust.	332/783(42%)	468/783(59%)	25/783(3%)	+3

```
Query  193       SIPLDRFIEKDIAEHYVCSICQGVPTTPCISPCSHIFCVGCIQQWLANSCACPSCREILE  252
                 SIP +RF   ++++ ++C+IC+GVP  P IS CSHIFC  CI  W + S ACP CR +L+
Sbjct  10207926  SIPTERFCNAEVSQSFMCTICRGVPCDPYISKCSHIFCKECIFGWFSLSSACPVCRSLLD  10208105

Query  253       CDDCQNLTGNHLNIYDSLRLRCTYSHLGCETMTTLPNYIDHELTCKYKAKGKRSTYG---  309
                   +   L G+ L IY++L + C   H  C     + N  +HE  C    KG +  Y
Sbjct  10208106  ESEVSPLHGHLLQIYETLLVHCI--HANCTQSHLIRNIDEHESICS--MKGTKFLYNVTT  10208273

Query  310       KTRV-KQSLRTADRQYCKQKRLKACYDFLRDFCTANSESTEDVLFFLLRSYLYDSGDRER  368
                 K+RV K  L +   ++ + +RLK     + +FC A  E+  DVLFF+L+ +L +  D  R
Sbjct  10208274  KSRVFKLPLHSVSAKHTRHRRLKPIISQVNEFCNAQEENKSDVLFFMLKDHLKEIND-PR  10208450

Query  369       SNLVDDLWSKTQSKMSADECLALRIDNLQTKNRYKAQYDMFKSKSVSVLVAPNQLDMLER  428
                    V+ LW    S +S ++CLALR+D LQ+K +Y++QYD     +V V  AP++++  E
Sbjct  10208451  WKQVESLWLGNNSTLSPEQCLALRVDLLQSKGQYRSQYDFLSQNNVHVFQAPSKMESCEN  10208630

Query  429       TYMPGTARYAIVGEENFEHIYQTPVKLHRKDLSISTTADSVEPIELNSEYPNDNKEFPGP  488
                  +MP  + + I+  +                L  ++     EP+ +N  +     E   P
Sbjct  10208631  LFMPSASIFQIIDNDG-------------NVLLQNSENPCTEPLNVNECFLPGFVELATP  10208771

Query  489       NLAGVRFRYTDAVAKTLEEIEPEISENLKSIGVSSTKTELVLRTFIKDGSDGMGDVEIHR  548
                 N  GVRF Y +A++ TL+E+EPEI   LK  G++    +++  T +KDG DGMG+V +++
Sbjct  10208772  NCMGVRFSYFEALSLTLQELEPEILFGLKKHGLNIE--DVLFLTTVKDGCDGMGEVSVYK  10208945

Query  549       RKGERTLPANAFRAAFAVVKCEVEVGNEIKTVWDQKNPNSVRCNRPLIEAIAEENNDSTV  608
                  K  + LP   FR +F +VK + E   ++  V+ +  PNSVR NRPL+E+I++ENN  +
Sbjct  10208946  EKDFKMLPDKVFRFSFCIVKIQAEYDGKLFDVFTEPLPNSVRTNRPLLESISDENNQVSN  10209125
```

```
Query  609       HYCLLTMEGERELMKNKVMKIDCGDYWRCHYLVFVTSMVDEKLDRSAGGLQGAGSGYPCT  668
                   C+L +E ERE++    M +   + W  H   F  SMVDEK DR   GLQG+GS Y CT
Sbjct  10209126  VVCILPIENEREILMQNRMHVKTKEGWMFHKFSFFNSMVDEKRDRGDSGLQGSGSKYLCT  10209305

Query  669       LCDCTREEAISKLGSFSISRKRTEIIEKAEIRRVNPKILSQNELNLSCKGVKKHPLLLSE  728
                 LCD  ++ A + LGSFSI+R  +E    AEI RVNP  LS+NEL    KGVK  PL   E
Sbjct  10209306  LCDADKQSAKALLGSFSINRSVSECSNIAEILRVNPNALSENELKKISKGVKCAPLSKIE  10209485

Query  729       PVERGIDSTHANINLASFFKKVLVREVAEITQWEKTAELKSSLDMAEKRLDDHLKAEIGI  788
                 P+++GID+THA+INL  FFKK++VRE+A +T+WE T ++K  +  AE   D H+K   GI
Sbjct  10209486  PIQKGIDATHADINLGQFFKKIIVREIAGVTKWELTQDVKPLVQNAEFLFDQHMKCNCGI  10209665

Query  789       NPSLMMPGNYARVFFDEKNEQAILSLIPQAQRREDFAAVLAKFRFLKKVYCAKLPKVDYK  848
                 NP LMMPGNYAR  F E     +L  I  + R+E+ +++L  F  L+KVY  K P  +
Sbjct  10209666  NPQLMMPGNYARTLF-ETPHSVLLEHISDSVRKENLSSILNIFLHLRKVYRCKDPLTECP  10209842

Query  849       DDIESVKTVGIEMGMLLIDKFGYARWPNYLHKVIEHTQELIEKEDSPGTIGGISGEGNEA  908
                  D+++ K   +EMG LL+  F Y  WPNYLHKVIEH Q+LIE  + PG++G  S EGNEA
Sbjct  10209843  FDVQNYKKCAVEMGALLLQHFDYVEWPNYLHKVIEHVQQLIEDPNGPGSVGAFSSEGNEA  10210022

Query  909       GNKLFRQFRKLHSRKGSVMGGLRDTIWLHWLYSSPKLCRHAEVAHRKNRCSACGCLGHNR  968
                 GNKLFR FRK  SR+G+  G L D + LHWLYSS  L + AEV H+K RCS C   GHN+
Sbjct  10210023  GNKLFRHFRKNLSRRGNTYGSLCDVLKLHWLYSSKALFKLAEVEHKKVRCSLCFTSGHNK  10210202

Query  969       LTC  971
                  TC
Sbjct  10210203  RTC  10210211
```

2.2 RAG2L

Query: **PflRAG2L_B from P.flava (Morales Poole et al, 2017)**
Subject: **MWPT03000007.1** **Length: 59691872** **Number of Matches: 1**

Score	Expect	Method	Identities	Positives	Gaps	Frame
226 bits(576)	2e-62	Compositional matrix adjust.	143/455(31%)	231/455(50%)	31/455(6%)	-1

```
Query  12        FRFIPLTEGYSDK-RKMSRKRFF-ELGEYFPPEGHMNVSIINGDGVVTVYTLGGGRWKEE  69
                 FR +P     ++  R+++RK    +   +FPPEGH+++ I       T    GG R  +E
Sbjct  10212014  FRALPFKSHSTNPLRRITRKSAASDDFNFFPPEGHVSLPI----NSYTYLYFGGARRGQE  10211847

Query  70        STWSLSNELYSLSFTLDDTDVDVESVQKFTTRGAMLSPLHAAVMLNISTPDKVKLLVWGG  129
                 STW++S  +Y +SF +DD DV+V+ + +    G     L ++    +S  +   L VWGG
Sbjct  10211846  STWNMSRNIYKISFIVDDNDVNVDFISEIKLSGGQFPQLQSSAGFYVSAENC--LFVWGG  10211673

Query  130       YHLGSLFCTNEAVTMEIQRKTATCVIYKDPNDMSFHLPDEKHQSGDIPSARCGHTLTPIP  189
                  +L     +NE   +++        I + P  +S      +   G+IPS RCGHTLT
Sbjct  10211672  LNLSCFSMSNELYIVKLNDNKGVVEIIQPPGGISV-----RELGGEIPSGRCGHTLTHYF  10211508

Query  190       GQHAAVLFGGAEMPNRFRRVPS--FEQDTKDGHFYLLNTDSLSWKKLNVPQLEPRAFHTA  247
                      +L GG   P+R   V S  F+  T D +FY+ + +SL W KL+V    PRA+HTA
Sbjct  10211507  -DSCVILHGGVCFPHRNSCVGSSLFKNVTNDNNFYMFDFESLFWTKLSVTGSAPRAYHTA  10211331

Query  248       TYLS--SSSTICYVGGVTYRDQKPYKQHQINEVTLLSISATNEYAVKSVLLSESLPYHVS  305
                   +      +I Y+GGVT + +   ++  ++ V +L + +   +  + +  + +    +S
Sbjct  10211330  NVMEIRGMKSIVYIGGVT-KTESALQRIPLSNVLVLKMDSNKHFHTEILTFANAPAVGIS  10211154
```

```
Query  306        MHGALQFNDQVIVYGGYVTPCALYQSNARPAKPSSSMFLLNTTSEVLTRLEAPDSFASAG  365
                   H         + V GG       L + N     +    S+ +LN  + +    ++     F SAG
Sbjct  10211153   YHSVGVIGPYIFVVGG------LDEDNL---QGRCSVSILNKDTFLCENVQFDRYFRSAG  10211001

Query  366        LSMVSLDKTAIMGLGGTHKNIYFCTSKAMCPVPCDLEDECTIKDTPEISPIAWIQCEGKC  425
                   S+ +L     +M   GG +    +   +SK M P PCD + EC I ++ E SPI+WIQCEG C
Sbjct  10211000   HSVCTLSDDCLMICGGMNLQYFVFSSKQMVPSPCDFDTECKIIESVETSPISWIQCEGAC  10210821

Query  426        KRWLHQFCIKLEVI--PKGKYVCNSC-KSTGGRKR  457
                  KRWLHQFC+ +   I    +   ++C +C KS+ G+KR
Sbjct  10210820   KRWLHQFCVGVLDIDMSRKNFICTTCSKSSRGKKR  10210716
```

3. TIR Detection info

Aligned C.virginica RAG1L-RAG2L flanking regions had been searched for homology variations both at left and right flanking regions. Detected homology droping regions were inspected for TIR and TSD presence. For more details please see methods section.

A. RAG1L facing margins alignment (left)

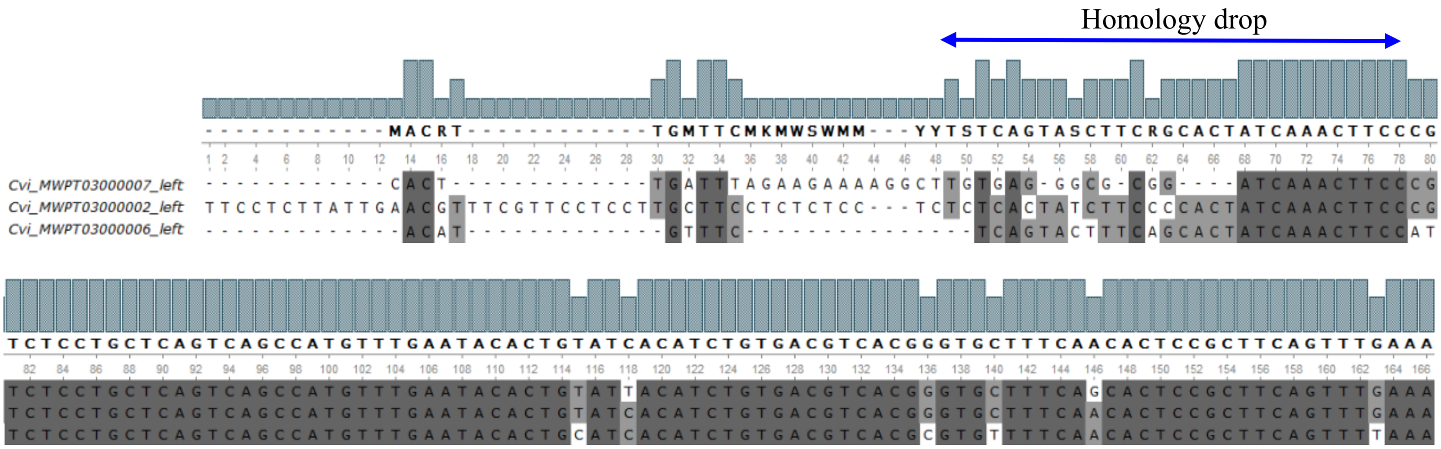

B. RAG2L facing margins alignment (right)

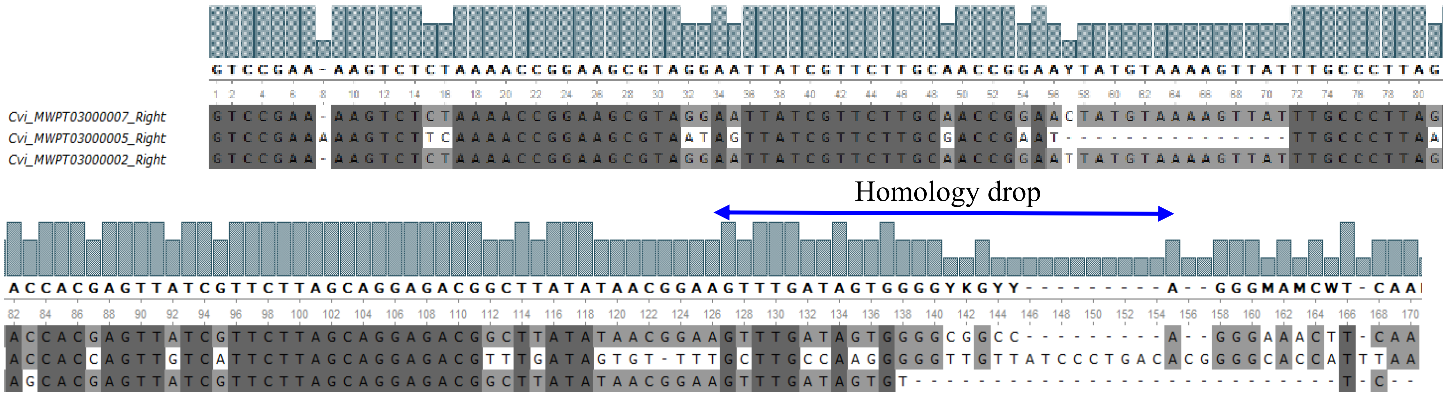

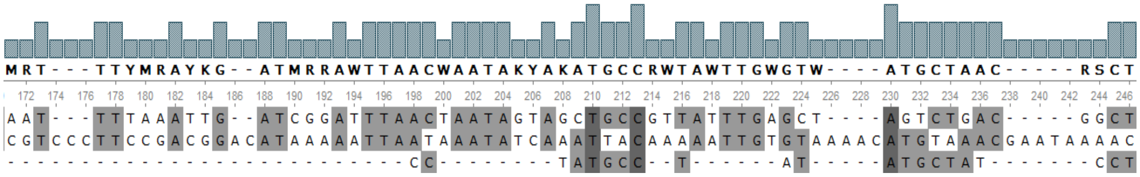

C. Alignment of Left homology drop regions with the reverse complement of right homology drop region in order to spot inverted repeat regions.

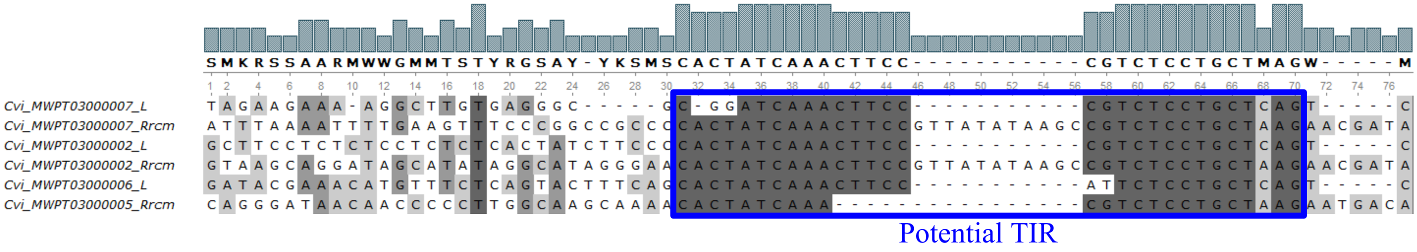

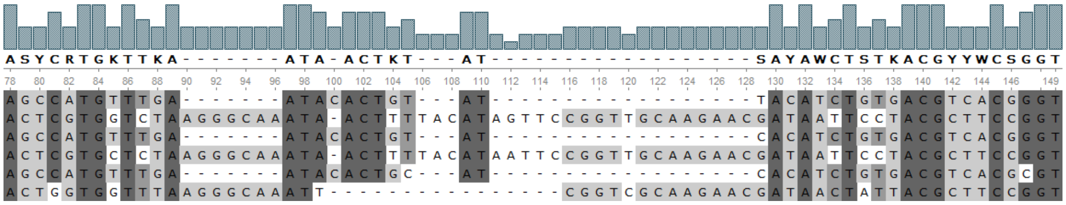

D. TSD pairs in MWPT03000007.1:

RAG1L side:

```
                TSD             Incomplete TIR5'
5' - GTGAGGGCGCGGATCAAACTTCCCGTCTCCTGCT - 3'
```

RAG2L side:

```
                                    TSD
5' - TATAACGGAAGTTTGATAGTGGGGCGGCCAGGGAAACT - 3'
3' - ATATTGCCTTCAAACTATCACCCCGCCGGTCCCTTTGA - 5'
                TIR3'
```

Crassostrea Virginica (Cvi)

(TaxID: 6565; Protostomia; Mollusca; Bivalvia; Ostreoida)

WGS: MWPT03000002.1 – copy 1

1. Summary

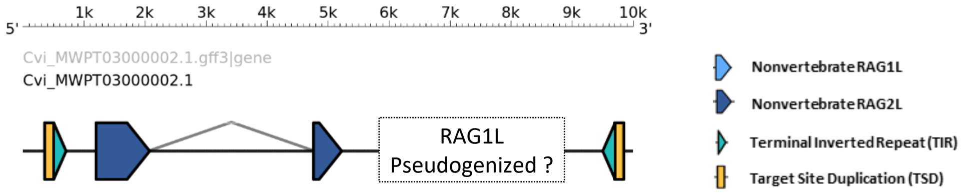

Detected configuration: TSD – TIR5' - RAG2L – TIR3' - TSD

Detected loci	Predicted CDS	start	end	strand	Observations
RAG1L	-	48299797	48297255	-	Pseudogenized (incomplete; stop codons)
RAG2L	1	48292771	48293649	+	complete; no stop codons
	2	48296320	48296787	+	

Predicted TIR	Distance from CDS (bp)	start	strand	TSD sequence	Assessment method
TIR5' (RAG1L side)	1395	48301192	-	5'-TTCCC-3'	Homology variation
TIR3' (RAG2L side)	691	48292080	+	5'-TTCCC-3'	Homology variation

CviTIR5'_0002_cp1

CviTIR3'_0002_cp1 (reverse complement)

```
5'CACTATCAAACTTC-----------CCGTCTCCTGCTCAG 3'
  ||||||||||||||           |||||||||||| ||
3'CACTATCAAACTTCCGTTATATAAGCCGTCTCCTGCTAAG 5'
```

Selected predicted protein product

CviRAG2L_B_Biv1_0002**

```
MMSFYKQFRALPFKSHSTNPLRRITRKSAASDDFNFFPPEGHVSLPINCYTYLYFGGARRGQ
ESTWNMSRNIYKISFIVDDNDVNVDFISEIKLSGGQFPQLQSSAGFYVSAENCLFVWGGLNL
SCFSMSNELYIVKLNDNKGVVEIIQPPGGISVRELGGEIPSGRCGHTLTHYFDSCVILHGGV
CFPHRNSCVGSSLFKNVTNDNSFYMFDFESLFWTKLSVTGSAPRAYHTANVVEIRGMKSIVY
IGGVTKTESALQRIPLSNVLVLKMDSNKHFHTEILTFATAPAVGISYHSVGVIGPYIFVVGG
LDEDNLQGRCSVSILNKDTFLCENVQFDRYFRSAGHSVCTLSDDCLMICGGMNLQYFVFSSK
QMVPSPCDFDTECKIIESVETSPLSWIQCEGACKRWLHQFCVGVLDIDMSRKNFICTTCSKT
SRVKKRKFLVQN
```

(predicted coding regions merging area is underlined as it is less confident)

** CviRAG2L_B_Biv1_0002 predicted protein product was not further included into analysis as it is 98 % identical with CviRAG2L_B_Biv1_0007

2. RAGL Detection info

Method: TBLASTN
Database searched: Whole-genome shotgun contigs (WGS) + only in Mollusca group (taxid: 6447)

2.1. RAG1L

Query: **CviRAG1L_B_Biv1_0007 (previously described)**
Subject: **MWPT03000002.1**

Score	Expect	Method	Identities	Positives	Gaps	Frame
710 bits(1833)	0.0	Compositional matrix adjust.	348/388(90%)	353/388(90%)	9/388(2%)	-2

```
Query  619       RTNRPLLESISDENNQVSNVVCILPIENEREILMQNRMHVKTKEGWMFHKFSFFNSMVDE  678
                 R NRPLLESISDENNQVSNVVCILPIENEREILMQNRMHVKTKEGWMFHKFSFFNSMVDE
Sbjct  48298391  RANRPLLESISDENNQVSNVVCILPIENEREILMQNRMHVKTKEGWMFHKFSFFNSMVDE  48298212

Query  679       KRDRGDSGLQGSGSKYLCTLCDADKQSAKALLGSFSINRSVSECSNIAEILRVNPNALSE  738
                 KRDRGDSGLQG    Y+  + +  K      LG F +         IAEILRVNPNALS+
Sbjct  48298211  KRDRGDSGLQGIYVLYVMQINNLQKH----YLGLFQLTGQF-----IAEILRVNPNALSK  48298059

Query  739       NELKKISKGVKCAPLSKIEPIQKGIDATHADINLGQFFKKIIVREIAGVTKWELTQDVKP  798
                 NE KKISKGVKCAPLSKIEPIQKGIDATHADINLGQF KKIIVREIAGVTKWELTQDVKP
Sbjct  48298058  NEFKKISKGVKCAPLSKIEPIQKGIDATHADINLGQFLKKIIVREIAGVTKWELTQDVKP  48297879

Query  799       LVQNAEFLFDQHMKCNCGINPQLMMPGNYARTLFETPHSVLLEHISDSVRKENLSSILNI  858
                 LVQNAEFLFDQHMKCNCGINPQLMMPGNY RTLFETPHSVLLE ISDSVRKENLSSILNI
Sbjct  48297878  LVQNAEFLFDQHMKCNCGINPQLMMPGNYTRTLFETPHSVLLERISDSVRKENLSSILNI  48297699

Query  859       FLHLRKVYRCKDPLTECPFDVQNYKKCAVEMGALLLQHFDYVEWPNYLHKVIEHVQQLIE  918
                  LHLRKVYRCKDPL ECPFDVQNYKKCAVEMGALLLQHFDYVEWPNYLHKVIEH QQLIE
Sbjct  48297698  ILHLRKVYRCKDPLIECPFDVQNYKKCAVEMGALLLQHFDYVEWPNYLHKVIEHFQQLIE  48297519

Query  919       DPNGPGSVGAFSSEGNEAGNKLFRHFRKNLSRRGNTYGSLCDVLKLHWLYSSKALFKLAE  978
                 DPNGPGSVGAFSSEGNEAGNKLFRHFRKNLSRRGNTYGSLCDVL LHWLYSSKALFKLAE
Sbjct  48297518  DPNGPGSVGAFSSEGNEAGNKLFRHFRKNLSRRGNTYGSLCDVL*LHWLYSSKALFKLAE  48297339

Query  979       VEHKKVRCSLCFTSGHNKRTCPLLNTSL  1006
                 VEHKKVRCSLCFTSGHNKRTCPLLNTSL
Sbjct  48297338  VEHKKVRCSLCFTSGHNKRTCPLLNTSL  48297255
```

Score	Expect	Method	Identities	Positives	Gaps	Frame
241 bits(616)	0.0	Compositional matrix adjust.	115/121(95%)	118/121(97%)	0/121(0%)	-3

```
Query  496       ENPCTEPLNVNECFLPGFVELATPNCMGVRFSYFEALSLTLQELEPEILFGLKKHGLNIE  555
                 ENPCTEPLNVNECFLPGFV+LATPNCMGVRFSYFEALS TLQ+LEPEILFGLKKHGLNIE
Sbjct  48298753  ENPCTEPLNVNECFLPGFVKLATPNCMGVRFSYFEALSTTLQKLEPEILFGLKKHGLNIE  48298574

Query  556       DVLFLTTVKDGCDGMGEVSVYKEKDFKMLPDKVFRFSFCIVKIQAEYDGKLFDVFTEPLP  615
                 +VLFLTTVKDGCDGMGEVSVYKEKDFKMLPDK FRFSFCIVKIQAEYDGKLFDVFTEPLP
Sbjct  48298573  NVLFLTTVKDGCDGMGEVSVYKEKDFKMLPDKAFRFSFCIVKIQAEYDGKLFDVFTEPLP  48298394

Query  616       N  616

Sbjct  48298393  T  48298391
```

Score	Expect	Method	Identities	Positives	Gaps	Frame
577 bits(1486)	3e-175	Compositional matrix adjust.	309/335(92%)	314/335(93%)	5/335(1%)	-3

```
Query  20        KNIQNFILSTDMEHGLKLKCMCRLCGVKIPINSKSHPKENFKSAITSLYNIDIDCDSENI  79
                 K    F  STDMEHGLKLKCMCRLC VKIPINSKSHPKENFKSAITSLYNIDIDCDSENI
Sbjct  48299797  KKYSKFYFSTDMEHGLKLKCMCRLCEVKIPINSKSHPKENFKSAITSLYNIDIDCDSENI  48299618

Query  80        HPPKICTACRLRLTRHSQAEDDIEINLPVLFEFLPHSINCNICFVKPGRPKKILKRKLSV  139
                 HPPKICTACRLRLTRHSQAEDDIEINLPVLFEFLPHSINCNICFVKPGRPK+ILKRKLSV
Sbjct  48299617  HPPKICTACRLRLTRHSQAEDDIEINLPVLFEFLPHSINCNICFVKPGRPKEILKRKLSV  48299438

Query  140       PSQSTKNIFIQQEHSYHSANKDSQDSSRPSTETRDITSPESPSSFTSFSSIVVPSASTSC  199
                 PSQSTKNIFIQQEHSYHS NK+SQ+SSRPSTETRDITSPESPSSFTSFSSIVVPSASTSC
Sbjct  48299437  PSQSTKNIFIQQEHSYHSGNKNSQESSRPSTETRDITSPESPSSFTSFSSIVVPSASTSC  48299258

Query  200       SFCPPVDSPPLPPVDKLSVVESCSSETAPEMFKNVVIRSIPTERFCNAEVSQSFMCTICR  259
                 SFCPPVDSPPLPPVDKLSV+ESCSSE APEMFKNVVIRSIPTERFCNAEVSQSFMCTICR
Sbjct  48299257  SFCPPVDSPPLPPVDKLSVIESCSSEPAPEMFKNVVIRSIPTERFCNAEVSQSFMCTICR  48299078

Query  260       GVPCDPYISKCSHIFCKECIFGWFSLSSACPVCRSLLDESEVSPLHGHLLQIYETLLVHC  319
                 GVPCDPYI KCSHIFCKECIFG      ACPVCRSLLDESEVSPLH HLLQIY+TLLVHC
Sbjct  48299077  GVPCDPYIPKCSHIFCKECIFGC-----ACPVCRSLLDESEVSPLHCHLLQIYDTLLVHC  48298913

Query  320       IHANCTQSHLIRNIDEHESICSMKGTKFLYNVTTK  354
                 IHANCTQSHLIRNIDEHESICSMKGTKFLY    K
Sbjct  48298912  IHANCTQSHLIRNIDEHESICSMKGTKFLYKCYNK  48298808
```

2.2 RAG2L

Query: **CviRAG2L_B_Biv1_0007 (previously described)**
Subject: **MWPT03000002.1**

Score	Expect	Method	Identities	Positives	Gaps	Frame
562 bits(1449)	3e-179	Compositional matrix adjust.	289/293(99%)	291/293(99%)	0/293(0%)	+1

```
Query  1         MMSFYKQFRALPFKSHSTNPLRRITRKSAASDDFNFFPPEGHVSLPINSYTYLYFGGARR  60
                 MMSFYKQFRALPFKSHSTNPLRRITRKSAASDDFNFFPPEGHVSLPIN YTYLYFGGARR
Sbjct  48292771  MMSFYKQFRALPFKSHSTNPLRRITRKSAASDDFNFFPPEGHVSLPINCYTYLYFGGARR  48292950

Query  61        GQESTWNMSRNIYKisfivddndvnvdfisEIKLSGGQFPQLQSSAGFYVSAENCLFVWG  120
                 GQESTWNMSRNIYKISFIVDDNDVNVDFISEIKLSGGQFPQLQSSAGFYVSAENCLFVWG
Sbjct  48292951  GQESTWNMSRNIYKISFIVDDNDVNVDFISEIKLSGGQFPQLQSSAGFYVSAENCLFVWG  48293130

Query  121       GLNLSCFSMSNELYIVKLNDNKGVVEIIQPPGGISVRELGGEIPSGRCGHTLTHYFDSCV  180
                 GLNLSCFSMSNELYIVKLNDNKGVVEIIQPPGGISVRELGGEIPSGRCGHTLTHYFDSCV
Sbjct  48293131  GLNLSCFSMSNELYIVKLNDNKGVVEIIQPPGGISVRELGGEIPSGRCGHTLTHYFDSCV  48293310

Query  181       ILHGGVCFPHRNSCVGSSLFKNVTNDNNFYMFDFESLFWTKLSVTGSAPRAYHTANVMEI  240
                 ILHGGVCFPHRNSCVGSSLFKNVTNDN+FYMFDFESLFWTKLSVTGSAPRAYHTANV+EI
Sbjct  48293311  ILHGGVCFPHRNSCVGSSLFKNVTNDNSFYMFDFESLFWTKLSVTGSAPRAYHTANVVEI  48293490

Query  241       RGMKSIVYIGGVTKTESALQRIPLSNVLVLKMDSNKHFHTEILTFANAPAVGI  293
                 RGMKSIVYIGGVTKTESALQRIPLSNVLVLKMDSNKHFHTEILTFA APAVGI
Sbjct  48293491  RGMKSIVYIGGVTKTESALQRIPLSNVLVLKMDSNKHFHTEILTFATAPAVGI  48293649
```

Score	Expect	Method	Identities	Positives	Gaps	Frame
301 bits(771)	2e-88	Compositional matrix adjust.	151/156(97%)	154/156(98%)	0/156(0%)	+1

```
Query  291       VGISYHSVGVIGPYIFVVGGLDEDNLQGRCSVSILNKDTFLCENVQFDRYFRSAGHSVCT  350
                 VGISYHSVGVIGPYIFVVGGLDEDNLQGRCSVSILNKDTFLCENVQFDRYFRSAGHSVCT
Sbjct  48296320  VGISYHSVGVIGPYIFVVGGLDEDNLQGRCSVSILNKDTFLCENVQFDRYFRSAGHSVCT  48296499

Query  351       LSDDCLMICGGMNLQYFVFSSKQMVPSPCDFDTECKIIESVETSPISWIQCEGACKRWLH  410
                 LSDDCLMICGGMNLQYFVFSSKQMVPSPCDFDTECKIIESVETSP+SWIQCEGACKRWLH
Sbjct  48296500  LSDDCLMICGGMNLQYFVFSSKQMVPSPCDFDTECKIIESVETSPLSWIQCEGACKRWLH  48296679

Query  411       QFCVGVLDIDMSRKNFICTTCskssrgkkrksLVQS  446
                 QFCVGVLDIDMSRKNFICTTCSK+SR KKRK LVQ+
Sbjct  48296680  QFCVGVLDIDMSRKNFICTTCSKTSRVKKRKFLVQN  48296787
```

3. TIR Detection info

See scaffold WGS: MWPT03000007.1 (page 5).

TSD pairs in MWPT03000002.1 copy1:

RAG1L side:

```
                TSD              TIR5`
5’ - CTCTCACTATCTTCCCCACTATCAAACTTCCCGTCTCCTGCTCAGT - 3’
```

RAG2L side:

```
                              TSD
5’ - CTTATATAACGGAAGTTTGATAGTGTTCCCTATGCCTATATGCTAT - 3’
3’ - GAATATATTGCCTTCAAACTATCACAAGGGATACGGATATACGATA - 5’
              TIR3`
```

C.virginica Potentially Pseudeogenized copies

WGS: MWPT03000002.1 – copy 2

1. Summary

Detected configuration: RAG2L - distantTIR3

Detected loci	Predicted CDS	start	end	strand	Observations
RAG2L	-	89840903	89841993	+	Pseudogenized (incomplete; stop codons)

Predicted TIR	Distance from CDS (bp)	start	strand	TSD sequence	Assessment method
TIR3' (RAG2L side)	4904	89835999	+	-	Blastn cassette margin

>CviTIR3_0002_cp2 (reverse complement)
ACTTTCAAAGTTACGTTATATAAGACGCCTCCTGTTAAG

2. TIR Detection info

TIR3 sequence was detected using BLASTN
Searched database was WGS in all Mollusca group

Query : reverse complement of TIR3' containing margin detected in scaffold WGS: MWPT03000002.1 copy1
Subject: scaffold WGS: MWPT03000002.1

Score	Expect	Identities	Gaps	Strand
130 bits(143)	5e-27	112/135(83%)	3/135(2%)	Plus/Plus

```
Query  2         ACTATCAAACTTCCGTTATATAAGCCGTCTCCTGCTAAGAACGATAACTCGTGCTCTAAG  61
                 ||| ||||| || ||||||||||| || |||||| ||||||||| |||  | || ||||
Sbjct  89835999  ACTTTCAAAGTTACGTTATATAAGACGCCTCCTGTTAAGAACGACAACCCATG-TCTATA  89836057

Query  62        GGCAAATAACTTTTACATAATTCCGGTTGCAAGAACGATAATTCCTACGCTTCCGGTTTT  121
                 |  ||||||| | |||||   ||||||||||||||||||| |||| ||||||| |||||||
Sbjct  89836058  GT-AAATAACATGTACATCGTTCCGGTTGCAAGAACGATCATTCATACGCTTTCGGTTTT  89836116

Query  122       AGAGACTTTTCGGAC  136
                  ||||| ||||||||
Sbjct  89836117  CGAGAC-TTTCGGAC  89836130
```

WGS: MWPT03000006.1 – copy 1

1. Summary

Detected configuration: TSD – TIR5 - RAG1L - RAG2L – distant TIR3 - TSD

Detected loci	Predicted CDS	start	end	strand	Observations
RAG1L	-	45389078	45387411	-	Pseudogenized (incomplete; fragmented; stop codons)
RAG2L	-	45384727	45385629	+	Pseudogenized (incomplete; fragmented; stop codons)

Predicted TIR	Distance from CDS (bp)	start	strand	TSD sequence	Assessment method
TIR5’ (RAG1L side)	888	45389966	-	CTTTCAG-TIR	Homology variation
TIR3’ (RAG2L side)	7415	45377312	+	TIR-TTTACAA	Blastn cassette margin

CviTIR5_0006.1_cp1

CviTIR3_0006.1_cp1 (reverse complement)

```
5’CACTATCAAACTTC-----------CATTCTCCTGCTCAG 3’
                              ||||||||| ||
3’-------------CCGTTATATAAGTCGTCTCCTGCTAAG 5’
```

3. TIR Detection info

TIR5 sequence was determined using homology variation (See scaffold WGS: MWPT03000007.1 - page 5).

TIR3 sequence was detected using BLASTN
Searched database was WGS in all Mollusca group

Query : reverse complement of TIR3’ containing margin detected in scaffold WGS: MWPT03000002.1 copy1
Subject: scaffold WGS: MWPT03000006.1

Score	Expect	Identities	Gaps	Strand
177 bits(195)	4e-41	120/135(89%)	0/135(0%)	Plus/Plus

```
Query  14         CCGTTATATAAGCCGTCTCCTGCTAAGAACGATAACTCGTGCTCTAAGGGCAAATAACTT  73
                  |||||||||||| ||||||||||||||||||| ||||| || || ||||||||||||| |
Sbjct  45377312   CCGTTATATAAGTCGTCTCCTGCTAAGAACGACAACTCATGGTCAAAGGGCAAATAACCT  45377371

Query  74         TTACATAATTCCGGTTGCAAGAACGATAATTCCTACGCTTCCGGTTTTAGAGACTTTTCG  133
                  ||||||| |||| |||||||||||||||||| ||||||||| |||||| |||||||||||
Sbjct  45377372   TTACATAGTTCCTGTTGCAAGAACGATAATTTCTACGCTTCAGGTTTTGGAGACTTTTCG  45377431

Query  134        GACTGTTTAACCGGT  148
                  |||   |||||||||
Sbjct  45377432   GACAAGGTAACCGGT  45377446
```

TSD pairs in MWPT03000006.1 copy1:

RAG1L side:

```
                                TSD             TIR5
5’ - TGTTTCTCAGTACTTTCAGCACTATCAAACTTCCATTCTCCTGCTCAG - 3’
```

RAG2L side:

```
                                         TSD
5’ - CTTAGCAGGAGACGACTTATATAACGGTTTACAATCGAA - 3’
3’ - GAATCGTCCTCTGCTGAATATATTGCCAAATGTTAGCTT - 5’
       Incomplete TIR3
```

WGS: MWPT03000006.1 – copy 2

1. Summary

Detected configuration: TSD - TIR5 - RAG1L - RAG2L – distant TIR3 - TSD

Detected loci	Predicted CDS	start	end	strand	Observations
RAG1L	-	45701199	45697958	-	Pseudogenized (incomplete; fragmented; stop codons)
RAG2L	-	45695276	45696177	+	Pseudogenized (incomplete; stop codons)

Predicted TIR	Distance from CDS (bp)	start	strand	TSD sequence	Assessment method
TIR5 (RAG1L side)	891	45702090	-	CTTTCAG-TIR	Blastn cassette margin
TIR3 (RAG2L side)	11753	45683523	+	TIR-TTTACAA	Blastn cassette margin

```
CviTIR5_0006.1_cp2                            5’CACTATCAAACTTC-----------CATTCTCCTGCTCAG 3’
                                                                         ||||||||| ||
CviTIR3_0006.1_cp2  (reverse complement)      3’-------------CCGTTATATAAGTCGTCTCCTGCTAAG 5’
```

2. TIR Detection info

TIR5 sequence was detected using BLASTN
Searched database was WGS_VDB://MWPT03 project.

Query: TIR5 containing margin detected in scaffold WGS: MWPT03000002.1 copy1
Subject: scaffold WGS: MWPT03000006.1

Score	Expect	Identities	Gaps	Strand
212 bits(234)	1e-53	132/142(93%)	0/142(0%)	Plus/Minus

```
Query  1         CACTATCAAACTTCCCGTCTCCTGCTCAGTCAGCCATGTTTGAATACACTGTATCACATC  60
                 ||||||||||||||   |||||||||||||||||||||||||||||||||| ||||||||
Sbjct  45702090  CACTATCAAACTTCCATTCTCCTGCTCAGTCAGCCATGTTTGAATACACTGCATCACATC  45702031
```

```
Query  61        TGTGACGTCACGGGTGCTTTCAACACTCCGCTTCAGTTTGAAAAGCATCAAACATGCTAC  120
                 |||||||||||| ||| |||||||||||||||||||||| |||||||| | |||||||||
Sbjct  45702030  TGTGACGTCACGCGTGTTTTCAACACTCCGCTTCAGTTTTAAAAGCATTAGACATGCTAC  45701971

Query  121       ATGCGCAAGGCGTGCCGAATGT  142
                 ||||  ||||||||||||||||
Sbjct  45701970  ATGCCTAAGGCGTGCCGAATGT  45701949
```

TIR3 sequence was detected using BLASTN
Searched database was WGS in all Mollusca group

Query : reverse complement of TIR3' containing margin detected in scaffold WGS: MWPT03000002.1 copy1
Subject: scaffold WGS: MWPT03000006.1

Score	Expect	Identities	Gaps	Strand
177 bits(195)	4e-41	120/135(89%)	0/135(0%)	Plus/Plus

```
Query  14        CCGTTATATAAGCCGTCTCCTGCTAAGAACGATAACTCGTGCTCTAAGGGCAAATAACTT  73
                 |||||||||||| ||||||||||||||||||| ||||| || || ||||||||||||| |
Sbjct  45683523  CCGTTATATAAGTCGTCTCCTGCTAAGAACGACAACTCATGGTCAAAGGGCAAATAACCT  45683582

Query  74        TTACATAATTCCGGTTGCAAGAACGATAATTCCTACGCTTCCGGTTTTAGAGACTTTTCG  133
                 ||||||| |||| |||||||||||||||||| ||||||||| |||||| |||||||||||
Sbjct  45683583  TTACATAGTTCCTGTTGCAAGAACGATAATTTCTACGCTTCAGGTTTTGGAGACTTTTCG  45683642

Query  134       GACTGTTTAACCGGT  148
                 |||    ||||||||
Sbjct  45683643  GACAAGGTAACCGGT  45683657
```

TSD pairs in MWPT03000006.1 copy2:

RAG1L side:

```
                  TSD             TIR5
5' - TGTTTCTCAGTACTTTCAGCACTATCAAACTTCCATTCTCCTGCTCAG - 3'
```

RAG2L side:

```
                                   TSD
5' - CTTAGCAGGAGACGACTTATATAACGGTTTACAATGAAA - 3'
3' - GAATCGTCCTCTGCTGAATATATTGCCAAATGTTACTTT - 5'
        Incomplete TIR3
```

WGS: MWPT03000005.1

1. Summary

Detected configuration: RAG1L - RAG2L

Detected loci	Predicted CDS	start	end	strand	Observations
RAG1L	-	33746540	33739309	-	Pseudogenized (incomplete; stop codons)
RAG2L	-	33738646	33739302	+	Pseudogenized (incomplete; stop codons)

WGS: MWPT03000001.1

1. Summary

Detected configuration: RAG1L

Detected loci	Predicted CDS	start	end	strand	Observations
RAG1L	-	7695514	7695876	+	Pseudogenized (incomplete; short segment)

Detected configuration: RAG1L

Detected loci	Predicted CDS	start	end	strand	Observations
RAG1L	-	63500927	63501286	+	Pseudogenized (incomplete; short segment)

Detected configuration: RAG2L

Detected loci	Predicted CDS	start	end	strand	Observations
RAG2L	-	92806530	93816947	+	Pseudogenized (incomplete; fragmented; stop codons)

Crassostrea Gigas (Cgi)

(TaxID: 29159; Protostomia; Mollusca; Bivalvia; Ostreoida)

Potentially Psudeogenized copies

WGS: AFTI01009375.1

1. Summary

Detected configuration: distant TIR5 - RAG1L

Detected loci	Predicted CDS	start	end	strand	Observations
RAG1L	-	10133	11489	+	Pseudogenized (incomplete; fragmented; stop codons)

Predicted TIR	Distance from CDS (bp)	start	strand	TSD sequence	Assessment method
TIR5 (RAG1L side)	3203	6930	+	-	Blastn cassette margin

CgiTIR5_9375 5’CACTATCAAACTTCCCGTCTCCTGCTCAG 3’

2. TIR Detection info

TIR5 sequence was detected using BLASTN
Searched database was WGS_VDB://MWPT03 project.

Query : TIR5 containing margin detected in scaffold WGS: MWPT03000002.1 copy1
Subject: WGS: AFTI01009375.1

Score	Expect	Identities	Gaps	Strand
123 bits(135)	2e-24	115/144(80%)	2/144(1%)	Plus/Plus

```
Query  1     CACTATCAAACTTCCCGTCTCCTGCTCAGTCAGCCATGT-TTGAATACACTGTATCACAT  59
             ||||||||||||||| ||||||||||||||  ||||| |    || | | |  || |||
Sbjct  6930  CACTATCAAACTTCCTGTCTCCTGCTCAGTTGGCCATTTAAAAAAAAAAATACATTACAA  6989

Query  60    CTGTGACGTCACGGGTGCTTTCAACACTCCGCTTCAGTTTGAAAAGCATCAAACATGCTA  119
             || ||||||||| ||||||||| |||||||||||||| || |||| ||  | | ||| ||
Sbjct  6990  CTATGACGTCAC-GGTGCTTTCCACACTCCGCTTCAGATTTAAAACCACAAGATATGGTA  7048

Query  120   CATGCGCAAGGCGTGCCGAATGTT  143
             ||||||||||| || |||||| ||
Sbjct  7049  CATGCGCAAGGAGTACCGAATATT  7072
```

WGS: AFTI01009376.1

1. Summary

Detected configuration: RAG2L + distant TIR3

Detected loci	Predicted CDS	start	end	strand	Observations
RAG2L	-	886	2	-	Pseudogenized (incomplete; fragmented; stop codons)

Predicted TIR	Distance from CDS (bp)	start	strand	TSD sequence	Assessment method
TIR3 (RAG2L side)	4089	4975	-		Blastn cassette margin

CgiTIR3_9376 (reverse complement) 3′ACTATCAAACTTCCGTTATATAAG 5′

TIR3 sequence was detected using BLASTN
Searched database was WGS in all Mollusca group

Query: **reverse complement** of TIR3 containing margin detected in scaffold WGS: MWPT03000002.1 copy1
Subject: WGS: AFTI01009375.1

Score	Expect	Identities	Gaps	Strand
92.4 bits(101)	1e-15	88/109(81%)	3/109(2%)	Plus/Minus

```
Query  2     ACTATCAAACTTCCGTTATATAAGCCGTCTCCTGCTAAGAACGATAACTCGTGCTCTAAG  61
             ||||||||||||||| | |||| | |||| |||||||||||| |||||||||||  ||| 
Sbjct  4975  ACTATCAAACTTCCGCTCTATACGGCGTCACCTGCTAAGAACAATAACTCGTGC-GTAAA  4917

Query  62    GGCAAATAACT-TTTACATAATTCCGGTTGCAAGAACGATAATTCCTAC  109
               | ||||||| ||| | || |||||||  ||||  |||||| ||||||
Sbjct  4916  AAC-AATAACTCTTTCCGTATTTCCGGTAACAAGTGCGATAACTCCTAC  4869
```

WGS: AFTI01023050.1

Summary

Detected configuration: RAG2L - TIR3

Detected loci	Predicted CDS	start	end	strand	Observations
RAG2L	-	23139	24802	+	Pseudogenized (incomplete; fragmented; stop codons)

Predicted TIR	Distance from CDS (bp)	start	strand	TSD sequence	Assessment method
TIR3 (RAG2L side)	790	22349	+		Blastn cassette margin

CgiTIR3_3050 (reverse complement) 3′ CACTATCAATATTCCGCTATATACGGCGTCTCCTGCTAAG 5′

TIR3 sequence was detected using BLASTN
Searched database was WGS in all Mollusca group

Query: **reverse complement** of TIR3 containing margin detected in scaffold WGS: MWPT03000002.1 copy1
Subject: WGS: AFTI01023050.1

Score	Expect	Identities	Gaps	Strand
70.7 bits(77)	4e-09	46/51(90%)	0/51(0%)	Plus/Plus

```
Query  1      CACTATCAAACTTCCGTTATATAAGCCGTCTCCTGCTAAGAACGATAACTC  51
              |||||||||   ||||| |||||| | ||||||||||||||||||||||||
Sbjct  22349  CACTATCAATATTCCGCTATATACGGCGTCTCCTGCTAAGAACGATAACTC  22399
```

WGS: AFTI01024488.1

Summary

Detected configuration: RAG2L

Detected loci	Predicted CDS	start	end	strand	Observations
RAG2L	-	43628	42308	-	Pseudogenized (incomplete; fragmented; stop codons)

WGS: AFTI01026957.1

Summary

Detected configuration: RAG1L - RAG2L

Detected loci	Predicted CDS	start	end	strand	Observations
RAG1L	-	920	633	-	Incomplete, short segment
RAG2L	-	2	415	+	Incomplete, short segment

WGS: AFTI01029017.1

Summary

Detected configuration: RAG2L

Detected loci	Predicted CDS	start	end	strand	Observations
RAG2L	-	5990	5578	-	Pseudogenized (incomplete; fragmented; stop codons)

Saccostrea glomerata (Sgl)

WGS: PRKT01001405.1

(TaxID: 157728; Protostomia; Mollusca; Bivalvia; Ostreoida)

1. Summary

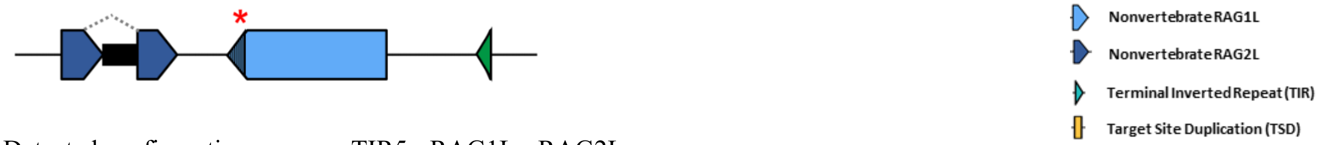

Detected configuration: TIR5 - RAG1L - RAG2L

Detected loci	Predicted CDS	start	end	strand	Observations
RAG1L (!)	1	86267	83718	-	early stop codon at C-ter
RAG2L (!)	1	80149	80600	+	complete; no stop codons;
	2	81601	82398	+	assembly gap between the 2 potential exons !

Predicted TIR	Distance from CDS (bp)	start	strand	TSD sequence	Assessment method
TIR5 (RAG1L side)	2027	88294	-	–	Blastn cassette margin

SglTIR5_1405   5′ CACTATCAAACTTCCCGTCTCCTGCTCAGTCAG 3′

Selected predicted protein product

SglRAG1L_B_Biv1_1405

```
MEHELKLKCMCRLCGVKIPVNSKSHPKENFKPAITSLYNIDIDSDSDNIHPTRICTACRLRLTRHSQ
AEDDIIVNLPVLYEFLPHSDSCDICFVKPGRPKKILKRKLNVPSQSKKNSFIRQEHSYHSADKDKSR
PSTETTDTTSSDCPSSSSSFCPLVNYSSVNVDHPSGSSSCSICPPVDSSPFPHVDKLSIADTSSASA
SEMFKNVIIRSIPVERFCNAEVAQSFMCTVCRGVPVDPYISKCNHIFCKECIFGWFSLSSACPVCRS
LLDESEVSPLHGQLLQIYETLLVHCIHADCSNSLLIRNIDEHEKTCSKRATKVLYDVTTKPKVFKLP
LHSVSAKHTRHKRLKPLISTVNEFCEMQDENKSDVLFFMLKDHLKELNDPRWKQIETLWLGNNNTLS
PEQCLAIRVDLLQSKGQYRSQYDFLSQNNVHIFQAPSKMESCENLFMPSAAISQVVNNEGNVLFQNC
KNPHTEPLNVNQCFMPGFVELATPNCMGVRFSYFEALSITLQELESEIANSLEKHGLDIENVLFHTT
VKDGCDGMGEVSVYREKDFKMLPDKAFRFSFCVIKIQAEYQGKMYDVFNEPFPNSVRTNRPLLESIS
DENNHVSNVVCILPIENEREILMQNCMHVKTKQGWMFHKFSFFNSMVDEKRDRGDSGLQGSGSKYLC
TLCDADKQSAKESLGSFVINRSVSECSNIAEILRVNPNSLSENELKSISKGVKSAPLTKIEPIQKGI
DATHADINLGQFFKKVIVREIAGVTKWELTQDVKPLVQNAESSFDQHMKCNCGINPQLMMPGNYART
LFETPHSVLLALISDSVRKENLSSVLKIFLNLRKVYRCKDPLKQCP
```

early stop codon !

Prediction performed with FGENESH+ (C.elegans dataset and CviRAG1L_B_Biv1_0007 as similarity reference)

SglRAG2L_B_Biv1_1405

```
MMSIYKQFRALPFKSHSTNPLRRITRKSAAADELNYFPPEGHVSLPINSYTCLYFGGARRGQESTWS
MSNKLYKVSFDVDDNDVNVDSISEIKLSGGQFPHLQSSAGFYITPQNCLFVWGGLNLSCFSMSNELY
IVKLNDKKGVVEIVQPPGGISLRELGGEIPSGRCGHTLTHIFNCCVILHGGVCFPNRNTCVGSSLFT
NVTNDSSFYMFDFESLFWTKLSVSGPEPRAYHTANIMDIKGMRSIVYIGGVTKTENDLHRIPL[659
bp ASSEMBLY GAP]
SNVLILKMDSDKHFHTEILIFANSPAVGVSYHSTGVIGPYIFVVGGVDENNLQGRNSVSILNVETFS
```

```
CEDIQFDQSFRSAGHSVCTLSDDCLMICGGMHLQYFVFTSKPMVPSPCDFENDCRIIESTETSPISW
IQCEGTCKRWLHQFCVGVLDIDMSRKRFICTTCSKTSRGKKRKSLT
```

Consensus of predicted potential genes using Augustus (multiple invertebrate bilaterian species datasets) and FGENESH+ (multiple invertebrate bilaterian species datasets), using CviRAG2L_B_Biv1_0007 as reference.

Despite the long assembly gap in the middle, the resulting protein sequence does not contain missing segments when compared to the reference used.

Predicted coding regions merging areas are underlined as they are less confident.

2. RAGL Detection info

Method: TBLASTN
Database searched: Whole-genome shotgun contigs (WGS) + only in Mollusca group (taxid: 6447)

2.1. RAG1L

Query: CviRAG1L_B_Biv1_0007 (previously described)
Subject: PRKT01001405.1 Length: 88294 Number of Matches: 1

Score	Expect	Method	Identities	Positives	Gaps	Frame
1393 bits(5287)	0.0	Compositional matrix adjust.	844/1013(83%)	938/1013(92%)	9/1013(0%)	-3

```
Query  2      AAYINTNCLHC-LEIMQMTKNIQNF--ILSTDMEHGLKLKCMCRLCGVKIPINSKSHPKE  58
               AY +T+ LHC ++ ++  K   +F  I ST+MEH LKLKCMCRLCGVKIP+NSKSHPKE
Sbjct  86363  VAYKHTHLLHCVIDDLKNEKIYIKFYFISSTEMEHELKLKCMCRLCGVKIPVNSKSHPKE  86184

Query  59     NFKSAITSLYNIDIDCDSENIHPPKICTACRLRLTRHSQAEDDIEINLPVLFEFLPHSIN  118
              NFK+AITSLYNIDID DS+NIHP +ICTACRLRLTRHSQAEDDI +NLPVL+EFLPHS +
Sbjct  86183  NFKPAITSLYNIDIDSDSDNIHPTRICTACRLRLTRHSQAEDDIIVNLPVLYEFLPHSDS  86004

Query  119    CNICFVKPGRPKKILKRKLSVPSQSTKNIFIQQEHSYHSANKDSQDSSRPSTETRDITSP  178
              C+ICFVKPGRPKKILKRKL+VPSQS KN FI+QEHSYHSA+KD   +S  +T+T     P
Sbjct  86003  CDICFVKPGRPKKILKRKLNVPSQSKKNSFIRQEHSYHSADKDKSRPSTETTDTTSSDCP  85824

Query  179    ESPSSF---TSFSSIVV--PSASTSCSFCPPVDSPPLPPVDKLSVVESCSSETAPEMFKN  233
               S+SSF    ++SS+ V  PS+S+SCS+CPPVDS+P+P VDKLS+ ++ SS +A+EMFKN
Sbjct  85823  SSSSSFCPLVNYSSVNVDHPSGSSSCSICPPVDSSPFPHVDKLSIADT-SSASASEMFKN  85647

Query  234    VVIRSIPTERFCNAEVSQSFMCTICRGVPCDPYISKCSHIFCKECIFGWFSLSSACPVCR  293
              V+IRSIP ERFCNAEV+QSFMCT+CRGVP DPYISKC+HIFCKECIFGWFSLSSACPVCR
Sbjct  85646  VIIRSIPVERFCNAEVAQSFMCTVCRGVPVDPYISKCNHIFCKECIFGWFSLSSACPVCR  85467

Query  294    SLLDESEVSPLHGHLLQIYETLLVHCIHANCTQSHLIRNIDEHESICSMKGTKFLYNVTT  353
              SLLDESEVSPLHG+LLQIYETLLVHCIHA+C++S LIRNIDEHE  CS ++TK LY+VTT
Sbjct  85466  SLLDESEVSPLHGQLLQIYETLLVHCIHADCSNSLLIRNIDEHEKTCSKRATKVLYDVTT  85287

Query  354    KSRVFKLPLHSVSAKHTRHRRLKPIISQVNEFCNAQEENKSDVLFFMLKDHLKEINDPRW  413
              K++VFKLPLHSVSAKHTRH+RLKP+IS VNEFC+ Q+ENKSDVLFFMLKDHLKE+NDPRW
Sbjct  85286  KPKVFKLPLHSVSAKHTRHKRLKPLISTVNEFCEMQDENKSDVLFFMLKDHLKELNDPRW  85107

Query  414    KQVESLWLGNNSTLSPEQCLALRVDLLQSKGQYRSQYDFLSQNNVHVFQAPSKMESCENL  473
              KQ+E+LWLGNN+TLSPEQCLA+RVDLLQSKGQYRSQYDFLSQNNVH+FQAPSKMESCENL
Sbjct  85106  KQIETLWLGNNNTLSPEQCLAIRVDLLQSKGQYRSQYDFLSQNNVHIFQAPSKMESCENL  84927
```

```
Query  474    FMPSASIFQIIDNDGNVLLQNSENPCTEPLNVNECFLPGFVELATPNCMGVRFSYFEALS  533
              FMPSA+I Q+++N+GNVL+QN  NP TEPLNVN+CF+PGFVELATPNCMGVRFSYFEALS
Sbjct  84926  FMPSAAISQVVNNEGNVLFQNCKNPHTEPLNVNQCFMPGFVELATPNCMGVRFSYFEALS  84747

Query  534    LTLQELEPEILFGLKKHGLNIEDVLFLTTVKDGCDGMGEVSVYKEKDFKMLPDKVFRFSF  593
              +TLQELE+EI  +L KHGL+IE+VLF TTVKDGCDGMGEVSVY+EKDFKMLPDK FRFSF
Sbjct  84746  ITLQELESEIANSLEKHGLDIENVLFHTTVKDGCDGMGEVSVYREKDFKMLPDKAFRFSF  84567

Query  594    CIVKIQAEYDGKLFDVFTEPLPNSVRTNRPLLESISDENNQVSNVVCILPIENEREILMQ  653
              C++KIQAEY+GK++DVF EP+PNSVRTNRPLLESISDENN+VSNVVCILPIENEREILMQ
Sbjct  84566  CVIKIQAEYQGKMYDVFNEPFPNSVRTNRPLLESISDENNHVSNVVCILPIENEREILMQ  84387

Query  654    NRMHVKTKEGWMFHKFSFFNSMVDEKRDRGDSGLQGSGSKYLCTLCDADKQSAKALLGSF  713
              N MHVKTK+GWMFHKFSFFNSMVDEKRDRGDSGLQGSGSKYLCTLCDADKQSAK  LGSF
Sbjct  84386  NCMHVKTKQGWMFHKFSFFNSMVDEKRDRGDSGLQGSGSKYLCTLCDADKQSAKESLGSF  84207

Query  714    SINRSVSECSNIAEILRVNPNALSENELKKISKGVKCAPLSKIEPIQKGIDATHADINLG  773
               INRSVSECSNIAEILRVNPN+LSENELK ISKGVK APL+KIEPIQKGIDATHADINLG
Sbjct  84206  VINRSVSECSNIAEILRVNPNSLSENELKSISKGVKSAPLTKIEPIQKGIDATHADINLG  84027

Query  774    QFFKKIIVREIAGVTKWELTQDVKPLVQNAEFLFDQHMKCNCGINPQLMMPGNYARTLFE  833
              QFFKK+IVREIAGVTKWELTQDVKPLVQNAE  FDQHMKCNCGINPQLMMPGNYARTLFE
Sbjct  84026  QFFKKVIVREIAGVTKWELTQDVKPLVQNAESSFDQHMKCNCGINPQLMMPGNYARTLFE  83847

Query  834    TPHSVLLEHISDSVRKENLSSILNIFLHLRKVYRCKDPLTECPFDVQNYKKCAVEMGALL  893
              TPHSVLL  ISDSVRKENLSS+L+IFL+LRKVYRCKDPL +CP DV+NYKK AVEMG+LL
Sbjct  83846  TPHSVLLALISDSVRKENLSSVLKIFLNLRKVYRCKDPLKQCP*DVKNYKKHAVEMGTLL  83667

Query  894    LQHFDYVEWPNYLHKVIEHVQQLIEDPNGPGSVGAFSSEGNEAGNKLFRHFRKNLSRRGN  953
              L++FDYVEWPNYLHKVIEHVQQLIEDPNGPGS+GAFSSEGNEAGNKLFRHFRKNLSRRGN
Sbjct  83666  LENFDYVEWPNYLHKVIEHVQQLIEDPNGPGSIGAFSSEGNEAGNKLFRHFRKNLSRRGN  83487

Query  954    TYGSLCDVLKLHWLYSSKALFKLAEVEHKKVRCSLCFTSGHNKRTCPLLNTSL  1006
              TYGSLCDVLKLHWLYSSKALFKLAEVEHKK+RCSLCFT GHNKRTCPLLNTSL
Sbjct  83486  TYGSLCDVLKLHWLYSSKALFKLAEVEHKKIRCSLCFTCGHNKRTCPLLNTSL  83328
```

2.2 RAG2L

Query: **CviRAG2L_B_Biv1_0007 (previously described)**
Subject: **PRKT01001405.1** **Length: 88294** **Number of Matches: 2**

Score	Expect	Method	Identities	Positives	Gaps	Frame
388 bits(1458)	4e-125	Compositional matrix adjust.	226/265(85%)	256/265(96%)	0/265(0%)	+1

```
Query  1      MMSFYKQFRALPFKSHSTNPLRRITRKSAASDDFNFFPPEGHVSLPINSYTYLYFGGARR  60
              MMS+YKQFRALPFKSHSTNPLRRITRKSAA+D++N+FPPEGHVSLPINSYT LYFGGARR
Sbjct  80149  MMSIYKQFRALPFKSHSTNPLRRITRKSAAADELNYFPPEGHVSLPINSYTCLYFGGARR  80328

Query  61     GQESTWNMSRNIYKISFIVDDNDVNVDFISEIKLSGGQFPQLQSSAGFYVSAENCLFVWG  120
              GQESTW+MS ++YK+SF VDDNDVNVD ISEIKLSGGQFP+LQSSAGFY++++NCLFVWG
Sbjct  80329  GQESTWSMSNKLYKVSFDVDDNDVNVDSISEIKLSGGQFPHLQSSAGFYITPQNCLFVWG  80508

Query  121    GLNLSCFSMSNELYIVKLNDNKGVVEIIQPPGGISVRELGGEIPSGRCGHTLTHYFDSCV  180
              GLNLSCFSMSNELYIVKLND+KGVVEI+QPPGGIS+RELGGEIPSGRCGHTLTH F+ CV
Sbjct  80509  GLNLSCFSMSNELYIVKLNDKKGVVEIVQPPGGISLRELGGEIPSGRCGHTLTHIFNCCV  80688

Query  181    ILHGGVCFPHRNSCVGSSLFKNVTNDNNFYMFDFESLFWTKLSVTGSAPRAYHTANVMEI  240
              ILHGGVCFP+RN+CVGSSLF NVTND++FYMFDFESLFWTKLSV+G+ PRAYHTAN+M+I
```

```
Sbjct  80689  ILHGGVCFPNRNTCVGSSLFTNVTNDSSFYMFDFESLFWTKLSVSGPEPRAYHTANIMDI  80868

Query  241    RGMKSIVYIGGVTKTESALQRIPLS  265
              +GM+SIVYIGGVTKTE+ L+RIPLS
Sbjct  80869  KGMRSIVYIGGVTKTENDLRRIPLS  80943
```

Score	Expect	Method	Identities	Positives	Gaps	Frame
177 bits(656)	3e-50	Compositional matrix adjust.	95/115(83%)	111/115(96%)	0/115(0%)	+1

```
Query  179    CVILHGGVCFPHRNSCVGSSLFKNVTNDNNFYMFDFESLFWTKLSVTGSAPRAYHTANVM  238
              CVILHGGVCFP+RN+CVGSSLF NVTND++FYMFDFESLFWTKLSV+G+ PRAYHTAN+M
Sbjct  81601  CVILHGGVCFPNRNTCVGSSLFTNVTNDSSFYMFDFESLFWTKLSVSGPEPRAYHTANIM  81780

Query  239    EIRGMKSIVYIGGVTKTESALQRIPLSNVLVLKMDSNKHFHTEILTFANAPAVGI  293
              +I+GM+SIVYIGGVTKTE+ L+RIPLSNVL+LKMDS+KHFHTEIL FAN+PAVG+
Sbjct  81781  DIKGMRSIVYIGGVTKTENDLHRIPLSNVLILKMDSDKHFHTEILIFANSPAVGV  81945
```

Potentially pseudogenized copies

WGS: PRKT01001316.1

1. Summary

Detected configuration: RAG1L - RAG2L - TIR3

Detected loci	Predicted CDS	start	end	strand	Observations
RAG1L	-	27334	24516	-	Pseudogenized (stop codons)
RAG2L	-	22216	23518	+	Pseudogenized (stop codons)

Predicted TIR	Distance from CDS (bp)	start	strand	TSD sequence	Assessment method
TIR3 (RAG2L side)	1024	21192			Blastn cassette margin

SglTIR3_1316 3′ CACTATCAAACTTCCGCTATATATGGCGTCTCCTGCTAAG 5′

WGS: PRKT01000024.1-copy1

Summary

Detected configuration: RAG1L

Detected loci	Predicted CDS	start	end	strand	Observations
RAG1L	-	841899	841552	-	incomplete; short segment

WGS: PRKT01000024.1-copy2

Summary

Detected configuration: RAG1L

Detected loci	Predicted CDS	start	end	strand	Observations
RAG1L	-	104967	105273	+	incomplete; short segment

WGS: PRKT01000035.1

Summary

Detected configuration: RAG1L

Detected loci	Predicted CDS	start	end	strand	Observations
RAG1L	-	1167395	1171536	+	Pseudogenized (complete; fragmented; stop codons)

WGS: PRKT01000326.1

Summary

Detected configuration: RAG1L

Detected loci	Predicted CDS	start	end	strand	Observations
RAG1L	-	358369	357875	-	Pseudogenized (incomplete; fragmented; stop codons)

WGS: PRKT01000409.1 - copy 1

Summary

Detected configuration: RAG1L

Detected loci	Predicted CDS	start	end	strand	Observations
RAG1L*	-	259013	259537	+	Pseudogenized (incomplete; stop codons)

WGS: PRKT01000409.1 - copy 2

Summary

Detected configuration: RAG1L

Detected loci	Predicted CDS	start	end	strand	Observations
RAG1L	-	311798	312197	+	Pseudogenized (incomplete; stop codons)

WGS: PRKT01001214.1

Summary

Detected configuration: RAG1L

Detected loci	Predicted CDS	start	end	strand	Observations
RAG1L	-	98934	99537	+	Pseudogenized (incomplete; stop codons)

WGS: PRKT01001234.1

Summary

Detected configuration: RAG2L

Detected loci	Predicted CDS	start	end	strand	Observations
RAG2L	-	5824	4954	-	Pseudogenized (incomplete; fragmented; stop codons)

M.philippinarum (Mph)

WGS: MJUU01063471.1

Summary

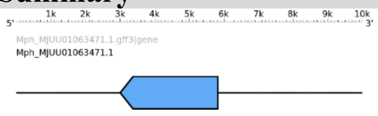

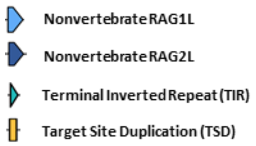

Detected configuration: RAG1L

Detected loci	Predicted CDS	start	end	strand	Observations
RAG1L(!)	-	3071	231	-	complete; no stop codons<br>RAG2L might be on a different scaffold as RAG1L ends near the beginning of the scaffold

Selected predicted protein product	
MphRAG1L_B_Biv1_3471	MKMSEEHSSKLKSLCRLCCSKIQKNCNKTNFSSEIKEIYDIDVVVESEQQ<br>YPQNICSSCRLRLTRFRQNKGSCGIQFCELSVFYPHDQDCKICCVKSGRP<br>KKVGKRKLSKVAVDLSNQDNSDTDSASECCDLDCGDLSRTSSFDQTNKHD<br>LSGSDSDNDTLLLKNLSLENEVSKSNILNFNNVIIDSLCIERFWDPQIAG<br>VFVCSICRGVPFVPVITSCNHIFCSECIRSWVNVSSACPVCRCCLDETDV<br>LPLSGNLLHIYDSLVVKCVHTNCQAKVALKNIKLHDVSCQHTLPTQIYNF<br>KKPKLPLHEVTSKHVKHRRLKPIISSIDDFCDTEGENKTDVLFSLLRNHL<br>KDSSDSRWKDIDMLWQGSSDTMSADQCLALRVNLLQSKGQYKCQYDFLKS<br>NNVHVFQCPSQLDKVESKYLPGHVEYSIIGDDFFENIYKTPCSSDPINIL<br>GDFCTNFPEFPSPNCMGVRWSYTDALAKTVEELSEELVSGLKYHNLNPDD<br>PSILFLTTVKDGGDGMGDCSVYRERSDRFLPDKAFRFSFSVVKIEAVSPS<br>GIFTVFTESSPNSVKNNKPLLESLADENNKSTISVCMAPIENERKYLQNK<br>IMKVKTSYGWHRHEFNFFNSMVDEKRDRSDGGLQGSGSKFLCTLCDADRD<br>TATQKLGSFEINRSIIETSKIYEYIRINPDKLSETELAKKSKGIKSEPMS<br>LMEPIKKGIDATHADINMGSFFKKIIVREIACVTKWELSEDIKEIVKNAE<br>SKFDFHMKEHCGINPQLMMPGNYARTIFTTPHDILLCLISDVERQQNLSV<br>ILDKFNFLRKIYRSNDPKSDFPNDISLFKVCAVEMGKLLLSNFDYVRWPN<br>YLHKLIEHTQQLIQDPAGPGAIGSFSSEGNEAGNKLFRHFRKNLSRRGST<br>YGSLCDVLKLHWLYSSKALGKLAEKEQKKNKCSLCFQTGHNRRTCPLL<br><br>Prediction performed with FGENESH+ (C.elegans dataset and CviRAG1L_B_Biv1_0007 as homologous reference)

RAGL Detection info

Method: TBLASTN
Database searched: Whole-genome shotgun contigs (WGS) + only in Mollusca group (taxid: 6447)

RAG1L

Query: **CviRAG1L_B_Biv1_0007 (previously described)**
Subject: **MJUU01063471.1 Length: 3414 Number of Matches: 1**

Score	Expect	Method	Identities	Positives	Gaps	Frame
857 bits(3244)	0.0	Compositional matrix adjust.	531/980(54%)	721/980(73%)	46/980(4%)	-2

```
Query  32    EHGLKLKCMCRLCGVKIPINSKSHPKENFKSAITSLYNIDIDCDSENIHPPKICTACRLR  91
```

```
                EH+ KLK +CRLC  KI    K+  K NF S I  +Y+ID+  +SE+  P +IC++CRLR
Sbjct  3059   EHSSKLKSLCRLCCSKIQ---KNCNKTNFSSEIKEIYDIDVVVESEQQYPQNICSSCRLR  2889

Query  92     LTRHSQAEDDIEINLPVLFEFLPHSINCNICFVKPGRPKKILKRKLS-VPSQSTKNIFIQ  150
              LTR  Q  +   I++  L  F PH  +C+IC VK+GRPKK+ KRKLS V+ + +++   +
Sbjct  2888   LTRFRQNKGSCGIQFCELSVFYPHDQDCKICCVKSGRPKKVGKRKLSKVAVDLSNQDNSD  2709

Query  151    QEHSYHSANKDSQDSSRPS----TETRDITSPESPSSFTSFSSIVVPSASTSCSFCPPVD  206
               + + +  + D  D SR S    T+ +D+++++S +    + ++ + +
Sbjct  2708   TDSASECCDLDCGDLSRTSSFDQTNKHDLSGSDSDNDTLLLKNLSLENE-----------  2562

Query  207    SPPLPPVDKLSVVESCSSETAPEMFKNVVIRSIPTERFCNAEVSQSFMCTICRGVPCDPY  266
                    V K ++++           F+NV+I S+  ERF +++++  F+C+ICRGVP  P
Sbjct  2561   ------VSKSNILN----------FNNVIIDSLCIERFWDPQIAGVFVCSICRGVPFVPV  2430

Query  267    ISKCSHIFCKECIFGWFSLSSACPVCRSLLDESEVSPLHGHLLQIYETLLVHCIHANCTQ  326
              I+ C+HIFC ECI +W ++SSACPVCR  LDE++V PL G+LL+IY++L+V C+H+NC
Sbjct  2429   ITSCNHIFCSECIRSWVNVSSACPVCRCCLDETDVLPLSGNLLHIYDSLVVKCVHTNCQA  2250

Query  327    SHLIRNIDEHESICSMKGTKFLYNVTTKSRVFKLPLHSVSAKHTRHRRLKPIISQVNEFC  386
                 ++NI  H+  C       +YN        KLPLH V++KH +HRRLKPIIS +++FC
Sbjct  2249   KVALKNIKLHDVSCQHTLPTQIYNFKKP----KLPLHEVTSKHVKHRRLKPIISSIDDFC  2082

Query  387    NAQEENKSDVLFFMLKDHLKEINDPRWKQVESLWLGNNSTLSPEQCLALRVDLLQSKGQY  446
              +++ ENK+DVLF +L++HLK+ +D+RWK+++ LW G++ T+S++QCLALRV+LLQSKGQY
Sbjct  2081   DTEGENKTDVLFSLLRNHLKDSSDSRWKDIDMLWQGSSDTMSADQCLALRVNLLQSKGQY  1902

Query  447    RSQYDFLSQNNVHVFQAPSKMESCENLFMPSASIFQIIDNDGNVLLQNS-ENPC-TEPLN  504
              + QYDFL  NNVHVFQ PS+++  E+ ++P+   + II++D   +++N    PC ++P+N
Sbjct  1901   KCQYDFLKSNNVHVFQCPSQLDKVESKYLPGHVEYSIIGDD---FFENIYKTPCSSDPIN  1731

Query  505    VNECFLPGFVELATPNCMGVRFSYFEALSLTLQELEPEILFGLKKHGLNIED--VLFLTT  562
              +   F   F E+++PNCMGVR SY +AL+ T++EL  E++ GLK H LN +D  +LFLTT
Sbjct  1730   ILGDFCTNFPEFPSPNCMGVRWSYTDALAKTVEELSEELVSGLKYHNLNPDDPSILFLTT  1551

Query  563    VKDGCDGMGEVSVYKEKDFKMLPDKVFRFSFCIVKIQAEYDGKLFDVFTEPLPNSVRTNR  622
              VKDG DGMG+ SVY+E+  + LPDK FRFSF +VKI+A   + +F VFTE+ PNSV+ N+
Sbjct  1550   VKDGGDGMGDCSVYRERSDRFLPDKAFRFSFSVVKIEAVSPSGIFTVFTESSPNSVKNNK  1371

Query  623    PLLESISDENNQVSNVVCILPIENEREILMQNRMHVKTKEGWMFHKFSFFNSMVDEKRDR  682
              PLLES++DENN+ +  VC+ PIENER  L ++ M VKT  GW  H F+FFNSMVDEKRDR
Sbjct  1370   PLLESLADENNKSTISVCMAPIENERKYLQNKIMKVKTSYGWHRHEFNFFNSMVDEKRDR  1191

Query  683    GDSGLQGSGSKYLCTLCDADKQSAKALLGSFSINRSVSECSNIAEILRVNPNALSENELK  742
              +D+GLQGSGSK+LCTLCDAD+++A   LGSF INRS+ E S+I E +R+NP+ LSE EL
Sbjct  1190   SDGGLQGSGSKFLCTLCDADRDTATQKLGSFEINRSIIETSKIYEYIRINPDKLSETELA  1011

Query  743    KISKGVKCAPLSKIEPIQKGIDATHADINLGQFFKKIIVREIAGVTKWELTQDVKPLVQN  802
              K SKG+K  P+S +EPI+KGIDATHADIN+G FFKKIIVREIA VTKWEL++D+K +V+N
Sbjct  1010   KKSKGIKSEPMSLMEPIKKGIDATHADINMGSFFKKIIVREIACVTKWELSEDIKEIVKN  831

Query  803    AEFLFDQHMKCNCGINPQLMMPGNYARTLFETPHSVLLEHISDSVRKENLSSILNIFLHL  862
              AE  FD HMK +CGINPQLMMPGNYART+F TPH +LL  ISD  R++NLS IL+ F  L
Sbjct  830    AESKFDFHMKEHCGINPQLMMPGNYARTIFTTPHDILLCLISDVERQQNLSVILDKFNFL  651

Query  863    RKVYRCKDPLTECPFDVQNYKKCAVEMGALLLQHFDYVEWPNYLHKVIEHVQQLIEDPNG  922
              RK+YR +DP ++ P D+  +K CAVEMG LLL +FDYV WPNYLHK+IEH QQLI+DP G
Sbjct  650    RKIYRSNDPKSDFPNDISLFKVCAVEMGKLLLSNFDYVRWPNYLHKLIEHTQQLIQDPAG  471

Query  923    PGSVGAFSSEGNEAGNKLFRHFRKNLSRRGNTYGSLCDVLKLHWLYSSKALFKLAEVEHK  982
              PG++G+FSSEGNEAGNKLFRHFRKNLSRRG+TYGSLCDVLKLHWLYSSKAL KLAE E+K
Sbjct  470    PGAIGSFSSEGNEAGNKLFRHFRKNLSRRGSTYGSLCDVLKLHWLYSSKALGKLAEKEQK  291
```

```
Query  983    KVRCSLCFTSGHNKRTCPLL  1002
              K +CSLCF +GHN+RTCPLL
Sbjct  290    KNKCSLCFQTGHNRRTCPLL  231
```

M.philippinarum (Mphi)

WGS: MJUU01034083.1

1. Summary

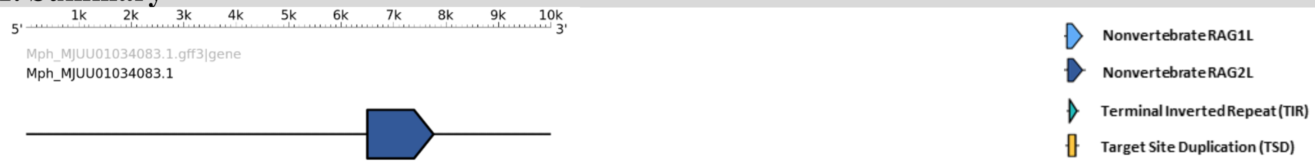

Detected configuration: RAG2L

Detected loci	Predicted CDS	start	end	strand	Observations
RAG2L	1	99677	101008	+	Complete, no stop codons

Selected predicted protein product	
MphRAG2L_B_Biv1_4083	MLMSFYRNFKALPFKSFSKYSTQKITRKRNDIHLQNND<br>FYPSEGHCALKCSENEFIFFGGARRLEESTWPSTCNIFKISFDIEDDDVS<br>IDSITQLKLIGSIFPPLQGSAAISINNSVYVWGGLNLETFKNTSELIELK<br>IQDGKCKVDIIQDIGQHSLSRRVMTGNVPSARCGHTMTPIDNSHAVLFGG<br>IEFVNKGCLLSFEHETVDDKFYIFDFNTFCWTPVAQLQVKPRAYHTATFV<br>NINNINSIVYIGGCLKKKSVIERISLSEIFALKILPDNQFQIDYLLYRNV<br>PDFGLSYHSSLWLGSVIFIFGGNTNDVLDGTQDIIILNNETMSANIITLE<br>SSFKCSGHSVIKVSDDCLMVCGGRQKQYFVYTSKPMVPGACDLHDDCKII<br>ESVETSPISWIQCEGVCKKWLHQFCVGVLNTVVRGKYMCKSCKSNSKSTR<br>SKKKRY<br><br>Prediction performed with FGENESH+ (C.elegans dataset and CviRAG2L_B_Biv1_0007 as homologous reference)

2. RAGL Detection info

Method: TBLASTN
Database searched: Whole-genome shotgun contigs (WGS) + only in Mollusca group (taxid: 6447)

2.1. RAG2L

Query: **CviRAG2L_B_Biv1_0007 (previously described)**
Subject: **MJUU01034083.1 Length: 156079 Number of Matches: 1**

Score	Expect	Method	Identities	Positives	Gaps	Frame
372 bits(956)	4e-113	Compositional matrix adjust.	203/435(47%)	282/435(64%)	10/435(2%)	+2

```
Query  1      MMSFYKQFRALPFKSHSTNPLRRITRK--SAASDDFNFFPPEGHVSLPINSYTYLYFGGA  58
              +MSFY+ F+ALPFKS S    ++ITRK         + +F+P EGH +L  +    +++FGGA
Sbjct  99680  LMSFYRNFKALPFKSFSKYSTQKITRKRNDIHLQNNDFYPSEGHCALKCSENEFIFFGGA  99859

Query  59     RRGQESTWNMSRNIYKisfivddndvnvdfisEIKLSGGQFPQLQSSAGFYVSAENCLFV  118
              RR +ESTW  + NI+KISF ++D+DV++D I+++KL G  FP LQ SA   +S  N ++V
```

```
Sbjct  99860   RRLEESTWPSTCNIFKISFDIEDDDVSIDSITQLKLIGSIFPPLQGSAA--ISINNSVYV  100033

Query  119     WGGLNLSCFSMSNELYIVKLNDNKGVVEIIQPPG--GISVRELGGEIPSGRCGHTLTHYF  176
               WGGLNL  F  ++EL  +K+ D K  V+IIQ  G   +S R + G +PS RCGHT+T
Sbjct  100034  WGGLNLETFKNTSELIELKIQDGKCKVDIIQDIGQHSLSRRVMTGNVPSARCGHTMTPID  100213

Query  177     DSCVILHGGVCFPHRNSCVGSSLFKNVTNDNNFYMFDFESLFWTKLSVTGSAPRAYHTAN  236
               +S  +L GG+ F ++  C+ S  F++ T D+ FY+FDF +  WT ++     PRAYHTA
Sbjct  100214  NSHAVLFGGIEFVNK-GCLLS--FEHETVDDKFYIFDFNTFCWTPVAQLQVKPRAYHTAT  100384

Query  237     VMEIRGMKSIVYIGGVTKTESALQRIPLSNVLVLKMDSNKHFHTEILTFANAPAVGISYH  296
                + I  + SIVYIGG  K +S ++RI LS +  LK+  +  F  + L + N P  G+SYH
Sbjct  100385  FVNINNINSIVYIGGCLKKKSVIERISLSEIFALKILPDNQFQIDYLLYRNVPDFGLSYH  100564

Query  297     SVGVIGPYIFVVGGLDEDNLQGRCSVSILNKDTFLCENVQFDRYFRSAGHSVCTLSDDCL  356
               S   +G  IF+ GG   D L G   + ILN +T     +  +  F+ +GHSV  +SDDCL
Sbjct  100565  SSLWLGSVIFIFGGNTNDVLDGTQDIIILNNETMSANIITLESSFKCSGHSVIKVSDDCL  100744

Query  357     MICGGMNLQYFVFSSKQMVPSPCDFDTECKIIESVETSPISWIQCEGACKRWLHQFCVGV  416
               M+CGG   QYFV++SK MVP  CD   +CKIIESVETSPISWIQCEG CK+WLHQFCVGV
Sbjct  100745  MVCGGRQKQYFVYTSKPMVPGACDLHDDCKIIESVETSPISWIQCEGVCKKWLHQFCVGV  100924

Query  417     LDIDMSRKNFICTTC  431
               L+  + R  ++C +C
Sbjct  100925  LNT-VVRGKYMCKSC  100966
```

Potentially pseudogenized copies

WGS: MJUU01020253.1

Summary

Detected configuration: RAG1L - RAG2L

Detected loci	Predicted CDS	start	end	strand	Observations
RAG1L	-	24459	30358	+	Pseudogenized (incomplete; fragmented; stop codons)
RAG2L	-	33773	32966	-	Pseudogenized (incomplete; stop codons)

WGS: MJUU01058305.1

Summary

Detected configuration: RAG2L

Detected loci	Predicted CDS	start	end	strand	Observations
RAG2L	-	18485	19078	+	Pseudogenized (incomplete; stop codons)

B. platifrons (Bpl)

Potentially pseudogenized copies

WGS: MJUT01007422.1

Summary

Detected configuration: RAG1L - RAG2L

Detected loci	Predicted CDS	start	end	strand	Observations
RAG1L	-	504446	507208	+	Pseudogenized (incomplete; fragmented; stop codons)
RAG2L	-	511141	510286	-	Pseudogenized (incomplete; fragmented; stop codons)

WGS: MJUT01028232.1

Summary

Detected configuration: RAG1L - RAG2L

Detected loci	Predicted CDS	start	end	strand	Observations
RAG1L	-	11345	9119	-	Pseudogenized (incomplete; fragmented; stop codons)
RAG2L	-	7322	7774	+	Pseudogenized (short fragment; stop codons)

WGS: MJUT01063358.1 copy 1

Summary

Detected configuration: RAG1L - RAG2L

Detected loci	Predicted CDS	start	end	strand	Observations
RAG1L	-	415930	418019	+	Pseudogenized (incomplete; fragmented; stop codons)
RAG2L	-	419792	418780	-	Pseudogenized (incomplete; fragmented; stop codons)

WGS: MJUT01063358.1 copy 2

Summary

Detected configuration: RAG1L - RAG2L

Detected loci	Predicted CDS	start	end	strand	Observations
RAG1L	-	501999	504203	+	Pseudogenized (incomplete; fragmented; stop codons)
RAG2L	-	506186	504891	-	Pseudogenized (incomplete; fragmented; stop codons)

WGS: MJUT01063358.1 copy 3

Summary

Detected configuration: RAG1L - RAG2L

Detected loci	Predicted CDS	start	end	strand	Observations
RAG1L	-	517228	519190	+	Pseudogenized (incomplete; fragmented; stop codons)
RAG2L	-	520252	519869	-	Pseudogenized (incomplete; fragmented; stop codons)

WGS: MJUT01050959.1

Summary

Detected configuration: RAG1L

Detected loci	Predicted CDS	start	end	strand	Observations
RAG1L	-	156785	156585	-	Pseudogenized (incomplete; short fragment)

WGS: MJUT01028541.1

Summary

Detected configuration: RAG2L

Detected loci	Predicted CDS	start	end	strand	Observations
RAG2L	-	26416	16851	-	Pseudogenized (incomplete; fragmented; stop codons)

Pinctada imbricata (Pim)

(TaxID: 66713; Protostomia; Mollusca; Bivalvia; Pterioida)

WGS: NIJJ01013975.1

1. Summary

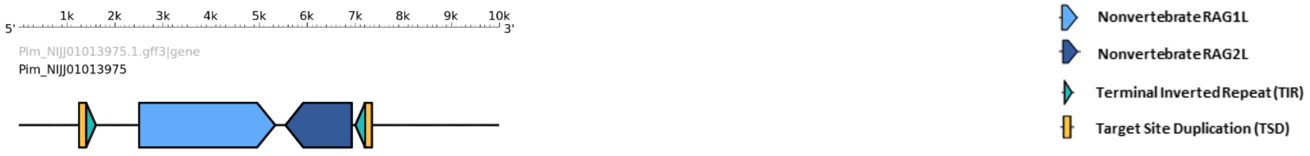

Detected configuration: TSD - TIR5 - RAG1L - RAG2L - TIR3 - TSD

Detected loci	Predicted CDS	start	end	strand	Observations
RAG1L	1	17416	20361	+	complete; no stop codons
RAG2L (!)	1	21932	20532	-	complete; no stop codons; potential longer N-ter

Predicted TIR	Distance from CDS (bp)	start	strand	TSD sequence	Assessment method
TIR5 (RAG1L side)	818	16598	+	5'-GTGAG-TIR	Homology variation
TIR3 (RAG2L side)	111	22043	-	TIR-GTGAG-3'	Homology variation

```
PimTIR5_3975            5'CACTTACGAACTTCTTTGCCTAAGATAAAGCC 3'
                          ||||||||||||  ||   |||||     ||
PimTIR3_3975 (revcmp)   3'CACTTACGAACTATTTATACTAAGTCCTTGCA 5'
```

Selected predicted protein product	
PimRAG1L_B_Biv2_3975	MLQIADQTSQVPACRAISMEASPHSEHAAHMEKLRNLCRLCGTK<br>IKGNKYLCKTLANKIAEVYNVNIVNDLADIHPEYLCGPCRCKMNRKTEGDFLKPYCFEEH<br>KFTGCMICNVHRGRPNKSKSACISRSDKADAQSSNDDIAFEKRPCSRHDESLSLDRINKR<br>EKSSCSRSVSSDPVNRDKYKKSSPSTNSTCNMSNTACKDISVEMDDVSFEKIEVTGTPAE<br>LYEQTHLADILKCSICLTVPKEPVYTVCQHIFCKSCIVAWCTVRQSCPVCRECIDVEQDL<br>LTLPNQLLVLFDTLTLKKNERGKSPGHFVENKVHLYSVSRKYAKQYRLRSVISDVGKFCE<br>THKENQTDVLFFMLRQRLEDLNDKRGEAIDSLWAGHFGSLSIDECLALRVDTLQSKGHYR<br>SQYQYLKQKGCLPFCTPHAVNNAEKNYLPQSVRFSVQANFFGHGDLYHTPLKPKEIALSH<br>EVTNDFKPINVTSCLSSPELPFPNLKGVRYNYVEAIAKTLEELDPYIKDGLVSNNIDPSD<br>PSLLLKTYIKDGADGMGEVSVYWEKSDCSLPDKAFRFAFAVVKVTTVASDGVEIVIFEEE<br>KPNSIRTNRPLLEAIADENHRSSSVFCMAPIETERSFLKDKILKVHMADGCRRHELKFYT<br>SMIDEKFDRAESGLEGSGSRYLCTLCTATREEAKSALGTFSITRTFAETKSVAEYIKVNP<br>DKLSQTQLDKLSYGVKSVPILTADAIEKGIDATHSDINMALFFKKVILREIAGVSVWELN<br>QDIKPLICEAETKFDKVMRSTTGIHPALMMPGNYARIMFDDKNEKHVTCLITCQVRRSLM<br>NELLSLFRKLRKIYRSRSPKVDYPDEVKAYKKIAVSMGQFLLQNYSYIEWPNYLHKIIEH<br>VQELIESVDGPGCIGSFSGEGNEGGNKVFRFFRKNLSRKGDVIGGLQDVLQLQWLYSSKK<br>LQALSNATQKQNRCSFCFELGHKRPSCPNKEKSDDFVE

Prediction performed with FGENESH+ (C.elegans dataset and CviRAG1L_B_Biv1_0007 as homologous reference)

PimRAG2L_B_Biv1_3975(!)

```
MAASTIHSVN
DSDAQFLPFKSSVPNKLKITRKRSEVELKNGDFYPGEGHKYIELHQDDVSEYYFLGGVRR
GEDAAWVDSSKIMKIKIVSTDDDIYIESIENVVTKGSMIPTLTSSGMVWGAEPGKYFVIW
GGLDVLNYTVVDDLYICKPVVERKKQIFDITQYTGSAKSFGQYGDHGKIQTNSPTARAGH
SLNKLPGKANVAIMFGGYEMPNREKEMAFAEPFAQVCVDGRFHMLKINGESMEWSVLPVP
SVKPRCFHTADFISDDKLAIVGGLAYTKNTASHRLPLHDVTVLYILNYQTNDYQMYSVLF
DMQPTFISFQSSCVVDGILYIFGGYLQSKEELDPDQDKIVSCKLFKLDFFTCKSSECDAG
SDYLSCGGSLLNVEPQTLLIVSGCFQKYYVYTSKPFVPGPCDLAQHGMCQIKDTPETSPI
QWVQCEGGCKQWHHFFCTGLKRIPKGKYLCKLCSKKK
```

Prediction performed with FGENESH+ (C.elegans dataset and CviRAG2L_B_Biv1_0007 as homologous reference)

(N-terminal could be longer by going beyond TIR3 location; also, this potential extension is not homologues with other RAG2Ls…)

2. RAGL Detection info

Method: TBLASTN
Database searched: Whole-genome shotgun contigs (WGS) + only in Mollusca group (taxid: 6447)

2.1. RAG1L

Query: **CviRAG1L_B_Biv1_0007 (previously described)**
Subject: **NIJJ01013975.1 Length: 23523 Number of Matches: 1**

Score	Expect	Method	Identities	Positives	Gaps	Frame
714 bits(1843)	0.0	Compositional matrix adjust.	405/993(41%)	572/993(57%)	75/993(7%)	+

```
Query  33     HGLKLKCMCRLCGVKIPINSKSHPKENFKSAITSLYNIDIDCDSENIHPPKICTACRLRL  92
              H  KL+ +CRLCG KI  N   +  +   + I  +YN++I  D  +IHP  +C  CR ++
Sbjct  17503  HMEKLRNLCRLCGTKIKGNK--YLCKTLANKIAEVYNVNIVNDLADIHPEYLCGPCRCKM  17676

Query  93     TRHSQAEDDIEINLPVLFEFLPHSI-NCNICFVKPGRPKK----ILKRKLSVPSQSTKNI  147
               R ++ +       P  FE   H    C IC V  GRP K     + R     +QS+ N
Sbjct  17677  NRKTEGD----FLKPYCFE--EHKFTGCMICNVHRGRPNKSKSACISRSDKADAQSS-ND  17835

Query  148    FIQQEHSYHSANKDSQDSSRPSTETRDitspespssftsfssivvpsASTSCSFCppvds  207
               I  E    S + +S    R +   +   S    S   +       S ST+ S C   ++
Sbjct  17836  DIAFEKRPCSRHDESLSLDRINKREKSSCSRSVSSDPVNRDKYKKSSPSTN-STCNMSNT  18012

Query  208    pplppvdklsvvESCSSETAPEMFKNVVIRSIPTERFCNAEVSQSFMCTICRGVPCDPYI  267
                          +  S E     F+ + +   P E +    ++    C+IC  VP +P
Sbjct  18013  AC----------KDISVEMDDVSFEKIEVTGTPAELYEQTHLADILKCSICLTVPKEPVY  18162

Query  268    SKCSHIFCKECIFGWFSLSSACPVCRSLLD-ESEVSPLHGHLLQIYETLLVHCIHANCTQ  326
              + C HIFCK CI  W ++  +CPVCR  +D E ++  L   LL +++TL +       +
Sbjct  18163  TVCQHIFCKSCIVAWCTVRQSCPVCRECIDVEQDLLTLPNQLLVLFDTLTLKKNERGKSP  18342

Query  327    SHLIRNIDEHESICSMKGTKFLYNVTTKSRVFKLPLHSVSAKHTRHRRLKPIISQVNEFC  386
               H + N                          K+ L+SVS K+ +  RL+ +IS V +FC
Sbjct  18343  GHFVEN--------------------------KVHLYSVSRKYAKQYRLRSVISDVGKFC  18444

Query  387    NAQEENKSDVLFFMLKDHLKEINDPRWKQVESLWLGNNSTLSPEQCLALRVDLLQSKGQY  446
```

```
                   +EN++DVLFFML+  L+++ND R + ++SLW G+  +LS ++CLALRVD LQSKG Y
Sbjct  18445  ETHKENQTDVLFFMLRQRLEDLNDKRGEAIDSLWAGHFGSLSIDECLALRVDTLQSKGHY  18624

Query  447    RSQYDFLSQNNVHVFQAPSKMESCENLFMPSASIFQIIDN-------------DGNVLLQ  493
              RSQY +L Q     F  P  + + E  ++P +  F +  N                + L
Sbjct  18625  RSQYQYLKQKGCLPFCTPHAVNNAEKNYLPQSVRFSVQANFFGHGDLYHTPLKPKEIALS  18804

Query  494    NSENPCTEPLNVNECFLPGFVELATPNCMGVRFSYFEALSLTLQELEPEILFGLKKHGLN  553
              +      +P+NV  C      EL  PN  GVR++Y EA++ TL+EL+P I  GL  + ++
Sbjct  18805  HEVTNDFKPINVTSCL--SSPELPFPNLKGVRYNYVEAIAKTLEELDPYIKDGLVSNNID  18978

Query  554    IED--VLFLTTVKDGCDGMGEVSVYKEKDFKMLPDKVFRFSFCIVKIQA-EYDGKLFDVF  610
                D  +L  T +KDG DGMGEVSVY EK    LPDK FRF+F +VK+     DG    +F
Sbjct  18979  PSDPSLLLKTYIKDGADGMGEVSVYWEKSDCSLPDKAFRFAFAVVKVTTVASDGVEIVIF  19158

Query  611    TEPLPNSVRTNRPLLESISDENNQVSNVVCILPIENEREILMQNRMHVKTKEGWMFHKFS  670
               E  PNS+RTNRPLLE+I+DEN++ S+V C+ PIE ER  L    + V   +G   H+
Sbjct  19159  EEEKPNSIRTNRPLLEAIADENHRSSSVFCMAPIETERSFLKDKILKVHMADGCRRHELK  19338

Query  671    FFNSMVDEKRDRGDSGLQGSGSKYLCTLCDADKQSAKALLGSFSINRSVSECSNIAEILR  730
              F+ SM+DEK DR +SGL+GSGS+YLCTLC A ++ AK+ LG+FSI R+ +E  ++AE ++
Sbjct  19339  FYTSMIDEKFDRAESGLEGSGSRYLCTLCTATREEAKSALGTFSITRTFAETKSVAEYIK  19518

Query  731    VNPNALSENELKKISKGVKCAPLSKIEPIQKGIDATHADINLGQFFKKIIVREIAGVTKW  790
              VNP+ LS+ +L K+S GVK  P+   + I+KGIDATH+DIN+  FFKK+I+REIAGV+ W
Sbjct  19519  VNPDKLSQTQLDKLSYGVKSVPILTADAIEKGIDATHSDINMALFFKKVILREIAGVSVW  19698

Query  791    ELTQDVKPLVQNAEFLFDQHMKCNCGINPQLMMPGNYARTLFET---PHSVLLEHISDSV  847
              EL QD+KPL+  AE  FD+ M+   GI+P LMMPGNYAR +F+     H   L  I+  V
Sbjct  19699  ELNQDIKPLICEAETKFDKVMRSTTGIHPALMMPGNYARIMFDDKNEKHVTCL--ITCQV  19872

Query  848    RKENLSSILNIFLHLRKVYRCKDPLTECPFDVQNYKKCAVEMGALLLQHFDYVEWPNYLH  907
              R+  ++ +L++F  LRK+YR + P  + P +V+ YKK AV MG  LLQ++ Y+EWPNYLH
Sbjct  19873  RRSLMNELLSLFRKLRKIYRSRSPKVDYPDEVKAYKKIAVSMGQFLLQNYSYIEWPNYLH  20052

Query  908    KVIEHVQQLIEDPNGPGSVGAFSSEGNEAGNKLFRHFRKNLSRRGNTYGSLCDVLKLHWL  967
              K+IEHVQ+LIE  +GPG +G+FS EGNE GNK+FR FRKNLSR+G+  G L DVL+L WL
Sbjct  20053  KIIEHVQELIESVDGPGCIGSFSGEGNEGGNKVFRFFRKNLSRKGDVIGGLQDVLQLQWL  20232

Query  968    YSSKALFKLAEVEHKKVRCSLCFTSGHNKRTCP  1000
              YSSK L  L+    K+ RCS CF  GH + +CP
Sbjct  20233  YSSKKLQALSNATQKQNRCSFCFELGHKRPSCP  20331
```

2.2 RAG2L

Query: CviRAG2L_B_Biv1_0007 (previously described)
Subject: NIJJ01013975.1 Length: 23523 Number of Matches: 1

Score	Expect	Method	Identities	Positives	Gaps	Frame
175 bits(444)	3e-45	Compositional matrix adjust.	143/458(31%)	226/458(49%)	48/458(10%)	-2

```
Query  11     LPFKSHSTNPLRRITRKSAASD--DFNFFPPEGHVSLPINS---YTYLYFGGARRGQEST  65
              LPFKS   N L+ ITRK +  +  + +F+P EGH  + ++      Y + GG RRG+++
Sbjct  21884  LPFKSSVPNKLK-ITRKRSEVELKNGDFYPGEGHKYIELHQDDVSEYYFLGGVRRGEDAA  21708

Query  66     WNMSRNIYKisfivddndvnvdfisEIKLSGGQFPQLQSSAGFYVSAENCLFV-WGGLNL  124
```

```
               W   S   I KI   +   D+D+ ++ I   +    G    P L SS    + +      FV WGGL++
Sbjct  21707  WVDSSKIMKIKIVSTDDDIYIESIENVVTKGSMIPTLTSSGMVWGAEPGKYFVIWGGLDV  21528

Query  125    SCFSMSNELYIVK--LNDNKGVVEIIQPPGGISVRELG-----GEI----PSGRCGHTLT  173
                +++ ++LYI K   +    K + +I Q   G   S +  G      G+I      P+ R GH+L
Sbjct  21527  LNYTVVDDLYICKPVVERKKQIFDITQYTG--SAKSFGQYGDHGKIQTNSPTARAGHSLN  21354

Query  174    HY--FDSCVILHGGVCFPHRNSCVG-SSLFKNVTNDNNFYMFDF--ESLFWTKLSVTGSA  228
                      +  I+ GG   P+R    +  +  F  V  D  F+M      ES+ W+ L V
Sbjct  21353  KLPGKANVAIMFGGYEMPNREKEMAFAEPFAQVCVDGRFHMLKINGESMEWSVLPVPSVK  21174

Query  229    PRAYHTANVMEIRGMKSIVYIGGVTKTE-SALQRIPLSNVLVLKM---DSNKHFHTEILT  284
               PR +HTA+ +         +  +GG+  T+ +A  R+PL +V VL +    +N +    +L
Sbjct  21173  PRCFHTADFI---SDDKLAIVGGLAYTKNTASHRLPLHDVTVLYILNYQTNDYQMYSVL-  21006

Query  285    FANAPAVGISYHSVGVIGPYIFVVGG-------LDEDNLQGRCSVSILNKDTFLCENVQF  337
               F   P   IS+ S  V+   +++ GG       LD D      S  +   D F C++ +
Sbjct  21005  FDMQPTF-ISFQSSCVVDGILYIFGGYLQSKEELDPDQ-DKIVSCKLFKLDFFTCKSSEC  20832

Query  338    D--RYFRSAGHSVCTLSDDCLMICGGMNLQYFVFSSKQMVPSPCDFDTE--CKIIESVET  393
               D     + S G S+  +     L+I  G   +Y+V++SK  VP PCD       C+I ++ ET
Sbjct  20831  DAGSDYLSCGGSLLNVEPQTLLIVSGCFQKYYVYTSKPFVPGPCDLAQHGMCQIKDTPET  20652

Query  394    SPISWIQCEGACKRWLHQFCVGVLDIDMSRKNFICTTC  431
               SPI W+QCEG CK+W H FC G+   I    +  ++C  C
Sbjct  20651  SPIQWVQCEGGCKQWHHFFCTGLKRI--PKGKYLCKLC  20544
```

3. TIR Detection info

Aligned P.imbricata RAG1L-RAG2L flanking regions had been searched for homology variations both at left and right flanking regions. Detected homology dropping regions were inspected for TIR and TSD presence. For more details please see methods section.

A. RAG1L facing margins alignment (left)

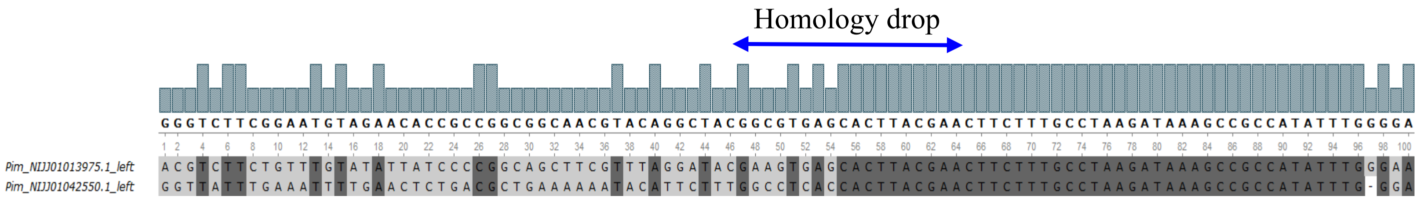

B. RAG2L facing margins alignment (right)

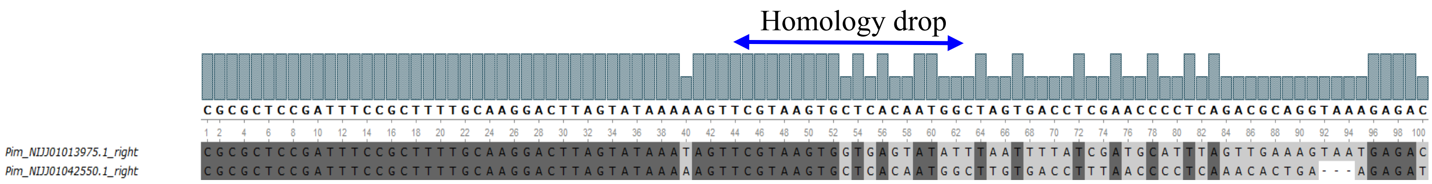

C. Alignment of Left homology drop regions with the reverse complement of right homology drop region in order to spot inverted repeat regions.

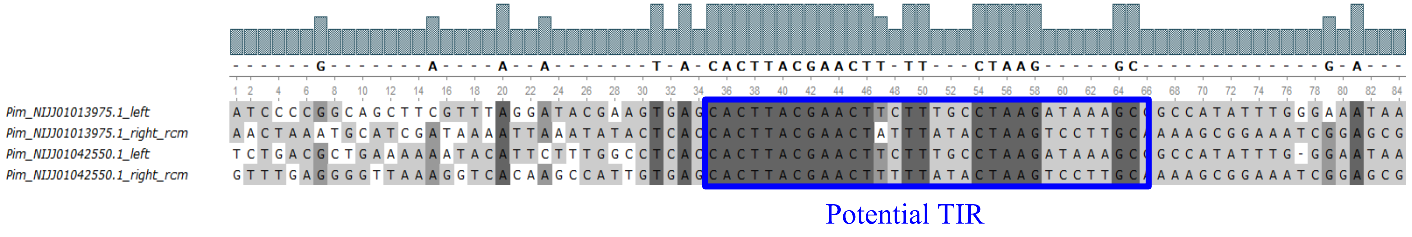

D. TSD pairs in NIJJ01013975.1:

RAG1L side:

```
        TSD       TIR5
5′ - ATACGAAGTGAGCACTTACGAACTTCTTTGCCTAAGATAAAGC - 3′
```

RAG2L side:

```
                         TSD
5′ - CTTAGTATAAATAGTTCGTAAGTGGTGAGTATATTTAATTTTAT - 3′
3′ - GAATCATATTTATCAAGCATTCACCACTCATATAAATTAAAATA - 5′
        TIR3
```

Pinctada imbricata (Pim)

WGS: NIJJ01042550.1

1. Summary

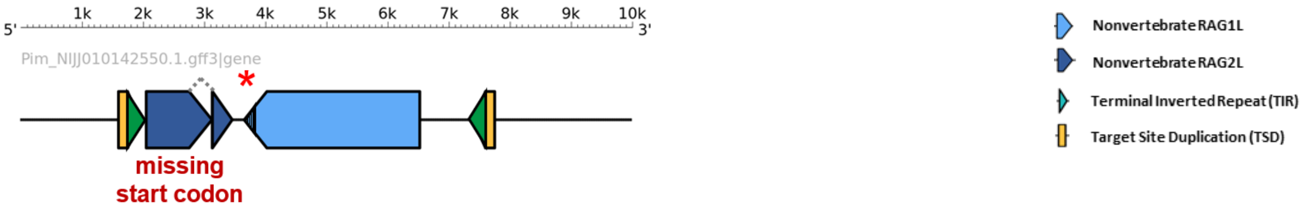

Detected configuration: TSD - TIR5 - RAG1L - RAG2L - TIR3 - TSD

Detected loci	Predicted CDS	start	end	strand	Observations
RAG1L (!)	1	6527	3772	-	Almost complete; early stop codon
RAG2L (!)	1	2060	3133	+	missing start codon; otherwise complete; no stop codons
	2	3136	3459		

Predicted TIR	Distance from CDS (bp)	start	strand	TSD sequence	Assessment method
TIR5 (RAG1L side)	864	7391	-	5'- CTCAC -TIR5	Homology variation
TIR3 (RAG2L side)	111	1949	+	TIR3- CTCAC -3'	Homology variation

```
PimTIR5_2550                           5’CACTTACGAACTTCTTTGCCTAAGATAAAGCCGCC 3’
                                         ||||||||||||| ||   |||||     ||
PimTIR3_2550 (reverse complement)      3’CACTTACGAACTTTTTATACTAAGTCCTTGCAAAA 5’
```

Selected predicted protein product	
PimRAG1L_B_Biv2_2550<br>(!)	MSMEASPHSEHAAHMEKLRNLCR<br>LCGTKIKGNKYLCKTLANIIAEVYNVNIVNDLADIHPEYLCGPCRCKMNRKTEGDFLKQY<br>CFEERKFTGCMICNVHRGRPNKRKSACISRSDKADAQSSNDDIAFEKRPCSRHDESLSLD<br>RLNKCEKSSCSRSVSSDPVNSDKYKKSSPSTTSTCNMSNTACKDISVEMDDVSFEKVEVT<br>GTPVELYEQTHLAEILKCSICLTVPKEPVYTVCQHIFCKSCIVAWCTVRQSCPVCRGCID<br>VEQDLLPLPNQLLVLFDTLTLKKNERGKSPGHFVENKVPLYSVSRKYAKQYRLRSVISDV<br>GKFCETHKENQTDVLFFMLRQRLEDLNDKSGEAIDSLWAEHFGSLSIDECLALRVDTLQS<br>KSHYRSQYQYLKQKGCLTFCTPHAVNNAEKNYLPQSVRFSVQANFFGHGDLYHTPLKPKE<br>IALSHEVTNDFKPINVTSCLSSPELPFPNLKGVRYNYVEAIAKTLEELDPYIKDGLVSNK<br>IDPSDPSLLLKTYIKDGADGMGEVSVYWEKSDCSLPDKAFRFAFAVVKVTRVASDGVEIV<br>IFEEEKPNSIRTNRPLLEAIADENHRSSSVFCMAPIETERSFLKDKILKVHMADGCRRHE<br>LKFYTSMIDEKFDRAESGLGGSGSRYLCTLCTATREKAKSALGTFSITRTFAETKSVAEY<br>IKVNPDKLSQTQLDKLSYGVKSVPILTADAIEKGIDATHSDINMALFFKKVILREIAGVS<br>VWELNQDIKPLICEAETKFDKVMRSTTGIHPALMMPGNYARIMFDEKNEKHVTCLITCQV<br>RRSLMNELLSLFRKLRKIYRSRSPKVDYPDEVKAYKKVAVSMGQFLLQNYSYIEWPNYLH<br>KIIEHVQELIESVDGPGCIGSFSGEGNEGGIKVFRFFRKNLSRKGDVIWGLQDVL*<br><br>Prediction performed with FGENESH+ (C.elegans dataset and CviRAG1L_B_Biv1_0007 as homologous reference)
PimRAG2L_B_Biv1_2550<br>(!)	Not further used as multiple issues:<br>– multiple exon merging solutions<br>– The region that shares homology with other RAG2Ls does not have a potential start codon inside the cassette (between the presumed TIRs). However, if we consider the first potential start codon, the extended N-terminal does not share homology with other RAG2Ls and it goes beyond the predicted TIR (see Additional-file-9-File-S2 region indicated with yellow highlight)

2. RAGL Detection info

Method: TBLASTN
Database searched: Whole-genome shotgun contigs (WGS) + only in Mollusca group (taxid: 6447)

2.1. RAG1L

Query: **CviRAG1L_B_Biv1_0007 (previously described)**
Subject: **NIJJ01042550.1 Length: 17559 Number of Matches: 1**

Score	Expect	Method	Identities	Positives	Gaps	Frame
708 bits(1827)	0.0	Compositional matrix.	401/990(41%)	567/990(57%)	69/990(6%)	-2

```
Query  33    HGLKLKCMCRLCGVKIPINSKSHPKENFKSAITSLYNIDIDCDSENIHPPKICTACRLRL  92
             H  KL+ +CRLCG KI  N    +    + I  +YN++I  D  +IHP  +C  CR ++
Sbjct  6488  HMEKLRNLCRLCGTKIKGNK--YLCKTLANIIAEVYNVNIVNDLADIHPEYLCGPCRCKM  6315

Query  93    TRHSQAEDDIEINLPVLFEFLPHSINCNICFVKPGRPKKILKRKLSVPSQSTKNIFIQQE  152
              R ++ +           FE    +  C IC V  GRP K   RK +  S+S K
Sbjct  6314  NRKTEGD----FLKQYCFEERKFT-GCMICNVHRGRPNK---RKSACISRSDKA------  6177
```

```
Query  153   HSYHSANKDSQDSSRPSTETRDitspespssftsfssivvpsAS--TSCSFCppvdsppl  210
                 S+N D      RP +    +   S +   +       S       S+     S   +       S
Sbjct  6176  -DAQSSNDDIAFEKRPCSRHDESLSLDRLNKCEKSSCSRSVSSDPVNSDKYKKSSPSTTS  6000

Query  211   ppvdklsvvESCSSETAPEMFKNVVIRSIPTERFCNAEVSQSFMCTICRGVPCDPYISKC  270
                   +  +  S E      F+ V +    P E +     +++    C+IC   VP +P   + C
Sbjct  5999  TCNMSNTACKDISVEMDDVSFEKVEVTGTPVELYEQTHLAEILKCSICLTVPKEPVYTVC  5820

Query  271   SHIFCKECIFGWFSLSSACPVCRSLLD-ESEVSPLHGHLLQIYETLLVHCIHANCTQSHL  329
              HIFCK CI   W ++   +CPVCR  +D E ++ PL    LL +++TL +          +   H
Sbjct  5819  QHIFCKSCIVAWCTVRQSCPVCRGCIDVEQDLLPLPNQLLVLFDTLTLKKNERGKSPGHF  5640

Query  330   IRNIDEHESICSMKGTKFLYNVTTKSRVFKLPLHSVSAKHTRHRRLKPIISQVNEFCNAQ  389
             + N                            K+PL+SVS K+ +   RL+ +IS V +FC
Sbjct  5639  VEN--------------------------KVPLYSVSRKYAKQYRLRSVISDVGKFCETH  5538

Query  390   EENKSDVLFFMLKDHLKEINDPRWKQVESLWLGNNSTLSPEQCLALRVDLLQSKGQYRSQ  449
             +EN++DVLFFML+   L+++ND     + ++SLW   +   +LS ++CLALRVD LQSK   YRSQ
Sbjct  5537  KENQTDVLFFMLRQRLEDLNDKSGEAIDSLWAEHFGSLSIDECLALRVDTLQSKSHYRSQ  5358

Query  450   YDFLSQNNVHVFQAPSKMESCENLFMPSASIFQIIDN-------------DGNVLLQNSE  496
             Y +L Q      F   P   + + E   ++P +   F +   N                   + L +
Sbjct  5357  YQYLKQKGCLTFCTPHAVNNAEKNYLPQSVRFSVQANFFGHGDLYHTPLKPKEIALSHEV  5178

Query  497   NPCTEPLNVNECFLPGFVELATPNCMGVRFSYFEALSLTLQELEPEILFGLKKHGLNIED  556
                 +P+NV   C        EL   PN   GVR++Y EA++ TL+EL+P I   GL   + ++   D
Sbjct  5177  TNDFKPINVTSCL--SSPELPFPNLKGVRYNYVEAIAKTLEELDPYIKDGLVSNKIDPSD  5004

Query  557   --VLFLTTVKDGCDGMGEVSVYKEKDFKMLPDKVFRFSFCIVKI-QAEYDGKLFDVFTEP  613
               +L   T +KDG DGMGEVSVY EK     LPDK FRF+F +VK+ +    DG     +F E
Sbjct  5003  PSLLLKTYIKDGADGMGEVSVYWEKSDCSLPDKAFRFAFAVVKVTRVASDGVEIVIFEEE  4824

Query  614   LPNSVRTNRPLLESISDENNQVSNVVCILPIENEREILMQNRMHVKTKEGWMFHKFSFFN  673
              PNS+RTNRPLLE+I+DEN++ S+V C+ PIE ER  L     + V    +G    H+   F+
Sbjct  4823  KPNSIRTNRPLLEAIADENHRSSSVFCMAPIETERSFLKDKILKVHMADGCRRHELKFYT  4644

Query  674   SMVDEKRDRGDSGLQGSGSKYLCTLCDADKQSAKALLGSFSINRSVSECSNIAEILRVNP  733
             SM+DEK DR +SGL GSGS+YLCTLC A ++ AK+ LG+FSI R+ +E  ++AE ++VNP
Sbjct  4643  SMIDEKFDRAESGLGGSGSRYLCTLCTATREKAKSALGTFSITRTFAETKSVAEYIKVNP  4464

Query  734   NALSENELKKISKGVKCAPLSKIEPIQKGIDATHADINLGQFFKKIIVREIAGVTKWELT  793
             + LS+ +L K+S GVK  P+    + I+KGIDATH+DIN+   FFKK+I+REIAGV+ WEL
Sbjct  4463  DKLSQTQLDKLSYGVKSVPILTADAIEKGIDATHSDINMALFFKKVILREIAGVSVWELN  4284

Query  794   QDVKPLVQNAEFLFDQHMKCNCGINPQLMMPGNYARTLFE---TPHSVLLEHISDSVRKE  850
             QD+KPL+   AE   FD+ M+    GI+P LMMPGNYAR +F+      H    L   I+   VR+
Sbjct  4283  QDIKPLICEAETKFDKVMRSTTGIHPALMMPGNYARIMFDEKNEKHVTCL--ITCQVRRS  4110

Query  851   NLSSILNIFLHLRKVYRCKDPLTECPFDVQNYKKCAVEMGALLLQHFDYVEWPNYLHKVI
              ++ +L++F   LRK+YR + P   + P +V+ YKK AV MG  LLQ++ Y+EWPNYLHK+I
Sbjct  4109  LMNELLSLFRKLRKIYRSRSPKVDYPDEVKAYKKVAVSMGQFLLQNYSYIEWPNYLHKII  3930

Query  911   EHVQQLIEDPNGPGSVGAFSSEGNEAGNKLFRHFRKNLSRRGNTYGSLCDVLKLHWLYSS  970
             EHVQ+LIE   +GPG +G+FS EGNE G K+FR FRKNLSR+G+     L DVL LHWLYSS
Sbjct  3929  EHVQELIESVDGPGCIGSFSGEGNEGGIKVFRFFRKNLSRKGDVIWGLQDVL*LHWLYSS  3750

Query  971   KALFKLAEVEHKKVRCSLCFTSGHNKRTCP  1000
             K L  L+     K+ RCS CF   GH + +CP
Sbjct  3749  KKLQALSNATQKQNRCSFCFELGHKRPSCP  3660
```

2.2 RAG2L

Query: CviRAG2L_B_Biv1_0007 (previously described)
Subject: NIJJ01042550.1 Length: 17559 Number of Matches: 2

Score	Expect	Method	Identities	Positives	Gaps	Frame
99.8 bits(247)	1e-34	Compositional matrix adjust.	103/355(29%)	168/355(47%)	40/355(11%)	+2

```
Query  11    LPFKSHSTNPLRRITRKSAASD--DFNFFPPEGHVSLPINS---YTYLYFGGARRGQEST   65
             LPFKS   N L+ ITR  +  +  + +F+P EGH  + ++      Y + GG RRG+++
Sbjct  2108  LPFKSSVPNKLK-ITRNRSEVELKNGDFYPGEGHKYIELHQDDVSEYYFLGGVRRGEDAA   2284

Query  66    WNMSRNIYKisfivddndvnvdfisEIKLSGGQFPQLQSSAGFYVSAENCLFV-WGGLNL   124
             W  S  I KI  +  D+D+ V+ I  +   G   P L SS   + +     FV WGGL++
Sbjct  2285  WVDSSKIMKIKIVSTDDDIYVESIENVLTKGSMIPTLTSSGMVWGAEPGEYFVIWGGLDV   2464

Query  125   SCFSMSNELYIVK--LNDNKGVVEIIQPPGGISVRELG-----GEI----PSGRCGHTLT   173
               +++ ++LYI K  +   K + +I Q  G  S +  G     G+I    P+ R GH+L
Sbjct  2465  LNYTIVDDLYICKSIVERKKQIFDITQYTG--SAKSYGQYGDHGKIQTNSPTARGGHSL-   2635

Query  174   HYFD---SCVILHGGVCFPHRNSCVG-SSLFKNVTNDNNFYMFDF--ESLFWTKLSVTGS   227
             H      +  I+ GG   P+R   +  +  F  V  D  F+M     ES+ W+ L V
Sbjct  2636  HKLPGKANVAIMFGGYEMPNREKEMAFAEPFAQVCVDGRFHMLKINGESMEWSVLPVPSV   2815

Query  228   APRAYHTANVMEIRGMKSIVYIGGVTKTE-SALQRIPLSNVLVLKM---DSNKHFHTEIL   283
              PR +HTA+ +       +  +GG+  T+ +A  R+PL +V VL +    +N +    +L
Sbjct  2816  KPRCFHTADFI---SDDKLAIVGGLAYTKNTASHRLPLHDVTVLYILNCQTNDYQMYSVL   2986

Query  284   TFANAPAVGISYHSVGVIGPYIFVVGGLDEDNLQGRCSVSILNKDTFLCENVQFD   338
              F   P   IS+    V+   +++ GG     LQ +  +      T  C+  + D
Sbjct  2987  -FDMQPTF-ISFQGSCVVDGILYIFGGY----LQSKEELDPDQDKTVSCKLFKLD   3133
```

Score	Expect	Method	Identities	Positives	Gaps	Frame
75.9 bits(185)	1e-34	Compositional matrix adjust.	40/106(38%)	59/106(55%)	6/106(5%)	+1

```
Query  330   FLCENVQFD--RYFRSAGHSVCTLSDDCLMICGGMNLQYFVFSSKQMVPSPCDFDTE--C   385
             F C++ + D    + S G S+  +    L+I  G   +Y+V++SK  VP PCD      C
Sbjct  3136  FTCKSSECDAGSDYLSCGGSLLNVEPQTLLIVSGCCQKYYVYTSKPFVPGPCDLAQHGMC   3315

Query  386   KIIESVETSPISWIQCEGACKRWLHQFCVGVLDIDMSRKNFICTTC   431
             +I  + ETSPI W+QCEG CK+W H FC G+  I   +  ++C  C
Sbjct  3316  QIKATPETSPIQWVQCEGGCKQWHHFFCTGLKRI--PKGKYVCKLC   3447
```

3. TIR Detection info

See scaffold WGS: NIJJ01013975.1 (page 33).
TSD pairs in NIJJ01042550.1:

RAG1L side:

```
                    TSD          TIR5
5’ - AATACATTCTTTGGCCTCACCACTTACGAACTTCTTTGCCTAAGAT - 3’
```

RAG2L side:

```
                                TSD
5’ - GACTTAGTATAAAAAGTTCGTAAGTGCTCACAATGGCTTGTGACCT- 3’
3’ - CTGAATCATATTTTTCAAGCATTCACGAGTGTTACCGAACACTGGA- 5’
               TIR3
```

Pinctada imbricata (Pim)

WGS: NIJJ01005135.1

1. Summary

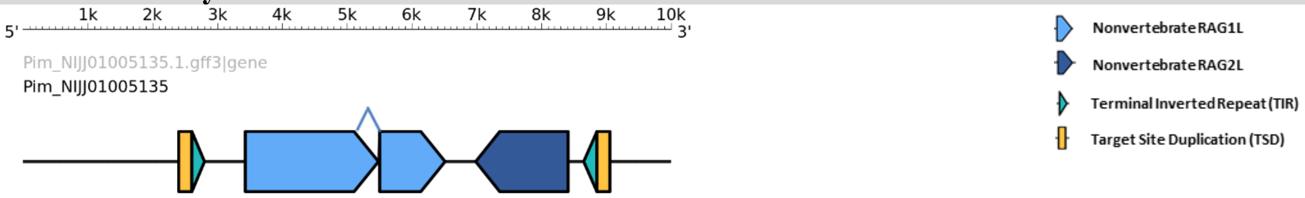

Detected configuration: TSD - TIR5 - RAG1L - RAG2L - TIR3 - TSD

Detected loci	Predicted CDS	start	end	strand	Observations
RAG1L	1	7626	9680	+	complete; no stop codons
	2	9700	10710		
RAG2L	1	12607	11186	-	complete; no stop codons

Predicted TIR	Distance from CDS (bp)	start	strand	TSD sequence	Assessment method
TIR5 (RAG1L side)	826	6800	+	5'-TAGTA-TIR	Homology variation
TIR3 (RAG2L side)	56	12663	-	TIR-TAGTA-3'	Homology variation

```
PimTIR5_5135                          5'CACTAACAAACGTCTATGCAACTTTTGCTTTT 3'
                                        ||||||||||||| ||    |||      |     |
PimTIR3_5135 (reverse complement)     3'CACTAACAAACGTTTACAGTACTCGCTATGCT 5'
```

Selected predicted protein product	
PimRAG1L_B_Biv2_5135	MEAADEMQ<br>KDADNIANHFANLTKFCRVCGSIQKRKSRAFCPVEPYKIELLSLFKIDIQYDDESIHPKK<br>LCNSCRSKCFHFRTKKGEGNTFSMNIPVKHLYIPHNANCKVCHRNSGRNATIELFQIHYN<br>PSSDVPIEHVDGGGTCSNTESDSEDQGSNDHSHSFSATCHVAPSTSSHFFAAEMDKSEIY<br>ASSLSENSNELCEPASKKLCRPKGLLDVTKQLDFDSTVHVPPSFTVTDLSSIPIEQAIEQ<br>SLAKIFQCTICLGIPTAPAITHCKHIFCRSCISSWLLNAGVCPICREPVMMNEISILTGQ<br>LSLMYDILHVSCRFDNCNEVLGLDKISDHENVCKYGKYPSKLLTPSSIVRRGKGLQKMPI<br>YDCKAKYVKQKRLKEVESSVEQFCSSKNEDKTDVLFFMLIHHLKSQNDNRFEQINDVWMG<br>GGVRKLTSHECLAIRIDTLQSKTQYKAQYDFLKAKGCNPFVAPYALDEIECQYVPGSVRY<br>SIEGQDQVINYYHTPVKVNRECTTSPLDNTFEPLDIVNVFPACTPELATPNVKGCRWRYP<br>DAIAKTLHELENDIIAGLQQHNIDPYDPTILLKTYIKDGADGLGDVSVHKEVGDRYLPDK<br>AFRFSFCVYRIEAILGDDEKVTIFVEKRPNSVKTNRPVLEAICDENNHGSSTLCLLPIEN<br>ERSYMKGKIIRIQTE[FG|FSNSMIGEKLDR]ANSGLAGSGSNYLCTLCDATRETALS<br>NLGNFVINRSYAETAEIAEYMRINPDNLTKADLNKISKGIKSTPLLQAESIEKGIDAT<br>HADINCGSFFRKLIIREIVQINKWEMSGDVKSIIKDGEVKFDLHLKKTVGINPQLMMPGN<br>YARAIFDSKNENSVLDLIEDDERKCKVRLLLQKFRFMRMIYRAVDPKVQYPSETKMYKET<br>AIEFGKILINDFDYAKWPNYIHKVIEHVQELIENENGPGTVGGLSSEGNEGGNKIFRHFR<br>KNLARRGSTYGGLRDVLWGHWLYSSPSLTKLSAKSKSEQRCSACRSLGHNIRNCPSSVENV

Prediction performed with FGENESH+ (*C.elegans* dataset and CviRAG1L_B_Biv1_0007 as homologous reference)

Predicted coding regions merging area is underlined as it is less confident. However, the region predicted to be intronic keeps homology with the reference sequence, therefore it is also provided between closed brackets and highlighted in black.

PimRAG2L_B_Biv2_5135

```
MRRSLLAYDNKQI
NRTRAIRMQVLPFKSIDLERGKSTRKRRDLELKHGDFFPPEGHVCIEIDRQNDDTNDESV
VSYLYFGGARRSDESSWEDSKDLFQIDYLVSSHDVNITDITKFETTGSIFPALQSTAAVV
NEHKIFVWGGYNTAQLTMETDLYILSKQGSKYNCEIVQQECTSLSRSKQYGNLPEGRSGH
SLTYIGNYMSVLYGGVTMPMRKTATLKSPFQKTCKDGKFYQLDIKTFEWKEINVPEVIPR
AFHSAEYFEEKKSIYIVGGMTYTGCQPDQRLPLDDVIVMKILDDNTFSLDRLKFTFRDIT
NYFLSYHSCCFLANNMFIIGGFYQSKSQMGELPGLNPNILSYNLTDLSVNHIEVNKIHRT
AGCTSMTLSNDCIMVVGGNTKNYFVYTSKPMIPSPCDFGIECNIRDSPEISPISWVQCEG
PCHRWLHQFCVGVLTSGLPKGKYICKDCSAKSRKRGRTAKK
```

Prediction performed with FGENESH (*C.elegans* dataset without similarity reference)

2. RAGL Detection info

Method: TBLASTN
Database searched: Whole-genome shotgun contigs (WGS) + only in Mollusca group (taxid: 6447)

2.1. RAG1L

Query: CviRAG1L_B_Biv1_0007 (previously described)
Subject: NIJJ01005135.1 Length: 19936 Number of Matches: 3

Score	Expect	Method	Identities	Positives	Gaps	Frame
62.8 bits(151)	1e-06	Compositional matrix adjust.	40/120(33%)	60/120(50%)	9/120(7%)	+3

```
Query  17    QMTKNIQNFILSTDMEHGLKLKCMCRLCGVKIPINSKSH-PKENFKSAITSLYNIDIDCD  75
             +M K+  N        H   L   CR+CG      S++  P E +K  + SL+ IDI  D
Sbjct  7641  EMQKDADNI-----ANHFANLTKFCRVCGSIQKRKSRAFCPVEPYKIELLSLFKIDIQYD  7805

Query  76    SENIHPPKICTACRLRL--TRHSQAEDD-IEINLPVLFEFLPHSINCNICFVKPGRPKKI  132
              E+IHP K+C +CR +    R  + E +   +N+PV   ++PH+ NC +C    GR   I
Sbjct  7806  DESIHPKKLCNSCRSKCFHFRTKKGEGNTFSMNIPVKHLYIPHNANCKVCHRNSGRNATI  7985
```

Score	Expect	Method	Identities	Positives	Gaps	Frame
332 bits(850)	0.0	Compositional matrix adjust.	180/464(39%)	272/464(58%)	25/464(5%)	+3

```
Query  223   SSETAPEMFKNVVIRSIPTERFCNAEVSQSFMCTICRGVPCDPYISKCSHIFCKECIFGW  282
             S+    P  F    + SIP E+     +++ F CTIC G+P  P I+ C HIFC+ CI  W
Sbjct  8295  STVHVPPSFTVTDLSSIPIEQAIEQSLAKIFQCTICLGIPTAPAITHCKHIFCRSCISSW  8474

Query  283   FSLSSACPVCRSLLDESEVSPLHGHLLQIYETLLVHCIHANCTQSHLIRNIDEHESICSM  342
                +  CP+CR  +  +E+S L G L  +Y+ L V C   NC +    +  I +HE++C
Sbjct  8475  LLNAGVCPICREPVMMNEISILTGQLSLMYDILHVSCRFDNCNEVLGLDKISDHENVC--  8648

Query  343   KGTKFLYNVTTKSRVF-------KLPLHSVSAKHTRHRRLKPIISQVNEFCNAQEENKSD  395
             K  K+   + T S +         K+P++   AK+ + +RLK + S V +FC+++ E+K+D
Sbjct  8649  KYGKYPSKLLTPSSIVRRGKGLQKMPIYDCKAKYVKQKRLKEVESSVEQFCSSKNEDKTD  8828

Query  396   VLFFMLKDHLKEINDPRWKQVESLWLGNN-STLSPEQCLALRVDLLQSKGQYRSQYDFLS  454
             VLFFML  HLK  ND R++Q+  +W+G      L+  +CLA+R+D LQSK QY++QYDFL
Sbjct  8829  VLFFMLIHHLKSQNDNRFEQINDVWMGGGVRKLTSHECLAIRIDTLQSKTQYKAQYDFLK  9008
```

```
Query  455   QNNVHVFQAPSKMESCENLFMPSASIFQIIDNDGNVLLQNS----ENPCT--------EP  502
                 + F AP  ++  E  ++P +  + I   D  +   ++       CT        EP
Sbjct  9009  AKGCNPFVAPYALDEIECQYVPGSVRYSIEGQDQVINYYHTPVKVNRECTTSPLDNTFEP  9188

Query  503   LNVNECFLPGFVELATPNCMGVRFSYFEALSLTLQELEPEILFGLKKHGLNIED--VLFL  560
             L++   F     ELATPN  G R+ Y +A++ TL ELE +I+ GL++H ++  D  +L
Sbjct  9189  LDIVNVFPACTPELATPNVKGCRWRYPDAIAKTLHELENDIIAGLQQHNIDPYDPTILLK  9368

Query  561   TTVKDGCDGMGEVSVYKEKDFKMLPDKVFRFSFCIVKIQAEY-DGKLFDVFTEPLPNSVR  619
             T +KDG DG+G+VSV+KE   + LPDK FRFSFC+ +I+A   D +   +F E  PNSV+
Sbjct  9369  TYIKDGADGLGDVSVHKEVGDRYLPDKAFRFSFCVYRIEAILGDDEKVTIFVEKRPNSVK  9548

Query  620   TNRPLLESISDENNQVSNVVCILPIENEREILMQNRMHVKTKEG  663
             TNRP+LE+I DENN  S+ +C+LPIENER  +    + ++T+ G
Sbjct  9549  TNRPVLEAICDENNHGSSTLCLLPIENERSYMKGKIIRIQTEFG  9680
```

Score	Expect	Method	Identities	Positives	Gaps	Frame
397 bits(1021)	0.0	Compositional matrix adjust.	184/331(56%)	243/331(73%)	1/331(0%)	+1

```
Query  671    FFNSMVDEKRDRGDSGLQGSGSKYLCTLCDADKQSAKALLGSFSINRSVSECSNIAEILR  730
              F NSM+ EK DR +SGL GSGS YLCTLCDA +++A + LG+F INRS +E + IAE +R
Sbjct  9700   FSNSMIGEKLDRANSGLAGSGSNYLCTLCDATRETALSNLGNFVINRSYAETAEIAEYMR  9879

Query  731    VNPNALSENELKKISKGVKCAPLSKIEPIQKGIDATHADINLGQFFKKIIVREIAGVTKW  790
              +NP+ L++ +L KISKG+K  PL + E I+KGIDATHADIN G FF+K+I+REI  + KW
Sbjct  9880   INPDNLTKADLNKISKGIKSTPLLQAESIEKGIDATHADINCGSFFRKLIIREIVQINKW  10059

Query  791    ELTQDVKPLVQNAEFLFDQHMKCNCGINPQLMMPGNYARTLFETPH-SVLLEHISDSVRK  849
              E++ DVK ++++ E  FD H+K   GINPQLMMPGNYAR +F++ + + +L+ I D  RK
Sbjct  10060  EMSGDVKSIIKDGEVKFDLHLKKTVGINPQLMMPGNYARAIFDSKNENSVLDLIEDDERK  10239

Query  850    ENLSSILNIFLHLRKVYRCKDPLTECPFDVQNYKKCAVEMGALLLQHFDYVEWPNYLHKV  909
                +  +L  F  +R +YR  DP  + P + + YK+ A+E G +L+  FDY +WPNY+HKV
Sbjct  10240  CKVRLLLQKFRFMRMIYRAVDPKVQYPSETKMYKETAIEFGKILINDFDYAKWPNYIHKV  10419

Query  910    IEHVQQLIEDPNGPGSVGAFSSEGNEAGNKLFRHFRKNLSRRGNTYGSLCDVLKLHWLYS  969
              IEHVQ+LIE+ NGPG+VG  SSEGNE GNK+FRHFRKNL+RRG+TYG L DVL  HWLYS
Sbjct  10420  IEHVQELIENENGPGTVGGLSSEGNEGGNKIFRHFRKNLARRGSTYGGLRDVLWGHWLYS  10599

Query  970    SKALFKLAEVEHKKVRCSLCFTSGHNKRTCP  1000
              S +L KL+     + RCS C + GHN R CP
Sbjct  10600  SPSLTKLSAKSKSEQRCSACRSLGHNIRNCP  10692
```

2.2 RAG2L

Query: **CviRAG2L_B_Biv1_0007 (previously described)**
Subject: **NIJJ01005135.1 Length: 19936 Number of Matches: 1**

Score	Expect	Method	Identities	Positives	Gaps	Frame
281 bits(718)	2e-81	Compositional matrix adjust.	168/451(37%)	252/451(55%)	35/451(7%)	-1

```
Query  7      QFRALPFKS------HSTNPLRRITRKSAASDDFNFFPPEGHVSLPINS----------Y  50
              + + LPFKS       ST   R +  K        +FFPPEGHV + I+
Sbjct  12550  RMQVLPFKSIDLERGKSTRKRRDLELKHG-----DFFPPEGHVCIEIDRQNDDTNDESVV  12386
```

```
Query  51     TYLYFGGARRGQESTWNMSRNIYKisfivddndvnvdfisEIKLSGGQFPQLQSSAGFYV  110
              +YLYFGGARR  ES+W  S+++++I ++V  +DVN+  I++ + +G  FP LQS+A    V
Sbjct  12385  SYLYFGGARRSDESSWEDSKDLFQIDYLVSSHDVNITDITKFETTGSIFPALQSTAA--V  12212

Query  111    SAENCLFVWGGLNLSCFSMSNELYIVKLNDNKGVVEIIQPP-GGISVRELGGEIPSGRCG  169
                E+ +FVWGG N +  +M  +LYI+    +K   EI+Q     +S   +  G +P GR G
Sbjct  12211  VNEHKIFVWGGYNTAQLTMETDLYILSKQGSKYNCEIVQQECTSLSRSKQYGNLPEGRSG  12032

Query  170    HTLTHYFDSCVILHGGVCFPHRNSCVGSSLFKNVTNDNNFYMFDFESLFWTKLSVTGSAP  229
              H+LT+  +   +L+GGV  P R +     S F+     D  FY  D ++  W +++V     P
Sbjct  12031  HSLTYIGNYMSVLYGGVTMPMRKTATLKSPFQKTCKDGKFYQLDIKTFEWKEINVPEVIP  11852

Query  230    RAYHTANVMEIRGMKSIVYIGGVTKTE-SALQRIPLSNVLVLKMDSNKHFHTEIL--TFA  286
              RA+H+A   E +  KSI  +GG+T T      QR+PL +V+V+K+  +  F  + L  TF
Sbjct  11851  RAFHSAEYFEEK--KSIYIVGGMTYTGCQPDQRLPLDDVIVMKILDDNTFSLDRLKFTFR  11678

Query  287    NAPAVGISYHSVGVIGPYIFVVGGLDEDNLQG------RCSVSILNKDTFLCENVQFDRY  340
              +     +SYHS    +   +F++GG  +   Q          ++    N        +++ ++
Sbjct  11677  DITNYFLSYHSCCFLANNMFIIGGFYQSKSQMGELPGLNPNILSYNLTDLSVNHIEVNKI  11498

Query  341    FRSAGHSVCTLSDDCLMICGGMNLQYFVFSSKQMVPSPCDFDTECKIIESVETSPISWIQ  400
               R+AG +  TLS+DC+M+ GG    YFV++SK M+PSPCDF  EC I +S E SPISW+Q
Sbjct  11497  HRTAGCTSMTLSNDCIMVVGGNTKNYFVYTSKPMIPSPCDFGIECNIRDSPEISPISWVQ  11318

Query  401    CEGACKRWLHQFCVGVLDIDMSRKNFICTTC  431
              CEG C RWLHQFCVGVL    + +  +IC  C
Sbjct  11317  CEGPCHRWLHQFCVGVLTSGLPKGKYICKDC  11225
```

3. TIR Detection info

Aligned P.imbricata RAG1L-RAG2L flanking regions had been searched for homology variations both at left and right flanking regions. Detected homology droping regions were inspected for TIR and TSD presence. For more details please see methods section.

A. RAG1L facing margins alignment (left)

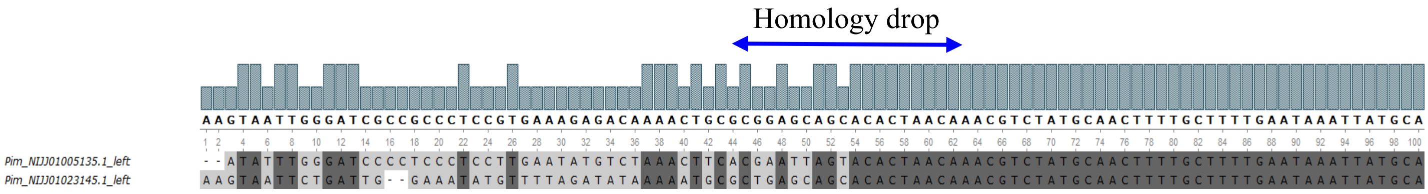

B. RAG2L facing margins alignment (right)

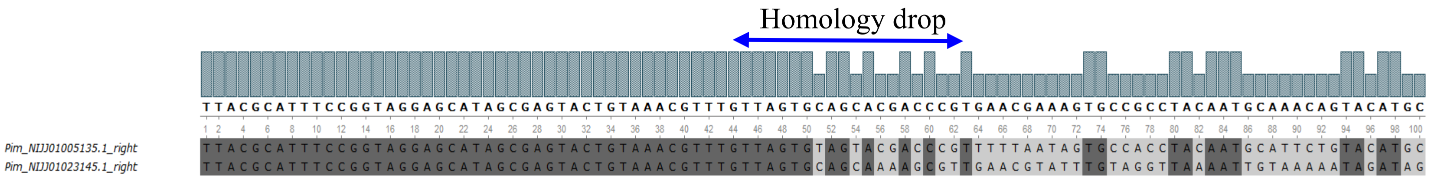

C. Alignment of Left homology drop regions with the reverse complement of right homology drop region in order to spot inverted repeat regions.

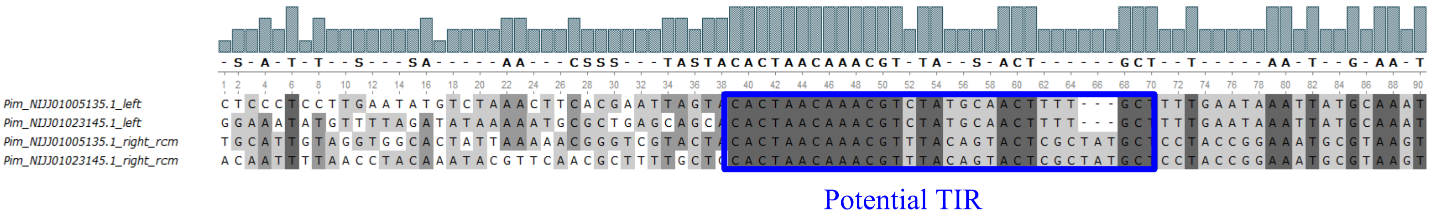

D. TSD pairs in NIJJ01005135.1:

RAG1L side:

TSD TIR5

5' - TGTCTAAACTTCACGAATTAGTACACTAACAAACGTCTATGCAAC - 3'

RAG2L side:

TSD

5' - AGCGAGTACTGTAAACGTTTGTTAGTGTAGTACGACCCGTTTTTA- 3'
3' - TCGCTCATGACATTTGCAAACAATCACATCATGCTGGGCAAAAAT- 5'

TIR3

Pinctada imbricata (Pim)

WGS: NIJJ01023145.1

1. Summary

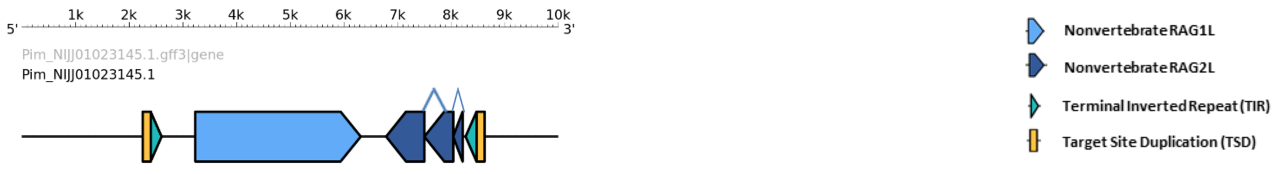

Detected configuration: TSD - TIR5 - RAG1L - RAG2L - TIR3 - TSD

Detected loci	Predicted CDS	start	end	strand	Observations
RAG1L	1	7234	10314	-	complete; no stop codon
RAG2L	1	12211	12035	+	complete; no stop codons
	2	12063	11502		
	3	11503	10790		

Predicted TIR	Distance from CDS (bp)	start	strand	TSD sequence	Assessment method
TIR5 (RAG1L side)	826	6408	+	5'- CAGCA -TIR5	Homology variation
TIR3 (RAG2L side)	56	12267	-	TIR3- CAGCA -3'	Homology variation

```
PimTIR5_3145                          5'CACTAACAAACGTCTATGCAACTTTTGCTTTTG 3'
                                        |||||||||||| ||     |||    ||| |
PimTIR3_3145 (reverse complement)     3'CACTAACAAACGTTTACAGTACTC--GCTATGC 5'
```

Selected predicted protein product

PimRAG1L_B_Biv2_3145

```
MEAADEMQKDADNIANHIANLTKFCRVCGTIQKRKSRAFCPVEPYKI
ELLSLFKIDIQYDDESIHPKKLCNSCRSKCFHFRTKKGEGNTFSMNIPVK
HLYIPHNANCKVCHRNSERNATIELFQIHYNPSSEVPIEHVDGGGTCSNT
ESDSEDQRSNDHSHSFSATCHVAPSTSSHFFAAEMDKSEIYASSLSENSN
ELCEPASKKLCRPKGILDVTKQLDFDSTVHVPPSFTVTDLSSIPIEQAIE
QSLAKIFQCTICLGIPTAPAITHCKHIFCRSCLSSWLLNAGVCPICREPV
VINEISILTGQLSLMYDILHVSCRFDNCNEVLGLDKISDHENVCKYGKYP
SKLLTPSSIVRRGKGLQKMPIYDCKAKYVKQKRLKEVESSVEQFCSSKNE
DKTDVLFFMLIHHLKSQNDNRFEQINDVWMGGGVRKLTSHECLAIRIDTL
QSKTQCKAQYDFLKAKGCNPFVAPYALDEIECQYVPGSVRYSIEGQDQVI
NYYHTPVKVNRECTSSPLDNTFEPLDIVNDFPACTPELATPNVKGCRWRY
PDAIAKTLHELENDIIAGLQQHNIDPYDPTILLKTYIKDGADGLGDVSVH
KEVGDRYLPDKAFRFSFCVYRIEAILGDDEKVTIFVEKRPNSVKTNRPVL
EAICDENNHGSSTLCLLPIENERSYMKGKIIRIQTEFGWRRHVLFFSNSM
IDEKLDRTNSGLAGSGSNYLCTLCDATRETALSNLGNFVINRSYAETAEI
AEYMRINPDNLTKADLNKISKGIKSTPLLQAESIEKGIDATHADINCGSF
FRKLIIREIVQINKWEMSGDVKSIIKDGEVKFDLHLKKTVGINPQLMMPG
NYARAIFDSKNENSVLDLIEDDERKCKVRLLLQKFRFMRMIYRAVDPKVQ
YPSETKMYKETAIEFGKILINDFDYAKWPNYIHKVIEHVQELNENGPGTV
GGLSSEGNEGGNKIFRHFRKNLARRGSTYGGLRDVLWGHWLYSSPSLTKL
SAKSKSEQRCSACRSLGHNIRNCPSSVENV
```

Prediction performed with FGENESH+ (*C.elegans* dataset and CviRAG1L_B_Biv1_0007 as homologous reference)

PimRAG2L_B_Biv2_3145	Many different predicted translation products, therefore, we did not further used this sequence in analysis.

2. RAGL Detection info

Method: TBLASTN

Database searched: Whole-genome shotgun contigs (WGS) + only in Mollusca group (taxid: 6447)

2.1. RAG1L

Query: **CviRAG1L_B_Biv1_0007 (previously described)**

Subject: **NIJJ01023145.1** **Length: 29196 Number of Matches: 2**

Score	Expect	Method	Identities	Positives	Gaps	Frame
61.2 bits(147)	4e-06	Compositional matrix adjust.	37/110(34%)	57/110(51%)	9/110(8%)	+1

```
Query  17    QMTKNIQNFILSTDMEHGLKLKCMCRLCGVKIPINSKSH-PKENFKSAITSLYNIDIDCD  75
             +M K+  N        H   L   CR+CG      S++  P E +K  + SL+ IDI  D
Sbjct  7249  EMQKDADNI-----ANHIANLTKFCRVCGTIQKRKSRAFCPVEPYKIELLSLFKIDIQYD  7413

Query  76    SENIHPPKICTACRLRL--TRHSQAEDD-IEINLPVLFEFLPHSINCNIC  122
              E+IHP K+C +CR +    R  + E +   +N+PV   ++PH+ NC +C
Sbjct  7414  DESIHPKKLCNSCRSKCFHFRTKKGEGNTFSMNIPVKHLYIPHNANCKVC  7563
```

Score	Expect	Method	Identities	Positives	Gaps	Frame
713 bits(1841)	0.0	Compositional matrix adjust.	364/802(45%)	515/802(64%)	28/802(3%)	+1

```
Query  223    SSETAPEMFKNVVIRSIPTERFCNAEVSQSFMCTICRGVPCDPYISKCSHIFCKECIFGW  282
              S+   P  F    + SIP E+     +++ F CTIC G+P  P I+ C HIFC+ C+  W
Sbjct  7903   STVHVPPSFTVTDLSSIPIEQAIEQSLAKIFQCTICLGIPTAPAITHCKHIFCRSCLSSW  8082

Query  283    FSLSSACPVCRSLLDESEVSPLHGHLLQIYETLLVHCIHANCTQSHLIRNIDEHESICSM  342
                 +  CP+CR  +  +E+S L G L  +Y+ L V C   NC +   +  I +HE++C
Sbjct  8083   LLNAGVCPICREPVVINEISILTGQLSLMYDILHVSCRFDNCNEVLGLDKISDHENVC--  8256

Query  343    KGTKFLYNVTTKSRVF-------KLPLHSVSAKHTRHRRLKPIISQVNEFCNAQEENKSD  395
              K  K+   + T S +        K+P++   AK+ + +RLK + S V +FC+++ E+K+D
Sbjct  8257   KYGKYPSKLLTPSSIVRRGKGLQKMPIYDCKAKYVKQKRLKEVESSVEQFCSSKNEDKTD  8436

Query  396    VLFFMLKDHLKEINDPRWKQVESLWLGNN-STLSPEQCLALRVDLLQSKGQYRSQYDFLS  454
              VLFFML  HLK  ND R++Q+  +W+G     L+  +CLA+R+D LQSK Q ++QYDFL
Sbjct  8437   VLFFMLIHHLKSQNDNRFEQINDVWMGGGVRKLTSHECLAIRIDTLQSKTQCKAQYDFLK  8616

Query  455    QNNVHVFQAPSKMESCENLFMPSASIFQIIDNDGNVLLQNS----ENPCT--------EP  502
                  + F AP  ++  E  ++P +  + I   D  +   ++       CT        EP
Sbjct  8617   AKGCNPFVAPYALDEIECQYVPGSVRYSIEGQDQVINYYHTPVKVNRECTSSPLDNTFEP  8796

Query  503    LNVNECFLPGFVELATPNCMGVRFSYFEALSLTLQELEPEILFGLKKHGLNIED--VLFL  560
              L++   F     ELATPN  G R+ Y +A++ TL ELE +I+ GL++H ++  D  +L
Sbjct  8797   LDIVNDFPACTPELATPNVKGCRWRYPDAIAKTLHELENDIIAGLQQHNIDPYDPTILLK  8976
```

```
Query  561    TTVKDGCDGMGEVSVYKEKDFKMLPDKVFRFSFCIVKIQAEY-DGKLFDVFTEPLPNSVR  619
              T +KDG DG+G+VSV+KE   + LPDK FRFSFC+ +I+A   D +   +F E  PNSV+
Sbjct  8977   TYIKDGADGLGDVSVHKEVGDRYLPDKAFRFSFCVYRIEAILGDDEKVTIFVEKRPNSVK  9156

Query  620    TNRPLLESISDENNQVSNVVCILPIENEREILMQNRMHVKTKEGWMFHKFSFFNSMVDEK  679
              TNRP+LE+I DENN  S+ +C+LPIENER  +    + ++T+ GW  H   F NSM+DEK
Sbjct  9157   TNRPVLEAICDENNHGSSTLCLLPIENERSYMKGKIIRIQTEFGWRRHVLFFSNSMIDEK  9336

Query  680    RDRGDSGLQGSGSKYLCTLCDADKQSAKALLGSFSINRSVSECSNIAEILRVNPNALSEN  739
               DR +SGL GSGS YLCTLCDA +++A + LG+F INRS +E + IAE +R+NP+ L++
Sbjct  9337   LDRTNSGLAGSGSNYLCTLCDATRETALSNLGNFVINRSYAETAEIAEYMRINPDNLTKA  9516

Query  740    ELKKISKGVKCAPLSKIEPIQKGIDATHADINLGQFFKKIIVREIAGVTKWELTQDVKPL  799
              +L KISKG+K  PL + E I+KGIDATHADIN G FF+K+I+REI   + KWE++ DVK +
Sbjct  9517   DLNKISKGIKSTPLLQAESIEKGIDATHADINCGSFFRKLIIREIVQINKWEMSGDVKSI  9696

Query  800    VQNAEFLFDQHMKCNCGINPQLMMPGNYARTLFETPH-SVLLEHISDSVRKENLSSILNI  858
              +++ E  FD H+K   GINPQLMMPGNYAR +F++ + + +L+ I D  RK  +  +L
Sbjct  9697   IKDGEVKFDLHLKKTVGINPQLMMPGNYARAIFDSKNENSVLDLIEDDERKCKVRLLLQK  9876

Query  859    FLHLRKVYRCKDPLTECPFDVQNYKKCAVEMGALLLQHFDYVEWPNYLHKVIEHVQQLIE  918
              F  +R +YR  DP  + P + + YK+ A+E G +L+  FDY +WPNY+HKVIEHVQ+L E
Sbjct  9877   FRFMRMIYRAVDPKVQYPSETKMYKETAIEFGKILINDFDYAKWPNYIHKVIEHVQELNE  10056

Query  919    DPNGPGSVGAFSSEGNEAGNKLFRHFRKNLSRRGNTYGSLCDVLKLHWLYSSKALFKLAE  978
                NGPG+VG  SSEGNE GNK+FRHFRKNL+RRG+TYG L DVL  HWLYSS +L KL+
Sbjct  10057  --NGPGTVGGLSSEGNEGGNKIFRHFRKNLARRGSTYGGLRDVLWGHWLYSSPSLTKLSA  10230

Query  979    VEHKKVRCSLCFTSGHNKRTCP  1000
                  + RCS C + GHN R CP
Sbjct  10231  KSKSEQRCSACRSLGHNIRNCP  10296
```

2.2 RAG2L

Query: **CviRAG2L_B_Biv1_0007 (previously described)**
Subject: **NIJJ01023145.1** **Length: 29196 Number of Matches: 2**

Score	Expect	Method	Identities	Positives	Gaps	Frame
115 bits(289)	5e-65	Compositional matrix adjust.	70/188(37%)	106/188(56%)	13/188(6%)	-1

```
Query  36     FFPPEGHVSLPINSY----------TYLYFGGARRGQESTWNMSRNIYKisfivddndvn  85
              FFPPEGHV + I+            +YLYFGGARR  ES+W  S+++++I ++V  +DVN
Sbjct  12063  FFPPEGHVCIEIDRQNDDTNDESVVSYLYFGGARRSDESSWEDSKDLFQIDYLVSSHDVN  11884

Query  86     vdfisEIKLSGGQFPQLQSSAGFYVSAENCLFVWGGLNLSCFSMSNELYIVKLNDNKGVV  145
              +  I++ + +G  FP LQS+    V  E+ +FVWGG N +  +M  +LYI+    +K
Sbjct  11883  ITDITKFETTGSIFPALQSTTA--VVIEHKIFVWGGYNTAQLTMETDLYIISKQGSKYNC  11710

Query  146    EIIQPP-GGISVRELGGEIPSGRCGHTLTHYFDSCVILHGGVCFPHRNSCVGSSLFKNVT  204
              EI+Q     +S  +  G +P GR GH+LT+  +   +L+GGV  P R +    S F+
Sbjct  11709  EIVQQECTSLSRSKQYGNLPEGRSGHSLTYIGNYMSVLYGGVTMPMRKTATLESPFQKTC  11530

Query  205    NDNNFYMF  212
               D  FY
Sbjct  11529  KDGKFYQL  11506
```

Score	Expect	Method	Identities	Positives	Gaps	Frame
161 bits(407)	5e-65	Compositional matrix adjust.	90/223(40%)	131/223(58%)	13/223(5%)	-3

```
Query  219    WTKLSVTGSAPRAYHTANVMEIRGMKSIVYIGGVTKTE-SALQRIPLSNVLVLKMDSNKH  277
              W +++V    PRA+H+A   E +  KSI  +GG+T T     QR+PL +V+V+K+  +
Sbjct  11488  WKEINVPEVIPRAFHSAEYFEEK--KSIYIVGGMTYTGCQPDQRLPLDDVIVMKILDDNT  11315

Query  278    FHTEIL--TFANAPAVGISYHSVGVIGPYIFVVGGLDEDNLQGRCSVSILNKD------T  329
              F  + L  TF +     +SYHS   +   +F++GG  +   Q    +  LN +      T
Sbjct  11314  FSLDRLKFTFRDITNYFLSYHSCCFLANNMFIIGGFYQSKSQMG-ELPGLNPNVLSYNLT  11138

Query  330    FLCEN-VQFDRYFRSAGHSVCTLSDDCLMICGGMNLQYFVFSSKQMVPSPCDFDTECKII  388
              +L  N ++ ++  R+AG +  TLS+DC+M+ GG    YFV++SK M PSPCDF  EC I
Sbjct  11137  YLSVNHIEVNKIHRTAGCTSMTLSNDCIMVVGGNTKNYFVYTSKPMTPSPCDFGIECNIR  10958

Query  389    ESVETSPISWIQCEGACKRWLHQFCVGVLDIDMSRKNFICTTC  431
              +S E SPISW+QCEG C RWLHQFCVGV    + +  +IC  C
Sbjct  10957  DSPEISPISWVQCEGPCHRWLHQFCVGVFTSGLPKGKYICKDC  10829
```

3. TIR Detection info

See scaffold WGS: NIJJ01005135.1 (page 41).

TSD pairs in NIJJ01023145.1:

RAG1L side:

```
                         TSD       TIR5
5’ - ATATAAAAATGCGCTGAGCAGCACACTAACAAACGTCTATGCAACT - 3’
```

RAG2L side:

```
                                   TSD
5’ - AGCGAGTACTGTAAACGTTTGTTAGTGCAGCAAAAGCGTTGAACGT- 3’
3’ - TCGCTCATGACATTTGCAAACAATCACGTCGTTTTCGCAACTTGCA- 5’
                TIR3
```

Pinctada imbricata (Pim)

WGS: NIJJ01003325.1

1. Summary

Detected configuration: TIR5 - RAG1L - RAG2L

Detected loci	Predicted CDS	start	end	strand	Observations
RAG1L (!)	1	32807	30945	-	complete; no stop codon; frameshift
	2	30946	29894		
RAG2L	-	28424	29767	+	complete; stop codon in the middle

Predicted TIR	Distance from CDS (bp)	start	strand	TSD sequence	Assessment method
TIR5 (RAG1L side)	634	33441	-		Blastn cassette margin
TIR3 (RAG2L side)					

>PimTIR5_3325
CACTAACAAAACCCAATGCTACTTTTGCTTTTGCATAATTAGGTAAA

Selected predicted protein product	
PimRAG1L_B_Biv2_3325 (!)	MSEVLELFEIEISSDSKDVHPSR<br>LCNACKMQCIRYRTSKQKKLKFSCNIPKPYDFAPHDSSSLCSICNRGKGRTYKQLMPVVF<br>DFSIAKSEDSTDTASDNEQVESTVAFAKVNDSDTDTASDMECGISSSNIAEYSIPGTSWD<br>SFEPSAKIPRKSQTVADVARVLDFNETAHSFSDKEVTSVNLDRAVEHHLAEIMQCSICKG<br>LPTEPIITLCNHIYCKACITAWLQIGSVCPVCRTVTDMDDISSLSGHLLLMYESLHVSCV<br>YTECTEVLSVHEVLKHEDTCKFGKYPKTILQSKSSRSRGEGLAKIPIYDCRPKYLRQKSL<br>KPVVQSVREFCSEKHENIGDVLFFMLSDHLSDTNDSRSKLIDSIWRGERSTSLTAHECLA<br>LRIDTLQSKTQYRSQYEFLKQKSHFNPFQPPKTLDSIECQYMPASTRYSLEGQEHFNTFY<br>HTPAKPTASIMSQTVNFSFEPYDVLSDFHKSVPEIPTPNVKGVRWNYPDSVAKTLQELEP<br>DIIRGLEAEGIDPLDPSLILKTYIKDGADGLGDVAVHKETGDRFLPDKAFRFSFCVFRIE<br>AVLEKGVELIFLEEKPNSVRTNRPLLEAICDENNKASSLL[CLQIIEQERLFMKDKIMS/<br>VQNQNGWRRHNLFFYNSMIDEKLDRS]CGGLAGSGSSFLCTLCYATRETAQSQLGSFSISRS<br>VADTTKIANYVKANPDNLSKSSLEKLSKGVKTVPILQTDASQKCLDATHADINCGSFFKKL<br>IIREISHVNTWDVTEDIKSIIKNAEVTFDNCLKKEIGINPQLMMSGNYSRSLFDPDNEQS<br>ILCLVDNEVRRKYLSDLLSKFRFMRKVYRASDPKTQYPEETKIYKQTAVSFGHALLENFS<br>YARWPNYIHKVIEHVQELIEDENGAGTVGGLSGEGNEGRNKIFRHFRKNLARRGDTYGGL<br>RDVLWGHWLYSSPELLRLANKTSNKKKCSICLQEGHNMRSCVQRKASTH<br><br>Frameshift is shown with red dash (/). However, various ORF predictions indicate potential transcripts with variable intronic size around the frameshift.<br><br>While less confident, above it was selected a consensus between a pool of predicted potential translations using Augustus (multiple invertebrate bilaterian species datasets) and FGENESH+ (multiple invertebrate bilaterian species datasets), using either CviRAG2L_B_Biv1_0007, PimRAG1L_B_Biv2_4498, PimRAG1L_B_Biv2_3145 as reference.

2. RAGL Detection info

Method: TBLASTN
Database searched: Whole-genome shotgun contigs (WGS) + only in Mollusca group (taxid: 6447)

2.1. RAG1L

Query: CviRAG1L_B_Biv1_0007 (previously described)
Subject: NIJJ01003325.1 **Length:46382 Number of Matches: 2**

Score	Expect	Method	Identities	Positives	Gaps	Frame
391 bits(1005)	7e-112	Compositional matrix adjust.	185/363(51%)	256/363(70%)	1/363(0%)	-2

```
Query  638    VVCILPIENEREILMQNRMHVKTKEGWMFHKFSFFNSMVDEKRDRGDSGLQGSGSKYLCT  697
              V C+    + ++ L + ++ V+ + GW  H   F+NSM+DEK DR   GL GSGS +LCT
Sbjct  31006  VCCVFK*LSRKDFL*KTKL*VQNQNGWRRHNLFFYNSMIDEKLDRSCGGLAGSGSSFLCT  30827

Query  698    LCDADKQSAKALLGSFSINRSVSECSNIAEILRVNPNALSENELKKISKGVKCAPLSKIE  757
              LC A +++A++ LGSFSI+RSV++ + IA  ++ NP+ LS++ L+K+SKGVK  P+ + +
Sbjct  30826  LCYATRETAQSQLGSFSISRSVADTTKIANYVKANPDNLSKSSLEKLSKGVKTVPILQTD  30647

Query  758    PIQKGIDATHADINLGQFFKKIIVREIAGVTKWELTQDVKPLVQNAEFLFDQHMKCNCGI  817
                QK +DATHADIN G FFKK+I+REI+ V  W++T+D+K +++NAE  FD  +K   GI
Sbjct  30646  ASQKCLDATHADINCGSFFKKLIIREISHVNTWDVTEDIKSIIKNAEVTFDNCLKKEIGI  30467

Query  818    NPQLMMPGNYARTLFETPH-SVLLEHISDSVRKENLSSILNIFLHLRKVYRCKDPLTECP  876
              NPQLMM GNY+R+LF+  +   +L  + + VR++ LS +L+ F  +RKVYR  DP T+ P
Sbjct  30466  NPQLMMSGNYSRSLFDPDNEQSILCLVDNEVRRKYLSDLLSKFRFMRKVYRASDPKTQYP  30287

Query  877    FDVQNYKKCAVEMGALLLQHFDYVEWPNYLHKVIEHVQQLIEDPNGPGSVGAFSSEGNEA  936
               + + YK+ AV  G  LL++F Y  WPNY+HKVIEHVQ+LIED NG G+VG  S EGNE
Sbjct  30286  EETKIYKQTAVSFGHALLENFSYARWPNYIHKVIEHVQELIEDENGAGTVGGLSGEGNEG  30107

Query  937    GNKLFRHFRKNLSRRGNTYGSLCDVLKLHWLYSSKALFKLAEVEHKKVRCSLCFTSGHNK  996
               NK+FRHFRKNL+RRG+TYG L DVL  HWLYSS  L +LA     K +CS+C   GHN
Sbjct  30106  RNKIFRHFRKNLARRGDTYGGLRDVLWGHWLYSSPELLRLANKTSNKKKCSICLQEGHNM  29927

Query  997    RTC  999
              R+C
Sbjct  29926  RSC  29918
```

Score	Expect	Method	Identities	Positives	Gaps	Frame
350 bits(899)	4e-98	Compositional matrix adjust.	180/456(39%)	282/456(61%)	23/456(5%)	-1

```
Query  231    FKNVVIRSIPTERFCNAEVSQSFMCTICRGVPCDPYISKCSHIFCKECIFGWFSLSSACP  290
              F +  + S+  +R     +++   C+IC+G+P +P I+ C+HI+CK CI  W  + S CP
Sbjct  32288  FSDKEVTSVNLDRAVEHHLAEIMQCSICKGLPTEPIITLCNHIYCKACITAWLQIGSVCP  32109

Query  291    VCRSLLDESEVSPLHGHLLQIYETLLVHCIHANCTQSHLIRNIDEHESICSMKG--TKFL  348
              VCR++ D  ++S L GHLL +YE+L V C++  CT+   +  + +HE  C         L
Sbjct  32108  VCRTVTDMDDISSLSGHLLLMYESLHVSCVYTECTEVLSVHEVLKHEDTCKFGKYPKTIL  31929

Query  349    YNVTTKSR---VFKLPLHSVSAKHTRHRRLKPIISQVNEFCNAQEENKSDVLFFMLKDHL  405
               + +++SR   + K+P++    K+ R + LKP++  V EFC+ + EN  DVLFFML DHL
Sbjct  31928  QSKSSRSRGEGLAKIPIYDCRPKYLRQKSLKPVVQSVREFCSEKHENIGDVLFFMLSDHL  31749

Query  406    KEINDPRWKQVESLWLGNNST-LSPEQCLALRVDLLQSKGQYRSQYDFLSQ-NNVHVFQA  463
               + ND R K ++S+W G  ST L+  +CLALR+D LQSK QYRSQY+FL Q ++ + FQ
Sbjct  31748  SDTNDSRSKLIDSIWRGERSTSLTAHECLALRIDTLQSKTQYRSQYEFLKQKSHFNPFQP  31569

Query  464    PSKMESCENLFMPSASIFQIIDNDG-NVLLQNSENPCT-----------EPLNVNECFLP  511
              P  ++S E  +MP+++ + +   +  N        P             EP +V   F
Sbjct  31568  PKTLDSIECQYMPASTRYSLEGQEHFNTFYHTPAKPTASIMSQTVNFSFEPYDVLSDFHK  31389
```

```
Query  512    GFVELATPNCMGVRFSYFEALSLTLQELEPEILFGLKKHGLNIED--VLFLTTVKDGCDG  569
                 E+ TPN  GVR++Y ++++ TLQELEP+I+ GL+  G++  D  ++   T +KDG DG
Sbjct  31388  SVPEIPTPNVKGVRWNYPDSVAKTLQELEPDIIRGLEAEGIDPLDPSLILKTYIKDGADG  31209

Query  570    MGEVSVYKEKDFKMLPDKVFRFSFCIVKIQAEYDGKLFDVFTEPLPNSVRTNRPLLESIS  629
              +G+V+V+KE    + LPDK FRFSFC+ +I+A   +   +F E  PNSVRTNRPLLE+I
Sbjct  31208  LGDVAVHKETGDRFLPDKAFRFSFCVFRIEAVLEKGVELIFLEEKPNSVRTNRPLLEAIC  31029

Query  630    DENNQVSNVVCILPIENEREILMQNRMHVKTKEGWM  665
              DENN+ S+++C+  IE ER + M++++ + +K  W+
Sbjct  31028  DENNKASSLLCLQIIEQER-LFMKDKI-MSSKPKWL  30927
```

3. TIR Detection info

TIR5 sequence was detected using BLASTN
Searched database was P.imbricata NIJJ WGS project.

Query: TIR5 containing margin (200bp) detected in scaffold WGS: NIJJ01005135.1
Subject: WGS: NIJJ01003325.1

Score	Expect	Identities	Gaps	Strand
43.7 bits(47)	0.020	39/48(81%)	1/48(2%)	Plus/Minus

```
Query  1      CACTAACAAACGTCTATGCAACTTTTGCTTTTGAATAAATTATGCAAA  48
              ||||||||||    | |||| ||||||||||||| || ||||| | |||
Sbjct  33441  CACTAACAAAACCCAATGCTACTTTTGCTTTTGCAT-AATTAGGTAAA  33395
```

Pinctada imbricata (Pim)

WGS: NIJJ01024498.1

1. Summary

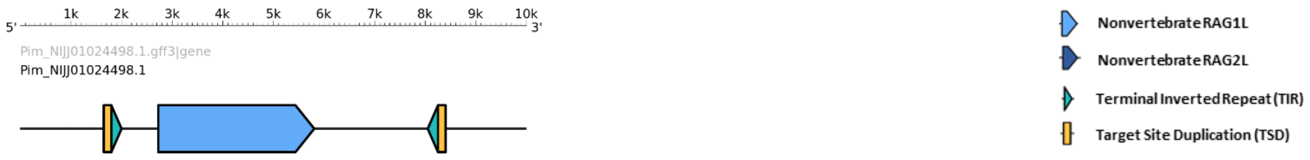

Detected configuration: TSD - TIR5 - RAG1L - TIR3 - TSD

Detected loci	Predicted CDS	start	end	strand	Observations
RAG1L	1	15723	18809	-	complete; no stop codon

Predicted TIR	Distance from CDS (bp)	start	strand	TSD sequence	Assessment method
TIR5 (RAG1L side)	826	14897	-	5'- AAACA -TIR5	Homology variation
TIR3 (RAG2L side)	2232	21041	+	TIR3- AAACA -3'	Homology variation

```
PimTIR5_4498                          5′CACTAACAAACGTCTATGCAACTTTTGCTTTT 3′
                                        ||||||||||||| ||    |||     |  |
PimTIR3_4498 (reverse complement)     3′CACTAACAAACGTTTACAGTACTCGCTATGCT 5′
```

Selected predicted protein product	
PimRAG1L_B_Biv2_4498	MEAADEMQKDADNIANHFANLTKFCRVCGTIQKRKSRAFCPVEPYKIELLSLFKIDIQYDDE<br>SIHPKKLCNSCRSKCFHFRTKKGEGNTFSMNIPVKHLYIPHNANCKVCHRNSGRNATIEL<br>FQIHYNPSSEVPIEHVDGGGTCSNTESDSEDQRSNDHSHSFSATCHVAPSTSSHFFAAEM<br>DKSEIYASSLSENSNELCEPASKKLCRPKGILDVTKQLDFDSTVHVPPSFTVTDLSSIPI<br>EQAIEQSLAKIFQCTICLGIPTAPAITHCKHIFCRSCISSWLLNAGVCPICREPVVINEI<br>SILTGQLSLMYDILHVSCRFDNCNEVLGLDKISDHENVCKYGKYPSKLLTPSSIVRRGKG<br>LQKMPIYDCKAKYVKQKRLKEVESSVEQFCSSKNEDKTDVLFFMLIHHLKSQNDNRFEQI<br>NDVWMGGGVRKLTSHECLAIRIDTLQSKTQYKAQYDFLKAKGCNPFVAPYALDEIECQYV<br>PGSVRYSIEGQDQVINYYHTPVKVNRECTTSPLDNTFEPLDIVNVFPACTPELATPNVKG<br>CRWRYPDAIAKTLHELENDIIAGLQQHNIDPYDPTILLKTYIKDGADGLGDVSVHKEVGD<br>RYLPDKAFRFSFCVYRIEAILGDDEKVTIFVEKRPNSVKTNRPVLEAICDENNHGSSTLC<br>LLPIENERSYMKGKIIRIQTEFGWRRHVLIFSNSMIDEKLDRANSGLAGSGSNYLCTLCD<br>ATRETALSNLGNFVINRSYAETAEIAEYMRINPDNLTKADLNKISKGIKSTPLLQAESIE<br>KGIDATHADINCGSFFRKLIIREIVQINKWEMSGDVKSIIKDGEVKFDLHLKKTVGINPQ<br>LMMPGNYARAIFDSKNENSILDLIEDDERKCKVRLLLQKFRFMRMIYRAVDPKVQYPSET<br>KMYKETAIEFGKILINDFDYAKWPNYIHKVIEHVQELIENENGPGTVGGLSSEGNEGGNK<br>IFRHFRKNLARRGSTYGGLRDVLWGHWLYSSPSLTKLSAKSKSEQRCSACRSLGHNIRNC<br>PSSVENV<br><br>Prediction performed with FGENESH+ (*C.elegans* dataset and CviRAG1L_B_Biv1_0007 as homologous reference

2. RAGL Detection info

Method: TBLASTN

Database searched: Whole-genome shotgun contigs (WGS) + only in Mollusca group (taxid: 6447)

2.1. RAG1L

Query: CviRAG1L_B_Biv1_0007 (previously described)
Subject: NIJJ01024498.1 Length: 35788 Number of Matches: 2

Score	Expect	Method	Identities	Positives	Gaps	Frame
729 bits(1882)	0.0	Compositional matrix adjust.	367/802(46%)	518/802(64%)	26/802(3%)	+3

```
Query  223    SSETAPEMFKNVVIRSIPTERFCNAEVSQSFMCTICRGVPCDPYISKCSHIFCKECIFGW  282
              S+   P  F    + SIP E+     +++ F CTIC G+P  P I+ C HIFC+ CI  W
Sbjct  16392  STVHVPPSFTVTDLSSIPIEQAIEQSLAKIFQCTICLGIPTAPAITHCKHIFCRSCISSW  16571

Query  283    FSLSSACPVCRSLLDESEVSPLHGHLLQIYETLLVHCIHANCTQSHLIRNIDEHESICSM  342
                 +  CP+CR  +  +E+S L G L  +Y+ L V C   NC +   +  I +HE++C
Sbjct  16572  LLNAGVCPICREPVVINEISILTGQLSLMYDILHVSCRFDNCNEVLGLDKISDHENVC--  16745

Query  343    KGTKFLYNVTTKSRVF-------KLPLHSVSAKHTRHRRLKPIISQVNEFCNAQEENKSD  395
              K  K+   + T S +        K+P++   AK+ + +RLK + S V +FC+++ E+K+D
Sbjct  16746  KYGKYPSKLLTPSSIVRRGKGLQKMPIYDCKAKYVKQKRLKEVESSVEQFCSSKNEDKTD  16925

Query  396    VLFFMLKDHLKEINDPRWKQVESLWLGNN-STLSPEQCLALRVDLLQSKGQYRSQYDFLS  454
              VLFFML  HLK  ND R++Q+  +W+G      L+  +CLA+R+D LQSK QY++QYDFL
Sbjct  16926  VLFFMLIHHLKSQNDNRFEQINDVWMGGGVRKLTSHECLAIRIDTLQSKTQYKAQYDFLK  17105

Query  455    QNNVHVFQAPSKMESCENLFMPSASIFQIIDNDGNVLLQNS----ENPCT--------EP  502
                  + F AP  ++  E  ++P +  + I   D  +   ++       CT        EP
Sbjct  17106  AKGCNPFVAPYALDEIECQYVPGSVRYSIEGQDQVINYYHTPVKVNRECTTSPLDNTFEP  17285

Query  503    LNVNECFLPGFVELATPNCMGVRFSYFEALSLTLQELEPEILFGLKKHGLNIED--VLFL  560
              L++   F     ELATPN  G R+ Y +A++ TL ELE +I+ GL++H ++  D  +L
Sbjct  17286  LDIVNVFPACTPELATPNVKGCRWRYPDAIAKTLHELENDIIAGLQQHNIDPYDPTILLK  17465

Query  561    TTVKDGCDGMGEVSVYKEKDFKMLPDKVFRFSFCIVKIQAEY-DGKLFDVFTEPLPNSVR  619
              T +KDG DG+G+VSV+KE   + LPDK FRFSFC+ +I+A   D +   +F E  PNSV+
Sbjct  17466  TYIKDGADGLGDVSVHKEVGDRYLPDKAFRFSFCVYRIEAILGDDEKVTIFVEKRPNSVK  17645

Query  620    TNRPLLESISDENNQVSNVVCILPIENEREILMQNRMHVKTKEGWMFHKFSFFNSMVDEK  679
              TNRP+LE+I DENN  S+ +C+LPIENER  +    + ++T+ GW  H   F NSM+DEK
Sbjct  17646  TNRPVLEAICDENNHGSSTLCLLPIENERSYMKGKIIRIQTEFGWRRHVLIFSNSMIDEK  17825

Query  680    RDRGDSGLQGSGSKYLCTLCDADKQSAKALLGSFSINRSVSECSNIAEILRVNPNALSEN  739
               DR +SGL GSGS YLCTLCDA +++A + LG+F INRS +E + IAE +R+NP+ L++
Sbjct  17826  LDRANSGLAGSGSNYLCTLCDATRETALSNLGNFVINRSYAETAEIAEYMRINPDNLTKA  18005

Query  740    ELKKISKGVKCAPLSKIEPIQKGIDATHADINLGQFFKKIIVREIAGVTKWELTQDVKPL  799
              +L KISKG+K  PL + E I+KGIDATHADIN G FF+K+I+REI  + KWE++ DVK +
Sbjct  18006  DLNKISKGIKSTPLLQAESIEKGIDATHADINCGSFFRKLIIREIVQINKWEMSGDVKSI  18185

Query  800    VQNAEFLFDQHMKCNCGINPQLMMPGNYARTLFETPH-SVLLEHISDSVRKENLSSILNI  858
              +++ E  FD H+K   GINPQLMMPGNYAR +F++ + + +L+ I D  RK  +  +L
Sbjct  18186  IKDGEVKFDLHLKKTVGINPQLMMPGNYARAIFDSKNENSILDLIEDDERKCKVRLLLQK  18365

Query  859    FLHLRKVYRCKDPLTECPFDVQNYKKCAVEMGALLLQHFDYVEWPNYLHKVIEHVQQLIE  918
              F  +R +YR  DP  + P + + YK+ A+E G +L+  FDY +WPNY+HKVIEHVQ+LIE
Sbjct  18366  FRFMRMIYRAVDPKVQYPSETKMYKETAIEFGKILINDFDYAKWPNYIHKVIEHVQELIE  18545

Query  919    DPNGPGSVGAFSSEGNEAGNKLFRHFRKNLSRRGNTYGSLCDVLKLHWLYSSKALFKLAE  978
              + NGPG+VG  SSEGNE GNK+FRHFRKNL+RRG+TYG L DVL  HWLYSS +L KL+
Sbjct  18546  NENGPGTVGGLSSEGNEGGNKIFRHFRKNLARRGSTYGGLRDVLWGHWLYSSPSLTKLSA  18725
```

```
Query  979    VEHKKVRCSLCFTSGHNKRTCP  1000
                  + RCS C + GHN R CP
Sbjct  18726  KSKSEQRCSACRSLGHNIRNCP  18791
```

Score	Expect	Method	Identities	Positives	Gaps	Frame
64.3 bits(155)	4e-07	Compositional matrix adjust.	40/120(33%)	60/120(50%)	9/120(7%)	+3

```
Query  17     QMTKNIQNFILSTDMEHGLKLKCMCRLCGVKIPINSKSH-PKENFKSAITSLYNIDIDCD  75
              +M K+   N         H    L   CR+CG       S++   P E +K   + SL+ IDI  D
Sbjct  15738  EMQKDADNI-----ANHFANLTKFCRVCGTIQKRKSRAFCPVEPYKIELLSLFKIDIQYD  15902

Query  76     SENIHPPKICTACRLRL--TRHSQAEDD-IEINLPVLFEFLPHSINCNICFVKPGRPKKI  132
               E+IHP K+C +CR +     R   + E +    +N+PV    ++PH+ NC +C    GR   I
Sbjct  15903  DESIHPKKLCNSCRSKCFHFRTKKGEGNTFSMNIPVKHLYIPHNANCKVCHRNSGRNATI  16082
```

3. TIR Detection info

TIR5 sequence was detected using BLASTN
Searched database was P.imbricata NIJJ WGS project.
Query: TIR5 containing margin (200bp) detected in scaffold WGS: NIJJ01005135.1
Subject: NIJJ01024498.1

Score	Expect	Identities	Gaps	Strand
345 bits(382)	1e-93	197/201(98%)	0/201(0%)	Plus/Plus

```
Query  1      CACTAACAAACGTCTATGCAACTTTTGCTTTTGAATAAATTATGCAAATTAAGTTaaaaa  60
              |||||||||||||||||||||||||||||||||||||||||||||||||||||||| ||||
Sbjct  14897  CACTAACAAACGTCTATGCAACTTTTGCTTTTGAATAAATTATGCAAATTAAGTTTAAAA  14956

Query  61     aaaTTAGAGTTGTCTTTCTTGCTCACCGCTTCAATTCATACCAGTACTCGTAGTGCTCCA  120
              ||||||||||||||||||||||||||||||||||||||||||||||||||||||||||||
Sbjct  14957  AAATTAGAGTTGTCTTTCTTGCTCACCGCTTCAATTCATACCAGTACTCGTAGTGCTCCA  15016

Query  121    AATTGCGGAGGAAATTCCCATAAGAATAATGTGGAGTTATCGTTCTTTGTTACTTCCTGT  180
              ||||||||||||||||||||||||||| |||||||   ||||||||||||||||||||||
Sbjct  15017  AATTGCGGAGGAAATTCCCATAAGAATCATGTGGAAATATCGTTCTTTGTTACTTCCTGT  15076

Query  181    TTTGATCGAAACATCAGTGAA  201
              |||||||||||||||||||||
Sbjct  15077  TTTGATCGAAACATCAGTGAA  15097
```

TIR3 sequence was detected using BLASTN
Searched database was P.imbricata NIJJ WGS project.

Query: **reverse complement** of TIR3 containing margin (200bp) detected in scaffold WGS: NIJJ01005135.1
Subject: NIJJ01024498.1

Score	Expect	Identities	Gaps	Strand
211 bits(233)	7e-53	118/119(99%)	0/119(0%)	Plus/Minus

```
Query  1      CACTAACAAACGTTTACAGTACTCGCTATGCTCCTACCGGAAATGCGTAAGTGCGCATGC  60
              |||||||||||||||||||||||||||||||||||||||||||||||||||||| |||||
Sbjct  21041  CACTAACAAACGTTTACAGTACTCGCTATGCTCCTACCGGAAATGCGTAAGTGCACATGC  20982

Query  61     GCAGGTCGTTATTAGCATATGATAATAAACAAATAAATCGTACACGTGCTATCAGAATG  119
              |||||||||||||||||||||||||||||||||||||||||||||||||||||||||||
Sbjct  20981  GCAGGTCGTTATTAGCATATGATAATAAACAAATAAATCGTACACGTGCTATCAGAATG  20923
```

TSD pairs in NIJJ01024498.1:

RAG1L side:

```
                    TSD      TIR5
5’ - AGGATTAAATATCGCAAACACACTAACAAACGTCTATGCAA- 3’
```

RAG2L side:

```
                             TSD
5’ - TACTGTAAACGTTTGTTAGTGAAACATGCATGTTGCAAATT- 3’
3’ - ATGACATTTGCAAACAATCACTTTGTACGTACAACGTTTAA- 5’
              TIR3
```

Pinctada imbricata (Pim)

Potentially pseudogenized copies

WGS: NIJJ01058024.1

1. Summary

Detected configuration: TIR5 - RAG1L

Detected loci	Predicted CDS	start	end	strand	Observations
RAG1L	1	7234	10314	+	Pseudogenized (incomplete)

Predicted TIR	Distance from CDS (bp)	start	strand	TSD sequence	Assessment method
TIR5 (RAG1L side)	986	11300	+		Blastn cassette margin

>PimTIR5_8024
CACTAACAAACGTCTATGCAACTTTTGCTTTTGAATAAATTATGCAAATTAAGTTAAAAAAAATTAGAGTTGTCTTT

2. TIR Detection info

TIR5 sequence was detected using BLASTN
Searched database was P.imbricata NIJJ WGS project.

Query : TIR5 containing margin (200bp) detected in scaffold WGS: NIJJ01005135.1
Subject: NIJJ01058024.1

Score	Expect	Identities	Gaps	Strand
363 bits(402)	5e-99	201/201(100%)	0/201(0%)	Plus/Plus

```
Query  1      CACTAACAAACGTCTATGCAACTTTTGCTTTTGAATAAATTATGCAAATTAAGTTaaaaa  60
              ||||||||||||||||||||||||||||||||||||||||||||||||||||||||||||
Sbjct  11300  CACTAACAAACGTCTATGCAACTTTTGCTTTTGAATAAATTATGCAAATTAAGTTAAAAA  11359

Query  61     aaaTTAGAGTTGTCTTTCTTGCTCACCGCTTCAATTCATACCAGTACTCGTAGTGCTCCA  120
              ||||||||||||||||||||||||||||||||||||||||||||||||||||||||||||
Sbjct  11360  AAATTAGAGTTGTCTTTCTTGCTCACCGCTTCAATTCATACCAGTACTCGTAGTGCTCCA  11419

Query  121    AATTGCGGAGGAAATTCCCATAAGAATAATGTGGAGTTATCGTTCTTTGTTACTTCCTGT  180
              ||||||||||||||||||||||||||||||||||||||||||||||||||||||||||||
Sbjct  11420  AATTGCGGAGGAAATTCCCATAAGAATAATGTGGAGTTATCGTTCTTTGTTACTTCCTGT  11479

Query  181    TTTGATCGAAACATCAGTGAA  201
              |||||||||||||||||||||
Sbjct  11480  TTTGATCGAAACATCAGTGAA  11500
```

WGS: NIJJ01060318.1

Summary

Detected configuration: RAG1L - RAG2L

Detected loci	Predicted CDS	start	end	strand	Observations
RAG1L	-	31685	31410	-	Pseudogenized (incomplete; short segment)
RAG2L	-	29488	30843	+	Pseudogenized (complete; stop codons)

WGS: NIJJ01009698.1

Summary

Detected configuration: RAG1L - RAG2L

Detected loci	Predicted CDS	start	end	strand	Observations
RAG1L	-	719	3280	+	Pseudogenized (incomplete; fragmented; stop codons)
RAG2L	-	3691	3395	-	Pseudogenized (incomplete; stop codons)

WGS: NIJJ01036769.1

Summary

Detected configuration: RAG1L - RAG2L

Detected loci	Predicted CDS	start	end	strand	Observations
RAG1L	-	1835	2311	+	Pseudogenized (incomplete; short segment)
RAG2L	-	3188	2251	-	Pseudogenized (incomplete; stop codons)

WGS: NIJJ01043600.1

Summary

Detected configuration: RAG1L - RAG2L

Detected loci	Predicted CDS	start	end	strand	Observations
RAG1L	-	4703	3166	-	Pseudogenized (incomplete; stop codons)
RAG2L	-	1744	2001	+	Pseudogenized (stop codons)

WGS: NIJJ01046456.1

Summary

Detected configuration: RAG1L - RAG2L

Detected loci	Predicted CDS	start	end	strand	Observations
RAG1L	-	31700	31008	-	Pseudogenized (incomplete; short segment)
RAG2L	-	29922	30756	+	Pseudogenized (incomplete; stop codons)

WGS: NIJJ01052071.1

Summary

Detected configuration: RAG1L - RAG2L

Detected loci	Predicted CDS	start	end	strand	Observations
RAG1L	-	4658	2763	-	Pseudogenized (incomplete)
RAG2L	-	912	1708	+	Pseudogenized (incomplete)

WGS: NIJJ01055763.1

Summary

Detected configuration: RAG1L - RAG2L

Detected loci	Predicted CDS	start	end	strand	Observations
RAG1L	-	1674	1871	+	Pseudogenized (incomplete; short segment)
RAG2L	-	4363	3719	-	Pseudogenized (incomplete; stop codons)

WGS: NIJJ01026157.1

Summary

Detected configuration: RAG1L

Detected loci	Predicted CDS	start	end	strand	Observations
RAG1L	-	17422	19295	+	Pseudogenized (incomplete)

WGS: NIJJ01013721.1

Summary

Detected configuration: RAG1L

Detected loci	Predicted CDS	start	end	strand	Observations
RAG1L	-	631	3580	+	Pseudogenized (incomplete; stop codons)

WGS: NIJJ01015382.1

Summary

Detected configuration: RAG1L

Detected loci	Predicted CDS	start	end	strand	Observations
RAG1L	-	1188	10789	+	Pseudogenized (incomplete; fragmented; stop codons)

WGS: NIJJ01024466.1

Summary

Detected configuration: RAG1L

Detected loci	Predicted CDS	start	end	strand	Observations
RAG1L	-	47426	51084	+	Pseudogenized (fragmented; stop codons)

WGS: NIJJ01024492.1

Summary

Detected configuration: RAG1L

Detected loci	Predicted CDS	start	end	strand	Observations
RAG1L	-	5825	2467	-	Pseudogenized (fragmented; stop codons)

WGS: NIJJ01030772.1

Summary

Detected configuration: RAG1L

Detected loci	Predicted CDS	start	end	strand	Observations
RAG1L	-	8581	6551	-	Pseudogenized (incomplete; fragmented; stop codons)

WGS: NIJJ01031045.1

Summary

Detected configuration: RAG1L

Detected loci	Predicted CDS	start	end	strand	Observations
RAG1L	-	561	6139	+	Pseudogenized (incomplete; fragmented; stop codons)

WGS: NIJJ01036768.1

Summary

Detected configuration: RAG1L

Detected loci	Predicted CDS	start	end	strand	Observations
RAG1L	-	42221	43638	+	Pseudogenized (incomplete; fragmented; stop codons)

WGS: NIJJ01061962.1

Summary

Detected configuration: RAG1L

Detected loci	Predicted CDS	start	end	strand	Observations
RAG1L	-	9990	14029	+	Pseudogenized (incomplete; fragmented; stop codons)

WGS: NIJJ01066387.1

Summary

Detected configuration: RAG1L

Detected loci	Predicted CDS	start	end	strand	Observations
RAG1L	-	7450	10108	+	Pseudogenized (incomplete; fragmented; stop codons)

WGS: NIJJ01020733.1

Summary

Detected configuration: RAG2L

Detected loci	Predicted CDS	start	end	strand	Observations
RAG2L	-	6236	6577	+	Pseudogenized (incomplete; short segment)

WGS: NIJJ01030768.1

Summary

Detected configuration: RAG2L

Detected loci	Predicted CDS	start	end	strand	Observations
RAG2L	-	4360	5558	+	Pseudogenized (fragmented; stop codons)

WGS: NIJJ01043599.1

Summary

Detected configuration: RAG2L

Detected loci	Predicted CDS	start	end	strand	Observations
RAG2L	-	1128	1838	+	Pseudogenized (incomplete; stop codons)

WGS: NIJJ01082005.1

Summary

Detected configuration: RAG2L

Detected loci	Predicted CDS	start	end	strand	Observations
RAG2L	-	13075	14243	+	Pseudogenized (incomplete; fragmented; stop codons)

PROTOSTOMIA - NEMERTEA

Notospermus Geniculatus (Nge)

(TaxID: 416868; Protostomia; Nemertea; Heteronemertea)

WGS: NMRB01003820.1

1. Summary

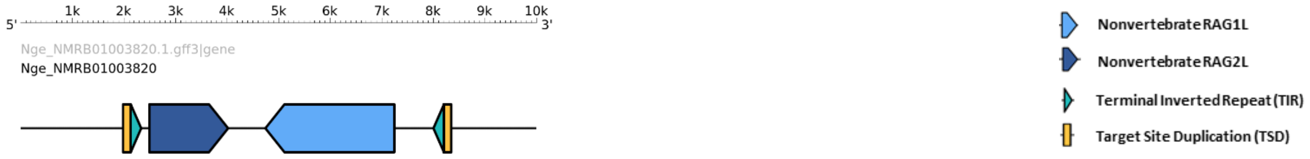

Detected configuration: TSD - TIR5 - RAG1L - RAG2L - TIR3 - TSD

Detected loci	Predicted CDS	start	end	strand	Observations
RAG1L	1	47679	45172	-	complete; no stop codon
RAG2L	1	42918	44450	+	complete; no stop codons

Predicted TIR	Distance from CDS (bp)	start	strand	TSD sequence	Assessment method
TIR5 (RAG1L side)	895	48574	-	5'-AGGGT-TIR5	Homology variation
TIR3 (RAG2L side)	156	42762	+	TIR3-AGGGT-3'	Homology variation

```
NgeTIR5_3820               5'CACACCGAAATGTTGTGTGTACGCGTAATTAGTCCTGTAGGCTGCC-AT 3'
                             |||| |||||||||    |||| || | ||   ||     || ||| |   ||
NgeTIR3_3820 (rev compl)   3'CACATCGAAATGTTTCCTGTA-GC-TCATGTGTGTAATAAGCTACGCAT 5'
```

Selected predicted protein product

NgeRAG1L_D_3820

```
MRVNTNIKSNLAGMSIDICFLLAFS
MDQYHKQVLQRICRLCSGDLKKVKRQNNFFDVSKAAVVLQLHYSISVGDESEAVFPRCICN
SCRNKVDRLKQTAVSKNLTAPKFLPHQECDCFCVGRGSLLKLPLPSEPVQIEPAEVVISSD
HSYSGSGPNECGTGTSNGTTNDV
MSPQPRKKCRLQQVSRHHIKQRRLKDLISSVGSFCDDRMEDPVDAMYAVLIQKLLDDGRK
KEANKILSVWQNKGEELEMSAQDCLAVRVRTLQSKTRYRQQYSLQRDNTNSASLKPPYQL
DAAEATFMPGHCAYKLMKDSVVIAEQSISTNDQYEPKDIMDQFKNFPTDFPVPNLAGVEF
SFVSAVAKTLWELESLIEMRLNELGLPLSTQIETIFKDGGDGLGDVCLKTQKADFARTDK
IFRYSFCCVSCSAVVGDKKHLLFRETFPNNTRPVILCHCDESDRPSISTLLANVSREREM
MQDGTLVLVDPDTGDDVRQHHLHFLYTMVDEKFERTSSGLQGSGSRFICTLCYAATNTCQ
ADVGSFSRCRTYDETVSLYKYRLENPDKLNENDLAERCKGVNSIPLIKSDATDRSVDSTH
ANINIGRVFKRILVREIAEIYEWAENDRNKKGLQHAEKNLDQHLMKTLGLQSKMIMPGNY
ARILFLDKNAETVSELIKNPDRKQHFKCILGLFSKLKAVYSSKDPTSSVPEETACYKENA
LQFAFHLGEHFNYVPWSNYLHKLIEHVDEIVQDPRCLHSVGLLSSEGGECGNKLYRFIRR
SLSRQDTAGDCDLRDALVFHWIYTSKCIQKLATSCKAPYNCRKCGQVGHNSRTCDENGDT
TTVDQGMDMNSNTSTSDDLEMDMYGDSDLSE
```

Prediction performed with FGENESH (*C.elegans* dataset)

NgeRAG2L_D_3820

```
MAEVSVLNRHDRESGDEFFNNARFYSVLKAVQNRRKVTKKAIVEEASKGALFSGSG
HLSWLIFYNDPRQHTAAVTNNGSGDEKTFSVAVLGGMHAMKPLTNLSGTVYSYAFKVNDD
DIKILSVAECKCSGAQIALRPFVHSACIVETKATRESSRSRRTGPGGARSAMISICSIWG
GLDPNRMLPHNDLTILENFGRDQFTARLVCQEGTKNVQTGTVPEARYGHTLSGLHYQGSL
VGAVLYGGVTVKNTTQGNEHSTQDGTLYFLDLENYQWKKLSELAPVAYHTSCEIRDLTLV
FIGGLSQLCDNRVTSTQRMSIQKITIVELNSTIDQVTVQNLSLSFLDPALDNVFLSGHSA
NICSPNQLLIFGGYQQHISDMNTKIPSRTYYLVDITSLSIQAKEAPVGFDMAGHTSVRLD
HCSLFFHGGANEHLFTMTTKRMELGVCEAEKCIVNDHFSPGEIVTKLQCTLCDAFFHSCC
DQGESHTSHSQEDIRFTCPKCRPKGKRQRKNKARN
```

Prediction performed with FGENESH (*C.elegans* dataset)

A very similar transcript was found (TSA: GFRY01012761 - 97% protein sequence ident with NgeRAG1L_D_3820). However, the differences along the UTR region indicates that **this transcript corresponds most likely to a different DNA loci than NgeRAG1L_D_3820, not detected in the WGS data.**

```
                1                                                                                                      90
NgeRAG1LD_3820  MRVNTNIKSN LAGMSIDICF LLAFSMDQYH KQVLQRICRL CSGDLKKVKR QNNFFDVSKA AVVLQLHYSI SVGDESEAVF PRCICNSCRN
GFRY01012761.1

                91                                                                                                    180
NgeRAG1LD_3820  KVDRLKQTAV SKNLTAPKFL PHQECDCFCV GRGSLLKLPL PSEPVQIEPA EVVISSDHSY SGSGPNECGT GTSNGTTNDV MSPQPRKKCR
GFRY01012761.1                                                                                         MSPQPRKKCR

                181                                                                                                   270
NgeRAG1LD_3820  LQQVSRHHIK QRRLKDLISS VGSFCDDRME DPVDAMYAVL IQKLLDDGRK KEANKILSVW QNKGEELEMS AQDCLAVRVR TLQSKTRYRQ
GFRY01012761.1  LQQVSRHHIK QRRLKDLISS VGSFCDDRME DPVDAMYAVL IQKLLDDGRK KEADKILSVW QNNGEELEMS AQDCLAVRVR TLQSKTRYRQ

                271                                                                                                   360
NgeRAG1LD_3820  QYSLQRDNTN SASLKPPYQL DAAEATFMPG HCAYKLMKDS VVIAEQSIST NDQYEPKDIM DQFKNFPTDF PVPNLAGVEF SFVSAVAKTL
GFRY01012761.1  QYSLQRDNTN SASLKPPYQL DAAEATFMPG HCAYKLMKDS VVIAEQSIST NDQYEPKDIM DQFKNFPTDF PVPNLAGVEF SFVSAVAKTL

                361                                                                                                   450
NgeRAG1LD_3820  WELESLIEMR LNELGLPLST QIETIFKDGG DGLGDVCLKT QKADFARTDK IFRYSFCCVS CSAVVGDKKH LLFRETFPN- --NTRPVILC
GFRY01012761.1  WELESLIEMR LNELGLPLST QIETIFKDGG DGLGDVSLKT QKADFARTDK IFRYSFCCVS CSAVVGDKKH LLFRETFPSS VDNTRPVILC

                451                                                                                                   540
NgeRAG1LD_3820  HCDESDRPSI STLLANVSRE REMMQDGTLV LVDPDTGDDV RQHHLHFLYT MVDEKFERTS SGLQGSGSRF ICTLCYAATN TCQADVGSFS
GFRY01012761.1  HCDESDSPSI STLLANVSRE REMMQDGTLV LVDPDTGDDV RQHHLHFLYT MVDEKFERTS SGLQGSGSRF ICTLCYAATN TCQADVGSFS

                541                                                                                                   630
NgeRAG1LD_3820  RCRTYDETVS LYKYRLENPD KLNENDLAER CKGVNSIPLI KSDATDRSVD STHANINIGR VFKRILVREI AEIYEWAEND RNKKGLQHAE
GFRY01012761.1  RCRTYDETVS LYKYRLENPD KLNENDLAER CKGVNSIPLI KSDATDRSVD STHANINIGR VFKRILVREI AEIYEWAEND GNKKGLQHAE

                631                                                                                                   720
NgeRAG1LD_3820  KNLDQHLMKT LGLQSKMIMP GNYARILFLD KNAETVSELI KNPDRKQHFK CILGLFSKLK AVYSSKDPTS SVPEETACYK ENALQFAFHL
GFRY01012761.1  KTLDQHLMKT LGLQSKLVMP GNYARILFLD KNAETVSELI KNPDRKQHFK SILGLFSKLK AVYSSKDPTS SVPEETACYK ENALQFAFHL

                721                                                                                                   810
NgeRAG1LD_3820  GEHFNYVPWS NYLHKLIEHV DEIVQDPRCL HSVGLLSSEG GECGNKLYRF IRRSLSRQDT AGDCDLRDAL VFHWIYTSKC IQKLATSCKA
GFRY01012761.1  GEHFNYVPWS NYLHKLIEHV DEIVQDPRCL HSVGLLSSEG GECGNKLYRF IRRSLSRQDT AGDCDLRDAL VFHWIYTSKC IQNLASSYKA

                811                                                  864
NgeRAG1LD_3820  PYNCRKCGQV GHNSRTCDEN GDTTTVDQGM DMNSNTSTSD DLEMDMYGDS DLSE
GFRY01012761.1  PYSCRKCGQV GHNSRTCDEN GDTTTVDQGM DMNSNTSTGD DLEMDIYGDS DLSE
```

2. RAGL Detection info

Method: TBLASTN

Database searched: Whole-genome shotgun contigs (WGS) + only in Nemertea clade (taxid:6217).

2.1. RAG1L

Query: CviRAG1L_B_Biv1_0007 (previously described)
Subject: NMRB01003820.1 Length: 51922 Number of Matches: 1

Score	Expect	Method	Identities	Positives	Gaps	Frame
403 bits(1035)	2e-117	Compositional matrix adjust.	244/654(37%)	363/654(55%)	20/654(3%)	-2

```
Query  359    KLPLHSVSAKHTRHRRLKPIISQVNEFCNAQEENKSDVLFFMLKDHLKEINDPRWKQ---  415
              K  L  VS  H + RRLK +IS V  FC+ + E+  D ++ +L   L  ++D R K+
Sbjct  47223  KCRLQQVSRHHIKQRRLKDLISSVGSFCDDRMEDPVDAMYAVLIQKL--LDDGRKKEANK  47050

Query  416    VESLWL--GNNSTLSPEQCLALRVDLLQSKGQYRSQYDFLSQN-NVHVFQAPSKMESCEN  472
              + S+W   G    +S + CLA+RV  LQSK +YR QY     N N    + P ++++ E
Sbjct  47049  ILSVWQNKGEELEMSAQDCLAVRVRTLQSKTRYRQQYSLQRDNTNSASLKPPYQLDAAEA  46870

Query  473    LFMPSASIFQIIDNDGNVLLQN-SENPCTEPLNVNECFLPGFVELATPNCMGVRFSYFEA  531
               FMP    ++++ +   +  Q+ S N   EP ++ + F     +   PN  GV FS+  A
Sbjct  46869  TFMPGHCAYKLMKDSVVIAEQSISTNDQYEPKDIMDQFKNFPTDFPVPNLAGVEFSFVSA  46690

Query  532    LSLTLQELEPEILFGLKKHGLNIEDVLFLTTVKDGCDGMGEVSVYKEKDFKMLPDKVFRF  591
              ++ TL ELE  I   L + GL +      T  KDG DG+G+V +  +K      DK+FR+
Sbjct  46689  VAKTLWELESLIEMRLNELGLPLS-TQIETIFKDGGDGLGDVCLKTQKADFARTDKIFRY  46513

Query  592    SFCIVKIQAEYDGKLFDVFTEPLPNSVRTNRPLLESISDENNQVSNVVCILPIENEREIL  651
              SFC V   A    K   +F E  PN+ R   P++    DE+++ S    +  +  ERE++
Sbjct  46512  SFCCVSCSAVVGDKKHLLFRETFPNNTR---PVILCHCDESDRPSISTLLANVSREREMM  46342

Query  652    MQNRMHV---KTKEGWMFHKFSFFNSMVDEKRDRGDSGLQGSGSKYLCTLCDADKQSAKA  708
                  + +    T +    H   F  +MVDEK +R  SGLQGSGS+++CTLC A   + +A
Sbjct  46341  QDGTLVLVDPDTGDDVRQHHLHFLYTMVDEKFERTSSGLQGSGSRFICTLCYAATNTCQA  46162

Query  709    LLGSFSINRSVSECSNIAEILRVNPNALSENELKKISKGVKCAPLSKIEPIQKGIDATHA  768
               +GSFS  R+  E  ++ +    NP+ L+EN+L +  KGV   PL K +   + +D+THA
Sbjct  46161  DVGSFSRCRTYDETVSLYKYRLENPDKLNENDLAERCKGVNSIPLIKSDATDRSVDSTHA  45982

Query  769    DINLGQFFKKIIVREIAGVTKWELTQDVKPLVQNAEFLFDQHMKCNCGINPQLMMPGNYA  828
              +IN+G+ FK+I+VREIA + +W      K  +Q+AE   DQH+    G+  +++MPGNYA
Sbjct  45981  NINIGRVFKRILVREIAEIYEWAENDRNKKGLQHAEKNLDQHLMKTLGLQSKMIMPGNYA  45802

Query  829    RTLF-ETPHSVLLEHISDSVRKENLSSILNIFLHLRKVYRCKDPLTECPFDVQNYKKCAV  887
              R LF +     + E I +  RK++   IL +F  L+ VY  KDP +  P +   YK+ A+
Sbjct  45801  RILFLDKNAETVSELIKNPDRKQHFKCILGLFSKLKAVYSSKDPTSSVPEETACYKENAL  45622

Query  888    EMGALLLQHFDYVEWPNYLHKVIEHVQQLIEDPNGPGSVGAFSSEGNEAGNKLFRHFRKN  947
              +    L +HF+YV W NYLHK+IEHV ++++DP    SVG  SSEG E GNKL+R  R++
Sbjct  45621  QFAFHLGEHFNYVPWSNYLHKLIEHVDEIVQDPRCLHSVGLLSSEGGECGNKLYRFIRRS  45442

Query  948    LSRRGNTYG--SLCDVLKLHWLYSSKALFKLAEVEHKKVRCSLCFTSGHNKRTC  999
              LSR+ +T G   L D L  HW+Y+SK + KLA        C  C   GHN RTC
Sbjct  45441  LSRQ-DTAGDCDLRDALVFHWIYTSKCIQKLATSCKAPYNCRKCGQVGHNSRTC  45283
```

2.2 RAG2L

Query: PflRAG2L_B from P.flava (Morales Poole et al, 2017)
Subject: NMRB01003820.1 Length: 51922 Number of Matches: 1

Score	Expect	Method	Identities	Positives	Gaps	Frame
117 bits(292)	3e-26	Compositional matrix adjust.	125/509(25%)	232/509(45%)	84/509(16%)	+3

```
Query  13     RFIPLTEGYSDKRKMSRKRFFE---LGEYFPPEGHMNVSII-------------NGDGVV  56
              RF  + +   ++RK+++K   E    G  F   GH++  I              NG G
Sbjct  42984  RFYSVLKAVQNRRKVTKKAIVEEASKGALFSGSGHLSWLIFYNDPRQHTAAVTNNGSGDE  43163

Query  57     TVYTLG--GGRWKEESTWSLSNELYSLSFTLDDTDVDVESVQKFTTRGAMLSP---LHAA  111
                +++   GG    +   +LS  +YS +F ++D D+ + SV +    GA ++    +H+A
Sbjct  43164  KTFSVAVLGGMHAMKPLTNLSGTVYSYAFKVNDDDIKILSVAECKCSGAQIALRPFVHSA  43343

Query  112    VMLNI-----------STPDKVK------LLVWGGYHLGSLFCTNEAVTME---IQRKTA  151
               ++             + P   +        +WGG     +   N+   +E     + TA
Sbjct  43344  CIVETKATRESSRSRRTGPGGARSAMISICSIWGGLDPNRMLPHNDLTILENFGRDQFTA  43523

Query  152    TCVIYKDPNDMSFHLPDEKHQSGDIPSARCGHTLTPIPGQHA---AVLFGGAEMPNRFRR  208
                V  +   ++         Q+G +P AR GHTL+ +  Q +   AVL+GG  + N
Sbjct  43524  RLVCQEGTKNV---------QTGTVPEARYGHTLSGLHYQGSLVGAVLYGGVTVKN---T  43667

Query  209    VPSFEQDTKDGHFYLLNTDSLSWKKLNVPQLEPRAFHTATYLSSSSTICYVGGVTYR-DQ  267
                  E  T+DG  Y L+ ++  WKKL+  +L P A+HT+  +    T+ ++GG++   D
Sbjct  43668  TQGNEHSTQDGTLYFLDLENYQWKKLS--ELAPVAYHTSCEI-RDLTLVFIGGLSQLCDN  43838

Query  268    K--PYKQHQINEVTLLSISAT-NEYAVKSVLLSESLP----YHVSMHGA-LQFNDQVIVY  319
              +    ++  I ++T++ +++T ++  V+++ LS   P      +S H A +   +Q++++
Sbjct  43839  RVTSTQRMSIQKITIVELNSTIDQVTVQNLSLSFLDPALDNVFLSGHSANICSPNQLLIF  44018

Query  320    GGYVTPCALYQSNARPAKPSSSMFLLNTTSEVLTRLEAPDSFASAGLSMVSLDKTAIMGL  379
              GGY      + S+     PS + +L++ TS  +   EAP  F  AG + V LD  ++
Sbjct  44019  GGY----QQHISDMNTKIPSRTYYLVDITSLSIQAKEAPVGFDMAGHTSVRLDHCSLFFH  44186

Query  380    GGTHKNIYFCTSKAMCPVPCDLEDECTIKDTPEISP---IAWIQCEGKCKRWLHQFCIKL  436
              GG +++++  T+K M    C+ E +C + D    SP   +  +QC   C  + H  C +
Sbjct  44187  GGANEHLFTMTTKRMELGVCEAE-KCIVND--HFSPGEIVTKLQCT-LCDAFFHSCCDQG  44354

Query  437    EVIPKG-----KYVCNSCKSTGGRKRSRK  460
              E          ++ C  C+  G R+R  K
Sbjct  44355  ESHTSHSQEDIRFTCPKCRPKGKRQRKNK  44441
```

3. TIR Detection info

Aligned N.geniculatus RAG1L-RAG2L flanking regions had been searched for homology variations both at left and right flanking regions. Detected homology dropping regions were inspected for TIR and TSD presence. For more details please see methods section.

A. RAG1L facing margins alignment (left)

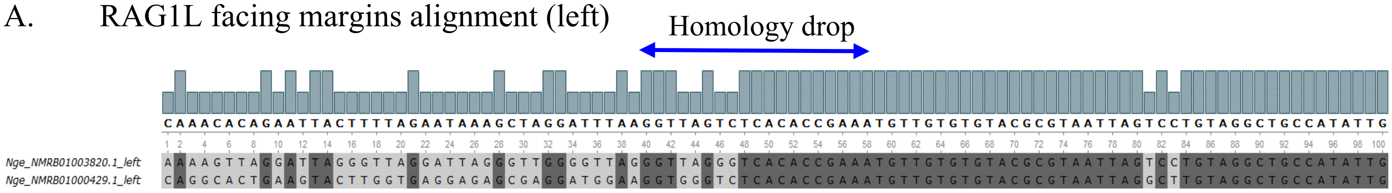

B. RAG2L facing margins alignment (right)

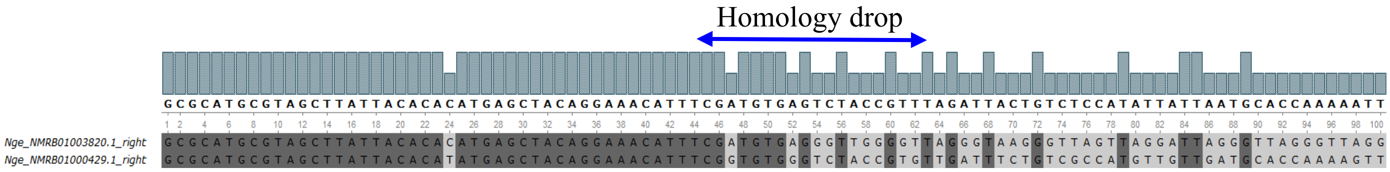

C. Alignment of Left homology drop regions with the reverse complement of right homology drop region in order to spot inverted repeat regions.

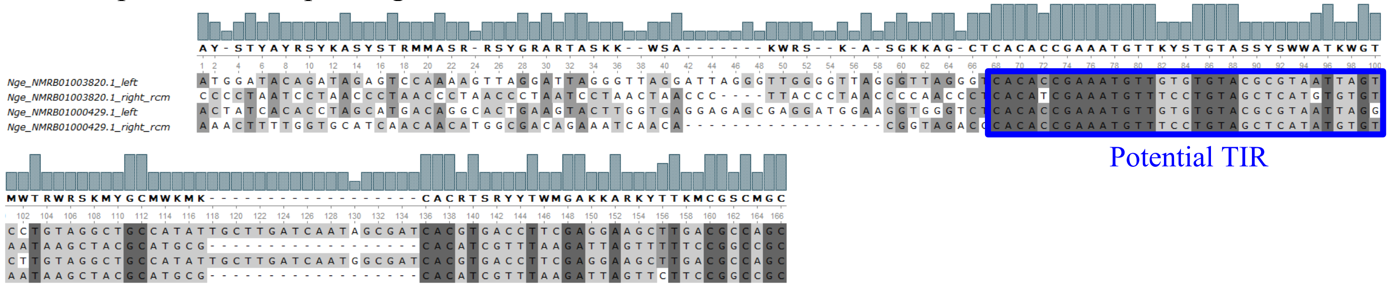

D. TSD pairs in NMRB01003820.1:

RAG1L side:

TSD TIR5

5′ - ATTAGGGTTAGGATTAGGGTTGGGGTTAGGGTTAGGGTCACACCGAAATGTTGTGTGTACGCGTAATTAGT - 3′

Short tandem repeats region

RAG2L side:

TSD Short tandem repeats region

5′ - ATTACACACATGAGCTACAGGAAACATTTCGATGTGAGGGTTGGGGTTAGGGTAAGGGTTAGTTAGGATTA - 3′

3′ - TAATGTGTGTACTCGATGTCCTTTGTAAAGCTACACTCCCAACCCCAATCCCATTCCCAATCAATCCTAAT - 5′

TIR3

WGS: NMRB01000429.1

1. Summary

Detected configuration: TSD - TIR5 - RAG1L - RAG2L - TIR3 - TSD

Detected loci	Predicted CDS	start	end	strand	Observations
RAG1L	1	179129	180331	+	Incomplete due to assembly gaps; no stop codon
RAG2L	1	181854	181090	-	Incomplete due to assembly gaps; no stop codons

These were not further used as they are incomplete, and > 95% identical with NgeRAG1/2L_D_3820

Predicted TIR	Distance from CDS (bp)	start	strand	TSD sequence	Assessment method
TIR5 (RAG1L side)	881	178248	+	5'- GGTCT-TIR5	Homology variation
TIR3 (RAG2L side)	156	182010	-	TIR3- GGTCT-3'	Homology variation

```
NgeTIR5_0049              5′CACACCGAAATGTTGTGTGTACGCGTAATTAGGCTTGTAGGCTGCCATATT 3′
                            ||||||||||||||   |||| || | ||  |  |  || ||| |
NgeTIR3_0049 (rev compl)  3′CACACCGAAATGTTTCCTGTA-GC-TCATATGTGTAATAAGCTACGCATGC 5′
```

2. RAGL Detection info

Method: TBLASTN
Database searched: Whole-genome shotgun contigs (WGS) + only in Nemertea clade (taxid:6217).

2.1. RAG1L

Query: **NgeRAG1L_D_3820 (previously described)**
Subject: **NMRB01000429.1 Length: 385858 Number of Matches: 1**

Score	Expect	Method	Identities	Positives	Gaps	Frame
814 bits(2102)	0.0	Compositional matrix adjust.	390/401(97%)	394/401(98%)	0/401(0%)	+2

```
Query  1       MDQYHKQVLQRICRLCSGDLKKVKRQNNFFDVSKAAVVLQLHYSISVGDESEAVFPRCIC  60
               MDQYHKQVLQRICRLCSGDLKKVKRQNNFFDVSKAAVVLQLHYSISVGDESEAVFPRCIC
Sbjct  179129  MDQYHKQVLQRICRLCSGDLKKVKRQNNFFDVSKAAVVLQLHYSISVGDESEAVFPRCIC  179308

Query  61      NSCRNKVDRLKQTAVSKNLTAPKFLPHQECDCFCVGRGSLLKLPLPSEPVQIEPAEVVIS  120
               N CRNKVDRLKQTAVSKNLTAP FLPHQ+CDCFCVGRGSLLKLPLPSEPVQIEP+EVVIS
Sbjct  179309  NKCRNKVDRLKQTAVSKNLTAPNFLPHQDCDCFCVGRGSLLKLPLPSEPVQIEPSEVVIS  179488

Query  121     SDHSYSGSGPNECGTGTSNGTTNDVMSPQPRKKCRLQQVSRHHIKQRRLKDLISSVGSFC  180
               SDHSYSGSGPN CGTGTSNG TNDVMSPQPRKKCRLQQVSRHHIKQRRLKDLISSVGSFC
Sbjct  179489  SDHSYSGSGPNACGTGTSNGNTNDVMSPQPRKKCRLQQVSRHHIKQRRLKDLISSVGSFC  179668

Query  181     DDRMEDPVDAMYAVLIQKLLDDGRKKEANKILSVWQNKGEELEMSAQDCLAVRVRTLQSK  240
               DDRMEDPVDAMYAVLIQKLLDDGRKKEA+KILSVWQN GEELEMSAQDCLAVRVRTLQSK
Sbjct  179669  DDRMEDPVDAMYAVLIQKLLDDGRKKEADKILSVWQNNGEELEMSAQDCLAVRVRTLQSK  179848
```

```
Query  241     TRYRQQYSLQRDNTNSASLKPPYQLDAAEATFMPGHCAYKLMKDSVVIAEQSISTNDQYE  300
               TRYRQQYSLQRDNTNSASL PPYQLDAAEATFMPGHCAYKLMKDSVVIAEQSISTN+QYE
Sbjct  179849  TRYRQQYSLQRDNTNSASLNPPYQLDAAEATFMPGHCAYKLMKDSVVIAEQSISTNEQYE  180028

Query  301     PKDIMDQFKNFPTDFPVPNLAGVEFSFVSAVAKTLWELESLIEMRLNELGLPLSTQIETI  360
               PKDIMDQFKNFPTDFPVPNLAGVEFSFVSAVAKTLWELESLIEMRLNELGLPLSTQIETI
Sbjct  180029  PKDIMDQFKNFPTDFPVPNLAGVEFSFVSAVAKTLWELESLIEMRLNELGLPLSTQIETI  180208

Query  361     FKDGGDGLGDVCLKTQKADFARTDKIFRYSFCCVSCSAVVG  401
               FKDGGDGLGDV LKTQKADFARTDKIFRYSFCCVSCSAVVG
Sbjct  180209  FKDGGDGLGDVSLKTQKADFARTDKIFRYSFCCVSCSAVVG  180331
```

2.2 RAG2L

Query: NgeRAG2L_D_3820 (previously described)
Subject: NMRB01000429.1 Length: 385858 Number of Matches: 1

Score	Expect	Method	Identities	Positives	Gaps	Frame
492 bits(1266)	1e-154	Compositional matrix adjust.	244/260(94%)	247/260(95%)	8/260(3%)	-2

```
Query  1       MAEVSVLNRHDRESGDEFFNNARFYSVLKAVQNRRKVTKKAIVEEASKGALFSGSGHLSW  60
               MAEVSV    +RESGDEFFNNARFYSVLKAV NRRKVTKKAIVEEASKGALFSG GHLSW
Sbjct  181854  MAEVSV----NRESGDEFFNNARFYSVLKAVHNRRKVTKKAIVEEASKGALFSGCGHLSW  181687

Query  61      LIFYNDPRQHTAAVTNNGSG---DEKTFSVAVLGGMHAMKPLTNLSGTVYSYAFKVNDDD  117
               LI YNDPRQHTAAVTNNGSG   +EK  SVAVLGGMHAMK LTNLSGTVYSYAFKVNDDD
Sbjct  181686  LI-YNDPRQHTAAVTNNGSGMSGEEKMLSVAVLGGMHAMKLLTNLSGTVYSYAFKVNDDD  181510

Query  118     IKILSVAECKCSGAQIALRPFVHSACIVETKATRESSRSRRTGPGGARSAMISICSIWGG  177
               IKILSVAECKCSGAQIALRPFVHSACIVETKATRESSRSRRTGPGGARSAMISICSIWGG
Sbjct  181509  IKILSVAECKCSGAQIALRPFVHSACIVETKATRESSRSRRTGPGGARSAMISICSIWGG  181330

Query  178     LDPNRMLPHNDLTILENFGRDQFTARLVCQEGTKNVQTGTVPEARYGHTLSGLHYQGSLV  237
               LDPNRMLPHNDLTILENFGRDQFTARLVCQEGTKNVQTGTVPEARYGHTLSG+HYQGSLV
Sbjct  181329  LDPNRMLPHNDLTILENFGRDQFTARLVCQEGTKNVQTGTVPEARYGHTLSGVHYQGSLV  181150

Query  238     GAVLYGGVTVKNTTQGNEHS  257
               GAVLYGGVTVKNTTQGNEHS
Sbjct  181149  GAVLYGGVTVKNTTQGNEHS  181090
```

3. TIR Detection info

See scaffold WGS: NMRB01003820.1 (page 60).

TSD pairs in NMRB010004291:

RAG1L side:

```
                                       TSD       TIR5
5' - GTACTTGGTGAGGAGAGCGAGGATGGAAGGTGGGTCTCACACCGAAATGTTGTGTGTACGCGTAATTAGGC - 3'
```

RAG2L side:

```
                                        TSD
5' - ACACATATGAGCTACAGGAAACATTTCGGTGTGGGTCTACCGTGTTGATTTCTGTCGCCATGTTGTTGATG - 3'
3' - TGTGTATACTCGATGTCCTTTGTAAAGCCACACCCAGATGGCACAACTAAAGACAGCGGTACAACAACTAC - 5'
             TIR3
```

WGS: NMRB01000029.1

1. Summary

Detected configuration: RAG1L - RAG2L

Detected loci	Predicted CDS	start	end	strand	Observations
RAG1L	1	885075	887105	+	N-term missing ; no stop codon
RAG2L	1	888649	887804	-	N-term missing; no stop codons

These were not further used as they are incomplete, and > 90% identical with NgeRAG1/2L_D_3820

2. RAGL Detection info

Method: TBLASTN
Database searched: Whole-genome shotgun contigs (WGS) + only in Nemertea clade (taxid:6217).

2.1. RAG1L

Query: NgeRAG1L_D_3820 (previously described)
Subject: NMRB01000029.1 Length: 995339 Number of Matches: 1

Score	Expect	Method	Identities	Positives	Gaps	Frame
1351 bits(3497)	0.0	Compositional matrix adjust.	649/677(96%)	659/677(97%)	3/677(0%)	+3

```
Query  163     HIKQRRLKDLISSVGSFCDDRMEDPVDAMYAVLIQKLLDDGRKKEANKILSVWQNKGEEL  222
               H+ ++    LISSVGSFCDDRMEDPVDAMYAVLIQKLLDDGRKKEA+KILSVWQN GEEL
Sbjct  885075  HLPKQWNTKLISSVGSFCDDRMEDPVDAMYAVLIQKLLDDGRKKEADKILSVWQNNGEEL  885254

Query  223     EMSAQDCLAVRVRTLQSKTRYRQQYSLQRDNTNSASLKPPYQLDAAEATFMPGHCAYKLM  282
               EMSAQDCLAVRVRTLQSKTRYRQQYSLQRDNTNSASL PPYQLDAAEATFMPGHCAYKLM
Sbjct  885255  EMSAQDCLAVRVRTLQSKTRYRQQYSLQRDNTNSASLNPPYQLDAAEATFMPGHCAYKLM  885434

Query  283     KDSVVIAEQSISTNDQYEPKDIMDQFKNFPTDFPVPNLAGVEFSFVSAVAKTLWELESLI  342
               KDSVVIAEQSISTN+QYEPKDIMDQFKNFPTDFPVPNLAGVEFSFVSAVAKTLWELESLI
Sbjct  885435  KDSVVIAEQSISTNEQYEPKDIMDQFKNFPTDFPVPNLAGVEFSFVSAVAKTLWELESLI  885614

Query  343     EMRLNELGLPLSTQIETIFKDGGDGLGDVCLKTQKADFARTDKIFRYSFCCVSCSAVVGD  402
               EMRLNELGLPLSTQIETIFKDGGDGLGDV LKTQKADFARTDKIFRYSFCCVSCSAVVGD
Sbjct  885615  EMRLNELGLPLSTQIETIFKDGGDGLGDVSLKTQKADFARTDKIFRYSFCCVSCSAVVGD  885794

Query  403     KKHLLFRETFPN---NTRPVILCHCDESDRPSISTLLANVSREREMMQDGTLVLVDPDTG  459
               KKHLLFRETFPN   NTRPVILCHCDESDRPSISTLLANVSREREMMQDGTLVLVDPDTG
Sbjct  885795  KKHLLFRETFPNSVDNTRPVILCHCDESDRPSISTLLANVSREREMMQDGTLVLVDPDTG  885974

Query  460     DDVRQHHLHFLYTMVDEKFERTSSGLQGSGSRFICTLCYAATNTCQADVGSFSRCRTYDE  519
               DDVRQHHLHFLYTMVDEKFERTSSGLQGSGSRFICTLCYAATNTCQADVGSFSRCRTYDE
Sbjct  885975  DDVRQHHLHFLYTMVDEKFERTSSGLQGSGSRFICTLCYAATNTCQADVGSFSRCRTYDE  886154

Query  520     TVSLYKYRLENPDKLNENDLAERCKGVNSIPLIKSDATDRSVDSTHANINIGRVFKRILV  579
               TVSLYKYRLENPDKLNENDLAERCKGVNSIPLIKSDATDRSVDSTHANINIGRVFKRILV
Sbjct  886155  TVSLYKYRLENPDKLNENDLAERCKGVNSIPLIKSDATDRSVDSTHANINIGRVFKRILV  886334
```

```
Query  580     REIAEIYEWAENDRNKKGLQHAEKTNDQHLMKTLGLQSKMIMPGNYARILFLDKNAETVS  639
               REIAEIYEWAEND NKKGLQHAEKT DQHLMKTLGLQSK++MPGNYARILFLDKNAETVS
Sbjct  886335  REIAEIYEWAENDGNKKGLQHAEKTLDQHLMKTLGLQSKLVMPGNYARILFLDKNAETVS  886514

Query  640     ELIKNPDRKQHFKCILGLFSKLKAVYSSKDPTSSVPEETACYKENALQFAFHLGEHFNYV  699
               ELIKNPDRKQHFK ILGLFSKLKAVYSSKDPTSSVPEETACYKENALQFAFHLGEHFNYV
Sbjct  886515  ELIKNPDRKQHFKSILGLFSKLKAVYSSKDPTSSVPEETACYKENALQFAFHLGEHFNYV  886694

Query  700     PWSNYLHKLIEHVDEIVQDPRCLHSVGLLSSEGGECGNKLYRFIRRSLSRQDTAGDCDLR  759
               PWSNYLHKLIEHVDEIVQDPRCLHSVGLLSSEGGECGNKLYRFIRRSLSRQDTAGDCDLR
Sbjct  886695  PWSNYLHKLIEHVDEIVQDPRCLHSVGLLSSEGGECGNKLYRFIRRSLSRQDTAGDCDLR  886874

Query  760     DALVFHWIYTSKCIQKLATSCKAPYNCRKCGQVGHNSRTCDENGDTTTVDQGMDMNSNTS  819
               DALVFHWIYTSKCIQ LA+S KAPY+CRKCGQVG NSRTCDENGDTTTVDQGMDMNSNTS
Sbjct  886875  DALVFHWIYTSKCIQNLASSYKAPYSCRKCGQVGQNSRTCDENGDTTTVDQGMDMNSNTS  887054

Query  820     TSDDLEMDMYGDSDLSE  836
               T DDLEMD+YGDSDLSE
Sbjct  887055  TGDDLEMDIYGDSDLSE  887105
```

2.2 RAG2L

Query: **NgeRAG2L_D_3820 (previously described)**
Subject: **NMRB01000029.1 Length: 995339 Number of Matches: 1**

Score	Expect	Method	Identities	Positives	Gaps	Frame
519 bits(1336)	6e-164	Compositional matrix adjust.	253/284(89%)	259/284(91%)	6/284(2%)	-2

```
Query  232     YQGSLVGAV-LYGGVTVKNTTQGNEHSTQDGTLYFLDLENYQWKKLSELAPVAYHTSCEI  290
               +   L G + L   +T     Q  EH  +   LYFLDLENYQWKKLSELAPVAYHTSCEI
Sbjct  888649  FPAGLSGLIKLLSKLTCSRFDQIGEHMLE--XLYFLDLENYQWKKLSELAPVAYHTSCEI  888476

Query  291     RDLTLVFIGGLSQLCDNRVTSTQRMSIQKITIVELNSTIDQVTVQNLSLSFLDPALDNVF  350
               RDLTLVFIGGLSQLCDNRVTSTQRMSIQKITIVELNST+D+VTVQNLSLSFLDP LDNVF
Sbjct  888475  RDLTLVFIGGLSQLCDNRVTSTQRMSIQKITIVELNSTMDKVTVQNLSLSFLDPTLDNVF  888296

Query  351     LSGHSANICSPNQLLIFGGYQQHISDMNTKIPSRTYYLVDITSLSIQAKEAPVGFDMAGH  410
               LSGHSANICSPNQLLIFGGYQQHISDMNTKIPSRTYYLVDITSLSIQAKEAPVGFDMAGH
Sbjct  888295  LSGHSANICSPNQLLIFGGYQQHISDMNTKIPSRTYYLVDITSLSIQAKEAPVGFDMAGH  888116

Query  411     TSVRLDHCSLFFHGGANEHLFTMTTKRMELGVCEAEKCIVNDHFSPGEIVTKLQCTLCDA  470
               TSVRLDHCSLFFHGGANEHLFTMTTKRMELGVCEAEKCIVNDHFSPGEIVTKLQCTLCDA
Sbjct  888115  TSVRLDHCSLFFHGGANEHLFTMTTKRMELGVCEAEKCIVNDHFSPGEIVTKLQCTLCDA  887936

Query  471     FFHSCCDQ---GESHTSHSQEDIRFTCPKCRPKGKRQRKNKARN  511
               FFHSCCDQ   GESHTSHSQEDIRFTCPKCRPKGKRQRKNKARN
Sbjct  887935  FFHSCCDQRGKGESHTSHSQEDIRFTCPKCRPKGKRQRKNKARN  887804
```

WGS: NMRB01002322.1

1. Summary

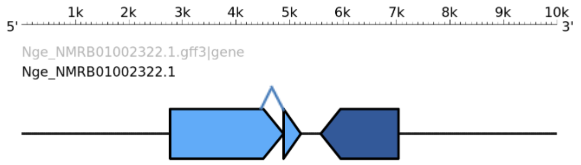

Detected configuration: RAG1L - RAG2L

Detected loci	Predicted CDS	start	end	strand	Observations
RAG1L	1	104772	106886	+	complete; no stop codon
	2	106892	107215	+	
RAG2L (!)	1	109039	107585	-	almost complete; no stop codons; N-terminal missing (?)

Selected predicted protein product

NgeRAG1L_D_ 2322

FGENESH+ gene prediction using the TSA transcript GFRY01014650.1 and C.elengans / A. californica):

```
MLSAMDDFHSEVLKHLCRICSGVLNSKACGAAPR
QKSFLSVEACASALLRIYGISVETEDNSVFPTSVCNKCRIKISREMKKTV
IEQQVLPHLFRPHQHDDLNCICHKLCSAVSVPLPPPTTESVAIEPSILTI
EHSYVHDIGAAAKPSAPSTPEVRSKKSLKELNRQYIKRTRLKDLFSNLHD
FCKEKKEDVVDVTFSLLIQELIDSDRKKEADRVMEIWSGSDKSKLSVEEC
LAMRVRNTLSKTHYAQMYAVHRDKTSLNSLQPVKQLTSIENLFFPGNCAY
KLFKNDCLIQEQDILSKVDCEPKDIMRNFANFPSDFPLPNVSGLEFDYIS
AVAKTLTELEESISARLKDCGLPIDTSVHTIIKDGGDGLGDVNIKTRKGE
FSLTDKVFRYSFCVLSCSATVNEKKYELFKEASASSVDCTRPVAICHCDE
SDRPSISITLANISKARDAMQNGVMKLRFGGGHIVPERSHTLQFLFTMVD
EKFERTSSGLQGSGSRYLCTLCEATTANCQCDLGSFTRTRTHDQTVSLYR
YRFENPEELNENDLAEKCKGVKAMPLISSDASERSLDSTHANINMGRFFK
RILVREAAEVYEWTESDRNRSRLQHVEKSLDAMLIEKLGIQPKMIMVGND
ARKLFNGANHDIVTSLIKNEERKGLVKHILSLYSKMHSVYSSTSPTVELP
ELTGNYREIAFQFSSCLGEHFP[#YITWSNYLHKVVEHVAE]KIISDPHGL
GSVGLLSSEGGESGNKLYRHIRKHLSRHDSVNDLRDALVFHWLYTSKRLQ
TLASTTHIKQHCTACGEAGHNCRTCTYDENAIG
```

# - 2 nucleotide insertion in WGS sequence (NMRB01002322.1)

However, TSA support was found for this sequence that cover the whole sequence in (See Additional File 9 nor nucleotide alignment) :

- GFRY01014650.1 (99% ident DNA)
- GFRY01014649.1 (99% ident DNA)

The protein translation of both sequences is identical and cover the predicted to be intronic region near the 2 nucleotide insertion (shown with black highlight between brackets).

As both TSA transcripts align with the WGS loci also in the UTR regions (different between the transcripts), we considered as reference sequence for this entry the translation from TSA data (See also Additional File 9 for nucleotide alignment) :

MLSAMDDFHSEVLKHLCRICSGVLNSKACGAAPRQKSFLSVEACASALLRIYGISVETEDN
SVFPTSVCNKCRIKISREMKKTVIEQQVLPHLFRPHQHDDLNCICHKLCSAVSVPLPPPTT
ESVAIEPSILTIEHSYVHDIGAAAKPSAPSTPEVRSKKSLKELNRQYIKRTRLKDLFSNLH
DFCKEKKEDVVDVTFSLLIQELIDSDRKKEADRVMEIWSGSDKSKLSVEECLAMRVRNTLS
KTHYAQMYAVHRDKTSLNSLQPVKQLTSIENLFFPGNCAYKLFKNDCLIQEQDILSKVDCE
PKDIMRNFANFPSDFPLPNVSGLEFDYISAVAKTLTELEESISARLKDCGLPIDTSVHTII
KDGGDGLGDVNIKTRKGEFSLTDKVFRYSFCVLSCSATVNEKKYELFKEASASSVDCTRPV
AICHCDESDRPSISITLANISKARDAMQNGVMKLRFGGGHIVPERSHTLQFLFTMVDEKFE
RTSSGLQGSGSRYLCTLCEATTANCQCDLGSFTRTRTHDQTVSLYRYRFENPEELNENDLA
EKCKGVKAMPLISSDASERSLDSTHANINMGRFFKRILVREAAEVYEWTESDRNRSRLQHV
EKSLDAMLIEKLGIQPKMIMVGNDARKLFNGANHDIVTSLIKNEERKGLVKHILSLYSKMH
SVYSSTSPTVELPELTGNYREIAFQFSSCLGEHFPYITWSNYLHKVVEHVAEIISDPHGLG
SVGLLSSEGGESGNKLYRHIRKHLSRHDSVNDLRDALVFHWLYTSKRLQTLASTTHIKQHC
TACGEAGHNCRTCTYDENAIG

NgeRAG1L_D_2322

IANRENFPRNARFFSILKAVQNRKKVTKKSMTDCISSLGLFSGKGHL
IWSRNDAELTPALRTAYALTDDVQGRIVDVFVTGGMHPRMPLTTTTASIFSYTFHIQESD
IKVLQVTEKKCTGAQISMRPFIHSANIVFHTRTRDVSVRRIGSVASAAAVKTSVYIWGGI
DPNIAMVPHNDLIVLENFGRGFNAKLIGTSDTVSLQTGSVPSPRYGHSLTLMNKEGKEVG
AILYGGVGCVEKGGNHATIDGKMYWLDMEKYHWQEIIIQGQQDLPPVAFHTGNEIKDCTV
VYLGGLLQVQEDNKTVIRRMSILDVSIIQCHLPQEGQNGIRAVISKIQLTFAGGLVNILL
SGHTTNVCSPSELLVYGGYQQVDNEIHSTVPSGQYFIVNIATASVTLMTAPPGFEMASHT
SIPLDSSSVFFHGGANQHLFTLTTKKMDPGRCEAETCVVDSEYTPGELVRSLQCVRCDKL
FHVCCTEEFRHSDQGKLDNIDFLCHNCKPKKTKGRKKQRK

TSA support: GFRY01034926.1 : the 5’UTR and first 21bp/7aa (blue highlight) are not covered by the scaffold. Otherwise the rest of the potential coding region, as well as 3’UTR region have 100% ident on DNA sequence (see also Additional File 9 for nucleotide alignment) :

MAAIMAMIANRENFPRNARFFSILKAVQNRKKVTKKSMTDCISSLGLFSGKGHLIWSRNDAEL
TPALRTAYALTDDVQGRIVDVFVTGGMHPRMPLTTTTASIFSYTFHIQESDIKVLQVTEKKCT
GAQISMRPFIHSANIVFHTRTRDVSVRRIGSVASAAAVKTSVYIWGGIDPNIAMVPHNDLIVL
ENFGRGFNAKLIGTSDTVSLQTGSVPSPRYGHSLTLMNKEGKEVGAILYGGVGCVEKGGNHAT
IDGKMYWLDMEKYHWQEIIIQGQQDLPPVAFHTGNEIKDCTVVYLGGLLQVQEDNKTVIRRMS
ILDVSIIQCHLPQEGQNGIRAVISKIQLTFAGGLVNILLSGHTTNVCSPSELLVYGGYQQVDN
EIHSTVPSGQYFIVNIATASVTLMTAPPGFEMASHTSIPLDSSSVFFHGGANQHLFTLTTKKM
DPGRCEAETCVVDSEYTPGELVRSLQCVRCDKLFHVCCTEEFRHSDQGKLDNIDFLCHNCKPK
KTKGRKKQRK

Further on, we considered as reference sequence for this entry the translation from TSA data.

2. RAGL Detection info

Method: TBLASTN
Database searched: Whole-genome shotgun contigs (WGS) + only in Nemertea clade (taxid:6217).

2.1. RAG1L

Query: **CviRAG1L_B_Biv1_0007 (previously described)**
Subject: **NMRB01002322.1 Length: 112446 Number of Matches: 2**

Score	Expect	Method	Identities	Positives	Gaps	Frame
290 bits(742)	1e-106	Compositional matrix adjust.	186/551(34%)	294/551(53%)	16/551(2%)	+3

```
Query  359     KLPLHSVSAKHTRHRRLKPIISQVNEFCNAQEENKSDVLFFMLKDHLKEINDPRWKQ---  415
               K  L  ++ ++ +  RLK + S +++FC  ++E+  DV F +L   L  I+  R K+
Sbjct  105246  KKSLKELNRQYIKRTRLKDLFSNLHDFCKEKKEDVVDVTFSLLIQEL--IDSDRKKEADR  105419

Query  416     VESLWLGNN-STLSPEQCLALRVDLLQSKGQYRSQYDF-LSQNNVHVFQAPSKMESCENL  473
               V  +W G++ S LS E+CLA+RV    SK  Y   Y     + +++  Q   ++ S ENL
Sbjct  105420  VMEIWSGSDKSKLSVEECLAMRVRNTLSKTHYAQMYAVHRDKTSLNSLQPVKQLTSIENL  105599

Query  474     FMPSASIFQIIDNDGNVLLQN--SENPCTEPLNVNECFLPGFVELATPNCMGVRFSYFEA  531
               F P    +++  ND  +  Q+  S+  C EP ++   F     +   PN  G+ F Y  A
Sbjct  105600  FFPGNCAYKLFKNDCLIQEQDILSKVDC-EPKDIMRNFANFPSDFPLPNVSGLEFDYISA  105776

Query  532     LSLTLQELEPEILFGLKKHGLNIEDVLFLTTVKDGCDGMGEVSVYKEKDFKMLPDKVFRF  591
               ++ TL ELE  I   LK  GL I D    T +KDG DG+G+V++   K    L DKVFR+
Sbjct  105777  VAKTLTELEESISARLKDCGLPI-DTSVHTIIKDGGDGLGDVNIKTRKGEFSLTDKVFRY  105953

Query  592     SFCIVKIQAEYDGKLFDVFTEPLPNSVRTNRPLLESISDENNQVSNVVCILPIENEREIL  651
               SFC++   A  + K +++F E   +SV   RP+     DE+++ S  + +  I   R+ +
Sbjct  105954  SFCVLSCSATVNEKKYELFKEASASSVDCTRPVAICHCDESDRPSISITLANISKARDAM  106133

Query  652     MQNRMHVKTKEGWMF----HKFSFFNSMVDEKRDRGDSGLQGSGSKYLCTLCDADKQSAK  707
                   M ++    G +     H   F  +MVDEK +R  SGLQGSGS+YLCTLC+A   + +
Sbjct  106134  QNGVMKLRFGGGHIVPERSHTLQFLFTMVDEKFERTSSGLQGSGSRYLCTLCEATTANCQ  106313

Query  708     ALLGSFSINRSVSECSNIAEILRVNPNALSENELKKISKGVKCAPLSKIEPIQKGIDATH  767
                 LGSF+  R+  +  ++      NP  L+EN+L +  KGVK  PL   +  ++ +D+TH
Sbjct  106314  CDLGSFTRTRTHDQTVSLYRYRFENPEELNENDLAEKCKGVKAMPLISSDASERSLDSTH  106493

Query  768     ADINLGQFFKKIIVREIAGVTKWELTQDVKPLVQNAEFLFDQHMKCNCGINPQLMMPGNY  827
               A+IN+G+FFK+I+VRE A V +W  +   +  +Q+ E   D  +    GI P+++M GN
Sbjct  106494  ANINMGRFFKRILVREAAEVYEWTESDRNRSRLQHVEKSLDAMLIEKLGIQPKMIMVGND  106673

Query  828     ARTLFETP-HSVLLEHISDSVRKENLSSILNIFLHLRKVYRCKDPLTECPFDVQNYKKCA  886
               AR LF    H ++   I +  RK  +  IL+++  +  VY    P  E P    NY++ A
Sbjct  106674  ARKLFNGANHDIVTSLIKNEERKGLVKHILSLYSKMHSVYSSTSPTVELPELTGNYREIA  106853

Query  887     VEMGALLLQHF  897
                +  + L +HF
Sbjct  106854  FQFSSCLGEHF  106886
```

Score	Expect	Method	Identities	Positives	Gaps	Frame
122 bits(305)	1e-106	Compositional matrix adjust.	57/103(55%)	71/103(68%)	1/103(0%)	+2

```
Query  898     DYVEWPNYLHKVIEHVQQLIEDPNGPGSVGAFSSEGNEAGNKLFRHFRKNLSRRGNTYGS  957
               +Y+ W NYLHKV+EHV ++I DP+G GSVG  SSEG E+GNKL+RH RK+LSR  ++
Sbjct  106889  EYITWSNYLHKVVEHVAEIISDPHGLGSVGLLSSEGGESGNKLYRHIRKHLSRH-DSVND  107065

Query  958     LCDVLKLHWLYSSKALFKLAEVEHKKVRCSLCFTSGHNKRTCP  1000
               L D L  HWLY+SK L  LA   H K  C+ C  +GHN RTC
Sbjct  107066  LRDALVFHWLYTSKRLQTLASTTHIKQHCTACGEAGHNCRTCT  107194
```

2.2 RAG2L

Query: PflRAG2L_B from P.flava (Morales Poole et al, 2017)
Subject: NMRB01003820.1 Length: 51922 Number of Matches: 1

Score	Expect	Method	Identities	Positives	Gaps	Frame
83.2 bits(204)	3e-15	Compositional matrix adjust.	95/443(21%)	192/443(43%)	70/443(15%)	-3

```
Query  55      VVTVYTLGGGRWKEESTWSLSNELYSLSFTLDDTDVDVESVQKFTTRGAMLSP---LHAA  111
               +V V+  GG   +   T + ++ ++S +F + ++D+ V  V +    GA +S    +H+A
Sbjct  108808  IVDVFVTGGMHPRMPLTTTTAS-IFSYTFHIQESDIKVLQVTEKKCTGAQISMRPFIHSA  108632

Query  112     -VMLNISTPD---------------KVKLLVWGGYHLG-SLFCTNEAVTMEIQRKTATCV  154
                ++ +  T D               K  + +WGG     ++   N+ + +E   +
Sbjct  108631  NIVFHTRTRDVSVRRIGSVASAAAVKTSVYIWGGIDPNIAMVPHNDLIVLENFGRGFNAK  108452

Query  155     IYKDPNDMSFHLPDEKHQSGDIPSARCGHTLTPIPGQH---AAVLFGGAEMPNRFRRVPS  211
               +    + +S        Q+G +PS R GH+LT +  +     A+L+GG     +
Sbjct  108451  LIGTSDTVSL-------QTGSVPSPRYGHSLTLMNKEGKEVGAILYGGVGCVEK-----G  108308

Query  212     FEQDTKDGHFYLLNTDSLSWKKLNV---PQLEPRAFHTATYLSSSSTICYVGG---VTYR  265
                   T DG  Y L+ +   W+++ +     L P AFHT   +    T+ Y+GG   V
Sbjct  108307  GNHATIDGKMYWLDMEKYHWQEIIIQGQQDLPPVAFHTGNEI-KDCTVVYLGGLLQVQED  108131

Query  266     DQKPYKQHQINEVTLLS--ISATNEYAVKSVLLSESLPYHVSMHGALQFN--------DQ  315
               ++   ++  I +V+++   +    +  +++V+    L +   +   L            +
Sbjct  108130  NKTVIRRMSILDVSIIQCHLPQEGQNGIRAVISKIQLTFAGGLVNILLSGHTTNVCSPSE  107951

Query  316     VIVYGGYVTPCALYQSNARPAKPSSSMFLLNTTSEVLTRLEAPDSFASAGLSMVSLDKTA  375
               ++VYGGY        +      PS   F++N  +  +T + AP  F  A  + + LD ++
Sbjct  107950  LLVYGGY----QQVDNEIHSTVPSGQYFIVNIATASVTLMTAPPGFEMASHTSIPLDSSS  107783

Query  376     IMGLGGTHKNIYFCTSKAMCPVPCDLEDECTIKDTPEISP---IAWIQCEGKCKRWLHQF  432
               +   GG +++++  T+K M P  C+ E  C +    E +P   +  +QC  +C +  H
Sbjct  107782  VFFHGGANQHLFTLTTKKMDPGRCEAE-TCVVDS--EYTPGELVRSLQCV-RCDKLFHVC  107615

Query  433     CI-KLEVIPKGK-----YVCNSC  449
               C  +     +GK     ++C++C
Sbjct  107614  CTEEFRHSDQGKLDNIDFLCHNC  107546
```

WGS: NMRB01004133.1

1. Summary

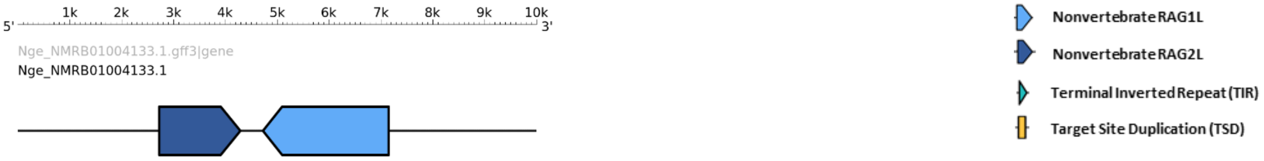

Detected configuration: RAG1L - RAG2L

Detected loci	Predicted CDS	start	end	strand	Observations
RAG1L	1	6152	3723	-	complete; no stop codon
RAG2L	1	1727	3283	+	complete; no stop codons

Selected predicted protein product

NgeRAG1L_D_ 4133

```
MVFFHFSTDMEDLHKFVLGQVCRVCGETLHNSGAAARQKTFRSV
STYVDVFKLHFGTDVENESSSVFPKTLCNKCRVRLDRLKLKLESKNLETYAFSSHQGDDC
FCLKVAGNLGIDLPPSPPNPITVNLDHSYNASEASIPAVNMPKTDRRRKKLSLKEANRQY
VKRVRLKPLIDSIETYCSESMEDDVDVLFFLLSPKLRDNQKFQMANKVSAIWKETDEFKQ
MTPEDCLAMRVTLGHSKTKYKKLYKVQKAQMGFPLLQPPKQVTAIEYKYLPGFCAFRLVD
SESDKVLAEQSLKTKAECEPTDIMGEYSHFPADFPLPNVCGFEFDYVSAVAKSLMDLEDD
ICQKLQELSLPADTKVKTFIKDGGDGLGDIKLKARKVDKNLPDKVFRYSFTVLLCTANVN
GVDHVLFRESNPNSVECTRPVLVANCDESDRPSLCVTLKSISRSRDLMKNNFLQILDANS
GSVVREHELTFWYTMIDEKFERNTSGLQGSGSMFICTLCHATTSSCQCDLGSFRRCRTNS
ETVNQYWYRLENPLSRNENDLAEECLGVKSLPLISTDATERSLDATHANINMGRIFKKIL
VRETASVYEWTENEQNKVKLQDAERRLDEHLRLRLGIQSSLIIPGNYARALFDVNNLDVV
TGLIKDEERAQHVKCLFTLLSKLQSVYSCKLPLETMPTECAEYKETAMQFGFCLGEHFPY
YTPWSNYTHRIIEHVGEIILSPGGVGSTGVLSSEGGESGNKLYRYIRQCLSRQDSTFDLR
DAIYFHWLFTCKSLQKRASVETSLQSCSLCGEPGHNRRRHILCHET
```

Predicted using FGENESH+ (*C.elegans* dataset) and NgeRAG1L_D_2322 as homolog.

NgeRAG1L_D_ 4133

```
MAAPVPVDFLHNSRFFSLAKAIKKRRKLTKKAIAADAAEKLFFSGKGHLIFSENVSEDDQ
GGQEEEGRLHSDDDEHERRVLVNVLGGLHDKEPMTTLSGTIFQYVFTVGEDDIAIESISE
KKCAGAQIHLRPFVYAASVVCKTFLRDTSSTRRQCRQQQVRSSKTEVYIWGGIDPNRMVA
HNDLTVLETYGLSYFATLVSSGNESMTKKLQSGSIPSPRYGHTMNIICNGGAILYGGMEI
DSQQIDIHTTQDGSLYVLDLHEFRWTKVVVLGQDELPHVVFHTTTTLKDFTLAMVGGISQ
TLQGDHVQYTRKSLLDVTCIVVEKTGVDITASIYHPQLQFNGPMDEMVYLSGHTAHLCSP
SCLFIFGGYQGNAADHNNPPSRQYFSVHLDTSLVSRFQAPEGFEMANHTCVSLDKSSLFF
HGGAHQHSFVFTSKTMEPNKCEAEECVVSDHYIPGETVIWVQCDKSSCEKWFHFCCTGLT
GEPDREKLETTEFYCNKCKPKRQGGGDREAKEEIKTEIR
```

Predicted using FGENESH (*C.elegans* dataset)

2. RAGL Detection info

Method: TBLASTN
Database searched: Whole-genome shotgun contigs (WGS) + only in Nemertea clade (taxid:6217).

2.1. RAG1L

Query: **NgeRAG1L_D_3820 (previously described)**
Subject: **NMRB01004133.1 Length: 44382 Number of Matches: 1**

Score	Expect	Method	Identities	Positives	Gaps	Frame
787 bits(2032)	0.0	Compositional matrix adjust.	397/806(49%)	540/806(66%)	22/806(2%)	-2

```
Query  1      MDQYHKQVLQRICRLCSGDLKK---VKRQNNFFDVSKAAVVLQLHYSISVGDESEAVFPR  57
              M+  HK VL ++CR+C   L       RQ  F  VS    V +LH+   V +ES +VFP+
Sbjct  6125   MEDLHKFVLGQVCRVCGETLHNSGAAARQKTFRSVSTYVDVFKLHFGTDVENESSSVFPK  5946

Query  58     CICNSCRNKVDRLKQTAVSKNLTAPKFLPHQECDCFCVGRGSLLKLPLPSEPVQIEPAEV  117
               +CN CR ++DRLK    SKNL    F  HQ  DCFC+     L + LP  P    P  +
Sbjct  5945   TLCNKCRVRLDRLKLKLESKNLETYAFSSHQGDDCFCLKVAGNLGIDLPPSP----PNPI  5778

Query  118    VISSDHSYSGSGPNECGTGTSNGTTNDVMSPQPRKKCRLQQVSRHHIKQRRLKDLISSVG  177
               ++ DHSY+ S   E      N    D    + RKK  L++ +R ++K+ RLK LI S+
Sbjct  5777   TVNLDHSYNAS---EASIPAVNMPKTD----RRRKKLSLKEANRQYVKRVRLKPLIDSIE  5619

Query  178    SFCDDRMEDPVDAMYAVLIQKLLDDGRKKEANKILSVWQNKGEELEMSAQDCLAVRVRTL  237
              ++C + MED VD ++ +L  KL D+ + + ANK+ ++W+   E  +M+ +DCLA+RV
Sbjct  5618   TYCSESMEDDVDVLFFLLSPKLRDNQKFQMANKVSAIWKETDEFKQMTPEDCLAMRVTLG  5439

Query  238    QSKTRYRQQYSLQRDNTNSASLKPPYQLDAAEATFMPGHCAYKLM--KDSVVIAEQSIST  295
               SKT+Y++ Y +Q+       L+PP Q+ A E  ++PG CA++L+  +   V+AEQS+ T
Sbjct  5438   HSKTKYKKLYKVQKAQMGFPLLQPPKQVTAIEYKYLPGFCAFRLVDSESDKVLAEQSLKT  5259

Query  296    NDQYEPKDIMDQFKNFPTDFPVPNLAGVEFSFVSAVAKTLWELESLIEMRLNELGLPLST  355
                + EP DIM ++ +FP DFP+PN+ G EF +VSAVAK+L +LE  I  +L EL LP  T
Sbjct  5258   KAECEPTDIMGEYSHFPADFPLPNVCGFEFDYVSAVAKSLMDLEDDICQKLQELSLPADT  5079

Query  356    QIETIFKDGGDGLGDVCLKTQKADFARTDKIFRYSFCCVSCSAVVGDKKHLLFRETFPNN  415
              +++T  KDGGDGLGD+ LK +K D    DK+FRYSF  + C+A V    H+LFRE+ PN+
Sbjct  5078   KVKTFIKDGGDGLGDIKLKARKVDKNLPDKVFRYSFTVLLCTANVNGVDHVLFRESNPNS  4899

Query  416    ---TRPVILCHCDESDRPSISTLLANVSREREMMQDGTLVLVDPDTGDDVRQHHLHFLYT  472
                 TRPV++ +CDESDRPS+   L ++SR R++M++  L ++D ++G  VR+H L F YT
Sbjct  4898   VECTRPVLVANCDESDRPSLCVTLKSISRSRDLMKNNFLQILDANSGSVVREHELTFWYT  4719

Query  473    MVDEKFERTSSGLQGSGSRFICTLCYAATNTCQADVGSFSRCRTYDETVSLYKYRLENPD  532
              M+DEKFER +SGLQGSGS FICTLC+A T++CQ D+GSF RCRT  ETV+ Y YRLENP
Sbjct  4718   MIDEKFERNTSGLQGSGSMFICTLCHATTSSCQCDLGSFRRCRTNSETVNQYWYRLENPL  4539

Query  533    KLNENDLAERCKGVNSIPLIKSDATDRSVDSTHANINIGRVFKRILVREIAEIYEWAEND  592
                NENDLAE C GV S+PLI +DAT+RS+D+THANIN+GR+FK+ILVRE A +YEW EN+
Sbjct  4538   SRNENDLAEECLGVKSLPLISTDATERSLDATHANINMGRIFKKILVRETASVYEWTENE  4359

Query  593    RNKKGLQHAEKTNDQHLMKTLGLQSKMIMPGNYARILFLDKNAETVSELIKNPDRKQHFK  652
              +NK  LQ AE+  D+HL   LG+QS +I+PGNYAR LF   N + V+ LIK+ +R QH K
Sbjct  4358   QNKVKLQDAERRLDEHLRLRLGIQSSLIIPGNYARALFDVNNLDVVTGLIKDEERAQHVK  4179

Query  653    CILGLFSKLKAVYSSKDPTSSVPEETACYKENALQFAFHLGEHF-NYVPWSNYLHKLIEH  711
              C+  L SKL++VYS K P  ++P E A YKE A+QF F LGEHF  Y PWSNY H++IEH
Sbjct  4178   CLFTLLSKLQSVYSCKLPLETMPTECAEYKETAMQFGFCLGEHFPYYTPWSNYTHRIIEH  3999

Query  712    VDEIVQDPRCLHSVGLLSSEGGECGNKLYRFIRRSLSRQDTAGDCDLRDALVFHWIYTSK  771
              V EI+  P  + S G+LSSEGGE GNKLYR+IR+ LSRQD+    DLRDA+ FHW++T K
Sbjct  3998   VGEIILSPGGVGSTGVLSSEGGESGNKLYRYIRQCLSRQDST--FDLRDAIYFHWLFTCK  3825
```

```
Query  772    CIQKLATSCKAPYNCRKCGQVGHNSR  797
               +QK A+   +  +C  CG+ GHN R
Sbjct  3824   SLQKRASVETSLQSCSLCGEPGHNRR  3747
```

2.2 RAG2L

Query: NgeRAG2L_D_3820 (previously described)
Subject: NMRB01004133.1 Length: 44382 Number of Matches: 1

Score	Expect	Method	Identities	Positives	Gaps	Frame
377 bits(968)	9e-115	Compositional matrix adjust.	212/516(41%)	313/516(60%)	30/516(5%)	+2

```
Query  17     EFFNNARFYSVLKAVQNRRKVTKKAIVEEASKGALFSGSGHL--SWLIFYNDP--RQHTA  72
              +F +N+RF+S+ KA++ RRK+TKKAI  +A++   FSG GHL  S  +  +D   ++
Sbjct  1748   DFLHNSRFFSLAKAIKKRRKLTKKAIAADAAEKLFFSGKGHLIFSENVSEDDQGGQEEEG  1927

Query  73     AVTNNGSGDEKTFSVAVLGGMHAMKPLTNLSGTVYSYAFKVNDDDIKILSVAECKCSGAQ  132
               + ++    E+   V VLGG+H  +P+T LSGT++ Y F V +DDI I S++E KC+GAQ
Sbjct  1928   RLHSDDDEHERRVLVNVLGGLHDKEPMTTLSGTIFQYVFTVGEDDIAIESISEKKCAGAQ  2107

Query  133    IALRPFVHSACIVETKATRESSRSRRTGPGGARSAMISICSIWGGLDPNRMLPHNDLTIL  192
              I LRPFV++A +V     R++S +RR         +  +   IWGG+DPNRM+ HNDLT+L
Sbjct  2108   IHLRPFVYAASVVCKTFLRDTSSTRRQCRQQQVRSSKTEVYIWGGIDPNRMVAHNDLTVL  2287

Query  193    ENFGRDQFTARLVC---QEGTKNVQTGTVPEARYGHTLSGLHYQGSLVGAVLYGGVTVKN  249
              E +G   F A LV    +  TK +Q+G++P  RYGHT++ +       GA+LYGG+ + +
Sbjct  2288   ETYGLSYF-ATLVSSGNESMTKKLQSGSIPSPRYGHTMNII----CNGGAILYGGMEI-D  2449

Query  250    TTQGNEHSTQDGTLYFLDLENYQWKKL-----SELAPVAYHTSCEIRDLTLVFIGGLSQL  304
              + Q + H+TQDG+LY LDL  ++W K+      EL  V +HT+  ++D TL  +GG+SQ
Sbjct  2450   SQQIDIHTTQDGSLYVLDLHEFRWTKVVVLGQDELPHVVFHTTTTLKDFTLAMVGGISQT  2629

Query  305    CDNRVTSTQRMSIQKITIVELNSTIDQVT--VQNLSLSFLDPALDNVFLSGHSANICSPN  362
                        R S+  +T + +  T   +T  + +  L F  P  + V+LSGH+A++CSP+
Sbjct  2630   LQGDHVQYTRKSLLDVTCIVVEKTGVDITASIYHPQLQFNGPMDEMVYLSGHTAHLCSPS  2809

Query  363    QLLIFGGYQQHISDMNTKIPSRTYYLVDITSLSIQAKEAPVGFDMAGHTSVRLDHCSLFF  422
               L IFGGYQ + +D N   PSR Y+ V + +  +   +AP GF+MA HT V LD  SLFF
Sbjct  2810   CLFIFGGYQGNAADHNNP-PSRQYFSVHLDTSLVSRFQAPEGFEMANHTCVSLDKSSLFF  2986

Query  423    HGGANEHLFTMTTKRMELGVCEAEKCIVNDHFSPGEIVTKLQC--TLCDAFFHSCCD--Q  478
              HGGA++H F  T+K ME   CEAE+C+V+DH+ PGE V  +QC  + C+ +FH CC
Sbjct  2987   HGGAHQHSFVFTSKTMEPNKCEAEECVVSDHYIPGETVIWVQCDKSSCEKWFHFCCTGLT  3166

Query  479    GESHTSHSQEDIRFTCPKCRPK----GKRQRKNKAR  510
              GE       E   F C KC+PK    G R+ K + +
Sbjct  3167   GEP-DREKLETTEFYCNKCKPKRQGGGDREAKEEIK  3271
```

WGS: NMRB01002297.1

1. Summary

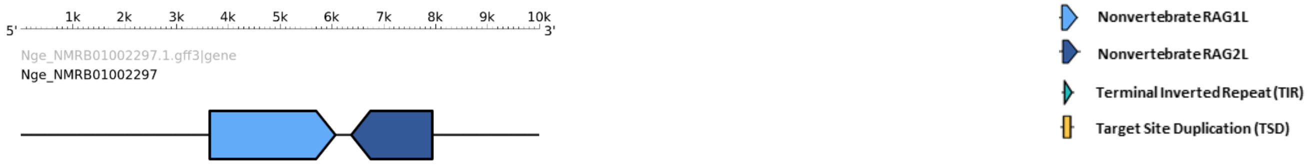

Detected configuration: RAG1L - RAG2L

Detected loci	Predicted CDS	start	end	strand	Observations
RAG1L	1	61617	64067	+	complete; no stop codon
RAG2L	1	65939	64332	-	complete; no stop codons

Selected predicted protein product

NgeRAG1L_D_ 2297

```
MASSAAAHQEVLKCLCRLCAEDLQSRAVVRQKSFRSVEKHEETLLLHFGIDVNKEDSEIF
PKLLCNKCRSKLDRLNSKSDPQHLESPVFSSHQEIDCFCLELINRMGIDVPSEPEPLEIG
IADVTAVDHPYTGGDHSATSTRGSSAANPTVRRAKRASLREANRRYVKGERLKDLITSVN
SFCEERDEDTLDALFFMLSQKLIDQGRKEEADKILSLWNCDSQDSSAEKLTMTPEECLAM
RVRTLQSKGHYRSVYKIQTEKTSCKTLCPPGKLNAIEHTFLPGHCEYELVTEDGETLVAQ
QAMLSEEDSEPKTILGAFNSFPSDFPLPNVHGVQFSYISCVAKALQELENVISEKLTSLG
LPISTKVETLIKDGGDGLGDVDVKKRKGDHALTDKVFRYSFCILTCTALIDGKRVVLFRE
EHPNSVDCTRPVVVANCDESDQPSIALTVKDVCRSREIMKNNVMKLLSSGTVVREHHLKF
MYTMVDEKFERTSCGLQGSGSRFICTLCCATSANCQADCGSFSRTRTNDETVRLYKYRLE
NPDNLSENDLAERTKGVKSMPMIFCDATERCFDATHANINMGRVLKKTLVREIAGVHEWT
ENDSNKIKLQHAEKTLDEHLREKLGLQPKLMLPGNYARALFTPENLDILCELIVDEERRH
HVATLLTYYSRLHSVYSCKTPLESMPEEAGNYGEIALQMAFTLGEHFPYFRWSNYLHKIV
EHGQEIIDDTSNDLHSIGAFSSEGGESGNKLYRFIRKSLSRSDSYNDMRDALLFHWLYTS
KQIQQYAQCSKIIQCCSLCGESNHNKRTCTYEPSTIG
```

Predicted using FGENESH (*C.elegans* dataset)

Green: TSA support GFRY01002319.1 (98% DNA, 98 % protein.
See Additional File 9 for alignments.

NgeRAG2L_D_ 2297

```
MAATVVN
NPEMGDNFRFFSLIRAIKKRRKLTKKAVSDDISSLCLFSGKGHLMWTEYGDRSASDPETG
HHDDDTAAAVHHHVHDVDRPNDYVVDDGSESKKVSVCVLGWMHDRLPLVSQAGAIYQYDF
KVELDDIQVEKISGVKCGGAQIGLRPFLHSAAVVRSVDTRVRTAPARRRRHARTAKKTEV
FVWGGMDPTRMEPHSDLMILESYGGGKYNATLVSSCGELPTKTQQTGTVPFPRYGHTLNA
VKSSHNGSIIGALLYGGIEVNVNEHHSEIHSTRDNNLYFLNFEDLKWTEVNVQGAGEGLC
PVAYHTGTEIRDSCIVFIGGLVQRADDDGNVDYNGKSVLDILMAQCHLREGVLTVTLTKP
KLTFSGPMNNDVHLSGHTANLCNQSSILVFGGYHNQDKEPQNEYFIISLDTSVVLRKVAP
VGFEMASHQTTYLDSDSIFFHGGANQHTFVLTEKQMEPSQCQARECVVNDHFIPGETVTW
LQCDGECEKWFHFCCSGLSGEPDTEELEQLEFICQSCQRNKPKKKRGKKK
```

Predicted using FGENESH+ (*C.elegans* dataset an NgeRAG2L_2322 as reference)

```
               1                                                                                         90
NgeRAG1L_D_2297   MASSAAAHQEVLKCLCRLCAEDLQSRAVVRQKSFRSVEKHEETLLLHFGIDVNKEDSEIFPKLLCNKCRSKLDRLNSKSDPQHLESPVFS
```

```
TSA_GFRY01002319
 Consensus   ...     .................................................................................

                   91                                                                               180
NgeRAG1L_D_2297    SHQEIDCFCLELINRMGIDVPSEPEPLEIGIADVTAVDHPYTGGDHSATSTRGSSAANPTVRRAKRASLREANRRYVKGERLKDLITSVN
TSA_GFRY01002319
 Consensus   ...   ..........................................................................................

                   181                                                                              270
NgeRAG1L_D_2297    SFCEERDEDTLDALFFMLSQKLIDQGRKEEADKILSLWNCDSQDSSAEKLTMTPEECLAMRVRTLQSKGHYRSVYKIQTEKTSCKTLCPP
TSA_GFRY01002319
 Consensus   ...   ..........................................................................................

                   271                                                                              360
NgeRAG1L_D_2297    GKLNAIEHTFLPGHCEYELVTEDGETLVAQQAMLSEEDSEPKTILGAFNSFPSDFPLPNVHGVQFSYISCVAKALQELENVISEKLTSLG
TSA_GFRY01002319                                     [redacted]VRKTPSILGAFNSFPSDFPLPNVHGVQFSYISCVAKALQELENVISEKLTSLG
 Consensus   ...   ....................................edrepksILGAFNSFPSDFPLPNVHGVQFSYISCVAKALQELENVISEKLTSLG

                   361                                                                              450
NgeRAG1L_D_2297    LPISTKVETLIKDGGDGLGDVDVKKRKGDHALTDKVFRYSFCILTCTALIDGKRVVLFREEHPNSVDCTRPVVVANCDESDQPSIALTVK
TSA_GFRY01002319   LPISTKVETLIKDGGDGLGDVDVKKRKGDHALTDKVFRYSFCILTCTALIDGKRVVLFREEHPNSVDCTRPVVVANCDESNQPSIALTVK
 Consensus   ...   LPISTKVETLIKDGGDGLGDVDVKKRKGDHALTDKVFRYSFCILTCTALIDGKRVVLFREEHPNSVDCTRPVVVANCDESdQPSIALTVK

                   451                                                                              540
NgeRAG1L_D_2297    DVCRSREIMKNNVMKLLSSGTVVREHHLKFMYTMVDEKFERTSCGLQGSGSRFICTLCCATSANCQADCGSFSRTRTNDETVRLYKYRLE
TSA_GFRY01002319   DASRSREIMKNNVMKLLSSGTVVREHHLKFMYTMVGEKFERTSCGLQGSGSRFICTLCRATSANCQADCGSYSRTRTNDETVRLYKYRLE
 Consensus   ...   DacRSREIMKNNVMKLLSSGTVVREHHLKFMYTMVdEKFERTSCGLQGSGSRFICTLCcATSANCQADCGSfSRTRTNDETVRLYKYRLE

                   541                                                                              630
NgeRAG1L_D_2297    NPDNLSENDLAERTKGVKSMPMIFCDATERCFDATHANINMGRVLKKTLVREIAGVHEWTENDSNKIKLQHAEKTLDEHLREKLGLQPKL
TSA_GFRY01002319   NPDNLSENDLAERTKGVKSMPMIFCDATERCFDATHANINMGRVLKKILVREIAGVHEWTENDSNKIKLQHAEKTLDEHLREKLGLQPKL
 Consensus   ...   NPDNLSENDLAERTKGVKSMPMIFCDATERCFDATHANINMGRVLKKiLVREIAGVHEWTENDSNKIKLQHAEKTLDEHLREKLGLQPKL

                   631                                                                              720
NgeRAG1L_D_2297    MLPGNYARALFTPENLDILCELIVDEERRHHVATLLTYYSRLHSVYSCKTPLESMPEEAGNYGEIALQMAFTLGEHFPYFRWSNYLHKIV
TSA_GFRY01002319   MLPGNYARALFTPENLDILCELIVDEERRHHVATLLKYYSRLHSVYSCKTPLESMPEEAGNYGEIALQMAFTLGEHFPYFRWSNYLHKIV
 Consensus   ...   MLPGNYARALFTPENLDILCELIVDEERRHHVATLLkYYSRLHSVYSCKTPLESMPEEAGNYGEIALQMAFTLGEHFPYFRWSNYLHKIV

                   721                                                                              810
NgeRAG1L_D_2297    EHGQEIIDDTSNDLHSIGAFSSEGGESGNKLYRFIRKSLSRSDSYNDMRDALLFHWLYTSKQIQQYAQCSKIIQCCSLCGESNHNKRTCT
TSA_GFRY01002319   EHGQEIIDDTSNDLHSIGAFSSEGGESGNKLYRFIRKSLSRSDSYNDMRDALLFHWLYTSKQIQQYAQCSKIIQCCSLCGESNHNKRTCT
 Consensus   ...   EHGQEIIDDTSNDLHSIGAFSSEGGESGNKLYRFIRKSLSRSDSYNDMRDALLFHWLYTSKQIQQYAQCSKIIQCCSLCGESNHNKRTCT

                   811
NgeRAG1L_D_2297    YEPSTIG
TSA_GFRY01002319   YEPSTIG
 Consensus   ...   YEPSTIG
```

2. RAGL Detection info

Method: TBLASTN
Database searched: Whole-genome shotgun contigs (WGS) + only in Nemertea clade (taxid:6217).

2.1. RAG1L

Query: **NgeRAG1L_D_3820 (previously described)**
Subject: **NMRB01002297.1 Length: 113872 Number of Matches: 1**

Score	Expect	Method	Identities	Positives	Gaps	Frame
826 bits(2134)	0.0	Compositional matrix adjust.	425/812(52%)	571/812(70%)	27/812(3%)	+3

```
Query  5      HKQVLQRICRLCSGDL--KKVKRQNNFFDVSKAAVVLQLHYSISVGDESEAVFPRCICNS  62
              H++VL+ +CRLC+ DL  + V RQ +F  V K    L LH+ I V  E  +FP+ +CN
Sbjct  61638  HQEVLKCLCRLCAEDLQSRAVVRQKSFRSVEKHEETLLLHFGIDVNKEDSEIFPKLLCNK  61817

Query  63     CRNKVDRLKQTAVSKNLTAPKFLPHQECDCFCVGRGSLLKLPLPSEPVQIEPAEV----V  118
```

```
                CR+K+DRL   +  ++L +P F  HQE DCFC+   + + + +PSEP   EP E+    V
Sbjct  61818  CRSKLDRLNSKSDPQHLESPVFSSHQEIDCFCLELINRMGIDVPSEP---EPLEIGIADV  61988

Query  119    ISSDHSYSGSGPNECGTGTSNGTTNDVMSPQPRKKCRLQQVSRHHIKQRRLKDLISSVGS  178
               + DH Y+G   +   T  S+     V   +  K+  L++ +R ++K  RLKDLI+SV S
Sbjct  61989  TAVDHPYTGGDHSATSTRGSSAANPTV---RRAKRASLREANRRYVKGERLKDLITSVNS  62159

Query  179    FCDDRMEDPVDAMYAVLIQKLLDDGRKKEANKILSVWQ-----NKGEELEMSAQDCLAVR  233
              FC++R ED +DA++ +L QKL+D GRK+EA+KILS+W       +  E+L M+ ++CLA+R
Sbjct  62160  FCEERDEDTLDALFFMLSQKLIDQGRKEEADKILSLWNCDSQDSSAEKLTMTPEECLAMR  62339

Query  234    VRTLQSKTRYRQQYSLQRDNTNSASLKPPYQLDAAEATFMPGHCAYKLMKD--SVVIAEQ  291
              VRTLQSK  YR  Y +Q + T+  +L PP +L+A E TF+PGHC Y+L+ +    ++A+Q
Sbjct  62340  VRTLQSKGHYRSVYKIQTEKTSCKTLCPPGKLNAIEHTFLPGHCEYELVTEDGETLVAQQ  62519

Query  292    SISTNDQYEPKDIMDQFKNFPTDFPVPNLAGVEFSFVSAVAKTLWELESLIEMRLNELGL  351
              ++ + +  EPK I+  F +FP+DFP+PN+ GV+FS++S VAK L ELE++I  +L  LGL
Sbjct  62520  AMLSEEDSEPKTILGAFNSFPSDFPLPNVHGVQFSYISCVAKALQELENVISEKLTSLGL  62699

Query  352    PLSTQIETIFKDGGDGLGDVCLKTQKADFARTDKIFRYSFCCVSCSAVVGDKKHLLFRET  411
              P+ST++ET+ KDGGDGLGDV +K +K D A TDK+FRYSFC ++C+A++  K+ +LFRE
Sbjct  62700  PISTKVETLIKDGGDGLGDVDVKKRKGDHALTDKVFRYSFCILTCTALIDGKRVVLFREE  62879

Query  412    FPNN---TRPVILCHCDESDRPSISTLLANVSREREMMQDGTLVLVDPDTGDDVRQHHLH  468
               PN+   TRPV++ +CDESD+PSI+  + +V R RE+M++  + L+   +G  VR+HHL
Sbjct  62880  HPNSVDCTRPVVVANCDESDQPSIALTVKDVCRSREIMKNNVMKLLS--SGTVVREHHLK  63053

Query  469    FLYTMVDEKFERTSSGLQGSGSRFICTLCYAATNTCQADVGSFSRCRTYDETVSLYKYRL  528
              F+YTMVDEKFERTS GLQGSGSRFICTLC A +  CQAD GSFSR RT DETV LYKYRL
Sbjct  63054  FMYTMVDEKFERTSCGLQGSGSRFICTLCCATSANCQADCGSFSRTRTNDETVRLYKYRL  63233

Query  529    ENPDKLNENDLAERCKGVNSIPLIKSDATDRSVDSTHANINIGRVFKRILVREIAEIYEW  588
              ENPD L+ENDLAER KGV S+P+I  DAT+R  D+THANIN+GRV K+ LVREIA ++EW
Sbjct  63234  ENPDNLSENDLAERTKGVKSMPMIFCDATERCFDATHANINMGRVLKKTLVREIAGVHEW  63413

Query  589    AENDRNKKGLQHAEKTNDQHLMKTLGLQSKMIMPGNYARILFLDKNAETVSELIKNPDRK  648
               END NK  LQHAEKT D+HL + LGLQ K+++PGNYAR LF  +N + + ELI + +R+
Sbjct  63414  TENDSNKIKLQHAEKTLDEHLREKLGLQPKLMLPGNYARALFTPENLDILCELIVDEERR  63593

Query  649    QHFKCILGLFSKLKAVYSSKDPTSSVPEETACYKENALQFAFHLGEHFNYVPWSNYLHKL  708
               H   +L  +S+L +VYS K P  S+PEE   Y E ALQ AF LGEHF Y  WSNYLHK+
Sbjct  63594  HHVATLLTYYSRLHSVYSCKTPLESMPEEAGNYGEIALQMAFTLGEHFPYFRWSNYLHKI  63773

Query  709    IEHVDEIVQDPRC-LHSVGLLSSEGGECGNKLYRFIRRSLSRQDTAGDCDLRDALVFHWI  767
              +EH  EI+ D    LHS+G  SSEGGE GNKLYRFIR+SLSR D+    D+RDAL+FHW+
Sbjct  63774  VEHGQEIIDDTSNDLHSIGAFSSEGGESGNKLYRFIRKSLSRSDSYN--DMRDALLFHWL  63947

Query  768    YTSKCIQKLATSCKAPYNCRKCGQVGHNSRTC  799
              YTSK IQ+ A   K    C  CG+  HN RTC
Sbjct  63948  YTSKQIQQYAQCSKIIQCCSLCGESNHNKRTC  64043
```

2.2 RAG2L

Query: **NgeRAG2L_D_3820 (previously described)**
Subject: **NMRB01002297.1 Length: 113872 Number of Matches: 1**

Score	Expect	Method	Identities	Positives	Gaps	Frame
367 bits(941)	4e-111	Compositional matrix adjust.	215/524(41%)	301/524(57%)	50/524(9%)	-3

```
Query  17     EFFNNARFYSVLKAVQNRRKVTKKAIVEEASKGALFSGSGHLSWLIF----YNDPR----  68
              E  +N RF+S+++A++ RRK+TKKA+ ++ S   LFSG GHL W  +     +DP
Sbjct  65912  EMGDNFRFFSLIRAIKKRRKLTKKAVSDDISSLCLFSGKGHLMWTEYGDRSASDPETGHH  65733

Query  69     -QHTAA---------------VTNNGSGDEKTFSVAVLGGMHAMKPLTNLSGTVYSYAFK  112
                 TAA               V ++GS + K  SV VLG MH   PL + +G +Y Y FK
Sbjct  65732  DDDTAAAVHHHVHDVDRPNDYVVDDGS-ESKKVSVCVLGWMHDRLPLVSQAGAIYQYDFK  65556

Query  113    VNDDDIKILSVAECKCSGAQIALRPFVHSACIVETKATRESSRSRRTGPGGARSAMISIC  172
              V  DDI++  ++  KC GAQI LRPF+HSA +V +  TR  +   R       +    +
Sbjct  65555  VELDDIQVEKISGVKCGGAQIGLRPFLHSAAVVRSVDTRVRTAPARRRRHARTAKKTEVF  65376

Query  173    SIWGGLDPNRMLPHNDLTILENFGRDQFTARLVCQEG---TKNVQTGTVPEARYGHTLSG  229
               +WGG+DP RM PH+DL ILE++G  ++ A LV   G   TK  QTGTVP  RYGHTL+
Sbjct  65375  -VWGGMDPTRMEPHSDLMILESYGGGKYNATLVSSCGELPTKTQQTGTVPFPRYGHTLNA  65199

Query  230    LH--YQGSLVGAVLYGGVTVKNTTQGNE-HSTQDGTLYFLDLENYQWKKLS------ELA  280
              +   + GS++GA+LYGG+ V      +E HST+D  LYFL+ E+ +W +++       L
Sbjct  65198  VKSSHNGSIIGALLYGGIEVNVNEHHSEIHSTRDNNLYFLNFEDLKWTEVNVQGAGEGLC  65019

Query  281    PVAYHTSCEIRDLTLVFIGGLSQLCDNRV-TSTQRMSIQKITIVE--LNSTIDQVTVQNL  337
              PVAYHT  EIRD  +VFIGGL Q  D+         S+  I + +  L   +  VT+
Sbjct  65018  PVAYHTGTEIRDSCIVFIGGLVQRADDDGNVDYNGKSVLDILMAQCHLREGVLTVTLTKP  64839

Query  338    SLSFLDPALDNVFLSGHSANICSPNQLLIFGGYQQHISDMNTKIPSRTYYLVDITSLSIQ  397
               L+F  P  ++V LSGH+AN+C+ + +L+FGGY         K P   Y+++ + +  +
Sbjct  64838  KLTFSGPMNNDVHLSGHTANLCNQSSILVFGGYHN-----QDKEPQNEYFIISLDTSVVL  64674

Query  398    AKEAPVGFDMAGHTSVRLDHCSLFFHGGANEHLFTMTTKRMELGVCEAEKCIVNDHFSPG  457
               K APVGF+MA H +  LD  S+FFHGGAN+H F +T K+ME   C+A +C+VNDHF PG
Sbjct  64673  RKVAPVGFEMASHQTTYLDSDSIFFHGGANQHTFVLTEKQMEPSQCQARECVVNDHFIPG  64494

Query  458    EIVTKLQCT-LCDAFFHSCCD--QGESHTSHSQEDIRFTCPKCR  498
              E VT LQC   C+ +FH CC    GE  T    E + F C  C+
Sbjct  64493  ETVTWLQCDGECEKWFHFCCSGLSGEPDTE-ELEQLEFICQSCQ  64365
```

WGS: NMRB01002705.1

3. Summary

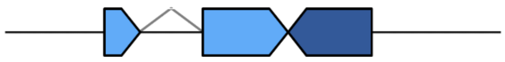

Detected configuration: RAG1L - RAG2L

Predicted gene	Predicted CDS	start	end	strand	Observations
RAG1L	1	27012	27730	+	complete; no stop codon
	2	29003	30704		
RAG2L	1	32386	30703	-	complete; no stop codons

Selected predicted protein product

NgeRAG1L_D_ 2705

```
MDQYHRDVLDHVCRICGDELQARAVKRQKKFYEVKGAAETIEVHYGISVRDEDEHQFPGY
ACNRCRNKLDRLKNVKESKNLTVFQFSPHADEDCFCIGVGSKLGLAVPQEPVPVDPVTIT
VDHAYQSSGSPSCSKPVIEPRKKIPLNQANRQYVKRQRLKGMISGVDQFCEEHKEDTVDA
LYFVLIQKLSDTGRKQEADRVRSMWNGRDEVELSVPECLGMRVRSLMSKAHYKQTYGIQR
DKTSAKSLQPRNQLTVLEPSFLPGHCKYRLLKDSVLLAEQDLLSAQDSQPLDIMRDYHSF
PADFPLPNVMGVELDYISAVAKTLLEIDNDVSKRLDALGLPQSTKLCTVIKDGGDGLGDV
NVKSRKADFALTDKVFRYSFCVLQCAASDAHGEKHTIFKEEFPNSVDCTRPVVVCNCDES
DRPSVAIILSHVSKARKQMQGATLQLLDPSSGSVIREHDLSFYYTMVDEKFERISSGLQG
SGSRFLCTLCHATTASCQADAGSHHRVRTHEETVSLYKYRLENPQNLGEDELAIQSKGVK
SFPLISSNANERSLDATHANINIGRVLKKVVVREIAEIHEWAENDNNKTSLQNAEKEFDA
KIMKEIGLQPKMMMAGNYARAVFAVENLGVVTSLIKNDERREHVSTLFNIFNRLHRVYSS
NTPLTSVPDETAAYRENALQFAFHLGENFPYVPWSNYIHRVVEHVDEILHDPSGVQSVGL
LSSEGGESGNKLYRFIRKSLSRQDSAFDLRDALVFHWLYTSKVLQKHAATEKVSKTCTIC
GMADHNRRTCAYKQDAAPEDDMMEES
```

Green: TSA support GFRY01095553.1, GFRY01095554.1, GFRY01095552.1 (99% identity nucleotide & protein sequence)

Predicted using FGENESH+ (*C.elegans* dataset an NgeRAG2L_3820 as reference) and corrected using the TSA entry at the merging area.

NgeRAG2L_D_ 2297

```
MMAEVNRTYDFWQDSRFYSILKAIKKRRKTTKKAVSGDIGNLALFSGPGHIIWTVE
SVATRETLTPALSHFFDERDVIDGKIITVSVLGGMHPRLPLTTLSGTIFNYSFHVQENDV
KVLNASEKKCSGAQIVLRPLLHAAACSDTTRTRDEAPRRRARGVARTGATKTLIFIWGGM
DLNRMEPHNDLVVLETFGRDQNYSAKLVGSDRPEGTCTTVKQMGDVPEGRYAHTLTGIHE
NGSLLGALLFGGVEKRPEGHGTFDKDLYFIDLLQYKWFQMVSEQLNPVAYHTGTELRDKV
VAYVGGLRQMPGANGATIERQSILEVKLAVLEFIDGQVSVTVSQLIMPNFLGKDTLLSGH
TSTALDESRVLVFGGYQQQDNQHPAKEPGGQYFVLDLATNSIQMKDIPSGFAMACHTAVM
MDPALIFFHGGANQHLFTLTTKRLEPGRCQSDKCIVTDNFSPGEIIESLQCETCDKWYHS
CCAGSKNGRSETESFVCVDCRPKQTKGKKKQRRRSEIQFEYFMYIV
```

Predicted using FGENESH+ (*C.elegans* dataset an NgeRAG2L_2322 as reference)

1 90

```
NgeRAG2L_D_ 2297  MDQYHRDVLDHVCRICGDELQARAVKRQKKFYEVKGAAETIEVHYGISVRDEDEHQFPGYACNRCRNKLDRLKNVKESKNLTVFQFSPHA
GFRY01095553
 Consensus        ..........................................................................................

                  91                                                                                     180
NgeRAG2L_D_ 2297  DEDCFCIGVGSKLGLAVPQEPVPVDPVTITVDHAYQSSGSPSCSKPVIEPRKKIPLNQANRQYVKRQRLKGMISGVDQFCEEHKEDTVDA
GFRY01095553                                                                             MISGVDQFCEEHKEDTVDA
 Consensus        .......................................................................MISGVDQFCEEHKEDTVDA

                  181                                                                                    270
NgeRAG2L_D_ 2297  LYFVLIQKLSDTGRKQEADRVRSMWNGRDEVELSVPECLGMRVRSLMSKAHYKQTYGIQRDKTSAKSLQPRNQLTVLEPSFLPGHCKYRL
GFRY01095553      LYFVLIQKLSDTGRKQEADRVRSMWNGRDEVELSVPECLGMRVRSLMSKAHYKQTYGIQRDKTSAKSLQPPNQLTVLEPSFLPGHCKYRL
 Consensus        LYFVLIQKLSDTGRKQEADRVRSMWNGRDEVELSVPECLGMRVRSLMSKAHYKQTYGIQRDKTSAKSLQPrNQLTVLEPSFLPGHCKYRL

                  271                                                                                    360
NgeRAG2L_D_ 2297  LKDSVLLAEQDLLSAQDSQPLDIMRDYHSFPADFPLPNVMGVELDYISAVAKTLLEIDNDVSKRLDALGLPQSTKLCTVIKDGGDGLGDV
GFRY01095553      LKDSVLLAEQDLLSAQDSQPLDIMRDYHSFPADFPLPNVMGVELDYISAVAKTLLEIDNDVSKRLDALGLPQSTKLCTVIKDGGDGLGDV
 Consensus        LKDSVLLAEQDLLSAQDSQPLDIMRDYHSFPADFPLPNVMGVELDYISAVAKTLLEIDNDVSKRLDALGLPQSTKLCTVIKDGGDGLGDV

                  361                                                                                    450
NgeRAG2L_D_ 2297  NVKSRKADFALTDKVFRYSFCVLQCAASDAHGEKHTIFKEEFPNSVDCTRPVVVCNCDESDRPSVAIILSHVSKARKQMQGATLQLLDPS
GFRY01095553      NVKSRKADFALTDKVFRYSFCVLQCAASDAHGEKHTIFKEEFPNSVDCTRPVVVCNCDESDRPSVAIILSHVSKARKQMQGATLQLLDPS
 Consensus        NVKSRKADFALTDKVFRYSFCVLQCAASDAHGEKHTIFKEEFPNSVDCTRPVVVCNCDESDRPSVAIILSHVSKARKQMQGATLQLLDPS

                  451                                                                                    540
NgeRAG2L_D_ 2297  SGSVIREHDLSFYYTMVDEKFERISSGLQGSGSRFLCTLCHATTASCQADAGSHHRVRTHEETVSLYKYRLENPQNLGEDELAIQSKGVK
GFRY01095553      SGSVIREHDLSFYYTMVDEKFERISSGLQGSGSRFLCTLCHATTASCQADVGSHHRVRTHEETVSLYKYRLENPQNLGEDELAIQSKEVK
 Consensus        SGSVIREHDLSFYYTMVDEKFERISSGLQGSGSRFLCTLCHATTASCQADaGSHHRVRTHEETVSLYKYRLENPQNLGEDELAIQSKeVK

                  541                                                                                    630
NgeRAG2L_D_ 2297  SFPLISSNANERSLDATHANINIGRVLKKVVVREIAEIHEWAENDNNKTSLQNAEKEFDAKIMKEIGLQPKMMMAGNYARAVFAVENLGV
GFRY01095553      SFPLISSNANERSLDATHANINIGRVLKKVVVREIAEIHEWAENDNNKTSLQNAEKEFDAKIMKEIGLQPKMMMPGNYARAVFAVENLGV
 Consensus        SFPLISSNANERSLDATHANINIGRVLKKVVVREIAEIHEWAENDNNKTSLQNAEKEFDAKIMKEIGLQPKMMMaGNYARAVFAVENLGV

                  631                                                                                    720
NgeRAG2L_D_ 2297  VTSLIKNDERREHVSTLFNIFNRLHRVYSSNTPLTSVPDETAAYRENALQFAFHLGENFPYVPWSNYIHRVVEHVDEILHDPSGVQSVGL
GFRY01095553      VTSLIKNDERREHVSTLFNIFNRLHRVYSSNTPLTSVPDETAAYRENALQFAFHLGENFPYVPWSNYIHRVVEHVDEILHDPSGVQSVGL
 Consensus        VTSLIKNDERREHVSTLFNIFNRLHRVYSSNTPLTSVPDETAAYRENALQFAFHLGENFPYVPWSNYIHRVVEHVDEILHDPSGVQSVGL

                  721                                                                                806
NgeRAG2L_D_ 2297  LSSEGGESGNKLYRFIRKSLSRQDSAFDLRDALVFHWLYTSKVLQKHAATEKVSKTCTICGMADHNRRTCAYKQDAAPEDDMMEES
GFRY01095553      LSSEGGESGNKLYRFIRKSLSRQDSAFDLRDALVFHWLYTSKVLQKHAATEKVSKTCTICGMADHNRRTCAYKQDAAPEDDMMEES
 Consensus        LSSEGGESGNKLYRFIRKSLSRQDSAFDLRDALVFHWLYTSKVLQKHAATEKVSKTCTICGMADHNRRTCAYKQDAAPEDDMMEES
```

4. RAGL Detection info

Method: TBLASTN
Database searched: Whole-genome shotgun contigs (WGS) + only in Nemertea clade (taxid:6217).

2.1. RAG1L

Query: **NgeRAG1L_D_3820 (previously described)**
Subject: **NMRB01002705.1 Length: 92969 Number of Matches: 2**

Score	Expect	Method	Identities	Positives	Gaps	Frame
234 bits(598)	1e-62	Compositional matrix adjust.	120/249(48%)	161/249(64%)	15/249(6%)	+2

```
Query  1      MDQYHKQVLQRICRLCSGDL--KKVKRQNNFFDVSKAAVVLQLHYSISVGDESEAVFPRC   58
```

```
                 MDQYH+ VL  +CR+C  +L  + VKRQ  F++V  AA  +++HY ISV DE E  FP
Sbjct  27011  MDQYHRDVLDHVCRICGDELQARAVKRQKKFYEVKGAAETIEVHYGISVRDEDEHQFPGY  27190

Query  59     ICNSCRNKVDRLKQTAVSKNLTAPKFLPHQECDCFCVGRGSLLKLPLPSEPVQIEPAEVV  118
               CN CRNK+DRLK    SKNLT  +F PH + DCFC+G GS L L +P EPV ++P  V
Sbjct  27191  ACNRCRNKLDRLKNVKESKNLTVFQFSPHADEDCFCIGVGSKLGLAVPQEPVPVDP--VT  27364

Query  119    ISSDHSYSGSGPNECGTGTSNGTTNDVMSPQPRKKCRLQQVSRHHIKQRRLKDLISSVGS  178
              I+ DH+Y  SG   C               +PRKK  L Q +R ++K++RLK +IS V
Sbjct  27365  ITVDHAYQSSGSPSCSKPVI----------EPRKKIPLNQANRQYVKRQRLKGMISGVDQ  27514

Query  179    FCDDRMEDPVDAMYAVLIQKLLDDGRKKEANKILSVWQNKGEELEMSAQDCLAVRVRTLQ  238
              FC++  ED VDA+Y VLIQKL D GRK+EA+++ S+W N  +E+E+S  +CL +RVR+L
Sbjct  27515  FCEEHKEDTVDALYFVLIQKLSDTGRKQEADRVRSMW-NGRDEVELSVPECLGMRVRSLM  27691

Query  239    SKTRYRQQY  247
              SK  Y+Q Y
Sbjct  27692  SKAHYKQTY  27718
```

Score	Expect	Method	Identities	Positives	Gaps	Frame
720 bits(1859)	0.0	Compositional matrix adjust.	337/571(59%)	434/571(76%)	6/571(1%)	+2

```
Query  247    YSLQRDNTNSASLKPPYQLDAAEATFMPGHCAYKLMKDSVVIAEQSISTNDQYEPKDIMD  306
              Y +QRD T++ SL+P  QL   E +F+PGHC Y+L+KDSV++AEQ + +    +P DIM
Sbjct  28988  YGIQRDKTSAKSLQPRNQLTVLEPSFLPGHCKYRLLKDSVLLAEQDLLSAQDSQPLDIMR  29167

Query  307    QFKNFPTDFPVPNLAGVEFSFVSAVAKTLWELESLIEMRLNELGLPLSTQIETIFKDGGD  366
               + +FP DFP+PN+ GVE  ++SAVAKTL E+++ +  RL+ LGLP ST++ T+ KDGGD
Sbjct  29168  DYHSFPADFPLPNVMGVELDYISAVAKTLLEIDNDVSKRLDALGLPQSTKLCTVIKDGGD  29347

Query  367    GLGDVCLKTQKADFARTDKIFRYSFCCVSCSAV-VGDKKHLLFRETFPNN---TRPVILC  422
              GLGDV +K++KADFA TDK+FRYSFC + C+A     +KH +F+E FPN+   TRPV++C
Sbjct  29348  GLGDVNVKSRKADFALTDKVFRYSFCVLQCAASDAHGEKHTIFKEEFPNSVDCTRPVVVC  29527

Query  423    HCDESDRPSISTLLANVSREREMMQDGTLVLVDPDTGDDVRQHHLHFLYTMVDEKFERTS  482
              +CDESDRPS++ +L++VS+ R+ MQ   TL L+DP +G  +R+H L F YTMVDEKFER S
Sbjct  29528  NCDESDRPSVAIILSHVSKARKQMQGATLQLLDPSSGSVIREHDLSFYYTMVDEKFERIS  29707

Query  483    SGLQGSGSRFICTLCYAATNTCQADVGSFSRCRTYDETVSLYKYRLENPDKLNENDLAER  542
              SGLQGSGSRF+CTLC+A T +CQAD GS  R RT++ETVSLYKYRLENP  L E++LA +
Sbjct  29708  SGLQGSGSRFLCTLCHATTASCQADAGSHHRVRTHEETVSLYKYRLENPQNLGEDELAIQ  29887

Query  543    CKGVNSIPLIKSDATDRSVDSTHANINIGRVFKRILVREIAEIYEWAENDRNKKGLQHAE  602
               KGV S PLI S+A +RS+D+THANINIGRV K+++VREIAEI+EWAEND NK  LQ+AE
Sbjct  29888  SKGVKSFPLISSNANERSLDATHANINIGRVLKKVVVREIAEIHEWAENDNNKTSLQNAE  30067

Query  603    KNLDQHLMKTLGLQSKMIMPGNYARILFLDKNAETVSELIKNPDRKQHFKCILGLFSKLK  662
              K  D  +MK +GLQ KM+M GNYAR +F  +N   V+ LIKN +R++H   +  +F++L
Sbjct  30068  KEFDAKIMKEIGLQPKMMMAGNYARAVFAVENLGVVTSLIKNDERREHVSTLFNIFNRLH  30247

Query  663    AVYSSKDPTSSVPEETACYKENALQFAFHLGEHFNYVPWSNYLHKLIEHVDEIVQDPRCL  722
               VYSS  P +SVP+ETA Y+ENALQFAFHLGE+F YVPWSNY+H+++EHVDEI+ DP  +
Sbjct  30248  RVYSSNTPLTSVPDETAAYRENALQFAFHLGENFPYVPWSNYIHRVVEHVDEILHDPSGV  30427

Query  723    HSVGLLSSEGGECGNKLYRFIRRSLSRQDTAGDCDLRDALVFHWIYTSKCIQKLATSCKA  782
               SVGLLSSEGGE GNKLYRFIR+SLSRQD+A   DLRDALVFHW+YTSK +QK A + K
Sbjct  30428  QSVGLLSSEGGESGNKLYRFIRKSLSRQDSA--FDLRDALVFHWLYTSKVLQKHAATEKV  30601
```

```
Query  783    PYNCRKCGQVGHNSRTCDENGDTTTVDQGMD  813
                 C  CG   HN RTC    D    D  M+
Sbjct  30602  SKTCTICGMADHNRRTCAYKQDAAPEDDMME  30694
```

2.2 RAG2L

Query: NgeRAG2L_D_3820 (previously described)
Subject: NMRB01002705.1 Length: 92969 Number of Matches: 1

Score	Expect	Method	Identities	Positives	Gaps	Frame
410 bits(1055)	3e-126	Compositional matrix adjust.	221/507(44%)	317/507(62%)	20/507(3%)	-3

```
Query  17     EFFNNARFYSVLKAVQNRRKVTKKAIVEEASKGALFSGSGHLSWLIFYNDPRQH-TAAVT  75
              +F+ ++RFYS+LKA++ RRK TKKA+  +    ALFSG GH+ W +     R+  T A++
Sbjct  32241  DFWQDSRFYSILKAIKKRRKTTKKAVSGDIGNLALFSGPGHIIWTVESVATRETLTPALS  32062

Query  76     N----NGSGDEKTFSVAVLGGMHAMKPLTNLSGTVYSYAFKVNDDDIKILSVAECKCSGA  131
              +        D K  +V+VLGGMH   PLT LSGT+++Y+F V ++D+K+L+ +E KCSGA
Sbjct  32061  HFFDERDVIDGKIITVSVLGGMHPRLPLTTLSGTIFNYSFHVQENDVKVLNASEKKCSGA  31882

Query  132    QIALRPFVHSACIVETKATRESSRSRRTGPGGARSAMISICSIWGGLDPNRMLPHNDLTI  191
              QI LRP +H+A   +T  TR+ +  RR         A  ++  IWGG+D NRM PHNDL +
Sbjct  31881  QIVLRPLLHAAACSDTTRTRDEAPRRRARGVARTGATKTLIFIWGGMDLNRMEPHNDLVV  31702

Query  192    LENFGRDQ-FTARLVCQ---EGTKNV--QTGTVPEARYGHTLSGLHYQGSLVGAVLYGGV  245
              LE FGRDQ ++A+LV     EGT     Q G VPE RY HTL+G+H  GSL+GA+L+GGV
Sbjct  31701  LETFGRDQNYSAKLVGSDRPEGTCTTVKQMGDVPEGRYAHTLTGIHENGSLLGALLFGGV  31522

Query  246    TVKNTTQGNEHSTQDGTLYFLDLENYQWKKL--SELAPVAYHTSCEIRDLTLVFIGGLSQ  303
                +       H T D  LYF+DL  Y+W ++   +L PVAYHT  E+RD  + ++GGL Q
Sbjct  31521  EKRP----EGHGTFDKDLYFIDLLQYKWFQMVSEQLNPVAYHTGTELRDKVVAYVGGLRQ  31354

Query  304    LCDNRVTSTQRMSIQKITIVELNSTIDQVTVQNLSLSFLDPALDNVFLSGHSANICSPNQ  363
              +      + +R SI ++ +  L     QV+V    L   +    +  LSGH++     ++
Sbjct  31353  MPGANGATIERQSILEVKLAVLEFIDGQVSVTVSQLIMPNFLGKDTLLSGHTSTALDESR  31174

Query  364    LLIFGGYQQHISDMNTKIPSRTYYLVDITSLSIQAKEAPVGFDMAGHTSVRLDHCSLFFH  423
              +L+FGGYQQ  +    K P   Y+++D+ + SIQ K+ P GF MA HT+V +D   +FFH
Sbjct  31173  VLVFGGYQQQDNQHPAKEPGGQYFVLDLATNSIQMKDIPSGFAMACHTAVMMDPALIFFH  30994

Query  424    GGANEHLFTMTTKRMELGVCEAEKCIVNDHFSPGEIVTKLQCTLCDAFFHSCCDQGESHT  483
              GGAN+HLFT+TTKR+E G C+++KCIV D+FSPGEI+  LQC  CD ++HSCC   ++
Sbjct  30993  GGANQHLFTLTTKRLEPGRCQSDKCIVTDNFSPGEIIESLQCETCDKWYHSCCAGSKNGR  30814

Query  484    SHSQEDIRFTCPKCRPKGKRQRKNKAR  510
              S ++    F C  CRPK  + +K + R
Sbjct  30813  SETES---FVCVDCRPKQTKGKKKQRR  30742
```

WGS: NMRB01001040.1

5. Summary

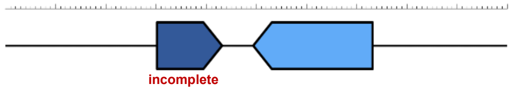

Detected configuration: RAG1L - RAG2L

Predicted gene	Predicted CDS	start	end	strand	Observations
RAG1L	1	70312	67937	-	complete; no stop codon
RAG2L(!)	1	66054	67316	+	incomplete (first 80 aa N-terminal are missing); no stop codons

Selected predicted protein product

NgeRAG1L_D_1040

```
MDAYEKALQDLCRLCGDQLKRHAAPRQITFASVNQHQQTILRHYHIKVEDEDNLPKRVCN
KCRSKLVRLSKKQESADISVPGPDDFARKIHQELGLVAPEPVAIDIVELDVSLEHSYSTT
SDGPIKPNTTERRQKTNLRAANRQYVKRQRLKKLIDSIDEFCDEHRENSVDSLFFILSQK
FLDLGRKKDADRVLSLWHDIDDSEEKLEMSADECLAMRVRTMQSKTQYKKMYKIQNKISA
RPLQPPKQLTKSETKYLPGHCKYKLVNSNGDILTEQEILSKEQCEPQQLLEKLNKFPSDF
PLPNISGVQFNYTSAIAKTLCELEGIIAGRLSEFGLPLDTPVYTLIKDGGDGLGDVDVKR
MRAAYSLPDKVFRYSFCVLEVRAKVNDTSYILFSEDMANSVDCTRPVVISHCNESDAPSM
ALVLSSVSKHRDAMNGNILQLHLESGSLRCHHLRLIYTMVDEKFERCSSGLQGSGSRYIC
TLCYATTQTCQCDHGSFQRTRTHAETVSLYHYRLENPNKLSENDLADKCKGVKSYPLIKS
DAADRSLDATHANINMGRVLKKIIVREMASVHEWVENDNNKAKLQHAERNLDEHLRLHLG
LQPKLIITGNDARALFSPENLDVVTSLIENAERREHVCSLLRLFSKLQKVYTCKYPLLSM
PEETALYSDTATEFASCLGTHFPYHNNWSNYTHKIVEHVAEIISDPNGIGSVGLLSSEGG
ESGNKLYRFIRKSLSRHDSGNDMRDVLVFHWLFTSKSVQKHAETSKQVQYCGNCGEPNHN
VRTCTNVSILEE
```

Predicted using FGENESH+ (*C.elegans* dataset an NgeRAG1L_3820 as reference)

NgeRAG2L_D_1040 (!)

```
MHHRMQLTDRSAAIHSYEFGVEEDSVTVLQSSGGIKCTGSQIGLRSLLYSACVKRSELTRS
ASQRRGRPGAQSERTRVYIWGGIGCNVFLPHSDLIILETYGRDYSAMLWGSNIATAAATVN
VQSGEVPSPRYGHTLTPLHSRTTGDLCGAILYGGVDVNNQLLGENVHETKDGTVYFLNLDS
CKWRKIEVENGLSLAPCAYHTGTEFRFGTVAFIGGLVQSFDGHQVTVSRNSIQELVLINIH
DIDLANDTDVVTAQLVKVDMAFLGIPRGDVYLSGHTATECLDSKILVYGGYQHSVSPAPEN
DKPGREYFVLDITNSTILRLEAPQGFEMANHCAVTMDSNSVFFHGGANQHLFILTTKSMEP
THCEAEDCVVFDNHSPGEAVTWLQCDGPCQKWYHYCCSGLSGEPDPEKIAEIEFHCKSCNK
PKKSRKKKV
```

Predicted using FGENESH+ (*C.elegans* dataset an NgeRAG2L_3820 as reference)

6. RAGL Detection info

Method: TBLASTN
Database searched: Whole-genome shotgun contigs (WGS) + only in Nemertea clade (taxid:6217).

2.1. RAG1L

Query: NgeRAG1L_D_3820 (previously described)
Subject: NMRB01001040.1 Length: 236192 Number of Matches: 1

Score	Expect	Method	Identities	Positives	Gaps	Frame
764 bits(1972)	0.0	Compositional matrix adjust.	407/812(50%)	555/812(68%)	35/812(4%)	-2

```
Query  23     AFSMDQYHKQVLQRICRLCSGDLKK--VKRQNNFFDVSKAAVVLQLHYSISVGDESEAVF  80
              A SMD Y K  LQ +CRLC   LK+    RQ  F  V++    +  HY I V DE
Sbjct  70321  APSMDAYEK-ALQDLCRLCGDQLKRHAAPRQITFASVNQHQQTILRHYHIKVEDEDN--L  70151

Query  81     PRCICNSCRNKVDRLKQTAVSKNLTAPKFLPHQECDCFCVGRGSLLKLPLPSEPVQIEPA  140
              P+ +CN CR+K+ RL +   S +++ P        D F       L L  P EPV I+
Sbjct  70150  PKRVCNKCRSKLVRLSKKQESADISVPG------PDDFARKIHQELGLVAP-EPVAIDIV  69992

Query  141    EVVISSDHSYSGSGPNECGTGTSNGTTNDVMSPQPRKKCRLQQVSRHHIKQRRLKDLISS  200
              E+ +S +HSYS +        T+          + R+K  L+  +R ++K++RLK LI S
Sbjct  69991  ELDVSLEHSYSTTSDGPIKPNTT----------ERRQKTNLRAANRQYVKRQRLKKLIDS  69842

Query  201    VGSFCDDRMEDPVDAMYAVLIQKLLDDGRKKEANKILSVWQN---KGEELEMSAQDCLAV  257
              +  FCD+  E+ VD+++ +L QK LD GRKK+A+++LS+W +     E+LEMSA +CLA+
Sbjct  69841  IDEFCDEHRENSVDSLFFILSQKFLDLGRKKDADRVLSLWHDIDDSEEKLEMSADECLAM  69662

Query  258    RVRTLQSKTRYRQQYSLQRDNTNSASLKPPYQLDAAEATFMPGHCAYKLMK-DSVVIAEQ  316
              RVRT+QSKT+Y++ Y +Q +  ++  L+PP QL  +E  ++PGHC YKL+  +  ++ EQ
Sbjct  69661  RVRTMQSKTQYKKMYKIQ-NKISARPLQPPKQLTKSETKYLPGHCKYKLVNSNGDILTEQ  69485

Query  317    SISTNDQYEPKDIMDQFKNFPTDFPVPNLAGVEFSFVSAVAKTLWELESLIEMRLNELGL  376
               I + +Q EP+ ++++   FP+DFP+PN++GV+F++ SA+AKTL ELE +I  RL+E GL
Sbjct  69484  EILSKEQCEPQQLLEKLNKFPSDFPLPNISGVQFNYTSAIAKTLCELEGIIAGRLSEFGL  69305

Query  377    PLSTQIETIFKDGGDGLGDVCLKTQKADFARTDKIFRYSFCCVSCSAVVGDKKHLLFRET  436
              PL T + T+ KDGGDGLGDV +K  +A ++  DK+FRYSFC +   A V D  ++LF E
Sbjct  69304  PLDTPVYTLIKDGGDGLGDVDVKRMRAAYSLPDKVFRYSFCVLEVRAKVNDTSYILFSED  69125

Query  437    FPNN---TRPVILCHCDESDRPSISTLLANVSREREMMQDGTLVLVDPDTGDDVRQHHLH  493
                N+   TRPV++ HC+ESD PS++ +L++VS+ R+ M +G ++ +  ++G  +R HHL
Sbjct  69124  MANSVDCTRPVVISHCNESDAPSMALVLSSVSKHRDAM-NGNILQLHLESGS-LRCHHLR  68951

Query  494    FLYTMVDEKFERTSSGLQGSGSRFICTLCYAATNTCQADVGSFSRCRTYDETVSLYKYRL  553
               +YTMVDEKFER SSGLQGSGSR+ICTLCYA T TCQ D GSF R RT+ ETVSLY YRL
Sbjct  68950  LIYTMVDEKFERCSSGLQGSGSRYICTLCYATTQTCQCDHGSFQRTRTHAETVSLYHYRL  68771

Query  554    ENPDKLNENDLAERCKGVNSIPLIKSDATDRSVDSTHANINIGRVFKRILVREIAEIYEW  613
              ENP+KL+ENDLA++CKGV S PLIKSDA DRS+D+THANIN+GRV K+I+VRE+A ++EW
Sbjct  68770  ENPNKLSENDLADKCKGVKSYPLIKSDAADRSLDATHANINMGRVLKKIIVREMASVHEW  68591

Query  614    AENDRNKKGLQHAEKNLDQHLMKTLGLQSKMIMPGNYARILFLDKNAETVSELIKNPDRK  673
               END NK  LQHAE+NLD+HL   LGLQ K+I+ GN AR LF  +N + V+ LI+N +R+
Sbjct  68590  VENDNNKAKLQHAERNLDEHLRLHLGLQPKLIITGNDARALFSPENLDVVTSLIENAERR  68411

Query  674    QHFKCILGLFSKLKAVYSSKDPTSSVPEETACYKENALQFAFHLGEHFNYV-PWSNYLHK  732
              +H   +L LFSKL+ VY+ K P  S+PEETA Y + A +FA  LG HF Y   WSNY HK
Sbjct  68410  EHVCSLLRLFSKLQKVYTCKYPLLSMPEETALYSDTATEFASCLGTHFPYHNNWSNYTHK  68231


Query  733    LIEHVDEIVQDPRCLHSVGLLSSEGGECGNKLYRFIRRSLSRQDTAGDCDLRDALVFHWI  792
```

```
                ++EHV EI+ DP  + SVGLLSSEGGE GNKLYRFIR+SLSR D+    D+RD LVFHW+
Sbjct  68230   IVEHVAEIISDPNGIGSVGLLSSEGGESGNKLYRFIRKSLSRHDSGN--DMRDVLVFHWL  68057

Query  793     YTSKCIQKLATSCKAPYNCRKCGQVGHNSRTC  824
               +TSK +QK A + K    C  CG+  HN RTC
Sbjct  68056   FTSKSVQKHAETSKQVQYCGNCGEPNHNVRTC  67961
```

2.2 RAG2L

Query: NgeRAG2L_D_3820 (previously described)
Subject: NMRB01001040.1 Length: 236192 Number of Matches: 1

Score	Expect	Method	Identities	Positives	Gaps	Frame
329 bits(844)	4e-98	Compositional matrix adjust.	193/438(44%)	262/438(59%)	30/438(6%)	+3

```
Query  85      FSVAVLGGMHAMKPLTNLSGTVYSYAFKVNDDDIKIL-SVAECKCSGAQIALRPFVHSAC  143
                 V  LGGMH    LT+ S  ++SY F V +D + +L S    KC+G+QI LR  ++SAC
Sbjct  66030   LQVFALGGMHHRMQLTDRSAAIHSYEFGVEEDSVTVLQSSGGIKCTGSQIGLRSLLYSAC  66209

Query  144     IVETKATRESSRSRRTGPGGARSAMISICSIWGGLDPNRMLPHNDLTILENFGRDQ----  199
               +  ++ TR  S S+R G  GA+S    +  IWGG+  N  LPH+DL ILE +GRD
Sbjct  66210   VKRSELTR--SASQRRGRPGAQSERTRV-YIWGGIGCNVFLPHSDLIILETYGRDYSAML  66380

Query  200     FTARLVCQEGTKNVQTGTVPEARYGHTLSGLHYQ--GSLVGAVLYGGVTVKNTTQG-NEH  256
               + + +     T NVQ+G VP  RYGHTL+ LH +  G L GA+LYGGV V N   G N H
Sbjct  66381   WGSNIATAAATVNVQSGEVPSPRYGHTLTPLHSRTTGDLCGAILYGGVDVNNQLLGENVH  66560

Query  257     STQDGTLYFLDLENYQWKKLS-----ELAPVAYHTSCEIRDLTLVFIGGLSQLCDNRVTS  311
                T+DGT+YFL+L++ +W+K+        LAP AYHT  E R  T+ FIGGL Q  D    +
Sbjct  66561   ETKDGTVYFLNLDSCKWRKIEVENGLSLAPCAYHTGTEFRFGTVAFIGGLVQSFDGHQVT  66740

Query  312     TQRMSIQKITIV-----ELNSTIDQVTVQ--NLSLSFLDPALDNVFLSGHSANICSPNQL  364
                 R SIQ++ ++     +L +  D VT Q    + ++FL      +V+LSGH+A  C  +++
Sbjct  66741   VSRNSIQELVLINIHDIDLANDTDVVTAQLVKVDMAFLGIPRGDVYLSGHTATECLDSKI  66920

Query  365     LIFGGYQQHISDM--NTKIPSRTYYLVDITSLSIQAKEAPVGFDMAGHTSVRLDHCSLFF  422
               L++GGYQ  +S    N K P R Y+++DIT+ +I   EAP GF+MA H +V +D  S+FF
Sbjct  66921   LVYGGYQHSVSPAPENDK-PGREYFVLDITNSTILRLEAPQGFEMANHCAVTMDSNSVFF  67097

Query  423     HGGANEHLFTMTTKRMELGVCEAEKCIVNDHFSPGEIVTKLQCT-LCDAFFHSCCD--QG  479
               HGGAN+HLF +TTK ME   CEAE C+V D+ SPGE VT LQC   C  ++H CC    G
Sbjct  67098   HGGANQHLFILTTKSMEPTHCEAEDCVVFDNHSPGEAVTWLQCDGPCQKWYHYCCSGLSG  67277

Query  480     ESHTSHSQEDIRFTCPKC  497
               E       E I F C  C
Sbjct  67278   EPDPEKIAE-IEFHCKSC  67328
```

WGS: NMRB01000727.1

1. Summary

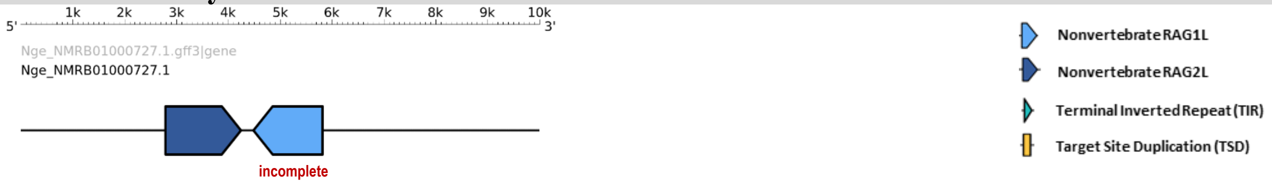

Detected configuration: RAG1L - RAG2L

Detected loci	Predicted CDS	start	end	strand	Observations
RAG1L(!)	1	45819	44482	-	Incomplete; missing N-terminal; no stop codon
RAG2L	1	42791	44245	+	complete; no stop codons

Selected predicted protein product

NgeRAG1L_D_0727

MVNETEHVLFKELFPNSVDSTRPVILCHCDESDRPSIATLLGKVSRDREKMKGATLQLYD
ASTGHCLREHELHFLYSMVDEKFERTSSGLQGSGSRYLCTLCHAHTGNCQSDVGSFTRCR
THEETVSLYKYRLENPDHLNDNDLAVKCKGVNSMPLIVSDATERSLDSTHANINIGRFFK
KILVREIAEVHQWSENEQNKTTLHHAEKTLDEHLMKTLGLQSKLIMVGNFARALFDPKNV
DIVTKLIRNEERKQHVKALLGHFSKMHAVYSCKNPVQTLPEEMAQYKENALQFAFLLGQQ
FPYVIWSNYVHKVVEHVAEIIQDPQGIHSVGALSSEGGECGNKLYRFIRKSLSRQDTSDD
CDLRDALVFQWLYTSKVLQKLAATKSFPKYCTACGGAGHNIRTCELAKKQNQTCHARQSD
DDDDSGLIENQEELEDEVAMICVNCM

Green: TSA support GFRY01046940.1 and GFRY01046941.1 (both 100% identity DNA and protein level)

Predicted using FGENESH (*C.elegans* dataset no reference) and supported by TSA entry

NgeRAG2L_D_0727
(incomplete)

MASGGVSNSEEKDFFEDSRFYSILRSVKKRRKVTKASIV
DEASKGALFSGKGHLVWSTRSGETTAAEVTPAMRAAIFGNQQEIGGEIVTVSVLGGMNSR
VPLTSLSGSIHSYTFHLNEHDIKVLSVCEKKCSGAQITLRPFVHSASISDTSLTRDGSTR
RTGRGNAATKTKIFIWGGLDPNFMTTHNDLTILETFGRDQSYNAKLMNGQQNRPGARYGH
TLTKVHHDGKLVGAVLYGGVSITGDIDHVTQDEKLYFLNLEHYTWESLTSIGESHPVPVA
YHTGTEICDGTLAYIGGLSQTLVGSQTVIKRLPIQEVMVVELSFAGDTISASVRNIKFSF
SGDPTKEVFLSGHTANVCGQDQILVFGGYQQKTPEENSDIPSGHYFLLDMNTLSMRHKEA
PEGFEMASHTTINLDSRSIFFHGGALQHLFTMTVKRMEPTICEAEKCVVKNHFSPGEATT
CLQCDACDKFFHPQKLEDINLCQMQN

Predicted using FGENESH (*C.elegans* dataset no reference)

```
                1                                                                                        90
NgeRAG1L_D_0727 MVNETEHVLFKELFPNSVDSTRPVILCHCDESDRPSIATLLGKVSRDREKMKGATLQLYDASTGHCLREHELHFLYSMVDEKFERTSSGL
TSA_GFRY01046940 MVNETEHVLFKELFPNSVDSTRPVILCHCDESDRPSIATLLGKVSRDREKMKGATLQLYDASTGHCLREHELHFLYSMVDEKFERTSSGL
TSA_GFRY01046941 MVNETEHVLFKELFPNSVDSTRPVILCHCDESDRPSIATLLGKVSRDREKMKGATLQLYDASTGHCLREHELHFLYSMVDEKFERTSSGL
 Consensus      MVNETEHVLFKELFPNSVDSTRPVILCHCDESDRPSIATLLGKVSRDREKMKGATLQLYDASTGHCLREHELHFLYSMVDEKFERTSSGL

                91                                                                                      180
NgeRAG1L_D_0727 QGSGSRYLCTLCHAHTGNCQSDVGSFTRCRTHEETVSLYKYRLENPDHLNDNDLAVKCKGVNSMPLIVSDATERSLDSTHANINIGRFFK
TSA_GFRY01046940 QGSGSRYLCTLCHAHTGNCQSDVGSFTRCRTHEETVSLYKYRLENPDHLNDNDLAVKCKGVNSMPLIVSDATERSLDSTHANINIGRFFK
TSA_GFRY01046941 QGSGSRYLCTLCHAHTGNCQSDVGSFTRCRTHEETVSLYKYRLENPDHLNDNDLAVKCKGVNSMPLIVSDATERSLDSTHANINIGRFFK
 Consensus      QGSGSRYLCTLCHAHTGNCQSDVGSFTRCRTHEETVSLYKYRLENPDHLNDNDLAVKCKGVNSMPLIVSDATERSLDSTHANINIGRFFK
```

```
                181                                                                                        270
NgeRAG1L_D_0727 KILVREIAEVHQWSENEQNKTTLHHAEKTLDEHLMKTLGLQSKLIMVGNFARALFDPKNVDIVTKLIRNEERKQHVKALLGHFSKMHAVY
TSA_GFRY01046940 KILVREIAEVHQWSENEQNKTTLHHAEKTLDEHLMKTLGLQSKLIMVGNFARALFDPKNVDIVTKLIRNEERKQHVKALLGHFSKMHAVY
TSA_GFRY01046941 KILVREIAEVHQWSENEQNKTTLHHAEKTLDEHLMKTLGLQSKLIMVGNFARALFDPKNVDIVTKLIRNEERKQHVKALLGHFSKMHAVY
 Consensus      KILVREIAEVHQWSENEQNKTTLHHAEKTLDEHLMKTLGLQSKLIMVGNFARALFDPKNVDIVTKLIRNEERKQHVKALLGHFSKMHAVY

                271                                                                                        360
NgeRAG1L_D_0727 SCKNPVQTLPEEMAQYKENALQFAFLLGQQFPYVIWSNYVHKVVEHVAEIIQDPQGIHSVGALSSEGGECGNKLYRFIRKSLSRQDTSDD
TSA_GFRY01046940 SCKNPVQTLPEEMAQYKENALQFAFLLGQQFPYVIWSNYVHKVVEHVAEIIQDPQGIHSVGALSSEGGECGNKLYRFIRKSLSRQDTSDD
TSA_GFRY01046941 SCKNPVQTLPEEMAQYKENALQFAFLLGQQFPYVIWSNYVHKVVEHVAEIIQDPQGIHSVGALSSEGGECGNKLYRFIRKSLSRQDTSDD
 Consensus      SCKNPVQTLPEEMAQYKENALQFAFLLGQQFPYVIWSNYVHKVVEHVAEIIQDPQGIHSVGALSSEGGECGNKLYRFIRKSLSRQDTSDD

                361                                                                                    446
NgeRAG1L_D_0727 CDLRDALVFQWLYTSKVLQKLAATKSFPKYCTACGGAGHNIRTCELAKKQNQTCHARQSDDDDDSGLIENQEELEDEVAMICVNCM
TSA_GFRY01046940 CDLRDALVFQWLYTSKVLQKLAATKSFPKYCTACGGAGHNIRTCELAKKQNQTCHARQSDDDDDSGLIENQEELEDEVAMICVNCM
TSA_GFRY01046941 CDLRDALVFQWLYTSKVLQKLAATKSFPKYCTACGGAGHNIRTCELAKKQNQTCHARQSDDDDDSGLIENQEELEDEVAMICVNCM
 Consensus      CDLRDALVFQWLYTSKVLQKLAATKSFPKYCTACGGAGHNIRTCELAKKQNQTCHARQSDDDDDSGLIENQEELEDEVAMICVNCM
```

2. RAGL Detection info

Method: TBLASTN
Database searched: Whole-genome shotgun contigs (WGS) + only in Nemertea clade (taxid:6217).

2.1. RAG1L

Query: NgeRAG1L_D_3820 (previously described)
Subject: NMRB01000727.1 Length: 293265 Number of Matches: 1

Score	Expect	Method	Identities	Positives	Gaps	Frame
641 bits(1654)	0.0	Compositional matrix adjust.	298/426(70%)	358/426(84%)	3/426(0%)	-1

```
Query  378    ADFARTDKIFRYSFCCVSCSAVVGDKKHLLFRETFPNN---TRPVILCHCDESDRPSIST  434
              ++FARTDKIFRYSFCC+ CS +V + +H+LF+E FPN+   TRPVILCHCDESDRPSI+T
Sbjct  45882  SEFARTDKIFRYSFCCLQCSVMVNETEHVLFKELFPNSVDSTRPVILCHCDESDRPSIAT  45703

Query  435    LLANVSREREMMQDGTLVLVDPDTGDDVRQHHLHFLYTMVDEKFERTSSGLQGSGSRFIC  494
              LL  VSR+RE M+  TL L D  TG  +R+H LHFLY+MVDEKFERTSSGLQGSGSR++C
Sbjct  45702  LLGKVSRDREKMKGATLQLYDASTGHCLREHELHFLYSMVDEKFERTSSGLQGSGSRYLC  45523

Query  495    TLCYAATNTCQADVGSFSRCRTYDETVSLYKYRLENPDKLNENDLAERCKGVNSIPLIKS  554
              TLC+A T  CQ+DVGSF+RCRT++ETVSLYKYRLENPD LN+NDLA +CKGVNS+PLI S
Sbjct  45522  TLCHAHTGNCQSDVGSFTRCRTHEETVSLYKYRLENPDHLNDNDLAVKCKGVNSMPLIVS  45343

Query  555    DATDRSVDSTHANINIGRVFKRILVREIAEIYEWAENDRNKKGLQHAEKTNDQHLMKTLG  614
              DAT+RS+DSTHANINIGR FK+ILVREIAE+++W+EN++NK  L HAEKT D+HLMKTLG
Sbjct  45342  DATERSLDSTHANINIGRFFKKILVREIAEVHQWSENEQNKTTLHHAEKTLDEHLMKTLG  45163

Query  615    LQSKMIMPGNYARILFLDKNAETVSELIKNPDRKQHFKCILGLFSKLKAVYSSKDPTSSV  674
              LQSK+IM GN+AR LF  KN + V++LI+N +RKQH K +LG FSK+ AVYS K+P  ++
Sbjct  45162  LQSKLIMVGNFARALFDPKNVDIVTKLIRNEERKQHVKALLGHFSKMHAVYSCKNPVQTL  44983

Query  675    PEETACYKENALQFAFHLGEHFNYVPWSNYLHKLIEHVDEIVQDPRCLHSVGLLSSEGGE  734
              PEE A YKENALQFAF LG+ F YV WSNY+HK++EHV EI+QDP+ +HSVG LSSEGGE
Sbjct  44982  PEEMAQYKENALQFAFLLGQQFPYVIWSNYVHKVVEHVAEIIQDPQGIHSVGALSSEGGE  44803

Query  735    CGNKLYRFIRRSLSRQDTAGDCDLRDALVFHWIYTSKCIQKLATSCKAPYNCRKCGQVGH  794
              CGNKLYRFIR+SLSRQDT+ DCDLRDALVF W+YTSK +QKLA +    P  C  CG  GH
Sbjct  44802  CGNKLYRFIRKSLSRQDTSDDCDLRDALVFQWLYTSKVLQKLAATKSFPKYCTACGGAGH  44623
```

```
Query  795    NSRTCD  800
              N RTC+
Sbjct  44622  NIRTCE  44605
```

2.2 RAG2L

Query: **NgeRAG2L_D_3820 (previously described)**
Sequence ID: NMRB01000727.1 Length: 293265 Number of Matches: 1

Score	Expect	Method	Identities	Positives	Gaps	Frame
476 bits(1225)	4e-149	Compositional matrix adjust.	253/488(52%)	328/488(67%)	32/488(6%)	+2

```
Query  1      MAEVSVLNRHDRESGDEFFNNARFYSVLKAVQNRRKVTKKAIVEEASKGALFSGSGHLSW  60
              MA   V N  +++    FF ++RFYS+L++V+ RRKVTK +IV+EASKGALFSG GHL W
Sbjct  42791  MASGGVSNSEEKD----FFEDSRFYSILRSVKKRRKVTKASIVDEASKGALFSGKGHLVW  42958

Query  61     LIFYND-------PRQHTAAVTNNGSGDEKTFSVAVLGGMHAMKPLTNLSGTVYSYAFKV  113
                   +       P    A   N      +  +V+VLGGM++  PLT+LSG+++SY F +
Sbjct  42959  STRSGETTAAEVTPAMRAAIFGNQQEIGGEIVTVSVLGGMNSRVPLTSLSGSIHSYTFHL  43138

Query  114    NDDDIKILSVAECKCSGAQIALRPFVHSACIVETKATRESSRSRRTGPGGARSAMISICS  173
              N+ DIK+LSV E KCSGAQI LRPFVHSA I +T  TR+ S +RRTG G A  A  +
Sbjct  43139  NEHDIKVLSVCEKKCSGAQITLRPFVHSASISDTSLTRDGS-TRRTGRGNA--ATKTKIF  43309

Query  174    IWGGLDPNRMLPHNDLTILENFGRDQ-FTARLVCQEGTKNVQTGTVPEARYGHTLSGLHY  232
              IWGGLDPN M  HNDLTILE FGRDQ + A+L+   G +N      P ARYGHTL+ +H+
Sbjct  43310  IWGGLDPNFMTTHNDLTILETFGRDQSYNAKLM--NGQQNR-----PGARYGHTLTKVHH  43468

Query  233    QGSLVGAVLYGGVTVKNTTQGNEHSTQDGTLYFLDLENYQWKKLSELA-----PVAYHTS  287
               G LVGAVLYGGV++   T   +H TQD  LYFL+LE+Y W+ L+ +      PVAYHT
Sbjct  43469  DGKLVGAVLYGGVSI---TGDIDHVTQDEKLYFLNLEHYTWESLTSIGESHPVPVAYHTG  43639

Query  288    CEIRDLTLVFIGGLSQLCDNRVTSTQRMSIQKITIVELNSTIDQVT--VQNLSLSFLDPA  345
               EI D TL +IGGLSQ      T  +R+ IQ++ +VEL+   D ++  V+N+  SF
Sbjct  43640  TEICDGTLAYIGGLSQTLVGSQTVIKRLPIQEVMVVELSFAGDTISASVRNIKFSFSGDP  43819

Query  346    LDNVFLSGHSANICSPNQLLIFGGYQQHISDMNTKIPSRTYYLVDITSLSIQAKEAPVGF  405
                 VFLSGH+AN+C  +Q+L+FGGYQQ   + N+ IPS  Y+L+D+ +LS++ KEAP GF
Sbjct  43820  TKEVFLSGHTANVCGQDQILVFGGYQQKTPEENSDIPSGHYFLLDMNTLSMRHKEAPEGF  43999

Query  406    DMAGHTSVRLDHCSLFFHGGANEHLFTMTTKRMELGVCEAEKCIVNDHFSPGEIVTKLQC  465
              +MA HT++ LD  S+FFHGGA +HLFTMT KRME  +CEAEKC+V +HFSPGE  T LQC
Sbjct  44000  EMASHTTINLDSRSIFFHGGALQHLFTMTVKRMEPTICEAEKCVVKNHFSPGEATTCLQC  44179

Query  466    TLCDAFFH  473
                CD FFH
Sbjct  44180  DACDKFFH  44203
```

WGS: NMRB01002182.1

1. Summary

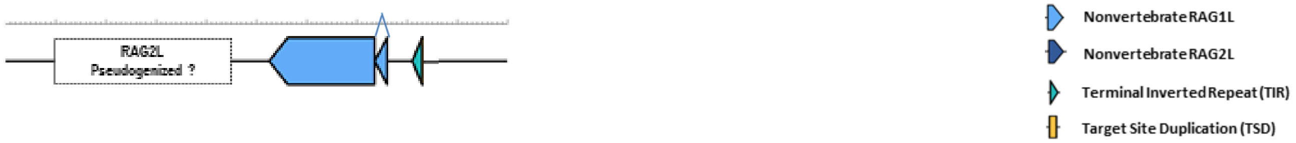

Detected configuration: RAG1L - RAG2L

Detected loci	Predicted CDS	start	end	strand	Observations
RAG1L	1	69296	69030	-	complete; no stop codons
	2	69057	66919	-	
RAG2L	-	59366	61236	+	Multiple fragments, probably 2 copies overlaid. no stop codons

Selected predicted protein product	
NgeRAG1L_D_2182	MDQYHAQVLQKICRLCNEALTPVKRQKSFYEVSEAADTIQLHYGISVAGESEEIFPKQIC NKCRNRVCRLMKNVSEGKGIDMPISINIEPSVVTVEADHAYLGPGRPNTPSCTSPTSESR KKPPLQQVTRHHIKQRRLKELILSVDNFCEEKKEDLVDSLYAILIQKLVDVGRKREADKV LSLWKSQGDDMELSPEECLAMRVRTLQSKVHYRHAYNIQRDKTCANSLKPPHQLTAAEPN FLPGHCAYKLCKNSEVIAQQDILSDKDCEPKDIMRRFKNFPEDFALPNLSGVEFSYISAV AKCLWELDDTISKSLSELGLPSSTVVKTIIKDGGDGLGDVNVKMQKADFARTDKVFRYSF CCLSCVAIVDGKEHVLFREEFASSVDNTRPVIVGHCDESDRPSISTLLFSLSREREKMKG ATLQLLDGSGACVREHQLHFIYSMVDEKFERTSSGLQGSGSSFLCTLCSATSSSCQPDVG SFSRTRTYDETVSLYKYRLENPDNLNDSDLAQKCKGVNSIPLITSDATERSLDSTHANIN LGRVFKKVLVRETAEVYAWTESEKNRTALQHAEKMLDEHLRKTLGLQPKLMMPGNYARTI FDRKNIGIVSALIKDPERKQHVATLFNLFSKMQAVYSCKNPTRTLPVETESYKEHALQFA FCLGEHFPYVNWSNYTHKVVEHVDEIIRAPHGIQSVGLLSSEGGEAGNKLYRFIRKALSR QDTSGDCDLRDALVFQWLYTSKSLQKLAATKKFPKYCTGCGQAGHNCRTCESVNQNEL

(predicted coding regions merging area is underlined as it is less confident)

Predicted using FGENESH+ (*C.elegans* dataset) and NgeRAG1L_D_2322 as homolog.

2. RAGL Detection info

Method: TBLASTN
Database searched: Whole-genome shotgun contigs (WGS) + only in Nemertea clade (taxid:6217).

2.1. RAG1L

Query: **NgeRAG1L_D_3820 (previously described)**
Subject: **NMRB01002182.1 Length: 120479 Number of Matches: 2**

Score	Expect	Method	Identities	Positives	Gaps	Frame
114 bits(285)	4e-24	Compositional matrix adjust.	54/89(61%)	67/89(75%)	1/89(1%)	-1

```
Query  1      MDQYHKQVLQRICRLCSGDLKKVKRQNNFFDVSKAAVVLQLHYSISVGDESEAVFPRCIC  60
              MDQYH QVLQ+ICRLC+  L  VKRQ +F++VS+AA  +QLHY ISV  ESE +FP+ IC
Sbjct  69296  MDQYHAQVLQKICRLCNEALTPVKRQKSFYEVSEAADTIQLHYGISVAGESEEIFPKQIC  69117
```

```
Query  61     NSCRNKVDRL-KQTAVSKNLTAPKFLPHQ  88
              N CRN+V RL K  +  K LTAP+F PH+
Sbjct  69116  NKCRNRVCRLMKNVSEGKGLTAPQFFPHK  69030
```

Score	Expect	Method	Identities	Positives	Gaps	Frame
932 bits(2408)	0.0	Compositional matrix adjust.	458/710(65%)	570/710(80%)	18/710(2%)	-3

```
Query  97     RGSLLKLPLPSEPVQIEPAEVVISSDHSYSGSG-PN--ECGTGTSNGTTNDVMSPQPRKK  153
              +G +L + +P   + IEP+ V + +DH+Y G G PN   C + TS          + RKK
Sbjct  69033  QGQVLGIDMPIS-INIEPSVVTVEADHAYLGPGRPNTPSCTSPTS----------ESRKK  68887

Query  154    CRLQQVSRHHIKQRRLKDLISSVGSFCDDRMEDPVDAMYAVLIQKLLDDGRKKEANKILS  213
                LQQV+RHHIKQRRLK+LI SV +FC+++ ED VD++YA+LIQKL+D GRK+EA+K+LS
Sbjct  68886  PPLQQVTRHHIKQRRLKELILSVDNFCEEKKEDLVDSLYAILIQKLVDVGRKREADKVLS  68707

Query  214    VWQNKGEELEMSAQDCLAVRVRTLQSKTRYRQQYSLQRDNTNSASLKPPYQLDAAEATFM  273
              +W+++G+++E+S ++CLA+RVRTLQSK  YR  Y++QRD T + SLKPP+QL AAE  F+
Sbjct  68706  LWKSQGDDMELSPEECLAMRVRTLQSKVHYRHAYNIQRDKTCANSLKPPHQLTAAEPNFL  68527

Query  274    PGHCAYKLMKDSVVIAEQSISTNDQYEPKDIMDQFKNFPTDFPVPNLAGVEFSFVSAVAK  333
              PGHCAYKL K+S VIA+Q I ++   EPKDIM +FKNFP DF +PNL+GVEFS++SAVAK
Sbjct  68526  PGHCAYKLCKNSEVIAQQDILSDKDCEPKDIMRRFKNFPEDFALPNLSGVEFSYISAVAK  68347

Query  334    TLWELESLIEMRLNELGLPLSTQIETIFKDGGDGLGDVCLKTQKADFARTDKIFRYSFCC  393
               LWEL+  I   L+ELGLP ST ++TI KDGGDGLGDV +K QKADFARTDK+FRYSFCC
Sbjct  68346  CLWELDDTISKSLSELGLPSSTVVKTIIKDGGDGLGDVNVKMQKADFARTDKVFRYSFCC  68167

Query  394    VSCSAVVGDKKHLLFRETFP---NNTRPVILCHCDESDRPSISTLLANVSREREMMQDGT  450
              +SC A+V  K+H+LFRE F    +NTRPVI+ HCDESDRPSISTLL ++SRERE M+  T
Sbjct  68166  LSCVAIVDGKEHVLFREEFASSVDNTRPVIVGHCDESDRPSISTLLFSLSREREKMKGAT  67987

Query  451    LVLVDPDTGDDVRQHHLHFLYTMVDEKFERTSSGLQGSGSRFICTLCYAATNTCQADVGS  510
              L L+D  +G  VR+H LHF+Y+MVDEKFERTSSGLQGSGS F+CTLC A +++CQ DVGS
Sbjct  67986  LQLLD-GSGACVREHQLHFIYSMVDEKFERTSSGLQGSGSSFLCTLCSATSSSCQPDVGS  67810

Query  511    FSRCRTYDETVSLYKYRLENPDKLNENDLAERCKGVNSIPLIKSDATDRSVDSTHANINI  570
              FSR RTYDETVSLYKYRLENPD LN++DLA++CKGVNSIPLI SDAT+RS+DSTHANIN+
Sbjct  67809  FSRTRTYDETVSLYKYRLENPDNLNDSDLAQKCKGVNSIPLITSDATERSLDSTHANINL  67630

Query  571    GRVFKRILVREIAEIYEWAENDRNKKGLQHAEKTNDQHLMKTLGLQSKMIMPGNYARILF  630
              GRVFK++LVRE AE+Y W E+++N+  LQHAEK  D+HL KTLGLQ K++MPGNYAR +F
Sbjct  67629  GRVFKKVLVRETAEVYAWTESEKNRTALQHAEKMLDEHLRKTLGLQPKLMMPGNYARTIF  67450

Query  631    LDKNAETVSELIKNPDRKQHFKCILGLFSKLKAVYSSKDPTSSVPEETACYKENALQFAF  690
                KN   VS LIK+P+RKQH   +  LFSK++AVYS K+PT ++P ET  YKE+ALQFAF
Sbjct  67449  DRKNIGIVSALIKDPERKQHVATLFNLFSKMQAVYSCKNPTRTLPVETESYKEHALQFAF  67270

Query  691    HLGEHFNYVPWSNYLHKLIEHVDEIVQDPRCLHSVGLLSSEGGECGNKLYRFIRRSLSRQ  750
               LGEHF YV WSNY HK++EHVDEI++ P  + SVGLLSSEGGE GNKLYRFIR++LSRQ
Sbjct  67269  CLGEHFPYVNWSNYTHKVVEHVDEIIRAPHGIQSVGLLSSEGGEAGNKLYRFIRKALSRQ  67090

Query  751    DTAGDCDLRDALVFHWIYTSKCIQKLATSCKAPYNCRKCGQVGHNSRTCD  800
              DT+GDCDLRDALVF W+YTSK +QKLA + K P  C  CGQ GHN RTC+
Sbjct  67089  DTSGDCDLRDALVFQWLYTSKSLQKLAATKKFPKYCTGCGQAGHNCRTCE  66940
```

WGS: NMRB01000732.1

1. Summary

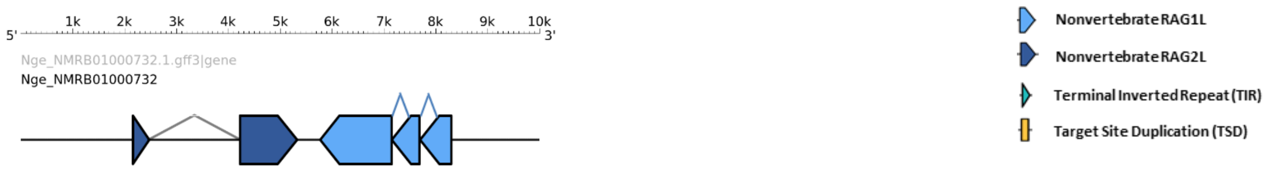

Detected configuration: RAG1L - RAG2L

Detected loci	Predicted CDS	start	end	strand	Observations
RAG1L (!)	1	230294	229693	-	complete; no stop codons;
	2	229686	229129	-	less confident protein prediction !
	3	229177	227765	-	
RAG2L	1	224156	224476	+	complete; no stop codons
	2	226220	227324	+	

Selected predicted protein product

NgeRAG1L_B_0732(!)

MASHDSLHKERVSKRCRVCGKRLPRDDFRQFSVDVYQHILQQLYSVDVMNEDKHVFPKKL
CKSCQLMCERAKNVGGDFRAKKKIAKFEKHPNEKCPLCAPAGGRCPTNEDNEMSLVARDE
FHGHVMTSAAAAEDMTSAAAAEDITLPSDPSVVTTPTAERQV[GLNFPKTPKKRKRPKSS
KKEHLSLAAPKYLKQKRLKPLISRINKFCE]ENKENKIDVLFCTLSITLYDKNEKEKSWK
VRDIWLEKMDTLPVEECLASRVENLQSKSQYRRQYSFLKSKGITALQPPNKLDEMEFHFM
PAAARYSLDVQEKESSFHRSLVKIDDAPSTFEPIDILSEFNDNLVELATPNCKGVR[WFY
PDAVAKTLMELDPLIA]KGIHQIGLDPGNPSLVIHTTIKDGADGMGDVSVHKESSDRFLP
DKAFRFAFAVLKCQIEIDGKLVTVFEEDKPNSVRTNRPLLEAIGDENSQATSVLCMLPIE
QERALMQSNTMKVKVLDTGMWRQHRLIFYNSMIDEKFDRAEGGLQGSGSSFLCTLCTAER
ATAKTCLGSFNITRTYEETRKMAEFIRTNPGNRSKAELDKLAKGVKSAPLSTSDAINKAL
DATHADINLAIFFRKLIVREISTVNKWEKTADVKSMIEDAEAKFDEHVKKTVGLNPQLCM
PGNYARIIFLAENANVILHLVPSEERKRYLAEVLAIFRELRKIYRAKAPKTDFGDEVGQY
KEKAVKMGKILIMHFDYAPWPNYLHKVIEHVQELIERGPGTIGALSSEGNEAGNKIFREI
RKNLSRKGKTSDGLRDVLWLHWLHSSPQLQRLSSVTPSKNKCSNCKLVGHKKSTCVQVRA
TKETDK

Consensus of predicted potential genes using Augustus (multiple invertebrate bilaterian species datasets) and FGENESH+ (multiple invertebrate bilaterian species datasets), using CviRAG1L_B_Biv1_0007, PimRAG1L_B_Biv1_4498 and SpuRAGL1 as homologous references.

Given the multitude of exon bindings variants this translation is less confident!

Predicted coding regions merging area is underlined as it is less confident. Regions that keep homology with the references, but are less likely to be part of the coding region are shown between brackets with black highlight (see Additional file 9).

NgeRAG2L_B_0732

MAQLSEERVFSRLPLDDFKFLPFKSYSYDSRKVTRKRAGLELLHDEYYPAEGHCMSAVQGE
NSVIVYFFGGARRKDEATWSMNNIIQEMVVTYDEEDVDIVTVKTLKTTGAEVTGLQSAGFL
AIPNNKCKPDFVIWGGFNMTHLKTENELLHLRYLKKEQKHHCTIFKDPNEKFKLDTDVYQS

```
GEVPSSRTGHSFTQISGTRLVALFGGLEMRFRERSAVTTPTLSPFTQTCQDAHFYVLNLAE
NKWTKLKTPEITARCYHSGTFIEIQGRQTIAIVGGIEYDGSAPCQRFPLDEIIILSFLDLD
TLDVAIQNIVIPLGPDKCFLSYHSCSKLENYLVIFGGFQQYTMELEKTSPSCALHLLDLEL
KQHKKLSAPPVYATAGHSVVIVDNSLMFCGGTEKQYTMFTRKPLVPSACDLGSNCHIVESP
EVSPIQWVQCEAKCNRWLHCFCINLKKIPKGKYICQDCKRETRTGRKK
```

***TSA support GFRY01056743.1 and GFRY01085303.1 (>99% identity DNA sequence)**

(predicted coding regions merging area is underlined as it is less confident)

2. RAGL Detection info

Method: TBLASTN
Database searched: Whole-genome shotgun contigs (WGS) + only in Nemertea clade (taxid:6217).

2.1. RAG1L

Query: NgeRAG1L_D_3820 (previously described)
Subject: NMRB01000732.1 Length: 292569 Number of Matches: 2

Score	Expect	Method	Identities	Positives	Gaps	Frame
104 bits(259)	1e-120	Compositional matrix adjust.	79/250(32%)	125/250(50%)	28/250(11%)	-1

```
Query  123     HSYSGSGPNECGTGTSNGTTNDVMSPQPRKKCRLQ----QVSRHHI---KQRRLKDLISS  175
                S SG  PN   TG S    +   + + +K  +LQ    ++ R  I   K+    +  S
Sbjct  229851  RSISGHNPNSRTTGRSKFPQD---TKKEKKA*KLQKRALELGRSKIFKTKEIEAPNN*SR  229681

Query  176     VGSFCDDRMEDPVDAMYAVLIQKLLDDGRKKEANKILSVWQNKGEELEMSAQDCLAVRVR  235
               +  FC++  E+ +D ++  L   L D   K+++ K+  +W  K + L +  ++CLA RV
Sbjct  229680  INKFCEENKENKIDVLFCTLSITLYDKNEKEKSWKVRDIWLEKMDTLPV--EECLASRVE  229507

Query  236     TLQSKTRYRQQYSLQRDNTNSASLKPPYQLDAAEATFMPGHCAYKL--------MKDSVV  287
                LQSK++YR+QYS  +    +A L+PP +LD  E  FMP    Y L           S+V
Sbjct  229506  NLQSKSQYRRQYSFLKSKGITA-LQPPNKLDEMEFHFMPAAARYSLDVQEKESSFHRSLV  229330

Query  288     IAEQSISTNDQYEPKDIMDQFKNFPTDFPVPNLAGVEFSFVSAVAKTLWELESLIEMRLN  347
                 + + ST   +EP DI+ +F +   +   PN  GV + +  AVAKTL EL+ LI
Sbjct  229329  KIDDAPST---FEPIDILSEFNDNLVELATPNCKGVRWFYPDAVAKTLMELDPLIAKGFT  229159

Query  348     EL----GLPL  353
               +L    G+P+
Sbjct  229158  KLD*IQGIPV  229129
```

Score	Expect	Method	Identities	Positives	Gaps	Frame
354 bits(908)	1e-120	Compositional matrix adjust.	181/464(39%)	279/464(60%)	7/464(1%)	-3

```
Query  354     STQIETIFKDGGDGLGDVCLKTQKADFARTDKIFRYSFCCVSCSAVVGDKKHLLFRETFP  413
```

```
               S  I T  KDG DG+GDV +  + +D    DK FR++F  + C    +  K   +F E  P
Sbjct  229132  SLVIHTTIKDGADGMGDVSVHKESSDRFLPDKAFRFAFAVLKCQIEIDGKLVTVFEEDKP  228953

Query  414     NNTR---PVILCHCDESDRPSISTLLANVSREREMMQDGTLVLVDPDTGDDVRQHHLHFL  470
               N+ R   P++    DE+ + +    +  + +ER +MQ  T+ +   DTG   RQH L F
Sbjct  228952  NSVRTNRPLLEAIGDENSQATSVLCMLPIEQERALMQSNTMKVKVLDTGM-WRQHRLIFY  228776

Query  471     YTMVDEKFERTSSGLQGSGSRFICTLCYAATNTCQADVGSFSRCRTYDETVSLYKYRLEN  530
                +M+DEKF+R   GLQGSGS F+CTLC A   T +   +GSF+  RTY+ET   + ++   N
Sbjct  228775  NSMIDEKFDRAEGGLQGSGSSFLCTLCTAERATAKTCLGSFNITRTYEETRKMAEFIRTN  228596

Query  531     PDKLNENDLAERCKGVNSIPLIKSDATDRSVDSTHANINIGRVFKRILVREIAEIYEWAE  590
               P   ++ +L +  KGV S PL  SDA ++++D+THA+IN+   F++++VREI+ + +W +
Sbjct  228595  PGNRSKAELDKLAKGVKSAPLSTSDAINKALDATHADINLAIFFRKLIVREISTVNKWEK  228416

Query  591     NDRNKKGLQHAEKTNDQHLMKTLGLQSKMIMPGNYARILFLDKNAETVSELIKNPDRKQH  650
                   K  ++ AE   D+H+ KT+GL  ++ MPGNYARI+FL +NA  +  L+ + +RK++
Sbjct  228415  TADVKSMIEDAEAKFDEHVKKTVGLNPQLCMPGNYARIIFLAENANVILHLVPSEERKRY  228236

Query  651     FKCILGLFSKLKAVYSSKDPTSSVPEETACYKENALQFAFHLGEHFNYVPWSNYLHKLIE  710
                  +L +F +L+ +Y +K P +   +E   YKE A++    L  HF+Y PW NYLHK+IE
Sbjct  228235  LAEVLAIFRELRKIYRAKAPKTDFGDEVGQYKEKAVKMGKILIMHFDYAPWPNYLHKVIE  228056

Query  711     HVDEIVQDPRCLHSVGLLSSEGGECGNKLYRFIRRSLSRQDTAGDCDLRDALVFHWIYTS  770
               HV E+++  R   ++G LSSEG E GNK++R IR++LSR+    D  LRD L  HW+++S
Sbjct  228055  HVQELIE--RGPGTIGALSSEGNEAGNKIFREIRKNLSRKGKTSD-GLRDVLWLHWLHSS  227885

Query  771     KCIQKLATSCKAPYNCRKCGQVGHNSRTCDENGDTTTVDQGMDM  814
                 +Q+L++   +   C  C  VGH   TC +   T   D+  DM
Sbjct  227884  PQLQRLSSVTPSKNKCSNCKLVGHKKSTCVQVRATKETDK*WDM  227753
```

2.2 RAG2L

Query: **NgeRAG2L_D_3820 (previously described)**
Subject: **NMRB01000732.1 Length: 292569 Number of Matches: 1**

Score	Expect	Method	Identities	Positives	Gaps	Frame
89.0 bits(219)	7e-17	Compositional matrix adjust.	88/319(28%)	143/319(44%)	36/319(11%)	+3

```
Query  174     IWGGLDPNRMLPHNDLTILENFGRDQFTARLVCQE-------GTKNVQTGTVPEARYGHT  226
               IWGG +    +    N+L  L     ++Q      + ++         T   Q+G VP +R GH+
Sbjct  226302  IWGGFNMTHLKTENELLHLRYLKKEQKHHCTIFKDPNEKFKLDTDVYQSGEVPSSRTGHS  226481

Query  227     LSGLHYQGSLVGAVLYGGVTVK---------NTTQGNEHSTQDGTLYFLDLENYQWKKLS  277
                + +    G+ + A L+GG+ ++            T       + QD    Y L+L    +W KL
Sbjct  226482  FTQI--SGTRLVA-LFGGLEMRFRERSAVTTPTLSPFTQTCQDAHFYVLNLAENKWTKLK  226652

Query  278     --ELAPVAYH--TSCEIRDL-TLVFIGGLSQLCDNRVTSTQRMSIQKITIVE-LNSTIDQ  331
                 E+     YH  T  EI+   T+  +GG+      +      QR  + +I I+  L+
Sbjct  226653  TPEITARCYHSGTFIEIQGRQTIAIVGGIEY---DGSAPCQRFPLDEIIILSFLDLDTLD  226823

Query  332     VTVQNLSLSFLDPALDNVFLSGHSANICSPNQLLIFGGYQQHISDMNTKIPSRTYYLVDI  391
               V +QN+ +   L P  D  FLS HS +    N L+IFGG+QQ+  ++     PS    +L+D+
Sbjct  226824  VAIQNIVIP-LGP--DKCFLSYHSCSKLE-NYLVIFGGFQQYTMELEKTSPSCALHLLDL  226991

Query  392     TSLSIQAKEAPVGFDMAGHTSVRLDHCSLFFHGGANEHLFTMTTKRMELGVCEAEKCIVN  451
                    +   AP  +  AGH+ V +D+ SL F GG  +     T K +    C+      N
```

```
Sbjct  226992  ELKQHKKLSAPPVYATAGHSVVIVDN-SLMFCGGTEKQYTMFTRKPLVPSACDLGS---N  227159

Query  452     DHFSPGEIVTKLQCTLCDA  470
                H       V+ +Q   C+A
Sbjct  227160  CHIVESPEVSPIQWVQCEA  227216
```

WGS: NMRB01005477.1

1. Summary

Detected configuration: RAG1L - RAG2L

Detected loci	Predicted CDS	start	end	strand	Observations
RAG1L	-	4848	6748	+	incomplete; missing middle region
RAG2L	3?	9014	7565	-	complete; potential 3CDS; no stop codons

Selected predicted protein product	
NgeRAG2L_D_5477	Not further used as conflicting protein translation predictions were obtained using different methods and learning sets.

2. RAGL Detection info

Method: TBLASTN
Database searched: Whole-genome shotgun contigs (WGS) + only in Nemertea clade (taxid:6217).

2.1 RAG2L

Query: NgeRAG2L_D_3820 (previously described)
Subject: NMRB01005477.1 Length: 21092 Number of Matches:3

Score	Expect	Method	Identities	Positives	Gaps	Frame
87.0 bits(214)	3e-16	Compositional matrix adjust.	43/113(38%)	70/113(61%)	4/113(3%)	-1

```
Query  17    EFFNNARFYSVLKAVQNRRKVTKKAIVEEASKGALFSGSGHLSWLIFYN----DPRQHTA  72
             + F+++RF+S+ KA++ RRK TKK+++++A   ALFSG GHL WL   +      P
Sbjct  8993  DLFDSSRFFSIRKAIKTRRKTTKKSLMDDAHHMALFSGKGHLLWLTKSDGTAVTPALRAM  8814

Query  73    AVTNNGSGDEKTFSVAVLGGMHAMKPLTNLSGTVYSYAFKVNDDDIKILSVAE  125
                NN + D +  +V+ LGGMH     LT  +GT+ +Y F +  ++I +L+V+E
Sbjct  8813  YFNNNDNIDGELVTVSALGGMHGRLLLTAQAGTIINYTFHMQQNEIHVLNVSE  8655
```

Score	Expect	Method	Identities	Positives	Gaps	Frame
96.7 bits(239)	9e-78	Compositional matrix adjust.	56/129(43%)	78/129(60%)	21/129(16%)	-3

```
Query  107   YSYAFKVNDDDIKILSVAEC---KCSGAQIALRPFVHSACIVETKATRESS--RSRRTGP  161
             +S+A K N       S  EC    K +GAQI LRPF++SA + +T   RE +    +RR
```

```
Sbjct  8700  FSHAAKRN-------SCPECVRKKSTGAQICLRPFLYSAAVFKTVDVREETGTAARRRAH  8542

Query  162   GGARSAMISICSIWGGLDPNRMLPHNDLTILENFGRDQ-FTARLVCQEGTKNVQTGTVPE  220
             GGA     ++  IWGG+DPN +LPHNDLT+L  +GRDQ + A+++        Q G +PE
Sbjct  8541  GGAAGTRTTV-YIWGGMDPNTILPHNDLTVLRTYGRDQDYKAQIM-------DQNGDIPE  8386

Query  221   ARYGHTLSG  229
             ARY HT++G
Sbjct  8385  ARYAHTVTG  8359
```

Score	Expect	Method	Identities	Positives	Gaps	Frame
218 bits(555)	9e-78	Compositional matrix adjust.	128/282(45%)	171/282(60%)	27/282(9%)	-2

```
Query  230   LHYQGSLVGAVLYGGVTVKNTTQGNEHSTQDGTLYFLDLENYQWKKLSELAPVAYHTSCE  289
             +H    L+G V+YGGV  ++++ G    T+DG LYFL LENY+W  L +L P+AYHT+ E
Sbjct  8359  VHKDSRLMGIVMYGGV--QSSSDGGRDKTRDGKLYFLTLENYKWTNLLDLKPLAYHTATE  8186

Query  290   IRDL--TLVFIGGLSQLCDNRVTSTQRMSIQKITIVELNSTIDQ---VTVQNLSLSF---  341
               D   T+VFIGGL+Q  DN V   +R SIQ IT+V L+  I      VTV+    SF
Sbjct  8185  TTDSDGTIVFIGGLTQ-DDNGV---RRQSIQDITMVHLDVDISGQVVVTVRKPDFSFNAE  8018

Query  342   --LDPALDNVFLSGHSANICSPNQLLIFGGYQQHISDMNTKIPSRTYYLVDITSLSIQAK  399
               +      V +SGH+ N    N+LL++GGYQ          P   ++LV+  +LSI  K
Sbjct  8017  RRVGATELPVLISGHTTNYLGSNKLLVYGGYQD-------DRPGGHHFLVNTDTLSITEK  7859

Query  400   EAPVGFDMAGHTSVRLDHCSLFFHGGANEHLFTMTTKRMELGVCEAEKCIVNDHFSPGEI  459
             +AP GF+MA HTS+     ++FFHGGA +HLF +TTKRM+ GVCEA+ CIV +HF+PGE
Sbjct  7858  QAPSGFEMASHTSLLFSPDAVFFHGGALQHLFVLTTKRMDPGVCEAKDCIVKNHFNPGET  7679

Query  460   VTKLQCT-LCDAFFHSCCDQ-GESHTSHSQEDIRFTCPKCRP  499
             +  L C   C  +FH CC   G++H     EDI FTC  CRP
Sbjct  7678  MHSLVCEGPCSKWFHLCCSGLGDAHG--RIEDISFTCSDCRP  7559
```

WGS: NMRB01001167.1

Summary

Detected configuration: RAG1L - RAG2L

Detected loci	Predicted CDS	start	end	strand	Observations
RAG1L	-	58607	48399	-	Pseudogenized (incomplete; stop codons)
RAG2L	-	47440	48263	+	incomplete, missing N-ter; no stop codons

WGS: NMRB01001002.1

Summary

Detected configuration: RAG1L - RAG2L

Detected loci	Predicted CDS	start	end	strand	Observations
RAG1L	-	73242	71971	-	Pseudogenized (incomplete; stop codons)
RAG2L	-	70338	71615	+	incomplete, missing N-ter; no stop codons

WGS: NMRB01000077.1

Summary

Detected configuration: RAG1L - RAG2L

Detected loci	Predicted CDS	start	end	strand	Observations
RAG1L	-	548203	548871	+	Pseudogenized (incomplete; stop codons)
RAG2L	-	551446	550051	-	Pseudogenized (incomplete; stop codons)

WGS: NMRB01001125.1

Summary

Detected configuration: RAG1L - RAG2L

Detected loci	Predicted CDS	start	end	strand	Observations
RAG1L	-	33660	35907	+	Pseudogenized (incomplete; stop codonst)
RAG2L	-	37583	37464	-	Pseudogenized (incomplete; stop codons)

WGS: NMRB01002873.1

Summary

Detected configuration: RAG1L

Detected loci	Predicted CDS	start	end	strand	Observations
RAG1L	-	25294	26654	+	Pseudogenized (incomplete; stop codons)

WGS: NMRB01002576.1

Summary

Detected configuration: RAG1L

Detected loci	Predicted CDS	start	end	strand	Observations
RAG1L	-	56736	53945	-	Pseudogenized (incomplete; stop codons)

-

WGS: NMRB01002346.1

Summary

Detected configuration: RAG1L

Detected loci	Predicted CDS	start	end	strand	Observations
RAG1L	-	17439	18799	+	Pseudogenized (incomplete; stop codons)

WGS: NMRB01002346.1

Summary

Detected configuration: RAG2L

Detected loci	Predicted CDS	start	end	strand	Observations
RAG2L	-	30187	29642	-	Pseudogenized (incomplete)

WGS: NMRB01002346.1

Summary

Detected configuration: RAG2L

Detected loci	Predicted CDS	start	end	strand	Observations
RAG2L	-	25666	26452	+	Pseudogenized (incomplete)

CNIDARIA

Aurelia Aurita (Aau)

(TaxID: 6145; Cnidaria; Scyphozoa; Semaeostomeae; Ulmaridae; Aurelia)

WGS: REGM01000520.1

1. Summary

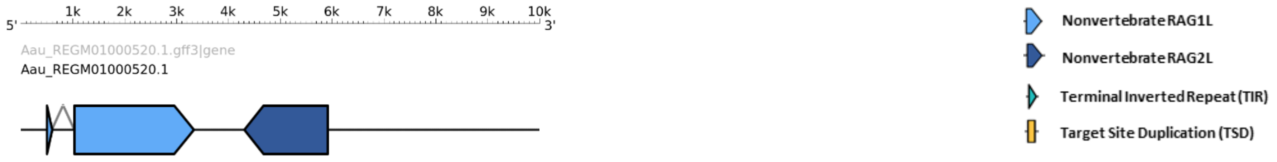

Detected configuration: RAG1L - RAG2L

Detected loci	Predicted CDS	start	end	strand	Observations
RAG1L	1	58496	58612	+	complete; no stop codons
	2	59025	61325	+	
RAG2L	1	63801	62311	-	complete; no stop codons; additional incomplete duplicate at 73811 - 72870

Selected predicted protein product

AauRAG1L_B_0520

MYIEEIKEIWCVDVNSEDEDVQSKHICRACEMKIKRLKRSKAHKKGRTNPPKILNAVPQV
AHNCASCNVCDHDLPRRSPRKANVAQPPSPFASMHAKKELFGSSSFLKEKELLTSTPRQG
MKASFGVDAGDQSPIPQSGESSKANTRGPSKAKIPLFDIADQKGCKKNRLSTVFQEFDDV
CKKQNENKIDVLFFMLRYEMRKNGDVQGAKELDNLWKRSDRQTALTADQCLALRVNTLLS
KGQYKSLYGFFEQNVEKSVLAPPSRLDKTEASYLPGQTEYKILDNEGNIMFHHLKTDPKP
RNALGSFNPMNDGVPTPNAKGVRWCYADAIAQTLKEADSHFDADILTNQSSNDSKIYSVI
KDGADGLGEVSIYKEKDDKFLPDKALRFSFGLVECSCISNGNENLSVYCAENPNSVRINR
PLLECIGDENNRGTLSALLKPIEQEREYLKDKTISVKVSEGHWREHKVRFFNSMIDEKYD
RSMSGYQGAGSNYMCVLCNATQSNCKEGLGTFKIERKVSDNLQLAHYVRINPDSLIEKKM
SETARGVKTIPISMSETKEKLIDATHTDINIGQFFRKLIIRLVAGVTQWESTKDITMILK
NAEQKFDVTMKNLVGINPSMMMPGNYARQLFDQKNEAAIVYLISDEGVKKSLIAVLQKYR
FLRSVYRATKPDKDDIKKYKATAVEMGQLLCKDFSFARWPNYLHKIIEHVQEMLEDPVGP
GSIGAFSSEGNEGGNKLFRLFRNQFSNRGDSYKGLEDVLKLHWLYSSLKLELLAEVDHRK
NRCSKCSELGHNMSSCQTKQTL

AauRAG2L_B_0520

MAHSPVAVIDVEDFKFISFKERSEDTRKLTRKKHLEDIERNEFYPPEGHLMFLMKDFGGD
KKDDEDGDAEMTANFSVRCFYLGGAQNSAGSTWSFASSLQYYTYEVTENDIFIEDAGFVK
TSGSQLPALSKAAFVTSMNEKEETEIFIWGGLDLMQLRPIDDLILLVSKKGHKRKLNAKI
FPSAQNAEIDFSNPLFKNVAVQSGSIPAARFGHTLTQMGDAFIMVGGLSMPNVTNRSFDC
SNIFQLAHPDPVIYNLCLEKKEDEILPKWSILDRNVAVATRTFHTATLVDDKILLIGGAF
FSDGKITRRISIKDAVSLKLGDSDKIEISSLDFPNLDVNIFISNHAAAVSPIHPSHLLIF
GGYQQSTELVSNRHPPSATLYIIDTISKLFLKVAAPQEFATAGHSMSFIDDDSLLICGGT
KQSLSLFTKKAMKPDQCELGDECIIDDKVLTPIPWIKCDGSCKRWLHQFCMQLKTLPKGK
FFCNDCQKPKTQRKKRA

(predicted coding regions merging area is underlined as it is less confident)

2.   RAGL Detection info

Method: TBLASTN
Database searched: Whole-genome shotgun contigs (WGS) + only in Cnidaria group

2.1. RAG1L

Query: **CviRAG1L_B_Biv1_0007 (previously described)**
Subject: **REGM01000520.1   Length: 113995 Number of Matches: 2**

Score	Expect	Method	Identities	Positives	Gaps	Frame
448 bits(1488)	1e-142	Compositional matrix adjust.	302/654(46%)	419/654(64%)	23/654(3%)	+3

```
Query  359    KLPLHSVS-AKHTRHRRLKPIISQVNEFCNAQEENKSDVLFFMLKDHLKEINDPRW-KQV  416
              K+PL  ++  K  +  RL  +  + ++ C  Q ENK DVLFFML+  +++  D +  K++
Sbjct  59376  KIPLFDIADQKGCKKNRLSTVFQEFDDVCKKQNENKIDVLFFMLRYEMRKNGDVQGAKEL  59555

Query  417    ESLWLGNN--STLSPEQCLALRVDLLQSKGQYRSQYDFLSQN-NVHVFQAPSKMESCENL  473
              ++LW  ++  + L+ +QCLALRV+ L SKGQY+S Y F  QN    V   PS+++  E
Sbjct  59556  DNLWKRSDRQTALTADQCLALRVNTLLSKGQYKSLYGFFEQNVEKSVLAPPSRLDKTEAS  59735

Query  474    FMPSASIFQIIDNDGNVLLQN-SENPCTEPLNVNECFLPGFVELATPNCMGVRFSYFEAL  532
              ++P  + ++I+DN+GN++  +   +P  +P N    F P    + TPN  GVR+ Y +A+
Sbjct  59736  YLPGQTEYKILDNEGNIMFHHLKTDP--KPRNALGSFNPMNDGVPTPNAKGVRWCYADAI  59909

Query  533    SLTLQE----LEPEILFGLKKHGLNIEDVLFLTTVKDGCDGMGEVSVYKEKDFKMLPDKV  588
              + TL+E     + +IL     +     D    + +KDG DG+GEVS+YKEKD K LPDK
Sbjct  59910  AQTLKEADSHFDADILTNQSSN-----DSKIYSVIKDGADGLGEVSIYKEKDDKFLPDKA  60074

Query  589    FRFSFCIVKIQAEYDG-KLFDVFTEPLPNSVRTNRPLLESISDENNQVSNVVCILPIENE  647
               RFSF +V+     +G +    V+    PNSVR NRPLLE I DENN+ +    + PIE E
Sbjct  60075  LRFSFGLVECSCISNGNENLSVYCAENPNSVRINRPLLECIGDENNRGTLSALLKPIEQE  60254

Query  648    REILMQNRMHVKTKEG-WMFHKFSFFNSMVDEKRDRGDSGLQGSGSKYLCTLCDADKQSA  706
              RE L    + VK  EG W  HK  FFNSM+DEK DR  SG QG+GS Y+C LC+A + +
Sbjct  60255  REYLKDKTISVKVSEGHWREHKVRFFNSMIDEKYDRSMSGYQGAGSNYMCVLCNATQSNC  60434

Query  707    KALLGSFSINRSVSECSNIAEILRVNPNALSENELKKISKGVKCAPLSKIEPIQKGIDAT  766
              K  LG+F I R VS+   +A  +R+NP++L E ++ + ++GVK  P+S  E  +K IDAT
Sbjct  60435  KEGLGTFKIERKVSDNLQLAHYVRINPDSLIEKKMSETARGVKTIPISMSETKEKLIDAT  60614

Query  767    HADINLGQFFKKIIVREIAGVTKWELTQDVKPLVQNAEFLFDQHMKCNCGINPQLMMPGN  826
              H DIN+GQFF+K+I+R +AGVT+WE T+D+  +++NAE  FD  MK   GINP +MMPGN
Sbjct  60615  HTDINIGQFFRKLIIRLVAGVTQWESTKDITMILKNAEQKFDVTMKNLVGINPSMMMPGN  60794

Query  827    YARTLFETPHSVLLEH-ISDSVRKENLSSILNIFLHLRKVYRCKDPLTECPFDVQNYKKC  885
              YAR LF+  +   + + ISD   K++L ++L  +  LR VYR   P  +   D++ YK
Sbjct  60795  YARQLFDQKNEAAIVYLISDEGVKKSLIAVLQKYRFLRSVYRATKPDKD---DIKKYKAT  60965

Query  886    AVEMGALLLQHFDYVEWPNYLHKVIEHVQQLIEDPNGPGSVGAFSSEGNEAGNKLFRHFR  945
              AVEMG LL + F +  WPNYLHK+IEHVQ+++EDP GPGS+GAFSSEGNE GNKLFR FR
Sbjct  60966  AVEMGQLLCKDFSFARWPNYLHKIIEHVQEMLEDPVGPGSIGAFSSEGNEGGNKLFRLFR  61145

Query  946    KNLSRRGNTYGSLCDVLKLHWLYSSKALFKLAEVEHKKVRCSLCFTSGHNKRTC  999
                 S RG++Y  L DVLKLHWLYSS  L  LAEV+H+K RCS C   GHN  +C
Sbjct  61146  NQFSNRGDSYKGLEDVLKLHWLYSSLKLELLAEVDHRKNRCSKCSELGHNMSSC  61307
```

Score	Expect	Method	Identities	Positives	Gaps	Frame
61.3 bits(184)	5e-07	Compositional matrix adjust.	39/69(57%)	44/69(63%)	0/69(0%)	+1

```
Query  936    AGNKLFRHFRKNLSRRGNTYGSLCDVLKLHWLYSSKALFKLAEVEHKKVRCSLCFTSGHN  995
               G KLFR FR   S RG++Y  L DVLKLHWLYSS  L  LAEV+H+K RCS C    GHN
Sbjct  61681  VGXKLFRLFRNQFSNRGDSYKGLEDVLKLHWLYSSLKLELLAEVDHRKNRCSKCSELGHN  61860

Query  996    KRTCPLLNT  1004
                 +C    T
Sbjct  61861  MSSCQTKQT  61887
```

2.2 RAG2L

Query: **CviRAG2L_B_Biv1_0007 (previously described)**
Subject: **REGM01000520.1 Length: 113995 Number of Matches: 1**

Score	Expect	Method	Identities	Positives	Gaps	Frame
131 bits(480)	3e-32	Compositional matrix adjust.	126/487(26%)	255/487(52%)	75/487(15%)	-2

```
Query  7      QFRALPFKSHSTNPLRRITRKS--AASDDFNFFPPEGHVSLPINSYTY------------  52
              +F+ ++FK +S +  R++TRK      +  +F+PPEGH+ + ++ +
Sbjct  63765  DFKFISFKERSEDT-RKLTRKKHLEDIERNEFYPPEGHLMFLMKDFGGDKKDDEDGDAEM  63589

Query  53     --------LYFGGARRGQESTWNMSRNI--YKisfivddndvnvdfisEIKLSGGQFPQL  102
                      +Y+GGA+ +  STW+ + ++  Y      +D  ++   +  +K SG+Q+P L
Sbjct  63588  TANFSVRCFYLGGAQNSAGSTWSFASSLQYYTYEVTENDIFIEDAGF--VKTSGSQLPAL  63415

Query  103    QSSA---GFYVSAENCLFVWGGLNLSCFSMSNELYIV--KLNDNKGVVEIIQPPGGISVR  157
                +A   +     E  +F+WGGL+L  +   ++L ++  K  +++ +   I P++  +
Sbjct  63414  SKAAFVTSMNEKEETEIFIWGGLDLMQLRPIDDLILLVSKKGHKRKLNAKIFPSAQNAEI  63235

Query  158    ELG-----------GEIPSGRCGHTLTHYFDSCVILHGGVCFPHRNS----CVGSSLFKN  202
              +++           G IP++R GHTLT+  +   I+ GG+  P+  +    C  S++F+
Sbjct  63234  DFSNPLFKNVAVQSGSIPAARFGHTLTQ-MGDAFIMVGGLSMPNVTNRSFDC--SNIFQL  63064

Query  203    VTNDNNFYMF----DFESLF--WTKLS-VTGSAPRAYHTANVMEIRGMKSIVYIGGVTKT  255
                 D  +Y +      + ++  W+ L    + A R++HTA +++ +    I+ IGG   +
Sbjct  63063  AHPDPVIYNLCLEKKEDEILPKWSILDRNVAVATRTFHTATLVDDK----ILLIGGAFFS  62896

Query  256    ESAL-QRIPLSNVLVLKMDSNKHFHTEILTFAN-APAVGISYHSVGVIG---PYIFVVGG  310
              ++ + +RI++ + + LK++ +  ++   L F+N    + IS H+ +V     + +++ GG
Sbjct  62895  DGKITRRISIKDAVSLKLGDSDKIEISSLDFPNLDVNIFISNHAAAVSPIHPSHLLIFGG  62716

Query  311    ------LDEDNLQGRCSVSILNKDTFLCENVQFDRYFRSAGHSVCTLSDDCLMICGGMNL  364
                    L  +      ++ I++  + L  +V   + F +AGHS+  + DD L+ICGG +
Sbjct  62715  YQQSTELVSNRHPPSATLYIIDTISKLFLKVAAPQEFATAGHSMSFIDDDSLLICGGTKQ  62536

Query  365    QYFVFSSKQMVPSPCDFDTECKIIESVETSPISWIQCEGACKRWLHQFCVGVLDIDMSRK  424
                 +F+ K M P  C+++ EC II+    +PI+WI+C+G+CKRWLHQFC+ +    +++
Sbjct  62535  SLSLFTKKAMKPDQCELGDEC-IIDDKVLTPIPWIKCDGSCKRWLHQFCMQL--KTLPKG  62365

Query  425    NFICTTC  431
              +F+C  C
Sbjct  62364  KFFCNDC  62344
```

Aurelia aurita complex sp. Pacific isolate jellyfish_7

WGS: REGL01002183.1

Summary

Detected configuration: RAG1L

Detected loci	Predicted CDS	start	end	strand	Observations
RAG1L	-	2755	4645	+	Almost complete; 1 stop codon at N-term

RAGL Detection info

Method: TBLASTN
Database searched: Whole-genome shotgun contigs (WGS) + only in Cnidaria group

2.1. RAG1L

Query: **AauRAG1L_0520 (previously described)**
Subject: **REGL01002183.1 Length: 48093 Number of Matches: 3**

Score	Expect	Method	Identities	Positives	Gaps	Frame
312 bits(1170)	5e-142	Compositional matrix adjust.	168/315(53%)	245/315(77%)	2/315(0%)	+2

```
Query  470   FFNSMIDEKYDRSMSGYQGAGSNYMCVLCNATQSNCKEGLGTFKIERKVSDNLQLAHYVR  529
             +F  M+DEK DR++SG QG+GS YMC LC+AT + CKE LGTF I R + +  +LA+YV
Sbjct  3707  YF--MLDEKHDRAISGLQGSGSRYMCNLCHATTALCKEKLGTFTICRTLEETRNLARYVI  3880

Query  530   INPDSLIEKKMSETARGVKTIPISMSETKEKLIDATHTDINIGQFFRKLIIRLVAGVTQW  589
             +NPD+L EK +S   +GVK++P+S S+ +EKLIDATH+DIN+ +FF+KLI+R  A V QW
Sbjct  3881  VNPDNLSEKSLSAKVKGVKALPFSTSDPNEKLIDATHSDINLVRFFKKLIVRESAEVHQW  4060

Query  590   ESTKDITMILKNAEQKFDVTMKNLVGINPSMMMPGNYARQLFDQKNEAAIVYLISDEGVK  649
             E  ++ +  LKNAE+K++  ++  +GINP ++MPGNYAR++F  +N++A++ +I DE+ K
Sbjct  4061  EERQENSPYLKNAEKKLNDHLRAKIGINPQLLMPGNYARHMFSPQNQGAFLHFIDDESRK  4240

Query  650   KSLIAVLQKYRFLRSVYRATKPDKDDIKKYKATAVEMGQLLCKDFSFARWPNYLHKIIEH  709
               L  +L+ +++LR VYRATKP K+++    K  AV MG LL K+F++A WPNYLHKI+EH
Sbjct  4241  DKLGEILDIFQILREVYRATKPTKEQVSTCKEKAVKMGSLLKKNFPYANWPNYLHKIMEH  4420

Query  710   VQEMLEDPVGPGSIGAFSSEGNEGGNKLFRLFRNQFSNRGDSYKGLEDVLKLHWLYSSLK  769
             VQE+LED  GPGSIG+FS+EGNE+GNK+FRLF+++FS R++SYK+LED+++LHW+YSS
Sbjct  4421  VQELLEDHNGPGSIGSFSGEGNEAGNKIFRLFKKNFSTRSNSYKALEDIIRLHWMYSSKT  4600

Query  770   LELLAEVDHRKNRCS  784
             L+  ++V+    + CS
Sbjct  4601  LQDHTHVEPAVHICS  4645
```

Score	Expect	Method	Identities	Positives	Gaps	Frame
197 bits(730)	5e-142	Compositional matrix adjust.	121/263(46%)	188/263(71%)	12/263(4%)	+3

```
Query  193   FFMLRYEMRKNGDVQGAKELDNLW-KRSDRQTALTADQCLALRVNTLLSKGQYKSLYGFF  251
             FFML  E+ ++    + +KE++NLW K ++++T L+A+Q L+LRV+ LL+K     +Y+++
```

```
Sbjct  2907  FFMLISELERSNPSK-SKEVQNLWTKTGNKETCLSAEQSLGLRVDFLLTK-----VYAIL  3068

Query  252   EQNVEKSVLAPPSRLDKTEASYLPGQTEYKILDNEGNIMFHHLKTDPKPRNALGSFNPMN  311
             E+++  +V+ PPS+L+  E  +LPG  E++++ N   +  HH   +PKP N +++F+ M+
Sbjct  3069  ENKIAANVVKPPSQLNEKEKEFLPGSVEFQVISNGNVLHHHHGEDEPKPINIISTFSEMS  3248

Query  312   DGVPTPNAKGVRWCYADAIAQTLKEADSHFDADILTNQSSNDSKIYSVIKDGADGLGEVS  371
             +  P PN KGVRW YA+A+AQ+LKE  + +D +I + Q    + I +++KDG+DGLGEVS
Sbjct  3249  HESPVPNIKGVRWNYAQALAQSLKEIYPFIDENIKSLQIDTPGIIKTIVKDGGDGLGEVS  3428

Query  372   IYKEKDDKFLPDKALRFSFGLVECSCISNGNENLSVYCAENPNSVRINRPLLECIGDENN  431
             +YKEK D+ LPDKA+RFSF +V  S +  G++   VY   NPNSVR+NRPLLE I+DENN
Sbjct  3429  VYKEKADRMLPDKAFRFSFCIV--SYMQGGQN---VYEEKNPNSVRLNRPLLESIADENN  3593

Query  432   RGTLSALLKPIEQEREYLKDKTI  454
             +++L A L+PI  ER+YLK+K I
Sbjct  3594  KASLVACLRPIGLERQYLKGKII  3662
```

Score	Expect	Method	Identities	Positives	Gaps	Frame
45.6 bits(153)	5e-142	Compositional matrix adjust.	21/53(40%)	39/53(73%)	0/53(0%)	+1

```
Query  142   SKANTRGPSKAKIPLFDIADQKGCKKNRLSTVFQEFDDVCKKQNENKIDVLFF  194
             S+   R P++ K+PL D+ D+ +C+K++L+  F+ ++ +C KQ+ENK+ VLF+
Sbjct  2755  SRIMER*PGQKKMPLSDVKDKTDCRKKKLAPFFESLNTLCEKQHENKMIVLFL  2913
```

WGS: REGL01004151.1

Summary

Detected configuration: RAG1L

Detected loci	Predicted CDS	start	end	strand	Observations
RAG1L	-	1794	4522	+	incomplete; stop codon

RAGL Detection info

Method: TBLASTN
Database searched: Whole-genome shotgun contigs (WGS) + only in Cnidaria group

2.1. RAG1L

Query: **AauRAG1L_0520 (previously described)**
Subject: **REGL01004151.1 Length: 11166 Number of Matches: 5**

Score	Expect	Method	Identities	Positives	Gaps	Frame
170 bits(628)	2e-43	Compositional matrix adjust.	103/193(53%)	141/193(73%)	5/193(2%)	+1

```
Query  360   IKDGADGLGEVSIYKEKDDKFLPDKALRFSFGLVECSCISNGNENLSVYCAENPNSVRIN  419
             +KDG+DGLGE+S+YKEK D+ LPDKA+RFSF +V     S+ +    VY   NPNSVR N
```

```
Sbjct  3871   FKDGGDGLGEMSVYKEKADRMLPDKAFRFSFCIV-----SSMQGGQHVYEEKNPNSVRSN  4035

Query  420    RPLLECIGDENNRGTLSALLKPIEQEREYLKDKTISVKVSEGHWREHKVRFFNSMIDEKY  479
              +PLLE I+DENN+++L A L+PIE ER+YLK+K I VKV+E++WR H ++F+NSM+DE+
Sbjct  4036   QPLLESIADENNKAALVACLRPIESERQYLKGKIIKVKVGEDQWRMHTLQFYNSMLDENH  4215

Query  480    DRSMSGYQGAGSNYMCVLCNATQSNCKEGLGTFKIERKVSDNLQLAHYVRINPDSLIEKK  539
              DR++SG QG+GS YMC LC+AT + CKE LGTF I R + +  +LA+Y  +NPD L
Sbjct  4216   DRAISGLQGSGSRYMCNLCHATTALCKEKLGTFTICRTLEETRNLARYAIVNPDKLEREV  4395

Query  540    MSETARGVKTIPI  552
                  + G ++ PI
Sbjct  4396   TVSKSEGCQSPPI  4434
```

Score	Expect	Method	Identities	Positives	Gaps	Frame
62.9 bits(219)	2e-14	Compositional matrix adjust.	33/73(45%)	58/73(79%)	2/73(2%)	+2

```
Query  193    FFMLRYEMRKNGDVQGAKELDNLW-KRSDRQTALTADQCLALRVNTLLSKGQYKSLYGFF  251
              FFML  E+ K+++   +KE+ NLW K ++++T L+A+Q L+LRV++LL+KGQYK +Y+++
Sbjct  1913   FFMLISEL-KHSNPSKSKEVLNLWTKTGNKETCLSAEQSLGLRVDSLLTKGQYKKVYAIL  2089

Query  252    EQNVEKSVLAPPS  264
              E+++  +V+ PPS
Sbjct  2090   ENKIAANVVKPPS  2128
```

Porites rus(Pru)

WGS: OKRP01001303.1

1.Summary

Detected configuration: RAG1L - RAG2L

Detected loci	Predicted CDS	start	end	strand	Observations
RAG1L	-	86887	79004	-	Large assembly gaps
RAG2L	-	77540	78888	+	Large assembly gaps

2. RAGL Detection info

Method: TBLASTN
Database searched: Whole-genome shotgun contigs (WGS) + only in Cnidaria group

2.1. RAG1L

Query: **CviRAG1L_B_Biv1_0007 (previously described)**
Subject: **OKRP01001303.1 Length: 114984 Number of Matches: 1**

Score	Expect	Method	Identities	Positives	Gaps	Frame
97.0 bits(349)	7e-28	Compositional matrix adjust.	59/184(32%)	108/184(58%)	8/184(4%)	-2

```
Query  311    IYETLLVHCIHA--NCTQSHLIRNIDEHESICSMKGTKFLYNVTTKSRVFKLPLHSVSAK  368
              ++E L V C  +   CT+      + E+E+ C     K       ++   K+ L  +S +
Sbjct  86369  VFELLTVKCKYSCNGCTEVLKGSLLTEREAHCKFSHIK----HKKRGPYNKVKLYDISRQ  86202

Query  369    HTRHRRLKPIISQVNEFCNAQEENKSDVLFFMLKDHLKEINDPRW-KQVESLWLGNNS-T  426
               ++ RRL P++S ++EFC+ ++EN  DVLF ML   L +       +++E LW+ +
Sbjct  86201  YAKGRRLLPLFSMLDEFCDLNNENTEDVLFSMLATTLYDNGKKELANKIEYLWVQQTDYI  86022

Query  427    LSPEQCLALRVDLLQSKGQYRSQYDFLSQNNVHVFQAPSKMESCENLFMPSASIFQIIDN  486
              L++++CLA RVDLLQ+K QYR Q+D+L+ +   VF+ P++++S E+ ++PS   ++++D
Sbjct  86021  LTADECLASRVDLLQTKNQYREQFDYLNSKGQYVFKLPNQVDSVEKTYLPSYVNYELLDG  85842

Query  487    DGNV  490
              D  +
Sbjct  85841  DKVI  85830
```

Score	Expect	Method	Identities	Positives	Gaps	Frame
55.3 bits(190)	7e-28	Compositional matrix adjust.	29/86(34%)	53/86(61%)	1/86(1%)	-3

```
Query  226    TAPEMFKNVVIRSIPTERFCNAEVSQSFMCTICRGVPCDPYI-SKCSHIFCKECIFGWFS  284
              T    ++NV + +I  +RF + + +Q F CT+C +VP ++ I + C H+FC  CI   ++
Sbjct  86626  TDTKSLNNVELTGIHLDRFVERDFAQIFQCTVCLDVPSNAVILAGCRHVFCEPCIRQ*LT  86447

Query  285    LSSACPVCRSLLDESEVSPLHGHLLQ  310
              +S+ CP C  + D  ++SPL  ++L+
Sbjct  86446  FSGVCPSCGQVTDLEDISPLKKQMLE  86369
```

**Score**	**Expect**	**Method**	**Identities**	**Positives**	**Gaps**	**Frame**
153 bits(563)	2e-36	Compositional matrix adjust.	93/206(45%)	143/206(69%)	0/206(0%)	-3

```
Query  598    IQAEYDGKLFDVFTEPLPNSVRTNRPLLESISDENNQVSNVVCILPIENEREILMQNRMH  657
              ++ E +G +  +  E  P SVRTNR L+ESI DEN+  + V C+ P+E ER  + ++ M+
Sbjct  82579  VKMEDGGEMKTIWEEEAPGSVRTNRCLIESICDENQTAAMVACVAPVEAERVEMSKSIMR  82400

Query  658    VKTKEGWMFHKFSFFNSMVDEKRDRGDSGLQGSGSKYLCTLCDADKQSAKALLGSFSINR  717
              ++    W    + F NSMVDEK  R+D+GLQG+GS YLC LC A +++AK+ +G+F I+R
Sbjct  82399  IQIGSIWRNFSLRFVNSMVDEK*TRADGGLQGAGSSYLCDLCYATQKTAKSDIGTFVISR  82220

Query  718    SVSECSNIAEILRVNPNALSENELKKISKGVKCAPLSKIEPIQKGIDATHADINLGQFFK  777
               + E  +IA++L+ NP+ L+ ++L  ++KGVK  P+ +IE  +  +DATHA INLG+F
Sbjct  82219  KLEETKQIADLLHFNPDKLNPSQLSAVAKGVKTHPILNIEHSEHKVDATHAKINLGKFVY  82040

Query  778    KIIVREIAGVTKWELTQDVKPLVQNA  803
              K+++RE+AGV +WE + DVK L+QNA
Sbjct  82039  KLLIREVAGVNQWEESADVKHLIQNA  81962
```

**Score**	**Expect**	**Method**	**Identities**	**Positives**	**Gaps**	**Frame**
152 bits(560)	4e-36	Compositional matrix adjust.	92/196(47%)	140/196(71%)	8/196(4%)	-3

```
Query  808    DQHMKCNCGINPQLMMPGNYARTLFETPHS---VLLEHISDSVRKENLSSILNIFLHLRK  864
              DQH K   GINP LMMPGNYAR LF+  +    V+L  ++   +K+N++ +L+ +  ++K
Sbjct  79591  DQHFKATIGINPCLMMPGNYARILFDPQNESKVVFL--LGTEEKKNNFTELLSKLQFMHK  79418

Query  865    VYRCKDPLTECPFDVQNYKKCAVEMGALLLQHFDYVEWPNYLHKVIEHVQQLIEDPNGPG  924
              ++ C+ P    P + ++YK+ AVE G +LL+++ Y  W+NY+HK IEHVQ++IE  +
Sbjct  79417  IFSCRSPKLKYPSEWNHYKQVAVEFGNILLNNYPYARWSNYVHKCIEHVQEVIETDD---  79247

Query  925    SVGAFSSEGNEAGNKLFRHFRKNLSRRGNTYGSLCDVLKLHWLYSSKALFKLAEVEHKKV  984
              ++ ++S+EGNEAGNK+FRH+RKN SR+G+T  S+ DVLK+HWLY S+ L KL++V ++K
Sbjct  79246  TLVGLSGEGNEAGNKIFRHLRKNHSRKGSTLHSVTDVLKVHWLYCSRKLRKLSHVSRRKY  79067

Query  985    RCSLCFTSGHNKRTCP  1000
              +CSLC   GHN  +CP
Sbjct  79066  KCSLCNQMGHNYMSCP  79019
```

2.2 RAG2L

Query: **CviRAG2L_B_Biv1_0007 (previously described)**
Subject: **OKRP01001303.1 Length: 114984 Number of Matches: 4**

**Score**	**Expect**	**Method**	**Identities**	**Positives**	**Gaps**	**Frame**
102 bits(371)	2e-22	Compositional matrix adjust.	108/433(25%)	221/433(51%)	64/433(14%)	+2

```
Query  55     FGGARRGQESTWNMSRNIYK---isfivddndvnvdfisEIKLSGGQFPQLQSSA--GFY  109
              FGGAR+ + STW+++  +          D +  +   I+  ++ G+ +  LQ+S+   F
Sbjct  77591  FGGARQTEPSTWELGNALTILQCSVSKDDVEVKSCVVITAERIRGSTLYPLQGSTMVNFL  77770

Query  110    VSAENC-------LFVWGGLNL---SCFSMSNELYIVKLNDNK----------GVVEIIQ  149
                 ++        +F+WGGLN+   +C S   EL + K  ++K          +  + ++
Sbjct  77771  DKDNENPNNDEYFIFIWGGLNIKTITCTSELVELKVSKTGESKRTRTGRKCTNASNDKFK  77950

Query  150    PP---------GGISVRELG--------GEIPSGRCGHTLTHYFDSCVILHGGVCFPHRN  192
              +P         +G+  R LG        G IPS+R GH+LT   D   +L GGV   +R+
Sbjct  77951  SPSFSVDATIFAGLEARVLGLSQINKQTGAIPSARTGHSLTMVSDHYAVLFGGVEMEQRD  78130

Query  193    SCVGSSLFKNVT-NDNNFYMFDFESLFWTKLSVTGSA---PRAYHTANVMEIRGMKSIVY  248
              +    ++FK+ T +D ++Y++D+    W+ L+ T ++    RAYHTA        +++
Sbjct  78131  N----GVFKQQTCKDGELYILDLKRKNWSSLASTNGGKLPTRAYHTAPFGSTS--NTLFI  78292

Query  249    IGGVTKTESALQRIP---LSNVLVLKMDSN-KHFHTEILTFANAPAVGISYHSVGVIGPY  304
              IGGV   ++ ++ +    +++V  +K +SN   F  E++ ++ + ++ +S H+  +
Sbjct  78293  IGGVQVPNPEINSVQYCNVGKVTTIKFESNFTSFTHEVIDITVSTPLFLSSHASCLSENI  78472

Query  305    IFVVGGLDEDN------LQGRCSVSILNKDTFLCENVQFDRYFRSAGHSVCTLSDDCLMI  358
              + + GG +  +       +   ++ +++ +  +  + +      +AG S+  LS+  ++I
Sbjct  78473  VHIFGGYQSKEEITTVKPETSKTMLLIDVEGRVAMKKDASEIHATAGLSLIKLSNSAFLI  78652

Query  359    CGGMNLQYFVFSSKQMVPSPCDFDTECKIIESVETSPISWIQCEGACKRWLHQFCVGVLD  418
              CGG N + ++F+S    + +CD++  C I  +  T P++W++C+G CKRW+HQ C G+
Sbjct  78653  CGGTNKEILLFTSLMPAADACDLNDKCIINTTGVTFPFPWLKCDGICKRWIHQACSGITI  78832

Query  419    IDMSRKNFICTTC  431
              I  ++ +++C  C
Sbjct  78833  I--PKDKYFCKDC  78865
```

Orbicella faveolata(Ofa)

WGS: MZGG01001906.1

1.Summary

Detected configuration: RAG1L - RAG2L

Detected loci	Predicted CDS	start	end	strand	Observations
RAG1L	-	95919	95410	-	Large assembly gaps; stop codon
RAG2L	-	82771	86830	+	Large assembly gaps; stop codon

2. RAGL Detection info

Method: TBLASTN
Database searched: Whole-genome shotgun contigs (WGS) + only in Cnidaria group

2.1. RAG1L

Query: **AauRAG1L_0520 (previously described)**
Subject: **MZGG01001906.1 Length: 317569 Number of Matches: 2**

Score	Expect	Method	Identities	Positives	Gaps	Frame
69.2 bits(243)	1e-19	Compositional matrix adjust.	58/129(45%)	81/129(62%)	0/129(0%)	-2

```
Query  467    KVRFFNSMIDEKYDRSMSGYQGAGSNYMCVLCNATQSNCKEGLGTFKIERKVSDNLQLAH  526
              +V F NSM DEK  R+ SG QGAGS+++  LC ATQ      LGTFKI    + D  Q+A+
Sbjct  95046  NVTFVNSMKDEKRARTDSG*QGAGSKFLRDLCYATQETAISELGTFKICCTLEDTKQIAD  94867

Query  527    YVRINPDSLIEKKMSETARGVKTIPISMSETKEKLIDATHTDINIGQFFRKLIIRLVAGV  586
               + +N D L   +++  A+GVKT +    E  E  +DATHT IN+G+F  +L+ + +AGV
Sbjct  94866  LLYFNLDKLTLTQLAALAKGVKTHSTLNIEHSECKVDATHTRINLGKFCYNLLTQEIAGV  94687

Query  587    TQWESTKDI  595
               QW+ T DI
Sbjct  94686  IQWNKTSDI  94660
```

Score	Expect	Method	Identities	Positives	Gaps	Frame
54.7 bits(188)	1e-19	Compositional matrix adjust.	34/108(31%)	64/108(59%)	5/108(4%)	-1

```
Query  600    KNAEQKFDVTMKNLVGINPSMMMPGNYARQLFDQKNEAAIVYLISDEGVKKSLIAVLQKY  659
              ++A +K++   K+ ++INP  + PGN AR LF Q N + I+  +  E  K ++  +L
Sbjct  94648  QQATNKLNTHRKQTIAINPCFLPPGNCARILFTQTNAPHILHPLQSEEKKPNFSNLLLSV  94469

Query  660    RFLRSVYRATKPDKDDIKK----YKATAVEMGQLLCKDFSFARWPNYL  703
              RF+  ++ + KP K+++      YK+  +E+G  L   +S+ARW++Y+
Sbjct  94468  RFMNKLFSSRKP-KEEFPDEWYLYKSRVIELGCQLTLYYSYARWSSYV  94328
```

2.2 RAG2L

Query: **AauRAG2L_0520 (previously described)**
Subject: **MZGG01001906.1 Length: 317569 Number of Matches: 1**

Score	Expect	Method	Identities	Positives	Gaps	Frame
64.7 bits(226)	1e-13	Compositional matrix adjust.	52/174(30%)	103/174(59%)	6/174(3%)	+3

```
Query  290    DDKILLIGGAFFSDGKITRRISIKDAVSLKLG-DSDKIEISSLDFPNLDVNIFISNHAAA  348
              +D + +IGG  + D+    +   +I +   ++++   D+++I   S   + P       ++S++AA+
Sbjct  86223  GDTLYIIGGVQLQDDSAVQYWNITEICAVQFNLDTNQIVSSVCEVPT-STPQYLSSQAAT  86399

Query  349    VSPIHPSHLLIFGGYQQSTELVSNRHPPSATLYIIDTISKLFLKVAAPQEFATAGHSMSF  408
              +S     + + +F+GY       ++ +    S TL+ ++     ++L+ +AP    ATA H+M
Sbjct  86400  ISD---NMVYVFDGYMAKDRSITTKPNVS*TLFAFGLDKLVYLNKTAPAMHATACHNMFA  86570

Query  409    IDDDSLLICGGTKQSLSLFTKKAMKPDQCELGDECIIDD-KVLTPIPWIKCDGS  461
              ++   +LLICGGTK   + +FTK    ++D   +LGD C I+   +V+ +IPW+KC G+
Sbjct  86571  LGSSTLLICGGTKMEIVMFTKLLPQADLRDLGDKCQINVFNVVYSIPWLKCVGP  86732
```

WGS: MPSW01001994.1

Summary

Detected configuration: RAG1L - RAG2L

Detected loci	Predicted CDS	start	end	strand	Observations
RAG1L	-	80657	80323	-	Large assembly gaps
RAG2L	-	79104	80093	+	Large assembly gaps; stop codon

WGS: MPSW01002212.1

Summary

Detected configuration: RAG1L - RAG2L

Detected loci	Predicted CDS	start	end	strand	Observations
RAG1L	-	44102	45693	+	Large assembly gaps; stop codon
RAG2L	-	53914	53191	-	Large assembly gaps; stop codon

Almost identical with MZGG01001906.1

WGS: MZGG01001116.1

Summary

Detected configuration: RAG1L - RAG2L

Detected loci	Predicted CDS	start	end	strand	Observations
RAG1L	-	212881	212296	-	Large assembly gaps
RAG2L	-	211076	212065	+	Large assembly gaps; stop codon

Almost identical with MPSW01001994.1

Nematostella vectensis (Nve)

WGS: ABAV01022117.1

1.Summary

Detected configuration: RAG1L

Detected loci	Predicted CDS	start	end	strand	Observations
RAG1L	-	1972	647	-	incomplete; stop codon

2.RAGL Detection info

Method: TBLASTN
Database searched: Whole-genome shotgun contigs (WGS) + only in Cnidaria group

2.1. RAG1L

Query: **AauRAG1L_0520 (previously described)**
Subject: **ABAV01022117.1 Length: 8883 Number of Matches: 1**

Score	Expect	Method	Identities	Positives	Gaps	Frame
258 bits(963)	4e-74	Compositional matrix adjust.	175/447(39%)	285/447(63%)	20/447(4%)	-3

```
Query  133   SPIPQSGESSKANTRGPSKAKIPLFDIADQKGCKKNRLSTVFQEFDDVCKKQNENKIDVL  192
              P  ++  + K +TR ++  K PL+ ++ + + +++RL  ++  ++  CK Q E+K DVL
Sbjct  1972  CPARHGKLPRKKETRTSNPFKEPLISVTAKYA-RQKRLQGIIGMLNSFCKEQFEDKGDVL  1796

Query  193   FFMLRYEMRKNGDVQGAKELDNLWKRSDRQTALTADQCLALRVNTLLSKGQYKSLYGFFE  252
             FF+L  ++R   D +++  +D L +R     +LT+DQCLALRV+TL +KGQYK+ Y+F+
Sbjct  1795  FFLLCQHLRDTQDERASS-IDGL*QRLS--YSLTTDQCLALRVDTLQTKGQYKAQYDFLK  1625

Query  253   QNVEKSVLAPPSRLDKTEASYLPGQTEYKILDNEGNIMFHH------------LKTDPK-  299
             Q++  +VL PP  +  +E +Y+PG   Y +  ++  + + H            + +DP
Sbjct  1624  QKLAITVLKPPLAVSESELAYMPGSVRYMVEAEDPLLSYYHTPV*QSGPVASGFSADPSC  1445

Query  300   -PRNALGSFNPMNDGVPTPNAKGVRWCYADAIAQTLKEADSHFDADILTNQSSNDSKIY-  357
              P N L  FN      PTPN KGVR  ++DA+A+TL E D+   + +  ++    + +
Sbjct  1444  EPINILQDFNDSIPEFPTPNVKGVRFRFTDALAKTLEELDPIVKSKLTEHNLDPATTLLK  1265

Query  358   SVIKDGADGLGEVSIYKEKDDKFLPDKALRFSFGLVECSCISNGNENLSVYCAENPNSVR  417
             + +K+GADG+G+VS+YKE +D+FL DKA+R SF+++    ++NGN  ++++  ++PNSVR
Sbjct  1264  TTVKNGADGMGDVSVYKETGDRFLHDKAFRHSFAVIKIEALTNGN-VIPLFLEDEPNSVR  1088

Query  418   INRPLLECIGDENNRGTLSALLKPIEQEREYLKDKTISVKVSEGHWREHKVRFFNSMIDE  477
              NRPLLE I+DENN ++    L P E+E+E+LK+K + ++V  G+WR H+V F NSMIDE
Sbjct  1087  TNRPLLEAIADENNNASSCVCLIPSEEEKEFLKGKQLKIQVDAGEWRTHQVVFHNSMIDE  908

Query  478   KYDRSMSGYQGAGSNYMCVLCNATQSNCKEGLGTFKIERKVSDNLQLAHYVRINPDSLIE  537
             K DR+ +G  G+GS Y+C LC+AT+   K  +G+F I R   +N ++A+YVR NPD+L
Sbjct  907   KLDRAEGGLAGSGSRYLCTLCKATRETAKREIGSFSICRTYQENAEIAEYVRTNPDNLSK  728

Query  538   KKMSETARGVKTIPISMSETKEKLIDA  564
              ++    +GVK+ PI  S++ EK IDA
Sbjct  727   EQLDTLEQGVKSAPILKSDAIEKGIDA  647
```

Pocillopora damicornis (Pda)
WGS: RCHS01004171.1

Summary

Detected configuration: RAG1L

Detected loci	Predicted CDS	start	end	strand	Observations
RAG1L	-	81	1671	+	N-term on a different scaffold ?

RAGL Detection info

Method: TBLASTN
Database searched: Whole-genome shotgun contigs (WGS) + only in Cnidaria group

2.1. RAG1L

Query: **AauRAG1L_0520 (previously described)**
Subject: **RCHS01004171.1 Length: 1840Number of Matches: 3**

Score	Expect	Method	Identities	Positives	Gaps	Frame
134 bits(490)	6e-59	Compositional matrix adjust.	77/169(46%)	119/169(70%)	0/169(0%)	+2

```
Query  484   SGYQGAGSNYMCVLCNATQSNCKEGLGTFKIERKVSDNLQLAHYVRINPDSLIEKKMSET  543
             +G QGAGS +MC LC ATQ + K +LGTFK  R + +  Q+A  +++NP+ L + ++S
Sbjct  728   GGLQGAGSRFMCDLCYATQDSAKSDLGTFKTCRTLEETKQIAALLHFNPNKLTQSQLSAV  907

Query  544   ARGVKTIPISMSETKEKLIDATHTDINIGQFFRKLIIRLVAGVTQWESTKDITMILKNAE  603
             A+GVKT P+   E  E  +DATH+ IN+G+F  KL+IR +AGVT+W+ T DI   + +A
Sbjct  908   AKGVKTHPVLNIEHAEHKVDATHARINLGKFCYKLLIREIAGVTRWNETSDIKHHIEQAT  1087

Query  604   QKFDVTMKNLVGINPSMMMPGNYARQLFDQKNEAAIVYLISDEGVKKSL  652
             +++++ +K+ +GINP +M+PGNYAR LF QKNE+ I+YL+  +   + +L
Sbjct  1088  NQLNIHLKKTIGINPCLMLPGNYARILFSQKNESHILYLLKSDEKRENL  1234
```

Score	Expect	Method	Identities	Positives	Gaps	Frame
121 bits(443)	6e-59	Compositional matrix adjust.	63/153(41%)	108/153(70%)	6/153(3%)	+1

```
Query  649   KKSLIAVLQKYRFLRSVYRATKPDKD---DIKKYKATAVEMGQLLCKDFSFARWPNYLHK  705
             K+ +  +L   RF++ ++ ++KP  +   +  +YK+ AVE G+ L   +++ARW NY+HK
Sbjct  1222  KRKFAKLLTLVRFMHKIFSSSKPKEEFPEEWSQYKSRAVEFGKHLIASYPYARWGNYVHK  1401

Query  706   IIEHVQEMLEDPVGPGSIGAFSSEGNEGGNKLFRLFRNQFSNRGDSYKGLEDVLKLHWLY  765
              IE VQE++E    + G++G+FS+EGNE+GNK+F+ +R++ S + +++ ++ DVLK+HWLY
Sbjct  1402  TIEYVQEVIE---SHGTLGGFSGEGNEAGNKIFQHLRKNHSRKTGTFESVSDVLKMHWLY  1572

Query  766   SSLKLELLAEVDHRKNRCSKCSELGHNMSSCQT  798
              S+KL  L EV +RK +CS C + GHN ++C +
Sbjct  1573  CSFKLKTLNEVARRKYKCSICKQNGHNCNTCPS  1671
```

Score	Expect	Method	Identities	Positives	Gaps	Frame
134 bits(491)	2e-33	Compositional matrix adjust.	92/237(39%)	154/237(64%)	3/237(1%)	+3

```
Query  266  LDKTEASYLPGQTEYKILDNEGNIMFHHLKTDPKPRNALGSFNPMNDGVPTPNAKGVRWC  325
            LDK+E +YLP+  +YKI DN +NI+F+H    + P N ++ F       P PN  GVRW
Sbjct  81   LDKAEDTYLPSYVNYKIKDNMDNIIFNHSACKSEPINLMADFREALPQFPVPNVVGVRWS  260

Query  326  YADAIAQTLKEADSHFDADILTNQSSNDSKIYSVIKDGADGLGEVSIYKEKDDKFLPDKA  385
            Y +A+A++L+E +S++D ++     + ++ +  +IKDG+DGLG+VS+YKEK+D++L DKA
Sbjct  261  YRNALAKSLQELGSEIDKGLDNCGITGNPTLQVLIKDGGDGLGDVSMYKEKGDRYLEDKA  440

Query  386  LRFSFGLVECSCISNGNENLSVYCAENPNSVRINRPLLECIGDENNRGTLSALLKPIEQE  445
             R+SF ++   + I   NE + +   + P SVR N+ L+E + DEN+ +++ A + P+E+E
Sbjct  441  YRYSFCVLKIT-IDRENERVVIWEEDTPGSVRTNKTLIEAVCDENQTAAMVACVVPVERE  617

Query  446  REYLKDKTISVKVSEGHWREHKVRFFNSMIDEKYDRSMSGYQGAGSNYMCVLCNATQ  502
            RE + ++ ISV  +   WR+ KV F NSM DE+ +  M + +G +++ +CV C   +
Sbjct  618  REQIANNLISVE-TGTLWRNFKVEFKNSMEDENGQALMVDCRGLDPD-LCVTCAMRH  782
```

DEUTEROSTOMIA

B.lanceolatum(Bla)

(TaxID: 7740; Deuterostomia; Chordata; Cephalochordata; Branchiostomidae)

WGS: FLLO01000298.1

1. Summary

Detected configuration: RAG1L - RAG2L

Detected loci	Predicted CDS	start	end	strand	Observations
RAG1L	1	156982	154142	-	complete; no stop codons
RAG2L	1	150607	150712	+	complete; no stop codons
	2	151182	151498	+	
	3	152047	153171	+	

Selected predicted protein product

BlaRAG1L_B_0298

MSSILHTEYIANVCRVCARNIPPKSQKTSVKRFKDAILETFSVNIQEDSSAVHPPSICQKCRMQ
LERLQKTSKRTSLQSVAVFQPHTEDDSCICSRKRGRPKPTEPVFRTDVGTESGVNVGASTSSGA
TSSHETASHKRKLFETEEEEEEVQSEDKTVIVNIEIERFVNQEVASTYTCIICRGLPYEVPLIT
SCDHIFCTKCISYWLEKAGSCPTCRSPMTTEDLNPLSGHLLNIFDTLKVKCLYSNKGCNMEDTV
KNIKDHESRCEYERKKTTTPGNLKWKSLRKAALYDVGRQYVKNKRLKPLLDNINSFCTEQYESK
EDVLFFALRSHLYDSNDRHRAEAVESLWFGAKPTRLTPEECLAMRVDLLMTKNSYAKEYALMKS
KDLHVLSPPSELNALEVLYMPGTARYSVQGDLHSDHYYHTPVKTSAATSTIHGKLDSLEPISIV
EDFNVSVPDFPSPNTKGVTFPYCDAVAKTLEELDDVIVSGLESLGIDPNDPSLVIHTIIKDGAD
GMGDVSVHKEASDRFLPDKALRFSFCVLKCSVEKDGEEVPVFTEDNPNSIRSNRPLLEAIGDEN
DKSTVSVCILPIEQERLVMKDKIMRVIVGDNVKRSHYLTFVNSMIDEKWDRSWGGLQGAGSKFL
CTLCDASRTEAFEKAGSYTVTRTLKQCKERANIARLNPDNLSEVALKDVCCGVKAQSMLTCEPK
ERAIDATHSDINMGSYFKTLIVREIAGVSQWGKTADVSDSISQAEAQFDRHLKATIGINPALMM
PGNYARELFDENNAAHVVSLVPSADKQAVLKEALSKFRTLRKVYRCTWPLKELPELVHQYKTVA
VEMVTMIRDNFPYASCTNYLHKITEHVQELIEDPSGAGSIGALSSEGNEAGNKLFRQLRLGHSR
KGNTLNGLRDVLWIHWLYSSKKLQSKASVTHNVYECGNCGGFGHNRRTCKN

Prediction performed with FGENESH+ (*B.floridae* dataset and BbeRAG1L as similarity reference)

BlaRAG2L_B_0298

MGRKRPDPSEAQKKGPGRKARKQKPPQMPRQLLQQVISRDFCRETKTNPRELQLVNMAEA
GRALFKCLPLLTPAVKCARKKNVAGGQVPVPGEGHSLLWDRAPGLDPDDNTSRYVLVVLA
QELNILLVDTTMDDVDVSNIAVNTRGLRQLRGVTGVWGPAEPVPSTRRGAVSRAFSRNAI
YWGGLDTEHMCLTDELVKVHLSVGGKSTKANVHVVFSPQSPTPSTSSSSTPATEEMQQGT
PPTPRTGHTLVSVSGTLAILFGGLEMPQRQGRFVSFAQTTKDGGFYSLHLPSNTWTTLPL
PKVTPRAYHSAAFVESSSELVVVGGVSYSGTEPSERISIREVLCVKISEGGYTMREVNIQ
GLPETGSYLSSLSCSILDSKVLLFGGYGQDSPPVSGKPCISSRLFCIDLEAGKGTVLQTP
GHMATAGHSSAALSDDLVLFVGGTAKALSCYTTKALVPSPCDLEKCIIASTEELSPIAWV
QCDACSKWVHQHCAGLVSAPKGSWYCCRSNRKRRK

Consensus prediction performed with FGENESH+ (*B.floridae* dataset and BbeRAG2L and SpuRAG2L as similarity reference) and Augustus (different species datasets).

(predicted coding regions merging area is underlined as it is less confident)

2. RAGL Detection info

Method: TBLASTN
Database searched: Whole-genome shotgun contigs (WGS) + only in Branchiostoma group

2.1. RAG1L

Query: **BbeRAG1_B from B.belcheri (Huang et al, 2016)**
Subject: **FLLO01000298.1 Length: 323267 Number of Matches: 1**

Score	Expect	Method	Identities	Positives	Gaps	Frame
632 bits(1630)	0.0	Compositional matrix adjust.	332/783(42%)	468/783(59%)	25/783(3%)	+3

```
Query  17      HKEYLKTVCRVCKLKLV-KGWNAKVLKFADVIRQTFDVDVSQDSPDVHPQFVCNKCRLKL  75
               H EY+  VCRVC  ++  K+ ++ V +F D I +TF V++ +DS+ VHP  +C+KCR++L
Sbjct  156967  HTEYIANVCRVCARNIPPKSQKTSVKRFKDAILETFSVNIQEDSSAVHPPSICQKCRMQL  156788

Query  76      QRKSKAG--TGIASMAIFQPHTTGD-CHCKRRRGRptssnttsqppsADVGATTSQHPTA  132
               +R  K++  T++ S+A+FQPHT +D C C R+RGRP +++    +    +DVG+ ++ +  A
Sbjct  156787  ERLQKTSKRTSLQSVAVFQPHTEDDSCICSRKRGRPKPTEPVFR---TDVGTESGVNVGA  156617

Query  133     DVRATTSQPGQNYGMTYKRKLFDESSEVESGAATETATEFDSMEVGRFVDEAVAFTFLCA  192
                  + ++ + +    + KRKLF+    E E    +E  T + ++E+ RFV++ VA T+ C
Sbjct  156616  STSSGATSSHET--ASHKRKLFETEEEEEE-VQSEDKTVIVNIEIERFVNQEVASTYTCI  156446

Query  193     VCHGVPCKSPVISNCQHIYCGNCIDFWLKRAGVCPSCRGAMTLDEDVNPLTGHLLNVYDT  252
               +C+G+P   P+I++C+HI+C +CI +WL +AG CP+CR++MT  ED+NPL+GHLLN++DT
Sbjct  156445  ICRGLPYEVPLITSCDHIFCTKCISYWLEKAGSCPTCRSPMT-TEDLNPLSGHLLNIFDT  156269

Query  253     LKVRCKYYANGCEVIEPLQHVGQHEVGCV---KTRATPESLQRKRLCKARLYDVTRHHVK  309
               LKV+C Y  +GC++ + ++++ +HE  C     K  +TP +L++K L KA LYDV R+ VK
Sbjct  156268  LKVKCLYSNKGCNMEDTVKNIKDHESRCEYERKKTTTPGNLKWKSLRKAALYDVGRQYVK  156089

Query  310     HKRLKPLIEHIDEYCNEKDENKGDVLFFLLRSHLYDTGNRSMAEQIDTLWHGESLSCMTP  369
               +KRLKPL+++I+ +C E+ E+K DVLFF LRSHLYD+ +R  AE +++LW G   + +TP
Sbjct  156088  NKRLKPLLDNINSFCTEQYESKEDVLFFALRSHLYDSNDRHRAEAVESLWFGAKPTRLTP  155909

Query  370     EECLGMRLDMLMTKNQYSKEYNILKERGFSTLCPPKQLDAIEKTLMPGTARYSIEGMDYS  429
               EECL+MR+D+LMTKN Y+KEY ++K +++  L PP +L+A+E   MPGTARYS++G   S
Sbjct  155908  EECLAMRVDLLMTKNSYAKEYALMKSKDLHVLSPPSELNALEVLYMPGTARYSVQGDLHS  155729

Query  430     EHYFHSPVKMTGDLEVHSG--ETLEPDSVTMDFHEYVPDFPCPNTKGVRFPYAHAVAKTL  487
               +HY+H+PVK ++      G  ++LEP S+  DF+  VPDFP PNTKGV FPY +AVAKTL
Sbjct  155728  DHYYHTPVKTSAATSTIHGKLDSLEPISIVEDFNVSVPDFPSPNTKGVTFPYCDAVAKTL  155549

Query  488     EELEDEIVNGLKKLGRDPNDPTLVIHTICKDGADGMGDVSVHKEKSDHLLPDKALRFSFC  547
               EEL+D IV+GL  LG DPNDP+LVIHTI KDGADGMGDVSVHKE SD++LPDKALRFSFC
Sbjct  155548  EELDDVIVSGLESLGIDPNDPSLVIHTIIKDGADGMGDVSVHKEASDRFLPDKALRFSFC  155369

Query  548     VLRCSVMHKDTEVTIYEDPNPNSVRSNRPVLECIGDENDDGTVAVCVGPIECQRLLMKDK  607
               VL+CSV   + EV ++ + NPNS+RSNRP+LE IGDEND +TV+VC+ PIE +RL+MKDK
Sbjct  155368  VLKCSVEKDGEEVPVFTEDNPNSIRSNRPLLEAIGDENDKSTVSVCILPIEQERLVMKDK  155189

Query  608     IMRVHMSDGTQRAHYLTFFNSMVDEKWDRAHGGLAGAGSKYLCTLCEAVRDEALEKAGSY  667
               IMRV ++D  +R+HYLTF NSM+DEKWDR+ GGL GAGSK+LCTLC+A R EA+EKAGSY
Sbjct  155188  IMRVIVGDNVKRSHYLTFVNSMIDEKWDRSWGGLQGAGSKFLCTLCDASRTEAFEKAGSY  155009
```

```
Query  668     KITRTLK--KIEVTASKMKYESQEKDTF-----GVKGYPLLTTEPWERGIDATHTDINMG  720
                +TRTLK  K     ++++ ++    ++     GVK+ ++LT EP ER+IDATH+DINMG
Sbjct  155008  TVTRTLKQCKERANIARLNPDNLSEVALKDVCCGVKAQSMLTCEPKERAIDATHSDINMG  154829

Query  721     NYFKSLIVREMAQVHSWAKTANVKKQIVDAESKLDKHLKESLGLNPTLMMAGNYARELFK  780
               +YFK+LIVRE+A V  W+KTA+V   I +AE+++D+HLK ++G+NP+LMM+GNYARELF
Sbjct  154828  SYFKTLIVREIAGVSQWGKTADVSDSISQAEAQFDRHLKATIGINPALMMPGNYARELFD  154649

Query  781     AEHADKLVALVDKPDRKSALVEVLAKFRQLRKVYRANWPLNDMSDEVRQYKAKAVEMAND  840
                ++A  +V+LV  +D+++ L E L+KFR LRKVYR  WPL+++++ V+QYK+ AVEM
Sbjct  154648  ENNAAHVVSLVPSADKQAVLKEALSKFRTLRKVYRCTWPLKELPELVHQYKTVAVEMVTM  154469

Query  841     LKTHFPYAPCTNYLHKVIEHVQELIEHPSGVGSVGALSSEGNEAGNKLFRQLRLGHARKG  900
               ++ +FPYA+CTNYLHK+ EHVQELIE+PSG GS+GALSSEGNEAGNKLFRQLRLGH+RKG
Sbjct  154468  IRDNFPYASCTNYLHKITEHVQELIEDPSGAGSIGALSSEGNEAGNKLFRQLRLGHSRKG  154289

Query  901     NTYNGLRDVLCTHWLYTSKTLRDKAAVTERSLCCGRCGGVGHNVRTC  947
               NT NGLRDVL  HWLY+SK L+ KA+VT+    CG CGG GHN RTC
Sbjct  154288  NTLNGLRDVLWIHWLYSSKKLQSKASVTHNVYECGNCGGFGHNRRTC  154148
```

2.2 RAG2L

Query: **BbeRAG2_B from B.belcheri (Huang et al, 2016)**
Subject: **FLLO01000298.1 Length: 323267 Number of Matches: 1**

Score	Expect	Method	Identities	Positives	Gaps	Frame
178 bits(661)	1e-50	Compositional matrix adjust.	119/314(38%)	207/314(65%)	31/314(9%)	+3

```
Query  80      EVKAMPHIASGCTAVWGPPLPPKPGKD-------TKQVILWGGLDKRRWCCSNDLTQVDI  132
               + +++ ++  G T+VWGP+ P  + +         +++ I WGGLD  + C +++L +V++
Sbjct  152049  NTRGLRQL-RGVTGVWGPAEPVPSTRRGAVSRAFSRNAIYWGGLDTEHMCLTDELVKVHL  152225

Query  133     TITPKTTTAKVSIL-----------------PADKQDGVP-SPRTGHTLVAISSLQAILF  174
               ++  K+T A+V ++                   + Q+G P +PRTGHTLV++S+  AILF
Sbjct  152226  SVGGKSTKANVHVVFSPQSPTPSTSSSSTPATEEMQQGTPPTPRTGHTLVSVSGTLAILF  152405

Query  175     GGLELASRHARLGTCAQSCKDGYFYLLDMTTLRWNKLPLPPLVPRAYHSSTWVPASSTMV  234
               GGLE++ R++R+ + AQ+ KDG FY L++ +  W  LPLP + PRAYHS++ V +SS +V
Sbjct  152406  GGLEMPQRQGRFVSFAQTTKDGGFYSLHLPSNTWTTLPLPKVTPRAYHSAAFVESSSELV  152585

Query  235     IVGGITYSGHCPSERLSVSDVVCLKISDTSQYTLTEIHMEGVRD--SYVssssasaLCDD  292
               +VGG++YSG  PSER+S+ +V+C+KIS+ + YT+ E++++G+ +  SY+SS S S L D
Sbjct  152586  VVGGVSYSGTEPSERISIREVLCVKISEGG-YTMREVNIQGLPETGSYLSSLSCSIL-DS  152759

Query  293     RFVLYGGYHHDKSGLHP-PEPSRDLYVMNLQTKKAVVHHAPTRMASAGHTCLRLIDNSVV  351
               + +L+GGY +D + +   P  S  L+ ++L++ K+ V ++P +MA+AGH+   L D+ V+
Sbjct  152760  KVLLFGGYGQDSPPVSGKPCISSRLFCIDLEAGKGTVLQTPGHMATAGHSSAALSDDLVL  152939

Query  352     MIGGTCKSVNCCTN  365
                +GGT K+++C T
Sbjct  152940  FVGGTAKALSCYTT  152981
```

WGS: FLLO01009573.1

Summary

Detected configuration: RAG1L - RAG2L

Detected loci	Predicted CDS	start	end	strand	Observations
RAG1L	-	16467	16054	-	incomplete; short fragment
RAG2L	-	13580	15137	+	incomplete; stop codon in the middle

WGS: FLLO01000085.1-copy 1

Summary

Detected configuration: RAG1L

Detected loci	Predicted CDS	start	end	strand	Observations
RAG1L	-	163179	167043	+	incomplete; stop codons

WGS: FLLO01000085.1-copy2

Summary

Detected configuration: RAG1L

Detected loci	Predicted CDS	start	end	strand	Observations
RAG1L	-	210857	214068	+	incomplete; stop codons

WGS: FLLO01000417.1

Summary

Detected configuration: RAG1L

Detected loci	Predicted CDS	start	end	strand	Observations
RAG1L	-	47929	46709	-	incomplete; stop codons

WGS: FLLO01000370.1

Summary

Detected configuration: RAG1L

Detected loci	Predicted CDS	start	end	strand	Observations
RAG1L	-	36710	34167	-	fragmented; stop codons

WGS: FLLO01000291.1

Summary

Detected configuration: RAG1L

Detected loci	Predicted CDS	start	end	strand	Observations
RAG1L	-	302870	303969	+	incomplete; stop codons

WGS: FLLO01000027.1

Summary

Detected configuration: RAG1L

Detected loci	Predicted CDS	start	end	strand	Observations
RAG1L	-	1914267	1912523	-	incomplete; stop codons

Hemicentrotus pulcherrimus (Hpu)

(TaxID: 7650; Deuterostomia; Echinodermata; Echinozoa)

WGS: BEXV01003119.1

3. Summary

Detected configuration: RAG1L - RAG2L

Detected loci	Predicted CDS	start	end	strand	Observations
RAG1L	1	29836	29655	-	complete; no stop codons
	2	29274	28818	-	
	3	28313	26514	-	
RAG2L	1	21238	21547	+	complete; no stop codons
	2	23565	23903	+	
	3	24507	25366	+	

Selected predicted protein product

HpuRAG1L_B_3119

```
MEEHLKALGMMCRICGEVVSSSKNRSRLDRSKAELLAIYHINLDDEHGDSIHPSIMCNKC
RHQLHRSYQAMQQGKQYQGKLAHLQLFSEHSPSCYVCDAHAAHNSSLLPEFKPPPPPPSP
SVSTTPVLGPTVATATKELFAEKNPGPGTSFKKVWNAKSSLKDVRRDWARKRTHEAASFI
SDFCDRQKEKKNDLLFFLLTESLQDSNDPRCKEVMSTWNDKKVVLTVDNCLAMRVTTMTS
KTRYKEQYDALKSSGVDILKPPHQLTECEKSYMPGSVRFRMGGDTVYTHHTPVKEGRQVE
YGGSSYEPLDLTSPHNPLVEIPFPNLKGVAWPYGHALAKTLEELDIDIAEGLLKVGLNPE
ENYEITTTIKDGADGMGDISVFREARDRLLPDKAFRVAFAVIKCEVTLGDERLTVYEPDK
PESTFITRPLVEAIADENNRASSILLLEGIECERAALRKSGLSVNIGNSWRRHNLRFYNS
MIDEKLDRADGGLQGSGSKFLCTLCHATRETAKSELGSFRITRTYEETDAIANYILINPD
DLSPAQLGEMSQGVKSRPVLQSDPREKLIDATHADINIGSFFKKLIICETARIQTWEIRQ
DVKESYEKAEKKFDDHLLSTLGLGPSLMMPGNFARALFALKNNDIVLQLVKDEERRERLK
EVLVLFSRLRSVYRAHHPKVDEVKVYKVNAVSMGNLLKTHFAYVSWPNYLHKVIEHVQEI
LEDPSGPQTVGGLSGEGNEASNKLFRDLRRHYARKHDVTQNLRDILWVHWLYTSPKIRRL
SSKTTRSYHCSKCDGLWHNARSCPGPSPSKDA

TSA: IACU01023786.1 (100% identical)
```

Consensus prediction performed with FGENESH+ (*S.purpuratus* dataset and SpuRAG1L as similarity reference) and TSA entry

HpuRAG2L_B_3119

```
MANLNYSDLEYIPLQAAKENKKKVTLKRSHLEMALGEHFPSEGHCSIFTPSSEDRDSQCR
ILTFGGARLREERTWQDGANITEFLLKADEDDVYIEASLCKTKGRHTPDHALLLYCNSRT
GIIMFKDAVYDLCVAGGTLPPLHGAAVVDIGQRQIIWGGLDLSEYRCTDDLMILQELTLP
RRGAPIFQITYIQTLKHQVPSRLGKDIPHQTGPVPSERSGHTLTMLPGTNKALLFGGLSM
GTHKGYYNNSRFCQTCKDGRFYILDTDTYEWQHIKVPLIQPRAYHSVTVMEKDNEFVAAL
IGGVVYEETAPTHREALNEIVVLTIDKEIQNFSLKEVSLQPSMPTTHNVFLSSHATTVHN
NVIIVAGGVQDQKKEMKEKSRKASSTVYSIDLGAKVFEAFPATCNASETFATHGHSIHHL
ASDTDNTFLVLGGSSRQISLLTDRSFEPESCDSEPCTIASSGDKVDPTWIQCNRKCQKWF
HIHCIKLTRIPEGDYHCKKCKK

TSA: IACU01028708.1(100% ident)
```

Consensus prediction performed with FGENESH+ (*S.purpuratus* dataset and SpuRAG1L as similarity reference) and TSA entry
(predicted coding regions merging area is underlined as it is less confident)

4. RAGL Detection info

Method: TBLASTN
Database searched: WGS project: BEXV

2.1. RAG1L

Query: **EchRAG1 from Morales Poole et al, 2017**
Subject: **BEXV01003119.1 Length: 48415 Number of Matches: 3**

Score	Expect	Method	Identities	Positives	Gaps	Frame
60.8 bits(146)	6e-08	Compositional matrix adjust.	27/59(46%)	41/59(69%)	3/59(5%)	-2

```
Query  11     HVAALSKVCRICATFISSKKNMSKIARYRNEVLGVYHVDIEKDDKG--IHPPSLCNKCR  67
              H+ AL  +CRIC   +SS KN S++ R + E+L +YH++++ D+ G  IHP  +CNKCR
Sbjct  29826  HLKALGMMCRICGEVVSSSKNRSRLDRSKAELLAIYHINLD-DEHGDSIHPSIMCNKCR  29653
```

Score	Expect	Method	Identities	Positives	Gaps	Frame
133 bits(334)	4e-30	Compositional matrix adjust.	73/166(44%)	107/166(64%)	12/166(7%)	-1

```
Query  67     RLSVTRAFTSLQSGKKYEGQIPNLTEFPNHQPSCPICLEHRVRLNSIQPVFKKPAALPST  126
              R  + R++ ++Q GK+Y+G++ +L  F  H PSC +C  H    +S+ P FK P   PS
Sbjct  29275  RHQLHRSYQAMQQGKQYQGKLAHLQLFSEHSPSCYVCDAHAAHNSSLLPEFKPPPPPPSP  29096

Query  127    LtsgssqrasqtssstqGPTVASTSRVLFSESVPDPGMAVHGRKRVWSAKASLKDVGRDY  186
                       S +++   GPTVA+ ++ LF+E  P PG +    K+VW+AK+SLKDV RD+
Sbjct  29095  ---------SVSTTPVLGPTVATATKELFAEKNPGPGTSF---KKVWNAKSSLKDVRRDW  28952

Query  187    ARNRSKGAEDFMTEYCMKQKEKKTDLLYFLLTEDLRNSNDHRFKEV  232
              AR R+  A  F++++C +QKEKK DLL+FLLTE L++SND R KEV
Sbjct  28951  ARKRTHEAASFISDFCDRQKEKKNDLLFFLLTESLQDSNDPRCKEV  28814
```

Score	Expect	Method	Identities	Positives	Gaps	Frame
760 bits(1962)	0.0	Compositional matrix adjust.	361/597(60%)	449/597(75%)	5/597(0%)	-1

```
Query  229    FKEVMASWGDELSANLTADDCLAMRISTLTSKRKYKEQYKLLKSKGNDFLKPPHLLDEME  288
              F +VM++W D+    LT D+CLAMR++T+TSK +YKEQY  LKS G D LKPPH L E E
Sbjct  28321  FLQVMSTWNDK-KVVLTVDNCLAMRVTTMTSKTRYKEQYDALKSSGVDILKPPHQLTECE  28145

Query  289    KSYMPGSVRFQLEGGADLIHHTPVKAGAAHIEYGNLSLEPAIIGSGGLHEEL-ELPPPNF  347
              KSYMPGSVRF++ G     HHTPVK G   +EYG  S EP  + S   H  L E+P PN
Sbjct  28144  KSYMPGSVRFRMGGDTVYTHHTPVKEGR-QVEYGGSSYEPLDLTSP--HNPLVEIPFPNL  27974

Query  348    KGVEWPYPHALAKTLEELDEEIQRGLMKAGLDPQGDDTIVTTVKDGSDGMGEISVTKEAS  407
              KGV WPY HALAKTLEELD +I  GL+K GL+P+ +  I TT+KDG+DGMG+ISV +EA
Sbjct  27973  KGVAWPYGHALAKTLEELDIDIAEGLLKVGLNPEENYEITTTIKDGADGMGDISVFREAR  27794

Query  408    DRLLPNKAFRAAFAVVKCEVEVGGSKVLVYEPDNPNSVFVTRPIVEAIADENNVGCGVAM  467
              DRLLP+KAFR AFAV+KCEV +G  ++ VYEPD P S F+TRP+VEAIADENN    + +
Sbjct  27793  DRLLPDKAFRVAFAVIKCEVTLGDERLTVYEPDKPESTFITRPLVEAIADENNRASSILL  27614
```

```
Query  468    LDSMEHSRDVMKNKEMRVFVDGWWRRHRLIFYNSMIDEKLDRAQGGLQGSGSKYLCTLCH  527
              L+ +E  R  ++   + V +   WRRH L FYNSMIDEKLDRA GGLQGSGSK+LCTLCH
Sbjct  27613  LEGIECERAALRKSGLSVNIGNSWRRHNLRFYNSMIDEKLDRADGGLQGSGSKFLCTLCH  27434

Query  528    ATRDTARSNVGSFSITRSFEETSDTANYIQTNPDNLSQNELAEFAKGVKDYPVLRMDPDH  587
              ATR+TA+S +GSF ITR++EET   ANYI  NPD+LS  +L E ++GVK  PVL+ DP
Sbjct  27433  ATRETAKSELGSFRITRTYEETDAIANYILINPDDLSPAQLGEMSQGVKSRPVLQSDPRE  27254

Query  588    KLIDATHADINLGSFFQKLIVCEIARTYTWEITRDVKVMFEDAEKRFNDHIRASLGLAPT  647
              KLIDATHADIN+GSFF+KLI+CE AR  TWEI +DVK  +E AEK+F+DH+ ++LGL P+
Sbjct  27253  KLIDATHADINIGSFFKKLIICETARIQTWEIRQDVKESYEKAEKKFDDHLLSTLGLGPS  27074

Query  648    LMMPGNYARQLFDSRNIDTILELIPDREKKEHLSEVMSLFRKLREVYRAHHPLQADFATY  707
              LMMPGN+AR LF  +N D +L+L+ D E++E L EV+ LF +LR VYRAHHP   +   Y
Sbjct  27073  LMMPGNFARALFALKNNDIVLQLVKDEERRERLKEVLVLFSRLRSVYRAHHPKVDEVKVY  26894

Query  708    KVKAVQMADLLDRHFPYASWPNYLHKIIEHVQEVLEDPRGPGTIGDLSGEGNEAANKLFR  767
              KV AV M +LL  HF Y SWPNYLHK+IEHVQE+LEDP GP T+G LSGEGNEA+NKLFR
Sbjct  26893  KVNAVSMGNLLKTHFAYVSWPNYLHKVIEHVQEILEDPSGPQTVGGLSGEGNEASNKLFR  26714

Query  768    DLRRNFSRKNCTLDSLRDILWMHWLYTSPKLRRMVIVASRSYKCTRCNDSGHNILTC  824
              DLRR+++RK+    +LRDILW+HWLYTSPK+RR+    +RSY C++C+   HN  +C
Sbjct  26713  DLRRHYARKHDVTQNLRDILWVHWLYTSPKIRRLSSKTTRSYHCSKCDGLWHNARSC  26543
```

2.2 RAG2L

Query: EchRAG2 from Morales Poole et al, 2017

Subject: BEXV01003119.1 **Length: 48415** **Number of Matches: 3**

Score	Expect	Method	Identities	Positives	Gaps	Frame
92.4 bits(228)	2e-18	Compositional matrix adjust.	46/92(50%)	67/92(72%)	3/92(3%)	+3

```
Query  7      ASIDNKRKVTRKRNNLEVKLGEKYPPEGHCSVTRCSAASAEGHNEYRILELGGARRKEEA  66
              A+ +NK+KVT KR++LE+ LGE +P EGHCS+   + +S +  ++ RIL  GGAR +EE
Sbjct  21282  AAKENKKKVTLKRSHLEMALGEHFPSEGHCSIF--TPSSEDRDSQCRILTFGGARLREER  21455

Query  67     TWNEGSTLTEFLVSSDEDDVSITSVTSCKTSG  98
              TW +G+ +TEFL+ +DEDDV I + + CKT G
Sbjct  21456  TWQDGANITEFLLKADEDDVYIEA-SLCKTKG  21548
```

Score	Expect	Method	Identities	Positives	Gaps	Frame
91.7 bits(226)	3e-18	Compositional matrix adjust.	41/88(47%)	60/88(68%)	2/88(2%)	+1

```
Query  96     TSGGTIRPLHGATMVDLDSKLLLWGGLDLKCYECRDDLLVIEELPRQRRGGALPHFDVTW  155
               +GGT+ PLHGA +VD+   + ++WGGLDL  Y C DDL++++EL   RRG   P F +T+
Sbjct  23653  VAGGTLPPLHGAAVVDIGQRQIIWGGLDLSEYRCTDDLMILQELTLPRRGA--PIFQITY  23826

Query  156    VQTEDSQTTSRMGLECKNQTGAIPSGRS  183
              +QT   Q  SR+G +  +QTG +PSG +
Sbjct  23827  IQTLKHQVPSRLGKDIPHQTGPVPSGMT  23910
```

Score	Expect	Method	Identities	Positives	Gaps	Frame
312 bits(800)	3e-93	Compositional matrix adjust.	144/286(50%)	204/286(71%)	7/286(2%)	+1

```
Query  182    RSGHTMTKIPGTNKVLLFGGYSINKNN---NTARWRKSCNDGRLYILDTDSYEWKTVRVP  238
              RSGHT+T +PGTNK LLFGG S+   +   N +R+ ++C DGR YILDTD+YEW+ ++VP
Sbjct  24508  RSGHTLTMLPGTNKALLFGGLSMGTHKGYYNNSRFCQTCKDGRFYILDTDTYEWQHIKVP  24687

Query  239    LIQARAFHTTNILEIDDRFVLPIIGGVIFDEGTPTFRESLSEITIVSMDKQLNQFSLSEI  298
              LIQ RA+H+  ++E D+ FV  +IGGV+++E  PT RE+L+EI ++++DK++  FSL E+
Sbjct  24688  LIQPRAYHSVTVMEKDNEFVAALIGGVVYEETAPTHREALNEIVVLTIDKEIQNFSLKEV  24867

Query  299    SLLPVSPDTHSVYLSSHASVVHNGQIIVVGGIQDLEREIKPDVTPKACAKAFVLNLTLNT  358
              SL P  P TH+V+LSSHA+ VHN  IIV GG+QD ++E+K + + KA +  + ++L
Sbjct  24868  SLQPSMPTTHNVFLSSHATTVHNNVIIVAGGVQDQKKEMK-EKSRKASSTVYSIDLGAKV  25044

Query  359    YQLLPKTSQAESIFRTYGATIHNLVG--DDTILILGGSSRQISLLSDRTYEPQECDYVPC  416
              ++  P T  A   F T+G +IH+L    D+T L+LGGSSRQISLL+DR++EP+ CD  PC
Sbjct  25045  FEAFPATCNASETFATHGHSIHHLASDTDNTFLVLGGSSRQISLLTDRSFEPESCDSEPC  25224

Query  417    TIADSPESGDPTWIECD-KCHKCLHTFCIKLKKNPDGKYFCTNCKR  461
              TIA S +  DPTWI+C+ KC K  H  CIKL + P+G Y C  CK+
Sbjct  25225  TIASSGDKVDPTWIQCNRKCQKWFHIHCIKLTRIPEGDYHCKKCKK  25362
```

WGS: BEXV01002133.1

5. Summary

Detected configuration: RAG1L - RAG2L

Detected loci	Predicted CDS	start	end	strand	Observations
RAG1L	1	4899	5134	+	complete; no stop codon
	2	5720	6800		
	3	7109	8902		
RAG2L	1	18125	12225	-	complete; 1 stop codon in the mjiddle

Selected predicted protein product

HpuRAG1L_B_2133

```
MSPSTLDSFLFCLPKVMETAELHRKALSHTCRLCGSYVKNKKSLSSKEKYEELILSVYGI
DFKLDDEDVHPPRICVSCRLWMTRSDSRNAEGPTYPTSGKTLANFSAHPELEPCSICEAT
TSTKRKAVGTDGLPPPKKPSAVVSGPDEQQASCSFTAPLPTTATSYQPIKPKTRSDSRNA
EGPTYHLCSICAATTLTKEKAVGTDDLPPPKIPSAAVSGPDEQIALCSFTAPLPPTATAP
LPPTATVLLTPTATVPLTPTATALLILTATAPLTLTATAPLTQAATVPLTPTATAPLPPT
ATVPLTPTVTAPLTPTATAPLTPTATAPLTPTATAPLTPTATDPLTPTATRYRPIVTKDR
AHFTRALFSPVLTVPARKSPVRAKGSLHYVRRDCAKNRAKGALDFMTSHSAAKNEDETDL
WFFGLHNRLRNEKDERAKMVMELWTERKNTTDLSIDDCLAMRVGTLCTKGMYAEKYRFLK
SKGDTTFKPPSQLTNRESYYMPGNVRFGLMDGGKCVYHTPEKSLEEFDDNSMYEPIRINV
RSKLTEFALPNCIGVAWSYPEAVAKTLEELDENIREGMLKVGLNPDDPSIIIDTTLKDGA
DGMGEIAVHKMKSDKFLPDKAFRASFVVLKCEVKRDDGTRDLVFKEPKPNSVMVNRPLLE
AIGDENSASTSAVLMRKMEKERLILQNSIMTIHARTHTRLHRLTIYNSMIDEKLARSSGG
LQGSGSHFICTLCHATKKSAKTQLGSFKIDRTLTETQQTSTYITTNPDNLTPDELATEAG
GVKRKPLLTSEPKQQLMDATHADISLGQFFKKIIVREIAGVHKWEASENVKQYIVDAERR
LDIHVRELLGTAPSIMMPGNYARALFKEKNEDIFLELIRNEERKALLRSVLQQFRALRKV
YREHQPNKKEVQGFKKKAVQMGRELLEHFEYVCWPNYLHKILEHTQESILSEDGPGSIGI
LSGEGSEAANKLFRKLRNNFSRRGDVWDGLRDILWFHWLYTSPKLLRLRDVTRRTYTCSR
CGAEGHNIKSCNVKAT
```

TSA support: IACU01059282.1 & IACU01059285.1 (99% identical)

Consensus prediction performed with FGENESH+ (*S.purpuratus* dataset and SpuRAG1L as similarity reference) and TSA entries

(predicted coding regions merging area is underlined as it is less confident)

```
                 1                                                                                                          110
                 |------------------------------------------------------------------------------------------------------------|
 HpuRAG1L_B_2133 MSPSTLDSFLFCLPKVMETAELHRKALSHTCRLCGSYVKNKKSLSSKEKYEELILSVYGIDFKLDDEDVHPPRICVSCRLWMTRSDSRNAEGPTYPTSGKTLANFSAHPE
  IACU01059285.1
  IACU01059282.1
       Consensus ..............................................................................................................

                 111                                                                                                        220
                 |------------------------------------------------------------------------------------------------------------|
 HpuRAG1L_B_2133 LEPCSICEATTSTKRKAVGTDGLPPPKKPSAVVSGPDEQQASCSFTAPLPTTATSYQPIKPKTRSDSRNAEGPTYHLCSICAATTLTKEKAVGTDDLPPPKIPSAAVSGP
  IACU01059285.1
  IACU01059282.1
       Consensus ..............................................................................................................

                 221                                                                                                        330
                 |------------------------------------------------------------------------------------------------------------|
 HpuRAG1L_B_2133 DEQIALCSFTAPLPPTATAPLPPTATVLLTPTATVPLTPTATALLILTATAPLTLTATAPLTQAATVPLTPTATAPLPPTATVPLTPTVTAPLTPTATAPLTPTATAPLT
  IACU01059285.1
  IACU01059282.1
       Consensus ..............................................................................................................

                 331                                                                                                        440
                 |------------------------------------------------------------------------------------------------------------|
 HpuRAG1L_B_2133 PTATAPLTPTATDPLTPTATRYRPIVTKDRAHFTRALFSPVLTVPARKSPVRAKGSLHYVRRDCAKNRAKGALDFMTSHSAAKNEDETDLWFFGLHNRLRNEKDERAKMV
  IACU01059285.1                                                                            MTSHSAAKNEDETDLWFFGLHNRLRNEKDERAKMV
  IACU01059282.1                                                                            MTSHSAAKNEDETDLWFFGLHNRLRNEKDERAKMV
       Consensus ...........................................................................MTSHSAAKNEDETDLWFFGLHNRLRNEKDERAKMV

                 441                                                                                                        550
                 |------------------------------------------------------------------------------------------------------------|
 HpuRAG1L_B_2133 MELWTERKNTTDLSIDDCLAMRVGTLCTKGMYAEKYRFLKSKGDTTFKPPSQLTNRESYYMPGNVRFGLMDGGKCVYHTPEKSLEEFDDNSMYEPIRINVRSKLTEFALP
  IACU01059285.1 MELWTERKNTTDLSIDDCLAMRVGTLCTKGMYAEKYRFLKSKGDTTFKPPSQLTNRESYYMPGNVRFGLMDGGKCVYHTPEKSLEEFDDNSMYEPIRINVRSKLTEFALP
  IACU01059282.1 MELWTERKNTTDLSIDDCLAMRVGTLCTKGMYAEKYRFLKSKGDTTFKPPSQLTNRESYYMPGNVRFGLMDGGKCVYHTPEKSLEEFDDNSMYEPIRINVRSKLTEFALP
       Consensus MELWTERKNTTDLSIDDCLAMRVGTLCTKGMYAEKYRFLKSKGDTTFKPPSQLTNRESYYMPGNVRFGLMDGGKCVYHTPEKSLEEFDDNSMYEPIRINVRSKLTEFALP

                 551                                                                                                        660
                 |------------------------------------------------------------------------------------------------------------|
 HpuRAG1L_B_2133 NCIGVAWSYPEAVAKTLEELDENIREGMLKVGLNPDDPSIIIDTTLKDGADGMGEIAVHKMKSDKFLPDKAFRASFVVLKCEVKRDDGTRDLVFKEPKPNSVMVNRPLLE
  IACU01059285.1 NCIGVAWSYPEAVAKTLEELDENIREGMLKVGLNPDDPSIIIDTTLKDGADGMGEIAVHKMKSDKFLPDKAFRASFVVLKCEVKRDDGTRDLVFKEPKPNSVMVNRPLLE
  IACU01059282.1 NCIGVAWSYPEAVAKTLEELDENIREGMLKVGLNPDDPSIIIDTTLKDGADGMGEIAVHKMKSDKFLPDKAFRASFVVLKCEVKRDDGTRDLVFKEPKPNSVMVNRPLLE
       Consensus NCIGVAWSYPEAVAKTLEELDENIREGMLKVGLNPDDPSIIIDTTLKDGADGMGEIAVHKMKSDKFLPDKAFRASFVVLKCEVKRDDGTRDLVFKEPKPNSVMVNRPLLE

                 661                                                                                                        770
                 |------------------------------------------------------------------------------------------------------------|
 HpuRAG1L_B_2133 AIGDENSASTSAVLMRKMEKERLILQNSIMTIHARTHTRLHRLTIYNSMIDEKLARSSGGLQGSGSHFICTLCHATKKSAKTQLGSFKIDRTLTETQQTSTYITTNPDNL
  IACU01059285.1 AIGDENSASTSAVLMRKMEKERLILQNSIMTIHARTHTRLHRLTIYNSMIDEKLARSSGGLQGSGSHFICTLCHATKTSAKTQLGSFKIDRTLTETQQTSTYITTNPDNL
  IACU01059282.1 AIGDENSASTSAVLMRKMEKERLILQNSIMTIHARTHTRLHRLTIYNSMIDEKLARSSGGLQGSGSHFICTLCHATKTSAKTQLGSFKIDRTLTETQQTSTYITTNPDNL
       Consensus AIGDENSASTSAVLMRKMEKERLILQNSIMTIHARTHTRLHRLTIYNSMIDEKLARSSGGLQGSGSHFICTLCHATKtSAKTQLGSFKIDRTLTETQQTSTYITTNPDNL

                 771                                                                                                        880
                 |------------------------------------------------------------------------------------------------------------|
 HpuRAG1L_B_2133 TPDELATEAGGVKRKPLLTSEPKQQLMDATHADISLGQFFKKIIVREIAGVHKWEASENVKQYIVDAERRLDIHVRELLGTAPSIMMPGNYARALFKEKNEDIFLELIRN
  IACU01059285.1 TPDELATEAGGVKRKPLLTSEPKQQLMDATHADINLGQFFKKIIVREIAGVHKWEASENVKQYIVDAERRLDIHVRELLGTAPSLMMPGNYARALFKEKNEDIFLELIRN
  IACU01059282.1 TPDELATEAGGVKRKPLLTSEPKQQLMDATHADINLGQFFKKIIVREIAGVHKWEASENVKQYIVDAERRLDIHVRELLGTAPSLMMPGNYARALFKEKNEDIFLELIRN
       Consensus TPDELATEAGGVKRKPLLTSEPKQQLMDATHADInLGQFFKKIIVREIAGVHKWEASENVKQYIVDAERRLDIHVRELLGTAPSlMMPGNYARALFKEKNEDIFLELIRN

                 881                                                                                                        990
                 |------------------------------------------------------------------------------------------------------------|
 HpuRAG1L_B_2133 EERKALLRSVLQQFRALRKVYREHQPNKKEVQGFKKKAVQMGRELLEHFEYVCWPNYLHKILEHTQESILSEDGPGSIGILSGEGSEAANKLFRKLRNNFSRRGDVWDGL
  IACU01059285.1 EERKALLRSVLQQFRALRKVYREHQPNKKEVQGFKKKAVQMGRELLEHFEYVCWPNYLHKILEHTQESILSEDGPGSIGILSGEGSEAANKLFRKLRNNFSRRGDVWDGL
  IACU01059282.1 EERKALLRSVLQQFRALRKVYREHQPNKKEVQGFKKKAVQMGRELLEHFEYVCWPNYLHKILEHTQESILSEDGPGSIGILSGEGSEAANKLFRKLRNNFSRRGDVWDGL
       Consensus EERKALLRSVLQQFRALRKVYREHQPNKKEVQGFKKKAVQMGRELLEHFEYVCWPNYLHKILEHTQESILSEDGPGSIGILSGEGSEAANKLFRKLRNNFSRRGDVWDGL

                 991                                        1036
                 |--------------------------------------------|
 HpuRAG1L_B_2133 RDILWFHWLYTSPKLLRLRDVTRRTYTCSRCGAEGHNIKSCNVKAT
  IACU01059285.1 RDILWFHWLYTSPKLLRLRDVTRRTYTCSRCGAEGHNIKSCNVKAT
  IACU01059282.1 RDILWFHWLYTSPKLLRLRDVTRRTYTCSRCGAEGHNIKSCNVKAT
       Consensus RDILWFHWLYTSPKLLRLRDVTRRTYTCSRCGAEGHNIKSCNVKAT
```

6. RAGL Detection info

Method: TBLASTN
Database searched: WGS project: BEXV

2.1. RAG1L

Query: **EchRAG1 from Morales Poole et al, 2017**
Subject: **BEXV01002133.1 Length: 164675 Number of Matches: 3**

Score	Expect	Method	Identities	Positives	Gaps	Frame
64.7 bits(156)	4e-09	Compositional matrix adjust.	28/80(35%)	48/80(60%)	3/80(3%)	+3

```
Query  6     EAADVHVAALSKVCRICATFISSKKNMSKIARYRNEVLGVYHVDIEKDDKGIHPPSLCNK  65
             E A++H  ALS  CR+C +++ +KK++S   +Y   +L VY +D + DD+ +HPP +C
Sbjct  4950  ETAELHRKALSHTCRLCGSYVKNKKSLSSKEKYEELILSVYGIDFKLDDEDVHPPRICVS  5129

Query  66    CRLS---VTRAFTSLQSGKK  82
             CR     +TR  +  ++  K
Sbjct  5130  CRYEHDHLTRVLSCYEAHIK  5189
```

Score	Expect	Method	Identities	Positives	Gaps	Frame
65.9 bits(159)	2e-09	Compositional matrix adjust.	38/82(46%)	46/82(56%)	5/82(6%)	+3

```
Query  151   SRVLFSESVPDPGMAVHGRKRVWSAKASLKDVGRDYARNRSKGAEDFMTEYCMKQKEKKT  210
             +R LFS     P + V  RK    AK SL  V RD A+NR+KGA DFMT +   + E +T
Sbjct  6573  TRALFS-----PVLTVPARKSPVRAKGSLHYVRRDCAKNRAKGALDFMTSHSAAKNEDET  6737

Query  211   DLLYFLLTEDLRNSNDHRFKEV  232
             DL +F L   LRN  D R K V
Sbjct  6738  DLWFFGLHNRLRNEKDERAKMV  6803
```

Score	Expect	Method	Identities	Positives	Gaps	Frame
596 bits(1536)	0.0	Compositional matrix adjust.	312/600(52%)	399/600(66%)	9/600(1%)	+2

```
Query  232   VMASWGDEL-SANLTADDCLAMRISTLTSKRKYKEQYKLLKSKGNDFLKPPHLLDEMEKS  290
             VM  W +   + +L+ DDCLAMR+ TL +K  Y E+Y+ LKSKG+   KPP  L   E
Sbjct  7109  VMELWTERKNTTDLSIDDCLAMRVGTLCTKGMYAEKYRFLKSKGDTTFKPPSQLTNRESY  7288

Query  291   YMPGSVRFQLEGGADLIHHTPVKAGAAHIEYGNLSL-EPAIIGSGGLHEELELPPPNFKG  349
             YMPG+VRF L  G   ++HTP K+     E+ + S+ EP  I       E  LP  N  G
Sbjct  7289  YMPGNVRFGLMDGGKCVYHTPEKSLE---EFDDNSMYEPIRINVRSKLTEFALP--NCIG  7453

Query  350   VEWPYPHALAKTLEELDEEIQRGLMKAGLDPQGDDTIV-TTVKDGSDGMGEISVTKEASD  408
             V W YP A+AKTLEELDE I+ G++K GL+P     I+ TT+KDG+DGMGEI+V K  SD
Sbjct  7454  VAWSYPEAVAKTLEELDENIREGMLKVGLNPDDPSIIIDTTLKDGADGMGEIAVHKMKSD  7633

Query  409   RLLPNKAFRAAFAVVKCEVEVG-GSKVLVYEPDNPNSVFVTRPIVEAIADENNVGCGVAM  467
             + LP+KAFRA+F V+KCEV+   G++ LV++   PNSV V RP++EAI DEN+      +
Sbjct  7634  KFLPDKAFRASFVVLKCEVKRDDGTRDLVFKEPKPNSVMVNRPLLEAIGDENSASTSAVL  7813

Query  468   LDSMEHSRDVMKNKEMRVFVDGWWRRHRLIFYNSMIDEKLDRAQGGLQGSGSKYLCTLCH  527
             +  ME  R +++N  M +      R HRL  YNSMIDEKL R+ GGLQGSGS ++CTLCH
Sbjct  7814  MRKMEKERLILQNSIMTIHARTHTRLHRLTIYNSMIDEKLARSSGGLQGSGSHFICTLCH  7993

Query  528   ATRDTARSNVGSFSITRSFEETSDTANYIQTNPDNLSQNELAEFAKGVKDYPVLRMDPDH  587
             AT+ +A++ +GSF I R+  ET  T+ YI TNPDNL+ +ELA  A GVK  P+L  +P
Sbjct  7994  ATKKSAKTQLGSFKIDRTLTETQQTSTYITTNPDNLTPDELATEAGGVKRKPLLTSEPKQ  8173

Query  588   KLIDATHADINLGSFFQKLIVCEIARTYTWEITRDVKVMFEDAEKRFNDHIRASLGLAPT  647
             +L+DATHADI+LG FF+K+IV EIA  + WE + +VK    DAE+R + H+R  LG AP+
Sbjct  8174  QLMDATHADISLGQFFKKIIVREIAGVHKWEASENVKQYIVDAERRLDIHVRELLGTAPS  8353
```

```
Query  648   LMMPGNYARQLFDSRNIDTILELIPDREKKEHLSEVMSLFRKLREVYRAHHPLQADFATY  707
             +MMPGNYAR LF  +N D  LELI + E+K  L  V+  FR LR+VYR H P + +    +
Sbjct  8354  IMMPGNYARALFKEKNEDIFLELIRNEERKALLRSVLQQFRALRKVYREHQPNKKEVQGF  8533

Query  708   KVKAVQMADLLDRHFPYASWPNYLHKIIEHVQEVLEDPRGPGTIGDLSGEGNEAANKLFR  767
             K KAVQM   L  HF Y  WPNYLHKI+EH QE +    GPG+IG LSGEG+EAANKLFR
Sbjct  8534  KKKAVQMGRELLEHFEYVCWPNYLHKILEHTQESILSEDGPGSIGILSGEGSEAANKLFR  8713

Query  768   DLRRNFSRKNCTLDSLRDILWMHWLYTSPKLRRMVIVASRSYKCTRCNDSGHNILTCTSK  827
              LR NFSR+    D LRDILW HWLYTSPKL R+  V  R+Y C+RC   GHNI +C  K
Sbjct  8714  KLRNNFSRRGDVWDGLRDILWFHWLYTSPKLLRLRDVTRRTYTCSRCGAEGHNIKSCNVK  8893
```

WGS: BEXV01001680.1

Summary

Detected configuration: RAG1L

Detected loci	Predicted CDS	start	end	strand	Observations
RAG1L	-	4830	3889	-	incomplete; stop codons

WGS: BEXV01001597.1

Summary

Detected configuration: RAG1L

Detected loci	Predicted CDS	start	end	strand	Observations
RAG1L	- -	41800	39748	-	incomplete; stop codons
