# Supplementary material for: Identification of RAG-like transposons in protostomes suggests their ancient bilaterian origin
Source: Mob DNA. 2020 May 6;11:17. doi: 10.1186/s13100-020-00214-y (PMC7204232; doi:10.1186/s13100-020-00214-y)
Supplement: Supplementary file 9 — Additional file 9: File S2. Identified RAGL loci mapped onto nucleotide sequence. [file 13100_2020_214_MOESM9_ESM.pdf]

File S2. Detected RAG1L & RAG2L loci mapped on nucleic acid sequence

LEGEND

- Target site duplication (TSD)
- Terminal inverted repeat (TIR)
- RAG1L
- RAG2L
- / Problem
- Potentially part of coding region
- RAG1L/RAG2L duplicated sequence

- M Start codon
- * Stop codon

Crassostrea virginica (Cvi)

WGS: MWPT03000007.1

10205871-10212733
Crassostrea virginica isolate RU13XGHG1-28 chromosome 4, whole genome shotgun sequence

```
          E  G  A  D  Q  T  S  R  L  L  L  S  Q  P  C  L  N  T  L  Y  Y  I  C  D  V  T  G  A  F  S  T  P  L  Q  F  E  K  H  Q  I  C  Y  M  R  K  A  C  R  M  F  V  Y  F  R  I  I  A  H  E  R  K  T  E  S  T  L  N    F1
           R  A  R  I  K  L  P  V  S  C  S  V  S  H  V  *  I  H  C  I  T  S  V  T  S  R  V  L  S  A  L  R  F  S  L  K  S  I  R  Y  A  T  C  A  R  R  A  E  C  L  S  T  F  V  S  L  R  M  N  E  K  Q  K  V  H  *  M   F2
            G  R  G  S  N  F  P  S  P  A  Q  S  A  M  F  E  Y  T  V  L  H  L  *  R  H  G  C  F  Q  H  S  A  S  V  *  K  A  S  D  M  L  H  A  Q  G  V  P  N  V  C  L  L  S  Y  H  C  A  *  T  K  N  R  K  Y  I  K    F3
   1 GAGGGCGCGGATCAAACTTCCCGTCTCCTGCTCAGTCAGCCATGTTTGAATACACTGTATTACATCTGTGACGTCACGGGTGCTTTCAGCACTCCGCTTCAGTTTGAAAAGCATCAGATATGCTACATGCGCAAGGCGTGCCGAATGTTTGTCTACTTTCGTATCATTGCGCATGAACGAAAAACAGAAAGTACATTAAA 200
     ----:----|----:----|----:----|----:----|----:----|----:----|----:----|----:----|----:----|----:----|----:----|----:----|----:----|----:----|----:----|----:----|----:----|----:----|----:----|----:----|
   1 CTCCCGCGCCTAGTTTGAAGGGCAGAGGACGAGTCAGTCGGTACAAACTTATGTGACATAATGTAGACACTGCAGTGCCCACGAAAGTCGTGAGGCGAAGTCAAACTTTTCGTAGTCTATACGATGTACGCGTTCCGCACGGCTTACAAACAGATGAAAGCATAGTAACGCGTACTTGCTTTTTGTCTTTCATGTAATTT 200
          S  P  A  S  *  V  E  R  R  R  S  L  *  G  H  K  F  V  S  Y  *  M  Q  S  T  V  P  A  K  L  V  G  S  *  N  S  F  C  *  I  H  *  M  R  L  A  H  R  I  N  T  *  K  R  I  M  A  C  S  R  F  V  S  L  V  N  F   F6
         X  P  R  P  D  F  K  G  D  G  A  *  D  A  M  N  S  Y  V  T  N  C  R  H  R  *  P  H  K  *  C  E  A  E  T  Q  F  A  D  S  I  S  C  A  C  P  T  G  F  T  Q  R  S  E  Y  *  Q  A  H  V  F  F  L  F  Y  M  L    F5
           L  A  R  I  L  S  G  T  E  Q  E  T  L  W  T  Q  I  C  Q  I  V  D  T  V  D  R  T  S  E  A  S  R  K  L  K  F  L  M  L  Y  A  V  H  A  L  R  A  S  H  K  D  V  K  T  D  N  R  M  F  S  F  C  F  T  C  *     F4

          V  E  P  D  A  M  T  L  V  *  I  G  R  K  K  K  I  E  T  V  I  *  N  *  F  C  S  L  P  V  Q  I  L  R  *  K  Y  A  L  V  N  N  N  K  R  I  Y  A  T  F  *  N  F  S  E  K  G  D  N  F  T  K  V  V  *  D  T    F1
           S  N  P  M  P  *  L  *  F  R  S  V  E  R  K  K  L  K  R  *  F  K  I  D  F  V  H  F  R  Y  K  F  *  D  K  S  M  L  S  *  I  I  I  K  E  F  M  L  R  F  E  I  F  L  R  K  A  I  I  L  P  R  S  F  R  T     F2
         C  R  T  R  C  H  D  F  S  L  D  R  S  K  E  K  N  *  N  G  N  L  K  L  I  L  F  T  S  G  T  N  F  E  I  K  V  C  S  R  E  *  *  *  K  N  L  C  Y  V  L  K  F  F  *  E  R  R  *  F  Y  Q  G  R  L  G  H    F3
 201 TGTCGAACCCGATGCCATGACTTTAGTTTAGATCGGTCGAAAGAAAAAAATTGAAACGGTAATTTAAAATTGATTTTGTTCACTTCCGGTACAAATTTTGAGATAAAAGTATGCTCTCGTGAATAATAATAAAAGAATTTATGCTACGTTTTGAAATTTTTCTGAGAAAGGCGATAATTTTACCAAGGTCGTTTAGGACA 400
     ----:----|----:----|----:----|----:----|----:----|----:----|----:----|----:----|----:----|----:----|----:----|----:----|----:----|----:----|----:----|----:----|----:----|----:----|----:----|----:----|
 201 ACAGCTTGGGCTACGGTACTGAAATCAAATCTAGCCAGCTTTCTTTTTTTAACTTTGCCATTAAATTTTAACTAAAACAAGTGAAGGCCATGTTTAAAACTCTATTTTCATACGAGAGCACTTATTATTATTTTCTTAAATACGATGCAAAACTTTAAAAAGACTCTTTCCGCTATTAAAATGGTTCCAGCAAATCCTGT 400
          T  S  G  S  A  M  V  K  T  *  I  P  R  F  F  F  I  S  V  T  I  *  F  Q  N  Q  E  S  G  T  C  I  K  L  Y  F  Y  A  R  T  F  L  L  L  L  I  *  A  V  N  Q  F  K  E  S  F  P  S  L  K  V  L  T  T  *  S    F6
         H  R  V  R  H  W  S  K  L  K  S  R  D  F  S  F  F  Q  F  P  L  K  F  N  I  K  N  V  E  P  V  F  K  S  I  F  T  H  E  R  S  Y  Y  Y  F  F  K  H  *  T  K  F  N  K  Q  S  L  R  Y  N  *  W  P  R  K  P  C    F5
         I  D  F  G  I  G  H  S  *  N  L  D  T  S  L  F  F  N  F  R  Y  N  L  I  S  K  T  *  K  R  Y  L  N  Q  S  L  L  I  S  E  H  I  I  I  F  S  N  I  S  R  K  S  I  K  R  L  F  A  I  I  K  G  L  D  N  L  V    F4

          *  I  Q  K  I  F  F  L  I  G  Y  T  T  D  K  Y  G  Y  T  L  T  E  T  Q  K  S  *  C  R  K  G  E  N  G  R  F  L  A  F  A  E  Q  T  E  V  K  M  R  N  K  *  K  Q  P  P  I  R  L  G  K  A  M  F  L  Y  Y    F1
         H  R  Y  R  K  Y  F  F  *  L  G  T  L  P  I  N  M  G  T  H  *  L  K  L  K  S  L  S  A  E  K  V  K  M  G  V  F  *  P  S  L  N  K  P  K  *  K  *  E  I  N  R  N  S  P  Q  S  D  W  E  R  Q  C  F  Y  I  T    F2
          I  D  T  E  N  I  F  F  N  W  V  H  Y  R  *  I  W  V  H  I  D  *  N  S  K  V  L  V  Q  K  R  *  K  W  A  F  F  S  L  R  *  T  N  R  S  K  N  E  K  *  I  E  T  A  P  N  Q  I  G  K  G  N  V  F  I  L  H   F3
 401 CATAGATACAGAAAATATTTTTTTTAATTGGGTACACTACCGATAAATATGGGTACACATTGACTGAAACTCAAAAGTCTTAGTGCAGAAAAGGTGAAAATGGGCGTTTTTTAGCCTTCGCTGAACAAACCGAAGTAAAAATGAGAAATAAATAGAAACAGCCCCCAATCAGATTGGGAAAGGCAATGTTTTTATATTAC 600
     ----:----|----:----|----:----|----:----|----:----|----:----|----:----|----:----|----:----|----:----|----:----|----:----|----:----|----:----|----:----|----:----|----:----|----:----|----:----|----:----|
 401 GTATCTATGTCTTTTATAAAAAAAATTAACCCATGTGATGGCTATTTATACCCATGTGTAACTGACTTTGAGTTTTCAGAATCACGTCTTTTCCACTTTTACCCGCAAAAAATCGGAAGCGACTTGTTTGGCTTCATTTTTACTCTTTATTTATCTTTGTCGGGGGTTAGTCTAACCCTTTCCGTTACAAAAATATAATG 600
         V  Y  I  C  F  I  N  K  K  I  P  Y  V  V  S  L  Y  P  Y  V  N  V  S  V  *  F  D  *  H  L  F  P  S  F  P  R  K  K  A  K  A  S  C  V  S  T  F  I  L  F  L  Y  F  C  G  G  I  L  N  P  F  A  I  N  K  Y  *    F6
          M  S  V  S  F  I  K  K  L  Q  T  C  *  R  Y  I  H  T  C  M  S  Q  F  E  F  T  K  T  C  F  L  H  F  H  A  N  K  L  R  R  Q  V  F  R  L  L  F  S  F  Y  I  S  V  A  G  L  *  I  P  F  P  L  T  K  I  N     F5
          C  L  Y  L  F  Y  K  K  *  N  P  V  S  G  I  F  I  P  V  C  Q  S  F  S  L  L  R  L  A  S  F  T  F  I  P  T  K  *  G  E  S  F  L  G  F  Y  F  H  S  I  F  L  F  L  G  W  D  S  Q  S  L  C  H  K  *  I  V   F4

         T  R  C  S  G  D  L  C  N  K  I  Y  H  *  K  V  *  F  K  H  *  T  L  *  I  W  R  G  H  S  I  I  H  T  A  C  E  I  L  S  S  L  L  I  V  A  C  Y  P  A  L  S  H  P  N  M  Y  A  P  T  P  L  C  R  C  L  G    F1
          L  G  V  L  V  T  C  V  I  K  Y  I  I  K  K  F  S  L  N  T  K  L  Y  E  Y  G  E  A  T  Q  *  Y  T  Q  L  V  K  Y  Y  L  A  S  *  L  L  H  V  I  L  H  Y  L  I  Q  I  C  M  P  P  P  P  F  A  D  A  *  V   F2
          *  V  F  W  *  L  V  *  *  N  I  S  L  K  S  L  V  *  T  L  N  S  M  N  M  E  R  P  L  N  N  T  H  S  L  *  N  T  I  *  P  L  N  C  C  M  L  S  C  I  I  S  S  K  Y  V  C  P  H  P  P  L  Q  M  P  R     F3
 601 ACTAGGTGTTCTGGTGACTTGTGTAATAAAATATATCATTAAAAAGTTTAGTTTAAACACTAAACTCTATGAATATGGAGAGGCCACTCAATAATACACACAGCTTGTGAAATACTATCTAGCCTCTTAATTGTTGCATGTTATCCTGCATTATCTCATCCAAATATGTATGCCCCCACCCCCCTTTGCAGATGCCTAGG 800
     ----:----|----:----|----:----|----:----|----:----|----:----|----:----|----:----|----:----|----:----|----:----|----:----|----:----|----:----|----:----|----:----|----:----|----:----|----:----|----:----|
 601 TGATCCACAAGACCACTGAACACATTATTTTATATAGTAATTTTTCAAATCAAATTTGTGATTTGAGATACTTATACCTCTCCGGTGAGTTATTATGTGTGTCGAACACTTTATGATAGATCGGAGAATTAACAACGTACAATAGGACGTAATAGAGTAGGTTTATACATACGGGGGTGGGGGAAACGTCTACGGATCC 800
         V  L  H  E  P  S  K  H  L  L  I  Y  *  *  F  T  *  N  L  C  *  V  R  H  I  H  L  P  W  E  I  I  C  V  A  Q  S  I  S  D  L  R  K  I  T  A  H  *  G  A  N  D  *  G  F  I  Y  A  G  V  G  R  Q  L  H  R  P    F6
         C  *  T  N  Q  H  S  T  Y  Y  F  I  D  N  F  L  K  T  *  V  S  F  E  I  F  I  S  L  G  S  L  L  V  C  L  K  H  F  V  I  *  G  R  L  Q  Q  M  N  D  Q  M  I  E  D  L  Y  T  H  G  W  G  G  K  C  I  G  L     F5
          S  P  T  R  T  V  Q  T  I  F  Y  I  M  L  F  N  L  K  F  V  L  S  *  S  Y  P  S  A  V  *  Y  Y  V  C  S  T  F  Y  *  R  A  E  *  N  N  C  T  I  R  C  *  R  M  W  I  H  I  G  G  G  G  K  A  S  A  *     F4

          S  C  C  M  K  M  S  P  Q  M  F  I  N  *  I  Q  Y  N  T  L  S  *  M  L  E  I  I  L  S  E  K  S  Y  *  T  *  F  K  H  L  *  R  I  F  K  H  T  C  T  Y  T  L  I  V  I  R  K  N  F  H  H  I  V  K  T  I  I    F1
           A  A  A  *  K  C  L  H  K  C  L  *  I  K  F  S  I  T  H  F  P  K  C  W  R  *  S  Y  L  K  S  H  I  E  H  N  L  N  I  C  E  E  F  S  N  I  H  V  H  I  H  *  L  *  L  E  K  T  F  T  I  *  *  R  L  *     F2
         *  L  L  H  E  N  V  S  T  N  V  Y  K  L  N  S  V  *  H  T  F  L  N  A  G  D  N  L  I  *  K  V  I  L  N  I  I  *  T  F  V  K  N  F  Q  T  Y  M  Y  I  Y  I  N  C  N  *  K  K  L  S  P  Y  S  K  D  Y  N    F3
 801 TAGCTGCTGCATGAAAATGTCTCCACAAATGTTTATAAATTAAATTCAGTATAACACACTTTCCTAAATGCTGGAGATAATCTTATCTGAAAAGTCATATTGAACATAATTTAAACATTTGTGAAGAATTTTCAAACATACATGTACATATACATTAATTGTAATTAGAAAAAACTTTCACCATATAGTAAAGACTATAA 1000
     ----:----|----:----|----:----|----:----|----:----|----:----|----:----|----:----|----:----|----:----|----:----|----:----|----:----|----:----|----:----|----:----|----:----|----:----|----:----|----:----|
 801 ATCGACGACGTACTTTTACAGAGGTGTTTACAAATATTTAATTTAAGTCATATTGTGTGAAAGGATTTACGACCTCTATTAGAATAGACTTTTCAGTATAACTTGTATTAAATTTGTAAACACTTCTTAAAAGTTTGTATGTACATGTATATGTAATTAACATTAATCTTTTTTGAAAGTGGTATATCATTTCTGATATT 1000
          L  Q  Q  M  F  I  D  G  C  I  N  I  F  *  I  *  Y  L  V  S  E  *  I  S  S  I  I  K  D  S  F  D  Y  Q  V  Y  N  L  C  K  H  L  I  K  L  C  V  H  V  Y  V  N  I  T  I  L  F  F  K  *  W  I  T  F  V  I    F6
          Y  S  S  C  S  F  T  E  V  F  T  *  L  N  F  E  T  Y  C  V  K  R  F  A  P  S  L  R  I  Q  F  T  M  N  F  M  I  *  V  N  T  F  F  K  *  V  Y  M  Y  M  Y  M  L  Q  L  *  F  F  S  E  G  Y  L  L  S  *  L   F5
         T  A  A  A  H  F  H  R  W  L  H  K  Y  I  L  N  L  I  V  C  K  G  L  H  Q  L  Y  D  *  R  F  L  *  I  S  C  L  K  F  M  Q  S  S  N  E  F  M  C  T  C  I  C  *  N  Y  N  S  F  V  K  V  M  Y  Y  L  S  Y    F4

          K  V  S  R  S  I  *  I  L  L  I  F  H  H  I  S  *  *  M  D  F  K  L  T  *  Y  K  M  F  K  E  *  L  *  K  N  F  *  G  C  I  M  H  Q  E  G  K  Y  F  F  K  I  F  S  E  R  *  K  L  S  V  L  L  I  C  G    F1
         S  K  F  Q  E  V  F  E  Y  Y  *  S  S  T  T  Y  L  D  E  W  I  S  S  *  L  N  I  K  C  L  K  N  N  Y  K  K  I  F  K  V  A  S  C  I  R  R  E  N  I  S  S  R  Y  L  V  K  D  E  S  C  L  Y  F  S  F  V  V    F2
         Q  S  F  K  K  Y  L  N  T  T  N  L  P  P  H  I  L  M  N  G  F  Q  V  D  L  I  *  N  V  *  R  I  I  I  K  K  F  L  R  L  H  H  A  S  G  G  K  I  F  L  Q  D  I  *  *  K  M  K  V  V  C  T  S  H  L  W  L    F3
1001 TCAAAGTTTCAAGAAGTATTTGAATACTACTAATCTTCCACCACATATCTTGATGAATGGATTTCAAGTTGACTTAATATAAAATGTTTAAAGAATAATTATAAAAAAATTTTTAAGGTTGCATCATGCATCAGGAGGGAAAATATTTCTTCAAGATATTTAGTGAAAGATGAAAGTTGTCTGTACTTCTCATTTGTGGT 1200
     ----:----|----:----|----:----|----:----|----:----|----:----|----:----|----:----|----:----|----:----|----:----|----:----|----:----|----:----|----:----|----:----|----:----|----:----|----:----|----:----|
1001 AGTTTCAAAGTTCTTCATAAACTTATGATTAGAAGGTGGTGTATAGAACTACTTACCTAAAGTTCAACTGAATTATATTTTACAAATTTCTTATTAATATTTTTTTAAAAATTCCAACGTAGTACGTAGTCCTCCCTTTTATAAAGAAGTTCTATAAATCACTTTCTACTTTCAACAGACATGAAGAGTAAACACCA 1200
         I  L  T  E  L  L  I  Q  I  S  S  I  K  W  W  M  D  Q  H  I  S  K  L  N  V  *  Y  L  I  N  L  S  Y  N  Y  F  F  K  *  P  Q  M  M  C  *  S  P  F  Y  K  K  L  I  N  L  S  L  H  F  N  D  T  S  R  M  Q  P    F6
          *  L  K  L  F  Y  K  F  V  V  L  R  G  G  C  I  K  I  F  P  N  *  T  S  K  I  Y  F  T  *  L  I  I  I  F  F  N  K  L  N  C  *  A  D  P  P  F  I  N  R  *  S  I  *  H  F  I  F  T  T  Q  V  E  *  K  H     F5
          D  F  N  *  S  T  N  S  Y  *  *  D  E  V  V  Y  R  S  S  H  I  E  L  Q  S  L  I  F  H  K  F  F  L  *  L  F  I  K  L  T  A  D  H  M  L  L  S  F  I  E  E  L  Y  K  T  F  S  S  L  Q  R  Y  K  E  N  T  T    F4

         *  S  *  V  N  D  G  Y  R  I  L  I  C  F  K  S  V  R  L  Y  A  V  F  C  I  S  L  S  Q  A  T  G  M  S  T  F  H  S  E  M  *  T  Y  P  W  L  A  T  M  A  A  Y  I  N  T  N  C  L  H  C  L  E  I  M  Q  M  T    F1
          E  V  K  L  M  M  D  T  G  Y  *  F  V  L  N  Q  C  V  Y  M  Q  F  S  A  Y  H  C  R  K  P  R  V  C  L  H  F  I  P  K  C  K  H  T  R  G  L  Q  R  W  L  H  T  *  I  L  I  V  Y  I  V  *  K  *  C  K  *  Q   F2
          K  L  S  *  *  W  I  P  D  I  N  L  F  *  I  S  A  F  I  C  S  F  L  H  I  I  V  A  S  H  G  Y  V  Y  I  S  F  R  N  V  N  I  P  V  A  C  N  D  G  C  I  H  K  Y  *  L  S  T  L  F  R  N  N  A  N  D     F3
1201 TGAAGTTAAGTTAATGATGGATACCGGATATTAATTTGTTTTAAATCAGTGCGTTTATATGCAGTTTTCTGCATATCATTGTCGCAAGCCACGGGTATGTCTACATTTCATTCCGAAATGTAAACATACCCGTGGCTTGCAACGATGGCTGCATACATAAATACTAATTGTCTACATTGTTTAGAAATAATGCAAATGAC 1400
     ----:----|----:----|----:----|----:----|----:----|----:----|----:----|----:----|----:----|----:----|----:----|----:----|----:----|----:----|----:----|----:----|----:----|----:----|----:----|----:----|
1201 ACTTCAATTCAATTACTACCTATGGCCTATAATTAAACAAAATTTAGTCACGCAAATATACGTCAAAAGACGTATAGTAACAGCGTTCGGTGCCCATACAGATGTAAAGTAAGGCTTTACATTTGTATGGGCACCGAACGTTGCTACCGACGTATGTATTTATGATTAACAGATGTAACAAATCTTTATTACGTTTACTG 1400
          Q  L  *  T  L  S  P  Y  R  I  N  I  Q  K  L  D  T  R  K  Y  A  T  K  Q  M  D  N  D  C  A  V  P  I  D  V  N  *  E  S  I  Y  V  Y  G  H  S  A  V  I  A  A  Y  M  F  V  L  Q  R  C  Q  K  S  I  I  C  I  V   F6
         N  F  N  L  *  H  H  I  G  S  I  L  K  N  *  I  L  A  N  I  H  L  K  R  C  I  M  T  A  L  W  P  Y  T  *  M  E  N  R  F  T  F  M  G  T  A  Q  L  S  P  Q  M  C  L  Y  *  N  D  V  N  N  L  F  L  A  F  S    F5
          S  T  L  N  I  I  S  V  P  Y  *  N  T  K  F  *  H  T  *  I  C  N  E  A  Y  *  Q  R  L  G  R  T  H  R  C  K  M  G  F  H  L  C  V  R  P  K  C  R  H  S  C  V  Y  I  S  I  T  *  M  T  *  F  Y  H  L  H     F4

          K  N  I  Q  N  F  I  L  S  T  D  M  E  H  G  L  K  L  K  C  M  C  R  L  C  G  V  K  I  P  I  N  S  K  S  H  P  K  E  N  F  K  S  A  I  T  S  L  Y  N  I  D  I  D  C  D  S  E  N  I  H  P  P  K  I  C  T    F1
          K  I  F  K  I  L  F  F  L  Q  T  W  S  M  D  *  S  *  S  V  C  A  G  S  V  G  *  K  F  P  S  I  Q  S  P  I  P  K  K  T  S  N  L  P  S  P  P  F  T  I  L  T  L  T  V  T  R  R  T  F  T  L  L  K  F  V     F2
         K  K  Y  S  K  F  Y  S  F  Y  R  H  G  A  W  I  K  V  K  V  Y  V  Q  A  L  W  G  E  N  S  H  Q  F  K  V  P  S  Q  R  K  L  Q  I  C  H  H  L  P  L  Q  Y  *  H  *  L  *  L  G  E  H  S  P  S  *  N  L  Y    F3
1401 AAAAAATATTCAAAATTTTATTCTTTCTACAGACATGGAGCATGGATTAAAGTTAAAGTGTATGTGCAGGCTCTGTGGGGTGAAAATTCCCATCAATTCAAAGTCCCATCCCAAAGAAAACTTCAAATCTGCCATCACCTCCCTTTACAATATTGACATTGACTGTGACTCGGAGAACATTCACCCTCCTAAAATTTGTA 1600
     ----:----|----:----|----:----|----:----|----:----|----:----|----:----|----:----|----:----|----:----|----:----|----:----|----:----|----:----|----:----|----:----|----:----|----:----|----:----|----:----|
1401 TTTTTTATAAGTTTTAAAATAAGAAAGATGTCTGTACCTCGTACCTAATTTCAATTTCACATACACGTCCGAGACACCCCACTTTTAAGGGTAGTTAAGTTTCAGGGTAGGGTTTCTTTTGAAGTTTAGACGGTAGTGGAGGGAAATGTTATAACTGTAACTGACACTGAGCCTCTTGTAAGTGGGAGGATTTTAAACAT 1600
          F  F  I  *  F  K  I  R  E  V  S  M  S  C  P  N  F  N  F  H  I  H  L  S  Q  P  T  F  I  G  M  L  E  F  D  W  G  L  S  F  K  L  D  A  M  V  E  R  *  L  I  S  M  S  Q  S  E  S  F  M  *  G  G  L  I  Q     F6
         L  F  Y  E  F  N  *  E  K  *  L  C  P  A  H  I  L  T  L  T  Y  T  C  A  R  H  P  S  F  E  W  *  N  L  T  G  D  W  L  F  S  *  I  Q  W  *  R  G  K  C  Y  Q  C  Q  S  H  S  P  S  C  E  G  E  *  F  K  Y    F5
         C  F  I  N  L  I  K  N  K  R  C  V  H  L  M  S  *  L  *  L  T  H  A  P  E  T  P  H  F  N  G  D  I  *  L  G  M  G  F  F  V  E  F  R  G  D  G  G  K  V  I  N  V  N  V  T  V  R  L  V  N  V  R  R  F  N  T     F4

           A  C  R  L  R  L  T  R  H  S  Q  A  E  D  D  I  E  I  N  L  P  V  L  F  E  F  L  P  H  S  I  N  C  N  I  C  F  V  K  P  G  R  P  K  K  I  L  K  R  K  L  S  V  P  S  Q  S  T  K  N  I  F  I  Q  Q  E    F1
         L  H  V  D  Y  D  *  Q  D  T  A  K  L  K  M  T  L  K  L  I  Y  L  F  C  L  N  S  C  L  I  L  L  T  V  T  F  V  L  *  N  L  A  D  R  R  K  Y  *  K  E  N  *  V  S  H  P  S  L  Q  K  T  F  S  S  N  K  N     F2
         C  M  *  T  T  T  N  K  T  Q  P  S  *  R  *  H  *  N  *  F  T  C  F  V  *  I  L  A  S  F  Y  *  L  *  H  L  F  C  E  T  W  Q  T  E  E  N  I  K  K  K  I  K  C  P  I  P  V  Y  K  K  H  F  H  P  T  R  T    F3
1601 CTGCATGTAGACTACGACTAACAAGACACAGCCAAGCTGAAGATGACATTGAAATTAATTTACCTGTTTTGTTTGAATTCTTGCCTCATTCTATTAACTGTAACATTTGTTTTGTGAAACCTGGCAGACCGAAGAAAATATTAAAAAGAAAATTAAGTGTCCCATCCCAGTCTACAAAAAACATTTTCATCCAACAAGAA 1800
     ----:----|----:----|----:----|----:----|----:----|----:----|----:----|----:----|----:----|----:----|----:----|----:----|----:----|----:----|----:----|----:----|----:----|----:----|----:----|----:----|
1601 GACGTACATCTGATGCTGATTGTTCTGTGTCGGTTCGACTTCTACTGTAACTTTAATTAAATGGACAAAACAAACTTAAGAACGGAGTAAGATAATTGACATTGTAAACAAAACACTTTGGACCGTCTGGCTTCTTTTATAATTTTTCTTTTAATTCACAGGGTAGGGTCAGATGTTTTTTGTAAAAGTAGGTTGTTCTT 1800
         V  A  H  L  S  R  S  V  L  C  L  W  A  S  S  S  M  S  I  L  K  G  T  K  N  S  N  K  G  *  E  I  L  Q  L  M  Q  K  T  F  G  P  L  G  F  F  I  N  F  L  F  N  L  T  G  D  W  D  V  F  F  M  K  M  W  C  S    F6
          Q  M  Y  V  V  V  L  L  V  C  G  L  Q  L  H  C  Q  F  *  N  V  Q  K  T  Q  I  R  A  E  N  *  *  S  Y  C  K  N  Q  S  V  Q  C  V  S  S  F  I  L  F  F  I  L  H  G  M  G  T  *  L  F  C  K  *  G  V  L     F5
          S  C  T  S  *  S  *  C  S  V  A  L  S  F  I  V  N  F  N  I  *  R  N  Q  K  F  E  Q  R  M  R  N  V  T  V  N  T  K  H  F  R  A  S  R  L  F  Y  *  F  S  F  *  T  D  W  G  L  R  C  F  V  N  E  D  L  L  F    F4
```

```
     H S Y H S A N K D S Q D S S R P S T E T R D I T S P E S P S S F T S F S S I V V V P S A S T S C S F C P P V D S P P L P P V D K L S V V   F1
      I L I T A R T K T V K T V Q D L R Q R Q E T S H P L N L L H P S H P L V P L * C H Q L Q H H A P F V P L * I V P L Y L L L I S C Q L L  F2
       F L S Q R E Q R Q S R Q F K T F D R D K R H H I P * I S F I L H I L * F H C S A I S F N I M L L S P C R * S P F T S C * * V V S C     F3
1801 CATTCTTATCACAGCGCGAACAAAGACAGTCAAGACAGTTCAAGACCTTCGACAGAGACAAGAGACATCACATCCCCTGAATCTCCTTCATCCTTCACATCCTTTAGTTCCATTGTAGTGCCATCAGCTTCAACATCATGCTCCTTTTGTCCCCCTGTAGATAGTCCCCCTTTACCTCCTGTTGATAAGTTGTCAGTTGT 2000
     ----:----|----:----|----:----|----:----|----:----|----:----|----:----|----:----|----:----|----:----|----:----|----:----|----:----|----:----|----:----|----:----|----:----|----:----|----:----|----:----|
1801 GTAAGAATAGTGTCGCGCTTGTTTCTGTCAGTTCTGTCAAGTTCTGGAAGCTGTCTCTGTTCTCTGTAGTGTAGGGGACTTAGAGGAAGTAGGAAGTGTAGGAAATCAAGGTAACATCACGGTAGTCGAAGTTGTAGTACGAGGAAAACAGGGGGACATCTATCAGGGGGAAATGGAGGACAACTATTCAACAGTCAACA 2000
      C E * * L A F L S L * S L E L G E V S V L S M V D G S D G E D K V D K L E M T T G D A E V D H E K Q G G T S L G G K G G T S L N D T T   F6
     V N K D C R S C L C D L C N L V K S L S L L C * M G Q I E K M R * M R * N W Q L A M L K L M M S R K D G Q L Y D G K V E Q Q Y T T L Q   F5
       M R I V A R V F V T L V T * S R R C L C S V D C G R F R R * G E C G K T G N Y H W * S * C * A G K T G R Y I T G R * R R N I L Q * N   F4

      E S C S S E T A P E M F K N V V I R S I P T E R F C N A E V S Q S F M C T I C R G V P C D P Y I S K C S H I F C K E C I F G W F S L S  F1
       S H V L L R L H L K C S K M L * L G V F L Q S D S A M Q K F H S L L C V L F V E V S L V I H I F P N V A T F F A R N V F L D G F L C  F2
     * V M F F * D C T * N V Q K C C N * E Y S Y R A I L Q C R S F T V F Y V Y Y L S R C P L * S I Y F Q M * P H F L Q G M Y F W M V F S V  F3
2001 TGAGTCATGTTCTTCTGAGACTGCACCTGAAATGTTCAAAAATGTTGTAATTAGGAGTATTCCTACAGAGCGATTCTGCAATGCAGAAGTTTCACAGTCTTTTATGTGTACTATTTGTCGAGGTGTCCCTTGTGATCCATATATTTCCAAATGTAGCCACATTTTTTGCAAGGAATGTATTTTTGGATGGTTTTCTCTGT 2200
     ----:----|----:----|----:----|----:----|----:----|----:----|----:----|----:----|----:----|----:----|----:----|----:----|----:----|----:----|----:----|----:----|----:----|----:----|----:----|----:----|
2001 ACTCAGTACAAGAAGACTCTGACGTGGACTTTACAAGTTTTTACAACATTAATCCTCATAAGGATGTCTCGCTAAGACGTTACGTCTTCAAAGTGTCAGAAAATACACATGATAAACAGCTCCACAGGGAACACTAGGTATATAAAGGTTTACATCGGTGTAAAAAACGTTCCTTACATAAAAACCTACCAAAAGAGACA 2200
       S D H E E S V A G S I N L F T T I L L I G V S R N Q L A S T E C D K I H V I Q R P T G Q S G Y I E L H L W M K Q L S H I K P H N E R   F6
      Q T M N K Q S Q V Q F T * F H Q L * S Y E * L A I R C H L L K V T K * T Y * K D L H G K H D M Y K W I Y G C K K C P I Y K Q I T K E T  F5
     N L * T R R L S C R F H E F I N Y N P T N R C L S E A I C F N * L R K H T S N T S T D R T I W I N G F T A V N K A L F T N K S P K R Q   F4

      S A C P V C R S L L D E S E V S P L H G H L L Q I Y E T L L V H C I H A N C T Q S H L I R N I D E H E S I C S M K G T K F L Y N V T   F1
     Q V H V L Y V V L C L M R V R C P P S M V I F F K F M K L C * C I V F M Q T V H S R I * L E I L M N N M K V S V Q * K A Q N F C T M L Q   F2
      K C M S C M S F S A * * E * G V P P P W S S S S N L * N F V S A L Y S C K L Y T V A F D * K Y * * T * K Y L F N E R H K I F V Q C Y N  F3
2201 CAAGTGCATGTCTGTATGTCGTCTCTGCTTGATGAGAGTGAGGTGTCCCCCCTCCATGGTCATCTTCTTCAAATTTATGAAACTTTGTTAGTGCATTGTATTCATGCAAACTGTACACAGTCGCATTTGATTAGAAATATTGATGAACATGAAAGTATCTGTTCAATGAAAGGCACAAAATTTTTGTACAATGTTACA 2400
     ----:----|----:----|----:----|----:----|----:----|----:----|----:----|----:----|----:----|----:----|----:----|----:----|----:----|----:----|----:----|----:----|----:----|----:----|----:----|----:----|
2201 GTTCACGTACAGGACATACAGCAGAGACGAACTACTCTCACTCCACAGGGGGGAGGTACCAGTAGAAGAAGTTTAAATACTTTGAAACAATCACGTAACATAAGTACGTTTGACATGTGTCAGCGTAAACTAATCTTTATAACTACTTGTACTTTCATAGACAAGTTACTTTCCGTGTTTTAAAAACATGTTACAATGT 2400
     D L A H G T H R E R S S S L S T D G R W P * R R * I * S V K N T C Q I * A F Q V C D C K I L F I S S C S L I Q E I F P V F N K Y L T V   F6
      L H M D Q I D N E A Q H S H P T G G G H D D E E F K H F K T L A N Y E H L S Y V T A N S * F Y Q H V H F Y R N L S L C L I K T C H *  F5
       * T C T R Y T T R Q K I L T L H G G E M T M K K L N I F S Q * H M T N M C V T C L R M Q N S I N I F M F T D T * H F A C F K Q V I N C  F4

      T K S R V F K L P L H S V S A K H T R H R R L K P I I S Q V N E F C N A Q E E N K S D V L F F M L K D H L K E I N D P R W K Q V E S L  F1
      Q N L G C S N Y H F I L F L L S I R A I D V * N P * F H K * M N F V M H K R K I S L M F C F L C * K T I * R K L M I H V G N K L R V C  F2
       K I * G V Q I T T S F C F C * A Y A P * T F E T H N F T S K * I L * C T R G K * V * C F V F Y V E R P F E G N * * S T L E T S * E F  F3
2401 ACAAAATCTAGGGTGTTCAAATTACCACTTCATTCTGTTTCTGCTAAGCATACGCGCCATAGACGTTTGAAACCCATAATTTCACAAGTAAATGAATTTTGTAATGCACAAGAGGAAAATAAGTCTGATGTTTTGTTTTTTATGTTGAAAGACCATTTGAAGGAAATTAATGATCCACGTTGGAAACAAGTTGAGAGTTT 2600
     ----:----|----:----|----:----|----:----|----:----|----:----|----:----|----:----|----:----|----:----|----:----|----:----|----:----|----:----|----:----|----:----|----:----|----:----|----:----|----:----|
2401 TGTTTTAGATCCCACAAGTTTAATGGTGAAGTAAGACAAAGACGATTCGTATGCGCGGTATCTGCAAACTTTGGGTATTAAAGTGTTCATTTACTTAAAACATTACGTGTTCTCCTTTTATTCAGACTACAAAACAAAAAATACAACTTTCTGGTAAACTTCCTTTAATTACTAGGTGCAACCTTTGTTCAACTCTCAAA 2600
      V F D L T N L N G S * E T E A L C V R W L R K F G M I E C T F S N Q L A C S S F L D S T K N K I N F S W K F S I L S G R Q F C T S L K  F6
     L L I * P T * I V V E N Q K Q * A Y A G Y V N S V W L K V L L H I K Y H V L P F Y T Q H K T K * T S L G N S P F * H D V N S V L Q S N   F5
       C F R P H E F * W K M R N R S L M R A M S T Q F G Y N * L Y I F K T I C L L F I L R I N Q K K H Q F V M Q L F N I I W T P F L N L T  F4

      W L G N N S T L S P E Q C L A L R V D L L Q S K G Q Y R S Q Y D F L S Q N N V H V F Q A P S K M E S C E N L F M P S A S I F Q I I D N  F1
       G * G I I A L F L Q N N V * P L E L T F Y K A R D N T D P S M T S F P K I M Y M F F K P L V K W K V V R I C L C L L R L F F K L L I  F2
     V V R E * * H S F S R T M F S P * S * P F T K Q G T I Q I P V * L P F P K * C T C F S S P * * N G K L * E F V Y A F C V Y F S N Y * *  F3
2601 GTGGTTAGGGAATAATAGCACTCTTTCTCCAGAACAATGTTTAGCCCTTAGAGTTGACCTTTTACAAAGCAAGGGACAATACAGATCCCAGTATGACTTCCTTTCCCAAAATAATGTACATGTTTTTCAAGCCCCTAGTAAAATGGAAAGTTGTGAGAATTTGTTTATGCCTTCTGCGTCTATTTTTCAAATTATTGATA 2800
     ----:----|----:----|----:----|----:----|----:----|----:----|----:----|----:----|----:----|----:----|----:----|----:----|----:----|----:----|----:----|----:----|----:----|----:----|----:----|----:----|
2601 CACCAATCCCTTATTATCGTGAGAAAGAGGTCTTGTTACAAATCGGGAATCTCAACTGGAAAATGTTTCGTTCCCTGTTATGTCTAGGGTCATACTGAAGGAAAGGGTTTTATTACATGTACAAAAAGTTCGGGGATCATTTTACCTTTCAACACTCTTAAACAAATACGGAAGACGCAGATAAAAAGTTTAATAACTAT 2800
       H N P F L L V R E G S C H K A R L T S R K C L L P C Y L D W Y S K R E W F L T C T K * A G L L I S L Q S F K N I G E A D I K * I I S  F6
      T T L S Y Y C E K E L V I N L G * L Q G K V F C P V I C I G T H S G K G F Y H V H K E L G * Y F P F N H S N T * A K Q T * K E F * Q Y F5
     Q P * P I I A S K R W F L T * G K S N V K * L A L S L V S G L I V E K G L I I Y M N K L G R T F H F T T L I Q K H R R R R N K L N N I  F4

       D G N V L L Q N S E N P C T E P L N V N E C F L P G F V E L A T P N C M G V R F S Y F E A L S L T L Q E L E P E I L F G L K K H G L  F1
     M M A M Y C Y K I Q K I H A Q S L * M L M N V F C Q V L L S W Q L Q T V W V L D F P I L R H S L L P C K N * S L R F Y L D * K N M G *  F2
      * W Q C I V T K F R K S M H R A F E C * * M F F A R F C * V G N S K L Y G C * I F L F * G T L S Y L A R T R A * D F I W I E K T W V K  F3
2801 ATGATGGCAATGTATTGTTACAAAATTCAGAAAATCCATGCACAGAGCCTTTGAATGTTAATGAATGTTTTTTGCCAGGTTTTGTTGAGTTGGCAACTCCAAACTGTATGGGTGTTAGATTTTCCTATTTTGAGGCACTCTCTCTTACCTTGCAAGAACTAGAGCCTGAGATTTTATTTGGATTGAAAAAACATGGGTTA 3000
     ----:----|----:----|----:----|----:----|----:----|----:----|----:----|----:----|----:----|----:----|----:----|----:----|----:----|----:----|----:----|----:----|----:----|----:----|----:----|----:----|
2801 TACTACCGTTACATAACAATGTTTTAAGTCTTTTAGGTACGTGTCTCGGAAACTTACAATTACTTACAAAAAACGGTCCAAAACAACTCAACCGTTGAGGTTTGACATACCCACAATCTAAAAGGATAAAACTCCGTGAGAGAGAATGGAACGTTCTTGATCTCGGACTCTAAAATAAACCTAACTTTTTTGTACCCAAT 3000
     L S P L T N N C F E S F G H V S G K F T L S H K G P K T S N A V G F Q I P T L N E * K S A S E R V K C S S S G S I K N P N F F C P N   F6
       H H C H I T V F N L F D M C L A K S H * H I N K A L N Q Q T P L E L S Y P H * I K R N Q P V R E * R A L V L A Q S K I Q I S F V H T  F5
      I I A I Y Q * L I * F I W A C L R Q I N I F T K Q W T K N L Q C S W V T H T N S K G I K L C E R K G Q L F * L R L N * K S Q F F M P *  F4

     N I E D V L F L T T V K D G C D G M G E V S V Y K E K D F K M L P D K V F R F S F C I V K I Q A E Y D G K L F D V F T E P L P N S V R   F1
      I L K M F F F L L L K M G V M E W G R S L F T K K K I S K C C L T R F F V F P F V L * K F R L N M T E N F L M F L N H C Q T V L E  F2
       Y * R C S F S Y Y C * R W V * W N G G G L C L Q R K R F Q N V A * Q G F S F F L L Y C E N S G * I * R K T F * C F Y * T I A K Q C *  F3
3001 AATATTGAAGATGTTCTTTTTCTTACTACTGTTAAAGATGGGTGTGATGGAATGGGGGAGGTCTCTGTTTACAAAGAAAAAGATTTCAAAATGTTGCCTGACAAGGTTTTTCGTTTTTCCTTTTGTATTGTGAAAATTCAGGCTGAATATGACGGAAAACTTTTTGATGTTTTTACTGAACCATTGCCAAACAGTGTTAG 3200
     ----:----|----:----|----:----|----:----|----:----|----:----|----:----|----:----|----:----|----:----|----:----|----:----|----:----|----:----|----:----|----:----|----:----|----:----|----:----|----:----|
3001 TTATAACTTCTACAAGAAAAAGAATGATGACAATTTCTACCCACACTACCTTACCCCCTCCAGAGACAAATGTTTCTTTTTCTAAAGTTTTACAACGGACTGTTCCAAAAAGCAAAAAGGAAAACATAACACTTTTAAGTCCGACTTATACTGCCTTTTGAAAAACTACAAAAATGACTTGGTAACGGTTTGTCACAATC 3200
       F I S S T R K R V V T L S P H S P I P S T E T * L S F S K L I N G S L T K R K E K Q I T F I * A S Y S P F S K S T K V S G N G F L T L  F6
      L Y Q L H E K E * * Q * L H T H H F P P P R Q K C L F L N * F T A Q C P K E N K R K Y Q S F E P Q I H R F V K Q H K * Q V M A L C H *  F5
       I N F I N K K K S S N F I P T I S H P L D R N V F F I E F H Q R V L N K T K G K T N H F N L S F I V S F K K I N K S F W Q W V T N  F4

      T N R P L L E S I S D E N N Q V S N V V C I L P I E N E R E I L M Q N R M H V K T K E G W M F H K F S F F N S M V D E K R D R G D S G  F1
       Q I D L F * N Q F L M K T I K F Q M L S V F Y L L K M K G K F * C K I A C M * K Q K R G G C S T N F H F L I L W L M R K E T G G L  F2
     N K * T S F R I N F * * K Q S S F K C C L F Y T Y * K * K G N F N A K S H A C E N K R G V D V P Q I F I F * F Y G * * E K R Q G G L W  F3
3201 AACAAATAGACCTCTTTTAGAATCAATTTCTGATGAAAACAATCAAGTTTCAAATGTTGTCTGTATTTTACCTATTGAAAATGAAAGGGAAATTTTAATGCAAAATCGCATGCATGTGAAAACAAAGGAGGGGTGGATGTTCCACAAATTTTCATTTTTAATTCTATGGTTGATGAGAAAAGAGACAGGGGGGACTCTG 3400
     ----:----|----:----|----:----|----:----|----:----|----:----|----:----|----:----|----:----|----:----|----:----|----:----|----:----|----:----|----:----|----:----|----:----|----:----|----:----|----:----|
3201 TTGTTTATCTGGAGAAAATCTTAGTTAAAGACTACTTTTGTTAGTTCAAAGTTTACAACAGACATAAAATGGATAACTTTTACTTTCCCTTTAAAATTACGTTTTAGCGTACGTACACTTTTGTTTTCTCCCCACCTACAAGGTGTTTAAAAGTAAAAATTAAGATACCAACTACTCTTTTCTCTGTCCCCCCTGAGAC 3400
       V F L G R K S D I E S S F L T E F T T Q I K G I S F S L S I K I C F R M C T F V F S P H I N W L N E N K L E I T S S F L S L P S E  F6
      F L Y V E K L I L K Q H F C D L K L H Q R Y K V * Q F H F P F K L A F D C A H S F L L P T S T G C I K M K * N * P Q H S F L C P P S Q  F5
     S C I S R K * F * N R I F V I L N * I N D T N * R N F I F P F N * H L I A H M H F C F L P P H E V F K * K K I R H N I L F S V P P V R   F4

       L Q G S G S K Y L C T L C D A D K Q S A K A L L G S F S I N R S V S E C S N I A E I L R V N P N A L S E N E L K K I S K G V K C A P  F1
     V F K V V V Q N I Y V L Y V M Q I N N L Q K H Y L G L F Q L T G Q F L N V Q I L L K F Y V L T Q M L C L K M N * K K F Q K V * N V L L  F2
      S S R * W F K I F M Y F M * C R * T I C K S I T W V F F N * Q V S F * M F K Y C * N F T C * P K C F V * K * I E K N F K R C K M C S S  F3
3401 GTCTTCAAGGTAGTGGTTCAAAATATTTATGTACTTTATGTGATGCAGATAAACAATCTGCAAAAGCATTACTTGGGTCTTTTTCAATTAACAGGTCAGTTTCTGAATGTTCAAATATTGCTGAAATTTTACGTGTTAACCCAAATGCTTTGTCTGAAAATGAATTGAAAAAAATTTCAAAGGTGTAAAATGTGCTCCT 3600
     ----:----|----:----|----:----|----:----|----:----|----:----|----:----|----:----|----:----|----:----|----:----|----:----|----:----|----:----|----:----|----:----|----:----|----:----|----:----|----:----|
3401 CAGAAGTTCCATCACCAAGTTTTATAAATACATGAAATACACTACGTCTATTTGTTAGACGTTTTCGTAATGAACCCAGAAAAAGTTAATTGTCCAGTCAAAGACTTACAAGTTTATAACGACTTTAAAATGCACAATTGGGTTTACGAAACAGACTTTTACTTAACTTTTTTTAAAGTTTTCCACATTTTACACGAGGA 3600
      P R * P L P E F Y K H V K H S A S L C D A F A N S P D K E I L L D T E S H E F I A S I K R T L G F A K D S F S N F F I E F P T F H A G  F6
       D E L Y H N L I N I Y K I H H L Y V I Q L L M V Q T K K L * C T L K Q I N L Y Q Q F K V H * G L H K T Q F H I S F F K L L H L I H E  F5
     T K L T T T * F I * T S * T I C I F L R C F C * K P R K * N V P * N R F T * I N S F N * T N V W I S Q R F I F Q F F N * F T Y F T S R   F4

     L S K I E P I Q K G I D A T H A D I N L G Q F F K K I I V R E I A G V T K W E L T Q D V K P L V Q N A E F L F D Q H M K C N C G I N P   F1
      F Q K * N Q F R K A * M P L M Q I * I * D S F L R K L L Y V K L Q V * Q N G N L H K M * N L * F K M L N F C L I N I * N V T V G * I L  F2
       F K N R T N S E R H R C H S C R Y K F R T V F * E N Y C T * N C R C N K M G T Y T R C K T F S S K C * I F V * S T Y E M * L W D K S  F3
3601 CTTTCAAAAATAGAACCAATTCAGAAAGGCATAGATGCCACTCATGCAGATATAAATTTAGGACAGTTTTTTAAGAAAATTATTGTACGTGAAATTGCAGGTGTAACAAAATGGGAACTTACACAAGATGTAAAACCTTTAGTTCAAAATGCTGAATTTTTGTTTGATCAACATATGAAATGTAACTGTGGGATAAATCC 3800
     ----:----|----:----|----:----|----:----|----:----|----:----|----:----|----:----|----:----|----:----|----:----|----:----|----:----|----:----|----:----|----:----|----:----|----:----|----:----|----:----|
3601 GAAAGTTTTTATCTTGGTTAAGTCTTTCCGTATCTACGGTGAGTACGTCTATATTTAAATCCTGTCAAAAAATTCTTTTAATAACATGCACTTTAACGTCCACATTGTTTTACCCTTGAATGTGTTCTACATTTTGGAAATCAAGTTTTACGACTTAAAAACAAACTAGTTGTATACTTTACATTGACACCCTATTTAGG 3800
       R E F I S G I * F P M S A V * A S I F K P C N K L F I I T R S I A P T V F H S S V C S T F G K T * F A S N K N S * C I F H L Q P I F G  F6
     E K L F L V L E S L C L H W E H L Y L N L V T K * S F * Q V H F Q L H L L I P V * V L H L V K L E F H Q I K T Q D V Y S I Y S H S L D   F5
      K * F Y F W N L F A Y I G S M C I Y I * S L K K L F N N Y T F N C T Y C F P F K C L I Y F R * N L I S F K Q K I L M H F T V T P Y I  F4

       Q L M M P G N Y A R T L F E T P H S V L L E H I S D S V R K E N L S S I L N I F L H L R K V Y R C K D P L T E C P F D V Q N Y K K C A  F1
      S L * C L V I M P E P F L K L P I L F C * N I F Q I L S G K R I C L L F * T Y F Y T F G K C I D V K T L * Q N V L L M Y K I T R N V  F2
      S A Y D A W * L C Q N P F * N S P F C F V R T Y F R F C Q E R E F V F Y F E H I F T P S E S V * M * R P S D R M S F * C T K L Q E M C  F3
3801 TCAGCTTATGATGCCTGGTAATTATGCCAGAACCCTTTTTGAAACTCCCCATTCTGTTTTGTTAGAACATATTTCAGATTCTGTCAGGAAAGAGAATTTGTCTTCTATTTTGAACATATTTTTACACCTTCGGAAAGTGTATAGATGTAAAGACCCTCTGACAGAATGTCCTTTTGATGTACAAAATTACAAGAAATGTG 4000
     ----:----|----:----|----:----|----:----|----:----|----:----|----:----|----:----|----:----|----:----|----:----|----:----|----:----|----:----|----:----|----:----|----:----|----:----|----:----|----:----|
3801 AGTCGAATACTACGGACCATTAATACGGTCTTGGGAAAAACTTTGAGGGGTAAGACAAAACAATCTTGTATAAAGTCTAAGACAGTCCTTTCTCTTAAACAGAAGATAAAACTTGTATAAAAATGTGGAAGCCTTTCACATATCTACATTTCTGGGAGACTGTCTTACAGGAAAACTACATGTTTTAATGTTCTTTACAC 4000
       * S I I G P L * A L V R K S V G W E T K N S C I E S E T L F S F K D E I K F M N K C R R F T Y L H L S G R V S H G K S T C F * L F H  F6
      E A * S A Q Y N H W F G K Q F E G N Q K T L V Y K L N Q * S L S N T K * K S C I K V G E S L T I Y L G E S L I D K Q H V F N C S I H  F5
     R L K H H R T I I G S G K K F S G M R N Q * F M N * I R D P F L I Q R K N Q V Y K * V K P F H I S T F V R Q C F T R K I Y L I V L F T   F4

       V E M G A L L L Q H F D Y V E W P N Y L H K V I E H V Q Q L I E D P N G P G S V G A F S S E G N E A G N K L F R H F R K N L S R R G  F1
     L L K W G P F Y F N I L I M * S G P I I Y T K S L N M S S N L L K I Q M G L G Q Q L G L S V V K A M R L A T N Y F V I L E K I S L V V E   F2
      C * N G G P F T S T F * L C R V A Q L F T Q S H * T C P A T Y * R S K W A W V S W G F Q * * R Q * G W Q Q T I S S F * K K S L S S W K  F3
4001 CTGTTGAAATGGGGGCCCTTTTACTTCAACATTTTGATTATGTAGAGTGGCCCAATTATTTACACAAAGTCATTGAACATGTCCAGCAACTTATTGAAGATCCAAATGGGCCTGGGTCAGTTGGGGCTTTCAGTAGTGAAGGCAATGAGGCTGGCAACAAACTATTTCGTCATTTTAGAAAAAATCTCTCTCGTCGTGGA 4200
     ----:----|----:----|----:----|----:----|----:----|----:----|----:----|----:----|----:----|----:----|----:----|----:----|----:----|----:----|----:----|----:----|----:----|----:----|----:----|----:----|
4001 GACAACTTTACCCCCGGGAAAATGAAGTTGTAAAACTAATACATCTCACCGGGTTAATAAATGTGTTTCAGTAACTTGTACAGGTCGTTGAATAACTTCTAGGTTTACCCGGACCCAGTCAACCCCGAAAGTCATCACTTCCGTTACTCCGACCGTTGTTTGATAAAGCAGTAAAATCTTTTTTAGAGAGAGCAGCACCT 4200
      A T S I P A R K S * C K S * T S H G L * K C L T M S C T W C S I S S G F P G P D T P A K L L S P L S A P L L S N R * K L F F R E R R P  F6
       Q Q F P P G K V E V N Q N H L T A W N N V C L * Q V H G A V * Q D L H A Q T L Q P K * Y H L C H P Q C C V I E D N * F F D R E D H  F5
      S N F H P G K * K L M K I I Y L P G I I * V F D N F M D L L K N F I W I P R P * N P S E T T F A I L S A V F * K T M K S F I E R T T S  F4

      N T Y G S L C D V L K L H W L Y S S K A L F K L A E V E H K K V R C S L C F T S G H N K R T C P L L N T S L * I L I S C L R L [redacted] * Y I  F1
       T R M G V C V M F * N F T G C I V A R L F L S L L K L S I R K F V V L Y V L L L V T T R E L A H F * I L V C K S * S V V * D Y D N T L  F2
      H V W E F V * C F E T S L A V * * Q G S F * A C * S * A * E S S L F F M F Y F W S Q Q E N L P T S E Y * F V N L N Q L F K I M I I H  F3
4201 AACACGTATGGGAGTTTGTGTGATGTTTTGAAACTTCACTGGCTGTATAGTAGCAAGGCTCTTTTTAAGCTTGCTGAAGTTGAGCATAAGAAAGTTCGTTGTTCTTTATGTTTTACTTCTGGTCACAACAAGAGAACTTGCCCACTTCTGAATACTAGTTTGTAAATCTTAATCAGTTGTTTAAGATTATGATAATACAT 4400
     ----:----|----:----|----:----|----:----|----:----|----:----|----:----|----:----|----:----|----:----|----:----|----:----|----:----|----:----|----:----|----:----|----:----|----:----|----:----|----:----|
4201 TTGTGCATACCCTCAAACACACTACAAAACTTTGAAGTGACCGACATATCATCGTTCCGAGAAAAATTCGAACGACTTCAACTCGTATTCTTTCAAGCAACAAGAAATACAAAATGAAGACCAGTGTTGTTCTCTTGAACGGGTGAAGACTTATGATCAAACATTTAGAATTAGTCAACAAATTCTAATACTATTATGTA 4400
      F V Y P L K H S T K F S * Q S Y L L A R K L S A S T S C L F T R Q E K H K V E P * L L V Q G S R F V L K Y I K I L Q K L N H Y Y M  F6
     F C T H S N T H H K S V E S A T Y Y C P E K * A Q Q L Q A Y S L E N N K I N * K Q D C C S F K G V E S Y * N T F R L * N N L I I I C  F5
      V R I P T Q T I N Q F K V P Q I T A L S K K L K S F N L M L F N T T R * T K S R T V V L S S A W K Q I S T Q L D * D T T * S * S L V  F4
```

```
     A F Y F I K Y * * S * * N L L H F S N L H V L Y Y M * L N N I C I M L Y V S S R G I Y K H T R S L * L * K F T S I C I * I Y I * V C I   F1
      H F I S L S I D D H N R I C Y T F L I Y M Y Y I T C N * T T Y V * C Y M S V L E V F T S I Q E V Y S Y K N L P V Y V Y R Y T Y R C V    F2
     C I L F H * V L M I I I E F V T L F * F T C T I L H V I K Q H M Y N V I C Q F * R Y L Q A Y K K F I V I K I Y Q Y M Y I D I H I G V Y   F3
4401 TGCATTTTATTTCATTAAGTATTGATGATCATAATAGAATTTGTTACACTTTTCTAATTTACATGTACTATATTACATGTAATTAAACAACATATGTATAATGTTATATGTCAGTTCTAGAGGTATTTACAAGCATACAAGAAGTTTATAGTTATAAAAATTTACCAGTATATGTATATAGATATACATATAGGTGTGTA 4600
     ----:----|----:----|----:----|----:----|----:----|----:----|----:----|----:----|----:----|----:----|----:----|----:----|----:----|----:----|----:----|----:----|----:----|----:----|----:----|----:----|
4401 ACGTAAAATAAAGTAATTCATAACTACTAGTATTATCTTAAACAATGTGAAAAGATTAAATGTACATGATATAATGTACATTAATTTGTTGTATACATATTACAATATACAGTCAAGATCTCCATAAATGTTCGTATGTTCTTCAAATATCAATATTTTTAAATGGTCATATACATATATCTATATGTATATCCACACAT 4600
      A N * K M L Y Q H D Y Y F K N C K E L K C T S Y * M Y N F L M H I I N Y T L E L P I * L C V L L K Y N Y F N V L I H I Y I Y M Y T H   F6
     Q M K N * * T N I I M I S N T V S K * N V H V I N C T I L C C I Y L T I H * N * L Y K C A Y L F N I T I F I * W Y I Y I S I C I P T Y   F5
     N C K I E N L I S S * L L I Q * V K R I * M Y * I V H L * V V Y T Y H * I D T R S T N V L M C S T * L * L F K G T Y T Y L Y V Y L H T   F4

      S R * Y R Y R C I N M I H Y L D L H L L V C I I S I C M S H C I H S L C L Y K * I N T N M Q I L K H Y Y N L I S L F K K Y E I Y N K   F1
     Y L D D I D I D V L I * Y I T * T F I F L Y V L F Q Y A C H I V Y I H Y V Y T N R * I Q T C R Y * N T T I I L F L C S K S M K Y T T R   F2
      I * M I * I * M Y * Y D T L L R P S S S C M Y Y F N M H V T L Y T F T M S I Q I D K Y K H A D I E T L L * S Y F F V Q K V * N I Q Q E   F3
4601 TATCTAGATGATATAGATATAGATGTATTAATATGATACATTACTTAGACCTTCATCTTCTTGTATGTATTATTTCAATATGCATGTCACATTGTATACATTCACTATGTCTATACAAATAGATAAATACAAACATGCAGATATTGAAACACTACTATAATCTTATTTCTTTGTTCAAAAAGTATGAAATATACAACAAG 4800
     ----:----|----:----|----:----|----:----|----:----|----:----|----:----|----:----|----:----|----:----|----:----|----:----|----:----|----:----|----:----|----:----|----:----|----:----|----:----|----:----|
4601 ATAGATCTACTATATCTATATCTACATAATTATACTATGTAATGAATCTGGAAGTAGAAGAACATACATAATAAAGTTATACGTACAGTGTAACATATGTAAGTGATACAGATATGTTTATCTATTTATGTTTGTACGTCTATAACTTTGTGATGATATTAGAATAAAGAAACAAGTTTTTCATACTTTATATGTTGTTC 4800
      I D L H Y L Y L H I L I I C * K S R * R R T H I I E I H M D C Q I C E S H R Y L Y I F V F M C I N F C * * L R I E K N L F Y S I Y L L   F6
       I * I I Y I Y I Y * Y S V N S L G E D E Q I Y * K L I C T V N Y Y V N V I D I C I S L Y L C A S I S V S S Y D * K K T * F T H F I C C   F5
      Y R S S I S I S T N I H Y M V * V K M K K Y T N N * Y A H * M T Y M * * T * V F L Y I C V H L Y Q F V V I I K N R Q E F L I F Y V V L   F4

     K Y * H T D * R L T L Y K R F S L L T S G G L G T C G T N K I F P T H V N I * N A N T K L M * P S F T S T F T L N P R Y R G C F N R F   F1
      S T N T Q T E D * L C T R D F L F L P L E D L E H V V Q I K F F R L M S I S K T P T Q N * C N H L L Q A P S H * I Q D I G D V S T D S   F2
       V L T H R L K I N S V Q E I F S S Y L W R T W N M W Y K * N F S D S C Q Y L K R Q H K T D V T I F Y K H L H I E S K I * G M F Q Q I   F3
4801 AAGTACTAACACACAGACTGAAGATTAACTCTGTACAAGAGATTTTCTCTTCTTACCTCTGGAGGACTTGGAACATGTGGTACAAATAAAATTTTTCCGACTCATGTCAATATCTAAAACGCCAACACAAAACTGATGTAACCATCTTTTACAAGCACCTTCACATTGAATCCAAGATATAGGGGATGTTTCAACAGATT 5000
     ----:----|----:----|----:----|----:----|----:----|----:----|----:----|----:----|----:----|----:----|----:----|----:----|----:----|----:----|----:----|----:----|----:----|----:----|----:----|----:----|
4801 TTCATGATTGTGTGTCTGACTTCTAATTGAGACATGTTCTCTAAAAGAGAAGAATGGAGACCTCCTGAACCTTGTACACCATGTTTATTTTAAAAAGGCTGAGTACAGTTATAGATTTTGCGGTTGTGTTTTGACTACATTGGTAGAAAATGTTCGTGGAAGTGTAACTTAGGTTCTATATCCCCTACAAAGTTGTCTAA 5000
      F Y * C V S Q L N V R Y L L N E R R V E P P S P V H P V F L I K G V * T L I * F A L V F S I Y G D K V L V K V N F G L Y L P H K L L N   F6
     S T S V C L S F I L E T C S I K E E * R Q L V Q F M H Y L Y F K E S E H * Y R F R W C L V S T V M K * L C R * M S D L I Y P I N * C I   F5
       L V L V C V S S * S Q V L S K R K K G R S S K S C T T C I F N K R S M D I D L V G V C F Q H L W R K C A G E C Q I W S I P S T E V S   F4

     N N F T F S I K I T W T W H H L L T T K N K I L * I H T T T Y H Q T I I R K C T N * M T S * P K I S V K L D I F T K K C V F I * N * H   F1
      I I L H S V S K S H G L G T I C L L L K T K Y C K F I P P H I I K Q S S E S V Q T E * P A D L K Y R S N W T F S Q R N V S L F K I D   F2
     Q * F Y I Q Y Q N H M D L A P F A Y Y * K Q N I V N S Y H H I S S N N H Q K V Y K L N D Q L T * N I G Q T G H F H K E M C L Y L K L T   F3
5001 CAATAATTTTACATTCAGTATCAAAATCACATGGACTTGGCACCATTTGCTTACTACTAAAAACAAAATATTGTAAATTCATACCACCACATATCATCAAACAATCATCAGAAAGTGTACAAACTGAATGACCAGCTGACCTAAAATATCGGTCAAACTGGACATTTTCACAAAGAAATGTGTCTTTATTTAAAATTGAC 5200
     ----:----|----:----|----:----|----:----|----:----|----:----|----:----|----:----|----:----|----:----|----:----|----:----|----:----|----:----|----:----|----:----|----:----|----:----|----:----|----:----|
5001 GTTATTAAAATGTAAGTCATAGTTTTAGTGTACCTGAACCGTGGTAAACGAATGATGATTTTTGTTTTATAACATTTAAGTATGGTGGTGTATAGTAGTTTGTTAGTAGTCTTTCACATGTTTGACTTACTGGTCGACTGGATTTTATAGCCAGTTTGACCTGTAAAAGTGTTTCTTTACACAGAAATAAATTTTAACTG 5200
      L L K V N L I L I V H V Q C W K S V V L F L I N Y I * V V V Y * * V I M L F H V F Q I V L Q G L I D T L S S M K V F F H T K I * F Q   F6
      * Y N * M * Y * F * M S K A G N A * * * F C F I T F E Y W W M D D F L * * F T Y L S F S W S V * F I P * V P C K * L S I H R * K F N V   F5
     E I I K C E T D F D C P S P V M Q K S S F V F Y Q L N M G G C I M L C D D S L T C V S H G A S R F Y R D F Q V N E C L F T D K N L I S   F4

      G T S A L K I I F V * T S N N K N V W P N H S H * M V * N T N S R G I G K C * N F C M K M L I R I H L Q N K Y I * * W Y S L K S T F   F1
     T E H L P * R L S S S K P P T T K M Y G P I T P T E W Y E I P T A G A L A N V R I S V * K C L F E S I F K T S T F D N G I L * R A L S   F2
      R N I C L E D Y L R L N L Q Q Q K C M A Q S L P L N G M K Y Q Q Q G H W Q M L E F L Y E N A Y S N P S S K Q V H L I M V F F E E H F Q   F3
5201 ACGGAACATCTGCCTTGAAGATTATCTTCGTCTAAACCTCCAACAACAAAAATGTATGGCCCAATCACTCCCACTGAATGGTATGAAATACCAACAGCAGGGGCATTGGCAAATGTTAGAATTTCTGTATGAAAATGCTTATTCGAATCCATCTTCAAAACAAGTACATTTGATAATGGTATTCTTTGAAGAGCACTTTC 5400
     ----:----|----:----|----:----|----:----|----:----|----:----|----:----|----:----|----:----|----:----|----:----|----:----|----:----|----:----|----:----|----:----|----:----|----:----|----:----|----:----|
5201 TGCCTTGTAGACGGAACTTCTAATAGAAGCAGATTTGGAGGTTGTTGTTTTTACATACCGGGTTAGTGAGGGTGACTTACCATACTTTATGGTTGTCGTCCCCGTAACCGTTTACAATCTTAAAGACATACTTTTACGAATAAGCTTAGGTAGAAGTTTTGTTCATGTAAACTATTACCATAAGAAACTTCTCGTGAAAG 5400
     C P V D A K F I I K T * V E L L L F T H G L * E W Q I T H F V L L L P M P L H * F K Q I F I S I R I W R * F L Y M Q Y H Y E K F L V K   F6
      R F M Q R S S * R R R F R W C C F H I A W D S G S F P I F Y W C C P C Q C I N S N R Y S F A * E F G D E F C T C K I I T N K S S C K    F5
      V S C R G Q L N D E D L G G V V F I Y P G I V G V S H Y S I G V A P A N A F T L I E T H F H K N S D M K L V L V N S L P I R Q L A S E   F4

     S F C D T T N I Y N R L H T S D F H Y I G G V I C S R C R S C H G E F G P K K T F K I K H V E I I I I G Y I F K K R * T N T * I S M W   F1
      V F V T P P I Y T I D F I P L I S I T L A V * Y A L G A D P V T E S L V Q K R L S K S N M * K L L S L V T F L K R D E P T H E F L C G   F2
       F L * H H Q Y I Q * T S Y L * F P L H W R C D M L * V Q I L S R R V W S K K D F Q N Q T C R N Y Y H W L H F * K E M N Q H M N F Y V   F3
5401 AGTTTTTGTGACACCACCAATATATACAATAGACTTCATACCTCTGATTTCCATTACATTGGCGGTGTGATATGCTCTAGGTGCAGATCCTGTCACGGAGAGTTTGGTCCAAAAAAGACTTTCAAAATCAAACATGTAGAAATTATTATCATTGGTTACATTTTTAAAAAGAGATGAACCAACACATGAATTTCTATGTG 5600
     ----:----|----:----|----:----|----:----|----:----|----:----|----:----|----:----|----:----|----:----|----:----|----:----|----:----|----:----|----:----|----:----|----:----|----:----|----:----|----:----|
5401 TCAAAAACACTGTGGTGGTTATATATGTTATCTGAAGTATGGAGACTAAAGGTAATGTAACCGCCACACTATACGAGATCCACGTCTAGGACAGTGCCTCTCAAACCAGGTTTTTTCTGAAAGTTTTAGTTTGTACATCTTTAATAATAGTAACCAATGTAAAAATTTTTCTCTACTTGGTTGTGTACTTAAAGATACAC 5600
      L K Q S V V L I Y L L S * V E S K W * M P P T I H E L H L D Q * P S N P G F F V K L I L C T S I I I M P * M K L F L H V L V H I E I H   F6
     * N K H C W W Y I C Y V E Y R Q N G N C Q R H S I S * T C I R D R L T Q D L F S K * F * V H L F * * * Q N C K * F S I F W C M F K * T   F5
       T K T V G G I Y V I S K M G R I E M V N A T H Y A R P A S G T V S L K T W F L S E F D F M Y F N N D N T V N K F L S S G V C S N R H    F4

      K T N S T M * N N T * I K I M S K C V P T P T R G N F T P * F S N R N T T W W L Y N L Y N T F V V I * F D N V Q F I * H R K T * Q I Q   F1
       K Q T P P C K I T H E S K * * V S V C P H L P E G I S P P N S L T E I P P G G C I I S T T P L L S F N L T M Y N S F D I E K H D K F   F2
     E N K L H H V K * H M N Q N N E * V C A H T Y Q R E F H P L I L * Q K Y H L V A V * S L Q H L C C H L I * Q C T I H L T * K N M T N S   F3
5601 GAAAACAAACTCCACCATGTAAAATAACACATGAATCAAAATAATGAGTAAGTGTGTGCCCACACCTACCAGAGGGAATTTCACCCCCTAATTCTCTAACAGAAATACCACCTGGTGGCTGTATAATCTCTACAACACCTTTGTTGTCATTTAATTTGACAATGTACAATTCATTTGACATAGAAAAACATGACAAATTC 5800
     ----:----|----:----|----:----|----:----|----:----|----:----|----:----|----:----|----:----|----:----|----:----|----:----|----:----|----:----|----:----|----:----|----:----|----:----|----:----|----:----|
5601 CTTTGTTTGAGGTGGTACATTTTATTGTGTACTTAGTTTTATTACTCATTCACACACGGGTGTGGATGGTCTCCCTTAAAGTGGGGGATTAAGAGATTGTCTTTATGGTGGACCACCGACATATTAGAGATGTTGTGGAAACAACAGTAAATTAAACTGTTACATGTTAAGTAAACTGTATCTTTTTGTACTGTTTAAG 5800
       F V F E V M Y F L V H I L I I L L H T G V G V L P F K V G * N E L L F V V Q H S Y L R * L V K T T M * N S L T C N M Q C L F V H C I   F6
      S F L S W W T F Y C M F * F L S Y T H A W V * W L S N * G R I R * C F Y W R T A T Y D R C C R Q Q * K I Q C H V I * K V Y F F M V F E   F5
      P F C V G G H L I V C S D F Y H T L T H G C R G S P I E G G L E R V S I G G P P Q I I E V V G K N D N L K V I Y L E N S M S F C S L N   F4

       T T P H K Q T I F S * N I K T S T * L * L W K L T T * K F D F R Y K I N I D I I V I Y N K R N F I N I S G H V P G A L L S S S G T T   F1
     R P P H T N K Q F S A E T * K P A L D C N C G N * P P E S L I S D I K S T L T S L S S T I K E I L * I F L D M F Q V L S C P L L A P P   F2
      D H P T Q T N N F Q L K H K N Q H L T V I V E I D H L K V * F Q I * N Q H * H H C H L Q * K K F Y K Y F W T C S R C S P V L F W H H Q   F3
5801 AGACCACCCCACACAAACAAACAATTTTCAGCTGAAACATAAAAACCAGCACTTGACTGTAATTGTGGAAATTGACCACCTGAAAGTTTGATTTCAGATATAAAATCAACATTGACATCATTGTCATCTACAATAAAAGAAATTTTATAAATATTTCTGGACATGTTCCAGGTGCTCTCCTGTCCTCTTCTGGCACCACC 6000
     ----:----|----:----|----:----|----:----|----:----|----:----|----:----|----:----|----:----|----:----|----:----|----:----|----:----|----:----|----:----|----:----|----:----|----:----|----:----|----:----|
5801 TCTGGTGGGGTGTGTTTGTTTGTTAAAAGTCGACTTTGTATTTTTGGTCGTGAACTGACATTAACACCTTTAACTGGTGGACTTTCAAACTAAAGTCTATATTTTAGTTGTAACTGTAGTAACAGTAGATGTTATTTTCTTTAAAATATTTATAAAGACCTGTACAAGGTCCACGAGAGGACAGGAGAAGACCGTGGTGG 6000
     * V V G C L C V I K L Q F M F V L V Q S Y N H F N V V Q F N S K L Y L I L M S M M T M * L L L F K I F I E P C T G P A R R D E E P V V   F6
      S W G V C V F L K * S F C L F W C K V T I T S I S W R F T Q N * I Y F * C Q C * Q * R C Y F F N * L Y K Q V H E L H E G T R K Q C W    F5
      L G G W V F L C N E A S V Y F G A S S Q L Q P F Q G G S L K I E S I F D V N V D N D D V I F S I K Y I N R S M N W T S E Q G R R A G G   F4

     K I Q I C I R I N G * R N M S F W W K K I K V I R C C T F S C N S P Q G I R * M G F K R * S T E L F V K T H H G I G T Q L I M * K A K   F1
      K Y K Y V * E L M G K E T C P S G G K K L K S S D A A L F L V I L L K G F V E W D L K G K A R N C L * K L I M E L E H S * * C K R P R   F2
      N T N M Y K N * W V K K H V L L V E K N * S H Q M L H F F L * F S S R D S L N G I * K V K H G I V C K N S S W N W N T V N N V K G Q    F3
6001 AAAATACAAATATGTATAAGAATTAATGGGTAAAGAAACATGTCCTTCTGGTGGAAAAAAATTAAAGTCATCAGATGCTGCACTTTTTCTTGTAATTCTCCTCAAGGGATTCGTTGAATGGGATTTAAAAGGTAAAGCACGGAATTGTTTGTAAAAACTCATCATGGAATTGGAACACAGTTAATAATGTAAAAGGCCAA 6200
     ----:----|----:----|----:----|----:----|----:----|----:----|----:----|----:----|----:----|----:----|----:----|----:----|----:----|----:----|----:----|----:----|----:----|----:----|----:----|----:----|
6001 TTTTATGTTTATACATATTCTTAATTACCCATTTCTTTGTACAGGAAGACCACCTTTTTTTAATTTCAGTAGTCTACGACGTGAAAAAGAACATTAAGAGGAGTTCCCTAAGCAACTTACCCTAAATTTTCCATTTCGTGCCTTAACAAACATTTTTGAGTAGTACCTTAACCTTGTGTCAATTATTACATTTTCCGGTT 6200
      L I C I H I L I L P Y L F M D K Q H F F I L T M L H Q V K E Q L E G * P I R Q I P N L L Y L V S N N T F V * * P I P V C N I I Y F A L   F6
     W F V F I Y L F * H T F F C T R R T S F F * L * * I S C K K K Y N E E L S E N F P I * F T F C P I T Q L F E D H F Q F V T L L T F P W   F5
      F Y L Y T Y S N I P L S V H G E P P F F N F D D S A A S K R T I R R L P N T S H S K F P L A R F Q K Y F S M M S N S C L * Y H L L G    F4

      D K Q K L S I K E Y S K Q T C K Q E Q Q Q I * L P R S P S P E Y I H A * I L * * E N I * Y I I * P C A I V * S Y E V H C A L * K F Y *   F1
       I N K S C P * R N I V N K R V N R N N N K F S Y L G A H H Q S I Y M H K Y C N E R I Y N I * Y N L V Q L C E A M K C T V H Y K N F T   F2
     G * T K A V H K G I * * T N V * T G T T T N L A T * E P I T R V Y T C I N T V M R E Y I I Y N I T L C N C V K L * S A L C I I K I L L   F3
6201 GGATAAACAAAAGCTGTCCATAAAGGAATATAGTAAACAAACGTGTAAACAGGAACAACAACAAATTTAGCTACCTAGGAGCCCATCACCAGAGTATATACATGCATAAATACTGTAATGAGAGAATATATAATATATAATATAACCTTGTGCAATTGTGTGAAGCTATGAAGTGCACTGTGCATTATAAAAATTTTACT 6400
     ----:----|----:----|----:----|----:----|----:----|----:----|----:----|----:----|----:----|----:----|----:----|----:----|----:----|----:----|----:----|----:----|----:----|----:----|----:----|----:----|
6201 CCTATTTGTTTTCGACAGGTATTTCCTTATATCATTTGTTTGCACATTTGTCCTTGTTGTTGTTTAAATCGATGGATCCTCGGGTAGTGGTCTCATATATGTACGTATTTATGACATTACTCTCTTATATATTATATATTATATTGGAACACGTTAACACACTTCGATACTTCACGTGACACGTAATATTTTTAAAATGA 6400
      S L C F S D M F S Y L L C V H L C S C C C I * S G L L G D G S Y I C A Y I S Y H S F I Y Y I I Y G Q A I T H L * S T C Q A N Y F N *   F6
      P Y V F A T W L P I Y Y V F T Y V P V V V F K A V * S G M V L T Y V H M F V T I L S Y I I Y L I V K H L Q T F S H L A S H M I F I K S   F5
     L I F L L Q G Y L F I T F L R T F L F L L L N L * R P A W * W L I Y M C L Y Q L S L I Y L I Y Y L R T C N H S A I F H V T C * L F K V   F4

      E S A L H C L F Q E N G Y S C T H L H V H * L V * R Y T G Y T I Y D I F * K K K R E S K N V A R H L H F E F Q D V L L Y S T I D I I   F1
     E N Q H C T V C F K K M D T H V L T Y M Y I D L F N D I Q D I Q Y T I F S E R K K G N L R T * L D I C I L N F K M F Y C I R P L I * S   F2
      R I S T A L F V S R K W I L M Y S P T C T L T C L T I Y R I Y N I R Y F L K E K K G I * E R S * T F A F * I S R C F I V F D H * Y N P   F3
6401 GAGAATCAGCACTGCACTGTTTGTTTCAAGAAAATGGATACTCATGTACTCACCTACATGTACATTGACTTGTTTAACGATATACAGGATATACAATATACGATATTTTCTGAAAGAAAAAAAGGGAATCTAAGAACGTAGCTAGACATTTGCATTTTGAATTTCAAGATGTTTTATTGTATTCGACCATTGATATAATC 6600
     ----:----|----:----|----:----|----:----|----:----|----:----|----:----|----:----|----:----|----:----|----:----|----:----|----:----|----:----|----:----|----:----|----:----|----:----|----:----|----:----|
6401 CTCTTAGTCGTGACGTGACAAACAAAGTTCTTTTACCTATGAGTACATGAGTGGATGTACATGTAACTGAACAAATTGCTATATGTCCTATATGTTATATGCTATAAAAGACTTTCTTTTTTTCCCTTAGATTCTTGCATCGATCTGTAAACGTAAAACTTAAAGTTCTACAAAATAACATAAGCTGGTAACTATATTAG 6600
     Q S D A S C Q K N * S F P Y E H V * R C T C Q S T * R Y V P Y V I Y S I K Q F F F L S D L F T A L C K C K S N * S T K N Y E V M S I I   F6
      L I L V A S N T E L F H I S M Y E G V H V N V Q K V I Y L I Y L I R Y K R F S F F P I * S R L * V N A N Q I E L H K I T N S W Q Y L   F5
     S F * C Q V T Q K L F I S V * T S V * M Y M S K N L S I C S I C Y V I N E S L F F P F R L V Y S S M Q M K F K L I N * Q I R G N I Y D   F4

     Q F Q S C Y Y L L * H N L L * N Y Y I S N T F I N N N K K K Q L T G * R G * T V R K V S K T G S V G I I V L A T G T M * K L F A L R P   F1
      N F S H A I T Y Y N T I S F K I T I S A I L L * I I I K K N S L P V N A V K Q S E K S L K P E A * E L S F L Q P E L C K S Y L P L D H   F2
      I S V M L L L I I T Q S P L K L L Y Q Q Y F Y K * * * K K T A Y R L T R L N S P K S L * N R K R R N Y R S C N R N Y V K V I C P * T   F3
6601 CAATTTCAGTCATGCTATTACTTATTATAACACAATCTCCTTTAAAATTACTATATCAGCAATACTTTTATAAATAATAATAAAAAAAAAACAGCTTACCGGTTAACGCGGTTAAACAGTCCGAAAAGTCTCTAAAACCGGAAGCGTAGGAATTATCGTTCTTGCAACCGGAACTATGTAAAAGTTATTTGCCCTTAGACC 6800
     ----:----|----:----|----:----|----:----|----:----|----:----|----:----|----:----|----:----|----:----|----:----|----:----|----:----|----:----|----:----|----:----|----:----|----:----|----:----|----:----|
6601 GTTAAAGTCAGTACGATAATGAATAATATTGTGTTAGAGGAAATTTTAATGATATAGTCGTTATGAAAATATTTATTATTATTTTTTTTTTGTCGAATGGCCAATTGCGCCAATTTGTCAGGCTTTTCAGAGATTTTGGCCTTCGCATCCTTAATAGCAAGAACGTTGGCCTTGATACATTTTCAATAAACGGGAATCTGG 6800
      W N * D H * * K N Y C L R R * F * * I L L V K I F L L L F F C S V P * R P * V T R F T E L V P L T P I I T R A V P V I Y F N N A R L G   F6
     G I E T M S N S I I V C D G K F N S Y * C Y K * L Y Y Y F F V A * R N V R N F L G F L R * F R F R L F * R E Q L R F * T F T I Q G * V   F5
      L K L * A I V * * L V I E K L I V I D A I S K Y I I I F F F L K G T L A T L C D S F D R F G S A Y S N D N K C G S S H L L * K G K S    F4

      R V I V L S R R R L I * R K F D S G A A X                                                                                        F1
       E L S F L A G D G L Y N G S L I V G R P X                                                                                       F2
     T S Y R S * Q E T A Y I T E V * * W G G Q                                                                                         F3
6801 ACGAGTTATCGTTCTTAGCAGGAGACGGCTTATATAACGGAAGTTTGATAGTGGGGCGGCCAG 6863
     ----:----|----:----|----:----|----:----|----:----|----:----|---
6801 TGCTCAATAGCAAGAATCGTCCTCTGCCGAATATATTGCCTTCAAACTATCACCCCGCCGGTC 6863
       R T I T R L L L R S I Y R F N S L P A A L                                                                                       F6
      V L * R E * C S V A * I V S T Q Y H P P W                                                                                        F5
     W S N D N K A P S P K Y L P L K I T P R G                                                                                         F4
```

Crassostrea virginica (Cvi)

WGS: MWPT03000002.1 – copy 1

48292070-48301202
Crassostrea virginica isolate RU13XGHG1-28 chromosome 5, whole genome shotgun sequence

```
     A * G T L S N F R Y I S R L L L R T I T R A L R A N N F Y I I P V A R T I I P T L P V L E T F R T V * P V S C F F Y Y Y L * K Y C * Y F1
     H R E H Y Q T S V I * A V S C * E R * L V L * G Q I T F T * F R L Q E R * F L R F R F * R L F G L F N R * A V F F I I I Y K S I A D I F2
     I G N T I K L P L Y K P S P A K N D N S C S K G K * L L H N S G C K N D N S Y A S G F R D F S D C L T G K L F F L L L F I K V L L I F3
   1 GCATAGGGAACACTATCAAACTTCCGTTATATAAGCCGTCTCCTGCTAAGAACGATAACTCGTGCTCTAAGGGCAAATAACTTTTACATAATTCCGGTTGCAAGAACGATAATTCCTACGCTTCCGGTTTTAGAGACTTTTCGGACTGTTTAACCGGTAAGCTGTTTTTTTTATTATTATTTATAAAAGTATTGCTGATA 200
     ----:----|----:----|----:----|----:----|----:----|----:----|----:----|----:----|----:----|----:----|----:----|----:----|----:----|----:----|----:----|----:----|----:----|----:----|----:----|----:----|
   1 CGTATCCCTTGTGATAGTTTGAAGGCAATATATTCGGCAGAGGACGATTCTTGCTATTGAGCACGAGATTCCCGTTTATTGAAAATGTATTAAGGCCAACGTTCTTGCTATTAAGGATGCGAAGGCCAAAATCTCTGAAAAGCCTGACAAATTGGCCATTCGACAAAAAAAATAATAATAAATATTTTCATAACGACTAT 200
     A Y P V S D F K R * I L R R R S L V I V R A R L A F L K * M I G T A L V I I G V S G T K S V K R V T * G T L Q K K * * * K Y F Y Q Q Y F6
     X M P F V I L S G N Y L G D G A L F S L E H E L P L Y S K C L E P Q L F S L E * A E P K L S K E S Q K V P L S N K K N N N I F T N S I F5
     C L S C * * V E T I Y A T E Q * S R Y S T S * P C I V K V Y N R N C S R Y N R R K R N * L S K P S N L R Y A T K K I I I * L L I A S F4

     S N F K G D C V I I S N S M T E I G L Y Q W S N T I K H L E I Q N A N V A V E G M * M S C L A T F L D S L F F L S E N I V Y R I L Y I F1
     V I L K E I V L * * V I A * L K L D Y I N G R I Q * N I L K F K M Q M * L * R A C K C H V * L R S * I P F F F F F Q K I S Y I V Y C I F2
     * * F * R R L C Y N K * * H D * N W I I S M V E Y N K T S * N S K C K C S C R G H V N V M S S Y V L R F P F F S F R K Y R I S Y I V Y F3
 201 TAGTAATTTTAAAGGAGATTGTGTTATAATAAGTAATAGCATGACTGAAATTGGATTATATCAATGGTCGAATACAATAAAACATCTTGAAATTCAAAATGCAAATGTAGCTGTAGAGGGCATGTAAATGTCATGTCTAGCTACGTTCTTAGATTCCCTTTTTTTTCTTTCAGAAAATATCGTATATCGTATATTGTATA 400
     ----:----|----:----|----:----|----:----|----:----|----:----|----:----|----:----|----:----|----:----|----:----|----:----|----:----|----:----|----:----|----:----|----:----|----:----|----:----|----:----|
 201 ATCATTAAAATTTCCTCTAACACAATATTATTCATTATCGTACTGACTTTAACCTAATATAGTTACCAGCTTATGTTATTTTGTAGAACTTTAAGTTTTACGTTTACATCGACATCTCCCGTACATTTACAGTACAGATCGATGCAAGAATCTAAGGGAAAAAAAAGAAAGTCTTTTATAGCATATAGCATATAACATAT 400
     L L K L P S Q T I I L L L M V S I P N Y * H D F V I F C R S I * F A F T A T S P M Y I D H R A V N K S E R K K R E S F I T Y R I N Y F6
     Y Y N * L L N H * L L Y Y C S Q F Q I I D I T S Y L L V D Q F E F H L H L Q L P C T F T M D L * T R L N G K K E K L F Y R I D Y I T Y F5
     I T I K F S I T N Y Y T I A H S F N S * I L P R I C Y F M K F N L I C I Y S Y L A H L H * T * S R E * I G K K K K * F I D Y I T Y Q I F4

     P Y I A I Q V N V H V G E Y P F S * N K Q C S A D S Q * N F Y N A Q C T S H N C T R L Y Y I F S Y Y S I N A C I Y S C D G L L G S * F1
     S R I S L Y K S M Y M * V S I H F L E T N S A V L I L S K I F I M H S A L H T I A Q G Y I I Y S L I T V L M H V Y T L V M G S * V A R F2
     P V Y R Y T S Q C T C R * V S I F L K Q T V Q C * F S V K F L * C T V H F T Q L H K V I L Y I L L L Q Y * C M Y I L L * W A P R * L D F3
 401 TCCCGTATATCGCTATACAAGTCAATGTACATGTAGGTGAGTATCCATTTTCTTGAAACAAACAGTGCAGTGCTGATTCTCAGTAAAATTTTTATAATGCACAGTGCACTTCACACAATTGCACAAGGTTATATTATATATTCTCTTATTACAGTATTAATGCATGTATATACTCTTGTGATGGGCTCCTAGGTAGCTAG 600
     ----:----|----:----|----:----|----:----|----:----|----:----|----:----|----:----|----:----|----:----|----:----|----:----|----:----|----:----|----:----|----:----|----:----|----:----|----:----|----:----|
 401 AGGGCATATAGCGATATGTTCAGTTACATGTACATCCACTCATAGGTAAAAGAACTTTGTTTGTCACGTCACGACTAAGAGTCATTTTAAAAATATTACGTGTCACGTGAAGTGTGTTAACGTGTTCCAATATAATATATAAGAGAATAATGTCATAATTACGTACATATATGAGAACACTACCCGAGGATCCATCGATC 600
     I G Y I A I C T L T C T P S Y G N E Q F L C H L A S E * Y F K * L A C H V E C L Q V L N Y * I N E * * L I L A H I Y E Q S P S R P L * F6
     G T Y R * V L * H V H L H T D M K K F C V T C H Q N E T F N K Y H V T C K V C N C L T I N Y I R K N C Y * H M Y I S K H H A G L Y S F5
     D R I D S Y L D I Y M Y T L I W K R S V F L A T S I R L L I K I I C L A S * V I A C P * I I Y E R I V T N I C T Y V R T I P E * T A L F4

     I C C C S S L H V C L L Y S F L D S F C L S L A F Y I I N C V P I P * * V F T N N S V L Y L L N P I R R I P * G E L Q E K V Q H L M T F1
     F V V V P V Y T F V Y Y I P F W T A F V Y P W P F T L L T V F Q F H D E F L Q T I P C F T F * I P F D E S L E E N Y K K K C S I * * L F2
     L L L F Q F T R L F T I F L F G Q L L F I L G L L H Y * L C S N S M M S F Y K Q F R A L P F K S H S T N P L R R I T R K S A A S D D F3
 601 ATTTGTTGTTGTTCCAGTTTACACGTTTGTTTACTATATTCCTTTTTGGACAGCTTTTGTTTATCCTTGGCCTTTTACATTATTAACTGTGTTCCAATTCCATGATGAGTTTTTACAAACAATTCCGTGCTTTACCTTTTAAATCCCATTCGACGAATCCCTTGAGGAGAATTACAAGAAAAAGTGCAGCATCTGATGAC 800
     ----:----|----:----|----:----|----:----|----:----|----:----|----:----|----:----|----:----|----:----|----:----|----:----|----:----|----:----|----:----|----:----|----:----|----:----|----:----|----:----|
 601 TAAACAACAACAAGGTCAAATGTGCAAACAAATGATATAAGGAAAAACCTGTCGAAAACAAATAGGAACCGGAAAATGTAATAATTGACACAAGGTTAAGGTACTACTCAAAAATGTTTGTTAAGGCACGAAATGGAAAATTTAGGGTAAGCTGCTTAGGGAACTCCTCTTAATGTTCTTTTTCACGTCGTAGACTACTG 800
     I Q Q Q E L K C T Q K S Y E K K S L K Q K D K A K * M I L Q T G I G H H T K V F L E T S * R K F G M R R I G Q P S N C S F T C C R I V F6
     S K N N N W N V R K N V I N R K P C S K N I R P R K C * * S H E L E M I L K * L C N R A K G K L D W E V F G K L L I V L F L A A D S S F5
     N T T T G T * V N T * * I G K Q V A K T * G Q G K V N N V T N W N W S S N K C V I G H K V K * I G N S S D R S S F * L F F H L M Q H F4

     L I F F H Q K D M F L Y Q L I V I H I C I L V V P E E D R R A P G T C P E I F I K F L L L * M T M M S M L I L Y L K S N F Q V V N F H F1
     * F F S T R R T C F F T N * L L Y I F V F W W C Q K R T G E H L E H V Q K Y L * N F F Y C R * Q * C Q C * F Y I * N Q T F R W S I S F2
     F N F F P P E G H V S L P I N C Y T Y L Y F G G A R R G Q E S T W N M S R N I Y K I S F I V D D N D V N V D F I S E I K L S G G Q F P F3
 801 TTTAATTTTTTTCCACCAGAAGGACATGTTTCTTTACCAATTAATTGTTATACATATTTGTATTTTGGTGGTGCCAGAAGAGGACAGGAGAGCACCTGGAACATGTCCAGAAATATTTATAAAATTTCTTTTATTGTAGATGACAATGATGTCAATGTTGATTTTATATCTGAAATCAAACTTTCAGGTGGTCAATTTCC 1000
     ----:----|----:----|----:----|----:----|----:----|----:----|----:----|----:----|----:----|----:----|----:----|----:----|----:----|----:----|----:----|----:----|----:----|----:----|----:----|----:----|
 801 AAATTAAAAAAAGGTGGTCTTCCTGTACAAAGAAATGGTTAATTAACAATATGTATAAACATAAAACCACCACGGTCTTCTCCTGTCCTCTCGTGGACCTTGTACAGGTCTTTATAAATATTTTAAAGAAAATAACATCTACTGTTACTACAGTTACAACTAAAATATAGACTTTAGTTTGAAAGTCCACCAGTTAAAGG 1000
     K I K K W W F S M N R * W N I T I C I Q I K T T G S S S L L A G P V H G S I N I F N R K N Y I V I I D I N I K Y R F D F K * T T L K F6
     K L K K G G S P C T E K G I L Q * V Y K Y K P P A L L P C S L V Q F M D L F I * L I E K I T S S L S T L T S K I D S I L S E P P * N G F5
     S * N K E V L L V H K K V L * N N Y M N T N Q H H W F L V P S C R S C T W F Y K Y F K K * Q L H C H H * H Q N * I Q F * V K L H D I E F4

     N Y S Q V L V F M F Q L K I V C L C G V V * I C H V F L C Q M N C T L S N * M T T K V L * R L Y S H Q V V F L L E N * G V K F P L V F1
     T I T V K C W F L C F S * K L F V C V G W S E F V M F F Y V K * I V H C Q I K * Q Q R C C R D Y T A T R W Y F C * R I R G * N S L W * F2
     Q L Q S S A G F Y V S A E N C L F V W G G L N L S C F S M S N E L Y I V K L N D N K G V V E I I Q P P G G I S V R E L G G E I P S G R F3
1001 ACAATTACAGTCAAGTGCTGGTTTTTATGTTTCAGCTGAAAATTGTTTGTTTGTGTGGGGTGGTCTGAATTTGTCATGTTTTTCTATGTCAAATGAATTGTACATTGTCAAATTAAATGACAACAAAGGTGTTGTAGAGATTATACAGCCACCAGGTGGTATTTCTGTTAGAGAATTAGGGGGTGAAATTCCCTCTGGTA 1200
     ----:----|----:----|----:----|----:----|----:----|----:----|----:----|----:----|----:----|----:----|----:----|----:----|----:----|----:----|----:----|----:----|----:----|----:----|----:----|----:----|
1001 TGTTAATGTCAGTTCACGACCAAAAATACAAAGTCGACTTTTAACAAACAAACACACCCCACCAGACTTAAACAGTACAAAAAGATACAGTTTACTTAACATGTAACAGTTTAATTTACTGTTGTTTCCACAACATCTCTAATATGTCGGTGGTCCACCATAAAGACAATCTCTTAATCCCCCACTTTAAGGGAGACCAT 1200
     W L * L * T S T K I N * S F I T Q K H P T T Q I Q * T K R H * I F Q V N D F * I V V F T N Y L N Y L W W T T N R N S F * P T F N G R T F6
     C N C D L A P K * T E A S F Q K N T H P P R F K D H K E I D F S N Y M T L N F S L L P T T S I I C G G P P I E T L S N P P S I G E P F5
     V I V T L H Q N K H K L Q F N N T Q T P H D S N T M N K * T L H I T C Q * I L H C C L H Q L S * V A V L H Y K Q * L I L P H F E R Q Y F4

     G V G T H L L I I L I H V L F Y M V E F V F H I E I H V L V H L F L K M * P M I I V S T C L I L K V F F G P N S L * Q D L H L E H I T F1
     V W A H T Y S L F * F M C Y F T W W S L F S T * K F M C W F I S F * K C N Q * * * F L H V * F * K S F L D Q T L C D R I C T * S I S H F2
     C G H T L T H Y F D S C V I L H G G V C F P H R N S C V G S S L F K N V T N D N S F Y M F D F E S L F W T K L S V T G S A P R A Y H F3
1201 GGTGTGGGCACACACTTACTCATTATTTTGATTCATGTGTTATTTTACATGGTGGAGTTTGTTTTCCACATAGAAATTCATGTGTTGGTTCATCTCTTTTTAAAAATGTAACCAATGATAATAGTTTCTACATGTTTGATTTTGAAAGTCTTTTTTGGACCAAACTCTCTGTGACAGGATCTGCACCTAGAGCATATCAC 1400
     ----:----|----:----|----:----|----:----|----:----|----:----|----:----|----:----|----:----|----:----|----:----|----:----|----:----|----:----|----:----|----:----|----:----|----:----|----:----|----:----|
1201 CCACACCCGTGTGTGAATGAGTAATAAAACTAAGTACACAATAAAATGTACCACCTCAAACAAAAGGTGTATCTTTAAGTACACAACCAAGTAGAGAAAAATTTTTACATTGGTTACTATTATCAAAGATGTACAAACTAAAACTTTCAGAAAAAACCTGGTTTGAGAGACACTGTCCTAGACGTGGATCTCGTATAGTG 1400
     P T P V C K S M I K I * T N N * M T S N T K W M S I * T N T * R K K F I Y G I I I T E V H K I K F T K K P G F E R H C S R C R S C I V F6
     L H P C V S V * * K S E H T I K C P P T Q K G C L F E H T P E D R K L F T V L S L L K * M N S K S L R K Q V L S E T V P D A G L A Y * F5
     T H A C V * E N N Q N M H * K V H H L K N E V Y F N M H Q N M E K * F H L W H Y Y N R C T Q N Q F D K K S W V R Q S L I Q V * L M D F4

     P P M * W K S E V * S L L Y I L V V S Q K L K V L F K E Y H Y Q M Y L F * R W I R I S I F I Q K S * H L P L P L L L V L G V H R Y I E F1
     R Q C S G N Q R Y E V Y C I Y W W C H K N * K C S S K N T I I K C T C F E D G F E * A F S Y R N P N I C H C P C C W Y * G C T D I S F2
     T A N V V E I R G M K S I V Y I G G V T K T E S A L Q R I P L S N V L V L K M D S N K H F H T E I L T F A T A P A V G I R G A P I Y R F3
1401 ACCGCCAATGTAGTGGAAATCAGAGGTATGAAGTCTATTGTATATATTGGTGGTGTCACAAAAACTGAAAGTGCTCTTCAAAGAATACCATTATCAAATGTACTTGTTTTGAAGATGGATTCGAATAAGCATTTTCATACAGAAATCCTAACATTTGCCACTGCCCCTGCTGTTGGTATTAGGGGTGCACCGATATATCG 1600
     ----:----|----:----|----:----|----:----|----:----|----:----|----:----|----:----|----:----|----:----|----:----|----:----|----:----|----:----|----:----|----:----|----:----|----:----|----:----|----:----|
1401 TGGCGGTTACATCACCTTTAGTCTCCATACTTCAGATAACATATATAACCACCACAGTGTTTTTGACTTTCACGAGAAGTTTCTTATGGTAATAGTTTACATGAACAAAACTTCTACCTAAGCTTATTCGTAAAAGTATGTCTTTAGGATTGTAAACGGTGACGGGGACGACAACCATAATCCCCACGTGGCTATATAGC 1600
     G G I Y H F D S T H L R N Y I N T T D C F S F T S K L S Y W * * I Y K N Q L H I R I L M K M C F D * C K G S G R S N T N P T C R Y I F6
     V A L T T S I L P I F D I T Y I P P T V F V S L A R * L I G N D F T S T K F I S E F L C K * V S I R V N A V A G A T P I L P A G I Y R F5
     C R W H L P F * L Y S T * Q I Y Q H H * L F Q F H E E F F V M I L H V Q K S S P N S Y A N E Y L F G L M Q W Q G Q Q Q Y * P H V S I D F4

     I S I H I D F H L H D E Y R * S T F D T P T N V F R L R V R D Y W T Y F R S G S T S L K L I T R M A S K K S K C W N Y F C N E K N R F1
     R S R Y I S I F T C M T S I D N Q P S I H R P M Y L G Y E F G I T G R I L D R D R P R * N * * H E W R Q K N R S A G I T F A M K R T D F2
     D L D T Y R F S P A * R V S I I N L R Y T D Q C I * V T S S G L L D V F * I G I D L V K I N N T N G V K K I E V L E L L L Q * K E P T F3
1601 AGATCTCGATACATATCGATTTTCACCTGCATGACGAGTATCGATAATCAACCTTCGATACACCGACCAATGTATTTAGGTTACGAGTTCGGGATTACTGGACGTATTTTAGATCGGGATCGACCTCGTTAAAATTAATAACACGAATGGCGTCAAAAAAATCGAAGTGCTGGAATTACTTTGCAATGAAAAGAACCGA 1800
     ----:----|----:----|----:----|----:----|----:----|----:----|----:----|----:----|----:----|----:----|----:----|----:----|----:----|----:----|----:----|----:----|----:----|----:----|----:----|----:----|
1601 TCTAGAGCTATGTATAGCTAAAAGTGGACGTACTGCTCATAGCTATTAGTTGGAAGCTATGTGGCTGGTTACATAAATCCAATGCTCAAGCCCTAATGACCTGCATAAAATCTAGCCCTAGCTGGAGCAATTTTAATTATTGTGCTTACCGCAGTTTTTTTAGCTTCACGACCTTAATGAAAACGTTACTTTTCTTGGCT 1800
     S I E I C I S K * R C S S Y R Y D V K S V G V L T N L N R T R S * Q V Y K L D P D V E N F N I V R I A D F F D F H Q F * K Q L S F F R F6
     S R S V Y R N E G A H R T D I I L R R Y V S W H I * T V L E P N S S T N * I P I S R T L I L L V F P T L F I S T S S N S K C H F S G F5
     L D R Y M D I K V Q M V L I S L * G E I C R G I Y K P * S N P I V P R I K S R S R G R * F * Y C S H R * F F R L A P I V K A I F L V S F4

     Q A K C N L C S A V V A T S G G T T N L I F H L N R H H N I S L R S E T T V T T R A S G D V G M C V I H E C L * L L F V * L N H L * R F1
     R Q N A I C V Q R W W R R Q G A L Q I * Y F I * T D I I T F R Y A A R Q L S Q L V P L E M S V C A L Y M S V Y D Y Y L F D * I I Y S E F2
     G K M Q S V F S G G G D V R G H Y K S D I S F K Q T S * H F V T Q R D N C H N S C L W R C R Y V R Y T * V F M I T I C L I K S F I A F3
1801 CAGGCAAAATGCAATCTGTGTTCAGCGGTGGTGGCGACGTCAGGGGGCACTACAAATCTGATATTTCATTTAAACAGACATCATAACATTTCGTTACGCAGCGAGACAACTGTCACAACTCGTGCCTCTGGAGATGTCGGTATGTGCGTTATACATGAGTGTTTATGATTACTATTTGTTTGATTAAATCATTTATAGCG 2000
     ----:----|----:----|----:----|----:----|----:----|----:----|----:----|----:----|----:----|----:----|----:----|----:----|----:----|----:----|----:----|----:----|----:----|----:----|----:----|----:----|
1801 GTCCGTTTTACGTTAGACACAAGTCGCCACCACCGCTGCAGTCCCCCGTGATGTTTAGACTATAAAGTAAATTTGTCTGTAGTATTGTAAAGCAATGCGTCGCTCTGTTGACAGTGTTGAGCACGGAGACCTCTACAGCCATACACGCAATATGTACTCACAAATACTAATGATAAACAAACTAATTTAGTAAATATCGC 2000
     C A F H L R H E A T T A V D P P V V F R I N * K F L C * L M E N R L S V V T V V R A E P S T P I H T I C S H K H N S N T Q N F * K Y R F6
     V P L I C D T N L P P P S T L P C * L D S I E N L C V D Y C K T V C R S L Q * L E H R Q L H R Y T R * V H T N I I V I Q K I L D N I A F5
     L C F A I Q T * R H H R R * P A S C I Q Y K M * V S M M V N R * A A L C S D C S T G R S I D T H A N Y M L T * S * * K N S * I M * L F4

     K Y * K T Y M A N * F L K H N F S P T T H * T K M * A W G E N Q P M D A F S F S A Q I A I V Q N K * N H N S R I A I Y Y S * S P A L L F1
     N I R K L T W L I N S * N T I L A Q Q P T E P K C E P G V K T N P W T L S A S Q R K L P L S R T N E I T T A V L Q Y I I H D L R P F F2
     K I L E N L H G * L I P E T Q F * P N N P L N Q N V S L G * K P T H G R F Q L L S A N C H C P E Q M K S Q Q P Y C N I L F M I S G P S F3
2001 AAAATATTAGAAAACTTACATGGCTAATTAATTCCTGAAACACAATTTTAGCCCAACAACCCACTGAACCAAAATGTGAGCCTGGGGTGAAAACCAACCCATGGACGCTTTCAGCTTCTCAGCGCAAATTGCCATTGTCCAGAACAAATGAAATCACAACAGCCGTATTGCAATATATTATTCATGATCTCCGGCCCTTC 2200
     ----:----|----:----|----:----|----:----|----:----|----:----|----:----|----:----|----:----|----:----|----:----|----:----|----:----|----:----|----:----|----:----|----:----|----:----|----:----|----:----|
2001 TTTTATAATCTTTTGAATGTACCGATTAATTAAGGACTTTGTGTTAAAATCGGGTTGTTGGGTGACTTGGTTTTACACTCGGACCCCACTTTTGGTTGGGTACCTGCGAAAGTCGAAGAGTCGCGTTTAACGGTAACAGGTCTTGTTTACTTTAGTGTTGTCGGCATAACGTTATATAATAAGTACTAGAGGCCGGGAAG 2200
     F Y * F V * M A L * N R F C L K L G V V W Q V L I H A Q P S F W G M S A K L K E A C I A M T W F L H F * L L R I A I Y * E H D G A R F6
     F I N S F K C P * N I G S V C N * G L L G S F W F T L R P H F G V W P R K * S R L A F Q W Q G S C I F D C C G Y Q L I N N M I E P G E F5
     S F I L F S V H S I L E Q F V I K A W C G V S G F H S G P T F V L G H V S E A E * R L N G N D L V F S I V V A T N C Y I I * S R R G K F4

     Y Y * E S C V * V R L T I K I K I C K P N A I I * C N E N C F P F * * * M N V I L S I K E D A F N T * A S L S A S K S N Q T K * R V F1
     S T I E S P V F R * D * Q * K * K F A N Q M Q L Y N A M K I V S H S N S K * M L Y Y L L R K M L S T L E P R Y Q P P N R T R L S E E L F2
     L L L R V L C L G K I D N K N K N L Q T K C N Y I M Q * K L F P I L I V N E C Y I I Y * G R C F Q H L S L V I S L Q I E P D * V K S * F3
2201 TCTACTATTGAGAGTCCTGTGTTTAGGTAAGATTGACAATAAAAATAAAAATTTGCAAACCAAATGCAATTATATAATGCAATGAAAATTGTTTCCCATTCTAATAGTAAATGAATGTTATATTATCTATTAAGGAAGATGCTTTCAACACTTGAGCCTCGTTATCAGCCTCCAAATCGAACCAGACTAAGTGAAGAGTT 2400
     ----:----|----:----|----:----|----:----|----:----|----:----|----:----|----:----|----:----|----:----|----:----|----:----|----:----|----:----|----:----|----:----|----:----|----:----|----:----|----:----|
2201 AGATGATAACTCTCAGGACACAAATCCATTCTAACTGTTATTTTTATTTTTAAACGTTTGGTTTACGTTAATATATTACGTTACTTTTAACAAAGGGTAAGATTATCATTTACTTACAATATAATAGATAATTCCTTCTACGAAAGTTGTGAACTCGGAGCAATAGTCGGAGGTTTAGCTTGGTCTGATTCACTTCTCAA 2400
     R * * Q S D Q T * T L N V I F I F I Q L G F A I I Y H L S F Q K G N * Y Y I F T I N D I L S S A K L V Q A E N D A E L D F W V L H L T F6
     R S N L T R H K P L I S L L F L F K C V L H L * I I C H F N N G M R I T F S H * I I * * P L H K * C K L R T I L R W I S G S * T F L F5
     E V I S L G T N L Y S Q C Y F Y F N A F W I C N Y L A I F I T E W E L L L H I N Y * R N L F I S E V S S G R * * G G F R V L S L S S N F4
```

```
     N S C P V * K H K K Q V I C * T T K Q * S S I P Y M R R L D I Q G N P V L S N S Y M S F C * * K L G I K F K N S T N R S F * R F T H R    F1
      I P A L Y E S I K S K L Y A E L Q S S E A V S L T C D G W T S R A T Q S F L T V T C H F V N K N W E L S S K I L Q T D H F E G S H T G   F2
       F L P C M K A * K A S Y M L N Y K A V K Q Y P L H A T A G H P G Q P S P F * Q L H V I L L I K T G N * V Q K F Y K Q I I L K V H T Q    F3
2401 AATTCCTGCCCTGTATGAAAGCATAAAAAGCAAGTTATATGCTGAACTACAAAGCAGTGAAGCAGTATCCCTTACATGCGACGGCTGGACATCCAGGGCAACCCAGTCCTTTCTAACAGTTACATGTCATTTTGTTAATAAAAACTGGGAATTAAGTTCAAAAATTCTACAAACAGATCATTTTGAAGGTTCACACACAG 2600
     ----:----|----:----|----:----|----:----|----:----|----:----|----:----|----:----|----:----|----:----|----:----|----:----|----:----|----:----|----:----|----:----|----:----|----:----|----:----|----:----|
2401 TTAAGGACGGGACATACTTTCGTATTTTTCGTTCAATATACGACTTGATGTTTCGTCACTTCGTCATAGGGAATGTACGCTGCCGACCTGTAGGTCCCGTTGGGTCAGGAAAGATTGTCAATGTACAGTAAAACAATTATTTTTGACCCTTAATTCAAGTTTTTAAGATGTTTGTCTAGTAAAACTTCCAAGTGTGTGTC 2600
      L E Q G T H F C L F C T I H Q V V F C H L L I G * M R R S S M W P L G T R E L L * M D N Q * Y F S P I L N L F E V F L D N Q L N V C L   F6
     * N R G Q I F A Y F A L * I S F * L A T F C Y G K C A V A P C G P C G L G K * C N C T M K N I F V P F * T * F N * L C I M K F T * V C    F5
      I G A R Y S L M F L L N Y A S S C L L S A T D R V H S P Q V D L A V W D K R V T V H * K T L L F Q S N L E F I R C V S * K S P E C V    F4

      S E N R I * T Q K R D D R L G Y F * Q S W N D Y S G * C Q * Y D S C S * S I W C * S Q T W L F C S H P * P G I K Q G S L Y T L A * Q N  F1
       Q K I G F E H K S V M T D W G I S D K V G M I T V D N A S N M T V A V E V S G V D L K L G C F A H T L N L A S N K A L Y I P S L D K  F2
     V R K * D L N T K A * * Q T G V F L T K L E * L Q W I M P V I * Q L Q L K Y L V L I S N L V V L L T P L T W H Q T R L F I Y P R L T K  F3
2601 GTCAGAAAATAGGATTTGAACACAAAAGCGTGATGACAGACTGGGGTATTTCTGACAAAGTTGGAATGATTACAGTGGATAATGCCAGTAATATGACAGTTGCAGTTGAAGTATCTGGTGTTGATCTCAAACTTGGTTGTTTTGCTCACACCCTTAACCTGGCATCAAACAAGGCTCTTTATATACCCTCGCTTGACAAA 2800
     ----:----|----:----|----:----|----:----|----:----|----:----|----:----|----:----|----:----|----:----|----:----|----:----|----:----|----:----|----:----|----:----|----:----|----:----|----:----|----:----|
2601 CAGTCTTTTATCCTAAACTTGTGTTTTCGCACTACTGTCTGACCCCATAAAGACTGTTTCAACCTTACTAATGTCACCTATTACGGTCATTATACTGTCAACGTCAACTTCATAGACCACAACTAGAGTTTGAACCAACAAAACGAGTGTGGGAATTGGACCGTAGTTTGTTCCGAGAAATATATGGGAGCGAACTGTTT 2800
       D S F L I Q V C F R S S L S P Y K Q C L Q F S * L P Y H W Y Y S L Q L Q L I Q H Q D * V Q N N Q E C G * G P M L C P E K Y V R A Q C  F6
      T L F Y S K F V F A H H C V P T N R V F N S H N C H I I G T I H C N C N F Y R T N I E F K T T K S V G K V Q C * V L S K I Y G R K V F  F5
     P * F I P N S C L L T I V S Q P I E S L T P I I V T S L A L L I V T A T S T D P T S R L S P Q K A * V R L R A D F L A R * I G E S S L  F4

      S W * S T F S C Y L L P Q K Q Y L K K S R L P F R C Q N T S * * L I A K Q G R T Q L I K C C K D F * S K D Q Q F * L L F W M T G * N  F1
     I L G K V R S V V T Y F H K S N T * R K A G C P S D A K T Q V D N * L Q N K V E L N L S N V A K I F R A K T S N S S Y S S G * Q G K T  F2
      F L V K Y V Q L L P T S T K A I L K E K Q V A L Q M P K H K L I I D C K T R S N S T Y Q M L Q R F L E Q R P A I L A T L L D D R V K R  F3
2801 ATTCTTGGTAAAGTACGTTCAGTTGTTACCTACTTCCACAAAAGCAATACTTAAAGAAAAGCAGGTTGCCCTTCAGATGCCAAAACACAAGTTGATAATTGATTGCAAAACAAGGTCGAACTCAACTTATCAAATGTTGCAAAGATTTTTAGAGCAAAGACCAGCAATTCTAGCTACTCTTCTGGATGACAGGGTAAAAC 3000
     ----:----|----:----|----:----|----:----|----:----|----:----|----:----|----:----|----:----|----:----|----:----|----:----|----:----|----:----|----:----|----:----|----:----|----:----|----:----|----:----|
2801 TAAGAACCATTTCATGCAAGTCAACAATGGATGAAGGTGTTTTCGTTATGAATTTCTTTTCGTCCAACGGGAAGTCTACGGTTTTGTGTTCAACTATTAACTAACGTTTTGTTCCAGCTTGAGTTGAATAGTTTACAACGTTTCTAAAAATCTCGTTTCTGGTCGTTAAGATCGATGAGAAGACCTACTGTCCCATTTTG 3000
      F E Q Y L V N L Q * R S G C F C Y K F F L L N G K L H W F V L Q Y N I A F C P R V * S I L H Q L S K * L L S W C N * S S K Q I V P Y F  F6
       N K T F Y T * N N G V E V F A I S L S F C T A R * I G F C L N I I S Q L V L D F E V * * I N C L N K S C L G A I R A V R R S S L T F  F5
      I R P L T R E T T V * K W L L L V * L F A P Q G E S A L V C T S L Q N C F L T S S L K D F T A F I K L A F V L L E L * E E P H C P L V F4

     E Q I K P R * L V A * V T L T F K F V R I L C P T W M * C L Q P L L S Y V K R S L L R L D L C F H F S T S * Q N I S N Q R L L T L H L  F1
      S R * N Q G S * W L E * L * H S N L * G F C V Q H G C N A Y S H S C L M * R E V S Y G W T C A S T S L Q A N R T F Q T K G Y * L Y I C F2
       A D K T K V V S G L S D S D I Q I C E D F V S N M D V M L T A T L V L C E E K S P T A G L V L P L L Y K L T E H F K P K A T D S T F  F3
3001 GAGCAGATAAAACCAAGGTAGTTAGTGGCTTGAGTGACTCTGACATTCAAATTTGTGAGGATTTTGTGTCCAACATGGATGTAATGCTTACAGCCACTCTTGTCTTATGTGAAGAGAAGTCTCCTACGGCTGGACTTGTGCTTCCACTTCTCTACAAGCTAACAGAACATTTCAAACCAAAGGCTACTGACTCTACATTT 3200
     ----:----|----:----|----:----|----:----|----:----|----:----|----:----|----:----|----:----|----:----|----:----|----:----|----:----|----:----|----:----|----:----|----:----|----:----|----:----|----:----|
3001 CTCGTCTATTTTGGTTCCATCAATCACCGAACTCACTGAGACTGTAAGTTTAAACACTCCTAAAACACAGGTTGTACCTACATTACGAATGTCGGTGAGAACAGAATACTTCTCTTCAGAGGATGCCGACCTGAACACGAAGGTGAAGAGATGTTCGATTGTCTTGTAAAGTTTGGTTTCCGATGACTGAGATGTAAA 3200
      S C I F G L Y N T A Q T V R V N L N T L K H G V H I Y H K C G S K D * T F L L R R S S K H K W K E V L * C F M E F W L S S V R C K  F6
     R A S L V L T T L P K L S E S M * I Q S S K T D L M S T I S V A V R T K H S S F D G V A P S T S G S R * L S V S C K L G F A V S E V N  F5
      L L Y F W P L * H S S H S Q C E F K H P N Q T W C P H L A * L W E Q R I H L S T E * P Q V Q A E V E R C A L L V N * V L P * Q S * M  F4

      L N P * K M L * V L T * L Q G T Q I Q T * D I F W K R L V H * I Q E Q R I R P V L M R K H G T G L R T K * L F L M S Q V * E * Q L N L  F1
       * I H E K C C R F * P N Y K V H R S K L E I F S G R G * C I R S K N K E * G L C * * G N M G Q D * G Q N D C S * C H R C R N N S * I  F2
     V K S M K N A V G S D L I T R Y T D P N L R Y F L E E A S A L D P R T K N K A C V D E E T W D R I E D K M T V L D V T G V G I T V K S  F3
3201 GTTAAATCCATGAAAAATGCTGTAGGTTCTGACCTAATTACAAGGTACACAGATCCAAACTTGAGATATTTTCTGGAAGAGGCTAGTGCATTAGATCCAAGAACAAAGAATAAGGCCTGTGTTGATGAGGAAACATGGGACAGGATTGAGGACAAAATGACTGTTCTTGATGTCACAGGTGTAGGAATAACAGTTAAATC 3400
     ----:----|----:----|----:----|----:----|----:----|----:----|----:----|----:----|----:----|----:----|----:----|----:----|----:----|----:----|----:----|----:----|----:----|----:----|----:----|----:----|
3201 CAATTTAGGTACTTTTTACGACATCCAAGACTGGATTAATGTTCCATGTGTCTAGGTTTGAACTCTATAAAAGACCTTCTCCGATCACGTAATCTAGGTTCTTGTTTCTTATTCCGGACACAACTACTCCTTTGTACCCTGTCCTAACTCCTGTTTTACTGACAAGAACTACAGTGTCCACATCCTTATTGTCAATTTAG 3400
       N F G H F I S Y T R V * N C P V C I W V Q S I K Q F L S T C * I W S C L I L G T N I L F C P V P N L V F H S N K I D C T Y S Y C N F  F6
      T L D M F F A T P E S R I V L Y V S G F K L Y K R S S A L A N S G L V F F L A Q T S S S V H S L I S S L I V T R S T V P T P I V T L D  F5
     Q * I W S F H Q L N Q G L * L T C L D L S S I N E P L P * H M L D L F L S Y P R H Q H P F M P C S Q P C F S Q E Q H * L H L F L L * I  F4

      K K L K V V K F L T H L L L * * S P H R F L L C L C W M R T * M Q M K R * Y S K G L L N I Y L L L R N T I I E F S S E E T A I S D  F1
     * K N * K W * N S S H I F C C S R V H T A S C F A C V G * G H K C R * K G D T L R A C * I F I Y Y Y * E T Q L * S F Q V K K Q Q * V M  F2
      E K T E S G E I P H T S S A V V E S T P L P A L P V L V D E D I N A D E K V I L * G L A K Y L F T T T K K H N Y R V F K * R N S N K * *  F3
3401 TGAAAAAACTGAAAGTGGTGAAATTCCTCACACATCTTCTGCTGTAGTAGAGTCCACACCGCTTCCTGCTTTGCCTGTGTTGGATGAGGACATAAATGCAGATGAAAAGGTGATACTCTAAGGGCTTGCTAAATATTTATTTACTACTACTAAGAAACACAATTATAGAGTTTTCAAGTGAAGAAACAGCAATAAGTGAT 3600
     ----:----|----:----|----:----|----:----|----:----|----:----|----:----|----:----|----:----|----:----|----:----|----:----|----:----|----:----|----:----|----:----|----:----|----:----|----:----|----:----|
3401 ACTTTTTTGACTTTCACCACTTTAAGGAGTGTGTAGAAGACGACATCATCTCAGGTGTGGCGAAGGACGAAACGGACACAACCTACTCCTGTATTTACGTCTACTTTTCCACTATGAGATTCCCGAACGATTTATAAATAAATGATGATGATTCTTTGTGTTAATATCTCAAAAGTTCACTTCTTTGTCGTTATTCACTA 3600
     R F F S F T T F N R V C R R S Y Y L G C R K R S Q R H Q I L V Y I C I F L H Y E L P K S F I * K S S S L F V I I S N E L S S V A I L S  F6
      S F V S L P S I G * V D E A T S D V G S G A K G T N S S S M F A S S F T I S * P S A L Y K N V V V L F C L * L T K L H L F L L L H  F5
     Q F F Q F H H F E E C M K Q Q L L T W V A E Q K A Q T P H P C L H L H F P S V R L A Q * I N I * * * * S V C N Y L K * T F F C C Y T I  F4

     E Y * S D E * I F Y L M H F H D T I F L N Q * F S C Q I S K S P P A K K N S P * D F V * * G N C Y N W T G T C T L C Q G K C Q T G A A  F1
      N I K V M N E Y F I * C I S M T L Y F * I S S F L V R F P N L H L Q K K T A L E T L F D E E I V I T G Q E P A L S A R E N A K R E L Q  F2
       I L K * * M N I L F N A F P * H Y I S K S V V F L S D F Q I S T C K K K Q P L R L C L M R K L L * L D R N L H S L P G K M P N G S C  F3
3601 GAATATTAAAGTGATGAATGAATATTTTATTTAATGCATTTCCATGACACTATATTTCTAAATCAGTAGTTTTCTTGTCAGATTTCCAAATCTCCACCTGCAAAAAAAAACAGCCCTTGAGACTTTGTTTGATGAGGAAATTGTTATAACTGGACAGGAACCTGCACTCTCTGCCAGGGAAAATGCCAAACGGGAGCTGC 3800
     ----:----|----:----|----:----|----:----|----:----|----:----|----:----|----:----|----:----|----:----|----:----|----:----|----:----|----:----|----:----|----:----|----:----|----:----|----:----|----:----|
3601 CTTATAATTTCACTACTTACTTATAAAATAAATTACGTAAAGGTACTGTGATATAAAGATTTAGTCATCAAAAGAACAGTCTAAAGGTTTAGAGGTGGACGTTTTTTTTTGTCGGGAACTCTGAAACAAACTACTCCTTTAACAATATTGACCTGTCCTTGGACGTGAGAGACGGTCCCTTTTACGGTTTGCCCTCGACG 3800
      S Y * L S S H I N * K I C K W S V I N R F * Y N E Q * I E L D G G A F F F L G Q S K T Q H P F Q * L Q V P V Q V R Q W P F H W V P A A  F6
     H I N F H H I F I K N L A N G H C * I E L D T T K K D S K W I E V Q L F F C G K L S Q K I L F N N Y S S L F R C E R G P F I G F P L Q  F5
      F I L T I F S Y K I * H M E M V S Y K * I L L K R T L N G F R W R C F F V A R S V K N S S S I T I V P C S G A S E A L S F A L R S S  F4

      A L Q R K E T S N N E R K P T A L V E D Q L S H V S T L I Q T S S Q I L L C A S Y I S S I R K S F Q H N W * H S I C N T C M P E N R K  F1
       L Y K E K K Q A T M K E N P L L W W K T N Y L M F P R L S K L A V R Y C C V Q A T S V A S E R V F S T T G D I V S A T R A C L K T E  F2
      S S T K K R N K Q Q * K K T H C F G G R P I I S C F H A Y P N * Q S D I A V C K L H Q * H Q K E F S A Q L V T * Y L Q H V H A * K Q K  F3
3801 AGCTCTACAAAGAAAAGAAACAAGCAACAATGAAAGAAAACCCACTGCTTTGGTGGAAGACCAATTATCTCATGTTTCCACGCTTATCCAAACTAGCAGTCAGATATTGCTGTGTGCAAGCTACATCAGTAGCATCAGAAAGAGTTTTCAGCACAACTGGTGACATAGTATCTGCAACACGTGCATGCCTGAAAACAGAA 4000
     ----:----|----:----|----:----|----:----|----:----|----:----|----:----|----:----|----:----|----:----|----:----|----:----|----:----|----:----|----:----|----:----|----:----|----:----|----:----|----:----|
3801 TCGAGATGTTTCTTTTCTTTGTTCGTTGTTACTTTCTTTTGGGTGACGAAACCACCTTCTGGTTAATAGAGTACAAAGGTGCGAATAGGTTTGATCGTCAGTCTATAACGACACACGTTCGATGTAGTCATCGTAGTCTTTCTCAAAAGTCGTGTTGACCACTGTATCATAGACGTTGTGCACGTACGGACTTTTGTCTT 4000
      A R C L F S V L L L S L F G V A K T S S W N D * T E V S I W V L L * I N S H A L * M L L M L F L K * C L Q H C L I Q L V H M G S F L  F6
      L E V F F L F L C C H F F V W Q K P F L G I E H K W A * G F * C D S I A T H L S C * Y C * F S N E A C S T V Y Y R C C T C A Q F C F  F5
     C S * L S F F C A V I F S F G S S Q H F V L * R M N G R K D L S A T L Y Q Q T C A V D T A D S L T K L V V P S M T D A V R A H R F V S  F4

      R * C S C V S K E K H A V L I S L I N Y N H F F T P S Q G E D K E K F V * Y C L L T I Y Y L F L F S F A L I K N I I Q Y I V Y R * L  F1
     N V N A L V F L K K N M Q F * F H * L T I I I F L P H L R E R I K K N L F D I V C L P F I I C F C L V L H * * K T * S N I S C I V N F  F2
      T L M L L C F * R K T C S F N F I D * L * S F F Y P I S G R G * R K I C L I L F A Y H L L S V F V * F C I N K K H N P I Y R V S L T L  F3
4001 AACGTTAATGCTCTTGTGTTTCTAAAGAAAAACATGCAGTTTTAATTTCATTGATTACTATAATCATTTTTTTACCCCATCTCAGGGAGAGGATAAAGAAAAATTTGTTTGATATTGTTTGCTTACCATTTATTATCTGTTTTTGTTTAGTTTTGCATTAATAAAAAACATAATCCAATATATCGTGTATCGTTACTT 4200
     ----:----|----:----|----:----|----:----|----:----|----:----|----:----|----:----|----:----|----:----|----:----|----:----|----:----|----:----|----:----|----:----|----:----|----:----|----:----|----:----|
4001 TTGCAATTACGAGAACACAAAGATTTCTTTTTGTACGTCAAAATTAAAGTAACTAATGATATTAGTAAAAAAATGGGGTAGAGTCCCTCTCCTATTTCTTTTTAAACAAACTATAACAAACGAATGGTAAATAATAGACAAAAACAAATCAAAACGTAATTATTTTTTGTATTAGGTTATATAGCACATAGCAATGAA 4200
      F R * H E Q T E L S F C A T K I E N I L * K K V G D * P S S L S F N T Q Y Q K S V M * * R N K N L K A N I F F M I W Y I T Y R * S  F6
      V N I S K H K * L F V H L K L K M S * S Y D N K * G M E P L P Y L F I Q K I N N A * W K N D T K T * N Q M L L F C L G I Y R T D N V  F5
      F T L A R T N R F F F M C N * N * Q N V I I M K K G W R L S L I F F F K N S I T Q K G N I I Q K Q K T K C * Y F V Y D L I D H I T L K  F4

     * H R I V S Y R I F * A N T Q L * L V F H T I Q * E * L G H T F L L L E V W T K I I F K A D V P C Q F * I K T H F F V K M S S L T D I  F1
      N I V S Y R I E F F K P I H S S S W Y F I P F S R S D W A I H F C C W R F G R R * S S R Q M F R V N F K * R H I S L * K C P V * P I F  F2
       T S Y R I V S N F L S Q Y T A L V G I S Y H S V G V I G P Y I F V V G G L D E D N L Q G R C S V S I L N K D T F L C E N V Q F D R Y  F3
4201 TAACATCGTATCGTATCGTATCGAATTTTTTAAGCCAATACACAGCTCTAGTTGGTATTTCATACCATTCAGTAGGAGTGATTGGGCCATACATTTTTGTTGTTGGAGGTTTGGACGAAGATAATCTTCAAGGCAGATGTTCCGTGTCAATTTTAAATAAAGACACATTTCTTTGTGAAAATGTCCAGTTTGACCGATAT 4400
     ----:----|----:----|----:----|----:----|----:----|----:----|----:----|----:----|----:----|----:----|----:----|----:----|----:----|----:----|----:----|----:----|----:----|----:----|----:----|----:----|
4201 ATTGTAGCATAGCATAGCATAGCTTAAAAAATTCGGTTATGTGTCGAGATCAACCATAAAGTATGGTAAGTCATCCTCACTAACCCGGTATGTAAAAACAACAACCTCCAAACCTGCTTCTATTAGAAGTTCCGTCTACAAGGCACAGTTAAAATTTATTTCTGTGTAAAGAAACACTTTTACAGGTCAAACTGGCTATA 4400
      * C R I T D Y R I K * A L V C S * N T N * V M * Y S H N P W V N K N N S T Q V F I I K L A S T G H * N * I F V C K K T F I D L K V S I  F6
      K V D Y R I T D F K K L W Y V A R T P I E Y W E T P T I P G Y M K T T P P K S S S L R * P L H E T D I K F L S V N R Q S F T W N S R Y  F5
      L M T D Y R I S N K L G I C L E L Q Y K M G N L L L S Q A M C K Q Q Q L N P R L Y D E L C I N R T L K L Y L C M E K H F H G T Q G I  F4

      L G Q L V I Q F V H F L M I V * * Y V V V * I Y N I L F L V V S K W C Q V H V I L I L N V K L L N L L K H P L Y L G F N V K V L V K D  F1
       * V S W S F S L Y T F * * L F D D M W W Y E F T I F C F * * * A N G A K S M * F * Y * M * N Y * I C * N I P F I L D S M * R C L * K  F2
      F R S A G H S V C T L S D D C L M I C G G M N L Q Y F V F S S K Q M V P S P C D F D T E C K I I E S V E T S P L S W I Q C E G A C K R  F3
4401 TTTAGGTCAGCTGGTCATTCAGTTTGTACACTTTCTGATGATTGTTTGATGATATGTGGTGGTATGAATTTACAATATTTTGTTTTTAGTAGTAAGCAAATGGTGCCAAGTCCATGTGATTTTGATACTGAATGTAAAATTATTGAATCTGTTGAAACATCCCCTTTATCTTGGATTCAATGTGAAGGTGCTTGTAAAAG 4600
     ----:----|----:----|----:----|----:----|----:----|----:----|----:----|----:----|----:----|----:----|----:----|----:----|----:----|----:----|----:----|----:----|----:----|----:----|----:----|----:----|
4401 AAATCCAGTCGACCAGTAAGTCAAACATGTGAAAGACTACTAACAAACTACTATACACCACCATACTTAAATGTTATAAAACAAAAATCATCATTCGTTTACCACGGTTCAGGTACACTAAAACTATGACTTACATTTTAATAACTTAGACAACTTTGTAGGGGAAATAGAACCTAAGTTACACTTCCACGAACATTTTC 4600
       K P * S T M * N T K R I T I Q H Y T T H I * L I K N K T T L L H H W T W T I K I S F T F N N F R N F C G R * R P N L T F H L H S T F  F6
      K L D A P * E T Q V S E S S Q K I I H P P I F K C Y K T K L L L C I T G L G H S K S V S H L I I S D T S V D G K D Q I * H S P A Q L L  F5
     N * T L Q D N L K Y V K Q H N N S S I H H Y S N V I N Q K * Y Y A F P A L D M H N Q Y Q I Y F * Q I Q Q F M G K I K S E I H L H K Y F  F4

       G Y I S F V L A F * I L T * V G K I L F V P H V P R P P E L R R E N F L Y R I N L Q S V C * Y F L L Y I S Y F L N K E I R L * * C F  F1
      M V T S V L C W R F R Y * H E S E K F Y L Y H M P Q D L Q S * E E K I S C T E L I F S L C V S T S C C I F H T F * T K K * D Y S S V S  F2
       W L H Q F C V G V L D I D M S R K N F I C T T C S K T S R V K K R K F L V Q N * S S V C V L V L L V V Y F I L F E Q R N K I I V V F Q  F3
4601 ATGGTTACATCAGTTTTGTGTTGGCGTTTTAGATATGACATGAGTCGGAAAAATTTTATTTGTACCACATGTTCCAAGACCTCCAGAGTTAAGAAGAGAAAATTTCTTGTACAGAATTAATCTTCAGTCTGTGTGTTAGTACTTCTTGTTGTATATTTCATACTTTTTGAACAAAGAAATAAGATTATAGTAGTGTTTC 4800
     ----:----|----:----|----:----|----:----|----:----|----:----|----:----|----:----|----:----|----:----|----:----|----:----|----:----|----:----|----:----|----:----|----:----|----:----|----:----|----:----|
4601 TACCAATGTAGTCAAAACACAACCGCAAAATCTATACTGTACTCAGCCTTTTTAAAATAAACATGGTGTACAAGGTTCTGGAGGTCTCAATTCTTCTCTTTTAAAGAACATGTCTTAATTAGAAGTCAGACACACAATCATGAAGAACAACATATAAAGTATGAAAAACTTGTTTCTTTATTCTAATATCATCACAAAG 4800
      S P * M L K T N A N * I N V H T P F I K N T G C T G L G G S N L L S F K K Y L I L R * D T H * Y K K N Y I E Y K K F L S I L N Y Y H K  F6
       H N C * N Q T P T K S I S M L R F F K I Q V V H E L V E L T L F L F N R T C F * D E T Q T N T S R T T Y K M S K S C L F L I I T T N  F5
      I T V D T K H Q R K L Y Q C S D S F N * K Y W M N W S R W L * S S F I E Q V S N I K L R H T L V E Q Q I N * V K Q V F F Y S * L L T E  F4

      N I C M F V F I Y L Y R H S E C I Q C D M H I E I I H T R R * R S K * C I I L I H L Y L Y H L D I H I Y M Y I Y I H I Y W * I F I T I  F1
       I S A C L Y L S I C I D I V N V Y N V T C I L K * Y I Q E D E G L S N V S Y * Y I Y I I * I Y T S I C I S I Y I Y T G K F L * L *  F2
      Y L H V C I Y L F V * T * * M Y T * H A Y * N N T Y K K M K V * V M Y H I N T S I S I S S R Y T H L Y V Y L Y T Y I V F Y N Y  F3
4801 AATATCTGCATGTTTGTATTTATCTATTTGTATAGACATAGTGAATGTATACAATGTGACATGCATATTGAAATAATACATACAAGAAGATGAAGGTCTAAGTAATGTATCATATTAATACATCTATATCTATATCATCTAGATATACACATCTATATGTATATCTATATACATATATACTGGTAAATTTTTATTTAACTAT 5000
     ----:----|----:----|----:----|----:----|----:----|----:----|----:----|----:----|----:----|----:----|----:----|----:----|----:----|----:----|----:----|----:----|----:----|----:----|----:----|----:----|
4801 TTATAGACGTACAAACATAAATAGATAAACATATCTGTATCACTTACATATGTTACACTGTACGTATAACTTTATTATGTATGTTCTTCTACTTCCAGATTCATTACATAGTATAATTATGTAGATATAGATATAGTAGATCTATATGTGTAGATATACATATAGATATATGTATATATGACCATTTAAAAATAAATTGATA 5000
      L I Q M N T N I * K Y L C L S H I C H S M C I S I I C V L L H L D L Y H I M N I C R Y R Y * R S I C M * I Y I * I C I Y Q Y I K I V I  F6
      * Y R C T Q I * R N T Y V Y H I Y V I H C A Y Q F L V Y L F I F T * T I Y * I L V D I D I D D L Y V C R Y T Y R Y V Y I S T F K * L *  F5
      I D A H K Y K D I Q I S M T F T Y L T V H M N F Y Y M C S S S P R L L T D Y * Y M * I * I M * I Y V D I H I D I Y M Y V P L N K Y S  F4
```

S F L Y A C K Y L * N * H I T L Y I C C L I T C N I V H V N * K S V T N S I M I I N T * * N * M Q C I I I I L N N * L R F T N * Y S E F1
A S C M L V N T S R T D I * H Y T Y V V * L H V I * Y M * I R K V * Q I L L * S S I L N E I K C N V L S * S * T T D * D L Q T S I Q F2
K L L V C L * I P L E L T Y N I I H M L F N Y M * Y S T C K L E K C N K F Y Y D H Q Y L M K L N A M Y Y H N L K Q L I K I Y K L V F R F3
5001 AAGCTTCTTGTATGCTTGTAAATACCTCTAGAACTGACATATAACATTATACATATGTTGTTTAATTACATGTAATATAGTACATGTAAATTAGAAAAGTGTAACAAATTCTATTATGATCATCAATACTTAATGAAATTAAATGCAATGTATTATCATAATCTTAAACAACTGATTAAGATTTACAAACTAGTATTCAG 5200
----:----|----:----|----:----|----:----|----:----|----:----|----:----|----:----|----:----|----:----|----:----|----:----|----:----|----:----|----:----|----:----|----:----|----:----|----:----|----:----|
5001 TTCGAAGAACATACGAACATTTATGGAGATCTTGACTGTATATTGTAATATGTATACAACAAATTAATGTACATTATATCATGTACATTTAATCTTTTCACATTGTTTAAGATAATACTAGTAGTTATGAATTACTTTAATTTACGTTACATAATAGTATTAGAATTTGTTGACTAATTCTAAATGTTTGATCATAAGTC 5200
L K K Y A Q L Y R * F Q C I V N Y M H Q K I V H L I T C T F * F L T V F E I I I M L V * H F * I C H I I M I K F L Q N L N V F * Y E F6
L S R T H K Y I G R S S V Y L M I C I N N L * M Y Y L V H L N S F H L L N * * S * * Y K I F N F A I Y * * L R L C S I L I * L S T N L F5
Y A E Q I S T F V E L V S M Y C * V Y T T * N C T I Y Y M Y I L F T Y C I R N H D D I S L S I L H L T N D Y D * V V S * S K C V L I * F4

V G K F S C C D Q K * N I K N N E L S Y A Q L Q Q A * K E P C Y Y T A S E V I K H H T N S H T C F H D E R D F F * N D E I V C C Q P F1
K W A S S L V V T R S K T * R T T N F L M L N F S K L K K S L A T I Q P V K L * N I T Q T P I R V S T T R E I F S K M T K * F V A S L F2
S G Q V L L L * P E V K H K E Q R T F L C S T S A S L K R A L L L Y S Q * S Y K T S H K L P Y V F P R R E R F F L K * R N S L L P A S F3
5201 AAGTGGGCAAGTTCTCTTGTTGTGACCAGAAGTAAAACATAAAGAACAACGAACTTTCTTATGCTCAACTTCAGCAAGCTTAAAAAGAGCCTTGCTACTATACAGCCAGTGAAGTTATAAAACATCACACAAACTCCCATACGTGTTTCCACGACGAGAGAGATTTTTTCTAAAATGACGAAATAGTTTGTTGCCAGCCT 5400
----:----|----:----|----:----|----:----|----:----|----:----|----:----|----:----|----:----|----:----|----:----|----:----|----:----|----:----|----:----|----:----|----:----|----:----|----:----|----:----|
5201 TTCACCCGTTCAAGAGAACAACACTGGTCTTCATTTTGTATTTCTTGTTGCTTGAAAGAATACGAGTTGAAGTCGTTCGAATTTTTCTCGGAACGATGATATGTCGGTCACTTCAATATTTTGTAGTGTGTTTGAGGGTATGCACAAAGGTGCTGCTCTCTCTAAAAAAGATTTTACTGCTTTATCAAACAACGGTCGGA 5400
S T P L N E Q Q S W F Y F M F F L S S E * A * S * C A * F S G Q * * V A L S T I F C * V F E W V H K W S S L S K K * F S S I T Q Q W G F6
L P C T R K N H G S T F C L S C R V K K H E V E A L K F L A K S S Y L W H L * L V D C L S G Y T N G R R S L N K R F H R F L K N G A F5
F H A L E R T T V L L L V Y L V V F K R I S L K L L S L F L R A V I C G T F N Y F M V C V G M R T E V V L S I K E L I V F Y N T A L R F4

H C L H Y * K L Q L T Q A H L D L Q * V A G N V Q * L C V N N W A T L H N Q N V E V K G P P F Q Q H I S C N F V H Q K D I L S E G L Y F1
I A F T T E S S N * P R P I W I F N K L L E M F N D F V * I I G P L Y I I K M L K * K G P H F N S T F L V I L Y I K R T F Y Q R V F T F2
L P S L L K A P T D P G P F G S S I S C W K C S M T L C K * L G H S T * S K C * S K R A P I S T A H F L * F C T S K G H S I R G S L F3
5401 CATTGCCTTCACTACTGAAAGCTCCAACTGACCCAGGCCCATTTGGATCTTCAATAAGTTGCTGGAAATGTTCAATGACTTTGTGTAAATAATTGGGCCACTCTACATAATCAAAATGTTGAAGTAAAAGGGCCCCCATTTCAACAGCACATTTCTTGTAATTTTGTACATCAAAAGGACATTCTATCAGAGGGTCTTTA 5600
----:----|----:----|----:----|----:----|----:----|----:----|----:----|----:----|----:----|----:----|----:----|----:----|----:----|----:----|----:----|----:----|----:----|----:----|----:----|----:----|
5401 GTAACGGAAGTGATGACTTTCGAGGTTGACTGGGTCCGGGTAAACCTAGAAGTTATTCAACGACCTTTACAAGTTACTGAAACACATTTATTAACCCGGTGAGATGTATTAGTTTTACAACTTCATTTTCCCGGGGGTAAAGTTGTCGTGTAAAGAACATTAAAACATGTAGTTTTCCTGTAAGATAGTCTCCCAGAAAT 5600
* Q R * * Q F S W S V W A W K S R * Y T A P F T * H S Q T F L Q A V R C L * F T S T F P G G N * C C M E Q L K T C * F S M R D S P R * F6
E N G E S S F A G V S G P G N P D E I L Q Q F H E I V K H L Y N P W E V Y D F H Q L L L A G M E V A C K K Y N Q V D F P C E I L P D K F5
M A K V V S L E L Q G L G M Q I K L L N S S I N L S K T Y I I P G S * M I L I N F Y F P G W K L L V N R T I K Y M L L V N * * L T K F4

I Y K L S E G V K L C S K * K T D S L S L Q N L K Y V L T K Q N G E F Q K G F W Y N Y Q A S * A E D L S H S Y I S Y V D Q T K I Q H F F1
S I N F P K V * N Y V Q N R R Q I L F P Y R I * N T F * Q N R M G S F K K G S G I I T R H H K L R I Y P T V T F H M L I K Q K F S I F2
H L * T F R R C K I M F K I E D R F S F L T E S E I R S N K T E W G V S K R V L V * L P G I I S * G F I P Q L H F I C * S N K N S A F F3
5601 CATCTATAAACTTTCCGAAGGTGTAAAATTATGTTCAAAATAGAAGACAGATTCTCTTTCCTTACAGAATCTGAAATACGTTCTAACAAAACAGAATGGGGAGTTTCAAAAAGGGTTCTGGTATAATTACCAGGCATCATAAGCTGAGGATTTATCCCACAGTTACATTTCATATGTTGATCAAACAAAAATTCAGCATT 5800
----:----|----:----|----:----|----:----|----:----|----:----|----:----|----:----|----:----|----:----|----:----|----:----|----:----|----:----|----:----|----:----|----:----|----:----|----:----|----:----|
5601 GTAGATATTTGAAAGGCTTCCACATTTTAATACAAGTTTTATCTTCTGTCTAAGAGAAAGGAATGTCTTAGACTTTATGCAAGATTGTTTTGTCTTACCCCTCAAAGTTTTTCCCAAGACCATATTAATGGTCCGTAGTATTCGACTCCTAAATAGGGTGTCAATGTAAAGTATACAACTAGTTTGTTTTTAAGTCGTAA 5800
M * L S E S P T F N H E F Y F V S E R E K C F R F Y T R V F C F P S N * F P N Q Y L * W A D Y A S S K D W L * M E Y T S * V F I * C F6
C R Y V K R L H L I I N L I S S L N E K R V S D S I R E L L V S H P T E F L T R T Y N G P M M L Q P N I G C N C K M H Q D F L F E A N F5
V D I F K G F T Y F * T * F L L C I R K G * L I Q F V N * C F L I P L K L F P E P I I V L C * L S L I * G V T V N * I N I L C F N L M F4

E L K V L H L V * V P I L L H L L F H V Q * F S L K T V L N L Y P H E W H L C L S E L V L F L K E E H I L H L L K F F * I H F * T K F1
L N * R F Y I L C K F P F C Y T C Y F T Y N N F L * K L S * I Y I R M S G I Y A F L N W F Y F * K R S T F Y T F * N F F K F I F R Q S F2
* T K G F T S C V S S H F V T P A I S R T I I F F K N C P K F I S A * V A S M P F * I G S I F E R G A H F T P F E I F L N S F L D K A F3
5801 TTGAACTAAAGGTTTTACATCTTGTGTAAGTTCCCATTTTGTTACACCTGCTATTTCACGTACAATAATTTTCTTTAAAAACTGTCCTAAATTTATATCCGCATGAGTGGCATCTATGCCTTTCTGAATTGGTTCTATTTTTGAAAGAGGAGCACATTTTACACCTTTTGAAATTTTTTTAAATTCATTTTTAGACAAAG 6000
----:----|----:----|----:----|----:----|----:----|----:----|----:----|----:----|----:----|----:----|----:----|----:----|----:----|----:----|----:----|----:----|----:----|----:----|----:----|----:----|
5801 AACTTGATTTCCAAAATGTAGAACACATTCAAGGGTAAAACAATGTGGACGATAAAGTGCATGTTATTAAAAGAAAATTTTTGACAGGATTTAAATATAGGCGTACTCACCGTAGATACGGAAAGACTTAACCAAGATAAAAACTTTCTCCTCGTGTAAAATGTGGAAAACTTTAAAAAAATTTAAGTAAAAATCTGTTTC 6000
K S S F T K C R T Y T G M K N C R S N * T C Y N E K F V T R F K Y G C S H C R H R E S N T R N K F S S C M K C R K F N K * I * K * V F F6
Q V L P K V D Q T L E W K T V G A I E R V I I K K L F Q G L N I D A H T A D I G K Q I P E I K S L P A C K V G K S I K K F E N K S L F5
K F * L N * M K H L N G N Q * V Q * K V Y L L K R * F S D * I * I R M L P M * A K R F Q N * K Q F L L V N * V K Q F K K L N M K L C L F4

H L G * H V K F Q Q * T D L L I E K D P S N A F A D C L S A S H K V H K Y L E D Q S P P C L F S H Q P * N * K M K I C G T S T P L L F F1
I W V N T * N F S N K L T C * L K K T Q V M L L Q I V Y L H H I K Y I N T L K T R V P P V S F L I N H R I K K * K F V E H P P L F C F F2
F G L T R K I S A I N * P V N * K R P K * C F C R L F I C I T * S T * I P * R P E S P L S L F S S S T I E L K N E N L W N I H P S F V F3
6001 CATTTGGGTTAACACGTAAAATTTCAGCAATAAACTGACCTGTTAATTGAAAAGACCCAAGTAATGCTTTTGCAGATTGTTTATCTGCATCACATAAAGTACATAAATACCTTGAAGACCAGAGTCCCCCCTGTCTCTTTTCTCATCAACCATAGAATTAAAAAATGAAAATTTGTGGAACATCCACCCCTCTTTTGTT 6200
----:----|----:----|----:----|----:----|----:----|----:----|----:----|----:----|----:----|----:----|----:----|----:----|----:----|----:----|----:----|----:----|----:----|----:----|----:----|----:----|
6001 GTAAACCCAATTGTGCATTTTAAAGTCGTTATTTGACTGGACAATTAACTTTTTCTGGGTTCATTACGAAAACGTCTAACAAATAGACGTAGTGTATTTCATGTATTTATGGAACTTCTGGTCTCAGGGGGGACAGAGAAAAGAGTAGTTGGTATCTTAATTTTTTACTTTTAAACACCTTGTAGGTGGGGAGAAAACAA 6200
C K P * C T F N * C Y V S R N I S F S G L L A K A S Q K D A D C L T C L Y R S S W L G G Q R K E * * G Y F * F I F I Q P V D V G R K N F6
A N P N V R L I E A I F Q G T L Q F L G L Y H K Q L N N I Q M V Y L V Y I G Q L G S D G R D R K E D V M S N F F S F K H F M W G G K R F5
M Q T L V Y F K L L L S V Q * N F F V W T I S K C I T * R C * M F Y M F V K F V L T G G T E K R M L W L I L F H F N T S C G G R K Q F4

S H A C D F A L K F P F H F Q * V K Y R Q H L K L D C F H Q K L I L K E V Y L L C W Q W F S K N I K K F S V I F S L N F H N T K G K T F1
H M H A I L H * N F P F I F N R * N T D N I * N L I V F I R N * F * K R S I C S V G N G S V K T S K S F P S Y S A * I F T I Q K E K F2
F T C M R F C I K I S L S F S I G K I Q T T F E T * L F S S E I D S K R G L F A L L A M V Q * K H Q K V F R H I Q P E F S Q Y K R K N F3
6201 TTCACATGCATGCGATTTGCATTAAAATTTCCCTTTCATTTTCAATAGGTAAAATACAGACAACATTTGAAACTTGATTGTTTTCATCAGAAATTGATTCTAAAAGAGGTCTATTTGCTCTGTTGGCAATGGTTCAGTAAAAACATCAAAAAGTTTTCCGTCATATTCAGCCTGAATTTTCACAATACAAAAGGAAAAA 6400
----:----|----:----|----:----|----:----|----:----|----:----|----:----|----:----|----:----|----:----|----:----|----:----|----:----|----:----|----:----|----:----|----:----|----:----|----:----|----:----|
6201 AAGTGTACGTACGCTAAAACGTAATTTTAAAGGGAAAGTAAAAGTTATCCATTTTATGTCTGTTGTAAACTTTGAACTAACAAAAGTAGTCTTTAACTAAGATTTTCTCCAGATAAACGAGACAACCGTTACCAAGTCATTTTTGTAGTTTTTCAAAAGGCAGTATAAGTCGGACTTAAAAGTGTTATGTTTTCCTTTTT 6400
E C A H S K A N F N G K * K * Y T F Y L C C K F S S Q K * F N I R F S T * K S Q Q C H N L L F M L F N E T M N L R F K * L V F P F F6
K V H M R N Q M L I E R E N E I P L I C V V N S V Q N N E D S I S E L L P R N A R N A I T * Y F C * F T K R * I * G S N E C Y L L F F F5
K * M C A I K C * F K G K M K L L Y F V S L M Q F K I T K M L F Q N * F L D I Q E T P L P E T F V D F L K G D Y E A Q I K V I C F S F F4

K S L V R Q H F E I F F F V N R D L P H S I T P I F N S S K K K N I F N I * P M F F Q S K * N K L N R L * F L Q G S R E C L K I G K S N F1
R K A L S G N I L K S F S L * T E T S P I P S H P S L T V V R K R T F S I F N P C F F N P N K I S G S S F C K V V E S A S K * E N L T F2
E K P C Q A T F * N L F L C K Q R P P P F H H T H L * Q * * E K E H F Q Y L T H V F S I Q I K S Q A L V F A R * * R V P Q N R K I * H F3
6401 CGAAAAGCCTTGTCAGGCAACATTTTGAAATCTTTTTCTTTGTAAACAGAGACCTCCCCCATTCCATCACACCCATCTTTAACAGTAGTAAGAAAAAGAACATTTTCAATATTTAACCCATGTTTTTTCAATCCAAATAAAATCTCAGGCTCTAGTTTTTGCAAGGTAGTAGAGAGTGCCTCAAAATAGGAAAATCTAAC 6600
----:----|----:----|----:----|----:----|----:----|----:----|----:----|----:----|----:----|----:----|----:----|----:----|----:----|----:----|----:----|----:----|----:----|----:----|----:----|----:----|
6401 GCTTTTCGGAACAGTCCGTTGTAAAACTTTAGAAAAAGAAACATTTGTCTCTGGAGGGGGTAAGGTAGTGTGGGTAGAAATTGTCATCATTCTTTTTCTTGTAAAAGTTATAAATTGGGTACAAAAAAGTTAGGTTTATTTTAGAGTCCGAGATCAAAAACGTTCCATCATCTCTCACGGAGTTTTATCCTTTTAGATTG 6600
V F L R T L C C K S I K K K T F L S R G W E M V G M K L L L F F F M K L I * G M N K * D L Y F R L S * N K C P L L S H R L I P F D L F6
S F G Q * A V N Q F R K R Q L C L G G G N W * V W R * C Y Y S F S C K * Y K V W T K E I W I F D * A R T K A L Y Y L T G * F L F I * F5
R F A K D P L M K F D K E K Y V S V E G M G D C G D K V T T L F L V N E I N L G H K K L G F L I E P E L K Q L T T S L A E F Y S F R V F4

T H T V R S C Q L N K T W Q K T F I N I Q R L C A W I F W R V C F A E T E * S G D L N T L D F V V T F V Q K F C A F H * T D T F M F I F1
P I Q F G V A N L T K P G K K H S L T F K G S V H G F S G A Y A L Q K Q N E V V I * T P * I L L * H L Y K N F V P F I E Q I L S C S S F2
P Y S S E L P T * Q N L A K N I H * H S K A L C M D F L A R M L C R N R M K W * F E H P R F C C N I C T K I L C L S L N R Y F H V H F3
6601 ACCCATACAGTTCGGAGTTGCCAACTTAACAAAACCTGGCAAAAAACATTCATTAACATTCAAAGGCTCTGTGCATGGATTTTCTGGCGCGTATGCTTTGCAGAAACAGAATGAAGTGGTGATTTGAACACCCTAGATTTTGTTGTAACATTTGTACAAAAATTTTGTGCCTTTCATTGAACAGATACTTTCATGTTCAT 6800
----:----|----:----|----:----|----:----|----:----|----:----|----:----|----:----|----:----|----:----|----:----|----:----|----:----|----:----|----:----|----:----|----:----|----:----|----:----|----:----|
6601 TGGGTATGTCAAGCCTCAACGGTTGAATTGTTTTGGACCGTTTTTTGTAAGTAATTGTAAGTTTCCGAGACACGTACCTAAAAGACCGCGCATACGAAACGTCTTTGTCTTACTTCACCACTAAACTTGTGGGATCTAAAACAACATTGTAAACATGTTTTTAAAACACGGAAAGTAACTTGTCTATGAAAGTACAAGTA 6800
V W V T R L Q W S L L V Q C F V N M L M * L S Q A H I K Q R T H K A S V S H L P S K F V R S K T T V N T C F N Q A K * Q V S V K M N M F6
C G Y L E S N G V * C F R A F F M * * C E F A R H M S K R A R I S Q L F L I F H H N S C G L N Q Q L M Q V F I K H R E N F L Y K * T * F5
G M C N P T A L K V F G P L F C E N V N L P E T C P N E P A Y A K C F C F S T T I Q V G * I K N Y C K Y L F K T G K M S C I S E H E F4

N I S N Q M R L C T V C M N T M H * Q S I I N L K K M T M E G G H L T L I K Q R T T Y R T C T S K N T F L A K N V A T F G N I W I T R F1
I F L I K C D C V Q F A * I Q C T N K V S * I * R R * Q W R G D T S L S S S R E R H T G H A H P K I H S L Q K M W L H L G I Y G S Q F2
Q Y F * S N A T V Y S L H E Y N A L T K Y H K F E E D D N G G G T P H S H Q A E N D I Q D M H I Q K Y I P C K K C G Y I W E Y M D H K F3
6801 CAATATTTCTAATCAAATGCGACTGTGTACAGTTTGCATGAATACAATGCACTAACAAAGTATCATAAATTTGAAGAAGATGACAATGGAGGGGGGACACCTCACTCTCATCAAGCAGAGAACGACATACAGGACATGCACATCCAAAAATACATTCCTTGCAAAAAATGTGGCTACATTTGGGAATATATGGATCACAA 7000
----:----|----:----|----:----|----:----|----:----|----:----|----:----|----:----|----:----|----:----|----:----|----:----|----:----|----:----|----:----|----:----|----:----|----:----|----:----|----:----|
6801 GTTATAAAGATTAGTTTACGCTGACACATGTCAAACGTACTTATGTTACGTGATTGTTTCATAGTATTTAAACTTCTTCTACTGTTACCTCCCCCCTGTGGAGTGAGAGTAGTTCGTCTCTTGCTGTATGTCCTGTACGTGTAGGTTTTTATGTAAGGAACGTTTTTTACACCGATGTAAACCCTTATATACCTAGTGTT 7000
L I E L * I R S H V T Q M F V I C * C L I M F K F F I V I S P P C R V R M L C L V V Y L V H V D L F V N R A F F T A V N P F I H I V F6
* Y K * D F A V T Y L K C S Y L A S V F Y * L N S S S S L P P P V G * E * * A S F S M C S M C M W F Y M G Q L F H P * M Q S Y I S * L F5
D I N R I L H S Q T C N A H I C H V L L T D Y I Q L L H C H L P S V E S E D L L S R C V P C A C G F I C E K C F I H S C K P I Y P D C F4

D T S T N S T H K R L * N F C I A E S L C R N T P N Y N I F E H F R C R L R R T * L N N * Q F I N R R * R G T I Y R G T K G A * C * F1
G T P R Q I V H I K D C E T S A L Q N R S V G I L L I T T F L N I S G A G S E E H D S I T D N L S T G G K G G L S T G G Q K E H D V E F2
G H L D K * Y T * K T V K L L H C R I A L * E Y S * L Q H F * T F Q V Q A Q K N M T Q * L T I Y Q Q E V K G D Y L Q G D K R S M M L K F3
7001 GGGACACCTCGACAAATAGTACACATAAAAGACTGTGAAACTTCTGCATTGCAGAATCGCTCTGTAGGAATACTCCTAATTACAACATTTTTGAACATTTCAGGTGCAGGCTCAGAAGAACATGACTCAATAACTGACAATTTATCAACAGGAGGTAAAGGGGGACTATCTACAGGGGGACAAAAGGAGCATGATGTTGA 7200
----:----|----:----|----:----|----:----|----:----|----:----|----:----|----:----|----:----|----:----|----:----|----:----|----:----|----:----|----:----|----:----|----:----|----:----|----:----|----:----|
7001 CCCTGTGGAGCTGTTTATCATGTGTATTTTCTGACACTTTGAAGACGTAACGTCTTAGCGAGACATCCTTATGAGGATTAATGTTGTAAAAACTTGTAAAGTCCACGTCCGAGTCTTCTTGTACTGAGTTATTGACTGTTAAATAGTTGTCCTCCATTTCCCCCTGATAGATGTCCCCCTGTTTTCCTCGTACTACAACT 7200
L S V E V F L V C L L S H F K Q M A S D S Q L F V G L * L M K S C K L H L S L L V H S L L Q C N I L L L Y L P V I * L P V F P A H H Q F6
P C R S L Y Y V Y F V T F S R C Q L I A R Y S Y E * N C C K Q V N * T C A * F F M V * Y S V I * * C S T F P S * R C P S L L L M I N F5
P V G R C I T C M F S Q S V E A N C F R E T P I S R I V V N K F M E P A P E S S C S E I V S L K D V P P L P P S D V P P C F S C S T S F4

S * W N Y N G T K G C E G * R R F R G C D V S C L C R R S * T L L T V F V P A V I R M F L L D E N I F C R L G W D T * F S F * Y F L W F1
A D G T T M E L K D V K D E G D S G D V M S L V S V E G L E L S * L F L F P L * * E C S C W M K I F F V D W D G T L N F L F N I S F G F2
L M E L Q W N * R M * R M K E I Q G M * C L L S L S K V L N S L D C F C S R C D K N V L V G * K Y F L * T G M G H L I F F L I F P L F3
7201 AGCTGATGGAACTACAATGGAACTAAAGGATGTGAAGGATGAAGGAGATTCAGGGGATGTGATGTCTCTTGTCTCTGTCGAAGGTCTTGAACTCTCTTGACTGTTTTTGTTCCCGCTGTGATAAGAATGTTCTTGTTGGATGAAAATATTTTTTGTAGACTGGGATGGGACACTTAATTTTCTTTTTAATATTTCCTTG 7400
----:----|----:----|----:----|----:----|----:----|----:----|----:----|----:----|----:----|----:----|----:----|----:----|----:----|----:----|----:----|----:----|----:----|----:----|----:----|----:----|
7201 TCGACTACCTTGATGTTACCTTGATTTCCTACACTTCCTACTTCCTCTAAGTCCCCTACACTACAGAGAACAGAGACAGCTTCCAGAACTTGAGAGAACTGACAAAAACAAGGGCGACACTATTCTTACAAGAACAACCTACTTTTATAAAAAACATCTGACCCTACCCTGTGAATTAAAAGAAAAATTATAAAGGAAC 7400
L Q H F * L P V L P H S P H L L N L P H S T E Q R Q R L D Q V R K V T K T G A T I L I N K N S S F I K Q L S P H S V * N K * Y K R Q F6
F S I S S C H F * L I H L I F S I * P I H H R K D R D F T K F E R S Q K Q E R Q S L F T R T P H F Y K K Y V P I P C K I K K K I N G K F5
A S P V V I S S F S T F S S P S E P S T I D R T E T S P R S S E Q S N K N G S H Y S H E Q Q I F I N K T S Q S P V S L K R K L I E K F4

S A R F H K T N V T V N R M R Q E F K Q N R * I N F N V I F S L A V S C * S * S T C S T N F R R V N V L R V T V N V N I V K G G D S R F1
L P G F T K Q M L Q L I E * G K N S N K T G K L I S M S S S A W L C L V S R S L H A V Q I L G G * M F S E S Q S M S I L * R E V I A F2
V C Q V S Q N K C Y S * * N E A R I Q T K Q V N * F Q C H L Q L G C V L L V V V Y M Q Y K F * E G E C S P S H S Q C Q Y C K G R * * Q F3
7401 GTCTGCCAGGTTTCACAAAACAAATGTTACAGTTAATAGAATGAGGCAAGAATTCAAACAAAACAGGTAAATTAATTTCAATGTCATCTTCAGCTTGGCTGTGTCTTGTTAGTCGTAGTCTACATGCAGTACAAATTTTAGGAGGGTGAATGTTCTCCGAGTCACAGTCAATGTCAATATTGTAAAGGGAGGTGATAGCA 7600
----:----|----:----|----:----|----:----|----:----|----:----|----:----|----:----|----:----|----:----|----:----|----:----|----:----|----:----|----:----|----:----|----:----|----:----|----:----|----:----|
7401 CAGACGGTCCAAAGTGTTTTGTTTACAATGTCAATTATCTTACTCCGTTCTTAAGTTTGTTTTGTCCATTTAATTAAAGTTACAGTAGAAGTCGAACCGACACAGAACAATCAGCATCAGATGTACGTCATGTTTAAAATCCTCCCACTTACAAGAGGCTCAGTGTCAGTTACAGTTATAACATTTCCCTCCACTATCGT 7600
D A L N * L V F T V L T L L I C S N L C F L Y I L K L T M K L K A T D Q * D Y D V H L V F K L L T F T R R T V T L T L I T F P P S L F6
T Q W T E C F L H * L * Y F S A L I * V F C T F * N * H * R * S P Q T K N T T T * M C Y L N * S P S H E G L * L * H * Y Q L P L H Y C F5
P R G P K V F C I N C N I S H P L F E F L V P L N I E I D D E A Q S H R T L R L R C A T C I K P P H I N E S D C D I D I N Y L S T I A F4

```
      F  E  V  F  F  G  M  R  L  *  I  D  G  N  F  H  L  T  E  P  A  H  T  L  *  L  *  S  M  L  H  V  C  R  K  I  K  F  *  I  F  F  V  I  C  I  I  C  K  Q  C  R  Q  L  V  F  M  Y  A  A  I  V  A  S  H  G     F1
     D  L  K  F  S  L  G  *  D  F  E  L  M  G  I  F  T  S  Q  S  L  H  I  H  F  N  F  N  P  C  S  M  S  V  E  K  *  N  F  E  Y  F  L  S  F  A  L  F  V  N  N  V  D  N  *  Y  L  C  M  Q  P  S  S  Q  A  M  G    F2
     I  *  S  F  L  W  D  E  T  L  N  *  W  E  F  S  P  H  R  A  C  T  Y  T  L  T  L  I  H  A  P  C  L  *  K  N  K  I  L  N  I  F  C  H  L  H  Y  L  *  T  M  *  T  I  S  I  Y  V  C  S  H  R  R  K  P  W  V   F3
7601 GATTTGAAGTTTTCTTTGGGATGAGACTTTGAATTGATGGGAATTTTCACCTCACAGAGCCTGCACATACACTTTAACTTTAATCCATGCTCCATGTCTGTAGAAAAATAAAATTTTGAATATTTTTTGTCATTTGCATTATTTGTAAACAATGTAGACAATTAGTATTTATGTATGCAGCCATCGTCGCAAGCCATGGG 7800
     ----:----|----:----|----:----|----:----|----:----|----:----|----:----|----:----|----:----|----:----|----:----|----:----|----:----|----:----|----:----|----:----|----:----|----:----|----:----|----:----|
7601 CTAAACTTCAAAAGAAACCCTACTCTGAAACTTAACTACCCTTAAAAGTGGAGTGTCTCGGACGTGTATGTGAAATTGAAATTAGGTACGAGGTACAGACATCTTTTTATTTTAAAACTTATAAAAAACAGTAAACGTAATAAACATTTGTTACATCTGTTAATCATAAATACATACGTCGGTAGCAGCGTTCGGTACCC 7800
     L  N  S  T  K  K  P  I  L  S  Q  I  S  P  F  K  *  R  V  S  G  A  C  V  S  *  S  *  D  M  S  W  T  Q  L  F  I  F  N  Q  I  N  K  T  M  Q  M  I  Q  L  C  H  L  C  N  T  N  I  Y  A  A  M  T  A  L  W  P   F6
      I  Q  L  K  R  Q  S  S  V  K  F  Q  H  S  N  E  G  *  L  A  Q  V  Y  V  K  V  K  I  W  A  G  H  R  Y  F  F  L  I  K  F  I  K  Q  *  K  C  *  K  Y  V  I  Y  V  I  L  I  *  T  H  L  W  R  R  L  G  H    F5
      S  K  F  N  E  K  P  H  S  K  S  N  I  P  I  K  V  E  C  L  R  C  M  C  K  L  K  L  G  H  E  M  D  T  S  F  Y  F  K  S  Y  K  K  D  N  A  N  N  T  F  L  T  S  L  *  Y  K  H  I  C  G  D  D  C  A  M  P   F4

     Y  V  Y  I  S  E  *  N  V  D  I  P  V  A  C  D  D  D  M  Q  Q  N  C  I  *  T  H  *  F  T  T  I  *  Y  P  V  S  I  I  N  L  T  S  T  T  N  E  K  Y  R  Q  L  S  S  F  T  K  Y  L  E  E  I  F  S  L  L  M   F1
      M  F  T  F  R  N  E  M  *  T  Y  P  W  L  A  T  M  I  C  S  K  T  A  Y  K  R  T  N  L  Q  Q  F  N  I  Q  Y  P  S  L  T  *  L  Q  P  Q  M  R  S  T  D  N  F  H  L  S  L  N  I  L  K  K  S  F  P  S  *  C   F2
      C  L  H  F  G  M  K  C  R  H  T  R  G  L  R  R  *  Y  A  A  K  L  H  I  N  A  L  I  Y  N  N  L  I  S  S  I  H  H  *  L  N  F  N  H  K  *  E  V  Q  T  T  F  I  F  H  *  I  S  *  R  N  L  F  P  P  D    F3
7801 TATGTTTACATTTCGGAATGAAATGTAGACATACCCGTGGCTTGCGACGATGATATGCAGCAAAACTGCATATAAACGCACTAATTTACAACAATTTAATATCCAGTATCCATCATTAACTTAACTTCAACCACAAATGAGAAGTACAGACAACTTTCATCTTTCACTAAATATCTTGAAGAAATCTTTTCCCTCCTGAT 8000
     ----:----|----:----|----:----|----:----|----:----|----:----|----:----|----:----|----:----|----:----|----:----|----:----|----:----|----:----|----:----|----:----|----:----|----:----|----:----|----:----|
7801 ATACAAATGTAAAGCCTTACTTTACATCTGTATGGGCACCGAACGCTGCTACTATACGTCGTTTTGACGTATATTTGCGTGATTAAATGTTGTTAAATTATAGGTCATAGGTAGTAATTGAATTGAAGTTGGTGTTTACTCTTCATGTCTGTTGAAAGTAGAAAGTGATTTATAGAACTTCTTTAGAAAAGGGAGGACTA 8000
      Y  T  *  M  E  S  H  F  T  S  M  G  T  A  Q  S  S  S  I  C  C  F  Q  M  Y  V  C  *  N  V  V  I  *  Y  G  T  D  M  M  L  K  V  E  V  V  F  S  F  Y  L  C  S  E  D  K  V  L  Y  R  S  S  I  K  E  R  R  I   F6
     T  H  K  C  K  P  I  F  H  L  C  V  R  P  K  R  R  H  Y  A  A  F  S  C  I  F  A  S  I  *  L  L  K  I  D  L  I  W  *  *  S  L  K  L  W  L  H  S  T  C  V  V  K  M  K  *  *  I  D  Q  L  F  R  K  G  G  S    F5
      I  N  V  N  R  F  S  I  Y  V  Y  G  H  S  A  V  I  I  H  L  L  V  A  Y  L  R  V  L  K  C  C  N  L  I  W  Y  G  D  N  V  *  S  *  G  C  I  L  L  V  S  L  K  *  R  E  S  F  I  K  F  F  D  K  G  E  Q    F4

      Q  P  *  F  F  F  S  N  Y  S  L  N  I  L  Y  K  V  N  L  K  S  I  H  Q  D  M  W  W  K  I  S  S  I  Q  I  L  L  E  T  L  I  I  G  L  C  Y  M  V  K  V  F  S  I  Y  N  *  C  I  C  T  C  S  T  C  M  F  E   F1
      N  L  N  F  F  L  V  I  I  L  *  T  F  Y  I  K  S  T  *  N  P  F  I  K  I  C  G  G  R  L  V  V  F  K  Y  F  L  K  L  *  L  *  V  F  A  I  W  *  K  F  F  L  F  T  I  N  V  Y  V  H  V  V  H  V  C  L    F2
     A  T  L  I  F  F  *  *  L  F  F  K  H  F  I  *  S  Q  L  E  I  H  S  S  R  Y  V  V  E  D  *  *  Y  S  N  T  S  *  N  F  D  Y  R  S  L  L  Y  G  E  S  F  F  Y  L  Q  L  M  Y  M  Y  M  *  Y  M  Y  V  *   F3
8001 GCAACCTTAATTTTTTTTTAGTAATTATTCTTTAAACATTTTATATAAAGTCAACTTGAAATCCATTCATCAAGATATGTGGTGGAAGATTAGTAGTATTCAAATACTTCTTGAAACTTTGATTATAGGTCTTTGCTATATGGTGAAAGTTTTTTCTATTTACAATTAATGTATATGTACATGTAGTACATGTATGTTTG 8200
     ----:----|----:----|----:----|----:----|----:----|----:----|----:----|----:----|----:----|----:----|----:----|----:----|----:----|----:----|----:----|----:----|----:----|----:----|----:----|----:----|
8001 CGTTGGAATTAAAAAAAAATCATTAATAAGAAATTTGTAAAATATATTTCAGTTGAACTTTAGGTAAGTAGTTCTATACACCACCTTCTAATCATCATAAGTTTATGAAGAACTTTGAAACTAATATCCAGAAACGATATACCACTTTCAAAAAAGATAAATGTTAATTACATATACATGTACATCATGTACATACAAAC 8200
      C  G  *  N  K  K  L  L  *  E  K  F  M  K  Y  L  T  L  K  F  D  M  *  *  S  I  H  H  F  I  L  L  I  *  I  S  R  S  V  K  I  I  P  R  Q  *  I  T  F  T  K  E  I  *  L  *  H  I  H  V  H  L  V  H  I  N    F6
     A  V  K  I  K  K  *  Y  N  N  K  L  C  K  I  Y  L  *  S  S  I  W  E  D  L  Y  T  T  S  S  *  Y  Y  E  F  V  E  Q  F  K  S  *  L  D  K  S  Y  P  S  L  K  K  *  K  C  N  I  Y  I  Y  M  Y  Y  M  Y  T  Q   F5
     H  L  R  L  K  K  K  T  I  I  R  *  V  N  *  I  F  D  V  Q  F  G  N  M  L  I  H  P  P  L  N  T  T  N  L  Y  K  K  F  S  Q  N  Y  T  K  A  I  H  H  F  N  K  R  N  V  I  L  T  Y  T  C  T  T  C  T  H  K    F4

      N  S  S  Q  M  F  K  L  C  S  I  *  L  F  R  *  D  Y  L  Q  H  L  G  K  C  V  I  L  N  I  I  *  T  F  V  E  T  F  S  C  S  S  Y  L  G  I  C  K  G  G  G  G  G  G  G  G  G  G  H  T  Y  L  D  E  I  M  Q    F1
     K  I  L  H  K  C  L  N  Y  V  Q  Y  D  F  S  D  K  I  I  S  S  I  *  E  S  V  L  Y  *  T  *  F  K  H  L  W  R  H  F  H  A  A  A  T  *  A  S  A  K  G  G  G  G  G  G  G  G  G  G  I  H  I  W  M  R  *  C  R   F2
     K  F  F  T  N  V  *  I  M  F  N  M  T  F  Q  I  R  L  S  P  A  F  R  K  V  C  Y  T  E  H  N  L  N  I  C  G  D  I  F  M  Q  Q  L  P  R  H  L  Q  R  G  G  G  G  G  G  G  G  G  A  Y  I  F  G  *  D  N  A  G   F3
8201 AAAATTCTTCACAAATGTTTAAATTATGTTCAATATGACTTTTCAGATAAGATTATCTCCAGCATTTAGGAAAGTGTGTTATACTGAACATAATTTAAACATTTGTGGAGACATTTTCATGCAGCAGCTACCTAGGCATCTGCAAAGGGGGGGGGGGGGGGGGGGGGGGGGGGGGGCATACATATTTGGATGAGATAATGCAG 8400
     ----:----|----:----|----:----|----:----|----:----|----:----|----:----|----:----|----:----|----:----|----:----|----:----|----:----|----:----|----:----|----:----|----:----|----:----|----:----|----:----|
8201 TTTTAAGAAGTGTTTACAAATTTAATACAAGTTATACTGAAAAGTCTATTCTAATAGAGGTCGTAAATCCTTTCACACAATATGACTTGTATTAAATTTGTAAACACCTCTGTAAAAGTACGTCGTCGATGGATCCGTAGACGTTTCCCCCCCCCCCCCCCCCCCCCCCCCCCCCCGTATGTATAAACCTACTCTATTACGTC 8400
     S  F  E  E  C  I  N  L  N  H  E  I  H  S  K  L  Y  S  *  R  W  C  K  P  F  H  T  I  S  F  M  I  *  V  N  T  S  V  N  E  H  L  L  *  R  P  M  Q  L  P  P  P  P  P  P  P  P  P  P  C  V  Y  K  S  S  I  I  C   F6
      F  N  K  V  F  T  *  I  I  N  L  I  V  K  *  I  L  N  D  G  A  N  L  F  T  H  *  V  S  C  L  K  F  M  Q  P  S  M  K  M  C  C  S  G  L  C  R  C  L  P  P  P  P  P  P  P  P  P  A  Y  M  N  P  H  S  L  A    F5
     F  I  R  *  L  H  K  F  *  T  *  Y  S  K  E  S  L  I  I  E  L  M  *  S  L  T  N  Y  Q  V  Y  N  L  C  K  H  L  C  K  *  A  A  A  V  *  A  D  A  F  P  P  P  P  P  P  P  P  P  P  M  C  I  Q  I  L  Y  H  L   F4

     D  N  M  Q  Q  L  R  G  *  I  E  F  H  *  L  C  V  L  L  S  G  L  S  L  F  I  E  F  S  V  *  T  K  L  F  N  D  I  F  Y  Y  T  S  H  Q  N  T  *  C  N  I  K  K  L  P  F  P  I  *  L  G  A  V  S  I  S  F   F1
      T  T  C  N  N  *  E  A  R  *  N  F  T  S  C  V  Y  Y  *  V  A  S  P  Y  S  *  S  L  V  F  K  L  N  F  L  M  I  Y  F  I  T  Q  V  T  R  T  P  S  V  I  *  K  N  C  L  S  Q  S  D  W  G  L  F  L  F  L  S   F2
      Q  H  A  T  I  K  R  L  D  R  I  S  L  A  V  C  I  I  E  W  P  L  P  I  H  R  V  *  C  L  N  *  T  F  *  *  Y  I  L  L  H  K  S  P  E  H  L  V  *  Y  K  K  I  A  F  P  N  L  I  G  G  C  F  Y  F  F    F3
8401 GACAACATGCAACAATTAAGAGGCTAGATAGAATTTCACTAGCTGTGTGTATTATTGAGTGGCCTCTCCCTATTCATAGAGTTTAGTGTTTAAACTAAACTTTTTAATGATATATTTTATTACACAAGTCACCAGAACACCTAGTGTAATATAAAAAAATTGCCTTTCCCAATCTGATTGGGGGCTGTTTCTATTTCTTT 8600
     ----:----|----:----|----:----|----:----|----:----|----:----|----:----|----:----|----:----|----:----|----:----|----:----|----:----|----:----|----:----|----:----|----:----|----:----|----:----|----:----|
8401 CTGTTGTACGTTGTTAATTCTCCGATCTATCTTAAAGTGATCGACACACATAATAACTCACCGGAGAGGGATAAGTATCTCAAATCACAAATTTGATTTGAAAAATTACTATATAAAATAATGTGTTCAGTGGTCTTGTGGATCACATTATATTTTTTTAACGGAAAGGGTTAGACTAACCCCCGACAAAGATAAAGAAA 8600
      S  L  M  C  C  N  L  P  *  I  S  N  *  *  S  H  T  N  N  L  P  R  E  R  N  M  S  N  L  T  *  V  L  S  K  L  S  I  N  *  *  V  L  *  W  F  V  *  H  L  I  F  F  N  G  K  G  I  Q  N  P  A  T  E  I  E  K   F6
     P  C  C  A  V  I  L  L  S  S  L  I  E  S  A  T  H  I  I  S  H  G  R  G  I  *  L  T  *  H  K  F  *  V  K  *  H  Y  I  K  N  C  L  D  G  S  C  R  T  Y  Y  L  F  I  A  K  G  L  R  I  P  P  Q  K  *  K  K   F5
      V  V  H  L  L  *  S  A  L  Y  F  K  V  L  Q  T  Y  *  Q  T  A  E  G  *  E  Y  L  K  T  N  L  S  F  K  K  I  I  Y  K  I  V  C  T  V  L  V  G  L  T  I  Y  F  F  Q  R  E  W  D  S  Q  P  S  N  R  N  R    F4

      S  F  L  L  R  F  V  Q  *  R  L  K  N  A  H  F  H  L  F  C  T  K  T  F  E  F  Q  S  M  C  T  H  I  Y  R  *  C  T  Q  L  K  K  I  F  S  V  S  M  C  P  K  R  P  W  *  N  Y  R  L  S  Q  K  N  F  K  T  *   F1
      H  F  Y  F  G  L  F  S  E  G  *  K  T  P  I  F  T  F  S  A  L  R  L  L  S  F  S  Q  C  V  P  I  F  I  G  S  V  P  N  *  K  K  Y  F  L  Y  L  C  V  L  N  D  L  G  K  I  I  A  F  L  R  K  I  S  K  R    F2
     L  I  F  T  S  V  C  S  V  K  A  K  K  R  P  F  S  P  F  L  H  *  D  F  *  V  S  V  N  V  Y  P  Y  L  S  V  V  V  Y  P  I  K  K  N  I  F  C  I  Y  V  S  *  T  T  L  V  K  L  S  P  F  S  E  K  F  Q  N  V   F3
8601 CTCATTTTTACTTCGGTTTGTTCAGTGAAGGCTAAAAAACGCCCATTTTCACCTTTTCTGCACTAAGACTTTTGAGTTTCAGTCAATGTGTACCCATATTTATCGGTAGTGTACCCAATTAAAAAAAATATTTTCTGTATCTATGTGTCCTAAACGACCTTGGTAAAATTATCGCCTTTCTCAGAAAAATTTCAAAACGT 8800
     ----:----|----:----|----:----|----:----|----:----|----:----|----:----|----:----|----:----|----:----|----:----|----:----|----:----|----:----|----:----|----:----|----:----|----:----|----:----|----:----|
8601 GAGTAAAAATGAAGCCAAACAAGTCACTTCCGATTTTTTGCGGGTAAAAGTGGAAAAGACGTGATTCTGAAAACTCAAAGTCAGTTACACATGGGTATAAATAGCCATCACATGGGTTAATTTTTTTTATAAAAGACATAGATACACAGGATTTGCTGGAACCATTTTAATAGCGGAAAGAGTCTTTTTAAAGTTTTGCA 8800
      E  N  K  S  R  N  T  *  H  L  S  F  F  A  W  K  *  R  K  Q  V  L  V  K  S  N  *  D  I  H  V  W  I  *  R  Y  H  V  W  N  F  F  I  N  E  T  D  I  H  G  L  R  G  Q  Y  F  *  R  R  E  *  F  F  K  L  V    F6
     R  M  K  V  E  T  Q  E  T  F  A  L  F  R  G  N  E  G  K  R  C  *  S  K  Q  T  E  T  L  T  Y  G  Y  K  D  T  T  Y  G  I  L  F  F  I  K  Q  I  *  T  D  *  V  V  K  T  F  N  D  G  K  E  S  F  N  *  F  T   F5
     E  *  K  *  K  P  K  N  L  S  P  *  F  V  G  M  K  V  K  E  A  S  L  S  K  L  K  L  *  H  T  G  M  N  I  P  L  T  G  L  *  F  F  Y  K  R  Y  R  H  T  R  F  S  R  P  L  I  I  A  K  R  L  F  I  E  F  R   F4

      Y  K  F  F  Y  F  Y  S  R  D  L  T  F  I  S  K  I  V  P  E  V  N  K  I  N  F  *  I  T  V  S  I  F  F  L  S  T  D  L  N  S  S  H  G  I  G  F  D  I  Q  C  T  F  C  F  S  *  V  D  K  H  S  A  R  L  A    F1
     S  I  N  S  F  I  F  I  H  E  T  L  L  L  S  Q  K  L  Y  R  K  *  T  K  S  I  F  K  L  P  F  Q  F  F  F  F  R  P  I  *  T  Q  V  M  A  S  G  S  T  F  N  V  L  S  V  F  R  K  *  T  N  I  R  H  A  L  R   F2
     V  *  I  L  L  F  L  F  T  R  P  Y  F  Y  L  K  N  C  T  G  S  E  Q  N  Q  F  L  N  Y  R  F  N  F  F  S  F  D  R  S  K  L  K  S  W  H  R  V  R  H  S  M  Y  F  L  F  F  V  S  R  Q  T  F  G  T  P  C  A   F3
8801 AGTATAAATTCTTTTATTTTTATTCACGAGACCTTACTTTTATCTCAAAAATTGTACCGGAAGTGAACAAAATCAATTTTTAAATTACCGTTTCAATTTTTTTTCTTTCGACCGATCTAAACTCAAGTCATGGCATCGGGTTCGACATTCAATGTACTTTCTGTTTTTCGTAAGTAGACAAACATTCGGCACGCCTTGCG 9000
     ----:----|----:----|----:----|----:----|----:----|----:----|----:----|----:----|----:----|----:----|----:----|----:----|----:----|----:----|----:----|----:----|----:----|----:----|----:----|----:----|
8801 TCATATTTAAGAAAATAAAAATAAGTGCTCTGGAATGAAAATAGAGTTTTTAACATGGCCTTCACTTGTTTTAGTTAAAAATTTAATGGCAAAGTTAAAAAAAAGAAAGCTGGCTAGATTTGAGTTCAGTACCGTAGCCCAAGCTGTAAGTTACATGAAAGACAAAAAGCATTCATCTGTTTGTAAGCCGTGCGGAACGC 9000
     Y  Y  L  N  K  *  K  *  E  R  S  R  V  K  I  E  F  I  T  G  S  T  F  L  I  L  K  *  I  V  T  E  I  K  K  R  E  V  S  R  F  E  L  *  P  M  P  N  S  M  *  H  V  K  Q  K  E  Y  T  S  L  C  E  A  R  R  A   F6
      T  Y  I  R  K  N  K  N  V  L  G  *  K  *  R  L  F  Q  V  P  L  S  C  F  *  N  K  F  *  R  K  L  K  K  E  K  S  R  D  L  S  L  D  H  C  R  T  R  C  E  I  Y  K  R  N  K  T  L  L  C  V  N  P  V  G  Q    F5
     L  I  F  E  K  I  K  I  *  S  V  K  S  K  D  *  F  N  Y  R  F  H  V  F  D  I  K  L  N  G  N  *  N  K  K  K  R  G  I  *  V  *  T  M  A  D  P  E  V  N  L  T  S  E  T  K  R  L  Y  V  F  M  R  C  A  K  R   F4

     H  V  A  C  L  M  L  F  K  L  K  R  S  V  E  S  T  R  D  V  T  D  V  I  Q  C  I  Q  T  W  L  T  E  Q  E  T  G  S  L  I  V  G  K  I  X   F1
      M  *  H  V  *  C  F  S  N  *  S  G  V  L  K  A  P  V  T  S  Q  M  *  Y  S  V  F  K  H  G  *  L  S  R  R  R  E  V  *  *  W  G  R  *   F2
      C  S  M  F  D  A  F  Q  T  E  A  E  C  *  K  H  P  *  R  H  R  C  D  T  V  Y  S  N  M  A  D  *  A  G  D  G  K  F  D  S  G  E  D  X   F3
9001 CATGTAGCATGTTTGATGCTTTTCAAACTGAAGCGGAGTGTTGAAAGCACCCGTGACGTCACAGATGTGATACAGTGTATTCAAACATGGCTGACTGAGCAGGAGACGGGAAGTTTGATAGTGGGGAAGATAG 9133
     ----:----|----:----|----:----|----:----|----:----|----:----|----:----|----:----|----:----|----:----|----:----|----:----|----:----|---
9001 GTACATCGTACAAACTACGAAAAGTTTGACTTCGCCTCACAACTTTCGTGGGCACTGCAGTGTCTACACTATGTCACATAAGTTTGTACCGACTGACTCGTCCTCTGCCCTTCAAACTATCACCCCTTCTATC 9133
      C  T  A  H  K  I  S  K  L  S  F  R  L  T  S  L  V  R  S  T  V  S  T  I  C  H  I  *  V  H  S  V  S  C  S  V  P  L  K  I  T  P  F  I   F6
     A  H  L  M  N  S  A  K  *  V  S  A  S  H  Q  F  C  G  H  R  *  L  H  S  V  T  Y  E  F  M  A  S  Q  A  P  S  P  F  N  S  L  P  S  S  L   F5
      M  Y  C  T  Q  H  K  E  F  Q  L  P  T  N  F  A  G  T  V  D  C  I  H  Y  L  T  N  L  C  P  Q  S  L  L  L  R  S  T  Q  Y  H  P  L  Y   F4
```

Saccostrea glomerata (Sgl)

WGS: PRKT01001405.1

80139-88294
Saccostrea glomerata isolate SGL-2013-L4 SGL_1404, whole genome shotgun sequence

```
     L T V H D E Y L Q T I P C I A F * I T F N K S F E K N Y K K K C R S R * I E L F S T R R T C F I T Y Q * L Y L F I F W W C Q T R A R E   F1
    * L C M M S I Y K Q F R A L P F K S H S T N P L R R I T R K S A A A D E L N Y F P P E G H V S L P I N S Y T C L Y F G G A R R G Q E S  F2
     N C A * * V F T N N S V H C L L N H I Q Q I L * E E L Q E K V P Q Q M N * I I F H Q K D M F H Y L S I V I L V Y I L V V P D E G K R   F3
   1 TTAACTGTGCATGATGAGTATTTACAAACAATTCCGTGCATTGCCTTTTAAATCACATTCAACAAATCCTTTGAGAAGAATTACAAGAAAAAGTGCCGCAGCAGATGAATTGAATTATTTTCCACCAGAAGGACATGTTTCATTACCTATCAATAGTTATACTTGTTTATATTTTGGTGGTGCCAGACGAGGGCAAGAGA 200
     ----:----|----:----|----:----|----:----|----:----|----:----|----:----|----:----|----:----|----:----|----:----|----:----|----:----|----:----|----:----|----:----|----:----|----:----|----:----|----:----|
   1 AATTGACACGTACTACTCATAAATGTTTGTTAAGGCACGTAACGGAAAATTTAGTGTAAGTTGTTTAGGAAACTCTTCTTAATGTTCTTTTTCACGGCGTCGTCTACTTAACTTAATAAAAGGTGGTCTTCCTGTACAAAGTAATGGATAGTTATCAATATGAACAAATATAAAACCACCACGGTCTGCTCCCGTTCTCT 200
     X V T C S S Y K C V I G H M A K * I V N L L D K S F F * L F F H R L L H I S N N E V L L V H K M V * * Y N Y K N I N Q H H W V L A L S   F6
     X L Q A H H T N V F L E T C Q R K F * M * C I R Q S S N C S F F T G C C I F Q I I K W W F S M N * * R D I T I S T * I K T T G S S P L L  F5
     * S H M I L I * L C N R A N G K L D C E V F G K L L I V L F L A A A S S N F * K G G S P C T E N G I L L * V Q K Y K P P A L R P C S    F4

     H L E Y V K Q I I Q S I L * C * * Q * C Q C * * Y I * N * A F R W A V S T L A V K C W F L Y Y T S E L F I C L G W S Q F V M F F Y V K   F1
     T W S M S N K L Y K V S F D V D D N D V N V D S I S E I K L S G G Q F P H L Q S S A G F Y I T P Q N C L F V W G G L N L S C F S M S   F2
     A L G V C Q T N Y T K Y P L M L M T M M S M L I V Y L K L S F Q V G S F H T C S Q V L V F I L H L R I V Y L F G V V S I C H V F L C Q   F3
 201 GCACTTGGAGTATGTCAAACAAATTATACAAAGTATCCTTTGATGTTGATGACAATGATGTCAATGTTGATAGTATATCTGAAATTAAGCTTTCAGGTGGGCAGTTTCCACACTTGCAGTCAAGTGCTGGTTTTTATATTACACCTCAGAATTGTTTATTTGTTTGGGGTGGTCTCAATTTGTCATGTTTTTCTATGTCA 400
     ----:----|----:----|----:----|----:----|----:----|----:----|----:----|----:----|----:----|----:----|----:----|----:----|----:----|----:----|----:----|----:----|----:----|----:----|----:----|----:----|
 201 CGTGAACCTCATACAGTTTGTTTAATATGTTTCATAGGAAACTACAACTACTGTTACTACAGTTACAACTATCATATAGACTTTAATTCGAAAGTCCACCCGTCAAAGGTGTGAACGTCAGTTCACGACCAAAAATATAATGTGGAGTCTTAACAAATAAACAAACCCCACCAGAGTTAAACAGTACAAAAAGATACAGT 400
     C K S Y T L C I I C L I R Q H Q H C H H * H Q Y Y I Q F * A K L H A T E V S A T L H Q N K Y * V E S N N I Q K P H D * N T M N K * T   F6
     A S P T H * V F * V F Y G K I N I V I I D I N I T Y R F N L K * T P L K W V Q L * T S T K I N C R L I T * K N P T T E I Q * T K R H *  F5
     L V Q L I D F L N Y L T D K S T S S L S T L T S L I D S I L S E P P C N G C K C D L A P K * I V G * F Q K N T Q P P R L K D H K E I D   F4

     * I V Y C Q I K * Q E R C S * N C T A T R W H F F A R I R G * N S L R * V W T Y T Y T Y F * L L C H F T W W R L F S K Q K Y M C W F   F1
     N E L Y I V K L N D K K G V V E I V Q P P G G I S L R E L G G E I P S G R C G H T L T H I F N C C V I L H G G V C F P N R N T C V G S   F2
     M N C I L S N * M T R K V * L K L Y S H Q V A F L C E N * G V K F P P V G V D I H L H I F L I V V S F Y M V A F V F Q T E I H V L V H   F3
 401 AATGAATTGTATATTGTCAAATTAAATGACAAGAAAGGTGTAGTTGAAATTGTACAGCCACCAGGTGGCATTTCTTTGCGAGAATTAGGGGGTGAAATTCCCTCCGGTAGGTGTGGACATACACTTACACATATTTTTAATTGTTGTGTCATTTTACATGGTGGCGTTTGTTTTCCAAACAGAAATACATGTGTTGGTTC 600
     ----:----|----:----|----:----|----:----|----:----|----:----|----:----|----:----|----:----|----:----|----:----|----:----|----:----|----:----|----:----|----:----|----:----|----:----|----:----|----:----|
 401 TTACTTAACATATAACAGTTTAATTTACTGTTCTTTCCACATCAACTTTAACATGTCGGTGGTCCACCGTAAAGAAACGCTCTTAATCCCCCACTTTAAGGGAGGCCATCCACACCTGTATGTGAATGTGTATAAAAATTAACAACACAGTAAAATGTACCACCGCAAACAAAAGGTTTGTCTTTATGTACACAACCAAG 600
     L H I T Y Q * I L H C S L H L Q F Q V A V L H C K K A L I L P H F E R R Y T H V Y V * V Y K * N N H * K V H H R K N E L C F Y M H Q N   F6
     I F Q I N D F * I V L F T Y N F N Y L W W T A N R Q S F * P T F N G G T P T S M C K C M N K I T T D N * M T A N T K W V S I C T N T  F5
     F S N Y I T L N F S L F P T T S I T C G G P P M E K R S N P P S I G E P L H P C V S V C I K L Q Q T M K C P P T Q K G F L F V H T P E   F4

     I F I Y K C N Q * * * F L Y V * F * K S L L D Q T F C V W T * A K S L S H C Q H Y G Y Q R Y E V Y C V Y W R C Y K D * K * S S Q N T V   F1
     S L F T N V T N D S S F Y M F D F E S L F W T K L S V S G P E P R A Y H T A N I M D I K G M R S I V Y I G G V T K T E N D L R R I P L   F2
     L Y L Q M * P M I V V F I C L I L K V S F G P N F L C L D L S Q E L I T L P T L W I S K V * G L L C I L A V L Q R L K M I F A E Y R   F3
 601 ATCTTTATTTACAAATGTAACCAATGATAGTAGTTTTTATATGTTTGATTTTGAAAGTCTCTTTTGGACCAAACTTTCTGTGTCTGGACCTGAGCCAAGAGCTTATCACACTGCCAACATTATGGATATCAAAGGTATGAGGTCTATTGTGTATATTGGCGGTGTTACAAAGACTGAAAATGATCTTCGCAGAATACCGT 800
     ----:----|----:----|----:----|----:----|----:----|----:----|----:----|----:----|----:----|----:----|----:----|----:----|----:----|----:----|----:----|----:----|----:----|----:----|----:----|----:----|
 601 TAGAAATAAATGTTTACATTGGTTACTATCATCAAAAATATACAAACTAAAACTTTCAGAGAAAACCTGGTTTGAAAGACACAGACCTGGACTCGGTTCTCGAATAGTGTGACGGTTGTAATACCTATAGTTTCCATACTCCAGATAACACATATAACCGCCACAATGTTTCTGACTTTTACTAGAAGCGTCTTATGGCA 800
     M K I * L H L W H Y Y N K Y T Q N Q F D R K S W V K Q T Q V Q A L L K D C Q W C * P Y * L Y S T * Q T Y Q R H * L S Q F H D E C F V T   F6
     * R * K C I Y G I I T T K I H K I K F T E K P G F K R H R S R L W S S I V S G V N H I D F T H P R N H I N A T N C L S F I I K A S Y R  F5
     D K N V F T V L S L L K * I N S K S L R K Q V L S E T D P G S G L A * * V A L M I S I L P I L D I T Y I P P T V F V S F S R R L I G   F4

     I X X X X X X X X X X X X X X X X X X X X X X X X X X X X X X X X X X X X X X X X X X X X X X X X X X X X X X X X X X X X X X X X X X   F1
     S X X X X X X X X X X X X X X X X X X X X X X X X X X X X X X X X X X X X X X X X X X X X X X X X X X X X X X X X X X X X X X X X X   F2
     Y X X X X X X X X X X X X X X X X X X X X X X X X X X X X X X X X X X X X X X X X X X X X X X X X X X X X X X X X X X X X X X X X X   F3
 801 TATCNNNNNNNNNNNNNNNNNNNNNNNNNNNNNNNNNNNNNNNNNNNNNNNNNNNNNNNNNNNNNNNNNNNNNNNNNNNNNNNNNNNNNNNNNNNNNNNNNNNNNNNNNNNNNNNNNNNNNNNNNNNNNNNNNNNNNNNNNNNNNNNNNNNNNNNNNNNNNNNNNNNNNNNNNNNNNNNNNNNNNNNNNNNNNNNNNNNNNNNNNN 1000
     ----:----|----:----|----:----|----:----|----:----|----:----|----:----|----:----|----:----|----:----|----:----|----:----|----:----|----:----|----:----|----:----|----:----|----:----|----:----|----:----|
 801 ATAGNNNNNNNNNNNNNNNNNNNNNNNNNNNNNNNNNNNNNNNNNNNNNNNNNNNNNNNNNNNNNNNNNNNNNNNNNNNNNNNNNNNNNNNNNNNNNNNNNNNNNNNNNNNNNNNNNNNNNNNNNNNNNNNNNNNNNNNNNNNNNNNNNNNNNNNNNNNNNNNNNNNNNNNNNNNNNNNNNNNNNNNNNNNNNNNNNNNNNNNNNNNNN 1000
     I X X X X X X X X X X X X X X X X X X X X X X X X X X X X X X X X X X X X X X X X X X X X X X X X X X X X X X X X X X X X X X X X X   F6
     * X X X X X X X X X X X X X X X X X X X X X X X X X X X X X X X X X X X X X X X X X X X X X X X X X X X X X X X X X X X X X X X X X  F5
     N D X X X X X X X X X X X X X X X X X X X X X X X X X X X X X X X X X X X X X X X X X X X X X X X X X X X X X X X X X X X X X X X X   F4

     X X X X X X X X X X X X X X X X X X X X X X X X X X X X X X X X X X X X X X X X X X X X X X X X X X X X X X X X X X X X X X X X X X   F1
     X X X X X X X X X X X X X X X X X X X X X X X X X X X X X X X X X X X X X X X X X X X X X X X X X X X X X X X X X X X X X X X X X X   F2
     X X X X X X X X X X X X X X X X X X X X X X X X X X X X X X X X X X X X X X X X X X X X X X X X X X X X X X X X X X X X X X X X X X   F3
1001 NNNNNNNNNNNNNNNNNNNNNNNNNNNNNNNNNNNNNNNNNNNNNNNNNNNNNNNNNNNNNNNNNNNNNNNNNNNNNNNNNNNNNNNNNNNNNNNNNNNNNNNNNNNNNNNNNNNNNNNNNNNNNNNNNNNNNNNNNNNNNNNNNNNNNNNNNNNNNNNNNNNNNNNNNNNNNNNNNNNNNNNNNNNNNNNNNNNNNNNNNNNNNNN 1200
     ----:----|----:----|----:----|----:----|----:----|----:----|----:----|----:----|----:----|----:----|----:----|----:----|----:----|----:----|----:----|----:----|----:----|----:----|----:----|----:----|
1001 NNNNNNNNNNNNNNNNNNNNNNNNNNNNNNNNNNNNNNNNNNNNNNNNNNNNNNNNNNNNNNNNNNNNNNNNNNNNNNNNNNNNNNNNNNNNNNNNNNNNNNNNNNNNNNNNNNNNNNNNNNNNNNNNNNNNNNNNNNNNNNNNNNNNNNNNNNNNNNNNNNNNNNNNNNNNNNNNNNNNNNNNNNNNNNNNNNNNNNNNNNNNNNNN 1200
     X X X X X X X X X X X X X X X X X X X X X X X X X X X X X X X X X X X X X X X X X X X X X X X X X X X X X X X X X X X X X X X X X X   F6
     X X X X X X X X X X X X X X X X X X X X X X X X X X X X X X X X X X X X X X X X X X X X X X X X X X X X X X X X X X X X X X X X X X   F5
     X X X X X X X X X X X X X X X X X X X X X X X X X X X X X X X X X X X X X X X X X X X X X X X X X X X X X X X X X X X X X X X X X X   F4

     X X X X X X X X X X X X X X X X X X X X X X X X X X X X X X X X X X X X X X X X X X X X X X X X X X X X X X X X X X X X X X X X X X   F1
     X X X X X X X X X X X X X X X X X X X X X X X X X X X X X X X X X X X X X X X X X X X X X X X X X X X X X X X X X X X X X X X X X X   F2
     X X X X X X X X X X X X X X X X X X X X X X X X X X X X X X X X X X X X X X X X X X X X X X X X X X X X X X X X X X X X X X X X X X   F3
1201 NNNNNNNNNNNNNNNNNNNNNNNNNNNNNNNNNNNNNNNNNNNNNNNNNNNNNNNNNNNNNNNNNNNNNNNNNNNNNNNNNNNNNNNNNNNNNNNNNNNNNNNNNNNNNNNNNNNNNNNNNNNNNNNNNNNNNNNNNNNNNNNNNNNNNNNNNNNNNNNNNNNNNNNNNNNNNNNNNNNNNNNNNNNNNNNNNNNNNNNNNNNNNNN 1400
     ----:----|----:----|----:----|----:----|----:----|----:----|----:----|----:----|----:----|----:----|----:----|----:----|----:----|----:----|----:----|----:----|----:----|----:----|----:----|----:----|
1201 NNNNNNNNNNNNNNNNNNNNNNNNNNNNNNNNNNNNNNNNNNNNNNNNNNNNNNNNNNNNNNNNNNNNNNNNNNNNNNNNNNNNNNNNNNNNNNNNNNNNNNNNNNNNNNNNNNNNNNNNNNNNNNNNNNNNNNNNNNNNNNNNNNNNNNNNNNNNNNNNNNNNNNNNNNNNNNNNNNNNNNNNNNNNNNNNNNNNNNNNNNNNNN 1400
     X X X X X X X X X X X X X X X X X X X X X X X X X X X X X X X X X X X X X X X X X X X X X X X X X X X X X X X X X X X X X X X X X X   F6
     X X X X X X X X X X X X X X X X X X X X X X X X X X X X X X X X X X X X X X X X X X X X X X X X X X X X X X X X X X X X X X X X X X   F5
     X X X X X X X X X X X X X X X X X X X X X X X X X X X X X X X X X X X X X X X X X X X X X X X X X X X X X X X X X X X X X X X X X X   F4

     X X X X X X X X X X X X X X X X X X X X L C H F T W W R L F S K Q K Y M C W F I F I Y K C N Q * * * F L Y V * F * K S L L D Q T F C V   F1
     X X X X X X X X X X X X X X X X X X X X C V I L H G G V C F P N R N T C V G S S L F T N V T N D S S F Y M F D F E S L F W T K L S V   F2
     X X X X X X X X X X X X X X X X X X X X X V S F Y M V A F V F Q T E I H V L V H L Y L Q M * P M I V V F I C L I L K V S F G P N F L C   F3
1401 NNNNNNNNNNNNNNNNNNNNNNNNNNNNNNNNNNNNNNNNNNNNNNNNNNNNNNNNNNNNNTTGTGTCATTTTACATGGTGGCGTTTGTTTTCCAAACAGAAATACATGTGTTGGTTCATCTTTATTTACAAATGTAACCAATGATAGTAGTTTTTATATGTTTGATTTTGAAAGTCTCTTTTGGACCAAACTTTCTGTG 1600
     ----:----|----:----|----:----|----:----|----:----|----:----|----:----|----:----|----:----|----:----|----:----|----:----|----:----|----:----|----:----|----:----|----:----|----:----|----:----|----:----|
1401 NNNNNNNNNNNNNNNNNNNNNNNNNNNNNNNNNNNNNNNNNNNNNNNNNNNNNNNNNNNNNAACACAGTAAAATGTACCACCGCAAACAAAAGGTTTGTCTTTATGTACACAACCAAGTAGAAATAAATGTTTACATTGGTTACTATCATCAAAAATATACAAACTAAAACTTTCAGAGAAAACCTGGTTTGAAAGACAC 1600
     X X X X X X X X X X X X X X X X X X X X X H * K V H H R K N E L C F Y M H Q N M K I * L H L W H Y Y N K Y T Q N Q F D R K S W V K Q   F6
     X X X X X X X X X X X X X X X X X X X X X T D N * M T A N T K W V S I C T N T * R * K C I Y G I I T T K I H K I K F T E K P G F K R H  F5
     X X X X X X X X X X X X X X X X X X X X X Q T M K C P P T Q K G F L F V H T P E D K N V F T V L S L L K * I N S K S L R K Q V L S E T   F4

     W T * A K S L S H C Q H Y G Y Q R Y E V Y C V Y W R C Y K D * K * S S Q N T V I E C T Y F E D G F * Q A F S H R N S N I C Q F S C C   F1
     S G P E P R A Y H T A N I M D I K G M R S I V Y I G G V T K T E N D L R R I P L S N V L I L K M D S D K H F H T E I L I F A N S P A V   F2
     L D L S Q E L I T L P T L W I S K V * G L L C I L A V L Q R L K M I F A E Y R Y R M Y L F * R W I L T S I F T Q K F * Y L P I L L L L   F3
1601 TCTGGACCTGAGCCAAGAGCTTATCACACTGCCAACATTATGGATATCAAAGGTATGAGGTCTATTGTGTATATTGGCGGTGTTACAAAGACTGAAAATGATCTTCGCAGAATACCGTTATCGAATGTACTTATTTTGAAGATGGATTCTGACAAGCATTTTCACACAGAAATTCTAATATTTGCCAATTCTCCTGCTGT 1800
     ----:----|----:----|----:----|----:----|----:----|----:----|----:----|----:----|----:----|----:----|----:----|----:----|----:----|----:----|----:----|----:----|----:----|----:----|----:----|----:----|
1601 AGACCTGGACTCGGTTCTCGAATAGTGTGACGGTTGTAATACCTATAGTTTCCATACTCCAGATAACACATATAACCGCCACAATGTTTCTGACTTTTACTAGAAGTGTCTTATGGCAATAGCTTACATGAATAAAACTTCTACCTAAGACTGTTCGTAAAAGTGTGTCTTTAAGATTATAAACGGTTAAGAGGACGACA 1800
     T Q V Q A L L K D C Q W C * P Y * L Y S T * Q T Y Q R H * L S Q F H D E C F V T I S H V * K S S P N Q C A N E C L F E L I Q W N E Q Q   F6
     R S R L W S S I V S G V N H I D F T H P R N H I N A T N C L S F I I K V S Y R * R I Y K N Q L H I R V L M K V C F N * Y K G I R R S  F5
     D P G S G L A * * V A L M I S I L P I L D I T Y I P P T V F V S F S R * L I G N D F T S I K F I S E S L C K * V S I R I N A L E G A T   F4

     W C F I P F N R S D W P I H I C C W R G G * E * S S R Q K F C F N I K C R N I F M * R Y S Q V * P V F * V S W S F S M Y T L S * * L F D D   F1
     G V S Y H S T G V I G P Y I F V V G G V D E N N L Q G R N S V S I L N V E T F S C E D I Q F D Q S F R S A G H S V C T L S D D C L M I   F2
     V F H T I Q Q E * L A H T Y L L L E G W M R I I F K A E I L F Q Y * M * K H F H V K I F S L T S L L G Q L V I Q Y V H C L M I V * M I   F3
1801 TGGTGTTTCATACCATTCAACAGGAGTGATTGGCCCATACATATTTGTTGTTGGAGGGGTGGATGAGAATAATCTTCAAGGCAGAAATTCTGTTTCAATATTAAATGTAGAAACATTTTCATGTGAAGATATTCAGTTTGACCAGTCTTTTAGGTCAGCTGGTCATTCAGTATGTACACTGTCTGATGATTGTTTGATGA 2000
     ----:----|----:----|----:----|----:----|----:----|----:----|----:----|----:----|----:----|----:----|----:----|----:----|----:----|----:----|----:----|----:----|----:----|----:----|----:----|----:----|
1801 ACCACAAAGTATGGTAAGTTGTCCTCACTAACCGGGTATGTATAAACAACAACCTCCCCACCTACTCTTATTAGAAGTTCCGTCTTTAAGACAAAGTTATAATTTACATCTTTGTAAAAGTACACTTCTATAAGTCAAACTGGTCAGAAAATCCAGTCGACCAGTAAGTCATACATGTGACAGACTACTAACAAACTACT 2000
     Q H K M G N L L L S Q G M C I Q Q Q L P P H S Y D E L C F N Q K L I L H L F M K M H L Y E T Q G T K * T L Q D N L I Y V T Q H N N S S   F6
     N T N * V M * C S H N A N N S P T * I I L H L I L K L A S I R N * Y * I Y F C K * T F I N L K V L R K P * S T M * Y T C Q R I I T Q H  F5
     P T E Y W E V P T I P G Y M N T T P P T S S F L R * P L F E T E I N F T S V N E H S S I * N S W D K L D A P * E T H V S D S S Q K I   F4

     M W W Y A F A I F C F Y * * T N G T K S M * F * K * L * N Y * I H * N I S N I L D S V * G Y L * K M V T S I L C W S F R Y R H E S * K   F1
     C G G M H L Q Y F V F T S K P M V P S P C D F E N D C R I I E S T E T S P I S W I Q C E G T C K R W L H Q F C V G V L D I D M S R K   F2
     Y V V V C I C N I L F L L V N Q W Y Q V H V I L K M I V E L L N P L K H L Q Y L G F S V R V L V K D G Y I N F V L E F * I S T * V V K   F3
2001 TATGTGGTGGTATGCATTTGCAATATTTTGTTTTTACTAGTAAACCAATGGTACCAAGTCCATGTGATTTTGAAAATGATTGTAGAATTATTGAATCCACTGAAACATCTCCAATATCTTGGATTCAGTGTGAGGGTACTTGTAAAAGATGGTTACATCAATTTTGTGTTGGAGTTTTAGATATCGACATGAGTCGTAAA 2200
     ----:----|----:----|----:----|----:----|----:----|----:----|----:----|----:----|----:----|----:----|----:----|----:----|----:----|----:----|----:----|----:----|----:----|----:----|----:----|----:----|
2001 ATACACCACCATACGTAAACGTTATAAAACAAAAATGATCATTTGGTTACCATGGTTCAGGTACACTAAAACTTTTACTAACATCTTAATAACTTAGGTGACTTTGTAGAGGTTATAGAACCTAAGTCACACTCCCATGAACATTTTCTACCAATGTAGTAAAACACAACCTCAAAATCTATAGCTGTACTCAGCATTT 2200
     I H H Y A N A I N K Q K * Y V L F V L D M H N Q F H N Y F Q I W Q F M E L I K S E T H P Y K Y F I T V D I K H Q L K L Y R C S D Y   F6
     Y T T T H M Q L I K N K S T F W H Y W T W T I K F I I T S N N F G S F C R W Y R P N L T L T S T F S P * M L K T N S N * I D V H T T F  F5
     I H P P I C K C Y K T K V L L G I T G L G H S K S F S Q L I I S D V S V D G I D Q I * H S P V Q L L H N C * N Q T P T K S I S M L R L   F4

     I Y L H Y L F * D I Q R * E E E I S H M I I F A V Y F A H V L Y I F M H T T T Y C F A A V C R S T * F P F I F F T T V A H T C Y T Y   F1
     R F I C T T C S K T S R G K K R K S L T * S F L L C I L P M C Y T F L C I P P H I A L L L S V G P L S F H S F S S Q L L H I H V T H M   F2
     D L F A L L V L R H P E V R R G N L S H D H F C C V F C P C A I H F Y A Y H H I L L C C C L S V H L V S I H F L H N C C T Y M L H I *   F3
2201 AGATTTATTTGCACTACTTGTTCTAAGACATCCAGAGGTAAGAAGAGGAAATCTCTCACATGATCATTTTTGCTGTGTATTTTGCCCATGTGCTATACATTTTTATGCATACCACCACATATTGCTTTGCTGCTGTCTGTCGGTCCACTTAGTTTCCATTCATTTTCTTCACAACTGTTGCACATACATGTTACACATAT 2400
     ----:----|----:----|----:----|----:----|----:----|----:----|----:----|----:----|----:----|----:----|----:----|----:----|----:----|----:----|----:----|----:----|----:----|----:----|----:----|----:----|
2201 TCTAAATAAACGTGATGAACAAGATTCTGTAGGTCTCCATTCTTCTCCTTTAGAGAGTGTACTAGTAAAAACGACACATAAAACGGGTACACGATATGTAAAAATACGTATGGTGGTGTATAACGAAACGACGACAGACAGCCAGGTGAATCAAAGGTAAGTAAAAGAAGTGTTGACAACGTGTATGTACAATGTGTATA 2400
     F I * K C * K N * S M W L Y S S I E * M I M K A T Y K A W T S Y M K I C V V Y Q K A A T Q R D V * N G N M K K V V T A C V H * V Y   F6
     S K N A S S T R L C G S T L L P F R E C S * K Q Q T N Q G H A I C K * A Y W W M N S Q Q R D T V K T E M * K R * S T M Q Q V Y M N C M  F5
     L N I Q V V Q E L V D L P L F L F D R V H D N K S H I K G M H * V N K H M G G C I A K S S D T P G S L K W E N E E C S N C M C T V C I   F4
```

```
 E I * R T D L * E V Y L S Q F C F G Y D R T I F D R V M S L R L * K I P I I C S F R S F S L Q R L H L L R * N L V Y K F I R * I F W A  F1
  K F N V Q I Y E R Y I * V N S V L G T I A P F L T E L C P L D F E K F Q L F A V S V H F L C R G C T Y * D E I W Y T N L S D K Y F G Q  F2
   N L T Y R F M R G I S K S I L F W V R S H H F * Q S Y V P * T L K N S N Y L Q F P F I F F A E V A L T E M K F G I Q I Y Q I N I L G  F3
2401 GAAATTTAACGTACAGATTTATGAGAGGTATATCTAAGTCAATTCTGTTTTGGGTACGATCGCACCATTTTTGACAGAGTTATGTCCCTTAGACTTTGAAAAATTCCAATTATTTGCAGTTTCCGTTCATTTTCTTTGCAGAGGTTGCACTTACTGAGATGAAATTTGGTATACAAATTTATCAGATAAATATTTTGGGC 2600
     ----:----|----:----|----:----|----:----|----:----|----:----|----:----|----:----|----:----|----:----|----:----|----:----|----:----|----:----|----:----|----:----|----:----|----:----|----:----|----:----|
2401 CTTTAAATTGCATGTCTAAATACTCTCCATATAGATTCAGTTAAGACAAAACCCATGCTAGCGTGGTAAAAACTGTCTCAATACAGGGAATCTGAAACTTTTTAAGGTTAATAAACGTCAAAGGCAAGTAAAAGAAACGTCTCCAACGTGAATGACTCTACTTTAAACCATATGTTTAAATAGTCTATTTATAAAACCCG 2600
  S I * R V S K H S T Y R L * N Q K P Y S R V M K S L T I D R L S Q F I G I I Q L K R E N E K C L N C K S L H F K T Y L N I L Y I N Q A  F6
 H F K V Y L N I L P I D L D I R N Q T R D C W K Q C L * T G * V K F F E L * K C N G N M K K A S T A S V S I F N P I C I * * I F I K P  F5
   F N L T C I * S L Y I * T L E T K P V I A G N K V S N H G K S K S F N W N N A T E T * K R Q L P Q V * Q S S I Q Y V F K D S L Y K P  F4

  S S V L G T V M P F W I * K I P I W G E G A * V F Y K H L L L F I I H T F Y V L N Y V K Q L F * G L L L * I A P P V V F C L L L T E Q  F1
   V L F W V Q L C P F G F K K F Q F G G R G H K C F T N I S C Y L L S I L F M Y * T T * N N C F K G F Y C R * H L L L F F A C Y * L N  F2
 K F C F G Y S Y A L L D L K N S N L G G G G I S V L Q T S L V I Y Y P Y F L C I E L R E T I V L R A F T V D S T S C C F L L V I N * T  F3
2601 AAGTTCTGTTTTGGGTACAGTTATGCCCTTTTGGATTTAAAAAATTCCAATTTGGGGGGAGGGGGCATAAGTGTTTTACAAACATCTCTTGTTATTTATTATCCATACTTTTTATGTATTGAACTACGTGAAACAATTGTTTTAAGGGCTTTTACTGTAGATAGCACCTCCTGTTGTTTTTTGCTTGTTATTAACTGAAC 2800
     ----:----|----:----|----:----|----:----|----:----|----:----|----:----|----:----|----:----|----:----|----:----|----:----|----:----|----:----|----:----|----:----|----:----|----:----|----:----|----:----|
2601 TTCAAGACAAAACCCATGTCAATACGGGAAAACCTAAATTTTTTAAGGTTAAACCCCCCTCCCCCGTATTCACAAAATGTTTGTAGAGAACAATAAATAATAGGTATGAAAAATACATAACTTGATGCACTTTGTTAACAAAATTCCCGAAAATGACATCTATCGTGGAGGACAACAAAAAACGAACAATAATTGACTTG 2800
   L E T K P V T I G K Q I * F I G I Q P S P A Y T N * L C R K N N I I W V K * T N F * T F C N N * P S K S Y I A G G T T K Q K N N V S  F6
  L N Q K P Y L * A R K S K F F E L K P P P P M L T K C V D R T I * * G Y K K H I S S R S V I T K L A K V T S L V E Q Q K K S T I L Q V  F5
 C T R N Q T C N H G K P N L F N W N P P L P C L H K V F M E Q * K N D M S K I Y Q V V H F L Q K L P K * Q L Y C R R N N K A Q * * S F  F4

  R N K I I I V F Q C A Y I Y I Y S Y D H M N V * L W D R H I E L N A Y K K L S F * V M C H I D L Y T L I Y L H I Y N F I Y K L V M G  F1
 K E I R S L * C F N V H I F I S I R M I I * M Y N Y G T G T L N * M H T R S * V S E * C V I S I Y I H * S I Y I Y I I L Y T N L L W A  F2
  K K * D H Y S V S M C I Y L Y L F V * S Y E C I T M G Q A H * T K C I Q E A K F L S N V S Y R S I Y I D L F T Y I * F Y I Q T C Y G H  F3
2801 AAAGAAATAAGATCATTATAGTGTTTCAATGTGCATATATTTATATCTATTCGTATGATCATATGAATGTATAACTATGGGACAGGCACATTGAACTAAATGCATACAAGAAGCTAAGTTTCTGAGTAATGTGTCATATCGATCTATATACATTGATCTATTTACATATATATAATTTTATATACAAACTTGTTATGGGC 3000
     ----:----|----:----|----:----|----:----|----:----|----:----|----:----|----:----|----:----|----:----|----:----|----:----|----:----|----:----|----:----|----:----|----:----|----:----|----:----|----:----|
2801 TTTCTTTATTCTAGTAATATCACAAAGTTACACGTATATAAATATAGATAAGCATACTAGTATACTTACATATTGATACCCTGTCCGTGTAACTTGATTTACGTATGTTCTTCGATTCAAAGACTCATTACACAGTATAGCTAGATATATGTAACTAGATAAATGTATATATATTAAAATATATGTTTGAACAATACCCG 3000
 C L F L I M I T N * H A Y I * I * E Y S * I F T Y S H S L C M S S F A Y L F S L K Q T I H * I S R Y V N I * K C I Y L K I Y L S T I P  F6
  F F Y S * * L T E I H M Y K Y R N T H D Y S H I V I P C A C Q V L H M C S A L N R L L T D Y R D I Y M S R N V Y I Y N * I C V Q * P  F5
   L S I L D N Y H K L T C I N I D I R I I M H I Y L * P V P V N F * I C V L L * T E S Y H T M D I * I C Q D I * M Y I I K Y V F K N H A  F4

 I * T T P Y * Y I K F Y I I W F K Y M * H V H V K V * I * K I S K E I Y Y D H E Q S Y * M K * I A L H D H I V V K Y S F E D L Q A S I  F1
  F K L L L T N T * N F T * F G S N T C N M Y M * K Y E F E K F Q K K S I M I M N K V I K * N K L L C M I I L * * N I H L K I Y K L V F  F2
   L N Y S L L I H K I L H N L V Q I H V T C T C K S M N L K N F K R N L L * S * T K L L N E I N C S A * S Y C S E I F I * R F T S * Y  F3
3001 ATTTAAACTACTCCTTACTAATACATAAAATTTTACATAATTTGGTTCAAATACATGTAACATGTACATGTAAAAGTATGAATTTGAAAAATTTCAAAAGAAATCTATTATGATCATGAACAAAGTTATTAAATGAAATAAATTGCTCTGCATGATCATATTGTAGTGAAATATTCATTTGAAGATTTACAAGCTAGTAT 3200
     ----:----|----:----|----:----|----:----|----:----|----:----|----:----|----:----|----:----|----:----|----:----|----:----|----:----|----:----|----:----|----:----|----:----|----:----|----:----|----:----|
3001 TAAATTTGATGAGGAATGATTATGTATTTTAAAATGTATTAAACCAAGTTTATGTACATTGTACATGTACATTTTCATACTTAAACTTTTTAAAGTTTTCTTTAGATAATACTAGTACTTGTTTCAATAATTTACTTTATTTAACGAGACGTACTAGTATAACATCACTTTATAAGTAAACTTCTAAATGTTCGATCATA 3200
  M * V V G * * Y M F N * M I Q N L Y M Y C T C T F T H I Q F I E F S I * * S * S C L * * I F Y I A R C S * I T T F Y E N S S K C A L I  F6
 C K F * E K S I C L I K C L K T * I C T V H V H L L I F K F F K L L F R N H D H V F N N F S I F Q E A H D Y Q L S I N M Q L N V L * Y  F5
   N L S S R V L V Y F K V Y N P E F V H L M Y M Y F Y S N S F N * F F D I I I M F L T I L H F L N S Q M I M N Y H F I * K F I * L S T  F4

  * K W T S S L I M T T S K T Q R T A N F L V L N L S K L K K S L A A I K P M K F * N I T Q T A I G V S T S G K I F S K M S K K F V A C  F1
   R S G Q V L L L * P Q V K H K E Q R I F L C S T S A S L K R A L L L * S Q * S F K T S H K L P * V F P R R E R F F L K C L K S L L P  F2
 L E V D K F S Y Y D H K * N T K N S E F S C A Q P Q Q A * K E P C C Y K A N E V L K H H T N C H R C F H V G K D F F * N V * K V C C L  F3
3201 TTAGAAGTGGACAAGTTCTCTTATTATGACCACAAGTAAAACACAAAGAACAGCGAATTTTCTTGTGCTCAACCTCAGCAAGCTTAAAAAGAGCCTTGCTGCTATAAAGCCAATGAAGTTTTAAAACATCACACAAACTGCCATAGGTGTTTCCACGTCGGGAAAGATTTTTTCTAAAATGTCTAAAAAGTTTGTTGCCT 3400
     ----:----|----:----|----:----|----:----|----:----|----:----|----:----|----:----|----:----|----:----|----:----|----:----|----:----|----:----|----:----|----:----|----:----|----:----|----:----|----:----|
3201 AATCTTCACCTGTTCAAGAGAATAATACTGGTGTTCATTTTGTGTTTCTTGTCGCTTAAAAGAACACGAGTTGGAGTCGTTCGAATTTTTCTCGGAACGACGATATTTCGGTTACTTCAAAATTTTGTAGTGTGTTTGACGGTATCCACAAAGGTGCAGCCCTTTCTAAAAAAGATTTTACAGATTTTTCAAACAACGGA 3400
  * F H V L E R I I V V L L V C L V A F K R T S L R L L S L F L R A A I F G I F N * F M V C V A M P T E V D P F I K E L I D L F N T A  F6
 K S T S L N E * * S W L Y F V F F L S N E Q A * G * C A * F S G Q Q * L A L S T K F C * V F Q W L H K W T P F S K K * F T * F T Q Q R  F5
 N L L P C T R K N H G C T F C L S C R I K K H E V E A L K F L A K S S Y L W H L K L V D C L S G Y T N G R R S L N K R F H R F L K N G  F4

  L I A L T T K S P Y * P R P I W V F Y K L L N M F N N F M * I I G P L Y I I K I F K * K S P H F N S M F F V I F Y I L W T L F Q R I  F1
 A S L P S L L K A P I D P G P F G S S I S C * T C S I T L C K * L G H S T * S K F S S K R V P I S T A C F L * F F T S Y G H C F R G S  F2
  P H C P H Y * K P L L T Q A H L G L L * V A E H V Q * L Y V N N W A T L H N Q N F Q V K E S P F Q Q H V F C N F L H L M D I V S E D P  F3
3401 GCCTCATTGCCCTCACTACTAAAAGCCCCTATTGACCCAGGCCCATTTGGGTCTTCTATAAGTTGCTGAACATGTTCAATAACTTTATGTAAATAATTGGGCCACTCTACATAATCAAAATTTTCAAGTAAAAGAGTCCCCATTTCAACAGCATGTTTTTTGTAATTTTTTACATCTTATGGACATTGTTTCAGAGGATC 3600
     ----:----|----:----|----:----|----:----|----:----|----:----|----:----|----:----|----:----|----:----|----:----|----:----|----:----|----:----|----:----|----:----|----:----|----:----|----:----|----:----|
3401 CGGAGTAACGGGAGTGATGATTTTCGGGGATAACTGGGTCCGGGTAAACCCAGAAGATATTCAACGACTTGTACAAGTTATTGAAATACATTTATTAACCCGGTGAGATGTATTAGTTTTAAAAGTTCATTTTCTCAGGGGTAAAGTTGTCGTACAAAAAACATTAAAAAATGTAGAATACCTGTAACAAAGTCTCCTAG 3600
 Q R M A R V V L L G * Q G L G M Q T K * L N S F M N L L K I Y I I P G S * M I L I K L Y F L G W K L L M N K T I K * M K H V N N * L I  F6
  G * Q G * * * F G R N V W A W K P R R Y T A S C T * Y S * T F L Q A V R C L * F K * T F S D G N * C C T K Q L K K C R I S M T E S S  F5
   A E N G E S S F A G I S G P G N P D E I L Q Q V H E I V K H L Y N P W E V Y D F N E L L L T G M E V A H K K Y N K V D * P C Q K L P D  F4

 L T S I N L S K V Q K Y F Q N R R Q I F F S D R I * N K C Q Q N R M R R F K K G S G I I T R H H K L G I N S T V T F H M L I K * R L C  F1
  L H L * T F L R F K N I F K T E D R F S F L T E S E I S A N K T E * G V S K R V L A * L P G I I S W G L I P Q L H F I C * S N E D S A  F2
   Y I Y K P F * G S K I F S K Q K T D F L F * Q N L K * V P T K Q N E A F Q K G F W H N Y Q A S * A G D * F H S Y I S Y V D Q M K T L  F3
3601 CTTACATCTATAAACCTTTCTAAGGTTCAAAAATATTTTCAAAACAGAAGACAGATTTTCTTTTCTGACAGAATCTGAAATAAGTGCCAACAAAACAGAATGAGGCGTTTCAAAAAGGGTTCTGGCATAATTACCAGGCATCATAAGCTGGGGATTAATTCCACAGTTACATTTCATATGTTGATCAAATGAAGACTCTG 3800
     ----:----|----:----|----:----|----:----|----:----|----:----|----:----|----:----|----:----|----:----|----:----|----:----|----:----|----:----|----:----|----:----|----:----|----:----|----:----|----:----|
3601 GAATGTAGATATTTGGAAAGATTCCAAGTTTTTATAAAAGTTTTGTCTTCTGTCTAAAAGAAAAGACTGTCTTAGACTTTATTCACGGTTGTTTTGTCTTACTCCGCAAAGTTTTTCCCAAGACCGTATTAATGGTCCGTAGTATTCGACCCCTAATTAAGGTGTCAATGTAAAGTATACAACTAGTTTACTTCTGAGAC 3800
  R V D I F R E L T * F Y K * F L L C I K K E S L I Q F L H W C F L I L R K L F P E P M I V L C * L S P I L E V T V N * I N I L H L S Q  F6
 G * M * L G K * P E F I N E F C F V S K R K Q C F R F Y T G V F C F S A N * F P N Q C L * W A D Y A P S * N W L * M E Y T S * I F V R  F5
   K C R Y V K R L N L F I K L V S S L N E K R V S D S I L A L L V S H P T E F L T R A Y N G P M M L Q P N I G C N C K M H Q D F S S E  F4

  I L N * W F D I L C K F P F S N T S Y F T N N N F L K K L S * I N I C M C G I Y T F L N W F N F C K W S R F D T F * N T F Q F I F * Q  F1
   F * T N G L T S C V S S H L V T P A I S R T I T F L K N C P K L I S A C V A S I P F * I G S I F V S G A D L T P F E I L F N S F S D  F2
 H F E L M V * H L V * V P I * * H Q L F H E Q * L S * K T V L N * Y L H V W H L Y L S E L V Q F L * V E Q I * H L L K Y F S I H F L T  F3
3801 CATTTTGAACTAATGGTTTGACATCTTGTGTAAGTTCCCATTTAGTAACACCAGCTATTTCACGAACAATAACTTTCTTAAAAAACTGTCCTAAATTAATATCTGCATGTGTGGCATCTATACCTTTCTGAATTGGTTCAATTTTTGTAAGTGGAGCAGATTTGACACCTTTTGAAATACTTTTCAATTCATTTTCTGAC 4000
     ----:----|----:----|----:----|----:----|----:----|----:----|----:----|----:----|----:----|----:----|----:----|----:----|----:----|----:----|----:----|----:----|----:----|----:----|----:----|----:----|
3801 GTAAAACTTGATTACCAAACTGTAGAACACATTCAAGGGTAAATCATTGTGGTCGATAAAGTGCTTGTTATTGAAAGAATTTTTTGACAGGATTTAATTATAGACGTACACACCGTAGATATGGAAAGACTTAACCAAGTTAAAAACATTCACCTCGTCTAAACTGTGGAAAACTTTATGAAAAGTTAAGTAAAAGACTG 4000
   M K F * H N S M K H L N G N L L V L * K V F L L K R L F S D * I L I Q M H P M * V K R F Q N L K Q L H L L N S V K Q F V K * N M K Q  F6
  C K S S I T Q C R T Y T G M * Y C W S N * S C Y S E * F V T R F * Y R C T H C R Y R E S N T * N K Y T S C I Q C R K F Y K E I * K R V  F5
 A N Q V L P K V D Q T L E W K T V G A I E R V I V K K F F Q G L N I D A H T A D I G K Q I P E I K T L P A S K V G K S I S K L E N E S  F4

  R V W I D T * N F S N I * T F R N R P V N N K R S Q T F F C R L L I C I T * C T * I F * P T T L K T R V S P I S L F V N H R I K K *  F1
 K E F G L T R K I S A I F E H S E T D L L I T K D P R L S F A D C L S A S H N V H K Y F D P L P * R P E S P L S L F S S T I E L K N E  F2
  K S L D * H V K F Q Q Y L N I Q K Q T C * * Q K I P D F L L Q I A Y L H H I M Y I N I L T H Y P E D Q S L P Y L S F R Q P * N * K M K  F3
4001 AAAGAGTTTGGATTGACACGTAAAATTTCAGCAATATTTGAACATTCAGAAACAGACCTGTTAATAACAAAAGATCCCAGACTTTCTTTTGCAGATTGCTTATCTGCATCACATAATGTACATAAATATTTTGACCCACTACCCTGAAGACCAGAGTCTCCCCTATCTCTCTTTTCGTCAACCATAGAATTAAAAAATGA 4200
     ----:----|----:----|----:----|----:----|----:----|----:----|----:----|----:----|----:----|----:----|----:----|----:----|----:----|----:----|----:----|----:----|----:----|----:----|----:----|----:----|
4001 TTTCTCAAACCTAACTGTGCATTTTAAAGTCGTTATAAACTTGTAAGTCTTTGTCTGGACAATTATTGTTTTCTAGGGTCTGAAAGAAAACGTCTAACGAATAGACGTAGTGTATTACATGTATTTATAAAACTGGGTGATGGGACTTCTGGTCTCAGAGGGGATAGAGAGAAAAGCAGTTGGTATCTTAATTTTTTACT 4200
 C L T Q I S V Y F K L L I Q V N L F L G T L L L L D W V K K Q L N S I Q M V Y H V Y I N Q G V V R F V L T E G I E R K T L W L I L F H  F6
  F L K S Q C T F N * C Y K F M * F C V Q * Y C F I G S K R K C I A * R C * M I Y M F I K V W * G S S W L R G * R E K R * G Y F * F I  F5
   L S N P N V R L I E A I N S C E S V S R N I V F S G L S E K A S Q K D A D C L T C L Y K S G S G Q L G S D G R D R K E D V M S N F F S  F4

 K F M E H P T L F C L N M H A I L H Q N F P F I F N R * N T N N I R Y M V V F V * N R F * K W P V C S D T I W K R F I K N I I H F A L  F1
  N L W N I Q P C F V L T C M Q F C I K I S L S F S I G K I Q T T L D T W L F S S E I D S K S G L F V L T L F G K G S L K T S Y I L P *  F2
   I Y G T S N P V L S * H A C N F A S K F P F H F Q * V K Y K Q H * I H G C F R L K * I L K V A C L F * H Y L E K V H * K H H T F C L  F3
4201 AAATTTATGGAACATCCAACCCTGTTTTGTCTTAACATGCATGCAATTTTGCATCAAAATTTCCCTTTCATTTTCAATAGGTAAAATACAAACAACATTAGATACATGGTTGTTTTCGTCTGAAATAGATTCTAAAAGTGGCCTGTTTGTTCTGACACTATTTGGAAAAGGTTCATTAAAAACATCATACATTTTGCCTT 4400
     ----:----|----:----|----:----|----:----|----:----|----:----|----:----|----:----|----:----|----:----|----:----|----:----|----:----|----:----|----:----|----:----|----:----|----:----|----:----|----:----|
4201 TTTAAATACCTTGTAGGTTGGGACAAAACAGAATTGTACGTACGTTAAAACGTAGTTTTAAAGGGAAAGTAAAAGTTATCCATTTTATGTTTGTTGTAATCTATGTACCAACAAAAGCAGACTTTATCTAAGATTTTCACCGGACAAACAAGACTGTGATAAACCTTTTCCAAGTAATTTTTGTAGTATGTAAAACGGAA 4400
  F N I S C G V R N Q R L M C A I K C * F K G K M K L L Y F V F L M L Y M T T K T Q F L N * F H G T Q E S V I Q F L N M L F M M C K A K  F6
 F I * P V D L G T K D * C A H L K A D F N G K * K * Y T F Y L C C * I C P Q K R R F Y I R F T A Q K N Q C * K S F T * * F C * V N Q R  F5
   F K H F M W G Q K T K V H M C N Q M L I E R E N E I P L I C V V N S V H N N E D S I S E L L P R N T R V S N P F P E N F V D Y M K G  F4

  I F S L N F D N T E R K P K S F V G * H F E I F F S I Y R N F T H S V T P I F N S C M K K D I L N I * P M F F * T I C N F G L * F L Q  F1
   Y S A * I L I T Q K E N R K A L S G N I L K S F S L Y T E T S P I P S H P S L T V V * K R T F S I S N P C F S K L F A I S D S S S C  F2
 D I Q L E F * * H R K K T E K L C R V T F * N L F L Y I Q K L H P F R H T H L * Q L Y E K G H S Q Y L T H V F L N Y L Q F R T L V L A  F3
4401 GATATTCAGCTTGAATTTTGATAACACAGAAAGAAAACCGAAAAGCTTTGTCGGGTAACATTTTGAAATCTTTTTCTCTATATACAGAAACTTCACCCATTCCGTCACACCCATCTTTAACAGTTGTATGAAAAAGGACATTCTCAATATCTAACCCATGTTTTTCTAAACTATTTGCAATTTCGGACTCTAGTTCTTGC 4600
     ----:----|----:----|----:----|----:----|----:----|----:----|----:----|----:----|----:----|----:----|----:----|----:----|----:----|----:----|----:----|----:----|----:----|----:----|----:----|----:----|
4401 CTATAAGTCGAACTTAAAACTATTGTGTCTTTCTTTTGGCTTTTCGAAACAGCCCATTGTAAAACTTTAGAAAAAGAGATATATGTCTTTGAAGTGGGTAAGGCAGTGTGGGTAGAAATTGTCAACATACTTTTTCCTGTAAGAGTTATAGATTGGGTACAAAAAGATTTGATAAACGTTAAAGCCTGAGATCAAGAACG 4600
  I N L K F K S L V S L F G F L K T P Y C K S I K K E I Y L F K V W E T V G M K L L Q I F F S M R L I * G M N K * V I Q L K P S * N K  F6
  S I * S S N Q Y C L F F V S F S Q R T V N Q F R K R * I C F S * G N R * V W R * C N Y S F P C E * Y R V W T K R F * K C N R V R T R A  F5
 Q Y E A Q I K I V C F S F R F A K D P L M K F D K E R Y V S V E G M G D C G D K V T T H F L V N E I D L G H K E L S N A I E S E L E Q  F4

  G N R K S F K I R K P N S H T V W S C Q L H K T W H K T L I D I E R L C V R V F T V L K * Y I A L I I N N L R N S R R G H K Q I L A  F1
 K V I E R A S K * E N L T P I Q F G V A N S T K P G I K H * L T L R G S V * G F L Q F * N N T L P S L L T T * E I A A E G I N K F S Q  F2
 R * * K E L Q N K K T * L P Y S L E L P T P Q N L A * N I D * H * E A L C E G F Y S F E I I H C P H Y * Q L E K * P Q R A * T N S R N  F3
4601 AAGGTAATAGAAAGAGCTTCAAAATAAGAAAACCTAACTCCCATACAGTTTGGAGTTGCCAACTCCACAAAACCTGGCATAAAACATTGATTGACATTGAGAGGCTCTGTGTGAGGGTTTTTACAGTTTTGAAATAATACATTGCCCTCATTATTAACAACTTGAGAAATAGCCGCAGAGGGCATAAACAAATTCTCGCA 4800
     ----:----|----:----|----:----|----:----|----:----|----:----|----:----|----:----|----:----|----:----|----:----|----:----|----:----|----:----|----:----|----:----|----:----|----:----|----:----|----:----|
4601 TTCCATTATCTTTCTCGAAGTTTTATTCTTTTGGATTGAGGGTATGTCAAACCTCAACGGTTGAGGTGTTTTGGACCGTATTTTGTAACTAACTGTAACTCTCCGAGACACACTCCCAAAAATGTCAAAACTTTATTATGTAACGGGAGTAATAATTGTTGAACTCTTTATCGGCGTCTCCCGTATTTGTTTAAGAGCGT 4800
 C P L L F L K L I L F G L E W V T Q L Q W S W L V Q C L V N I S M S L S Q T L T K V T K F Y Y M A R M I L L K L F L R L P C L C I R A  F6
  L Y Y F S S * F L F V * S G Y L K S N G V G C F R A Y F M S Q C Q S A R H S P K * L K S I I C Q G * * * C S S F Y G C L A Y V F E R  F5
   L T I S L A E F Y S F R V G M C N P T A L E V F G P M F C Q N V N L P E T H P N K C N Q F L V N G E N N V V Q S I A A S P M F L N E C  F4

 T F H F T R G L E N M Y I V L * K K I V L G S I L S L T L Q K I N S N C * T L F W R K S V I I S * P Q S L N L L P T W I I Q F L Q V I  F1
  L S I L L G A W K I C T L F C E R K S Y W D L Y C P L L C K R S T L I A R H C S G E R V L L F P S H K V S I C F Q R G S F N S F K * S  F2
   F P F Y * G L G K Y V H C F V K E N R T G I Y I V P Y F A K D Q L * L L D T V L E K E C Y Y F L A T K S Q F A S N V D H S I P S S D  F3
4801 ACTTTCCATTTTACTAGGGGCTTGGAAAATATGTACATTGTTTTGTGAAAGAAAATCGTACTGGGATCTATATTGTCCCTTACTTTGCAAAAGATCAACTCTAATTGCTAGACACTGTTCTGGAGAAAGAGTGTTATTATTTCCTAGCCACAAAGTCTCAATTTGCTTCCAACGTGGATCATTCAATTCCTTCAAGTGAT 5000
     ----:----|----:----|----:----|----:----|----:----|----:----|----:----|----:----|----:----|----:----|----:----|----:----|----:----|----:----|----:----|----:----|----:----|----:----|----:----|----:----|
4801 TGAAAGGTAAAATGATCCCCGAACCTTTTATACATGTAACAAAACACTTTCTTTTAGCATGACCCTAGATATAACAGGGAATGAAACGTTTTCTAGTTGAGATTAACGATCTGTGACAAGACCTCTTTCTCACAATAATAAAGGATCGGTGTTTCAGAGTTAAACGAAGGTTGCACCTAGTAAGTTAAGGAAGTTCACTA 5000
  V K W K V L P K S F I Y M T K H F F I T S P D I N D R V K C F I L E L Q * V S N Q L F L T I I E * G C L R L K S G V H I M * N R * T I  F6
 L K G N * * P S P F Y T C Q K T F S F R V P I * I T G * K A F S * S * N S S V T R S F S H * * K R A V F D * N A E L T S * E I G E L S  F5
   S E M K S P A Q F I H V N N Q S L F D Y Q S R Y Q G K S Q L L D V R I A L C Q E P S L T N N N G L W L T E I Q K W R P D N L E K L H  F4
```

```
      F * H K K Q N I R L V L V L H F T K F I D C R Y K R F K T L M T G V L S R Y R V K R * F E H L R F G R D I I Q N F C G P L * T S F F M   F1
      F N I K N K T S D L F S S C I S Q N S L T V D I R G L R R L * R V C L A D T E * R G N L N T L G L V V T S Y K T F V A L F E Q V F S    F2
     L L T * K T K H Q T C S R L A F H K I H * L * I * E V * D A Y D G C A * Q I Q S E E V I * T P * V W S * H H T K L L W P S L N K F F H  F3
5001 CTTTTAACATAAAAAACAAAACATCAGACTTGTTCTCGTCTTGCATTTCACAAAATTCATTGACTGTAGATATAAGAGGTTTAAGACGCTTATGACGGGTGTGCTTAGCAGATACAGAGTGAAGAGGTAATTTGAACACCTTAGGTTTGGTCGTGACATCATACAAAACTTTTGTGGCCCTCTTTGAACAAGTTTTTCA 5200
     ----:----|----:----|----:----|----:----|----:----|----:----|----:----|----:----|----:----|----:----|----:----|----:----|----:----|----:----|----:----|----:----|----:----|----:----|----:----|----:----|
5001 GAAAATTGTATTTTTTGTTTTGTAGTCTGAACAAGAGCAGAACGTAAAGTGTTTTAAGTAACTGACATCTATATTCTCCAAATTCTGCGAATACTGCCCACACGAATCGTCTATGTCTCACTTCTCCATTAAACTTGTGGAATCCAAACCAGCACTGTAGTATGTTTTGAAAACACCGGGAGAAACTTGTTCAAAAAGT 5200
      K * L F C F M L S T R T K C K V F N M S Q L Y L L N L V S I V P T S L L Y L T F L Y N S C R L N P R S M M C F K Q P G R Q V L K K   F6
     R K V Y F V F C * V Q E R R A N * L I * Q S Y I Y S T * S A * S P H A * C I C L S S T I Q V G * T Q D H C * V F S K H G E K F L N K *  F5
     D K L M F F L V D S K N E D Q M E C F E N V T S I L P K L R K H R T H K A S V S H L P L K F V K P K T T V D Y L V K T A R K S C T K E  F4

      F I Y I S N * Q * I * A V C M N T M H * * C F I N L E K L T M E G G H L T L S S I K Q R S A Y R T C T * Q R K P T K N A F L T K N V I T   F1
     C S S I F L I N S E F E Q S A * I Q C T N N V S * I W R S * P W R G D T S L S S S R D L H T G H A L D K E N Q P K M H S L Q K M * L H  F2
      V H L Y F * L T V N L S S L H E Y N A L I M F H K F G E V D H G G G T P H S H Q A E I C I Q D M H L T K K T N Q K C I P Y K K C D Y I  F3
5201 TGTTCATCTATATTTCTAATTAACAGTGAATTTGAGCAGTCTGCATGAATACAATGCACTAATAATGTTTCATAAATTTGGAGAAGTTGACCATGGAGGGGGGACACCTCACTCTCATCAAGCAGAGATCTGCATACAGGACATGCACTTGACAAAGAAAACCAACCAAAAATGCATTCCTTACAAAAAATGTGATTACA 5400
     ----:----|----:----|----:----|----:----|----:----|----:----|----:----|----:----|----:----|----:----|----:----|----:----|----:----|----:----|----:----|----:----|----:----|----:----|----:----|----:----|
5201 ACAAGTAGATATAAAGATTAATTGTCACTTAAACTCGTCAGACGTACTTATGTTACGTGATTATTACAAAGTATTTAAACCTCTTCAACTGGTACCTCCCCCCTGTGGAGTGAGAGTAGTTCGTCTCTAGACGTATGTCCTGTACGTGAACTGTTTCTTTTGGTTGGTTTTTACGTAAGGAATGTTTTTTACACTAATGT 5400
     M N M * I E L * C H I Q A T Q M F V I C * Y H K M F K S F N V M S P P C R V R M L C L D A Y L V H V Q C L F G V L F A N R V F F T I V  F6
       T * R Y K * N V T F K L L R C S Y L A S I I N * L N P S T S W P P P V G * E * * A S I Q M C S M C K V F F V L W F H M G * L F H S *  F5
      H E D I N R I L L S N S C D A H I C H V L L T E Y I Q L L Q G H L P S V E S E D L L S R C V P C A S S L S F W G F I C E K C F I H N C  F4

     F R N V G V N R D A S T N C T H K R L C N F C I A E A L H R N T S N N N I L E H F R C R R R G S I S N * Q F I Y M G K R G T I Y R G T  F1
      L E M * G S T G T P L Q T V H I K D C A T S A L Q K R S T G I L L I I T F L N I S D A D A E E V S A I D N L S T W G K G E L S T G G Q  F2
      * K C R G Q Q G R L Y K L Y T * K V Q L L H C R S A P Q E Y F * * * H S * T F Q M Q T Q R K Y Q Q L T I Y L H G E K G N Y L Q G D  F3
5401 TTTAGAAATGTAGGGGTCAACAGGGACGCCTCTACAAACTGTACACATAAAAGATTGTGCAACTTCTGCATTGCAGAAGCGCTCCACAGGAATACTTCTAATAATAACATTCTTGAACATTTCAGATGCAGACGCAGAGGAAGTATCAGCAATTGACAATTTATCTACATGGGGAAAAGGGGAACTATCTACAGGGGGAC 5600
     ----:----|----:----|----:----|----:----|----:----|----:----|----:----|----:----|----:----|----:----|----:----|----:----|----:----|----:----|----:----|----:----|----:----|----:----|----:----|----:----|
5401 AAATCTTTACATCCCCAGTTGTCCCTGCGGAGATGTTTGACATGTGTATTTTCTAACACGTTGAAGACGTAACGTCTTCGCGAGGTGTCCTTATGAAGATTATTATTGTAAGAACTTGTAAAGTCTACGTCTGCGTCTCCTTCATAGTCGTTAACTGTTAAATAGATGTACCCCTTTTCCCCTTGATAGATGTCCCCCTG 5600
      N L F T P T L L S A E V F Q V C L L N H L K Q M A S A S W L F V E L L M R S C K L H L R L P L I L L Q C N I * M P F L P V I * L P V  F6
     M * F H L P * C P R R * L S Y V Y F I T C S R C Q L L A G C S Y K * Y Y C E Q V N * I C V C L F Y * C N V I * R C P S F P F * R C P S  F5
       K S I Y P D V P V G R C V T C M F S Q A V E A N C F R E V P I S R I I V N K F M E S A S A S S T D A I S L K D V H P F P S S D V P P   F4

      N G A * * * T R W M I Y I N R G I I H K G T K * G * R * R T V R G C G V C R L S R R S * L V F V R A V I R V F L S D E T V L F G L R W  F1
      M E H D D E P D G * S T L T E E * F T R G Q N E D E D E G Q S E D V V S V V S V E G L D L S L S A L * * E C S C L M K L F F L D C D   F2
     K W S M M M N P M D D L H * Q R N N S Q G D K M R M K M K D S Q R M W C L S S Q S K V L T C L C P R C D K S V L V * * N C S F W T A M  F3
5601 AAATGGAGCATGATGATGAACCCGATGGATGATCTACATTAACAGAGGAATAATTCACAAGGGGACAAAATGAGGATGAAGATGAAGGACAGTCAGAGGATGTGGTGTCTGTCGTCTCAGTCGAAGGTCTTGACTTGTCTTTGTCCGCGCTGTGATAAGAGTGTTCTTGTCTGATGAAACTGTTCTTTTTGGACTGCGAT 5800
     ----:----|----:----|----:----|----:----|----:----|----:----|----:----|----:----|----:----|----:----|----:----|----:----|----:----|----:----|----:----|----:----|----:----|----:----|----:----|----:----|
5601 TTTACCTCGTACTACTACTTGGGCTACCTACTAGATGTAATTGTCTCCTTATTAAGTGTTCCCCTGTTTTACTCCTACTTCTACTTCCTGTCAGTCTCCTACACCACAGACAGCAGAGTCAGCTTCCAGAACTGAACAGAAACAGGCGCGACACTATTCTCACAAGAACAGACTACTTTGACAAGAAAAACCTGACGCTA 5800
       F P A H H H V R H I I * M L L P I I * L P V F H P H L H L V T L P H P T Q R R L R L D Q S T K T R A T I L T N K D S S V T R K P S R  F6
      L H L M I I F G I S S R C * C L F L E C P S L I L I F I F S L * L I H H R D D * D F T K V Q R Q G R Q S L L T R T Q H F Q E K Q V A I  F5
      C I S C S S G S P H D V N V S S Y N V L P C F S S S S S P C D S S T T D T T E T S P R S K D K D A S H Y S H E Q R I F S N K K S Q S  F4

       D V K L S L * Y F L G S S R F Y K T N V T T V * M R Q K F I Q N R Q I N N N V I F S L A V S R Q S * S T C S A N S C R V N V V R V T   F1
     G T L S F L F N I F L G L P G F T K Q M S Q L S E * G K N S Y K T G K L T I M S S S A W L C L V S L S L H A V Q I L V G * M L S E S L  F2
      G R * A F S L I F S W V F Q V L Q N K C H N C L N E A K I H T K Q A N * Q * C H L Q L G C V S S V L V Y M Q C K F L * G E C C Q S H C  F3
5801 GGGACGTTAAGCTTTCTCTTTAATATTTTCTTGGGTCTTCCAGGTTTTACAAAACAAATGTCACAACTGTCTGAATGAGGCAAAAATTCATACAAAACAGGCAAATTAACAATAATGTCATCTTCAGCTTGGCTGTGTCTCGTCAGTCTTAGTCTACATGCAGTGCAAATTCTTGTAGGGTGAATGTTGTCAGAGTCACT 6000
     ----:----|----:----|----:----|----:----|----:----|----:----|----:----|----:----|----:----|----:----|----:----|----:----|----:----|----:----|----:----|----:----|----:----|----:----|----:----|----:----|
5801 CCCTGCAATTCGAAAGAGAAATTATAAAAGAACCCAGAAGGTCCAAAATGTTTTGTTTACAGTGTTGACAGACTTACTCCGTTTTTAAGTATGTTTTGTCCGTTTAATTGTTATTACAGTAGAAGTCGAACCGACACAGAGCAGTCAGAATCAGATGTACGTCACGTTTAAGAACATCCCACTTACAACAGTCTCAGTGA 6000
     H S T L S E R * Y K R P D E L N * L V F T V V T Q I L C F N M C F L C I L L L T M K L K A T D R * D * D V H L A F E Q L T F T T L T V  F6
       P R * A K E K I N E Q T K W T K C F L H * L Q R F S A F I * V F C A F * C Y H * R * S P Q T E D T K T * M C H L N K Y P S H Q * L *  F5
      P V N L K R K L I K K P R G P K V F C I D C S D S H P L F E Y L V P L N V I I D D E A Q S H R T L R L R C A T C I R T P H I N D S D S  F4

     V N V N V V E G G N S R L E V F F G M G F R I H R D F Y T T K S A H T L * L * F M L H F C R R N E I K F Y I N F L I F * I I Y Y T M *  F1
      S M S M L * R E V I A G L K F S L G W D L E F T G I F T P Q S L H I H F N F N S C S I S V E E M K * N F I * I F S F F K S S I T Q C S  F2
       Q C Q C C R G R * * Q A * S F L W D G I * N S Q G F L H H K V C T Y T L T L I H A P F L * K K * N K I L Y K F S H F L N H L L H N V  F3
6001 GTCAATGTCAATGTTGTAGAGGGAGGTAATAGCAGGCTTGAAGTTTTCTTTGGGATGGGATTTAGAATTCACAGGGATTTTTACACCACAAAGTCTGCACATACACTTTAACTTTAATTCATGCTCCATTTCTGTAGAAGAAATGAAATAAAATTTTATATAAATTTTCTCATTTTTTAAATCATCTATTACACAATGTA 6200
     ----:----|----:----|----:----|----:----|----:----|----:----|----:----|----:----|----:----|----:----|----:----|----:----|----:----|----:----|----:----|----:----|----:----|----:----|----:----|----:----|
6001 CAGTTACAGTTACAACATCTCCCTCCATTATCGTCCGAACTTCAAAAGAAACCCTACCCTAAATCTTAAGTGTCCCTAAAAATGTGGTGTTTCAGACGTGTATGTGAAATTGAAATTAAGTACGAGGTAAAGACATCTTCTTTACTTTATTTTAAAATATATTTAAAAGAGTAAAAAATTTAGTAGATAATGTGTTACAT 6200
      T L T L T T S P P L L S S T K K P I P N L I * L S K * V V F D A C V S * S * N M S W K Q L L F S I F N * I F K R M K * I M * * V I Y  F6
     Q * H * H Q L P L Y Y C A Q L K R Q S P I * F E C P N K C W L T Q V Y V K V K I * A G N R Y F F H F L I K Y L N E * K K F * R N C L T  F5
       D I D I N Y L S T I A P K F N E K P H S K S N V P I K V G C L R C M C K L K L E H E M E T S S I F Y F K I Y I K E N K L D D I V C H  F4

      Q M S M F I C N S L I Y C T F H * K G T T I * F * G E A R Q N F E * I N * P F * M L K I V * L * L A * K * S H F F F A C Q F Y F S I D  F1
      K * V V C L Y A T L * Y T V L S I K R G Q P F N S E G K P D R I L N K S T S L F K C * K L S N C S W H E N N H I F F L L V N F I F Q L  F2
     A N E Y V Y M Q L S N I L Y F P L K G D N H L I L R G S P T E F * I N Q L A F L N V E N C L T V V G M K I I T F F F C L S I L F F N *  F3
6201 GCAAATGAGTATGTTTATATGCAACTCTCTAATATACTGTACTTTCCATTAAAAGGGGACAACCATTTAATTCTGAGGGGAAGCCCGACAGAATTTTGAATAAATCAACTAGCCTTTTTAAATGTTGAAAATTGTCTAACTGTAGTTGGCATGAAAATAATCACATTTTTTTTTTGCTTGTCAATTTTATTTTTCAATTG 6400
     ----:----|----:----|----:----|----:----|----:----|----:----|----:----|----:----|----:----|----:----|----:----|----:----|----:----|----:----|----:----|----:----|----:----|----:----|----:----|----:----|
6201 CGTTTACTCATACAAATATACGTTGAGAGATTATATGACATGAAAGGTAATTTTCCCCTGTTGGTAAATTAAGACTCCCCTTCGGGCTGTCTTAAAACTTATTTAGTTGATCGGAAAAATTTACAACTTTTAACAGATTGACATCAACCGTACTTTTATTAGTGTAAAAAAAAAACGAACAGTTAAAATAAAAAGTTAAC 6400
       C I L N I H L E R I Y Q V K W * F P V V M * N Q P S A R C F K S Y I L * G K * I N F I T * S Y N A H F Y D C K K K A Q * N * K E I   F6
      A F S Y T * I C S E L I S Y K G N F P S L W K I R L P L G V S N Q I F * S A K K F T S F Q R V T T P M F I I V N K K Q K D I K N K L Q  F5
     L L H T H K Y A V R * Y V T S E M L L P C G N L E S P F G S L I K F L D V L R K L H Q F N D L Q L Q C S F L * M K K K S T L K I K * N  F4

       E K N I P K I L P A P S I L S G C L L I * S K N F F * L L H M N K L L F A T F Y V H L C A Q R S T E N S L S F S K * L D D L Y E L N   F1
     M K K I S Q K Y C Q P P V Y * V V V S * Y N Q K I F F N C C I * T N Y Y L Q H F M Y I Y V L R E V Q K T H Y L F P N N L M T F M N * T  F2
      * K K Y P K N I A S P Q Y I E W L S P N I I K K F F L T V A Y E Q T I I C N I L C T F M C S E K Y R K L I I F F Q I T * * P L * T E Q  F3
6401 ATGAAAAAAATATCCCAAAAATATTGCCAGCCCCCAGTATATTGAGTGGTTGTCTCCTAATATAATCAAAAAATTTTTTTAACTGTTGCATATGAACAAACTATTATTTGCAACATTTTATGTACATTTATGTGCTCAGAGAAGTACAGAAAACTCATTATCTTTTTCCAAATAACTTGATGACCTTTATGAACTGAAC 6600
     ----:----|----:----|----:----|----:----|----:----|----:----|----:----|----:----|----:----|----:----|----:----|----:----|----:----|----:----|----:----|----:----|----:----|----:----|----:----|----:----|
6401 TACTTTTTTATAGGGTTTTTATAACGGTCGGGGGTCATATAACTCACCAACAGAGGATTATATTAGTTTTTTAAAAAAAAATTGACAACGTATACTTGTTTGATAATAAACGTTGTAAAATACATGTAAATACACGAGTCTCTTCATGTCTTTTGAGTAATAGAAAAAGGTTTATTGAACTACTGGAAATACTTGACTTG 6600
      S S F F I G F I N G A G L I N L P Q R R I Y D F F K K * S N C I F L S N N A V N * T C K H A * L L V S F E N D K E L Y S S S R * S S F  F6
       H F F Y G L F I A L G W Y I S H N D G L I I L F N K K V T A Y S C V I I Q L M K H V N I H E S F Y L F S M I K K W I V Q H G K H V S  F5
       I F F I D W F Y Q W G G T Y Q T T E * Y L * F I K K L Q Q M H V F * * K C C K I Y M * T S L S T C F V * * R K G F L K I V K I F Q V  F4

     S L I K I K S S L A P F Y F D C R M A I K I K R S * * R F D F N Q T V N * K S N H Q D V W R K I K T I Q I L L K K L L W V Y I R K P K  F1
      V * L K S N L R * L L F I L I V A W Q S K * R G A S E G L I L I R L * T E N P I I K M Y G E R * R L F K Y F L K N Y Y G S I L E S Q N  F2
       S D * N Q I F A S S F L F * L S H G N Q N K E E L V K V * F * S D C E L K I Q S S R C M E K D K D Y S N T S * K I T M G L Y * K A K  F3
6601 AGTCTGATTAAAATCAAATCTTCGCTAGCTCCTTTTTATTTTGATTGTCGCATGGCAATCAAAATAAAGAGGAGCTAGTGAAGGTTTGATTTTAATCAGACTGTGAACTGAAAATCCAATCATCAAGATGTATGGAGAAAGATAAAGACTATTCAAATACTTCTTAAAAAATTACTATGGGTCTATATTAGAAAGCCAAA 6800
     ----:----|----:----|----:----|----:----|----:----|----:----|----:----|----:----|----:----|----:----|----:----|----:----|----:----|----:----|----:----|----:----|----:----|----:----|----:----|----:----|
6601 TCAGACTAATTTTAGTTTAGAAGCGATCGAGGAAAAATAAAACTAACAGCGTACCGTTAGTTTTATTTCTCCTCGATCACTTCCAAACTAAAATTAGTCTGACACTTGACTTTTAGGTTAGTAGTTCTACATACCTCTTTCTATTTCTGATAAGTTTATGAAGAATTTTTTAATGATACCCAGATATAATCTTTCGGTTT 6800
       L R I L I L D E S A G K * K S Q R M A I L I F L L * H L N S K L * V T F Q F D L * * S T H L F I F V I * I S R L F N S H T * I L F G F  F6
      C D S * F * I K A L E K K N Q N D C P L * F L S S S T F T Q N * D S Q S S F I W D D L H I S F S L S * E F V E * F I V I P R Y * F A L  F5
       T Q N F D F R R * S R K I K I T A H C D F Y L P A L S P K I K I L S H S V S F G I M L I Y P S L Y L S N L Y K K F F * * P D I N S L W  F4

       F I Y K C * A R L Q I C D K I K * K K N F F F S I * F I E N K N L * N H W Q M V N I C P T C F * L S R N L W K I L S R I F S H L Y L *  F1
       L F T N V E Q G Y R S V T K L S E K K I F F S L Y N S L K T K I C K I T G K W S I F V Q L V F D Y Q E I C G K F C Q E F F H T C T C   F2
     I Y L Q M L S K A T D L * Q N * V K K K F F L Y I I H * K Q K F V K S L A N G Q Y L S N L F L I I K K F V E N S V K N F F T P V P V  F3
6801 ATTTATTTACAAATGTTGAGCAAGGCTACAGATCTGTGACAAAATTAAGTGAAAAAAAAATTTTTTTTCTCTATATAATTCATTGAAAACAAAAATTTGTAAAATCACTGGCAAATGGTCAATATTTGTCCAACTTGTTTTTGATTATCAAGAAATTTGTGGAAAATTCTGTCAAGAATTTTTTCACACCTGTACCTGT 7000
     ----:----|----:----|----:----|----:----|----:----|----:----|----:----|----:----|----:----|----:----|----:----|----:----|----:----|----:----|----:----|----:----|----:----|----:----|----:----|----:----|
6801 TAAATAAATGTTTACAACTCGTTCCGATGTCTAGACACTGTTTTAATTCACTTTTTTTTTAAAAAAAAGAGATATATTAAGTAACTTTTGTTTTTAAACATTTTAGTGACCGTTTACCAGTTATAAACAGGTTGAACAAAAACTAATAGTTCTTTAAACACCTTTTAAGACAGTTCTTAAAAAAGTGTGGACATGGACA 7000
       N I * L H Q A L S C I Q S L I L H F F F K K K E I Y N M S F L F K Y F * Q C I T L I Q G V Q K Q N D L F K H F I R D L I K E C R Y R  F6
      I * K C I N L L A V S R H C F * T F F F N K K R * I * Q F C F N T F D S A F P * Y K D L K N K I I L F N T S F E T L F K K V G T G T  F5
     F K N V F T S C P * L D T V F N L S F F I K K E R Y L E N F V F I Q L I V P L H D I N T W S T K S * * S I Q P F N Q * S N K * V Q V Q  F4

       F Y K M F S M I Y I * K K N Y L Q Y F G V H L * V I N K L I Y M I L C K F A C K M H I * K C S E * L Q I T T * R V C N C M Q L Q L L   F1
     D F T K C F Q * Y I F R K K I I F N I L A C I C K * S T N * F I * Y F V S L H V K C I S K N A Q N D F K L Q L R G Y A T A C S C S F Y  F2
      I L Q N V F N D I Y L E K K L S S I F W R A S V S D Q Q I N L Y D T L * V C M * N A Y L K M L R M T S N Y N L E G M Q L H A A A A F T  F3
7001 GATTTTACAAAATGTTTTCAATGATATATATTTAGAAAAAAAATTATCTTCAATATTTTGGCGTGCATCTGTAAGTGATCAACAAATTAATTTATATGATACTTTGTAAGTTTGCATGTAAAATGCATATCTAAAAATGCTCAGAATGACTTCAAATTACAACTTAGAGGGTATGCAACTGCATGCAGCTGCAGCTTTA 7200
     ----:----|----:----|----:----|----:----|----:----|----:----|----:----|----:----|----:----|----:----|----:----|----:----|----:----|----:----|----:----|----:----|----:----|----:----|----:----|----:----|
7001 CTAAAATGTTTTACAAAAGTTACTATATATAAATCTTTTTTTTAATAGAAGTTATAAAACCGCACGTAGACATTCACTAGTTGTTTAATTAAATATACTATGAAACATTCAAACGTACATTTTACGTATAGATTTTTACGAGTCTTACTGAAGTTTAATGTTGAATCTCCCATACGTTGACGTACGTCGACGTCGAAAT 7200
      H N * L I N E I I Y I * F F F * R * Y K P T C R Y T I L L N I * I I S Q L N A H L I C I * F H E S H S * I V V * L T H L Q M C S C S K  F6
       I K C F T K L S I Y K S F F N D E I N Q R A D T L S * C I L K Y S V K Y T Q M Y F A Y R F I S L I V E F * L K S P I C S C A A A K  F5
      S K V F H K * H Y I N L F F I I K L I K A H M Q L H D V F * N I H Y K T L K C T F H M D L F A * F S K L N C S L P Y A V A H L Q L K *  F4

     L L Y I Y I * * C I Y Y L K G Q S D Y S R N I C F F Y K A Q L S C M F H I S G S * L Y K E E K Y L E K S P C T * C T D D I F K K * * G  F1
      C Y I Y I Y N N V Y I T * K D K A T I L E I F V F F I K H N F P V C F I L V E V D F I K K K N I L R K A H V L N V Q M I F L K N D E V  F2
      A I Y I Y I I M L Y I L L K R T K L F K S K Y L F F L * S T T F L Y V S Y * W K L T L * R R K I S * E K P M Y L M Y R * Y F * K M M R  F3
7201 CTGCTATATATATATATAATAATGTATATATTACTTAAAAGGACAAAGCGACTATTCTCGAAATATTTGTTTTTTTATAAAGCACAACTTTCCTGTATGTTTCATATTAGTGGAGTTGACTTTATAAAGAAGAAAAATATCTTGAGAAAAGCCCATGTACTTAATGTACAGATGATATTTTTAAAAAATGATGAGG 7400
     ----:----|----:----|----:----|----:----|----:----|----:----|----:----|----:----|----:----|----:----|----:----|----:----|----:----|----:----|----:----|----:----|----:----|----:----|----:----|----:----|
7201 GACGATATATATATATATATTATTACATATATAATGAATTTTCCTGTTTCGCTGATAAGAGCTTTATAAACAAAAAAATATTTCGTGTTGAAAGGACATACAAAGTATAATCACCTCAACTGAAATATTTCTTCTTTTTATAGAACTCTTTTCGGGTACATGAATTACATGTCTACTATAAAAATTTTTTACTACTCC 7400
       S S Y I Y I Y Y H I Y * K F P C L S * E R F I Q K K * L A C S E Q I N * I L P L Q S * L S S F Y R S F L G H V * H V S S I K L F H H P  F6
      V A I Y I Y I I I Y I N S L L V F R S N E F Y K N K K Y L V V K R Y T E Y * H F N V K Y L L F I D Q S F G M Y K I Y L H Y K * F I I L  F5
       Q * I Y I Y L L T Y I V * F S L A V I R S I N T K K I F C L K G T H K M N T S T S K I F F F F I K L F A W T S L T C I I N K F F S S  F4

      V R W C E D F S T G Q Q * S R * N T I S V Y E L L R Q H S T V Y Y * V I K Q Y S L M N F Y S H K V S E H F I * C K M L S F P S F C N N  F1
      * G G V K I F Q Q V N N K V D E I L S Q Y M N Y * D N T Q Q Y I I K * L N N I L * * I F I H T R Y Q N T L Y D V K C C P F L H S A I   F2
     C K V V * R F F N R S T I K * M K Y Y L S I * I I E T T L N S I L L S D * T I F F N E F L F T Q G I R T L Y M M * N V V L S F I L Q *  F3
7401 TGTAAGGTGGTGTGAAGATTTTTCAACAGGTCAACAATAAAGTAGATGAAATACTATCTCAGTATATGAATTATTGAGACAACACTCAACAGTATATTATTAAGTGATTAAACAATATTCTTTAATGAATTTTTATTCACACAAGGTATCAGAACACTTTATATGATGTAAAATGTTGTCCTTTCCTTCATTCTGCAATA 7600
     ----:----|----:----|----:----|----:----|----:----|----:----|----:----|----:----|----:----|----:----|----:----|----:----|----:----|----:----|----:----|----:----|----:----|----:----|----:----|----:----|
7401 ACATTCCACCACACTTCTAAAAAGTTGTCCAGTTGTTATTTCATCTACTTTATGATAGAGTCATATACTTAATAACTCTGTTGTGAGTTGTCATATAATAATTCACTAATTTGTTATAAGAAATTACTTAAAAATAAGTGTGTTCCATAGTCTTGTGAAATATACTACATTTTACAACAGGAAAGGAAGTAAGACGTTAT 7600
       T L H H S S K E V P * C Y L L H F V I E T Y S N N L C C E V T Y * * T I L C Y E K I F K * E C L T D S C K I H H L I N D K G E N Q L  F6
      H L T T H L N K L L D V I F Y I F Y * R L I H I I S V V S L L I N N L S * V I N K L S N K N V C P I L V S * I I Y F T T R E K M R C Y  F5
      T Y P P T F I K * C T L L L T S S I S D * Y I F * Q S L V * C Y I I L H N F L I R * H I K I * V L Y * F V K Y S T F H Q G K R * E A I  F4
```

```
      G  I  G  I  S  I  F  K  D  S  V  C  *  F  I  C  Y  Q  W  P  F  S  L  Y  L  Y  Q  D  F  *  V  L  M  N  V  *  P  C  L  S  V  V  Y  P  N  K  Q  K  F  S  *  I  Y  V  S  C  M  T  L  V  K  L  S  L  F  T     F1
     M  V  *  V  F  L  F  L  K  T  Q  F  A  N  S  F  A  I  S  G  H  F  H  F  I  C  T  K  T  F  E  F  *  *  M  C  N  H  V  Y  R  *  C  T  Q  I  N  K  N  F  H  E  S  T  C  P  V  *  P  W  *  N  Y  L  F  S  H     F2
      W  Y  R  Y  F  Y  F  *  R  L  S  L  L  I  H  L  L  S  V  A  I  F  T  L  F  V  P  R  L  L  S  F  N  E  C  V  T  M  F  I  G  S  V  P  K  *  T  K  I  F  M  N  L  R  V  L  Y  D  L  G  K  I  I  S  F  H  T   F3
7601 ATGGTATAGGTATTTCTATTTTTAAAGACTCAGTTTGCTAATTCATTTGCTATCAGTGGCCATTTTCACTTTATTTGTACCAAGACTTTTGAGTTTTAATGAATGTGTAACCATGTTTATCGGTAGTGTACCCAAATAAACAAAAATTTTCATGAATCTACGTGTCCTGTATGACCTTGGTAAAATTATCTCTTTTCACA 7800
     ----:----|----:----|----:----|----:----|----:----|----:----|----:----|----:----|----:----|----:----|----:----|----:----|----:----|----:----|----:----|----:----|----:----|----:----|----:----|----:----|
7601 TACCATATCCATAAAGATAAAAATTTCTGAGTCAAACGATTAAGTAAACGATAGTCACCGGTAAAAGTGAAATAAACATGGTTCTGAAAACTCAAAATTACTTACACATTGGTACAAATAGCCATCACATGGGTTTATTTGTTTTTAAAAGTACTTAGATGCACAGGACATACTGGAACCATTTTAATAGAGAAAAGTGT 7800
     L  P  I  P  I  E  I  K  L  S  E  T  Q  *  N  M  Q  *  *  H  G  N  E  S  *  K  Y  W  S  K  Q  T  K  I  F  T  Y  G  H  K  D  T  T  Y  G  F  L  C  F  N  E  H  I  *  T  D  Q  I  V  K  T  F  N  D  R  K  V    F6
      H  Y  L  Y  K  *  K  *  L  S  L  K  S  I  *  K  S  D  T  A  M  K  V  K  N  T  G  L  S  K  L  K  L  S  H  T  V  M  N  I  P  L  T  G  L  Y  V  F  I  K  M  F  R  R  T  R  Y  S  R  P  L  I  I  E  K  *     F5
      I  T  Y  T  N  R  N  K  F  V  *  N  A  L  E  N  A  I  L  P  W  K  *  K  I  Q  V  L  V  K  S  N  *  H  I  H  L  W  T  *  R  Y  H  V  W  I  F  L  F  K  *  S  D  V  H  G  T  H  G  Q  Y  F  *  R  K  E  C    F4


     Q  K  F  H  K  A  V  *  N  M  *  I  F  I  R  *  S  I  F  L  S  Q  K  K  H  R  K  S  T  I  S  V  L  N  Y  R  F  K  L  F  F  R  T  I  Q  T  Q  V  I  P  S  G  S  L  L  C  V  L  S  V  F  S  S  C  A  I  I    F1
      K  N  F  I  K  Q  Y  K  I  C  K  Y  L  L  G  R  A  Y  F  Y  H  K  K  S  T  G  S  Q  Q  Y  Q  F  *  I  T  V  S  N  F  S  F  G  R  S  K  L  K  S  Y  H  R  V  R  Y  C  V  Y  F  L  F  L  A  H  A  Q  *  Y   F2
      K  I  S  *  S  S  I  K  Y  V  N  I  Y  *  V  E  H  I  F  I  T  K  K  A  P  E  V  N  N  I  S  F  K  L  P  F  Q  T  F  L  S  D  D  P  N  S  S  H  T  I  G  F  V  I  V  C  T  F  C  F  *  L  M  R  N  N     F3
7801 CAAAAATTTCATAAAGCAGTATAAAATATGTAAATATTTATTAGGTAGAGCATATTTTTATCACAAAAAAAGCACCGGAAGTCAACAATATCAGTTTTAAATTACCGTTTCAAACTTTTCTTTCGGACGATCCAAACTCAAGTCATACCATCGGGTTCGTTATTGTGTGTACTTTCTGTTTTTAGCTCATGCGCAATAAT 8000
     ----:----|----:----|----:----|----:----|----:----|----:----|----:----|----:----|----:----|----:----|----:----|----:----|----:----|----:----|----:----|----:----|----:----|----:----|----:----|----:----|
7801 GTTTTTAAAGTATTTCGTCATATTTTATACATTTATAAATAATCCATCTCGTATAAAAATAGTGTTTTTTTCGTGGCCTTCAGTTGTTATAGTCAAAATTTAATGGCAAAGTTTGAAAAGAAAGCCTGCTAGGTTTGAGTTCAGTATGGTAGCCCAAGCAATAACACACATGAAAGACAAAAATCGAGTACGCGTTATTA 8000
      C  F  N  *  L  A  T  Y  F  I  Y  I  N  I  L  Y  L  M  N  K  D  C  F  F  C  R  F  D  V  I  D  T  K  F  *  R  K  L  S  K  K  R  V  I  W  V  *  T  M  G  D  P  E  N  N  H  T  S  E  T  K  L  E  H  A  I  I   F6
     V  F  I  E  Y  L  L  I  F  Y  T  F  I  *  *  T  S  C  I  K  I  V  F  F  A  G  S  T  L  L  I  L  K  L  N  G  N  *  V  K  R  E  S  S  G  F  E  L  *  V  M  P  N  T  I  T  H  V  K  Q  K  *  S  M  R  L  L    F5
      L  F  K  M  F  C  Y  L  I  H  L  Y  K  N  P  L  A  Y  K  *  *  L  F  L  V  P  L  *  C  Y  *  N  *  I  V  T  E  F  K  E  K  P  R  D  L  S  L  D  Y  W  R  T  R  *  Q  T  Y  K  R  N  K  A  *  A  C  Y     F4


      R  K  *  S  I  I  R  Y  P  L  R  I  *  H  I  V  *  L  S  I  *  S  G  V  W  R  A  S  V  T  S  Q  L  *  C  I  V  F  K  H  G  *  L  S  R  R  R  E  V  *  *  X                                                       F1
      E  N  R  V  S  F  G  T  P  C  A  S  S  I  S  Y  S  Y  R  F  E  A  E  C  G  E  H  P  *  R  H  S  C  N  V  L  F  L  N  M  A  D  *  A  G  D  G  K  F  D  S  X                                                      F2
     T  K  I  E  Y  H  S  V  P  L  A  H  L  A  Y  R  I  V  I  D  L  K  R  S  V  E  S  I  R  D  V  T  V  V  M  Y  C  F  *  T  W  L  T  E  Q  E  T  G  S  L  I  V                                                       F3
8001 ACGAAAATAGAGTATCATTCGGTACCCCTTGCGCATCTAGCATATCGTATAGTTATCGATTTGAAGCGGAGTGTGGAGAGCATCCGTGACGTCACAGTTGTAATGTATTGTTTTTAAACATGGCTGACTGAGCAGGAGACGGGAAGTTTGATAGTG 8156
     ----:----|----:----|----:----|----:----|----:----|----:----|----:----|----:----|----:----|----:----|----:----|----:----|----:----|----:----|----:----|----:-
8001 TGCTTTTATCTCATAGTAAGCCATGGGGAACGCGTAGATCGTATAGCATATCAATAGCTAAACTTCGCCTCACACCTCTCGTAGGCACTGCAGTGTCAACATTACATAACAAAAATTTGTACCGACTGACTCGTCCTCTGCCCTTCAAACTATCAC 8156
      R  F  Y  L  I  M  R  Y  G  K  R  M  *  C  I  T  Y  N  D  I  Q  L  P  T  H  L  A  D  T  V  D  C  N  Y  H  I  T  K  L  C  P  Q  S  L  L  L  R  S  T  Q  Y  H                                                       F6
      V  F  I  S  Y  *  E  T  G  R  A  C  R  A  Y  R  I  T  I  S  K  F  R  L  T  S  L  M  R  S  T  V  T  T  I  Y  Q  K  *  V  H  S  V  S  C  S  V  P  L  K  I  T                                                       F5
     Y  S  F  L  T  D  N  P  V  G  Q  A  D  L  M  D  Y  L  *  R  N  S  A  S  H  P  S  C  G  H  R  *  L  Q  L  T  N  N  K  F  M  A  S  Q  A  P  S  P  F  N  S  L                                                        F4
```

M.philippinarum (Mph)

WGS: MJUU01063471.1

1-3100
Modiolus philippinarum isolate TK14-01 Mph_scaf_63473, whole genome shotgun sequence

```
      * K Q W Y E N I S K * E F K F C E K * M K * S M N Y I K R S K * R * F L F V H V F N K K V V * N * F I Y G S K V Q A K V L Y N L I I I   F1
      E N N G T K T F Q N E N S N F V K N R * N N L * I T * K G Q N R D D F Y L Y M Y L T K K L F E I N S S M D P K F R P K F C T T L * * F  F2
      K T M V R K H F K M R I Q I L * K I D E I I Y E L H K K V K I E M I S I C T C I * Q K S C L K L I H L W I Q S S G Q S S V Q P Y N N   F3
   1 TGAAAACAATGGTACGAAAACATTTCAAAATGAGAATTCAAATTTTGTGAAAAATAGATGAAATAATCTATGAATTACATAAAAAGGTCAAAATAGAGATGATTTCTATTTGTACATGTATTTAACAAAAAAGTTGTTTGAAATTAATTCATCTATGGATCCAAAGTTCAGGCCAAAGTTCTGTACAACCTTATAATAAT 200
     ----:----|----:----|----:----|----:----|----:----|----:----|----:----|----:----|----:----|----:----|----:----|----:----|----:----|----:----|----:----|----:----|----:----|----:----|----:----|----:----|
   1 ACTTTTGTTACCATGCTTTTGTAAAGTTTTACTCTTAAGTTTAAAACACTTTTTATCTACTTTATTAGATACTTAATGTATTTTTCCAGTTTTATCTCTACTAAAGATAAACATGTACATAAATTGTTTTTTCAACAAACTTTAATTAAGTAGATACCTAGGTTTCAAGTCCGGTTTCAAGACATGTTGGAATATTATTA 200
      X F C H Y S F M E F H S N L N Q S F Y I F Y D I F * M F L D F Y L H N R N T C T N L L F T T Q F * N M * P D L T * A L T R Y L R I I I  F6
      X F V I T R F C K L I L I * I K H F I S S I I * S N C L F T L I S I I E I Q V H I * C F L Q K F N I * R H I W L E P W L E T C G * L L  F5
       S F L P V F V N * F S F E F K T F F L H F L R H I V Y F P * F L S S K * K Y M Y K V F F N N S I L E D I S G F N L G F N Q V V K Y Y   F4

      C N V N F K Y F I H R R G Q V R L L W P V W K H N E H L F F F C S F S A S F P R A L L E Y S Q W S F R T S H K L P Y V L P L L D K F L  F1
       V M S I S N T L F T E E D K C V C C G Q F G N I M N T Y S F F A L F Q Q V F Q G L C L N I A S G V S G H H T N C H M Y C L F W T S F   F2
      L * C Q F Q I L Y S Q K R T S A S V V A S L E T * * T L I L F L L F F S K F S K G F A * I * P V E F Q D I T Q T A I C T A S S G Q V F  F3
 201 TTGTAATGTCAATTTCAAATACTTTATTCACAGAAGAGGACAAGTGCGTCTGTTGTGGCCAGTTTGGAAACATAATGAACACTTATTCTTTTTTTGCTCTTTTTCAGCAAGTTTTCCAAGGGCTTTGCTTGAATATAGCCAGTGGAGTTTCAGGACATCACACAAACTGCCATATGTACTGCCTCTTCTGGACAAGTTTT 400
     ----:----|----:----|----:----|----:----|----:----|----:----|----:----|----:----|----:----|----:----|----:----|----:----|----:----|----:----|----:----|----:----|----:----|----:----|----:----|----:----|
 201 AACATTACAGTTAAAGTTTATGAAATAAGTGTCTTCTCCTGTTCACGCAGACAACACCGGTCAAACCTTTGTATTACTTGTGAATAAGAAAAAAACGAGAAAAAGTCGTTCAAAAGGTTCCCGAAACGAACTTATATCGGTCACCTCAAAGTCCTGTAGTGTGTTTGACGGTATACATGACGGAGAAGACCTGTTCAAAA 400
       Q L T L K L Y K I * L L P C T R R N H G T Q F C L S C K N K K Q E K E A L K G L A K S S Y L W H L K L V D C L S G Y T S G R R S L N  F6
      K Y H * N * I S * E C F L V L A D T T A L K S V Y H V S I R K K S K K L L N E L P K A Q I Y G T S N * S M V C V A M H V A E E P C T K  F5
      N T I D I E F V K N V S S S L H T Q Q P W N P F M I F V * E K K A R K * C T K W P S Q K F I A L P T E P C * V F Q W I Y Q R K Q V L K  F4

       R K C R K S L L P A S L P S E L K L P M A P G P A G S C I N C C V C S I N L * R * F G Q R T * S K F E S N S L P I S T A Q T L N K L   F1
      Y G S A E K V C C L P H Y L Q N * S F L W H Q D Q R D L V L I A V C V R S T Y E G N S A N E H S Q N L R V I V Y P S P Q H K P * I N *  F2
       T E V P K K F V A C L I T F R T K A S Y G T R T S G I L Y * L L C V F D Q L M K V I R P T N I V K I * E * * F T H L H S T N L K * T N F3
 401 TACGGAAGTGCCGAAAAAGTTGTTGCCTGCCTCATTACCTTCAGAACTAAAGCTTCCTATGGCACCAGGACCAGCGGGATCTTGTATTAATTGCTGTGTGTGTTCGATCAACTTATGAAGGTAATTCGGCCAACGAACATAGTCAAAATTTGAGAGTAATAGTTTACCCATCTCCACAGCACAAACCTTAAATAAACTA 600
     ----:----|----:----|----:----|----:----|----:----|----:----|----:----|----:----|----:----|----:----|----:----|----:----|----:----|----:----|----:----|----:----|----:----|----:----|----:----|----:----|
 401 ATGCCTTCACGGCTTTTTCAAACAACGGACGGAGTAATGGAAGTCTTGATTTCGAAGGATACCGTGGTCCTGGTCGCCCTAGAACATAATTAACGACACACACAAGCTAGTTGAATACTTCCATTAAGCCGGTTGCTTGTATCAGTTTTAAACTCTCATTATCAAATGGGTAGAGGTGTCGTGTTTGGAATTTATTTGAT 600
       K R F H R F L K N G A E N G E S S F S G I A G P G A P D Q I L Q Q T H E I L K H L Y N P W R V Y D F N S L L L K G M E V A C V K F L S  F6
       V S T G F F N T A Q R M V K L V L A E * P V L V L P I K Y * N S H T N S * S I F T I R G V F M T L I Q S Y Y N V W R W L V F R L Y V   F5
      * P L A S F T Q Q R G * * R * F * L K R H C W S W R S R T N I A T H T R D V * S P L E A L S C L * F K L T I T * G D G C C L G * I F *  F4

      M S L G K S D F G S L D L Y I F L K K L N L S K M T D K F C C L S T S E I R H K S M S * G V V K I V L A * L P G I I S * G L M P Q C S  F1
      C H W G N L I L G H * T Y I F S L K N * I C L K * Q I N S V A F L H Q K * D I K V C H E E W * K * S * H N Y Q A S S V E D * C H N A L  F2
       V I G E I * F W V I R P I Y F P * K I K F V * N D R * I L L P F Y I R N K T * K Y V M R S G K N S P S I I T R H H Q L R I D A T M L   F3
 601 ATGTCATTGGGGAAATCTGATTTTGGGTCATTAGACCTATATATTTTCCTTAAAAAATTAAATTTGTCTAAAATGACAGATAAATTCTGTTGCCTTTCTACATCAGAAATAAGACATAAAAGTATGTCATGAGGAGTGGTAAAAATAGTCCTAGCATAATTACCAGGCATCATCAGTTGAGGATTGATGCCACAATGCTC 800
     ----:----|----:----|----:----|----:----|----:----|----:----|----:----|----:----|----:----|----:----|----:----|----:----|----:----|----:----|----:----|----:----|----:----|----:----|----:----|----:----|
 601 TACAGTAACCCCTTTAGACTAAAACCCAGTAATCTGGATATATAAAAGGAATTTTTTAATTTAAACAGATTTTACTGTCTATTTAAGACAACGGAAAGATGTAGTCTTTATTCTGTATTTTCATACAGTACTCCTCACCATTTTTATCAGGATCGTATTAATGGTCCGTAGTAGTCAACTCCTAACTACGGTGTTACGAG 800
       I D N P F D S K P D N S R Y I K R L F N F K D L I V S L N Q Q R E V D S I L C L L I D H P T T F I T R A Y N G P M M L Q P N I G C H E  F6
      L T M P S I Q N Q T M L G I Y K G * F I L N T * F S L Y I R N G K * M L F L V Y F Y T M L L P L F L G L M I V L C * * N L I S A V I S  F5
       H * Q P F R I K P * * V * I N E K F F * I Q R F H C I F E T A K R C * F Y S M F T H * S S H Y F Y D * C L * W A D D T S S Q H W L A   F4

       F M W K S N L L S A F F T I S F M S S D N S H L V T H A I S L T I I F L K K L P I L M S A C V A S I P F F I G S I K L M G S D F M P F  F1
       S C G N Q I C F L H F S L F P L C P L I I P I * L H M L F L L L L F S * K N Y P Y * C L H V W H Q S P S L L V P L N S W V Q I L C L   F2
      F H V E I K F A F C I F H Y F L Y V L * * F P F S Y T C Y F S Y Y Y F L K K T T H I D V C M C G I N P L L Y W F H * T H G F R F Y A F  F3
 801 TTTCATGTGGAAATCAAATTTGCTTTCTGCATTTTTCACTATTTCCTTTATGTCCTCTGATAATTCCCATTTAGTTACACATGCTATTTCTCTTACTATTATTTTCTTAAAAAAACTACCCATATTGATGTCTGCATGTGTGGCATCAATCCCCTTCTTTATTGGTTCCATTAAACTCATGGGTTCAGATTTTATGCCTT 1000
     ----:----|----:----|----:----|----:----|----:----|----:----|----:----|----:----|----:----|----:----|----:----|----:----|----:----|----:----|----:----|----:----|----:----|----:----|----:----|----:----|
 801 AAAGTACACCTTTAGTTTAAACGAAAGACGTAAAAAGTGATAAAGGAAATACAGGAGACTATTAAGGGTAAATCAATGTGTACGATAAAGAGAATGATAATAAAAGAATTTTTTTGATGGGTATAACTACAGACGTACACACCGTAGTTAGGGGAAGAAATAACCAAGGTAATTTGAGTACCCAAGTCTAAAATACGGAA 1000
       K M H F D F K S E A N K V I E K I D E S L E W K T V C A I E R V I I K K F F S G M N I D A H T A D I G K K I P E M L S M P E S K I G   F6
      K * T S I L N A K Q M K * * K R * T R Q Y N G N L * V H * K E * * * K R L F V V W I S T Q M H P M L G R R * Q N W * V * P N L N * A K  F5
      R E H P F * I Q K R C K E S N G K H G R I I G M * N C M S N R K S N N E * F F * G Y Q H R C T H C * D G E K N T G N F E H T * I K H R  F4

       D F F A N S V S D S L S G L I L I Y S * I L D V S M M D L L I S K E P N F C V A V S R S A S H R V H K N F D P L P C K P P S D L S R   F1
      L I F L P I L F L T V C Q D * F L Y I H K F W M F Q * W T C * F Q R N L T F V L L C L D Q H H I E Y T K T L I H Y L A N H H L I Y P V  F2
      * F F C Q F C F * Q F V R I N S Y I F I N F G C F N D G P V N F K G T * L L C C C V S I S I T * S T Q K L * S T T L Q T T I * S I P F  F3
1001 TTGATTTTTTTGCCAATTCTGTTTCTGACAGTTTGTCAGGATTAATTCTTATATATTCATAAATTTTGGATGTTTCAATGATGGACCTGTTAATTTCAAAGGAACCTAACTTTTGTGTTGCTGTGTCTCGATCAGCATCACATAGAGTACACAAAAACTTTGATCCACTACCTTGCAAACCACCATCTGATCTATCCCGT 1200
     ----:----|----:----|----:----|----:----|----:----|----:----|----:----|----:----|----:----|----:----|----:----|----:----|----:----|----:----|----:----|----:----|----:----|----:----|----:----|----:----|
1001 AACTAAAAAAACGGTTAAGACAAAGACTGTCAAACAGTCCTAATTAAGAATATATAAGTATTTAAAACCTACAAAGTTACTACCTGGACAATTAAAGTTTCCTTGGATTGAAAACACAACGACACAGAGCTAGTCGTAGTGTATCTCATGTGTTTTTGAAACTAGGTGATGGAACGTTTGGTGGTAGACTAGATAGGGCA 1200
      K S K K A L E T E S L K D P N I R I Y E Y I K S T E I I S R N I E F S G L K Q T A T D R D A D C L T C L F K S G S G Q L G G D S R D R  F6
       Q N K Q W N Q K Q C N T L I L E * I N M F K P H K L S P G T L K L P V * S K H Q Q T E I L M V Y L V C F S Q D V V K C V V M Q D I G   F5
      K I K K G I R N R V T Q * S * N K Y I * L N Q I N * H H V Q * N * L F R V K T N S H R S * C * M S Y V F V K I W * R A F W W R I * G T  F4

      F S S T I E L K K L N S W R C H P Y E V F T F M I L F C K Y F L S F S I G A M H T E I V D L L F S S A N D S S N G L L F F T E L G E D  F1
       F H L P * N * K N * I H G D A I H M K F L L S * F Y F V N I F S H F Q L V P C I L K L L T Y C F H L P M I L V M V C C F L Q S W E R T F2
      F I Y H R I E K I K F M A M P S I * S F Y F H D F I L * I F S L I F N W C H A Y * N C * L I V F I C Q * F * * W F V V F Y R V G R G  F3
1201 TTTTCATCTACCATAGAATTGAAAAAATTAAATTCATGGCGATGCCATCCATATGAAGTTTTTACTTTCATGATTTTATTTTGTAAATATTTTCTCTCATTTTCAATTGGTGCCATGCATACTGAAATTGTTGACTTATTGTTTTCATCTGCCAATGATTCTAGTAATGGTTTGTTGTTTTTTACAGAGTTGGGAGAGGA 1400
     ----:----|----:----|----:----|----:----|----:----|----:----|----:----|----:----|----:----|----:----|----:----|----:----|----:----|----:----|----:----|----:----|----:----|----:----|----:----|----:----|
1201 AAAAGTAGATGGTATCTTAACTTTTTTAATTTAAGTACCGCTACGGTAGGTATACTTCAAAAATGAAAGTACTAAAATAAAACATTTATAAAAGAGAGTAAAAGTTAACCACGGTACGTATGACTTTAACAACTGAATAACAAAAGTAGACGGTTACTAAGATCATTACCAAACAACAAAAAATGTCTCAACCCTCTCCT 1400
      K E D V M S N F F N F E H R H W G Y S T K V K M I K N Q L Y K R E N E I P A M C V S I T S K N N E D A L S E L L P K N N K V S N P S S  F6
      N K M * W L I S F I L N M A I G D M H L K * K * S K I K Y I N E R M K L Q H W A Y Q F Q Q S I T K M Q W H N * Y H N T T K * L T P L P  F5
       K * R G Y F Q F F * I * P S A M W I F N K S E H N * K T F I K E * K * N T G H M S F N N V * Q K * R G I I R T I T Q Q K K C L Q S L   F4

       S V N T V K M P E G E T A S I L T T E K E N L K A L S G R N L S L R S L Y T E Q S P I P S P P S L T V V R N K I D G S S G F K L * Y F F1
      Q * T L * K C Q K G K Q L Q S * Q Q K K K T * R L C R E E I Y H C V P Y I L N S L P Y H L H H L * L * L E I K * M D H Q D L N Y D I   F2
      L S E H C E N A R R G N S F N L D N R K R K P K G F V G K K S I T A F P I Y * T V S H T I S T I F N C S * K * N R W I I R I * I M I F  F3
1401 CTCAGTGAACACTGTGAAAATGCCAGAAGGGGAAACAGCTTCAATCTTGACAACAGAAAAAGAAAACCTAAAGGCTTTGTCGGGAAGAAATCTATCACTGCGTTCCCTATATACTGAACAGTCTCCCATACCATCTCCACCATCTTTAACTGTAGTTAGAAATAAAATAGATGGATCATCAGGATTTAAATTATGATATT 1600
     ----:----|----:----|----:----|----:----|----:----|----:----|----:----|----:----|----:----|----:----|----:----|----:----|----:----|----:----|----:----|----:----|----:----|----:----|----:----|----:----|
1401 GAGTCACTTGTGACACTTTACGGTCTTCCCCTTTGTCGAAGTTAGAACTGTTGTCTTTTTCTTTTGGATTTCCGAAACAGCCCTTCTTTAGATAGTGACGCAAGGGATATATGACTTGTCAGAGGGTATGGTAGAGGTGGTAGAAATTGACATCAATCTTTATTTTATCTACCTAGTAGTCCTAAATTTAATACTATAA 1600
       E T F V T F I G S P S V A E I K V V S F S F R F A K D P L F R D S R E R Y V S C D G M G D G G D K V T T L F L I S P D D P N L N H Y  F6
       S L S C Q S F A L L P F L K L R S L L F L F G L P K T P F F D I V A N G I Y Q V T E W V M E V M K L Q L * F Y F L H I M L I * I I I N F5
      V * H V S H F H W F P F C S * D Q C C F F F V * L S Q R S S I * * Q T G * I S F L R G Y W R W W R * S Y N S I F Y I S * * S K F * S I  F4

       K P E T S S S E S S S T V F A R A S V Y D Q R T P I Q L G D G N S G K F V Q K S P R I L I G S D E Q G V L Y I F S K K S S P I I E Y   F1
      S N L K L V L Q R A P L Q F L Q G H L Y M T N E H P Y N * V M E T R G S L Y K N L Q G Y * L G Q M N R E S Y I Y F Q K N H L Q * L N T  F2
      Q T * N * F F R E L L Y S F C K G I C I * P T N T H T I R * W K L G E V C T K I S K D I D W V R * T G S L I Y I F K K I I S N N * I L  F3
1601 TCAAACCTGAAACTAGTTCTTCAGAGAGCTCCTCTACAGTTTTTGCAAGGGCATCTGTATATGACCAACGAACACCCATACAATTAGGTGATGGAAACTCGGGGAAGTTTGTACAAAAATCTCCAAGGATATTGATTGGGTCAGATGAACAGGGAGTCTTATATATATTTTCAAAAAAATCATCTCCAATAATTGAATAC 1800
     ----:----|----:----|----:----|----:----|----:----|----:----|----:----|----:----|----:----|----:----|----:----|----:----|----:----|----:----|----:----|----:----|----:----|----:----|----:----|----:----|
1601 AGTTTGGACTTTGATCAAGAAGTCTCTCGAGGAGATGTCAAAAACGTTCCCGTAGACATATACTGGTTGCTTGTGGGTATGTTAATCCACTACCTTTGAGCCCCTTCAAACATGTTTTTAGAGGTTCCTATAACTAACCCAGTCTACTTGTCCCTCAGAATATATATAAAAGTTTTTTTAGTAGAGGTTATTAACTTATG 1800
      K L G S V L E E S L E E V T K A L A D T Y S W R V G M C N P S P F E P F N T C F D D G L I N I P D S S C P T K Y I N E F F D D G I I S Y  F6
      * V Q F * N K L S S R * L K Q L P M Q I H G V F V W V I L H H F S P S T Q V F I E L S I S Q T L H V P L R I Y I K L F I M E L L Q I   F5
       E F R F S T R * L A G R C N K C P C R Y I V L S C G Y L * T I S V R P L K Y L F R W P Y Q N P * I F L S D * I Y K * F F * R W Y N F V F4

      S T * P G R Y L L S T L S S * L G H * N T * T L F D F K K S Y * H L Y C P L L C R R F T L S A K H * S A D I V S L E P C Q S M S I S F  F1
      Q H D Q E D I C F Q L C P A D * D T E T H E H Y L I L R S H T D I C T A P Y F A E D L P L V L N I D Q Q T * Y H L N L A R A C Q Y L S  F2
       N M T R K I F A F N F V Q L T R T L K H M N I I * F * E V I L T F V L P L T L Q K I Y P * C * T L I S R H S I T * T L P E H V N I F   F3
1801 TCAACATGACCAGGAAGATATTTGCTTTCAACTTTGTCCAGCTGACTAGGACACTGAAACACATGAACATTATTTGATTTTAAGAAGTCATACTGACATTTGTACTGCCCCTTACTTTGCAGAAGATTTACCCTTAGTGCTAAACATTGATCAGCAGACATAGTATCACTTGAACCTTGCCAGAGCATGTCAATATCTTT 2000
     ----:----|----:----|----:----|----:----|----:----|----:----|----:----|----:----|----:----|----:----|----:----|----:----|----:----|----:----|----:----|----:----|----:----|----:----|----:----|----:----|
1801 AGTTGTACTGGTCCTTCTATAAACGAAAGTTGAAACAGGTCGACTGATCCTGTGACTTTGTGTACTTGTAATAAACTAAAATTCTTCAGTATGACTGTAAACATGACGGGGAATGAAACGTCTTCTAAATGGGAATCACGATTTGTAACTAGTCGTCTGTATCATAGTGAACTTGGAACGGTCTCGTACAGTTATAGAAA 2000
       E V H G P L Y K S E V K D L Q S P C Q F V H V N N S K L F D Y Q C K Y Q G K S Q L L N V R L A L C Q D A S M T D S S G Q W L M D I D K F6
      S L M V L F I N A K L K T W S V L V S F C M F M I Q N * S T M S V N T S G R V K C F I * G * H * V N I L L C L I V Q V K G S C T L I K  F5
       * C S W S S I Q K * S Q G A S * S V S V C S C * K I K L L * V S M Q V A G * K A S S K G K T S F M S * C V Y Y * K F R A L A H * Y R   F4

       Q R E S D E S F K * F L N N E N R T S V L F S P S V S Q K S S I E L M I G L S L L C L T C F E V T S C K G N L G F L K L Y I C V G S V F1
       S E N Q T N L L N D F S T M K I E R Q F C F L L L C H K S H R * N * * L V * A F C V * H V L K * L H V R V I * V F * N C I F V L V V   F2
      P A R I R R I F * M I S Q Q * K * N V S F V F S F C V T K V I D R T D D W F E P S V F N M F * S D F M * G * F R F F K I V Y L C W * C  F3
2001 CCAGCGAGAATCAGACGAATCTTTTAAATGATTTCTCAACAATGAAAATAGAACGTCAGTTTTGTTTTCTCCTTCTGTGTCACAAAAGTCATCGATAGAACTGATGATTGGTTTGAGCCTTCTGTGTTTAACATGTTTTGAAGTGACTTCATGTAAGGGTAATTTAGGTTTTTTAAAATTGTATATTTGTGTTGGTAGTG 2200
     ----:----|----:----|----:----|----:----|----:----|----:----|----:----|----:----|----:----|----:----|----:----|----:----|----:----|----:----|----:----|----:----|----:----|----:----|----:----|----:----|
2001 GGTCGCTCTTAGTCTGCTTAGAAAATTTACTAAAGAGTTGTTACTTTTATCTTGCAGTCAAAACAAAAGAGGAAGACACAGTGTTTTCAGTAGCTATCTTGACTACTAACCAAACTCGGAAGACACAAATTGTACAAAACTTCACTGAAGTACATTCCCATTAAATCCAAAAAATTTTAACATATAAACACAACCATCAC 2200
       W R S D S S D K L H N R L L S F L V D T K N E G E T D C F D D I S S I I P K L R R H K V H K S T V E H L P L K P K K F N Y I Q T P L  F6
       G A L I L R I K * I I E * C H F Y F T L K T K E K Q T V F T M S L V S S Q N S G E T N L M N Q L S K M Y P Y N L N K L I T Y K H Q Y H F5
      E L S F * V F R K K F S K E V I F I S R * N Q K R R R H * L L * R Y F Q H N T Q A K Q T * C T K F H S * T L T I * T K * F Q I N T N T T F4

       C * H E T S C N L I F F K A T F A * Q F V W T H L T T K L S Y I C N K F P L S G K T S V S S K Q H L Q T G H A D D T L T Q L L I H S   F1
      C V D M R H H A I * Y F S K Q P L L D N L Y G H I * Q L N C H I Y V I N F H * V A K H Q S H L N N T Y K L D M Q M I H * P N F * Y I L  F2
      V L T * D I M Q F N I F Q S N L C L T I C M D T F N N * T V I Y M * * I S T K W Q N I S L I * T T P T N W T C R * Y I N P T S N T F *  F3
2201 TGTGTTGACATGAGACATCATGCAATTTAATATTTTTCAAAGCAACCTTTGCTTGACAATTTGTATGGACACATTTAACAACTAAACTGTCATATATATGTAATAAATTTCCACTAAGTGGCAAAACATCAGTCTCATCTAAACAACACCTACAAACTGGACATGCAGATGATACATTAACCCAACTTCTAATACATTCT 2400
     ----:----|----:----|----:----|----:----|----:----|----:----|----:----|----:----|----:----|----:----|----:----|----:----|----:----|----:----|----:----|----:----|----:----|----:----|----:----|----:----|
2201 ACACAACTGTACTCTGTAGTACGTTAAATTATAAAAAGTTTCGTTGGAAACGAACTGTTAAACATACCTGTGTAAATTGTTGATTTGACAGTATATATACATTATTTAAAGGTGATTCACCGTTTTGTAGTCAGAGTAGATTTGTTGTGGATGTTTGACCTGTACGTCTACTATGTAATTGGGTTGAAGATTATGTAAGA 2400
      T H Q C S V D H L K I N K L A V K A Q C N T H V C K V V L S D Y I H L L N G S L P L V D T E D L C C R C V P C A S S V N V W S R I C E  F6
       T N V H S M M C N L I K * L L R Q K V I Q I S V N L L * V T M Y I Y Y I E V L H C F M L R M * V V G V F Q V H L H Y M L G V E L V N  F5
       H T S M L C * A I * Y K E F C G K S S L K Y P C M * C S F Q * I Y T I F K W * T A F C * D * R F L V * L S S M C I I C * G L K * Y M R F4
```

```
      E Q K I * L H E V I T G T N G T P L Q I E Q T N T P A I * G S Q N L S I H K L S I I T L L K F K I L L F E T S F S N D K F F N N K V S    F1
       S K K Y D Y M R L * Q V Q M E H L Y K * N K Q T L Q Q F E D P K I F Q Y T N Y Q * L H C * N S K Y Y F L K P H F L M T N F S I I K Y H   F2
        A K N M I T * G Y N R Y K W N T S T N R T N K H S S N L R I P K S F N T Q T I N N Y I V K I Q N I T F * N L I F * * Q I F Q * * S I    F3
2401 GAGCAAAAAATATGATTACATGAGGTTATAACAGGTACAAATGGAACACCTCTACAAATAGAACAAACAAACACTCCAGCAATTTGAGGATCCCAAAATCTTTCAATACACAAACTATCAATAATTACATTGTTAAAATTCAAAATATTACTTTTTGAAACCTCATTTTCTAATGACAAATTTTTCAATAATAAAGTATC 2600
     ----:----|----:----|----:----|----:----|----:----|----:----|----:----|----:----|----:----|----:----|----:----|----:----|----:----|----:----|----:----|----:----|----:----|----:----|----:----|----:----|
2401 CTCGTTTTTTATACTAATGTACTCCAATATTGTCCATGTTTACCTTGTGGAGATGTTTATCTTGTTTGTTTGTGAGGTCGTTAAACTCCTAGGGTTTTAGAAAGTTATGTGTTTGATAGTTATTAATGTAACAATTTTAAGTTTTATAATGAAAAACTTTGGAGTAAAAGATTACTGTTTAAAAAGTTATTATTTCATAG 2600
       S C F I H N C S T I V P V F P V G R C I S C V F V G A I Q P D W F R E I C L S D I I V N N F N L I N S K S V E N E L S L N K L L L T D   F6
      Q A F F I I V H P * L L Y L H F V E V F L V F L C E L L K L I G F D K L V C V I L L * M T L I * F I V K Q F R M K * H C I K * Y Y L I    F5
        L L F Y S * M L N Y C T C I S C R * L Y F L C V S W C N S S G L I K * Y V F * * Y N C Q * F E F Y * K K F G * K R I V F K E I I F Y     F4

       L S E S D P L K S C L L V * S K L L V R D K S P Q S R S Q H S E A L S V S L L S * F D R S T A T L D N F L F P T F F G L P L L T Q Q I   F1
        Y Q N L I H L N H V Y W F D Q N Y * C E T N H H S Q D H N T L K H C L C H Y C P D L I D Q L Q L W T T S F S Q L F L A F H F * H N K     F2
      I I R I * S T * I M F I G L I K T T S A R Q I T T V K I T T L * S T V C V T I V L I * * I N C N F G Q L P F P N F F W P S T F N T T N   F3
2601 ATTATCAGAATCTGATCCACTTAAATCATGTTTATTGGTTTGATCAAAACTACTAGTGCGAGACAAATCACCACAGTCAAGATCACAACACTCTGAAGCACTGTCTGTGTCACTATTGTCCTGATTTGATAGATCAACTGCAACTTTGGACAACTTCCTTTTCCCAACTTTTTTTGGCCTTCCACTTTTAACACAACAAA 2800
     ----:----|----:----|----:----|----:----|----:----|----:----|----:----|----:----|----:----|----:----|----:----|----:----|----:----|----:----|----:----|----:----|----:----|----:----|----:----|----:----|
2601 TAATAGTCTTAGACTAGGTGAATTTAGTACAAATAACCAAACTAGTTTTGATGATCACGCTCTGTTTAGTGGTGTCAGTTCTAGTGTTGTGAGACTTCGTGACAGACACAGTGATAACAGGACTAAACTATCTAGTTGACGTTGAAACCTGTTGAAGGAAAAGGGTTGAAAAAAACCGGAAGGTGAAAATTGTGTTGTTT 2800
        N D S D S G S L D H K N T Q D F S S T R S L D G C D L D C C E S A S D T D S N D Q N S L D V A V K S L K R K G V K K P R G S K V C C     F6
       M I L I Q D V * I M N I P K I L V V L A L C I V V T L I V V S Q L V T Q T V I T R I Q Y I L Q L K P C S G K G L K K Q G E V K L V V F   F5
      * * * F R I W K F * T * Q N S * F * * H S V F * W L * S * L V R F C Q R H * * Q G S K I S * S C S Q V V E K E W S K K A K W K * C L L    F4

        L Q S W S * G * K T D N S Q N * I P Q D P L F C L N R V R R S L Q E L Q I F * G Y C C S L S T T T S M S * I S F I S D E K L V L L Q    F1
      F Y N L G H E G K K L T T H K T E Y H K T P C F V * T E * D V A C R N Y R Y F E D I V V H F Q Q P H Q C H K F P L F Q M K N W F C Y S   F2
       F T I L V M R V K N * Q L T K L N T T R P L V L S K P S K T * P A G T T D I L R I L L F T F N N H I N V I N F L Y F R * K I G F V T V  F3
2801 TTTTACAATCTTGGTCATGAGGGTAAAAAACTGACAACTCACAAAACTGAATACCACAAGACCCCTTGTTTTGTCTAAACCGAGTAAGACGTAGCCTGCAGGAACTACAGATATTTTGAGGATATTGTTGTTCACTTTCAACAACCACATCAATGTCATAAATTTCCTTTATTTCAGATGAAAAATTGGTTTTGTTACAG 3000
     ----:----|----:----|----:----|----:----|----:----|----:----|----:----|----:----|----:----|----:----|----:----|----:----|----:----|----:----|----:----|----:----|----:----|----:----|----:----|----:----|
2801 AAAATGTTAGAACCAGTACTCCCATTTTTTGACTGTTGAGTGTTTTGACTTATGGTGTTCTGGGGAACAAAACAGATTTGGCTCATTCTGCATCGGACGTCCTTGATGTCTATAAAACTCCTATAACAACAAGTGAAAGTTGTTGGTGTAGTTACAGTATTTAAAGGAAATAAAGTCTACTTTTTAACCAAAACAATGTC 3000
      I K C D Q D H P Y F V S L E C F Q I G C S G K N Q R F R T L R L R C S S C I N Q P Y Q Q E S E V V V D I D Y I E K I E S S F N T K N C   F6
        K V I K T M L T F F Q C S V F S F V V L G R T K D L G L L V Y G A P V V S I K L I N N N V K L L W M L T M F K R * K L H F I P K T V    F5
       N * L R P * S P L F S V V * L V S Y W L V G Q K T * V S Y S T A Q L F * L Y K S S I T T * K * C G C * H * L N G K N * I F F Q N Q * L  F4

      F F * I L L Q H N R H R L F S L L L C S S L I L L Y F K L K P T M X                                                                       F1
       S F E S C Y N T T D I D S S A Y Y C V L H S F Y F I S N * N Q Q C                                                                        F2
        L L N L A T T Q P T * T L Q L T T V F F T H F T L F Q T E T N N V                                                                       F3
3001 TTCTTTTGAATCTTGCTACAACACAACCGACATAGACTCTTCAGCTTACTACTGTGTTCTTCACTCATTTTACTTTATTTCAAACTGAAACCAACAATGT 3100
     ----:----|----:----|----:----|----:----|----:----|----:----|----:----|----:----|----:----|----:----|
3001 AAGAAAACTTAGAACGATGTTGTGTTGGCTGTATCTGAGAAGTCGAATGATGACACAAGAAGTGAGTAAAATGAAATAAAGTTTGACTTTGGTTGTTACA 3100
       N K Q I K S C C L R C L S K L K S S H E E S M K S * K L S F G V I                                                                        F6
      T R K F R A V V C G V Y V R * S V V T N K V * K V K N * V S V L L T                                                                       F5
       E K S D Q * L V V S M S E E A * * Q T R * E N * K I E F Q F W C H                                                                        F4
```

M.philippinarum (Mph)

WGS: MJUU01034083.1

99577-101108
Modiolus philippinarum isolate TK14-01 Mph_scaf_34084, whole genome shotgun sequence

```
       K  C  T  N  *  N  F  A  W  F  T  C  F  S  L  S  D  I  *  V  V  T  L  F  L  L  Y  *  I  V  V  C  L  H  A  N  E  F  L  Q  K  L  Q  G  I  A  F  *  I  V  F  E  I  F  N  S  E  N  N  Q  E  K  E  *  Y  T  F   F1
        S  V  Q  I  K  I  L  H  G  L  L  V  F  P  F  Q  I  Y  E  W  *  H  C  F  C  C  I  K  L  L  F  V  C  M  L  M  S  F  Y  R  N  F  K  A  L  P  F  K  S  F  S  K  Y  S  T  Q  K  I  T  R  K  R  N  D  I  H  L   F2
         V  Y  K  L  K  F  C  M  V  Y  L  F  F  P  F  R  Y  M  S  G  D  I  V  F  V  V  L  N  C  C  L  F  A  C  *  *  V  F  T  E  T  S  R  H  C  L  L  N  R  F  R  N  I  Q  L  R  K  *  P  G  K  G  M  I  Y  I    F3
   1 AAGTGTACAAATTAAAATTTTGCATGGTTTACTTGTTTTTCCCTTTCAGATATATGAGTGGTGACATTGTTTTTGTTGTATTAAATTGTTGTTTGTTTGCATGCTAATGAGTTTTTACAGAAACTTCAAGGCATTGCCTTTTAAATCGTTTTCGAAATATTCAACTCAGAAAATAACCAGGAAAAGGAATGATATACATT 200
     ----:----|----:----|----:----|----:----|----:----|----:----|----:----|----:----|----:----|----:----|----:----|----:----|----:----|----:----|----:----|----:----|----:----|----:----|----:----|----:----|
   1 TTCACATGTTTAATTTTAAAACGTACCAAATGAACAAAAAGGGAAAGTCTATATACTCACCACTGTAACAAAAACAACATAATTTAACAACAAACAAACGTACGATTACTCAAAAATGTCTTTGAAGTTCCGTAACGGAAAATTTAGCAAAAGCTTTATAAGTTGAGTCTTTTATTGGTCCTTTTCCTTACTATATGTAA 200
       X  H  V  F  *  F  K  A  H  N  V  Q  K  E  R  E  S  I  H  T  T  V  N  N  K  N  Y  *  I  T  T  Q  K  C  A  L  S  N  K  C  F  S  *  P  M  A  K  *  I  T  K  S  I  N  L  E  S  F  L  W  S  F  S  H  Y  V  N   F6
       X  T  Y  L  N  F  N  Q  M  T  *  K  N  K  G  K  L  Y  I  L  P  S  M  T  K  T  T  N  F  Q  Q  K  N  A  H  *  H  T  K  V  S  V  E  L  C  Q  R  K  F  R  K  R  F  I  *  S  L  F  Y  G  P  F  P  I  I  Y  M   F5
        L  T  C  I  L  I  K  C  P  K  S  T  K  G  K  *  I  Y  S  H  H  C  Q  K  Q  Q  I  L  N  N  N  T  Q  M  S  I  L  K  *  L  F  K  L  A  N  G  K  L  D  N  E  F  Y  E  V  *  F  I  V  L  F  L  F  S  I  C    F4

        T  E  Q  *  L  L  S  I  R  R  T  L  C  I  K  M  F  *  K  *  I  Y  L  F  W  W  R  Q  K  V  R  G  I  Y  M  A  K  H  L  *  H  F  *  N  F  L  *  Y  *  R  *  *  C  Q  Y  *  Q  Y  N  T  I  K  I  N  W  V  N   F1
         Q  N  N  D  F  Y  P  S  E  G  H  C  A  L  K  C  S  E  N  E  F  I  F  F  G  G  A  R  R  L  E  E  S  T  W  P  S  T  C  N  I  F  K  I  S  F  D  I  E  D  D  D  V  S  I  D  S  I  T  Q  L  K  L  I  G  S    F2
       Y  R  T  M  T  S  I  H  Q  K  D  I  V  H  *  N  V  L  K  M  N  L  S  F  L  V  A  P  E  G  *  R  N  L  H  G  Q  A  L  V  T  F  L  K  F  P  L  I  L  K  M  M  M  S  V  L  T  V  *  H  N  *  N  *  L  G  Q   F3
 201 TACAGAACAATGACTTCTATCCATCAGAAGGACATTGTGCATTAAAATGTTCTGAAAATGAATTTATCTTTTTTGGTGGCGCCAGAAGGTTAGAGGAATCTACATGGCCAAGCACTTGTAACATTTTTAAAATTTCCTTTGATATTGAAGATGATGATGTCAGTATTGACAGTATAACACAATTAAAATTAATTGGGTCA 400
     ----:----|----:----|----:----|----:----|----:----|----:----|----:----|----:----|----:----|----:----|----:----|----:----|----:----|----:----|----:----|----:----|----:----|----:----|----:----|----:----|
 201 ATGTCTTGTTACTGAAGATAGGTAGTCTTCCTGTAACACGTAATTTTACAAGACTTTTACTTAAATAGAAAAAACCACCGCGGTCTTCCAATCTCCTTAGATGTACCGGTTCGTGAACATTGTAAAAATTTTAAAGGAAACTATAACTTCTACTACTACAGTCATAACTGTCATATTGTGTTAATTTTAATTAACCCAGT 400
       V  S  C  H  S  R  D  M  L  L  V  N  H  M  L  I  N  Q  F  H  I  *  R  K  Q  H  R  W  F  T  L  P  I  *  M  A  L  C  K  Y  C  K  *  F  K  R  Q  Y  Q  L  H  H  H  *  Y  Q  C  Y  L  V  I  L  I  L  Q  T   F6
      *  L  V  I  V  E  I  W  *  F  S  M  T  C  *  F  T  R  F  I  F  K  D  K  K  T  A  G  S  P  *  L  F  R  C  P  W  A  S  T  V  N  K  F  N  G  K  I  N  F  I  I  I  D  T  N  V  T  Y  C  L  *  F  *  N  P  *   F5
     K  C  F  L  S  K  *  G  D  S  P  C  Q  A  N  F  H  E  S  F  S  N  I  K  K  P  P  A  L  L  N  S  S  D  V  H  G  L  V  Q  L  M  K  L  I  E  K  S  I  S  S  S  S  T  L  I  S  L  I  V  C  N  F  N  I  P  D    F4

        L  P  T  S  S  R  I  S  C  Y  I  Y  K  Q  F  C  I  C  M  G  W  T  E  F  G  N  F  *  K  Y  K  *  T  Y  R  V  K  N  T  R  W  *  M  Q  S  *  Y  N  S  R  Y  W  S  T  Q  F  K  P  E  G  Y  D  W  K  C  A    F1
     I  F  P  P  L  Q  G  S  A  A  I  S  I  N  N  S  V  Y  Y  V  W  G  G  L  N  L  E  T  F  K  N  T  S  E  L  I  E  L  K  I  Q  D  G  K  C  K  V  D  I  I  Q  D  I  G  Q  H  S  L  S  R  R  V  M  T  G  N  V  P   F2
       S  S  H  L  F  K  D  Q  L  L  Y  L  *  T  I  L  Y  M  Y  G  V  D  *  I  W  K  L  L  K  I  Q  V  N  L  S  S  *  K  Y  K  M  V  N  A  K  L  I  *  F  K  I  L  V  N  T  V  *  A  G  G  L  *  L  E  M  C  R   F3
 401 ATCTTCCCACCTCTTCAAGGATCAGCTGCTATATCTATAAACAATTCTGTATATGTATGGGGTGGACTGAATTTGGAAACTTTTAAAAATACAAGTGAACTTATCGAGTTAAAAATACAAGATGGTAAATGCAAAGTTGATATAATTCAAGATATTGGTCAACACAGTTTAAGCCGGAGGGTTATGACTGGAAATGTGCC 600
     ----:----|----:----|----:----|----:----|----:----|----:----|----:----|----:----|----:----|----:----|----:----|----:----|----:----|----:----|----:----|----:----|----:----|----:----|----:----|----:----|
 401 TAGAAGGGTGGAGAAGTTCCTAGTCGACGATATAGATATTTGTTAAGACATATACATACCCCACCTGACTTAAACCTTTGAAAATTTTTATGTTCACTTGAATAGCTCAATTTTTATGTTCTACCATTTACGTTTCAACTATATTAAGTTCTATAACCAGTTGTGTCAAATTCGGCCTCCCAATACTGACCTTTACACGG 600
     L  R  G  V  E  E  L  I  L  Q  *  I  *  L  C  N  Q  I  H  I  P  H  V  S  N  P  F  K  *  F  Y  L  H  V  *  R  T  L  F  V  L  H  Y  I  C  L  Q  Y  L  E  L  Y  Q  D  V  C  N  L  G  S  P  *  S  Q  F  H  A   F6
      D  E  W  R  K  L  S  *  S  S  Y  R  Y  V  I  R  Y  I  Y  P  T  S  Q  I  Q  F  S  K  F  I  C  T  F  K  D  L  *  F  Y  L  I  T  F  A  F  N  I  Y  N  L  I  N  T  L  V  T  *  A  P  P  N  H  S  S  I  H    F5
       I  K  G  G  R  *  P  D  A  A  I  D  I  F  L  E  T  Y  T  H  P  P  S  F  K  S  V  K  L  F  V  L  S  S  I  S  N  F  I  C  S  P  L  H  L  T  S  I  I  *  S  I  P  *  C  L  K  L  R  L  T  I  V  P  F  T  G   F4

       V  S  *  M  W  S  Y  Y  D  S  Y  R  *  F  T  C  S  I  I  R  W  Y  *  I  C  K  Q  R  V  F  T  F  F  *  T  *  N  C  *  *  Q  I  L  H  F  *  F  *  Y  I  L  L  D  S  C  C  T  V  A  G  *  T  K  S  L  S  H    F1
        S  A  R  C  G  H  T  M  T  P  I  D  N  S  H  A  V  L  F  G  G  I  E  F  V  N  K  G  C  L  L  S  F  E  H  E  T  V  D  D  K  F  Y  I  F  D  F  N  T  F  C  W  T  P  V  A  Q  L  Q  V  K  P  R  A  Y  H  T   F2
        Q  L  D  V  V  I  L  *  L  L  *  I  I  H  M  Q  Y  Y  S  V  V  L  N  L  *  T  K  G  V  Y  F  L  L  N  M  K  L  L  M  T  N  F  T  F  L  I  L  I  H  F  V  G  L  L  L  H  S  C  R  L  N  Q  E  L  I  T    F3
 601 GTCAGCTAGATGTGGTCATACTATGACTCCTATAGATAATTCACATGCAGTATTATTCGGTGGTATTGAATTTGTAAACAAAGGGTGTTTACTTTCTTTTGAACATGAAACTGTTGATGACAAATTTTACATTTTTGATTTTAATACATTTTGTTGGACTCCTGTTGCACAGTTGCAGGTTAAACCAAGAGCTTATCACA 800
     ----:----|----:----|----:----|----:----|----:----|----:----|----:----|----:----|----:----|----:----|----:----|----:----|----:----|----:----|----:----|----:----|----:----|----:----|----:----|----:----|
 601 CAGTCGATCTACACCAGTATGATACTGAGGATATCTATTAAGTGTACGTCATAATAAGCCACCATAACTTAAACATTTGTTTCCCACAAATGAAAGAAAACTTGTACTTTGACAACTACTGTTTAAAATGTAAAAACTAAAATTATGTAAAACAACCTGAGGACAACGTGTCAACGTCCAATTTGGTTCTCGAATAGTGT 800
       T  L  *  I  H  D  Y  *  S  E  *  L  Y  N  V  H  L  I  I  R  H  Y  Q  I  Q  L  C  L  T  N  V  K  K  Q  V  H  F  Q  Q  H  C  I  K  C  K  Q  N  *  Y  M  K  N  S  E  Q  Q  V  T  A  P  *  V  L  L  K  D  C   F6
     R  *  S  S  T  T  M  S  H  S  R  Y  I  I  *  M  C  Y  *  E  T  T  N  F  K  Y  V  F  P  T  *  K  R  K  F  M  F  S  N  I  V  F  K  V  N  K  I  K  I  C  K  T  P  S  R  N  C  L  Q  L  N  F  W  S  S  I  V   F5
      D  A  L  H  P  *  V  I  V  G  I  S  L  E  C  A  T  N  N  P  P  I  S  N  T  F  L  P  H  K  S  E  K  S  C  S  V  T  S  S  L  N  *  M  K  S  K  L  V  N  Q  Q  V  G  T  A  C  N  C  T  L  G  L  A  *  *    F4

        C  Y  I  C  E  H  *  *  H  *  *  Y  S  L  Y  W  R  L  F  K  K  K  I  C  N  *  K  D  I  S  F  R  N  F  C  S  E  N  F  T  R  *  S  I  S  D  *  L  F  V  V  *  E  C  S  R  L  W  T  F  V  P  L  I  I  M  V   F1
         A  T  F  V  N  I  N  N  I  N  S  I  V  Y  I  G  G  C  L  K  K  K  S  V  I  E  R  I  S  L  S  E  I  F  A  L  K  I  L  P  D  N  Q  F  Q  I  D  Y  L  L  Y  R  N  V  P  D  F  G  L  S  Y  H  S  S  L  W    F2
       L  L  H  L  *  T  L  I  T  L  I  V  *  F  I  L  E  V  V  *  K  K  N  L  *  L  K  G  Y  L  F  Q  K  F  L  L  *  K  F  Y  Q  I  I  N  F  R  L  I  I  C  C  I  G  M  F  Q  T  L  D  F  R  T  T  H  H  Y  G   F3
 801 CTGCTACATTTGTGAACATTAATAACATTAATAGTATAGTTTATATTGGAGGTTGTTTAAAAAAAAAATCTGTAATTGAAAGGATATCTCTTTCAGAAATTTTTGCTCTGAAAATTTTACCAGATAATCAATTTCAGATTGATTATTTGTTGTATAGGAATGTTCCAGACTTTGGACTTTCGTACCACTCATCATTATGG 1000
     ----:----|----:----|----:----|----:----|----:----|----:----|----:----|----:----|----:----|----:----|----:----|----:----|----:----|----:----|----:----|----:----|----:----|----:----|----:----|----:----|
 801 GACGATGTAAACACTTGTAATTATTGTAATTATCATATCAAATATAACCTCCAACAAATTTTTTTTTTAGACATTAACTTTCCTATAGAGAAAGTCTTTAAAAACGAGACTTTTAAAATGGTCTATTAGTTAAAGTCTAACTAATAAACAACATATCCTTACAAGGTCTGAAACCTGAAAGCATGGTGAGTAGTAATACC 1000
        Q  *  M  Q  S  C  *  Y  C  *  Y  Y  L  K  Y  Q  L  N  N  L  F  F  I  Q  L  Q  F  S  I  E  K  L  F  K  Q  E  S  F  K  V  L  Y  D  I  E  S  Q  N  N  T  T  Y  S  H  E  L  S  Q  V  K  T  G  S  M  M  I   F6
       S  S  C  K  H  V  N  I  V  N  I  T  Y  N  I  N  S  T  T  *  F  F  F  R  Y  N  F  P  Y  R  K  *  F  N  K  S  Q  F  N  *  W  I  I  L  K  L  N  I  I  Q  Q  I  P  I  N  W  V  K  S  K  R  V  V  *  *  *  P   F5
     V  A  V  N  T  F  M  L  L  M  L  L  I  T  *  I  P  P  Q  K  F  F  F  D  T  I  S  L  I  D  R  E  S  I  K  A  R  F  I  K  G  S  L  *  N  *  I  S  *  K  N  Y  L  F  T  G  S  K  P  S  E  Y  W  E  D  N  H   F4

         R  L  S  Y  F  Y  F  W  W  Q  Y  K  *  C  F  R  W  N  T  R  Y  Y  Y  T  *  Q  *  N  Y  V  C  K  H  Y  Y  T  *  I  K  F  *  M  F  W  A  F  S  H  K  S  I  *  *  L  S  Y  G  L  W  W  *  T  K  A  V  F   F1
     L  G  S  V  I  F  I  F  G  G  N  T  N  D  V  L  D  G  T  Q  D  I  I  I  L  N  N  E  T  M  S  A  N  I  I  T  L  E  S  S  F  K  C  S  G  H  S  V  I  K  V  S  D  D  C  L  M  V  C  G  G  R  Q  K  Q  Y  F   F2
      *  A  Q  L  F  L  F  L  V  A  I  Q  M  M  F  *  M  E  H  K  I  L  L  Y  L  T  M  K  L  C  L  Q  T  L  L  H  L  N  Q  V  L  N  V  L  G  I  Q  S  *  K  Y  L  M  I  V  L  W  F  V  V  V  D  K  S  S  I  L   F3
1001 TTAGGCTCAGTTATTTTTATTTTTGGTGGCAATACAAATGATGTTTTAGATGGAACACAAGATATTATTATACTTAACAATGAAACTATGTCTGCAAACATTATTACACTTGAATCAAGTTTTAAATGTTCTGGGCATTCAGTCATAAAAGTATCTGATGATTGTCTTATGGTTTGTGGTGGTAGACAAAAGCAGTATTT 1200
     ----:----|----:----|----:----|----:----|----:----|----:----|----:----|----:----|----:----|----:----|----:----|----:----|----:----|----:----|----:----|----:----|----:----|----:----|----:----|----:----|
1001 AATCCGAGTCAATAAAAATAAAAACCACCGTTATGTTTACTACAAAATCTACCTTGTGTTCTATAATAATATGAATTGTTACTTTGATACAGACGTTTGTAATAATGTGAACTTAGTTCAAAATTTACAAGACCCGTAAGTCAGTATTTTCATAGACTACTAACAGAATACCAAACACCACCATCTGTTTTCGTCATAAA 1200
     T  L  S  L  *  K  *  K  Q  H  C  Y  L  H  H  K  L  H  F  V  L  Y  *  *  V  *  C  H  F  *  T  Q  L  C  *  *  V  Q  I  L  N  *  I  N  Q  A  N  L  *  L  L  I  Q  H  N  D  *  P  K  H  H  Y  V  F  A  T  N   F6
      *  A  *  N  N  K  N  K  T  A  I  C  I  I  N  *  I  S  C  L  I  N  N  Y  K  V  I  F  S  H  R  C  V  N  N  C  K  F  *  T  K  F  T  R  P  M  *  D  Y  F  Y  R  I  I  T  K  H  N  T  T  T  S  L  L  L  I    F5
       N  P  E  T  I  K  I  K  P  P  L  V  F  S  T  K  S  P  V  C  S  I  I  I  S  L  L  S  V  I  D  A  F  M  I  V  S  S  D  L  K  L  H  E  P  C  E  T  M  F  T  D  S  S  Q  R  I  T  Q  P  P  L  C  F  C  Y  K   F4

     C  L  H  Q  *  T  Y  G  A  W  G  L  *  S  P  *  *  L  *  N  N  *  I  S  G  N  L  P  N  I  L  D  S  M  *  R  C  V  *  K  V  V  T  P  V  L  C  R  S  F  K  Y  S  C  A  W  E  I  Y  V  Q  E  L  Q  V  K  *   F1
      V  Y  T  S  K  P  M  V  P  G  A  C  D  L  H  D  D  C  K  I  I  E  S  V  E  T  S  P  I  S  W  I  Q  C  E  G  V  C  K  K  W  L  H  Q  F  C  V  G  V  L  N  T  V  V  R  G  K  Y  M  C  K  S  C  K  S  N  S   F2
       F  T  P  V  N  L  W  C  L  G  L  V  I  S  M  M  I  V  K  *  L  N  Q  W  K  P  P  Q  Y  P  G  F  N  V  K  V  C  V  K  S  G  Y  T  S  F  V  *  E  F  *  I  Q  L  C  V  G  N  I  C  A  R  V  A  S  Q  I    F3
1201 TGTTTACACCAGTAAACCTATGGTGCCTGGGGCTTGTGATCTCCATGATGATTGTAAAATAATTGAATCAGTGGAAACCTCCCCAATATCCTGGATTCAATGTGAAGGTGTGTGTAAAAAGTGGTTACACCAGTTTTGTGTAGGAGTTTTAAATACAGTTGTGCGTGGGAAATATATGTGCAAGAGTTGCAAGTCAAATA 1400
     ----:----|----:----|----:----|----:----|----:----|----:----|----:----|----:----|----:----|----:----|----:----|----:----|----:----|----:----|----:----|----:----|----:----|----:----|----:----|----:----|
1201 ACAAATGTGGTCATTTGGATACCACGGACCCCGAACACTAGAGGTACTACTAACATTTTATTAACTTAGTCACCTTTGGAGGGGTTATAGGACCTAAGTTACACTTCCACACACATTTTTCACCAATGTGGTCAAAACACATCCTCAAAATTTATGTCAACACGCACCCTTTATATACACGTTCTCAACGTTCAGTTTAT 1400
      Q  K  C  W  Y  V  *  P  A  Q  P  K  H  D  G  H  H  N  Y  F  L  Q  I  L  P  F  R  G  L  I  R  S  E  I  H  L  H  T  Y  F  T  T  V  G  T  K  H  L  L  K  L  Y  L  Q  A  H  S  I  Y  T  C  S  N  C  T  L  Y   F6
     K  N  V  G  T  F  R  H  H  R  P  S  T  I  E  M  I  I  T  F  Y  N  F  *  H  F  G  G  W  Y  G  P  N  L  T  F  T  H  T  F  L  P  *  V  L  K  T  Y  S  N  *  I  C  N  H  T  P  F  I  H  A  L  T  A  L  *  I   F5
       T  *  V  L  L  G  I  T  G  P  A  Q  S  R  W  S  S  Q  L  I  I  S  D  T  S  V  E  G  I  D  Q  I  *  H  S  P  T  H  L  F  H  N  C  W  N  Q  T  P  T  K  F  V  T  T  R  P  F  Y  I  H  L  L  Q  L  D  F    F4

      Q  V  H  T  *  *  K  E  K  I  L  M  S  M  S  *  C  K  F  L  Y  A  C  *  K  D  R  H  I  M  V  C  L  L  S  V  K  F  I  F  G  F  Q  X                                                                        F1
       K  S  T  R  S  K  K  K  R  Y  *  C  Q  *  V  D  V  N  F  Y  T  P  V  E  K  T  D  I  L  W  Y  A  F  C  L  L  N  L  F  L  V  S  R  X                                                                       F2
     A  S  P  H  V  V  K  R  K  D  T  D  V  N  E  L  M  *  I  F  I  R  L  L  K  R  Q  T  Y  Y  G  M  P  S  V  C  *  I  Y  F  W  F  P  E                                                                          F3
1401 GCAAGTCCACACGTAGTAAAAAGAAAAGATACTGATGTCAATGAGTTGATGTAAATTTTTATACGCCTGTTGAAAAGACAGACATATTATGGTATGCCTTCTGTCTGTTAAATTTATTTTTGGTTTCCAGAG 1532
     ----:----|----:----|----:----|----:----|----:----|----:----|----:----|----:----|----:----|----:----|----:----|----:----|----:----|--
1401 CGTTCAGGTGTGCATCATTTTTCTTTTCTATGACTACAGTTACTCAACTACATTTAAAAATATGCGGACAACTTTTCTGTCTGTATAATACCATACGGAAGACAGACAATTTAAATAAAAACCAAAGGTCTC 1532
      C  T  W  V  Y  Y  F  S  F  I  S  I  D  I  L  Q  H  L  N  K  Y  A  Q  Q  F  S  L  C  I  I  T  H  R  R  D  T  L  N  I  K  P  K  W  L                                                                        F6
      A  L  G  C  T  T  F  L  F  S  V  S  T  L  S  N  I  Y  I  K  I  R  R  N  F  L  C  V  Y  *  P  I  G  E  T  Q  *  I  *  K  Q  N  G  S                                                                         F5
     L  L  D  V  R  L  L  F  F  L  Y  Q  H  *  H  T  S  T  F  K  *  V  G  T  S  F  V  S  M  N  H  Y  A  K  Q  R  N  F  K  N  K  T  E  L                                                                          F4
```

Pinctada imbricata (Pim)

WGS: NIJJ01013975.1

16588-22053
Pinctada imbricata breed pearl oyster-OUG PIN_chr12_3667, whole genome shotgun sequence

```
       T K * A L T N F F A * D K A A I F G K * L C K A A R V T S R N V L R S N L F R Y V K A L F W G W G D S H * H A Q S L F P H R R E T E L   F1
        R S E H L R T S L P K I K P P Y L G N N S V K P P E L P P V T C F V Q I Y F G T * K L Y F G V G G I P T D M R S R Y F R I E E K P S L  F2
         E V S T Y E L L C L R * S R H I W E I T L * S R Q S Y L P * R A S F K S I S V R E S S I L G L G G F P L T C A V A I S A * K R N R A  F3
   1 ACGAAGTGAGCACTTACGAACTTCTTTGCCTAAGATAAAGCCGCCATATTTGGGAAATAACTCTGTAAAGCCGCCAGAGTTACCTCCCGTAACGTGCTTCGTTCAAATCTATTTCGGTACGTGAAAGCTCTATTTTGGGGTTGGGGGGATTCCCACTCACATGCGCAGTCGCTATTTCCGCATAGAAGAGAAACCGAGCT 200
     ----:----|----:----|----:----|----:----|----:----|----:----|----:----|----:----|----:----|----:----|----:----|----:----|----:----|----:----|----:----|----:----|----:----|----:----|----:----|----:----|
   1 TGCTTCACTCGTGAATGCTTGAAGAAACGGATTCTATTTCGGCGGTATAAACCCTTTATTGAGACATTTCGGCGGTCTCAATGGAGGGCATTGCACGAAGCAAGTTTAGATAAAGCCATGCACTTTCGAGATAAAACCCCAACCCCCCTAAGGGTGACTGTACGCGTCAGCGATAAAGGCGTATCTTCTCTTTGGCTCGA 200
      V F H A S V F K K A * S L A A M N P F Y S Q L A A L T V E R L T S R E F R N R Y T F A R N Q P Q P S E W Q C A C D S N G C L L S V S S  F6
     X S T L V * S S R Q R L Y L R W I Q S I V R Y L R W L * R G Y R A E N L D I E T R S L E I K P N P P N G S V H A T A I E A Y F L F R A   F5
       R L S C K R V E K G L I F G G Y K P F L E T F G G S N G G T V H K T * I * K P V H F S * K P T P P I G V S M R L R * K R M S S F G L  F4

      V V D I F R L K I G K F I D F C R N F E V F V F Q Q * K L E D I S C S G I Y V L E S K E D F F V R K R D F F P R S S I L C L S F Y I *  F1
       W W I F S A * K S V S L * I F A G I L K F L Y F S N E N * K I * A A A G Y M C W N R K R I F L * G K E I F F H E A R F F V Y R S I Y  F2
     C G G Y F P L E N R * V Y R F L P E F * S F C I S A M K I R R Y K L Q R D I C V G I E R G F F C E E K R F F S T K L D S L S I V L Y I   F3
 201 TGTGGTGGATATTTTCCGCTTGAAAATCGGTAAGTTTATAGATTTTTGCCGGAATTTTGAAGTTTTTGTATTTCAGCAATGAAAATTAGAAGATATAAGCTGCAGCGGGATATATGTGTGGAATCGAAAGAGGATTTTTTTGTGAGGAAAAGAGATTTTTTTCCACGAAGCTCGATTCTTTGTCTATCGTTCTATATAT 400
     ----:----|----:----|----:----|----:----|----:----|----:----|----:----|----:----|----:----|----:----|----:----|----:----|----:----|----:----|----:----|----:----|----:----|----:----|----:----|----:----|
 201 ACACCACCTATAAAAGGCGAACTTTTAGCCATTCAAATATCTAAAAACGGCCTTAAAACTTCAAAAACATAAAGTCGTTACTTTTAATCTTCTATATTCGACGTCGCCCTATATACACACCTTAGCTTTCCTCCTAAAAAAACACTCCTTTTCTCTAAAAAAAGGTGCTTCGAGCTAAGAAACAGATAGCAAGATATATA 400
       T T S I K R K F I P L N I S K Q R F K S T K T N * C H F N S S I L Q L P I Y T N S D F S S K K T L F L S K K G R L E I R Q R D N * I  F6
      Q P P Y K G S S F R Y T * L N K G S N Q L K Q I E A I F I L L Y L S C R S I H T P I S L P N K Q S S F L N K E V F S S E K D I T R Y I  F5
     K H H I N E A Q F D T L K Y I K A P I K F N K Y K L L S F * F I Y A A A P Y I H Q F R F L I K K H P F S I K K W S A R N K T * R E I Y   F4

       * P R * T W Q F R R * T R P V F T C T * E L F K I K W P P N Q L * I I T D I * I L L C T E G I T S Q I * N I T M I L S W G H M G L *  F1
      S N Q G K H G S L E G E Q G Q Y L H A P E N Y L R * N G R R I S S E L * L T Y R Y C F A Q K E * Q V K Y E I L Q * F * V G V I W A C K  F2
     V T K V N M A V * K V N K A S I Y M H L R I I * D K M A A E S A L N Y N * H I D I A L H R R N N K S N M K Y Y N D F E L G S Y G P V S   F3
 401 AGTAACCAAGGTAAACATGGCAGTTTAGAAGGTGAACAAGGCCAGTATTTACATGCACCTGAGAATTATTTAAGATAAAATGGCCGCCGAATCAGCTCTGAATTATAACTGACATATAGATATTGCTTTGCACAGAAGGAATAACAAGTCAAATATGAAATATTACAATGATTTTGAGTTGGGGTCATATGGGCCTGTAA 600
     ----:----|----:----|----:----|----:----|----:----|----:----|----:----|----:----|----:----|----:----|----:----|----:----|----:----|----:----|----:----|----:----|----:----|----:----|----:----|----:----|
 401 TCATTGGTTCCATTTGTACCGTCAAATCTTCCACTTGTTCCGGTCATAAATGTACGTGGACTCTTAATAAATTCTATTTTACCGGCGGCTTAGTCGAGACTTAATATTGACTGTATATCTATAACGAAACGTGTCTTCCTTATTGTTCAGTTTATACTTTATAATGTTACTAAAACTCAACCCCAGTATACCCGGACATT 600
     Y Y G L Y V H C N L L H V L G T N V H V Q S N N L I F H G G F * S Q I I V S M Y I N S Q V S P I V L * I H F I V I I K L Q P * I P R Y   F6
       T V L T F M A T * F T F L A L I * M C R L I I * S L I A A S D A R F * L Q C I S I A K C L L F L L D F I F Y * L S K S N P D Y P G T  F5
      L L W P L C P L K S P S C P W Y K C A G S F * K L Y F P R R I L E S N Y S V Y L Y Q K A C F S Y C T L Y S I N C H N Q T P T M H A Q L  F4

     V V I M S G Q A M D T T S N I R C P R L N C F Y P D Y F * N L E S L H D R H C M L H S F T A A M N R C * V H R * Y S L L Q K N * K L L   F1
      W L S C Q A R L W T L H P I * D V Q D * I V F T L I I F K I W N H Y M I D T A C C T A L Q Q L * T D A R C T D N I H Y Y R K I K S Y  F2
       G Y H V R P G Y G H Y I Q Y K M S K I K L F L P * L F L K S G I I T * * T L H A A Q L Y S S Y E Q M L G A Q I I F I I T E K L K V I  F3
 601 GTGGTTATCATGTCAGGCCAGGCTATGGACACTACATCCAATATAAGATGTCCAAGATTAAATTGTTTTTACCCTGATTATTTTTAAAATCTGGAATCATTACATGATAGACACTGCATGCTGCACAGCTTTACAGCAGCTATGAACAGATGCTAGGTGCACAGATAATATTCATTATTACAGAAAAATTAAAAGTTATT 800
     ----:----|----:----|----:----|----:----|----:----|----:----|----:----|----:----|----:----|----:----|----:----|----:----|----:----|----:----|----:----|----:----|----:----|----:----|----:----|----:----|
 601 CACCAATAGTACAGTCCGGTCCGATACCTGTGATGTAGGTTATATTCTACAGGTTCTAATTTAACAAAAATGGGACTAATAAAAATTTTAGACCTTAGTAATGTACTATCTGTGACGTACGACGTGTCGAAATGTCGTCGATACTTGTCTACGATCCACGTGTCTATTATAAGTAATAATGTCTTTTTAATTTCAATAA 800
       T T I M D P W A I S V V D L I L H G L N F Q K * G S * K * F R S D N C S L C Q M S C L K V A A I F L H * T C L Y Y E N N C F F * F N N  F6
     L P * T L G P * P C * M W Y L I D L I L N N K Q N N K F D P I M V H Y V S C A A C S * L L * S C I S P A C I I N M I V S F N F T I   F5
      H N D H * A L S W S C G I Y S T W S * I T K V R I I K L I Q F * * M I S V A H Q V A K C C S H V S A L H V S L I * * * L F I L L *  F4

      N Y Y F Q E Y T T M L Q I A D Q T S Q V P A C R A I S M E A S P H S E H A A H M E K L R N L C R L C G T K I K G N K Y L C K T L A N K  F1
       I I T F R N T Q P C C K * Q I R P V K F R R A V R L V W K P V H T V N M R H I W K S Y V T C V A Y V V Q K S K A T N I F A K L L Q T  F2
     K L L S G I H N H A A N S R S D Q S S S G V P C D * Y G S Q S T Q * T C G T Y G K V T * L V S L M W Y K N Q R Q Q I S L Q N S C K Q   F3
 801 AAATTATTACTTTCAGGAATACACAACCATGCTGCAAATAGCAGATCAGACCAGTCAAGTTCCGGCGTGCCGTGCGATTAGTATGGAAGCCAGTCCACACAGTGAACATGCGGCACATATGGAAAAGTTACGTAACTTGTGTCGCTTATGTGGTACAAAAATCAAAGGCAACAAATATCTTTGCAAAACTCTTGCAAACA 1000
     ----:----|----:----|----:----|----:----|----:----|----:----|----:----|----:----|----:----|----:----|----:----|----:----|----:----|----:----|----:----|----:----|----:----|----:----|----:----|----:----|
 801 TTTAATAATGAAAGTCCTTATGTGTTGGTACGACGTTTATCGTCTAGTCTGGTCAGTTCAAGGCCGCACGGCACGCTAATCATACCTTCGGTCAGGTGTGTCACTTGTACGCCGTGTATACCTTTTCAATGCATTGAACACAGCGAATACACCATGTTTTTAGTTTCCGTTGTTTATAGAAACGTTTTGAGAACGTTTGT 1000
       F * * K * S Y V V M S C I A S * V L * T G A H R A I L I S A L G C L S C A A C I S F N R L K H R K H P V F I L P L Y R Q L V R A F  F6
      L N N S E P I C L W A A F L L D S W D L E P T G H S * Y P L W D V C H V H P V Y P F T V Y S T D S I H Y L F * L C C I D K C F E Q L C  F5
     * I I V K L F V C G H Q L Y C I L G T L N R R A T R N T H F G T W V T F M R C M H F L * T V Q T A * T T C F D F A V F I K A F S K C V   F4

       I A E V Y N V N I V N D L A D I H P E Y L C G P C R C K M N R K T E G D F L K P Y C F E E H K F T G C M I C N V H R G R P N K S K S  F1
      R L Q R S I T * I * * M T W P I F T Q S T Y V V R V D V K * I V K Q R V I F * N H I V S R N I N L L A V * Y V M Y T E E G Q T R V S Q  F2
     D C R G L * R K Y S E * L G R Y S P R V P M W S V S M * N E S * N R G * F F K T I L F R G T * I Y W L Y D M * C T Q R K A K Q E * V S   F3
1001 AGATTGCAGAGGTCTATAACGTAAATATAGTGAATGACTTGGCCGATATTCACCCAGAGTACCTATGTGGTCCGTGTCGATGTAAAATGAATCGTAAAACAGAGGGTGATTTTTTAAAACCATATTGTTTCGAGGAACATAAATTTACTGGCTGTATGATATGTAATGTACACAGAGGAAGGCCAAACAAGAGTAAGTCA 1200
     ----:----|----:----|----:----|----:----|----:----|----:----|----:----|----:----|----:----|----:----|----:----|----:----|----:----|----:----|----:----|----:----|----:----|----:----|----:----|----:----|
1001 TCTAACGTCTCCAGATATTGCATTTATATCACTTACTGAACCGGCTATAAGTGGGTCTCATGGATACACCAGGCACAGCTACATTTTACTTAGCATTTTGTCTCCCACTAAAAAATTTGGTATAACAAAGCTCCTTGTATTTAAATGACCGACATACTATACATTACATGTGTCTCCTTCCGGTTTGTTCTCATTCAGT 1200
      L I A S T * L T F I T F S K A S I * G S Y R H P G H R H L I F R L V S P S K K F G Y Q K S S C L N V P Q I I H L T C L P L G F L L L D  F6
       S Q L P R Y R L Y L S H S P R Y E G L T G I H D T D I Y F S D Y F L P H N K L V M N N R P V Y I * Q S Y S I Y H V C L F A L C S Y T  F5
     L N C L D I V Y I Y H I V Q G I N V W L V * T T R T S T F H I T F C L T I K * F W I T E L F M F K S A T H Y I Y V S S P W V L T L *   F4

     A C I S R S D K A D A Q S S N D D I A F E K R P C S R H D E S L S L D R I N K R E K S S C S R S V S S D P V N R D K Y K K S S P S T N   F1
      H V F L E V T R L M H N P V M M T L H L K S G P V H V T M N R F H S T V * T S V R S H L V H A V C L L I Q * T V T S I K S R L Q V L I  F2
       M Y F * K * Q G * C T I Q * * * H D I * K A A L F T S R * I A F T R P Y K Q A * E V I L F T P Q C V F * S S K P Q V * K V V S K Y *  F3
1201 GCATGTATTTCTAGAAGTGACAAGGCTGATGCACAATCCAGTAATGATGACATTGCATTTGAAAAGCGGCCCTGTTCACGTCACGATGAATCGCTTTCACTCGACCGTATAAACAAGCGTGAGAAGTCATCTTGTTCACGCAGTGTGTCTTCTGATCCAGTAAACCGTGACAAGTATAAAAAGTCGTCTCCAAGTACTAA 1400
     ----:----|----:----|----:----|----:----|----:----|----:----|----:----|----:----|----:----|----:----|----:----|----:----|----:----|----:----|----:----|----:----|----:----|----:----|----:----|----:----|
1201 CGTACATAAAGATCTTCACTGTTCCGACTACGTGTTAGGTCATTACTACTGTAACGTAAACTTTTCGCCGGGACAAGTGCAGTGCTACTTAGCGAAAGTGAGCTGGCATATTTGTTCGCACTCTTCAGTAGAACAAGTGCGTCACACAGAAGACTAGGTCATTTGGCACTGTTCATATTTTTCAGCAGAGGTTCATGATT 1400
      A H I E L L S L A S A C D L L S S N A M S F R S G Q E R * S S D S E S S R I F L R S F D D Q E R L T D E S G T F R S L Y L F D D G L V L  F6
     L M Y K * F H C P Q H V I W Y H H C Q M Q F A A R N V D R H I A K V R G Y L C A H S T M K N V C H T K Q D L L G H C T Y F T T E L Y *   F5
       C T N R S T V L S I C L G T I I V N C K F L P G T * T V I F R K * E V T Y V L T L L * R T * A T H R R I W Y V T V L I F L R R W T S  F4

       S T C N M S N T A C K D I S V E M D D V S F E K I E V T G T P A E L Y E Q T H L A D I L K C S I C L T V P K E P V Y T V C Q H I F C K  F1
      V H V T C Q T L H V K T F Q L K W M T F H L K K L K L L E R Q Q N C M N K H T L Q I F * N A L F A * P Y Q K S R C I Q F V N T Y F V  F2
     * Y M * H V K H C M * K R H F S * N G * R F I * K N * S Y W N A S R I V * T N T P C R Y F E M L Y L L N R T K R A G V Y S L S T H I L *   F3
1401 TAGTACATGTAACATGTCAAACACTGCATGTAAAGACATTTCAGTTGAAATGGATGACGTTTCATTTGAAAAAATTGAAGTTACTGGAACGCCAGCAGAATTGTATGAACAAACACACCTTGCAGATATTTTGAAATGCTCTATTTGCTTAACCGTACCAAAAGAGCCGGTGTATACAGTTTGTCAACACATATTTTGTA 1600
     ----:----|----:----|----:----|----:----|----:----|----:----|----:----|----:----|----:----|----:----|----:----|----:----|----:----|----:----|----:----|----:----|----:----|----:----|----:----|----:----|
1401 ATCATGTACATTGTACAGTTTGTGACGTACATTTCTGTAAAGTCAACTTTACCTACTGCAAAGTAAACTTTTTTAACTTCAATGACCTTGCGGTCGTCTTAACATACTTGTTTGTGTGGAACGTCTATAAAACTTTACGAGATAAACGAATTGGCATGGTTTTCTCGGCCACATATGTCAAACAGTTGTGTATAAAACAT 1600
       L V H L M D F V A H L S M E T S I S S T E N S F I S T V P V G A S N Y S C V C R A S I K F H E I Q K V T G F S G T Y V T Q * C M N Q  F6
      Y Y M Y C T L C Q M Y L C K L Q F P H R K M Q F F Q L * Q F A L L I T H V F V G Q L Y K S I S * K S L A R V L L A P T Y L K D V C I K Y  F5
     I T C T V H * V S C T F V N * N F H I V N * K F F N F N S S R W C F Q I F L C V K C I N Q F A R N A * G Y W F L R H I C N T L V Y K T   F4

       S C I V A W C T V R Q S C P V C R E C I D V E Q D L L T L P N Q L L V L F D T L T L K K N E R G K S P G H F V E N K V H L Y S V S R  F1
      N R V L L P G V L S G S H A L F V G N V * M * N K I C * H Y Q T S F W Y Y L I P * L * R K M K G V K V L D I L L K I K S I F I L C P G  F2
     I V Y C C L V C Q A V M P C L * G M Y R C R T R F V N T T K P A S G I I * Y L D F E K E K * K G * K S W T F C * K * S P S L F C V Q E   F3
1601 AATCGTGTATTGTTGCCTGGTGTACTGTCAGGCAGTCATGCCCTGTTTGTAGGGAATGTATAGATGTAGAACAAGATTTGTTAACACTACCAAACCAGCTTCTGGTATTATTTGATACCTTGACTTTAAAGAAAAATGAAAGGGGTAAAAGTCCTGGACATTTTGTTGAAAATAAAGTCCATCTTTATTCTGTGTCCAGG 1800
     ----:----|----:----|----:----|----:----|----:----|----:----|----:----|----:----|----:----|----:----|----:----|----:----|----:----|----:----|----:----|----:----|----:----|----:----|----:----|----:----|
1601 TTAGCACATAACAACGGACCACATGACAGTCCGTCAGTACGGGACAAACATCCCTTACATATCTACATCTTGTTCTAAACAATTGTGATGGTTTGGTCGAAGACCATAATAAACTATGGAACTGAAATTTCTTTTTACTTTCCCCATTTTCAGGACCTGTAAAACAACTTTTATTTCAGGTAGAATAAGACACAGGTCC 1800
      L D H I T A Q H V T L C D H G T Q L S H I S T S C S K N V S G F W S R T N N S V K V K F F F S L P L L G P C K T S F L T W K * E T D L  F6
       I T Y Q Q R T Y Q * A T M G Q K Y P I Y L H L V L N T L V V L G A E P I I Q Y R S K L S F H F P Y F D Q V N Q Q F Y L G D K N Q T W  F5
     F R T N N G P T S D P L * A R N T P F T Y I Y F L I Q * C * W V L K Q Y * K I G Q S * L F I F P T F T R S M K N F I F D M K I R H G P   F4

     K Y A K Q Y R L R S V I S D V G K F C E T H K E N Q T D V L F F M L R Q R L E D L N D K R G E A I D S L W A G H F G S L S I D E C L A   F1
      S M Q N N I D C D L * F Q M S V N F V K H T K K T K L M C Y F L C C V A S V * R I * M I R E G K L L I V F G L G I L G V C P L M S V L L  F2
       V C K T I * I A I C D F R C R * I L * N T Q R K P N * C A I F Y A A S A F R G F E * * E R G S Y * * S L G W A F W E S V H * * V S C  F3
1801 AAGTATGCAAAACAATATAGATTGCGATCTGTGATTTCAGATGTCGGTAAATTTTGTGAAACACACAAAGAAAACCAAACTGATGTGCTATTTTTTATGCTGCGTCAGCGTTTAGAGGATTTGAATGATAAGAGAGGGGAAGCTATTGATAGTCTTTGGGCTGGGCATTTTGGGAGTCTGTCCATTGATGAGTGTCTTGC 2000
     ----:----|----:----|----:----|----:----|----:----|----:----|----:----|----:----|----:----|----:----|----:----|----:----|----:----|----:----|----:----|----:----|----:----|----:----|----:----|----:----|
1801 TTCATACGTTTTGTTATATCTAACGCTAGACACTAAAGTCTACAGCCATTTAAAACACTTTGTGTGTTTCTTTTGGTTTGACTACACGATAAAAAATACGACGCAGTCGCAAATCTCCTAAACTTACTTATTCTCTCCCCTTCGATAACTATCAGAAACCCGACCCGTAAAACCCTCAGACAGGTAACTACTCACAGAACG 2000
       F Y A F C Y L N R D T I E S T P L N Q S V C L S F W V S T S N K I S R * R K S S K F S L L P S A I S L R Q A P C K P L R D M S S H R A  F6
      S T H L V I Y I A I Q S K L H R Y K I H F V C L F G F Q H A I K * A A D A N L P N S H Y S L P L * Q Y D K P Q A N Q S D T W Q H T D Q  F5
     L I C F L I S Q S R H N * I D T F K T F C V F F V L S I H * K K H Q D T L T * L I Q I I L S P F S N I T K P S P M K P T Q G N I L T K   F4

       L R V D T L Q S K G H Y R S Q Y Q Y L K Q K G C L P F C T P H A V N N A E K N Y L P Q S V R F S V Q A N F F G H G D L Y H T P L K P K  F1
      * G W T L C K V K V T I G H N I S T * N R K V A C L F V P H M Q L T M L R K I T C H N L L D S L F R Q I S L V T V I S I T P L * N R  F2
     F K G G H F A K * R S L * V I S V P E T E R L L A F L Y P T C S * Q C * E K L L A T I C * I L C S G K F L W S R * S L S H P F E *   F3
2001 TTTAAGGGTGGACACTTTGCAAAGTAAAGGTCACTATAGGTCACAATATCAGTACCTGAAACAGAAAGGTTGCTTGCCTTTTTGTACCCCACATGCAGTTAACAATGCTGAGAAAAATTACTTGCCACAATCTGTTAGATTCTCTGTTCAGGCAAATTTCTTTGGTCACGGTGATCTCTATCACACCCCTTTGAAACCGA 2200
     ----:----|----:----|----:----|----:----|----:----|----:----|----:----|----:----|----:----|----:----|----:----|----:----|----:----|----:----|----:----|----:----|----:----|----:----|----:----|----:----|
2001 AAATTCCCACCTGTGAAACGTTTCATTTCAGTGATATCCAGTGTTATAGTCATGGACTTTGTCTTTCCAACGAACGGAAAAACATGGGGTGTACGTCAATTGTTACGACTCTTTTTAATGAACGGTGTTAGACAATCTAAGAGACAAGTCCGTTTAAAGAAACCAGTGCCACTAGAGATAGTGTGGGGAAACTTTGGCT 2200
       K L T S V K C L L P * * L D C Y * Y R C F P Q K G K Q V G C A T L L A S F F * K G C D T L N E T * A F K K P * P S R * * V G K F G  F6
      K L P P C K A F Y L D S Y T V I D T G S V S L N S A K K Y G V H L * C H Q S F N S A V I Q * I R Q E P L N R Q D R H D R D C G K S V S  F5
     S * P H V S Q L T F T V I P * L I L V Q F L F T A Q R K T G W M C N V I S L F I V Q W L R N S E R N L C I E K T V T I E I V G R Q F R   F4

       E I A L S H E V T N D F K P I N V T S C L S S P E L P F P N L K G V R Y N Y V E A I A K T L E E L D P Y I K D G L V S N N I D P S D  F1
      K K * R F H M K * Q M I S N P S M L P H V C R H Q S Y L F Q I * K V Y A I I M L K L * Q R H L R S * I P I S K M V L C Q I I * I P Q T  F2
     R N S A F T * S D K * F Q T H Q C Y L M S V V T R V T F S K F E R C T L * L C * S Y S K D T * G A R S L Y Q R W S C V K * Y R S L R P   F3
2201 AAGAAATAGCGCTTTCACATGAAGTGACAAATGATTTCAAACCCATCAATGTTACCTCATGTCTGTCGTCACCAGAGTTACCTTTTCCAAATTTGAAAGGTGTACGCTATAATTATGTTGAAGCTATAGCAAAGACACTTGAGGAGCTAGATCCCTATATCAAAGATGGTCTTGTGTCAAATAATATAGATCCCTCAGAC 2400
     ----:----|----:----|----:----|----:----|----:----|----:----|----:----|----:----|----:----|----:----|----:----|----:----|----:----|----:----|----:----|----:----|----:----|----:----|----:----|----:----|
2201 TTCTTTATCGCGAAAGTGTACTTCACTGTTTACTAAAGTTTGGGTAGTTACAATGGAGTACAGACAGCAGTGGTCTCAATGGAAAAGGTTTAAACTTTCCACATGCGATATTAATACAACTTCGATATCGTTTCTGTGAACTCCTCGATCTAGGGATATAGTTTCTACCAGAACACAGTTTATTATATCTAGGGAGTCTG 2400
       F S I A S E C S T V F S K L G M L T V E H R D D G S N G K G F K F P T R * L * T S A I F V S S S S S G * I L S P R T D F L I S G E S  F6
      L F L A K V H L S L H N * V W * H * R M D T T V L T V K E L N S L H V S Y N H Q L * L L S V Q P A L D R Y * L H D Q T L Y Y L D R L  F5
     F F Y R K * M F H C I I E F G D I N G * T Q R * W L * R K W I Q F T Y A I I I N F S Y C L C K L L * I G I D F I T K H * I I Y G * V   F4
```

```
      P S L L L K T Y I K D G A D G M G E V S V Y W E K S D C S L P D K A F R F A F A V V K V T T V A S D G V E I V I F E E E K P N S I R T   F1
      H L C F * K P T L K M V Q T E W E K Y R F T G R K V I V V S Q I K H F D L L S Q L L K S Q Q W L L M V L K * * Y L K K K S Q I P F V L   F2
       I F A F K N L H * R W C R R N G R S I G L L G E K * L * S P R * S I S I C F R S C * S H N S G F * W C * N S D I * R R K A K F H S Y    F3
2401 CCATCTTTGCTTTTAAAAACCTACATTAAAGATGGTGCAGACGGAATGGGAGAAGTATCGGTTTACTGGGAGAAAAGTGATTGTAGTCTCCCAGATAAAGCATTTCGATTTGCTTTCGCAGTTGTTAAAGTCACAACAGTGGCTTCTGATGGTGTTGAAATAGTGATATTTGAAGAAGAAAAGCCAAATTCCATTCGTAC 2600
     ----:----|----:----|----:----|----:----|----:----|----:----|----:----|----:----|----:----|----:----|----:----|----:----|----:----|----:----|----:----|----:----|----:----|----:----|----:----|----:----|
2401 GGTAGAAACGAAAATTTTTGGATGTAATTTCTACCACGTCTGCCTTACCCTCTTCATAGCCAAATGACCCTCTTTTCACTAACATCAGAGGGTCTATTTCGTAAAGCTAAACGAAAGCGTCAACAATTTCAGTGTTGTCACCGAAGACTACCACAACTTTATCACTATAAACTTCTTCTTTTCGGTTTAAGGTAAGCATG 2600
      G D K S K F V * M L S P A S P I P S T D T * Q S F L S Q L R G S L A N R N A K A T T L T V V T A E S P T S I T I N S S S F G F E M R V   F6
     G M K A K L F R C * L H H L R F P L L I P K S P S F H N Y D G L Y L M E I Q K R L Q * L * L L P K Q H H Q F L S I Q L L F A L N W E Y   F5
       W R Q K * F G V N F I T C V S H S F Y R N V P L F T I T T E W I F C K S K S E C N N F D C C H S R I T N F Y H Y K F F F L W I G N T    F4

       N R P L L E A I A D E N H R S S S V F C M A P I E T E R S F L K D K I L K V H M A D G C R R H E L K F Y T S M I D E K F D R A E S G L   F1
       I D P Y L K L L Q M K I I G L Q V S S A W L Q L K L S A V S * R I K Y * R F T W Q M V V V G M N L N S I H L * * M K N L T V L N Q G    F2
      * S T L T * S Y C R * K S * V F K C L L H G S N * N * A Q F P K G * N T E G S H G R W L S * A * T * I L Y I Y D R * K I * P C * I R A   F3
2601 TAATCGACCCTTACTTGAAGCTATTGCAGATGAAAATCATAGGTCTTCAAGTGTCTTCTGCATGGCTCCAATTGAAACTGAGCGCAGTTTCCTAAAGGATAAAATACTGAAGGTTCACATGGCAGATGGTTGTCGTAGGCATGAACTTAAATTCTATACATCTATGATAGATGAAAAATTTGACCGTGCTGAATCAGGGC 2800
     ----:----|----:----|----:----|----:----|----:----|----:----|----:----|----:----|----:----|----:----|----:----|----:----|----:----|----:----|----:----|----:----|----:----|----:----|----:----|----:----|
2601 ATTAGCTGGGAATGAACTTCGATAACGTCTACTTTTAGTATCCAGAAGTTCACAGAAGACGTACCGAGGTTAACTTTGACTCGCGTCAAAGGATTTCCTATTTTATGACTTCCAAGTGTACCGTCTACCAACAGCATCCGTACTTGAATTTAAGATATGTAGATACTATCTACTTTTTAAACTGGCACGACTTAGTCCCG 2800
       L R G K S S A I A S S F * L D E L T K Q M A G I S V S R L K R F S L I S F T * M A S P Q R L C S S L N * V D I I S S F N S R A S D P    F6
      * D V R V Q L * Q L H F D Y T K L H R R C P E L Q F Q A C N G L P Y F V S P E C P L H N D Y A H V * I R Y M * S L H F I Q G H Q I L A   F5
      S I S G * K F S N C I F I M P R * T D E A H S W N F S L A T E * L I F Y Q L N V H C I T T T P M F K F E I C R H Y I F F K V T S F * P   F4

       E G S G G S R Y L C T L C T A T R E E A K S A L G T F S I T R T F A E T K S V A E Y I K V N P D K L S Q T Q L D K L S Y G V K S V P I   F1
      L R G V G R V I C V H C V Q P Q G R R Q S L P L E P L V L H V L L Q R Q S L * L N I S R * I L I N L V K H N * I N C H M V * K V Y Q F   F2
      * G E W V A L F V Y T V Y S H K G G G K V C P W N L * Y Y T Y F C R D K V C S * I Y Q G E S * * T * S N T I R * T V I W C K K C T N S   F3
2801 TTGAGGGGAGTGGGTCGCGTTATTTGTGTACACTGTGTACAGCCACAAGGGAGGAGGCAAAGTCTGCCCTTGGAACCTTTAGTATTACACGTACTTTTGCAGAGACAAAGTCTGTAGCTGAATATATCAAGGTGAATCCTGATAAACTTAGTCAAACACAATTAGATAAACTGTCATATGGTGTAAAAAGTGTACCAATT 3000
     ----:----|----:----|----:----|----:----|----:----|----:----|----:----|----:----|----:----|----:----|----:----|----:----|----:----|----:----|----:----|----:----|----:----|----:----|----:----|----:----|
2801 AACTCCCCTCACCCAGCGCAATAAACACATGTGACACATGTCGGTGTTCCCTCCTCCGTTTCAGACGGGAACCTTGGAAATCATAATGTGCATGAAAACGTCTCTGTTTCAGACATCGACTTATATAGTTCCACTTAGGACTATTTGAATCAGTTTGTGTTAATCTATTTGACAGTATACCACATTTTTCACATGGTTAA 3000
      S S P L P D R * K H V S H V A V L S S A F D A R P V K L I V R V K A S V F D T A S Y I L T F G S L S L * V C N S L S D Y P T F L T G I   F6
       Q P S H T A N N T Y V T Y L W L P P P L T Q G Q F R * Y * V Y K Q L S L T Q L Q I Y * P S D Q Y V * D F V I L Y V T M H H L F H V L    F5
       K L P T P R T I Q T C Q T C G C P L L C L R G K S G K T N C T S K C L C L R Y S F I D L H I R I F K T L C L * I F Q * I T Y F T Y W N   F4

      L T A D A I E K G I D A T H S D I N M A L F F K K V I L R E I A G V S V W E L N Q D I K P L I C E A E T K F D K V M R S T T G I H P A   F1
      L L L M Q * R R G * M Q H T L T * I W H C F S K K * Y * G K L Q V * V F G S * I K T S N L L F A R Q R Q N L I R S * G Q L L A F I Q H   F2
       Y C * C N R E G D R C N T L * H K Y G T V F Q K S N I K G N C R C K C L G V K S R H Q T S Y L R G R D K I * * G H E V N Y W H S S S    F3
3001 CTTACTGCTGATGCAATAGAGAAGGGGATAGATGCAACACACTCTGACATAAATATGGCACTGTTTTTCAAAAAAGTAATATTAAGGGAAATTGCAGGTGTAAGTGTTTGGGAGTTAAATCAAGACATCAAACCTCTTATTTGCGAGGCAGAGACAAAATTTGATAAGGTCATGAGGTCAACTACTGGCATTCATCCAGC 3200
     ----:----|----:----|----:----|----:----|----:----|----:----|----:----|----:----|----:----|----:----|----:----|----:----|----:----|----:----|----:----|----:----|----:----|----:----|----:----|----:----|
3001 GAATGACGACTACGTTATCTCTTCCCCTATCTACGTTGTGTGAGACTGTATTTATACCGTGACAAAAAGTTTTTCATTATAATTCCCTTTAACGTCCACATTCACAAACCCTCAATTTAGTTCTGTAGTTTGGAGAATAAACGCTCCGTCTCTGTTTTAAACTATTCCAGTACTCCAGTTGATGACCGTAAGTAGGTCG 3200
      R V A S A I S F P I S A V C E S M F I A S N K L F T I N L S I A P T L T Q S N F * S M L G R I Q S A S V F N S L T M L D V V P M * G A   F6
      E * Q Q H L L S P S L H L V S Q C L Y P V T K * F L L I L P F Q L H L H K P T L D L C * V E * K R P L S L I Q Y P * S T L * Q C E D L   F5
       K S S I C Y L L P Y I C C V R V Y I H C Q K E F F Y Y * P F N C T Y T N P L * I L V D F R K N A L C L C F K I L D H P * S S A N M W    F4

       L M M P G N Y A R I M F D D K N E K H V T C L I T C Q V R R S L M N E L L S L F R K L R K I Y R S R S P K V D Y P D E V K A Y K K I A   F1
       L * C L A T M P E L C L M I K M K N M S H V S L H A R L E E V L * M S F F L F S E S * G K Y T G Q G H R K * I T Q T R S R L T R K L    F2
      T Y D A W Q L C Q N Y V * * * K * K T C H M S H Y M P G * K K S Y E * A S F S F Q K V E E N I Q V K V T E S R L P R R G Q G L Q E N C   F3
3201 ACTTATGATGCCTGGCAACTATGCCAGAATTATGTTTGATGATAAAAATGAAAAACATGTCACATGTCTCATTACATGCCAGGTTAGAAGAAGTCTTATGAATGAGCTTCTTTCTCTTTTCAGAAAGTTGAGGAAAATATACAGGTCAAGGTCACCGAAAGTAGATTACCCAGACGAGGTCAAGGCTTACAAGAAAATTG 3400
     ----:----|----:----|----:----|----:----|----:----|----:----|----:----|----:----|----:----|----:----|----:----|----:----|----:----|----:----|----:----|----:----|----:----|----:----|----:----|----:----|
3201 TGAATACTACGGACCGTTGATACGGTCTTAATACAAACTACTATTTTTACTTTTTGTACAGTGTACAGAGTAATGTACGGTCCAATCTTCTTCAGAATACTTACTCGAAGAAAGAGAAAAGTCTTTCAACTCCTTTTATATGTCCAGTTCCAGTGGCTTTCATCTAATGGGTCTGCTCCAGTTCCGAATGTTCTTTTAAC 3400
       S I I G P L * A L I I N S S L F S F C T V H R M V H W T L L L R I F S S R E R K L F N L F I Y L D L D G F T S * G S S T L A * L F I    F6
      V * S A Q C S H W F * T Q H Y F H F V H * M D * * M G P * F F D * S H A E K E K * F T S S F I C T L T V S L L N G L R P * P K C S F Q   F5
      C K H H R A V I G S N H K I I F I F F M D C T E N C A L N S S T K H I L K K R K E S L Q P F Y V P * P * R F Y I V W V L D L S V L F N   F4

       V S M G Q F L L Q N Y S Y I E W P N Y L H K I I E H V Q E L I E S V D G P G C I G S F S G E G N E G G N K V F R F F R K N L S R K G   F1
      Q F Q W V S F C C K I I L I L S G Q I I C I R * * N M F K N S S R V * M A Q V A L E V S L V R E M K G G I K F F A F L E K I S L E R G   F2
      S F N G S V F V A K L F L Y * V A K L S A * D N R T C S R T H R E C R W P R L H W K F L W * G K * R G E * S F S L F * K K S L * K G G   F3
3401 CAGTTTCAATGGGTCAGTTTTTGTTGCAAAATTATTCTTATATTGAGTGGCCAAATTATCTGCATAAGATAATAGAACATGTTCAAGAACTCATCGAGAGTGTAGATGGCCCAGGTTGCATTGGAAGTTTCTCTGGTGAGGGAAATGAAGGGGGGAATAAAGTTTTCGCTTTTTAGAAAAAATCTCTCTAGAAAGGGG 3600
     ----:----|----:----|----:----|----:----|----:----|----:----|----:----|----:----|----:----|----:----|----:----|----:----|----:----|----:----|----:----|----:----|----:----|----:----|----:----|----:----|
3401 GTCAAAGTTACCCAGTCAAAAACAACGTTTTAATAAGAATATAACTCACCGGTTTAATAGACGTATTCTATTATCTTGTACAAGTTCTTGAGTAGCTCTCACATCTACCGGGTCCAACGTAACCTTCAAAGAGACCACTCCCTTTACTTCCCCCCTTATTTCAAAAGCGAAAAATCTTTTTAGAGAGATCTTTCCCC 3600
      A T E I P * N K N C F * E * I S H G F * R C L I I S C T * S S M S L T S P G P Q M P L K E P S P F S P P F L T K R K K L F F R E L F P   F6
       L K L P D T K T A F N N K Y Q T A L N D A Y S L L V H E L V * R S H L H G L N C Q F N R Q H P F H L P S Y L K E S K * F F D R * F P    F5
      C N * H T L K Q Q L I I R I N L P W I I Q M L Y Y F M N L F E D L T Y I A W T A N S T E R T L S I F P P I F N K A K K S F I E R S L P   F4

     D V I G G L Q D V L Q L Q W L Y S S K K L Q A L S N A T Q K Q N R C S F C F E L G H K R P S C P N K E K S D D F V E * F I C L C I K C   F1
      M L L G V F K M Y Y N Y N G C T V V K S Y R L C Q M L H R S K I D A L S V L N L A T K D L H V P I K R N Q M I L L N D L Y V Y A * N V   F2
      C Y W G S S R C I T I T M A V Q * * K A T G F V K C Y T E A K * M L F L F * T W P Q K T F M S Q * R E I R * F C * M I Y M F M H K M   F3
3601 GATGTTATTGGGGGTCTTCAAGATGTATTACAATTACAATGGCTGTACAGTAGTAAAAAGCTACAGGCTTTGTCAAATGCTACACAGAAGCAAAATAGATGCTCTTTCTGTTTTGAACTTGGCCACAAAAGACCTTCATGTCCCAATAAAGAGAAATCAGATGATTTTGTTGAATGATTTATATGTTTATGCATAAAATG 3800
     ----:----|----:----|----:----|----:----|----:----|----:----|----:----|----:----|----:----|----:----|----:----|----:----|----:----|----:----|----:----|----:----|----:----|----:----|----:----|----:----|
3601 CTACAATAACCCCCAGAAGTTCTACATAATGTTAATGTTACCGACATGTCATCATTTTTCGATGTCCGAAACAGTTTACGATGTGTCTTCGTTTTATCTACGAGAAAGACAAAACTTGAACCGGTGTTTTCTGGAAGTACAGGGTTATTTCTCTTTAGTCTACTAAAACAACTTACTAAATATACAAATACGTATTTTAC 3800
      S T I P P R * S T N C N C H S Y L L L F S C A K D F A V C F C F L H E K Q K S S P W L L G E H G L L S F D S S K T S H N I H K H M F H   F6
      P H * Q P D E L H I V I V I A T C Y Y F A V P K T L H * V S A F Y I S K R N Q V Q G C F V K M D W Y L S I L H N Q Q I I * I N I C L I   F5
       I N N P T K L I Y * L * L P Q V T T F L * L S Q * I S C L L L I S A R E T K F K A V F S R * T G I F L F * I I K N F S K Y T * A Y F    F4

       Q I K S C N D L V * S * N E S N K I M K L S F F I M K C I T V D P V K * N K S H N D R M K C N Y F F L E Q S L H K Y F P F G I R F R P   F1
       R * N H V T I L F D H E M N Q I K S * N C P F L S * N A * L L I L S N E I K V T M I E * N V I I S S W S R V C T N T F L L V F A L D    F2
      S D K I M * R S C L I M K * I K * N H E T V L F Y H E M H N C * S C Q M K * K S Q * * N E M * L F L L G A E F A Q I L S F W Y S L * T   F3
3801 TCAGATAAAATCATGTAACGATCTTGTTTGATCATGAAATGAATCAAATAAAATCATGAAACTGTCCTTTTTTATCATGAAATGCATAACTGTTGATCCTGTCAAATGAAATAAAAGTCACAATGATAGAATGAAATGTAATTATTTCTTCTTGGAGCAGAGTTTGCACAAATACTTTCCTTTTGGTATTCGCTTTAGAC 4000
     ----:----|----:----|----:----|----:----|----:----|----:----|----:----|----:----|----:----|----:----|----:----|----:----|----:----|----:----|----:----|----:----|----:----|----:----|----:----|----:----|
3801 AGTCTATTTTAGTACATTGCTAGAACAAACTAGTACTTTACTTAGTTTATTTTAGTACTTTGACAGGAAAAAATAGTACTTTACGTATTGACAACTAGGACAGTTTACTTTATTTTCAGTGTTACTATCTTACTTTACATTAATAAAGAAGAACCTCGTCTCAAACGTGTTTATGAAAGGAAAACCATAAGCGAAATCTG 4000
       * I F D H L S R T Q D H F S D F L I M F S D K K I M F H M V T S G T L H F L L * L S L I F H L * K K K S C L K C L Y K G K P I R K L   F6
       D S L I M Y R D Q K I M F H I L Y F * S V T R K * * S I C L Q Q D Q * I F Y F D C H Y F S I Y N N R R P A S N A C I S E K Q Y E S * V   F5
      T L Y F * T V I K N S * S I F * I F D H F Q G K K D H F A Y S N I R D F S I F T V I I S H F T I I E E Q L L T Q V F V K R K T N A K S   F4

       V Q K K W C H C L H P P S H * T H C I G E V S G V S F I W H I P C W A K S H G P G T N G L L V Y T * Y F * K H P L T I S R V W G S T   F1
      Q Y R K N G A T V Y T H L H T E P I V L V K S L G C L L F G T S H V G P N H M V Q A Q M V Y L C T H S T S E N I H S L * V E S G V Q H   F2
      S T E K M V P L F T P T F T L N P L Y W * S L W G V F Y L A H P M L G Q I T W S R H K W F T C V H I V L L K T S T H Y K * S L G F N I   F3
4001 CAGTACAGAAAAAAATGGTGCCACTGTTTACACCCACCTTCACACTGAACCCATTGTATTGGTGAAGTCTCTGGGGTGTCTTTTATTTGGCACATCCCATGTTGGGCCAAATCACATGGTCCAGGCACAAATGGTTTACTTGTGTACACATAGTACTTCTGAAAACATCCACTCACTATAAGTAGAGTCTGGGGTTCAACA 4200
     ----:----|----:----|----:----|----:----|----:----|----:----|----:----|----:----|----:----|----:----|----:----|----:----|----:----|----:----|----:----|----:----|----:----|----:----|----:----|----:----|
4001 GTCATGTCTTTTTTTACCACGGTGACAAATGTGGGTGGAAGTGTGACTTGGGTAACATAACCACTTCAGAGACCCCACAGAAAATAAACCGTGTAGGGTACAACCCGGTTTAGTGTACCAGGTCCGTGTTTACCAAATGAACACATGTGTATCATGAAGACTTTTGTAGGTGAGTGATATTCATCTCAGACCCCAAGTTGT 4200
      G T C F F H H W Q K C G G E C Q V W Q I P S T E P T D K I Q C M G H Q A L D C P G P V F P K S T Y V Y Y K Q F C G S V I L L T Q P E V   F6
       L V S F I T G S N V G V K V S F G N Y Q H L R Q P T K * K A C G M N P W I V H D L C L H N V Q T C M T S R F V D V * * L Y L R P N L    F5
       W Y L F F P A V T * V W R * V S G M T N T F D R P H R K N P V D W T P G F * M T W A C I T * K H V C L V E S F M W E S Y T S D P T * C   F4

      F S S D P P H D K * S E P A S H S D D L H V K K S S L K S L H D T I L S * S G S S S S L D C K * P P N M Y R I P S T T Q E L W N D I N   F1
      S A V T H H M T S S R N L H H I L M T Y M * K N Q V * R V C M I Q F C P D L D Q V P L W T A S N P Q I C T E F H R L H R N F G M I * M   F2
       Q Q * P T T * Q V V G T C I T F * * L T C K K I K F K E F A * Y N F V L I W I K F L F G L Q V T P K Y V Q N S I D Y T G T L E * Y K    F3
4201 TTCAGCAGTGACCCACCACATGACAAGTAGTCGGAACCTGCATCACATTCTGATGACTTACATGTAAAAAAATCAAGTTTAAAGAGTTTGCATGATACAATTTTGTCCTGATCTGGATCAAGTTCCTCTTTGGACTGCAAGTAACCCCCAAATATGTACAGAATTCCATCGACTACACAGGAACTTTGGAATGATATAAA 4400
     ----:----|----:----|----:----|----:----|----:----|----:----|----:----|----:----|----:----|----:----|----:----|----:----|----:----|----:----|----:----|----:----|----:----|----:----|----:----|----:----|
4201 AAGTCGTCACTGGGTGGTGTACTGTTCATCAGCCTTGGACGTAGTGTAAGACTACTGAATGTACATTTTTTTAGTTCAAATTTCTCAAACGTACTATGTTAAAACAGGACTAGACCTAGTTCAAGGAGAAACCTGACGTTCATTGGGGGTTTATACATGTCTTAAGGTAGCTGATGTGTCCTTGAAACCTTACTATATTT 4400
       N L L S G G C S L Y D S G A D C E S S K C T F F D L K F L K C S V I K D Q D P D L E E K S Q L Y G G F I Y L I G D V V C S S Q F S I F   F6
      M * C H G V V H C T T P V Q M V N Q H S V H L F I L N L S N A H Y L K T R I Q I L N R K P S C T V G L Y T C F E M S * V P V K S H Y L   F5
       E A T V W W M V L L R F R C * M R I V * M Y F F * T * L T Q M I C N Q G S R S * T G R Q V A L L G W I H V S N W R S C L F K P I I Y    F4

       V G C M S N S T L Y I * * S F V * * F N M Y R T V T S C K G S L C E A V F F V * A N P P T M A N L S S D M K S A V W K H L G L T E G T   F1
       L A A C Q T V H C T F D N H L F D S L I C T E Q L H H A R E V C V K Q C F L C K L T L Q L W L I Y H L I * N L Q C G N T * V * Q R G    F2
      C W L H V K Q Y T V H L I I I C L I V * Y V Q N S Y I M Q G K S V * S S V F C V S * P S N Y G * F I I * Y E I C S V E T P R F D R G D   F3
4401 TGTTGGCTGCATGTCAAACAGTACACTGTACATTTGATAATCATTTGTTTGATAGTTTAATATGTACAGAACAGTTACATCATGCAAGGGAAGTCTGTGTGAAGCAGTGTTTTTTTGTGTAAGCTAACCCTCCAACTATGGCTAATTTATCATCTGATATGAAATCTGCAGTGTGGAAACACCTAGGTTTGACAGAGGGGA 4600
     ----:----|----:----|----:----|----:----|----:----|----:----|----:----|----:----|----:----|----:----|----:----|----:----|----:----|----:----|----:----|----:----|----:----|----:----|----:----|----:----|
4401 ACAACCGACGTACAGTTTGTCATGTGACATGTAAACTATTAGTAAACAAACTATCAAATTATACATGTCTTGTCAATGTAGTACGTTCCCTTCAGACACACTTCGTCACAAAAAAACACATTCGATTGGGAGGTTGATACCGATTAAATAGTAGACTATACTTTAGACGTCACACCTTTGTGGATCCAAACTGTCTCCCCT 4600
       T P Q M D F L V S Y M Q Y D N T Q Y N L I Y L V T V D H L P L R H S A T N K T Y A L G G V I A L K D D S I F D A T H F C R P K V S P   F6
      H Q S C T L C Y V T C K I I M Q K I T * Y T C F L * M M C P F D T H L L T K Q T L * G E L * P * N I M Q Y S I Q L T S V G L N S L P S   F5
      I N A A H * V T C Q V N S L * K N S L K I H V S C N C * A L S T Q T F C H K K H L S V R W S H S I * * R I H F R C H P F V * T Q C L P   F4

       G S T L H S I D S P L I F N I * N L P S T Q T C A N G S A K A I S F S R F G I S Y P P N M I A T F A F P G S L L R E C P A L A V G L   F1
      L E V H S I P L I L H * S L T Y E T C H P H R L V Q M V L Q R P F P F H G S A F H I H Q T * L Q R L L S Q A V Y * E N A Q L L Q L D C   F2
      W K Y T P F H * F S I N L * H M K P A I H T D L C K W F C K G H F L F T V R H F I S T K H D C N V C F P R Q F I K R M P S S C S W T V   F3
4601 CTGGAAGTACACTCCATTCCATTGATTCTCCATTAATCTTTAACATATGAAACCTGCCATCCACACAGACTTGTGCAAATGGTTCTGCAAAGGCCATTTCCTTTTCACGGTTCGGCATTTCATATCCACCAAACATGATTGCAACGTTTGCTTTCCCAGGCAGTTTATTAAGAGAATGCCCAGCTCTTGCAGTTGGACTG 4800
     ----:----|----:----|----:----|----:----|----:----|----:----|----:----|----:----|----:----|----:----|----:----|----:----|----:----|----:----|----:----|----:----|----:----|----:----|----:----|----:----|
4601 GACCTTCATGTGAGGTAAGGTAACTAAGAGGTAATTAGAAATTGTATACTTTGGACGGTAGGTGTGTCTGAACACGTTTACCAAGACGTTTCCGGTAAAGGAAAAGTGCCAAGCCGTAAAGTATAGGTGGTTTGTACTAACGTTGCAAACGAAAGGGTCCGTCAAATAATTCTCTTACGGGTCGAGAACGTCAACCTGAC 4800
      V P L V S W E M S E G N I K L M H F R G D V C V Q A F P E A F A M E K E R N P M E Y G G F M I A V N A K G P L K N L S H G A R A T P S   F6
       Q F Y V G N W Q N E M L R * C I F G A M W V S K H L H N Q L P W K R K V T R C K M D V L C S Q L T Q K G L C N I L L I G L E Q L Q V    F5
       S S T C E M G N I R W * D K V Y S V Q W G C L S T C I T R C L G N G K * P E A N * I W W V H N C R K S E W A T * * S F A W S K C N S Q   F4

      F V C I F P W S P Y W P K D F A L P V Y C V I S K I C F F L S T T G L Q M Y K S S T T V * L R T S S P P H I T K Y F P G S A P H T I P   F1
       L S V F S H G P H T G Q R I L H C L C T V * Y Q K F A S F S L Q L V Y R C T N R L Q Q C N * E R Q V H P I * Q N T S L A P L P T P Y P   F2
       C L Y F P M V P I L A K G F C T A C V L C D I K N L L L S L Y N W F T D V Q I V Y N S V I E N V K S T P Y N K I L P W L R S P H H T    F3
4801 TTTGTCTGTATTTTCCCATGGTCCCCATACTGGCCAAAGGATTTTGCACTGCCTGTGTACTGTGTGATATCAAAAATTTGCTTCTTTCTCTCTACAACTGGTTTACAGATGTACAAATCGTCTACAACAGTGTAATTGAGAACGTCAAGTCCACCCCATATAACAAAATACTTCCCTGGCTCCGCTCCCCACACCATACC 5000
     ----:----|----:----|----:----|----:----|----:----|----:----|----:----|----:----|----:----|----:----|----:----|----:----|----:----|----:----|----:----|----:----|----:----|----:----|----:----|----:----|
4801 AAACAGACATAAAAGGGTACCAGGGGTATGACCGGTTTCCTAAAACGTGACGGACACATGACACACTATAGTTTTTAAACGAAGAAAGAGAGATGTTGACCAAATGTCTACATGTTTAGCAGATGTTGTCACATTAACTCTTGCAGTTCAGGTGGGGTATATTGTTTTATGAAGGGACCGAGGCGAGGGGTGTGGTATGG 5000
       N T Q I K G H D G Y Q G F S K A S G T Y Q T I D F I Q K K R E V V P K C I Y L D D V V T Y N L V D L G G W I V F Y K G P E A G W V M G   F6
      T Q R Y K G M T G M S A L P N Q V A Q T S H S I L F K S R E R * L Q N V S T C I T * L L T I S F T L D V G Y L L I S G Q S R E G C W V   F5
       K D T N E W P G W V P W L I K C Q R H V T H Y * F N A E K E R C S T * L H V F R R C C H L Q S R * T W G M Y C F V E R A G S G V G Y    F4
```

```
       D  E  V  K  V  G  I  I  E  P  F  V  T  T  F  S  I  D  S  I  Y  I  S  S  S  V  E  T  I  F  I  F  I  I  F  E  E  S  T  Q  A  A  S  S  P  R  L  T  P  P  K  K  *  Y  S  E  T  S  S  W  C  N  S  I  Y  L  C   F1
        M  R  S  R  L  E  S  L  N  L  L  L  Q  H  F  Q  L  T  R  Y  I  Y  H  H  L  L  K  R  S  L  F  S  *  F  L  K  N  Q  P  K  Q  H  L  L  H  V  *  L  H  P  R  N  N  I  Q  K  H  H  L  G  A  I  Q  Y  I  C     F2
      R  *  G  Q  G  W  N  H  *  T  F  C  Y  N  I  F  N  *  L  D  I  Y  I  I  I  C  *  N  D  L  Y  F  H  N  F  *  R  I  N  P  S  S  I  F  S  T  S  N  S  T  Q  E  I  I  F  R  N  I  I  L  V  Q  F  N  I  F  V    F3
 5001 CGATGAGGTCAAGGTTGGAATCATTGAACCTTTTGTTACAACATTTTCAATTGACTCGATATATATATCATCATCTGTTGAAACGATCTTTATTTTCATAATTTTTGAAGAATCAACCCAAGCAGCATCTTCTCCACGTCTAACTCCACCCAAGAAATAATATTCAGAAACATCATCTTGGTGCAATTCAATATATTTGT 5200
      ----:----|----:----|----:----|----:----|----:----|----:----|----:----|----:----|----:----|----:----|----:----|----:----|----:----|----:----|----:----|----:----|----:----|----:----|----:----|----:----|
 5001 GCTACTCCAGTTCCAACCTTAGTAACTTGGAAAACAATGTTGTAAAAGTTAACTGAGCTATATATATAGTAGTAGACAACTTTGCTAGAAATAAAAGTATTAAAAACTTCTTAGTTGGGTTCGTCGTAGAAGAGGTGCAGATTGAGGTGGGTTCTTTATTATAAGTCTTTGTAGTAGAACCACGTTAAGTTATATAAACA 5200
        S  S  T  L  T  P  I  M  S  G  K  T  V  V  N  E  I  S  E  I  Y  I  D  D  D  T  S  V  I  K  I  K  M  I  K  S  S  D  V  W  A  A  D  E  G  R  R  V  G  G  L  F  Y  Y  E  S  V  D  D  Q  H  L  E  I  Y  K    F6
       R  H  P  *  P  Q  F  *  Q  V  K  Q  *  L  M  K  L  Q  S  S  I  Y  I  M  M  Q  Q  F  S  R  *  K  *  L  K  Q  L  I  L  G  L  L  M  K  E  V  D  L  E  V  W  S  I  I  N  L  F  M  M  K  T  C  N  L  I  N  T   F5
      G  I  L  D  L  N  S  D  N  F  R  K  N  C  C  K  *  N  V  R  Y  I  Y  *  *  R  N  F  R  D  K  N  E  Y  N  K  F  F  *  G  L  C  C  R  R  W  T  *  S  W  G  L  F  L  I  *  F  C  *  R  P  A  I  *  Y  I  Q     F4

        P  S  P  G  *  K  S  P  F  F  N  S  T  S  D  L  F  L  V  I  F  N  L  L  G  T  E  D  L  K  G  K  N  C  A  S  L  S  F  T  L  C  I  V  L  A  A  I  I  A  S  L  V  I  P  L  N  G  T  G  R  D  R  E  C  A     F1
      A  L  P  Q  D  K  N  P  H  S  L  T  P  P  L  T  S  F  L  L  F  L  I  Y  *  E  Q  R  T  *  R  A  K  I  V  H  R  C  R  L  R  Y  V  L  Y  W  P  P  L  *  R  P  *  *  S  R  *  T  G  Q  D  E  I  A  S  A  Q    F2
       P  F  P  R  I  K  I  P  I  L  *  L  H  L  *  P  L  S  C  Y  F  *  F  I  R  N  R  G  L  E  G  Q  K  L  C  I  A  V  V  Y  A  M  Y  C  T  G  R  H  Y  S  V  L  S  D  P  V  E  R  D  R  T  R  S  R  V  R  K   F3
 5201 GCCCTTCCCCAGGATAAAAATCCCCATTCTTTAACTCCACCTCTGACCTCTTTCTTGTTATTTTTAATTTATTAGGAACAGAGGACTTGAAGGGCAAAAATTGTGCATCGCTGTCGTTTACGCTATGTATTGTACTGGCCGCCATTATAGCGTCCTTAGTGATCCCGTTGAACGGGACAGGACGAGATCGCGAGTGCGCA 5400
      ----:----|----:----|----:----|----:----|----:----|----:----|----:----|----:----|----:----|----:----|----:----|----:----|----:----|----:----|----:----|----:----|----:----|----:----|----:----|----:----|
 5201 CGGGAAGGGGTCCTATTTTTAGGGGTAAGAAATTGAGGTGGAGACTGGAGAAAGAACAATAAAAATTAAATAATCCTTGTCTCCTGAACTTCCCGTTTTTAACACGTAGCGACAGCAAATGCGATACATAACATGACCGGCGGTAATATCGCAGGAATCACTAGGGCAACTTGCCCTGTCCTGCTCTAGCGCTCACGCGT 5400
      H  G  E  G  P  Y  F  D  G  N  K  L  E  V  E  S  R  K  R  T  I  K  L  K  N  P  V  S  S  K  F  P  L  F  Q  A  D  S  D  N  V  S  H  I  T  S  A  A  M  I  A  D  K  T  I  G  N  F  P  V  P  R  S  R  S  H  A    F6
        G  K  G  L  I  F  I  G  M  R  *  S  W  R  Q  G  R  E  Q  *  K  *  N  I  L  F  L  P  S  S  P  C  F  N  H  M  A  T  T  *  A  I  Y  Q  V  P  R  W  *  L  T  R  L  S  G  T  S  R  S  L  V  L  D  R  T  R     F5
       A  R  G  W  S  L  F  G  W  E  K  V  G  G  R  V  E  K  K  N  N  K  I  *  *  S  C  L  V  Q  L  A  F  I  T  C  R  Q  R  K  R  *  T  N  Y  Q  G  G  N  Y  R  G  *  H  D  R  Q  V  P  C  S  S  I  A  L  A  C   F4

      S  A  R  S  D  F  R  F  C  K  D  L  V  *  I  V  R  K  W  *  V  Y                                                                                                                                             F1
       A  R  A  P  I  S  A  F  A  R  T  *  Y  K  *  F  V  S  G  E  Y  X                                                                                                                                            F2
        R  A  L  R  F  P  L  L  Q  G  L  S  I  N  S  S  *  V  V  S  I  X                                                                                                                                           F3
 5401 AGCGCGCGCTCCGATTTCCGCTTTTGCAAGGACTTAGTATAAATAGTTCGTAAGTGGTGAGTATAT 5466
      ----:----|----:----|----:----|----:----|----:----|----:----|----:-
 5401 TCGCGCGCGAGGCTAAAGGCGAAAACGTTCCTGAATCATATTTATCAAGCATTCACCACTCATATA 5466
       L  A  R  E  S  K  R  K  Q  L  S  K  T  Y  I  T  R  L  H  H  T  Y                                                                                                                                            F6
      L  R  A  S  R  N  G  S  K  C  P  S  L  I  F  L  E  Y  T  T  L  I                                                                                                                                             F5
        A  R  A  G  I  E  A  K  A  L  V  *  Y  L  Y  N  T  L  P  S  Y  I                                                                                                                                           F4
```

Pinctada imbricata (Pim)

WGS: NIJJ01042550.1

1939-7401
Pinctada imbricata breed pearl oyster-OUG PIN_chr3_13601, whole genome shotgun sequence

```
     P L * A L T N F L Y * V L A K A E I G A R A C A L T I S S C P V Q R D H * G R Y N C G Q Y N T * R K R Q R C T I F A L Q V L C S * * I   F1
      H C E H L R T F Y T K S L Q K R K S E R A L A H S R S R P V P F N G I T K D A I I A A S T I H S V N D S D A Q F L P F K S S V P N K L  F2
      I V S T Y E L F I L S P C K S G N R S A R L R T H D L V L S R S T G S L R T L * L R P V Q Y I A * T T A M H N F C P S S P L F L I N   F3
   1 CCATTGTGAGCACTTACGAACTTTTTATACTAAGTCCTTGCAAAAGCGGAAATCGGAGCGCGCGCTTGCGCACTCACGATCTCGTCCTGTCCCGTTCAACGGGATCACTAAGGACGCTATAATTGCGGCCAGTACAATACATAGCGTAAACGACAGCGATGCACAATTTTTGCCCTTCAAGTCCTCTGTTCCTAATAAAT 200
     ----:----|----:----|----:----|----:----|----:----|----:----|----:----|----:----|----:----|----:----|----:----|----:----|----:----|----:----|----:----|----:----|----:----|----:----|----:----|----:----|
   1 GGTAACACTCGTGAATGCTTGAAAAATATGATTCAGGAACGTTTTCGCCTTTAGCCTCGCGCGCGAACGCGTGAGTGCTAGAGCAGGACAGGGCAAGTTGCCCTAGTGATTCCTGCGATATTAACGCCGGTCATGTTATGTATCGCATTTGCTGTCGCTACGTGTTAAAAACGGGAAGTTCAGGAGACAAGGATTATTTA 200
      G N H A S V F K K Y * T R A F A S I P A R A Q A S V I E D Q G T * R S * * P R * L Q P W Y L V Y R L R C R H V I K A R * T R Q E * Y I   F6
     X M T L V * S S K I S L G Q L L P F R L A R K R V * S R T R D R E V P D S L V S Y N R G T C Y M A Y V V A I C L K Q G E L G R N R I F   F5
      W Q S C K R V K * V L D K C F R F D S R A S A C E R D R G T G N L P I V L S A I I A A L V I C L T F S L S A C N K G K L D E T G L L   F4

      K N N K K Q V R G G V K E W G F L S W G R A Q I Y * I A P R * C F * I L F L G W S * T W R R C C L G * F F K N Y E N K D R F N R * * Y   F1
       K I T R N R S E V E L K N G D F Y P G E G H K Y I E L H Q D D V S E Y Y F L G G V R R G E D A A W V D S S K I M K I K I V S T D D D  F2
      * K * Q E T G Q R W S * R M G I F I L G K G T N I L N C T K M M F L N I I S W V E L D V E K M L L G L I L Q K L * K * R S F Q Q M M I  F3
 201 TAAAAATAACAAGAAACAGGTCAGAGGTGGAGTTAAAGAATGGGGATTTTTATCCTGGGGAAGGGCACAAATATATTGAATTGCACCAAGATGATGTTTCTGAATATTATTTTCTTGGGTGGAGTTAGACGTGGAGAAGATGCTGCTTGGGTTGATTCTTCAAAAATTATGAAAATAAAGATCGTTTCAACAGATGATGAT 400
     ----:----|----:----|----:----|----:----|----:----|----:----|----:----|----:----|----:----|----:----|----:----|----:----|----:----|----:----|----:----|----:----|----:----|----:----|----:----|----:----|
 201 ATTTTTATTGTTCTTTGTCCAGTCTCCACCTCAATTTCTTACCCCTAAAAATAGGACCCCTTCCCGTGTTTATATAACTTAACGTGGTTCTACTACAAAGACTTATAATAAAGAACCCACCTCAATCTGCACCTCTTCTACGACGAACCCAACTAAGAAGTTTTTAATACTTTTATTTCTAGCAAAGTTGTCTACTACTA 400
      L F L L F C T L P P T L S H P N K D Q P L A C I Y Q I A G L H H K Q I N N R P H L * V H L L H Q K P Q N K L F * S F L S R K L L H H   F6
      * F Y C S V P * L H L * L I P I K I R P F P V F I N F Q V L I I N R F I I E Q T S N S T S F I S S P N I R * F N H F Y L D N * C I I I   F5
     N F I V L F L D S T S N F F P S K * G P S P C L Y I S N C W S S T E S Y * K K P P T L R P S S A A Q T S E E F I I F I F I T E V S S S   F4

      I C R V N * K C F N K R F N D S N L D L I G Y G V G S G A R G V F C Y M G W T * R S Q L H Y C R R F V H L * I N C R E K E A N F * Y   F1
      I Y V E S I E N V L T K G S M I P T L T S S G M V W G A E P G E Y F V I W G G L D V L N Y T I V D D L Y I C K S I V E R K K Q I F D I   F2
      Y M S S Q L K M F * Q K V Q * F Q P * P H R V W C G E R S Q G S I L L Y G V D L T F S I T L L * T I C T S V N Q L * R E R S K F L I S  F3
 401 ATATATGTCGAGTCAATTGAAAATGTTTTAACAAAAGGTTCAATGATTCCAACCTTGACCTCATCGGGTATGGTGTGGGGAGCGGAGCCAGGGGAGTATTTTGTTATATGGGGTGGACTTGACGTTCTCAATTACACTATTGTAGACGATTTGTACATCTGTAAATCAATTGTAGAGAGAAAGAAGCAAATTTTTGATAT 600
     ----:----|----:----|----:----|----:----|----:----|----:----|----:----|----:----|----:----|----:----|----:----|----:----|----:----|----:----|----:----|----:----|----:----|----:----|----:----|----:----|
 401 TATATACAGCTCAGTTAACTTTTACAAAATTGTTTTCCAAGTTACTAAGGTTGGAACTGGAGTAGCCCATACCACACCCCTCGCCTCGGTCCCCTCATAAAACAATATACCCCACCTGAACTGCAAGAGTTAATGTGATAACATCTGCTAAACATGTAGACATTTAGTTAACATCTCTCTTTCTTCGTTTAAAAACTATA 600
     Y I H R T L Q F H K L L L N L S E L R S R M P Y P T P L P A L P T N Q * I P H V Q R E * N C * Q L R N T C R Y I L Q L S F S A F K Q Y   F6
      Y I D L * N F I N * C F T * H N W G Q G * R T H H P S R L W P L I K N Y P T S K V N E I V S N Y V I Q V D T F * N Y L S L L L N K I   F5
      I Y T S D I S F T K V F P E I I G V K V E D P I T H P A S G P S Y K T I H P P S S T R L * V I T S S K Y M Q L D I T S L F F C I K S I  F4

     H T V H R Q C K I L W P V W G P W K N T D K Q S N C K R W A F S S * T A W E S K R C N H V W W I * N A E P * K G N G L C R T I C T S L   F1
      T Q Y T G S A K S Y G Q Y G D H G K I Q T N S P T A R G G H S L H K L P G K A N V A I M F G G Y E M P N R E K E M A F A E P F A Q V C  F2
      H S T Q A V Q N P M A S M G T M E K Y R Q T V Q L Q E V G I L F I N C L G K Q T L Q S C L V D M K C R T V K R K W P L Q N H L H K S   F3
 601 CACACAGTACACAGGCAGTGCAAAATCCTATGGCCAGTATGGGGACCATGGAAAAATACAGACAAACAGTCCAACTGCAAGAGGTGGGCATTCTCTTCATAAACTGCCTGGGAAAGCAAACGTTGCAATCATGTTTGGTGGATATGAAATGCCGAACCGTGAAAAGGAAATGGCCTTTGCAGAACCATTTGCACAAGTCT 800
     ----:----|----:----|----:----|----:----|----:----|----:----|----:----|----:----|----:----|----:----|----:----|----:----|----:----|----:----|----:----|----:----|----:----|----:----|----:----|----:----|
 601 GTGTGTCATGTGTCCGTCACGTTTTAGGATACCGGTCATACCCCTGGTACCTTTTTATGTCTGTTTGTCAGGTTGACGTTCTCCACCCGTAAGAGAAGTATTTGACGGACCCTTTCGTTTGCAACGTTAGTACAAACCACCTATACTTTACGGCTTGGCACTTTTCCTTTACCGGAAACGTCTTGGTAAACGTGTTCAGA 800
      * V T C L C H L I R H G T H P G H F F V S L C D L Q L L H A N E E Y V A Q S L L R Q L * T Q H I H F A S G H F P F P R Q L V M Q V L R   F6
     D C L V C A T C F G I A L I P V M S F Y L C V T W S C S T P M R K M F Q R P F C V N C D H K T S I F H R V T F L F H G K C F W K C L D   F5
      V C Y V P L A F D * P W Y P S W P F I C V F L G V A L P P C E R * L S G P F A F T A I M N P P Y S I G F R S F S I A K A S G N A C T   F4

     C G W Q V S Y V K D * W R I N G M E C T S S P L C Q T * V F P H C R F H I R * * I S H S W R V S L H K E H C F T Q T S L A * C N C S V   F1
      V D G R F H M L K I N G E S M E W S V L P V P S V K P R C F H T A D F I S D D K L A I V G G L A Y T K N T A S H R L P L H D V T V L   F2
     V W M A G F I C * R L M E N Q W N G V Y F Q S P L S N L G V S T L Q I S Y Q M I N * P * L E G * L T Q R T L L H T D F P C M M * L F C   F3
 801 GTGTGGATGGCAGGTTTCATATGTTAAAGATTAATGGAGAATCAATGGAATGGAGTGTACTTCCAGTCCCCTCTGTCAAACCTAGGTGTTTCCACACTGCAGATTTCATATCAGATGATAAATTAGCCATAGTTGGAGGGTTAGCTTACACAAAGAACACTGCTTCACACAGACTTCCCTTGCATGATGTAACTGTTCTG 1000
     ----:----|----:----|----:----|----:----|----:----|----:----|----:----|----:----|----:----|----:----|----:----|----:----|----:----|----:----|----:----|----:----|----:----|----:----|----:----|----:----|
 801 CACACCTACCGTCCAAAGTATACAATTTCTAATTACCTCTTAGTTACCTTACCTCACATGAAGGTCAGGGGAGACAGTTTGGATCCACAAAGGTGTGACGTCTAAAGTATAGTCTACTATTTAATCGGTATCAACCTCCCAATCGAATGTGTTTCTTGTGACGAAGTGTGTCTGAAGGGAACGTACTACATTGACAAGAC 1000
      H P H C T E Y T L S * H L I L P I S H V E L G R Q * V * T N G C Q L N * I L H Y I L W L Q L T L K C L S C Q K V C V E R A H H L Q E   F6
      T H I A P K M H * L N I S F * H F P T Y K W D G R D F R P T E V S C I E Y * I I F * G Y N S P * S V C L V S S * V S K G Q M I Y S N Q  F5
     Q T S P L N * I N F I L P S D I S H L T S G T G E T L G L H K W V A S K M D S S L N A M T P P N A * V F F V A E C L S G K C S T V T R   F4

      H I K L S N K * L S N V Q C T V * H A A N I Y I I P R F L C S R W N S V H I W G L L A V Q R G T * S R S G Q N C I M Q T L * T * F F  F1
     Y I L N C Q T N D Y Q M Y S V L F D M Q P T F I S F Q G S C V V D G I L Y I F G G Y L Q S K E E L D P D Q D K T V S C K L F K L D F L   F2
      T Y * T V K Q M I I K C T V Y C L T C S Q H L Y H S K V P V * S M E F C T Y L G V T C S P K R N L I Q I R T K L Y H A N S L N L I F Y  F3
1001 TACATATTAAACTGTCAAACAAATGATTATCAAATGTACAGTGTACTGTTTGACATGCAGCCAACATTTATATCATTCCAAGGTTCCTGTGTAGTCGATGGAATTCTGTACATATTTGGGGGTTACTTGCAGTCCAAAGAGGAACTTGATCCAGATCAGGACAAAACTGTATCATGCAAACTCTTTAAACTTGATTTTT 1200
     ----:----|----:----|----:----|----:----|----:----|----:----|----:----|----:----|----:----|----:----|----:----|----:----|----:----|----:----|----:----|----:----|----:----|----:----|----:----|----:----|
1001 ATGTATAATTTGACAGTTTGTTTACTAATAGTTTACATGTCACATGACAAACTGTACGTCGGTTGTAAATATAGTAAGGTTCCAAGGACACATCAGCTACCTTAAGACATGTATAAACCCCCAATGAACGTCAGGTTTCTCCTTGAACTAGGTCTAGTCCTGTTTTGACATAGTACGTTTGAGAAATTTGAACTAAAAAA 1200
     T C I L S D F L H N D F T C H V T Q C A A L M * I M G L N R H L R H F E T C I Q P N S A T W L P V Q D L D P C F Q I M C V R * V Q N K   F6
      V Y * V T L C I I I L H V T Y Q K V H L W C K Y * E L T G T Y D I S N Q V Y K P T V Q L G F L F K I W I L V F S Y * A F E K F K I K   F5
      Y M N F Q * V F S * * I Y L T S N S M C G V N I D N W P E Q T T S P I R Y M N P P * K C D L S S S S G S * S L V T D H L S K L S S K K  F4

     T C K S S E C D A G S D Y L S C G G S L L N V E P Q T L L I V S G C C Q K Y Y V Y T S K P F V P G P C D L A Q H G M C Q I K A T P E T   F1
     H V S H Q N V M Q V P T T C H V V G H C * M L N P R L Y L * * V D V V R S T M C T Q V N H L C L D H V I W P N M G C A K * K P P Q R L   F2
     M * V I R M * C R F R L L V M W W V T A E C * T P D S T Y S E W M L S E V L C V H K * T I C A W T M * F G P T W D V P N K S H P R D   F3
1201 ACATGTAAGTCATCAGAATGTGATGCAGGTTCCGACTACTTGTCATGTGGTGGGTCACTGCTGAATGTTGAACCCCAGACTCTACTTATAGTGAGTGGATGTTGTCAGAAGTACTATGTGTACACAAGTAAACCATTTGTGCCTGGACCATGTGATTTGGCCCAACATGGGATGTGCCAAATAAAAGCCACCCCAGAGAC 1400
     ----:----|----:----|----:----|----:----|----:----|----:----|----:----|----:----|----:----|----:----|----:----|----:----|----:----|----:----|----:----|----:----|----:----|----:----|----:----|----:----|
1201 TGTACATTCAGTAGTCTTACACTACGTCCAAGGCTGATGAACAGTACACCACCCAGTGACGACTTACAACTTGGGGTCTGAGATGAATATCACTCACCTACAACAGTCTTCATGATACACATGTGTTCATTTGGTAAACACGGACCTGGTACACTAAACCGGGTTGTACCCTACACGGTTTATTTTCGGTGGGGTCTCTG 1400
     V H L D D S H S A P E S * K D H P P D S S F T S G W V R S I T L P H Q * F Y * T Y V L L G N T G P G H S K A W C P I H W I F A V G S V   F6
     * M Y T M L I H H L N R S S T M H H T V A S H Q V G S E V * L S H I N D S T S H T C L Y V M Q A Q V M H N P G V H S T G F L L W G L S   F5
      C T L * * F T I C T G V V Q * T T P * Q Q I N F G L S * K Y H T S T T L L V I H V C T F W K H R S W T I Q G L M P H A L Y F G G W L   F4

     S P I Q W V Q C E G G C K Q W H H F F C T G L K R I P K G K Y V C K L C S K K K * L H F I L S L * L L F H L T G S T V M H F I I K K D   F1
     H Q Y N G F S V K V G V N S G T I F S V L V * S E Y Q K E S M C A N S V P R R N N Y I S F Y H C D F Y F I * Q D Q Q L C I S L * K R   F2
     F T N T M G S V * R W V * T V A P F F L Y W S K A N T K R K V C V Q T L F Q E E I I T F H S I I V T F I S F D R I N S Y A F H Y K K G   F3
1401 TTCACCAATACAATGGGTTCAGTGTGAAGGTGGGTGTAAACAGTGGCACCATTTTTTCTGTACTGGTCTAAAGCGAATACCAAAAGGAAAGTATGTGTGCAAACTCTGTTCCAAGAAGAAATAATTACATTTCATTCTATCATTGTGACTTTTATTTTCATTTGACAGGATCAACAGTTATGCATTTCATTATAAAAAAGG 1600
     ----:----|----:----|----:----|----:----|----:----|----:----|----:----|----:----|----:----|----:----|----:----|----:----|----:----|----:----|----:----|----:----|----:----|----:----|----:----|----:----|
1401 AAGTGGTTATGTTACCCAAGTCACACTTCCACCCACATTTGTCACCGTGGTAAAAAAGACATGACCAGATTTCGCTTATGGTTTTCCTTTCATACACACGTTTGAGACAAGGTTCTTCTTTATTAATGTAAAGTAAGATAGTAACACTGAAAATAAAGTAAACTGTCCTAGTTGTCAATACGTAAAGTAATATTTTTTCC 1600
      E G I C H T * H S P P H L C H C W K K Q V P R F R I G F P F Y T H L S Q E L F F Y N C K M R D N H S K N * K V P D V T I C K M I F F   F6
      K V L V I P E T H L H T Y V T A G N K R Y Q D L A F V L L F T H T C V R N W S S I I V N * E I M T V K I E N S L I L L * A N * * L F P  F5
     S * W Y L P N L T F T P T F L P V M K E T S T * L S Y W F S L I H A F E T G L L F L * M E N * * Q S K * K M Q C S * C N H M E N Y F L   F4

      S F M I L F D S F H D Q T R S V H D F I * H F M H K H I N Y S T K S S G F S L L G H E G L L W P S S K Q K E H L F C F C V A F D K A   F1
     T V S * F Y L I H F M I K Q D R Y M I L S D I L C I N I * I I Q Q N H L V S L Y W D M K V F C G Q V Q N R K S T Y F A S V * H L T K P   F2
     Q F H D F I * F I S * S N K I G T * F Y L T F Y A * T Y K L F N K I I W F L F I G T * R S F V A K F K T E R A P I L L L C S I * Q S L   F3
1601 ACAGTTTCATGATTTTATTTGATTCATTTCATGATCAAACAAGATCGGTACATGATTTTATCTGACATTTTATGCATAAACATATAAATTATTCAACAAAATCATCTGGTTTCTCTTTATTGGGACATGAAGGTCTTTTGTGGCCAAGTTCAAAACAGAAAGAGCACCTATTTTGCTTCTGTGTAGCATTTGACAAAGCC 1800
     ----:----|----:----|----:----|----:----|----:----|----:----|----:----|----:----|----:----|----:----|----:----|----:----|----:----|----:----|----:----|----:----|----:----|----:----|----:----|----:----|
1601 TGTCAAAGTACTAAAATAAACTAAGTAAAGTACTAGTTTGTTCTAGCCATGTACTAAAATAGACTGTAAAATACGTATTTGTATATTTAATAAGTTGTTTTAGTAGACCAAAGAGAAATAACCCTGTACTTCCAGAAAACACCGGTTCAAGTTTTGTCTTTCTCGTGGATAAAACGAAGACACATCGTAAACTGTTTCGG 1800
     S L K M I K N S E N * S * V L D T C S K I Q C K I C L C I F * E V F D D P K E K N P C S P R K H G L E F C F S C R N Q K Q T A N S L A   F6
      C N * S K I Q N M E H D F L I P V H N * R V N * A Y V Y L N N L L I M Q N R K I P V H L D K T A L N L V S L A G I K S R H L M Q C L   F5
      V T E H N * K I * K M I L C S R Y M I K D S M K H M F M Y I I * C F * R T E R * Q S M F T K Q P W T * F L F L V * K A E T Y C K V F G  F4

     C S F L L L Y N Q C N Y N T S * R P Q I T S P F L E R F F L K K R K T L I P P S F P S P E K L P M Q P G P S T L S M S S * T C S I I L   F1
     V A F Y Y C T T S V I I I H L E D P K * H P P F * R D F F * K S E K L * S P L H F P H Q R N F Q C N L G H L H S R * V L E H V L L S Y   F2
      * L F T T V Q P V * L * Y I L K T P N N I P L S R E I F S K K A K N F N P P F I S L T R E T S N A T W A I Y T L D E F L N M F Y Y L  F3
1801 TGTAGCTTTTTACTACTGTACAACCAGTGTAATTATATAATACATCTTGAAGACCCCAAATAACATCCCCCTTTCTAGAGAGATTTTTTCTAAAAAAGCGAAAAACTTTAATCCCCCCTTCATTTCCCTCACCAGAGAAACTTCCAATGCAACCTGGGCCATCTACACTCTCGATGAGTTCTTGAACATGTTCTATTATCTT 2000
     ----:----|----:----|----:----|----:----|----:----|----:----|----:----|----:----|----:----|----:----|----:----|----:----|----:----|----:----|----:----|----:----|----:----|----:----|----:----|----:----|
1801 ACATCGAAAAATGATGACATGTTGGTCACATTAATATTATGTAGAACTTCTGGGGTTTATTGTAGGGGGAAAGATCTCTCTAAAAAAGATTTTTTCGCTTTTTGAAATTAGGGGGGAAGTAAAGGGAGTGGTCTCTTTGAAGGTTACGTTGGACCCGGTAGATGTGAGAGCTACTCAAGAACTTGTACAAGATAATAGAA 2000
      Q L K K S S Y L W H L * L V D Q L G W I V D G K R S L N K R F F R F V K I G G E N G E G S F S G I C G P G D V S E I L E Q V H E I I K  F6
     R Y S K V V T C G T Y N Y Y M K F V G F L M G R E L S I K E L F A F F K L G G K M E R V L S V E L A V Q A M * V R S S N K F M N * * R   F5
      T A K * * Q V V L T I I I C R S S G L Y C G G K * L S K K * F L S F S * D G R * K G * W L F K W H L R P W R C E R H T R S C T R N D  F4

     C R * F G H S I * E * F C N K N * P I E T A T F L * A L T S S G * S T F G D L D L Y I F L N F L K R E R S S F I R L L L T W H V M R H   F1
      A D N L A T Q Y K N N F A T K T D P L K L Q L S C K P * P R L G N L L S V T L T C I F S S T F * R E K E A H S * D F F * P G M * * D  F2
     M Q I I W P L N I R I I L Q Q K L T H * N C N F L V S L D L V W V I Y F R * P * P V Y F P Q L S K E R K K L I H K T S S N L A C N E T   F3
2001 ATGCAGATAATTTGGCCACTCAATATAAGAATAATTTTGCAACAAAAACTGACCCATTGAAACTGCAACTTTCTTGTAAGCCTTGACCTCGTCTGGGTAATCTACTTTCGGTGACCTTGACCTGTATATTTTCCTCAACTTTCTAAAGAGAGAAAGAAGCTCATTCATAAGACTTCTTCTAACCTGGCATGTAATGAGAC 2200
     ----:----|----:----|----:----|----:----|----:----|----:----|----:----|----:----|----:----|----:----|----:----|----:----|----:----|----:----|----:----|----:----|----:----|----:----|----:----|----:----|
2001 TACGTCTATTAAACCGGTGAGTTATATTCTTATTAAAACGTTGTTTTTGACTGGGTAACTTTGACGTTGAAAGAACATTCGGAACTGGAGCAGACCCATTAGATGAAAGCCACTGGAACTGGACATATAAAAGGAGTTGAAAGATTTCTCTCTTTCTTCGAGTAAGTATTCTGAAGAAGATTGGACCGTACATTACTCTG 2200
      H L Y N P W E I Y S Y N Q L L F Q G M S V A V K K Y A K V E D P Y D V K P S R S R Y I K R L K R F L S L L E N M L S R R V Q C T I L   F6
      I C I I Q G S L I L I I K C C F S V W Q F Q L K R T L R S R T Q T I * K R H G Q G T Y K G * S E L S L F F S M * L V E E L R A H L S V  F5
      * A S L K A V * Y L F L K A V F V S G N F S C S E Q L G Q G R R P L R S E T V K V Q I N E E V K * L S F S A * E Y S K K * G P M Y H S  F4

      V T C F S F F S S N I I L A * L P G I I S A G * M P V V D L M T L S N F V S A S Q I R G L M S * F N S Q T L T P A I S L N I T F L K   F1
     M * H V F H F F H Q T * F W H S C Q A S * V L D E C Q * L T S * P Y Q I L S L P R K * E V * C L D L T P K H L H L Q F P L I L L F * K   F2
      C D M F F I F F I K H N S G I V A R H H K C W M N A S S * P H D L I K F C L C L A N K R F D V L I * L P N T Y T C N F P * Y Y F F E K  F3
2201 ATGTGACATGTTTTTCATTTTTTTCATCAAACATAATTCTGGCATAGTTGCCAGGCATCATAAGTGCTGGATGAATGCCAGTAGTTGACCTCATGACCTTATCAAATTTTGTCTCTGCCTCGCAAATAAGAGGTTTGATGTCTTGATTTAACTCCCAAACACTTACACCTGCAATTTCCCTTAATATTACTTTTTTGAAA 2400
     ----:----|----:----|----:----|----:----|----:----|----:----|----:----|----:----|----:----|----:----|----:----|----:----|----:----|----:----|----:----|----:----|----:----|----:----|----:----|----:----|
2201 TACACTGTACAAAAAGTAAAAAAAGTAGTTTGTATTAAGACCGTATCAACGGTCCGTAGTATTCACGACCTACTTACGGTCATCAACTGGAGTACTGGAATAGTTTAAAACAGAGACGGAGCGTTTATTCTCCAAACTACAGAACTAAATTGAGGGTTTGTGAATGTGGACGTTAAAGGGAATTATAATGAAAAAACTTT 2400
      C T V H K E N K E D F M I R A Y N G P M M L A P H I G T T S R M V K D F K T E A E C I L P K I D Q N L E W V S V G A I E R L I V K K F   F6
      H S M N K M K K M L C L E P M T A L C * L H Q I F A L L Q G * S R I L N Q R Q R A F L L N S T K I * S G F V * V Q L K G * Y * K K S   F5
      M H C T K * K K * * V Y N Q C L Q W A D Y T S S S H W Y N V E H G * * I K D R G R L Y S T Q H R S K V G L C K C R C N G K I N S K Q F   F4
```

```
     N S A I F M S E C V A S I P F S I A S A V R I G T L F T P Y D S L S N C V * L S L S G F T L I Y S A T D F V S A K V R V I L K V P R A   F1
      T V P Y L C Q S V L H L S P S L L H Q Q * E L V H F L H H M T V Y L I V F D * V Y Q D S P * Y I Q L Q T L S L Q K Y V * Y * R F Q G Q  F2
      Q C H I Y V R V C C I Y P L L Y C I S S K N W Y T F Y T I * Q F I * L C L T K F I R I H L D I F S Y R L C L C K S T C N T K G S K G   F3
2401 AACAGTGCCATATTTATGTCAGAGTGTGTTGCATCTATCCCCTTCTCTATTGCATCAGCAGTAAGAATTGGTACACTTTTTACACCATATGACAGTTTATCTAATTGTGTTTGACTAAGTTTATCAGGATTCACCTTGATATATTCAGCTACAGACTTTGTCTCTGCAAAAGTACGTGTAATACTAAAGGTTCCAAGGGC 2600
     ----:----|----:----|----:----|----:----|----:----|----:----|----:----|----:----|----:----|----:----|----:----|----:----|----:----|----:----|----:----|----:----|----:----|----:----|----:----|----:----|
2401 TTGTCACGGTATAAATACAGTCTCACACAACGTAGATAGGGGAAGAGATAACGTAGTCGTCATTCTTAACCATGTGAAAAATGTGGTATACTGTCAAATAGATTAACACAAACTGATTCAAATAGTCCTAAGTGGAACTATATAAGTCGATGTCTGAAACAGAGACGTTTTCATGCACATTATGATTTCCAAGGTTCCCG 2600
      F L A M N I D S H T A D I G K E I A D A T L I P V S K V G Y S L K D L Q T Q S L K D P N V K I Y E A V S K T E A F T R T I S F T G L A  F6
     F C H W I * T L T H Q M * G R R * Q M L L L F Q Y V K * V M H C N I * N H K V L N I L I * R S I N L * L S Q R Q L L V H L V L P E L P   F5
      V T G Y K H * L T N C R D G E R N C * C Y S N T C K K C W I V T * R I T N S * T * * S E G Q Y I * S C V K D R C F Y T Y Y * L N W P  F4

      D F A F S L V A V H S V H K * R D P L P P S P D S A R S N F S S I I D V * N L S S C L R Q P S A M * T F S I L S F R K L R S V S I G A  F1
      T L P S P L W L Y T V Y T N N A T H S P Q A L I Q H G Q I F H L S * M Y R I * V H A Y D N H L P C E P S V F Y L L G N C A Q F Q L E  F2
     R L C L L P C G C T Q C T Q I T R P T P P K P * F S T V K F F I Y H R C I E F K F M P T T T I C H V N L Q Y F I F * E T A L S F N W S   F3
2601 AGACTTTGCCTTCTCCCTTGTGGCTGTACACAGTGTACACAAATAACGCGACCCACTCCCCCCAAGCCCTGATTCAGCACGGTCAAATTTTTCATCTATCATAGATGTATAGAATTTAAGTTCATGCCTACGACAACCATCTGCCATGTGAACCTTCAGTATTTTATCTTTTAGGAAACTGCGCTCAGTTTCAATTGGAG 2800
     ----:----|----:----|----:----|----:----|----:----|----:----|----:----|----:----|----:----|----:----|----:----|----:----|----:----|----:----|----:----|----:----|----:----|----:----|----:----|----:----|
2601 TCTGAAACGGAAGAGGGAACACCGACATGTGTCACATGTGTTTATTGCGCTGGGTGAGGGGGGTTCGGGACTAAGTCGTGCCAGTTTAAAAAGTAGATAGTATCTACATATCTTAAATTCAAGTACGGATGCTGTTGGTAGACGGTACACTTGGAAGTCATAAAATAGAAAATCCTTTGACGCGAGTCAAAGTTAACCTC 2800
      S K A K E R T A T C L T C L Y R S G S G G L G S E A R D F K E D I M S T Y F K L E H R R C G D A M H V K L I K D K L F S R E T E I P  F6
      L S Q R R G Q P Q V C H V C I V R G V G G L G Q N L V T L N K M * * L H I S N L N M G V V V M Q W T F R * Y K I K * S V A S L K L Q L  F5
     C V K G E G K H S Y V T Y V F L A V W E G W A R I * C P * I K * R D Y I Y L I * T * A * S L W R G H S G E T N * R K P F Q A * N * N S   F4

      I Q K T L E D L * F S S A I A S S K G R L V R M E F G F S S S N I T I S T P S E A T L V T L T T A K A N R N A L S G R L Q S L F S Q  F1
     P Y R R H L K T Y D F H L Q * L Q V R V D * Y E W N L A F L L Q I S L F Q H H Q K P L L * L * Q L R K Q I E M L Y L G D Y N H F S P S   F2
      H T E D T * R P M I F I C N S F K * G S I S T N G I W L F F F K Y H Y F N T I R S H S C D F N N C E S K S K C F I W E T T I T F L P V  F3
2801 CCATACAGAAGACACTTGAAGACCTATGATTTTCATCTGCAATAGCTTCAAGTAAGGGTCGATTAGTACGAATGGAATTTGGCTTTTCTTCTTCAAATATCACTATTTCAACACCATCAGAAGCCACTCTTGTGACTTTAACAACTGCGAAAGCAAATCGAAATGCTTTATCTGGGAGACTACAATCACTTTTCTCCCAG 3000
     ----:----|----:----|----:----|----:----|----:----|----:----|----:----|----:----|----:----|----:----|----:----|----:----|----:----|----:----|----:----|----:----|----:----|----:----|----:----|----:----|
2801 GGTATGTCTTCTGTGAACTTCTGGATACTAAAAGTAGACGTTATCGAAGTTCATTCCCAGCTAATCATGCTTACCTTAAACCGAAAAGAAGAAGTTTATAGTGATAAAGTTGTGGTAGTCTTCGGTGAGAACACTGAAATTGTTGACGCTTTCGTTTAGCTTTACGAAATAGACCCTCTGATGTTAGTGAAAAGAGGGTC 3000
     A M C F V S S S R H N E D A I A E L L P R N T R I S N P K E E E F I V I E V G D S A V R T V K V V A F A F R F A K D P L S C D S K E W   F6
      W V S S V Q L G I I K M Q L L K L Y P D I L V F P I Q S K K K L Y * * K L V M L L W E Q S K L L Q S L L D F H K I Q S V V I V K R G  F5
      G Y L L C K F V * S K * R C Y S * T L T S * Y S H F K A K R R * I D S N * C W * F G S K H S * C S R F C I S I S * R P S * L * K E G L  F4

     * T D T S P I P S A P S L M * V F K S K D G S E G S I L F D T R P S L M * G S S S S S V F A I A S T * L * R T P F K F G K G N S G D D   F1
      K P I L L P F H L H H L * C R F L K A K M G L R D L F Y L T Q D H L * C R D L A P Q V S L L * L Q H N Y S V H L S N L E K V T L V T T  F2
      N R Y F S H S I C T I F N V G F * K Q R W V * G I Y F I * H K T I F D V G I * L L K C L C Y S F N I I I A Y T F Q I W K R * L W * R  F3
3001 TAAACCGATACTTCTCCCATTCCATCTGCACCATCTTTAATGTAGGTTTTTAAAAGCAAAGATGGGTCTGAGGGATCTATTTTATTTGACACAAGACCATCTTTGATGTAGGGATCTAGCTCCTCAAGTGTCTTTGCTATAGCTTCAACATAATTATAGCGTACACCTTTCAAATTTGGAAAAGGTAACTCTGGTGACGA 3200
     ----:----|----:----|----:----|----:----|----:----|----:----|----:----|----:----|----:----|----:----|----:----|----:----|----:----|----:----|----:----|----:----|----:----|----:----|----:----|----:----|
3001 ATTTGGCTATGAAGAGGGTAAGGTAGACGTGGTAGAAATTACATCCAAAAATTTTCGTTTCTACCCAGACTCCCTAGATAAAATAAACTGTGTTCTGGTAGAAACTACATCCCTAGATCGAGGAGTTCACAGAAACGATATCGAAGTTGTATTAATATCGCATGTGGAAAGTTTAAACCTTTTCCATTGAGACCACTGCT 3200
      Y V S V E G M G D A G D K I Y T K L L L S P D S P D I K N S V L G D K I Y P D L E E L T K A I A E V Y N Y R V G K L N P F P L E P S S  F6
     T F R Y K E W E M Q V M K L T P K * F C L H T Q P I * K I Q C L V M K S T P I * S R L H R Q * L K L M I I A Y V K * I Q F L Y S Q H R   F5
      L G I S R G N W R C W R * H L N K F A F I P R L S R N * K V C S W R Q H L S R A G * T D K S Y S * C L * L T C R E F K S F T V R T V  F4

      R H E V T L M G L K S F V T S C E S A I S F G F K G V * * R S P * P K K F A * T E N L T D C G K * F F S A L L T A C G V Q K V K Q P F  F1
      D M R * H * W V * N H L S L H V K A L F L S V S K G C D R D H R D Q R N L P E Q R I * Q I V A S N F F Q H C * L H V G Y K K S S N L  F2
     Q T * G N I D G F E I I C H F M * K R Y F F R F Q R G V I E I T V T K E I C L N R E S N R L W Q V I F F S I V N C M W G T K S Q A T F   F3
3201 CAGACATGAGGTAACATTGATGGGTTTGAAATCATTTGTCACTTCATGTGAAAGCGCTATTTCTTTCGGTTTCAAAGGGGTGTGATAGAGATCACCGTGACCAAAGAAATTTGCCTGAACAGAGAATCTAACAGATTGTGGCAAGTAATTTTTTTCAGCATTGTTAACTGCATGTGGGGTACAAAAAGTCAAGCAACCTT 3400
     ----:----|----:----|----:----|----:----|----:----|----:----|----:----|----:----|----:----|----:----|----:----|----:----|----:----|----:----|----:----|----:----|----:----|----:----|----:----|----:----|
3201 GTCTGTACTCCATTGTAACTACCCAAACTTTAGTAAACAGTGAAGTACACTTTCGCGATAAAGAAAGCCAAAGTTTCCCCACACTATCTCTAGTGGCACTGGTTTCTTTAAACGGACTTGTCTCTTAGATTGTCTAACACCGTTCATTAAAAAAAGTCGTAACAATTGACGTACACCCCATGTTTTTCAGTTCGTTGGAA 3400
      L C S T V N I P K F D N T V E H S L A I E K P K L P T H Y L D G H G F F N A Q V S F R V S Q P L Y N K E A N N V A H P T C F T L C G  F6
      C V H P L M S P N S I M Q * K M H F R * K K R N * L P T I S I V T V L S I Q R F L S D L L N H C T I K K L M T L Q M H P V F L * A V K  F5
     V S M L Y C Q H T Q F * K D S * T F A S N R E T E F P H S L S * R S W L F K G S C L I * C I T A L L K K * C Q * S C T P Y L F D L L R   F4

      C F R Y * Y C D L * * L L L C K V S T L K A R H S S M D R L P K C S A Q R L S I A S P L L S F K S S K R * R S I K N S T S V * F S L  F1
     S V S G T D I V T Y S D S Y F V K C P P L K Q D T H Q W T D S Q N A Q P K D Y Q * L P H S Y H S N P L N A D A A * K I A H Q F D F L C   F2
      L F Q V L I L * P I V T L T L * S V H P * S K T L I N G Q T P K M L S P K T I N S F P T L I I Q I L * T L T Q H K K * H I S L I F F V  F3
3401 TCTGTTTCAGGTACTGATATTGTGACCTATAGTGACTCTTACTTTGTAAAGTGTCCACCCTTAAAGCAAGACACTCATCAATGGACAGACTCCCAAAATGCTCAGCCCAAAGACTATCAATAGCTTCCCCACTCTTATCATTCAAATCCTCTAAACGCTGACGCAGCATAAAAAATAGCACATCAGTTTGATTTTCTTTG 3600
     ----:----|----:----|----:----|----:----|----:----|----:----|----:----|----:----|----:----|----:----|----:----|----:----|----:----|----:----|----:----|----:----|----:----|----:----|----:----|----:----|
3401 AGACAAAGTCCATGACTATAACACTGGATATCACTGAGAATGAAACATTTCACAGGTGGGAATTTCGTTCTGTGAGTAGTTACCTGTCTGAGGGTTTTACGAGTCGGGTTTCTGATAGTTATCGAAGGGGTGAGAATAGTAAGTTTAGGAGATTTGCGACTGCGTCGTATTTTTTATCGTGTAGTCAAACTAAAAGAAAC 3600
     K Q K L Y Q Y Q S R Y H S K S Q L T D V R L A L C E D I S L S G F H E A W L S D I A E G S K D N L D E L R Q R L M F F L V D T Q N E K   F6
      R N * T S I N H G I T V R V K Y L T W G * L L V S M L P C V G L I S L G F V I L L K G V R I M * I R * V S V C C L F Y C M L K I K K  F5
      E T E P V S I T V * L S E * K T F H G G K F C S V * * H V S E W F A * G L S * * Y S G W E * * E F G R F A S A A Y F I A C * N S K R Q  F4

     C V S Q N L P T S E I T D R N L Y C F A Y F L D T E * R G T L F S T K C P G L L P L S F F F K V K V S N N T R S W F G S G N K S C S T   F1
      V F H K I Y R H L K S Q I A I Y I V L H T S W T Q N K E G L Y F Q Q N V Q D F Y P F H F S L K S R Y Q I I P E A G L V V V T N L V L H  F2
      C F T K F T D I * N H R S Q S I L F C I L P G H R I K R D F I F N K M S R T F T P F I F L * S Q G I K * Y Q K L V W * W * Q I L F Y  F3
3601 TGTGTTTCACAAAATTTACCGACATCTGAAATCACAGATCGCAATCTATATTGTTTTGCATACTTCCTGGACACAGAATAAAGAGGGACTTTATTTTCAACAAAATGTCCAGGACTTTTACCCCTTTCATTTTTCTTTAAAGTCAAGGTATCAAATAATACCAGAAGCTGGTTTGGTAGTGGTAACAAATCTTGTTCTAC 3800
     ----:----|----:----|----:----|----:----|----:----|----:----|----:----|----:----|----:----|----:----|----:----|----:----|----:----|----:----|----:----|----:----|----:----|----:----|----:----|----:----|
3601 ACACAAAGTGTTTTAAATGGCTGTAGACTTTAGTGTCTAGCGTTAGATATAACAAAACGTATGAAGGACCTGTGTCTTATTTCTCCCTGAAATAAAAGTTGTTTTACAGGTCCTGAAAATGGGGAAAGTAAAAAGAAATTTCAGTTCCATAGTTTATTATGGTCTTCGACCAAACCATCACCATTGTTTAGAACAAGATG 3800
      H T E C F K G V D S I V S R L R Y Q K A Y K R S V S Y L P V K N E V F H G P S K G R E N K K L T L T D F L V L L Q N P L P L L D Q E V  F6
     T H K V F N V S M Q F * L D C D I N N Q M S G P C L I F L S K I K L L I D L V K V G K M K R * L * P I L Y Y W F S T Q Y H Y C I K N *   F5
      T N * L I * R C R F D C I A I * I T K C V E Q V C F L S P S * K * C F T W S K * G K * K E K F D L Y * I I G S A P K T T T V F R T R  F4

     S I H P L Q T G H D C L T V H Q A T I H D L Q N M C * Q T V Y T G S F G T V K Q I E H F K I S A R C V C S Y N S T G V P V T S T F S N   F1
      L Y I P Y K Q G M T A * Q Y T R Q Q Y T I Y K I C V D K L Y T P A L L V R L S K * S I S K F L Q G V F V H T I L L A F Q * L Q L F Q  F2
     I Y T S P T N R A * L P D S T P G N N T R F T K Y V L T N C I H R L F W Y G * A N R A F Q N F C K V C L F I Q F Y W R S S N F N F F K   F3
3801 ATCTATACATCCCCTACAAACAGGGCATGACTGCCTGACAGTACACCAGGCAACAATACACGATTTACAAAATATGTGTTGACAAACTGTATACACCGGCTCTTTTGGTACGGTTAAGCAAATAGAGCATTTCAAAATTTCTGCAAGGTGTGTTTGTTCATACAATTCTACTGGCGTTCCAGTAACTTCAACTTTTCAA 4000
     ----:----|----:----|----:----|----:----|----:----|----:----|----:----|----:----|----:----|----:----|----:----|----:----|----:----|----:----|----:----|----:----|----:----|----:----|----:----|----:----|
3801 TAGATATGTAGGGGATGTTTGTCCCGTACTGACGGACTGTCATGTGGTCCGTTGTTATGTGCTAAATGTTTTATACACAACTGTTTGACATATGTGGCCGAGAAAACCATGCCAATTCGTTTATCTCGTAAAGTTTTAAAGACGTTCCACACAAACAAGTATGTTAAGATGACCGCAAGGTCATTGAAGTTGAAAAAGTT 4000
      D I C G R C V P C S Q R V T C W A V I C S K C F I H Q C V T Y V P E K P V T L C I S C K L I E A L H T Q E Y L E V P T G T V E V K E  F6
     M * V D G V F L A H S G S L V G P L L V R N V F Y T N V F Q I C R S K Q Y P * A F L A N * F K Q L T H K N M C N * Q R E L L K L K K L   F5
     C R Y M G * L C P M V A Q C Y V L C C Y V I * L I H T S L S Y V G A R K T R N L L Y L M E F N R C P T N T * V I R S A N W Y S * S K *   F4

      E T S S I S T E M S L H A V F D M L H V L V V L G D D F L Y L S L F T G S E D T L R E Q D D F S H L F K R S S E S D S S * R E Q G R  F1
     M K R H P F Q L K C L Y M Q C L T C Y M Y * * Y L E T T F Y T C H C L L D Q K T H C V N K M T S H T C L N G R V K A I H R D V N R A A   F2
      * N V I H F N * N V F T C S V * H V T C T S S T W R R L F I L V T V Y W I R R H T A * T R * L L T L V * T V E * K R F I V T * T G P L  F3
4001 ATGAAACGTCATCCATTTCAACTGAAATGTCTTTACATGCAGTGTTTGACATGTTACATGTACTAGTAGTACTTGGAGACGACTTTTTATACTTGTCACTGTTTACTGGATCAGAAGACACACTGCGTGAACAAGATGACTTCTCACACTTGTTTAAACGGTCGAGTGAAAGCGATTCATCGTGACGTGAACAGGGCCGC 4200
     ----:----|----:----|----:----|----:----|----:----|----:----|----:----|----:----|----:----|----:----|----:----|----:----|----:----|----:----|----:----|----:----|----:----|----:----|----:----|----:----|
4001 TACTTTGCAGTAGGTAAAGTTGACTTTACAGAAATGTACGTCACAAACTGTACAATGTACATGATCATCATGAACCTCTGCTGAAAAATATGAACAGTGACAAATGACCTAGTCTTCTGTGTGACGCACTTGTTCTACTGAAGAGTGTGAACAAATTTGCCAGCTCACTTTCGCTAAGTAGCACTGCACTTGTCCCGGCG 4200
     F S V D D M E V S I D K C A T N S M N C T S T T S P S S K K Y K D S N V P D S S V S R S C S S K E C K N L R D L S L S E D H R S C P R   F6
      H F T M W K L Q F T K V H L T Q C T V H V L L V Q L R S K I S T V T * Q I L L C V A H V L H S R V S T * V T S H F R N M T V H V P G  F5
      I F R * G N * S F H R * M C H K V H * M Y * Y Y K S V V K * V Q * Q K S S * F V C Q T F L I V E * V Q K F P R T F A I * R S T F L A A  F4

     F S N A M S S L L D C A S A L S L L E M H A D F L L F G L P L C T L H I I Q P V N L R S S K Q Y C F K K S P S V L R F I L H R H G P H   F1
      F Q M Q C H H Y W I V H Q P C H F * K C M L T S S C L A F L C V H Y I S Y S Q * I Y V P R N N I V L K N H P L F Y D S F Y I D T D H I  F2
      F K C N V I I T G L C I S L V T S R N A C * L P L V W P S S V Y I T Y H T A S K F T F L E T I L F * K I T L C F T I H F T S T R T T  F3
4201 TTTTCAAATGCAATGTCATCATTACTGGATTGTGCATCAGCCTTGTCACTTCTAGAAATGCATGCTGACTTCCTCTTGTTTGGCCTTCCTCTGTGTACATTACATATCATACAGCCAGTAAATTTACGTTCCTCGAAACAATATTGTTTTAAAAAATCACCCTCTGTTTTACGATTCATTTTACATCGACACGGACCACA 4400
     ----:----|----:----|----:----|----:----|----:----|----:----|----:----|----:----|----:----|----:----|----:----|----:----|----:----|----:----|----:----|----:----|----:----|----:----|----:----|----:----|
4201 AAAAGTTTACGTTACAGTAGTAATGACCTAACACGTAGTCGGAACAGTGAAGATCTTTACGTACGACTGAAGGAGAACAAACCGGAAGGAGACACATGTAATGTATAGTATGTCGGTCATTTAAATGCAAGGAGCTTTGTTATAACAAAATTTTTTAGTGGGAGACAAAATGCTAAGTAAAATGTAGCTGTGCCTGGTGT 4400
      K E F A I D D N S S Q A D A K D S R S I C A S K R K N P R G R H V N C I M C G T F K R E E F C Y Q K L F D G E T K R N M K C R C P G C  F6
     S K L H L T M M V P N H M L R T V E L F A H Q S G R T Q G E E T Y M V Y * V A L L N V N R S V I N N * F I V R Q K V I * K V D V R V V   F5
      K * I C H * * * Q I T C * G Q * K * F H M S V E E Q K A K R Q T C * M D Y L W Y I * T G R F L I T K F F * G R N * S E N * M S V S W  F4

      R Y S G * I S A K S F T I F T L * T S A I M F A R V L Q R Y L F P L I F V P H K R H K L R S F S I C A A C S L C G L A S I L I A R H S  F1
      G T L G E Y R P S H S L Y L R Y R P L Q L C L Q E F C K D T C F L * F L Y H I S D T S Y V A F P Y V P H V H C V D W L P Y S S H G T  F2
     * V L W V N I G Q V I H Y I Y V I D L C N Y V C K S F A K I L V S F D F C T T * A T Q V T * L F H M C R M F T V W T G F H T H R T A L   F3
4401 TAGGTACTCTGGGTGAATATCGGCCAAGTCATTCACTATATTTACGTTATAGACCTCTGCAATTATGTTTGCAAGAGTTTTGCAAAGATACTTGTTTCCTTTGATTTTTGTACCACATAAGCGACACAAGTTACGTAGCTTTTCCATATGTGCCGCATGTTCACTGTGTGGACTGGCTTCCATACTCATCGCACGGCACT 4600
     ----:----|----:----|----:----|----:----|----:----|----:----|----:----|----:----|----:----|----:----|----:----|----:----|----:----|----:----|----:----|----:----|----:----|----:----|----:----|----:----|
4401 ATCCATGAGACCCACTTATAGCCGGTTCAGTAAGTGATATAAATGCAATATCTGGAGACGTTAATACAAACGTTCTCAAAACGTTTCTATGAACAAAGGAAACTAAAAACATGGTGTATTCGCTGTGTTCAATGCATCGAAAAGGTATACACGGCGTACAAGTGACACACCTGACCGAAGGTATGAGTAGCGTGCCGTGA 4600
      L Y E P H I D A L D N V I N V N Y V E A I I N A L T K C L Y K N G K I K T G C L R C L N R L K E M H A A H E S H P S A E M S M A R C  F6
      Y T S Q T F I P W T M * * I * T I S R Q L * T Q L L K A F I S T E K S K Q V V Y A V C T V Y S K W I H R M N V T H V P K W V * R V A S  F5
     M P V R P S Y R G L * E S Y K R * L G R C N H K C S N Q L S V Q K R Q N K Y W M L S V L * T A K G Y T G C T * Q T S Q S G Y E D C P V   F4

      T G L I C Y L Q H G C V F L K V I I * * L L I F L * L * I L S V H L A S V H S C C K A V Q H A V S I M * * F * I L K I I R V K K I *  F1
     R L V * S A I Y S M V V Y S * K * * F N N F * F F C N Y E Y Y L C T * H L F I A A V K L C S M Q C L S C N D S R F * K * S G * K K F N   F2
      D W S D L L F T A W L C I P E S N N L I T F N F S V I M N I I C A P S I C S * L L * S C A A C S V Y H V M I L D F K N N Q G K K N L I  F3
4601 CGACTGGTCTGATCTGCTATTTACAGCATGGTTGTGTATTCCTGAAAGTAATAATTTAATAACTTTTAATTTTTCTGTAATTATGAATATTATCTGTGCACCTAGCATCTGTTCATAGCTGCTGTAAAGCTGTGCAGCATGCAGTGTCTATCATGTAATGATTCTAGATTTTAAAAATAATCAGGGTAAAAAAAATTTAA 4800
     ----:----|----:----|----:----|----:----|----:----|----:----|----:----|----:----|----:----|----:----|----:----|----:----|----:----|----:----|----:----|----:----|----:----|----:----|----:----|----:----|
4601 GCTGACCAGACTAGACGATAAATGTCGTACCAACACATAAGGACTTTCATTATTAAATTATTGAAAATTAAAAAGACATTAATACTTATAATAGACACGTGGATCGTAGACAAGTATCGACGACATTTCGACACGTCGTACGTCACAGATAGTACATTACTAAGATCTAAAATTTTTATTAGTCCCATTTTTTTTAAATT 4800
     E V P R I Q * K C C P Q T N R F T I I * Y S K I K R Y N H I N D T C R A D T * L Q Q L A T C C A T D I M Y H N * I K F I I L T F F I *   F6
      S Q D S R S N V A H N H I G S L L L K I V K L K E T I I F I I Q A G L M Q E Y S S Y L Q A A H L T * * T I I R S K L F L * P L F F K  F5
      R S T Q D A I * L M T T Y E Q F Y Y N L L K * N K Q L * S Y * R H V * C R N M A A T F S H L M C H R D H L S E L N * F Y D P Y F F N L  F4

     S W T S Y I G C C V H S L A * H D N H I K A H M T P T Q N H Y N I S Y L T C Y S F C A * Q Y L Y V S Y N S E L I R P I R R P F Y L K *   F1
      L G H L I L D V V S I A W P D M I T T * R P I * P Q L K I I I I F H I * L V I P S V H S N I Y M S V I I Q S * F G Q F G G H F I L N N  F2
      L D I L Y W M L C P * P G L T * * P H K G P Y D P N S K S L * Y F I F D L L F L L C I A I S I C Q L * F R A D S A N S A A I L S * I  F3
4801 TCTTGGACATCTTATATTGGATGTTGTGTCCATAGCCTGGCCTGACATGATAACCACATAAAGGCCCATATGACCCCAACTCAAAATCATTATAATATTTCATATTTGACTTGTTATTCCTTCTGTGCATAGCAATATCTATATGTCAGTTATAATTCAGAGCTGATTCGGCCAATTCGGCGGCCATTTTATCTTAAATA 5000
     ----:----|----:----|----:----|----:----|----:----|----:----|----:----|----:----|----:----|----:----|----:----|----:----|----:----|----:----|----:----|----:----|----:----|----:----|----:----|----:----|
4801 AGAACCTGTAGAATATAACCTACAACACAGGTATCGGACCGGACTGTACTATTGGTGTATTTCCGGGTATACTGGGGTTGAGTTTTAGTAATATTATAAAGTATAAACTGAACAATAAGGAAGACACGTATCGTTATAGATATACAGTCAATATTAAGTCTCGACTAAGCCGGTTAAGCCGCCGGTAAAATAGAATTTAT 5000
      D Q V D * I P H Q T W L R A Q C S L W M F A W I V G V * F * * L I E Y K V Q * E K Q A Y C Y R Y T L * L E S S I R G I R R G N * R L Y  F6
     I K S M K Y Q I N H G Y G P R V H Y G C L P G Y S G L E F D N Y Y K M N S K N N R R H M A I D I H * N Y N L A S E A L E A A M K D * I   F5
      R P C R I N S T T D M A Q G S M I V V Y L G M H G W S L I M I I N * I Q S T I G E T C L L I * I D T I I * L Q N P W N P P W K I K F  F4
```

```
      F  S  G  A  C  K  Y  W  P  C  S  P  S  K  L  P  C  L  P  W  L  L  Y  I  E  R  *  T  K  N  R  A  S  W  K  K  I  S  F  P  H  K  E  S  S  F  D  S  N  T  Y  I  P  L  Q  L  I  S  S  N  F  H  C  *  N  T  K   F1
       S  Q  V  H  V  N  T  G  L  V  H  L  L  N  C  H  V  Y  L  G  Y  Y  I  *  N  D  R  Q  R  I  E  L  R  G  K  K  S  L  F  L  T  K  N  P  L  S  I  P  T  H  I  S  R  C  S  L  Y  L  L  I  F  I  A  E  I  Q    F2
     I  L  R  C  M  *  I  L  A  L  F  T  F  *  T  A  M  F  T  L  V  T  I  Y  R  T  I  D  K  E  S  S  F  V  E  K  N  L  F  S  S  Q  R  I  L  F  R  F  Q  H  I  Y  P  A  A  A  Y  I  F  *  F  S  L  L  K  Y  K   F3
5001 ATTCTCAGGTGCATGTAAATACTGGCCTTGTTCACCTTCTAAACTGCCATGTTTACCTTGGTTACTATATATAGAACGATAGACAAAGAATCGAGCTTCGTGGAAAAAAATCTCTTTTCCTCACAAAGAATCCTCTTTCGATTCCAACACATATATCCCGCTGCAGCTTATATCTTCTAATTTTCATTGCTGAAATACAA 5200
     ----:----|----:----|----:----|----:----|----:----|----:----|----:----|----:----|----:----|----:----|----:----|----:----|----:----|----:----|----:----|----:----|----:----|----:----|----:----|----:----|
5001 TAAGAGTCCACGTACATTTATGACCGGAACAAGTGGAAGATTTGACGGTACAAATGGAACCAATGATATATATCTTGCTATCTGTTTCTTAGCTCGAAGCACCTTTTTTTAGAGAAAAGGAGTGTTTCTTAGGAGAAAGCTAAGGTTGTGTATATAGGGCGACGTCGAATATAGAAGATTAAAAGTAACGACTTTATGTT 5200
       N  E  P  A  H  L  Y  Q  G  Q  E  G  E  L  S  G  H  K  G  Q  N  S  Y  I  S  R  Y  V  F  F  R  A  E  H  F  F  I  E  K  G  *  L  S  D  E  K  S  E  L  V  Y  I  G  S  C  S  I  D  E  L  K  *  Q  Q  F  V    F6
      I  R  L  H  M  Y  I  S  A  K  N  V  K  *  V  A  M  N  V  K  T  V  I  Y  L  V  I  S  L  S  D  L  K  T  S  F  F  R  K  E  E  C  L  I  R  K  R  N  W  C  M  Y  G  A  A  A  *  I  K  *  N  E  N  S  F  Y  L   F5
     L  E  *  T  C  T  F  V  P  R  T  *  R  R  F  Q  W  T  *  R  P  *  *  I  Y  F  S  L  C  L  I  S  S  R  P  F  F  D  R  K  R  V  F  F  G  R  E  I  G  V  C  I  D  R  Q  L  K  Y  R  R  I  K  M  A  S  I  C    F4


       T  S  K  F  R  Q  K  S  I  N  L  P  I  F  K  R  K  V  P  P  P  N  S  V  S  L  L  Y  G  N  S  D  C  A  C  Q  W  E  S  P  N  P  K  I  E  L  S  R  T  E  I  D  L  N  E  A  R  Y  G  R  *  L  W  R  L  Y    F1
     K  L  Q  N  S  G  K  N  L  *  T  Y  R  F  S  S  G  K  F  H  H  Q  T  Q  F  L  F  Y  T  E  I  A  T  A  H  V  S  G  N  L  P  T  P  K  *  S  F  H  V  P  K  *  I  *  T  K  H  V  T  G  G  N  S  G  G  F  T    F2
      N  F  K  I  P  A  K  I  Y  K  L  T  D  F  Q  A  E  S  S  T  T  K  L  S  F  S  S  I  R  K  *  R  L  R  M  S  V  G  I  S  Q  P  Q  N  R  A  F  T  Y  R  N  R  F  E  R  S  T  L  R  E  V  T  L  A  A  L  Q   F3
5201 AAACTTCAAAATTCCGGCAAAAATCTATAAACTTACCGATTTTCAAGCGGAAAGTTCCACCACCAAACTCAGTTTCTCTTCTATACGGAAATAGCGACTGCGCATGTCAGTGGGAATCTCCCAACCCCAAAATAGAGCTTTCACGTACCGAAATAGATTTGAACGAAGCACGTTACGGGAGGTAACTCTGGCGGCTTTAC 5400
     ----:----|----:----|----:----|----:----|----:----|----:----|----:----|----:----|----:----|----:----|----:----|----:----|----:----|----:----|----:----|----:----|----:----|----:----|----:----|----:----|
5201 TTTGAAGTTTTAAGGCCGTTTTTAGATATTTGAATGGCTAAAAGTTCGCCTTTCAAGGTGGTGGTTTGAGTCAAAGAGAAGATATGCCTTTATCGCTGACGCGTACAGTCACCCTTAGAGGGTTGGGGTTTTATCTCGAAAGTGCATGGCTTTATCTAAACTTGCTTCGTGCAATGCCCTCCATTGAGACCGCCGAAATG 5400
     F  V  E  F  N  R  C  F  D  I  F  K  G  I  K  L  R  F  T  G  G  G  F  E  T  E  R  R  Y  P  F  L  S  Q  A  H  *  H  S  D  G  L  G  L  I  S  S  E  R  V  S  I  S  K  F  S  A  R  *  P  L  Y  S  Q  R  S  *    F6
       F  K  L  I  G  A  F  I  *  L  S  V  S  K  *  A  S  L  E  V  V  L  S  L  K  E  E  I  R  F  Y  R  S  R  M  D  T  P  I  E  W  G  W  F  L  A  K  V  Y  R  F  L  N  S  R  L  V  N  R  S  T  V  R  A  A  K    F5
      F  S  *  F  E  P  L  F  R  Y  V  *  R  N  E  L  P  F  N  W  W  W  V  *  N  R  K  *  V  S  I  A  V  A  C  T  L  P  F  R  G  V  G  F  Y  L  K  *  T  G  F  Y  I  Q  V  F  C  T  V  P  P  L  E  P  P  K  V   F4


     R  V  I  P  K  Y  G  G  F  I  L  G  K  E  V  R  K  W  *  G  Q                                                                                                                                         F1
      E  L  F  P  N  M  A  A  L  S  *  A  K  K  F  V  S  G  E  A  X                                                                                                                                        F2
       S  Y  S  Q  I  W  R  L  Y  L  R  Q  R  S  S  *  V  V  R  P  X                                                                                                                                       F3
5401 AGAGTTATTCCCAAATATGGCGGCTTTATCTTAGGCAAAGAAGTTCGTAAGTGGTGAGGCCAA 5463
     ----:----|----:----|----:----|----:----|----:----|----:----|---
5401 TCTCAATAAGGGTTTATACCGCCGAAATAGAATCCGTTTCTTCAAGCATTCACCACTCCGGTT 5463
      L  T  I  G  L  Y  P  P  K  I  K  P  L  S  T  R  L  H  H  P  W                                                                                                                                        F6
     C  L  *  E  W  I  H  R  S  *  R  L  C  L  L  E  Y  T  T  L  G                                                                                                                                         F5
       S  N  N  G  F  I  A  A  K  D  *  A  F  F  N  T  L  P  S  A  L                                                                                                                                       F4
```

Pinctada imbricata (Pim)

WGS: NIJJ01005135.1

6790-12673
Pinctada imbricata breed pearl oyster-OUG PIN_chr10_10269, whole genome shotgun sequence

```
     R I S T L T N V Y A T F A F E * I M Q I K L K K I R V V F L A H R F N S Y Q Y S * C S K L R R K F P * E * C G V I V L C Y F L F * S K   F1
     E L V H * Q T S M Q L L L L N K L C K L S * K K L E L S F L L T A S I H T S T R S A P N C G G N S H K N N V E L S F F V T S C F D R N   F2
     N * Y T N K R L C N F C F * I N Y A N * V K K N * S C L S C S P L Q F I P V L V V L Q I A E E I P I R I M W S Y R S L L L P V L I E   F3
   1 CGAATTAGTACACTAACAAACGTCTATGCAACTTTTGCTTTGAATAAATTATGCAAATTAAGTTAAAAAAAATTAGAGTTGTCTTTCTTGCTCACCGCTTCAATTCATACCAGTACTCGTAGTGCTCCAAATTGCGGAGGAAATTCCCATAAGAATAATGTGGAGTTATCGTTCTTGTTACTTCCTGTTTTGATCGAA 200
     ----:----|----:----|----:----|----:----|----:----|----:----|----:----|----:----|----:----|----:----|----:----|----:----|----:----|----:----|----:----|----:----|----:----|----:----|----:----|----:----|
   1 GCTTAATCATGTGATTGTTTGCAGATACGTTGAAAACGAAAACTTATTTAATACGTTTAATTCAATTTTTTTTAATCTCAACAGAAAGAACGAGTGGCGAAGTTAAGTATGGTCATGAGCATCACGAGGTTTAACGCCTCCTTTAAGGGTATTCTTATTACACCTCAATAGCAAGAACAATGAAGGACAAAACTAGCTT 200
     R I L V S V F T * A V K A K S Y I I C I L N F F I L T T K R A * R K L E Y W Y E Y H E L N R L F N G Y S Y H P T I T R Q * K R N Q D F   F6
     X F * Y V L L R R H L K Q K Q I F * A F * T L F F * L Q R E Q E G S * N M G T S T T S W I A S S I G M L I I H L * R E K N S G T K I S   F5
     S N T C * C V D I C S K S K F L N H L N L * F F N S N D K K S V A E I * V L V R L A G F Q P P F E W L F L T S N D N K T V E Q K S R   F4

     H Q * K S Y * N R P R N G F L A I L * S L V N I L E K I Y T F E V * N K T N C I K S S A N V T D T V Q F F S D S P E I L L S F R I G L   F1
     I S E K V I E I D Q E M D F * Q F Y E V W * I F * K K Y T H L R Y E I K Q I V * N Q V Q M L Q I L C N F F Q I L Q K F Y F H F V * V   F2
     T S V K K L L K S T K K W I F S N F M K F G E Y F R K N I H I * G M K * N K L Y K I K C K C Y R Y C A I F F R F S R N S T F I S Y R S   F3
 201 ACATCAGTGAAAAAGTTATTGAAATCGACCAAGAAATGGATTTTTAGCAATTTTATGAAGTTTGGTGAATATTTTAGAAAAAATATACACATTTGAGGTATGAAATAAAACAAATTGTATAAAATCAAGTGCAAATGTTACAGATACTGTGCAATTTTTTTCAGATTCTCCAGAAATTCTACTTTCATTTCGTATAGGTC 400
     ----:----|----:----|----:----|----:----|----:----|----:----|----:----|----:----|----:----|----:----|----:----|----:----|----:----|----:----|----:----|----:----|----:----|----:----|----:----|----:----|
 201 TGTAGTCACTTTTTCAATAACTTTAGCTGGTTCTTTACCTAAAAATCGTTAAAATACTTCAAACCACTTATAAAATCTTTTTTATATGTGTAAACTCCATACTTTATTTTGTTTAACATATTTTAGTTCACGTTTACAATGTCTATGACACGTTAAAAAAAGTCTAAGAGGTCTTTAAGATGAAAGTAAAGCATATCCAG 400
     C * H F L * Q F R G L F P N K A I K H L K T F I K S F I Y V N S T H F L V F Q I F D L A F T V S V T C N K E S E G S I R S E N R I P   F6
     V D T F F N N F D V L F H I K L L K I F N P S Y K L F F I C M Q P I F Y F L N Y L I L H L H * L Y Q A I K K L N E L F E V K M E Y L D   F5
     F M L S F T I S I S W S I S K * C N * S T Q H I N * F F Y V C K L Y S I F C I T Y F * T C I N C I S H L K K * I R W F N * K * K T Y T   F4

     N V R H S I V F C V N S L E T R H V P L * Y A K R I I D G K S I I P Y I D F N C C S N V T N Y N I E N E G C M H G K K G V S Q P F *   F1
     S M C V I L S F F A S T L L K L G M S H Y S M Q N E S L M E S Q * Y H I * I L I V A A M L L I T I * K M R D A C M V K R G C H S L S D   F2
     Q C A S F Y R F L R Q L S * N * A C P I I V C K T N H * W K V N N T I Y R F * L L Q Q C Y * L Q Y R K * G M H A W * K G G V T A F L T   F3
 401 TCAATGTGCGTCATTCTATCGTTTTTTGCGTCAACTCTCTTGAAACTAGGCATGTCCCATTATAGTATGCAAAACGAATCATTGATGGAAAGTCAATAATACCATATATAGATTTTAATTGTTGCAGCAATGTTACTAATTACAATATAGAAAATGAGGGATGCATGCATGGTAAAAAGGGGGTGTCACAGCCTTTCTGA 600
     ----:----|----:----|----:----|----:----|----:----|----:----|----:----|----:----|----:----|----:----|----:----|----:----|----:----|----:----|----:----|----:----|----:----|----:----|----:----|----:----|
 401 AGTTACACGCAGTAAGATAGCAAAAAACGCAGTTGAGAGAACTTTGATCCGTACAGGGTAATATCATACGTTTTGCTTAGTAACTACCTTTCAGTTATTATGGTATATATCTAAAATTAACAACGTCGTTACAATGATTAATGTTATATCTTTTACTCCCTACGTACGTACCATTTTTCCCCCACAGTGTCGGAAAGACT 600
     R L T R * E I T K Q T L E R S V L C T G N Y Y A F R I M S P F D I I G Y I S K L Q Q L L T V L * L I S F S P H M C P L F P T D C G K Q   F6
     * H A D N * R K K R * S E Q F * A H G M I T H L V F * Q H F T L L V M Y L N * N N C C H * * N C Y L F H P I C A H Y F P P T V A K R   F5
     E I H T M R D N K A D V R K F S P M D W * L I C F S D N I S L * Y Y W I Y I K I T A A I N S I V I Y F I L S A H M T F L P H * L R E S   F4

     P N N V I F S * E N R * F C K L N C T R I C T I Q K E I F V * H * Q A G N I A V * F N F Q D Y I * L T H I Y Q N L S K I H I F H R L I L   F1
     Q I M L F F P E K T G D S A S * I V P V F V Q S K K S L C S T S R Q A T L Q F N S T F K T T S N * H I Y I K T Y Q R F I F S I D * Y L   F2
     K * C Y F F L R K Q V I L Q A E L Y P Y L Y N P R N L C V A L A G R Q H C S L I Q L S R L H L I D T Y I S K P I K D S Y F P * I N T   F3
 601 CCAAATAATGTTATTTTTTCCTGAGAAAACAGGTGATTCTGCAAGCTGAATTGTACCCGTATTTGTACAATCCAAGAAATCTTTGTGTAGCACTAGCAGGCAGGCAACATTGCAGTTTAATTCAACTTTCAAGACTACATCTAATTGACACATATATATCAAAACCTATCAAAGATTCATATTTCCATAGATTAATACT 800
     ----:----|----:----|----:----|----:----|----:----|----:----|----:----|----:----|----:----|----:----|----:----|----:----|----:----|----:----|----:----|----:----|----:----|----:----|----:----|----:----|
 601 GGTTTATTACAATAAAAAAGGACTCTTTTGTCCACTAAGACGTTCGACTTAACATGGGCATAAACATGTTAGGTTCTTTAGAAACACATCGTGATCGTCCGTCCGTTGTAACGTCAAATTAAGTTGAAAGTTCTGATGTAGATTAACTGTGTATATATAGTTTTGGATAGTTTCTAAGTATAAAGGTATCTAATTATGA 800
     G F L T I K E Q S F L H N Q L S F Q V R I Q V I W S I K T Y C * C A P L M A T * N L K * S * M * N V C I Y * F R D F I * I K W L N I S   F6
     V L Y H * K K R L F C T I R C A S N Y G Y K Y L G L F R Q T A S A P L C C Q L K I * S E L S C R I S V Y I D F G I L S E Y K G Y I L V   F5
     W I I N N K G S F V P S E A L Q I T G T N T C D L F D K H L V L L C A V N C N L E V K L V V D L Q C M Y I L V * * L N M N E M S * Y   F4

     V S N F S G I F E T K P W R L L M K C K R M R I T L P I T L Q I * P N F V G C V V A S K R G R V E L F V Q L N L I K * N F Y H Y S K L   F1
     * V T F Q E F L R P N H G G C * * N A K G C G * H C Q S L C K F D Q I L * G V W * H P K E E E * S F L S S * T L * N R T F I I I Q N   F2
     C K * L F R N F * D Q T M E A A D E M Q K D A D N I A N H F A N L T K F C R V C G S I Q K R K S R A F C P V E P Y K I E L L S L F K I   F3
 801 TGTAAGTAACTTTTCAGGAATTTTTGAGACCAAACCATGGAGGCTGCTGATGAAATGCAAAAGGATGCGGATAACATTGCCAATCACTTTGCAAATTTGACCAAATTTTGTAGGGTGTGTGGTAGCATCCAAAAGAGGAAGAGTAGAGCTTTTTGTCCAGTTGAACCTTATAAAATAGAACTTTTATCATTATTCAAAAT 1000
     ----:----|----:----|----:----|----:----|----:----|----:----|----:----|----:----|----:----|----:----|----:----|----:----|----:----|----:----|----:----|----:----|----:----|----:----|----:----|----:----|
 801 ACATTCATTGAAAAGTCCTTAAAAACTCTGGTTTGGTACCTCCGACGACTACTTTACGTTTTCCTACGCCTATTGTAACGGTTAGTGAAACGTTTAAACTGGTTTAAAACATCCCACACACCATCGTAGGTTTTCTCCTTCTCATCTCGAAAAACAGGTCAACTTGGAATATTTTATCTTGAAAATAGTAATAAGTTTTA 1000
     T L K E P I K S V L G H L S S I F H L L I R I V N G I V K C I Q G F K T P H T T A D L L P L T S S K T W N F R I F Y F K * * E F   F6
     Q L Y S K L F K Q S W V M S A A S S I C F S A S L M A L * K A F K V L N Q L T H P L M W F L F L A K Q G T S G * L I S S K D N N L I   F5
     K Y T V K * S N K L G F W P P Q Q H F A F P H P Y C Q W D S Q L N S W I K Y P T H Y C G F S S S Y L K K D L Q V K Y F L V K I M I * F   F4

     I S N M M M K V F I L R N C A T H A G Q N A F I F A Q R K A K E I H F L * T F L L S I C T F L I M Q I V K F V I G I L D E M L P * S   F1
     * Y P I * * K Y S S * E I V Q L M P V K M L S F S H K E R R K Y I F Y E H S C * A F V H S S * C K L * S L S * E F W T K C Y H R V   F2
     D I Q Y D D E S I H P K K L C N S C R S K C F H F R T K K G E G N T F S M N I P V K H L Y I P H N A N C K V C H R N S G R N A T I E L   F3
1001 TGATATCCAATATGATGATGAAAGTATTCATCCTAAGAAATTGTGCAACTCATGCCGGTCAAAATGCTTTCATTTTCGCACAAAGAAAGGCGAAGGAAATACATTTTCTATGAACATTCCTGTTAAGCATTTGTACATTCCTCATAATGCAAATTGTAAAGTTTGTCATAGGAATTCTGGACGAAATGCTACCATAGAGT 1200
     ----:----|----:----|----:----|----:----|----:----|----:----|----:----|----:----|----:----|----:----|----:----|----:----|----:----|----:----|----:----|----:----|----:----|----:----|----:----|----:----|
1001 ACTATAGGTTATACTACTACTTTCATAAGTAGGATTCTTTAACACGTTGAGTACGGCCAGTTTTACGAAAGTAAAAGCGTGTTTCTTTCCGCTTCCTTTATGTAAAAGATACTTGTAAGGACAATTCGTAAACATGTAAGGAGTATTACGTTTAACATTTCAAACAGTATCCTTAAGACCTGCTTTACGATGGTATCTCA 1200
     N I D L I I I F T N M R L F Q A V * A P * F A K M K A C L F A F S I C K R H V N R N L M Q V N R M I C I T F N T M P I R S S I S G Y L   F6
     S I W Y S S S L I * G L F N H L E H R D F H K * K R V F F P S P F V N E I F M G T L C K Y M G * L A F Q L T Q * L F E P R F A V M S   F5
     Q Y G I H H H F Y E D * S I T C S M G T L I S E N E C L S L R L F Y M K * S C E Q * A N T C E E Y H L N Y L K D Y S N Q V F H * W L T   F4

     C F K F I I I H L Q M F P * N M * M G E V L V A T L N Q T V R I K D Q M I I L I H F Q L L A M * L H Q Q V A I F L Q L K W T N Q K F M   F1
     V S N S L * S I F R C S H R T C R W G R Y L * Q H * I R Q * G S R I K * S F S F I F S Y L P C S S I N K * P F F C S * N G Q I R N L C   F2
     F Q I H Y N P S S D V P I E H V D G G G T C S N T E S D S E D Q G S N D H S H S F S A T C H V A P S T S S H F F A A E M D K S E I Y   F3
1201 TGTTTCAAATTCATTATAATCCATCTTCAGATGTTCCCATAGAACATGTAGATGGGGGAGGTACTTGTAGCAACACTGAATCAGACAGTGAGGATCAAGGATCAAATGATCATTCTCATTCATTTTCAGCTACTTGCCATGTAGCTCCATCAACAAGTAGCCATTTTTTTGCAGCTGAAATGGACAAATCAGAAATTTAT 1400
     ----:----|----:----|----:----|----:----|----:----|----:----|----:----|----:----|----:----|----:----|----:----|----:----|----:----|----:----|----:----|----:----|----:----|----:----|----:----|----:----|
1201 ACAAAGTTTAAGTAATATTAGGTAGAAGTCTACAAGGGTATCTTGTACATCTACCCCCTCCATGAACATCGTTGTGACTTAGTCTGTCACTCCTAGTTCCTAGTTTACTAGTAAGAGTAAGTAAAAGTCGATGAACGGTACATCGAGGTAGTTGTTCATCGGTAAAAAAACGTCGACTTTACCTGTTTAGTCTTTAAATA 1400
     Q K L N M I I W R * I N G Y F M Y I P S T S T A V S F * V T L I L S * I I M R M * K * S S A M Y S W * C T A M K K C S F H F V * F N I   F6
     N N * I * * L G D E S T G M S C T S P P P V Q L L V S D S L S S * P D F S * E * E N E A V Q W T A G D V L L W K K A A S I S L D S I *   F5
     T E F E N Y D M K L H E W L V H L H P L Y K Y C C Q I L C H P D L I L H D N E N M K L * K G H L E M L L Y G N K K Q L Q F P C I L F K   F4

     H L L F Q K I Q M N Y V N Q L Q R N Y V D P K D Y L M L L N N * I L T A Q Y M C H P H S L * L I Y Q A F P * N K L L N S H W P R Y S S   F1
     I F S F R K F K * T M * T S F K E I M * T Q R I T * C Y * T I R F * Q H S T C A T L I H C N * F I K H S H R T S Y * T V I G Q D I P   F2
     A S S L S E N S N E L C E P A S K K L C R P K G L L D V T K Q L D F D S T V H V P P S F T V T D L S S I P I E Q A I E Q S L A K I F Q   F3
1401 GCATCTTCTCTTTCAGAAAATTCAAATGAACTATGTGAACCAGCTTCAAAGAAATTATGTAGACCCAAAGGATTACTTGATGTTACTAAACAATTAGATTTTGACAGCACAGTACATGTGCCACCCTCATTCACTGTAACTGATTTATCAAGCATTCCCATAGAACAAGCTATTGAACAGTCATTGGCCAAGATATTCCA 1600
     ----:----|----:----|----:----|----:----|----:----|----:----|----:----|----:----|----:----|----:----|----:----|----:----|----:----|----:----|----:----|----:----|----:----|----:----|----:----|----:----|
1401 CGTAGAGAGAAAGTCTTTTAAGTTTACTTGATACACTTGGTCGAAGTTTCTTTAATACATCTGGGTTTCCTAATGAACTACAATGATTTGTTAATCTAAAACTGTCGTGTCATGTACACGGTGGGAGTAAGTGACATTGACTAAATAGTTCGTAAGGGTATCTTGTTCGATAACTTGTCAGTAACCGGTTCTATAAGGT 1600
     C R R K * F I * I F * T F W S * L F * T S G F S * K I N S F L * I K V A C Y M H W G * E S Y S I * A N G Y F L S N F L * Q G L Y E   F6
     A D E R E S F E F S S H S G A E F F N H L G L P N S S T V L C N S K S L V T C T G G E N V T V S K D L M G M S C A I S C D N A L I N W   F5
     H M K E K L F N L H V I H V L K L S I I Y V W L I V Q H * * V I L N Q C C L V H A V R M * Q L Q N I L C E W L V L * Q V T M P W S I G   F4

     A Q F V L V Y Q L L L L * L I V N I F F A D H V Y L H G Y * M Q V F V Q F V E N Q * * * M R * A F L Q A N Y P * C M I F Y M F H V G   F1
     V H N L S W Y T N C S C Y N S L * T Y F L Q I M Y I F M A I E C R C L S N L * R T S D D E * D K H S Y R P I I L D V * Y F T C F M * V   F2
     C T I C L G I P T A P A I T H C K H I F C R S C I S S W L L N A G V C P I C R E P V M M N E I S I L T G Q L S L M Y D I L H V S C R F   F3
1601 GTGCACAATTTGTCTTGGTATACCAACTGCTCCTGCTATAACTCATTGTAAACATATTTTTTGCAGATCATGTATATCTTCATGGCTATTGAATGCAGGTGTTTGTCCAATTTGTAGAGAACCAGTGATGATGAATGAGATAAGCATTCTTACAGGCCAATTATCCTTGATGTATGATATTTTACATGTTTCATGTAGGT 1800
     ----:----|----:----|----:----|----:----|----:----|----:----|----:----|----:----|----:----|----:----|----:----|----:----|----:----|----:----|----:----|----:----|----:----|----:----|----:----|----:----|
1601 CACGTGTTAAACAGAACCATATGGTTGACGAGGACGATATTGAGTAACATTTGTATAAAAAACGTCTAGTACATATAGAAGTACCGATAACTTACGTCCACAAACAGGTTAAACATCTCTTGGTCACTACTACTTACTCTATTCGTAAGAATGTCCGGTTAATAGGAACTACATACTATAAAATGTACAAAGTACATCCA 1800
     L A C N T K T Y W S S R S Y S M T F M N K A S * T Y R * P * Q I C T N T W N T S F W H H H I L Y A N K C A L * G Q H I I N * M N * T P   F6
     H V I Q R P I G V A G A I V * Q L C I K Q L D H I D E H S N F A P T Q G I Q L S G T I I F S I L M R V P W N D K I Y S I K C T E H L   F5
     T C L K D Q Y V L Q E Q * L E N Y V Y K K C I M Y I K M A I S H L H K D L K Y L V L S S S H S L C E * L G I I R S T H Y K V H K M Y T   F4

     L I I V M K S * D L I K F Q I M K M Y V N M E S I P Q S F * H L H Q * * E E E R G C K K C L F M I A R Q N M L S K K G L K K * K A Q L   F1
     * * L * * S L R T * * N F R S * K C M * I W K V S L K A F D T F I N S K K R K G V A K N A Y L * L Q G K I C * A K K A * R S R K L S *   F2
     D N C N E V L G L D K I S D H E N V C K Y G K Y P S K L L T P S S I V R R G K G L Q K M P I Y D C K A K Y V K Q K R L K E V E S S V   F3
1801 TTGATAATTGTAATGAAGTCTTAGGACTTGATAAAATTTCAGATCATGAAAATGTATGTAAATATGGAAAGTATCCCTCAAAGCTTTTGACACCTTCATCAATAGTAAGAAGAGGAAAGGGGTTGCAAAAAATGCCTATTTATGATTGCAAGGCAAAATATGTTAAGCAAAAAAGGCTTAAAGAAGTAGAAAGCTCAGTT 2000
     ----:----|----:----|----:----|----:----|----:----|----:----|----:----|----:----|----:----|----:----|----:----|----:----|----:----|----:----|----:----|----:----|----:----|----:----|----:----|----:----|
1801 AACTATTAACATTACTTCAGAATCCTGAACTATTTTAAAGTCTAGTACTTTTACATACATTTATACCTTTCATAGGGAGTTTCGAAAACTGTGGAAGTAGTTATCATTCTTCTCCTTTCCCCAACGTTTTTTACGGATAAATACTAACGTTCCGTTTTATACAATTCGTTTTTTCCGAATTTCTTCATCTTTCGAGTCAA 2000
     K I T I F D * S K I F N * I M F I Y T F I S L I G * L K Q C R * * Y S S S L P Q L F H R N I I A L C F I N L L P K F F Y F A * N   F6
     N S L Q L S T K P S S L I E S * S F T H L Y P F Y G E F S K V G E D I T L L P F P N C F I G I * S Q L A F Y T L C F L S L S T S L E T   F5
     Q Y N Y H L R L V Q Y F K L D H F H I Y I H F T D R L A K S V K M L L L F L F P T A F F A * K H N C P L I H * A F F A * L L L F S L   F4

     N N F V L Q K M R I K Q M F Y F S C L F I I * R A K M I I D L N K L M M F G W E G E S E N * Q V M N A L Q S E * T L Y R V K L N I K H   F1
     T I L F F K K * G * N R C F I F H A Y S S F E E P K * * I * T N * * C L D G R G S Q K I N K S * M P C N Q N R H F T E * N S I * S   F2
     E Q F C S S K N E D K T D V L F F M L I H H L K S Q N D N R F E Q I N D V W M G G G V R K L T S H E C L A I R I D T L Q S K T Q Y K A   F3
2001 GAACAATTTTGTTCTTCAAAAAATGAGGATAAAACAGATGTTTTATTTTTCATGCTTATTCATCATTTGAAGAGCCAAAATGATAATAGATTTGAACAAATTAATGATGTTTGGATGGGAGGGGGAGTCAGAAAATTAACAAGTCATGAATGCCTTGCAATCAGAATAGACACTTTACAGAGTAAAACTCAATATAAAGC 2200
     ----:----|----:----|----:----|----:----|----:----|----:----|----:----|----:----|----:----|----:----|----:----|----:----|----:----|----:----|----:----|----:----|----:----|----:----|----:----|----:----|
2001 CTTGTTAAAACAAGAAGTTTTTTACTCCTATTTTGTCTACAAAATAAAAAGTACGAATAAGTAGTAAACTTCTCGGTTTTACTATTATCTAAACTTGTTTAATTACTACAAACCTACCCTCCCCCTCAGTCTTTTAATTGTTCAGTACTTACGGAACGTTAGTCTTATCTGTGAAATGTCTCATTTTGAGTTATATTTCG 2200
     F L K T R * F I L F C I N * K E H K N M M Q L A L I I S K F L N I I N P H S P S D S F * C T M F A K C D S Y V S * L T F S L I F   F6
     S C N Q E E F F S S L V S T K N K M S I * * K F L W F S L L N S C I L S T Q I P P P T L F N V L * S H R A I L I S V K C L L V * Y L A   F5
     Q V I K N K L F H P Y F L H K I K * A * E D N S S G F H Y Y I Q V F * H H K S P L P L * F I L L D H I G Q L * F L C K V S Y F E I Y L   F4

     S M I S * R L R D V T H L * L H M H * M K L S V S M S L A Q L D I Q L K V R I K S L T I I T H Q S K * I E N V L L H P * T I H L N H   F1
     T V * F L K G * G M * P I C S S I C I R * N * V S V C P W L S * I F N * R S G S S H * L L S H T S Q S K * R M Y Y F T P R Q Y I * T I   F2
     Q Y D F L K A K G C N P F V A P Y A L D E I E C Q Y V P G S V R Y S I E G Q D Q V I N Y Y H T P V K V N R E C T T S P L D N T F E P L   F3
2201 ACAGTATGATTTCTTAAAGGCTAAGGGATGTAACCCATTTGTAGCTCCATATGCATTAGATGAAATTGAGTGTCAGTATGTCCCTGGCTCAGTTAGATATTCAATTGAAGGTCAGGATCAAGTCATTAACTATTATCACACACCAGTCAAAGTAAATAGAGAATGTACTACTTCACCCCTAGACAATACATTTGAACCAT 2400
     ----:----|----:----|----:----|----:----|----:----|----:----|----:----|----:----|----:----|----:----|----:----|----:----|----:----|----:----|----:----|----:----|----:----|----:----|----:----|----:----|
2201 TGTCATACTAAAGAATTTCCGATTCCCTACATTGGGTAAACATCGAGGTATACGTAATCTACTTTAACTCACAGTCATACAGGGACCGAGTCAATCTATAAGTTAACTTCCAGTCCTAGTTCAGTAATTGATAATAGTGTGTGGTCAGTTTCATTTATCTCTTACATGATGAAGTGGGGATCTGTTATGTAAACTTGGTA 2400
     C L I E * L S L S T V W K Y S W I C * I F N L T L I D R A * N S I * N F T L I L D N V I I V C W D F Y I S F T S S * G * V I C K F W   F6
     C Y S K K F A L P H L G N T A G Y A N S S I S H * Y T G P E T L Y E I S P * S * T M L * * V G T L T F L S H V V E G R S L V N S G   F5
     V T H N R L P * P I Y G M Q L E M H M L H F Q T D T H G Q S L * I N L Q L D P D L * * S N D C V L * L L Y L I Y * K V G L C Y M Q V M   F4

     * T L * M Y S L L V L L N * Q L L M * R A V V G D I R M Q * P K H C M N * K M T * L Q D Y N N T I L I L M I L Q S C * R P T L R M G L   F1
```

```
R H C K C I P C L Y S * I S N S * C E G L S L E I S G C N S Q N I A * I R K * H N C R I T T T Q Y * S L * S Y N P V K D L H * G W G * F2
D I V N V F P A C T P E L A T P N V K G C R W R Y P D A I A K T L H E L E N D I I A G L Q Q H N I D P Y D P T I L L K T Y I K D G A F3
2401 TAGACATTGTAAATGTATTCCCTGCTTGTACTCCTGAATTAGCAACTCCTAATGTGAAGGGCTGTCGTTGGAGATATCCGGATGCAATAGCCAAAACATTGCATGAATTAGAAAATGACATAATTGCAGGATTACAACAACACAATATTGATCCTTATGATCCTACAATCCTGTTAAAGACCTACATTAAGGATGGGGCT 2600
----:----|----:----|----:----|----:----|----:----|----:----|----:----|----:----|----:----|----:----|----:----|----:----|----:----|----:----|----:----|----:----|----:----|----:----|----:----|----:----|
2401 ATCTGTAACATTTACATAAGGGACGAACATGAGGACTTAATCGTTGAGGATTACACTTCCCGACAGCAACCTCTATAGGCCTACGTTATCGGTTTTGTAACGTACTTAATCTTTTACTGTATTAACGTCCTAATGTTGTTGTGTTATAACTAGGAATACTAGGATGTTAGGACAATTTCTGGATGTAATTCCTACCCCGA 2600
* V N Y I Y E R S T S R F * C S R I H L A T T P S I R I C Y G F C Q M F * F I V Y N C S * L L V I N I R I I R C D Q * L G V N L I P S F6
N S M T F T N G A Q V G S N A V G L T F P Q R Q L Y G S A I A L V N C S N S F S M I A P N C C C L I S G * S G V I R N F V * M L S P A F5
L C Q L H I G Q K Y E Q I L L E * H S P S D N S I D P H L L W F M A H I L F H C L Q L I V V V C Y Q D K H D * L G T L S R C * P H P F4

M V L V M C L Y T K K * G I D I C Q T R H S D F P F V Y I E * R Q Y * E M M K R S Q S L L K K G Q I L S K Q T D L Y L K Q F V T K I I F1
W S W * C V C T Q R S R G * I F A R Q G I Q I F L L C I * N R G N I R R * * K G H N L C * K K A K F C Q N K Q T C T * S N L * R K * F2
D G L G D V S V H K E V G D R Y L P D K A F R F S F C V Y R I E A I L G D D E K V T I F V E K R P N S V K T N R P V L E A I C D E N N F3
2601 GATGGTCTTGGTGATGTGTCTGTACACAAAGAAGTAGGGGATAGATATTTGCCAGACAAGGCATTCAGATTTTCCTTTTGTGTATATAGAATAGAGGCAATATTAGGAGATGATGAAAAGGTCACAATCTTTGTTGAAAAAAGGCCAAATTCTGTCAAAACAAACAGACCTGTACTTGAAGCAATTTGTGACGAAAATAA 2800
----:----|----:----|----:----|----:----|----:----|----:----|----:----|----:----|----:----|----:----|----:----|----:----|----:----|----:----|----:----|----:----|----:----|----:----|----:----|----:----|
2601 CTACCAGAACCACTACACAGACATGTGTTTCTTCATCCCCTATCTATAAACGGTCTGTTCCGTAAGTCTAAAAGGAAAACACATATATCTTATCTCCGTTATAATCCTCTACTACTTTTCCAGTGTTAGAAACAACTTTTTTCCGGTTTAAGACAGTTTTGTTTGTCTGGACATGAACTTCGTTAAACACTGCTTTTATT 2800
I T K T I H R Y V F F Y P I S I Q W V L C E S K G K T Y I S Y L C Y * S I I F L D C D K N F F P W I R D F C V S R Y K F C N T V F I F6
S P R P S T D T C L S T P S L Y K G S L A N L N E K Q T Y L I S A I N P S S S F T V I K T S F L G F E T L V F L G T S S A I Q S S F L F5
Q H D Q H H T Q V C L L L P Y I N A L C P M * I K R K H I Y F L P L I L L H H F P * L R Q Q F F A L N Q * F L C V Q V Q L L K H R F Y F4

M A P V L F A Y C Q * K M K G L I * K V R L S G F K Q S L A E K A C T V F S N S M I G E K L D R A N S G L A G S G S N Y L C T L C D F1
S W L Q Y S L P I A N R K * K V L Y E R * D Y Q D S N R V W L R R H V L F F L T Q * L V K N W I G P T V D W L V V G P T I Y A H C V M F2
H G S S T L C L L P I E N E R S Y M K G K I I R I Q T E F G * E G M Y C F F * L N D W * K I G * G Q Q W I G W * W V Q L S M H I V * C F3
2801 TCATGGCTCCAGTACTCTTTGCCTATTGCCAATAGAAAATGAAAGGTCTTATATGAAAGGTAAGATTATCAGGATTCAAACAGAGTTTGGCTGAGAAGGCATGTACTGTTTTTTCTAACTCAATGATTGGTGAAAAATTGGATAGGGCCAACAGTGGATTGGCTGGTAGTGGGTCCAACTATCTATGCACATTGTGTGAT 3000
----:----|----:----|----:----|----:----|----:----|----:----|----:----|----:----|----:----|----:----|----:----|----:----|----:----|----:----|----:----|----:----|----:----|----:----|----:----|----:----|
2801 AGTACCGAGGTCATGAGAAACGGATAACGGTTATCTTTTACTTTCCAGAATATACTTTCCATTCTAATAGTCCTAAGTTTGTCTCAAACCGACTCTTCCGTACATGACAAAAAAGATTGAGTTACTAACCACTTTTTAACCTATCCCGGTTGTCACCTAACCGACCATCACCCAGGTTGATAGATACGTGTAACACACTA 3000
I M A G T S K A * Q W Y F I F P R I H F T L N D P N L C L K A S F A H V T K E L E I I P S F N S L A L L P N A P L P D L * R H V N H S F6
* P E L V R Q R N G I S F S L D * I F P L I I L I * V S N P Q S P M Y Q K K * S L S Q H F I P Y P W C H I P Q Y H T W S D I C M T H F5
D H S W Y E K G I A L L F H F T K Y S L Y S * * S E F L T Q S L L C T S N K R V * H N T F F Q I P G V T S Q S T T P G V I * A C Q T I F4

A T R E T A L S N L G N F V I N R S Y A E T A E I A E Y M R I N P D N L T K A D L N K I S K G I K S T P L L Q A E S I E K G I D A T H F1
Q Q E K R P Y P I W V I L S * I V H M L K Q Q K L L S T * G * I L I I L P R L I * T R F L R A L N Q P L S S R L N Q * K R A * M L L M F2
N K R N G P I Q F G * F C H K S F I C * N S R N C * V H E D K S * * S Y Q G * F K Q D F * G H * I N P S P P G * I N R K G H R C Y S F3
3001 GCAACAAGAGAAACGGCCCTATCCAATTTGGGTAATTTTGTCATAAATCGTTCATATGCTGAAACAGCAGAAATTGCTGAGTACATGAGGATAAATCCTGATAATCTTACCAAGGCTGATTTAAACAAGATTTCTAAGGGCATTAAATCAACCCCTCTCCTCCAGGCTGAATCAATAGAAAAGGGCATAGATGCTACTCA 3200
----:----|----:----|----:----|----:----|----:----|----:----|----:----|----:----|----:----|----:----|----:----|----:----|----:----|----:----|----:----|----:----|----:----|----:----|----:----|----:----|
3001 CGTTGTTCTCTTTGCCGGGATAGGTTAAACCCATTAAAACAGTATTTAGCAAGTATACGACTTTGTCGTCTTTAACGACTCATGTACTCCTATTTAGGACTATTAGAATGGTTCCGACTAAATTTGTTCTAAAGATTCCCGTAATTTAGTTGGGGAGAGGAGGTCCGACTTAGTTATCTTTTCCCGTATCTACGATGAGT 3200
A V L S V A R D L K P L K T M F R E Y A S V A S I A S Y M L I F G S L R V L A S K F L I E L P M L D V G R R W A S D I S F P M S A V * F6
H L L L F P G I W N P Y N Q * L D N M H Q F L L F Q Q T C S S L D Q Y D * W P Q N L C S K * P C * I L G E G G P Q I L L F P C L H * E F5
C C S F R G * G I Q T I K D Y I T * I S F C C F N S L V H P Y I R I I K G L S I * V L N R L A N F * G R E E L S F * Y F L A Y I S S F4

A D I N C G S F F R K L I I R E I V Q I N K W E M S G D V K S I I K D G E V K F D L H L K K T V G I N P Q L M M P G N Y A R A I F D S F1
Q I S T V G L F F E S * S L G K * C K L I N G K * V G M * N L L L K M G R L S L I Y I S R K Q * A * T H S * * C L V T M H E Q S L T F2
C R Y Q L W V F F S K A N H * G N S A N * * M G N E W G C E I Y Y * R W G G * V * F T S Q E N S R H K P T V D D A W * L C T S N L * L F3
3201 TGCAGATATCAACTGTGGGTCTTTTTTTCGAAAGCTAATCATTAGGGAAATAGTGCAAATTAATAAATGGGAAATGAGTGGGGATGTGAAATCTATTATTAAAGATGGGGAGGTTAAGTTTGATTTACATCTCAAGAAAACAGTAGGCATAAACCCACAGTTGATGATGCCTGGTAACTATGCACGAGCAATCTTTGACT 3400
----:----|----:----|----:----|----:----|----:----|----:----|----:----|----:----|----:----|----:----|----:----|----:----|----:----|----:----|----:----|----:----|----:----|----:----|----:----|----:----|
3201 ACGTCTATAGTTGACACCCAGAAAAAAAGCTTTCGATTAGTAATCCCTTTATCACGTTTAATTATTTACCCTTTACTCACCCCTACACTTTAGATAATAATTTCTACCCCTCCAATTCAAACTAAATGTAGAGTTCTTTTGTCATCCGTATTTGGGTGTCAACTACTACGGACCATTGATACGTGCTCGTTAGAAACTGA 3400
A S I L Q P D K K R F S I M L S I T C I L L H S I L P S T F D I I L S P S T L N S K C R L F V T P M F G C N I I G P L * A R A I K S F6
H L Y * S H T K K E F A L * * P F L A F * Y I P F S H P H S I * * * L H P P * T Q N V D * S F L L C L G V T S S A Q Y S H V L L R Q S F5
M C I D V T P R K K S L * D N P F Y H L N I F P P F H T P I H F R N N F I P L N L K I * M E L F C Y A Y V W L Q H H R T V I C S C D K V F4

K N E N S V L D L I E D D E R K C K V R L L L Q K F R F M R M I Y R A V D P K V Q Y P S E T K M Y K E T A I E F G K I L I N D F D Y F1
L K M R I L F L I * S K M M R E S A K L D Y Y F K N S D S * E * F I E L L T P K Y S I H Q R L K C T K K L P * S L G K Y * L M T L I M F2
* K * E F C S * F N R R * * E K V Q S * I I T S K I Q I H E N D L * S C * P Q S T V S I R D * N V Q R N C H R V W E N I D * * L * L C F3
3401 CTAAAAATGAGAATTCTGTTCTTGATTTAATCGAAGATGATGAGAGAAAGTGCAAAGTTAGATTATTACTTCAAAAATTCAGATTCATGAGAATGATTTATAGAGCTGTTGACCCCAAAGTACAGTATCCATCAGAGACTAAAATGTACAAAGAAACTGCCATAGAGTTTGGGAAAATATTGATTAATGACTTTGATTAT 3600
----:----|----:----|----:----|----:----|----:----|----:----|----:----|----:----|----:----|----:----|----:----|----:----|----:----|----:----|----:----|----:----|----:----|----:----|----:----|----:----|
3401 GATTTTTACTCTTAAGACAAGAACTAAATTAGCTTCTACTACTCTCTTTCACGTTTCAATCTAATAATGAAGTTTTTAAGTCTAAGTACTCTTACTAAATATCTCGACAACTGGGGTTTCATGTCATAGGTAGTCTCTGATTTTACATGTTTCTTTGACGGTATCTCAAACCCTTTTATAACTAATTACTGAAACTAATA 3600
E L F S F E T R S K I S S S S L F H L T L N N S * F N L N M L I I * L A T S G L T C Y G D S V L I Y L S V A M S N P F I N I L S K S * F6
* F H S N Q E Q N L R L H H S F T C L * I I V E F I * I * S F S K Y L Q Q G W L V T D M L S * F T C L F Q W L T Q S F I S * H S Q N F5
R F I L I R N K I * D F I I L S L A F N S * * K L F E S E H S H N I S S N V G F Y L I W * L S F H V F F S G Y L K P F Y Q N I V K I I F4

A K W P N Y I H K V I E H V Q E L I E N E N G P G T V G G L S S E G N E G G N K I F R H F R K N L A R R G S T Y G G L R D V L W G H W F1
L N G P I T Y I K * L N M Y R N * L K M K M D L E L L E G L V V K G M K E G T R F F D I L G R I W Q G G V V H M G V * E M Y F G V I G F2
* M A Q L H T * S D * T C T G I N * K * K W T W N C W R A * * * R E * R R E Q D F S T F * E E F G K E G * Y I W G S K R C T L G S L F3
3601 GCTAAATGGCCCAATTACATACATAAAGTGATTGAACATGTACAGGAATTAATTGAAAATGAAAATGGACCTGGAACTGTTGGAGGGCTTAGTAGTGAAGGGAATGAAGGAGGGAACAAGATTTTTCGACATTTTAGGAAGAATTTGGCAAGGAGGGGTAGTACATATGGGGGTCTAAGAGATGTACTTTGGGGTCATTG 3800
----:----|----:----|----:----|----:----|----:----|----:----|----:----|----:----|----:----|----:----|----:----|----:----|----:----|----:----|----:----|----:----|----:----|----:----|----:----|----:----|
3601 CGATTTACCGGGTTAATGTATGTATTTCACTAACTTGTACATGTCCTTAATTAACTTTTACTTTTACCTGGACCTTGACAACCTCCCGAATCATCACTTCCCTTACTTCCTCCCTTGTTCTAAAAAGCTGTAAAATCCTTCTTAAACCGTTCCTCCCCATCATGTATACCCCCAGATTCTCTACATGAAACCCCAGTAAC 3800
A L H G L * M C L T I S C T C S N I S F S F P G P V T P P S L L S P F S P P F L I K R C K L F F K A L L P L V Y P P R L S T S Q P * Q F6
H * I A W N C V Y L S Q V H V P I L Q F H F H V Q F Q Q L A * Y H L S H L L S C S K E V N * S S N P L S P Y Y M H P D L L H V K P D N F5
S F P G I V Y M F H N F M Y L F * N F I F I S R S S N S P K T T F P I F S P V L N K S M K P L I Q C P P T T C I P T * S I Y K P T M F4

L Y S S P S L T K L S A K S K S E Q R C S A C R S L G H N I R N C P S S V E N V * I C Y S I Y V S I S E N I L C Y S V H S N F I D T F F1
Y T V V H L * Q N Y Q L N Q N L N K D A V H V D L W D I I L E T V H L L * K M S K F V I A Y M Y L F L K I F C V T V Y T P I L L T P F2
V I Q * S I S N K T I S * I K I * T K M Q C M * I F G T * Y * K L S I F C R K C L N L L * H I C I Y F * K Y F V L Q C T L Q F Y * H L F3
3801 GTTATACAGTAGTCCATCTCTAACAAAACTATCAGCTAAATCAAAATCTGAACAAAGATGCAGTGCATGTAGATCTTTGGGACATAATATTAGAAACTGTCCATCTTCTGTAGAAAATGTCTAAATTTGTTATAGCATATATGTATCTATTTCTGAAAATATTTTGTGTTACAGTGTACACTCCAATTTTATTGACACCT 4000
----:----|----:----|----:----|----:----|----:----|----:----|----:----|----:----|----:----|----:----|----:----|----:----|----:----|----:----|----:----|----:----|----:----|----:----|----:----|----:----|
3801 CAATATGTCATCAGGTAGAGATTGTTTTGATAGTCGATTTAGTTTTAGACTTGTTTCTACGTCACGTACATCTAGAAACCCTGTATTATAATCTTTGACAGGTAGAAGACATCTTTTACAGATTTAAACAATATCGTATATACATAGATAAAGACTTTTATAAAACACAATGTCACATGTGAGGTTAAAATAACTGTGGA 4000
N Y L L G D R V F S D A L D F D S C L H L A H L D K P C L I L F Q G D E T S F T * I Q * L M Y T D I E S F I K H * L T C E L K I S V F6
T I C Y D M E L L V I L * I L I Q V F I C H M Y I K P V Y Y * F S D M K Q L F H R F K N Y C I H I * K Q F Y K T N C H V S W N * Q C R F5
P * V T T W R * C F * * S F * F R F L S A T C T S R Q S M I N S V T W R R Y F I D L N T I A Y I Y R N R F I N Q T V T Y V G I K N V G F4

P A D V G V N N Y L V H S C I F Q L I V C S L F I L I I L N H I F L Q M L M A G I A * P R Q H I L F Y F Y Y S N I H L F Y S T * Q N F1
F Q Q M L E L I I I W Y I H V Y F S S L F A V C L F * * Y * I I F S F R C * W P G * P S L D S T S S F T S T I V I F I C F I P L N R I F2
S S R C W S * * L S G T F M Y I S A H C L Q F V Y F N N I K S Y F P S D V D G R D S L A S T A H P L L L L L * * Y S F V L F H L T E Y F3
4001 TTCCAGCAGATGTTGGAGTTAATAATTATCTGGTACATTCATGTATATTTCAGCTCATTGTTTGCAGTTTGTTTATTTTAATAATATTAAATCATATTTTCCTTCAGATGTTGATGGCCGGGATAGCCTAGCCTCGACAGCACATCCTCTTTTACTTCTACTATAGTAATATTCATTTGTTTATTCCACTTAACAGAAT 4200
----:----|----:----|----:----|----:----|----:----|----:----|----:----|----:----|----:----|----:----|----:----|----:----|----:----|----:----|----:----|----:----|----:----|----:----|----:----|----:----|
4001 AAGGTCGTCTACAACCTCAATTATTAATAGACCATGTAAGTACATATAAAGTCGAGTAACAAACGTCAAACAAATAAAATTATTATAATTTAGTATAAAAGGAAGTCTACAACTACCGGCCCTATCGGATCGGAGCTGTCGTGTAGGAGAAAATGAAGATGATATCATTATAAGTAAACAAAATAAGGTGAATTGTCTTA 4200
K G A S T P T L L * R T C E H I N * S M T Q L K N I K I I N F * I K R * I N I A P I A * G R C C M R K * K * * L L I * K N * E V * C F F6
E L L H Q L * Y N D P V N M Y I E A * Q K C N T * K L L I L D Y K G E S T S P R S L R A E V A C G R K S R S Y Y Y E N T K N W K V S F5
K W C I N S N I I I Q Y M * T Y K L E N N A T Q K N * Y Y * I M N E K L H Q H G P Y G L R S L V D E K V E V I T I N M Q K I G S L L I F4

T L F A L M L P C A R L * V K H F D * Y S C V I F I N K * Y M S E I I * L S N N N K K H N N S T * F L S D F F K L I * Y F D M F F I S F1
L Y L H * C C H V Q G Y R S N T L T N I H V * F S L T S N T C L R L F N F Q I I T K N I I I L H S F C L I S S N * S D I L I C F S F L F2
F I C I N V A M C K V I G Q T L * L I F M C N F H * Q V I H V * D Y L T F K * * Q K T * * F Y I V F V * F L Q T D L I F * Y V F H F F3
4201 ACTTTATTTGCATTAATGTTGCCATGTGCAAGGTTATAGGTCAAACACTTTGACTAATATTCATGTGTAATTTTCATTAACAAGTAATACATGTCTGAGATTATTTAACTTTCAAATAATAACAAAAAACATAATAATTCTACATAGTTTTTGTCTGATTTCTTCAAACTGATCTGATATTTTGATATGTTTTTCATTTC 4400
----:----|----:----|----:----|----:----|----:----|----:----|----:----|----:----|----:----|----:----|----:----|----:----|----:----|----:----|----:----|----:----|----:----|----:----|----:----|----:----|
4201 TGAAATAAACGTAATTACAACGGTACACGTTCCAATATCCAGTTTGTGAAACTGATTATAAGTACACATTAAAAGTAATTGTTCATTATGTACAGACTCTAATAAATTGAAAGTTTATTATTGTTTTTTGTATTATTAAGATGTATCAAAAACAGACTAAAGAAGTTTGACTAGACTATAAAACTATACAAAAAGTAAAG 4400
V K N A N I N G H A L N Y T L C K S * Y E H T I K M L L Y Y M D S I I * S E F L L L F C L L E V Y N K D S K K L S I Q Y K S I N K M E F6
Y K I Q M L T A M H L T I P * V S Q S I N M H L K * * C T I C T Q S * K V K L Y Y C F V Y Y N * M T K T Q N R * V S R I N Q Y T K * K F5
S * K C * H Q W T C P * L D F V K V L I * T Y N E N V L L V H R L N N L K * I I V F F M I I R C L K Q R I E E F Q D S I K I H K E N F4

W L F F P S F Y F L L N S L C I C T F P L V I Q M L K H Q H K I D E A I Y D M V L R T E P M K L E R F L E N L * C Y I L F Q S H M D S F1
G C S S P L S T F C * T V F A Y V L S L W * S R C * N T N T K L M K P S M T W S F A L N P * N W R D F W R I S N V T F Y S K V T W T F2
L A V L P L F L L F A E Q S L H M Y F P F G N P D V K T P T Q N * * S H L * H G P S H * T H E I G E I S G E S L M L H S I P K S H G L F3
4401 TTGGCTGTTCTTCCCCTCTTTCTACTTTTTGCTGAACAGTCTTTGCATATGTACTTTCCCTTTGGTAATCCAGATGTTAAAACACCAACACAAAATTGATGAAGCCATCTATGACATGGTCCTTCGCACTGAACCCATGAAATTGGAGAGATTTCTGGAGAATCTCTAATGTTACATTCTATTCCAAAGTCACATGGACT 4600
----:----|----:----|----:----|----:----|----:----|----:----|----:----|----:----|----:----|----:----|----:----|----:----|----:----|----:----|----:----|----:----|----:----|----:----|----:----|----:----|
4401 AACCGACAAGAAGGGGAGAAAGATGAAAAACGACTTGTCAGAAACGTATACATGAAAGGGAAACCATTAGGTCTACAATTTTGTGGTTGTGTTTTAACTACTTCGGTAGATACTGTACCAGGAAGCGTGACTTGGGTACTTTAACCTCTCTAAAGACCTCTTAGAGATTACAATGTAAGATAAGGTTTCAGTGTACCTGA 4600
Q S N K G E K * K K S F L R Q M H V K G K T I W I N F C W C L I S S A M * S M T R R V S G M F N S L N R S F R * H * M R N W L * M S F6
K A T R G R K R S K A S C D K C I Y K G K P L G S T L V G V C F Q H L W R H C P G E C Q V W S I P S I E P S D R I N C E I G F D C P S F5
R P Q E E G R E V K Q Q V T K A Y T S E R Q Y D L H * F V L V F N I F G D I V H D K A S F G H F Q L S K Q L I E L T V N * E L T V H V F4

V S * V L M C T Q S S F * Y F H L L P * C S H Y L E S L K C I L Q S Y G F C L L Q Y D S Q R D L S N C M T I C W D S N L A I L P F G F1
R Y H R F * C V H K V V F S I S T Y Y H N A V I T * S H * S A S C S P M D F V Y F N M I H R E I C Q I V * Q Y V G I Q T W Q F S H L A F2
G I I G F D V Y T K * F L V F P P T T I M Q S L L R V I E V H P A V L W I L F T S I * F T E R S V K L Y D N M L G F K P G N S P I W L F3
4601 CGGTATCATAGGTTTTGATGTGTACACAAAGTAGTTTTTAGTATTTCCACCTACTACCATAATGCAGTCATTACTTAGAGTCATTGAAGTGCATCCTGCAGTCCTATGGATTTTGTTTACTTCAATATGATTCACAGAGAGATCTGTCAAATTGTATGACAATATGTTGGGATTCAAACCTGGCAATTCTCCCATTTGGC 4800
----:----|----:----|----:----|----:----|----:----|----:----|----:----|----:----|----:----|----:----|----:----|----:----|----:----|----:----|----:----|----:----|----:----|----:----|----:----|----:----|
4601 GCCATAGTATCCAAAACTACACATGTGTTTCATCAAAAATCATAAAGGTGGATGATGGTATTACGTCAGTAATGAATCTCAGTAACTTCACGTAGGACGTCAGGATACCTAAAACAAATGAAGTTATACTAAGTGTCTCTCTAGACAGTTTAACATACTGTTATACAACCCTAAGTTTGGACCGTTAAGAGGGTAAACCG 4800
E T D Y T K I H V C L L K * Y K W R S G Y H L * * K S D N F H M R C D * P N Q K S * Y S E C L S R D F Q I V I H Q S E F R A I R G N P F6
P I M P K S T Y V F Y N K T N G G V V M I C D N S L T M S T C G A T R H I K N V E I H N V S L D T L N Y S L I N P N L G P L E G M Q F5
R Y * L N Q H T C L T T K L I E V * * W L A T M V * L * Q L A D Q L G I S K T * K L I I * L S I Q * I T H C Y T P I * V Q C N E W K A F4

S Y S G K N L Q L * T C Y L Q E S N M S D K K E S N L * C L * M * T S S D Q V R K Y C H L K F S * L * H H Q V G D A D Q V G S Q C K S F1
L T L V K T S N Y K H V I C K K A T * V I R K K V I C N V S E C K L Q A I K * E S I V I * N F H D Y D I I K W E T L I R L A A S V S H F2
L L W * K P P I I N M L F A R K Q H E * * E R K * F V M S L N V N F K R S S E K V L S S K I F M T M T S S S G R R * S G W Q P V * V F3
4801 TCTTACTCTGGTAAAAACCTCCAATTATAAACATGTTATTTGCAAGAAAGCAACATGAGTGATAAGAAAGAAAGTAATTTGTAATGTCTCTGAATGTAAACTTCAAGCGATCAAGTGAGAAAGTATTGTCATCTAAAATTTTCATGACTATGACATCATCAAGTGGGAGACGCTGATCAGGTTGGCAGCCAGTGTAAGTC 5000
----:----|----:----|----:----|----:----|----:----|----:----|----:----|----:----|----:----|----:----|----:----|----:----|----:----|----:----|----:----|----:----|----:----|----:----|----:----|----:----|
4801 AGAATGAGACCATTTTTGGAGGTTAATATTTGTACAATAAACGTTCTTTCGTTGTACTCACTATTCTTTCTTTCATTAAACATTACAGAGACTTACATTTGAAGTTCGCTAGTTCACTCTTTCATAACAGTAGATTTTAAAAGTACTGATACTGTAGTAGTTCACCCTCTGCGACTAGTCCAACCGTCGGTCACATTCAG 5000
E * E P L F R W N Y V H * K C S L L M L S L F S L L K Y H R Q I Y V E L S * T L F Y Q * R F N E H S H C * * T P S A S * T P L W H L D F6
S K S Q Y F G G I I F M N N A L F C C S H Y S L F Y N T I D R F T F K L R D L S F T N D D L I K M V I V D D L P L R Q D P Q C G T Y T F5
R V R T F V E L * L C T I Q L F A V H T I L F F T I Q L T E S H L S * A I L H S L I T M * F K * S * S M M L H S V S I L N A A L T L F4

Y L Q L C K L T F S P Q N I Q Q N G R L G V * L R V H L F L S I R K F L C P V D R I Y H L C K F F E M V I S M W L F S S W A * * L H H F1
```

```
      T  S  N  Y  V  N  *  L  F  L  L  K  I  F  S  R  M  E  G  S  G  Y  N  F  G  Y  I  Y  F  F  P  F  E  S  F  Y  V  Q  L  I  E  F  T  I  F  A  S  F  L  K  W  *  F  Q  C  G  C  F  P  H  G  H  S  D  S  T    F2
     I  P  P  T  M  *  I  D  F  F  S  S  K  Y  S  A  E  W  K  A  R  G  I  T  S  G  T  F  I  S  F  H  S  K  V  F  M  S  S  *  *  N  L  P  S  L  Q  V  F  *  N  G  D  F  N  V  A  V  F  L  M  G  I  V  T  P  P   F3
5001 ATACCTCCAACTATGTAAATTGACTTTTTCTCCTCAAAATATTCAGCAGAATGGAAGGCTCGGGGTATAACTTCGGGTACATTTATTTCTTTCCATTCGAAAGTTTTTATGTCCAGTTGATAGAATTTACCATCTTTGCAAGTTTTTTGAAATGGTGATTTCAATGTGGCTGTTTTCCTCATGGGCATAGTGACTCCACC 5200
     ----:----|----:----|----:----|----:----|----:----|----:----|----:----|----:----|----:----|----:----|----:----|----:----|----:----|----:----|----:----|----:----|----:----|----:----|----:----|----:----|
5001 TATGGAGGTTGATACATTTAACTGAAAAAGAGGAGTTTTATAAGTCGTCTTACCTTCCGAGCCCCATATTGAAGCCCATGTAAATAAAGAAAGGTAAGCTTTCAAAAATACAGGTCAACTATCTTAAATGGTAGAAACGTTCAAAAAACTTTACCACTAAAGTTACACCGACAAAAGGAGTACCCGTATCACTGAGGTGG 5200
      Y  R  W  S  H  L  N  V  K  E  G  *  F  I  *  C  F  P  L  S  P  T  Y  S  R  T  C  K  N  R  E  M  R  F  N  K  H  G  T  S  L  I  *  W  R  Q  L  N  K  S  I  T  I  E  I  H  S  N  E  E  H  A  Y  H  S  W    F6
      M  G  G  V  I  Y  I  S  K  K  E  E  F  Y  E  A  S  H  F  A  R  P  I  V  E  P  V  N  I  E  K  W  E  F  T  K  I  D  L  Q  Y  F  K  G  D  K  C  T  K  Q  F  P  S  K  L  T  A  T  K  R  M  P  M  T  V  G  G   F5
     *  V  E  L  *  T  F  Q  S  K  R  R  L  I  N  L  L  I  S  P  E  P  Y  L  K  P  Y  M  *  K  K  G  N  S  L  K  *  T  W  N  I  S  N  V  M  K  A  L  K  K  F  H  H  N  *  H  P  Q  K  G  *  P  C  L  S  E  V   F4

      I  K  L  T  Y  N  C  Q  Y  K  S  V  N  D  Q  I  F  P  L  E  G  F  H  I  V  L  T  L  K  E  M  Y  I  L  V  V  Q  S  H  N  Y  T  L  N  P  V  L  K  V  Y  K  G  Q  S  P  W  L  A  V  L  Y  C  I  H  P  K    F1
     I  *  N  *  H  I  I  A  N  I  S  Q  *  M  T  R  S  S  L  W  K  V  S  I  L  F  *  P  *  K  R  C  T  F  L  L  Y  N  L  T  I  I  L  *  T  L  F  *  K  Y  I  K  V  S  L  H  G  *  L  C  C  I  V  S  T  P  N   F2
      Y  K  T  D  I  *  L  P  I  *  V  S  E  *  P  D  L  P  S  G  R  F  P  Y  C  F  D  L  E  R  D  V  H  S  C  C  T  I  S  Q  L  Y  F  E  P  C  F  E  S  I  *  R  S  V  S  M  V  S  C  A  V  L  Y  P  P  Q  T   F3
5201 ATATAAAACTGACATATAATTGCCAATATAAGTCAGTGAATGACCAGATCTTCCCTCTGGAAGGTTTCCATATTGTTTTGACCTTGAAAGAGATGTACATTCTTGTTGTACAATCTCACAATTATACTTTGAACCCTGTTTTGAAAGTATATAAAGGTCAGTCTCCATGGTTAGCTGTGCTGTATTGTATCCACCCCAAA 5400
     ----:----|----:----|----:----|----:----|----:----|----:----|----:----|----:----|----:----|----:----|----:----|----:----|----:----|----:----|----:----|----:----|----:----|----:----|----:----|----:----|
5201 TATATTTTGACTGTATATTAACGGTTATATTCAGTCACTTACTGGTCTAGAAGGGAGACCTTCCAAAGGTATAACAAAACTGGAACTTTCTCTACATGTAAGAACAACATGTTAGAGTGTTAATATGAAACTTGGGACAAAACTTTCATATATTTCCAGTCAGAGGTACCAATCGACACGACATAACATAGGTGGGGTTT 5400
     W  I  F  S  V  Y  L  Q  W  Y  L  D  T  F  S  W  I  K  G  R  S  P  K  W  I  T  K  V  K  F  S  I  Y  M  R  T  T  C  D  *  L  *  V  K  F  G  T  K  F  T  Y  L  P  *  D  G  H  N  A  T  S  Y  Q  I  W  G  L   F6
      Y  L  V  S  M  Y  N  G  I  Y  T  L  S  H  G  S  R  G  E  P  L  N  G  Y  Q  K  S  R  S  L  S  T  C  E  Q  Q  V  I  E  C  N  Y  K  S  G  Q  K  S  L  I  Y  L  D  T  E  M  T  L  Q  A  T  N  Y  G  G  W    F5
      M  Y  F  Q  C  I  I  A  L  I  L  *  H  I  V  L  D  E  R  Q  F  T  E  M  N  N  Q  G  Q  F  L  H  V  N  K  N  Y  L  R  V  I  I  S  Q  V  R  N  Q  F  Y  I  F  T  L  R  W  P  *  S  H  Q  I  T  D  V  G  F   F4

     Q  R  F  Y  V  H  L  P  L  L  L  L  T  V  G  Q  G  K  L  T  Q  *  F  Q  I  L  *  Y  Q  *  Y  *  H  H  G  C  L  L  N  N  Q  S  E  I  N  P  L  S  P  P  T  K  I  R  P  I  V  W  P  H  Q  N  I  S  K  T  L    F1
      K  D  F  M  F  I  Y  H  C  C  C  *  L  *  G  R  E  N  *  P  S  S  F  K  F  C  D  I  S  N  I  N  I  M  A  A  Y  *  I  I  N  L  K  *  I  L  *  V  L  P  R  R  F  V  R  S  S  G  P  T  K  I  *  V  R  H  Y   F2
      K  I  L  C  S  F  T  T  A  A  V  D  C  R  A  G  K  I  D  P  V  V  S  N  F  V  I  S  V  I  L  T  S  W  L  L  T  K  *  S  I  *  N  K  S  F  E  S  S  H  E  D  S  S  D  R  L  A  P  P  K  Y  K  *  D  T    F3
5401 CAAAGATTTTATGTTCATTTACCACTGCTGCTGTTGACTGTAGGGCAGGGAAAATTGACCCAGTAGTTTCAAATTTTGTGATATCAGTAATATTAACATCATGGCTGCTTACTAAATAATCAATCTGAAATAAATCCTTTGAGTCCTCCCACGAAGATTCGTCCGATCGTCTGGCCCCACCAAAATATAAGTAAGACACT 5600
     ----:----|----:----|----:----|----:----|----:----|----:----|----:----|----:----|----:----|----:----|----:----|----:----|----:----|----:----|----:----|----:----|----:----|----:----|----:----|----:----|
5401 GTTTCTAAAATACAAGTAAATGGTGACGACGACAACTGACATCCCGTCCCTTTTAACTGGGTCATCAAAGTTTAAAACACTATAGTCATTATAATTGTAGTACCGACGAATGATTTATTAGTTAGACTTTATTTAGGAAACTCAGGAGGGTGCTTCTAAGCAGGCTAGCAGACCGGGGTGGTTTTATATTCATTCTGTGA 5600
      C  L  N  *  T  *  K  G  S  S  S  N  V  T  P  C  P  F  N  V  W  Y  N  *  I  K  H  Y  *  Y  Y  *  C  *  P  Q  K  S  F  L  *  D  S  I  F  G  K  L  G  G  V  F  I  R  G  I  T  Q  G  W  W  F  I  L  L  V  S   F6
     V  F  I  K  H  E  N  V  V  A  A  T  S  Q  L  A  P  F  I  S  G  T  T  E  F  K  T  I  D  T  I  N  V  D  H  S  S  V  L  Y  D  I  Q  F  L  D  K  S  D  E  W  S  S  E  D  S  R  R  A  G  G  F  Y  L  Y  S  V   F5
      L  S  K  I  N  M  *  W  Q  Q  Q  Q  S  Y  P  L  S  F  Q  G  L  L  K  L  N  Q  S  I  L  L  I  L  M  M  A  A  *  *  I  I  L  R  F  Y  I  R  Q  T  R  G  R  L  N  T  R  D  D  P  G  V  L  I  Y  T  L  C    F4

      R  S  R  H  L  Y  H  H  F  A  C  Q  F  L  Y  I  H  V  P  P  V  E  K  N  H  R  V  L  I  L  S  P  F  F  F  W  L  I  S  P  S  L  N  L  S  I  *  M  V  R  L  A  F  *  *  H  V  Y  D  L  F  V  Y  Y  H  M  L   F1
      A  L  V  I  C  I  I  I  L  P  V  N  F  Y  T  Y  M  S  L  R  W  K  K  I  T  V  F  *  F  *  V  P  S  F  S  G  *  F  P  P  L  *  I  Y  R  F  K  W  *  D  L  H  S  D  S  T  C  T  I  Y  L  F  I  I  I  C    F2
     T  L  S  S  F  V  S  S  F  C  L  S  I  S  I  H  T  C  P  S  G  G  K  K  S  P  C  F  N  S  K  S  L  L  F  L  V  D  F  P  L  S  K  S  I  D  L  N  G  K  T  C  I  L  I  A  R  V  R  F  I  C  L  L  S  Y  A   F3
5601 ACGCTCTCGTCATTTGTATCATCATTTTGCCTGTCAATTTCTATACATACATGTCCCTCCGGTGGAAAAAAATCACCGTGTTTTAATTCTAAGTCCCTTCTTTTTCTGGTTGATTTCCCCCTCTCTAAATCTATCGATTTAAATGGTAAGACTTGCATTCTGATAGCACGTGTACGATTTATTTGTTTATTATCATATGC 5800
     ----:----|----:----|----:----|----:----|----:----|----:----|----:----|----:----|----:----|----:----|----:----|----:----|----:----|----:----|----:----|----:----|----:----|----:----|----:----|----:----|
5601 TGCGAGAGCAGTAAACATAGTAGTAAAACGGACAGTTAAAGATATGTATGTACAGGGAGGCCACCTTTTTTTAGTGGCACAAAATTAAGATTCAGGGAAGAAAAAGACCAACTAAAGGGGGAGAGATTTAGATAGCTAAATTTACCATTCTGAACGTAAGACTATCGTGCACATGCTAAATAAACAAATAATAGTATACG 5800
      R  E  R  *  K  Y  *  *  K  A  Q  *  N  R  Y  M  C  T  G  G  T  S  F  F  *  R  T  K  I  R  L  G  K  K  K  Q  N  I  E  G  E  R  F  R  D  I  *  I  T  L  S  A  N  Q  Y  C  T  Y  S  K  N  T  *  *  *  I   F6
      V  S  E  D  N  T  D  D  N  Q  R  D  I  E  I  C  V  H  G  E  P  P  F  F  D  G  H  K  L  E  L  D  R  R  K  R  T  S  K  G  R  E  L  D  I  S  K  F  P  L  V  Q  M  R  I  A  R  T  R  N  I  Q  K  N  D  Y  A   F5
     *  A  R  T  M  Q  I  M  M  K  G  T  L  K  *  V  Y  M  D  R  R  H  F  F  I  V  T  N  *  N  *  T  G  E  K  E  P  Q  N  G  G  R  *  I  *  R  N  L  H  Y  S  K  C  E  S  L  V  H  V  I  *  K  N  I  I  M  H   F4

      I  T  T  C  A  C  A  L  T  H  F  R  *  E  H  S  E  Y  C  K  R  L  L  V  *  Y  D  X                                                                                                                                  F1
     *  *  R  P  A  H  A  H  L  R  I  S  G  R  S  I  A  S  T  V  N  V  C  *  C  S  T  T                                                                                                                                   F2
      N  N  D  L  R  M  R  T  Y  A  F  P  V  G  A  *  R  V  L  *  T  F  V  S  V  V  R  P                                                                                                                                  F3
5801 TAATAACGACCTGCGCATGCGCACTTACGCATTTCCGGTAGGAGCATAGCGAGTACTGTAAACGTTTGTTAGTGTAGTACGACC 5884
     ----:----|----:----|----:----|----:----|----:----|----:----|----:----|----:----|----
5801 ATTATTGCTGGACGCGTACGCGTGAATGCGTAAAGGCCATCCTCGTATCGCTCATGACATTTGCAAACAATCACATCATGCTGG 5884
     S  I  V  V  Q  A  H  A  S  V  C  K  R  Y  S  C  L  S  Y  Q  L  R  K  N  T  Y  Y  S                                                                                                                                   F6
      L  L  S  R  R  M  R  V  *  A  N  G  T  P  A  Y  R  T  S  Y  V  N  T  L  T  T  R  G                                                                                                                                  F5
      *  Y  R  G  A  C  A  C  K  R  M  E  P  L  L  M  A  L  V  T  F  T  Q  *  H  L  V  V                                                                                                                                  F4
```

Pinctada imbricata (Pim)

WGS: NIJJ01003325.1

28400-33451
Pinctada imbricata breed pearl oyster-OUG PIN_chr10_6649, whole genome shotgun sequence

```
       I L F T A T E F R M E N F K I I P F K S I D E K K V K G T R K R S E I D I I N G D F F P P E G H C A V L I K E V Q S E D D L L K S F L  F1
      S Y S Q L L N L E W K I S R L Y P L N R L M K K R * K E P E N D R R * T L * M E I F F L Q K D T A Q Y * L K K C N Q R T I Y * S H F Y   F2
     L I H S Y * I * N G K F Q D Y T L * I D * * K K G E R N Q K T I G D R H Y K W R F F S S R R T L R S I D * R S A I R G R F I E V I S    F3
   1 ATCTTATTCACAGCTACTGAATTTAGAATGGAAAATTTCAAGATTATACCCTTTAAATCGATTGATGAAAAAAAGGTGAAAGGAACCAGAAAACGATCGGAGATAGACATTATAAATGGAGATTTTTTTCCTCCAGAAGGACACTGCGCAGTATTGATTAAAGAAGTGCAATCAGAGGACGATTTATTGAAGTCATTTCT 200
     ----:----|----:----|----:----|----:----|----:----|----:----|----:----|----:----|----:----|----:----|----:----|----:----|----:----|----:----|----:----|----:----|----:----|----:----|----:----|----:----|
   1 TAGAATAAGTGTCGATGACTTAAATCTTACCTTTTAAAGTTCTAATATGGGAAATTTAGCTAACTACTTTTTTTCCACTTTCCTTGGTCTTTTGCTAGCCTCTATCTGTAATATTTACCTCTAAAAAAAGGAGGTCTTCCTGTGACGCGTCATAACTAATTTCTTCACGTTAGTCTCCTGCTAAATAACTTCAGTAAAGA 200
      X K N V A V S N L I S F K L I I G K L D I S S F F T F P V L F R D S I S M I F P S K K G G S P C Q A T N I L S T C D S S S K N F D N R  F6
     X R I * L * Q I * F P F N * S * V R * I S Q H F F P S L F W F V I P S L C * L H L N K E E L L V S R L I S * L L A I L P R N I S T M E   F5
     D * E C S S F K S H F I E L N Y G K F R N I F F L H F S G S F S R L Y V N Y I S I K K R W F S V A C Y Q N F F H L * L V I * Q L * K    F4

       Y F G G S R H T E E S T W S L G S S P F I I S Y I V A E N D V E I S Q I K K C N T L G A S F P S L A S S A V I V G S N D K I Y V W G G  F1
      I L G D P G I R R S Q H G L L G P H H S * F H T S W L K M M L K S V K * K N V T H L E H H F H L L P L Q Q * L L D Q M I K Y M Y G E   F2
     I F W G I Q A Y G G V N M V S W V L T I H N F I H R G * K * C * N Q S N K K M * H T W S I I S I S C L F S S N C W I K * * N I C M G R    F3
 201 ATATTTTGGGGGATCCAGGCATACGGAGGAGTCAACATGGTCTCTTGGGTCCTCACCATTCATAATTTCATACATCGTGGCTGAAAATGATGTTGAAATCAGTCAAATAAAAAAATGTAACACACTTGGAGCATCATTTCCATCTCTTGCCTCTTCAGCAGTAATTGTTGGATCAAATGATAAAATATATGTATGGGGAG 400
     ----:----|----:----|----:----|----:----|----:----|----:----|----:----|----:----|----:----|----:----|----:----|----:----|----:----|----:----|----:----|----:----|----:----|----:----|----:----|----:----|
 201 TATAAAACCCCCTAGGTCCGTATGCCTCCTCAGTTGTACCAGAGAACCCAGGAGTGGTAAGTATTAAAGTATGTAGCACCGACTTTTACTACAACTTTAGTCAGTTTATTTTTTTACATTGTGTGAACCTCGTAGTAAAGGTAGAGAACGGAGAAGTCGTCATTAACAACCTAGTTTACTATTTTATATACATACCCCTC 400
      Y K P P D L C V S S D V H D R P D E G N M I E Y M T A S F S T S I L * I F F H L V S P A D N G D R A E E A T I T P D F S L I Y T H P  F6
     I N Q P I W A Y P P T L M T E Q T R V M * L K M C R P Q F H H Q F * D F L F I Y C V Q L M M E M E Q R K L L L Q Q I L H Y F I H I P L   F5
     * I K P S G P M R L L * C P R K P G * W E Y N * V D H S F I I N F D T L Y F F T V C K S C * K W R K G R * C Y N N S * I I F Y I Y P S    F4

       M D T S S M K A L H D L Y L L K W N D A K I Q V Q L V Q N S K D D M C V T Q F K Q T G D V P L G R F G H S L T K L N D Q Y A I L H G  F1
      G W I L H L * K R C M T C I F L N G M M Q R S K F N L Y K T V K M I C V * L N S N R Q V M S H * A D L D I H L Q N * M I S M Q F Y M G   F2
     D G Y F I Y E S V A * P V S S * M E * C K D P S S T C T K Q * R * Y V C D S I Q T D R * C P T R Q I W T F T Y K I K * S V C N F T W G    F3
 401 GGATGGATACTTCATCTATGAAAGCGTTGCATGACCTGTATCTTCTTAAATGGAATGATGCAAAGATCCAAGTTCAACTTGTACAAAACAGTAAAGATGATATGTGTGTGACTCAATTCAAACAGACAGGTGATGTCCCACTAGGCAGATTTGGACATTCACTTACAAAATTAAATGATCAGTATGCAATTTTACATGG 600
     ----:----|----:----|----:----|----:----|----:----|----:----|----:----|----:----|----:----|----:----|----:----|----:----|----:----|----:----|----:----|----:----|----:----|----:----|----:----|----:----|
 401 CCTACCTATGAAGTAGATACTTTCGCAACGTACTGGACATAGAAGAATTTACCTTACTACGTTTCTAGGTTCAAGTTGAACATGTTTTGTCATTTCTACTATACACACACTGAGTTAAGTTTGTCTGTCCACTACAGGGTGATCCGTCTAAACCTGTAAGTGAATGTTTTAATTTACTAGTCATACGTTAAAATGTACC 600
      P I S V E D I F A N C S R Y R R L H F S A F I W T * S T C F L L S S I H T V * N L C V P S T G S P L N P C E S V F N F S * Y A I K C P  F6
     S P Y K M * S L T A H G T D E * I S H H L S G L E V Q V F C Y L H Y T H S E I * V S L H H G V L C I Q V N V * L I L H D T H L K V H   F5
     P H I S * R H F R Q M V Q I K K F P I I C L D L N L K Y L V T F I I H T H S L E F L C T I D W * A S K S M * K C F * I I L I C N * M P    F4

       G I S L P F R E A G L V S P F Q Q A C N D G K F Y L F D L N T L S W S F I N V P D V D S R A F H S A T Y D H R M N C I Y I I G G V T  F1
      G Y H Y H F V K Q D D L Y P H F N K L A M M G N S I Y L I * T P * A G P L S M S Q M S I A G H F I V L L M T I G * I V Y T * L E G S P   F2
     D I I T I S * S R M T C I P I S T S L Q * W E I L F I * F E H L E L V L Y Q C P R C R * Q G I S * C Y L * P * D E L Y I H N W R G H    F3
 601 GGGATATCATTACCATTTCGTGAAGCAGGATGACTTGTATCCCCATTTCAACAAGCTTGCAATGATGGGAAATTCTATTTATTTGATTTGAACACCTTGAGCTGGTCCTTTATCAATGTCCCAGATGTCGATAGCAGGGCATTTCATAGTGCTACTTATGACCATAGGATGAATTGTATATACATAATTGGAGGGGTCAC 800
     ----:----|----:----|----:----|----:----|----:----|----:----|----:----|----:----|----:----|----:----|----:----|----:----|----:----|----:----|----:----|----:----|----:----|----:----|----:----|----:----|
 601 CCCTATAGTAATGGTAAAGCACTTCGTCCTACTGAACATAGGGGTAAAGTTGTTCGAACGTTACTACCCTTTAAGATAAATAAACTAAACTTGTGGAACTCGACCAGGAAATAGTTACAGGGTCTACAGCTATCGTCCCGTAAAGTATCACGATGAATACTGGTATCCTACTTAACATATATGTATTAACCTCCCCAGTG 800
      P I D N G N R S A P H S T D G N * C A Q L S P F N * K N S K F V K L Q D K I L T G S T S L L A N * L A V * S W L I F Q I Y M I P P T V  F6
     P S I M V M E H L L I V Q T I G M E V L K C H H S I R N I Q N S C R S S T R * * H G L H R Y C P M E Y H * K H G Y S S N Y I C L Q L P *   F5
     P Y * * W K T F C S S K Y G W K L L S A I I P F E I * K I Q V G Q A P G K D I D W I D I A P C K M T S S I V M P H I T Y V Y N S P D    F4

       Y Q E C T P R S R L P V H D V L V L R I H E K S D M T L D R V I F Q S P P N I E N L G I S Y H S V C L D D N N L I I V G G F T Q I G Q  F1
      T R N A P Q G H A C Q F M M S W Y * E S M R K V T * P W T G S Y F N H H Q T * K T L G Y H T T V S V * M I I T * L L L G V S H K * D   F2
     L P G M H P K V T L A S S * C P G T K N P * E K * H D L G Q G H I S I T T K H R K P W D I I P Q C L F R * * * P D Y C W G F H T N R T    F3
 801 CTACCAGGAATGCACCCCAAGGTCACGCTTGCCAGTTCATGATGTCCTGGTACTAAGAATCCATGAGAAAAGTGACATGACCTTGGACAGGGTCATATTTCAATCACCACCAAACATAGAAAACCTTGGGATATCATACCACAGTGTCTGTTTAGATGATAATAACCTGATTATTGTGGGGGGTTTCACACAAATAGGAC 1000
     ----:----|----:----|----:----|----:----|----:----|----:----|----:----|----:----|----:----|----:----|----:----|----:----|----:----|----:----|----:----|----:----|----:----|----:----|----:----|----:----|
 801 GATGGTCCTTACGTGGGGTTCCAGTGCGAACGGTCAAGTACTACAGGACCATGATTCTTAGGTACTCTTTTCACTGTACTGGAACCTGTCCCAGTATAAAGTTAGTGGTGGTTTGTATCTTTTGGAACCCTATAGTATGGTGTCACAGACAAATCTACTATTATTGGACTAATAACACCCCCCAAAGTGTGTTTATCCTG 1000
      * W S H V G L D R K G T * S T R T S L I W S F L S M V K S L T M N * D G G F M S F R P I D Y W L T Q K S S L R I I T P P K V C I P  F6
     R G P I C G L T V R S A L E H H G P V L F G H S F H C S R P C P * I E I V V L C L F G Q S I M G C H R N L H Y Y G S * Q Q P N * V F L V   F5
     G V L F A G W P * A Q W N M I D Q Y * S D M L F T V H G Q V P D Y K L * W W V Y F V K P Y * V V T D T * I I I V Q N N N P T E C L Y S    F4

       L K T D V K P D L N P Y V L L M D L T F K S I L Y A D I S P E Y L T A G N S L L C L S S D C F L S C G G I I K S Y F V Y T T K A M K  F1
      S L K Q M * N L T L I H M F Y S W I * L S N P Y Y M L I L V Q N I * Q Q G I H C F V Y Q V T A S S H V V V * S K V T L F I Q Q K P * N   F2
     A * N R C K T * P * S I C F T H G F N F Q I H T I C * Y * S R I S N S R E F I A L F I K * L L P L M W W Y N Q K L L C L Y N K S H E T    F3
1001 AGCTTAAAACAGATGTAAAACCTGACCTTAATCCATATGTTTTACTCATGGATTTAACTTTCAAATCCATACTATATGCTGATATTAGTCCAGAATATCTAACAGCAGGGAATTCATTGCTTTGTTTATCAAGTGACTGCTTCCTCTCATGTGGTGGTATAATCAAAAGTTACTTTGTTTATACAACAAAAGCCATGAAA 1200
     ----:----|----:----|----:----|----:----|----:----|----:----|----:----|----:----|----:----|----:----|----:----|----:----|----:----|----:----|----:----|----:----|----:----|----:----|----:----|----:----|
1001 TCGAATTTTGTCTACATTTTGGACTGGAATTAGGTATACAAAATGAGTACCTAAATTGAAAGTTTAGGTATGATATACGACTATAATCAGGTCTTATAGATTGTCGTCCCTTAAGTAACGAAACAAATAGTTCACTGACGAAGGAGAGTACACCACCATATTAGTTTTCAATGAAACAAATATGTTGTTTTCGGTACTTT 1200
      C S L V S T F G S R L G Y T K S M S K V K L D M S Y A S I L G S Y R V A P F E N S Q K D L S Q K R E H P P I I L L * K T * V V F A M F  F6
     A * F L H L V Q G * D M K H K V * P N L K * I W V I H Q Y * D L I D L L L S N M A K N I L H S S G R M H H Y L * F N S Q K Y L L W S   F5
     L K F C I Y F R V K I W I N * E H I * S E F G Y * I S I N T W F I * C C P I * Q K T * * T V A E E * T T T Y D F T V K N I C C F G H F    F4

       P I P C D L Q S E C L I D N S P E I S P I S W I Q C E G T C K R W L H Q F C I G L L N K D L P K G K Y I C L T C K S S R K R K S K K *  F1
      L F L V T F S Q N A L L I I H Q R Y P Q F H G Y N V K G L V K G G F T N F A L D F * T K T C L K G N I F A * L A N Q V E K E N Q K N   F2
     Y S L * P S V R M P Y * * F T R D I P N F M D T M * R D L * K V A S P I L H W T F E Q R L A * R E I Y L P D L Q I K * K K K I K K I    F3
1201 CCTATTCCTTGTGACCTTCAGTCAGAATGCCTTATTGATAATTCACCAGAGATATCCCCAATTTCATGGATACAATGTGAAGGGACTTGTAAAAGGTGGCTTCACCAATTTTGCATTGGACTTTTGAACAAAGACTTGCCTAAAGGGAAATATATTTGCCTGACTTGCAAATCAAGTAGAAAAAGAAAATCAAAAAATA 1400
     ----:----|----:----|----:----|----:----|----:----|----:----|----:----|----:----|----:----|----:----|----:----|----:----|----:----|----:----|----:----|----:----|----:----|----:----|----:----|----:----|
1201 GGATAAGGAACACTGGAAGTCAGTCTTACGGAATAACTATTAAGTGGTCTCTATAGGGGTTAAAGTACCTATGTTACACTTCCCTGAACATTTTCCACCGAAGTGGTTAAAACGTAACCTGAAAACTTGTTTCTGAACGGATTTCCCTTTATATAAACGGACTGAACGTTTAGTTCATCTTTTTCTTTTAGTTTTTTAT 1400
      G I G Q S R * D S H R I S L E G S I D G I E H I C H S P V Q L L H S * W N Q M P S K F L S K G L P F Y I Q R V Q L D L L F L F D F F Y  F6
     V * E K H G E T L I G * Q Y N V L S I G L K M S V I H L S K Y F T A E G I K C Q V K S C L S A * L S I Y K G S K C I L Y F F F I L F I   F5
     R N R T V K L * F A K N I I * W L Y G W N * P Y L T F P S T F P P K V L K A N S K Q V F V Q R F P F I N A Q S A F * T S F S F * F F    F4

       S M Y I L I C R H V * V L Y C I D I L Q T H I I Q Y K I N C S M C R S F T L Y A * S H V M T F L * T Y A A L L L V T C L V C K P E Q F  F1
      A C T F * Y V D M F K Y C I A * T F C R L T * F S T R L I V Q C V E A L R C T H D L M L * P S C K H M L H F F L L L V L F A S L S S   F2
     E H V H F N M * T C L S T V L H R H F A D S H N S V Q D * L F N V * K L Y V V R M I S C Y D L L V N I C C T S S C Y L S C L Q A * A V    F3
1401 GAGCATGTACATTTTAATATGTAGACATGTTTAAGTACTGTATTGCATAGACATTTTGCAGACTCACATAATTCAGTACAAGATTAATTGTTCAATGTGTAGAAGCTTTACGTTGTACGCATGATCTCATGTTATGACCTTCTTGTAAACATATGCTGCACTTCTTCTTGTTACTTGTCTTGTTTGCAAGCCTGAGCAGT 1600
     ----:----|----:----|----:----|----:----|----:----|----:----|----:----|----:----|----:----|----:----|----:----|----:----|----:----|----:----|----:----|----:----|----:----|----:----|----:----|----:----|
1401 CTCGTACATGTAAAATTATACATCTGTACAAATTCATGACATAACGTATCTGTAAAACGTCTGAGTGTATTAAGTCATGTTCTAATTAACAAGTTACACATCTTCGAAATGCAACATGCGTACTAGAGTACAATACTGGAAGAACATTTGTATACGACGTGAAGAAGAACAATGAACAGAACAAACGTTCGGACTCGTCA 1600
      L M Y M K I H L C T * T S Y Q M S M K C V * M I * Y L I L Q E I H L L K V N Y A H D * T I V K K Y V Y A A S R R T V R Q T Q L G S C  F6
     S C T C K L I Y V H K L V T N C L C K A S E C L E T C S * N N L T Y F S * T T R M I E H * S R R T F M H Q V E E Q * K D Q K C A Q A T   F5
     L A H V N * Y T S M N L Y Q I A Y V N Q L S V Y N L V L N I T * H T S A K R Q V C S R M N H G E Q L C I S C K K K N S T K N A L R L L    F4

       R T A V K P V T P K H I S K S P I S V S S P G Q I L P K M S K Y F I S P F I T L P T * P S H S S S P I F I F N Q F L N M L N N F V N  F1
      S G L L * S Q * P Q S T S R S P P * * V S P L L A K F F L K C L N I L F L P S L P S P L N P P T V P A P F S S S I S S * T C S I T L * I   F2
     Q D C C K A S D P K A H L E V P H K C L L S W P N S S * N V * I F Y F S L H Y P P H L T L P Q F Q P H F H L Q S V L E H A Q * L C E Y    F3
1601 TCAGGACTGCTGTAAAGCCAGTGACCCCAAAGCACATCTCGAAGTCCCCCATAAGTGTCTCCTCTCCTGGCCAAATTCTTCCTAAAATGTCTAAATATTTTATTTCTCCCTTCATTACCCTCCCCACTAACCCCTCCCCACAGTTCCAGCCCCATTTTCATCTTCAATCAGTTCTTGAACATGCTCAATAACTTTGTGAAT 1800
     ----:----|----:----|----:----|----:----|----:----|----:----|----:----|----:----|----:----|----:----|----:----|----:----|----:----|----:----|----:----|----:----|----:----|----:----|----:----|----:----|
1601 AGTCCTGACGACATTTCGGTCACTGGGGTTTCGTGTAGAGCTTCAGGGGGTATTCACAGAGGAGAGGACCGGTTTAAGAAGGATTTTACAGATTTATAAAATAAAGAGGGAAGTAATGGGAGGGGTGATTGGGGAGGGGTGTCAAGGTCGGGGTAAAAGTAGAAGTTAGTCAAGAACTTGTACGAGTTATTGAAACACTTA 1800
      N L V A T F G T V G F C M E F D G M L T E E G P W I R G L I D L Y K I E G K M V R G V * G E W L E L G M K M K L * N K F M S L L K T F  F6
     * S Q Q L A L S G L A C R S T G W L H R R E Q G F E E * F T * I N * K E R * * G G W K V R G C N W G W K * R * D T R S C A * Y S Q S   F5
     E P S S Y L W H G W L V D R L G G Y T D G R R A L N K R F H R F I K N R G E N G E G S L G G V T G A G N E D E I L E Q V H E I V K H I    F4

       I I R P S S I T E I F K K C M S K * N C S L F V D F C F F R I L S F G V T G S I H L S H K S K F * E * I * Q V L S S D L I I N Q T * N  F1
      * L G H L A * L K F S R S A C P N E T A V C L * I F V S S G Y * V L G S L A L Y T F R I N R N F E S R S D K Y F L L T S L S T K H K I   F2
     N * A I * H N * N F Q E V H V Q M K L Q F V C R F L F L Q D T E F W G H W L Y T P F A * I E I L R V D L T S T F F * P H Y Q P N I K    F3
1801 ATAATTAGGCCATCTAGCATAACTGAAATTTTCAAGAAGTGCATGTCCAAATGAAACTGCAGTTTGTTTGTAGATTTTTGTTTCTTCAGGATACTGAGTTTTGGGGTCACTGGCTCTATACACCTTTCGCATAAATCGAAATTTTGAGAGTAGATCTGACAAGTACTTTCTTCTGACCTCATTATCAACCAAACATAAAA 2000
     ----:----|----:----|----:----|----:----|----:----|----:----|----:----|----:----|----:----|----:----|----:----|----:----|----:----|----:----|----:----|----:----|----:----|----:----|----:----|----:----|
1801 TATTAATCCGGTAGATCGTATTGACTTTAAAAGTTCTTCACGTACAGGTTTACTTTGACGTCAAACAAACATCTAAAAACAAAGAAGTCCTATGACTCAAAACCCCAGTGACCGAGATATGTGGAAAGCGTATTTAGCTTTAAAACTCTCATCTAGACTGTTCATGAAAGAAGACTGGAGTAATAGTTGGTTTGTATTTT 2000
      I I L G D L M V S I K L F H M D L H F Q L K N T S K Q K K L I S L K P T V P E I C R E C L D F N Q S Y I Q C T S E E S R M I L W V Y F  F6
     Y L * A M * C L Q F K * S T C T W I F S C N T Q L N K N R * S V S N Q P * Q S * V G K A Y I S I K L T S R V L V K K Q G * * * G F M F   F5
     Y N P W R A Y S F N E L L A H G F S V A T Q K Y I K T E E P Y Q T K P D S A R Y V K R M F R F K S L L D S L Y K R R V E N D V L C L    F4

       T L L I V W V K Q * P * V I T R H H * L R I Y A N F L F K A I I K C Y F C I L N D * F N V L C N I P C I D M * N F S N Y E F F K K * A  F1
      L C S L S G G S N N D L E * L P D I I N * G F M P I S F L R Q L S N V T S A F L M I D L M S S V T S H V L T C E I S L I M S F L K N E   F2
     Y S A H C L G Q T M T L S N Y P T S L T E D L C Q F P F F * G N Y Q M L L L H S * * L I * C P L * H P M Y * H V K F L * L * V F * K M S    F3
2001 TACTCTGCTCATTGTCTGGGTCAAACAATGACCTTGAGTAATTACCCGACATCATTAACTGAGGATTTATGCCAATTTCCTTTTTAAGGCAATTATCAAATGTTACTTCTGCATTCTTAATGATTGATTTAATGTCCTCTGTAACATCCCATGTATTGACATGTGAAATTTCTCTAATTATGAGTTTTTTAAAAAATGAG 2200
     ----:----|----:----|----:----|----:----|----:----|----:----|----:----|----:----|----:----|----:----|----:----|----:----|----:----|----:----|----:----|----:----|----:----|----:----|----:----|----:----|
2001 ATGAGACGAGTAACAGACCCAGTTTGTTACTGGAACTCATTAATGGGCTGTAGTAATTGACTCCTAAATACGGTTAAAGGAAAAATTCCGTTAATAGTTTACAATGAAGACGTAAGAATTACTAACTAAATTACAGGAGACATTGTAGGGTACATAACTGTACACTTTAAAGAGATTAATACTCAAAAAATTTTTTACTC 2200
      V R S M T Q T L C H G Q T I V R C * * S L I * A L K R K L A I I L H * K Q M R L S Q N L T R Q L M G H I S M H F K E L * S N K L F H  F6
     Y E A * Q R P * V I V K L L * G V D N V S S K H W N G K * P L * * I N S R C E * H N I * H G R Y C G M Y Q C T F N R * N H T K * F I L   F5
     I S Q E N D P D F L S R S Y N G S M M L Q P N I G I E K K L C N D F T V E A N K I I S K I D E T V D W T N V H S I E R I I L K K F F S    F4

       T V Y I S M G C I * A F L T G I C L E N R N S F H S F R Q F F K R R F * * I I W I C F D I I C Y F C S I S Y R S * N A E * T Q L R L  F1
      P Q F I S A W V A S K H F * L A S V W R I G T V F T P L D S F S R E D F D K L S G F A L T * F A I F V V S A T D L E M L N E P S * D *   F2
     H S L Y Q H G L H L S I S D W H L F G E * E Q F S L L * T V F Q E K I L I N Y L D L L * H N L L F L * Y Q L Q I L K C * M N P A E T E    F3
2201 CCACAGTTTATATCAGCATGGGTTGCATCTAAGCATTTCTGACTGGCATCTGTTTGGAGAATAGGAACAGTTTTCACTCCTTTAGACAGTTTTTCAAGAGAAGATTTTGATAAATTATCTGGATTTGCTTTGACATAATTTGCTATTTTTGTAGTATCAGCTACAGATCTTGAAATGCTGAATGAACCCAGCTGAGACTG 2400
     ----:----|----:----|----:----|----:----|----:----|----:----|----:----|----:----|----:----|----:----|----:----|----:----|----:----|----:----|----:----|----:----|----:----|----:----|----:----|----:----|
2201 GGTGTCAAATATAGTCGTACCCAACGTAGATTCGTAAAGACTGACCGTAGACAAACCTCTTATCCTTGTCAAAAGTGAGGAAATCTGTCAAAAAGTTCTCTTCTAAAACTATTTAATAGACCTAAACGAAACTGTATTAAACGATAAAAACATCATAGTCGATGTCTAGAACTTTACGACTTACTTGGGTCGACTCTGAC 2400
      A V T * I L M P Q M * A N R V P M Q K S F L F L K * E K L C N K L L N Q Y I Q I I Q K S M I Q * K Q L I L * L D Q F A S H V W S L S  F6
     W L K Y * C P N C R L M E S Q C R N P S Y S C N E S R * V T K * S F I K I F * R S K S Q C L K S N K Y Y * S C I K F H Q I F G A S V   F5
     G C N I D A H T A D L C K Q S A D T Q L I P V T K V G K S L K E L S S K S L N D P N A K V Y N A I K T T D A V S R S I S F S G L Q S Q    F4
```

```
      S  C  L  S  S  S  I  A  Q  G  T  Q  E  G  *  T  T  P  C  *  T  P  T  *  P  V  *  F  F  I  Y  H  *  V  I  K  E  *  I  V  P  S  P  A  I  L  V  L  N  S  *  F  C  L  S  *  K  V  F  P  A  Q  L  F  E  D  T   F1
       A  V  S  L  V  A  *  H  K  V  H  K  K  D  E  P  L  P  A  R  P  P  H  D  L  S  N  F  S  S  I  I  E  L  *  K  N  K  L  C  L  L  Q  P  F  W  F  *  T  H  N  F  V  F  H  K  K  S  F  L  L  N  Y  L  K  T  Q   F2
        L  S  L  *  *  H  S  T  R  Y  T  R  R  M  N  H  S  L  L  D  P  H  M  T  C  L  I  F  H  L  S  L  S  Y  K  R  I  N  C  A  F  S  S  H  F  G  F  E  L  I  I  L  S  F  I  K  S  L  S  C  S  I  I  *  R  H    F3
2401 AGCTGTCTCTCTAGTAGCATAGCACAAGGTACACAAGAAGGATGAACCACTCCCTGCTAGACCCCCACATGACCTGTCTAATTTTTCATCTATCATTGAGTTATAAAAGAATAAATTGTGCCTTCTCCAGCCATTTTGGTTTTGAACTCATAATTTTGTCTTTCATAAAAAGTCTTTCCTGCTCAATTATTTGAAGACAC 2600
     ----:----|----:----|----:----|----:----|----:----|----:----|----:----|----:----|----:----|----:----|----:----|----:----|----:----|----:----|----:----|----:----|----:----|----:----|----:----|----:----|
2401 TCGACAGAGAGATCATCGTATCGTGTTCCATGTGTTCTTCCTACTTGGTGAGGGACGATCTGGGGGTGTACTGGACAGATTAAAAAGTAGATAGTAACTCAATATTTTCTTATTTAACACGGAAGAGGTCGGTAAAACCAAAACTTGAGTATTAAAACAGAAAGTATTTTTCAGAAAGGACGAGTTAATAAACTTCTGTG 2600
      L  Q  R  E  L  L  M  A  C  P  V  C  S  P  H  V  V  G  Q  *  V  G  V  H  G  T  *  N  K  M  *  *  Q  T  I  F  S  Y  I  T  G  E  G  A  M  K  T  K  F  E  Y  N  Q  R  E  Y  F  T  K  G  A  *  N  N  S  S  V   F6
     S  S  D  R  *  Y  C  L  V  L  Y  V  L  L  I  F  W  E  R  S  S  G  W  M  V  Q  R  I  K  *  R  D  N  L  *  L  L  I  F  Q  A  K  E  L  W  K  P  K  S  S  M  I  K  D  K  M  F  L  R  E  Q  E  I  I  Q  L  C    F5
       A  T  E  R  T  A  Y  C  L  T  C  L  F  S  S  G  S  G  A  L  G  G  C  S  R  D  L  K  E  D  I  M  S  N  Y  F  F  L  N  H  R  R  W  G  N  Q  N  Q  V  *  L  K  T  K  *  L  F  D  K  R  S  L  *  K  F  V     F4

      T  D  *  M  L  C  Y  F  H  R  K  L  P  L  I  M  A  Y  S  F  *  P  N  L  A  F  L  P  K  I  S  V  Q  P  P  F  L  K  L  P  R  F  *  K  H  K  M  K  T  *  R  L  Y  Q  G  G  T  Y  H  L  F  P  C  A  L  L  H   F1
       Q  T  R  C  F  V  I  F  I  A  N  C  L  *  *  W  P  I  R  S  D  R  I  W  L  F  F  Q  K  Y  Q  F  N  P  L  F  *  N  C  L  D  S  E  N  T  K  *  K  P  E  G  F  I  R  E  E  P  I  T  C  F  L  V  H  C  Y   F2
     N  R  L  D  A  L  L  F  S  S  Q  I  A  S  N  N  G  L  F  V  L  T  E  F  G  F  S  S  K  N  I  S  S  T  P  F  S  K  T  A  S  I  L  K  T  Q  N  E  N  L  K  A  L  S  G  R  N  L  S  P  V  S  L  C  T  A  T    F3
2601 AACAGACTAGATGCTTTGTTATTTTCATCGCAAATTGCCTCTAATAATGGCCTATTCGTTCTGACCGAATTTGGCTTTTCTTCCAAAAATATCAGTTCAACCCCCTTTTCTAAAACTGCCTCGATTCTGAAAACACAAAATGAAAACCTGAAGGCTTTATCAGGGAGGAACCTATCACCTGTTTCCTTGTGCACTGCTAC 2800
     ----:----|----:----|----:----|----:----|----:----|----:----|----:----|----:----|----:----|----:----|----:----|----:----|----:----|----:----|----:----|----:----|----:----|----:----|----:----|----:----|
2601 TTGTCTGATCTACGAAACAATAAAAGTAGCGTTTAACGGAGATTATTACCGGATAAGCAAGACTGGCTTAAACCGAAAAGAAGGTTTTTATAGTCAAGTTGGGGGAAAAGATTTTGACGGAGCTAAGACTTTTGTGTTTTACTTTTGGACTTCCGAAATAGTCCCTCCTTGGATAGTGGACAAAGGAACACGTGACGATG 2800
       V  S  *  I  S  Q  *  K  *  R  L  N  G  R  I  I  A  *  E  N  Q  G  F  K  A  K  R  G  F  I  D  T  *  G  G  K  R  F  S  G  R  N  Q  F  C  L  I  F  V  Q  L  S  *  *  P  P  V  *  *  R  N  G  Q  A  S  S   F6
      L  L  S  S  A  K  N  N  E  D  C  I  A  E  L  L  P  R  N  T  R  V  S  N  P  K  E  E  L  F  I  L  E  V  G  K  E  L  V  A  E  I  R  F  V  C  F  S  F  R  F  A  K  D  P  L  F  R  D  G  T  E  K  H  V  A  V  F5
     C  C  V  L  H  K  T  I  K  M  A  F  Q  R  *  Y  H  G  I  R  E  S  R  I  Q  S  K  K  W  F  Y  *  N  L  G  R  K  *  F  Q  R  S  E  S  F  V  F  H  F  G  S  P  K  I  L  S  S  G  I  V  Q  K  R  T  C  Q  *    F4

      H  P  I  H  Q  P  H  P  L  Y  M  F  *  E  *  V  K  G  Q  E  G  Q  Y  L  L  L  P  N  L  *  *  Y  Q  V  L  I  P  A  V  F  *  P  H  Y  L  D  N  S  I  *  L  L  *  H  W  G  L  V  F  L  V  Q  I  C  E  N   F1
     I  T  Q  S  I  S  P  I  L  Y  I  C  F  K  N  K  *  R  V  K  R  V  N  T  F  C  F  Q  T  S  D  N  I  R  F  *  F  L  Q  C  F  S  H  T  I  W  I  I  P  S  D  S  F  N  I  G  G  W  Y  F  W  Y  R  F  V  K  I   F2
      S  P  N  P  S  A  P  S  F  I  Y  V  L  R  I  S  E  G  S  R  G  S  I  P  S  A  S  K  P  L  I  I  S  G  S  N  S  C  S  V  L  A  T  L  S  G  *  F  H  L  T  P  L  T  L  G  V  G  I  S  G  T  D  L  *  K  S  F3
2801 ATCACCCAATCCATCAGCCCCATCCTTTATATATGTTTTAAGAATAAGTGAAGGGTCAAGAGGGTCAATACCTTCTGCTTCCAAACCTCTGATAATATCAGGTTCTAATTCCTGCAGTGTTTTAGCCACACTATCTGGATAATTCCATCTGACTCCTTTAACATTGGGGGTTGGTATTTCTGGTACAGATTTGTGAAAAT 3000
     ----:----|----:----|----:----|----:----|----:----|----:----|----:----|----:----|----:----|----:----|----:----|----:----|----:----|----:----|----:----|----:----|----:----|----:----|----:----|----:----|
2801 TAGTGGGTTAGGTAGTCGGGGTAGGAAATATATACAAAATTCTTATTCACTTCCCAGTTCTCCCAGTTATGGAAGACGAAGGTTTGGAGACTATTATAGTCCAAGATTAAGGACGTCACAAAATCGGTGTGATAGACCTATTAAGGTAGACTGAGGAAATTGTAACCCCCAACCATAAAGACCATGTCTAAACACTTTTA 3000
     C  *  G  I  W  *  G  W  G  K  Y  I  N  *  S  Y  T  F  P  *  S  P  *  Y  R  R  S  G  F  R  Q  Y  Y  *  T  R  I  G  A  T  N  *  G  C  *  R  S  L  E  M  Q  S  R  *  C  Q  P  N  T  N  R  T  C  I  Q  S  F   F6
      D  G  L  G  D  A  G  D  K  I  Y  T  K  L  I  L  S  P  D  L  P  D  I  G  E  A  E  L  G  R  I  I  D  P  E  L  E  Q  L  T  K  A  V  S  D  P  Y  N  W  R  V  G  K  V  N  P  T  P  I  E  P  V  S  K  H  F   F5
      M  V  W  D  M  L  G  M  R  *  I  H  K  L  F  L  H  L  T  L  L  T  L  V  K  Q  K  W  V  E  S  L  I  L  N  *  N  R  C  H  K  L  W  V  I  Q  I  I  G  D  S  E  K  L  M  P  P  Q  Y  K  Q  Y  L  N  T  F  I   F4

     H  L  K  H  H  M  V  Q  K  K  N  S  L  S  V  T  L  L  K  Q  *  V  W  L  G  C  G  K  K  Y  *  N  A  L  D  P  P  M  N  I  *  L  M  L  A  Y  T  D  I  Q  *  N  L  M  F  W  E  A  E  T  D  *  S  D  S  F  V    F1
      T  *  N  I  I  W  F  K  R  K  I  H  C  L  *  H  Y  *  S  S  R  F  G  W  G  V  V  K  S  I  E  M  L  L  T  L  Q  *  I  S  S  *  C  W  H  I  L  T  F  N  R  I  *  C  F  G  R  L  K  R  I  E  V  T  L  L  F   F2
      L  K  T  S  Y  G  S  K  E  K  F  T  V  C  D  I  I  E  A  V  G  L  A  G  V  W  *  K  V  L  K  C  S  *  P  S  N  E  Y  L  V  D  A  G  I  Y  *  H  S  I  E  S  N  V  L  G  G  *  N  G  L  K  *  L  F  C    F3
3001 CACTTAAAACATCATATGGTTCAAAAGAAAAATTCACTGTCTGTGACATTATTGAAGCAGTAGGTTTGGCTGGGGTGTGGTAAAAAGTATTGAAATGCTCTTGACCCTCCAATGAATATCTAGTTGATGCTGGCATATACTGACATTCAATAGAATCTAATGTTTTGGGAGGCTGAAACGGATTGAAGTGACTCTTTTGT 3200
     ----:----|----:----|----:----|----:----|----:----|----:----|----:----|----:----|----:----|----:----|----:----|----:----|----:----|----:----|----:----|----:----|----:----|----:----|----:----|----:----|
3001 GTGAATTTTGTAGTATACCAAGTTTTCTTTTTAAGTGACAGACACTGTAATAACTTCGTCATCCAAACCGACCCCACACCATTTTTCATAACTTTACGAGAACTGGGAGGTTACTTATAGATCAACTACGACCGTATATGACTGTAAGTTATCTTAGATTACAAAACCCTCCGACTTTGCCTAACTTCACTGAGAAAACA 3200
      *  K  F  C  *  I  T  *  F  F  F  E  S  D  T  V  N  N  F  C  Y  T  Q  S  P  H  P  L  F  Y  Q  F  A  R  S  G  G  I  F  I  *  N  I  S  A  Y  V  S  M  *  Y  F  R  I  N  Q  S  A  S  V  S  Q  L  S  E  K  T   F6
     D  S  L  V  D  Y  P  E  F  S  F  N  V  T  Q  S  M  I  S  A  T  P  K  A  P  T  H  Y  F  T  N  F  H  E  Q  G  E  L  S  Y  R  T  S  A  P  M  Y  Q  C  E  I  S  D  L  T  K  P  P  Q  F  P  N  F  H  S  K  Q   F5
       V  *  F  M  M  H  N  L  L  F  I  *  Q  R  H  C  *  Q  L  L  L  N  P  Q  P  T  T  F  L  I  S  I  S  K  V  R  W  H  I  D  L  Q  H  Q  C  I  S  V  N  L  L  I  *  H  K  P  L  S  F  R  I  S  T  V  R  K   F4

      L  K  T  H  T  E  S  D  I  G  S  C  S  A  V  Y  Q  C  A  M  P  N  I  H  V  Q  S  M  M  L  T  F  H  L  S  K  *  S  Q  *  V  L  T  S  N  H  W  C  Q  I  D  D  Q  I  T  *  K  K  E  H  L  Q  Y  F  H  V  F   F1
       *  K  L  I  L  R  A  I  L  G  L  A  L  Q  C  I  N  A  Q  C  Q  T  F  M  C  S  Q  *  C  *  P  F  T  S  P  N  R  V  N  K  F  *  P  R  I  I  G  V  R  *  M  I  R  *  H  E  K  K  N  I  S  N  I  F  M  F   F2
     F  K  N  S  Y  *  E  R  Y  W  V  L  L  C  S  V  S  M  R  N  A  K  H  S  C  A  V  N  D  V  D  L  S  P  L  Q  I  E  S  I  S  F  D  L  E  S  L  V  S  D  R  *  S  D  N  M  K  K  R  T  S  P  I  F  S  C  F   F3
3201 TTTAAAAACTCATACTGAGAGCGATATTGGGTCTTGCTCTGCAGTGTATCAATGCGCAATGCCAAACATTCATGTGCAGTCAATGATGTTGACCTTTCACCTCTCCAAATAGAGTCAATAAGTTTTGACCTCGAATCATTGGTGTCAGATAGATGATCAGATAACATGAAAAAAAGAACATCTCCAATATTTTCATGTTT 3400
     ----:----|----:----|----:----|----:----|----:----|----:----|----:----|----:----|----:----|----:----|----:----|----:----|----:----|----:----|----:----|----:----|----:----|----:----|----:----|----:----|
3201 AAATTTTTGAGTATGACTCTCGCTATAACCCAGAACGAGACGTCACATAGTTACGCGTTACGGTTTGTAAGTACACGTCAGTTACTACAACTGGAAAGTGGAGAGGTTTATCTCAGTTATTCAAAACTGGAGCTTAGTAACCACAGTCTATCTACTAGTCTATTGTACTTTTTTTCTTGTAGAGGTTATAAAAGTACAAA 3400
      K  F  V  *  V  S  L  S  I  P  D  Q  E  A  T  Y  *  H  A  I  G  F  M  *  T  C  D  I  I  N  V  K  *  R  E  L  Y  L  *  Y  T  K  V  E  F  *  Q  H  *  I  S  S  *  I  V  H  F  F  S  C  R  W  Y  K  *  T   F6
      K  L  F  E  Y  Q  S  R  Y  Q  T  K  S  Q  L  T  D  I  R  L  A  L  C  E  H  A  T  L  S  T  S  R  E  G  R  W  I  S  D  I  L  K  S  R  S  D  N  T  D  S  L  H  D  S  L  M  F  F  L  V  D  G  I  N  E  H  K   F5
     N  *  F  S  M  S  L  A  I  N  P  R  A  R  C  H  I  L  A  C  H  W  V  N  M  H  L  *  H  H  Q  G  K  V  E  G  F  L  T  L  L  N  Q  G  R  I  M  P  T  L  Y  I  I  L  Y  C  S  F  F  F  M  E  L  I  K  M  N   F4

      H  C  R  I  H  G  Q  T  V  L  L  A  L  N  F  S  V  S  N  I  S  V  Y  S  H  K  *  E  F  L  L  G  L  L  L  V  T  D  *  I  L  T  A  E  L  F  *  G  I  S  Q  T  C  T  C  L  H  V  S  V  P  H  V  P  T  A   F1
     F  T  A  E  F  T  D  R  L  Y  Y  W  L  *  T  F  L  S  Q  I  F  R  S  T  V  I  N  R  N  F  C  *  A  F  S  S  *  P  T  R  F  *  L  Q  N  C  F  R  V  F  P  K  L  A  R  V  F  M  F  Q  Y  L  M  Y  R  Q  H   F2
      S  L  Q  N  S  R  T  D  C  T  T  G  F  K  L  F  C  L  K  Y  F  G  L  Q  S  *  I  G  I  F  A  R  P  S  P  R  D  R  L  D  F  D  C  R  I  V  L  G  Y  F  P  N  L  H  V  S  S  C  F  S  T  S  C  T  D  S  T   F3
3401 TTCACTGCAGAATTCACGGACAGACTGTACTACTGGCTTTAAACTTTTCTGTCTCAAATATTTCGGTCTACAGTCATAAATAGGAATTTTTGCTAGGCCTTCTCCTCGTGACCGACTAGATTTTGACTGCAGAATTGTTTTAGGGTATTTCCCAAACTTGCACGTGTCTTCATGTTTCAGTACCTCATGTACCGACAGCA 3600
     ----:----|----:----|----:----|----:----|----:----|----:----|----:----|----:----|----:----|----:----|----:----|----:----|----:----|----:----|----:----|----:----|----:----|----:----|----:----|----:----|
3401 AAGTGACGTCTTAAGTGCCTGTCTGACATGATGACCGAAATTTGAAAAGACAGAGTTTATAAAGCCAGATGTCAGTATTTATCCTTAAAAACGATCCGGAAGAGGAGCACTGGCTGATCTAAAACTGACGTCTTAACAAAATCCCATAAAGGGTTTGAACGTGCACAGAAGTACAAAGTCATGGAGTACATGGCTGTCGT 3600
     K  *  Q  L  I  *  P  C  V  T  S  S  A  K  F  K  E  T  E  F  I  E  T  *  L  *  L  Y  S  N  K  S  P  R  R  R  T  V  S  *  I  K  V  A  S  N  N  *  P  I  E  W  V  Q  V  H  R  *  T  E  T  G  *  T  G  V  A   F6
      E  S  C  F  E  R  V  S  Q  V  V  P  K  L  S  K  Q  R  L  Y  K  P  R  C  D  Y  I  P  I  K  A  L  G  E  G  R  S  R  S  S  K  S  Q  L  I  T  K  P  Y  K  G  F  K  C  T  D  E  H  K  L  V  E  H  V  S  L   F5
      K  V  A  S  N  V  S  L  S  Y  *  Q  S  *  V  K  R  D  *  I  N  R  D  V  T  M  F  L  F  K  Q  *  A  K  E  E  H  G  V  L  N  Q  S  C  F  Q  K  L  T  N  G  L  S  A  R  T  K  M  N  *  Y  R  M  Y  R  C  C   F4

     L  L  C  I  L  Y  K  H  T  K  H  E  D  S  H  T  L  E  V  N  D  H  L  N  S  R  Y  H  P  Y  L  *  L  S  D  T  Q  D  K  H  F  L  S  E  A  M  R  *  Y  K  L  Y  N  K  C  G  C  K  V  L  L  L  A  R  L  V  D   F1
      F  C  A  F  C  I  N  T  R  N  M  K  T  L  I  H  *  K  *  M  T  T  *  T  R  D  I  I  H  I  C  D  C  P  T  H  R  T  N  T  S  Y  L  K  P  C  G  N  T  S  F  T  I  N  V  V  A  K  C  Y  Y  W  L  G  W  *  T   F2
       S  V  H  S  V  *  T  H  E  T  *  R  L  S  Y  I  R  S  K  *  P  L  K  L  E  I  S  S  I  S  V  T  V  R  H  T  G  Q  T  L  P  I  *  S  H  A  V  I  Q  A  L  Q  *  M  W  L  Q  S  V  I  I  G  S  V  G  R    F3
3601 CTTCTGTGCATTCTGTATAAACACACGAAACATGAAGACTCTCATACATTAGAAGTAAATGACCACTTAAACTCGAGATATCATCCATATCTGTGACTGTCCGACACACAGGACAAACACTTCCTATCTGAAGCCATGCGGTAATACAAGCTTTACAATAAATGTGGTTGCAAAGTGTTATTATTGGCTCGGTTGGTAGA 3800
     ----:----|----:----|----:----|----:----|----:----|----:----|----:----|----:----|----:----|----:----|----:----|----:----|----:----|----:----|----:----|----:----|----:----|----:----|----:----|----:----|
3601 GAAGACACGTAAGACATATTTGTGTGCTTTGTACTTCTGAGAGTATGTAATCTTCATTTACTGGTGAATTTGAGCTCTATAGTAGGTATAGACACTGACAGGCTGTGTGTCCTGTTTGTGAAGGATAGACTTCGGTACGCCATTATGTTCGAAATGTTATTTACACCAACGTTTCACAATAATAACCGAGCCAACCATCT 3800
      S  R  H  M  R  Y  L  C  V  F  C  S  S  E  *  V  N  S  T  F  S  W  K  F  E  L  Y  *  G  Y  R  H  S  D  S  V  C  S  L  C  K  R  D  S  A  M  R  Y  Y  L  S  *  L  L  H  P  Q  L  T  N  N  N  A  R  N  T  S   F6
     V  E  T  C  E  T  Y  V  C  S  V  H  L  S  E  Y  M  L  L  L  H  G  S  L  S  S  I  D  D  M  D  T  V  T  R  C  V  P  C  V  S  G  I  Q  L  W  A  T  I  C  A  K  C  Y  I  H  N  C  L  T  I  I  P  E  T  P  L   F5
       K  Q  A  N  Q  I  F  V  R  F  M  F  V  R  M  C  *  F  Y  I  V  V  *  V  R  S  I  M  W  I  Q  S  Q  G  V  C  L  V  F  V  E  *  R  F  G  H  P  L  V  L  K  V  I  F  T  T  A  F  H  *  *  Q  S  P  Q  Y   F4

      L  Y  I  C  C  I  A  *  F  Q  Q  D  D  A  L  Q  P  C  L  N  *  H  *  *  L  P  Y  L  R  M  S  G  Q  F  H  *  N  P  T  H  G  Q  H  Q  Q  Q  F  D  F  F  L  E  F  *  Q  M  V  Q  T  S  P  N  *  C  Q  G  Y   F1
       F  T  Y  A  A  L  H  N  F  S  K  M  M  L  Y  S  P  V  *  I  N  T  S  N  F  L  I  *  E  *  V  G  S  F  I  K  I  Q  H  T  G  N  I  S  N  S  L  T  F  S  W  N  F  S  R  W  F  K  R  V  P  T  S  A  R  D   F2
     P  L  H  M  L  H  C  I  I  S  A  R  *  C  S  T  A  L  S  K  L  T  L  V  T  S  L  S  E  N  E  W  A  V  S  L  K  S  N  T  R  A  T  S  A  T  V  *  L  F  L  G  I  L  A  D  G  S  N  E  S  Q  L  V  P  G  I   F3
3801 CCTTTACATATGCTGCATTGCATAATTTCAGCAAGATGATGCTCTACAGCCCTGTCTAAATTAACACTAGTAACTTCCTTATCTGAGAATGAGTGGGCAGTTTCATTAAAATCCAACACACGGGCAACATCAGCAACAGTTTGACTTTTTCTTGGAATTTTAGCAGATGGTTCAAACGAGTCCCAACTAGTGCCAGGGAT 4000
     ----:----|----:----|----:----|----:----|----:----|----:----|----:----|----:----|----:----|----:----|----:----|----:----|----:----|----:----|----:----|----:----|----:----|----:----|----:----|----:----|
3801 GGAAATGTATACGACGTAACGTATTAAAGTCGTTCTACTACGAGATGTCGGGACAGATTTAATTGTGATCATTGAAGGAATAGACTCTTACTCACCCGTCAAAGTAATTTTAGGTTGTGTGCCCGTTGTAGTCGTTGTCAAACTGAAAAAGAACCTTAAAATCGTCTACCAAGTTTGCTCAGGGTTGATCACGGTCCCTA 4000
      R  *  M  H  Q  M  A  Y  N  *  C  S  S  A  R  C  G  Q  R  F  *  C  *  Y  S  G  *  R  L  I  L  P  C  N  *  *  F  G  V  C  P  C  C  *  C  C  N  S  K  K  K  S  N  *  C  I  T  *  V  L  G  L  *  H  W  P   F6
      G  K  C  I  S  C  Q  M  I  E  A  L  H  H  E  V  A  R  D  L  N  V  S  T  V  E  K  D  S  F  S  H  A  T  E  N  F  D  L  V  R  A  V  D  A  V  T  Q  S  K  R  P  I  K  A  S  P  E  F  S  D  W  S  T  G  P  I   F5
     V  K  V  Y  A  A  N  C  L  K  L  L  I  I  S  *  L  G  T  *  I  L  V  L  L  K  R  I  Q  S  H  T  P  L  K  M  L  I  W  C  V  P  L  M  L  L  L  K  V  K  E  Q  F  K  L  L  H  N  L  R  T  G  V  L  A  L  S   F4

      Y  I  Q  R  Y  C  Y  L  K  C  H  I  P  Y  P  K  L  Y  Q  Y  H  Y  H  S  L  L  Q  R  L  L  Y  F  Q  L  V  H  Y  L  M  L  Y  Q  Y  Y  P  L  I  L  Q  *  K  N  Q  R  Q  L  A  L  I  V  Y  R  Y  D  L  F   F1
     T  I  F  S  D  I  A  T  *  N  A  T  F  H  I  R  S  C  I  S  I  T  I  I  H  F  C  K  G  Y  C  T  F  N  L  F  I  I  *  C  C  I  S  T  I  L  *  F  C  N  R  K  I  K  D  N  W  H  *  L  F  I  G  T  T  F  S   F2
      L  Y  S  A  I  L  L  L  E  M  P  H  S  I  S  E  A  V  S  V  S  L  S  F  T  F  A  K  A  T  V  L  S  T  C  S  L  S  D  A  V  S  V  L  S  S  D  F  A  I  E  K  S  K  T  T  G  I  N  C  L  *  V  R  P  F  P   F3
4001 ACTATATTCAGCGATATTGCTACTTGAAATGCCACATTCCATATCCGAAGCTGTATCAGTATCACTATCATTCACTTTTGCAAAGGCTACTGTACTTTCAACTTGTTCATTATCTGATGCTGTATCAGTACTATCCTCTGATTTTGCAATAGAAAAATCAAAGACAACTGGCATTAATTGTTTATAGGTACGACCTTTTC 4200
     ----:----|----:----|----:----|----:----|----:----|----:----|----:----|----:----|----:----|----:----|----:----|----:----|----:----|----:----|----:----|----:----|----:----|----:----|----:----|----:----|
4001 TGATATAAGTCGCTATAACGATGAACTTTACGGTGTAAGGTATAGGCTTCGACATAGTCATAGTGATAGTAAGTGAAAACGTTTCCGATGACATGAAAGTTGAACAAGTAATAGACTACGACATAGTCATGATAGGAGACTAAAACGTTATCTTTTTAGTTTCTGTTGACCGTAATTAACAAATATCCATGCTGGAAAAG 4200
     Y  *  I  *  R  Y  Q  *  K  F  H  W  M  G  Y  G  F  S  Y  *  Y  *  *  *  E  S  K  C  L  S  S  Y  K  *  S  T  *  *  R  I  S  Y  *  Y  *  G  R  I  K  C  Y  F  F  *  L  C  S  A  N  I  T  *  L  Y  S  R  K   F6
      S  Y  E  A  I  N  S  S  S  I  G  C  E  M  D  S  A  T  D  T  D  S  D  N  V  K  A  F  A  V  T  S  E  V  Q  E  N  D  S  A  T  D  T  S  D  E  S  K  A  I  S  F  D  F  V  V  P  M  L  Q  K  Y  T  R  G  K   F5
      V  I  N  L  S  I  A  V  Q  F  A  V  N  W  I  R  L  Q  I  L  I  V  I  M  *  K  Q  L  P  *  Q  V  K  L  K  N  M  I  Q  H  Q  I  L  V  I  R  Q  N  Q  L  L  F  I  L  S  L  Q  C  *  N  N  I  P  V  V  K  E   F4

     L  Y  Y  K  C  Y  T  D  *  N  C  H  E  E  Q  S  H  K  A  L  G  Y  Y  K  K  T  *  V  S  F  A  L  K  F  Y  I  L  C  T  A  F  Y  R  H  C  T  V  C  L  D  V  R  P  L  N  H  *  K  F  Q  S  Q  K  V  P  K  P   F1
      S  I  T  N  A  T  Q  T  R  T  V  M  R  S  K  V  I  R  L  W  D  I  T  R  K  L  K  F  L  L  L  *  S  S  I  S  Y  A  L  H  F  T  G  I  A  Q  S  A  W  M  Y  V  L  *  I  T  R  N  F  N  L  K  K  F  Q  N  L   F2
      L  L  Q  M  L  H  R  L  E  L  S  *  G  A  K  S  *  G  F  G  I  L  Q  E  N  L  S  F  F  C  F  E  V  L  Y  L  M  H  C  I  L  Q  A  L  H  S  L  L  G  C  T  S  F  E  S  L  E  I  S  I  S  K  S  S  K  T   F3
4201 CTCTATTACAAATGCTACACAGACTAGAACTGTCATGAGGAGCAAAGTCATAAGGCTTTGGGATATTACAAGAAAACTTAAGTTTCTTTTGCTTTGAAGTTCTATATCTTATGCACTGCATTTTACAGGCATTGCACAGTCTGCTTGGATGTACGTCCTTTGAATCACTAGAAATTTCAATCTCAAAAAGTTCCAAAACC 4400
     ----:----|----:----|----:----|----:----|----:----|----:----|----:----|----:----|----:----|----:----|----:----|----:----|----:----|----:----|----:----|----:----|----:----|----:----|----:----|----:----|
4201 GAGATAATGTTTACGATGTGTCTGATCTTGACAGTACTCCTCGTTTCAGTATTCCGAAACCCTATAATGTTCTTTTGAATTCAAAGAAAACGAAACTTCAAGATATAGAATACGTGACGTAAAATGTCCGTAACGTGTCAGACGAACCTACATGCAGGAAACTTAGTGATCTTTAAAGTTAGAGTTTTTCAAGGTTTTGG 4400
     R  *  *  L  H  *  V  S  *  F  Q  *  S  S  C  L  *  L  A  K  P  Y  *  L  F  V  *  T  E  K  A  K  F  N  *  I  K  H  V  A  N  *  L  C  Q  V  T  Q  K  S  T  R  G  K  F  *  *  F  N  *  D  *  F  T  G  F  G   F6
     G  R  N  C  I  S  C  L  S  S  S  D  H  P  A  F  D  Y  P  K  P  I  N  C  S  F  K  L  K  K  Q  K  S  T  R  Y  R  I  C  Q  M  K  C  A  N  C  L  R  S  P  H  V  D  K  S  D  S  S  I  E  I  E  F  L  E  L  V   F5
      E  I  V  F  A  V  C  V  L  V  T  M  L  L  L  T  M  L  S  Q  S  I  V  L  F  S  L  N  R  K  S  Q  L  E  I  D  *  A  S  C  K  V  P  M  A  C  D  A  Q  I  Y  T  R  Q  I  V  L  F  K  L  R  L  F  N  W  F   F4

      Q  T  C  M  G  S  K  E  K  I  F  Y  T  S  V  H  I  P  C  I  N  H  V  Y  F  H  F  *  H  L  Q  H  *  T  T  V  L  G  L  Y  S  I  D  K  A  T  Q  D  C  R  Q  N  G  R  S  D  L  V  Q  L  S  S  L  G  L  S  K   F1
      R  H  V  W  V  Q  R  K  R  F  F  T  L  L  S  T  Y  P  A  *  T  M  C  T  S  I  F  D  I  Y  N  I  K  R  P  Y  *  A  C  I  Q  S  I  K  Q  L  K  T  V  D  K  M  D  D  Q  T  L  F  S  S  V  R  L  V  *  V   F2
     S  D  M  Y  G  F  K  G  K  D  F  L  H  F  C  P  H  T  L  H  K  P  C  V  L  P  F  L  T  F  T  T  L  N  D  R  I  R  L  V  F  N  R  *  S  N  S  R  L  *  T  K  W  T  I  R  P  C  S  A  Q  F  A  W  F  K  *   F3
4401 TCAGACATGTATGGGTTCAAAGGAAAAGATTTTTTACACTTCTGTCCACATACCCTGCATAAACCATGTGTACTTCCATTTTTGACATTTACAACATTAAACGACCGTATTAGGCTTGTATTCAATCGATAAAGCAACTCAAGACTGTAGACAAAATGGACGATCAGACCTTGTTCAGCTCAGTTCGCTTGGTTTAAGTA 4600
     ----:----|----:----|----:----|----:----|----:----|----:----|----:----|----:----|----:----|----:----|----:----|----:----|----:----|----:----|----:----|----:----|----:----|----:----|----:----|----:----|
4401 AGTCTGTACATACCCAAGTTTCCTTTTCTAAAAAATGTGAAGACAGGTGTATGGGACGTATTTGGTACACATGAAGGTAAAAACTGTAAATGTTGTAATTTGCTGGCATAATCCGAACATAAGTTAGCTATTTCGTTGAGTTCTGACATCTGTTTTACCTGCTAGTCTGGAACAAGTCGAGTCAAGCGAACCAAATTCAT 4600
      *  V  H  I  P  E  F  S  F  I  K  *  V  E  T  W  M  G  Q  M  F  W  T  Y  K  W  K  Q  C  K  C  C  *  V  V  T  N  P  K  Y  E  I  S  L  A  V  *  S  Q  L  C  F  P  R  D  S  R  T  *  S  L  E  S  P  K  L   F6
      E  S  M  Y  P  N  L  P  F  S  K  K  C  K  Q  G  C  V  R  C  L  G  H  T  S  G  N  K  V  N  V  V  N  F  S  R  I  L  S  T  N  L  R  Y  L  L  E  L  S  Y  V  F  H  V  I  L  G  Q  E  A  *  N  A  Q  N  L  Y   F5
     R  L  C  T  H  T  *  L  F  L  N  K  V  S  R  D  V  Y  G  A  Y  V  M  H  V  E  M  K  S  M  *  L  M  L  R  G  Y  *  A  Q  I  *  D  I  F  C  S  L  V  T  S  L  I  S  S  *  V  K  N  L  E  T  R  K  T  *  T   F4

      H  T  L  N  S  M  *  I  P  Q  L  C  Q  *  *  N  E  S  R  A  F  T  L  F  K  T  K  Y  L  F  F  I  Y  K  S  P  F  Y  L  L  I  F  K  I  I  Q  S  F  S  I  H  A  A  K  P  N  I  A  D  L  Q  T  R  I  F  Q   F1
     N  I  P  *  I  L  C  K  Y  P  S  C  V  N  N  K  T  K  V  E  H  S  L  Y  L  K  L  N  T  S  F  S  Y  I  N  H  L  F  I  Y  *  F  S  K  L  Y  S  H  F  P  S  M  Q  P  S  Q  T  S  P  I  F  K  R  E  Y  F  N   F2
      T  Y  P  K  F  Y  V  N  T  P  A  V  S  I  I  K  R  K  *  S  I  H  F  I  *  N  *  I  P  L  F  H  I  *  I  T  F  L  F  I  N  F  Q  N  Y  T  V  I  F  H  P  C  S  Q  A  K  H  R  R  S  S  N  A  N  I  S  T   F3
4601 AACATACCCTAAATTCTATGTAAATACCCCAGCTGTGTCAATAATAAAACGAAAGTAGAGCATTCACTTTATTTAAAACTAAATACCTCTTTTTCATATATAAATCACCTTTTTATTTATTAATTTTCAAAATTATACAGTCATTTTCCATCCATGCAGCCAAGCCAAACATCGCCGATCTTCAAACGCGAATATTTCAA 4800
     ----:----|----:----|----:----|----:----|----:----|----:----|----:----|----:----|----:----|----:----|----:----|----:----|----:----|----:----|----:----|----:----|----:----|----:----|----:----|----:----|
4601 TTGTATGGGATTTAAGATACATTTATGGGGTCGACACAGTTATTATTTTGCTTTCATCTCGTAAGTGAAATAAATTTTGATTTATGGAGAAAAAGTATATATTTAGTGGAAAAATAAATAATTAAAAGTTTTAATATGTCAGTAAAAGGTAGGTACGTCGGTTCGGTTTGTAGCGGCTAGAAGTTTGCGCTTATAAAGTT 4800
     L  C  V  R  F  E  I  Y  I  G  W  S  H  *  Y  Y  F  S  L  L  A  N  V  K  N  L  V  L  Y  R  K  K  M  Y  L  D  G  K  *  K  N  I  K  L  I  I  C  D  N  E  M  W  A  A  L  G  F  M  A  S  R  *  V  R  I  N  *   F6
      V  Y  G  L  N  *  T  F  V  G  A  T  D  I  I  F  R  F  Y  L  M  *  K  I  *  F  *  I  G  R  K  *  I  Y  I  V  K  K  N  I  L  K  *  F  *  V  T  M  K  W  G  H  L  W  A  L  C  R  R  D  E  F  A  F  I  E   F5
      F  M  G  *  I  R  H  L  Y  G  L  Q  T  L  L  L  V  F  T  S  C  E  S  *  K  F  S  F  V  E  K  E  Y  I  F  *  R  K  I  *  *  N  E  F  N  Y  L  *  K  G  D  M  C  G  L  W  V  D  G  I  K  L  R  S  Y  K  L   F4

     L  L  L  T  Q  C  P  P  F  F  L  L  E  L  V  *  R  Q  E  I  L  L  L  Q  L  R  M  L  R  G  Y  F  L  I  L  Y  L  A  R  Q  V  T  *  I  A  V  Q  S  E  S  G  S  S  G  I  S  V  P  T  F  D  F  T  S  L  F  T   F1
      F  Y  *  L  N  V  R  H  S  F  F  L  N  W  F  R  D  R  K  F  Y  F  Y  N  C  A  C  S  E  G  I  S  S  F  Y  I  W  P  D  R  *  H  K  *  P  S  N  Q  K  A  E  V  R  E  F  P  F  R  P  L  I  L  R  H  C  L  P   F2
      S  I  N  S  M  S  A  I  L  S  S  *  T  G  L  E  T  G  N  S  T  F  T  T  A  H  A  Q  R  V  F  P  H  F  I  F  G  Q  T  G  N  I  N  S  R  P  I  R  K  R  K  F  G  N  F  R  S  D  L  *  F  Y  V  I  V  Y   F3
4801 CTTCTATTAACTCAATGTCCGCCATTCTTTCTTCTTGAACTGGTTTAGAGACAGGAAATTCTACTTTTACAACTGCGCATGCTCAGAGGGTATTTCCTCATTTTATATTTGGCCAGACAGGTAACATAAATAGCCGTCCAATCAGAAAGCGGAAGTTCGGGAATTTCCGTTCCGACCTTTGATTTTACGTCATTGTTTAC 5000
     ----:----|----:----|----:----|----:----|----:----|----:----|----:----|----:----|----:----|----:----|----:----|----:----|----:----|----:----|----:----|----:----|----:----|----:----|----:----|----:----|
4801 GAAGATAATTGAGTTACAGGCGGTAAGAAAGAAGAACTTGACCAAATCTCTGTCCTTTAAGATGAAAATGTTGACGCGTACGAGTCTCCCATAAAGGAGTAAAATATAAACCGGTCTGTCCATTGTATTTATCGGCAGGTTAGTCTTTCGCCTTCAAGCCCTTAAAGGCAAGGCTGGAAACTAAAATGCAGTAACAAATG 5000
      S  R  N  V  *  H  G  G  N  K  R  R  S  S  T  *  L  C  S  I  R  S  K  C  S  R  M  S  L  P  Y  K  R  M  K  Y  K  A  L  C  T  V  Y  I  A  T  W  D  S  L  P  L  E  P  I  E  T  G  V  K  S  K  V  D  N  N  V   F6
     V  E  I  L  E  I  D  A  M  R  E  E  Q  V  P  K  S  V  P  F  E  V  K  V  V  A  C  A  *  L  T  N  G  *  K  I  N  P  W  V  P  L  M  F  L  R  G  I  L  F  R  F  N  P  F  K  R  E  S  R  Q  N  *  T  M  T  *   F5
      K  *  *  S  L  T  R  W  E  K  K  K  F  Q  N  L  S  L  F  N  *  K  *  L  Q  A  H  E  S  P  I  E  E  N  *  I  Q  G  S  L  Y  C  L  Y  G  D  L  *  F  A  S  T  R  S  N  G  N  R  G  K  I  K  R  *  Q  K   F4
```

```
      *  L  C  K  S  K  S  S  I  G  F  C  *  W  I  V  L         F1
       N  Y  A  K  A  K  V  A  L  G  F  V  S  G  L  C  X        F2
     L  I  M  Q  K  Q  K  *  H  W  V  L  L  V  D  C  A  X       F3
5001 CTAATTATGCAAAAGCAAAAGTAGCATTGGGTTTTGTTAGTGGATTGTGCTA 5052
     ----:----|----:----|----:----|----:----|----:----|--
5001 GATTAATACGTTTTCGTTTTCATCGTAACCCAAAACAATCACCTAACACGAT 5052
       *  N  H  L  L  L  L  L  M  P  N  Q  *  H  I  T  S        F6
      R  I  I  C  F  C  F  Y  C  Q  T  K  N  T  S  Q  A         F5
     G  L  *  A  F  A  F  T  A  N  P  K  T  L  P  N  H  *       F4
```

Pinctada imbricata (Pim)

WGS: NIJJ01023145.1

6390-12300
Pinctada imbricata breed pearl oyster-OUG PIN_chr1_1727, whole genome shotgun sequence

```
       K M R * A A H * Q T S M Q L L L L N K L C K L S L K K I E L S F L L T A S I H T S T R S A P N C G G N S H K N N V E L S F F V T S C F    F1
        K C A E Q H T N K R L C N F C F * I N Y A N * V * K K * S C L S C S P L Q F I P V L V V L Q I A E E I P I R I M W S Y R S L L L P V L   F2
      N A L S S T L T N V Y A T F A F E * I M Q I K F K K N R V V F L A H R F N S Y Q Y S * C S K L R R K F P * E * C G V I V L C Y F L F      F3
   1 AAAATGCGCTGAGCAGCACACTAACAAACGTCTATGCAACTTTTGCTTTTGAATAAATTATGCAAATTAAGTTTAAAAAAAAATAGAGTTGTCTTTCTTGCTCACCGCTTCAATTCATACCAGTACTCGTAGTGCTCCAAATTGCGGAGGAAATTCCCATAAGAATAATGTGGAGTTATCGTTCTTTGTTACTTCCTGTTT 200
     ----:----|----:----|----:----|----:----|----:----|----:----|----:----|----:----|----:----|----:----|----:----|----:----|----:----|----:----|----:----|----:----|----:----|----:----|----:----|----:----|
   1 TTTTACGCGACTCGTCGTGTGATTGTTTGCAGATACGTTGAAAACGAAAACTTATTTAATACGTTTAATTCAAATTTTTTTTTATCTCAACAGAAAGAACGAGTGGCGAAGTTAAGTATGGTCATGAGCATCACGAGGTTTAACGCCTCCTTTAAGGGTATTCTTATTACACCTCAATAGCAAGAAACAATGAAGGACAAA 200
      X I R Q A A C * C V D I C S K S K F L N H L N L K F F I S N D K K S V A E I * V L V R L A G F Q P P F E W L F L T S N D N K T V E Q K   F6
     X F A S L L V S V F T * A V K A K S Y I I C I L N L F F L T T K R A * R K L E Y W Y E Y H E L N R L F N G Y S Y H P T I T R Q * K R N    F5
       F H A S C C V L L R R H L K Q K Q I F * A F * T * F F Y L Q R E Q E G S * N M G T S T T S W I A S S I G M L I I H L * R E K N S G T     F4

       D R N I S E K V I E I D Q E M D F * Q F Y E V W * I F * K K Y T H L R Y E I K K I V * N Q V Q M L Q I L C N F F Q I L Q K F Y F H F V   F1
        I E T S V K K L L K S T K K W I F S N F M K F G E Y F R K N I H I * G M K * K K L Y K I K C K C Y R Y C A I F F R F S R N S T F I S     F2
     * S K H Q * K S Y * N R P R N G F L A I L * S L V N I L E K I Y T F E V * N K K N C I K S S A N V T D T V Q F F S D S P E I L L S F R    F3
 201 TGATCGAAACATCAGTGAAAAAGTTATTGAAATCGACCAAGAAATGGATTTTTAGCAATTTTATGAAGTTTGGTGAATATTTTAGAAAAAATATACACATTTGAGGTATGAAATAAAAAAAAATTGTATAAAATCAAGTGCAAATGTTACAGATACTGTGCAATTTTTTTCAGATTCTCCAGAAATTCTACTTTCATTTCG 400
     ----:----|----:----|----:----|----:----|----:----|----:----|----:----|----:----|----:----|----:----|----:----|----:----|----:----|----:----|----:----|----:----|----:----|----:----|----:----|----:----|
 201 ACTAGCTTTGTAGTCACTTTTTCAATAACTTTAGCTGGTTCTTTACCTAAAAATCGTTAAAATACTTCAAACCACTTATAAAATCTTTTTTATATGTGTAAACTCCATACTTTATTTTTTTTAACATATTTTAGTTCACGTTTACAATGTCTATGACACGTTAAAAAAAGTCTAAGAGGTCTTTAAGATGAAAGTAAAGC 400
      S R F M L S F T I S I S W S I S K * C N * S T Q H I N * F F Y V C K L Y S I F F I T Y F * T C I N C I S H L K K * I R W F N * K * K    F6
      Q D F C * H F L * Q F R G L F P N K A I K H L K T F I K S F I Y V N S T H F L F F Q I F D L A F T V S V T C N K E S E G S I R S E N R   F5
     K I S V D T F F N N F D V L F H I K L L K I F N P S Y K L F F I C M Q P I F Y F F N Y L I L H L H * L Y Q A I K K L N E L F E V K M E    F4

      * V S M C V I L S F F A S S L L K L G M S H Y S M Q N E S L M E S Q * Y H I * I L I V A A M L L I T L * K L R D A C M V K R G C H S     F1
     Y R S Q C A S F Y R F L R Q V S * N * A C P I I V C K T N H * W K V N N T I Y R F * L L Q Q C Y * L H Y R N * G M H A W * K G G V T A    F2
      I G L N V R H S I V F C V K S L E T R H V P L * Y A K R I I D G K S I I P Y I D F N C C S N V T N Y I I E I E G C M H G K K G V S Q P  F3
 401 TATAGGTCTCAATGTGCGTCATTCTATCGTTTTTTGCGTCAAGTCTCTTGAAACTAGGCATGTCCCATTATAGTATGCAAAACGAATCATTGATGGAAAGTCAATAATACCATATATAGATTTTAATTGTTGCAGCAATGTTACTAATTACATTATAGAAATTGAGGGATGCATGCATGGTAAAAAGGGGGTGTCACAGC 600
     ----:----|----:----|----:----|----:----|----:----|----:----|----:----|----:----|----:----|----:----|----:----|----:----|----:----|----:----|----:----|----:----|----:----|----:----|----:----|----:----|
 401 ATATCCAGAGTTACACGCAGTAAGATAGCAAAAAACGCAGTTCAGAGAACTTTGATCCGTACAGGGTAATATCATACGTTTTGCTTAGTAACTACCTTTCAGTTATTATGGTATATATCTAAAATTAACAACGTCGTTACAATGATTAATGTAATATCTTTAACTCCCTACGTACGTACCATTTTTCCCCCACAGTGTCG 600
     T Y T E I H T M R D N K A D L R K F S P M D W * L I C F S D N I S L * Y Y W I Y I K I T A A I N S I V N Y F N L S A H M T F L P H * L   F6
       I P R L T R * E I T K Q T L D R S V L C T G N Y Y A F R I M S P F D I I G Y I S K L Q Q L L T V L * M I S I S P H M C P L F P T D C   F5
      Y L D * H A D N * R K K R * T E Q F * A H G M I T H L V F * Q H F T L L V M Y L N * N N C C H * * N C * L F Q P I C A H Y F P P T V A  F4

     L S D Q I M L F F P E K T G D S A S * I V P V F V Q S K K S L C S T S R Q A T L Q F N S T L K T T S N * H I Y I K T Y Q R F I F S I D    F1
      F L T K * C Y F F L R K Q V I L Q A E L Y P Y L Y N P R N L C V A L A G R Q H C S L I Q L * R L H L I D T Y I S K P I K D S Y F P * I  F2
       F * P N N V I F S * E N R * F C K L N C T R I C T I Q E I F V * H * Q A G N I A V * F N F E D Y I * L T H I Y Q N L S K I H I F H R  F3
 601 CTTTCTGACCAAATAATGTTATTTTTTCCTGAGAAAACAGGTGATTCTGCAAGCTGAATTGTACCCGTATTTGTACAATCCAAGAAATCTTTGTGTAGCACTAGCAGGCAGGCAACATTGCAGTTTAATTCAACTTTGAAGACTACATCTAATTGACACATATATATCAAAACCTATCAAAGATTCATATTTTCCATAGA 800
     ----:----|----:----|----:----|----:----|----:----|----:----|----:----|----:----|----:----|----:----|----:----|----:----|----:----|----:----|----:----|----:----|----:----|----:----|----:----|----:----|
 601 GAAAGACTGGTTTATTACAATAAAAAAGGACTCTTTTGTCCACTAAGACGTTCGACTTAACATGGGCATAAACATGTTAGGTTCTTTAGAAACACATCGTGATCGTCCGTCCGTTGTAACGTCAAATTAAGTTGAAACTTCTGATGTAGATTAACTGTGTATATATAGTTTTGGATAGTTTCTAAGTATAAAAGGTATCT 800
       R E S W I I N N K G S F V P S E A L Q I T G T N T C D L F D K H L V L L C A V N C N L E V K F V V D L Q C M Y I L V * * L N M N E M S  F6
     G K Q G F L T I K E Q S F L H N Q L S F Q V R I Q V I W S I K T Y C * C A P L M A T * N L K S S * M * N V C I Y * F R D F I * I K W L   F5
       K R V L Y H * K K R L F C T I R C A S N Y G Y K Y L G L F R Q T A S A P L C C Q L K I * S Q L S C R I S V Y I D F G I L S E Y K G Y   F4

      * Y L * V T F Q E F L R P N H G G C * * N A K G C G * H C Q S H C K F D Q I L * G V W Y H P K E E E * S F L S S * T L * N R T F I I I  F1
       N T C K * L F R N F * D Q T M E A A D E M Q K D A D N I A N H I A N L T K F C R V C G T I Q K R K S R A F C P V E P Y K I E L L S L   F2
     L I L V S N F S G I F E T K P W R L L M K C K R M R I T L P I T L Q I * P N F V G C V V P S K R G R V E L F V Q L N L I K * N F Y H Y  F3
 801 TTAATACTTGTAAGTAACTTTTCAGGAATTTTTGAGACCAAACCATGGAGGCTGCTGATGAAATGCAAAAGGATGCGGATAACATTGCCAATCACATTGCAAATTTGACCAAATTTTGTAGGGTGTGTGGTACCATCCAAAAGAGGAAGAGTAGAGCTTTTTGTCCAGTTGAACCTTATAAAATAGAACTTTTATCATTA 1000
     ----:----|----:----|----:----|----:----|----:----|----:----|----:----|----:----|----:----|----:----|----:----|----:----|----:----|----:----|----:----|----:----|----:----|----:----|----:----|----:----|
 801 AATTATGAACATTCATTGAAAAGTCCTTAAAAACTCTGGTTTGGTACCTCCGACGACTACTTTACGTTTTCCTACGCCTATTGTAACGGTTAGTGTAACGTTTAAACTGGTTTAAAACATCCCACACACCATGGTAGGTTTTCTCCTTCTCATCTCGAAAAACAGGTCAACTTGGAATATTTTATCTTGAAAATAGTAAT 1000
      * Y K Y T V K * S N K L G F W P P Q Q H F A F P H P Y C Q W D C Q L N S W I K Y P T H Y W G F S S S Y L K K D L Q V K Y F L V K I M   F6
      N I S T L L K E P I K S V L G H L S S I F H L L I R I V N G I V N C I Q G F K T P H T T G D L L P L T S S K T W N F R I F Y F K * * *  F5
     I L V Q L Y S K L F K Q S W V M S A A S S I C F S A S L M A L * M A F K V L N Q L T H P V M W F L F L L A K Q G T S G * L I S S K D N   F4

       Q N * Y P I * * * K Y S S * E I V Q L M P V K M L S F S H K E R R R K Y I F Y E H S C * A F V H S S * C K L * S L S * E F * A K C Y   F1
      F K I D I Q Y D D E S I H P K K L C N S C R S K C F H F R T K K G E G N T F S M N I P V K H L Y I P H N A N C K V C H R N S E R N A T   F2
      S K L I S N M M M K V F I L R N C A T H A G Q N A F I F A Q R K A K E I H F L * T F L L S I C T F L I M Q I V K F V I G I L S E M L P  F3
1001 TTCAAAATTGATATCCAATATGATGATGAAAGTATTCATCCTAAGAAATTGTGCAACTCATGCCGGTCAAAATGCTTTCATTTTCGCACAAAGAAAGGCGAAGGAAATACATTTTCTATGAACATTCCTGTTAAGCATTTGTACATTCCTCATAATGCAAATTGTAAAGTTTGTCATAGGAATTCTGAGCGAAATGCTAC 1200
     ----:----|----:----|----:----|----:----|----:----|----:----|----:----|----:----|----:----|----:----|----:----|----:----|----:----|----:----|----:----|----:----|----:----|----:----|----:----|----:----|
1001 AAGTTTTAACTATAGGTTATACTACTACTTTCATAAGTAGGATTCTTTAACACGTTGAGTACGGCCAGTTTTACGAAAGTAAAAGCGTGTTTCTTTCCGCTTCCTTTATGTAAAAGATACTTGTAAGGACAATTCGTAAACATGTAAGGAGTATTACGTTTAACATTTCAAACAGTATCCTTAAGACTCGCTTTACGATG 1200
     I * F Q Y G I H H H F Y E D * S I T C S M G T L I S E N E C L S L R L F Y M K * S C E Q * A N T C E E Y H L N Y L K D Y S N Q A F H *   F6
      E F N I D L I I I F T N M R L F Q A V * A P * F A K M K A C L F A F S I C K R H V N R N L M Q V N R M I C I T F N T M P I R L S I S   F5
      N L I S I W Y S S S L I * G L F N H L E H R D F H K * K R V F F P S P F V N E I F M G T L C K Y M G * L A F Q L T Q * L F E S R F A V  F4

     H R V V S N S L * S I F R S S H R T C R W G R Y M * Q H * I R Q * G S K I K * S F S F I F S Y L P C S S I N K * P F F C S * N G Q I R   F1
      I E L F Q I H Y N P S S E V P I E H V D G G G T C S N T E S D S E D Q R S N D H S H S F S A T C H V A P S T S S H F F A A E M D K S E  F2
      * S C F K F I I I H L Q K F P * N M * M G E V H V A T L N Q T V R I K D Q M I I L I H F Q L L A M * L H Q Q V A I F L Q L K W T N Q   F3
1201 CATAGAGTTGTTTCAAATTCATTATAATCCATCTTCAGAAGTTCCCATAGAACATGTAGATGGGGGAGGTACATGTAGCAACACTGAATCAGACAGTGAGGATCAAAGATCAAATGATCATTCTCATTCATTTTCAGCTACTTGCCATGTAGCTCCATCAACAAGTAGCCATTTTTTTGCAGCTGAAATGGACAAATCAG 1400
     ----:----|----:----|----:----|----:----|----:----|----:----|----:----|----:----|----:----|----:----|----:----|----:----|----:----|----:----|----:----|----:----|----:----|----:----|----:----|----:----|
1201 GTATCTCAACAAAGTTTAAGTAATATTAGGTAGAAGTCTTCAAGGGTATCTTGTACATCTACCCCCTCCATGTACATCGTTGTGACTTAGTCTGTCACTCCTAGTTTCTAGTTTACTAGTAAGAGTAAGTAAAAGTCGATGAACGGTACATCGAGGTAGTTGTTCATCGGTAAAAAAACGTCGACTTTACCTGTTTAGTC 1400
      W L T T E F E N Y D M K L L E W L V H L H P L Y M Y C C Q I L C H P D F I L H D N E N M K L * K G H L E M L L Y G N K Q L Q F P C I L  F6
     G Y L Q K L N M I I W R * F N G Y F M Y I P S T C T A V S F * V T L I L S * I I M R M * K * S S A M Y S W * C T A M K K C S F H V F *   F5
       M S N N * I * * L G D E S T G M S C T S P P P V H L L V S D S L S S * L D F S * E * E N E A V Q W T A G D V L L W K K A A S I S L D   F4

      N L C I F S F R K F K * T M * T S F K E I M * T Q R N T * C Y * T I R F * Q H S T C A T L I H C N * F I K H S H R T S Y * T V I G Q D  F1
       I Y A S S L S E N S N E L C E P A S K K L C R P K G I L D V T K Q L D F D S T V H V P P S F T V T D L S S I P I E Q A I E Q S L A K   F2
     K F M H L L F Q K I Q M N Y V N Q L Q R N Y V D P K E Y L M L L N N * I L T A Q Y M C H P H S L * L I Y Q A F P * N K L L N S H W P R   F3
1401 AAATTTATGCATCTTCTCTTTCAGAAAATTCAAATGAACTATGTGAACCAGCTTCAAAGAAATTATGTAGACCCAAAGGAATACTTGATGTTACTAAACAATTAGATTTTGACAGCACAGTACATGTGCCACCCTCATTCACTGTAACTGATTTATCAAGCATTCCCATAGAACAAGCTATTGAACAGTCATTGGCCAAG 1600
     ----:----|----:----|----:----|----:----|----:----|----:----|----:----|----:----|----:----|----:----|----:----|----:----|----:----|----:----|----:----|----:----|----:----|----:----|----:----|----:----|
1401 TTTAAATACGTAGAAGAGAAAGTCTTTTAAGTTTACTTGATACACTTGGTCGAAGTTTCTTTAATACATCTGGGTTTCCTTATGAACTACAATGATTTGTTAATCTAAAACTGTCGTGTCATGTACACGGTGGGAGTAAGTGACATTGACTAAATAGTTCGTAAGGGTATCTTGTTCGATAACTTGTCAGTAACCGGTTC 1600
       F K H M K E K L F N L H V I H V L K L S I I Y V W L F V Q H * * V I L N Q C C L V H A V R M * Q L Q N I L C E W L V L * Q V T M P W   F6
      F N I C R R K * F I * I F * T F W S * L F * T S G F S Y K I N S F L * I K V A C Y M H W G * E S Y S I * * A N G Y F L S N F L * Q G L  F5
     S I * A D E R E S F E F S S H S G A E F F N H L G L P I S S T V L C N S K S L V T C T G G E N V T V S K D L M G M S C A I S C D N A L   F4

       I P V H N L S W Y T N C S C Y N S L * T Y F L Q I M S I F M A I E C R C L S N L * R T S S D K * D * H S Y R P I I L D V * Y F T C F   F1
      I F Q C T I C L G I P T A P A I T H C K H I F C R S C L S S W L L N A G V C P I C R E P V V I N E I S I L T G Q L S L M Y D I L H V S  F2
      Y S S A Q F V L V Y Q L L L L * L I V N I F F A D H V Y L H G Y * M Q V F V Q F V E N Q * * * M R L A F L Q A N Y P * C M I F Y M F H  F3
1601 ATATTCCAGTGCACAATTTGTCTTGGTATACCAACTGCTCCTGCTATAACTCATTGTAAACATATTTTTTGCAGATCATGTCTATCTTCATGGCTATTGAATGCAGGTGTTTGTCCAATTTGTAGAGAACCAGTAGTGATAAATGAGATTAGCATTCTTACAGGCCAATTATCCTTGATGTATGATATTTTACATGTTTC 1800
     ----:----|----:----|----:----|----:----|----:----|----:----|----:----|----:----|----:----|----:----|----:----|----:----|----:----|----:----|----:----|----:----|----:----|----:----|----:----|----:----|
1601 TATAAGGTCACGTGTTAAACAGAACCATATGGTTGACGAGGACGATATTGAGTAACATTTGTATAAAAAACGTCTAGTACAGATAGAAGTACCGATAACTTACGTCCACAAACAGGTTAAACATCTCTTGGTCATCACTATTTACTCTAATCGTAAGAATGTCCGGTTAATAGGAACTACATACTATAAAATGTACAAAG 1800
      S I G T C L K D Q Y V L Q E Q * L E N Y V Y K K C I M D I K M A I S H L H K D L K Y L V L L S L H S * C E * L G I I R S T H Y K V H K  F6
       Y E L A C N T K T Y W S S R S Y S M T F M N K A S * T * R * P * Q I C T N T W N T S F W Y H Y I L N A N K C A L * G Q H I I N * M N   F5
       I N W H V I Q R P I G V A G A I V * Q L C I K Q L D H R D E H S N F A P T Q G I Q L S G T T I F S I L M R V P W N D K I Y S I K C T E  F4

     M * V * * L * * S L R T * * N F R S * K C M * I W K V S L K A F D T F I N S K K R K G V A K N A Y L * L Q G K I C * A K K A * R S R K   F1
      C R F D N C N E V L G L D K I S D H E N V C K Y G K Y P S K L L T P S S I V R R G K G L Q K M P I Y D C K A K Y V K Q K R L K E V E S  F2
      V G L I I V M K S * D L I K F Q I M K M Y V N M E S I P Q S F * H L H Q * * E E E R G C K K C L F M I A R Q N M L S K K G L K K * K   F3
1801 ATGTAGGTTTGATAATTGTAATGAAGTCTTAGGACTTGATAAAATTTCAGATCATGAAAATGTATGTAAATATGGAAAGTATCCCTCAAAGCTTTTGACACCTTCATCAATAGTAAGAAGAGGAAAGGGGTTGCAAAAAATGCCTATTTATGATTGCAAGGCAAAATATGTTAAGCAAAAAAGGCTTAAAGAAGTAGAAA 2000
     ----:----|----:----|----:----|----:----|----:----|----:----|----:----|----:----|----:----|----:----|----:----|----:----|----:----|----:----|----:----|----:----|----:----|----:----|----:----|----:----|
1801 TACATCCAAACTATTAACATTACTTCAGAATCCTGAACTATTTTAAAGTCTAGTACTTTTACATACATTTATACCTTTCATAGGGAGTTTCGAAAACTGTGGAAGTAGTTATCATTCTTCTCCTTTCCCCAACGTTTTTTACGGATAAATACTAACGTTCCGTTTTATACAATTCGTTTTTTCCGAATTTCTTCATCTTT 2000
      M Y T Q Y N Y H L R L V Q Y F K L D H F H I Y I H F T D R L A K S V K M L L L F L F P T A F F A * K H N C P L I H * A F F A * L L L F   F6
     * T P K I I T I F D * S K I F N * I M F I Y T F I S L I G * L K Q C R * * Y Y S S S L P Q L F H R N I I A L C F I N L L F P K F F Y F   F5
       H L N S L Q L S T K P S S L I E S * S F T H L Y P F Y G E F S K V G E D I T L L P F P N C F I G I * S Q L A F Y T L C F L S L S T S   F4

      L S * T I L F F K K * G * N R C F I F H A Y S S F E E P K * * * I * T N * * C L D G R G S Q K I N K S * M P C N Q N R H F T E * N S M  F1
       S V E Q F C S S K N E D K T D V L F F M L I H H L K S Q N D N R F E Q I N D V W M G G G V R K L T S H E C L A I R I D T L Q S K T Q   F2
      A Q L N N F V L Q K M R I K Q M F Y F S C L F I I * R A K M I I D L N K L M M F G W E G E S E N * Q V M N A L Q S E * T L Y R V K L N  F3
2001 GCTCAGTTGAACAATTTTGTTCTTCAAAAAATGAGGATAAAACAGATGTTTTATTTTTCATGCTTATTCATCATTTGAAGAGCCAAAATGATAATAGATTTGAACAAATTAATGATGTTTGGATGGGAGGGGGAGTCAGAAAATTAACAAGTCATGAATGCCTTGCAATCAGAATAGACACTTTACAGAGTAAAACTCAA 2200
     ----:----|----:----|----:----|----:----|----:----|----:----|----:----|----:----|----:----|----:----|----:----|----:----|----:----|----:----|----:----|----:----|----:----|----:----|----:----|----:----|
2001 CGAGTCAACTTGTTAAAACAAGAAGTTTTTTACTCCTATTTTGTCTACAAAATAAAAAGTACGAATAAGTAGTAAACTTCTCGGTTTTACTATTATCTAAACTTGTTTAATTACTACAAACCTACCCTCCCCCTCAGTCTTTTAATTGTTCAGTACTTACGGAACGTTAGTCTTATCTGTGAAATGTCTCATTTTGAGTT 2200
      S L Q V I K N K L F H P Y F L H K I K * A * E D N S S G F H Y Y I Q V F * H H K S P L P L * F I L L D H I G Q L * F L C K V S Y F E   F6
      A * N F L K T R * F I L I F C I N * K E H K N M M Q L A L I I I S K F L N I I N P H S P S D S F * C T M F A K C D S Y V S * L T F S L  F5
     L E T S C N Q E E F F S S L V S T K N K M S I * * K F L W F S L L N S C I L S T Q I P P P T L F N V L * S H R A I L I S V K C L L V *   F4

      * S T V * F L K G * G M * P I C S S I C I R * N * V S V C P W L S * I F N * R S G S S H * L L S H T S Q S K * R M Y F F T P R Q Y I   F1
     C K A Q Y D F L K A K G C N P F V A P Y A L D E I E C Q Y V P G S V R Y S I E G Q D Q V I N Y Y H T P V K V N R E C T S S P L D N T F   F2
      V K H S M I S * R L R D V T H L * L H M H * M K L S V S M S L A Q L D I Q L K V R I K S L T I I T H Q S K * I E N V L L H P * T I H L  F3
2201 TGTAAAGCACAGTATGATTTCTTAAAGGCTAAGGGATGTAACCCATTTGTAGCTCCATATGCATTAGATGAAATTGAGTGTCAGTATGTCCCTGGCTCAGTTAGATATTCAATTGAAGGTCAGGATCAAGTCATTAACTATTATCACACACCAGTCAAAGTAAATAGAGAATGTACTTCTTCACCCCTAGACAATACATT 2400
     ----:----|----:----|----:----|----:----|----:----|----:----|----:----|----:----|----:----|----:----|----:----|----:----|----:----|----:----|----:----|----:----|----:----|----:----|----:----|----:----|
2201 ACATTTCGTGTCATACTAAAGAATTTCCGATTCCCTACATTGGGTAAACATCGAGGTATACGTAATCTACTTTAACTCACAGTCATACAGGGACCGAGTCAATCTATAAGTTAACTTCCAGTCCTAGTTCAGTAATTGATAATAGTGTGTGGTCAGTTTCATTTATCTCTTACATGAAGAAGTGGGGATCTGTTATGTAA 2400
      I Y L V T H N R L P * P I Y G M Q L E M H M L H F Q T D T H G Q S L * I N L Q L D P D L * * S N D C V L * L L Y L I Y K K V G L C Y M  F6
       T F C L I I E * L S L S T V W K Y S W I C * I F N L T L I D R A * N S I * N F T L I L D N V I I V C W D F Y I S F T S R * G * V I C   F5
       H L A C Y S K K F A L P H L G N T A G Y A N S S I S H * Y T G P E T L Y E I S P * S * T M L * * * V G T L T F L S H V E E G R S L V N  F4
```

```
     * T I R H C K * L P C L Y S * I S N S * C E G L S L E I S G C N S Q N I A * I R K * H N C R I T T T Q Y * S L * S Y N P V K D L H * G    F1
      E P L D I V N D F P A C T P E L A T P N V K G C R W R Y P D A I A K T L H E L E N D I I A G L Q Q H N I D P Y D P T I L L K T Y I K D   F2
      N H * T L * M T S L L V L L N * Q L L M * R A V V G D I R M Q * P K H C M N * K M T * L Q D Y N N T I L I L M I L Q S C * R P T L R  F3
2401 TGAACCATTAGACATTGTAAATGACTTCCCTGCTTGTACTCCTGAATTAGCAACTCCTAATGTGAAGGGCTGTCGTTGGAGATATCCGGATGCAATAGCCAAAACATTGCATGAATTAGAAAATGACATAATTGCAGGATTACAACAACACAATATTGATCCTTATGATCCTACAATCCTGTTAAAGACCTACATTAAGG 2600
     ----:----|----:----|----:----|----:----|----:----|----:----|----:----|----:----|----:----|----:----|----:----|----:----|----:----|----:----|----:----|----:----|----:----|----:----|----:----|----:----|
2401 ACTTGGTAATCTGTAACATTTACTGAAGGGACGAACATGAGGACTTAATCGTTGAGGATTACACTTCCCGACAGCAACCTCTATAGGCCTACGTTATCGGTTTTGTAACGTACTTAATCTTTTACTGTATTAACGTCCTAATGTTGTTGTGTTATAACTAGGAATACTAGGATGTTAGGACAATTTCTGGATGTAATTCC 2600
      Q V M L C Q L H S G Q K Y E Q I L L E * H S P S D N S I D P H L L W F M A H I L F H C L Q L I V V V C Y Q D K H D * L G T L S R C * P  F6
     K F W * V N Y I V E R S T S R F * C S R I H L A T T P S I R I C Y G F C Q M F * F I V Y N C S * L L V I N I R I I R C D Q * L G V N L   F5
      S G N S M T F S K G A Q V G S N A V G L T F P Q R Q L Y G S A I A L V N C S N S F S M I A P N C C C L I S G * S G V I R N F V * M L    F4

      W G * W S W * C V C T Q R S R G * I F A R Q G I Q I F L L C I * N R G H I R R * * K G H N L C * K K A K F C Q N K Q T C T * S N L * R   F1
       G A D G L G D V S V H K E V G D R Y L P D K A F R F S F C V Y R I E A I L G D D E K V T I F V E K R P N S V K T N R P V L E A I C D   F2
     M G L M V L V M C L Y T K K * G I D I C Q T R H S D F P F V Y I E * R P Y * E M M K R S Q S L L K K G Q I L S K Q T D L Y L K Q F V T   F3
2601 ATGGGGCTGATGGTCTTGGTGATGTGTCTGTACACAAAGAAGTAGGGGATAGATATTTGCCAGACAAGGCATTCAGATTTTCCTTTTGTGTATATAGAATAGAGGCCATATTAGGAGATGATGAAAAGGTCACAATCTTTGTTGAAAAAAGGCCAAATTCTGTCAAAACAAACAGACCTGTACTTGAAGCAATTTGTGAC 2800
     ----:----|----:----|----:----|----:----|----:----|----:----|----:----|----:----|----:----|----:----|----:----|----:----|----:----|----:----|----:----|----:----|----:----|----:----|----:----|----:----|
2601 TACCCCGACTACCAGAACCACTACACAGACATGTGTTTCTTCATCCCCTATCTATAAACGGTCTGTTCCGTAAGTCTAAAAGGAAAACACATATATCTTATCTCCGGTATAATCCTCTACTACTTTTCCAGTGTTAGAAACAACTTTTTTCCGGTTTAAGACAGTTTTGTTTGTCTGGACATGAACTTCGTTAAACACTG 2800
      H P Q H D Q H H T Q V C L L L P Y I N A L C P M * I K R K H I Y F L P W I L L H H F P * L R Q Q F F A L N Q * F L C V Q V Q L L K H  F6
     I P S I T K T I H R Y V F F Y P I S I Q W V L C E S K G K T Y I S Y L G Y * S I I F L D C D K N F F P W I R D F C V S R Y K F C N T V   F5
     S P A S P R P S T D T C L S T P S L Y K G S L A N L N E K Q T Y L I S A M N P S S S F T V I K T S F L G F E T L V F L G T S S A I Q S   F4

       K * S W L Q Y S L P I A N R K * K V L Y E R * D Y Q D S N R V W L E K A C T V F F * L N D * * K I G * D Q Q W I G W * W V Q L S M H   F1
     E N N H G S S T L C L L P I E N E R S Y M K G K I I R I Q T E F G W R R H V L F F S N S M I D E K L D R T N S G L A G S G S N Y L C T   F2
      K I I M A P V L F A Y C Q * K M K G L I * K V R L S G F K Q S L A G E G M Y C F F L T Q * L M K N W I G P T V D W L V V G P T I Y A H  F3
2801 GAAAATAATCATGGCTCCAGTACTCTTTGCCTATTGCCAATAGAAAATGAAAGGTCTTATATGAAAGGTAAGATTATCAGGATTCAAACAGAGTTTGGCTGGAGAAGGCATGTACTGTTTTTTCTAACTCAATGATTGATGAAAAATTGGATAGGACCAACAGTGGATTGGCTGGTAGTGGGTCCAACTATCTATGCAC 3000
     ----:----|----:----|----:----|----:----|----:----|----:----|----:----|----:----|----:----|----:----|----:----|----:----|----:----|----:----|----:----|----:----|----:----|----:----|----:----|----:----|
2801 CTTTTATTAGTACCGAGGTCATGAGAAACGGATAACGGTTATCTTTTACTTTCCAGAATATACTTTCCATTCTAATAGTCCTAAGTTTGTCTCAAACCGACCTCTTCCGTACATGACAAAAAAGATTGAGTTACTAACTACTTTTTAACCTATCCTGGTTGTCACCTAACCGACCATCACCCAGGTTGATAGATACGTG 3000
     R F Y D H S W Y E K G I A L L F H F T K Y S L Y S * * S E F L T Q S S F A H V T K K * S L S Q S H F I P Y S W C H I P Q Y H T W S D I C   F6
      F I I M A G T S K A * Q W Y F I F P R I H F T L N D P N L C L K A P S P M Y Q K K R V * H N I F F Q I P G V T S Q S T T P G V I * A   F5
      S F L * P E L V R Q R N G I S F S L D * I F F P L I I L I * V S N P Q L L C T S N K E L E I I S S F N S L V L L P N A P L P P D L * R H V  F4

     I V * C N K R N G P I Q F G * F C H K S F I C * N S R N C * V Y E D K S * * S Y Q G * F K Q D F * G H * I N P S P P G * I N R K G H R   F1
      L C D A T R E T A L S N L G N F V I N R S Y A E T A E I A E Y M R I N P D N L T K A D L N K I S K G I K S T P L L Q A E S I E K G I D  F2
      C V M Q Q E K R P Y P I W V I L S * I V H M L K Q Q K L L S I * G * I L I I L P R L I * T R F L R A L N Q P L S S R L N Q * K R A *  F3
3001 ATTGTGTGATGCAACAAGAGAAACGGCCCTATCCAATTTGGGTAATTTTGTCATAAATCGTTCATATGCTGAAACAGCAGAAATTGCTGAGTATATGAGGATAAATCCTGATAATCTTACCAAGGCTGATTTAAACAAGATTTCTAAGGGCATTAAATCAACCCCTCTCCTCCAGGCTGAATCAATAGAAAAGGGCATAG 3200
     ----:----|----:----|----:----|----:----|----:----|----:----|----:----|----:----|----:----|----:----|----:----|----:----|----:----|----:----|----:----|----:----|----:----|----:----|----:----|----:----|
3001 TAACACACTACGTTGTTCTCTTTGCCGGGATAGGTTAAACCCATTAAAACAGTATTTAGCAAGTATACGACTTTGTCGTCTTTAACGACTCATATACTCCTATTTAGGACTATTAGAATGGTTCCGACTAAATTTGTTCTAAAGATTCCCGTAATTTAGTTGGGGAGAGGAGGTCCGACTTAGTTATCTTTTCCCGTATC 3200
      M T H H L L L F P G I W N P Y N Q * L D N M H Q F L L F Q Q T Y S S L D Q Y D * W P Q N L C S K * P C * I L G E G G P Q I L L F P C L  F6
     C Q T I C C S F R G * G I Q T I K D Y I T * I S F C C F N S L I H P Y I R I I K G L S I * V L N R L A N F * G R E E L S F * Y F L A Y   F5
      N H S A V L S V A R D L K P L K T M F R E Y A S V A S I A S Y I L I F G S L R V L A S K F L I E L P M L D V G R R W A S D I S F P M  F4

      C Y S C R Y Q L W V F F S K A N H * G N S A N * * M G N E W G C E I Y Y * R W G G * V * F T S Q E N S R H K P T V D D A W * L C T S N  F1
       A T H A D I N C G S F F R K L I I R E I V Q I N K W E M S G D V K S I I K D G E V K F D L H L K K T V G I N P Q L M M P G N Y A R A  F2
     M L L M Q I S T V G L F F E S * S L G K * C K L I N G K * V G M * N L L L K M G R L S L I Y I S R K Q * A * T H S * * C L V T M H E Q   F3
3201 ATGCTACTCATGCAGATATCAACTGTGGGTCTTTTTTTCGAAAGCTAATCATTAGGGAAATAGTGCAAATTAATAAATGGGAAATGAGTGGGGATGTGAAATCTATTATTAAAGATGGGGAGGTTAAGTTTGATTTACATCTCAAGAAAACAGTAGGCATAAACCCACAGTTGATGATGCCTGGTAACTATGCACGAGCA 3400
     ----:----|----:----|----:----|----:----|----:----|----:----|----:----|----:----|----:----|----:----|----:----|----:----|----:----|----:----|----:----|----:----|----:----|----:----|----:----|----:----|
3201 TACGATGAGTACGTCTATAGTTGACACCCAGAAAAAAAGCTTTCGATTAGTAATCCCTTTATCACGTTTAATTATTTACCCTTTACTCACCCCTACACTTTAGATAATAATTTCTACCCCTCCAATTCAAACTAAATGTAGAGTTCTTTTGTCATCCGTATTTGGGTGTCAACTACTACGGACCATTGATACGTGCTCGT 3400
      H * E H L Y * S H T K K E F A L * * P F L A F * Y I P F S H P H S I * * * L H P P * T Q N V D * S F L L C L G V T S S A Q Y S H V L  F6
      I S S M C I D V T P R K K S L * D N P F Y H L N I F P F H T P I H F R N N F I P L N L K I * M E L F C Y A Y V W L Q H H R T V I C S C  F5
     S A V * A S I L Q P D K K R F S I M L S I T C I L L H S I L P S T F D I I L S P S T L N S K C R L F V T P M F G C N I I G P L * A R A   F4

      L * L * K * E F C S * F N R R * * E K V Q S * I I T S K I Q I H E N D L * S C * P Q S T V S I R D * N V Q R N C H R V W E N I D * *  F1
     I F D S K N E N S V L D L I E D D E R K C K V R L L L Q K F R F M R M I Y R A V D P K V Q Y P S E T K M Y K E T A I E F G K I L I N D   F2
      S L T L K M R I L F L I * S K M M R E S A K L D Y Y F K N S D S * E * F I E L L T P K Y S I H Q R L K C T K K L P * S L G K Y * L M T  F3
3401 ATCTTTGACTCTAAAAATGAGAATTCTGTTCTTGATTTAATCGAAGATGATGAGAGAAAGTGCAAAGTTAGATTATTACTTCAAAAATTCAGATTCATGAGAATGATTTATAGAGCTGTTGACCCCAAAGTACAGTATCCATCAGAGACTAAAATGTACAAAGAAACTGCCATAGAGTTTGGGAAAATATTGATTAATGA 3600
     ----:----|----:----|----:----|----:----|----:----|----:----|----:----|----:----|----:----|----:----|----:----|----:----|----:----|----:----|----:----|----:----|----:----|----:----|----:----|----:----|
3401 TAGAAACTGAGATTTTTACTCTTAAGACAAGAACTAAATTAGCTTCTACTACTCTCTTTCACGTTTCAATCTAATAATGAAGTTTTTAAGTCTAAGTACTCTTACTAAATATCTCGACAACTGGGGTTTCATGTCATAGGTAGTCTCTGATTTTACATGTTTCTTTGACGGTATCTCAAACCCTTTTATAACTAATTACT 3600
     L R Q S * F H S N Q E Q N L R L H H S F T C L * I I V E F I * I * S F S K Y L Q Q G W L V T D M L S * F T C L F Q W L T Q S F I S * H   F6
      D K V R F I L I R N K I * D F I I L S L A F N S * * K L F E S E H S H N I S S N V G F Y L I W * L S F H V F F S G Y L K P F Y Q N I  F5
      I K S E L F S F E T R S K I S S S S L F H L T L N N S * F N L N M L I I * L A T S G L T C Y G D S V L I Y L S V A M S N P F I N I L S  F4

     L * L C * M A Q L H T * S D * T C T G I K * K W T W N C W R A * * * R E * R R E Q D F S T F * E E F G K E G * Y I W G S K R C T L G S   F1
      F D Y A K W P N Y I H K V I E H V Q E L N E N G P G T V G G L S S E G N E G G N K I F R H F R K N L A R R G S T Y G G L R D V L W G H  F2
      L I M L N G P I T Y I K * L N M Y R N * M K M D L E L L E G L V V K G M K E G T R F F D I L G R I W Q G G V V H M G V * E M Y F G V  F3
3601 CTTTGATTATGCTAAATGGCCCAATTACATACATAAAGTGATTGAACATGTACAGGAATTAAATGAAAATGGACCTGGAACTGTTGGAGGGCTTAGTAGTGAAGGGAATGAAGGAGGGAACAAGATTTTTCGACATTTTAGGAAGAATTTGGCAAGGAGGGGTAGTACATATGGGGGTCTAAGAGATGTACTTTGGGGTC 3800
     ----:----|----:----|----:----|----:----|----:----|----:----|----:----|----:----|----:----|----:----|----:----|----:----|----:----|----:----|----:----|----:----|----:----|----:----|----:----|----:----|
3601 GAAACTAATACGATTTACCGGGTTAATGTATGTATTTCACTAACTTGTACATGTCCTTAATTTACTTTTACCTGGACCTTGACAACCTCCCGAATCATCACTTCCCTTACTTCCTCCCTTGTTCTAAAAAGCTGTAAAATCCTTCTTAAACCGTTCCTCCCCATCATGTATACCCCCAGATTCTCTACATGAAACCCCAG 3800
      S Q N H * I A W N C V Y L S Q V H V P I L H F H V Q F Q Q L A * Y H L S H L L S C S K E V N * S S N P L S P Y Y M H P D L L H V K P D  F6
     V K I S F P G I V Y M F H N F M Y L F * I F I S R S S N S P K T T F P I F S P V L N K S M K P L I Q C P P T T C I P T * S I Y K P T   F5
      K S * A L H G L * M C L T I S C T C S N F S F P G P V T P P S L L S P F S P P F L I K R C K L F F K A L L P L V Y P P R L S T S Q P  F4

      L V I Q * S I S N K T I S * I K I * T K M Q C M * I F G T * Y * K L S I F C R K C L N L L * H I C I Y F * K Y F V L Q C T L Q F Y * H  F1
       W L Y S S P S L T K L S A K S K S E Q R C S A C R S L G H N I R N C P S S V E N V * I C Y S I Y V S I S E N I L C Y S V H S N F I D  F2
     I G Y T V V H L * Q N Y Q L N Q N L N K D A V H V D L W D I I L E T V H L L * K M S K F V I A Y M Y L F L K I F C V T V Y T P I L L T   F3
3801 ATTGGTTATACAGTAGTCCATCTCTAACAAAACTATCAGCTAAATCAAAATCTGAACAAAGATGCAGTGCATGTAGATCTTTGGGACATAATATTAGAAACTGTCCATCTTCTGTAGAAAATGTCTAAATTTGTTATAGCATATATGTATCTATTTCTGAAAATATTTTGTGTTACAGTGTACACTCCAATTTTATTGAC 4000
     ----:----|----:----|----:----|----:----|----:----|----:----|----:----|----:----|----:----|----:----|----:----|----:----|----:----|----:----|----:----|----:----|----:----|----:----|----:----|----:----|
3801 TAACCAATATGTCATCAGGTAGAGATTGTTTTGATAGTCGATTTAGTTTTAGACTTGTTTCTACGTCACGTACATCTAGAAACCCTGTATTATAATCTTTGACAGGTAGAAGACATCTTTTACAGATTTAAACAATATCGTATATACATAGATAAAGACTTTTATAAAACACAATGTCACATGTGAGGTTAAAATAACTG 4000
      N T I C Y D M E L L V I L * I L I Q V F I C H M Y I K P V Y Y * F S D M K Q L F H R F K N Y C I H I * K Q F Y K T N C H V S W N * Q  F6
     M P * V T T W R * C F * * S F * F R F L S A T C T S R Q S M I N S V T W R R Y F I D L N T I A Y I Y R N R F I N Q T V T Y V G I K N V   F5
     * Q N Y L L G D R V F S D A L D F D S C L H L A H L D K P C L I L F Q G D E T S F T * I Q * L M Y T D I E S F I K H * L T C E L K I S   F4

      L S S R C G S * * L S G I F M Y I S A H C L Q F V Y F N N I K S Y F P S D V D G R D S L A S T A H P L L L L L * * Y S F V L F H L T  F1
     T F P V D V G V N N Y L V Y S C I F Q L I V C S L F I L I I L N H I F L Q M L M A G I A * P R Q H I L F Y F Y Y S N I H L F Y S T * Q   F2
      P F Q * M W E L I I I W Y I H V Y F S S L F A V C L F * * Y * I I F S F R C * W P G * P S L D S T S S F T S T I V I F I C F I P L N R  F3
4001 ACCTTTCCAGTAGATGTGGGAGTTAATAATTATCTGGTATATTCATGTATATTTCAGCTCATTGTTTGCAGTTTGTTTATTTTAATAATATTAAATCATATTTTCCTTCAGATGTTGATGGCCGGGATAGCCTAGCCTCGACAGCACATCCTCTTTTACTTCTACTATAGTAATATTCATTTGTTTTATTCCACTTAACA 4200
     ----:----|----:----|----:----|----:----|----:----|----:----|----:----|----:----|----:----|----:----|----:----|----:----|----:----|----:----|----:----|----:----|----:----|----:----|----:----|----:----|
4001 TGGAAAGGTCATCTACACCCTCAATTATTAATAGACCATATAAGTACATATAAAGTCGAGTAACAAACGTCAAACAAATAAAATTATTATAATTTAGTATAAAAGGAAGTCTACAACTACCGGCCCTATCGGATCGGAGCTGTCGTGTAGGAGAAAATGAAGATGATATCATTATAAGTAAACAAAATAAGGTGAATTGT 4200
     C R E L L H P L * Y N D P I N M Y I E A * Q K C N T * K L L I L D Y K G E S T S P R S L R A E V A C G R K S R S Y Y E N T K N W K V   F6
      G K W Y I H S N I I I Q Y I * T Y K L E N N A T Q K N * Y Y * I M N E K L H Q H G P Y G L R S L V D E K V E V I T I N M Q K I G S L  F5
      V K G T S T P T L L * R T Y E H I N * S M T Q L K N I K I I N F * I K R * I N I A P I A * G R C C M R K * K * * L L I * K N * E V * C  F4

     E Y F I C I N V A M C K V I G Q T L * L I F M C N F H * Q V I H V * D Y L T F K * * Q K T * * F Y I V F V * F L Q T D L I F * Y V F H   F1
      N T L F A L M L P C A R L * V K H F D S Y S C V I F I N K * Y M S E I I * L S N N N K K H N N F T * F L S D F F K L I * Y F D M F F I  F2
       I L Y L H * C C H V Q G Y R S N T L T H I H V * F S L T S N T C L R L F N F Q I I T K N I I I L H S F C L I S S N * S D I L I C F S  F3
4201 GAATACTTTATTTGCATTAATGTTGCCATGTGCAAGGTTATAGGTCAAACACTTTGACTCATATTCATGTGTAATTTTCATTAACAAGTAATACATGTCTGAGATTATTTAACTTTCAAATAATAACAAAAAACATAATAATTTTACATAGTTTTTGTCTGATTTCTTCAAACTGATCTGATATTTTGATATGTTTTTCA 4400
     ----:----|----:----|----:----|----:----|----:----|----:----|----:----|----:----|----:----|----:----|----:----|----:----|----:----|----:----|----:----|----:----|----:----|----:----|----:----|----:----|
4201 CTTATGAAATAAACGTAATTACAACGGTACACGTTCCAATATCCAGTTTGTGAAACTGAGTATAAGTACACATTAAAAGTAATTGTTCATTATGTACAGACTCTAATAAATTGAAAGTTTATTATTGTTTTTTGTATTATTAAAATGTATCAAAAACAGACTAAAGAAGTTTGACTAGACTATAAAACTATACAAAAAGT 4400
      S Y K I Q M L T A M H L T I P * V S Q S M N M H L K * * C T I C T Q S * K V K L Y C F V Y Y N * M T K T Q N R * V S R I N Q Y T K *   F6
     L I S * K C * H Q W T C P * L D F V K V * I * T Y N E N V L V H R L N N L K * I I V F F M I I K C L K Q R I E E F Q D S I K I H K E   F5
      F V K N A N I N G H A L N Y T L C K S E Y E H T I K M L L Y M D S I I * S E F L L L F C L L K V Y N K D S K K L S I Q Y K S I N K   F4

      F L A V L P L F L L F A E Q S L H M Y F P F G N P D V K T P T Q N * * S H L * H G P S H * T H E M G E I S G E S L M L H S I P K S H G  F1
       S W L F F P S F Y F L L N S L C I C T F P L V I Q M * K H Q H K I D E A I Y D M V L R T E P M K W E R F L E N L * C Y I L F Q S H M  F2
     F L G C S S P L S T F C * T V F A Y V L S L W * S R C K N T N T K L M K P S M T W S F A L N P * N G R D F W R I S N V T F Y S K V T W   F3
4401 TTTCTTGGCTGTTCTTCCCCTCTTTCTACTTTTTGCTGAACAGTCTTTGCATATGTACTTTCCCTTTGGTAATCCAGATGTAAAAACACCAACACAAAATTGATGAAGCCATCTATGACATGGTCCTTCGCACTGAACCCATGAAATGGGAGAGATTTCTGGAGAATCTCTAATGTTACATTCTATTCCAAAGTCACATG 4600
     ----:----|----:----|----:----|----:----|----:----|----:----|----:----|----:----|----:----|----:----|----:----|----:----|----:----|----:----|----:----|----:----|----:----|----:----|----:----|----:----|
4401 AAAGAACCGACAAGAAGGGGAGAAAGATGAAAAACGACTTGTCAGAAACGTATACATGAAAGGGAAACCATTAGGTCTACATTTTTGTGGTTGTGTTTTAACTACTTCGGTAGATACTGTACCAGGAAGCGTGACTTGGGTACTTTACCCTCTCTAAAGACCTCTTAGAGATTACAATGTAAGATAAGGTTTCAGTGTAC 4600
      K K A T R G R K R S K A S C D K C I Y K G K P L G S T F V G V C F D H L W R H C P G E C Q V W S I P S I E P S D R I N C E I G F D C  F6
      N R P Q E E G R E V K Q Q V T K A Y T S E R Q Y D L H L F V L V F N I F G D I V H D K A S F G H F P L S K Q L I E L T V N * E L T V H  F5
     M E Q S N K G E K * K K S F L R Q M H V K G K T I W I Y F C W C L I S S A M * S M T R R V S G M F H S L N R S F R * H M R N W L * M   F4

      L G V I G F D V Y T K * F L V F P P T T I M Q S L L R V I E V H P A V L W I L F T S I * F T E R Y V K L Y D N T L G F K P G N S P I  F1
     D S V S * V L M C T Q S S F * Y F H L L P * C S H Y L E S L K C I L Q S Y G F C L L Q Y D S Q R D M S N C M T I R W D S N L A I L P F   F2
      T R C H R F * C V H K V V F S I S T Y Y H N A V I T * S H * S A S C S P M D F V Y F N M I H R E I C Q I V * Q Y V G I Q T W Q F S H L  F3
4601 GACTCGGTGTCATAGGTTTTGATGTGTACACAAAGTAGTTTTTAGTATTTCCACCTACTACCATAATGCAGTCATTACTTAGAGTCATTGAAGTGCATCCTGCAGTCCTATGGATTTTGTTTACTTCAATATGATTCACAGAGAGATATGTCAAATTGTATGACAATACGTTGGGATTCAAACCTGGCAATTCTCCCATT 4800
     ----:----|----:----|----:----|----:----|----:----|----:----|----:----|----:----|----:----|----:----|----:----|----:----|----:----|----:----|----:----|----:----|----:----|----:----|----:----|----:----|
4601 CTGAGCCACAGTATCCAAAACTACACATGTGTTTCATCAAAAATCATAAAGGTGGATGATGGTATTACGTCAGTAATGAATCTCAGTAACTTCACGTAGGACGTCAGGATACCTAAAACAAATGAAGTTATACTAAGTGTCTCTCTATACAGTTTAACATACTGTTATGCAACCCTAAGTTTGGACCGTTAAGAGGGTAA 4800
      P S P T M P K S T Y V F Y N K T N G G V V M I C D N S L T M S T C G A T R H I K N V E I H N V S L Y T L N Y S L V N P N L G P L E G M  F6
       V R H * L N Q H T C L T T K L I E V * * W L A T M V * L * Q L A D Q L G I S K T * K L I I * L S I H * I T H C Y T P I * V Q C N E W  F5
      S E T D Y T K I H V C L L K * Y K W R S G Y H L * * K S D N F H M R C D * P N Q K S * Y S E C L S I D F Q I V I R Q S E F R A I R G N  F4

     W L L L W * K P P I I N M L F A R K Q H E * * E R K * F V M S L N V N F K R S S E K V L S S K I F M T M T S S S G R R * S G W Q P V *   F1
      G S Y S G K N L Q L * T C Y L Q E S N M S D K K E S N L * C L * M * T S S D Q V R K Y C H L K F S * L * H H Q V G D A D Q V G S Q C K  F2
       A L T L V K T S N Y K H V I C K K A T * V I R K K V I C N V S E C K L Q A I K * E S I V I * N F H D Y D I I K W E T L I R L A A S V  F3
4801 TGGCTCTTACTCTGGTAAAAACCTCCAATTATAAACATGTTATTTGCAAGAAAGCAACATGAGTGATAAGAAAGAAAGTAATTTGTAATGTCTCTGAATGTAAACTTCAAGCGATCAAGTGAGAAAGTATTGTCATCTAAAATTTTCATGACTATGACATCATCAAGTGGGAGACGCTGATCAGGTTGGCAGCCAGTGTA 5000
     ----:----|----:----|----:----|----:----|----:----|----:----|----:----|----:----|----:----|----:----|----:----|----:----|----:----|----:----|----:----|----:----|----:----|----:----|----:----|----:----|
4801 ACCGAGAATGAGACCATTTTTGGAGGTTAATATTTGTACAATAAACGTTCTTTCGTTGTACTCACTATTCTTTCTTTCATTAAACATTACAGAGACTTACATTTGAAGTTCGCTAGTTCACTCTTTCATAACAGTAGATTTTAAAAGTACTGATACTGTAGTAGTTCACCCTCTGCGACTAGTCCAACCGTCGGTCACAT 5000
      Q S K S Q Y F G G I I F M N N A L F C C S H Y S L F Y N T I D R F T F K L R D L S T N D D L I K M V I V D D L P L R Q D P Q C G T Y  F6
     K A R V R T F V E L * L C T I Q L F A V H T I L F F T I Q L T E S H L S * A I L H S L I T M * F K * S S M M L H S V S I L N A A L T   F5
      P E * E P L F R W N Y V H * K C S L L M L S L F S L L K Y H R Q I Y V E L S * T L F Y Q * R F N E H S H C * * T P S A S * T P L W H  F4
```

```
 V I P P T M * I D F F S S K Y S A E W K A R G I T S G T F I S F H S K V F M I V D R I Y H L C K F F E M V I P M W L F S S W A * * L H   F1
  S Y L Q Q C K L T F S P Q N I Q Q N G R L V V * L R V H L F L S I R K F L * * L I E F T I F A S F L K W * F Q C G C F P H G H S D S   F2
 S H T S N N V N * L F L L K I F S R M E G S W Y N F G Y I Y F F P F E S F Y D S * * N L P S L Q V F * N G D S N V A V F L M G I V T P   F3
5001 AGTCATACCTCCAACAATGTAAATTGACTTTTTCTCCTCAAAATATTCAGCAGAATGGAAGGCTCGTGGTATAACTTCGGGTACATTTATTTCTTTCCATTCGAAAGTTTTTATGATAGTTGATAGAATTTACCATCTTTGCAAGTTTTTTGAAATGGTGATTCCAATGTGGCTGTTTTCCTCATGGGCATAGTGACTCC 5200
     ----:----|----:----|----:----|----:----|----:----|----:----|----:----|----:----|----:----|----:----|----:----|----:----|----:----|----:----|----:----|----:----|----:----|----:----|----:----|----:----|
5001 TCAGTATGGAGGTTGTTACATTTAACTGAAAAAGAGGAGTTTTATAAGTCGTCTTACCTTCCGAGCACCATATTGAAGCCCATGTAAATAAAGAAAGGTAAGCTTTCAAAAATACTATCAACTATCTTAAATGGTAGAAACGTTCAAAAAACTTTACCACTAAGGTTACACCGACAAAAGGAGTACCCGTATCACTGAGG 5200
  T M G G V I Y I S K K E E F Y E A S H F A R P I V E P V N I E K W E F T K I I T S L I * W R Q L N K S I T I G I H S N E E H A Y H S   F6
 L * V E L L T F Q S K R R L I N L L I S P E H Y L K P Y M * K K G N S L K * S L Q Y F K G D K C T K Q F P S E L T A T K R M P M T V G F5
 L D Y R W C H L N V K E G * F I * C F P L S T T Y S R T C K N R E M R F N K H Y N I S N V M K A L K K F H H N W H P Q K G * P C L S E   F4

  H I K L T Y N C Q Y K S V N D Q I F P L E G F H I V L T L K E M Y I L V V Q S H N Y T L N P V L K L Y K G Q S P W L A V L Y C I H P   F1
 T I * N * H I I A N I S Q * M T R S S L W K V S I L F * P * K R C T F L L Y N L T I I L * T L F * N Y I K V S L H G * L C C I V S T P   F2
  P Y K T D I * L P I * V S E * P D L P S G R F P Y C F D L E R D V H S C C T I S Q L Y F E P C F E I I * R S V S M V S C A V L Y P P Q F3
5201 ACCATATAAAACTGACATATAATTGCCAATATAAGTCAGTGAATGACCAGATCTTCCCTCTGGAAGGTTTCCATATTGTTTTGACCTTGAAAGAGATGTACATTCTTGTTGTACAATCTCACAATTATACTTTGAACCCTGTTTTGAAATTATATAAAGGTCAGTCTCCATGGTTAGCTGTGCTGTATTGTATCCACCCC 5400
     ----:----|----:----|----:----|----:----|----:----|----:----|----:----|----:----|----:----|----:----|----:----|----:----|----:----|----:----|----:----|----:----|----:----|----:----|----:----|----:----|
5201 TGGTATATTTTGACTGTATATTAACGGTTATATTCAGTCACTTACTGGTCTAGAAGGGAGACCTTCCAAAGGTATAACAAAACTGGAACTTTCTCTACATGTAAGAACAACATGTTAGAGTGTTAATATGAAACTTGGGACAAAACTTTAATATATTTCCAGTCAGAGGTACCAATCGACACGACATAACATAGGTGGGG 5400
 W W I F S V Y L Q W Y L D T F S W I K G R S P K W I T K V K F S I Y M R T T C D * L * V K F G T K F N Y L P * D G H N A T S Y Q I W G F6
  G Y L V S M Y N G I Y T L S H G S R G E P L N G Y Q K S R S L S T C E Q Q V I E C N Y K S G Q K S I I Y L D T E M T L Q A T N Y G G   F5
 V M Y F Q C I I A L I L * H I V L D E R Q F T E M N N Q G Q F L H V N K N Y L R V I I S Q V R N Q F * I F T L R W P * S H Q I T D V G F4

 K Q R F Y V Q L P L L L L T V G Q G K L T Q * F Q I L * Y Q * Y * H H G C L L N N Q S E I N P L S P P T K I R P I V W P H Q N I S K T F1
  N K D F M F N Y H C C C * L * G R E N * P S S F K F C D I S N I N I M A A Y * I I N L K * I L * V L P R R F V R S S G P T K I * V R H F2
  T K I L C S I T T A V V D C R A G K I D P V V S N F V I S V I L T S W L L T K * S I * N K S F E S S H E D S S D R L A P P K Y K * D   F3
5401 AAACAAAGATTTTATGTTCAATTACCACTGCTGTTGTTGACTGTAGGGCAGGGAAAATTGACCCAGTAGTTTCAAATTTTGTGATATCAGTAATATTAACATCATGGCTGCTTACTAAATAATCAATCTGAAATAAATCCTTTGAGTCCTCCCACGAAGATTCGTCCGATCGTCTGGCCCCACCAAAATATAAGTAAGAC 5600
     ----:----|----:----|----:----|----:----|----:----|----:----|----:----|----:----|----:----|----:----|----:----|----:----|----:----|----:----|----:----|----:----|----:----|----:----|----:----|----:----|
5401 TTTGTTTCTAAAATACAAGTTAATGGTGACGACAACAACTGACATCCCGTCCCTTTTAACTGGGTCATCAAAGTTTAAAACACTATAGTCATTATAATTGTAGTACCGACGAATGATTTATTAGTTAGACTTTATTTAGGAAACTCAGGAGGGTGCTTCTAAGCAGGCTAGCAGACCGGGGTGGTTTTATATTCATTCTG 5600
 L C L N * T * N G S S N N V T P C P F N V W Y N * I K H Y * Y Y * C * P Q K S F L * D S I F G K L G G V F I R G I T Q G W W F I L L V F6
 W V F I K H E I V V A T T S Q L A P F I S G T T E F K T I D T I N V D H S S V L Y D I Q F L D K S D E W S S E D S R R A G G F Y L Y S F5
  F L S K I N L * W Q Q Q Q S Y P L S F Q G L L K L N Q S I L L I L M M A A * * I I L R F Y I R Q T R G R L N T R D D P G V L I Y T L   F4

 L R S R H L Y H H F A C Q F L Y I H V P P V E K K S P C F N S K S L L L F L V D F P L S K S I D L N G K T C I L I A R V R F T C L L S Y F1
  Y A L V I C I I I L P V N F Y T Y M S L R W K K N H R V L I L S P F F F W L I S P S L N L S I * M V R L A F * * H V Y D L L V Y Y H   F2
 T T L S S F V S S F C L S I S I H T C P S G G K K I T V F * F * V P S F S G * F P P L * I Y R F K W * D L H S D S T C T I Y L F I I I F3
5601 ACTACGCTCTCGTCATTTGTATCATCATTTTGCCTGTCAATTTCTATACATACATGTCCCTCCGGTGGAAAAAAAATCACCGTGTTTAATTCTAAGTCCCTTCTTTTTCTGGTTGATTTCCCCCTCTCTAAATCTATCGATTTAAATGGTAAGACTTGCATTCTGATAGCACGTGTACGATTTACTTGTTTATTATCAT 5800
     ----:----|----:----|----:----|----:----|----:----|----:----|----:----|----:----|----:----|----:----|----:----|----:----|----:----|----:----|----:----|----:----|----:----|----:----|----:----|----:----|
5601 TGATGCGAGAGCAGTAAACATAGTAGTAAAACGGACAGTTAAAGATATGTATGTACAGGGAGGCCACCTTTTTTTTAGTGGCACAAAATTAAGATTCAGGGAAGAAAAAGACCAACTAAAGGGGGAGAGATTTAGATAGCTAAATTTACCATTCTGAACGTAAGACTATCGTGCACATGCTAAATGAACAAATAATAGTA 5800
  S R E R * K Y * * K A Q * N R Y M C T G G T S F F D G H K L E L D R R K R T S K G R E L D I S K F P L V Q M R I A R T R N V Q K N D F6
 V V S E D N T D D N Q R D I E I C V H G E P P F F I V T N * N * T G E K E P Q N G G R * I * R N L H Y S K C E S L V H V I * K N I I M F5
 C * A R T M Q I M M K G T L K * V Y M D R R H F F F * R T K I R L G K K K Q N I E G E R F R D I * I T L S A N Q Y C T Y S K S T * * * F4

  A N N D L R M R T Y A F P V G A * R V L * T F V S A A K A L N V F V G * X   F1
 M L I T T C A C A L T H F R * E H S E Y C K R L L V Q Q K R * T Y L * V K   F2
  C * * R P A H A H L R I S G R S I A S T V N V C * C S K S V E R I C R L X  F3
5801 ATGCTAATAACGACCTGCGCATGCGCACTTACGCATTTCCGGTAGGAGCATAGCGAGTACTGTAAACGTTTGTTAGTGCAGCAAAAGCGTTGAACGTATTTGTAGGTTAAA 5911
     ----:----|----:----|----:----|----:----|----:----|----:----|----:----|----:----|----:----|----:----|----:----|-
5801 TACGATTATTGCTGGACGCGTACGCGTGAATGCGTAAAGGCCATCCTCGTATCGCTCATGACATTTGCAAACAATCACGTCGTTTTCGCAACTTGCATAAACATCCAATTT 5911
 Y A L L S R R M R V * A N G T P A Y R T S Y V N T L A A F A N F T N T P *   F6
  H * Y R G A C A C K R M E P L L M A L V T F T Q * H L L L T S R I Q L N F  F5
 I S I V V Q A H A S V C K R Y S C L S Y Q L R K N T C C F R Q V Y K Y T L   F4
```

Pinctada imbricata (Pim)

WGS: NIJJ01024498.1

14887-21051
Pinctada imbricata breed pearl oyster-OUG PIN_chr1_4433, whole genome shotgun sequence

```
     I A N T L T N V Y A T F A F E * I M Q I K F K K I R V V F L A H R F N S Y Q Y S * C S K L R R K F P * E S C G N I V L C Y F L F * S K   F1
      S Q T H * Q T S M Q L L L L N K L C K L S L K K L E L S F L L T A S I H T S T R S A P N C G G N S H K N H V E I S F F V T S C F D R N  F2
       R K H T N K R L C N F C F * I N Y A N * V * K N * S C L S C S P L Q F I P V L V V L Q I A E E I P I R I M W K Y R S L L L P V L I E   F3
   1 ATCGCAAACACACTAACAAACGTCTATGCAACTTTTGCTTTTGAATAAATTATGCAAATTAAGTTTAAAAAAATTAGAGTTGTCTTTCTTGCTCACCGCTTCAATTCATACCAGTACTCGTAGTGCTCCAAATTGCGGAGGAAATTCCCATAAGAATCATGTGGAAATATCGTTCTTTGTTACTTCCTGTTTTGATCGAA 200
     ----:----|----:----|----:----|----:----|----:----|----:----|----:----|----:----|----:----|----:----|----:----|----:----|----:----|----:----|----:----|----:----|----:----|----:----|----:----|----:----|
   1 TAGCGTTTGTGTGATTGTTTGCAGATACGTTGAAAACGAAAACTTATTTAATACGTTTAATTCAAATTTTTTTAATCTCAACAGAAAGAACGAGTGGCGAAGTTAAGTATGGTCATGAGCATCACGAGGTTTAACGCCTCCTTTAAGGGTATTCTTAGTACACCTTTATAGCAAGAAACAATGAAGGACAAAACTAGCTT 200
      X A F V S V F T * A V K A K S Y I I C I L N L F I L T T K R A * R K L E Y W Y E Y H E L N R L F N G Y S D H P F I T R Q * K R N Q D F  F6
     X R L C V L L R R H L K Q K Q I F * A F * T * F F * L Q R E Q E G S * N M G T S T T S W I A S S I G M L I M H F Y R E K N S G T K I S   F5
       D C V C * C V D I C S K S K F L N H L N L K F F N S N D K K S V A E I * V L V R L A G F Q P P F E W L F * T S I D N K T V E Q K S R   F4

      H Q * K S Y * N R P R N G F L A I L * S L V N I L E K I Y T F E V * N K K N C I K S S A N I T D T V Q F F S D S P E I L L S F R I G L  F1
       I S E K V I E I D Q E M D F * Q F Y E V W * I F * K K Y T H L R Y E I K K I V * N Q V Q I L Q I L C N F F Q I L Q K F Y F H F V * V   F2
     T S V K K L L K S T K K W I F S N F M K F G E Y F R K N I H I * G M K * K K L Y K I K C K Y Y R Y C A I F F R F S R N S T F I S Y R S   F3
 201 ACATCAGTGAAAAAGTTATTGAAATCGACCAAGAAATGGATTTTTAGCAATTTTATGAAGTTTGGTGAATATTTTAGAAAAAATATACACATTTGAGGTATGAAATAAAAAAAATTGTATAAAATCAAGTGCAAATATTACAGATACTGTGCAATTTTTTTCAGATTCTCCAGAAATTCTACTTTCATTTCGTATAGGTC 400
     ----:----|----:----|----:----|----:----|----:----|----:----|----:----|----:----|----:----|----:----|----:----|----:----|----:----|----:----|----:----|----:----|----:----|----:----|----:----|----:----|
 201 TGTAGTCACTTTTTCAATAACTTTAGCTGGTTCTTTACCTAAAAATCGTTAAAATACTTCAAACCACTTATAAAATCTTTTTTATATGTGTAAACTCCATACTTTATTTTTTTTAACATATTTTAGTTCACGTTTATAATGTCTATGACACGTTAAAAAAAGTCTAAGAGGTCTTTAAGATGAAAGTAAAGCATATCCAG 400
       C * H F L * Q F R G L F P N K A I K H L K T F I K S F I Y V N S T H F L F F Q I F D L A F I V S V T C N K E S E G S I R S E N R I P   F6
      V D T F F N N F D V L F H I K L L K I F N P S Y K L F F I C M Q P I F Y F F N Y L I L H L Y * L Y Q A I K K L N E L F E V K M E Y L D  F5
     F M L S F T I S I S W S I S K * C N * S T Q H I N * F F Y V C K L Y S I F F I T Y F * T C I N C I S H L K K * I R W F N * K * K T Y T   F4

       N V R H S I V F C V N S L E T R H V P L * Y A K R I I D G K S I I P Y I D F N C C S N V T N Y N I E N E G C M H G K K G V S Q P F *  F1
     S M C V I L S F F A S T L L K L G M S H Y S M Q N E S L M E S Q * Y H I * I L I V A A M L L I T I * K M R D A C M V K R G C H S L S D   F2
      Q C A S F Y R F L R Q L S * N * A C P I I V C K T N H * W K V N N T I Y R F * L L Q Q C Y * L Q Y R K * G M H A W * K G G V T A F L T  F3
 401 TCAATGTGCGTCATTCTATCGTTTTTGCGTCAACTCTCTTGAAACTAGGCATGTCCCATTATAGTATGCAAAACGAATCATTGATGGAAAGTCAATAATACCATATATAGATTTAATTGTTGCAGCAATGTTACTAATTACAATATAGAAAATGAGGGATGCATGCATGGTAAAAAGGGGGTGTCACAGCCTTTCTGA 600
     ----:----|----:----|----:----|----:----|----:----|----:----|----:----|----:----|----:----|----:----|----:----|----:----|----:----|----:----|----:----|----:----|----:----|----:----|----:----|----:----|
 401 AGTTACACGCAGTAAGATAGCAAAAACGCAGTTGAGAGAACTTTGATCCGTACAGGGTAATATCATACGTTTTGCTTAGTAACTACCTTTCAGTTATTATGGTATATATCTAAAATTAACAACGTCGTTACAATGATTAATGTTATATCTTTTACTCCCTACGTACGTACCATTTTTCCCCCACAGTGTCGGAAAGACT 600
     R L T R * E I T K Q T L E R S V L C T G N Y Y A F R I M S P F D I I G Y I S K L Q Q L L T V L * L I S F S P H M C P L F P T D C G K Q   F6
      * H A D N * R K K R * S E Q F * A H G M I T H L V F * Q H F T L L V M Y L N * N N C C H * * N C Y L F H P I C A H Y F P P T V A K R  F5
       E I H T M R D N K A D V R K F S P M D W * L I C F S D N I S L * Y Y W I Y I K I T A A I N S I V I Y F I L S A H M T F L P H * L R E S   F4

     P N N V I F S * E N R * F C K L N C T R I C T I Q E I F V * H * Q A G N I A V * F N F Q D Y I * L T H I Y Q N L S K I H I F H R L I L   F1
      Q I M L F F P E K T G D S A S * I V P V F V Q S K K S L C S T S R Q A T L Q F N S T F K T T S N * H I Y I K T Y Q R F I F S I D * Y L  F2
       K * C Y F F L R K Q V I L Q A E L Y P Y L Y N P R N L C V A L A G R Q H C S L I Q L S R L H L I D T Y I S K P I K D S Y F P * I N T   F3
 601 CCAAATAATGTTATTTTTTCCTGAGAAAACAGGTGATTCTGCAAGCTGAATTGTACCCGTATTTGTACAATCCAAGAAATCTTTGTGTAGCACTAGCAGGCAGGCAACATTGCAGTTTAATTCAACTTTCAAGACTACATCTAATTGACACATATATATCAAAACCTATCAAAGATTCATATTTTCCATAGATTAATACT 800
     ----:----|----:----|----:----|----:----|----:----|----:----|----:----|----:----|----:----|----:----|----:----|----:----|----:----|----:----|----:----|----:----|----:----|----:----|----:----|----:----|
 601 GGTTTATTACAATAAAAAAGGACTCTTTTGTCCACTAAGACGTTCGACTTAACATGGGCATAAACATGTTAGGTTCTTTAGAAACACATCGTGATCGTCCGTCCGTTGTAACGTCAAATTAAGTTGAAAGTTCTGATGTAGATTAACTGTGTATATATAGTTTTGGATAGTTTCTAAGTATAAAAGGTATCTAATTATGA 800
      G F L T I K E Q S F L H N Q L S F Q V R I Q V I W S I K T Y C * C A P L M A T * N L K * S * M * N V C I Y * F R D F I * I K W L N I S  F6
     V L Y H * K K R L F C T I R C A S N Y G Y K Y L G L F R Q T A S A P L C C Q L K I * S E L S C R I S V Y I D F G I L S E Y K G Y I L V   F5
       W I I N N K G S F V P S E A L Q I T G T N T C D L F D K H L V L L C A V N C N L E V K L V V D L Q C M Y I L V * * L N M N E M S * Y   F4

       V S N F S G I F E T K P W R L L M K C K R M R I T L P I T L Q I * P N F V G C V V P S K R G R V E L F V Q L N L I K * N F Y H Y S K L  F1
      * V T F Q E F L R P N H G G C * * N A K G C G * H C Q S L C K F D Q I L * G V W Y H P K E E E * S F L S S * T L * N R T F I I I Q N   F2
     C K * L F R N F * D Q T M E A A D E M Q K D A D N I A N H F A N L T K F C R V C G T I Q K R K S R A F C P V E P Y K I E L L S L F K I   F3
 801 TGTAAGTAACTTTTCAGGAATTTTTGAGACCAAACCATGGAGGCTGCTGATGAAATGCAAAAGGATGCGGATAACATTGCCAATCACTTTGCAAATTTGACCAAATTTTGTAGGGTGTGTGGTACCATCCAAAAGAGGAAGAGTAGAGCTTTTTGTCCAGTTGAACCTTATAAAATAGAACTTTTATCATTATTCAAAAT 1000
     ----:----|----:----|----:----|----:----|----:----|----:----|----:----|----:----|----:----|----:----|----:----|----:----|----:----|----:----|----:----|----:----|----:----|----:----|----:----|----:----|
 801 ACATTCATTGAAAAGTCCTTAAAAACTCTGGTTTGGTACCTCCGACGACTACTTTACGTTTTCCTACGCCTATTGTAACGGTTAGTGAAACGTTTAAACTGGTTTAAAACATCCCACACACCATGGTAGGTTTTCTCCTTCTCATCTCGAAAAACAGGTCAACTTGGAATATTTTATCTTGAAAATAGTAATAAGTTTTA 1000
       T L L K E P I K S V L G H L S S I F H L L I R I V N G I V K C I Q G F K T P H T T G D L L P L T S S K T W N F R I F Y F K * * * E F   F6
      Q L Y S K L F K Q S W V M S A A S S I C F S A S L M A L * K A F K V L N Q L T H P V M W F L F L L A K Q G T S G * L I S S K D N N L I  F5
     K Y T V K * S N K L G F W P P Q Q H F A F P H P Y C Q W D S Q L N S W I K Y P T H Y W G F S S S Y L K K D L Q V K Y F L V K I M I * F   F4

       I S N M M M K V F I L R N C A T H A G Q N A F I F A Q R K A K E I H F L * T F L L S I C T F L I M Q I V K F V I G I L D E M L P * S   F1
      * Y P I * * * K Y S S * E I V Q L M P V K M L S F S H K E R R R K Y I F Y E H S C * A F V H S S * C K L * S L S * E F W T K C Y H R V  F2
       D I Q Y D D E S I H P K K L C N S C R S K C F H F R T K K G E G N T F S M N I P V K H L Y I P H N A N C K V C H R N S G R N A T I E L  F3
1001 TGATATCCAATATGATGATGAAAGTATTCATCCTAAGAAATTGTGCAACTCATGCCGGTCAAAATGCTTTCATTTTCGCACAAAGAAAGGCGAAGGAAATACATTTTCTATGAACATTCCTGTTAAGCATTTGTACATTCCTCATAATGCAAATTGTAAAGTTTGTCATAGGAATTCTGGACGAAATGCTACCATAGAGT 1200
     ----:----|----:----|----:----|----:----|----:----|----:----|----:----|----:----|----:----|----:----|----:----|----:----|----:----|----:----|----:----|----:----|----:----|----:----|----:----|----:----|
1001 ACTATAGGTTATACTACTACTTTCATAAGTAGGATTCTTTAACACGTTGAGTACGGCCAGTTTTACGAAAGTAAAAGCGTGTTTCTTTCCGCTTCCTTTATGTAAAAGATACTTGTAAGGACAATTCGTAAACATGTAAGGAGTATTACGTTTAACATTTCAAACAGTATCCTTAAGACCTGCTTTACGATGGTATCTCA 1200
     N I D L I I I F T N M R L F Q A V * A P * F A K M K A C L F A F S I C K R H V N R N L M Q V N R M I C I T F N T M P I R S S I S G Y L   F6
       S I W Y S S S L I * G L F N H L E H R D F H K * K R V F F P S P F V N E I F M G T L C K Y M G * L A F Q L T Q * L F E P R F A V M S   F5
      Q Y G I H H H F Y E D * S I T C S M G T L I S E N E C L S L R L F Y M K * S C E Q * A N T C E E Y H L N Y L K D Y S N Q V F H * W L T  F4

     C F K F I I I H L Q K F P * N M * M G E V H V A T L N Q T V R I K D Q M I I L I H F Q L L A M * L H Q Q V A I F L Q L K W T N Q K F M   F1
      V S N S L * S I F R S S H R T C R W G R Y M * Q H * I R Q * G S K I K * S F S F I F S Y L P C S S I N K * P F F C S * N G Q I R N L C  F2
       F Q I H Y N P S S E V P I E H V D G G G T C S N T E S D S E D Q R S N D H S H S F S A T C H V A P S T S S H F F A A E M D K S E I Y   F3
1201 TGTTTCAAATTCATTATAATCCATCTTCAGAAGTTCCCATAGAACATGTAGATGGGGGAGGTACATGTAGCAACACTGAATCAGACAGTGAGGATCAAAGATCAAATGATCATTCTCATTCATTTTCAGCTACTTGCCATGTAGCTCCATCAACAAGTAGCCATTTTTTTGCAGCTGAAATGGACAAATCAGAAATTTAT 1400
     ----:----|----:----|----:----|----:----|----:----|----:----|----:----|----:----|----:----|----:----|----:----|----:----|----:----|----:----|----:----|----:----|----:----|----:----|----:----|----:----|
1201 ACAAAGTTTAAGTAATATTAGGTAGAAGTCTTCAAGGGTATCTTGTACATCTACCCCCTCCATGTACATCGTTGTGACTTAGTCTGTCACTCCTAGTTTCTAGTTTACTAGTAAGAGTAAGTAAAAGTCGATGAACGGTACATCGAGGTAGTTGTTCATCGGTAAAAAAACGTCGACTTTACCTGTTTAGTCTTTAAATA 1400
      Q K L N M I I W R * F N G Y F M Y I P S T C T A V S F * V T L I L S * I I M R M * K * S S A M Y S W * C T A M K K C S F H V F * F N I  F6
     N N * I * * L G D E S T G M S C T S P P P V H L L V S D S L S S * L D F S * E * E N E A V Q W T A G D V L L W K K A A S I S L D S I *   F5
       T E F E N Y D M K L L E W L V H L H P L Y M Y C C Q I L C H P D F I L H D N E N M K L * K G H L E M L L Y G N K Q L Q F P C I L F K   F4

      H L L F Q K I Q M N Y V N Q L Q R N Y V D P K E Y L M L L N N * I L T A Q Y M C H P H S L * L I Y Q A F P * N K L L N S H W P R Y S S  F1
       I F S F R K F K * T M * T S F K E I M * T Q R N T * C Y * T I R F * Q H S T C A T L I H C N * F I K H S H R T S Y * T V I G Q D I P   F2
     A S S L S E N S N E L C E P A S K K L C R P K G I L D V T K Q L D F D S T V H V P P S F T V T D L S S I P I E Q A I E Q S L A K I F Q   F3
1401 GCATCTTCTCTTTCAGAAAATTCAAATGAACTATGTGAACCAGCTTCAAAGAAATTATGTAGACCCAAAGGAATACTTGATGTTACTAAACAATTAGATTTTGACAGCACAGTACATGTGCCACCCTCATTCACTGTAACTGATTTATCAAGCATTCCCATAGAACAAGCTATTGAACAGTCATTGGCCAAGATATTCCA 1600
     ----:----|----:----|----:----|----:----|----:----|----:----|----:----|----:----|----:----|----:----|----:----|----:----|----:----|----:----|----:----|----:----|----:----|----:----|----:----|----:----|
1401 CGTAGAAGAGAAAGTCTTTTAAGTTTACTTGATACACTTGGTCGAAGTTTCTTTAATACATCTGGGTTTCCTTATGAACTACAATGATTTGTTAATCTAAAACTGTCGTGTCATGTACACGGTGGGAGTAAGTGACATTGACTAAATAGTTCGTAAGGGTATCTTGTTCGATAACTTGTCAGTAACCGGTTCTATAAGGT 1600
       C R R K * F I * I F * T F W S * L F * T S G F S Y K I N S F L * I K V A C Y M H W G * E S Y S I * * A N G Y F L S N F L * Q G L Y E   F6
      A D E R E S F E F S S H S G A E F F N H L G L P I S S T V L C N S K S L V T C T G G E N V T V S K D L M G M S C A I S C D N A L I N W  F5
     H M K E K L F N L H V I H V L K L S I I Y V W L F V Q H * * V I L N Q C C L V H A V R M * Q L Q N I L C E W L V L * Q V T M P W S I G   F4

       A Q F V L V Y Q L L L L * L I V N I F F A D H V Y L H G Y * M Q V F V Q F V E N Q * * * M R L A F L Q A N Y P * C M I F Y M F H V G   F1
      V H N L S W Y T N C S C Y N S L * T Y F L Q I M Y I F M A I E C R C L S N L * R T S S D K * D * H S Y R P I I L D V * Y F T C F M * V  F2
      C T I C L G I P T A P A I T H C K H I F C R S C I S S W L L N A G V C P I C R E P V V I N E I S I L T G Q L S L M Y D I L H V S C R F  F3
1601 GTGCACAATTTGTCTTGGTATACCAACTGCTCCTGCTATAACTCATTGTAAACATATTTTTTGCAGATCATGTATATCTTCATGGCTATTGAATGCAGGTGTTTGTCCAATTTGTAGAGAACCAGTAGTGATAAATGAGATTAGCATTCTTACAGGCCAATTATCCTTGATGTATGATATTTTACATGTTTCATGTAGGT 1800
     ----:----|----:----|----:----|----:----|----:----|----:----|----:----|----:----|----:----|----:----|----:----|----:----|----:----|----:----|----:----|----:----|----:----|----:----|----:----|----:----|
1601 CACGTGTTAAACAGAACCATATGGTTGACGAGGACGATATTGAGTAACATTTGTATAAAAAACGTCTAGTACATATAGAAGTACCGATAACTTACGTCCACAAACAGGTTAAACATCTCTTGGTCATCACTATTTACTCTAATCGTAAGAATGTCCGGTTAATAGGAACTACATACTATAAAATGTACAAAGTACATCCA 1800
     L A C N T K T Y W S S R S Y S M T F M N K A S * T Y R * P * Q I C T N T W N T S F W Y H Y I L N A N K C A L * G Q H I I N * M N * T P   F6
       H V I Q R P I G V A G A I V * Q L C I K Q L D H I D E H S N F A P T Q G I Q L S G T T I F S I L M R V P W N D K I Y S I K C T E H L   F5
       T C L K D Q Y V L Q E Q * L E N Y V Y K K C I M Y I K M A I S H L H K D L K Y L V L L S L H S * C E * L G I I R S T H Y K V H K M Y T  F4

     L I I V M K S * D L I K F Q I M K M Y V N M E S I P Q S F * H L H Q * * E E E R G C K K C L F M I A R Q N M L S K K G L K K * K A Q L   F1
      * * L * * S L R T * * N F R S * K C M * I W K V S L K A F D T F I N S K K R K G V A K N A Y L * L Q G K I C * A K K A * R S R K L S *  F2
       D N C N E V L G L D K I S D H E N V C K Y G K Y P S K L L T P S S I V R R G K G L Q K M P I Y D C K A K Y V K Q K R L K E V E S S V   F3
1801 TTGATAATTGTAATGAAGTCTTAGGACTTGATAAAATTTCAGATCATGAAAATGTATGTAAATATGGAAAGTATCCCTCAAAGCTTTTGACACCTTCATCAATAGTAAGAAGAGGAAAGGGGTTGCAAAAAATGCCTATTTATGATTGCAAGGCAAAATATGTTAAGCAAAAAAGGCTTAAAGAAGTAGAAAGCTCAGTT 2000
     ----:----|----:----|----:----|----:----|----:----|----:----|----:----|----:----|----:----|----:----|----:----|----:----|----:----|----:----|----:----|----:----|----:----|----:----|----:----|----:----|
1801 AACTATTAACATTACTTCAGAATCCTGAACTATTTTAAAGTCTAGTACTTTTACATACATTTATACCTTTCATAGGGAGTTTCGAAAACTGTGGAAGTAGTTATCATTCTTCTCCTTTCCCCAACGTTTTTTACGGATAAATACTAACGTTCCGTTTTATACAATTCGTTTTTTCCGAATTTCTTCATCTTTCGAGTCAA 2000
      K I I T I F D * S K I F N * I M F I Y T F I S L I G * L K Q C R * * Y Y S S S L P Q L F H R N I I A L C F I N L L F P K F F Y F A * N  F6
     N S L Q L S T K P S S L I E S * S F T H L Y P F Y G E F S K V G E D I T L L P F P N C F I G I * S Q L A F Y T L C F L S L S T S L E T   F5
       Q Y N Y H L R L V Q Y F K L D H F H I Y I H F T D R L A K S V K M L L L F L F P T A F F A * K H N C P L I H * A F F A * L L L F S L   F4

       N N F V L Q K M R I K Q M F Y F S C L F I I * R A K M I I D L N K L M M F G W E G E S E N * Q V M N A L Q S E * T L Y R V K L N I K H  F1
        T I L F F K K * G * N R C F I F H A Y S S F E E P K * * * I * T N * * C L D G R G S Q K I N K S * M P C N Q N R H F T E * N S I * S   F2
     E Q F C S S K N E D K T D V L F F M L I H H L K S Q N D N R F E Q I N D V W M G G G V R K L T S H E C L A I R I D T L Q S K T Q Y K A   F3
2001 GAACAATTTTGTTCTTCAAAAAATGAGGATAAAACAGATGTTTTATTTTTCATGCTTATTCATCATTTGAAGAGCCAAAATGATAATAGATTTGAACAAATTAATGATGTTTGGATGGGAGGGGGAGTCAGAAAATTAACAAGTCATGAATGCCTTGCAATCAGAATAGACACTTTACAGAGTAAAACTCAATATAAAGC 2200
     ----:----|----:----|----:----|----:----|----:----|----:----|----:----|----:----|----:----|----:----|----:----|----:----|----:----|----:----|----:----|----:----|----:----|----:----|----:----|----:----|
2001 CTTGTTAAAACAAGAAGTTTTTTACTCCTATTTTGTCTACAAAATAAAAAGTACGAATAAGTAGTAAACTTCTCGGTTTTACTATTATCTAAACTTGTTTAATTACTACAAACCTACCCTCCCCCTCAGTCTTTTAATTGTTCAGTACTTACGGAACGTTAGTCTTATCTGTGAAATGTCTCATTTTGAGTTATATTTCG 2200
       F L K T R * F I L I F C I N * K E H K N M M Q L A L I I I S K F L N I I N P H S P S D S F * C T M F A K C D S Y V S * L T F S L I F   F6
      S C N Q E E F F S S L V S T K N K M S I * * K F L W F S L L N S C I L S T Q I P P P T L F N V L * S H R A I L I S V K C L L V * Y L A  F5
     Q V I K N K L F H P Y F L H K I K * A * E D N S S G F H Y Y I Q V F * H H K S P L P L * F I L L D H I G Q L * F L C K V S Y F E I Y L   F4

       S M I S * R L R D V T H L * L H M H * M K L S V S M S L A Q L D I Q L K V R I K S L T I I T H Q S K * I E N V L L H P * T I H L N H   F1
     T V * F L K G * G M * P I C S S I C I R * N * V S V C P W L S * I F N * R S G S S H * L L S H T S Q S K * R M Y Y F T P R Q Y I * T I   F2
      Q Y D F L K A K G C N P F V A P Y A L D E I E C Q Y V P G S V R Y S I E G Q D Q V I N Y Y H T P V K V N R E C T T S P L D N T F E P L  F3
2201 ACAGTATGATTTCTTAAAGGCTAAGGGATGTAACCCATTTGTAGCTCCATATGCATTAGATGAAATTGAGTGTCAGTATGTCCCTGGCTCAGTTAGATATTCAATTGAAGGTCAGGATCAAGTCATTAACTATTATCACACACCAGTCAAAGTAAATAGAGAATGTACTACTTCACCCCTAGACAATACATTTGAACCAT 2400
     ----:----|----:----|----:----|----:----|----:----|----:----|----:----|----:----|----:----|----:----|----:----|----:----|----:----|----:----|----:----|----:----|----:----|----:----|----:----|----:----|
2201 TGTCATACTAAAGAATTTCCGATTCCCTACATTGGGTAAACATCGAGGTATACGTAATCTACTTTAACTCACAGTCATACAGGGACCGAGTCAATCTATAAGTTAACTTCCAGTCCTAGTTCAGTAATTGATAATAGTGTGTGGTCAGTTTCATTTATCTCTTACATGATGAAGTGGGGATCTGTTATGTAAACTTGGTA 2400
     C L I I E * L S L S T V W K Y S W I C * I F N L T L I D R A * N S I * N F T L I L D N V I I V C W D F Y I S F T S S * G * V I C K F W   F6
       C Y S K K F A L P H L G N T A G Y A N S S I S H * Y T G P E T L Y E I S P * S * T M L * * * V G T L T F L S H V V E G R S L V N S G   F5
      V T H N R L P * P I Y G M Q L E M H M L H F Q T D T H G Q S L * I N L Q L D P D L * * S N D C V L * L L Y L I Y * K V G L C Y M Q V M  F4

     * T L * M Y S L L V L L N * Q L L M * R A V V G D I R M Q * P K H C M N * K M T * L Q D Y N N T I L I L M I L Q S C * R P T L R M G L   F1
```

```
      R H C K C I P C L Y S * I S N S * C E G L S L E I S G C N S Q N I A * I R K * H N C R I T T T Q Y * S L * S Y N P V K D L H * G W G *  F2
       D I V N V F P A C T P E L A T P N V K G C R W R Y P D A I A K T L H E L E N D I I A G L Q Q H N I D P Y D P T I L L K T Y I K D G A   F3
2401 TAGACATTGTAAATGTATTCCCTGCTTGTACTCCTGAATTAGCAACTCCTAATGTGAAGGGCTGTCGTTGGAGATATCCGGATGCAATAGCCAAAACATTGCATGAATTAGAAAATGACATAATTGCAGGATTACAACAACACAATATTGATCCTTATGATCCTACAATCCTGTTAAAGACCTACATTAAGGATGGGGCT 2600
     ----:----|----:----|----:----|----:----|----:----|----:----|----:----|----:----|----:----|----:----|----:----|----:----|----:----|----:----|----:----|----:----|----:----|----:----|----:----|----:----|
2401 ATCTGTAACATTTACATAAGGGACGAACATGAGGACTTAATCGTTGAGGATTACACTTCCCGACAGCAACCTCTATAGGCCTACGTTATCGGTTTTGTAACGTACTTAATCTTTTACTGTATTAACGTCCTAATGTTGTTGTGTTATAACTAGGAATACTAGGATGTTAGGACAATTTCTGGATGTAATTCCTACCCCGA 2600
      * V N Y I Y E R S T S R F * C S R I H L A T T P S I R I C Y G F C Q M F * F I V Y N C S * L L V I N I R I I R C D Q * L G V N L I P S  F6
     N S M T F T N G A Q V G S N A V G L T F P Q R Q L Y G S A I A L V N C S N S F S M I A P N C C C L I S G * S G V I R N F V * M L S P A   F5
      L C Q L H I G Q K Y E Q I L L E * H S P S D N S I D P H L L W F M A H I L F H C L Q L I V V V C Y Q D K H D * L G T L S R C * P H P   F4

      M V L V M C L Y T K K * G I D I C Q T R H S D F P F V Y I E * R Q Y * E M M K R S Q S L L K K G Q I L S K Q T D L Y L K Q F V T K I I  F1
       W S W * C V C T Q R S R G * I F A R Q G I Q I F L L C I * N R G N I R R * * K G H N L C * K K A K F C Q N K Q T C T * S N L * R K *   F2
      D G L G D V S V H K E V G D R Y L P D K A F R F S F C V Y R I E A I L G D D E K V T I F V E K R P N S V K T N R P V L E A I C D E N N  F3
2601 GATGGTCTTGGTGATGTGTCTGTACACAAAGAAGTAGGGGATAGATATTTGCCAGACAAGGCATTCAGATTTTCCTTTTGTGTATATAGAATAGAGGCAATATTAGGAGATGATGAAAAGGTCACAATCTTTGTTGAAAAAAGGCCAAATTCTGTCAAAACAAACAGACCTGTACTTGAAGCAATTTGTGACGAAAATAA 2800
     ----:----|----:----|----:----|----:----|----:----|----:----|----:----|----:----|----:----|----:----|----:----|----:----|----:----|----:----|----:----|----:----|----:----|----:----|----:----|----:----|
2601 CTACCAGAACCACTACACAGACATGTGTTTCTTCATCCCCTATCTATAAACGGTCTGTTCCGTAAGTCTAAAAGGAAAACACATATATCTTATCTCCGTTATAATCCTCTACTACTTTTCCAGTGTTAGAAACAACTTTTTTCCGGTTTAAGACAGTTTTGTTTGTCTGGACATGAACTTCGTTAAACACTGCTTTTATT 2800
       I T K T I H R Y V F F Y P I S I Q W V L C E S K G K T Y I S Y L C Y * S I I F L D C D K N F F P W I R D F C V S R Y K F C N T V F I   F6
      S P R P S T D T C L S T P S L Y K G S L A N L N E K Q T Y L I S A I N P S S S F T V I K T S F L G F E T L V F L G T S S A I Q S S F L  F5
     Q H D Q H H T Q V C L L L P Y I N A L C P M * I K R K H I Y F L P L I L L H H F P * L R Q Q F F A L N Q * F L C V Q V Q L L K H R F Y   F4

       M A P V L F A Y C Q * K M K G L I * K V R L S G F K Q S L A G E G M Y * F F L T Q * L M K N W I G P T V D W L V V G P T I Y A H C V   F1
      S W L Q Y S L P I A N R K * K V L Y E R * D Y Q D S N R V W L E K A C T N F F * L N D * * K I G * G Q Q W I G W * W V Q L S M H I V *  F2
       H G S S T L C L L P I E N E R S Y M K G K I I R I Q T E F G W R R H V L I F S N S M I D E K L D R A N S G L A G S G S N Y L C T L C D  F3
2801 TCATGGCTCCAGTACTCTTTGCCTATTGCCAATAGAAAATGAAAGGTCTTATATGAAAGGTAAGATTATCAGGATTCAAACAGAGTTTGGCTGGAGAAGGCATGTACTAATTTTTTCTAACTCAATGATTGATGAAAAATTGGATAGGGCCAACAGTGGATTGGCTGGTAGTGGGTCCAACTATCTATGCACATTGTGTG 3000
     ----:----|----:----|----:----|----:----|----:----|----:----|----:----|----:----|----:----|----:----|----:----|----:----|----:----|----:----|----:----|----:----|----:----|----:----|----:----|----:----|
2801 AGTACCGAGGTCATGAGAAACGGATAACGGTTATCTTTTACTTTCCAGAATATACTTTCCATTCTAATAGTCCTAAGTTTGTCTCAAACCGACCTCTTCCGTACATGATTAAAAAAGATTGAGTTACTAACTACTTTTTAACCTATCCCGGTTGTCACCTAACCGACCATCACCCAGGTTGATAGATACGTGTAACACAC 3000
      I M A G T S K A * Q W Y F I F P R I H F T L N D P N L C L K A P S P M Y * N K R V * H N I F F Q I P G V T S Q S T T P G V I * A C Q T  F6
       * P E L V R Q R N G I S F S L D * I F P L I I L I * V S N P Q L L C T S I K E L E I I S S F N S L A L L P N A P L P D L * R H V N H   F5
      D H S W Y E K G I A L L F H F T K Y S L Y S * * S E F L T Q S S F A H V L K K * S L S Q H F I P Y P W C H I P Q Y H T W S D I C M T H  F4

      M Q Q E K R P Y P I W V I L S * I V H M L K Q Q K L L S T * G * I L I I L P R L I * T R F L R A L N Q P L S S R L N Q * K R A * M L L  F1
       C N K R N G P I Q F G * F C H K S F I C * N S R N C * V H E D K S * * S Y Q G * F K Q D F * G H * I N P S P P G * I N R K G H R C Y S  F2
       A T R E T A L S N L G N F V I N R S Y A E T A E I A E Y M R I N P D N L T K A D L N K I S K G I K S T P L L Q A E S I E K G I D A T   F3
3001 ATGCAACAAGAGAAACGGCCCTATCCAATTTGGGTAATTTTGTCATAAATCGTTCATATGCTGAAACAGCAGAAATTGCTGAGTACATGAGGATAAATCCTGATAATCTTACCAAGGCTGATTTAAACAAGATTTCTAAGGGCATTAAATCAACCCCTCTCCTCCAGGCTGAATCAATAGAAAAGGGCATAGATGCTACT 3200
     ----:----|----:----|----:----|----:----|----:----|----:----|----:----|----:----|----:----|----:----|----:----|----:----|----:----|----:----|----:----|----:----|----:----|----:----|----:----|----:----|
3001 TACGTTGTTCTCTTTGCCGGGATAGGTTAAACCCATTAAAACAGTATTTAGCAAGTATACGACTTTGTCGTCTTTAACGACTCATGTACTCCTATTTAGGACTATTAGAATGGTTCCGACTAAATTTGTTCTAAAGATTCCCGTAATTTAGTTGGGGAGAGGAGGTCCGACTTAGTTATCTTTTCCCGTATCTACGATGA 3200
      I C C S F R G * G I Q T I K D Y I T * I S F C C F N S L V H P Y I R I I K G L S I * V L N R L A N F * G R E E L S F * Y F L A Y I S S  F6
      S A V L S V A R D L K P L K T M F R E Y A S V A S I A S Y M L I F G S L R V L A S K F L I E L P M L D V G R R W A S D I S F P M S A V  F5
       H L L L F P G I W N P Y N Q * L D N M H Q F L L F Q Q T C S S L D Q Y D * W P Q N L C S K * P C * I L G E G G P Q I L L F P C L H *   F4

       M Q I S T V G L F F E S * S L G K * C K L I N G K * V G M * N L L L K M G R L S L I Y I S R K Q * A * T H S * * C L V T M H E Q S L T  F1
       C R Y Q L W V F F S K A N H * G N S A N * * M G N E W G C E I Y Y * R W G G * V * F T S Q E N S R H K P T V D D A W * L C T S N L *   F2
      H A D I N C G S F F R K L I I R E I V Q I N K W E M S G D V K S I I K D G E V K F D L H L K K T V G I N P Q L M M P G N Y A R A I F D  F3
3201 CATGCAGATATCAACTGTGGGTCTTTTTTTCGAAAGCTAATCATTAGGGAAATAGTGCAAATTAATAAATGGGAAATGAGTGGGGATGTGAAATCTATTATTAAAGATGGGGAGGTTAAGTTTGATTTACATCTCAAGAAAACAGTAGGCATAAACCCACAGTTGATGATGCCTGGTAACTATGCACGAGCAATCTTTGA 3400
     ----:----|----:----|----:----|----:----|----:----|----:----|----:----|----:----|----:----|----:----|----:----|----:----|----:----|----:----|----:----|----:----|----:----|----:----|----:----|----:----|
3201 GTACGTCTATAGTTGACACCCAGAAAAAAAGCTTTCGATTAGTAATCCCTTTATCACGTTTAATTATTTACCCTTTACTCACCCCTACACTTTAGATAATAATTTCTACCCCTCCAATTCAAACTAAATGTAGAGTTCTTTTGTCATCCGTATTTGGGTGTCAACTACTACGGACCATTGATACGTGCTCGTTAGAAACT 3400
       M C I D V T P R K K S L * D N P F Y H L N I F P F H T P I H F R N N F I P L N L K I * M E L F C Y A Y V W L Q H H R T V I C S C D K   F6
      * A S I L Q P D K K R F S I M L S I T C I L L H S I L P S T F D I I L S P S T L N S K C R L F V T P M F G C N I I G P L * A R A I K S  F5
      E H L Y * S H T K K E F A L * * P F L A F * Y I P F S H P H S I * * * L H P P * T Q N V D * S F L L C L G V T S S A Q Y S H V L L R Q  F4

       L K M R I L F L I * S K M M R E S A K L D Y Y F K N S D S * E * F I E L L T P K Y S I H Q R L K C T K K L P * S L G K Y * L M T L I   F1
      L * K * E F Y S * F N R R * * E K V Q S * I I T S K I Q I H E N D L * S C * P Q S T V S I R D * N V Q R N C H R V W E N I D * * L * L  F2
       S K N E N S I L D L I E D D E R K C K V R L L L Q K F R F M R M I Y R A V D P K V Q Y P S E T K M Y K E T A I E F G K I L I N D F D Y  F3
3401 CTCTAAAAATGAGAATTCTATTCTTGATTTAATCGAAGATGATGAGAGAAAGTGCAAAGTTAGATTATTACTTCAAAAATTCAGATTCATGAGAATGATTTATAGAGCTGTTGACCCCAAAGTACAGTATCCATCAGAGACTAAAATGTACAAAGAAACTGCCATAGAGTTTGGGAAAATATTGATTAATGACTTTGATT 3600
     ----:----|----:----|----:----|----:----|----:----|----:----|----:----|----:----|----:----|----:----|----:----|----:----|----:----|----:----|----:----|----:----|----:----|----:----|----:----|----:----|
3401 GAGATTTTTACTCTTAAGATAAGAACTAAATTAGCTTCTACTACTCTCTTTCACGTTTCAATCTAATAATGAAGTTTTTAAGTCTAAGTACTCTTACTAAATATCTCGACAACTGGGGTTTCATGTCATAGGTAGTCTCTGATTTTACATGTTTCTTTGACGGTATCTCAAACCCTTTTATAACTAATTACTGAAACTAA 3600
      V R F I L I R N K I * D F I I L S L A F N S * * K L F E S E H S H N I S S N V G F Y L I W * L S F H V F F S G Y L K P F Y Q N I V K I  F6
       E L F S F E I R S K I S S S S S L F H L T L N N S * F N L N M L I I * L A T S G L T C Y G D S V L I Y L S V A M S N P F I N I L S K S  F5
      S * F H S N * E Q N L R L H H S F T C L * I I V E F I * I * S F S K Y L Q Q G W L V T D M L S * F T C L F Q W L T Q S F I S * H S Q N  F4

      M L N G P I T Y I K * L N M Y R N * L K M K M D L E L L E G L V V K G M K E G T R F F D I L G R I W Q G G V V H M G V * E M Y F G V I  F1
      C * M A Q L H T * S D * T C T G I N * K * K W T W N C W R A * * * R E * R R E Q D F S T F * E E F G K E G * Y I W G S K R C T L G S L  F2
       A K W P N Y I H K V I E H V Q E L I E N E N G P G T V G G L S S E G N E G G N K I F R H F R K N L A R R G S T Y G G L R D V L W G H  F3
3601 ATGCTAAATGGCCCAATTACATACATAAAGTGATTGAACATGTACAGGAATTAATTGAAAATGAAAATGGACCTGGAACTGTTGGAGGGCTTAGTAGTGAAGGGAATGAAGGAGGGAACAAGATTTTTCGACATTTTAGGAAGAATTTGGCAAGGAGGGGTAGTACATATGGGGGTCTAAGAGATGTACTTTGGGGTCAT 3800
     ----:----|----:----|----:----|----:----|----:----|----:----|----:----|----:----|----:----|----:----|----:----|----:----|----:----|----:----|----:----|----:----|----:----|----:----|----:----|----:----|
3601 TACGATTTACCGGGTTAATGTATGTATTTCACTAACTTGTACATGTCCTTAATTAACTTTTACTTTTACCTGGACCTTGACAACCTCCCGAATCATCACTTCCCTTACTTCCTCCCTTGTTCTAAAAAGCTGTAAAATCCTTCTTAAACCGTTCCTCCCCATCATGTATACCCCCAGATTCTCTACATGAAACCCCAGTA 3800
      I S F P G I V Y M F H N F M Y L F * N F I F I S R S S N S P K T T F P I F S P V L N K S M K P L I Q C P P T T C I P T * S I Y K P T M  F6
      * A L H G L * M C L T I S C T C S N I S F S F P G P V T P P S L L S P F S P P F L I K R C K L F F K A L L P L V Y P P R L S T S Q P *  F5
       H * I A W N C V Y L S Q V H V P I L Q F H F H V Q F Q Q L A * Y H L S H L L S C S K E V N * S S N P L S P Y Y M H P D L L H V K P D   F4

       G Y T V V H L * Q N Y Q L N Q N L N K D A V H V D L W D I I L E T V H L L * K M S K F V I A Y M Y L F L K I F C V T V Y T P I L L T P  F1
       V I Q * S I S N K T I S * I K I * T K M Q C M * I F G T * Y * K L S I F C R K C L N L L * H I C I Y F * K Y F V L Q C T L Q F Y * H  F2
      W L Y S S P S L T K L S A K S K S E Q R C S A C R S L G H N I R N C P S S V E N V * I C Y S I Y V S I S E N I L C Y S V H S N F I D T  F3
3801 TGGTTATACAGTAGTCCATCTCTAACAAAACTATCAGCTAAATCAAAATCTGAACAAAGATGCAGTGCATGTAGATCTTTGGGACATAATATTAGAAACTGTCCATCTTCTGTAGAAAATGTCTAAATTTGTTATAGCATATATGTATCTATTTCTGAAAATATTTTGTGTTACAGTGTACACTCCAATTTTATTGACAC 4000
     ----:----|----:----|----:----|----:----|----:----|----:----|----:----|----:----|----:----|----:----|----:----|----:----|----:----|----:----|----:----|----:----|----:----|----:----|----:----|----:----|
3801 ACCAATATGTCATCAGGTAGAGATTGTTTTGATAGTCGATTTAGTTTTAGACTTGTTTCTACGTCACGTACATCTAGAAACCCTGTATTATAATCTTTGACAGGTAGAAGACATCTTTTACAGATTTAAACAATATCGTATATACATAGATAAAGACTTTTATAAAACACAATGTCACATGTGAGGTTAAAATAACTGTG 4000
       P * V T T W R * C F * * S F * F R F L S A T C T S R Q S M I N S V T W R R Y F I D L N T I A Y I Y R N R F I N Q T V T Y V G I K N V   F6
       Q N Y L L G D R V F S D A L D F D S C L H L A H L D K P C L I L F Q G D E T S F T * I Q * L M Y T D I E S F I K H * L T C E L K I S V  F5
      N T I C Y D M E L L V I L * I L I Q V F I C H M Y I K P V Y Y * F S D M K Q L F H R F K N Y C I H I * K Q F Y K T N C H V S W N * Q C  F4

       F Q Q M L E L I I I W Y I H V Y F S S L F A V C L F * * Y * I I F S F R C * W P G * P S L D S T S S F T S T I V I F I C F I P L N R   F1
      L S S R C W S * * L S G T F M Y I S A H C L Q F V Y F N N I K S Y F P S D V D G R D S L A S T A H P L L L L L * * Y S F V L F H L T E  F2
       F P A D V G V N N Y L V H S C I F Q L I V C S L F I L I I L N H I F L Q M L M A G I A * P R Q H I L F Y F Y Y S N I H L F Y S T * Q N  F3
4001 CTTTCCAGCAGATGTTGGAGTTAATAATTATCTGGTACATTCATGTATATTTCAGCTCATTGTTTGCAGTTTGTTTATTTTAATAATATTAAATCATATTTCCTTCAGATGTTGATGGCCGGGATAGCCTAGCCTCGACAGCACATCCTCTTTTACTTCTACTATAGTAATATTCATTTGTTTATTCCACTTAACAGA 4200
     ----:----|----:----|----:----|----:----|----:----|----:----|----:----|----:----|----:----|----:----|----:----|----:----|----:----|----:----|----:----|----:----|----:----|----:----|----:----|----:----|
4001 GAAAGGTCGTCTACAACCTCAATTATTAATAGACCATGTAAGTACATATAAAGTCGAGTAACAAACGTCAAACAAATAAAATTATTATAATTTAGTATAAAAGGAAGTCTACAACTACCGGCCCTATCGGATCGGAGCTGTCGTGTAGGAGAAAATGAAGATGATATCATTATAAGTAAACAAAATAAGGTGAATTGTCT 4200
      G K W C I N S N I I I Q Y M * T Y K L E N N A T Q K N * Y Y * I M N E K L H Q H G P Y G L R S L V D E K V E V I T I N M Q K I G S L L  F6
       K G A S T P T L L * R T C E H I N * S M T Q L K N I K I I N F * I K R * I N I A P I A * G R C C M R K * K * * L L I * K N * E V * C   F5
       R E L L H Q L * Y N D P V N M Y I E A * Q K C N T * K L L I L D Y K G E S T S P R S L R A E V A C G R K S R S Y Y Y E N T K N W K V S  F4

      I L Y L H * C C H V Q G Y R S N T L T N I H V * F S L T S N T C L R L F N F Q I I T K N I I I L H S F C L I S S N * S D I L I C F S F  F1
       Y F I C I N V A M C K V I G Q T L * L I F M C N F H * Q V I H V * D Y L T F K * * Q K T * * F Y I V F V * F L Q T D L I F * Y V F H F  F2
       T L F A L M L P C A R L * V K H F D * Y S C V I F I N K * Y M S E I I * L S N N N K K H N N S T * F L S D F F K L I * Y F D M F F I   F3
4201 ATACTTTATTTGCATTAATGTTGCCATGTGCAAGGTTATAGGTCAAACACTTTGACTAATATTCATGTGTAATTTTCATTAACAAGTAATACATGTCTGAGATTATTTAACTTTCAAATAATAACAAAAAACATAATAATTCTACATAGTTTTTGTCTGATTTCTTCAAACTGATCTGATATTTTGATATGTTTTTCATT 4400
     ----:----|----:----|----:----|----:----|----:----|----:----|----:----|----:----|----:----|----:----|----:----|----:----|----:----|----:----|----:----|----:----|----:----|----:----|----:----|----:----|
4201 TATGAAATAAACGTAATTACAACGGTACACGTTCCAATATCCAGTTTGTGAAACTGATTATAAGTACACATTAAAAGTAATTGTTCATTATGTACAGACTCTAATAAATTGAAAGTTTATTATTGTTTTTTGTATTATTAAGATGTATCAAAAACAGACTAAAGAAGTTTGACTAGACTATAAAACTATACAAAAAGTAA 4400
       I S * K C * H Q W T C P * L D F V K V L I * T Y N E N V L L V H R L N N L K * I I V F F M I I R C L K Q R I E E F Q D S I K I H K E N  F6
      F V K N A N I N G H A L N Y T L C K S * Y E H T I K M L L Y Y M D S I I * S E F L L L F C L L E V Y N K D S K K L S I Q Y K S I N K M  F5
       Y K I Q M L T A M H L T I P * V S Q S I N M H L K * * C T I C T Q S * K V K L Y Y C F V Y Y N * M T K T Q N R * V S R I N Q Y T K *  F4

       L G * Y S R P S P H S F R * L G N A L P F Q V F I L L R L S S I L C K F Q V G N G I G G * S T M V W K G E D L L Y S E T S P W G M I Y  F1
       L A D I V D H P P I L F V N S E M P Y R F K F S F Y Y A Y H Q F C V N S K S V T V * G D D L L W F G R G K I C Y I V K H P P G G * S   F2
      S W L I * * T I P P F F S L T R K C L T V S S F H F I T L I I N F V * I P S R * R Y R G M I Y Y G L E G G R S A I * * N I P L G D D L  F3
4401 TCTTGGCTGATATAGTAGACCATCCCCCCATTCTTTTCGTTAACTCGGAAATGCCTTACCGTTTCAAGTTTTCATTTTATTACGCTTATCATCAATTTTGTGTAAATTCCAAGTCGGTAACGGTATAGGGGGATGATCTACTATGGTTTGGAAGGGGGAAGATCTGCTATATAGTGAAACATCCCCCTGGGGGATGATCT 4600
     ----:----|----:----|----:----|----:----|----:----|----:----|----:----|----:----|----:----|----:----|----:----|----:----|----:----|----:----|----:----|----:----|----:----|----:----|----:----|----:----|
4401 AGAACCGACTATATCATCTGGTAGGGGGGTAAGAAAAGCAATTGAGCCTTTACGGAATGGCAAAGTTCAAAAGTAAAATAATGCGAATAGTAGTTAAAACACATTTAAGGTTCAGCCATTGCCATATCCCCCTACTAGATGATACCAAACCTTCCCCCTTCTAGACGATATATCACTTTGTAGGGGGACCCCCTACTAGA 4600
       R P Q Y L L G D G W E K R * S P F A K G N * T K M K N R K D D I K H L N W T P L P I P P H D V I T Q F P S S R S Y L S V D G Q P I I   F6
       E Q S I Y Y V M G G N K E N V R F H R V T E L K * K I V S I M L K T Y I G L R Y R Y L P I I * * P K S P P L D A I Y H F M G R P S S R  F5
      K K A S I T S W G G M R K T L E S I G * R K L N E N * * A * * * N Q T F E L D T V T Y P S S R S H N P L P F I Q * I T F C G G P P H D   F4

       Y I A D H P G G R P A M G D D P L Y N T G I L L F K S V L K Y L K C F Q T C E * I T * C K L G T L R H S * S L I R N T H V W Y K T F   F1
      T I * Q I I R G E D L L W G M I H Y I T P A Y Y F S S P F L N I * N V S K H V N K L H N V N * G H * D I H N P L S E T R M S G T R H L   F2
       L Y S R S S G G K T C Y G G * S T I * H R H T T F Q V R S * I F K M F P N M * I N Y I M * I R D I E T F I I P Y P K H A C L V Q D I *  F3
4601 ACTATATAGCAGATCATCCGGGGGGAAGACCTGCTATGGGGGATGATCCACTATATAACACCGGCATACTACTTTTCAAGTCCGTTCTTAAATATTTAAAATGTTTCCAAACATGTGAATAAATTACATAATGTAAATTAGGGACATTGAGACATTCATAATCCCTTATCCGAAACACGCATGTCTGGTACAAGACATTT 4800
     ----:----|----:----|----:----|----:----|----:----|----:----|----:----|----:----|----:----|----:----|----:----|----:----|----:----|----:----|----:----|----:----|----:----|----:----|----:----|----:----|
4601 TGATATATCGTCTAGTAGGCCCCCCTTCTGGACGATACCCCCTACTAGGTGATATATTGTGGCCGTATGATGAAAAGTTCAGGCAAGAATTTATAAATTTTACAAAGGTTTGTACACTTATTTAATGTATTACATTTAATCCCTGTAACTCTGTAAGTATTAGGGAATAGGCTTTGTGCGTACAGACCATGTTCTGTAAA 4800
      * * I A S * G P P L G A I P S S G S Y L V P M S S K L D T R L Y K F H K W V H S Y I V Y H L N P V N L C E Y D R I R F V C T Q Y L V N  F6
       S Y L L D D P P F V Q * P P H D V I Y C R C V V K * T R E * I N L I N G F M H I F * M I Y I L S M S V N M I G * G F C A H R T C S M   F5
       V I Y C I M R P S S R S H P I I W * I V G A Y * K E L G N K F I * F T E L C T F L N C L T F * P C Q S M * L G K D S V R M D P V L C K  F4

      R K F S Q K A F L * Q Q F W T L R F S L L H G R Y T * L Y I F N T G M K Y H R F V T N P F L N T * K K P F L L Q V Y I K N R T L P I *  F1
       E N S P R R R F F D N N F G H F V F L Y Y T V D T H N Y T S L T Q G * S I I D S S Q T L S S T H E K N P F F C K S I L K T G H F L Y N  F2
       K I L P E G V S L T T I L D T S F F F I T R S I H I I I H L * H R D E V S S I R H K P F P Q H M K K T L S S A S L Y * K Q D T S Y I   F3
4801 AGAAAATTCTCCCAGAAGGCGTTTCTTTGACAACAATTTTGGACACTTCGTTTTTCTTTATTACACGGTCGATACACATAATTATACATCTTTAACACAGGGATGAAGTATCATCGATTCGTCACAAACCCTTTCCTCAACACATGAAAAAAACCCTTTCTTCTGCAAGTCTATATTAAAAACAGGACACTTCCTATATA 5000
     ----:----|----:----|----:----|----:----|----:----|----:----|----:----|----:----|----:----|----:----|----:----|----:----|----:----|----:----|----:----|----:----|----:----|----:----|----:----|----:----|
4801 TCTTTTAAGAGGGTCTTCCGCAAAGAAACTGTTGTTAAAACCTGTGAAGCAAAAAGAAATAATGTGCCAGCTATGTGTATTAATATGTAGAAATTGTGTCCCTACTTCATAGTAGCTAAGCAGTGTTTGGGAAAGGAGTTGTGTACTTTTTTTGGGAAAGAAGACGTTCAGATATAATTTTTGTCCTGTGAAGGATATAT 5000
       L F N E W F A N R Q C C N Q V S R K E K N C P R Y V Y N Y M K L V P I F Y * R N T V F G K R L V H F F G K R R C T * I L F L V S G I Y  F6
      * F I R G S P T E K V V I K S V E N K K I V R D I C M I I C R * C L S S T D D I R * L G K G * C M F F V R E E A L R Y * F C S V E * I  F5
       S F E G L L R K K S L L K P C K T K R * * V T S V C L * V D K V C P H L I M S E D C V R E E V C S F F G K K Q L D I N F V P C K R Y   F4

      * L Y I S F V H N R G F N F * R S A F G R * N F * D K F F D L V M F Y T F I L R * Q I K F * T K N R I N W A V C S A C R K E K R K L L  F1
```

```
  S Y I S L L Y T I V V S T F S D L H S V G E T F R I N F S I W L C F I H S S * D N K L S F K P K I V S I G P C V Q P V G R K K E S Y   F2
 I A I Y L F C T Q S W F Q L L A I C I R S V K L L G * I F R F G Y V L Y I H L K I T N * V L N Q K S Y Q L G R V F S L * E G K K K A I   F3
5001 ATAGCTATATATCTCTTTTGTACACAATCGTGGTTTCAACTTTTAGCGATCTGCATTCGGTCGGTGAAACTTTTAGGATAAATTTTTCGATTTGGTTATGTTTTATACATTCATCTTAAGATAACAAATTAAGTTTTAAACCAAAAATCGTATCAATTGGGCCGTGTGTTCAGCCTGTAGGAAGGAAAAAAGAAAGCTAT 5200
     ----:----|----:----|----:----|----:----|----:----|----:----|----:----|----:----|----:----|----:----|----:----|----:----|----:----|----:----|----:----|----:----|----:----|----:----|----:----|----:----|
5001 TATCGATATATAGAGAAAACATGTGTTAGCACCAAAGTTGAAAATCGCTAGACGTAAGCCAGCCACTTTGAAAATCCTATTTAAAAAGCTAAACCAATACAAAATATGTAAGTAGAATTCTATTGTTTAATTCAAAATTTGGTTTTTAGCATAGTTAACCCGGCACACAAGTCGGACATCCTTCCTTTTTTCTTTCGATA 5200
  Y S Y I E K T C L R P K L K * R D A N P R H F K * S L N K S K T I N * V N M K L Y C I L N * V L F R I L Q A T H E A Q L F S F L F S   F6
 I A I Y R K Q V C D H N * S K A I Q M R D T F S K P Y I K R N P * T K Y M * R L I V F * T K F W F D Y * N P R T N L R Y S P F F F A I   F5
L L * I D R K Y V I T T E V K L S R C E T P S V K L I F K E I Q N H K I C E D * S L L N L K L G F I T D I P G H T * G T P L F F S L *   F4
```

```
  W C T K R V S A L G V F * * Y L K R K E F V P R K G F V T K L W Y P I P V I F D D C * Y D L R S S D S F F D L S G K I H P * D N C *   F1
Y G V R R E C P P L G F F N S T * R G K N L C Q G K G L L P S Y G T P S L * S L M T V N M T C D H Q T P F L T F L E K S T L E I I A R   F2
 M V Y E E S V R P W G F L I V L E E E R I C A K E R V C Y Q A M V P H P C D L * * L L I * P A I I R L L F * P F W K N P P L R * L L G   F3
5201 TATGGTGTACGAAGAGAGTGTCCGCCCTTGGGGTTTTTAATAGTACTTGAAGAGGAAAGAATTTGTGCCAAGGAAAGGGTTTGTTACCAAGCTATGGTACCCCATCCCTGTGATCTTTGATGACTGTTAATATGACCTGCGATCATCAGACTCCTTTTTTGACCTTTCTGGAAAAATCCACCCTTGAGATAATTGCTAG 5400
     ----:----|----:----|----:----|----:----|----:----|----:----|----:----|----:----|----:----|----:----|----:----|----:----|----:----|----:----|----:----|----:----|----:----|----:----|----:----|----:----|
5201 ATACCACATGCTTCTCTCACAGGCGGAACCCCAAAAAATTATCATGAACTTCTCCTTTCTTAAACACGGTTCCTTTCCCAAACAATGGTTCGATACCATGGGGTAGGGACACTAGAAACTACTGACAATTATACTGGACGCTAGTAGTCTGAGGAAAAAACTGGAAAGACCTTTTTAGGTGGGAACTCTATTAACGATC 5400
N H H V F L T D A R P T K * Y Y K F L F S N T G L F P N T V L S H Y G M G T I K S S Q * Y S R R D D S E K K S R E P F I W G Q S L Q *   F6
  I T Y S S L T R G Q P K K I T S S S S L I Q A L S L T Q * W A I T G W G Q S R Q H S N I H G A I M L S R K Q G K Q F F G G K L Y N S   F5
 * P T R L S H G G K P N K L L V Q L P F F K H W P F P K N G L * P V G D R H D K I V T L I V Q S * * V G K K V K R S F D V R S I I A L   F4
```

```
E A Y F Y F D R G Y * L Q P S I W H E Y H N G Y I Q L F L D I S K T W A L * M I S H P S C Y V F R C T F R H N F H T L * Q Y V D C R A   F1
 K H I S T L T G D T D F N P Q F G M S I T M V T F S Y F L T F L K R G P C K * Y P I H P A T S F G V L L D I I F T L F D N M L T V G Q   F2
  S I F L L * Q G I L T S T L N L A * V S Q W L H S A I S * H F * N V G P V N D I P S I L L R L S V Y F * T * F S H S L T I C * L * G   F3
5401 GAAGCATATTTCTACTTTGACAGGGGATACTGACTTCAACCCTCAATTTGGCATGAGTATCACAATGGTTACATTCAGCTATTTCTTGACATTTCTAAAACGTGGGCCCTGTAAATGATATCCCATCCATCCTGCTACGTCTTTCGGTGTACTTTTAGACATAATTTTCACACTCTTTGACAATATGTTGACTGTAGGGC 5600
     ----:----|----:----|----:----|----:----|----:----|----:----|----:----|----:----|----:----|----:----|----:----|----:----|----:----|----:----|----:----|----:----|----:----|----:----|----:----|----:----|
5401 CTTCGTATAAAGATGAAACTGTCCCCTATGACTGAAGTTGGGAGTTAAACCGTACTCATAGTGTTACCAATGTAAGTCGATAAAGAACTGTAAAGATTTTGCACCCGGGACATTTACTATAGGGTAGGTAGGACGATGCAGAAAGCCACATGAAAATCTGTATTAAAAGTGTGAGAAACTGTTATACAACTGACATCCCG 5600
  S A Y K * K S L P Y Q S * G E I Q C S Y * L P * M * S N R S M E L V H A R Y I I D W G D Q * T K R H V K L C L K * V R Q C Y T S Q L A   F6
P L M N R S Q C P I S V E V R L K A H T D C H N C E A I E Q C K * F T P G T F S I G D M R S R R E T Y K * V Y N E C E K V I H Q S Y P   F5
  F C I E V K V P S V S K L G * N P M L I V I T V N L * K K V N R F R P G Q L H Y G M W G A V D K P T S K S M I K V S K S L I N V T P   F4
```

```
 G K I D P V V S N F V I S V I L T S W L L T K * S I * N K S F E S S H E D S S D R L A P P K Y K Q D T T L S S F V S S F C N F * N V G   F1
  G K L T Q * F Q I L * Y Q * Y * H H G C L L N N Q S E I N P S S P P T K I R L I V W P H Q N I S K T L R S H H L Y H H F A I S K T W   F2
R E N * P S S F K F C D I S N I N I M A A Y * I I N L K * I L R V L P R R F V * S S G P T K I * A R H Y A L I I C I I I L Q F L K R G   F3
5601 AGGGAAAATTGACCCAGTAGTTTCAAATTTTGTGATATCAGTAATATTAACATCATGGCTGCTTACTAAATAATCAATCTGAAATAAATCCTTCGAGTCCTCCCACGAAGATTCGTCTGATCGTCTGGCCCCACCAAAATATAAGCAAGACACTACGCTCTCATCATTTGTATCATCATTTTGCAATTTCTAAAACGTGG 5800
     ----:----|----:----|----:----|----:----|----:----|----:----|----:----|----:----|----:----|----:----|----:----|----:----|----:----|----:----|----:----|----:----|----:----|----:----|----:----|----:----|
5601 TCCCTTTTAACTGGGTCATCAAAGTTTAAAACACTATAGTCATTATAATTGTAGTACCGACGAATGATTTATTAGTTAGACTTTATTTAGGAAGCTCAGGAGGGTGCTTCTAAGCAGACTAGCAGACCGGGGTGGTTTTATATTCGTTCTGTGATGCGAGAGTAGTAAACATAGTAGTAAAACGTTAAAGATTTTGCACC 5800
  P F I S G T T E F K T I D T I N V D H S S V L Y D I Q F L D K S D E W S S E D S R R A G G F Y L C S V V S E D N T D D N Q L K * F T   F6
 L S F Q G L L K L N Q S I L L I L M M A A * * I I L R F Y I R R T R G R L N T Q D D P G V L I Y A L C * A R M M Q I M M K C N R F R P   F5
C P F N V W Y N * I K H Y * Y Y * C * P Q K S F L * D S I F G E L G G V F I R R I T Q G W W F I L L V S R E * * K Y * * K A I E L V H   F4
```

```
  P V A S Y P T H S A T S F G T F L D I I I F I L F G R I T L F R L R I T D E C L V P E T C M S G P C C K F I R F * I M L L N L K T F   F1
V L * H H I P P I L L R L L V H F W T * * F S Y F L A V L H C L D L G S L T S V * Y P K R A C L V H A V N L S G F R L C Y * I * K R L   F2
 S C S I I S H P F C Y V F W Y I F G H N N F H T F W P Y Y I V * T * D H * R V F S T R N V H V W S M L * I Y Q V L D Y A I E F K N V *   F3
5801 GTCCTGTAGCATCATATCCCACCCATTCTGCTACGTCTTTTGGTACATTTTTGGACATAATAATTTCATACTTTTTGGCCGTATTACATTGTTTAGACTTAGGATCACTGACGAGTGTTTAGTACCCGAAACGTGCATGTCTGGTCCATGCTGTAAATTTATCAGGTTTTAGATTATGCTATTGAATTTAAAAACGTTT 6000
     ----:----|----:----|----:----|----:----|----:----|----:----|----:----|----:----|----:----|----:----|----:----|----:----|----:----|----:----|----:----|----:----|----:----|----:----|----:----|----:----|
5801 CAGGACATCGTAGTATAGGGTGGGTAAGACGATGCAGAAAACCATGTAAAAACCTGTATTATTAAAAGTATGAAAAACCGGCATAATGTAACAAATCTGAATCCTAGTGACTGCTCACAAATCATGGGCTTTGCACGTACAGACCAGGTACGACATTTAAATAGTCCAAAATCTAATACGATAACTTAAATTTTTGCAAA 6000
P G T A D Y G V W E A V D K P V N K S M I I K M S K P R I V N N L S L I V S S H K T G S V H M D P G H Q L N I L N * I I S N F K F V N   F6
  D Q L M M D W G N Q * T K Q Y M K P C L L K * V K Q G Y * M T * V * S * Q R T N L V R F T C T Q D M S Y I * * T K S * A I S N L F T   F5
 T R Y C * I G G M R S R R K T C K Q V Y Y N E Y K K A T N C Q K S K P D S V L T * Y G F R A H R T W A T F K D P K L N H * Q I * F R K   F4
```

```
N N C L D * G S L T R I H S D S T C T I Y L F I I I C * * R P A H V H L R I S G R S I A S T V N V C * * N M H   F1
 T I V * I E D H * R G F I L I A R V R F I C L L S Y A N N D L R M C T Y A F P V G A * R V L * T F V S E T C X   F2
  Q L F R L R I T D E D S F * * H V Y D L F V Y Y H M L I T T C A C A L T H F R * E H S E Y C K R L L V K H A X   F3
6001 AACAATTGTTTAGATTGAGGATCACTGACGAGGATTCATTCTGATAGCACGTGTACGATTTATTTGTTTATTATCATATGCTAATAACGACCTGCGCATGTGCACTTACGCATTTCCGGTAGGAGCATAGCGAGTACTGTAAACGTTTGTTAGTGAAACATGCAT 6165
     ----:----|----:----|----:----|----:----|----:----|----:----|----:----|----:----|----:----|----:----|----:----|----:----|----:----|----:----|----:----|----:----|----:
6001 TTGTTAACAAATCTAACTCCTAGTGACTGCTCCTAAGTAAGACTATCGTGCACATGCTAAATAAACAAATAATAGTATACGATTATTGCTGGACGCGTACACGTGAATGCGTAAAGGCCATCCTCGTATCGCTCATGACATTTGCAAACAATCACTTTGTACGTA 6165
 L L Q K S Q P D S V L I * E S L V H V I * K N I I M H * Y R G A C T C K R M E P L L M A L V T F T Q * H F M C   F6
* C N N L N L I V S S S E N Q Y C T Y S K N T * * * I S I V V Q A H A S V C K R Y S C L S Y Q L R K N T F C A   F5
  V I T * I S S * Q R P N M R I A R T R N I Q K N D Y A L L S R R M H V * A N G T P A Y R T S Y V N T L S V H M   F4
```

Notospermus geniculatus (Nge)

WGS: NMRB01003820.1

42752-48584

Notospermus geniculatus strain Ushimado scaffold3820, whole genome shotgun sequence

```
     P Q P S H R N V S C S S C V * * A T H A H I V * D * F F R P P P R S L G M L A C T V R S L R W * Q I N K H H K H G G G E R I E S T * S   F1
      P N P H I E M F P V A H V C N K L R M R T S F K I S F S G R R H V L S V C S H A Q Y D R C A G N R * T N I I N M A E V S V L N R H D R   F2
       P T L T S K C F L * L M C V I S Y A C A H R L R L V F P A A A T F S R Y A R M H S T I V A L V T D K Q T S * T W R R * A Y * I D M I   F3
   1 CCCCAACCCTCACATCGAAATGTTTCCTGTAGCTCATGTGTGTAATAAGCTACGCATGCGCACATCGTTTAAGATTAGTTTTTCCGGCCGCCGCCACGTTCTCTCGGTATGCTCGCATGCACAGTACGATCGTTGCGCTGGTAACAGATAAACAAACATCATAAACATGGCGGAGGTGAGCGTATTGAATCGACATGATC 200
     ----:----|----:----|----:----|----:----|----:----|----:----|----:----|----:----|----:----|----:----|----:----|----:----|----:----|----:----|----:----|----:----|----:----|----:----|----:----|----:----|
   1 GGGGTTGGGAGTGTAGCTTTACAAAGGACATCGAGTACACACATTATTCGATGCGTACGCGTGTAGCAAATTCTAATCAAAAAGGCCGGCGGCGGTGCAAGAGAGCCATACGAGCGTACGTGTCATGCTAGCAACGCGACCATTGTCTATTTGTTTGTAGTATTTGTACCGCCTCCACTCGCATAACTTAGCTGTACTAG 200
      G W G E C R F T E Q L E H T Y Y A V C A C M T * S * N K R G G G R E R P I S A H V T R D N R Q Y C I F L C * L C P P P S R I S D V H D   F6
     X G V R V D F H K R Y S M H T I L * A H A C R K L N T K G A A A V N E R Y A R M C L V I T A S T V S L C V D Y V H R L H A Y Q I S M I   F5
       G L G * M S I N G T A * T H L L S R M R V D N L I L K E P R R W T R E T H E C A C Y S R Q A P L L Y V F M M F M A S T L T N F R C S   F4

      R I W R R I L Q * C T F L F C S E G S S K S * K G D K K G N R R R G K Q G S P F F G V W T F I L V D F L Q R S Q A A H C C C H K Q R F   F1
       E S G D E F F N N A R F Y S V L K A V Q N R R K V T K K A I V E E A S K G A L F S G S G H L S W L I F Y N D P R Q H T A A V T N N G   F2
     E N L A T N S S I M H V F I L F * R Q F K I V E R * Q K R Q S S K R Q A R E P F F R G L D I Y L G * F F T T I P G S T L L L S Q T T V   F3
 201 GAGAATCTGGCGACGAATTCTTCAATAATGCACGTTTTTATTCTGTTCTGAAGGCAGTTCAAAATCGTAGAAAGGTGACAAAAAAGGCAATCGTCGAAGAGGCAAGCAAGGGAGCCCTTTTTTCGGGGTCTGGACATTTATCTTGGTTGATTTTTTACAACGATCCCAGGCAGCACACTGCTGCTGTCACAAACAACGGT 400
     ----:----|----:----|----:----|----:----|----:----|----:----|----:----|----:----|----:----|----:----|----:----|----:----|----:----|----:----|----:----|----:----|----:----|----:----|----:----|----:----|
 201 CTCTTAGACCGCTGCTTAAGAAGTTATTACGTGCAAAAATAAGACAAGACTTCCGTCAAGTTTTAGCATCTTTCCACTGTTTTTTCCGTTAGCAGCTTCTCCGTTCGTTCCCTCGGGAAAAAAGCCCCAGACCTGTAAATAGAACCAACTAAAAAATGTTGCTAGGGTCCGTCGTGTGACGACGACAGTGTTTGTTGCCA 400
      L I Q R R I R * Y H V N K N Q E S P L E F D Y F P S L F P L R R L P L C P L G K K P T Q V N I K T S K K C R D W A A C Q Q Q * L C R   F6
       S F R A V F E E I I C T K I R N Q L C N L I T S L H C F L C D D F L C A L S G K K R P R S M * R P Q N K V V I G P L V S S S D C V V T   F5
     R S D P S S N K L L A R K * E T R F A T * F R L F T V F F A I T S S A L L P A R K E P D P C K D Q N I K * L S G L C C V A A T V F L P   F4

       G * R K N V L C C G A W W D A R N E T T D K F I G D C L Q L R I * S E * R * H * D T V C G R V Q V F R C T D S S E A L C A L R M H C   F1
     S G D E K T F S V A V L G G M H A M K P L T N L S G T V Y S Y A F K V N D D D I K I L S V A E C K C S G A Q I A L R P F V H S A C I V   F2
      R V T K K R S L L R C L V G C T Q * N H * Q I Y R G L S T V T H L K * M T M T L R Y C L W Q S A S V Q V H R * L * G P L C T P H A L L   F3
 401 TCGGGTGACGAAAAAACGTTCTCTGTTGCGGTGCTTGGTGGGATGCACGCAATGAAACCACTGACAAATTTATCGGGGACTGTCTACAGTTACGCATTTAAAGTGAATGACGATGACATTAAGATACTGTCTGTGGCAGAGTGCAAGTGTTCAGGTGCACAGATAGCTCTGAGGCCCTTTGTGCACTCCGCATGCATTGT 600
     ----:----|----:----|----:----|----:----|----:----|----:----|----:----|----:----|----:----|----:----|----:----|----:----|----:----|----:----|----:----|----:----|----:----|----:----|----:----|----:----|
 401 AGCCCACTGCTTTTTTGCAAGAGACAACGCCACGAACCACCCTACGTGCGTTACTTTGGTGACTGTTTAAATAGCCCCTGACAGATGTCAATGCGTAAATTTCACTTACTGCTACTGTAATTCTATGACAGACACCGTCTCACGTTCACAAGTCCACGTGTCTATCGAGACTCCGGGAAACACGTGAGGCGTACGTAACA 600
      N P H R F F T R Q Q P A Q H S A R L S V V S L N I P S Q R C N R M * L S H R H C * S V T Q P L T C T N L H V S L E S A R Q A S R M C Q   F6
       R T V F F R E R N R H K T P H V C H F W Q C I * R P S D V T V C K F H I V I V N L Y Q R H C L A L T * T C L Y S Q P G K H V G C A N   F5
     E P S S F V N E T A T S P P I C A I F G S V F K D P V T * L * A N L T F S S S M L I S D T A S H L H E P A C I A R L G K T C E A H M T   F4

     * N E G N T G I F Q I Q A N R A W G S Q E C H D I D L L H L G R P G S Q S Y V T T * * S D N T * K L W K G S I Y R * V S L P G R D * E   F1
      E T K A T R E S S R S R R T G P G G A R S A M I S I C S I W G G L D P N R M L P H N D L T I L E N F G R D Q F T A R L V C Q E G T K N   F2
       K R R Q H G N L P D P G E P G L G E P G V P * Y R F A P F G A A W I P I V C Y H I M I * Q Y L K T L E G I N L P L G * F A R K G L R   F3
 601 TGAAACGAAGGCAACACGGGAATCTTCCAGATCCAGGCGAACCGGGCCTGGGGGAGCCAGGAGTGCCATGATATCGATTTGCTCCATTTGGGGCGGCCTGGATCCCAATCGTATGTTACCACATAATGATCTGACAATACTTGAAAACTTTGGAGGGATCAATTTACCGCTAGGTTAGTTTGCCAGGAAGGGACTAAGA 800
     ----:----|----:----|----:----|----:----|----:----|----:----|----:----|----:----|----:----|----:----|----:----|----:----|----:----|----:----|----:----|----:----|----:----|----:----|----:----|----:----|
 601 ACTTTGCTTCCGTTGTGCCCTTAGAAGGTCTAGGTCCGCTTGGCCCGGACCCCCTCGGTCCTCACGGTACTATAGCTAAACGAGGTAAACCCCGCCGGACCTAGGGTTAGCATACAATGGTGTATTACTAGACTGTTATGAACTTTTGAAACCTCCCTAGTTAAATGGCGATCCAATCAAACGGTCCTTCCCTGATTCT 800
      Q F S P L V P I K W I W A F R A Q P L W S H W S I S K S W K P R G P D W D Y T V V Y H D S L V Q F S Q F P D I * R * T L K G P L S * S   F6
     N F R L C P F R G S G P S G P R P S G P T G H Y R N A G N P A A Q I G I T H * W M I I Q C Y K F V K S P I L K G S P * N A L F P S L   F5
       S V F A V R S D E L D L R V P G P P A L L A M I D I Q E M Q P P R S G L R I N G C L S R V I S S F K P L S * N V A L N T Q W S P V L   F4

       C T N W D C P * S T L W A Y I K W S P L P R Q S C G S S T V W G S H C E K H Y P G Q * T F H P G W Y T L L P * P R K L S V E K T V * T   F1
     V Q T G T V P E A R Y G H T L S G L H Y Q G S L V G A V L Y G G V T V K N T T Q G N E H S T Q D G T L Y F L D L E N Y Q W K K L S E   F2
      M Y K L G L S L K H V M G I H * V V S I T K A V L W E Q Y C M G E S L * K T L P R A M N I P P R M V H S T S L T * K I I S G K N C L N   F3
 801 ATGTACAAACTGGGACTGTCCCTGAAGCACGTTATGGGCATACATTAAGTGGTCTCCATTACCAAGGCAGTCTTGTGGGAGCAGTACTGTATGGGGGAGTCACTGTGAAAAACACTACCCAGGGCAATGAACATTCCACCCAGGATGGTACACTCTACTTCCTTGACCTAGAAAATTATCAGTGGAAAAAACTGTCTGAA 1000
     ----:----|----:----|----:----|----:----|----:----|----:----|----:----|----:----|----:----|----:----|----:----|----:----|----:----|----:----|----:----|----:----|----:----|----:----|----:----|----:----|
 801 TACATGTTTGACCCTGACAGGGACTTCGTGCAATACCCGTATGTAATTCACCAGAGGTAATGGTTCCGTCAGAACACCCTCGTCATGACATACCCCCTCAGTGACACTTTTTGTGATGGGTCCCGTTACTTGTAAGGTGGGTCCTACCATGTGAGATGAAGGAACTGGATCTTTTAATAGTCACCTTTTTTGACAGACTT 1000
       H V F Q S Q G Q L V N H A Y M L H D G N G L C D Q P L L V T H P L * Q S F C * G P C H V N W G P H Y V R S G Q G L F N D T S F V T Q   F6
      I Y L S P S D R F C T I P M C * T T E M V L A T K H S C Y Q I P S D S H F V S G L A I F M G G L I T C E V E K V * F I I L P F F Q R F   F5
     F T C V P V T G S A R * P C V N L P R W * W P L R T P A T S Y P P T V T F F V V W P L S C E V W S P V S * K R S R S F * * H F F S D S   F4

       C S S C L P H K L * N Q G P D S R F H R G T V S A V * Q * G D F Y T A Y V H P E D N H C G T E F N N * P S Y C A K S Q S V L P * P C   F1
     L A P V A Y H T S C E I R D L T L V F I G G L S Q L C D N R V T S T Q R M S I Q K I T I V E L N S T I D Q V T V Q N L S L S F L D P A   F2
      L L Q L P T T Q A V K S G T * L S F S * G D C L S C V T I G * L L H S V C P S R R * P L W N * I Q Q L T K L L C K I S V C P S L T L H   F3
1001 CTTGCTCCAGTTGCCTACCACACAAGCTGTGAAATCAGGGACCTGACTCTCGTTTTCATAGGGGGACTGTCTCAGCTGTGTGACAATAGGGTGACTTCTACACAGCGTATGTCCATCCAGAAGATAACCATTGTGGAACTGAATTCAACAATTGACCAAGTTACTGTGCAAAATCTCAGTCTGTCTTCCTTGACCCTGC 1200
     ----:----|----:----|----:----|----:----|----:----|----:----|----:----|----:----|----:----|----:----|----:----|----:----|----:----|----:----|----:----|----:----|----:----|----:----|----:----|----:----|
1001 GAACGAGGTCAACGGATGGTGTGTTCGACACTTTAGTCCCTGGACTGAGAGCAAAAGTATCCCCCTGACAGAGTCGACACACTGTTATCCCACTGAAGATGTGTCGCATACAGGTAGGTCTTCTATTGGTAACACCTTGACTTAAGTTGTTAACTGGTTCAATGACACGTTTTAGAGTCAGACAGAAGGAACTGGGACG 1200
     V Q E L Q R G C L S H F * P G S E R K * L P V T E A T H C Y P S K * V A Y T W G D S S L W Q P V S N L L Q G L * Q A F D * D T R G Q G Q   F6
      K S W N G V V C A T F D P V Q S E N E Y P S Q R L Q T V I P H S R C L T H G D L L Y G N H F Q I * C N V L N S H L I E T Q G E K V R   F5
       S A G T A * W V L Q S I L S R V R T K M P P S D * S H S L L T V E V C R I D M W F I V M T S S F E V I S W T V T C F R L R D K R S G A   F4

       T G * C L S L R A Q C K H L F S K S A T D F R G V P T A Y F R Y E H Q D S K Q N L L S C G H Y * P Q H S S Q G S S C R F R Y G W S H F   F1
     L D N V F L S G H S A N I C S P N Q L L I F G G G Y Q Q H I S D M N T K I P S R T Y Y L V D I T S L S I Q A K E A P V G F D M A G H T S   F2
      W I M S F S Q G T V Q T S V L Q I S Y * F S G G T N S I F Q I * T P R F Q A E L T I L W T L L A S A F K P R K L L S V S I W L V T L   F3
1201 ACTGGATAATGTCTTTCTCTCAGGGCACAGTGCAAACATCTGTTCTCCAAATCAGCTACTGATTTTCGGGGGGTACCAACAGCATATTTCAGATATGAACACCAAGATTCCAAGCAGAACTTACTATCTTGTGGACATTACTAGCCTCAGCATTCAAGCCAAGGAAGCTCCTGTCGGTTTCGATATGGCTGGTCACACTT 1400
     ----:----|----:----|----:----|----:----|----:----|----:----|----:----|----:----|----:----|----:----|----:----|----:----|----:----|----:----|----:----|----:----|----:----|----:----|----:----|----:----|
1201 TGACCTATTACAGAAAGAGAGTCCCGTGTCACGTTTGTAGACAAGAGGTTTAGTCGATGACTAAAAGCCCCCCATGGTTGTCGTATAAAGTCTATACTTGTGGTTCTAAGGTTCGTCTTGAATGATAGAACACCTGTAATGATCGGAGTCGTAAGTTCGGTTCCTTCGAGGACAGCCAAAGCTATACCGACCAGTGTGAA 1400
       V P Y H R E R L A C H L C R N E L D A V S K R P T G V A Y K L Y S C W S E L C F K S D P Q C * * G * C E L W P L E Q R N R Y P Q D C K   F6
      C Q I I D K E * P V T C V D T R W I L * Q N E P P V L L M N * I H V G L N W A S S V I K H V N S A E A N L G L F S R D T E I H S T V S   F5
     S S L T K R E P C L A F M Q E G F * S S I K P P Y W C C I E S I F V L I G L L V * * R T S M V L R L M * A L S A G T P K S I A P * V   F4

       C E I R P L L P V L P W W S Q * T F V H N D N Q T D G A W C L * S * K M H R K * S F L P G R N C H K T S V Y L M * C I F P L M L * P G   F1
     V R L D H C S L F F H G G A N E H L F T M T T K R M E L W C V E A E K C I V N D H F S P G E I V T K L Q C T L C D A F F H S C C D Q   F2
     L * D * T T A P C S S M V E P M N I C S Q * Q P N G W S L V F V K L K N A S * M I I S P R E K L S Q N F S V P Y V M H F S T H A V T R   F3
1401 CTGTGAGATTAGACCACTGCTCCCTGTTCTTCCATGGTGGAGCCAATGAACATTTGTTCACAATGACAACCAAACGGATGGAGCTTGGTGTTTGTGAAGCTGAAAAATGCATCGTAAATGATCATTTCTCCCCGGGAGAAATTGTCACAAAACTTCAGTGTACCTTATGTGATGCATTTTTCCACTCATGCTGTGACCAG 1600
     ----:----|----:----|----:----|----:----|----:----|----:----|----:----|----:----|----:----|----:----|----:----|----:----|----:----|----:----|----:----|----:----|----:----|----:----|----:----|----:----|
1401 GACACTCTAATCTGGTGACGAGGGACAAGAAGGTACCACCTCGGTTACTTGTAAACAAGTGTTACTGTTGGTTTGCCTACCTCGAACCACAAACACTTCGACTTTTTACGTAGCATTTACTAGTAAAGAGGGGGCCCTCTTTAACAGTGTTTTGAAGTCACATGGAATACACTACGTAAAAAGGTGAGTACGACACTGGTC 1600
       Q S I L G S S G T R G H H L W H V N T * L S L W V S P A Q H K H L Q F I C R L H D N R G P L F Q * L V E T Y R I H H M K G S M S H G   F6
      R H S * V V A G Q E E M T S G I F M Q E C H C G F P H L K T N T F S F F A D Y I I M E G R S P N D C F K L T G * T I C K E V * A T V L   F5
     E T L N S W Q E R N K W P P A L S C K N V I V V L R I S S P T Q S A S F H M T F S * K E G P S I T V F S * H V K H S A N K W E H Q S W   F4

       R I S H E S * P G G H Q I Y M S * V Q T K R * K T K K E Q S Q K L S T T I M * F V Q C I E N W T S N L S Y K N * L H C S T V H P R V   F1
     G E S H T S H S Q E D I R F T C P K C R P K G K R Q R K N K A R N * V Q Q S C N L C N A L K I G L P I S L I R T S C I A A L Y I P G W   F2
      E N L T R V I A R R T S D L H V L S A D Q K V K D K E R T K P E I E Y N N H V I C A M H * K L D F Q S L L * E L A A L Q H C T S Q G G   F3
1601 GGAGAATCTCACACGAGTCATAGCCAGGAGGACATCAGATTTACATGTCCTAAGTGCAGACCAAAAGGTAAAAGACAAAGAAAGAACAAAGCCAGAAATTGAGTACAACAATCATGTAATTTGTGCAATGCATTGAAAATTGGACTTCCAATCTCTCTTATAAGAACTAGCTGCATTGCAGCACTGTACATCCCAGGGTG 1800
     ----:----|----:----|----:----|----:----|----:----|----:----|----:----|----:----|----:----|----:----|----:----|----:----|----:----|----:----|----:----|----:----|----:----|----:----|----:----|----:----|
1601 CCTCTTAGAGTGTGCTCAGTATCGGTCCTCCTGTAGTCTAAATGTACAGGATTCACGTCTGGTTTTCCATTTTCTGTTTCTTTCTTGTTTCGGTCTTTAACTCATGTTGTTAGTACATTAAACACGTTACGTAACTTTTAACCTGAAGGTTAGAGAGAATATTCTTGATCGACGTAACGTCGTGACATGTAGGGTCCCAC 1800
       P L I E C S D Y G P P C * I * M D * T C V L L Y F V F F S C L W F N L V V I M Y N T C H M S F Q V E L R E * L F * S C Q L V T C G L T   F6
      S F R V R T M A L L V D S K C T R L A S W F T F S L S L V F G S I S Y L L * T I Q A I C Q F N S K W D R K Y S S A N C C Q V D W P   F5
     P S D * V L * L W S S M L N V H G L H L G F P L L C L F F L A L F Q T C C D H L K H L A N F I P S G I E R I L V L Q M A A S Y M G P H   F4

     G Q * K C P V N Y H I V C L C F P I V H W T F Y * S I L V A L V H S P S L K F R G H P F S I C V H H Q T I K F N L I V G D F Q T P P I T T   F1
      A N K N V Q * T T I V Y V F L L F I G H F I D Q F W L L W Y I H R A * N L G A I H L V Y V C T T K Q S N S I * L L V I F R P H P S L Q   F2
       P I K M S S E L P L F M F S Y C S L D I L L I N S G C F G T F T E P K I * G P S I * Y M C A P P N N Q I Q F D C W * F S D P T H H Y   F3
1801 GGCCAATAAAAATGTCCAGTGAACTACCATTGTTTATGTTTTCCTATTGTTCATTGGACATTTTATTGATCAATTCTGGTTGCTTTGGTACATTCACCGAGCCTAAAATTTAGGGGCCATCCATTTAGTATATGTGTGCACCACCAAACAATCAAATTCAATTTGATTGTTGGTGATTTTCAGACCCCACCCATCACTAC 2000
     ----:----|----:----|----:----|----:----|----:----|----:----|----:----|----:----|----:----|----:----|----:----|----:----|----:----|----:----|----:----|----:----|----:----|----:----|----:----|----:----|
1801 CCGGTTATTTTTACAGGTCACTTGATGGTAACAAATACAAAAGGATAACAAGTAACCTGTAAAATAACTAGTTAAGACCAACGAAACCATGTAAGTGGCTCGGATTTTAAATCCCCGGTAGGTAAATCATATACACACGTGGTGGTTTGTTAGTTTAAGTTAAACTAACAACCACTAAAAGTCTGGGGTGGGTAGTGATG 2000
       P W Y F H G T F * W Q K H K G I T * Q V N * Q D I R T A K T C E G L R F N L P W G N L I H T C W W V I L N L K I T P S K * V G G M V   F6
      P G I F I D L S S G N N I N E * Q E N S M K N I L E P Q K P V N V S G L I * P G D M * Y I H A G G F L * I * N S Q Q H N E S G V W *   F5
     A L L F T W H V V M T * T K R N N M P C K I S * N Q N S Q Y M * R A * F K P A M W K T Y T H V V L C D F E I Q N N T I K L G W G D S   F4

      E S K Q N V H N N * N Q * Q S I H T Y M Y Y M D S A * Q I N N * Q * S N V S I K Q I M W * K L C G G K N L K K * M N K S I Q E N H I S   F1
       K V N K M Y I I I K I N N S Q F T H T C T T W I A L N K L I I N S K V M * V * N K * C G K N Y V V V K T * R N K * I N Q S K K I T F   F2
     R K * T K C T * * L K S I T V N S H I H V L H G * R L T N * * L T V K * C K Y K T N N V V K T M * K L E E I N E * I N P R K S H F   F3
2001 AGAAAGTAAACAAAATGTACATAATAATTAAAATCAATAACAGTCAATTCACACATACATGTACTACATGGATAGCGCTTAACAAATTAATAATTAACAGTAAAGTAATGTAAGTAATAAAACAAATAATGTGGTAAAAACTATGTGGTGGTAAAAACTTGAAGAAATAAATGAATAAATCAATCCAAGAAAATCACATT 2200
     ----:----|----:----|----:----|----:----|----:----|----:----|----:----|----:----|----:----|----:----|----:----|----:----|----:----|----:----|----:----|----:----|----:----|----:----|----:----|----:----|
2001 TCTTTCATTTGTTTTACATGTATTATTAATTTTAGTTATTGTCAGTTAAGTGTGTATGTACATGATGTACCTATCGCGAATTGTTTAATTATTAATTGTCATTTCATTACATTCATTATTTTGTTTATTACACCATTTTTGATACACCACCATTTTTGAACTTCTTTATTTACTTATTTAGTTAGGTTCTTTTAGTGTAA 2200
       S L L C F T C L L * F * Y C D I * V Y M Y * M S L A * C I L L * C Y L L T L I F C I I H Y F S H P P L F K F F Y I F L F D L I W S F * M   F6
      L F Y V F H V Y Y N F D I V T L E C M C T S C P Y R K V F * Y N V T F Y H L Y L V F L T T F V I H H Y F S S S I F S Y I L G L F D C K   F5
     C F T F L I Y M I I L I L L L * N V C V H V H I A S L L N I I L L L T I Y T Y F L Y H P L F * T T T F V Q L F L H I F * D L F I V N   F4

       F E C F S A V W T N V * C D P R E * V S I I A L I N S E I Q L K Y T * L K Y T Y S H * * P G L I F N I L T F M W T T * N I E N V K *   F1
     H L N V F Q Q C G L M C N V I Q G N E F Q L L H * S T L K Y S * N T P S * N T P T L T D N Q A * F S I F * H L C G P H R I * K M * S S   F2
      I * M F F S S V D * C V M * S K G M S F N Y C I D Q L * N T A E I H L A E I H L L S L I T R P N F Q Y F D I Y V D H I E Y R K C E V V   F3
2201 CATTTGAATGTTTTTCAGCAGTGTGGACTAATGTGTAATGTGATCCAAGGGAATGAGTTTCAATTATTGCATTGATCAACTCTGAAATACAGCTGAAATACACCTAGCTGAAATACACCTACTCTCACTGATAACCAGGCCTAATTTTCAATATTTTGACATTTATGTGGACCACATAGAATATAGAAAATGTGAAGTAG 2400
     ----:----|----:----|----:----|----:----|----:----|----:----|----:----|----:----|----:----|----:----|----:----|----:----|----:----|----:----|----:----|----:----|----:----|----:----|----:----|----:----|
2201 GTAAACTTACAAAAAGTCGTCACACCTGATTACACATTACACTAGGTTCCCTTACTCAAAGTTAATAACGTAACTAGTTGAGACTTTATGTCGACTTTATGTGGATCGACTTTATGTGGATGAGAGTGACTATTGGTCCGGATTAAAAGTTATAAAACTGTAAATACACCTGGTGTATCTTATATCTTTTACACTTCATC 2400
      E N S H K E A T H V L T Y H S G L S H T E I I A N I L E S I C S F Y V * S F Y V * E * Q Y G P R I K L I K V N I H V V Y F I S F T F Y   F6
      M Q I N K L L T S * H T I H D L P I L K L * Q M S * S Q F V A S I C R A S I C R S E S I V L G L K * Y K S M * T S W M S Y L F H S T   F5
      * K F T K * C H P S I H L T I W P F S N * N N C Q D V R F Y L Q F V G L Q F V G V R V S L W A * N E I N Q C K H P G C L I Y F I H L L   F4
```

```
     W Y M * F P Y S D K S E S P Y M S I S K S S L V L V L L F M S M P W S T V V V S P F S S Q V R L L * P T C P H F L Q L * G A L Q E V A   F1
     G T C N S L I Q T N Q N H H T C P S P N H H * Y W C Y C S C P C P G Q Q * * C P H F H H K C D C C D Q P V H I S Y S Y K G P Y K K L L   F2
     V H V I P L F R Q I R I T I H V H L Q I I T S T G V T V H V H A L V N S S S V P I F I T S A T V V T N L S T F P T V I R G L T R S C   F3
2401 TGGTACATGTAATTCCCTTATTCAGACAAATCAGAATCACCATACATGTCCATCTCCAAATCATCACTAGTACTGGTGTTACTGTTCATGTCCATGCCCTGGTCAACAGTAGTAGTGTCCCCATTTTCATCACAAGTGCGACTGTTGTGACCAACCTGTCCACATTTCCTACAGTTATAAGGGGCCTTACAAGAAGTTGC 2600
     ----:----|----:----|----:----|----:----|----:----|----:----|----:----|----:----|----:----|----:----|----:----|----:----|----:----|----:----|----:----|----:----|----:----|----:----|----:----|----:----|
2401 ACCATGTACATTAAGGGAATAAGTCTGTTTAGTCTTAGTGGTATGTACAGGTAGAGGTTTAGTAGTGATCATGACCACAATGACAAGTACAGGTACGGGACCAGTTGTCATCATCACAGGGGTAAAAGTAGTGTTCACGCTGACAACACTGGTTGGACAGGTGTAAAGGATGTCAATATTCCCCGGAATGTTCTTCAACG 2600
     H Y M Y N G * E S L D S D G Y M D M E L D D S T S T N S N M D M G Q D V T T T D G N E D C T R S N H G V Q G C K R C N Y P A K C S T A   F6
     T T C T I G K N L C I L I V M C T W R W I M V L V P T V T * T W A R T L L L L T G M K M V L A V T T V L R D V N G V T I L P R V L L Q   F5
     P V H L E R I * V F * F * W V H G D G F * * * Y Q H * Q E H G H G P * C Y Y H G W K * * L H S Q Q S W G T W M E * L * L P G * L F N   F4

     S F * M H L L V * I Q W K T R A S L K S Q S P A V S C L L R D L R M N L Y N L F P H S P P S L E S N P T L W R H L G S W T I S S T C S   F1
     V S E C I Y S C K S N G K P G H L S N H S H Q L C P A Y * G T F E * T Y T I C F H T R H P H L K A T P H Y G D I L G P G L F H P H V   F2
     * F L N A F T R V N P M E N Q G I S Q I T V T S C V L P T E G P S N E P I Q F V S T L A T L T * K Q P H T M E T S W V L D Y F I H M F   F3
2601 TAGTTTCTGAATGCATTTACTCGTGTAAATCCAATGGAAAACCAGGGCATCTCTCAAATCACAGTCACCAGCTGTGTCCTGCCTACTGAGGGACCTTCGAATGAACCTATACAATTTGTTTCCACACTCGCCACCCTCACTTGAAAGCAACCCCACACTATGGAGACATCTTGGGTCCTGGACTATTTCATCCACATGTT 2800
     ----:----|----:----|----:----|----:----|----:----|----:----|----:----|----:----|----:----|----:----|----:----|----:----|----:----|----:----|----:----|----:----|----:----|----:----|----:----|----:----|
2601 ATCAAAGACTTACGTAAATGAGCACATTTAGGTTACCTTTTGGTCCCGTAGAGAGTTTAGTGTCAGTGGTCGACACAGGACGGATGACTCCCTGGAAGCTTACTTGGATATGTTAAACAAAGGTGTGAGCGGTGGGAGTGAACTTTCGTTGGGGTGTGATACCTCTGTAGAACCCAGGACCTGATAAAGTAGGTGTACAA 2800
     L K Q I C K S T Y I W H F V L A D R L D C D G A T D Q R S L S R R I F R Y L K N G C E G G E S S L L G V S H L C R P D Q V I E D V H   F6
     * N R F A N V R T F G I S F W P M E * I V T V L Q T R G V S P G E F S G I C N T E V S A V R V Q F C G W V I S V D Q T R S * K M W M N   F5
     S T E S H M * E H L D L P F G P C R E F * L * W S H G A * Q P V K S H V * V I Q K W V R W G * K F A V G C * P S M K P G P S N * G C T   F4

     I N L * R * F D H G T * L K C S P K * N A N C S A F S L * Q A V S S G T E L V G S L L E Y T A F N L L N K P K M H L K C C F L S G F   F1
     Q S T C E D N L T M E H N * N A P P N E M Q I A V H S P C N R Q F L L A L S * W D L C * N T L P S T C * I N L K C I * N A A F Y Q D F   F2
     N Q L V K I I * P W N I I E M L P Q M K C K L Q C I L L V T G S F F W H * A S G I F A R I H C L Q L A E * T * N A F E M L L S I R I F   F3
2801 CAATCAACTTGTGAAGATAATTTGACCATGGAACATAATTGAAATGCTCCCCCAAATGAAATGCAAATTGCAGTGCATTCTCCTTGTAACAGGCAGTTTCTTCTGGCACTGAGCTAGTGGGATCTTTGCTAGAATACACTGCCTTCAACTTGCTGAATAAACCTAAAATGCATTTGAAATGCTGCTTTCTATCAGGATTT 3000
     ----:----|----:----|----:----|----:----|----:----|----:----|----:----|----:----|----:----|----:----|----:----|----:----|----:----|----:----|----:----|----:----|----:----|----:----|----:----|----:----|
2801 GTTAGTTGAACACTTCTATTAAACTGGTACCTTGTATTAACTTTACGAGGGGGTTTACTTTACGTTTAACGTCACGTAAGAGGAACATTGTCCGTCAAAGAAGACCGTGACTCGATCACCCTAGAAACGATCTTATGTGACGGAAGTTGAACGACTTATTTGGATTTTACGTAAACTTTACGACGAAAGATAGTCCTAAA 3000
     E I L K H L Y N S W P V Y N F H E G L H F A F Q L A N E K Y C A T E E P V S S T P D K S S Y V A K L K S F L G L I C K F H Q K R D P N   F6
     L * S T F I I Q G H F M I S I S G W I F H L N C H M R R T V P L K K Q C Q A L P I K A L I C Q R * S A S Y V * F A N S I S S E I L I   F5
     * D V Q S S L K V M S C L Q F A G G F S I C I A T C E G Q L L C N R R A S L * H S R Q * F V S G E V Q Q I F R F H M Q F A A K * * S K   F4

     L I N S L T V S A F L S K N R M R A * L P G M I I L D C K P K V F I K C * S K F F S A C C R P F L F L S F S A H S * I S A I S L T S I   F1
     * S I R S L S Q H S Y L K T E C G H N C L A * S S W T A S P K F S S N A D L S F F Q H V V G L F C F Y H S Q P T H R S L L F P * Q V S   F2
     D Q F A H C L S I L I * K Q N A G I I A W H D H L G L Q A Q S F H Q M L I * V F F S M L * A F F V S I I L S P L I D L C Y F P D K Y   F3
3001 TTGATCAATTCGCTCACTGTCTCAGCATTCTTATCTAAAAACAGAATGCGGGCATAATTGCCTGGCATGATCATCTTGGACTGCAAGCCCAAAGTTTTCATCAAATGCTGATCTAAGTTTTTTTCAGCATGTTGTAGGCCTTTTTTGTTTCTATCATTCTCAGCCCACTCATAGATCTCTGCTATTTCCCTGACAAGTAT 3200
     ----:----|----:----|----:----|----:----|----:----|----:----|----:----|----:----|----:----|----:----|----:----|----:----|----:----|----:----|----:----|----:----|----:----|----:----|----:----|----:----|
3001 AACTAGTTAAGCGAGTGACAGAGTCGTAAGAATAGATTTTTGTCTTACGCCCGTATTAACGGACCGTACTAGTAGAACCTGACGTTCGGGTTTCAAAAGTAGTTTACGACTAGATTCAAAAAAAGTCGTACAACATCCGGAAAAAAACAAAGATAGTAAGAGTCGGGTGAGTATCTAGAGACGATAAAGGGACTGTTCATA 3200
     K I L E S V T E A N K D L F L I R A Y N G P M I M K S Q L G L T K M L H Q D L N K E A H Q L G K K N R D N E A W E Y I E A I E R V L I   F6
     K S * N A * Q R L M R I * F C F A P M I A Q C S * R P S C A W L K * * I S I * T K K L M N Y A K K T E I M R L G S M S R Q * K G S L Y   F5
     Q D I R E S D * C E * R F V S H P C L Q R A H D D Q V A L G F N E D F A S R L K K * C T T P R K Q K * * E * G V * L D R S N G Q C T   F4

     L L K T L P I L I L A * V E S T D L S V A S D L I R G M L L T P L H L S A R S F S F S L S G F S R R Y L Y R E T V S S Y V L H L E K E   F1
     S * K P C Q Y * Y W H E W N Q L I C L W H L I * S E V C Y * P P C I F L P D H F H L V C Q G F Q G D T C T G K L S H H M F Y I W R R   F2
     P L E N P A N I D I G M S G I N * S V C G I * F N Q R Y A I N P L A S F C Q I I F I * F V R V F K A I L V Q G N C L I I C S T S G E G   F3
3201 CCTCTTGAAAACCCTGCCAATATTGATATTGGCATGAGTGGAATCAACTGATCTGTCTGTGGCATCTGATTTAATCAGAGGTATGCTATTAACCCCCTTGCATCTTTCTGCCAGATCATTTTCATTTAGTTTGTCAGGGTTTTCAAGGCGATACTTGTACAGGGAAACTGTCTCATCATATGTTCTACATCTGGAGAAGG 3400
     ----:----|----:----|----:----|----:----|----:----|----:----|----:----|----:----|----:----|----:----|----:----|----:----|----:----|----:----|----:----|----:----|----:----|----:----|----:----|----:----|
3201 GGAGAACTTTTGGGACGGTTATAACTATAACCGTACTCACCTTAGTTGACTAGACAGACACCGTAGACTAAATTAGTCTCCATACGATAATTGGGGGAACGTAGAAAGACGGTCTAGTAAAAGTAAATCAAACAGTCCCAAAAGTTCCGCTATGAACATGTCCCTTTGACAGAGTAGTATACAAGATGTAGACCTCTTCC 3400
     R K F V R G I N I N A H T S D V S R D T A D S K I L P I S N V G K C R E A L D N E N L K D P N E L R Y K Y L S V T E D Y T R C R S F   F6
     G R S F G A L I S I P M L P I L Q D T Q P M Q N L * L Y A I L G R A D K Q W I M K M * N T L T K L A I S T C P F Q R M M H E V D P S P   F5
     D E Q F G Q W Y Q Y Q C S H F * S I Q R H C R I * D S T H * * G G Q M K R G S * K * K T Q * P K * P S V Q V P F S D * * I N * M Q L L   F4

     P T S A * Q V F V A A * Q R V Q M N L D P L P * R P D D V R S N F S S T M V Y R K C R * C C L T S S P V S G S T N T K V P S C I I S   F1
     N Q H Q L D K C L L P H N K E C K * I * I H C P E G L M M Y A Q I F H P P W Y T G N V G D V V S H H H L Y Q D P P T P K C H L A S S L   F2
     T N I S L T S V C C R I T K S A N E S R S T A L K A * * C T L K F F I H H G I Q E M * V M L S H I I T C I R I H Q H Q S A I L H H L S   F3
3401 AACCAACATCAGCTTGACAAGTGTTTGTTGCCGCATAACAAAGAGTGCAAATGAATCTAGATCCACTGCCCTGAAGGCCTGATGATGTACGCTCAAATTTTTCATCCACCATGGTATACAGGAAATGTAGGTGATGTTGTCTCACATCATCACCTGTATCAGGATCCACCAACACCAAAGTGCCATCTTGCATCATCTCT 3600
     ----:----|----:----|----:----|----:----|----:----|----:----|----:----|----:----|----:----|----:----|----:----|----:----|----:----|----:----|----:----|----:----|----:----|----:----|----:----|----:----|
3401 TTGGTTGTAGTCGAACTGTTCACAAACAACGGCGTATTGTTTCTCACGTTTACTTAGATCTAGGTGACGGGACTTCCGGACTACTACATGCGAGTTTAAAAAGTAGGTGGTACCATATGTCCTTTACATCCACTACAACAGAGTGTAGTAGTGGACATAGTCCTAGGTGGTTGTGGTTTCACGGTAGAACGTAGTAGAGA 3600
     S G V D A Q C T N T A A Y C L T C I F R S G S G Q L G S S T R E F K E D V M T Y L F H L H H Q R V D D G T D P D V L V L T G D Q M M E   F6
     V L M L K V L T Q Q R M V F L A F S D L D V A R F A Q H H V S L N K M W W P I C S I Y T I N D * M M V Q I L I W W C W L A M K C * R   F5
     F W C * S S L H K N G C L L S H L H I * I W Q G S P R I I Y A * I K * G G H Y V P F T P S T T E C * * R Y * S G G V G F H W R A D D R   F4

     L S R D T L A S K V E I L G L S L S S Q * Q R I T G L V L L G N V S R K R R C F L S P T T A L H E T Q Q K E Y L K I L S V L A K S A F   F1
     F P E I H W P A K L K Y W A C H S R H S D R E * Q A * C Y * E T F L G K E D A S Y L Q Q Q H Y M K H S K R S I * K S C Q F * Q N L P F   F2
     F P R Y I G Q Q S * N T G P V T L V T V T E N N R P S V I R K R F S E K K M L L I S N N S T T * N T A K G V S K N L V S S S K I C L   F3
3601 CTTTCCCGAGATACATTGGCCAGCAAAGTTGAAATACTGGGCCTGTCACTCTCGTCACAGTGACAGAGAATAACAGGCCTAGTGTTATTAGGAAACGTTTCTCGGAAAAGAAGATGCTTCTTATCTCCAACAACAGCACTACATGAAACACAGCAAAAGGAGTATCTAAAAATCTTGTCAGTTCTAGCAAAATCTGCCTT 3800
     ----:----|----:----|----:----|----:----|----:----|----:----|----:----|----:----|----:----|----:----|----:----|----:----|----:----|----:----|----:----|----:----|----:----|----:----|----:----|----:----|
3601 GAAAGGGCTCTATGTAACCGGTCGTTTCAACTTTATGACCCGGACAGTGAGAGCAGTGTCACTGTCTCTTATTGTCCGGATCACAATAATCCTTTGCAAAGAGCCTTTTCTTCTACGAAGAATAGAGGTTGTTGTCGTGATGTACTTTGTGTCGTTTTCCTCATAGATTTTTAGAACAGTCAAGATCGTTTTAGACGGAA 3800
     R E R S V N A L L T S I S P R D S E D C H C L I V P R T N N P F T E R F L L H K K D G V V A S C S V C C F S Y R F I K D T R A F D A K   F6
     E K G L Y M P W C L Q F V P G T V R T V T V S F L L G L T I L F R K E S F F I S R I E L L L V V H F V A F P T D L F R T L E L L I Q R   F5
     K G S I C Q G A F N F Y Q A Q * E R * L S L S Y C A * H * * S V N R P F S S A E * R W C C C * M F C L L L L I * F D Q * N * C F R G   F4

     C V F K H T S P S P S P P S L N I V S I C V E S G N P S S F R R I S I K L S N S H N V F A T A E T K L N S T P A R L G T G K S V G K F   F1
     V F S S I H H Q V R H H H P * I L S L F V L K V V I Q V H L G A S Q S N F P I P I M S L Q Q R K R N * I Q H Q P D * A Q E S Q S E N   F2
     L C F Q A Y I T K S V T T I L E Y C L Y L C * K W * S K F I * A H L N Q T F Q F P * C L C N S G N E T E F N T S Q I R H R K V S R K I   F3
3801 TTGTGTTTTCAAGCATACATCACCAAGTCCGTCACCACCATCCTTGAATATTGTCTCTATTTGTGTTGAAAGTGGTAATCCAAGTTCATTTAGGCGCATCTCAATCAAACTTTCCAATTCCCATAATGTCTTTGCAACAGCGGAAACGAAACTGAATTCAACACCAGCCAGATTAGGCACAGGAAAGTCAGTCGGAAAAT 4000
     ----:----|----:----|----:----|----:----|----:----|----:----|----:----|----:----|----:----|----:----|----:----|----:----|----:----|----:----|----:----|----:----|----:----|----:----|----:----|----:----|
3801 AACACAAAAGTTCGTATGTAGTGGTTCAGGCAGTGGTGGTAGGAACTTATAACAGAGATAAACACAACTTTCACCATTAGGTTCAAGTAAATCCGCGTAGAGTTAGTTTGAAAGGTTAAGGGTATTACAGAAACGTTGTCGCCTTTGCTTTGACTTAAGTTGTGGTCGGTCTAATCCGTGTCCTTTCAGTCAGCCTTTTA 4000
     Q T K L C V D G L G D G G D K F I T E I Q T S L P L G L E N L R M E I L S E L E W L T K A V A S V F S F E V G A L N P V P F D T P F   F6
     K H K * A Y M V L D T V V M R S Y Q R * K H Q F H Y D L N M * A C R L * V K W N G Y H R Q L L P F S V S N L V L W I L C L F T L R F I   F5
     K T N E L M C * W T R * W W G Q I N D R N T N F T T I W T * K P A D * D F K G I G M I D K C C R F R F Q I * C W G S * A C S L * D S F   F4

     L N * S M I S F G S Y W S L V E M D C S A M T T E S F I S L * A Q * P G I N V A S A A S S W * G G F R D A E L V L S R C R L Y C C L   F1
     S * T D P * Y P L A H T G H W W R W I V Q Q * Q Q N L S L A C K H S D Q A * M L P Q Q H L A G R G V S E M L S W Y C R V V D Y I V A Y   F2
     L K L I H D I L W L I L V I G G D G L F S N D N R I F H * L V S T V T R H K C C L S S I * L V G G F Q R C * V G I V A L * T I L L P I   F3
4001 TCTTAAACTGATCCATGATATCCTTTGGCTCATACTGGTCATTGGTGGAGATGGATTGTTCAGCAATGACAACAGAATCTTTCATTAGCTTGTAAGCACAGTGACCAGGCATAAATGTTGCCTCAGCAGCATCTAGCTGGTAGGGGGGTTTCAGAGATGCTGAGTTGGTATTGTCGCGTTGTAGACTATATTGTTGCCTA 4200
     ----:----|----:----|----:----|----:----|----:----|----:----|----:----|----:----|----:----|----:----|----:----|----:----|----:----|----:----|----:----|----:----|----:----|----:----|----:----|----:----|
4001 AGAATTTGACTAGGTACTATAGGAAACCGAGTATGACCAGTAACCACCTCTACCTAACAAGTCGTTACTGTTGTCTTAGAAAGTAATCGAACATTCGTGTCACTGGTCCGTATTTACAACGGAGTCGTCGTAGATCGACCATCCCCCCAAAGTCTCTACGACTCAACCATAACAGCGCAACATCTGATATAACAACGGAT 4200
     N K F Q D M I D K P E Y Q D N T S I S Q E A I V V S D K M L K Y A C H G P M F T A E A A D L Q Y P P K L S A S N T N D R Q L S Y Q Q R   F6
     R L S I W S I R Q S M S T M P P S P N N L L S L L I K * * S T L V T V L C L H Q R L L M * S T P P N * L H Q T P I T A N Y V I N N G   F5
     E * V S G H Y G K A * V P * Q H L H I T * C H C C F R E N A Q L C L S W A Y I N G * C C R A P L P T E S I S L Q Y Q R T T S * I T A *   F4

     Y R V F D C N V R T R T A R Q S * A D I S S S S P L F C H T D K I L F A S F F L P S S R S F W M R T A Y I A S T G S S I L S S Q K E P   F1
     T E S L T A M S E H A L Q D N P E Q T F P A P P L C F A T R T K F C L P L F F S H H Q E V F G * E Q R T L H Q L D L P S C H H K K N Q   F2
     P S L * L Q C P N T H C K T I L S R H F Q L L P F V L P H G Q N F V C L F F S P I I K K F L D E N S V H C I N W I F H P V I T K R T   F3
4201 TACCGAGTCTTTGACTGCAATGTCCGAACACGCACTGCAAGACAATCCTGAGCAGACATTTCCAGCTCCTCCCCTTTGTTTTGCCACACGGACAAAATTTTGTTTGCCTCTTTTTTTCTCCCATCATCAAGAAGTTTTTGGATGAGAACAGCGTACATTGCATCAACTGGATCTTCCATCCTGTCATCACAAAAAGAACC 4400
     ----:----|----:----|----:----|----:----|----:----|----:----|----:----|----:----|----:----|----:----|----:----|----:----|----:----|----:----|----:----|----:----|----:----|----:----|----:----|----:----|
4201 ATGGCTCAGAAACTGACGTTACAGGCTTGTGCGTGACGTTCTGTTAGGACTCGTCTGTAAAGGTCGAGGAGGGGAAACAAAACGGTGTGCCTGTTTTAAAACAAACGGAGAAAAAAAGAGGGTAGTAGTTCTTCAAAAACCTACTCTTGTCGCATGTAACGTAGTTGACCTAGAAGGTAGGACAGTAGTGTTTTTCTTGG 4400
     Y R T K S Q L T R V R V A L C D Q A S M E L E E G K N Q W V S L I K N A E K K R G D D L L K Q I L V A Y M A D V P D E M R D D C F S G   F6
     I G L R Q S C H G F V C Q L V I R L L C K W S R G K T K G C P C F K T Q R K K E G M M L F N K S S F L T C Q M L Q I K W G T M V F L V   F5
     V S D K V A I D S C A S C S L G S C V N G A G G R Q K A V R V F N Q K G R K K E W * * S T K P H S C R V N C * S S R G D Q * * L F F   F4

     T D D I K S L R R R C F M * * R E T C C S R H F F R G * G D I T S L V V P L D V P V P H S F G P D P E * E * S L E M T T S A G S I C T   F1
     L M I S S L * D V A A L C D D V K P A A V D I F F A V E E T * H R * W C H * M Y R Y H I H L G Q I L N R N D H * K * P P L R A Q F A   F2
     N * * Y Q V F E T S L L Y V M T * N L L Q S T F F S R L R R H N I V S G A I R C T G T T F I W A R S * I G M I T R N D H L C G L N L H   F3
4401 AACTGATGATATCAAGTCTTTGAGACGTCGCTGCTTTATGTGATGACGTGAAACCTGCTGCAGTCGACATTTTTTTCGCGGTTGAGGAGACATAACATCGTTAGTGGTGCCATTAGATGTACCGGTACCACATTCATTTGGGCCAGATCCTGAATAGGAATGATCACTAGAAATGACCACCTCTGCGGGCTCAATTTGCA 4600
     ----:----|----:----|----:----|----:----|----:----|----:----|----:----|----:----|----:----|----:----|----:----|----:----|----:----|----:----|----:----|----:----|----:----|----:----|----:----|----:----|
4401 TTGACTACTATAGTTCAGAAACTCTGCAGCGACGAAATACACTACTGCACTTTGGACGACGTCAGCTGTAAAAAAAGCGCCAACTCCTCTGTATTGTAGCAATCACCACGGTAATCTACATGGCCATGGTGTAAGTAAACCCGGTCTAGGACTTATCCTTACTAGTGATCTTTACTGGTGGAGACGCCCGAGTTAAACGT 4600
     V S S I L D K L R R Q K I H H R S V Q Q L R C K K R P Q P S M V D N T T G N S T G T G C E N P G S G S Y S H D S S I V V E A P E I Q   F6
     L Q H Y * T K S V D S S * T I V H F R S C D V N K E R N L L C L M T L P A M L H V P V V N M Q A L D Q I P I I V L F S W R Q P S L K C   F5
     W S I I D L R Q S T A A K H S S T F G A A T S M K K A T S S V Y C R * H H W * I Y R Y W M * K P W I R F L F S * * F H G G R R A * N A   F4

     G S L G K G N F R S E P L P T Q K Q S H S W W G K N L G A V R F L E T A V C F R R S T L L R Q L L H I H R G K T A S D S S P T E I L   F1
     Q A H W V K V T L G V S L F L H K S N H I P G G V K I * G P S D S * K P L S V L G D Q P C Y D N C C I Y I V G R Q L P T H H R Q R Y C   F2
     R L T G * R * L * E * A S S Y T K A I T F L V G * K F R G R Q I L R N R C L F * A I N L V T T T V A Y T S W E D S F R L I T D R D T V   F3
4601 CAGGCTCACTGGGTAAAGGTAACTTTAGGAGTGAGCCTCTTCCTACACAAAAGCAATCACATTCCTGGTGGGGTAAAAATTTAGGGGCCGTCAGATTCTTAGAAACCGCTGTCTGTTTTAGGCGATCAACCTTGTTACGACAACTGTTGCATATACATCGTGGGAAGACAGCTTCCGACTCATCACCGACAGAGATACTG 4800
     ----:----|----:----|----:----|----:----|----:----|----:----|----:----|----:----|----:----|----:----|----:----|----:----|----:----|----:----|----:----|----:----|----:----|----:----|----:----|----:----|
4601 GTCCGAGTGACCCATTTCCATTGAAATCCTCACTCGGAGAAGGATGTGTTTTCGTTAGTGTAAGGACCACCCCATTTTTAAATCCCCGGCAGTCTAAGAATCTTTGGCGACAGACAAAATCCGCTAGTTGGAACAATGCTGTTGACAACGTATATGTAGCACCCTTCTGTCGAAGGCTGAGTAGTGGCTGTCTCTATGAC 4800
     V P E S P L P L K L L S G R G V C F C D C E Q H P L F K P A T L N K S V A T Q K L R D V K N R C S N C I C R P F V A E S E D G V S I S   F6
     L S V P Y L Y S * S H A E E * V F A I V N R T P Y F N L P R * I R L F R Q R N * A I L R T V V V T A Y V D H S S L K R S M V S L S V   F5
     C A * Q T F T V K P T L R K R C L L L * M G P P T F I * P G D S E * F G S D T K P S * G Q * S L Q Q M Y M T P L C S G V * * R C L Y Q   F4

     * C N * R T T A A L E T S K K L F C L L T F L R S P E Q R R H I R W R T C L * Y W S I L K A R R K Q M S M L M P A K F D L I F V F T L   F1
     S A T E G Q L L L W K H Q R N Y S V F * L S * D H Q N K D D I S A G G L A C D T G P Y * K Q E G N K C Q C S C Q L N L I * Y L Y L L S   F2
     V Q L K D N C C F G N I K E I I L S F D F L E I T R T K T T Y P L E D L L V I L V H T E S K K E T N V N A H A S * I * F D I C I Y S   F3
4801 TAGTGCAACTGAAGGACAACTGCTGCTTTGGAAACATCAAAGAAATTATTCTGTCTTTTGACTTTCTTGAGATCACCAGAACAAAGACGACATATCCGCTGGAGGACTTGCTTGTGATACTGGTCCATACTGAAAGCAAGAAGGAAACAAATGTCAATGCTCATGCCAGCTAAATTTGATTTGATATTTGTATTTACTCT 5000
     ----:----|----:----|----:----|----:----|----:----|----:----|----:----|----:----|----:----|----:----|----:----|----:----|----:----|----:----|----:----|----:----|----:----|----:----|----:----|----:----|
4801 ATCACGTTGACTTCCTGTTGACGACGAAACCTTTGTAGTTTCTTTAATAAGACAGAAAACTGAAAGAACTCTAGTGGTCTTGTTTCTGCTGTATAGGCGACCTCCTGAACGAACACTATGACCAGGTATGACTTTCGTTCTTCCTTTGTTTACAGTTACGAGTACGGTCGATTTAAACTAAACTATAAACATAAATGAGA 5000
     Y H L Q L V V A A K S V D F F N N Q R K V K K L D G S C L R C I R Q L V Q K H Y Q D M S F A L L F C I D I S M G A L N S K I N T N V R   F6
     T T C S F S L Q Q K P F M L S I I R D K S K R S I V L V F V V Y G S S S K S T I S T W V S L L F S V F T L A * A L * I Q N S I Q I * E   F5
     L A V S P C S S S Q F C * L F * E T K Q S E Q S * W F L S S M D A P P S A Q S V P G Y Q F C S P F L H * H E H W S F K I Q Y K Y K S   F4
```

```
      I  S  R  K  Y  Q  Y  P  F  Y  F  S  *  E  I  L  N  T  M  N  D  S  F  T  F  P  K  L  V  I  L  N  H  I  I  T  S  E  I  Y  K  L  G  H  I  P  R  Q  L  S  Q  T  I  S  *  F  T  K  T  E  A  Q  S  Q  I  S  L   F1
       S  A  E  N  I  N  T  H  S  I  S  P  E  R  F  *  T  Q  *  M  I  H  S  H  F  P  S  W  S  F  S  T  I  *  *  L  L  K  Y  T  S  L  D  I  S  Q  D  N  S  L  K  L  S  A  D  L  P  K  Q  K  H  R  A  K  F  P    F2
     H  Q  Q  K  I  S  I  P  I  L  F  L  L  R  D  S  K  H  N  E  *  F  I  H  I  S  Q  A  G  H  S  Q  P  Y  N  N  F  *  N  I  Q  A  W  T  Y  P  K  T  T  L  S  N  Y  Q  L  I  Y  Q  N  R  S  T  E  P  N  F  L   F3
5001 CATCAGCAGAAAATATCAATACCCATTCTATTTCTCCTGAGAGATTCTAAACACAATGAATGATTCATTCACATTTCCCAAGCTGGTCATTCTCAACCATATAATAACTTCTGAAATATACAAGCTTGGACATATCCCAAGACAACTCTCTCAAACTATCAGCTGATTTACCAAAACAGAAGCACAGAGCCAAATTTCCT 5200
     ----:----|----:----|----:----|----:----|----:----|----:----|----:----|----:----|----:----|----:----|----:----|----:----|----:----|----:----|----:----|----:----|----:----|----:----|----:----|----:----|
5001 GTAGTCGTCTTTTATAGTTATGGGTAAGATAAAGAGGACTCTCTAAGATTTGTGTTACTTACTAAGTAAGTGTAAAGGGTTCGACCAGTAAGAGTTGGTATATTATTGAAGACTTTATATGTTCGAACCTGTATAGGGTTCTGTTGAGAGAGTTTGATAGTCGACTAAATGGTTTTGTCTTCGTGTCTCGGTTTAAAGGA 5200
      M  L  L  F  Y  *  Y  G  N  *  K  E  Q  S  I  R  F  V  I  F  S  E  N  V  N  G  L  S  T  M  R  L  W  I  I  V  E  S  I  Y  L  S  P  C  I  G  L  C  S  E  *  V  I  L  Q  N  V  L  V  S  A  C  L  W  I  E    F6
      *  *  C  F  I  D  I  G  M  R  N  R  R  L  S  E  L  C  L  S  H  N  M  *  M  E  W  A  P  *  E  *  G  Y  L  L  K  Q  F  I  C  A  Q  V  Y  G  L  V  V  R  E  F  *  *  S  I  *  W  F  L  L  V  S  G  F  K  R   F5
     E  D  A  S  F  I  L  V  W  E  I  E  G  S  L  N  *  V  C  H  I  I  *  E  C  K  G  L  Q  D  N  E  V  M  Y  Y  S  R  F  Y  V  L  K  S  M  D  W  S  L  E  R  L  S  D  A  S  K  G  F  C  F  C  L  A  L  N  G    F4

       R  I  Y  R  E  I  P  S  K  K  V  K  V  I  L  N  V  P  Q  N  N  K  R  I  *  R  T  R  E  S  L  P  S  Y  P  H  G  P  S  M  G  T  F  R  S  W  L  L  T  K  S  C  P  L  R  M  G  T  S  V  K  N  T  D  S  I    F1
     Y  G  S  T  E  K  F  L  A  R  R  S  R  S  S  *  M  Y  P  K  I  T  N  V  Y  D  V  H  E  K  A  C  L  P  T  P  M  A  L  Q  W  E  L  S  E  V  G  Y  *  P  N  H  V  L  *  G  W  E  Q  A  *  K  T  P  T  A  F   F2
      T  D  L  P  R  N  S  *  Q  E  G  Q  G  H  P  K  C  T  P  K  *  Q  T  Y  M  T  Y  T  R  K  P  A  F  L  P  P  W  P  F  N  G  N  F  Q  K  L  V  I  D  Q  I  M  S  S  E  D  G  N  K  R  K  K  H  R  Q  H  F   F3
5201 TACGGATCTACCGAGAAATTCCTAGCAAGAAGGTCAAGGTCATCCTAAATGTACCCCAAAATAACAAACGTATATGACGTACACGAGAAAGCCTGCCTTCCTACCCCCATGGCCCTTCAATGGGAACTTTCAGAAGTTGGTTATTGACCAAATCATGTCCTCTGAGGATGGGAACAAGCGTAAAAAACACCGACAGCATT 5400
     ----:----|----:----|----:----|----:----|----:----|----:----|----:----|----:----|----:----|----:----|----:----|----:----|----:----|----:----|----:----|----:----|----:----|----:----|----:----|----:----|
5201 ATGCCTAGATGGCTCTTTAAGGATCGTTCTTCCAGTTCCAGTAGGATTTACATGGGGTTTTATTGTTTGCATATACTGCATGTGCTCTTTCGGACGGAAGGATGGGGGTACCGGGAAGTTACCCTTGAAAGTCTTCAACCAATAACTGGTTTAGTACAGGAGACTCCTACCCTTGTTCGCATTTTTTGTGGCTGTCGTAA 5400
     K  R  I  *  R  S  I  G  L  L  F  T  L  T  M  R  F  T  G  W  F  L  L  R  I  H  R  V  R  S  L  R  G  E  *  G  W  P  G  E  I  P  V  K  L  L  Q  N  N  V  L  D  H  G  R  L  I  P  V  L  T  F  F  V  S  L  M   F6
       V  S  R  G  L  F  E  *  C  S  P  *  P  *  G  L  H  V  G  F  Y  C  V  Y  I  V  Y  V  L  F  G  A  K  R  G  G  H  G  K  L  P  F  K  *  F  N  T  I  S  W  I  M  D  E  S  S  P  F  L  R  L  F  C  R  C  C    F5
      *  P  D  V  S  F  N  R  A  L  L  D  L  D  D  *  I  Y  G  L  I  V  F  T  Y  S  T  C  S  F  A  Q  R  G  V  G  M  A  R  *  H  S  S  E  S  T  P  *  Q  G  F  *  T  R  Q  P  H  S  C  A  Y  F  V  G  V  A  N   F4

     F  I  Y  R  G  S  T  A  C  R  M  S  M  E  R  A  K  L  R  L  S  A  A  S  N  H  F  L  L  S  L  T  G  *  N  T  A  C  T  D  S  G  V  S  Y  L  P  N  T  T  V  N  P  Q  E  N  V  *  N  T  V  K  T  S  N  L  G   F1
      L  F  T  E  E  A  Q  H  V  A  C  Q  W  K  G  Q  S  F  A  F  P  L  H  P  T  I  F  C  C  R  *  Q  V  R  T  Q  L  A  Q  T  L  G  F  R  T  Y  L  I  Q  L  L  I  H  K  K  M  F  K  T  P  *  K  R  Q  I  L  A   F2
       Y  L  P  R  K  H  S  M  S  H  V  N  G  K  G  K  A  S  P  F  R  C  I  Q  P  F  F  A  V  A  N  R  L  E  H  S  L  H  R  L  W  G  F  V  P  T  *  Y  N  C  *  S  T  R  K  C  L  K  H  R  K  N  V  K  S  W    F3
5401 TTTATTTACCGAGGAAGCACAGCATGTCGCATGTCAATGGAAAGGGCAAAGCTTCGCCTTTCCGCTGCATCCAACCATTTTTGCTGTCGCTAACAGGTTAGAACACAGCTTGCACAGACTCTGGGGTTTCGTACCTACCTAATACAACTGTTAATCCACAAGAAAATGTTTAAAACACCGTAAAAACGTCAAATCTTGG 5600
     ----:----|----:----|----:----|----:----|----:----|----:----|----:----|----:----|----:----|----:----|----:----|----:----|----:----|----:----|----:----|----:----|----:----|----:----|----:----|----:----|
5401 AAATAAATGGCTCCTTCGTGTCGTACAGCGTACAGTTACCTTTCCCGTTTCGAAGCGGAAAGGCGACGTAGGTTGGTAAAAAACGACAGCGATTGTCCAATCTTGTGTCGAACGTGTCTGAGACCCCAAAGCATGGATGGATTATGTTGACAATTAGGTGTTCTTTTACAAATTTTGTGGCATTTTTGCAGTTTAGAACC 5600
      K  I  *  R  P  L  V  A  H  R  M  D  I  S  L  A  F  S  R  R  E  A  A  D  L  W  K  K  S  D  S  V  P  *  F  V  A  Q  V  S  E  P  T  E  Y  R  G  L  V  V  T  L  G  C  S  F  T  *  F  V  T  F  V  D  F  R  P   F6
     K  *  K  G  L  F  C  L  M  D  C  T  L  P  F  P  L  A  E  G  K  R  Q  M  W  G  N  K  A  T  A  L  L  N  S  C  L  K  C  L  S  Q  P  K  T  G  V  *  Y  L  Q  *  D  V  L  F  H  K  F  C  R  L  F  T  L  D  Q   F5
       K  N  V  S  S  A  C  C  T  A  H  *  H  F  P  C  L  K  A  K  G  S  C  G  V  M  K  Q  Q  R  *  C  T  L  V  C  S  A  C  V  R  P  N  R  V  *  R  I  C  S  N  I  W  L  F  I  N  L  V  G  Y  F  R  *  I  K    F4

      R  F  *  R  L  K  L  H  V  L  V  Y  F  K  M  Q  E  M  A  A  S  L  R  P  R  Q  R  E  P  P  E  K  W  E  M  K  L  E  G  *  C  W  R  Q  A  S  S  K  V  T  *  S  L  L  I  K  Q  Y  G  S  L  Q  D  *  L  R  V   F1
       D  S  E  D  *  N  C  M  Y  *  F  I  S  R  C  K  R  W  R  P  V  Y  G  H  V  N  E  N  H  L  R  N  G  K  *  S  W  K  G  D  A  G  V  K  L  P  R  R  S  R  D  R  Y  *  S  S  N  M  A  A  Y  R  T  N  Y  A    F2
     Q  I  L  K  T  E  T  A  C  I  S  L  F  Q  D  A  R  D  G  G  Q  F  T  A  T  S  T  R  T  T  *  E  M  G  N  E  V  G  R  V  M  L  A  S  S  F  L  E  G  H  V  I  A  I  D  Q  A  I  W  Q  P  T  G  L  I  T  R   F3
5601 CAGATTCTGAAGACTGAAACTGCATGTATTAGTTTATTTCAAGATGCAAGAGATGGCGGCCAGTTTACGGCCACGTCAACGAGAACCACCTGAGAAATGGGAAATGAAGTTGGAAGGGTGATGCTGGCGTCAAGCTTCCTCGAAGGTCACGTGATCGCTATTGATCAAGCAATATGGCAGCCTACAGGACTAATTACGCG 5800
     ----:----|----:----|----:----|----:----|----:----|----:----|----:----|----:----|----:----|----:----|----:----|----:----|----:----|----:----|----:----|----:----|----:----|----:----|----:----|----:----|
5601 GTCTAAGACTTCTGACTTTGACGTACATAATCAAATAAAGTTCTACGTTCTCTACCGCCGGTCAAATGCCGGTGCAGTTGCTCTTGGTGGACTCTTTACCCTTTACTTCAACCTTCCCACTACGACCGCAGTTCGAAGGAGCTTCCAGTGCACTAGCGATAACTAGTTCGTTATACCGTCGGATGTCCTGATTAATGCGC 5800
       L  N  Q  L  S  F  S  C  T  N  T  *  K  L  I  C  S  I  A  A  L  K  R  G  R  *  R  S  G  G  S  F  H  S  I  F  N  S  P  H  H  Q  R  *  A  E  E  F  T  V  H  D  S  N  I  L  C  Y  P  L  R  C  S  *  N  R    F6
      C  I  R  F  V  S  V  A  H  I  L  K  N  *  S  A  L  S  P  P  W  N  V  A  V  D  V  L  V  V  Q  S  I  P  F  S  T  P  L  T  I  S  A  D  L  K  R  S  P  *  T  I  A  I  S  *  A  I  H  C  G  V  P  S  I  V  R   F5
     A  S  E  S  S  Q  F  Q  M  Y  *  N  I  E  L  H  L  L  H  R  G  T  *  P  W  T  L  S  F  W  R  L  F  P  F  H  L  Q  F  P  S  A  P  T  L  S  G  R  L  D  R  S  R  *  Q  D  L  L  I  A  A  *  L  V  L  *  A    F4

       H  T  T  F  R  C  D  P  N  P  X                                                                                                                                                                                      F1
     Y  T  Q  H  F  G  V  T  L  T  L                                                                                                                                                                                        F2
      T  H  N  I  S  V  *  P  *  P  X                                                                                                                                                                                       F3
5801 TACACACAACATTTCGGTGTGACCCTAACCCTA 5833
     ----:----|----:----|----:----|---
5801 ATGTGTGTTGTAAAGCCACACTGGGATTGGGAT 5833
     T  C  V  V  N  R  H  S  G  L  G                                                                                                                                                                                        F6
       V  C  L  M  E  T  H  G  *  G  *                                                                                                                                                                                      F5
      Y  V  C  C  K  P  T  V  R  V  R                                                                                                                                                                                       F4
```

Notospermus geniculatus (Nge)

WGS: NMRB01002322.1

104762-109049
Notospermus geniculatus strain Ushimado scaffold2322, whole genome shotgun sequence

```
     I  F  R  N  V  V  C  D  G  *  F  S  F  R  S  P  Q  T  P  V  S  H  L  F  W  C  P  *  F  Q  G  M  W  C  S  T  K  T  E  E  F  P  F  S  G  S  M  C  F  S  S  S  Q  D  L  W  D  I  S  G  N  *  G  Q  Q  C  I    F1
      F  S  E  M  L  S  A  M  D  D  F  H  S  E  V  L  K  H  L  C  R  I  C  S  G  V  L  N  S  K  A  C  G  A  A  P  R  Q  K  S  F  L  S  V  E  A  C  A  S  A  L  L  R  I  Y  G  I  S  V  E  T  E  D  N  S  V  F   F2
       F  Q  K  C  C  L  R  W  M  I  F  I  Q  K  S  S  N  T  C  V  A  F  V  L  V  S  L  I  P  R  H  V  V  Q  H  Q  D  R  R  V  S  F  Q  W  K  H  V  L  Q  L  F  S  G  S  M  G  Y  Q  W  K  L  R  T  T  V  Y    F3
   1 ATTTTCAGAAATGTTGTCTGCGATGGATGATTTTCATTCAGAAGTCCTCAAACACCTGTGTCGCATTTGTTCTGGTGTCCTTAATTCCAAGGCATGTGGTGCAGCACCAAGACAGAAGAGTTTCCTTTCAGTGGAAGCATGTGCTTCAGCTCTTCTCAGGATCTATGGGATATCAGTGGAAACTGAGGACAACAGTGTAT 200
     ----:----|----:----|----:----|----:----|----:----|----:----|----:----|----:----|----:----|----:----|----:----|----:----|----:----|----:----|----:----|----:----|----:----|----:----|----:----|----:----|
   1 TAAAAGTCTTTACAACAGACGCTACCTACTAAAAGTAAGTCTTCAGGAGTTTGTGGACACAGCGTAAACAAGACCACAGGAATTAAGGTTCCGTACACCACGTCGTGGTTCGTGTCTTCTCAAAGGAAAGTCACCTTCGTACACGAAGTCGAGAAGAGTCCTAGATACCCTATAGTCACCTTTGACTCCTGTTGTCACATA 200
      X  K  L  F  T  T  Q  S  P  H  N  E  N  L  L  G  *  V  G  T  D  C  K  N  Q  H  G  *  N  W  P  M  H  H  L  V  L  V  S  S  N  G  K  L  P  L  M  H  K  L  E  E  *  S  R  H  S  I  L  P  F  Q  P  C  C  H  I   F6
     X  K  *  F  H  Q  R  R  H  I  I  K  M  *  F  D  E  F  V  Q  T  A  N  T  R  T  D  K  I  G  L  C  T  T  C  C  W  S  L  L  T  E  K  *  H  F  C  T  S  *  S  K  E  P  D  I  P  Y  *  H  F  S  L  V  V  T  Y    F5
       N  E  S  I  N  D  A  I  S  S  K  *  E  S  T  R  L  C  R  H  R  M  Q  E  P  T  R  L  E  L  A  H  P  A  A  G  L  C  F  L  K  R  E  T  S  A  H  A  E  A  R  R  L  I  *  P  I  D  T  S  V  S  S  L  L  T    F4

      S  N  F  S  V  Q  Q  V  Q  N  *  N  *  *  G  N  E  K  D  C  N  *  A  A  G  V  T  S  F  V  P  S  P  S  A  *  *  F  E  L  Y  L  P  Q  V  M  L  S  C  Q  C  S  I  A  T  P  N  H  R  I  G  R  Y  *  T  I  N   F1
       P  T  S  V  C  N  K  C  R  I  K  I  S  R  E  M  K  K  T  V  I  E  Q  Q  V  L  P  H  L  F  R  P  H  Q  H  D  D  L  N  C  I  C  H  K  L  C  S  A  V  S  V  P  L  P  P  P  T  T  E  S  V  A  I  E  P  S    F2
      F  Q  L  Q  C  A  T  S  A  E  L  K  L  V  G  K  *  K  R  L  *  L  S  S  R  C  Y  L  I  C  S  V  P  I  S  M  M  I  *  T  V  F  A  T  S  Y  A  Q  L  S  V  F  H  C  H  P  Q  P  Q  N  R  S  L  L  N  H  Q    F3
 201 TTCCAACTTCAGTGTGCAACAAGTGCAGAATTAAAATTAGTAGGGAAATGAAAAAGACTGTAATTGAGCAGCAGGTGTTACCTCATTTGTTCCGTCCCCATCAGCATGATGATTTGAACTGTATTTGCCACAAGTTATGCTCAGCTGTCAGTGTTCCATTGCCACCCCCAACCACAGAATCGGTCGCTATTGAACCATCA 400
     ----:----|----:----|----:----|----:----|----:----|----:----|----:----|----:----|----:----|----:----|----:----|----:----|----:----|----:----|----:----|----:----|----:----|----:----|----:----|----:----|
 201 AAGGTTGAAGTCACACGTTGTTCACGTCTTAATTTTAATCATCCCTTTACTTTTTCTGACATTAACTCGTCGTCCACAATGGAGTAAACAAGGCAGGGGTAGTCGTACTACTAAACTTGACATAAACGGTGTTCAATACGAGTCGACAGTCACAAGGTAACGGTGGGGGTTGGTGTCTTAGCCAGCGATAACTTGGTAGT 400
       E  L  K  L  T  C  C  T  C  F  *  F  *  Y  P  F  S  F  S  Q  L  Q  A  A  P  T  V  E  N  T  G  D  G  D  A  H  H  N  S  S  Y  K  G  C  T  I  S  L  Q  *  H  E  M  A  V  G  L  W  L  I  P  R  *  Q  V  M    F6
      K  W  S  *  H  A  V  L  A  S  N  F  N  T  P  F  H  F  L  S  Y  N  L  L  L  H  *  R  M  Q  E  T  G  M  L  M  I  I  Q  V  T  N  A  V  L  *  A  *  S  D  T  N  W  Q  W  G  W  G  C  F  R  D  S  N  F  W  *   F5
     N  G  V  E  T  H  L  L  H  L  I  L  I  L  L  S  I  F  F  V  T  I  S  C  C  T  N  G  *  K  N  R  G  W  *  C  S  S  K  F  Q  I  Q  W  L  N  H  E  A  T  L  T  G  N  G  G  G  V  V  S  D  T  A  I  S  G  D    F4

      S  D  Y  *  T  *  L  R  S  *  Y  W  C  C  C  K  T  K  C  T  F  N  T  *  G  S  *  *  K  K  F  E  G  V  E  P  T  V  H  K  E  N  P  F  E  R  P  F  L  K  P  S  *  L  L  *  R  E  K  R  G  C  G  R  C  Y    F1
     I  L  T  I  E  H  S  Y  V  H  D  I  G  A  A  A  K  P  S  A  P  S  T  P  E  V  R  S  K  K  S  L  K  E  L  N  R  Q  Y  I  K  R  T  R  L  K  D  L  F  S  N  L  H  D  F  C  K  E  K  K  E  D  V  V  D  V  T    F2
      F  *  L  L  N  I  A  T  F  M  I  L  V  L  L  Q  N  Q  V  H  L  Q  H  L  R  F  V  V  K  K  V  *  R  S  *  T  D  S  T  *  R  E  P  V  *  K  T  F  S  Q  T  F  M  T  F  V  K  R  K  K  R  M  W  *  M  L  L   F3
 401 ATTCTGACTATTGAACATAGCTACGTTCATGATATTGGTGCTGCTGCAAAACCAAGTGCACCTTCAACACCTGAGGTTCGTAGTAAAAAAAGTTTGAAGGAGTTGAACCGACAGTACATAAAGAGAACCCGTTTGAAAGACCTTTTCTCAAACCTTCATGACTTTTGTAAAGAGAAAAAAGAGGATGTGGTAGATGTTAC 600
     ----:----|----:----|----:----|----:----|----:----|----:----|----:----|----:----|----:----|----:----|----:----|----:----|----:----|----:----|----:----|----:----|----:----|----:----|----:----|----:----|
 401 TAAGACTGATAACTTGTATCGATGCAAGTACTATAACCACGACGACGTTTTGGTTCACGTGGAAGTTGTGGACTCCAAGCATCATTTTTTTCAAACTTCCTCAACTTGGCTGTCATGTATTTCTCTTGGGCAAACTTTCTGGAAAAGAGTTTGGAAGTACTGAAAACATTTCTCTTTTTTCTCCTACACCATCTACAATG 600
     L  E  S  *  Q  V  Y  S  R  E  H  Y  Q  H  Q  Q  L  V  L  H  V  K  L  V  Q  P  E  Y  Y  F  F  N  S  P  T  S  G  V  T  C  L  S  F  G  N  S  L  G  K  R  L  G  E  H  S  K  Y  L  S  F  L  P  H  P  L  H  *   F6
       N  Q  S  N  F  M  A  V  N  M  I  N  T  S  S  C  F  W  T  C  R  *  C  R  L  N  T  T  F  F  T  Q  L  L  Q  V  S  L  V  Y  L  S  G  T  Q  F  V  K  E  *  V  K  M  V  K  T  F  L  F  F  L  I  H  Y  I  N    F5
      I  R  V  I  S  C  L  *  T  *  S  I  P  A  A  A  F  G  L  A  G  E  V  G  S  T  R  L  L  F  L  K  F  S  N  F  R  C  Y  M  F  L  V  R  K  F  S  R  K  E  F  R  *  S  K  Q  L  S  F  F  S  S  T  T  S  T  V   F4

     F  *  S  P  N  T  G  T  Y  *  F  R  S  *  K  G  S  *  S  C  H  G  D  L  E  R  E  *  Q  I  K  I  V  S  *  G  M  F  S  H  E  G  E  K  H  T  L  Q  D  A  L  C  A  D  V  C  S  S  Q  G  Q  D  Q  F  E  F  P    F1
      F  S  L  L  I  Q  E  L  I  D  S  D  R  K  K  E  A  D  R  V  M  E  I  W  S  G  S  D  K  S  K  L  S  V  E  E  C  L  A  M  R  V  R  N  T  L  S  K  T  H  Y  A  Q  M  Y  A  V  H  R  D  K  T  S  L  N  S  L   F2
       L  V  S  *  Y  R  N  L  L  I  Q  I  V  K  R  K  L  I  V  S  W  R  F  G  A  G  V  T  N  Q  N  C  Q  L  R  N  V  *  P  *  G  *  E  T  H  S  P  R  R  T  M  R  R  C  M  Q  F  T  G  T  R  P  V  *  I  P    F3
 601 TTTTAGTCTCCTAATACAGGAACTTATTGATTCAGATCGTAAAAAGGAAGCTGATCGTGTCATGGAGATTTGGAGCGGGAGTGACAAATCAAAATTGTCAGTTGAGGAATGTTTAGCCATGAGGGTGAGAAACACACTCTCCAAGACGCACTATGCGCAGATGTATGCAGTTCACAGGGACAAGACCAGTTTGAATTCCC 800
     ----:----|----:----|----:----|----:----|----:----|----:----|----:----|----:----|----:----|----:----|----:----|----:----|----:----|----:----|----:----|----:----|----:----|----:----|----:----|----:----|
 601 AAAATCAGAGGATTATGTCCTTGAATAACTAAGTCTAGCATTTTTCCTTCGACTAGCACAGTACCTCTAAACCTCGCCCTCACTGTTTAGTTTTAACAGTCAACTCCTTACAAATCGGTACTCCCACTCTTTGTGTGAGAGGTTCTGCGTGATACGCGTCTACATACGTCAAGTGTCCCTGTTCTGGTCAAACTTAAGGG 800
      K  *  D  G  L  V  P  V  *  Q  N  L  D  Y  F  P  L  Q  D  H  *  P  S  K  S  R  S  H  C  I  L  I  T  L  Q  P  I  N  L  W  S  P  S  F  C  V  R  W  S  A  S  H  A  S  T  H  L  E  C  P  C  S  W  N  S  N  G    F6
     S  K  T  E  *  Y  L  F  K  N  I  *  I  T  F  L  F  S  I  T  D  H  L  N  P  A  P  T  V  F  *  F  Q  *  N  L  F  T  *  G  H  P  H  S  V  C  E  G  L  R  V  I  R  L  H  I  C  N  V  P  V  L  G  T  Q  I  G    F5
       K  L  R  R  I  C  S  S  I  S  E  S  R  L  F  S  A  S  R  T  M  S  I  Q  L  P  L  S  L  D  F  N  D  T  S  S  H  K  A  M  L  T  L  F  V  S  E  L  V  C  *  A  C  I  Y  A  T  *  L  S  L  V  L  K  F  E    F4

      T  T  S  E  T  I  D  I  H  *  K  L  I  F  P  R  *  L  C  L  Q  V  I  Q  K  *  L  F  N  S  G  T  G  Y  I  K  Q  G  *  L  *  T  K  G  H  N  E  K  L  C  K  L  S  K  *  F  S  I  T  Q  C  V  R  F  G  V  R   F1
       Q  P  V  K  Q  L  T  S  I  E  N  L  F  F  P  G  N  C  A  Y  K  L  F  K  N  D  C  L  I  Q  E  Q  D  I  L  S  K  V  D  C  E  P  K  D  I  M  R  N  F  A  N  F  P  S  D  F  P  L  P  N  V  S  G  L  E  F    F2
     Y  N  Q  *  N  N  *  H  P  L  K  T  Y  F  S  Q  V  T  V  L  T  S  Y  S  K  M  T  V  *  F  R  N  R  I  Y  *  A  R  L  I  V  N  Q  R  T  *  *  E  T  L  Q  T  F  Q  V  I  F  H  Y  P  M  C  Q  V  W  S  S    F3
 801 TACAACCAGTGAAACAATTGACATCCATTGAAAACTTATTTTTCCCAGGTAACTGTGCTTACAAGTTATTCAAAAATGACTGTTTAATTCAGGAACAGGATATATTAAGCAAGGTTGATTGTGAACCAAAGGACATAATGAGAAACTTTGCAAACTTTCCAAGTGATTTTCCATTACCCAATGTGTCAGGTTTGGAGTTC 1000
     ----:----|----:----|----:----|----:----|----:----|----:----|----:----|----:----|----:----|----:----|----:----|----:----|----:----|----:----|----:----|----:----|----:----|----:----|----:----|----:----|
 801 ATGTTGGTCACTTTGTTAACTGTAGGTAACTTTTGAATAAAAAGGGTCCATTGACACGAATGTTCAATAAGTTTTTACTGACAAATTAAGTCCTTGTCCTATATAATTCGTTCCAACTAACACTTGGTTTCCTGTATTACTCTTTGAAACGTTTGAAAGGTTCACTAAAAGGTAATGGGTTACACAGTCCAAACCTCAAG 1000
       V  V  L  S  V  I  S  M  W  Q  F  S  I  K  G  L  Y  S  H  K  C  T  I  *  F  H  S  N  L  E  P  V  P  Y  I  L  C  P  Q  N  H  V  L  P  C  L  S  F  S  Q  L  S  E  L  H  N  E  M  V  W  H  T  L  N  P  T    F6
      *  L  W  H  F  L  Q  C  G  N  F  V  *  K  E  W  T  V  T  S  V  L  *  E  F  I  V  T  *  N  L  F  L  I  Y  *  A  L  N  I  T  F  W  L  V  Y  H  S  V  K  C  V  K  W  T  I  K  W  *  G  I  H  *  T  Q  L  E   F5
     R  C  G  T  F  C  N  V  D  M  S  F  K  N  K  G  P  L  Q  A  *  L  N  N  L  F  S  Q  K  I  *  S  C  S  I  N  L  L  T  S  Q  S  G  F  S  M  I  L  F  K  A  F  K  G  L  S  K  G  N  G  L  T  D  P  K  S  N    F4

      L  H  I  S  S  G  K  N  I  D  *  I  R  R  E  Y  Q  C  S  A  K  R  L  W  S  S  N  *  H  I  C  P  H  H  N  *  R  W  W  *  W  S  W  R  R  E  H  *  D  S  *  G  R  I  L  F  N  *  Q  S  L  *  V  L  I  L    F1
     D  Y  I  S  A  V  A  K  T  L  T  E  L  E  E  S  I  S  A  R  L  K  D  C  G  L  P  I  D  T  S  V  H  T  I  I  K  D  G  G  D  G  L  G  D  V  N  I  K  T  R  K  G  E  F  S  L  T  D  K  V  F  R  Y  S  F  C    F2
      T  T  Y  Q  Q  W  Q  K  H  *  L  N  *  K  R  V  S  V  L  G  *  K  T  V  V  F  Q  L  T  H  L  S  T  P  *  L  K  M  V  V  M  V  L  A  T  *  T  L  R  L  V  R  A  N  S  L  *  L  T  K  S  L  G  T  H  S  A   F3
1001 GACTACATATCAGCAGTGGCAAAAACATTGACTGAATTAGAAGAGAGTATCAGTGCTCGGCTAAAAGACTGTGGTCTTCCAATTGACACATCTGTCCACACCATAATTAAAGATGGTGGTGATGGTCTTGGCGACGTGAACATTAAGACTCGTAAGGGCGAATTCTCTTTAACTGACAAAGTCTTTAGGTACTCATTCTG 1200
     ----:----|----:----|----:----|----:----|----:----|----:----|----:----|----:----|----:----|----:----|----:----|----:----|----:----|----:----|----:----|----:----|----:----|----:----|----:----|----:----|
1001 CTGATGTATAGTCGTCACCGTTTTTGTAACTGACTTAATCTTCTCTCATAGTCACGAGCCGATTTTCTGACACCAGAAGGTTAACTGTGTAGACAGGTGTGGTATTAATTTCTACCACCACTACCAGAACCGCTGCACTTGTAATTCTGAGCATTCCCGCTTAAGAGAAATTGACTGTTTCAGAAATCCATGAGTAAGAC 1200
     R  S  C  I  L  L  P  L  F  M  S  Q  I  L  L  S  Y  *  H  E  A  L  L  S  H  D  E  L  Q  C  M  Q  G  C  W  L  *  L  H  H  H  H  D  Q  R  R  S  C  *  S  E  Y  P  R  I  R  K  L  Q  C  L  R  *  T  S  M  R    F6
       V  V  Y  *  C  H  C  F  C  Q  S  F  *  F  L  T  D  T  S  P  *  F  V  T  T  K  W  N  V  C  R  D  V  G  Y  N  F  I  T  T  I  T  K  A  V  H  V  N  L  S  T  L  A  F  E  R  *  S  V  F  D  K  P  V  *  E    F5
     S  *  M  D  A  T  A  F  V  N  V  S  N  S  S  L  I  L  A  R  S  F  S  Q  P  R  G  I  S  V  D  T  W  V  M  I  L  S  P  P  S  P  R  P  S  T  F  M  L  V  R  L  P  S  N  E  K  V  S  L  T  K  L  Y  E  N  Q   F4

     R  P  F  M  *  C  N  S  E  *  E  K  I  *  I  V  Q  G  S  I  S  *  F  C  *  L  H  P  P  G  S  Y  L  P  L  *  *  K  R  Q  T  E  H  F  Y  H  I  G  *  Y  L  Q  S  K  R  C  D  A  E  W  C  D  E  V  E  V  W   F1
      V  L  S  C  S  A  T  V  N  E  K  K  Y  E  L  F  K  E  A  S  A  S  S  V  D  C  T  R  P  V  A  I  C  H  C  D  E  S  D  R  P  S  I  S  I  T  L  A  N  I  S  K  A  R  D  A  M  Q  N  G  V  M  K  L  R  F  G   F2
       S  F  H  V  V  Q  Q  *  M  R  K  N  M  N  C  S  R  K  H  Q  L  V  L  L  I  A  P  A  R  *  L  F  A  I  V  M  K  A  T  D  R  A  F  L  S  H  W  L  I  S  P  K  Q  E  M  R  C  R  M  V  *  *  S  *  G  L    F3
1201 CGTCCTTTCATGTAGTGCAACAGTGAATGAGAAAAAATATGAATTGTTCAAGGAAGCATCAGCTAGTTCTGTTGATTGCACCCGCCCGGTAGCTATTTGCCATTGTGATGAAAGCGACAGACCGAGCATTTCTATCACATTGGCTAATATCTCCAAAGCAAGAGATGCGATGCAGAATGGTGTGATGAAGTTGAGGTTTG 1400
     ----:----|----:----|----:----|----:----|----:----|----:----|----:----|----:----|----:----|----:----|----:----|----:----|----:----|----:----|----:----|----:----|----:----|----:----|----:----|----:----|
1201 GCAGGAAAGTACATCACGTTGTCACTTACTCTTTTTTATACTTAACAAGTTCCTTCGTAGTCGATCAAGACAACTAACGTGGGCGGGCCATCGATAAACGGTAACACTACTTTCGCTGTCTGGCTCGTAAAGATAGTGTAACCGATTATAGAGGTTTCGTTCTCTACGCTACGTCTTACCACACTACTTCAACTCCAAAC 1400
      R  G  K  M  Y  H  L  L  S  H  S  F  I  H  I  T  *  P  L  M  L  *  N  Q  Q  N  C  G  G  P  L  *  K  G  N  H  H  F  R  C  V  S  C  K  *  *  M  P  *  Y  R  W  L  L  L  H  S  A  S  H  H  S  S  T  S  T  Q   F6
     A  D  K  *  T  T  C  C  H  I  L  F  F  I  F  Q  E  L  F  C  *  S  T  R  N  I  A  G  A  R  Y  S  N  A  M  T  I  F  A  V  S  R  A  N  R  D  C  Q  S  I  D  G  F  C  S  I  R  H  L  I  T  H  H  L  Q  P  K    F5
       T  R  E  H  L  A  V  T  F  S  F  F  Y  S  N  N  L  S  A  D  A  L  E  T  S  Q  V  R  G  T  A  I  Q  W  Q  S  S  L  S  L  G  L  M  E  I  V  N  A  L  I  E  L  A  L  S  A  I  C  F  P  T  I  F  N  L  N    F4

      W  W  T  H  C  T  *  E  V  P  H  T  A  V  S  L  Y  H  G  *  *  E  I  *  T  D  I  L  R  A  P  G  *  W  V  Q  I  P  L  Y  S  V  R  S  H  H  C  Q  L  P  V  *  F  G  F  I  H  S  Y  K  N  T  *  S  D  R  L   F1
       G  G  H  I  V  P  E  R  S  H  T  L  Q  F  L  F  T  M  V  D  E  K  F  E  R  T  S  S  G  L  Q  G  S  G  S  R  Y  L  C  T  L  C  E  A  T  T  A  N  C  Q  C  D  L  G  S  F  T  R  T  R  T  H  D  Q  T  V    F2
     V  V  D  T  L  Y  L  R  G  P  T  H  C  S  F  S  L  P  W  L  M  R  N  L  N  G  H  P  Q  G  S  R  V  V  G  P  D  T  F  V  L  C  A  K  P  P  L  P  I  A  S  V  I  W  V  H  S  L  V  Q  E  H  M  I  R  P  S    F3
1401 GTGGTGGACACATTGTACCTGAGAGGTCCCACACACTGCAGTTTCTCTTTACCATGGTTGATGAGAAATTTGAACGGACATCCTCAGGGCTCCAGGGTAGTGGGTCCAGATACCTTTGTACTCTGTGCGAAGCCACCACTGCCAATTGCCAGTGTGATTTGGGTTCATTCACTCGTACAAGAACACATGATCAGACCGTC 1600
     ----:----|----:----|----:----|----:----|----:----|----:----|----:----|----:----|----:----|----:----|----:----|----:----|----:----|----:----|----:----|----:----|----:----|----:----|----:----|----:----|
1401 CACCACCTGTGTAACATGGACTCTCCAGGGTGTGTGACGTCAAAGAGAAATGGTACCAACTACTCTTTAAACTTGCCTGTAGGAGTCCCGAGGTCCCATCACCCAGGTCTATGGAAACATGAGACACGCTTCGGTGGTGACGGTTAACGGTCACACTAAACCCAAGTAAGTGAGCATGTTCTTGTGTACTAGTCTGGCAG 1600
      H  H  V  C  Q  V  Q  S  T  G  C  V  A  T  E  R  *  W  P  Q  H  S  I  Q  V  S  M  R  L  A  G  P  Y  H  T  W  I  G  K  Y  E  T  R  L  W  W  Q  W  N  G  T  H  N  P  N  M  *  E  Y  L  F  V  H  D  S  R    F6
       T  T  S  V  N  Y  R  L  P  G  V  C  Q  L  K  E  K  G  H  N  I  L  F  K  F  P  C  G  *  P  E  L  T  T  P  G  S  V  K  T  S  Q  A  F  G  G  S  G  I  A  L  T  I  Q  T  *  E  S  T  C  S  C  M  I  L  G  D   F5
     P  P  P  C  M  T  G  S  L  D  W  V  S  C  N  R  K  V  M  T  S  S  F  N  S  R  V  D  E  P  S  W  P  L  P  D  L  Y  R  Q  V  R  H  S  A  V  V  A  L  Q  W  H  S  K  P  E  N  V  R  V  L  V  C  S  *  V  T    F4

      P  L  Q  V  S  F  *  K  S  *  R  T  K  *  K  *  L  G  *  E  M  Q  R  G  *  G  Y  A  T  D  F  F  R  C  I  *  T  F  T  *  F  Y  P  C  K  Y  K  H  G  E  I  F  Q  E  D  T  C  K  G  S  G  *  S  V  R  V    F1
     S  L  Y  R  Y  R  F  E  N  P  E  E  L  N  E  N  D  L  A  E  K  C  K  G  V  K  A  M  P  L  I  S  S  D  A  S  E  R  S  L  D  S  T  H  A  N  I  N  M  G  R  F  F  K  R  I  L  V  R  E  A  A  E  V  Y  E  W    F2
      P  S  T  G  I  V  L  K  I  L  K  N  *  M  K  M  T  W  L  R  N  A  K  G  L  R  L  C  H  *  F  L  P  M  H  L  N  V  H  L  I  L  P  M  Q  I  *  T  W  G  D  F  S  R  G  Y  L  *  G  K  R  L  K  C  T  S  G    F3
1601 TCCCTCTACAGGTATCGTTTTGAAAATCCTGAAGAACTAAATGAAAATGACTTGGCTGAGAAATGCAAAGGGGTTAAGGCTATGCCACTGATTTCTTCCGATGCATCTGAACGTTCACTTGATTCTACCCATGCAAATATAAACATGGGGAGATTTTTCAAGAGGATACTTGTAAGGGAAGCGGCTGAAGTGTACGAGTG 1800
     ----:----|----:----|----:----|----:----|----:----|----:----|----:----|----:----|----:----|----:----|----:----|----:----|----:----|----:----|----:----|----:----|----:----|----:----|----:----|----:----|
1601 AGGGAGATGTCCATAGCAAAACTTTTAGGACTTCTTGATTTACTTTTACTGAACCGACTCTTTACGTTTCCCCAATTCCGATACGGTGACTAAAGAAGGCTACGTAGACTTGCAAGTGAACTAAGATGGGTACGTTTATATTTGTACCCCTCTAAAAAGTTCTCCTATGAACATTCCCTTCGCCGACTTCACATGCTCAC 1800
     R  G  R  C  T  D  N  Q  F  D  Q  L  V  L  H  F  H  S  P  Q  S  I  C  L  P  *  P  *  A  V  S  K  K  R  H  M  Q  V  N  V  Q  N  *  G  H  L  Y  L  C  P  S  I  K  *  S  S  V  Q  L  P  L  P  Q  L  T  R  T    F6
       G  E  V  P  I  T  K  F  I  R  F  F  *  I  F  I  V  Q  S  L  F  A  F  P  N  L  S  H  W  Q  N  R  G  I  C  R  F  T  *  K  I  R  G  M  C  I  Y  V  H  P  S  K  E  L  P  Y  K  Y  P  F  R  S  F  H  V  L    F5
      E  R  *  L  Y  R  K  S  F  G  S  S  S  F  S  F  S  K  A  S  F  H  L  P  T  L  A  I  G  S  I  E  E  S  A  D  S  R  E  S  S  E  V  W  A  F  I  F  M  P  L  N  K  L  L  I  S  T  L  S  A  A  S  T  Y  S  H   F4

     D  G  I  R  Q  K  Q  I  S  A  P  A  C  *  K  V  F  R  C  H  V  D  R  E  I  R  Y  P  A  K  N  D  Y  G  R  Q  *  C  Q  E  T  F  Q  W  S  K  P  *  Y  C  H  I  T  D  Q  K  *  G  K  E  G  T  C  E  A  H  I    F1
      T  E  S  D  R  N  R  S  R  L  Q  H  V  E  K  S  L  D  A  M  L  I  E  K  L  G  I  Q  P  K  M  I  M  V  G  N  D  A  R  K  L  F  N  G  A  N  H  D  I  V  T  S  L  I  K  N  E  E  R  K  G  L  V  K  H  I  L   F2
       R  N  Q  T  E  T  D  L  G  S  S  M  L  K  S  L  *  M  P  C  *  *  R  N  *  V  S  S  Q  K  *  L  W  *  A  M  M  P  G  N  F  S  M  E  Q  T  M  I  L  S  H  H  *  S  K  M  R  K  G  R  D  L  *  S  T  Y    F3
1801 GACGGAATCAGACAGAAACAGATCTCGGCTCCAGCATGTTGAAAAGTCTTTAGATGCCATGTTGATAGAGAAATTAGGTATCCAGCCAAAAATGATTATGGTAGGCAATGATGCCAGGAAACTTTTCAATGGAGCAAACCATGATATTGTCACATCACTGATCAAAAATGAGGAAAGGAAGGGACTTGTGAAGCACATAT 2000
     ----:----|----:----|----:----|----:----|----:----|----:----|----:----|----:----|----:----|----:----|----:----|----:----|----:----|----:----|----:----|----:----|----:----|----:----|----:----|----:----|
1801 CTGCCTTAGTCTGTCTTTGTCTAGAGCCGAGGTCGTACAACTTTTCAGAAATCTACGGTACAACTATCTCTTTAATCCATAGGTCGGTTTTTACTAATACCATCCGTTACTACGGTCCTTTGAAAAGTTACCTCGTTTGGTACTATAACAGTGTAGTGACTAGTTTTTACTCCTTTCCTTCCCTGAACACTTCGTGTATA 2000
      S  P  I  L  C  F  C  I  E  A  G  A  H  Q  F  T  K  L  H  W  T  S  L  S  I  L  Y  G  A  L  F  S  *  P  L  C  H  H  W  S  V  K  *  H  L  L  G  H  Y  Q  *  M  V  S  *  F  H  P  F  S  P  V  Q  S  A  C  I    F6
     P  R  F  *  V  S  V  S  R  P  E  L  M  N  F  L  R  *  I  G  H  Q  Y  L  F  *  T  D  L  W  F  H  N  H  Y  A  I  I  G  P  F  K  E  I  S  C  V  M  I  N  D  C  *  Q  D  F  I  L  F  P  L  S  K  H  L  V  Y    F5
       V  S  D  S  L  F  L  D  R  S  W  C  T  S  F  D  K  S  A  M  N  I  S  F  N  P  I  W  G  F  I  I  I  T  P  L  S  A  L  F  S  K  L  P  A  F  W  S  I  T  V  D  S  I  L  F  S  S  L  F  P  S  T  F  C  M    F4

                                                                                                                                                covered by transcripts (see bellow)
      V  T  V  F  *  N  A  F  S  V  F  F  Y  L  T  H  S  R  A  S  R  V  D  R  K  L  Q  R  N  S  L  P  I  F  I  L  S  W  *  T  L  S  E  Y  I  T  W  S  N  Y  L  H  K  V  V  E  H  V  A  E  I  I  S  D  P  H  G   F1
      S  L  Y  S  K  M  H  S  V  Y  S  S  T  S  P  T  V  E  L  P  E  L  T  G  N  Y  R  E  I  A  F  Q  F  S  S  C  L  G  E  H  F  P  S  T  L  P  G  R  T  T  C  I  R  W  W  S  M  L  Q  K  L  S  V  T  P  M   F2
     C  H  C  I  L  K  C  I  Q  C  I  L  L  P  H  P  Q  *  S  F  Q  S  *  Q  E  I  T  E  K  *  P  S  N  F  H  L  V  L  V  N  T  F  R  V  H  Y  L  V  E  L  P  A  *  G  G  G  A  C  C  R  N  Y  Q  *  P  P  W    F3
2001 TGTCACTGTATTCTAAAATGCATTCAGTGTATTCTTCTACCTCACCCACAGTAGAGCTTCCAGAGTTGACAGGAAATTACAGAGAAATAGCCTTCCAATTTTCATCTTGTCTTGGTGAACACTTTCCGAGTACATTACCTGGTCGAACTACCTGCATAAGGTGGTGGAGCATGTTGCAGAAATTATCAGTGACCCCCATG 2200
     ----:----|----:----|----:----|----:----|----:----|----:----|----:----|----:----|----:----|----:----|----:----|----:----|----:----|----:----|----:----|----:----|----:----|----:----|----:----|----:----|
2001 ACAGTGACATAAGATTTTACGTAAGTCACATAAGAAGATGGAGTGGGTGTCATCTCGAAGGTCTCAACTGTCCTTTAATGTCTCTTTATCGGAAGGTTAAAAGTAGAACAGAACCACTTGTGAAAGGCTCATGTAATGGACCAGCTTGATGGACGTATTCCACCACCTCGTACAACGTCTTTAATAGTCACTGGGGGTAC 2200
       T  V  T  N  *  F  A  N  L  T  N  K  *  R  V  W  L  L  A  E  L  T  S  L  F  N  C  L  F  L  R  G  I  K  M  K  D  Q  H  V  S  E  S  Y  M  V  Q  D  F  *  R  C  L  T  T  S  C  T  A  S  I  I  L  S  G  W    F6
      Q  *  Q  I  R  F  H  M  *  H  I  R  R  G  *  G  C  Y  L  K  W  L  Q  C  S  I  V  S  F  Y  G  E  L  K  *  R  T  K  T  F  V  K  R  T  C  *  R  T  S  S  G  A  Y  P  P  P  A  H  Q  L  F  *  *  H  G  G  H   F5
     N  D  S  Y  E  L  I  C  E  T  Y  E  E  V  E  G  V  T  S  S  G  S  N  V  P  F  *  L  S  I  A  K  W  N  E  D  Q  R  P  S  C  K  G  L  V  N  G  P  R  V  V  Q  M  L  H  H  L  M  N  C  F  N  D  T  V  G  M    F4

       L  G  S  V  G  L  L  S  S  E  G  G  E  S  G  N  K  L  Y  R  H  I  R  K  H  L  S  R  H  D  S  V  N  D  L  R  D  A  L  V  F  H  W  L  Y  T  S  K  R  L  Q  T  L  A  S  T  T  H  I  K  Q  H  C  T  A  C    F1
     G  W  A  V  L  A  C  C  Q  V  R  V  G  N  L  E  I  N  S  T  V  T  S  E  N  I  C  L  G  M  T  V  *  M  T  C  E  M  P  L  F  F  T  G  S  I  P  A  K  G  S  R  H  L  P  A  P  L  T  S  S  N  T  V  Q  P  V    F2
      A  G  Q  C  W  L  V  V  K  *  G  W  G  I  W  K  *  T  L  P  S  H  Q  K  T  F  V  *  A  *  Q  C  K  *  P  A  R  C  P  C  F  S  L  A  L  Y  Q  Q  K  A  P  D  T  C  Q  H  H  S  H  Q  A  T  L  Y  S  L  W   F3
2201 GGCTGGGCAGTGTTGGCTTGTTGTCAAGTGAGGGTGGGGAATCTGGAAATAAACTCTACCGTCACATCAGAAAACATTTGTCTAGGCATGACAGTGTAAATGACCTGCGAGATGCCCTTGTTTTCACTGGCTCTATACCAGCAAAAGGCTCCAGACACTTGCCAGCACCACTCACATCAAGCAACACTGTACAGCCTGT 2400
     ----:----|----:----|----:----|----:----|----:----|----:----|----:----|----:----|----:----|----:----|----:----|----:----|----:----|----:----|----:----|----:----|----:----|----:----|----:----|----:----|
2201 CCGACCCGTCACAACCGAACAACAGTTCACTCCCACCCCTTAGACCTTTATTTGAGATGGCAGTGTAGTCTTTTGTAAACAGATCCGTACTGTCACATTTACTGGACGCTCTACGGGAACAAAAAGTGACCGAGATATGGTCGTTTTCCGAGGTCTGTGAACGGTCGTGGTGAGTGTAGTTCGTTGTGACATGTCGGACA 2400
     P  S  P  L  T  P  K  N  D  L  S  P  P  S  D  P  F  L  S  *  R  *  M  L  F  C  K  D  L  C  S  L  T  F  S  R  R  S  A  R  T  K  *  Q  S  *  V  L  L  L  S  W  V  S  A  L  V  V  *  M  L  C  C  Q  V  A  Q    F6
       A  P  C  H  Q  S  T  T  L  H  P  H  P  I  Q  F  Y  V  R  G  D  C  *  F  V  N  T  *  A  H  C  H  L  H  G  A  L  H  G  Q  K  E  S  A  R  Y  W  C  F  A  G  S  V  Q  W  C  W  E  C  *  A  V  S  Y  L  R    F5
      P  Q  A  T  N  A  Q  Q  *  T  L  T  P  F  R  S  I  F  E  V  T  V  D  S  F  M  Q  R  P  M  V  T  Y  I  V  Q  S  I  G  K  N  K  V  P  E  I  G  A  F  P  E  L  C  K  G  A  G  S  V  D  L  L  V  T  C  G  T    F4
```

```
      G  E  A  G  H  N  C  R  T  C  T  Y  D  E  N  A  I  G  *  F  M  A  S  S  G  *  R  G  K  K  G  S  S  S  *  *  V  L  V  K  N  V  Y  D  C  P  S  S  E  G  M  S  T  G  *  N  C  P  Y  W  G  L  N  L  G  C  *   F1
       G  R  L  A  T  I  A  A  P  V  R  M  M  K  M  P  *  G  D  S  W  H  P  R  D  E  G  A  K  R  G  R  R  R  D  K  Y  *  *  K  M  F  M  I  V  L  A  A  K  E  C  P  Q  G  E  I  V  L  T  G  D  *  T  W  A  A  R    F2
        G  G  W  P  Q  L  P  H  L  Y  V  *  *  K  C  H  R  V  I  H  G  I  L  G  M  K  G  Q  K  G  V  V  V  V  I  S  I  S  K  K  C  L  *  L  S  *  Q  R  R  N  V  H  R  V  K  L  S  L  L  G  T  E  P  G  L  L   F3
2401 GGGGAGGCTGGCCACAATTGCCGCACCTGTACGTATGATGAAAATGCCATAGGGTGATTCATGGCATCCTCGGGATGAAGGGGCAAAAAGGGGTCGTCGTCGTGATAAGTATTAGTAAAAAATGTTTATGATTGTCCTAGCAGCGAAGGAATGTCCACAGGGTGAAATTGTCCTTACTGGGGACTGAACTGGGCTGCTA 2600
     ----:----|----:----|----:----|----:----|----:----|----:----|----:----|----:----|----:----|----:----|----:----|----:----|----:----|----:----|----:----|----:----|----:----|----:----|----:----|----:----|
2401 CCCCTCCGACCGGTGTTAACGGCGTGGACATGCATACTACTTTTACGGTATCCCACTAAGTACCGTAGGAGCCCTACTTCCCCGTTTTTCCCCAGCAGCAGCACTATTCATAATCATTTTTACAAATACTAACAGGATCGTCGCTTCCTTACAGGTGTCCCACTTTAACAGGAATGACCCCTGACTTGGACCCGACGAT 2600
      P  S  A  P  W  L  Q  R  V  Q  V  Y  S  S  F  A  M  P  H  N  M  A  D  E  P  H  L  P  L  F  P  D  D  D  H  Y  T  N  T  F  F  T  *  S  Q  G  L  L  S  P  I  D  V  P  H  F  Q  G  *  Q  P  S  F  R  P  Q  *   F6
     H  P  P  Q  G  C  N  G  C  R  Y  T  H  H  F  H  W  L  T  I  *  P  M  R  P  I  F  P  C  F  P  T  T  T  T  I  L  I  L  F  H  K  H  N  D  *  C  R  L  F  T  W  L  T  F  N  D  K  S  P  V  S  G  P  S  S    F5
       P  L  S  A  V  I  A  A  G  T  R  I  I  F  I  G  Y  P  S  E  H  C  G  R  S  S  P  A  F  L  P  R  R  R  S  L  Y  *  Y  F  I  N  I  I  T  R  A  A  F  S  H  G  C  P  S  I  T  R  V  P  S  Q  V  Q  A  A    F4

       A  K  H  N  H  F  G  K  T  N  R  L  I  Q  N  Y  G  T  A  E  V  K  *  L  S  C  I  F  L  V  M  Y  E  N  I  H  V  V  P  Q  R  E  L  R  R  V  M  L  F  S  L  F  F  S  A  L  C  F  L  R  F  A  V  M  T  *  E   F1
        P  S  T  I  I  L  V  K  L  T  D  S  F  K  T  M  E  Q  Q  K  *  N  D  C  L  V  F  S  W  *  C  M  K  I  Y  M  L  Y  L  K  E  N  C  V  E  L  C  Y  F  L  C  F  F  L  P  F  V  F  L  G  L  Q  L  *  H  R    F2
      G  Q  A  Q  S  F  W  *  N  *  Q  T  H  S  K  L  W  N  S  R  S  K  M  I  V  L  Y  F  P  G  D  V  *  K  Y  T  C  C  T  S  K  R  I  A  *  S  Y  A  I  F  F  V  F  F  C  P  L  F  S  *  V  C  S  Y  D  I  G   F3
2601 GGCCAAGCACAATCATTTTGGTAAAACTAACAGACTCATTCAAAACTATGGAACAGCAGAAGTAAAATGATTGTCTTGTATTTTCCTGGTGATGTATGAAAATATACATGTTGTACCTCAAAGAGAATTGCGTAGAGTTATGCTATTTTCTTTGTTTTTTTCTGCCCTTTGTTTTCTTAGGTTTGCAGTTATGACATAGG 2800
     ----:----|----:----|----:----|----:----|----:----|----:----|----:----|----:----|----:----|----:----|----:----|----:----|----:----|----:----|----:----|----:----|----:----|----:----|----:----|----:----|
2601 CCGGTTCGTGTTAGTAAAACCATTTTGATTGTCTGAGTAAGTTTTGATACCTTGTCGTCTTCATTTTACTAACAGAACATAAAAGGACCACTACATACTTTTATATGTACAACATGGAGTTTCTCTTAACGCATCTCAATACGATAAAAGAAACAAAAAAAGACGGGAAACAAAAGAATCCAAACGTCAATACTGTATCC 2800
      A  L  C  L  *  K  P  L  V  L  L  S  M  *  F  *  P  V  A  S  T  F  H  N  D  Q  I  K  R  T  I  Y  S  F  I  C  T  T  G  *  L  S  N  R  L  T  I  S  N  E  K  N  K  E  A  R  Q  K  R  L  N  A  T  I  V  Y    F6
     P  W  A  C  D  N  Q  Y  F  *  C  V  *  E  F  S  H  F  L  L  L  L  I  I  T  K  Y  K  G  P  S  T  H  F  Y  V  H  Q  V  E  F  L  I  A  Y  L  *  A  I  K  K  T  K  K  Q  G  K  N  E  *  T  Q  L  *  S  M  P   F5
     L  G  L  V  I  M  K  T  F  S  V  S  E  N  L  V  I  S  C  C  F  Y  F  S  Q  R  T  N  E  Q  H  H  I  F  I  Y  M  N  Y  R  L  S  F  Q  T  S  N  H  *  K  R  Q  K  K  R  G  K  T  K  K  P  K  C  N  H  C  L    F4

       V  N  V  V  K  F  P  L  I  *  V  S  E  F  F  C  T  A  D  M  E  Q  F  V  T  S  H  T  L  Q  R  S  D  K  F  T  W  S  I  L  G  V  N  H  T  S  F  S  F  A  P  S  W  V  H  F  L  C  G  Q  C  K  Q  M  L  I    F1
      K  S  M  L  S  S  F  P  *  S  E  C  L  N  S  S  V  Q  Q  T  W  N  N  L  S  H  L  T  H  C  K  D  L  T  S  S  P  G  V  Y  S  E  S  T  T  Q  V  S  A  S  H  L  P  G  S  I  F  F  V  V  S  V  N  R  C  *  F   F2
       S  Q  C  C  Q  V  S  L  D  L  S  V  *  I  L  L  Y  S  R  H  G  T  I  C  H  I  S  H  T  A  K  I  *  Q  V  H  L  E  Y  T  R  S  Q  P  H  K  F  Q  L  R  T  F  L  G  P  F  S  L  W  S  V  *  T  D  V  D  L   F3
2801 AAGTCAATGTTGTCAAGTTTCCCTTGATCTGAGTGTCTGAATTCTTCTGTACAGCAGACATGGAACAATTTGTCACATCTCACACACTGCAAAGATCTGACAAGTTCACCTGGAGTATACTCGGAGTCAACCACACAAGTTTCAGCTTCGCACCTTCCTGGGTCCATTTTCTTTGTGGTCAGTGTAAACAGATGTTGATT 3000
     ----:----|----:----|----:----|----:----|----:----|----:----|----:----|----:----|----:----|----:----|----:----|----:----|----:----|----:----|----:----|----:----|----:----|----:----|----:----|----:----|
2801 TTCAGTTACAACAGTTCAAAGGGAACTAGACTCACAGACTTAAGAAGACATGTCGTCTGTACCTTGTTAAACAGTGTAGAGTGTGACGTTTCTAGACTGTTCAAGTGGACCTCATATGAGCCTCAGTTGGTGTGTTCAAAGTCGAAGCGTGGAAGGACCCAGGTAAAAGAAACACCAGTCACATTTGTCTACAACTAA 3000
      S  T  L  T  T  L  N  G  K  I  Q  T  D  S  N  K  Q  V  A  S  M  S  C  N  T  V  D  *  V  S  C  L  D  S  L  N  V  Q  L  I  S  P  T  L  W  V  L  K  L  K  A  G  E  Q  T  W  K  R  Q  P  *  H  L  C  I  N  I   F6
      L  *  H  Q  *  T  E  R  S  R  L  T  Q  I  R  R  Y  L  L  C  P  V  I  Q  *  M  E  C  V  A  F  I  Q  C  T  *  R  S  Y  V  R  L  *  G  C  L  N  *  S  R  V  K  R  P  G  N  E  K  H  D  T  Y  V  S  T  S    F5
      F  D  I  N  D  L  K  G  Q  D  S  H  R  F  E  E  T  C  C  V  H  F  L  K  D  C  R  V  C  Q  L  S  R  V  L  E  G  P  T  Y  E  S  D  V  V  C  T  E  A  E  C  R  G  P  D  M  K  K  T  T  L  T  F  L  H  Q  N    F4

      C  T  T  M  K  K  Y  R  *  T  V  Q  R  D  G  S  V  A  G  H  F  K  S  W  R  C  S  H  *  S  D  R  C  R  G  Y  V  Y  N  K  I  L  S  T  G  N  S  R  M  Y  F  I  V  N  L  L  I  T  P  I  Y  Q  Q  F  T  G  T   F1
       A  P  P  *  K  N  T  D  E  L  S  K  G  M  E  V  W  L  A  I  S  N  P  G  G  A  V  I  K  V  T  D  A  V  A  M  F  T  I  K  Y  C  P  L  G  T  V  E  C  I  S  L  S  T  C  *  *  P  P  Y  T  N  S  S  L  G  L    F2
        H  H  E  K  I  Q  M  N  C  P  K  G  W  K  C  G  W  P  F  Q  I  L  E  V  Q  S  L  K  *  Q  M  P  W  L  C  L  Q  *  N  T  V  H  W  E  Q  *  N  V  F  H  C  Q  L  A  D  N  P  H  I  P  T  V  H  W  D   F3
3001 TGCACCACCATGAAAAAATACAGATGAACTGTCCAAAGGGATGGAAGTGTGGCTGGCCATTTCAAATCCTGGAGGTGCAGTCATTAAAGTGACAGATGCCGTGGCTATGTTTACAATAAAATACTGTCCACTGGGAACAGTAGAATGTATTTCATTGTCAACTTGCTGATAACCCCCATATACCAACAGTTCACTGGGAC 3200
     ----:----|----:----|----:----|----:----|----:----|----:----|----:----|----:----|----:----|----:----|----:----|----:----|----:----|----:----|----:----|----:----|----:----|----:----|----:----|----:----|
3001 ACGTGGTGGTACTTTTTTATGTCTACTTGACAGGTTTCCCTACCTTCACACCGACCGGTAAAGTTTAGGACCTCCACGTCAGTAATTTCACTGTCTACGGCACCGATACAAATGTATTTTATGACAGGTGACCCTTGTCATCTTACATAAAGTAACAGTTGAACGACTATTGGGGGTATATGGTTGTCAAGTGACCCTG 3200
      Q  V  V  M  F  F  Y  L  H  V  T  W  L  S  P  L  T  A  P  W  K  L  D  Q  L  H  L  *  *  L  S  L  H  R  P  *  T  *  L  L  I  S  D  V  P  F  L  L  I  Y  K  M  T  L  K  S  I  V  G  M  Y  W  C  N  V  P  V   F6
     K  C  W  W  S  F  I  C  I  F  Q  G  F  P  H  F  H  P  Q  G  N  *  I  R  S  T  C  D  N  F  H  C  I  G  H  S  H  K  C  Y  F  V  T  W  Q  S  C  Y  F  T  N  *  Q  *  S  A  S  L  G  W  I  G  V  T  *  Q  S   F5
       A  G  G  H  F  F  V  S  S  S  D  L  P  I  S  T  H  S  A  M  E  F  G  P  P  A  T  M  L  T  V  S  A  T  A  I  N  V  I  F  Y  Q  G  S  P  V  T  S  H  I  E  N  D  V  Q  Q  Y  G  G  Y  V  L  L  E  S  P    F4

       A  D  I  C  S  M  A  R  E  K  N  V  Y  Q  T  T  S  K  C  Q  L  Y  F  T  Y  D  C  P  Y  A  I  L  T  F  L  W  Q  V  T  L  N  N  G  N  I  Q  N  G  H  S  S  D  H  S  F  I  V  L  L  Y  L  *  Q  A  P  *  V   F1
        Q  T  F  V  V  W  P  E  R  R  M  F  T  K  P  P  A  N  V  S  C  I  L  L  M  T  A  L  M  P  F  *  P  S  C  G  K  *  H  *  I  M  E  T  S  R  M  D  I  L  L  I  T  V  L  L  S  S  C  T  C  N  R  P  P  K    F2
      C  R  H  L  *  Y  G  Q  R  E  E  C  L  P  N  H  Q  Q  M  S  V  V  F  Y  L  *  L  P  L  C  H  F  D  L  P  V  A  S  D  T  E  *  W  K  H  P  E  W  T  F  F  *  S  Q  F  Y  C  P  L  V  P  V  T  G  P  L  S   F3
3201 TGCAGACATTTGTAGTATGGCCAGAGAGAAGAATGTTTACCAAACCACCAGCAAATGTCAGTTGTATTTTACTTATGACTGCCCTTATGCCATTTTGACCTTCCTGTGGCAAGTGACACTGAATAATGGAAACATCCAGAATGGACATTCTTCTGATCACAGTTTTATTGTCCTCTTGTACCTGTAACAGGCCCCCTAAG 3400
     ----:----|----:----|----:----|----:----|----:----|----:----|----:----|----:----|----:----|----:----|----:----|----:----|----:----|----:----|----:----|----:----|----:----|----:----|----:----|----:----|
3201 ACGTCTGTAAACATCATACCGGTCTCTCTTCTTACAAATGGTTTGGTGGTCGTTTACAGTCAACATAAAATGAATACTGACGGGAATACGGTAAAACTGGAAGGACACCGTTCACTGTGACTTATTACCTTTGTAGGTCTTACCTGTAAGAAGACTAGTGTCAAAATAACAGGAGAACATGGACATTGTCCGGGGGATTC 3400
      A  S  M  Q  L  I  A  L  S  F  F  T  *  W  V  V  L  L  H  *  N  Y  K  V  *  S  Q  G  *  A  M  K  V  K  R  H  C  T  V  S  F  L  P  F  M  W  F  P  C  E  E  S  *  L  K  I  T  R  K  Y  R  Y  C  A  G  *   F6
      Q  L  C  K  Y  Y  P  W  L  S  S  H  K  G  F  W  W  C  I  D  T  T  N  *  K  H  S  G  K  H  W  K  S  R  G  T  A  L  S  V  S  Y  H  F  C  G  S  H  V  N  K  Q  D  C  N  *  Q  G  R  T  G  T  V  P  G  R  L   F5
      S  C  V  N  T  T  H  G  S  L  L  I  N  V  L  G  G  A  F  T  L  Q  I  K  S  I  V  A  R  I  G  N  Q  G  E  Q  P  L  H  C  Q  I  S  V  D  L  I  S  M  R  R  I  V  T  H  K  N  D  E  Q  V  Q  L  L  G  G  L   F4

       N  N  C  A  V  F  N  F  I  S  S  M  E  S  N  G  R  E  I  L  L  S  L  D  Y  Y  L  L  P  V  V  F  L  H  I  *  P  V  H  F  T  I  N  S  C  M  I  S  S  L  L  H  T  S  H  S  P  I  Q  Y  C  S  N  L  L  A   F1
      *  T  T  V  Q  S  L  I  S  F  P  V  W  K  A  T  G  G  R  S  C  C  P  W  I  I  I  S  C  Q  W  Y  F  S  I  S  S  Q  Y  I  L  P  S  I  V  A  *  F  P  P  F  S  T  H  P  T  P  P  Y  N  I  A  P  T  S  L  P   F2
      K  Q  L  C  S  L  *  F  H  F  Q  Y  G  K  Q  R  E  G  D  P  V  V  L  G  L  L  S  L  A  S  G  I  S  P  Y  L  A  S  T  F  Y  H  Q  *  L  H  D  F  L  P  S  P  H  I  P  L  H  T  I  L  L  Q  P  P  C  P   F3
3401 TAAACAACTGTGCAGTCTTTAATTTCATTTCCAGTATGGAAAGCAACGGGAGGGAGATCCTGTTGTCCTTGGATTATTATCTTCTTCCAGTGGTATTTCTCCATATCTAGCCAGTACATTTTACCATCAATAGTTGCATGATTTCCTCCCTTCTCCACACATCCCACTCCCCCATACAATATTGCTCCAACCTCCTTGCC 3600
     ----:----|----:----|----:----|----:----|----:----|----:----|----:----|----:----|----:----|----:----|----:----|----:----|----:----|----:----|----:----|----:----|----:----|----:----|----:----|----:----|
3401 ATTTGTTGACACGTCAGAAATTAAAGTAAAGGTCATACCTTTCGTTGCCCTCCCTCTAGGACAACAGGAACCTAATAATAGAGAACGGTCACCATAAAGAGGTATAGATCGGTCATGTAAAATGGTAGTTATCAACGTACTAAAGGAGGGAAGAGGTGTGTAGGGTGAGGGGGTATGTTATAACGAGGTTGGAGGAACGG 3600
      T  F  L  Q  A  T  K  L  K  M  E  L  I  S  L  L  P  L  S  I  R  N  D  K  S  *  *  R  K  G  T  T  N  R  W  I  *  G  T  C  K  V  M  L  L  Q  M  I  E  E  R  R  W  V  D  W  E  G  M  C  Y  Q  E  L  R  R  A   F6
      L  C  S  H  L  R  *  N  *  K  W  Y  P  F  C  R  S  P  S  G  T  T  R  P  N  N  D  R  A  L  P  I  E  G  Y  R  A  L  V  N  *  W  *  Y  N  C  S  K  R  G  E  G  C  M  G  S  G  W  V  I  N  S  W  G  G  Q   F5
      Y  V  V  T  C  D  K  I  E  N  G  T  H  F  A  V  P  P  L  D  Q  Q  G  Q  I  I  E  Q  W  H  Y  K  E  M  D  L  W  Y  M  K  G  D  I  T  A  H  N  G  G  K  E  V  C  G  V  G  G  Y  L  I  A  G  V  E  K  G   F4

      L  L  V  H  *  S  K  G  M  S  I  P  W  T  R  N  T  S  S  L  K  R  H  C  I  R  C  P  Y  *  F  G  I  E  T  P  A  K  I  F  K  Y  N  *  I  I  M  R  Y  H  G  Y  V  W  I  N  A  S  P  N  V  D  R  C  F  H  R   F1
       S  L  F  I  K  V  R  E  C  P  Y  L  G  L  G  T  L  P  V  *  R  D  T  V  S  D  V  P  I  S  L  A  L  K  P  L  P  K  F  S  S  T  I  K  S  L  *  G  T  M  A  M  F  G  S  M  P  P  Q  M  *  T  D  V  F  L   F2
        P  C  S  L  K  *  G  N  V  H  T  L  D  *  E  H  F  Q  S  E  E  T  L  Y  P  M  S  L  L  V  W  H  *  N  P  C  Q  N  F  Q  V  Q  L  N  Y  E  V  P  W  L  C  L  D  Q  C  L  P  K  C  R  Q  M  F  S  P   F3
3601 CTCCTTGTTCATTAAAGTAAGGGAATGTCCATACCTTGGACTAGGAACACTTCCAGTCTGAAGAGACACTGTATCCGATGTCCCTATTAGTTTGGCATTGAAACCCCTGCCAAAATTTTCAAGTACAATTAAATCATTATGAGGTACCATGGCTATGTTTGGATCAATGCCTCCCCAAATGTAGACAGATGTTTTCACCG 3800
     ----:----|----:----|----:----|----:----|----:----|----:----|----:----|----:----|----:----|----:----|----:----|----:----|----:----|----:----|----:----|----:----|----:----|----:----|----:----|----:----|
3601 GAGGAACAAGTAATTTCATTCCCTTACAGGTATGGAACCTGATCCTTGTGAAGGTCAGACTTCTCTGTGACATAGGCTACAGGGATAATCAAACCGTAACTTTGGGGACGGTTTTAAAAGTTCATGTTAATTTAGTAATACTCCATGGTACCGATACAAACCTAGTTACGGAGGGGTTTACATCTGTCTACAAAAGTGGC 3800
      R  R  T  *  *  L  P  I  D  M  G  Q  V  L  F  V  E  L  R  F  L  C  Q  I  R  H  G  *  *  N  P  M  S  V  G  A  L  I  K  L  Y  L  *  I  M  I  L  Y  W  P  *  T  Q  I  L  A  E  G  F  T  S  L  H  K  *  R   F6
      G  G  Q  E  N  F  Y  P  F  T  W  V  K  S  *  S  C  K  W  D  S  S  V  S  Y  G  I  D  R  N  T  Q  C  Q  F  G  Q  W  F  K  *  T  C  N  F  *  *  S  T  G  H  S  H  K  S  *  H  R  G  L  H  L  C  I  N  E  G   F5
       E  K  N  M  L  T  L  S  H  G  Y  R  P  S  P  V  S  G  T  Q  L  S  V  T  D  S  T  G  I  L  K  A  N  F  G  R  G  F  N  E  L  V  I  L  D  N  H  P  V  M  A  I  N  P  D  I  G  G  W  I  Y  V  S  T  K  V   F4

       S  S  T  S  N  R  T  Y  P  P  N  *  H  I  P  S  S  C  V  E  Y  Y  V  I  S  R  M  N  E  W  S  H  T  Y  L  R  T  S  A  F  F  F  C  H  L  Q  H  L  N  V  T  L  L  Y  M  K  S  V  T  K  Y  R  C  S  R  R  R  E   F1
        A  A  L  A  T  E  P  I  L  L  T  D  T  S  R  V  L  V  W  N  T  M  L  A  E  *  M  N  G  L  I  L  I  C  A  P  V  H  F  F  S  V  T  C  N  T  L  M  S  L  S  C  I  *  K  V  *  L  N  I  D  A  V  V  V  V   F2
      Q  Q  H  *  Q  Q  N  L  S  S  *  L  T  H  P  E  F  L  C  G  I  L  C  *  P  N  E  *  M  V  S  Y  L  S  A  H  Q  C  I  F  F  L  S  P  A  T  P  *  C  H  S  P  V  Y  E  K  C  N  *  I  S  M  Q  S  S  S  *   F3
3801 CAGCAGCACTAGCAACAGAACCTATCCTCCTAACTGACACATCCCGAGTTCTTGTGTGGAATACTATGTTAGCCGAATGAATGAATGGTCTCATACTTATCTGCGCACCAGTGCATTTTTTTCTGTCACCTGCAACACCTTAATGTCACTCTCCTGTATATGAAAAGTGTAACTAAATATCGATGCAGTCGTCGTCGTG 4000
     ----:----|----:----|----:----|----:----|----:----|----:----|----:----|----:----|----:----|----:----|----:----|----:----|----:----|----:----|----:----|----:----|----:----|----:----|----:----|----:----|
3801 GTCGTCGTGATCGTTGTCTTGGATAGGAGGATTGACTGTGTAGGGCTCAAGAACACACCTTATGATACAATCGGCTTACTTACTTACCAGAGTATGAATAGACGCGTGGTCACGTAAAAAAAGACAGTGGACGTTGTGGAATTACAGTGAGAGGACATATACTTTTCACATTGATTTATAGCTACGTCAGCAGCAGCAC 4000
      L  L  V  L  L  L  V  *  G  G  L  Q  C  M  G  L  E  Q  T  S  Y  *  T  L  R  I  F  S  H  D  *  V  *  R  R  V  L  A  N  K  K  Q  *  R  C  C  R  L  T  V  R  R  Y  I  F  L  T  V  L  Y  R  H  L  R  R  R   F6
      C  C  C  *  C  C  F  R  D  E  *  S  V  C  G  S  N  K  H  P  I  S  H  *  G  F  S  H  I  T  E  Y  K  D  A  C  W  H  M  K  K  R  D  G  A  V  G  *  H  *  E  G  T  Y  S  F  H  L  *  I  D  I  C  D  D  D  H   F5
      A  A  A  S  A  V  S  G  I  R  R  V  S  V  D  R  T  R  T  H  F  V  I  N  A  S  H  I  F  P  R  M  S  I  Q  A  G  T  C  K  K  E  T  V  Q  L  V  K  I  D  S  E  Q  I  H  F  T  Y  S  F  I  S  A  T  T  T   F4

       W  H  S  W  M  H  A  A  C  N  K  H  I  H  N  T  T  L  Y  I  I  C  Q  C  I  C  S  S  *  S  R  R  K  F  R  V  I  S  R  P  N  *  M  A  L  P  *  K  Q  P  Q  *  A  D  A  I  C  H  R  F  L  R  H  L  F  T   F1
      S  G  I  L  G  C  M  P  P  V  T  N  T  S  T  I  R  P  C  T  S  S  V  S  A  Y  A  V  R  K  A  G  V  S  S  A  S  F  L  D  Q  I  K  W  P  F  P  E  N  S  P  S  E  L  M  Q  S  V  I  D  F  F  V  T  F  L  R   F2
       V  A  F  L  D  A  C  R  L  *  Q  T  H  P  Q  Y  D  P  V  H  H  L  S  V  H  M  Q  F  V  K  Q  A  *  V  P  R  H  F  *  T  K  L  N  G  P  S  L  K  T  A  P  V  S  *  C  N  L  S  S  I  S  S  S  P  F  Y  D   F3
4001 AGTGGCATTCTTGGATGCATGCCGCCTGTAACAAACACATCCACAATACGACCCTGTACATCATCTGTCAGTGCATATGCAGTTCGTAAAGCAGGCGTAAGTTCCGCGTCATTTCTAGACCAAATTAAATGGCCCTTCCCTGAAAACAGCCCCAGTGAGCTGATGCAATCTGTCATCGATTTCTTCGTCACCTTTTTACG 4200
     ----:----|----:----|----:----|----:----|----:----|----:----|----:----|----:----|----:----|----:----|----:----|----:----|----:----|----:----|----:----|----:----|----:----|----:----|----:----|----:----|
4001 TCACCGTAAGAACCTACGTACGGCGGACATTGTTTGTGTAGGTGTTATGCTGGGACATGTAGTAGACAGTCACGTATACGTCAAGCATTTCGTCCGCATTCAAGGCGCAGTAAAGATCTGGTTTAATTTACCGGGAAGGGACTTTTGTCGGGGTCACTCGACTACGTTAGACAGTAGCTAAAGAAGCAGTGGAAAAATGC 4200
      S  H  C  E  Q  I  C  A  A  Q  L  L  C  M  W  L  V  V  R  Y  M  W  N  Q  *  H  M  H  L  E  Y  L  L  R  N  R  T  M  E  L  G  F  *  I  A  R  G  Q  F  C  G  W  H  A  S  A  I  *  R  N  R  R  *  R  K  V   F6
      T  A  N  K  S  A  H  R  R  Y  C  V  C  G  C  Y  S  G  T  C  *  R  D  T  C  I  C  N  T  F  C  A  Y  T  G  R  *  K  *  V  L  N  F  P  G  E  R  F  V  A  G  T  L  Q  H  L  R  D  D  I  E  E  D  G  K  *   F5
      L  P  M  R  P  H  M  G  G  T  V  F  V  D  V  I  R  G  Q  V  D  D  T  L  A  Y  A  T  R  L  A  P  T  L  E  A  D  N  R  S  W  I  L  H  G  K  G  S  F  L  G  L  S  S  I  C  D  T  M  S  K  K  T  V  K  K  R   F4

      I  L  Y  S  L  *  D  G  K  K  P  C  I  S  R  K  I  F  A  I  G  N  Q  A  *  S  Q  K  V  Q  A  *  R  L  K  S  G  V  G  S     F1
       F  C  T  A  F  R  M  E  K  N  R  A  F  R  G  K  F  S  R  L  A  I  K  L  E  V  R  R  F  K  L  E  D  *  S  Q  G  L  V  Q     F2
        F  V  Q  P  L  G  W  K  K  T  V  H  F  E  E  N  F  R  D  W  Q  S  S  L  K  S  E  G  S  S  L  K  I  E  V  R  G  W  F  K    F3
4201 ATTTGTACAGCCTTTAGGATGGAAAAAAACCGTGCATTTCGAGGAAAATTTTCGCGATTGGCAATCAAGCTTGAAGTCAGAAGGTTCAAGCTTGAAGATTGAAGTCAGGGGTTGGTTCA
     ----:----|----:----|----:----|----:----|----:----|----:----|----:----|----:----|----:----|----:----|----:----|----:----|
4201 TAAACATGTCGGAAATCCTACCTTTTTTTGGCACGTAAAGCTCCTTTTAAAAGCGCTAACCGTTAGTTCGAACTTCAGTCTTCCAAGTTCGAACTTCTAACTTCAGTCCCCAACCAAGT
      I  K  Y  L  R  *  S  P  F  F  G  H  M  E  L  F  I  K  A  I  P  L  *  A  Q  L  *  F  T  *  A  Q  L  N  F  D  P  T  P  E     F6
      S  K  T  C  G  K  P  H  F  F  V  T  C  K  S  S  F  K  R  S  Q  C  D  L  K  F  D  S  P  E  L  K  F  I  S  T  L  P  Q  N     F5
     N  Q  V  A  K  L  I  S  F  F  R  A  N  R  P  F  N  E  R  N  A  I  L  S  S  T  L  N  L  S  S  S  Q  L  *  P  N  T  *      F4
```

Matching transcriptomic data (TSA)

RAG1L

>WGS: NMRB01002322.1:102000-107600
>TSA: GFRY01014649.1:beg-end
>TSA: GFRY01014650.1:beg-end

```
               1                                                                                                                                                                                                              200
NMRB01002322.1  CACTGCATCTGCAGTTCCGATTGTCGGACAACCTCCATACCGTATAGGCCTAGGCCTATATACTTTCTCCAATAATTTACGCTTTGTTCGACCAACTAAAAACACTTACACGATCACGTACAGAACTGCGTCAGTAGGACGCCTCGCATCATGAACAACATGATAAAAGACAGCTGATGATTGTCATGCAGGATGTGGAA
GFRY01014649.1
GFRY01014650.1
   Consensus    ........................................................................................................................................................................................................

               201                                                                                                                                                                                                            400
NMRB01002322.1  TGTGTGGCGTGTCGCGTTTTGTTTGTTTTGTTTTGTTTCACAGGCGGCCGCGTTGGATCCGTATCCCACTCCCCGCTTTGCCCTTCCTCCAGCCTCTAGTACCACCCCCACGCCCAGGCGGCAGCACTGTACGGTGTCGCTCGTCGTCGTCTCTTATGAAAATCATGAAATTTTACATGAACTCCATGCGAAAGACATA
GFRY01014649.1
GFRY01014650.1                                                                                  CTACTCATGTACTAGGCCTAGCCTACATCATGTA------------------------------------------------------------------------------------------
   Consensus    ...............................................................................c.actc.....ct..gcc...cct.ca.c.t.ta..........................................................................................

               401                                                                                                                                                                                                            600
NMRB01002322.1  CCAATACAGCGCAGCTCAAAAGAGACCTCAGGTATTAATAACTAGACCTGTGCAAAGCTAACATGTTGTTTATTGCTAACGGAAGAGTTGGAAATGACAAAAGAGTAGGTTCAGTCACATGTAAGAAAAGTGTAGATGACGTGGTAATATGTGATCCAAATTCTTTATGCAGTATGGGTTCCTTTGATGTTGATACTTTC
GFRY01014649.1
GFRY01014650.1  --------------------------------------------------------------------------------------------------------------------------------------------------------------------------------------------------------
   Consensus    ........................................................................................................................................................................................................
```

```
601                                                                                                                                                                                                       800
NMRB01002322.1 AACCCTTTATTATCAGATGTTCATTCACCTGTAATCTTTAATTTGTGTAAGTCCACCAAATCTGGTCGGGCTGATGTCCAGGAAAATGAAGATCATCATACGATGAAACCAATTACTGGTGATATCAATTCTTCTCGTCCCACTTGGAAAGCTGAGAAAAGCAATGATTTCATAAGCGTCGTGCGAAAAAACCTGAATAT
GFRY01014649.1
GFRY01014650.1 --------------------------------------------------------------------------------------------------------------------------------------------------------------------------------------------------------
Consensus      ........................................................................................................................................................................................................

801                                                                                                                                                                                                       1000
NMRB01002322.1 TGTTACCAAGGCGTTCAATGAACAACAAGATGTGAACCTAATGGCAGAAGCAATCAGCGAAGTTTTCATAAAGGCCTCTGAAGAGTCATCTCAAATGAGGAGGTCCAATGTGTCAGGTAAAATGAATGAGTCTAGAAACACATGTAGATCTAGGAACAGGAGTTGGTTTGATAGAGACTGCGCAGAAGCTAAGAAACGAT
GFRY01014649.1
GFRY01014650.1 --------------------------------------------------------------------------------------------------------------------------------------------------------------------------------------------------------
Consensus      ........................................................................................................................................................................................................

1001                                                                                                                                                                                                      1200
NMRB01002322.1 TTGAAACTAGTCATAACTTGTACAGAAGAAATAAAACCAATGAGAGGTTTGCAGAAATGAAATGGAGAAGTAAAGAATACAAACGACAACTTAACAGGAGTCACAACAAATACAAGAAAGACCTGGGAAAAAAGTTAAAAAAATCTTAGGAGTTCTAATCCTAAAGATTTCTGGAAACTGCTGACCCCCCCCCCAAAAA
GFRY01014649.1
GFRY01014650.1 --------------------------------------------------------------------------------------------------------------------------------------------------------------------------------------------------------
Consensus      ........................................................................................................................................................................................................

1201                                                                                                                                                                                                      1400
NMRB01002322.1 AAAATCACATGTTAATTGTGAAGTAAGCATCGAGTTGTTCAAGAAACACTTTGCTAAACTAAATACTGCTCCTGATGATCATTATCTTCCTGATTCTGATCAAAACCTGACACAGCACTTTAGTGAAAATGGGAACGCTAGCTTGAATGATCCAATAACTGAGAAAGAAATAGCTAAAGCTATCAAAGCCTTGAAAAAT
GFRY01014649.1
GFRY01014650.1 --------------------------------------------------------------------------------------------------------------------------------------------------------------------------------------------------------
Consensus      ........................................................................................................................................................................................................

1401                                                                                                                                                                                                      1600
NMRB01002322.1 GGGAAGGCCTTTGGTTGTGACAAGATCTTGAACGAACATTTAAAAACCTCCACGGACACCATGACGCCATACTTTCTTCAGCTTTTCAACGCAGTCCTCCTGACTGGAGAAATCCCCAAATCTTGGTCATATGGGACGATCAAGCCGATATACAAAAATAAGGGTGATAACAAAAACCCTGACAATTACAGGGGCATTAC
GFRY01014649.1
GFRY01014650.1 --------------------------------------------------------------------------------------------------------------------------------------------------------------------------------------------------------
Consensus      ........................................................................................................................................................................................................

1601                                                                                                                                                                                                      1800
NMRB01002322.1 AATCTTAAGCTGCCTTGGGAAAGTTTTCACGTCCATTATCAATGATAGGCTGAATGCCTTTGCTTCTGACGTTAACCTGATTGGGAAAGAGCAAGCAGGTTTCAGATCAGGTTTTTGTACTGTAGACCATATGATTACCATGAAGTTTTTGCTTGATATATACCTCTCAAAAGGATTAAAAATTTTCTGTGCCTTCATTG
GFRY01014649.1
GFRY01014650.1 --------------------------------------------------------------------------------------------------------------------------------------------------------------------------------------------------------
Consensus      ........................................................................................................................................................................................................

1801                                                                                                                                                                                                      2000
NMRB01002322.1 ATTATCGGAAGGCCTTTGACACTGTGTGGAGAGCTGGGGGGGGGGGGGGAGGCTGAATACCTTTGCTTCTGACGTTAACCTGATTGGGAAAGAGCAAGCAGGTTTCAGATCAGGTTATTGTACTGTAGACCATATGATTACCCACACCGAAACCTGTAGTGTAAATAAAACCCGCCCTGGGCGCCGCCATAATTAGATGC
GFRY01014649.1
GFRY01014650.1 ------------------------------------------------------------------------------------------------------------------------------------TGTAGCTACTCACACCGAAACCTGTAGTGTAAATAAAACCCGCCCTGGGCGCCGCCATAATTAGATGC
Consensus      ....................................................................................................................................t.t...tac.cacaccgaaacctgtagtgtaaataaaacccgccctgggcgccgccataattagatgc

2001                                                                                                                                                                                                      2200
NMRB01002322.1 GTCGCGCTTGTCACGTGACCCACGCTCAACCCTGCGTGGAGACTACCACCCCTCATTTACAGACCTCCCAGCGGGTCTCTTTACCCGTAACGCATGCGCAGTTACAATCAGGAAGTGTGCGGTCGACGGAGCTTTGTCTGGCTGTTTATTTCATAAAAATCGGCCTAGAAATGCGATTTTTGTGAATTTTTTTGCGTTTT
GFRY01014649.1
GFRY01014650.1 GTCGCGCTTGTCACGTGACCCACGCTCAACCCTGCGTGGAGACTACCACCCCTCATTTACAGACCTCCCAGCGGGTCTCTTTACCCGTAACGCATGCGCAGTTACAATCAGGAAGTGTGCGGTCGACGGAGCTTTGTCTGGCTGTTTATTTCATAAAAATCGGCCTAGAAATGCGATTTTTGTGAATTTTTTTGCGTTTT
Consensus      gtcgcgcttgtcacgtgacccacgctcaaccctgcgtggagactaccacccctcatttacagacctcccagcgggtctctttacccgtaacgcatgcgcagttacaatcaggaagtgtgcggtcgacggagctttgtctggctgtttatttcataaaaatcggcctagaaatgcgatttttgtgaatttttttgcgtttt

2201                                                                                                                                                                                                      2400
NMRB01002322.1 TCACGTGAAAATCAGGGCGGAAGGCATCCGTGGTACCCCTAGAAGATTTATCAGGTAACTTTTTTCGGTTTATGTTAAGCAAAATGATTCAAACGCAGATTGAAAGTTGAGATTCCATGCTTCCCGTTGATGTAAAACACTTCATGGTTGCCTTCAGGACAAATTGTTTAACAATTCTTGATTCTGCCAGTCGGGTAGCA
GFRY01014649.1 AATTGTTTAACAATTCTTGATTCTGCCAGTCGGGTAGCA
GFRY01014650.1 TCACGTGAAAATCAGGGCGGAAGGCATCCGTGGTACCCCTAGAAGATTTATCAG--------------------------------------------------------------------------------------------------------------------------------------------------
Consensus      tcacgtgaaaatcagggcggaaggcatccgtggtacccctagaagatttatcag...........................................................................................................aattgtttaacaattcttgattctgccagtcgggtagca

2401                                                                                                                                                                                                      2600
NMRB01002322.1 TGAAATAGGTCTGGTTGGAATGAAAAATTGATTCAAGTGGGTTTAAGATGATATTTCTTGGCAAATATTGCAGATTTATCGCATTTTGTGAATGACCTTGACCTTCTACCACGCTGTATTGATTTTTCCTGAGGAACGAAAGGATTTTGGGCAGTTTTTGATCAGCTGATTGTATGTCAACATCATATTGTGTCCTCAGA
GFRY01014649.1 TGAAATAGGTCTGGTTGGAATGAAAAATTGATTCAAGTGGGTTTAAGATGATATTTCTTGGCAAATATTGCAGATTTATCGCATTTTGTGAATGACCTTGACCTTCTACCACGCTGTATTGATTTTTCCTGAGGAACGAAAGGATTTTGGGCAGTTTTTGATCAGCTGATTGTATGTCAACATCATATTGTGTCCTCAGA
GFRY01014650.1 --------------------------------------------------------------------------------------------------------------------------------------------------------------------------------------------------------
Consensus      tgaaataggtctggttggaatgaaaaattgattcaagtgggtttaagatgatatttcttggcaaatattgcagatttatcgcattttgtgaatgaccttgaccttctaccacgctgtattgatttttcctgaggaacgaaaggattttgggcagtttttgatcagctgattgtatgtcaacatcatattgtgtcctcaga

2601                                                                                                                                                                                                      2800
NMRB01002322.1 TATATGATGAGTTCATTCATGTTGTCACCCCTGGAGCTGTGTTGATCATTGCTGTCCTCAAATATGCTCATATCCACATTTATGTCTGTGGTTAAATAATATGGGACTTCTGTTATCCCTCAGGCTTCCATCAGAAATACAATTTTAATCCAATTCTTTTCTATTTTCAGAAATGTTGTCTGCGATGGATGATTTTCATT
GFRY01014649.1 TATATGATGAGTTCATTCATGTTGTCACCCCTGGAGCTGTGTTGATCATTGCTGTCCTCAAATATGCTCATATCCACATTTATGTCTGTGGTTAAATAATATGGGACTTCTGTTATCCCTCAGGCTTCCGTCAGAAATACAATTTTAATCCAATTCTTTTCTATTTTCAGAAATGTTGTCTGCGATGGATGATTTTCATT
GFRY01014650.1 ------------------------------------------------------------------------------------------------------------------------------------------------------------------------AAATGTTGTCTGCGATGGATGATTTTCATT
Consensus      tatatgatgagttcattcatgttgtcacccctggagctgtgttgatcattgctgtcctcaaatatgctcatatccacatttatgtctgtggttaaataatatgggacttctgttatccctcaggcttcc.tcagaaatacaattttaatccaattcttttctattttcagAAATGTTGTCTGCGATGGATGATTTTCATT

2801                                                                                                                                                                                                      3000
NMRB01002322.1 CAGAAGTCCTCAAACACCTGTGTCGCATTTGTTCTGGTGTCCTTAATTCCAAGGCATGTGGTGCAGCACCAAGACAGAAGAGTTTCCTTTCAGTGGAAGCATGTGCTTCAGCTCTTCTCAGGATCTATGGGATATCAGTGGAAACTGAGGACAACAGTGTATTTCCAACTTCAGTGTGCAACAAGTGCAGAATTAAAATT
GFRY01014649.1 CAGAAGTCCTCAAACACCTGTGTCGCATTTGTTCTGGTGTCCTTAATTCCAAGGCATGTGGTGCAGCACCAAGACAGAAGAGTTTCCTTTCAGTGGAAGCATGTGCTTCAGCTCTTCTCAGGATCTATGGGATATCAGTGGAAACTGAGGACAACAGTGTATTTCCAACTTCAGTGTGCAACAAGTGCAGAATTAAAATT
GFRY01014650.1 CAGAAGTCCTCAAACACCTGTGTCGCATTTGTTCTGGTGTCCTTAATTCCAAGGCATGTGGTGCAGCACCAAGACAGAAGAGTTTCCTTTCAGTGGAAGCATGTGCTTCAGCTCTTCTCAGGATCTATGGGATATCAGTGGAAACTGAGGACAACAGTGTATTTCCAACTTCAGTGTGCAACAAGTGCAGAATTAAAATT
Consensus      CAGAAGTCCTCAAACACCTGTGTCGCATTTGTTCTGGTGTCCTTAATTCCAAGGCATGTGGTGCAGCACCAAGACAGAAGAGTTTCCTTTCAGTGGAAGCATGTGCTTCAGCTCTTCTCAGGATCTATGGGATATCAGTGGAAACTGAGGACAACAGTGTATTTCCAACTTCAGTGTGCAACAAGTGCAGAATTAAAATT

3001                                                                                                                                                                                                      3200
NMRB01002322.1 AGTAGGGAAATGAAAAAGACTGTAATTGAGCAGCAGGTGTTACCTCATTTGTTCCGTCCCCATCAGCATGATGATTTGAACTGTATTTGCCACAAGTTATGCTCAGCTGTCAGTGTTCCATTGCCACCCCCAACCACAGAATCGGTCGCTATTGAACCATCAATTCTGACTATTGAACATAGCTACGTTCATGATATTGG
GFRY01014649.1 AGTAGGGAAATGAAAAAGACTGTAATTGAGCAGCAGGTGTTACCTCATTTGTTCCGTCCCCATCAGCATGATGATTTGAACTGTATTTGCCACAAGTTATGCTCAGCTGTCAGTGTTCCATTGCCACCCCCAACCACAGAATCGGTCGCTATTGAACCATCAATTCTGACTATTGAACATAGCTACGTTCATGATATTGG
GFRY01014650.1 AGTAGGGAAATGAAAAAGACTGTAATTGAGCAGCAGGTGTTACCTCATTTGTTCCGTCCCCATCAGCATGATGATTTGAACTGTATTTGCCACAAGTTATGCTCAGCTGTCAGTGTTCCATTGCCACCCCCAACCACAGAATCGGTCGCTATTGAACCATCAATTCTGACTATTGAACATAGCTACGTTCATGATATTGG
Consensus      AGTAGGGAAATGAAAAAGACTGTAATTGAGCAGCAGGTGTTACCTCATTTGTTCCGTCCCCATCAGCATGATGATTTGAACTGTATTTGCCACAAGTTATGCTCAGCTGTCAGTGTTCCATTGCCACCCCCAACCACAGAATCGGTCGCTATTGAACCATCAATTCTGACTATTGAACATAGCTACGTTCATGATATTGG

3201                                                                                                                                                                                                      3400
NMRB01002322.1 TGCTGCTGCAAAACCAAGTGCACCTTCAACACCTGAGGTTCGTAGTAAAAAAAGTTTGAAGGAGTTGAACCGACAGTACATAAAGAGAACCCGTTTGAAAGACCTTTTCTCAAACCTTCATGACTTTTGTAAAGAGAAAAAAGAGGATGTGGTAGATGTTACTTTTAGTCTCCTAATACAGGAACTTATTGATTCAGATC
GFRY01014649.1 TGCTGCTGCAAAACCAAGTGCACCTTCAACACCTGAGGTTCGTAGTAAAAAAAGTTTGAAGGAGTTGAACCGACAGTACATAAAGAGAACCCGTTTGAAAGACCTTTTCTCAAACCTTCATGACTTTTGTAAAGAGAAAAAAGAGGATGTGGTAGATGTTACTTTTAGTCTCCTAATACAGGAACTTATTGATTCAGATC
GFRY01014650.1 TGCTGCTGCAAAACCAAGTGCACCTTCAACACCTGAGGTTCGTAGTAAAAAAAGTTTGAAGGAGTTGAACCGACAGTACATAAAGAGAACCCGTTTGAAAGACCTTTTCTCAAACCTTCATGACTTTTGTAAAGAGAAAAAAGAGGATGTGGTAGATGTTACTTTTAGTCTCCTAATACAGGAACTTATTGATTCAGATC
Consensus      TGCTGCTGCAAAACCAAGTGCACCTTCAACACCTGAGGTTCGTAGTAAAAAAAGTTTGAAGGAGTTGAACCGACAGTACATAAAGAGAACCCGTTTGAAAGACCTTTTCTCAAACCTTCATGACTTTTGTAAAGAGAAAAAAGAGGATGTGGTAGATGTTACTTTTAGTCTCCTAATACAGGAACTTATTGATTCAGATC

3401                                                                                                                                                                                                      3600
NMRB01002322.1 GTAAAAAGGAAGCTGATCGTGTCATGGAGATTTGGAGCGGGAGTGACAAATCAAAATTGTCAGTTGAGGAATGTTTAGCCATGAGGGTGAGAAACACACTCTCCAAGACGCACTATGCGCAGATGTATGCAGTTCACAGGGACAAGACCAGTTTGAATTCCCTACAACCAGTGAAACAATTGACATCCATTGAAAACTTA
GFRY01014649.1 GTAAAAAGGAAGCTGATCGTGTCATGGAGATTTGGAGCGGGAGTGACAAATCAAAATTGTCAGTTGAGGAATGTTTAGCCATGAGGGTGAGAAACACACTCTCCAAGACGCACTATGCGCAGATGTATGCAGTTCACAGGGACAAGACCAGTTTGAATTCCCTACAACCAGTGAAACAATTGACATCCATTGAAAACTTA
GFRY01014650.1 GTAAAAAGGAAGCTGATCGTGTCATGGAGATTTGGAGCGGGAGTGACAAATCAAAATTGTCAGTTGAGGAATGTTTAGCCATGAGGGTGAGAAACACACTCTCCAAGACGCACTATGCGCAGATGTATGCAGTTCACAGGGACAAGACCAGTTTGAATTCCCTACAACCAGTGAAACAATTGACATCCATTGAAAACTTA
Consensus      GTAAAAAGGAAGCTGATCGTGTCATGGAGATTTGGAGCGGGAGTGACAAATCAAAATTGTCAGTTGAGGAATGTTTAGCCATGAGGGTGAGAAACACACTCTCCAAGACGCACTATGCGCAGATGTATGCAGTTCACAGGGACAAGACCAGTTTGAATTCCCTACAACCAGTGAAACAATTGACATCCATTGAAAACTTA

3601                                                                                                                                                                                                      3800
NMRB01002322.1 TTTTTCCCAGGTAACTGTGCTTACAAGTTATTCAAAAATGACTGTTTAATTCAGGAACAGGATATATTAAGCAAGGTTGATTGTGAACCAAAGGACATAATGAGAAACTTTGCAAACTTTCCAAGTGATTTTCCATTACCCAATGTGTCAGGTTTGGAGTTCGACTACATATCAGCAGTGGCAAAAACATTGACTGAATT
GFRY01014649.1 TTTTTCCCAGGTAACTGTGCTTACAAGTTATTCAAAAATGACTGTTTAATTCAGGAACAGGATATATTAAGCAAGGTTGATTGTGAACCAAAGGACATAATGAGAAACTTTGCAAACTTTCCAAGTGATTTTCCATTACCCAATGTGTCAGGTTTGGAGTTCGACTACATATCAGCAGTGGCAAAAACATTGACTGAATT
GFRY01014650.1 TTTTTCCCAGGTAACTGTGCTTACAAGTTATTCAAAAATGACTGTTTAATTCAGGAACAGGATATATTAAGCAAGGTTGATTGTGAACCAAAGGACATAATGAGAAACTTTGCAAACTTTCCAAGTGATTTTCCATTACCCAATGTGTCAGGTTTGGAGTTCGACTACATATCAGCAGTGGCAAAAACATTGACTGAATT
Consensus      TTTTTCCCAGGTAACTGTGCTTACAAGTTATTCAAAAATGACTGTTTAATTCAGGAACAGGATATATTAAGCAAGGTTGATTGTGAACCAAAGGACATAATGAGAAACTTTGCAAACTTTCCAAGTGATTTTCCATTACCCAATGTGTCAGGTTTGGAGTTCGACTACATATCAGCAGTGGCAAAAACATTGACTGAATT

3801                                                                                                                                                                                                      4000
NMRB01002322.1 AGAAGAGAGTATCAGTGCTCGGCTAAAAGACTGTGGTCTTCCAATTGACACATCTGTCCACACCATAATTAAAGATGGTGGTGATGGTCTTGGCGACGTGAACATTAAGACTCGTAAGGGCGAATTCTCTTTAACTGACAAAGTCTTTAGGTACTCATTCTGCGTCCTTTCATGTAGTGCAACAGTGAATGAGAAAAAAT
GFRY01014649.1 AGAAGAGAGTATCAGTGCTCGGCTAAAAGACTGTGGTCTTCCAATTGACACATCTGTCCACACCATAATTAAAGATGGTGGTGATGGTCTTGGCGACGTGAACATTAAGACTCGTAAGGGCGAATTCTCTTTAACTGACAAAGTCTTTAGGTACTCATTCTGCGTCCTTTCATGTAGTGCAACAGTGAATGAGAAAAAAT
GFRY01014650.1 AGAAGAGAGTATCAGTGCTCGGCTAAAAGACTGTGGTCTTCCAATTGACACATCTGTCCACACCATAATTAAAGATGGTGGTGATGGTCTTGGCGACGTGAACATTAAGACTCGTAAGGGCGAATTCTCTTTAACTGACAAAGTCTTTAGGTACTCATTCTGCGTCCTTTCATGTAGTGCAACAGTGAATGAGAAAAAAT
Consensus      AGAAGAGAGTATCAGTGCTCGGCTAAAAGACTGTGGTCTTCCAATTGACACATCTGTCCACACCATAATTAAAGATGGTGGTGATGGTCTTGGCGACGTGAACATTAAGACTCGTAAGGGCGAATTCTCTTTAACTGACAAAGTCTTTAGGTACTCATTCTGCGTCCTTTCATGTAGTGCAACAGTGAATGAGAAAAAAT

4001                                                                                                                                                                                                      4200
NMRB01002322.1 ATGAATTGTTCAAGGAAGCATCAGCTAGTTCTGTTGATTGCACCCGCCCGGTAGCTATTTGCCATTGTGATGAAAGCGACAGACCGAGCATTTCTATCACATTGGCTAATATCTCCAAAGCAAGAGATGCGATGCAGAATGGTGTGATGAAGTTGAGGTTTGGTGGTGGACACATTGTACCTGAGAGGTCCCACACACTG
GFRY01014649.1 ATGAATTGTTCAAGGAAGCATCAGCTAGTTCTGTTGATTGCACCCGCCCGGTAGCTATTTGCCATTGTGATGAAAGCGACAGACCGAGCATTTCTATCACATTGGCTAATATCTCCAAAGCAAGAGATGCGATGCAGAATGGTGTGATGAAGTTGAGGTTTGGTGGTGGACACATTGTACCTGAGAGGTCCCACACACTG
GFRY01014650.1 ATGAATTGTTCAAGGAAGCATCAGCTAGTTCTGTTGATTGCACCCGCCCGGTAGCTATTTGCCATTGTGATGAAAGCGACAGACCGAGCATTTCTATCACATTGGCTAATATCTCCAAAGCAAGAGATGCGATGCAGAATGGTGTGATGAAGTTGAGGTTTGGTGGTGGACACATTGTACCTGAGAGGTCCCACACACTG
Consensus      ATGAATTGTTCAAGGAAGCATCAGCTAGTTCTGTTGATTGCACCCGCCCGGTAGCTATTTGCCATTGTGATGAAAGCGACAGACCGAGCATTTCTATCACATTGGCTAATATCTCCAAAGCAAGAGATGCGATGCAGAATGGTGTGATGAAGTTGAGGTTTGGTGGTGGACACATTGTACCTGAGAGGTCCCACACACTG

4201                                                                                                                                                                                                      4400
NMRB01002322.1 CAGTTTCTCTTTACCATGGTTGATGAGAAATTTGAACGGACATCCTCAGGGCTCCAGGGTAGTGGGTCCAGATACCTTTGTACTCTGTGCGAAGCCACCACTGCCAATTGCCAGTGTGATTTGGGTTCATTCACTCGTACAAGAACACATGATCAGACCGTCTCCCTCTACAGGTATCGTTTTGAAAATCCTGAAGAACT
GFRY01014649.1 CAGTTTCTCTTTACCATGGTTGATGAGAAATTTGAACGGACATCCTCAGGGCTCCAGGGTAGTGGGTCCAGATACCTTTGTACTCTGTGCGAAGCCACCACTGCCAATTGCCAGTGTGATTTGGGTTCGTTCACTCGTACAAGAACACATGATCAGACCGTCTCCCTCTACAGGTATCGTTTTGAAAATCCTGAAGAACT
GFRY01014650.1 CAGTTTCTCTTTACCATGGTTGATGAGAAATTTGAACGGACATCCTCAGGGCTCCAGGGTAGTGGGTCCAGATACCTTTGTACTCTGTGCGAAGCCACCACTGCCAATTGCCAGTGTGATTTGGGTTCGTTCACTCGTACAAGAACACATGATCAGACCGTCTCCCTCTACAGGTATCGTTTTGAAAATCCTGAAGAACT
Consensus      CAGTTTCTCTTTACCATGGTTGATGAGAAATTTGAACGGACATCCTCAGGGCTCCAGGGTAGTGGGTCCAGATACCTTTGTACTCTGTGCGAAGCCACCACTGCCAATTGCCAGTGTGATTTGGGTTCgTTCACTCGTACAAGAACACATGATCAGACCGTCTCCCTCTACAGGTATCGTTTTGAAAATCCTGAAGAACT

4401                                                                                                                                                                                                      4600
NMRB01002322.1 AAATGAAAATGACTTGGCTGAGAAATGCAAAGGGGTTAAGGCTATGCCACTGATTTCTTCCGATGCATCTGAACGTTCACTTGATTCTACCCATGCAAATATAAACATGGGGAGATTTTTCAAGAGGATACTTGTAAGGGAAGCGGCTGAAGTGTACGAGTGGACGGAATCAGACAGAACAGATCTCGGCTCCAGCATG
GFRY01014649.1 AAATGAAAATGACTTGGCTGAGAAATGCAAAGGGGTTAAGGCTATGCCACTGATTTCTTCCGATGCATCTGAACGTTCACTTGATTCTACCCATGCAAATATAAACATGGGGAGATTTTTCAAGAGGATACTTGTAAGGGAAGCGGCTGAAGTGTACGAGTGGACGGAATCAGACAGAACAGATCTCGGCTCCAGCATG
GFRY01014650.1 AAATGAAAATGACTTGGCTGAGAAATGCAAAGGGGTTAAGGCTATGCCACTGATTTCTTCCGATGCATCTGAACGTTCACTTGATTCTACCCATGCAAATATAAACATGGGGAGATTTTTCAAGAGGATACTTGTAAGGGAAGCGGCTGAAGTGTACGAGTGGACGGAATCAGACAGAACAGATCTCGGCTCCAGCATG
Consensus      AAATGAAAATGACTTGGCTGAGAAATGCAAAGGGGTTAAGGCTATGCCACTGATTTCTTCCGATGCATCTGAACGTTCACTTGATTCTACCCATGCAAATATAAACATGGGGAGATTTTTCAAGAGGATACTTGTAAGGGAAGCGGCTGAAGTGTACGAGTGGACGGAATCAGACAGAACAGATCTCGGCTCCAGCATG

4601                                                                                                                                                                                                      4800
NMRB01002322.1 TTGAAAGTCTTTAGATGCCATGTTGATAGAGAAATTAGGTATCCAGCCAAAAATGATTATGGTAGGCAATGATGCCAGGAAACTTTTCAATGGAGCAAACCATGATATTGTCACATCACTGATCAAAAATGAGGAAAGGAGGGACTTGTGAAGCACATATTGTCACTGTATTCTAAAATGCATTCAGTGTATTCTTCT
GFRY01014649.1 TTGAAAGTCTTTAGATGCCATGTTGATAGAGAAATTAGGTATCCAGCCAAAAATGATTATGGTAGGCAATGATGCCAGGAAACTTTTCAATGGAGCAAACCATGATATTGTCACATCACTGATCAAAAATGAGGAAAGGAGGGACTTGTGAAGCACATATTGTCACTGTATTCTAAAATGCATTCAGTGTATTCTTCT
GFRY01014650.1 TTGAAAGTCTTTAGATGCCATGTTGATAGAGAAATTAGGTATCCAGCCAAAAATGATTATGGTAGGCAATGATGCCAGGAAACTTTTCAATGGAGCAAACCATGATATTGTCACATCACTGATCAAAAATGAGGAAAGGAGGGACTTGTGAAGCACATATTGTCACTGTATTCTAAAATGCATTCAGTGTATTCTTCT
Consensus      TTGAAAGTCTTTAGATGCCATGTTGATAGAGAAATTAGGTATCCAGCCAAAAATGATTATGGTAGGCAATGATGCCAGGAAACTTTTCAATGGAGCAAACCATGATATTGTCACATCACTGATCAAAAATGAGGAAAGGAGGGACTTGTGAAGCACATATTGTCACTGTATTCTAAAATGCATTCAGTGTATTCTTCT

4801                                                                                                                                                                                                      5000
NMRB01002322.1 ACCTCACCCACAGTAGAGCTTCCAGAGTTGACAGGAAATTACAGAGAAATAGCCTTCCAATTTTCATCTTGTCTTGGTGAACACTTTCCGAGTACATTACCTGGTCGAACTACCTGCATAAGGTGGTGGAGCATGTTGCAGAAATTATCAGTGACCCCCATGGGCTGGGCAGTGTTGGCTTGTTGTCAAGTGAGGTGGG
GFRY01014649.1 ACCTCACCCACAGTAGAGCTTCCAGAGTTGACAGGAAATTACAGAGAAATAGCCTTCCAATTTTCATCTTGTCTTGGTGAACACTTTCCG--TACATTACCTGGTCGAACTACCTGCATAAGGTGGTGGAGCATGTTGCAGAAATTATCAGTGACCCCCATGGGCTGGGCAGTGTTGGCTTGTTGTCAAGTGAGGTGGG
GFRY01014650.1 ACCTCACCCACAGTAGAGCTTCCAGAGTTGACAGGAAATTACAGAGAAATAGCCTTCCAATTTTCATCTTGTCTTGGTGAACACTTTCCG--TACATTACCTGGTCGAACTACCTGCATAAGGTGGTGGAGCATGTTGCAGAAATTATCAGTGACCCCCATGGGCTGGGCAGTGTTGGCTTGTTGTCAAGTGAGGTGGG
Consensus      ACCTCACCCACAGTAGAGCTTCCAGAGTTGACAGGAAATTACAGAGAAATAGCCTTCCAATTTTCATCTTGTCTTGGTGAACACTTTCCG..TACATTACCTGGTCGAACTACCTGCATAAGGTGGTGGAGCATGTTGCAGAAATTATCAGTGACCCCCATGGGCTGGGCAGTGTTGGCTTGTTGTCAAGTGAGGTGGG

5001                                                                                                                                                                                                      5200
NMRB01002322.1 GAATCTGGAAATAAACTCTACCGTCACATCAGAAAACATTTGTCTAGGCATGACAGTGTAAATGACCTGCGAGATGCCCTTGTTTTTCACTGGCTCTATACCAGCAAAAGGCTCCAGACACTTGCCAGCACCACTCACATCAAGCAACACTGTACAGCCTGTGGGGAGGCTGGCCACAATTGCGCGCACCTGTACGTATGA
GFRY01014649.1 GAATCTGGAAATAAACTCTACCGTCACATCAGAAAACATTTGTCTAGGCATGACAGTGTAAATGACCTGCGAGATGCCCTTGTTTTTCACTGGCTCTATACCAGCAAAAGGCTCCAGACACTTGCCAGCACCACTCACATCAAGCAACACTGTACAGCCTGTGGGGAGGCTGGCCACAATTGCGCGCACCTGTACGTATGA
GFRY01014650.1 GAATCTGGAAATAAACTCTACCGTCACATCAGAAAACATTTGTCTAGGCATGACAGTGTAAATGACCTGCGAGATGCCCTTGTTTTTCACTGGCTCTATACCAGCAAAAGGCTCCAGACACTTGCCAGCACCACTCACATCAAGCAACACTGTACAGCCTGTGGGGAGGCTGGCCACAATTGCGCGCACCTGTACGTATGA
Consensus      GAATCTGGAAATAAACTCTACCGTCACATCAGAAAACATTTGTCTAGGCATGACAGTGTAAATGACCTGCGAGATGCCCTTGTTTTTCACTGGCTCTATACCAGCAAAAGGCTCCAGACACTTGCCAGCACCACTCACATCAAGCAACACTGTACAGCCTGTGGGGAGGCTGGCCACAATTGCGCGCACCTGTACGTATGA

5201                                                                                                                                                                                                      5400
NMRB01002322.1 TGAAAATGCCATAGGGTGATTCATGGCATCCTCGGGATGAAGGGGCAAAAAGGGGTCGTCGTCGTGATAAGTATTAGTAAAAAATGTTTATGATTGTCCTAGCAGCGAAGGAATGTCCACAGGGTGAAATTGTCCTTACTGGGGACTGAACCTGGGCTGCTAGGCCAAGCACAATCATTTTGGTAAAACTAACAGACTCA
GFRY01014649.1 TGAAAATGCCATAGGGTGATTCATGGCATCCTCGGGATGAAGGGGCAAAAAGGGGTCGTCGTCGTGATAAGTATTAGTAAAAAATGTTTATGATTGTCCTAGCAGCGAAGGAATGTCCACAGGGTGAAATTGTCCTTACTGGGGACTGAACCTGGGCTGCTAGGCCAAGCACAATCATTTTGGTAAAACTAACAGACTCA
GFRY01014650.1 TGAAAATGCCATAGGGTGATTCATGGCATCCTCGGGATGAAGGGGCAAAAAGGGGTCGTCGTCGTGATAAGTATTAGTAAAAAATGTTTATGATTGTCCTAGCAGCGAAGGAATGTCCACAGGGTGAAATTGTCCTTACTGGGGACTGAACCTGGGCTGCTAGGCCAAGCACAATCATTTTGGTAAAACTAACAGACTCA
Consensus      TGAAAATGCCATAGGGTGATTCATGGCATCCTCGGGATGAAGGGGCAAAAAGGGGTCGTCGTCGTGATAAGTATTAGTAAAAAATGTTTATGATTGTCCTAGCAGCGAAGGAATGTCCACAGGGTGAAATTGTCCTTACTGGGGACTGAACCTGGGCTGCTAGGCCAAGCACAATCATTTTGGTAAAACTAACAGACTCA
```

```
                  5401                                                                                                                                                                                                       5600
NMRB01002322.1    TTCAAAACTATGGAACAGCAGAAGTAAAATGATTGTCTTGTATTTTCCTGGTGATGTATGAAAATATACATGTTGTACCTCAAAGAGAATTGCGTAGAGTTATGCTATTTTCTTTGTTTTTTTCTGCCCTTTGTTTTCTTAGGTTTGCAGTTATGACATAGGAAGTCAATGTTGTCAAGTTTCCCTTGATCTGAGTGTCT
GFRY01014649.1    TTCAAAACTATGGAACAGCAGAAGTAAAATGATTGTCTTGTATTTTCCTGGTGATGTATGAAAATATACATGTTGTACCTCAAAGAGAATTGCGTAGAGTTATGCTATTTTCTTTGTTTTTTTCTGCCCTTTGTTTTCTT
GFRY01014650.1    TTCAAAACTATGGAACAGCAGAAGTAAAATGATTGTCTTGTATTTTCCTGGTGATGTATGAAAATATACATGTTGTACCTCAAAGAGAATTGCGTAGAGTTATGCTATTTTCTTTGTTTTTTTCTGCCCTTTGTTTTCTT
 Consensus        TTCAAAACTATGGAACAGCAGAAGTAAAATGATTGTCTTGTATTTTCCTGGTGATGTATGAAAATATACATGTTGTACCTCAAAGAGAATTGCGTAGAGTTATGCTATTTTCTTTGTTTTTTTCTGCCCTTTGTTTTCTT............................................................

                  5601
NMRB01002322.1    G
GFRY01014649.1
GFRY01014650.1
 Consensus        .
```

>TSA: GFRY01014649.1:translation

5'3' Frame 1

```
NCLTILDSASRVA-NRSGWNEKLIQVGLR-YFLANIADLSHFVNDLDLLPRCIDFS-GTKGFWAVFDQLIVCQHHIVSSDI--VHSCCHPWSCVDHCCPQICSYPHLCLWLNNMGLLLSLRLPSEIQF-SNSFLFSEMLSAMDDFHSEVLKHLCRICSGVLNSKACGAAPRQKSFLSVEACASALLRIYGISVETEDNSVFPTSVCNKCRIKISREMKK
TVIEQQVLPHLFRPHQHDDLNCICHKLCSAVSVPLPPPTTESVAIEPSILTIEHSYVHDIGAAAKPSAPSTPEVRSKKSLKELNRQYIKRTRLKDLFSNLHDFCKEKKEDVVDVTFSLLIQELIDSDRKKEADRVMEIWSGSDKSKLSVEECLAMRVRNTLSKTHYAQMYAVHRDKTSLNSLQPVKQLTSIENLFFPGNCAYKLFKNDCLIQEQDILSK
VDCEPKDIMRNFANFPSDFPLPNVSGLEFDYISAVAKTLTELEESISARLKDCGLPIDTSVHTIIKDGGDGLGDVNIKTRKGEFSLTDKVFRYSFCVLSCSATVNEKKYELFKEASASSVDCTRPVAICHCDESDRPSISITLANISKARDAMQNGVMKLRFGGGHIVPERSHTLQFLFTMVDEKFERTSSGLQGSGSRYLCTLCEATTANCQCDLGSF
TRTRTHDQTVSLYRYRFENPEELNENDLAEKCKGVKAMPLISSDASERSLDSTHANINMGRFFKRILVREAAEVYEWTESDRNRSRLQHVEKSLDAMLIEKLGIQPKMIMVGNDARKLFNGANHDIVTSLIKNEERKGLVKHILSLYSKMHSVYSSTSPTVELPELTGNYREIAFQFSSCLGEHFPYITWSNYLHKVVEHVAEIISDPHGLGSVGLLSS
EGGESGNKLYRHIRKHLSRHDSVNDLRDALVFHWLYTSKRLQTLASTTHIKQHCTACGEAGHNCRTCTYDENAIG-FMASSG-RGKKGSSS--VLVKNVYDCPSSEGMSTG-NCPYWGLNLGC-AKHNHFGKTNRLIQNYGTAEVK-LSCIFLVMYENIHVVPQRELRRVMLFSLFFSALCFL
```

>TSA: GFRY01014650.1:translation

5'3' Frame 2

```
YSCTRPSLHHVCSYSHRNL-CK-NPPWAPP-LDASRLSRDPRSTLRGDYHPSFTDLPAGLFTRNACAVTIRKCAVDGALSGCLFHKNRPRNAIFVNFFAFFT-KSGRKASVVPLEDLSEMLSAMDDFHSEVLKHLCRICSGVLNSKACGAAPRQKSFLSVEACASALLRIYGISVETEDNSVFPTSVCNKCRIKISREMKKTVIEQQVLPHLFRPHQHD
DLNCICHKLCSAVSVPLPPPTTESVAIEPSILTIEHSYVHDIGAAAKPSAPSTPEVRSKKSLKELNRQYIKRTRLKDLFSNLHDFCKEKKEDVVDVTFSLLIQELIDSDRKKEADRVMEIWSGSDKSKLSVEECLAMRVRNTLSKTHYAQMYAVHRDKTSLNSLQPVKQLTSIENLFFPGNCAYKLFKNDCLIQEQDILSKVDCEPKDIMRNFANFPSD
FPLPNVSGLEFDYISAVAKTLTELEESISARLKDCGLPIDTSVHTIIKDGGDGLGDVNIKTRKGEFSLTDKVFRYSFCVLSCSATVNEKKYELFKEASASSVDCTRPVAICHCDESDRPSISITLANISKARDAMQNGVMKLRFGGGHIVPERSHTLQFLFTMVDEKFERTSSGLQGSGSRYLCTLCEATTANCQCDLGSFTRTRTHDQTVSLYRYRFE
NPEELNENDLAEKCKGVKAMPLISSDASERSLDSTHANINMGRFFKRILVREAAEVYEWTESDRNRSRLQHVEKSLDAMLIEKLGIQPKMIMVGNDARKLFNGANHDIVTSLIKNEERKGLVKHILSLYSKMHSVYSSTSPTVELPELTGNYREIAFQFSSCLGEHFPYITWSNYLHKVVEHVAEIISDPHGLGSVGLLSSEGGESGNKLYRHIRKHLS
RHDSVNDLRDALVFHWLYTSKRLQTLASTTHIKQHCTACGEAGHNCRTCTYDENAIG-FMASSG-RGKKGSSS--VLVKNVYDCPSSEGMSTG-NCPYWGLNLGC-AKHNHFGKTNRLIQNYGTAEVK-LSCIFLVMYENIHVVPQRELRRVMLFSLFFSALCFL
```

RAG2L

>WGS: NMRB01002322.1:107000-112446 (end)
>TSA: GFRY01034926.1:beg-end (reverse complement)

```
                  1                                                                                                                                                                                                          200
NMRB01002322.1    GAATCTGGAAATAAACTCTACCGTCACATCAGAAAACATTTGTCTAGGCATGACAGTGTAAATGACCTGCGAGATGCCCTTGTTTTTCACTGGCTCTATACCAGCAAAAGGCTCCAGACACTTGCCAGCACCACTCACATCAAGCAACACTGTACAGCCTGTGGGGAGGCTGGCCACAATTGCCGCACCTGTACGTATGA
GFRY01034926.1
 Consensus        ........................................................................................................................................................................................................

                  201                                                                                                                                                                                                        400
NMRB01002322.1    TGAAAATGCCATAGGGTGATTCATGGCATCCTCGGGATGAAGGGGCAAAAAGGGGTCGTCGTCGTGATAAGTATTAGTAAAAAATGTTTATGATTGTCCTAGCAGCGAAGGAATGTCCACAGGGTGAAATTGTCCTTACTGGGGACTGAACCTGGGCTGCTAGGCCAAGCACAATCATTTTGGTAAAACTAACAGACTCA
GFRY01034926.1
 Consensus        ........................................................................................................................................................................................................

                  401                                                                                                                                                                                                        600
NMRB01002322.1    TTCAAAACTATGGAACAGCAGAAGTAAAATGATTGTCTTGTATTTTCCTGGTGATGTATGAAAATATACATGTTGTACCTCAAAGAGAATTGCGTAGAGTTATGCTATTTTCTTTGTTTTTTTCTGCCCTTTGTTTTCTTAGGTTTGCAGTTATGACATAGGAAGTCAATGTTGTCAAGTTTCCCTTGATCTGAGTGTCT
GFRY01034926.1                             AAAATGATTGTCTTGTATTTTCCTGGTGATGTATGAAAATATACATGTTGTACCTCAAAGAGAATTGCGTAGAGTTATGCTATTTTCTTTGTTTTTTTCTGCCCTTTGTTTTCTTAGGTTTGCAGTTATGACATAGGAAGTCAATGTTGTCAAGTTTCCCTTGATCTGAGTGTCT
 Consensus        .........................AAAATGATTGTCTTGTATTTTCCTGGTGATGTATGAAAATATACATGTTGTACCTCAAAGAGAATTGCGTAGAGTTATGCTATTTTCTTTGTTTTTTTCTGCCCTTTGTTTTCTTAGGTTTGCAGTTATGACATAGGAAGTCAATGTTGTCAAGTTTCCCTTGATCTGAGTGTCT

                  601                                                                                                                                                                                                        800
NMRB01002322.1    GAATTCTTCTGTACAGCAGACATGGAACAATTTGTCACATCTCACACACTGCAAAGATCTGACAAGTTCACCTGGAGTATACTCGGAGTCAACCACACAAGTTTCAGCTTCGCACCTTCCTGGGTCCATTTTCTTTGTGGTCAGTGTAAACAGATGTTGATTTGCACCACCATGAAAAAATACAGATGAACTGTCCAAAG
GFRY01034926.1    GAATTCTTCTGTACAGCAGACATGGAACAATTTGTCACATCTCACACACTGCAAAGATCTGACAAGTTCACCTGGAGTATACTCGGAGTCAACCACACAAGTTTCAGCTTCGCACCTTCCTGGGTCCATTTTCTTTGTGGTCAGTGTAAACAGATGTTGATTTGCACCACCATGAAAAAATACAGATGAACTGTCCAAAG
 Consensus        GAATTCTTCTGTACAGCAGACATGGAACAATTTGTCACATCTCACACACTGCAAAGATCTGACAAGTTCACCTGGAGTATACTCGGAGTCAACCACACAAGTTTCAGCTTCGCACCTTCCTGGGTCCATTTTCTTTGTGGTCAGTGTAAACAGATGTTGATTTGCACCACCATGAAAAAATACAGATGAACTGTCCAAAG

                  801                                                                                                                                                                                                        1000
NMRB01002322.1    GGATGGAAGTGTGGCTGGCCATTTCAAATCCTGGAGGTGCAGTCATTAAAGTGACAGATGCCGTGGCTATGTTTACAATAAAATACTGTCCACTGGGAACAGTAGAATGTATTTCATTGTCAACTTGCTGATAACCCCCATATACCAACAGTTCACTGGGACTGCAGACATTTGTAGTATGGCCAGAGAGAAGAATGTTT
GFRY01034926.1    GGATGGAAGTGTGGCTGGCCATTTCAAATCCTGGAGGTGCAGTCATTAAAGTGACAGATGCCGTGGCTATGTTTACAATAAAATACTGTCCACTGGGAACAGTAGAATGTATTTCATTGTCAACTTGCTGATAACCCCCATATACCAACAGTTCACTGGGACTGCAGACATTTGTAGTATGGCCAGAGAGAAGAATGTTT
 Consensus        GGATGGAAGTGTGGCTGGCCATTTCAAATCCTGGAGGTGCAGTCATTAAAGTGACAGATGCCGTGGCTATGTTTACAATAAAATACTGTCCACTGGGAACAGTAGAATGTATTTCATTGTCAACTTGCTGATAACCCCCATATACCAACAGTTCACTGGGACTGCAGACATTTGTAGTATGGCCAGAGAGAAGAATGTTT

                  1001                                                                                                                                                                                                       1200
NMRB01002322.1    ACCAAACCACCAGCAAATGTCAGTTGTATTTTACTTATGACTGCCCTTATGCCATTTTGACCTTCCTGTGGCAAGTGACACTGAATAATGGAAACATCCAGAATGGACATTCTTCTGATCACAGTTTTATTGTCCTCTTGTACCTGTAACAGGCCCCCTAAGTAAACAACTGTGCAGTCTTTAATTTCATTTCCAGTATG
GFRY01034926.1    ACCAAACCACCAGCAAATGTCAGTTGTATTTTACTTATGACTGCCCTTATGCCATTTTGACCTTCCTGTGGCAAGTGACACTGAATAATGGAAACATCCAGAATGGACATTCTTCTGATCACAGTTTTATTGTCCTCTTGTACCTGTAACAGGCCCCCTAAGTAAACAACTGTGCAGTCTTTAATTTCATTTCCAGTATG
 Consensus        ACCAAACCACCAGCAAATGTCAGTTGTATTTTACTTATGACTGCCCTTATGCCATTTTGACCTTCCTGTGGCAAGTGACACTGAATAATGGAAACATCCAGAATGGACATTCTTCTGATCACAGTTTTATTGTCCTCTTGTACCTGTAACAGGCCCCCTAAGTAAACAACTGTGCAGTCTTTAATTTCATTTCCAGTATG

                  1201                                                                                                                                                                                                       1400
NMRB01002322.1    GAAAGCAACGGGAGGGAGATCCTGTTGTCCTTGGATTATTATCTCTTGCCAGTGGTATTTCTCCATATCTAGCCAGTACATTTTACCATCAATAGTTGCATGATTTCCTCCCTTCTCCACACATCCCACTCCCCCATACAATATTGCTCCAACCTCCTTGCCCTCCTTGTTCATTAAAGTAAGGGAATGTCCATACCTTG
GFRY01034926.1    GAAAGCAACGGGAGGGAGATCCTGTTGTCCTTGGATTATTATCTCTTGCCAGTGGTATTTCTCCATATCTAGCCAGTACATTTTACCATCAATAGTTGCATGATTTCCTCCCTTCTCCACACATCCCACTCCCCCATACAATATTGCTCCAACCTCCTTGCCCTCCTTGTTCATTAAAGTAAGGGAATGTCCATACCTTG
 Consensus        GAAAGCAACGGGAGGGAGATCCTGTTGTCCTTGGATTATTATCTCTTGCCAGTGGTATTTCTCCATATCTAGCCAGTACATTTTACCATCAATAGTTGCATGATTTCCTCCCTTCTCCACACATCCCACTCCCCCATACAATATTGCTCCAACCTCCTTGCCCTCCTTGTTCATTAAAGTAAGGGAATGTCCATACCTTG

                  1401                                                                                                                                                                                                       1600
NMRB01002322.1    GACTAGGAACACTTCCAGTCTGAAGAGACACTGTATCCGATGTCCCTATTAGTTTGGCATTGAAACCCCTGCCAAAATTTTCAAGTACAATTAAATCATTATGAGGTACCATGGCTATGTTTGGATCAATGCCTCCCCAAATGTAGACAGATGTTTTCACCGCAGCAGCACTAGCAACAGAACCTATCCTCCTAACTGAC
GFRY01034926.1    GACTAGGAACACTTCCAGTCTGAAGAGACACTGTATCCGATGTCCCTATTAGTTTGGCATTGAAACCCCTGCCAAAATTTTCAAGTACAATTAAATCATTATGAGGTACCATGGCTATGTTTGGATCAATGCCTCCCCAAATGTAGACAGATGTTTTCACCGCAGCAGCACTAGCAACAGAACCTATCCTCCTAACTGAC
 Consensus        GACTAGGAACACTTCCAGTCTGAAGAGACACTGTATCCGATGTCCCTATTAGTTTGGCATTGAAACCCCTGCCAAAATTTTCAAGTACAATTAAATCATTATGAGGTACCATGGCTATGTTTGGATCAATGCCTCCCCAAATGTAGACAGATGTTTTCACCGCAGCAGCACTAGCAACAGAACCTATCCTCCTAACTGAC

                  1601                                                                                                                                                                                                       1800
NMRB01002322.1    ACATCCCGAGTTCTTGTGTGGAATACTATGTTAGCCGAATGAATGAATGGTCTCATACTTATCTGCGCACCAGTGCATTTTTTTTCTGTCACCTGCAACACCTTAATGTCACTCTCCTGTATATGAAAAGTGTAACTAAATATCGATGCAGTCGTCGTCGTGAGTGGCATTCTTGGATGCATGCCGCCTGTAACAAACAC
GFRY01034926.1    ACATCCCGAGTTCTTGTGTGGAATACTATGTTAGCCGAATGAATGAATGGTCTCATACTTATCTGCGCACCAGTGCATTTTTTTTCTGTCACCTGCAACACCTTAATGTCACTCTCCTGTATATGAAAAGTGTAACTAAATATCGATGCAGTCGTCGTCGTGAGTGGCATTCTTGGATGCATGCCGCCTGTAACAAACAC
 Consensus        ACATCCCGAGTTCTTGTGTGGAATACTATGTTAGCCGAATGAATGAATGGTCTCATACTTATCTGCGCACCAGTGCATTTTTTTTCTGTCACCTGCAACACCTTAATGTCACTCTCCTGTATATGAAAAGTGTAACTAAATATCGATGCAGTCGTCGTCGTGAGTGGCATTCTTGGATGCATGCCGCCTGTAACAAACAC

                  1801                                                                                                                                                                                                       2000
NMRB01002322.1    ATCCACAATACGACCCTGTACATCATCTGTCAGTGCATATGCAGTTCGTAAAGCAGGCGTAAGTTCCGCGTCATTTCTAGACCAAATTAAATGGCCCTTCCCTGAAAACAGCCCCAGTGAGCTGATGCAATCTGTCATCGATTTCTTCGTCACCTTTTTACGATTTTGTACAGCCTTTAGGATGGAAAAAAACCGTGCAT
GFRY01034926.1    ATCCACAATACGACCCTGTACATCATCTGTCAGTGCATATGCAGTTCGTAAAGCAGGCGTAAGTTCCGCGTCATTTCTAGACCAAATTAAATGGCCCTTCCCTGAAAACAGCCCCAGTGAGCTGATGCAATCTGTCATCGATTTCTTCGTCACCTTTTTACGATTTTGTACAGCCTTTAGGATGGAAAAAAACCGTGCAT
 Consensus        ATCCACAATACGACCCTGTACATCATCTGTCAGTGCATATGCAGTTCGTAAAGCAGGCGTAAGTTCCGCGTCATTTCTAGACCAAATTAAATGGCCCTTCCCTGAAAACAGCCCCAGTGAGCTGATGCAATCTGTCATCGATTTCTTCGTCACCTTTTTACGATTTTGTACAGCCTTTAGGATGGAAAAAAACCGTGCAT

                  2001                                                                                                                                                                                                       2200
NMRB01002322.1    TTCGAGGAAAATTTTCGCGATTGGCAATCAAGCTTGAAGTCAGAAGGTTCAAGCTTGAAGATTGAAGTCAGGGGTTGGTTCAAGCTTGAAGATTGAAGTCCGGGGTTCAAGCTTGAAGACTGAAGTCTGAGGGTTCAAGCTTGAAGATTGAAGTTCCTGGTTCAAGCTTGAAGTCAGAAGGTTCAAGCTTGAAGATCGAA
GFRY01034926.1    TTCGAGGAAAATTTTCGCGATTGGCAATCAT-------------------------------------------------------------------------------------------------------------------------------------------------------------------------
 Consensus        TTCGAGGAAAATTTTCGCGATTGGCAATCAa.........................................................................................................................................................................

                  2201                                                                                                                                                                                                       2400
NMRB01002322.1    GTCAGGGGTTGGTTCAAGCTTGAAGATTGAAATCCGGGGTTCAAGCTTGAAGATTGAAGTCTGAGAGTTCAAGCTTGAAGATTGAACTCCGGATTCAAGTTTGAAGTAGGAAGGTTCAAGTTTGAAGATTGGAGTGGAAATAATTAATATTGTACATTTTTTGATAGCAATCCGCGGAAATGGTTGGAATTGGATCATAT
GFRY01034926.1    --------------------------------------------------------------------------------------------------------------------------------------------------------------------------------------------------------
 Consensus        ........................................................................................................................................................................................................

                  2401                                                                                                                                                                                                       2600
NMRB01002322.1    TAAACATATCGAATACTAAATGTTGCAATTTCTTAGGGATGAAGGTCTATATAACTCATAAATTGGAATGATTTGTCTGGCTTCAATATTGAAGTCGGTGGTTCAAGCTTGAAGATTGAAGTCGGAGTTACTTCAAGCTTGAAGATTGAAGTTCTACTTCAAGCTTGAAGATTGAAGATTGAATGAGCCTTCTGGGCTTT
GFRY01034926.1    --------------------------------------------------------------------------------------------------------------------------------------------------------------------------------------------------------
 Consensus        ........................................................................................................................................................................................................

                  2601                                                                                                                                                                                                       2800
NMRB01002322.1    CGTACTCTGGACCGACCCCTTGGAGGTGAGGATAAAGTATACTAACTCATTTGAAGATGATTATGTGGATTAGCTGAGAGGTTAACTGGTGCGTGCAAGACTTCACTTGCTTTCATCATGTTGGTAGACTGACATAATCTACGCAGTCAAAAGGCTTTCAGGTCATGAACCAAACAAAATGTGACGTCACTTATGCAAAC
GFRY01034926.1    --------------------------------------------------------------------------------------------------------------------------------------------------------------------------------------------------------
 Consensus        ........................................................................................................................................................................................................

                  2801                                                                                                                                                                                                       3000
NMRB01002322.1    AACATGAGAAGTTCTGCGCGTGCGTGTTTTGTAATTGGCACTTTCAGGCAGACGGCATTCGATTGGTCATTTCTTAAACCAGATTACCAATACAATGATGTGATTTCGTTGATGTCATGTGCAGTTCTGTGTTTGCATCTCAGCCTGGAATGTTCAACCATAAACGTTTTCGACTATGGCGGACATTTAATATGTCAGCT
GFRY01034926.1    ----------------------------------------------------------------------------------------------------------------TGCCATGATCGCCGCCATGTTTGTTTATCCTGGATACGCCCCCATGCGCATGCGCTGTTGAGTGAGGCCGCCTATTGTTGTTGCG--
 Consensus        ................................................................................................................TGcCATGagCaccgCcaTGTTTGcaTaTCagccagaaaccccCAacCacAaaCGcTgTcGAcTaaGGCcGaCaaTTaaTaTgTcaG..

                  3001                                                                                                                                                                                                       3200
NMRB01002322.1    GAGTCAAAAGAAAGGAACTTGTATGCAAAAAGAAGAAGCGATACACTGCAGTGGAGCTCGGATGTATGAAAGAAAGGTAAGCAACTGACATCTGACCTCTAATCTGCGTATTTTAATGGGACACAGATATGGGGACATAAAATGCCTGTGATGTGGTGGGGATTACACCCATAATTGTTTTTAAGTTCGCCCTTAATGGG
GFRY01034926.1    --------------------------------------------------------------------------------------------------------------------------------------------------------------------------------------------------------
 Consensus        ........................................................................................................................................................................................................

                  3201                                                                                                                                                                                                       3400
NMRB01002322.1    GTAGCCCCCAAATCTTTCCTAACCAGGTGAAAATTATAAATGGAGCGATGAGAACCAAATACGACATTGTTCGCCCTATCGACAAGATTGGGCAAGACCAGGTGAAAATTATAAATGGAGCGATGAGAACCAAATACGGCATTGTTCGCCCTATCGACAAGATTGGGCATGTAAATCGAGCAGGTGGAAACCGGAAATGC
GFRY01034926.1    --------------------------------------------------------------------------------------------------------------------------------------------------------------------------------------------------------
 Consensus        ........................................................................................................................................................................................................

                  3401                                                                                                                                                                                                       3600
NMRB01002322.1    CGGAGAAGAAGTAGGCTGTCAATGAGAGTCGCCCTCTCTACAACATCGTGAATGGAACTCGGAATCTCAGAGGTGACAGGAGCTGATGTTTCCACTGAACTACTACTGATGATTGAACATGTATACATGTCATTTCTTGTACATGTATATTCGTACTATCTCACCCGGGTCAGAAATCAATTCGGAGGAAAACGTTCAAC
GFRY01034926.1    --------------------------------------------------------------------------------------------------------------------------------------------------------------------------------------------------------
 Consensus        ........................................................................................................................................................................................................

                  3601                                                                                                                                                                                                       3800
NMRB01002322.1    ATTTTCAAAAAGGTTTTAAATGAGTAATTAGTCTAAACATTAATGTAATTGAGATACAGAGTGAATATTGGTTTAACCCTGTGTACCCGTTAGCTACGCTAATTGTGAGGGCGACTAATCTCTAAAATGTACTTCCTAAAGACACATTTTTCAGTGCGTTTTATTGCTTTTTCATGAGGGAAGTAGACTTTTTTGTCAT
GFRY01034926.1    ------------------------------------------------------------------------------------------TAACTACGTTTTTTATCACTGCG------------------------------------------------------------------------------------CTT
 Consensus        ..........................................................................................TAaCTACGcTaaTTaTcAcgGCG....................................................................................CaT

                  3801                                                                                                                                                                                                       4000
NMRB01002322.1    ACAGGAACATTATGAAAGGCAAATAAAGACTGGGGGTGCTGAAAAATTAATCCCTTTGAAAATGGATCGGTGGTCACAACAAGGATGGAGTGATAGAAGAATAAATGGAGTCGTTCACCAAAAATTAGTTATAGAAATATCATGAAAACATACAATTCGTAAGATCGTCAAGAAAATATTAAAGATAATGTATCATAACT
GFRY01034926.1    GCGCGCGCTGTTTGATAGGCAACGCTGTACACAGGTTTCGG
 Consensus        aCacGaaCagTaTGAaAGGCAAagaaagACacaGGgTgCgG...............................................................................................................................................................

                  4001                                                                                                                                                                                                       4200
NMRB01002322.1    TCATTGTTTACATTCAATATTCGTAAACATGGATTTGTAAACAGGGATACATTGTGTTAGTTTGGGCTTTTGGGACATGCCAACCCACAAAAATGAATACATTCAGTGTCAAACGAATTTCTGGAGGGCATGTCAACAACGAACTGTGTACAATGTACTCTTAAGACAAACAAAGAGACTGGGCAGAAACAAACAAATAA
GFRY01034926.1
```

```
 Consensus      ........................................................................................................................................................................................................

                4201                                                                                                                                                                                                4400
NMRB01002322.1  ACAATGACGGCCGGAGAAAATACCAGTGATACATTATTCTTTAATAACTACAGTGTACAATTTTTACCAATAAATGTAGAGCCGAAGAGGAGATTTTCGAATGTACGATAAGTTTTTATTGATTCCAAAGTACAGTGGAAACGGACTAGCATGTCGTATCCGTTTCCATCCCACTCTGCCCGTGATTATTTTCATCCAAC
GFRY01034926.1
 Consensus      ........................................................................................................................................................................................................

                4401                                                                                                                                                                                                4600
NMRB01002322.1  GTGATCTGCACGCTCACCGTTATCGATGTCTTTCGTACGCTCTCGACGCAGACGGCACAAGTTACAGGCTGCTTCACAATGTGACGTACGTCTGCAGACATCAGACATCGGTCCTACGTACCATCGCCATTACCGTGAAATACATTGTAATGTTCAAGGTGCAGAAGATGGGGTTGAGAAAATGGCGACTGAAACCAGTT
GFRY01034926.1
 Consensus      ........................................................................................................................................................................................................

                4601                                                                                                                                                                                                4800
NMRB01002322.1  TTGTCCCTGTACGACTTCAGGACAGGAAATTACGTCGGTCACATATTGATCAACAAGAGAGACAGGTGTTTGGAATGACGGAGCCGTGGTGGTCCTGGAACTGTCGCTATCGCCAGGTGGAAGGTACACCTGGACATGGAAAGTGTTGTTTCGATTTGCCCAGTGGCTCAGTACGATTTTATTATGACAATATACAATTG
GFRY01034926.1
 Consensus      ........................................................................................................................................................................................................

                4801                                                                                                                                                                                                5000
NMRB01002322.1  TCACGCACAGCGCACAGGACATGAAATGGTTTACCGTCTATCATTGAATGTTTCTGACAAGATTAGTGTTTGGGGGGTCAGATCTGCGGCCGTACACTTTCACAGATTGGATGTCACCATTGCATGTCAGGTATTCCTCAGAACGATGTGGTTTAAACTGGTTTTGTTTCATCGATCAAAAACTGGTTGGTGGTTGAGGG
GFRY01034926.1
 Consensus      ........................................................................................................................................................................................................

                5001                                                                                                                                                                                                5200
NMRB01002322.1  AAAACGATACTATCATACCACTTCCCGGTAAGGCTAAAATATTAGCTAGAAACACCACCGAAGGAATTGTTTTGTGTCAGTTTAGGAACTTACTATTAAAAAACCAACTTACCACTGTTGATCTAGGATGCTTTAGAGGAGGAGCGATTGTTTCGAATGATATTCCATTAAAATTAACACTTTTCAAGATAAGAGGTCCT
GFRY01034926.1
 Consensus      ........................................................................................................................................................................................................

                5201                                                                                                                                                                                                5400
NMRB01002322.1  GTTTCAATACTGAATCAATTGTCATATCATAACTTAGCAGTTGATGGGAAAAAGTCCCTGATGGTGATACATGGGTTTCTCGATAGCACTAAGAGGCGCCGTATTTACAAAACGGTTTATATTTTCAAACAAGTTCTTTGAATAGCATTTCCCGTTTCATCCAGAGTAAACGGAACCAGAATTCGAGAATTAGGCTTTGG
GFRY01034926.1
 Consensus      ........................................................................................................................................................................................................

                5401                                      5447
NMRB01002322.1  CAATGTTAGGCCTTTATTGGTAACATTTTTGTGAATCTTATATGTTG
GFRY01034926.1
 Consensus      ...............................................
```

>TSA: GFRY01034926.1 translation

5'3' Frame 1

```
PKPVYSVAYQTARASAVIKNVVTQTTIGGLTQQRMRMGAYPG-TNMAAIMAMIANRENFPRNARFFSILKAVQNRKKVTKKSMTDCISSLGLFSGKGHLIWSRNDAELTPALRTAYALTDDVQGRIVDVFVTGGMHPRMPLTTTTASIFSYTFHIQESDIKVLQVTEKKCTGAQISMRPFIHSANIVFHTRTRDVSVRRIGSVASAAAVKTSVYIWGGI
DPNIAMVPHNDLIVLENFGRGFNAKLIGTSDTVSLQTGSVPSPRYGHSLTLMNKEGKEVGAILYGGVGCVEKGGNHATIDGKMYWLDMEKYHWQEIIIQGQQDLPPVAFHTGNEIKDCTVVYLGGLLQVQEDNKTVIRRMSILDVSIIQCHLPQEGQNGIRAVISKIQLTFAGGLVNILLSGHTTNVCSPSELLVYGGYQQVDNEIHSTVPSGQYFIVN
IATASVTLMTAPPGFEMASHTSIPLDSSSVFFHGGANQHLFTLTTKKMDPGRCEAETCVVDSEYTPGELVRSLQCVRCDKLFHVCCTEEFRHSDQGKLDNIDFLCHNCKPKKTKGRKKQRK-HNSTQFSLRYNMYIFIHHQENTRQSF
```

Notospermus geniculatus (Nge)

WGS: NMRB01002297.1

61607-65949
Notospermus geniculatus strain Ushimado scaffold2297, whole genome shotgun sequence

```
      E S H H G K * C C C S P G G L K V P L S S L R * G P A V K S C C T A E I L S I S * K A * R D S V A A F W Y * C * * G G F * N I S K K V T   F1
       R V I M A S S A A A H Q E V L K C L C R L C A E D L Q S R A V V R Q K S F R S V E K H E E T L L L H F G I D V N K E D S E I F P K L L  F2
        E S S W Q V V L L L T R R S * S A F V V F A L R T C S Q E L L Y G R N P F D Q L K S M K R L C C C I L V L M L I R R I L K Y F Q S Y   F3
   1 GAGAGTCATCATGGCAAGTAGTGCTGCTGCTCACCAGGAGGTCTTAAAGTGCCTTTGTCGTCTTTGCGCTGAGGACCTGCAGTCAAGAGCTGTTGTACGGCAGAAATCCTTTCGATCAGTTGAAAAGCATGAAGAGACTCTGTTGCTGCATTTTGGTATTGATGTTAATAAGGAGGATTCTGAAATATTTCCAAAGTTAC 200
     ----:----|----:----|----:----|----:----|----:----|----:----|----:----|----:----|----:----|----:----|----:----|----:----|----:----|----:----|----:----|----:----|----:----|----:----|----:----|----:----|
   1 CTCTCAGTAGTACCGTTCATCACGACGACGAGTGGTCCTCCAGAATTTCACGGAAACAGCAGAAACGCGACTCCTGGACGTCAGTTCTCGACAACATGCCGTCTTTAGGAAAGCTAGTCAACTTTTCGTACTTCTCTGAGACAACGACGTAAAACCATAACTACAATTATTCCTCCTAAGACTTTATAAAGGTTTCAATG 200
       S L * * P L Y H Q Q E G P P R L T G K D D K R Q P G A T L L Q Q V A S I R E I L Q F A H L S E T A A N Q Y Q H * Y P P N Q F I E L T V  F6
     X S D D H C T T S S S V L L D * L A K T T K A S L V Q L * S S N Y P L F G K S * N F L M F L S Q Q Q M K T N I N L L I R F Y K W L *   F5
      L T M M A L L A A A * W S T K F H R Q R R Q A S S R C D L A T T R C F D K R D T S F C S S V R N S C K P I S T L L S S E S I N G F N    F4

      V Q * M Q I * V G P T * Q Q V * S T A F G I S C I F * P P R N * L F L F R I N Q * D G N R C T I R T G A L G N R H C * R Y C C * S S I   F1
       C N K C R S K L D R L N S K S D P Q H L E S P V F S S H Q E I D C F C L E L I N R M G I D V P S E P E P L E I G I A D V T A V D H P  F2
     C A I N A D L S W T D L T A S L I H S I W N L L Y F L A T K K L T V S V * N * S I G W E S M Y H Q N R S P W K S A L L T L L L L I I H   F3
 201 TGTGCAATAAATGCAGATCTAAGTTGGACCGACTTAACAGCAAGTCTGATCCACAGCATTTGGAATCTCCTGTATTTTCTAGCCACCAAGAAATTGACTGTTTCTGTTTAGAATTAATCAATAGGATGGGAATCGATGTACCATCAGAACCGGAGCCCTTGGAAATCGGCATTGCTGACGTTACTGCTGTTGATCATCCA 400
     ----:----|----:----|----:----|----:----|----:----|----:----|----:----|----:----|----:----|----:----|----:----|----:----|----:----|----:----|----:----|----:----|----:----|----:----|----:----|----:----|
 201 ACACGTTATTTACGTCTAGATTCAACCTGGCTGAATTGTCGTTCAGACTAGGTGTCGTAAACCTTAGAGGACATAAAAGATCGGTGGTTCTTTAACTGACAAAGACAAATCTTAATTAGTTATCCTACCCTTAGCTACATGGTAGTCTTGGCCTCGGGAACCTTTAGCCGTAACGACTGCAATGACGACAACTAGTAGGT 400
       T C Y I C I * T P G V * C C T Q D V A N P I E Q I K * G G L F Q S N R N L I L * Y S P F R H V M L V P A R P F R C Q Q R * Q Q Q D D  F6
      Q A I F A S R L Q V S K V A L R I W L M Q F R R Y K R A V L F N V T E T * F * D I P H S D I Y W * F R L G Q F D A N S V N S S N I M W   F5
     S H L L H L D L N S R S L L L D S G C C K S D G T N E L W W S I S Q K Q K S N I L L I P I S T G D S G S G K S I P M A S T V A T S * G    F4

       H W W * S F C Y K Y T R F F S S Q S N C P P C K K G K P * R S Q P S I C E R G T T * G F N N K C E Q F L * R T * * R Y I G C T V F Y   F1
     Y T G G D H S A S T S T R G S S A A N P T V R R A K R A S L R E A N R R Y V K G E R L K D L I T S V N S F C E E R D E D T L D A L F F M  F2
      T L V V I I L L Q V H E V L Q Q P I Q L S A V Q K G Q A L E K P T V D M * K G N D L R I * * Q V * T V S V K N V M K I H W M H C F L C   F3
 401 TACACTGGTGGTGATCATTCTGCTACAAGTACACGAGGTTCTTCAGCAGCCAATCCAACTGTCCGCCGTGCAAAAAGGGCAAGCCTTAGAGAAGCCAACCGTCGATATGTGAAAGGGGAACGACTTAAGGATTTAATAACAAGTGTGAACAGTTTCTGTGAAGAACGTGATGAAGATACATTGGATGCACTGTTTTTTAT 600
     ----:----|----:----|----:----|----:----|----:----|----:----|----:----|----:----|----:----|----:----|----:----|----:----|----:----|----:----|----:----|----:----|----:----|----:----|----:----|----:----|
 401 ATGTGACCACCACTAGTAAGACGATGTTCATGTGCTCCAAGAAGTCGTCGGTTAGGTTGACAGGCGGCACGTTTTTCCCGTTCGGAATCTCTTCGGTTGGCAGCTATACACTTTCCCCTTGCTGAATTCCTAAATTATTGTTCACACTTGTCAAAGACACTTCTTGCACTACTTCTATGTAACCTACGTGACAAAAAATA 600
     M C Q H H D N Q * L Y V L V N K L L W D L Q G G H L F P L G * L L W G D I H S L P V V * P N L L L H S C N R H L V H H L Y M P H V T K *  F6
      V S T T I M R S C T C S T R * C G I W S D A T C F P C A K S F G V T S I H F P F S K L I * Y C T H V T E T F F T I F I C Q I C Q K K   F5
       Y V P P S * E A V L V R P E E A A L G V T R R A F L A L R L S A L R R Y T F P S R S L S K I V L T F L K Q S S R S S S V N S A S N K I  F4

      V V T K T D * S G * E R R G * Q N F V S M E L * Q S G Q L C R E T N N D P R G V S G H E S Q N F T V K G P L * V S L * D S D G E N * L   F1
       L S Q K L I D Q G R K E E A D K I L S L W N C D S Q D S S A E K L T M T P E E C L A M R V R T L Q S K G H Y R S V Y K I Q T E K T S C  F2
        C H K N * L I R V G K K R L T K F C L Y G I V T V R T A L Q R N * Q * P Q R S V W P * E S E L Y S Q R A T I G Q F I R F R R R K L V   F3
 601 GTTGTCACAAAAACTGATTGATCAGGGTAGGAAAGAAGAGGCTGACAAAATTTTGTCTCTATGGAATTGTGACAGTCAGGACAGCTCTGCAGAGAAACTAACAATGACCCCAGAGGAGTGTCTGGCCATGAGAGTCAGAACTTTACAGTCAAAGGGCACTATAGGTCAGTTTATAAGATTCAGACGGAGAAAACTAGTT 800
     ----:----|----:----|----:----|----:----|----:----|----:----|----:----|----:----|----:----|----:----|----:----|----:----|----:----|----:----|----:----|----:----|----:----|----:----|----:----|----:----|
 601 CAACAGTGTTTTTGACTAACTAGTCCCATCCTTTCTTCTCCGACTGTTTTAAAACAGAGATACCTTAACACTGTCAGTCCTGTCGAGACGTCTCTTTGATTGTTACTGGGGTCTCCTCACAGACCGGTACTCTCAGTCTTGAAATGTCAGTTTCCCGTGATATCCAGTCAAATATTCTAAGTCTGCCTCTTTTGATCAA 800
       T T V F V S Q D P Y S L L P Q C F K T E I S N H C D P C S Q L S V L L S G L P T D P W S L * F K V T L P G S Y T L K Y S E S P S F * N  F6
     H Q * L F Q N I L T P F F L S V F N Q R * P I T V T L V A R C L F * C H G W L L T Q G H S D S S * L * L A V I P * N I L N L R L F S T   F5
      N D C F S I S * P L F S S A S L I K D R H F Q S L * S L E A S F S V I V G S S H R A M L T L V K C D F P W * L D T * L I * V S F V L    F4

       * N I V P S R * T K C Y * T H L F L A W P L * V * V G N R G W * N P C S T A G H V K * G R L * T K N Y P R C F Q F I S E * F S T S K C P   F1
     K T L C P P G K L N A I E H T F L P G H C E Y E L V T E D G E T L V A Q Q A M L S E E D S E P K T I L G A F N S F P S D F L P N V  F2
      V K H C A L Q V N * M L L N T P F C L A I V S M S W * P R M V K P L * H S R P C * V R K T L N Q K L S * V L S I H F R V I F H F Q M S   F3
 801 GTAAAACATTGTGCCCTCCAGGTAAACTAAATGCTATTGAACACACCTTTTTGCCTGGCCATTGTGAGTATGAGTTGGTAACCGAGGATGGTGAAACCCTTGTAGCACAGCAGGCCATGTTAAGTGAGGAAGACTCTGAACCAAAAACTATCCTAGGTGCTTTCAATTCATTTCCGAGTGATTTTCCACTTCCAAATGTC 1000
     ----:----|----:----|----:----|----:----|----:----|----:----|----:----|----:----|----:----|----:----|----:----|----:----|----:----|----:----|----:----|----:----|----:----|----:----|----:----|----:----|
 801 CATTTTGTAACACGGGAGGTCCATTTGATTTACGATAACTTGTGTGGAAAAACGGACCGGTAACACTCATACTCAACCATTGGCTCCTACCACTTTGGGAACATCGTGTCGTCCGGTACAATTCACTCCTTCTGAGACTTGGTTTTTGATAGGATCCACGAAAGTTAAGTAAAGGCTCACTAAAAGGTGAAGGTTTACAG 1000
       Y F M T G E L Y V L H * Q V C R K A Q G N H T H T P L R P H H F G Q L V A P W T L H P L S Q V L F * G L H K * N M E S H N E V E L H  F6
      T F C Q A R W T F * I S N F V G K Q R A M T L I L Q Y G L I T F G K Y C L L G H * T L F V R F W F S D * T S E I * K R T I K W K W I D   F5
     Q L V N H G G P L S F A I S C V K K G P W Q S Y S N T V S S P S V R T A C C A M N L S S S E S G F V I R P A K L E N G L S K G G S G F T    F4

       W S T I Q L H F V C G * G T T G V G E C Y F R K I N I S W F T N L H * G * N P D Q G W G * W S W G C G R K K K E R G S C T Y G Q S V   F1
      H G V Q F S Y I S C V A K A L Q E L E N V I S E K L T S L G L P I S T K V E T L I K D G G D G L G D V D V K K R K G D H A L T D K V F  F2
     M E Y N S V T F R V W L R H Y R S W R M L F Q K N * H L L V Y Q S P L R L K P * S R M G V M V L G M W T * K K G K G I M H L R T K C F   F3
1001 CATGGAGTACAATTCAGTTACATTTCGTGTGTGGCTAAGGCACTACAGGAGTTGGAGAATGTTATTTCAGAAAAATTAACATCTCTTGGTTTACCAATCTCCACTAAGGTTGAAACCCTGATCAAGGATGGGGGTGATGGTCTTGGGGATGTGGACGTAAAAAAAAGGAAAGGGGATCATGCACTTACGGACAAAGTGTT 1200
     ----:----|----:----|----:----|----:----|----:----|----:----|----:----|----:----|----:----|----:----|----:----|----:----|----:----|----:----|----:----|----:----|----:----|----:----|----:----|----:----|
1001 GTACCTCATGTTAAGTCAATGTAAAGCACACACCGATTCCGTGATGTCCTCAACCTCTTACAATAAAGTCTTTTTAATTGTAGAGAACCAAATGGTTAGAGGTGATTCCAACTTTGGGACTAGTTCCTACCCCCACTACCAGAACCCCTACACCTGCATTTTTTTTCCTTTCCCCTAGTACGTGAATGCCTGTTTCACAA 1200
      G H L V I * N C K T H P * P V V P T P S H * K L F I L M E Q N V L R W * P Q F G S * P H P H H D Q P H P R L F F S L P D H V * P C L T  F6
       M S Y L E T V N R T H S L C * L L Q L I N N * F F * C R K T * W D G S L N F G Q D L I P T I T K P I H V Y F F P F P I M C K R V F H   F5
     W P T C N L * M E H T A L A S C S N S F T I E S F N V D R P K K G I E V L T S V R I L S P P S P R P S T S T F F L F P S * A S V S L T N    F4

       S I F V L Y S D L H R T H R W K T S G F V S * R T S K L C G L H K T C R R G * L * * K * P T K H C P H C E G G C V * I S R N Y E K * C D   F1
      R Y S F C I L T C T A L I D G K R V V L F R E E H P N S V D C T Q R P V V V A N C D E S D Q P S I A L T V K D V C R S R E I M K N N V M  F2
     D I R F V F * P A P H S * M E N E W F C F V K N I Q T L W T A Q D L S S W L I V M K V T N Q A L P S L * R M C V D L A K L * K I M *   F3
1201 TCGATATTCGTTTTGTATTCTGACCTGCACCGCACTCATAGATGGAAAACGAGTGGTTTTGTTTTCGTGAAGAACATCCAAACTCTGTGGACTGCACAAGACCTGTCGTCGTGGCTAATTGTGATGAAAGTGACCAACCAAGCATTGCCCTCACTGTGAAGGATGTGTGTAGATCTCGCGAAATTATGAAAAATAATGTGA 1400
     ----:----|----:----|----:----|----:----|----:----|----:----|----:----|----:----|----:----|----:----|----:----|----:----|----:----|----:----|----:----|----:----|----:----|----:----|----:----|----:----|
1201 AGCTATAAGCAAAACATAAGACTGGACGTGGCGTGAGTATCTACCTTTTGCTCACCAAAACAAAGCACTTCTTGTAGGTTTGAGACACCTGACGTGTTCTGGACAGCAGCACCGATTAACACTACTTTCACTGGTTGGTTCGTAACGGGAGTGACACTTCCTACACACATCTAGAGCGCTTTAATACTTTTTATTACACT 1400
       E I N T K Y E S R C R V * L H F V L P K T E H L V D L S Q P S C L V Q R R P * N H H F H G V L C Q G * Q S P H T Y I E R F * S F Y H S  F6
      K S I R K T N Q G A G C E Y I S F S H N Q K T F F M W V R H V A C S R D D H S I T I F T V L W A N G E S H L I H T S R A F N H F I I H   F5
       R Y E N Q I R V Q V A S M S P F R T T K N R S S C G F E T S Q V L G T T T A L Q S S L S W G L M A R V T F S T H L D R S I I F F L T   F4

       E V A F L R D C C E R T S P Q I Y V H N G R * K I * A H L V W S P G E W V S V H L H I V L C H K R Q L S G * L W V I * P H K N Q * * N   F1
      K L L S S G T V R E H H L K F M Y T M V D E K F E R T S C G L Q G S G S R F I C T L C C A T S A N C Q A D C G S F S R T R T N D E  F2
     * S C F P Q G L L * E N I T S N L C T Q W * M K N L S A P R V V S R G V G L G S S A H C A V P Q A P T V R L I V G H L A A Q E P M M K   F3
1401 TGAAGTTGCTTTCCTCAGGGACTGTTGTGAGAGAACATCACCTCAAATTTATGTACACAATGGTAGATGAAAAATTTGAGCGCACCTCGTGTGGTCTCCAGGGGAGTGGGTCTCGGTTCATCTGCACATTGTGCTGTGCCACAAGCGCCAACTGTCAGGCTGATTGTGGGTCATTTAGCCGCACAAGAACCAATGATGAA 1600
     ----:----|----:----|----:----|----:----|----:----|----:----|----:----|----:----|----:----|----:----|----:----|----:----|----:----|----:----|----:----|----:----|----:----|----:----|----:----|----:----|
1401 ACTTCAACGAAAGGAGTCCCTGACAACACTCTCTTGTAGTGGAGTTTAAATACATGTGTTACCATCTACTTTTTAAACTCGCGTGGAGCACACCAGAGGTCCCCTCACCCAGAGCCAAGTAGACGTGTAACACGACACGGTGTTCGCGGTTGACAGTCCGACTAACACCCAGTAAATCGGCGTGTTCTTGGTTACTACTT 1600
       S T A K R L S Q Q S L V D G * I * T C L P L H F I Q A C R T H D G P S H T E T * R C M T S H W L R W S D P Q N H T M * G C L F W H H  F6
      H L Q K G * P S N H S F M V E F K H V C H Y I F F K L A G R T T E L P T P R P E D A C Q A T G C A G V T L S I T P * K A A C S G I I F   F5
     I F N S E E P V T T L S C * R L N I Y V I T S S F N S R V E H P R W P L P D R N M Q V N H Q A V L A L Q * A S Q P D N L R V L V L S S    F4

       C T S L Q I S S * E P R Q P L * K * S C R K D K R C Q K H A N D I L * C N G T L F * C N P C K Y K H G Q G S Q E D T C T R N S W G T   F1
      T V R L Y K Y R L E N P D N L S E N D L A E R T K G V K S M P M I F C D A T E R C F D A T H A N I N M G R V L K K T L V R E I A G V H  F2
     L Y V S T N I V L R T Q T T S L K M I L Q K G Q K V S K A C Q * Y F V M Q R N A V L M Q P M Q I * T W A G F S R R H L Y E K * L G Y M   F3
1601 ACTGTACGTCTCTACAAATATCGTCTTGAGAACCCAGACAACCTCTCTGAAAATGATCTTGCAGAAAGGACAAAAGGTGTCAAAAGCATGCCAATGATATTTTGTGATGCAACGGAACGCTGTTTTGATGCAACCCATGCAAATATAAACATGGGCAGGGTTCTCAAGAAGACACTTGTACGAGAAATAGCTGGGGTACA 1800
     ----:----|----:----|----:----|----:----|----:----|----:----|----:----|----:----|----:----|----:----|----:----|----:----|----:----|----:----|----:----|----:----|----:----|----:----|----:----|----:----|
1601 TGACATGCAGAGATGTTTATAGCAGAACTCTTGGGTCTGTTGGAGAGACTTTTACTAGAACGTCTTTCCTGTTTTCCACAGTTTTCGTACGGTTACTATAAAACACTACGTTGCCTTGCGACAAAACTACGTTGGGTACGTTTATATTTGTACCCGTCCCAAGAGTTCTTCTGTGAACATGCTCTTTATCGACCCCATGT 1800
       F Q V D R C I D D Q S G L C G R Q F H D Q L F S L L H * F C A L S I K H H L P V S N Q H L G H L Y L C P C P E * S S V Q V L F L Q P V  F6
      S Y T E V F I T K L V W V V E R F I I K C F P C F T D F A H W H Y I T C R F A T K I C G M C I Y V H A P N E L L C K Y S F Y S P Y   F5
     V T R R * L Y R R S F G S L R E S F S R A S L V F P T L L M G I I N Q S A V S R Q K S A V W A F I F M P L T R L F V S T R S I A P T C    F4

       * M D * E N D * F K * N K A P T C R K N S * * A S P * K T W P S A Q T Y A A R * L C P C P L H S * K P * Y P M * T D C G * G A K T P C C N   F1
      E W T E N D S N K I K L Q H A E K T L D E H L R E K L G L Q P K L M L P G N Y A R A L F T P E N L D I L C E L I V D E E R R H V A T  F2
     N G L R M I Q I K * S S N M Q K K L L M S I S V K N L A F S P N L C C Q V I M P V P S S L L K T L I S Y V N * L W M R S E D T M L Q   F3
1801 TGAATGGACTGAGAATGATTCAAATAAAATAAAGCTCCAACATGCAGAAAAAACTCTTGATGAGCATCTCCGTGAAAAACTTGGCCTTCAGCCCAAACTTATGCTGCCAGGTAATTATGCCCGTGCCCTCTTCACTCCTGAAAACCTTGATATCCTATGTGAACTGATTGTGGATGAGGAGCGAAGACACCATGTTGCAA 2000
     ----:----|----:----|----:----|----:----|----:----|----:----|----:----|----:----|----:----|----:----|----:----|----:----|----:----|----:----|----:----|----:----|----:----|----:----|----:----|----:----|
1801 ACTTACCTGACTCTTACTAAGTTTATTTTATTTCGAGGTTGTACGTCTTTTTTGAGAACTACTCGTAGAGGCACTTTTTGAACCGGAAGTCGGGTTTGAATACGACGGTCCATTAATACGGGCACGGGAGAAGTGAGGACTTTTGGAACTATAGGATACACTTGACTAACACCTACTCCTCGCTTCTGTGGTACAACGTT 2000
       H I S Q S H N L Y F L A G V H L F F E Q H A D G H F V Q G E A W V * A A L Y N H G H G R * E Q F G Q Y G I H V S Q P H P A F V G H Q L  F6
      M F P S L I I * I F Y L E L M C F F S K I L M E T F F K A K L G F K H Q W T I I G T G E E S R F V K I * D T F Q N H I L L S S V M N C   F5
     S H V S F S E F L I F S W C A S F V R S S C R R S F S P R * G L S I S G P L * A R A R K V G S F R S I R H S S I T S S S R L C W T A    F4

       F V D I L F K A T F S V L L * N S F G V Y A R G S W E L W R D S T S D G V H T G R T F P L F Q V V * L P A * D C * A W P R D H * * Y E   F1
      L L T Y Y S R L H S V Y S C K T P L E S M P E E A G N Y G E I A L Q M A F T L G E H F P Y F R W S N Y L H K I V E H G Q E I I D D T  F2
     L C * H T I Q G Y I Q C T P V K L L W S L C Q R K L G T M E R * H F R W R S H W E N I S L I S G G L I T C I R L L S M A K R S L M I R   F3
2001 CTTTGTTGACATACTATTCAAGGCTACATTCAGTGTACTCCTGTAAAACTCCTTTGGAGTCTATGCCAGAGGAAGCTGGGAACTATGGAGAGATAGCACTTCAGATGGCGTTCACACTGGGAGAACATTTCCCTTATTTCAGGTGGTCTAATTACCTGCATAAGATTGTTGAGCATGGCCAAGAGATCATTGATGATACG 2200
     ----:----|----:----|----:----|----:----|----:----|----:----|----:----|----:----|----:----|----:----|----:----|----:----|----:----|----:----|----:----|----:----|----:----|----:----|----:----|----:----|
2001 GAAACAACTGTATGATAAGTTCCGATGTAAGTCACATGAGGACATTTTGAGGAAACCTCAGATACGGTCTCCTTCGACCCTTGATACCTCTCTATCGTGAAGTCTACCGCAAGTGTGACCCTCTTGTAAAGGGAATAAAGTCCACCAGATTAATGGACGTATTCTAACAACTCGTACCGGTTCTCTAGTAACTACTATGC 2200
       K T S M S N L A V N L T S R Y F E K P T * A L P L Q S S H L S L V E S P T * P V L V N G K N * T T * N G A Y S S Q Q A H G L S * Q H Y  F6
      S Q Q C V I * P * M * H V G T F S R Q L R H W L F S P V I S L Y C K L H R E C Q S F M E R I E P P R I V Q M L N N L M A L L D N I I R   F5
     V K N V Y * E L S C E T Y E Q L V G K S D I G S S A P F * P S I A S * I A N V S P S C K G * K L H D L * R C L I T S C P W S I M S S V    F4

       * * P A Q Y W S I F Q * G G * K W Q Q T V P L H Q K V T V * V * F L * * H E R C T P F S L A V Y K Q T D T A I C S V L Q D H P V L *   F1
      S N D L H S I G A F S S E G G E S G N K L Y R F I R K S L S R S D S Y N D M R D A L L F H W L Y T S K Q I Q Y A Q C S K I I Q C C S  F2
     V M T C T V L E H F P V R G V K V A T N C T A S S E S H C L G L I P I M T * E M H S F F I G C I Q A N R Y S N M L S A P R S S S V V A   F3
2201 AGTAATGACCTGCACAGTATTGGAGCATTTTCCAGTGAGGGGGGTGAAAGTGGCAACAAACTGTACCGCTTCATCAGAAAGTCACTGTCTAGGTCTGATTCCTATAATGACATGAGAGATGCACTCCTTTTTCATTGGCTGTATACAAGCAAACAGATACAGCAATATGCTCAGTGCTCCAAGATCATCCAGTGTTGTAG 2400
     ----:----|----:----|----:----|----:----|----:----|----:----|----:----|----:----|----:----|----:----|----:----|----:----|----:----|----:----|----:----|----:----|----:----|----:----|----:----|----:----|
2201 TCATTACTGGACGTGTCATAACCTCGTAAAAGGTCACTCCCCCCACTTTCACCGTTGTTTGACATGGCGAAGTAGTCTTTCAGTGACAGATCCAGACTAAGGATATTACTGTACTCTCTACGTGAGGAAAAAGTAACCGACATATGTTCGTTTGTCTATGTCGTTATACGAGTCACGAGGTTCTAGTAGGTCACAACATC 2400
       S Y H G A C Y Q L M K W H P P H F H C C V T G S * * F T V T * T Q N R Y H C S L H V G K E N A T Y L C V S V A I H E T S W S * G T N Y  F6
      T I V Q V T N S C K G T L P T F T A V F Q V A E D S L * Q R P R I G I I V H S I C E K K M P Q I C A F L Y L L I S L G L D D L T T   F5
     L L S R C L I P A N E L S P P S L P L L S Y R K M L F D S D L D S E * L S M L S A S R K * Q S Y V L L C I C C Y A * H E L I M W H Q L    F4
```

```
     P  L  R  G  E  *  P  Q  *  E  D  M  Y  I  *  A  K  Y  H  R  L  N  S  N  T  W  F  H  I  H  E  H  V  Q  V  K  V  *  Y  I  L  I  L  C  *  T  Q  K  I  N  I  K  L  R  H  V  Y  E  C  G  P  C  E  S  V  L  R   F1
      L  C  G  E  S  N  H  N  K  R  T  C  T  Y  E  P  S  T  I  G  *  I  L  T  P  G  S  I  F  M  N  M  F  K  S  K  C  D  T  Y  L  S  F  V  E  H  K  R  *  I  S  N  F  D  M  F  M  N  V  G  L  V  N  Q  F  *  E   F2
    F  A  G  R  V  T  T  I  R  G  H  V  H  M  S  Q  V  P  *  A  E  F  *  H  L  V  P  Y  S  *  T  C  S  S  Q  S  V  I  H  T  Y  P  L  L  N  T  K  D  K  Y  Q  T  S  T  C  L  *  M  W  A  L  *  I  S  S  E   F3
2401 CCTTTGCGGGGAGAGTAACCACAATAAGAGGACATGTACATATGAGCCAAGTACCATAGGCTGAATTCTAACACCTGGTTCCATATTCATGAACATGTTCAAGTCAAAGTGTGATACATACTTATCCTTTGTTGAACACAAAAGATAAATATCAAATCTCGACATGTTTATGAATGTGGGCCTTGTGAATCAGTTCTGAG 2600
2401 GGAAACGCCCCTCTCATTGGTGTTATTCTCCTGTACATGTATACTCGGTTCATGGTATCCGACTTAAGATTGTGGACCAAGGTATAAGTACTTGTACAAGTTCAGTTTCACACTATGTATGAATAGGAAACAACTTGTGTTTTCTATTTATAGTTTGAAGCTGTACAAATACTTACACCCGGAACACTTAGTCAAGACTC 2600
    G  K  R  P  S  Y  G  C  Y  S  S  M  Y  M  H  A  L  Y  W  L  S  F  E  L  V  Q  N  W  I  *  S  C  T  *  T  L  T  H  Y  M  S  I  R  Q  Q  V  C  F  I  F  I  L  S  R  C  T  *  S  H  P  G  Q  S  D  T  R  L   F6
   A  K  A  P  L  T  V  V  I  L  P  C  T  C  I  L  W  T  G  Y  A  S  N  *  C  R  T  G  Y  E  H  V  H  E  L  *  L  T  I  C  V  *  G  K  N  F  V  F  S  L  Y  *  V  E  V  H  K  H  I  H  A  K  H  I  L  E  S   F5
     R  Q  P  S  L  L  W  L  L  L  V  H  V  Y  S  G  L  V  M  P  Q  I  R  V  G  P  E  M  N  M  F  M  N  L  D  F  H  S  V  Y  K  D  K  T  S  C  L  L  Y  I  D  F  K  S  M  N  I  F  T  P  R  T  F  *  N  Q   F4

     S  N  H  S  L  Q  N  *  D  T  V  N  *  D  K  S  I  T  V  F  G  M  *  L  Y  *  I  T  S  S  S  S  H  L  Y  M  C  I  H  F  I  F  S  F  L  F  F  S  L  A  C  F  S  G  S  F  G  R  *  T  P  T  A  Q  V  L  Q   F1
      V  T  I  A  C  K  I  R  T  R  *  I  K  T  N  Q  L  L  C  S  V  C  D  F  I  K  S  H  H  H  H  H  T  C  T  C  A  S  I  S  F  F  L  S  S  F  F  L  W  L  V  S  L  A  A  L  A  D  E  L  Q  L  L  K  F  F   F2
    K  *  P  *  P  A  K  L  G  H  G  K  L  R  Q  I  N  Y  C  V  R  Y  V  T  L  L  N  H  I  I  I  I  T  L  V  H  V  H  P  F  H  F  F  F  P  L  F  F  F  G  L  F  L  W  Q  L  W  Q  M  N  S  N  C  S  S  S  S   F3
2601 AAGTAACCATAGCCTGCAAAATTAGGACACGGTAAATTAAGACAAATCAATTACTGTGTTCGGTATGTGACTTTATTAAATCACATCATCATCATCACACTTGTACATGTGCATCCATTTCATTTTTTCTTTCCTCTTTTTTTCTTTGGCTTGTTTCTCTGGCAGCTTTGGCAGATGAACTCCAACTGCTCAAGTTCTTC 2800
2601 TTCATTGGTATCGGACGTTTTAATCCTGTGCCATTTAATTCTGTTTAGTTAATGACACAAGCCATACACTGAAATAATTTAGTGTAGTAGTAGTAGTAGTGTGAACATGTACACGTAGGTAAAGTAAAAAAGAAAGGAGAAAAAAAGAAACCGAACAAAGAGACCGTCGAAACCGTCTACTTGAGGTTGACGAGTTCAAGAAG 2800
     L  L  W  L  R  C  F  *  S  V  T  F  *  S  L  D  I  V  T  N  P  I  H  S  *  *  I  V  D  D  D  D  C  K  Y  M  H  M  W  K  M  K  E  K  R  K  K  E  K  A  Q  K  E  P  L  K  P  L  H  V  G  V  A  *  T  R   F6
    F  Y  G  Y  G  A  F  N  P  C  P  L  N  L  C  I  L  *  Q  T  R  Y  T  V  K  N  F  *  M  M  M  M  V  S  T  C  T  C  G  N  *  K  K  K  G  R  K  K  K  P  K  N  R  Q  C  S  Q  C  I  F  E  L  Q  E  L  E  E   F5
   S  T  V  M  A  Q  L  I  L  V  R  Y  I  L  V  F  *  N  S  H  E  T  H  S  K  I  L  D  C  *  *  *  *  V  Q  V  H  A  D  M  E  N  K  R  E  E  K  K  R  Q  S  T  E  R  A  A  K  A  S  S  S  W  S  S  L  N  K   F4

     C  L  V  P  Q  K  D  H  Y  S  K  S  E  T  T  F  H  T  P  H  H  I  A  T  K  L  Q  F  P  L  G  *  N  G  H  *  Q  H  I  P  L  L  D  I  D  L  A  P  F  V  F  Q  S  E  Q  M  C  V  G  W  H  P  H  G  K  I   F1
    S  V  W  F  P  R  K  T  T  T  A  K  V  K  P  L  F  T  L  P  I  T  L  Q  P  S  Y  S  F  P  W  D  E  M  V  I  N  N  T  F  P  C  L  T  L  T  W  L  H  L  F  F  S  Q  N  K  C  V  L  V  G  T  P  M  E  K  Y   F2
     V  S  G  S  P  E  R  P  L  Q  Q  K  *  N  H  F  S  H  S  P  S  H  C  N  Q  V  T  V  S  P  G  M  K  W  S  L  T  T  H  S  L  A  *  H  *  L  G  S  I  C  F  S  V  R  T  N  V  C  W  L  A  P  P  W  K  N  I   F3
2801 AGTGTCTGGTTCCCCAGAAAGACCACTACAGCAAAAGTGAAACCACTTTTCACACTCCCCATCACATTGCAACCAAGTTACAGTTTCCCCTGGGATGAAATGGTCATTAACAACACATTCCCTTGCTTGACATTGACTTGGCTCCATTTGTTTTTCAGTCAGAACAAATGTGTGTTGGTTGGCACCCCCATGGAAAAATA 3000
2801 TCACAGACCAAGGGGTCTTTCTGGTGATGTCGTTTTCACTTTGGTGAAAAGTGTGAGGGGTAGTGTAACGTTGGTTCAATGTCAAAGGGGACCCTACTTTACCAGTAATTGTTGTGTAAGGGAACGAACTGTAACTGAACCGAGGTAAACAAAAAGTCAGTCTTGTTTACACACAACCAACCGTGGGGGTACCTTTTTAT 3000
    *  H  R  T  G  W  F  S  L  V  V  V  A  F  T  F  G  S  K  V  S  G  M  V  N  C  G  L  *  L  K  G  Q  S  S  I  T  M  L  L  V  N  G  Q  K  V  N  V  Q  S  W  K  N  K  L  *  F  L  H  T  N  T  P  V  G  M  S  F  Y   F6
     T  D  P  E  G  S  L  G  S  C  C  F  H  F  W  K  E  C  E  G  D  C  Q  L  W  T  V  T  E  G  P  I  F  H  D  N  V  V  C  E  R  A  Q  C  Q  S  P  E  M  Q  K  E  T  L  V  F  T  H  Q  N  A  G  G  H  F  F   F5
    L  T  Q  N  G  L  F  V  V  V  A  F  T  F  G  S  K  V  S  G  M  V  N  C  G  L  *  L  K  G  Q  S  S  I  T  M  L  L  V  N  G  Q  K  V  N  V  Q  S  W  K  N  K  L  *  F  L  H  T  N  T  P  V  G  M  S  F  Y   F4

     L  N  P  S  P  N  K  L  S  G  D  *  P  F  Q  T  L  Q  E  L  L  S  S  K  Q  Q  K  C  P  N  *  *  *  S  T  H  F  E  V  L  C  P  G  C  D  S  H  Q  R  L  E  L  N  F  G  C  R  D  W  L  C  A  P  I  D  G  H   F1
      *  I  R  V  Q  I  S  C  L  V  T  S  H  F  K  P  Y  R  S  Y  F  P  Q  N  N  R  S  V  Q  T  D  N  E  V  L  I  L  R  F  F  V  L  V  V  I  A  T  K  D  *  N  *  T  L  V  A  E  I  G  C  V  P  R  *  M  D  I   F2
     E  S  E  S  K  *  V  V  W  *  L  A  I  S  N  P  T  G  A  T  F  L  K  T  T  E  V  S  K  L  I  M  K  Y  S  F  *  G  S  L  S  W  L  *  *  P  P  K  T  R  I  E  L  W  L  Q  R  L  A  V  C  P  D  R  W  T   F3
3001 TTGAATCCGAGTCCAAATAAGTTGTCTGGTGACTAGCCATTTCAAACCCTACAGGAGCTACTTTCCTCAAAACAACAGAAGTGTCCAAACTGATAATGAAGTACTCATTTTGAGGTTCTTTGTCCTGGTTGTGATAGCCACCAAAGACTAGAATTGAACTTTGGTTGCAGAGATTGGCTGTGTGCCCCGATAGATGGACA 3200
3001 AACTTAGGCTCAGGTTTATTCAACAGACCACTGATCGGTAAAGTTTGGGATGTCCTCGATGAAAGGAGTTTTGTTGTCTTCACAGGTTTGACTATTACTTCATGAGTAAAACTCCAAGAAACAGGACCAACACTATCGGTGGTTTCTGATCTTAACTTGAAACCAACGTCTCTAACCGACACACGGGGCTATCTACCTGT 3200
     N  F  G  L  G  F  L  N  D  P  S  *  G  N  *  V  R  C  S  S  S  E  E  F  C  C  F  H  G  F  Q  Y  H  L  V  *  K  S  T  R  Q  G  P  Q  S  L  W  W  L  S  S  N  F  K  P  Q  L  S  Q  S  H  A  G  I  S  P  C   F6
    I  S  D  S  D  L  Y  T  T  Q  H  S  A  M  E  F  G  V  P  A  V  K  R  L  V  V  S  T  D  L  S  I  I  F  Y  E  N  Q  P  E  K  D  Q  N  H  Y  G  G  F  V  L  I  S  S  Q  N  C  L  N  A  T  H  G  S  L  H  V   F5
   Q  I  R  T  W  I  L  Q  R  T  V  L  W  K  L  G  *  L  L  *  K  G  *  F  L  L  T  W  V  S  L  S  T  S  M  K  L  N  K  T  R  T  T  I  A  V  L  S  *  F  Q  V  K  T  A  S  I  P  Q  T  G  R  Y  I  S   F4

     H  C  S  L  V  H  *  M  L  V  L  V  *  S  E  *  Q  S  V  H  L  L  L  N  G  T  V  P  S  E  Y  R  E  Q  I  C  R  Y  S  Q  H  F  H  H  H  L  P  S  G  P  D  H  Q  *  R  Q  C  M  S  P  L  F  L  Y  Q  C  G   F1
      I  V  H  W  S  T  K  C  *  F  W  F  S  Q  S  N  S  Q  Y  T  F  S  *  M  A  L  C  H  Q  N  I  E  N  R  F  A  V  I  V  N  I  S  I  I  I  C  P  L  D  Q  T  T  N  K  D  N  A  *  V  P  Y  F  C  T  S  V   F2
     S  L  F  I  G  P  L  N  V  S  F  G  L  V  R  V  T  V  S  T  P  S  L  K  W  H  C  A  I  R  I  S  R  T  D  L  P  L  *  S  T  F  P  S  S  S  A  L  W  T  R  P  P  I  K  T  M  H  E  S  L  I  S  V  P  V  W   F3
3201 TCATTGTTCATTGGTCCACTAAATGTTAGTTTTGGTTTAGTCAGAGTAACAGTCAGTACACCTTCTCTTAAATGGCACTGTGCCATCAGAATATCGAGAACAGATTTGCCGTTATAGTCAACATTTCCATCATCATCTGCCCTCTGGACCAGACCACCAATAAAGACAATGCATGAGTCCCTTATTTCTGTACCAGTGTG 3400
3201 AGTAACAAGTAACCAGGTGATTTACAATCAAAACCAAATCAGTCTCATTGTCAGTCATGTGGAAGAGAATTTACCGTGACACGGTAGTCTTATAGCTCTTGTCTAAACGGCAATATCAGTTGTAAAGGTAGTAGTAGACGGGAGACCTGGTCTGGTGGTTATTTCTGTTACGTACTCAGGGAATAAAGACATGGTCACAC 3400
    *  Q  E  N  T  W  *  I  N  T  K  T  *  D  S  Y  C  D  T  C  R  R  K  F  P  V  T  G  D  S  Y  R  S  C  I  Q  R  *  L  *  C  K  W  *  *  R  G  E  P  G  S  W  W  Y  L  C  H  M  L  G  K  N  R  Y  W  H   F6
     D  N  N  M  P  G  S  F  T  L  K  P  K  T  L  T  V  T  L  V  G  E  R  L  H  C  Q  A  M  L  I  D  L  V  S  K  G  N  Y  D  V  N  G  D  D  D  A  R  Q  V  L  G  G  I  F  V  I  C  S  D  R  I  E  T  G  T  H   F5
   M  M  T  *  Q  D  V  L  H  *  N  Q  N  L  *  L  L  *  Y  V  K  E  *  I  A  S  H  W  *  F  I  S  F  L  N  A  T  I  T  L  M  E  M  M  M  Q  G  R  S  W  V  V  L  L  S  L  A  H  T  G  *  K  Q  V  L  T   F4

     M  P  R  D  K  V  L  L  L  P  P  E  H  S  L  L  S  I  L  N  P  R  N  S  R  S  T  D  C  C  L  W  C  Y  E  F  L  S  D  V  H  S  H  L  L  Q  F  H  H  T  I  M  H  Q  *  L  I  H  C  D  W  I  L  L  H  S   F1
    V  C  H  G  T  K  S  F  S  C  P  L  N  I  H  F  C  P  F  *  I  L  E  I  Q  E  V  Q  I  V  V  S  G  A  M  N  F  *  V  M  F  I  H  I  Y  F  N  S  T  I  Q  *  C  T  N  D  *  S  I  V  T  G  F  Y  C  I  Q   F2
     Y  A  T  G  Q  S  P  S  P  A  P  *  T  F  T  S  V  H  F  K  S  S  K  F  K  K  Y  R  L  L  S  L  V  L  *  I  S  E  *  C  S  F  T  F  T  S  I  P  P  Y  N  N  A  P  M  I  D  P  L  *  L  D  F  T  A  F  R   F3
3401 GTATGCCACGGGACAAAGTCCTTCTCCTGCCCCCTGAACATTCACTTCTGTCCATTTTAAATCCTCGAAATTCAAGAAGTACAGATTGTTGTCTCTGGTGCTATGAATTTCTGAGTGATGTTCATTCACATTTACTTCAATTCCACCATACAATAATGCACCAATGATTGATCCATTGTGACTGGATTTTACTGCATTCA 3600
3401 CATACGGTGCCCTGTTTCAGGAAGAGGACGGGGGACTTGTAAGTGAAGACAGGTAAAATTTAGGAGCTTTAAGTTCTTCATGTCTAACAACAGAGACCACGATACTTAAAGACTCACTACAAGTAAGTGTAAATGAAGTTAAGGTGGTATGTTATTACGTGGTTACTAACTAGGTAACACTGACCTAAAATGACGTAAGT 3600
    P  I  G  R  S  L  T  R  R  R  G  G  S  C  E  S  R  D  M  K  F  G  R  F  E  L  L  V  S  Q  Q  R  Q  H  *  S  N  R  L  S  T  *  E  C  K  S  *  N  W  W  V  I  I  C  W  H  N  I  W  Q  S  Q  I  K  S  C  E   F6
     Y  A  V  P  C  L  G  E  G  A  G  Q  V  N  V  E  T  W  K  L  D  E  F  N  L  F  Y  L  N  N  D  R  T  S  H  I  E  S  H  H  E  N  V  N  V  E  I  G  G  Y  L  L  A  G  I  I  S  G  N  H  S  S  K  V  A  N   F5
   T  H  W  P  V  F  D  K  E  Q  G  R  F  M  *  K  Q  G  N  *  I  R  S  I  *  S  T  C  I  T  T  E  P  A  I  F  K  Q  T  I  N  M  *  M  *  K  L  E  V  M  C  Y  H  V  L  S  Q  D  M  T  V  P  N  *  Q  M  *   F4

     E  C  D  R  I  W  E  K  E  L  F  L  S  A  A  S  *  L  E  V  R  H  N  Y  *  P  M  L  H  C  T  C  R  H  R  T  I  L  K  S  S  N  R  C  G  V  P  C  G  *  G  P  S  L  P  T  Q  K  P  Q  S  F  *  Q  F  W  H   F1
      S  V  T  V  S  G  K  R  N  C  S  C  L  L  R  L  S  W  K  F  A  T  T  T  D  Q  C  C  I  V  L  A  A  T  V  R  F  *  N  H  Q  I  A  V  G  F  H  A  G  R  V  H  P  S  P  H  K  N  L  S  L  F  S  S  S  G  M   F2
     V  *  P  Y  L  G  K  G  T  V  P  V  C  V  L  V  G  S  S  P  Q  L  L  T  N  V  A  L  Y  L  P  P  P  Y  D  S  K  I  I  K  S  L  W  G  S  M  R  V  G  S  I  P  P  H  T  K  T  S  V  F  L  A  V  L  A   F3
3601 GAGTGTGACCGTATCTGGGAAAAGGAACTGTTCCTGTCTGCTGCGTCTTAGTTGGAAGTCGCCACAACTACTGACCAATGTTGCATTGTACTTGCCGCCACCGTACGATTCTAAAATCATCAAATCGCTGTGGGGTTCCATGCGGGTAGGGTCCATCCCTCCCCACACAAAAACCTCAGTCTTTTTAGCAGTTCTGGCA 3800
3601 CTCACACTGGCATAGACCCTTTTCCTTGACAAGGACAGACGACGCAGAATCAACCTTCAAGCGGTGTTGATGACTGGTTACAACGTAACATGAACGGCGGTGGCATGCTAAGATTTTAGTAGTTTAGCGACACCCCAAGGTACGCCCATCCCAGGTAGGGAGGGGTGTGTTTTTGGAGTCAGAAAAATCGTCAAGACCGT 3800
     S  H  S  R  I  Q  S  F  S  S  N  R  D  A  A  D  *  N  S  T  R  W  L  *  Q  G  I  N  C  Q  V  Q  R  W  R  V  I  R  F  D  D  F  R  Q  P  T  G  H  P  Y  P  G  D  R  G  V  C  F  G  *  D  K  *  C  N  Q  C   F6
    L  T  H  G  Y  R  P  F  P  V  T  G  T  Q  Q  T  K  T  P  L  E  G  C  S  S  V  L  T  A  N  Y  K  G  G  G  Y  S  E  L  I  M  L  D  S  H  P  E  M  R  T  P  D  M  G  G  W  V  F  V  E  T  K  K  A  T  R  A   F5
   L  T  V  T  D  P  F  L  F  Q  E  Q  R  S  R  R  L  Q  F  N  A  V  V  V  S  W  H  Q  M  T  S  A  A  V  T  R  N  *  F  *  *  I  A  T  P  N  W  A  P  L  T  W  G  E  G  C  L  F  R  L  R  K  L  L  E  P   F4

     G  D  G  V  W  Q  G  Q  S  *  P  L  C  Q  Q  T  E  R  Q  L  L  N  E  E  T  D  G  D  Q  F  V  P  H  H  I  *  L  L  K  F  F  P  L  E  C  H  P  T  L  P  *  N  H  T  G  R  L  H  P  L  G  K  P  M  V  V  D   F1
      A  T  A  S  G  R  G  S  P  D  P  C  V  N  R  P  N  D  S  C  *  M  K  K  R  T  E  T  N  L  C  P  T  T  F  N  S  *  N  F  F  H  L  N  V  I  Q  L  Y  L  E  I  I  L  V  D  C  T  R  L  G  N  Q  W  *  S   F2
     W  R  R  L  A  G  A  V  L  T  L  V  S  T  D  R  T  T  A  A  E  *  N  G  R  R  P  I  C  A  P  P  H  L  T  P  E  I  F  S  T  *  M  S  S  N  S  T  L  K  S  Y  W  *  I  A  P  A  W  E  T  N  G  S  R   F3
3801 TGGCGACGGCGTCTGGCAGGGGCAGTCCTGACCCTTGTGTCAACAGACCGAACGACAGCTGCTGAATGAAGAAACGGACGGAGACCAATTTGTGCCCCACCACATTTAACTCCTGAAATTTTTTCCACTTGAATGTCATCCAACTCTACCTTGAAATCATACTGGTAGATTGCACCCGCTTGGGAAACCAATGGTAGTCG 4000
3801 ACCGCTGCCGCAGACCGTCCCCGTCAGGACTGGGAACACAGTTGTCTGGCTTGCTGTCGACGACTTACTTCTTTGCCTGCCTCTGGTTAAACACGGGGTGGTGTAAATTGAGGACTTTAAAAAAGGTGAACTTACAGTAGGTTGAGATGGAACTTTAGTATGACCATCTAACGTGGGCGAACCCTTTGGTTACCATCAGC 4000
     P  S  P  T  Q  C  P  C  D  Q  G  K  H  *  C  V  S  R  C  S  S  F  S  S  V  S  P  S  W  N  T  G  W  W  M  *  S  R  F  N  K  G  S  S  H  *  G  V  R  G  Q  F  *  V  P  L  N  C  G  S  P  F  G  I  T  T   F6
    H  R  R  R  R  A  P  A  T  R  V  R  T  D  V  S  R  V  V  A  A  S  H  L  F  P  R  L  G  I  Q  A  G  G  C  K  V  G  S  I  K  E  V  Q  I  D  D  L  E  V  K  F  D  Y  Q  Y  I  A  G  A  Q  S  V  L  P  L  R   F5
   M  A  V  A  D  P  L  P  L  G  S  G  Q  T  L  L  G  F  S  L  Q  Q  I  F  F  R  V  S  V  L  K  H  G  V  V  N  L  E  Q  F  K  K  W  K  F  T  M  W  S  *  R  S  I  M  S  T  S  Q  V  R  K  P  F  W  H  Y  D   F4

     R  A  S  N  Q  A  H  K  R  S  L  F  L  T  L  T  H  H  P  P  R  N  H  *  A  C  Q  H  H  V  H  D  D  V  L  L  L  L  C  R  R  H  D  D  Q  F  Q  D  H  L  H  F  Y  R  R  I  R  S  T  L  G  D  P  Y  Q  K   F1
      I  V  H  P  T  K  H  T  N  A  H  F  F  *  L  *  P  I  I  H  V  I  R  P  V  N  I  M  Y  M  M  Y  C  C  C  C  V  V  M  M  T  S  F  R  I  T  C  T  S  I  A  V  F  G  P  H  *  V  T  L  T  R  K   F2
     S  C  I  Q  P  S  T  Q  T  L  T  F  F  D  S  D  P  S  S  T  T  *  S  L  G  L  S  T  S  C  T  *  *  C  T  A  A  A  V  S  S  S  *  *  P  V  S  G  S  L  A  L  L  S  P  Y  S  V  H  I  R  *  P  L  P  E  N   F3
4001 ATCGTGCATCCAACCAAGCACACAAACGCTCACTTTTTTTGACTCTGACCCATCATCCACCACGTAATCATTAGGCCTGTCAACATCATGTACATGATGATGTACTGCTGCTGCTGTGTCGTCGTCATGATGACCAGTTTCAGGATCACTTGCACTTCTATCGCCGTATTCGGTCCACATTAGGTGACCCTTACCAGAAA 4200
4001 TAGCACGTAGGTTGGTTCGTGTGTTTGCGAGTGAAAAAAACTGAGACTGGGTAGTAGGTGGTGCATTAGTAATCCGGACAGTTGTAGTACATGTACTACTACATGACGACGACGACGACACAGCAGCAGTACTACTGGTCAAAGTCCTAGTGAACGTGAAGATAGCGGCATAAGCCAGGTGTAATCCACTGGGAATGGTCTTT 4200
     S  R  A  D  L  W  A  C  L  R  E  S  K  K  V  R  V  W  *  G  G  R  L  *  *  A  Q  *  C  *  T  C  S  S  T  S  S  S  S  H  R  R  *  S  S  W  N  *  S  *  K  C  K  *  R  R  I  R  D  V  N  P  S  G  *  W  F   F6
    D  H  M  W  G  L  V  C  V  S  V  K  K  S  E  S  G  D  D  V  V  Y  D  N  P  R  D  V  D  H  V  H  H  V  A  A  A  T  D  D  D  H  H  G  T  E  P  D  S  A  S  R  D  G  Y  E  T  W  M  L  H  G  K  G  S   F5
   I  T  C  G  V  L  C  V  F  A  *  K  K  Q  S  Q  G  M  M  W  W  T  I  M  L  G  T  L  M  M  Y  M  I  I  Y  Q  Q  Q  Q  T  T  T  M  I  V  L  K  L  I  V  Q  V  E  I  A  T  N  P  G  C  *  T  V  R  V  L  F   F4

     T  D  I  N  S  I  Y  H  R  *  L  P  F  L  *  V  S  S  A  S  L  L  P  V  *  V  K  K  S  E  N  Y  P  P  F  P  G  C  *  Q  L  S  P  P  C  L  F  X   F1
      Q  T  *  T  R  Y  I  I  A  D  C  L  F  C  K  F  P  P  L  L  Y  C  P  Y  E  *  K  K  A  K  I  I  P  H  F  R  V  V  D  N  C  R  R  H  V  C  S  X   F2
     R  H  K  L  D  I  S  S  L  T  A  F  V  S  F  L  R  F  F  I  A  R  M  S  E  K  K  R  K  L  S  P  I  S  G  L  L  T  T  V  A  A  M  F  V  Q   F3
4201 ACAGACATAAACTCGATATATCATCGCTGACTGCCTTTTTTGTAAGTTTCCTCCGCTTCTTTATTGCCCGTATGAGTGAAAAAAAGCGAAAATTATCCCCCATTTCCGGGTTGTTGACAACTGTCGCCGCCATGTTTGTTCAA 4343
4201 TGTCTGTATTTGAGCTATATAGTAGCGACTGACGGAAAAAACATTCAAAGGAGGCGAAGAAATAACGGGCATACTCACTTTTTTTCGCTTTTAATAGGGGGTAAAGGCCCAACAACTGTTGACAGCGGCGGTACAAACAAGTT 4343
     V  S  M  F  E  I  Y  *  R  Q  S  G  K  K  Y  T  E  E  A  E  K  N  G  T  H  T  F  F  L  S  F  *  G  G  N  G  P  Q  Q  C  S  D  G  G  H  K  N  L   F6
    F  L  C  L  S  S  I  D  D  S  V  A  K  K  T  L  K  R  R  K  K  I  A  R  I  L  S  F  F  R  F  N  D  G  M  E  P  N  N  V  V  T  A  A  M  N  T  *   F5
   C  V  Y  V  R  Y  I  M  A  S  Q  R  K  Q  L  N  G  G  S  R  *  Q  G  Y  S  H  F  F  A  F  I  I  G  W  K  R  T  T  S  L  Q  R  R  W  T  Q  E   F4
```

RAG1L

>WGS: NMRB01002297.1:61607-64500
>TSA: GFRY01002319.1:beg-end

```
               1                                                                                                                                                                                                    200
NMRB01002297   GAGAGTCATCATGGCAAGTAGTGCTGCTGCTCACCAGGAGGTCTTAAAGTGCCTTTGTCGTCTTTGCGCTGAGGACCTGCAGTCAAGAGCTGTTGTACGGCAGAAATCCTTTCGATCAGTTGAAAAGCATGAAGAGACTCTGTTGCTGCATTTTGTATTGATGTTAATAAGGAGGATTCTGAAATATTTCCAAAGTTAC
GFRY01002319
 Consensus     ........................................................................................................................................................................................................

               201                                                                                                                                                                                                  400
NMRB01002297   TGTGCAATAAATGCAGATCTAAGTTGGACCGACTTAACAGCAAGTCTGATCCACAGCATTTGGAATCTCCTGTATTTTCTAGCCACCAAGAAATTGACTGTTTCTGTTTAGAATTAATCAATAGGATGGGAATCGATGTACCATCAGAACCGGAGCCCTTGGAAATCGGCATTGCTGACGTTACTGCTGTTGATCATCCA
GFRY01002319
 Consensus     ........................................................................................................................................................................................................

               401                                                                                                                                                                                                  600
NMRB01002297   TACACTGGTGGTGATCATTCTGCTACAAGTACACGAGGTTCTTCAGCAGCCAATCCAACTGTCCGCCGTGCAAAAAGGGCAAGCCTTAGAGAAGCCAACCGTCGATATGTGAAAGGGGGAACGACTTAAGGATTTAATAACAAGTGTGAACAGTTTCTGTGAAGAACGTGATGAAGATACATTGGATGCACTGTTTTTTAT
GFRY01002319                                                                                                                                                                                     GTTTCTGTGAAGAACGTGATGAAGATACATTGGATGCACTGTTTTTTAT
 Consensus     .........................................................................................................................................................GTTTCTGTGAAGAACGTGATGAAGATACATTGGATGCACTGTTTTTTAT

               601                                                                                                                                                                                                  800
NMRB01002297   GTTGTCACAAAAACTGATTGATCAGGGTAGGAAAGAAGAGGCTGACAAAATTTTGTCTCTATGGAATTGTGACAGTCAGGACAGCTCTGCAGAGAAACTAACAATGACCCCAGAGGAGTGTCTGGCCATGAGAGTCAGAACTTTACAGTCAAAGGGCCACTATAGGTCAGTTTATAAAGATTCAGACGGAGAAAACTAGTT
GFRY01002319   GTTGTCACAAAAACTGATTGATCAGGGTAGGAAAGAAGAGGCTGACAAAATTTTGTCTCTATGGAATTGTGACAGTCAGGACAGCTCTGCAGAGAAACTAACAATGACCCCAGAGGAGTGTCTGGCCATGAGAGTCAGAACTTTACAGTCAAAGGGCCACTATAGGTCAGTTTATAAAGATTCAGACGGAGAAAACTAGTT
 Consensus     GTTGTCACAAAAACTGATTGATCAGGGTAGGAAAGAAGAGGCTGACAAAATTTTGTCTCTATGGAATTGTGACAGTCAGGACAGCTCTGCAGAGAAACTAACAATGACCCCAGAGGAGTGTCTGGCCATGAGAGTCAGAACTTTACAGTCAAAGGGCCACTATAGGTCAGTTTATAAAGATTCAGACGGAGAAAACTAGTT

               801                                                                                                                                                                                                  1000
NMRB01002297   GTAAAACATTGTGCCCTCCAGGTAAACTAAATGCTATTGAACACACCTTTTTGCCTGGCCATTGTGAGTATGAGTTGGTAACCGAGGATGGTGAAACCCTTGTAGCACAGCAGGCCATGTTAAGTGAGGAGACTCTGAACCAAAAACTATCCTAGGTGCTTTCAATTCATTTCCGAGTGATTTTCCACTTCCAAATGTC
GFRY01002319   GTAAAACATTGTGCCCTCCAGGTAAACTAAATGCTATTGAACACACCTTTTTGCCTGGCCATTGTGAGTATGAGTTGGTAACTGAGGATGGTGAAACCCTTGTAGCACAGCAGGCCATGTTAAGTAAGGAGACTCC--------AAGTATCCTAGGTGCTTTCAATTCATTTCCGAGTGATTTTCCACTTCCAAATGTC
 Consensus     GTAAAACATTGTGCCCTCCAGGTAAACTAAATGCTATTGAACACACCTTTTTGCCTGGCCATTGTGAGTATGAGTTGGTAACcGAGGATGGTGAAACCCTTGTAGCACAGCAGGCCATGTTAAGTaAGGAGACTCc........AActATCCTAGGTGCTTTCAATTCATTTCCGAGTGATTTTCCACTTCCAAATGTC

               1001                                                                                                                                                                                                 1200
NMRB01002297   CATGGAGTACAATTCAGTTACATTTCGTGTGTGGCTAAGGCACTACAGGAGTTGGAGAATGTTATTTCAGAAAAATTAACATCTCTTGGTTTACCAATCTCCACTAAGGTTGAAACCCTGATCAAGGATGGGGGTGATGGTCTTGGGGATGTGGACGTAAAAAAAAGGAAAGGGGATCATGCACTTACGGACAAAGTGTT
GFRY01002319   CATGGAGTACAATTCAGTTACATTTCGTGTGTGGCTAAGGCACTACAGGAGTTGGAGAATGTTATTTCAGAAAAATTAACATCTCTTGGTTTACCAATCTCCACTAAGGTTGAAACCCTGATCAAGGATGGGGGTGATGGTCTTGGGGATGTGGACGTAAAAAAAAGGAAAGGGGATCATGCACTTACGGACAAAGTGTT
```

```
 Consensus    CATGGAGTACAATTCAGTTACATTTCGTGTGTGGCTAAGGCACTACAGGAGTTGGAGAATGTTATTTCAGAAAAATTAACATCTCTTGGTTTACCAATCTCCACTAAGGTTGAAACCCTGATCAAGGATGGGGGTGATGGTCTTGGGGATGTGGACGTAAAAAAAAGGAAAGGGGATCATGCACTTACGGACAAAGTGTT

              1201                                                                                                                                                                                                    1400
NMRB01002297  TCGATATTCGTTTTGTATTCTGACCTGCACCGCACTCATAGATGGAAAACGAGTGGTTTTGTTTCGTGAAGAACATCCAAACTCTGTGGACTGCACAAGACCTGTCGTCGTGGCTAATTGTGATGAAAGTGACCAACCAAGCATTGCCCTCACTGTGAAGGATGTGTGTAGATCTCGCGAAATTATGAAAAATAATGTGA
GFRY01002319  TCGATATTCGTTTTGTATTCTGACCTGCACCGCACTCATAGATGGAAAACGAGTGGTTTTGTTTCGTGAAGAACATCCAAACTCTGTGGACTGCACAAGACCTGTCGTCGTGGCTAATTGTGATGAAAGTAATCAACCAAGCATTGCCCTCACTGTGAAGGATGCGTCTAGATCTCGTGAAATTATGAAAAATAATGTGA
 Consensus    TCGATATTCGTTTTGTATTCTGACCTGCACCGCACTCATAGATGGAAAACGAGTGGTTTTGTTTCGTGAAGAACATCCAAACTCTGTGGACTGCACAAGACCTGTCGTCGTGGCTAATTGTGATGAAAGTaAcCAACCAAGCATTGCCCTCACTGTGAAGGATGcGTcTAGATCTCGcGAAATTATGAAAAATAATGTGA

              1401                                                                                                                                                                                                    1600
NMRB01002297  TGAAGTTGCTTTCCTCAGGGACTGTTGTGAGAGAACATCACCTCAAATTTATGTACACAATGGTAGATGAAAAATTTGAGCGCACCTCGTGTGGTCTCCAGGGGAGTGGGTCTCGGTTCATCTGCACATTGTGCTGTGCCACAAGCGCCAACTGTCAGGCTGATTGTGGGTCATTTAGCCGCACAAGAACCAATGATGAA
GFRY01002319  TGAAGTTGCTTTCCTCAGGGACTGTTGTGAGAGAACATCACCTCAAATTTATGTACACAATGGTGGGTGAAAAATTTGAGCGCACCTCGTGTGGTCTCCAGGGGAGTGGGTCTCGGTTCATCTGCACATTGTGCCGTGCCACAAGCGCCAACTGTCAGGCTGATTGTGGGTCCTATAGCCGCACAAGAACCAATGATGAA
 Consensus    TGAAGTTGCTTTCCTCAGGGACTGTTGTGAGAGAACATCACCTCAAATTTATGTACACAATGGTaGaTGAAAAATTTGAGCGCACCTCGTGTGGTCTCCAGGGGAGTGGGTCTCGGTTCATCTGCACATTGTGCcGTGCCACAAGCGCCAACTGTCAGGCTGATTGTGGGTCaTaTAGCCGCACAAGAACCAATGATGAA

              1601                                                                                                                                                                                                    1800
NMRB01002297  ACTGTACGTCTCTACAAATATCGTCTTGAGAACCCAGACAACCTCTCTGAAAATGATCTTGCAGAAAGGACAAAAGGTGTCAAAAGCATGCCAATGATATTTTGTGATGCAACGGAACGCTGTTTTGATGCAACCCATGCAAATATAAACATGGGCAGGGTTCTCAAGAAGACACTTGTACGAGAAATAGCTGGGGTACA
GFRY01002319  ACTGTACGTCTCTACAAATATCGTCTTGAGAACCCAGACAACCTCTCTGAAAATGATCTTGCAGAAAGGACAAAAGGTGTCAAAAGCATGCCAATGATATTTTGTGATGCAACGGAACGCTGTTTTGATGCAACCCATGCAAATATAAACATGGGCAGGGTTCTCAAGAAGATACTTGTACGAGAAATAGCTGGGGTACA
 Consensus    ACTGTACGTCTCTACAAATATCGTCTTGAGAACCCAGACAACCTCTCTGAAAATGATCTTGCAGAAAGGACAAAAGGTGTCAAAAGCATGCCAATGATATTTTGTGATGCAACGGAACGCTGTTTTGATGCAACCCATGCAAATATAAACATGGGCAGGGTTCTCAAGAAGAcACTTGTACGAGAAATAGCTGGGGTACA

              1801                                                                                                                                                                                                    2000
NMRB01002297  TGAATGGACTGAGAATGATTCAAATAAAATAAAGCTCCAACATGCAGAAAAAACTCTTGATGAGCATCTCCGTGAAAAACTTGGCCTTCAGCCCAAACTTATGCTGCCAGGTAATTATGCCCGTGCCCTCTTCACTCCTGAAAACCTTGATATCCTATGTGAACTGATTGTGGATGAGGAGCGAAGACACCATGTTGCAA
GFRY01002319  TGAATGGACTGAGAATGATTCAAATAAAATAAAGCTCCAACATGCAGAAAAAACTCTTGATGAGCATCTCCGTGAAAAACTTGGCCTTCAGCCCAAACTAATGCTGCCAGGTAATTATGCCCGTGCCCTCTTCACTCCTGAAAACCTTGATATCCTATGTGAACTGATTGTGGATGAGGAGCGAAGACACCATGTTGCAA
 Consensus    TGAATGGACTGAGAATGATTCAAATAAAATAAAGCTCCAACATGCAGAAAAAACTCTTGATGAGCATCTCCGTGAAAAACTTGGCCTTCAGCCCAAACTaATGCTGCCAGGTAATTATGCCCGTGCCCTCTTCACTCCTGAAAACCTTGATATCCTATGTGAACTGATTGTGGATGAGGAGCGAAGACACCATGTTGCAA

              2001                                                                                                                                                                                                    2200
NMRB01002297  CTTTGTTGACATACTATTCAAGGCTACATTCAGTGTACTCCTGTAAAACTCCTTTGGAGTCTATGCCAGAGGAAGCTGGGAACTATGGAGAGATAGCACTTCAGATGGCGTTCACACTGGGAGAACATTTCCCTTATTTCAGGTGGTCTAATTACCTGCATAAGATTGTTGAGCATGGCCAAGAGATCATTGATGATACG
GFRY01002319  CTTTGTTGAAATACTATTCAAGGCTACATTCAGTGTACTCCTGTAAAACTCCTTTGGAGTCTATGCCAGAGGAAGCTGGGAACTATGGAGAGATAGCACTTCAGATGGCGTTCACACTGGGAGAACATTTCCCTTATTTCAGGTGGTCTAATTACCTGCATAAGATTGTTGAGCATGGCCAAGAAATCATTGATGATACG
 Consensus    CTTTGTTGAaATACTATTCAAGGCTACATTCAGTGTACTCCTGTAAAACTCCTTTGGAGTCTATGCCAGAGGAAGCTGGGAACTATGGAGAGATAGCACTTCAGATGGCGTTCACACTGGGAGAACATTTCCCTTATTTCAGGTGGTCTAATTACCTGCATAAGATTGTTGAGCATGGCCAAGAaATCATTGATGATACG

              2201                                                                                                                                                                                                    2400
NMRB01002297  AGTAATGACCTGCACAGTATTGGAGCATTTTCCAGTGAGGGGGGTGAAAGTGGCAACAAACTGTACCGCTTCATCAGAAAGTCACTGTCTAGGTCTGATTCCTATAATGACATGAGAGATGCACTCCTTTTTCATTGGCTGTATACAAGCAAACAGATACAGCAATATGCTCAGTGCTCCAAGATCATCCAGTGTTGTAG
GFRY01002319  AGTAATGACCTGCACAGTATTGGAGCATTTTCCAGTGAGGGGGGTGAAAGTGGCAACAAATTGTACCGCTTCATCAGAAAGTCACTGTCTAGGTCTGATTCCTATAATGACATGAGAGATGCACTCCTTTTTCATTGGCTGTATACAAGCAAACAGATACAGCAATATGCTCAGTGCTCCAAGATCATCCAGTGTTGTAG
 Consensus    AGTAATGACCTGCACAGTATTGGAGCATTTTCCAGTGAGGGGGGTGAAAGTGGCAACAAAcTGTACCGCTTCATCAGAAAGTCACTGTCTAGGTCTGATTCCTATAATGACATGAGAGATGCACTCCTTTTTCATTGGCTGTATACAAGCAAACAGATACAGCAATATGCTCAGTGCTCCAAGATCATCCAGTGTTGTAG

              2401                                                                                                                                                                                                    2600
NMRB01002297  CCTTTGCGGGGAGAGTAACCACAATAAGAGGACATGTACATATGAGCCAAGTACCATAGGCTGAATTCTAACACCTGGTTCCATATTCATGAACATGTTCAAGTCAAAGTGTGATACATACTTATCCTTTGTTGAACACAAAAGATAAATATCAAACTTCGACATGTTTATGAATGTGGGCCTTGTGAATCAGTTCTGAG
GFRY01002319  CCTTTGTGGGGAGAGTAACCACAATAAGAGGACATGTACATATGAGCCAAGTACCATAGGCTGAATTCTAACACCTGGTTCCATATTCATGAACATGTTTAAGTCAAAGTGTGA
 Consensus    CCTTTGcGGGGAGAGTAACCACAATAAGAGGACATGTACATATGAGCCAAGTACCATAGGCTGAATTCTAACACCTGGTTCCATATTCATGAACATGTTcAAGTCAAAGTGTGA.......................................................................................

              2601                                                                                                                                                                                                    2800
NMRB01002297  AAGTAACCATAGCCTGCAAAATTAGGACACGGTAAATTAAGACAAATCAATTACTGTGTTCGGTATGTGACTTTATTAAATCACATCATCATCATCACACTTGTACATGTGCATCCATTTCATTTTTTCTTTCCTCTTTTTTTCTTTGGCTTGTTTCTCTGGCAGCTTTGGCAGATGAACTCCAACTGCTCAAGTTCTTC
GFRY01002319
 Consensus    ........................................................................................................................................................................................................

              2801                                                                                          2894
NMRB01002297  AGTGTCTGGTTCCCCAGAAAGACCACTACAGCAAAAGTGAAACCACTTTTCACACTCCCCATCACATTGCAACCAAGTTACAGTTTCCCCTGGG
GFRY01002319
 Consensus    ..............................................................................................
```

>TSA: GFRY01002319.1:beg-end
5'3' Frame 1

VSVKNVMKIHWMHCFLCCHKN-LIRVGKKRLTKFCLYGIVTVRTALQRN-Q-PQRSVWP-ESELYSQRATIGQFIRFRRRKLVVKHCALQVN-MLLNTPFCLAIVSMSW-LRMVKPL-HSRPC-
VRKTPSILGAFNSFPSDFPLPNVHGVQFSYISCVAKALQELENVISEKLTSLGLPISTKVETLIKDGGDGLGDVDVKKRKGDHALTDKVFRYSFCILTCTALIDGKRVVLFREEHPNSVDCTRPVVVANCDESNQPSIALTVKDASRSREIMKNNVMKLLSSGTVVREHHLKFMYTMVGEKFERTSCGLQGSGSRFICTLCRATSANCQADCGSYSRTRT
NDETVRLYKYRLENPDNLSENDLAERTKGVKSMPMIFCDATERCFDATHANINMGRVLKKILVREIAGVHEWTENDSNKIKLQHAEKTLDEHLREKLGLQPKLMLPGNYARALFTPENLDILCELIVDEERRHHVATLLKYYSRLHSVYSCKTPLESMPEEAGNYGEIALQMAFTLGEHFPYFRWSNYLHKIVEHGQEIIDDTSNDLHSIGAFSSEGGE
SGNKLYRFIRKSLSRSDSYNDMRDALLFHWLYTSKQIQQYAQCSKIIQCCSLCGESNHNKRTCTYEPSTIG-ILTPGSIFMNMFKSKC

Notospermus geniculatus (Nge)

WGS: NMRB01001040.1

66004-70332
Notospermus geniculatus strain Ushimado scaffold1040, whole genome shotgun sequence

[illegible]

```
     M F S T L S I F Y E R G N N I Q I F W R E * R S S I I A S D D * F R L Q A Q M Q S * V F I K I S F S M L Q F C F V V I I L N P L M H *   F2
      C S L R S A F S M R E V T T S R F S G E N N A L A S L P V M I N L G C K P K C S R R C S S R F R S A C C N F A L L L S F S T H S C T    F3
2401 CATGTTCTCTACGCTCAGCATTTTCTATGAGAGAGGTAACAACATCCAGATTTTCTGGAGAGAATAACGCTCTAGCATCATTGCCAGTGATGATTAATTTAGGCTGCAAGCCCAAATGCAGTCGTAGGTGTTCATCAAGATTTCGTTCAGCATGCTGCAATTTTGCTTTGTTGTTATCATTCTCAACCCACTCATGCACT 2600
     ----:----|----:----|----:----|----:----|----:----|----:----|----:----|----:----|----:----|----:----|----:----|----:----|----:----|----:----|----:----|----:----|----:----|----:----|----:----|----:----|
2401 GTACAAGAGATGCGAGTCGTAAAAGATACTCTCTCCATTGTTGTAGGTCTAAAAGACCTCTCTTATTGCGAGATCGTAGTAACGGTCACTACTAATTAAATCCGACGTTCGGGTTTACGTCAGCATCCACAAGTAGTTCTAAAGCAAGTCGTACGACGTTAAAACGAAACAACAATAGTAAGAGTTGGGTGAGTACGTGA 2600
      C T R * A * C K R H S L Y C C G S K R S L I V S * C * Q W H H N I * A A L G F A T T P T * * S K T * C A A I K S Q Q * * E * G V * A S   F6
      V H E R R E A N E I L S T V V D L N E P S F L A R A D N G T I I L K P Q L G L H L R L H E D L N R E A H Q L K A K N N D N E V W E H V    F5
       M N E V S L M K * S L P L L M W I K Q L S Y R E L M M A L S S * N L S C A W I C D Y T N M L I E N L M S C N Q K T T I M R L G S M C     F4

      T P F L G Q L F F S E P Y P C L Y * R V L H L R N G L Q H L I L S V G N F * H P C I C Q P G H F L K V Y W D S Q D D S D T D L L S Q H   F1
       R H F S D N Y F F Q N P T H V Y I S V C C I * G T V C S I * F Y Q W V T F D T L A F V S Q V I F * K F I G I L K T I V I Q T Y C L S    F2
      D A I S R T I I F F R T L P M F I L A C V A S K E R S A A S D F I S G * L L T P L H L S A R S F S E S L L G F S R R * * Y R L T V S A   F3
2601 GACGCCATTTCTCGGACAATTATTTTTTTCAGAACCCTACCCATGTTTATATTAGCGTGTGTTGCATCTAAGGAACGGTCTGCAGCATCTGATTTTATCAGTGGGTAACTTTTGACACCCTTGCATTTGTCAGCCAGGTCATTTTCTGAAAGTTTATTGGGATTCTCAAGACGATAGTGATACAGACTTACTGTCTCAGC 2800
     ----:----|----:----|----:----|----:----|----:----|----:----|----:----|----:----|----:----|----:----|----:----|----:----|----:----|----:----|----:----|----:----|----:----|----:----|----:----|----:----|
2601 CTGCGGTAAAGAGCCTGTTAATAAAAAAAGTCTTGGGATGGGTACAAATATAATCGCACACAACGTAGATTCCTTGCCAGACGTCGTAGACTAAAATAGTCACCCATTGAAAACTGTGGGAACGTAAACAGTCGGTCCAGTAAAAGACTTTCAAATAACCCTAAGAGTTCTGCTATCACTATGTCTGAATGACAGAGTCG 2800
       V G N R P C N N K E S G * G H K Y * R T N C R L F P R C C R I K D T P L K Q C G Q M Q * G P * K R F T * Q S E * S S L S V S K S D *   F6
       S A M E R V I I K K L V R G M N I N A H T A D L S R D A A D S K I L P Y S K V G K C K D A L D N E S L K N P N E L R Y H Y L S V T E A   F5
      Q R W K E S L * K K * F G V W T * I L T H Q M * P V T Q L M Q N * * H T V K S V R A N T L W T M K Q F N I P I R L V I T I C V * Q R L    F4

       G F * C V E M S H G H T D K S V L L H N T M C K Y T * S H S L A I L M N T V Q T S H P P L C K * V * D D D T * V N H S P S A T V K Y    F1
      M G S S A L K * A M V T L T S L C C C I T Q C A N I P R A T P L Q S * * T P F K L L I H H C V N E S K M M T P K * T T L Q V Q L * N I   F2
       W V L V R * N E P W S H * Q V C V V A * H N V Q I Y L E P L P C N P D E H R S N F S S T I V * M S L R * * H L S E P L S K C N C K I L  F3
2801 ATGGGTTCTAGTGCGTTGAAATGAGCCATGGTCACACTGACAAGTCTGTGTTGTTGCATAACACAATGTGCAAATATACCTAGAGCCACTCCCTTGCAATCCTGATGAACACCGTTCAAACTTCTCATCCACCATTGTGTAAATGAGTCTAAGATGATGACACCTAAGTGAACCACTCTCCAAGTGCAACTGTAAAATAT 3000
     ----:----|----:----|----:----|----:----|----:----|----:----|----:----|----:----|----:----|----:----|----:----|----:----|----:----|----:----|----:----|----:----|----:----|----:----|----:----|----:----|
2801 TACCCAAGATCACGCAACTTTACTCGGTACCAGTGTGACTGTTCAGACACAACAACGTATTGTGTTACACGTTTATATGGATCTCGGTGAGGGAACGTTAGGACTACTTGTGGCAAGTTTGAAGAGTAGGTGGTAACACATTTACTCAGATTCTACTACTGTGGATTCACTTGGTGAGAGGTTCACGTTGACATTTTATA 3000
      C P N * H T S I L W P * V S L D T N N C L V I H L Y V * L W E R A I R I F V T * V E * G G N H L H T * S S S V * T F W E G L A V T F Y   F6
       H T R T R Q F S G H D C Q C T Q T T A Y C L T C I Y R S G S G Q L G S S C R E F K E D V M T Y I L R L H H C R L S G S E L H L Q L I   F5
      M P E L A N F H A M T V S V L R H Q Q M V C H A F I G L A V G K C D Q H V G N L S R M W W Q T F S D L I I V G L H V V R W T C S Y F I   F4

      Y H S W H P Y A L K Q N L T P E P Y * E H H F H Y N G K S Q L V W C S Q L S * P C L Q K I G Y R M C H S L * P * P Q E H R K S T E T P   F1
       T I H G I P M L * N R T * H Q S H T R S I T F I T M G N H N W S G A V N * V S H V F R K * D I G C V I H F S P D L K N T E R V P K H L  F2
       P F M A S L C F E T E L N T R A I L G A S L S L Q W E I T T G L V Q S T E L A M S S E N R I * D V S F T L A L T S R T Q K E Y R N T    F3
3001 TACCATTCATGGCATCCCTATGCTTTGAAACAGAACTTAACACCAGAGCCATACTAGGAGCATCACTTTCATTACAATGGGAAATCACAACTGGTCTGGTGCAGTCAACTGAGTTAGCCATGTCTTCAGAAAATAGGATATAGGATGTGTCATTCACTTTAGCCCTGACCTCAAGAACACAGAAAGAGTACCGAAACACC 3200
     ----:----|----:----|----:----|----:----|----:----|----:----|----:----|----:----|----:----|----:----|----:----|----:----|----:----|----:----|----:----|----:----|----:----|----:----|----:----|----:----|
3001 ATGGTAAGTACCGTAGGGATACGAAACTTTGTCTTGAATTGTGGTCTCGGTATGATCCTCGTAGTGAAAGTAATGTTACCCTTTAGTGTTGACCAGACCACGTCAGTTGACTCAATCGGTACAGAAGTCTTTTATCCTATATCCTACACAGTAAGTGAAATCGGGACTGGAGTTCTTGTGTCTTTCTCATGGCTTTGTGG 3200
      * W E H C G * A K F C F K V G S G Y * S C * K * * L P F D C S T Q H L * S L * G H R * F I P Y L I H * E S * G Q G * S C L F L V S V G   F6
      N G N M A D R H K S V S S L V L A M S P A D S E N C H S I V V P R T C D V S N A M D E S F L I Y S T D N V K A R V E L V C F S Y R F V   F5
       V M * P M G I S Q F L V * C W L W V L L M V K M V I P F * L Q D P A T L Q T L W T K L F Y S I P H T M * K L G S R L F V S L T G F C    F4

       Y L V K S K Q L S F F * H Q H H Q G H H P R L * L M C I Q V C P K E G Q T Q T S A Q Q L Y P Q V H T E S L Q W Q K C S * T V L Q K Y L  F1
       I W * R V S S S H S F D I N I T K A I T P V F D * C V Y R C V Q R K A K L R Q A P S N Y T L K F T Q S L C N G R S V V K L Y S R N I   F2
      L S G K E * A A L I L L T S T S P R P S P P S L I N V Y T G V S K G R P N S D K R P A I I P S S S H R V F A M A E V * L N C T P E I F  F3
3201 TTATCTGGTAAAGAGTAAGCAGCTCTCATTCTTTTGACATCAACATCACCAAGGCCATCACCCCCGTCTTTGATTAATGTGTATACAGGTGTGTCCAAAGGAAGGCCAAACTCAGACAAGCGCCCAGCAATTATACCCTCAAGTTCACACAGAGTCTTTGCAATGGCAGAAGTGTAGTTAAACTGTACTCCAGAAATATT 3400
     ----:----|----:----|----:----|----:----|----:----|----:----|----:----|----:----|----:----|----:----|----:----|----:----|----:----|----:----|----:----|----:----|----:----|----:----|----:----|----:----|
3201 AATAGACCATTTCTCATTCGTCGAGAGTAAGAAAACTGTAGTTGTAGTGGTTCCGGTAGTGGGGGCAGAAACTAATTACACATATGTCCACACAGGTTTCCTTCCGGTTTGAGTCTGTTCGCGGGTCGTTAATATGGGAGTTCAAGTGTGTCTCAGAAACGTTACCGTCTTCACATCAATTTGACATGAGGTCTTTATAA 3400
       * R T F L L C S E N K Q C * C * W P W * G R R Q N I H I C T H G F S P W V * V L A W C N Y G * T * V S D K C H C F H L * V T S W F Y  F6
       K D P L S Y A A R M R K V D V D G L G D G G D K I L T Y V P T D L P L G F E S L R G A I I G E L E C L T K A I A S T Y N F Q V G S I N  F5
      R I Q Y L T L L E * E K S M L M V L A M V G T K S * H T Y L H T W L F A L S L C A G L L * V R L N V C L R Q L P L L T T L S Y E L F I   F4

       V V E N L K E T C S I S Q V V A G V H T V P L K E F P A L * G Y H H L N * P V C T C N A Q V N I S F P T L * A A L V A E V D G H L F   F1
      W * W K I * R K L V Q F L K * L L G F T L F L * K N F L L C K D I T I * I D Q F V L A M P R * I F R F R L C K L L W W L K W T G T Y F  F2
      G S G K S E G N L F N F S S S C W G S H C S F E R I S C S V R I S P F E L T S L Y L Q C P G K Y F V S D F V S C F G G * S G R A L I L  F3
3401 TGGTAGTGGAAAATCTGAAGGAAACTTGTTCAATTTCTCAAGTAGTTGCTGGGGTTCACACTGTTCCTTTGAAAGAATTTCCTGCTCTGTAAGGATATCACCATTTGAATTGACCAGTTTGTACTTGCAATGCCCAGGTAAATATTTCGTTTCCGACTTTGTAAGCTGCTTTGGTGGCTGAAGTGGACGGGCACTTATTT 3600
     ----:----|----:----|----:----|----:----|----:----|----:----|----:----|----:----|----:----|----:----|----:----|----:----|----:----|----:----|----:----|----:----|----:----|----:----|----:----|----:----|
3401 ACCATCACCTTTTAGACTTCCTTTGAACAAGTTAAAGAGTTCATCAACGACCCCAAGTGTGACAAGGAAACTTTCTTAAAGGACGAGACATTCCTATAGTGGTAAACTTAACTGGTCAAACATGAACGTTACGGGTCCATTTATAAAGCAAAGGCTGAAACATTCGACGAAACCACCGACTTCACCTGCCCGTGAATAAA 3600
      K T T S F R F S V Q E I E * T T A P T * V T G K F S N G A R Y P Y * W K F Q G T Q V Q L A W T F I E N G V K Y A A K T A S T S P C K N   F6
        P L P F D S P F K N L K E L L Q Q P E C Q E K S L I E Q E T L I D G N S N V L K Y K C H G P L Y K T E S K T L Q K P P Q L P R A S I   F5
       Q Y H F I Q L F S T * N R L Y N S P N V S N R Q F F K R S Q L S I V M Q I S W N T S A I G L Y I N R K R S Q L S S Q H S F H V P V * K  F4

      C S E F C T F F Y I E F W T A L F S P A S Q D I H Q Q T F L T F L L S H Q C H A R E K E P C L H P S S F P N P G T F V T E * R K E N L   F1
       V L N F V H F F I L S F G L H C S H P H R K T F I S R H F * L F F * V I N V M P E R K N P V C I L L P S Q I Q E L L * Q N K E K R I Y  F2
       F * I L Y I F L Y * V L D C I V L T R I A R H S S A D I S N F S S E S S M S C Q R E R T L S A S F F L P K S R N F C D R I K K R E S    F3
3601 TGTTCTGAATTTTGTACATTTTTTTATATTGAGTTTTGGACTGCATTGTTCTCACCCGCATCGCAAGACATTCATCAGCAGACATTTCTAACTTTTCTTCTGAGTCATCAATGTCATGCCAGAGAGAAAGAACCCTGTCTGCATCCTTCTTCCTTCCCAAATCCAGGAACTTTTGTGACAGAATAAAGAAAAGAGAATCT 3800
     ----:----|----:----|----:----|----:----|----:----|----:----|----:----|----:----|----:----|----:----|----:----|----:----|----:----|----:----|----:----|----:----|----:----|----:----|----:----|----:----|
3601 ACAAGACTTAAAACATGTAAAAAAATATAACTCAAAACCTGACGTAACAAGAGTGGGCGTAGCGTTCTGTAAGTAGTCGTCTGTAAAGATTGAAAAGAAGACTCAGTAGTTACAGTACGGTCTCTCTTTCTTGGGACAGACGTAGGAAGAAGGAAGGGTTTAGGTCCTTGAAAACACTGTCTTATTTCTTTTCTCTTAGA 3800
       Q E S N Q V N K * I S N Q V A N N E G A D C S M * * C V N R V K R R L * * H * A L S F S G Q R C G E E K G F G P V K T V S Y L F S F R  F6
      K N Q I K Y M K K Y Q T K S Q M T R V R M A L C E D A S M E L K E E S D D I D H W L S L V R D A D K K R G L D L F K Q S L I F F L S D   F5
       T R F K T C K K I N L K P S C Q E * G C R L V N M L L C K * S K K Q T M L T M G S L F F G T Q M R R G E W I W S S K H C F L S F L I    F4

       L N F P G V H H K T H Q L N R S V F S T S A V * H T V D L L H V S W S S V S F L * C W V * S A H Q M W S S N Y V P E R H P T L Q Y Q *  F1
       * I F P V F I T K L I N * I D Q F F Q P L P F N I L S I C C T * V G L L S P F C S V G F D R P I R C G R V T M F Q R D I Q L Y N I N    F2
      T E F S R C S S Q N S S I E S I S F F N L C R L T Y C R F A A R K L V F C L L S V V L G L I G P S D V V E * L C S R E T S N S T I S I  F3
3801 ACTGAATTTTCCCGGTGTTCATCACAAAACTCATCAATTGAATCGATCAGTTTTTTCAACCTCTGCCGTTTAACATACTGTCGATTTGCTGCACGTAAGTTGGTCTTCTGTCTCCTTTCTGTAGTGTTGGGTTTGATCGGCCCATCAGATGTGGTCGAGTAACTATGTTCCAGAGAGACATCCAACTCTACAATATCAAT 4000
     ----:----|----:----|----:----|----:----|----:----|----:----|----:----|----:----|----:----|----:----|----:----|----:----|----:----|----:----|----:----|----:----|----:----|----:----|----:----|----:----|
3801 TGACTTAAAAGGGCCACAAGTAGTGTTTTGAGTAGTTAACTTAGCTAGTCAAAAAAGTTGGAGACGGCAAATTGTATGACAGCTAAACGACGTGCATTCAACCAGAAGACAGAGGAAAGACATCACAACCCAAACTAGCCGGGTAGTCTACACCAGCTCATTGATACAAGGTCTCTCTGTAGGTTGAGATGTTATAGTTA 4000
       S F K G P T * * L V * * N F R D T K E V E A T * C V T S K S C T L Q D E T E K R Y H Q T Q D A W * I H D L L * T G S L C G V R C Y *   F6
      V S N E R H E D C F E D I S D I L K K L R Q R K V Y Q R N A A R L N T K Q R R E T T N P K I P G D S T T S Y S H E L S V D L E V I D I  F5
      * Q I K G T N M V F S M L Q I S * N K * G R G N L M S D I Q Q V Y T P R R D G K Q L T P N S R G M L H P R T V I N W L S M W S * L I L  F4

       Q L V Q A P Q A P A P D V F S W Q S H L A Q G Q R C Q L T P A F S K V S L I C C G I C C I H V L V N C P H P R L * C G S A S G L F A    F1
      S N W F R R H K P Q L L M Y F P G K V I W P R D R D V S * L L L F R K S H * F A A A F V A Y T F W * I V L I L D F N V V V P Q D C L L   F2
      A T G S G A T S P S S * C I F L A K S S G P G T E M S A D S C F F E S L T N L L R H L L H T R F G K L S S S S T L M W * C L R I V C *  F3
4001 AGCAACTGGTTCAGGCGCCACAAGCCCCAGCTCCTGATGTATTTTCCTGGCAAAGTCATCTGGCCCAGGGACAGAGATGTCAGCTGACTCCTGCTTTTTCGAAAGTCTCACTAATTTGCTGCGGCATTTGTTGCATACACGTTTTGGTAAATTGTCCTCATCCTCGACTTTAATGTGGTAGTGCCTCAGGATTGTTTGCT 4200
     ----:----|----:----|----:----|----:----|----:----|----:----|----:----|----:----|----:----|----:----|----:----|----:----|----:----|----:----|----:----|----:----|----:----|----:----|----:----|----:----|
4001 TCGTTGACCAAGTCCGCGGTGTTCGGGGTCGAGGACTACATAAAAGGACCGTTTCAGTAGACCGGGTCCCTGTCTCTACAGTCGACTGAGGACGAAAAAGCTTTCAGAGTGATTAAACGACGCCGTAAACAACGTATGTGCAAAACCATTTAACAGGAGTAGGAGCTGAAATTACACCATCACGGAGTCCTAACAAACGA 4200
      Y C S T * A G C A G A G S T N E Q C L * R A W P C L H * S V G A K E F T E S I Q Q P M Q Q M C T K T F Q G * G R S * H P L A E P N N A   F6
       A V P E P A V L G L E Q H I K R A F D D P G P V S I D A S E Q K K S L R V L K S R C K N C V R K P L N D E D E V K I H Y H R L I T Q   F5
       L L Q N L R W L G W S R I Y K G P L T M Q G L S L S T L Q S R S K R F D * * N A A A N T A Y V N Q Y I T R M R S K L T T T G * S Q K S F4

      D V D S Q K Q M L F A L V L H A A S V D P H T A D T S P E V L S H R H P Y W V L K I Q                                                     F1
       M L I H R S K C Y L P W C C M P L Q L I P T Q P T Q V L K C F L I G I H T G C * K Y X                                                    F2
        C * F T E A N V I C L G A A C R F S * S P H S R H K S * S A F S * A S I L G A E N T X                                                   F3
4201 GATGTTGATTCACAGAAGCAAATGTTATTTGCCTTGGTGCTGCATGCCGCTTCAGTTGATCCCCACACAGCCGACACAAGTCCTGAAGTGCTTTCTCATAGGCATCCATACTGGGTGCTGAAAATACAA 4329
     ----:----|----:----|----:----|----:----|----:----|----:----|----:----|----:----|----:----|----:----|----:----|----:----|----:----
4201 CTACAACTAAGTGTCTTCGTTTACAATAAACGGAACCACGACGTACGGCGAAGTCAACTAGGGGTGTGTCGGCTGTGTTCAGGACTTCACGAAAGAGTATCCGTAGGTATGACCCACGACTTTTATGTT 4329
      S T S E C F C I N N A K T S C A A E T S G W V A S V L G S T S E * L C G Y Q T S F I C                                                     F6
      Q H Q N V S A F T I Q R P A A H R K L Q D G C L R C L D Q L A K E Y A D M S P A S F V                                                     F5
       I N I * L L L H * K G Q H Q M G S * N I G V C G V C T R F H K R M P M W V P H Q F Y L                                                    F4
```

Notospermus geniculatus (Nge)

WGS: NMRB01000727.1

42781-45829
Notospermus geniculatus strain Ushimado scaffold727, whole genome shotgun sequence

```
   C  K  Q  D  G  V  G  G  R  K  *  L  R  R  E  R  F  F  R  R  F  T  L  L  F  D  S  E  V  S  E  K  A  P  K  G  Y  K  S  I  N  S  *  *  S  K  Q  R  C  A  L  F  W  E  G  T  P  G  M  V  H  E  I  G  *  N  Y   F1
   V  N  K  M  A  S  G  G  V  S  N  S  E  E  K  D  F  F  E  D  S  R  F  Y  S  I  L  R  S  V  K  K  R  R  K  V  T  K  A  S  I  V  D  E  A  S  K  G  A  L  F  S  G  K  G  H  L  V  W  S  T  R  S  G  E  T  T   F2
   *  T  R  W  R  R  G  A  *  V  T  Q  K  R  K  I  F  S  K  I  H  A  S  I  R  F  *  G  Q  *  K  S  A  E  R  L  Q  K  H  Q  *  L  M  K  Q  A  K  V  R  S  F  L  G  R  D  T  W  Y  G  P  R  D  R  V  K  L   F3
1 TGTAAACAAGATGGCGTCGGGGGGCGTAAGTAACTCAGAAGAGAAAGATTTTTTCGAAGATTCACGCTTCTATTCGATTCTGAGGTCAGTGAAAAAGCGCCGAAAGGTTACAAAAGCATCAATAGTTGATGAAGCAAGCAAAGGTGCGCTCTTTTCTGGGAAGGGACACCTGGTATGGTCCACGAGATCGGGTGAAACTA 200
  ----:----|----:----|----:----|----:----|----:----|----:----|----:----|----:----|----:----|----:----|----:----|----:----|----:----|----:----|----:----|----:----|----:----|----:----|----:----|----:----|
1 ACATTTGTTCTACCGCAGCCCCCCGCATTCATTGAGTCTTCTCTTTCTAAAAAAGCTTCTAAGTGCGAAGATAAGCTAAGACTCCAGTCACTTTTTCGCGGCTTTCCAATGTTTTCGTAGTTATCAACTACTTCGTTCGTTTCCACGCGAGAAAAGACCCTTCCCTGTGGACCATACCAGGTGCTCTAGCCCACTTTGAT 200
   X  L  C  S  P  T  P  P  R  L  Y  S  L  L  S  L  N  K  R  L  N  V  S  R  N  S  E  S  T  L  S  F  A  G  F  P  *  L  L  M  L  L  Q  H  L  L  C  L  H  A  R  K  Q  S  P  V  G  P  I  T  W  S  I  P  H  F  *   F6
   X  Y  V  L  H  R  R  P  A  Y  T  V  *  F  L  F  I  K  E  F  I  *  A  E  I  R  N  Q  P  *  H  F  L  A  S  L  N  C  F  C  *  Y  N  I  F  C  A  F  T  R  E  K  R  P  L  S  V  Q  Y  P  G  R  S  R  T  F  S   F5
    T  F  L  I  A  D  P  P  T  L  L  E  S  S  F  S  K  K  S  S  E  R  K  *  E  I  R  L  D  T  F  F  R  R  F  T  V  F  A  D  I  T  S  S  A  L  L  P  A  S  K  E  P  F  P  C  R  T  H  D  V  L  D  P  S  V   F4

   R  C  *  S  D  A  C  N  E  G  C  D  F  W  *  P  A  G  N  R  R  *  N  C  D  S  F  C  S  W  R  D  E  F  E  G  T  A  D  I  F  I  G  K  H  S  Q  L  H  V  S  P  E  *  A  *  Y  K  S  T  L  C  V  R  E  K  V   F1
   A  A  E  V  T  P  A  M  R  A  A  I  F  G  N  Q  Q  E  I  G  G  E  I  V  T  V  S  V  L  G  G  M  N  S  R  V  P  L  T  S  L  S  G  S  I  H  S  Y  T  F  H  L  N  E  H  D  I  K  V  L  S  V  C  E  K  K   F2
   P  L  K  *  R  L  Q  *  G  L  R  F  L  V  T  S  R  K  *  A  V  K  L  *  Q  F  L  F  L  E  G  *  I  R  G  Y  R  *  H  L  Y  R  E  A  F  T  V  T  R  F  T  *  M  S  M  I  *  K  Y  S  L  C  A  R  K  S   F3
201 CCGCTGCTGAAGTGACGCCTGCAATGAGGGCTGCGATTTTTGGTAACCAGCAGGAAATAGGCGGTGAAATTGTGACAGTTTCTGTTCTTGGAGGGATGAATTCGAGGGTACCGCTGACATCTTTATCGGGAAGCATTCACAGTTACACGTTTCACCTGAATGAGCATGATATAAAAGTACTCTCTGTGTGCGAGAAAAAG 400
    ----:----|----:----|----:----|----:----|----:----|----:----|----:----|----:----|----:----|----:----|----:----|----:----|----:----|----:----|----:----|----:----|----:----|----:----|----:----|----:----|
201 GGCGACGACTTCACTGCGGACGTTACTCCCGACGCTAAAAACCATTGGTCGTCCTTTATCCGCCACTTTAACACTGTCAAAGACAAGAACCTCCCTACTTAAGCTCCCATGGCGACTGTAGAAATAGCCCTTCGTAAGTGTCAATGTGCAAAGTGGACTTACTCGTACTATATTTTCATGAGAGACACACGCTCTTTTTC 400
   R  Q  Q  L  S  A  Q  L  S  P  Q  S  K  Q  Y  G  A  P  F  L  R  H  F  Q  S  L  K  Q  E  Q  L  S  S  N  S  P  V  A  S  M  K  I  P  F  C  E  C  N  C  T  E  G  S  H  A  H  Y  L  L  V  R  Q  T  R  S  F   F6
   G  S  S  F  H  R  R  C  H  P  S  R  N  K  T  V  L  L  F  Y  A  T  F  N  H  C  N  R  N  K  S  P  H  I  R  P  Y  R  Q  C  R  *  R  S  A  N  V  T  V  R  K  V  Q  I  L  M  I  Y  F  Y  E  R  H  A  L  F  L   F5
   V  A  A  S  T  V  G  A  I  L  A  A  I  K  P  L  W  C  S  I  P  P  S  I  T  V  T  E  T  R  P  P  I  F  E  L  T  G  S  V  D  K  D  P  L  M  *  L  *  V  N  *  R  F  S  C  S  I  F  T  S  E  T  H  S  F  F   F4

   F  R  G  P  D  N  P  E  A  I  C  A  L  C  L  Y  Q  *  Y  Q  P  H  S  *  W  I  Y  P  A  H  R  Q  G  Q  C  C  N  Q  N  *  N  L  Y  L  G  W  L  G  P  Q  L  H  D  Y  P  *  *  F  D  H  L  G  N  L  W  E   F1
   C  S  G  A  Q  I  T  L  R  P  F  V  H  S  A  S  I  S  D  T  S  L  T  R  D  G  S  T  R  R  T  G  R  G  N  A  A  T  K  T  K  I  F  I  W  G  G  L  D  P  N  F  M  T  T  H  N  D  L  T  I  L  E  T  F  G  R   F2
   V  Q  G  P  R  *  P  *  G  H  L  C  T  L  P  L  S  V  I  P  A  S  L  V  M  D  L  P  G  A  Q  A  G  A  M  L  Q  P  K  L  K  S  L  S  G  V  A  W  T  P  T  S  *  L  P  I  M  I  *  P  S  W  K  P  L  G  G   F3
401 TGTTCAGGGGCCCAGATAACCCTGAGGCCATTTGTGCACTCTGCCTCTATCAGTGATACCAGCCTCACTCGTGATGGATCTACCCGGCGCACAGGCAGGGGCAATGCTGCAACCAAAACTAAAATCTTTATCTGGGGTGGCTTGGACCCCAACTTCATGACTACCCATAATGATTTGACCATCTTGGAAACCTTTGGGAG 600
    ----:----|----:----|----:----|----:----|----:----|----:----|----:----|----:----|----:----|----:----|----:----|----:----|----:----|----:----|----:----|----:----|----:----|----:----|----:----|----:----|
401 ACAAGTCCCCGGGTCTATTGGGACTCCGGTAAACACGTGAGACGGAGATAGTCACTATGGTCGGAGTGAGCACTACCTAGATGGGCCGCGTGTCCGTCCCCGTTACGACGTTGGTTTTGATTTTAGAAATAGACCCCACCGAACCTGGGGTTGAAGTACTGATGGGTATTACTAAACTGGTAGAACCTTTGGAAACCCTC 600
   T  N  L  P  G  S  L  G  S  A  M  Q  A  S  Q  R  *  *  H  Y  W  G  *  E  H  H  I  *  G  A  C  L  C  P  C  H  Q  L  W  F  *  F  R  *  R  P  H  S  P  G  W  S  *  S  *  G  Y  H  N  S  W  R  P  F  R  Q  S   F6
   T  *  P  G  L  Y  G  Q  P  W  K  H  V  R  G  R  D  T  I  G  A  E  S  T  I  S  R  G  P  A  C  A  P  A  I  S  C  G  F  S  F  D  K  D  P  T  A  Q  V  G  V  E  H  S  G  M  I  I  Q  G  D  Q  F  G  K  P   F5
   H  E  P  A  W  I  V  R  L  G  N  T  C  E  A  E  I  L  S  V  L  R  V  R  S  P  D  V  R  R  V  P  L  P  L  A  A  V  L  V  L  I  K  I  Q  P  P  K  S  G  L  K  M  V  V  W  L  S  K  V  M  K  S  V  K  P  L   F4

   G  S  V  I  *  C  Q  T  Y  E  W  S  A  E  S  S  W  G  K  I  W  A  H  L  D  E  S  P  P  R  R  K  T  C  G  S  C  S  I  W  W  G  V  N  Y  W  *  H  *  S  C  H  S  G  *  K  V  V  L  S  E  L  G  T  L  Y  M   F1
   D  Q  S  Y  N  A  K  L  M  N  G  Q  Q  N  R  P  G  A  R  Y  G  H  T  L  T  K  V  H  H  D  G  K  L  V  G  A  V  L  Y  G  G  V  S  I  T  G  D  I  D  H  V  T  Q  D  E  K  L  Y  F  L  N  L  E  H  Y  T  W   F2
   I  S  H  I  M  P  N  L  *  M  V  S  R  I  V  L  G  Q  D  M  G  T  P  *  R  K  S  T  T  T  E  N  L  W  E  L  F  Y  M  V  G  C  Q  L  L  V  T  L  I  M  S  L  R  M  K  S  C  T  F  *  T  W  N  I  I  H   F3
601 GGATCAGTCATATAATGCCAAACTTATGAATGGTCAGCAGAATCGTCCTGGGGCAAGATATGGGCACACCTTGACGAAAGTCCACCACGACGGAAAACTTGTGGGAGCTGTTCTATATGGTGGGGTGTCAATTACTGGTGACATTGATCATGTCACTCAGGATGAAAAGTTGTACTTTCTGAACTTGGAACATTATACAT 800
    ----:----|----:----|----:----|----:----|----:----|----:----|----:----|----:----|----:----|----:----|----:----|----:----|----:----|----:----|----:----|----:----|----:----|----:----|----:----|----:----|
601 CCTAGTCAGTATATTACGGTTTGAATACTTACCAGTCGTCTTAGCAGGACCCGTTCTATACCCGTGTGGAACTGCTTTCAGGTGGTGCTGCCTTTTGAACACCCTCGACAAGATATACCACCCCACAGTTAATGACCACTGTAACTAGTACAGTGAGTCCTACTTTTCAACATGAAAGACTTGAACCTTGTAATATGTA 800
   P  D  T  M  Y  H  W  V  *  S  H  D  A  S  D  D  Q  P  L  I  H  A  C  R  S  S  L  G  G  R  R  F  V  Q  P  L  Q  E  I  H  H  P  T  L  *  Q  H  C  Q  D  H  *  E  P  H  F  T  T  S  E  S  S  P  V  N  Y  M   F6
   P  I  L  *  I  I  G  F  K  H  I  T  L  L  I  T  R  P  C  S  I  P  V  G  Q  R  F  D  V  V  V  S  F  K  H  S  S  N  *  I  T  P  H  *  N  S  T  V  N  I  M  D  S  L  I  F  L  Q  V  K  Q  V  Q  F  M  I  C   F5
   S  *  D  Y  L  A  L  S  I  F  P  *  C  F  R  G  P  A  L  Y  P  C  V  K  V  F  T  W  W  S  P  F  S  T  P  A  T  R  Y  P  P  T  D  I  V  P  S  M  S  *  T  V  *  S  S  F  N  Y  K  R  F  K  S  C  *  V   F4

   G  K  F  N  F  N  R  *  I  A  S  S  A  S  G  L  S  H  W  N  R  N  L  *  W  D  T  G  L  H  W  R  T  V  P  D  T  S  W  *  S  N  S  D  Q  A  T  A  N  S  G  S  H  G  G  *  T  Q  F  C  R  R  Y  D  F  C  I   F1
   E  S  L  T  S  I  G  E  S  H  P  V  P  V  A  Y  H  T  G  T  E  I  C  D  G  T  L  A  Y  I  G  G  L  S  Q  T  L  V  G  S  Q  T  V  I  K  R  L  P  I  Q  E  V  M  V  V  E  L  S  F  A  G  D  T  I  S  A   F2
   G  K  V  *  L  Q  *  V  N  R  I  Q  C  Q  W  L  I  T  L  E  Q  K  S  V  M  G  H  W  L  T  L  E  D  C  P  R  H  *  L  V  V  K  Q  *  S  S  D  C  Q  F  R  K  S  W  W  L  N  S  V  L  Q  E  I  R  F  L  H   F3
801 GGGAAAGTTTAACTTCAATAGGTGAATCGCATCCAGTGCCAGTGGCTTATCACACTGGAACAGAAATCTGTGATGGGACACTGGCTTACATTGGAGGACTGTCCCAGACACTAGTTGGTAGTCAAACAGTGATCAAGCGACTGCCAATTCAGGAAGTCATGGTGGTTGAACTCAGTTTTGCAGGAGATACGATTTCTGCA 1000
    ----:----|----:----|----:----|----:----|----:----|----:----|----:----|----:----|----:----|----:----|----:----|----:----|----:----|----:----|----:----|----:----|----:----|----:----|----:----|----:----|
801 CCCTTTCAAATTGAAGTTATCCACTTAGCGTAGGTCACGGTCACCGAATAGTGTGACCTTGTCTTTAGACACTACCCTGTGACCGAATGTAACCTCCTGACAGGGTCTGTGATCAACCATCAGTTTGTCACTAGTTCGCTGACGGTTAAGTCCTTCAGTACCACCAACTTGAGTCAAAACGTCCTCTATGCTAAAGACGT 1000
   P  F  N  L  K  L  L  H  I  A  D  L  A  L  P  K  D  C  Q  F  L  F  R  H  H  S  V  P  K  C  Q  L  V  T  G  S  V  L  Q  Y  D  F  L  S  *  A  V  A  L  E  P  L  *  P  P  Q  V  *  N  Q  L  L  Y  S  K  Q   F6
   P  F  T  *  S  *  Y  T  F  R  M  W  H  W  H  S  I  V  S  S  C  F  D  T  I  P  C  Q  S  V  N  S  S  Q  G  L  C  *  N  T  T  L  C  H  D  L  S  Q  W  N  L  F  D  H  H  N  F  E  T  K  C  S  I  R  N  R  C   F5
   H  S  L  K  V  E  I  P  S  D  C  G  T  G  T  A  *  V  P  V  S  I  Q  S  P  V  S  A  *  M  P  P  S  D  W  V  S  T  P  L  *  V  T  I  L  R  S  G  I  *  S  T  M  T  T  S  S  L  K  A  P  S  V  I  E  A   F4

   S  P  K  Y  *  I  F  I  *  W  *  S  D  *  R  S  V  S  V  W  A  H  C  Q  R  M  W  P  G  P  N  T  C  L  R  W  L  S  T  E  N  T  *  G  E  Q  *  H  S  E  W  A  L  F  P  P  G  Y  E  H  S  *  H  E  A  Q   F1
   S  V  R  N  I  K  F  S  F  S  G  D  P  T  K  E  V  F  L  S  G  H  T  A  N  V  C  G  Q  D  Q  I  L  V  F  G  G  Y  Q  Q  K  T  P  E  E  N  S  D  I  P  S  G  H  Y  F  L  L  D  M  N  T  L  S  M  R  H  K   F2
   Q  S  E  I  L  N  F  H  L  V  V  I  R  L  K  K  C  F  C  L  G  T  I  L  P  T  Y  V  A  R  T  K  Y  L  S  S  V  A  I  N  R  K  H  L  R  R  T  V  T  F  R  V  G  I  I  S  S  W  I  *  T  L  A  *  G  T  R   F3
1001 TCAGTCCGAAATATTAAATTTTCATTTAGTGGTGATCCGACTAAAGAAGTGTTTCTGTCTGGGCACACTGCCAACGTATGTGGCCAGGACCAAATACTTGTCTTCGGTGGCTATCAACAGAAAACACCTGAGGAGAACAGTGACATTCCGAGTGGGCATTATTTCCTCCTGGATATGAACACTCTTAGCATGAGGCACAA 1200
     ----:----|----:----|----:----|----:----|----:----|----:----|----:----|----:----|----:----|----:----|----:----|----:----|----:----|----:----|----:----|----:----|----:----|----:----|----:----|----:----|
1001 AGTCAGGCTTTATAATTTAAAAGTAAATCACCACTAGGCTGATTTCTTCACAAAGACAGACCCGTGTGACGGTTGCATACACCGGTCCTGGTTTATGAACAGAAGCCACCGATAGTTGTCTTTTGTGGACTCCTCTTGTCACTGTAAGGCTCACCCGTAATAAAGGAGGACCTATACTTGTGAGAATCGTACTCCGTGTT 1200
   M  L  G  F  Y  *  I  K  M  *  H  H  D  S  *  L  L  T  E  T  Q  A  C  Q  W  R  I  H  G  P  G  F  V  Q  R  R  H  S  D  V  S  F  V  Q  P  S  C  H  C  E  S  H  A  N  N  G  G  P  Y  S  C  E  *  C  S  A  C   F6
   *  D  S  I  N  F  K  *  K  T  I  R  S  F  F  H  K  Q  R  P  V  S  G  V  Y  T  A  L  V  L  Y  K  D  E  T  A  I  L  F  C  R  L  L  V  T  V  N  R  T  P  M  I  E  E  Q  I  H  V  S  K  A  H  P  V   F5
   D  T  R  F  I  L  N  E  N  L  P  S  G  V  L  S  T  N  R  D  P  C  V  A  L  T  H  P  W  S  W  I  S  T  K  P  P  *  *  C  F  V  G  S  S  F  L  S  M  G  L  P  C  *  K  R  R  S  I  F  V  R  L  M  L  C  L   F4

   G  S  S  *  G  F  *  N  G  Q  S  H  N  N  *  L  G  L  K  V  N  F  F  P  W  R  G  T  S  A  S  F  H  N  D  C  E  E  D  G  T  N  H  L  *  S  *  E  V  C  S  E  K  P  L  L  T  W  *  S  H  Y  M  F  A  M  *   F1
   E  A  P  E  G  F  E  M  A  S  H  T  T  I  N  L  D  S  R  S  I  F  F  H  G  G  A  L  Q  H  L  F  T  M  T  V  K  R  M  E  P  T  I  C  E  A  E  K  C  V  V  K  N  H  F  S  P  G  E  A  T  T  C  L  Q  C  D   F2
   K  L  L  R  V  L  K  W  P  V  T  Q  Q  L  T  W  T  Q  G  Q  F  F  S  M  E  G  H  F  S  I  F  S  Q  *  L  *  R  G  W  N  Q  P  F  V  K  L  R  S  V  *  *  K  T  T  S  H  L  V  K  P  L  H  V  C  N  V   F3
1201 GGAAGCTCCTGAGGGTTTTGAAATGGCCAGTCACACAACAATTAACTTGGACTCAAGGTCAATTTTTTTCCATGGAGGGGCACTTCAGCATCTTTTCACAATGACTGTGAAGAGGATGGAACCAACCATTTGTGAAGCTGAGAAGTGTGTAGTGAAAAACCACTTCTCACCTGGTGAAGCCACTACATGTTTGCAATGTG 1400
     ----:----|----:----|----:----|----:----|----:----|----:----|----:----|----:----|----:----|----:----|----:----|----:----|----:----|----:----|----:----|----:----|----:----|----:----|----:----|----:----|
1201 CCTTCGAGGACTCCCAAAACTTTACCGGTCAGTGTGTTGTTAATTGAACCTGAGTTCCAGTTAAAAAAAGGTACCTCCCCGTGAAGTCGTAGAAAAGTGTTACTGACACTTCTCCTACCTTGGTTGGTAAACACTTCGACTCTTCACACATCACTTTTTGGTGAAGAGTGGACCACTTCGGTGATGTACAAACGTTACAC 1400
   P  L  E  Q  P  N  Q  F  P  W  D  C  L  L  *  S  P  S  L  T  L  K  K  G  H  L  P  V  E  A  D  K  *  L  S  Q  S  S  S  P  V  L  W  K  H  L  Q  S  T  H  L  S  F  G  S  R  V  Q  H  L  W  *  M  N  A  I  H   F6
   L  F  S  R  L  T  K  F  H  G  T  V  C  C  N  V  Q  V  *  P  *  N  K  E  M  S  P  C  K  L  M  K  E  C  H  S  H  L  P  H  F  W  G  N  T  F  S  L  L  T  Y  H  F  V  V  E  *  R  T  F  G  S  C  T  Q  L  T   F5
   S  A  G  S  P  K  S  I  A  L  *  V  V  I  L  K  S  E  L  D  I  K  K  W  P  P  A  S  *  C  R  K  V  I  V  T  F  L  I  S  G  V  M  Q  S  A  S  F  H  T  T  F  F  W  K  E  G  P  S  A  V  V  H  K  C  H   F4

   C  L  R  *  I  F  P  P  S  K  T  R  R  H  Q  L  V  P  N  A  K  L  R  K  M  Q  N  *  E  K  D  W  K  K  E  N  E  E  V  K  F  Q  H  D  I  T  C  L  V  F  T  N  K  A  H  H  M  Y  G  T  K  N  K  M  S  T  *   F1
   A  C  D  K  F  F  H  P  Q  K  L  E  D  I  N  L  C  Q  M  Q  N  *  E  K  C  K  T  K  K  K  T  G  K  R  K  T  K  K  *  N  F  N  M  I  S  H  V  L  Y  S  P  T  R  P  T  I  C  M  A  Q  R  I  K  C  P  L   F2
   M  P  A  I  N  F  S  T  L  K  N  *  K  T  S  T  C  A  K  C  K  T  K  K  N  A  K  L  R  K  R  L  E  K  G  K  R  R  S  K  I  S  T  *  Y  H  M  S  C  I  H  Q  Q  G  P  P  Y  V  W  H  K  E  *  N  V  H  L   F3
1401 ATGCCTGCGATAAATTTTTCCACCCTCAAAAACTAGAAGACATCAACTTGTGCCAAATGCAAAACTAAGAAAAATGCAAAACTAAGAAAAAGACTGGAAAAAGGAAAACGAAGAAGTAAAATTTCAACATGATATCACATGTCTTGTATTCACCAACAAGGCCCACCATATGTATGGCACAAAGAATAAAATGTCCACTT 1600
     ----:----|----:----|----:----|----:----|----:----|----:----|----:----|----:----|----:----|----:----|----:----|----:----|----:----|----:----|----:----|----:----|----:----|----:----|----:----|----:----|
1401 TACGGACGCTATTTAAAAAGGTGGGAGTTTTTGATCTTCTGTAGTTGAACACGGTTTACGTTTTGATTCTTTTTACGTTTTGATTCTTTTTCTGACCTTTTTCCTTTTGCTTCTTCATTTTAAAGTTGTACTATAGTGTACAGAACATAAGTGGTTGTTCCGGGTGGTATACATACCGTGTTTCTTATTTTACAGGTGAA 1600
   H  R  R  Y  I  K  G  G  E  F  V  L  L  C  *  S  T  G  F  A  F  S  L  F  I  C  F  *  S  F  S  Q  F  F  S  F  S  S  T  F  N  *  C  S  I  V  H  R  T  N  V  L  L  A  W  W  I  Y  P  V  F  F  L  I  D  V   F6
   I  G  A  I  F  K  E  V  R  L  F  *  F  V  D  V  Q  A  L  H  L  V  L  F  A  F  S  L  F  L  S  S  F  P  F  R  L  L  L  I  E  V  H  Y  *  M  D  Q  I  *  W  C  P  G  G  Y  T  H  C  L  S  Y  F  T  W  K   F5
   S  A  Q  S  L  N  K  W  G  *  F  S  S  S  M  L  K  H  W  I  C  F  *  S  F  H  L  V  L  F  F  V  P  F  L  F  V  F  F  Y  F  K  L  M  I  D  C  T  K  Y  E  G  V  L  G  V  M  H  I  A  C  L  I  F  H  G  S   F4

   S  *  L  N  T  H  S  S  F  H  F  S  G  K  S  L  L  H  F  C  V  M  S  W  A  *  G  F  T  F  H  F  L  H  T  I  H  T  N  H  G  Y  F  I  L  *  L  L  L  V  F  N  Q  T  *  I  V  I  I  I  T  L  S  G  M  A   F1
   N  H  N  *  I  P  T  V  H  F  I  S  Q  A  R  V  F  Y  I  F  V  *  C  L  G  P  R  V  S  H  F  T  F  Y  I  Q  F  T  Q  I  M  A  T  S  S  S  S  S  S  W  F  S  I  K  P  E  S  S  S  S  S  L  C  L  A  W  H   F2
   I  I  I  E  Y  P  Q  F  I  S  F  L  R  Q  E  S  S  T  F  L  C  N  V  L  G  L  G  F  H  I  S  L  F  T  Y  N  S  H  K  S  W  L  L  H  P  L  A  P  P  G  F  Q  S  N  L  N  R  H  H  H  H  S  V  W  H  G  M   F3
1601 AATCATAATTGAATACCCACAGTTCATTTCATTTCTCAGGCAAGAGTCTTCTACATTTTTGTGTAATGTCTTGGGCCTAGGGTTTCACATTTCACTTTTTACATACAATTCACACAAATCATGGCTACTTCATCCTCTAGCTCCTCCTGGTTTTCAATCAAACCTGAATCGTCATCATCATCACTCTGTCTGGCATGGCA 1800
     ----:----|----:----|----:----|----:----|----:----|----:----|----:----|----:----|----:----|----:----|----:----|----:----|----:----|----:----|----:----|----:----|----:----|----:----|----:----|----:----|
1601 TTAGTATTAACTTATGGGTGTCAAGTAAAGTAAAGAGTCCGTTCTCAGAAGATGTAAAAACACATTACAGAACCCGGATCCCAAAGTGTAAAGTGAAAAATGTATGTTAAGTGTGTTTAGTACCGATGAAGTAGGAGATCGAGGAGGACCAAAAGTTAGTTTGGACTTAGCAGTAGTAGTAGTGAGACAGACCGTACCGT 1800
   *  D  Y  N  F  V  W  L  E  N  *  K  E  P  L  L  R  R  C  K  Q  T  I  D  Q  A  *  P  K  V  N  *  K  K  C  V  I  *  V  F  *  P  *  K  M  R  *  S  R  R  T  K  L  *  V  Q  I  T  M  M  M  V  R  D  P  M  A   F6
   I  M  I  S  Y  G  C  N  M  E  N  R  L  C  S  D  E  V  N  K  H  L  T  K  P  R  P  N  *  M  E  S  K  V  Y  L  E  C  L  D  H  S  S  *  G  R  A  G  G  P  K  *  D  F  R  F  R  *  *  *  E  T  Q  C  P   F5
   L  *  L  Q  I  G  V  T  *  K  M  E  *  A  L  T  K  *  M  K  T  Y  H  R  P  G  L  T  E  C  K  V  K  *  M  C  N  V  C  I  M  A  V  E  D  E  L  E  E  Q  N  E  I  L  G  S  D  D  D  D  S  Q  R  A  H  C   F4

   C  L  V  L  L  L  C  K  F  T  S  A  N  I  M  A  S  S  S  T  G  C  A  V  L  W  K  *  F  S  G  S  *  F  L  *  Y  L  T  G  V  Q  P  L  K  N  Q  C  I  S  K  V  A  V  I  R  S  V  L  P  A  Q  R  F  T  N  K   F1
   V  W  F  C  F  F  A  S  S  Q  V  R  I  L  W  P  A  P  P  Q  A  V  Q  Y  F  G  N  D  L  V  A  A  N  F  C  S  T  L  L  V  Y  S  H  *  K  T  N  A  S  L  R  S  Q  S  S  E  V  S  C  L  L  R  D  L  R  I  N   F2
   S  G  S  A  S  L  Q  V  H  K  C  E  Y  Y  G  Q  L  L  H  R  L  C  S  T  L  E  M  I  *  W  Q  L  I  S  V  V  P  Y  W  C  T  A  I  E  K  P  M  H  L  *  G  R  S  H  Q  K  C  L  A  C  S  E  I  Y  E  *   F3
1801 TGTCTGGTTCTGCTTCTTTGCAAGTTCACAAGTGCGAATATTATGGCCAGCTCCTCCACAGGCTGTGCAGTACTTTGGAAATGATTTAGTGGCAGCTAATTTCTGTAGTACCTTACTGGTGTACAGCCATTGAAAAACCAATGCATCTCTAAGGTCGCAGTCATCAGAAGTGTCTTGCCTGCTCAGAGATTTACGAATAA 2000
     ----:----|----:----|----:----|----:----|----:----|----:----|----:----|----:----|----:----|----:----|----:----|----:----|----:----|----:----|----:----|----:----|----:----|----:----|----:----|----:----|
1801 ACAGACCAAGACGAAGAAACGTTCAAGTGTTCACGCTTATAATACCGGTCGAGGAGGTGTCCGACACGTCATGAAACCTTTACTAAATCACCGTCGATTAAAGACATCATGGAATGACCACATGTCGGTAACTTTTGGTTACGTAGAGATTCCAGCGTCAGTAGTCTTCACAGAACGGACGAGTCTCTAAATGCTTATT 2000
   H  R  T  R  S  R  Q  L  N  V  L  A  F  I  I  A  L  E  E  V  P  Q  A  T  S  Q  F  H  N  L  P  L  *  N  R  Y  R  V  P  T  C  G  N  F  F  W  H  M  E  L  T  A  T  M  L  L  T  K  G  A  *  L  N  V  F  L   F6
   M  D  P  E  A  E  K  C  T  *  L  H  S  Y  *  P  W  S  R  W  L  S  H  L  V  K  S  I  I  *  H  C  S  I  E  T  T  G  *  Q  H  V  A  M  S  F  G  I  C  R  *  P  R  L  *  *  F  H  R  A  Q  E  S  I  *  S  Y   F5
   T  Q  N  Q  K  K  A  L  E  C  T  R  I  N  H  G  A  G  G  C  A  T  C  Y  K  P  F  S  K  T  A  A  L  K  Q  L  V  K  S  T  Y  L  W  Q  F  V  L  A  D  R  L  D  C  D  D  S  T  D  Q  R  S  L  S  K  R  I   F4

   P  I  Q  L  I  A  T  L  P  T  F  T  G  E  S  T  N  T  V  N  S  L  R  I  L  N  Y  F  C  H  M  L  H  N  F  V  D  I  I  G  P  N  H  I  R  K  L  L  S  Q  K  E  C  E  L  Q  C  I  F  F  V  L  C  H  F  L  W   F1
   L  Y  N  L  L  P  H  S  P  P  S  L  E  R  A  P  T  L  *  I  P  *  G  S  *  I  I  S  A  T  C  S  T  T  L  W  T  *  L  D  Q  I  T  *  G  N  C  C  P  R  R  N  A  N  C  S  A  F  S  L  Y  C  A  I  S  S   F2
   T  Y  T  T  Y  C  H  T  P  H  L  H  W  R  E  H  Q  H  C  E  F  P  E  D  P  E  L  F  L  P  H  A  P  Q  L  C  G  H  N  W  T  K  S  H  K  E  I  V  V  P  E  G  M  R  T  A  V  H  F  L  C  T  V  P  F  P  L   F3
2001 ACCTATACAACTTATTGCCACACTCCCCACCTTCACTGGAGAGAGCACCAACACTGTGAATTCCCTGAGGATCCTGAATTATTTCTGCCACATGCTCCACAACTTTGTGGACATAATTGGACCAAATCACATAAGGAAATTGTTGTCCCAGAAGGAATGCGAACTGCAGTGCATTTTCTTTGTACTGTGCCATTTCCTCT 2200
     ----:----|----:----|----:----|----:----|----:----|----:----|----:----|----:----|----:----|----:----|----:----|----:----|----:----|----:----|----:----|----:----|----:----|----:----|----:----|----:----|
2001 TGGATATGTTGAATAACGGTGTGAGGGGTGGAAGTGACCTCTCTCGTGGTTGTGACACTTAAGGGACTCCTAGGACTTAATAAAGACGGTGTACGAGGTGTTGAAACACCTGTATTAACCTGGTTTAGTGTATTCCTTTAACAACAGGGTCTTCCTTACGCTTGACGTCACGTAAAAGAAACATGACACGGTAAAGGAGA 2200
   G  I  C  S  I  A  V  S  G  V  K  V  P  S  L  V  L  V  T  F  E  R  L  I  R  F  *  K  Q  W  M  S  W  L  K  T  S  M  I  P  G  F  *  M  L  F  N  N  D  W  F  S  H  S  S  C  H  M  K  K  T  S  H  W  K  R   F6
   V  *  V  V  *  Q  W  V  G  W  R  *  Q  L  S  C  W  C  Q  S  N  G  S  S  G  S  N  N  R  G  C  A  G  C  S  Q  P  C  L  Q  V  L  D  C  L  S  I  T  T  G  S  P  I  R  V  A  T  C  K  R  Q  V  T  G  N  G  R   F5
   F  R  Y  L  K  N  G  C  E  G  G  E  S  S  L  A  G  V  S  H  I  G  Q  P  D  Q  I  I  E  A  V  H  E  V  V  K  H  V  Y  N  S  W  I  V  Y  P  F  Q  Q  G  L  L  F  A  F  Q  L  A  N  E  K  Y  Q  A  M  E  E   F4

   *  C  L  D  R  I  F  T  R  I  H  C  M  H  L  A  E  V  T  K  E  S  F  N  M  L  L  S  F  F  I  S  D  *  L  C  D  N  I  N  I  L  G  V  K  *  C  S  G  K  I  S  N  H  Y  K  F  *  L  Q  T  K  S  F  H  E   F1
   G  N  V  W  T  G  F  L  Q  E  Y  T  A  C  I  L  L  K  *  P  R  R  A  L  T  C  C  F  L  S  S  F  L  I  N  F  V  T  I  S  T  F  L  G  S  N  S  A  L  A  K  F  P  T  I  I  S  F  D  C  R  P  R  V  F  M  R   F2
   V  M  S  G  Q  D  F  Y  K  N  T  L  H  A  S  C  *  S  D  Q  G  E  L  *  H  A  A  F  F  L  H  F  *  L  T  L  *  Q  Y  Q  H  S  W  G  Q  I  V  L  W  Q  N  F  Q  P  L  *  V  L  T  A  D  Q  E  F  S  *  G   F3
2201 GGTAATGTCTGGACAGGATTTTTACAAGAATACACTGCATGCATCTTGCTGAAGTGACCAAGGAGAGCTTTAACATGCTGCTTTCTTCTTCATTTCTGATTAACTTTGTGACAATATCAACATTCTTGGGGTCAAATAGTGCTCTGGCAAAATTTCCAACCATTATAAGTTTTGACTGCAGACCAAGAGTTTTCATGAG 2400
     ----:----|----:----|----:----|----:----|----:----|----:----|----:----|----:----|----:----|----:----|----:----|----:----|----:----|----:----|----:----|----:----|----:----|----:----|----:----|----:----|
2201 CCATTACAGACCTGTCCTAAAAATGTTCTTATGTGACGTACGTAGAACGACTTCACTGGTTCCTCTCGAAATTGTACGACGAAAGAAAGAAGTAAAGACTAATTGAAACACTGTTATAGTTGTAAGAACCCCAGTTTATCACGAGACCGTTTTAAAGGTTGGTAATATTCAAAACTGACGTCTGGTTCTCAAAAGTACTC 2400
   Q  Y  H  R  S  L  I  K  V  L  I  C  Q  M  C  R  A  S  T  V  L  S  L  K  L  M  S  S  E  K  K  M  E  S  *  S  Q  S  L  I  L  M  R  P  T  L  Y  H  E  P  L  I  E  L  W  *  L  N  Q  S  C  V  L  L  K  *  S   F6
   T  I  D  P  C  S  K  *  L  F  V  S  C  A  D  Q  Q  L  S  W  P  S  S  *  C  A  A  K  K  R  *  K  Q  N  V  K  H  C  Y  *  C  E  Q  P  *  I  T  S  Q  C  F  K  W  G  N  Y  T  K  V  A  S  W  S  N  E  H   F5
   P  L  T  Q  V  P  N  K  C  S  Y  V  A  H  M  K  S  F  H  G  L  L  A  K  V  H  Q  K  R  E  E  N  R  I  L  K  T  V  I  D  V  N  K  P  D  F  L  A  R  A  F  N  G  V  M  I  L  K  S  Q  L  G  L  T  K  M  L   F4
```

```
        V  F  I  Q  C  L  L  S  M  M  Q  C  S  F  I  L  L  I  F  *  P  L  M  D  F  S  N  F  P  H  K  Y  L  L  E  K  P  A  N  I  Y  Y  V  C  M  S  *  V  *  *  S  F  S  S  I  *  D  N  Q  R  H  A  V  H  T  F  A  F   F1
         C  S  S  N  V  F  S  A  *  C  N  V  V  L  F  C  S  F  S  D  H  *  W  T  S  A  I  S  L  T  S  I  F  L  K  N  L  P  I  F  M  F  A  *  V  E  S  N  D  L  S  V  A  S  E  T  I  R  G  M  L  F  T  P  L  H  F   F2
          V  H  P  M  S  S  Q  H  D  A  M  *  F  Y  S  A  H  F  L  T  I  D  G  L  Q  Q  F  P  S  Q  V  S  S  *  K  T  C  Q  Y  L  C  L  H  E  L  S  L  M  I  F  Q  *  H  L  R  Q  S  E  A  C  C  S  H  L  C  I    F3
2401 GTGTTCATCCAATGTCTTCTCAGCATGATGCAATGTAGTTTTATTCTGCTCATTTTCTGACCATTGATGGACTTCAGCAATTTCCCTCACAAGTATCTTCTTGAAAAACCTGCCAATATTTATGTTTGCATGAGTTGAGTCTAATGATCTTTCAGTAGCATCTGAGACAATCAGAGGCATGCTGTTCACACCTTTGCATT 2600
     ----:----|----:----|----:----|----:----|----:----|----:----|----:----|----:----|----:----|----:----|----:----|----:----|----:----|----:----|----:----|----:----|----:----|----:----|----:----|----:----|
2401 CACAAGTAGGTTACAGAAGAGTCGTACTACGTTACATCAAAATAAGACGAGTAAAAGACTGGTAACTACCTGAAGTCGTTAAAGGGAGTGTTCATAGAAGAACTTTTTGGACGGTTATAAATACAAACGTACTCAACTCAGATTACTAGAAAGTCATCGTAGACTCTGTTAGTCTCCGTACGACAAGTGTGGAAACGTAA 2600
      T  N  M  W  H  R  R  L  M  I  C  H  L  K  I  R  S  M  K  Q  G  N  I  S  K  L  L  K  G  *  L  Y  R  R  S  F  G  A  L  I  *  T  Q  M  L  Q  T  *  H  D  K  L  L  M  Q  S  L  *  L  C  A  T  *  V  K  A  N   F6
     P  T  *  G  I  D  E  *  C  S  A  I  Y  N  *  E  A  *  K  R  V  M  S  P  S  *  C  N  G  E  C  T  D  E  Q  F  V  Q  W  Y  K  H  K  C  S  N  L  R  I  I  K  *  Y  C  R  L  C  D  S  A  H  Q  E  C  R  Q  M    F5
       H  E  D  L  T  K  E  A  H  H  L  T  T  K  N  Q  E  N  E  S  W  Q  H  V  E  A  I  E  R  V  L  I  K  K  F  F  R  G  I  N  I  N  A  H  T  S  D  L  S  R  E  T  A  D  S  V  I  L  P  M  S  N  V  G  K  C     F4

         Y  C  K  V  I  I  I  Q  M  V  R  I  F  K  A  I  L  V  E  G  N  C  F  F  V  C  P  A  P  C  K  G  A  N  I  T  L  T  V  A  S  M  C  M  A  Q  S  T  Q  I  P  R  A  T  S  L  K  S  G  G  C  S  L  E  L  L  I   F1
          T  A  R  S  L  S  F  R  W  S  G  F  S  R  R  Y  L  *  R  E  T  V  S  S  C  V  L  H  L  V  K  E  P  T  S  L  *  Q  L  P  V  C  A  W  H  K  V  H  R  Y  R  E  P  L  P  *  S  P  E  D  V  R  S  N  F  S    F2
        L  L  Q  G  H  Y  H  S  D  G  Q  D  F  Q  G  D  T  C  R  G  K  L  F  L  R  V  S  C  T  L  *  R  S  Q  H  H  F  D  S  C  Q  Y  V  H  G  T  K  Y  T  D  T  A  S  H  F  L  E  V  R  R  M  F  A  R  T  S  H   F3
2601 TTACTGCAAGGTCATTATCATTCAGATGGTCAGGATTTTCAAGGCGATACTTGTAGAGGGAAACTGTTTCTTCGTGTGTCCTGCACCTTGTAAAGGAGCCAACATCACTTTGACAGTTGCCAGTATGTGCATGGCACAAAGTACACAGATACCGCGAGCCACTTCCTTGAAGTCCGGAGGATGTTCGCTCGAACTTCTCA 2800
     ----:----|----:----|----:----|----:----|----:----|----:----|----:----|----:----|----:----|----:----|----:----|----:----|----:----|----:----|----:----|----:----|----:----|----:----|----:----|----:----|
2601 AATGACGTTCCAGTAATAGTAAGTCTACCAGTCCTAAAAGTTCCGCTATGAACATCTCCCTTTGACAAAGAAGCACACAGGACGTGGAACATTTCCTCGGTTGTAGTGAAACTGTCAACGGTCATACACGTACCGTGTTTCATGTGTCTATGGCGCTCGGTGAAGGAACTTCAGGCCTCCTACAAGCGAGCTTGAAGAGT 2800
        *  Q  L  T  M  I  M  *  I  T  L  I  K  L  A  I  S  T  S  P  F  Q  K  K  T  H  G  A  G  Q  L  P  A  L  M  V  K  V  T  A  L  I  H  M  A  C  L  V  C  I  G  R  A  V  E  K  F  D  P  P  H  E  S  S  S  R    F6
       K  S  C  P  *  *  *  E  S  P  *  S  K  *  P  S  V  Q  L  P  F  S  N  R  R  T  D  Q  V  K  Y  L  L  W  C  *  K  S  L  Q  W  Y  T  C  P  V  F  Y  V  S  V  A  L  W  K  R  S  T  R  L  I  N  A  R  V  E  *   F5
     K  V  A  L  D  N  D  N  L  H  D  P  N  E  L  R  Y  K  Y  L  S  V  T  E  E  H  T  R  C  R  T  F  S  G  V  D  S  Q  C  N  G  T  H  A  H  C  L  T  C  L  Y  R  S  G  S  G  Q  L  G  S  S  T  R  E  F  K  E   F4

          N  H  T  V  K  E  M  *  F  M  L  S  Q  A  V  T  S  T  G  I  I  *  L  Q  C  G  S  F  H  F  L  P  V  S  *  D  F  P  Q  K  C  C  N  T  W  P  V  T  F  I  T  M  T  E  N  D  R  P  S  T  I  N  R  I  G  E    F1
       S  T  I  L  *  R  K  C  S  S  C  S  L  R  Q  *  P  V  L  A  S  Y  N  C  N  V  A  P  F  I  F  S  L  S  L  E  T  F  P  R  S  V  A  I  L  G  L  S  L  S  S  Q  *  Q  R  M  T  G  R  V  L  S  T  E  L  G  N   F2
        Q  P  Y  C  K  G  N  V  V  H  A  L  S  G  S  D  Q  Y  W  H  H  I  I  A  M  W  L  L  S  F  S  P  C  L  L  R  L  S  P  E  V  L  Q  Y  L  A  C  H  F  H  H  N  D  R  E  *  Q  A  E  Y  Y  Q  Q  N  W  G  T   F3
2801 TCAACCATACTGTAAAGGAAATGTAGTTCATGCTCTCTCAGGCAGTGACCAGTACTGGCATCATATAATTGCAATGTGGCTCCTTTCATTTTCTCCCTGTCTCTTGAGACTTTCCCCAGAAGTGTTGCAATACTTGGCCTGTCACTTTCATCACAATGACAGAGAATGACAGGCCGAGTACTATCAACAGAATTGGGGAA 3000
     ----:----|----:----|----:----|----:----|----:----|----:----|----:----|----:----|----:----|----:----|----:----|----:----|----:----|----:----|----:----|----:----|----:----|----:----|----:----|----:----|
2801 AGTTGGTATGACATTTCCTTTACATCAAGTACGAGAGAGTCCGTCACTGGTCATGACCGTAGTATATTAACGTTACACCGAGGAAAGTAAAAGAGGGACAGAGAACTCTGAAAGGGGTCTTCACAACGTTATGAACCGGACAGTGAAAGTAGTGTTACTGTCTCTTACTGTCCGGCTCATGATAGTTGTCTTAACCCCTT 3000
     M  L  W  V  T  F  S  I  Y  N  M  S  E  *  A  T  V  L  V  P  M  M  Y  N  C  H  P  E  K  *  K  R  G  T  E  Q  S  K  G  W  F  H  Q  L  V  Q  G  T  V  K  M  V  I  V  S  F  S  L  G  L  V  I  L  L  I  P  S    F6
       *  G  Y  Q  L  P  F  T  T  *  A  R  E  P  L  S  W  Y  Q  C  *  I  I  A  I  H  S  R  E  N  E  G  Q  R  K  L  S  E  G  S  T  N  C  Y  K  A  Q  *  K  *  *  L  S  L  S  H  C  A  S  Y  *  *  C  F  Q  P    F5
        D  V  M  S  Y  L  F  H  L  E  H  E  R  L  C  H  G  T  S  A  D  Y  L  Q  L  T  A  G  K  M  K  E  R  D  R  S  V  K  G  L  L  T  A  I  S  P  R  D  S  E  D  C  H  C  L  I  V  P  R  T  S  D  V  S  N  P  F   F4

     Q  L  L  E  *  N  M  L  C  F  I  Y  H  D  R  T  X   F1
      N  S  L  N  R  T  C  S  V  S  F  T  M  T  E  H   F2
       T  P  *  I  E  H  A  L  F  H  L  P  *  Q  N  T   F3
3001 CAACTCCTTGAATAGAACATGCTCTGTTTCATTTACCATGACAGAACAC 3049
     ----:----|----:----|----:----|----:----|----:----
3001 GTTGAGGAACTTATCTTGTACGAGACAAAGTAAATGGTACTGTCTTGTG 3049
      C  S  R  S  Y  F  M  S  Q  K  M  *  W  S  L  V   F6
     V  V  G  Q  I  S  C  A  R  N  *  K  G  H  C  F  V   F5
       L  E  K  F  L  V  H  E  T  E  N  V  M  V  S  C   F4
```

Matching TSA transcripts

RAG1L

>NMRB01000727.1:44100-293265
>TSA:GFRY01046940.1:beg-end
>TSA:GFRY01046941.1:beg-end

```
               1                                                                                                                                                                                                      200
NMRB01000727.1 AACCAACCATTTGTGAAGCTGAGAAGTGTGTAGTGAAAAACCACTTCTCACCTGGTGAAGCCACTACATGTTTGCAATGTGATGCCTGCGATAAATTTTTCCACCCTCAAAAACTAGAAGACATCAACTTGTGCCAAATGCAAAACTAAGAAAAATGCAAAACTAAGAAAAAGACTGGAAAAAGGAAAACGAAGAAGTAA
GFRY01046940.1                                                   CCTGGTGAAGCCACTACATGTTTGCAATGTGATGCCTGCGATAAATTTTTCCACCCTCAAAAACTAGAAGACATCAACTTGTGCCAAATGCAAAACTAAGAAAAATGCAAAACTAAGAAAAAGACTGGAAAAAGGAAAACGAAGAAGTAA
GFRY01046941.1                                                   CCTGGTGAAGCCACTACATGTTTGCAATGTGATGCCTGCGATAAATTTTTCCACCCTCAAAAACTAGAAGACATCAACTTGTGCCAAATGCAAAACTAAGAAAAATGCAAAACTAAGAAAAAGACTGGAAAAAGGAAAACGAAGAAGTAA
 Consensus     ..................................................CCTGGTGAAGCCACTACATGTTTGCAATGTGATGCCTGCGATAAATTTTTCCACCCTCAAAAACTAGAAGACATCAACTTGTGCCAAATGCAAAACTAAGAAAAATGCAAAACTAAGAAAAAGACTGGAAAAAGGAAAACGAAGAAGTAA

               201                                                                                                                                                                                                    400
NMRB01000727.1 AATTTCAACATGATATCACATGTCTTGTATTCACCAACAAGGCCCACCATATGTATGGCACAAAGAATAAAATGTCCACTTAATCATAATTGAATACCCACAGTTCATTTCATTTCTCAGGCAAGAGTCTTCTACATT-TTTGTGTAATGTCTTGGGCCTAGGGTTTCACATTTCACTTTTTACATACAATTCACACAAA
GFRY01046940.1 AATTTCAACATGATATCACATGTCTTGTATTCACCAACAAGGCCCACCATATGTATGGCACAAAGAATAAAATGTCCACTTAATCATAATTGAATACCCACAGTTCATTTCATTTCTCAGGCAAGAGTCTTCTACATTATTTGTGTAATGTCTTGGGCCTAGGGTTTCACATTTCACTTTTTACATACAATTCACACAAA
GFRY01046941.1 AATTTCAACATGATATCACATGTCTTGTATTCACCAACAAGGCCCACCATATGTATGGCACAAAGAATAAAATGTCCACTTAATCATAATTGAATACCCACAGTTCATTTCATTTCTCAGGCAAGAGTCTTCTACATTATTTGTGTAATGTCTTGGGCCTAGGGTTTCACATTTCACTTTTTACATACAATTCACACAAA
 Consensus     AATTTCAACATGATATCACATGTCTTGTATTCACCAACAAGGCCCACCATATGTATGGCACAAAGAATAAAATGTCCACTTAATCATAATTGAATACCCACAGTTCATTTCATTTCTCAGGCAAGAGTCTTCTACATTaTTTGTGTAATGTCTTGGGCCTAGGGTTTCACATTTCACTTTTTACATACAATTCACACAAA

               401                                                                                                                                                                                                    600
NMRB01000727.1 TCATGGCTACTTCATCCTCTAGCTCCTCCTGGTTTTCAATCAAACCTGAATCGTCATCATCATCACTCTGTCTGGCATGGCATGTCTGGTTCTGCTTCTTTGCAAGTTCACAAGTGCGAATATTATGGCCAGCTCCTCCACAGGCTGTGCAGTACTTTGGAAATGATTTAGTGGCAGCTAATTTCTGTAGTACCTTACTG
GFRY01046940.1 TCATGGCTACTTCATCCTCTAGCTCCTCCTGGTTTTCAATCAAACCTGAATCGTCATCATCATCACTCTGTCTGGCATGGCATGTCTGGTTCTGCTTCTTTGCAAGTTCACAAGTGCGAATATTATGGCCAGCTCCTCCACAGGCTGTGCAGTACTTTGGAAATGATTTAGTGGCAGCTAATTTCTGTAGTACCTTACTG
GFRY01046941.1 TCATGGCTACTTCATCCTCTAGCTCCTCCTGGTTTTCAATCAAACCTGAATCGTCATCATCATCACTCTGTCTGGCATGGCATGTCTGGTTCTGCTTCTTTGCAAGTTCACAAGTGCGAATATTATGGCCAGCTCCTCCACAGGCTGTGCAGTACTTTGGAAATGATTTAGTGGCAGCTAATTTCTGTAGTACCTTACTG
 Consensus     TCATGGCTACTTCATCCTCTAGCTCCTCCTGGTTTTCAATCAAACCTGAATCGTCATCATCATCACTCTGTCTGGCATGGCATGTCTGGTTCTGCTTCTTTGCAAGTTCACAAGTGCGAATATTATGGCCAGCTCCTCCACAGGCTGTGCAGTACTTTGGAAATGATTTAGTGGCAGCTAATTTCTGTAGTACCTTACTG

               601                                                                                                                                                                                                    800
NMRB01000727.1 GTGTACAGCCATTGAAAAACCAATGCATCTCTAAGGTCGCAGTCATCAGAAGTGTCTTGCCTGCTCAGAGATTTACGAATAAACCTATACAACTTATTGCCACACTCCCCACCTTCACTGGAGAGAGCACCAACACTGTGAATTCCCTGAGGATCCTGAATTATTTCTGCCACATGCTCCACAACTTTGTGGACATAATT
GFRY01046940.1 GTGTACAGCCATTGAAAAACCAATGCATCTCTAAGGTCGCAGTCATCAGAAGTGTCTTGCCTGCTCAGAGATTTACGAATAAACCTATACAACTTATTGCCACACTCCCCACCTTCACTGGAGAGAGCACCAACACTGTGAATTCCCTGAGGATCCTGAATTATTTCTGCCACATGCTCCACAACTTTGTGGACATAATT
GFRY01046941.1 GTGTACAGCCATTGAAAAACCAATGCATCTCTAAGGTCGCAGTCATCAGAAGTGTCTTGCCTGCTCAGAGATTTACGAATAAACCTATACAACTTATTGCCACACTCCCCACCTTCACTGGAGAGAGCACCAACACTGTGAATTCCCTGAGGATCCTGAATTATTTCTGCCACATGCTCCACAACTTTGTGGACATAATT
 Consensus     GTGTACAGCCATTGAAAAACCAATGCATCTCTAAGGTCGCAGTCATCAGAAGTGTCTTGCCTGCTCAGAGATTTACGAATAAACCTATACAACTTATTGCCACACTCCCCACCTTCACTGGAGAGAGCACCAACACTGTGAATTCCCTGAGGATCCTGAATTATTTCTGCCACATGCTCCACAACTTTGTGGACATAATT

               801                                                                                                                                                                                                    1000
NMRB01000727.1 GGACCAAATCACATAAGGAAATTGTTGTCCCAGAAGGAATGCGAACTGCAGTGCATTTTCTTTGTACTGTGCCATTTCCTCTGGTAATGTCTGGACAGGATTTTTACAAGAATACACTGCATGCATCTTGCTGAAGTGACCAAGGAGAGCTTTAACATGCTGCTTTCTTTCTTCATTTCTGATTAACTTTGTGACAATAT
GFRY01046940.1 GGACCAAATCACATAAGGAAATTGTTGTCCCAGAAGGAATGCGAACTGCAGTGCATTTTCTTTGTACTGTGCCATTTCCTCTGGTAATGTCTGGACAGGATTTTTACAAGAATACACTGCATGCATCTTGCTGAAGTGACCAAGGAGAGCTTTAACATGCTGCTTTCTTTCTTCATTTCTGATTAACTTTGTGACAATAT
GFRY01046941.1 GGACCAAATCACATAAGGAAATTGTTGTCCCAGAAGGAATGCGAACTGCAGTGCATTTTCTTTGTACTGTGCCATTTCCTCTGGTAATGTCTGGACAGGATTTTTACAAGAATACACTGCATGCATCTTGCTGAAGTGACCAAGGAGAGCTTTAACATGCTGCTTTCTTTCTTCATTTCTGATTAACTTTGTGACAATAT
 Consensus     GGACCAAATCACATAAGGAAATTGTTGTCCCAGAAGGAATGCGAACTGCAGTGCATTTTCTTTGTACTGTGCCATTTCCTCTGGTAATGTCTGGACAGGATTTTTACAAGAATACACTGCATGCATCTTGCTGAAGTGACCAAGGAGAGCTTTAACATGCTGCTTTCTTTCTTCATTTCTGATTAACTTTGTGACAATAT

               1001                                                                                                                                                                                                   1200
NMRB01000727.1 CAACATTCTTGGGGTCAAATAGTGCTCTGGCAAAATTTCCAACCATTATAAGTTTTGACTGCAGACCAAGAGTTTTCATGAGGTGTTCATCCAATGTCTTCTCAGCATGATGCAATGTAGTTTTATTCTGCTCATTTTCTGACCATTGATGGACTTCAGCAATTTCCCTCACAAGTATCTTCTTGAAAAACCTGCCAATA
GFRY01046940.1 CAACATTCTTGGGGTCAAATAGTGCTCTGGCAAAATTTCCAACCATTATAAGTTTTGACTGCAGACCAAGAGTTTTCATGAGGTGTTCATCCAATGTCTTCTCAGCATGATGCAATGTAGTTTTATTCTGCTCATTTTCTGACCATTGATGGACTTCAGCAATTTCCCTCACAAGTATCTTCTTGAAAAACCTGCCAATA
GFRY01046941.1 CAACATTCTTGGGGTCAAATAGTGCTCTGGCAAAATTTCCAACCATTATAAGTTTTGACTGCAGACCAAGAGTTTTCATGAGGTGTTCATCCAATGTCTTCTCAGCATGATGCAATGTAGTTTTATTCTGCTCATTTTCTGACCATTGATGGACTTCAGCAATTTCCCTCACAAGTATCTTCTTGAAAAACCTGCCAATA
 Consensus     CAACATTCTTGGGGTCAAATAGTGCTCTGGCAAAATTTCCAACCATTATAAGTTTTGACTGCAGACCAAGAGTTTTCATGAGGTGTTCATCCAATGTCTTCTCAGCATGATGCAATGTAGTTTTATTCTGCTCATTTTCTGACCATTGATGGACTTCAGCAATTTCCCTCACAAGTATCTTCTTGAAAAACCTGCCAATA

               1201                                                                                                                                                                                                   1400
NMRB01000727.1 TTTATGTTTGCATGAGTTGAGTCTAATGATCTTTCAGTAGCATCTGAGACAATCAGAGGCATGCTGTTCACACCTTTGCATTTTACTGCAAGGTCATTATCATTCAGATGGTCAGGATTTTCAAGGCGATACTTGTAGAGGGAAACTGTTTCTTCGTGTGTCCTGCACCTTGTAAAGGAGCCAACATCACTTTGACAGTT
GFRY01046940.1 TTTATGTTTGCATGAGTTGAGTCTAATGATCTTTCAGTAGCATCTGAGACAATCAGAGGCATGCTGTTCACACCTTTGCATTTTACTGCAAGGTCATTATCATTCAGATGGTCAGGATTTTCAAGGCGATACTTGTAGAGGGAAACTGTTTCTTCGTGTGTCCTGCACCTTGTAAAGGAGCCGACATCACTTTGACAGTT
GFRY01046941.1 TTTATGTTTGCATGAGTTGAGTCTAATGATCTTTCAGTAGCATCTGAGACAATCAGAGGCATGCTGTTCACACCTTTGCATTTTACTGCAAGGTCATTATCATTCAGATGGTCAGGATTTTCAAGGCGATACTTGTAGAGGGAAACTGTTTCTTCGTGTGTCCTGCACCTTGTAAAGGAGCCGACATCACTTTGACAGTT
 Consensus     TTTATGTTTGCATGAGTTGAGTCTAATGATCTTTCAGTAGCATCTGAGACAATCAGAGGCATGCTGTTCACACCTTTGCATTTTACTGCAAGGTCATTATCATTCAGATGGTCAGGATTTTCAAGGCGATACTTGTAGAGGGAAACTGTTTCTTCGTGTGTCCTGCACCTTGTAAAGGAGCCgACATCACTTTGACAGTT

               1401                                                                                                                                                                                                   1600
NMRB01000727.1 GCCAGTATGTGCATGGCACAAAGTACACAGATACCGCGAGCCACTTCCTTGAAGTCCGGAGGATGTTCGCTCGAACTTCTCATCAACCATACTGTAAAGGAAATGTAGTTCATGCTCTCTCAGGCAGTGACCAGTACTGGCATCATATAATTGCAATGTGGCTCCTTTCATTTTCTCCCTGTCTCTTGAGACTTTCCCCA
GFRY01046940.1 GCCAGTATGTGCATGGCACAAAGTACACAGATACCGCGAGCCACTTCCTTGAAGTCCGGAGGATGTTCGCTCGAACTTCTCATCAACCATACTGTAAAGGAAATGTAGTTCATGCTCTCTTAGGCAGTGACCAGTACTGGCATCATATAATTGCAATGTGGCTCCTTTCATTTTCTCCCTGTCTCTTGAGACTTTCCCCA
GFRY01046941.1 GCCAGTATGTGCATGGCACAAAGTACACAGATACCGCGAGCCACTTCCTTGAAGTCCGGAGGATGTTCGCTCGAACTTCTCATCAACCATACTGTAAAGGAAATGTAGTTCATGCTCTCTTAGGCAGTGACCAGTACTGGCATCATATAATTGCAATGTGGCTCCTTTCATTTTCTCCCTGTCTCTTGAGACTTTCCCCA
 Consensus     GCCAGTATGTGCATGGCACAAAGTACACAGATACCGCGAGCCACTTCCTTGAAGTCCGGAGGATGTTCGCTCGAACTTCTCATCAACCATACTGTAAAGGAAATGTAGTTCATGCTCTCTtAGGCAGTGACCAGTACTGGCATCATATAATTGCAATGTGGCTCCTTTCATTTTCTCCCTGTCTCTTGAGACTTTCCCCA

               1601                                                                                                                                                                                                   1800
NMRB01000727.1 GAAGTGTTGCAATACTTGGCCTGTCACTTTCATCACAATGACAGAGAATGACAGGCCGAGTACTATCAACAGAATTGGGGAACAACTCCTTGAATAGAACATGCTCTGTTTCATTTACCATGACAGAACACTGAAGACAACAGAAAGAATACCTGAAAATCTTGTCTGTTCTTGCAAATTCAGATTNTGATTGTTAACGA
GFRY01046940.1 GAAGTGTTGCAATACTTGGCCTGTCACTTTCATCACAATGACAGAGAATGACAGGCCGAGTACTATCAACAGAATTGGGGAACAACTCCTTGAATAGAACATGCTCTGTTTCATTTACCATGACAGAACACTGAAGACAACAGAAAGAATACCTGAAAATCTTGTCTGTTCTTGCAAATTCAGATTT-------------
GFRY01046941.1 GAAGTGTTGCAATACTCGGCCTGTCACTTTCATCACAATGACAGAGAATGACAGGCCGAGTACTATCAACAGAATTGGGGAACAACTCCTTGAATAGAACATGCTCTGTTTCATTTACCATGACAGAACACTGAAGACAACAGAAAGAATACCTGAAAATCTTGTCTGTTCTTGCAAATTCAGATTT-------------
 Consensus     GAAGTGTTGCAATACTtGGCCTGTCACTTTCATCACAATGACAGAGAATGACAGGCCGAGTACTATCAACAGAATTGGGGAACAACTCCTTGAATAGAACATGCTCTGTTTCATTTACCATGACAGAACACTGAAGACAACAGAAAGAATACCTGAAAATCTTGTCTGTTCTTGCAAATTCAGATTt.............

               1801                                                                                                                                                                                                   2000
NMRB01000727.1 ACTCCTGTGACTTCAGCAGTAGACTGATCCTGATAGCTGCCCAGAAGACCAGATACCAGTCTACCTCAGTAGTATCAGGTAAAGTCGGGTACTCGAACGAACTAAACTGGAACAAGTGTAATACTACAAGTGCCAGGTCAACAGTGACAACTCAGGCACTCAAACGAACTAAACTGTAATGGCTGGCACATCATGACATG
GFRY01046940.1 --------------------------------------------------------------------------------------------------------------------------------------------------------------------------------------------------------
GFRY01046941.1 --------------------------------------------------------------------------------------------------------------------------------------------------------------------------------------------------------
 Consensus     ........................................................................................................................................................................................................

               2001                                                                                                                                                                                                   2200
NMRB01000727.1 TGGCATGGCTTGGAAAGTCCATGCTTTGGGCATGTTTCTTCGCTGAATTTTGTGCTTATTTATGAAAATAGGCCTATGGGATGATGCAGGCCTTAATCCACATAGGAACCGCACTGTTGTATCACCCGCACTGGCGATTTTGGGGCCTTTTCGCGGAAAAAGTCGCGGGTCTTATGCCGAAAAATACGGTACAAGAAT
GFRY01046940.1 ------------------------------------------CTGAGTTTTTACATTAAT--------------------ATCACCCAGGCC----------------------------ATCCCCCCCCATCTTTGATGAAGGTCTTGACAGGAGTTAATGGTGGCAGGTC----------------------------
GFRY01046941.1 ------------------------------------------CTGAGTTTTTACATTAAT--------------------ATCACCCAGGCC----------------------------ATCCCCCCCCATCTTTGATGAAGGTCTTGACAGGAGTTAATGGTGGCAGGTC----------------------------
 Consensus     ..........................................CTGAgTTTTtacaTTAaT....................ATcAccCAGGCC............................ATCcCCCcCcCatctttgaTgaaGGtCtTgacaGgaGttAAtgGTgGcAGGTC............................

               2201                                                                                                                                                                                                   2400
NMRB01000727.1 GGCGCCTCATTGAAAATTAGCTCTTTCATTTTTTCACTCTGTTCAAAAGAGCAGTCCCTAAATTAGCTCTTTTAGAGCCAATTTCGCTCTTCCTTTTTTAGAGAGCACCGCGAGGGAGTGGGCGGTCGTAAAGGGCATGGACATTCAATCTCGCACCCAGATCTACCTCATCCGAGGATATCTCTGGCAAGGGCTACGAC
GFRY01046940.1 ----------------------------------------------AAGTTCACGAAGTGAACATGCTATTTTATCATCCAATTCAGACAACGATTTTGCAACTGA----------------------------------------------------------------------------------------------
GFRY01046941.1 ----------------------------------------------AAGTTCACGAAGTGAACATGCTATTTTATCATCCAATTCAGACAACGATTTTGCAACTGA----------------------------------------------------------------------------------------------
 Consensus     ..............................................AAGttCAcgaagTgAAcatGCTaTTTTAtcatCcAaTTCagaCaaCgaTTTTgcaActGa..............................................................................................

               2401                                                                                                                                                                                                   2600
NMRB01000727.1 ATGACCAAAATTTCTGGTCAACCGCTGGTAGCGTGCCAAAATGTTTGTTTCATATATGCCTAAAAGCCATATAATACACCCAAATCACGAAGTTGTTTGAGTATTAGAGCAATTTCAAACGAATAGTTTAGTTGGTAACATCATGCCGCAGGTGCCAGCCTCGGCGTGATTTGCCTCAAAATTAGTACTGTATATCGCG
GFRY01046940.1 ------------------------------------------------------------TGAAACATAATCGAATTCAACCCCAGCAAGATTTGGA---------AGAGGAAAATCAGCAGGAAAATTTTTGTATTTTCTCATTAT--------------------GTCCTTTGGTTCACAATCTTTCCCTTTAA----
GFRY01046941.1 ------------------------------------------------------------TGAAACATAATCGAATTCAACCCCAGCAAGATTTGGA---------AGAGGAAAATCAGCAGGAAAATTTTTGTATTTTCTCATTAT--------------------GTCCTTTGGTTCACAATCTTTCCCTTTAA----
 Consensus     ............................................................TgAAAcataATcgAATtCAaCCccAgCAaGAttTgGa.........AGAGgAAaaTCAgcaGgAaAaTTTttgTatTttCtcatTat....................GTccTTTGgtTCAcAATcttTcCctTtaA....

               2601                                                                                                                                                                                                   2800
NMRB01000727.1 CATGCGCAGTATTCCTGAAGTCGTCAGCCCTTTCCAGTGGTGTTGTAGGAACGTAAACCCACTGGGGACGAGTTTGCTGGACATTTGACACATCTTTCTCATGCAGTGTTTCCGTGACAGGGTAAGGGTACTATGGTACAGTACTGCTTGAATTTAAGTGACTTCAGATCGCGATCAGAACCCACACTCGGATCGCGATC
GFRY01046940.1 -------------------------------------------------------------------GAATATCCTGTTGGGCAATTACTTCTGAATTCTTAACAAGTCTGTAGGCACAATGGCCAGGCAAATATGATG---------------------------GTTCAGCATCAGTCAATTGATTGGGA--------
GFRY01046941.1 -------------------------------------------------------------------GAATATCCTGTTGGGCAATTACTTCTGAATTCTTAACAAGTCTGTAGGCACAATGGCCAGGCAAATATGATG---------------------------GTTCAGCATCAGTCAATTGATTGGGA--------
 Consensus     ...................................................................GAatAtccTGtTGGgCAaTTacttCtgaaTTCTtAacaAGTcTgTagGcacaAtGGccAGGcaAaTATGaTg...........................GtTCagcATCAGtcaattgAtTgGGA........

               2801                                                                                                                                                                                                   3000
NMRB01000727.1 AGAGCGCGCACTTCTTACTTCGGGTGGNGGATCGCATTGGGTAACGTTTACATGCAAGAGTTTTGAACCACAATCGGATTCGATCGGATCGCATGTGGATTTTGCCTGTCGTCTAAACGGTCCTAGTTAGAACATGGTATATTGTTTTGAGCCAAGTACATTTTTCAAGATGGACGACTGGTGGCTATATTTGAAGTCCA
```

```
GFRY01046940.1  --------------------------------------GGCTTTAATGATGAAGCAGTAGTTTTGTCCCGC----------------------------------------------------------------------------TGAATACTGTACATCTGCCTGTAGTGAGTTTTTGACTGTAAAGTCCTAATCCG
GFRY01046941.1  --------------------------------------GGCTTTAATGATGAAGCAGTAGTTTTGTCCCGC----------------------------------------------------------------------------TGAATACTGTACATCTGCCTGTAGTGAGTTTTTGACTGTAAAGTCCTAATCCG
 Consensus      ......................................GGcTttaaTgatgAaGCAgtAGTTTTGtcCCgC............................................................................TGAatactGTACATcTgcCtgtAgtGAgtttTtGactgTAaAgTcctAaTCCg

                3001                                                                                                                                                                                                        3200
NMRB01000727.1  ATCCGGCTGAAATTTCTTGTATTTGTATAGGGTACCTTATATTCATACCAAGATACAACATTCTAGCTGCTGTTCTTCATGAGATATCGGCTAAATAGTCCCGTTTTCAAGATGGCCGACTGACCACCATATTTGAAGATTGAAAACGGTCAAAATTCAATGTACGCATAGTGGGTACCGAGATACATACACTTTCTACG
GFRY01046940.1  AATTGCCAGGCATT-----------------------------CATCCGCTGATAATTCATTCCTTCCCGAGGCCCATATAGACACTACACTATTCGCCTCTCTCTTCCGTCCAGCATCAAGGAGTTTTTGAATTAACATAGCAAACA----------------------------------ATACATCAACTTGCTCCT
GFRY01046941.1  AATTGCCAGGCATT-----------------------------CATCCGCTGATAATTCATTCCTTCCCGAGGCCCATATAGACACTACACTATTCGCCTCTCTCTTCCGTCCAGCATCAAGGAGTTTTTGAATTAACATAGCAAACA----------------------------------ATACATCAACTTGCTCCT
 Consensus      AattGcCaGgcATT.............................CATcCgctGATAattCATTCcttCccgaGgcCcatATagacacTacaCTAttcgcctCtcTcTTCcgtccaGCatcaaGgagttttTgaaTtAAcATaGcAAACa..................................ATACATcaACTTgCTcCt

                3201                                                                                                                                                                                                        3400
NMRB01000727.1  TCAAAATATTCTAGTTGGAGTAATTTACGAGATACCGGCCAAAAGCCATTTCCAAGATGGACGCCCGGCAGCCATGTTTGAGGTAGAATCGGGCCGAAATTTGCTGAACACATAGAGGGAATCTAGATACATAATCACAGCAAGTTAGAAGATTCCAGGTGAAATAGCCACAAAACACGCAAACAAATGGCAAAATTTG
GFRY01046940.1  CCTTTCTTTCCTCACAGAAAATGCTCACAGAAGAA------------------AAAAGGTCCTTAAGCCTCCTTTGCCTGATGTGGTGTC-----------TGCTGACCTGTTACAAATTTGCTTTGGAAATATTTCCAGC-----------------------------------------------------------
GFRY01046941.1  CCTTTCTTTCCTCACAGAAAATGCTCACAGAAGAA------------------AAAAGGTCCTTAAGCCTCCTTTGCCTGATGTGGTGTC-----------TGCTGACCT------------------------------------------------------------------------------------------
 Consensus      cCtttcTtTcCTcacaGaAaatgcTcACagaAgAa..................AAaAgGtcCttaaGcCtcCttTGccTGAtGTgGtgTC...........TGCTGAcCt..ta.a......ct....a.ata.t..cagc...........................................................

                3401                                                                                                                                                                                                        3600
NMRB01000727.1  AACTTTGACACTGCGACTTAAAAGCAGGTCAAATCAAAAAGCCGTTGATATGTCATGTATCCTTATTAGATGTGCACCTATGATAAAAATTTGGAGATGATCCAGCCAGTACTTCAGGAGTTATTGCACTTTTTGTGTTTTCGGTTTGGGGTTCCTAATGGCCAAGCCAACGAATCGGACCGAAACATGGTGCC--ATGG
GFRY01046940.1  --------------------------------------------------------GGCTCGTCAATGACTGAAATTCCGTAATGCACCTTGACGGTTTCTGCAGCCAGGGTGATATCATAGAAGCTTTTCTGTCGCTTTACTGCCTTCAGATCTTCATTACAGAGGCGACAAATACGACTTAAAACGTCCACATGATGT
GFRY01046941.1  ------------------------------------------------------------------------------------------TGACGGTTTCTGCAGCCAGGGTGATATCATAGAAGCTTTTCTGTCGCTTTACTGCCTTCAGATCTTCATTACAGAGGCGACAAATACGACTTAAAACGTCCACATGATGT
 Consensus      ........................................................g..tc.t.a.t...tg.....c..t.at..a..tTgacgGtTtcTgCAGCCAGggtgatAtcAtagAagcttttTcTgTcgcTTTaCtGccTtcaGaTCtTcATtaCagAGgCgACaAATacGACttAAAacgtccaCatgATGt

                3601                                                                                                                                                                                                        3800
NMRB01000727.1  GTCAGCTGCCCTATAGGGGTATACATCTATATATACAAAGTTTCAGGTCAATAGCCACAGCTGTTTACGAAACGTGCTCCGCTAAAAACTGGCGTCCACTAGGCAGCGGACAAGAGGCGGAATGCAAAATTAGTAAATCCGCATTCATTCAGCAACCTCTCCGCATATTCCTGAAAGATTCCATTGACTGTCTTTATCAT
GFRY01046940.1  TGCAGCTCCTCCATGGTGGTGCCTTGC-------------------------------------------------------------CTTGGTGACACTTGACAGCTTAGTAAAATCCCAAAAATCAGCTTAAAAATCCATA-----------------------------GAAAAACTAATTTTGCTTCGATTTTCGA
GFRY01046941.1  TGCAGCTCCTCCATGGTGGTGCCTTGC-------------------------------------------------------------CTTGGTGACACTTGACAGCTTAGTAAAATCCCAAAAATCAGCTTAAAAATCCATA-----------------------------GAAAAACTAATTTTGCTTCGATTTTCGA
 Consensus      tgCAGCTcCtCcATgGtGGTgccttgC.............................................................CTtGgtgaCACTtGaCAGCttAgtAaAatCccAAaaatcAgcTtaaAAATCCatA.............................GAAAaAcTaatTTtgCTtcgaTTtTCga

                3801                                                                                                                                                                                                        4000
NMRB01000727.1  TCCTAAACATTTCCCGGCATTTCTGCACTTTCGGGAGAGGGCAAAATGACCAATAGAGAGTTTTCGCAAAGCCCCCGAAACGACGACGCGAACGCCTATCCTGTTGTTCGCTGCTCCTTTCGTGGCATCGAACAATGAGATTGGCGTTCGCGTTGGCGTTTCGGGGGCTTTGCGAAAACTCCCTATTGGTCATTGACGCG
GFRY01046940.1  GCTTGAAGCGGCTCCG----TGTAGCTCGTTCTGTGCCACACAAACTTAGTGACATTGCGCATCCGTAATTATCATAGAAGACCGTAAGGAGA-------------------------TTCGGGCATGGGACCAAGTGTCATATCGCGCAGCTTCCTATCGGGGGTCACGTGATTAGGGATTAGTAGTGTTATGGCGGCG
GFRY01046941.1  GCTTGAAGCGGCTCCG----TGTAGCTCGTTCTGTGCCACACAAACTTAGTGACATTGCGCATCCGTAATTATCATAGAAGACCGTAAGGAGA-------------------------TTCGGGCATGGGACCAAGTGTCATATCGCGCAGCTTCCTATCGGGGGTCACGTGATTAGGGATTAGTAGTGTTATGGCGGCG
 Consensus      gCtTgAAgcggctCCG....TgtaGCtCgTTCtGtgccacaCAAAcTtAgtgAcAttGcGcaTcCGtAAttatCatagAAgacCGtaagGAga.........................TTCGgGcatggGAcCAAgtgtcaTatCGcgCagcTTcctaTcggGGGtcacgTGattAgggattagtagtGTtATgGcgGCG

                4001                                                                                                                                                                                                        4200
NMRB01000727.1  GAAAGAATGCCGAAATGACATGATTTGCTTCCGCAACCCTTCCTAATCTTGTCGCCGCTCTATAGAGGACGCCACCCATAACGGACGAGGGAAGACGAAGCTCAACGTGAGGATTTTTTAGTAATGTGGTTTCTCGCAGTCATATGCATAAAATAACAAAAAAATCGCGTTTAAGATATATTTTTCCACGTTTTGGGAT
GFRY01046940.1  TACAGA------------------TAGATTTAGCTACACACGCTATGTTTCGGTGTGGTATCCCTAGCTCCGCATCCATCACGTCCAGCAG---------------------------TGACAATTTGCAATCATTAAGAAAAATAAATTACATATGAAACAGAGGAAGTAGCATTCAGGAATTCTAA
GFRY01046941.1  TACAGA------------------TAGATTTAGCTACACACGCTATGTTTCGGTGTGGTATCCCTAGCTCCGCATCCATCACGTCCAGCAG---------------------------TGACAATTTGCAATCATTAAGAAAAATAAATTACATATGAAACAGAGGAAGTAGCATTCAGGAATTCTAA
 Consensus      tAcAGA..................TaGaTTtaGCtACaCacgCTAtgtTTcggtgtGgTaTccctAGctCcgCAtCCATcACGtcCagcaG...........................TgacAATtTGcaaTCattaAGaaAaATaaATtAcATAtgAAAcAgAggaaGTagcAttcAggaaTTcTaa............

                4201                                                                                                                                                                                                        4400
NMRB01000727.1  AAGATTGGTCTCTCCTACCAGTCTAGAGTCCGGCCGATGTTGAGTGCACTGCCAGATCTGATCGCCCTCCTAACGTCGGCCGGATCGCGAGGCCCACGACAGCTTGTGCAACAGGTCAGTGCAGTAGGCNNNNNNNNNNNNNNNNNNNNNNNNNNNNNNNNNNNNNNNNNNNNNNNNNNNNNNNNNNNNNNNNNNNNN
GFRY01046940.1
GFRY01046941.1
 Consensus      ........................................................................................................................................................................................................
```

```
>TSA:GFRY01046940.1:beg-end
```

5'3' Frame 1

```
LEFLNATSSVSYVIYFS--LQIVTAGRDGCGARDTTPKHSVCS-IYLYAAITLLIPNHVTPDRKLRDMTLGPMPESPYGLL--LRMRNVTKFVWHRTSYTEPLQARKSKQN-FFYGFLS-FLGFY-AVKCHQGKAPPWRSCNIMWTF-VVFVASVMKI-RQ-SDRKASMISPWLQKPSRCITEFQSLTSRWKYFQSKFVTGQQTPHQAKEA-GPFFFCE
HFL-GKKGGAS-CIVCYVNSKTP-CWTEERGE-CSVYMGLGKE-IISG-MPGNSD-DFTVKNSLQADVQYSAGQNYCFIIKASQSID-C-TIIFAWPLCLQTC-EFRSNCPTGYS-RERL-TKGHNEKIQKFSC-FSSSKSCWG-IRLCFISCKIVV-IG--NSMFTS-T-PATINSCQDLHQRWGGDGLGDINVKTQKSEFARTDKIFRYSFCCLQCS
VMVNETEHVLFKELFPNSVDSTRPVILCHCDESDRPSIATLLGKVSRDREKMKGATLQLYDASTGHCLREHELHFLYSMVDEKFERTSSGLQGSGSRYLCTLCHAHTGNCQSDVGSFTRCRTHEETVSLYKYRLENPDHLNDNDLAVKCKGVNSMPLIVSDATERSLDSTHANINIGRFFKKILVREIAEVHQWSENEQNKTTLHHAEKTLDEHLMKTL
GLQSKLIMVGNFARALFDPKNVDIVTKLIRNEERKQHVKALLGHFSKMHAVYSCKNPVQTLPEEMAQYKENALQFAFLLGQQFPYVIWSNYVHKVVEHVAEIIQDPQGIHSVGALSSEGGECGNKLYRFIRKSLSRQDTSDDCDLRDALVFQWLYTSKVLQKLAATKSFPKYCTACGGAGHNIRTCELAKKQNQTCHARQSDDDDDSGLIENQEELEDE
VAMICVNCM-KVKCETLGPRHYTNNVEDSCLRNEMNCGYSIMIKWTFYSLCHTYGGPCW-IQDM-YHVEILLLRFPFSSLFLSFAFFLVLHLAQVDVF-FLRVEKFIAGITLQTCSGFTR
```

```
>TSA:GFRY01046941.1:beg-end
```

5'3' Frame 2

```
-NS-MLLPLFHM-FIFLNDCKLSLLDVMDAELGIPHRNIACVAKSICTPP-HY-SLIT-PPIGSCAI-HLVPCPNLLTVFYDNYGCAMSLSLCGTERATRSRFKLENRSKISFSMDF-ADFWDFTKLSSVTKARHHHGGAATSCGRFKSYLSPL--RSEGSKATEKLL-YHPGCRNRQGQQTPHQAKEA-GPFFFCEHFL-GKKGGAS-CIVCYVNSKT
P-CWTEERGE-CSVYMGLGKE-IISG-MPGNSD-DFTVKNSLQADVQYSAGQNYCFIIKASQSID-C-TIIFAWPLCLQTC-EFRSNCPTGYS-RERL-TKGHNEKIQKFSC-FSSSKSCWG-IRLCFISCKIVV-IG--NSMFTS-T-PATINSCQDLHQRWGGDGLGDINVKTQKSEFARTDKIFRYSFCCLQCSVMVNETEHVLFKELFPNSVDST
RPVILCHCDESDRPSIATLLGKVSRDREKMKGATLQLYDASTGHCLREHELHFLYSMVDEKFERTSSGLQGSGSRYLCTLCHAHTGNCQSDVGSFTRCRTHEETVSLYKYRLENPDHLNDNDLAVKCKGVNSMPLIVSDATERSLDSTHANINIGRFFKKILVREIAEVHQWSENEQNKTTLHHAEKTLDEHLMKTLGLQSKLIMVGNFARALFDPKNV
DIVTKLIRNEERKQHVKALLGHFSKMHAVYSCKNPVQTLPEEMAQYKENALQFAFLLGQQFPYVIWSNYVHKVVEHVAEIIQDPQGIHSVGALSSEGGECGNKLYRFIRKSLSRQDTSDDCDLRDALVFQWLYTSKVLQKLAATKSFPKYCTACGGAGHNIRTCELAKKQNQTCHARQSDDDDDSGLIENQEELEDEVAMICVNCM-KVKCETLGPRHY
TNNVEDSCLRNEMNCGYSIMIKWTFYSLCHTYGGPCW-IQDM-YHVEILLLRFPFSSLFLSFAFFLVLHLAQVDVF-FLRVEKFIAGITLQTCSGFTR
```

Notospermus geniculatus (Nge)

WGS: NMRB01004133.1

1717-6162
Notospermus geniculatus strain Ushimado scaffold4133, whole genome shotgun sequence

```
        H  R  Q  N  G  S  A  R  S  G  R  F  S  A  Q  F  T  L  F  F  A  C  K  S  N  *  K  A  Q  K  A  N  K  K  S  N  C  C  *  C  C  R  E  T  L  F  L  W  E  R  A  S  H  F  L  *  K  R  Q  *  G  R  P  R  R  P  G   F1
         T  D  K  M  A  A  P  V  P  V  D  F  L  H  N  S  R  F  F  S  L  A  K  A  I  K  K  R  R  K  L  T  K  K  A  I  A  A  D  A  A  E  K  L  F  F  S  G  K  G  H  L  I  F  S  E  N  V  S  E  D  D  Q  G  G  Q  E  F2
       Q  T  K  W  Q  R  P  F  R  *  I  F  C  T  I  H  A  F  F  R  L  Q  K  Q  L  K  S  A  E  S  *  Q  K  K  Q  L  L  L  M  L  Q  R  N  S  F  S  L  G  K  G  I  S  F  S  L  K  T  S  V  R  T  T  K  A  A  R    F3
   1 CACAGACAAAATGGCAGCGCCCGTTCCGGTAGATTTTCTGCACAATTCACGCTTTTTTTCGCTTGCAAAAGCAATTAAAAAGCGCAGAAAGCTAACAAAAAAAGCAATTGCTGCTGATGCTGCAGAGAAACTCTTTTTCTCTGGGAAAGGGCATCTCATTTTCTCTGAAAACGTCAGTGAGGACGACCAAGGCGGCCAGG 200
     ----:----|----:----|----:----|----:----|----:----|----:----|----:----|----:----|----:----|----:----|----:----|----:----|----:----|----:----|----:----|----:----|----:----|----:----|----:----|----:----|
   1 GTGTCTGTTTTACCGTCGCGGGCAAGGCCATCTAAAAGACGTGTTAAGTGCGAAAAAAAGCGAACGTTTTCGTTAATTTTTCGCGTCTTTCGATTGTTTTTTTCGTTAACGACGACTACGACGTCTCTTTGAGAAAAAGAGACCCTTTCCCGTAGAGTAAAAGAGACTTTTGCAGTCACTCCTGCTGGTTCCGCCGGTCC 200
       X  L  C  F  P  L  A  R  E  P  L  N  E  A  C  N  V  S  K  K  A  Q  L  L  L  *  F  A  C  F  A  L  L  F  L  L  Q  Q  Q  H  Q  L  S  V  R  K  R  Q  S  L  A  D  *  K  R  Q  F  R  *  H  P  R  G  L  R  G  P   F6
      X  C  V  F  H  C  R  G  N  R  Y  I  K  Q  V  I  *  A  K  K  R  K  C  F  C  N  F  L  A  S  L  *  C  F  F  C  N  S  S  I  S  C  L  F  E  K  E  R  P  F  P  M  E  N  E  R  F  V  D  T  L  V  V  L  A  A  L    F5
     V  S  L  I  A  A  G  T  G  T  S  K  R  C  L  E  R  K  K  E  S  A  F  A  I  L  F  R  L  F  S  V  F  F  A  I  A  A  S  A  A  S  F  S  K  K  E  P  F  P  C  R  M  K  E  S  F  T  L  S  S  S  W  P  P  W     F4

       G  G  G  *  T  S  Q  *  *  *  *  A  *  T  A  C  A  C  Q  C  A  W  W  T  A  *  *  R  A  D  D  Y  T  V  R  H  N  F  S  V  C  F  Y  G  G  *  R  *  H  C  H  R  I  H  F  *  K  K  M  C  W  R  S  D  S  S  T   F1
        E  E  G  R  L  H  S  D  D  D  E  H  E  R  R  V  L  V  N  V  L  G  G  L  H  D  K  E  P  M  T  T  L  S  G  T  I  F  Q  Y  V  F  T  V  G  E  D  D  I  A  I  E  S  I  S  E  K  K  C  A  G  A  Q  I  H  L    F2
      R  R  V  D  F  T  V  M  M  M  S  M  N  G  V  C  L  S  M  C  L  V  D  C  M  I  K  S  R  *  L  H  C  Q  A  Q  F  F  S  M  F  L  R  W  V  K  M  T  L  P  *  N  P  F  L  K  K  N  V  L  A  L  R  F  I  Y     F3
 201 AGGAGGAGGGTAGACTTCACAGTGATGATGATGAGCATGAACGGCGTGTGCTTGTCAATGTGCTTGGTGGACTGCATGATAAAGAGCCGATGACTACACTGTCAGGCACAATTTTTCAGTATGTTTTTACGGTGGGTGAAGATGACATTGCCATAGAATCCATTTCTGAAAAAAAATGTGCTGGCGCTCAGATTCATCTA 400
     ----:----|----:----|----:----|----:----|----:----|----:----|----:----|----:----|----:----|----:----|----:----|----:----|----:----|----:----|----:----|----:----|----:----|----:----|----:----|----:----|
 201 TCCTCCTCCCATCTGAAGTGTCACTACTACTACTCGTACTTGCCGCACACGAACAGTTACACGAACCACCTGACGTACTATTTCTCGGCTACTGATGTGACAGTCCGTGTTAAAAAGTCATACAAAAATGCCACCCACTTCTACTGTAACGGTATCTTAGGTAAAGACTTTTTTTTACACGACCGCGAGTCTAAGTAGAT 400
       P  P  P  Y  V  E  C  H  H  H  A  H  V  A  H  A  Q  *  H  A  Q  H  V  A  H  Y  L  A  S  S  *  V  T  L  C  L  K  E  T  H  K  *  P  P  H  L  H  C  Q  W  L  I  W  K  Q  F  F  I  H  Q  R  E  S  E  D    F6
      L  L  L  T  S  K  V  T  I  I  I  L  M  F  P  T  H  K  D  I  H  K  T  S  Q  M  I  F  L  R  H  S  C  Q  *  A  C  N  K  L  I  N  K  R  H  T  F  I  V  N  G  Y  F  G  N  R  F  F  F  T  S  A  S  L  N  M  *    F5
     S  S  S  P  L  S  *  L  S  S  S  S  C  S  R  R  T  S  T  L  T  S  P  P  S  C  S  L  S  G  I  V  V  S  D  P  V  I  K  *  Y  T  K  V  T  P  S  S  S  M  A  M  S  D  M  E  S  F  F  H  A  P  A  *  I  *  R     F4

       T  F  R  V  C  C  I  S  C  L  Q  N  F  S  E  R  Y  F  L  H  K  A  S  V  P  S  A  T  S  Q  E  Q  Q  D  R  S  L  Y  L  G  W  N  *  P  K  *  D  G  G  T  Q  *  P  D  S  S  R  D  L  R  F  V  L  F  C  D   F1
        R  P  F  V  Y  A  A  S  V  V  C  K  T  F  L  R  D  T  S  T  R  R  Q  C  R  Q  Q  Q  V  R  S  S  K  T  E  V  Y  I  W  G  G  I  D  P  N  R  M  V  A  H  N  D  L  T  V  L  E  T  Y  G  L  S  Y  F  A  T    F2
      D  L  S  C  M  L  H  Q  L  F  A  K  L  F  *  E  I  L  P  P  Q  G  V  S  A  V  S  N  K  S  G  A  A  R  Q  K  F  I  F  G  V  E  L  T  Q  I  G  W  W  H  T  M  T  *  Q  F  *  R  L  T  V  C  P  I  L  R  L     F3
 401 CGACCTTTCGTGTATGCTGCATCAGTTGTTTGCAAAACTTTTCTGAGAGATACTTCCTCCACAAGGCGTCAGTGCCGTCAGCAACAAGTCAGGAGCAGCAAGACAGAAGTTTATATTTGGGGTGGAATTGACCCAAATAGGATGGTGGCACACAATGACCTGACAGTTCTAGAGACTTACGGTTTGTCCTATTTTGCGAC 600
     ----:----|----:----|----:----|----:----|----:----|----:----|----:----|----:----|----:----|----:----|----:----|----:----|----:----|----:----|----:----|----:----|----:----|----:----|----:----|----:----|
 401 GCTGGAAAGCACATACGACGTAGTCAACAAACGTTTTGAAAAGACTCTCTATGAAGGAGGTGTTCCGCAGTCACGGCAGTCGTTGTTCAGTCCTCGTCGTTCTGTCTTCAAATATAAACCCCACCTTAACTGGGTTTATCCTACCACCGTGTGTTACTGGACTGTCAAGATCTCTGAATGCCAAACAGGATAAAACGCTG 600
       V  V  K  R  T  H  Q  M  L  Q  K  C  F  K  E  S  L  Y  K  R  W  L  A  D  T  G  D  A  V  L  *  S  C  C  S  L  L  K  Y  K  P  H  F  Q  G  L  Y  S  P  P  V  C  H  G  S  L  E  L  S  K  R  N  T  R  N  Q  S    F6
      S  R  E  H  I  S  C  *  N  N  A  F  S  K  Q  S  I  S  G  G  C  P  T  L  A  T  L  L  L  D  P  A  A  L  C  F  N  I  N  P  T  S  N  V  W  I  P  H  H  C  V  I  V  Q  C  N  *  L  S  V  T  Q  G  I  K  R    F5
     R  G  K  T  Y  A  A  D  T  Q  L  V  K  R  L  S  V  E  E  V  L  R  *  H  R  *  C  C  T  L  L  L  L  V  S  T  *  I  Q  P  P  I  S  G  F  L  I  T  A  C  L  S  R  V  T  R  S  V  *  P  K  D  *  K  A  V     F4

       S  S  *  *  R  *  *  K  Y  D  Q  E  T  A  E  W  Q  H  T  L  S  T  L  R  T  H  Y  E  H  Y  M  *  W  W  C  H  S  V  W  G  N  G  N  R  Q  P  A  N  *  Y  S  Y  N  T  G  W  V  T  L  C  F  G  F  A  *  I  Q   F1
        L  V  S  S  G  N  E  S  M  T  K  K  L  Q  S  G  S  I  P  S  P  R  Y  G  H  T  M  N  I  I  C  N  G  G  A  I  L  Y  G  G  M  E  I  D  S  Q  Q  I  D  I  H  T  T  Q  D  G  S  L  Y  V  L  D  L  H  E  F  R    F2
      *  L  V  A  V  M  K  V  *  P  R  N  C  R  V  A  A  Y  P  L  H  A  T  D  T  L  *  T  L  Y  V  M  V  V  P  F  C  M  G  E  W  K  S  T  A  S  K  L  I  F  I  Q  H  R  M  G  H  F  M  F  W  I  C  M  N  S     F3
 601 TCTAGTTAGTAGCGGTAATGAAAGTATGACCAAGAAACTGCAGAGTGGCAGCATACCCTCTCCACGCTACGGACACACTATGAACATTATATGTAATGGTGGTGCCATTCTGTATGGGGGAATGGAAATCGACAGCCAGCAAATTGATATTCATACAACACAGGATGGGTCACTTTATGTTTTGGATTTGCATGAATTCA 800
     ----:----|----:----|----:----|----:----|----:----|----:----|----:----|----:----|----:----|----:----|----:----|----:----|----:----|----:----|----:----|----:----|----:----|----:----|----:----|----:----|
 601 AGATCAATCATCGCCATTACTTTCATACTGGTTCTTTGACGTCTCACCGTCGTATGGGAGAGGTGCGATGCCTGTGTGATACTTGTAATATACATTACCACCACGGTAAGACATACCCCCTTACCTTTAGCTGTCGGTCGTTTAACTATAAGTATGTTGTGTCCTACCCAGTGAAATACAAAACCTAAACGTACTTAAGT 800
       E  L  *  Y  R  Y  H  F  Y  S  W  S  V  A  S  H  C  C  V  R  E  V  S  R  V  C  *  S  C  *  I  Y  H  H  H  W  E  T  H  P  F  P  F  R  C  G  A  F  Q  Y  E  Y  L  V  P  H  T  V  K  H  K  P  N  A  H  I  *    F6
      S  *  N  T  A  T  I  F  T  H  G  L  F  Q  L  T  A  A  Y  G  R  W  A  V  S  V  S  H  V  N  Y  T  I  T  T  G  N  Q  I  P  S  H  F  D  V  A  L  L  N  I  N  M  C  C  L  I  P  *  K  I  N  Q  I  Q  M  F  E    F5
     R  T  L  L  P  L  S  L  I  V  L  F  S  C  L  P  L  M  G  E  G  R  *  P  C  V  I  F  M  I  H  L  P  P  A  M  R  Y  P  P  I  S  I  S  L  W  C  I  S  I  *  V  V  C  S  P  D  S  *  T  K  S  K  C  S  N     F4

       V  D  Q  G  G  S  V  R  S  G  *  T  P  P  C  C  V  P  H  H  Y  N  I  E  R  F  Y  S  S  N  G  W  R  H  F  A  N  P  A  R  *  P  C  P  V  H  K  E  I  T  T  R  C  Y  M  H  C  C  *  K  N  W  C  *  H  N  C   F1
        W  T  K  V  V  V  L  G  Q  D  E  L  P  H  V  V  F  H  T  T  T  T  L  K  D  F  T  L  A  M  V  G  G  I  S  Q  T  L  Q  G  D  H  V  Q  Y  T  R  K  S  L  L  D  V  T  C  I  V  V  E  K  T  G  V  D  I  T    F2
      G  G  P  R  W  *  C  *  V  R  M  N  S  P  M  L  C  S  T  P  L  Q  H  *  K  I  L  L  *  Q  W  L  E  A  F  R  K  P  C  K  V  T  M  S  S  T  Q  G  N  H  Y  *  M  L  H  A  L  L  L  K  K  L  V  L  T  *  L     F3
 801 GGTGGACCAAGGTGGTAGTGTTAGGTCAGGATGAACTCCCCCATGTTGTGTTCCACACCACTACAACATTGAAAGATTTTACTCTAGCAATGGTTGGAGGCATTTCGCAAACCCTGCAAGGTGACCATGTCCAGTACACAAGGAAATCACTACTAGATGTTACATGCATTGTTGTTGAAAAAACTGGTGTTGACATAACT 1000
     ----:----|----:----|----:----|----:----|----:----|----:----|----:----|----:----|----:----|----:----|----:----|----:----|----:----|----:----|----:----|----:----|----:----|----:----|----:----|----:----|
 801 CCACCTGGTTCCACCATCACAATCCAGTCCTACTTGAGGGGGTACAACACAAGGTGTGGTGATGTTGTAACTTTCTAAAATGAGATCGTTACCAACCTCCGTAAAGCGTTTGGGACGTTCCACTGGTACAGGTCATGTGTTCCTTTAGTGATGATCTACAATGTACGTAACAACAACTTTTTTGACCACAACTGTATTGA 1000
       T  S  W  P  P  L  T  L  D  P  H  V  G  G  H  Q  T  G  C  W  *  L  M  S  L  N  *  E  L  L  P  Q  L  C  K  A  F  G  A  L  H  G  H  G  T  C  L  S  I  V  V  L  H  *  M  C  Q  Q  Q  F  F  Q  H  Q  C  L    F6
      P  P  G  L  H  Y  H  *  T  L  I  F  E  G  M  N  H  E  V  G  S  C  C  Q  F  I  K  S  *  C  H  N  S  A  N  R  L  G  Q  L  T  V  M  D  L  V  C  P  F  *  *  *  I  N  C  A  N  N  N  F  F  S  T  N  V  Y  S    F5
     L  H  V  L  T  T  T  N  P  *  S  S  S  G  W  T  T  N  W  V  V  V  V  N  F  S  K  V  R  A  I  T  P  P  M  E  C  V  R  C  P  S  W  T  W  Y  V  L  F  D  S  S  S  T  V  H  M  T  T  S  F  V  P  T  S  M  V     F4

       I  N  L  P  P  S  T  S  I  Q  W  T  Y  G  *  D  G  V  F  I  R  T  Y  C  A  S  V  *  S  *  L  S  F  H  F  W  R  V  S  G  E  C  C  R  P  Q  Q  S  T  K  Q  A  I  L  L  S  S  L  G  Y  I  S  C  K  Q  V   F1
        A  S  I  Y  H  P  Q  L  Q  F  N  G  P  M  D  E  M  V  Y  L  S  G  H  T  A  H  L  C  S  P  S  C  L  F  I  F  G  G  Y  Q  G  N  A  A  D  H  N  N  P  P  S  R  Q  Y  F  S  V  H  L  D  T  S  L  V  S  R  F    F2
      H  Q  F  T  T  L  N  F  N  S  M  D  L  W  M  R  W  C  I  Y  Q  D  I  L  R  I  C  V  V  L  A  V  F  S  F  L  A  G  I  R  G  M  L  Q  T  T  T  I  H  Q  A  G  N  T  S  Q  F  T  W  I  H  L  L  *  A  G  S     F3
1001 GCATCAATTTACCACCCTCAACTTCAATTCAATGGACCTATGGATGAGATGGTGTATTTATCAGGACATACTGCGCATCTGTGTAGTCCTAGCTGTCTTTTCATTTTTGGCGGGTATCAGGGGAATGCTGCAGACCACAACAATCCACCAAGCAGGCAATACTTCTCAGTTCACTTGGATACATCTCTTGTAAGCAGGT 1200
     ----:----|----:----|----:----|----:----|----:----|----:----|----:----|----:----|----:----|----:----|----:----|----:----|----:----|----:----|----:----|----:----|----:----|----:----|----:----|----:----|
1001 CGTAGTTAAATGGTGGGAGTTGAAGTTAAGTTACCTGGATACCTACTCTACCACATAAATAGTCCTGTATGACGCGTAGACACATCAGGATCGACAGAAAAGTAAAAACCGCCCATAGTCCCCTTACGACGTCTGGTGTTGTTAGGTGGTTCGTCCGTTATGAAGAGTCAAGTGAACCTATGTAGAGAACATTCGTCCA 1200
       Q  M  L  K  G  G  E  V  E  I  *  H  V  *  P  H  S  P  T  N  I  L  V  Y  Q  A  D  T  Y  D  *  S  D  K  *  K  Q  R  T  D  P  S  H  Q  L  G  C  C  D  V  L  C  A  I  S  R  L  E  S  P  Y  M  E  Q  L  C  T    F6
      C  *  N  V  V  R  L  K  L  E  I  S  R  H  I  L  H  H  I  *  *  S  M  S  R  M  Q  T  T  R  A  T  K  E  N  K  A  P  I  L  P  I  S  C  V  V  V  I  W  W  A  P  L  V  E  *  N  V  Q  I  C  R  K  Y  A  P    F5
     A  D  I  *  W  G  *  S  *  N  L  P  G  I  S  S  I  T  Y  K  D  P  C  V  A  C  R  H  L  G  L  Q  R  K  M  K  P  P  Y  *  P  F  A  A  S  W  L  L  G  G  L  L  C  Y  K  E  T  *  K  S  V  D  R  T  L  L  N     F4

       P  S  P  R  G  F  *  N  G  Q  P  Y  L  C  F  V  R  *  I  L  T  L  F  S  W  R  C  P  S  T  F  L  C  V  H  L  K  N  Y  G  T  Q  *  V  *  G  R  G  M  C  G  Q  *  S  L  H  S  R  R  N  C  H  L  G  A  V  *   F1
        Q  A  P  E  G  F  E  M  A  N  H  T  C  V  S  L  D  K  S  S  L  F  F  H  G  G  A  H  Q  H  S  F  V  F  T  S  K  T  M  E  P  N  K  C  E  A  E  E  C  V  V  S  D  H  Y  I  P  G  E  T  V  I  W  V  Q  C  D    F2
      K  P  Q  R  V  L  K  W  P  T  I  L  V  F  R  *  I  N  P  H  S  F  F  M  E  V  P  I  N  I  P  L  C  S  P  Q  K  L  W  N  P  I  S  V  R  P  R  N  V  W  S  V  I  I  T  F  Q  E  K  L  S  F  G  C  S  V     F3
1201 CCAAGCCCCAGAGGGTTTTGAAATGGCCAACCATACTTGTGTTTCGTTAGATAAATCCTCACTCTTTTTTCATGGAGGTGCCCATCAACATTCCTTTGTGTTCACCTCAAAAACTATGGAACCCAATAAGTGTGAGGCCGAGGAATGTGTGGTCAGTGATCATTACATTCCAGGAGAAACTGTCATTTGGGTGCAGTGTG 1400
     ----:----|----:----|----:----|----:----|----:----|----:----|----:----|----:----|----:----|----:----|----:----|----:----|----:----|----:----|----:----|----:----|----:----|----:----|----:----|----:----|
1201 GGTTCGGGGTCTCCCAAAACTTTACCGGTTGGTATGAACACAAAGCAATCTATTTAGGAGTGAGAAAAAAGTACCTCCACGGGTAGTTGTAAGGAAACACAAGTGGAGTTTTTGATACCTTGGGTTATTCACACTCCGGCTCCTTACACACCAGTCACTAGTAATGTAAGGTCCTCTTTGACAGTAAACCCACGTCACAC 1400
       G  L  G  L  P  N  Q  F  P  W  G  Y  K  H  K  T  L  Y  I  R  V  R  K  E  H  L  H  G  D  V  N  R  Q  T  *  R  L  F  *  P  V  W  Y  T  H  P  R  P  I  H  P  *  H  D  N  C  E  L  L  F  Q  *  K  P  A  T  H    F6
      E  L  G  W  L  T  K  F  H  G  V  M  S  T  N  R  *  I  F  G  *  E  K  K  M  S  T  G  M  L  M  G  K  H  E  G  *  F  S  H  F  G  I  L  T  L  G  L  F  T  H  D  T  I  M  V  N  W  S  F  S  D  N  P  H  L  T    F5
     W  A  G  S  P  K  S  I  A  L  W  V  Q  T  E  N  S  L  D  E  S  K  K  *  P  P  A  W  *  C  E  K  T  N  V  E  F  V  I  S  G  L  L  H  S  A  S  S  H  T  T  L  S  *  *  M  G  P  S  V  T  M  Q  T  C  H     F4

       *  V  F  M  *  K  V  V  S  L  L  L  H  W  V  D  R  T  R  Q  R  K  T  *  N  Y  R  V  L  L  *  *  M  Q  A  K  K  A  R  G  R  G  Q  R  G  K  R  G  N  Q  N  R  N  *  V  A  S  P  Y  L  Q  *  R  P  W  K   F1
        K  S  S  C  E  K  W  F  H  F  C  C  T  G  L  T  G  E  P  D  R  E  K  L  E  T  T  E  F  Y  C  N  K  C  K  P  K  R  Q  G  G  G  D  R  E  A  K  E  E  I  K  T  E  I  R  *  P  L  R  I  Y  N  E  G  H  G    F2
      I  S  L  H  V  K  S  G  F  T  F  V  A  L  G  *  Q  E  N  Q  T  E  K  N  L  K  L  Q  S  S  I  V  I  N  A  S  Q  K  G  K  G  A  G  T  E  R  Q  K  R  K  S  K  Q  K  L  G  S  L  S  V  F  T  M  K  A  M  E     F3
1401 ATAAGTCTTCATGTGAAAAGTGGTTTCACTTTTGTTGCACTGGGTTGACAGGAGAACCAGACAGAGAAAAACTTGAAACTACAGAGTTCTATTGTAATAAATGCAAGCCAAAAAGGCAAGGGGGCGGGGACAGAGAGGCAAAAGAGGAAATCAAAACAGAAATTAGGTAGCCTCTCCGTATTTACAATGAAGGCCATGGA 1600
     ----:----|----:----|----:----|----:----|----:----|----:----|----:----|----:----|----:----|----:----|----:----|----:----|----:----|----:----|----:----|----:----|----:----|----:----|----:----|----:----|
1401 TATTCAGAAGTACACTTTTCACCAAAGTGAAAACAACGTGACCCAACTGTCCTCTTGGTCTGTCTCTTTTTGAACTTTGATGTCTCAAGATAACATTATTTACGTTCGGTTTTTCCGTTCCCCCGCCCCTGTCTCTCCGTTTTCTCCTTTAGTTTTGTCTTTAATCCATCCGGAGAGGCATAAATGTTACTTCCGGTACCT 1600
       Y  T  K  M  H  F  T  T  E  S  K  N  C  Q  T  S  L  L  V  L  C  L  F  V  Q  F  *  L  T  R  N  Y  Y  I  C  A  L  F  A  L  P  R  P  C  L  P  L  L  P  F  *  F  L  F  *  T  A  E  G  Y  K  C  H  L  G  H    F6
      I  L  R  *  T  F  L  P  K  V  K  T  A  S  P  Q  C  S  F  W  V  S  F  F  K  F  S  C  L  E  I  T  I  F  A  L  W  F  L  P  L  A  P  V  S  L  C  F  L  F  D  F  C  F  N  P  L  R  E  T  N  V  I  F  A  M  S    F5
     S  L  D  E  H  S  F  H  N  *  K  Q  Q  V  P  N  V  P  S  G  S  L  S  F  S  S  V  V  S  N  *  Q  L  L  H  L  G  F  L  C  P  P  P  S  L  S  A  F  S  S  I  L  V  S  I  L  Y  G  R  R  I  *  L  S  P  W  P     F4

       E  K  I  Q  A  I  F  V  T  H  V  L  R  N  A  *  M  Q  *  I  S  R  A  K  V  T  S  K  I  I  R  *  R  I  *  F  C  S  H  A  T  R  T  *  K  K  H  M  Y  I  *  Y  Q  L  M  K  I  L  L  N  F  Y  A  A  F  S   F1
        K  K  K  F  R  Q  F  L  *  R  M  C  L  E  M  H  K  C  S  K  F  L  G  L  R  *  P  L  R  S  F  V  E  E  F  N  F  V  H  M  Q  Q  G  H  E  R  N  T  C  I  Y  N  I  S  *  *  K  F  Y  *  I  S  M  L  P  S  L    F2
      R  K  N  S  G  N  F  C  D  A  C  A  *  K  C  I  N  A  V  N  F  *  G  G  D  L  *  D  H  S  L  K  N  L  I  L  F  T  C  N  K  D  M  K  E  T  H  V  I  Y  I  I  S  A  D  E  N  F  I  K  F  L  C  C  L  L  S     F3
1601 AAGAAAAAATTCAGGCAATTTTTGTGACGCATGTGCTTAGAAATGCATAAATGCAGTAAATTTCTAGGGCTAAGGTGACCTCTAAGATCATTCGTTGAAGAATTTAATTTTGTTCACATGCAACAAGGACATGAAAGAAACACATGTATATATAATATCAGCTGATGAAAATTTTATTAAATTTCTATGCTGCCTTCTCT 1800
     ----:----|----:----|----:----|----:----|----:----|----:----|----:----|----:----|----:----|----:----|----:----|----:----|----:----|----:----|----:----|----:----|----:----|----:----|----:----|----:----|
1601 TTCTTTTTTAAGTCCGTTAAAAACACTGCGTACACGAATCTTTACGTATTTACGTCATTTAAAGATCCCGATTCCACTGGAGATTCTAGTAAGCAACTTCTTAAATTAAAACAAGTGTACGTTGTTCCTGTACTTTCTTTGTGTACATATATATTATAGTCGACTACTTTTAAAATAATTTAAAGATACGACGGAGAGA 1800
       F  S  F  I  *  A  I  K  T  V  C  T  S  L  F  A  Y  I  C  Y  I  L  A  L  T  V  E  L  I  M  R  Q  L  I  *  N  Q  E  C  A  V  L  V  H  F  F  C  M  Y  I  Y  Y  *  S  I  F  I  K  N  F  K  *  A  A  K  E    F6
      L  F  F  E  P  L  K  Q  S  A  H  A  *  F  H  M  F  A  T  F  K  *  P  *  P  S  R  *  S  *  E  N  F  F  K  I  K  N  V  H  L  L  S  M  F  S  V  C  T  Y  I  I  D  A  S  S  F  K  I  L  N  R  H  Q  R  R    F5
     F  F  F  N  L  C  N  K  H  R  M  H  K  S  I  C  L  H  L  L  N  R  P  S  L  H  G  R  L  D  N  T  S  S  N  L  K  T  *  M  C  P  C  S  L  F  V  H  I  Y  L  I  L  Q  H  F  N  *  *  I  E  I  S  G  E  R     F4

       L  L  F  D  Q  C  T  P  T  R  Q  N  C  R  *  W  C  T  Y  M  Y  I  R  T  C  K  K  Q  T  C  L  L  R  I  S  L  I  Q  S  I  S  S  K  F  L  S  S  *  S  L  Y  W  *  H  N  T  K  Q  T  K  F  A  L  D  F  S  N   F1
        S  Y  L  I  S  A  L  P  P  D  R  I  A  D  S  G  V  H  T  C  T  L  E  L  A  K  N  R  P  V  Y  *  G  S  H  L  S  S  P  Y  P  A  N  F  Y  H  H  K  V  Y  T  G  N  I  T  L  N  R  Q  N  L  H  W  I  L  A  T    F2
      L  I  *  S  V  H  S  H  Q  T  E  L  Q  I  V  V  Y  I  H  V  H  *  N  L  Q  K  T  D  L  S  I  K  D  L  T  Y  P  V  H  I  Q  Q  I  S  I  I  I  K  S  I  L  V  T  *  H  *  T  D  K  I  C  I  G  F  *  Q     F3
1801 CTCTTATTTGATCAGTGCACTCCCACCAGACAGAATTGCAGATAGTGGTGTACATACATGTACATTAGAACTTGCAAAAAACAGACCTGTCTATTAAGGATCTCACTTATCCAGTCCATATCCAGCAAATTTCTATCATCATAAAGTCTATACTGGTAACATAACACTAAACAGACAAAATTTGCATTGGATTTTAGCAA 2000
     ----:----|----:----|----:----|----:----|----:----|----:----|----:----|----:----|----:----|----:----|----:----|----:----|----:----|----:----|----:----|----:----|----:----|----:----|----:----|----:----|
1801 GAGAATAAACTAGTCACGTGAGGGTGGTCTGTCTTAACGTCTATCACCACATGTATGTACATGTAATCTTGAACGTTTTTTGTCTGGACAGATAATTCCTAGAGTGAATAGGTCAGGTATAGGTCGTTTAAAGATAGTAGTATTTCAGATATGACCATTGTATTGTGATTTGTCTGTTTTAAACGTAACCTAAAATCGTT 2000
       R  K  N  S  *  H  V  G  V  L  C  F  Q  L  Y  H  H  V  Y  M  Y  M  L  V  Q  L  F  C  V  Q  R  N  L  I  E  S  I  W  D  M  D  L  L  N  R  D  D  Y  L  R  Y  Q  Y  C  L  V  L  C  V  F  N  A  N  S  K  L  L    F6
      E  R  I  Q  D  T  C  E  W  W  V  S  N  C  I  T  T  Y  M  C  T  C  *  F  K  C  F  V  S  R  D  I  L  S  R  V  *  G  T  W  I  W  C  I  E  I  M  M  F  D  I  S  T  V  Y  C  *  V  S  L  I  Q  M  P  N  *  C    F5
     E  *  K  I  L  A  S  G  G  S  L  I  A  S  L  P  T  C  V  H  V  N  S  A  F  F  L  G  T  *  *  P  D  *  K  D  L  G  Y  G  A  F  K  *  *  *  L  T  *  V  P  L  M  V  S  F  L  C  F  K  C  Q  I  K  A     F4

       I  *  V  S  *  H  N  I  *  R  R  R  L  C  P  G  S  P  Q  S  E  Q  L  C  K  D  V  S  T  E  A  L  F  C  R  D  L  H  V  N  S  Q  *  K  *  I  A  S  L  R  S  N  V  L  S  C  L  E  R  H  W  R  I  *  R  *  S   F1
        S  K  F  H  D  I  I  Y  D  D  A  G  C  V  Q  A  H  H  R  V  N  S  S  A  R  M  F  Q  Q  R  P  F  S  A  G  I  Y  M  *  T  A  S  E  S  R  L  H  L  S  G  Q  M  C  C  L  A  L  R  D  I  G  E  Y  N  D  K    F2
      H  L  S  F  M  T  *  Y  M  T  T  P  V  V  S  R  L  T  T  E  *  T  A  L  Q  G  C  F  N  R  G  P  F  L  Q  G  F  T  C  E  Q  P  V  K  V  D  C  I  S  Q  V  K  C  A  V  L  P  *  E  T  L  A  N  I  T  I  K     F3
2001 CATCTAAGTTTCATGACATAATATATGACGACGCCGGTTGTGTCCAGGCTCACCACAGAGTGAACAGCTCTGCAAGGATGTTTCAACAGAGGCCCTTTTCTGCAGGGATTTACATGTGAACAGCCAGTGAAAGTAGATTGCATCTCTCAGGTCAAATGTGCTGTCTTGCCTTGAGAGACATTGGCGAATATAACGATAAA 2200
     ----:----|----:----|----:----|----:----|----:----|----:----|----:----|----:----|----:----|----:----|----:----|----:----|----:----|----:----|----:----|----:----|----:----|----:----|----:----|----:----|
2001 GTAGATTCAAAGTACTGTATTATATACTGCTGCGGCCAACACAGGTCCGAGTGGTGTCTCACTTGTCGAGACGTTCCTACAAAGTTGTCTCCGGGAAAAGACGTCCCTAAATGTACACTTGTCGGTCACTTTCATCTAACGTAGAGAGTCCAGTTTACACGACAGAACGGAACTCTCTGTAACCGCTTATATTGCTATTT 2200
       M  *  T  E  H  C  L  I  H  R  R  R  N  H  G  P  E  G  C  L  S  C  S  Q  L  S  T  E  V  S  A  R  K  Q  L  S  K  C  T  F  L  W  H  F  Y  I  A  D  R  L  D  F  T  S  D  Q  R  S  L  C  Q  R  I  Y  R  Y    F6
      C  R  L  K  M  V  Y  Y  I  V  V  G  T  T  D  L  S  V  V  S  H  V  A  R  C  P  H  K  L  L  P  G  K  R  C  P  N  V  H  S  C  G  T  F  T  S  Q  M  E  C  T  L  H  A  T  K  G  Q  S  V  N  A  F  I  V  I  F    F5
     V  D  L  N  *  S  M  I  Y  S  S  A  P  Q  T  W  A  *  W  L  T  F  L  E  A  L  I  N  *  C  L  G  K  E  A  P  I  *  M  H  V  A  L  S  L  L  N  C  R  E  P  *  I  H  Q  R  A  K  L  S  M  P  S  Y  L  S  L     F4

       L  L  P  L  S  P  P  S  L  D  R  T  P  V  L  P  T  P  P  G  L  S  M  I  S  P  T  C  S  M  I  R  *  V  *  F  D  H  G  V  *  Y  G  K  C  S  P  R  Q  K  P  N  C  M  A  V  S  L  Y  S  A  H  S  V  G  I   F1
        V  C  C  H  S  P  P  L  R  *  I  E  H  Q  Y  F  Q  H  H  Q  D  S  V  *  S  R  Q  R  A  Q  *  Y  D  E  Y  S  L  T  M  V  Y  S  M  G  N  V  P  L  D  K  N  K  Q  I  A  W  L  F  P  Y  I  Q  H  I  Q  W  A  L    F2
      F  V  A  T  L  P  P  F  A  R  *  N  T  S  T  S  N  T  T  R  T  Q  Y  D  L  A  N  V  L  N  D  T  M  S  I  V  *  P  W  C  I  V  W  E  M  F  P  *  T  K  T  K  L  H  G  C  F  L  I  F  S  T  F  S  G  H  C     F3
2201 GTTTGTTGCCACTCTCCCCCCCTTCGCTAGATAGAACACCAGTACTTCCAACACCACCAGGACTCAGTATGATCTCGCCAACGTGCTCAATGATACGATGAGTATAGTTTGACCATGGTGTATAGTATGGGAAATGTTCCCCTAGACAAAAACCAAATTGCATGGCTGTTTCCTTATATTCAGCACATTCAGTGGGCATT 2400
     ----:----|----:----|----:----|----:----|----:----|----:----|----:----|----:----|----:----|----:----|----:----|----:----|----:----|----:----|----:----|----:----|----:----|----:----|----:----|----:----|
2201 CAAACAACGGTGAGAGGGGGGGAAGCGATCTATCTTGTGGTCATGAAGGTTGTGGTGGTCCTGAGTCATACTAGAGCGGTTGCACGAGTTACTATGCTACTCATATCAAACTGGTACCACATATCATACCCTTTACAAGGGGATCTGTTTTTGGTTTAACGTACCGACAAAGGAATATAAGTCGTGTAAGTCACCCGTAA 2400
       L  K  N  G  S  E  G  G  E  S  S  L  V  G  T  S  G  V  G  G  P  S  L  I  I  E  G  V  H  E  I  I  R  H  T  Y  N  S  W  P  T  Y  Y  P  F  H  E  G  L  C  F  G  F  Q  M  A  T  E  K  Y  E  A  C  E  T  P  M    F6
      N  T  A  V  R  G  G  K  A  L  Y  F  V  L  V  E  L  V  V  L  V  *  Y  S  R  A  L  T  S  L  S  V  I  L  I  T  Q  G  H  I  T  H  I  S  I  N  G  *  F  V  L  N  C  P  Q  K  R  I  N  L  V  N  L  P  C    F5
     T  Q  Q  W  E  G  G  R  R  *  I  S  C  W  Y  K  W  C  W  W  S  E  T  H  D  R  W  R  A  *  H  Y  S  S  Y  L  K  V  M  T  Y  L  I  P  F  T  G  R  S  L  F  W  I  A  H  S  N  G  *  I  *  C  M  *  H  A  N     F4
```

```
     V  S  R  G  S  L  Q  E  *  T  D  C  N  L  E  R  R  V  N  K  H  F  T  C  W  A  L  S  S  S  L  M  S  P  V  T  T  S  R  L  F  T  S  K  S  A  L  A  *  F  P  G  I  I  S  E  D  *  I  P  R  R  S  L  K  C  S   F1
      F  P  E  E  A  C  R  S  R  L  T  A  T  W  K  G  E  *  T  N  T  S  H  V  G  L  F  L  H  L  *  *  V  L  *  Q  H  Q  G  Y  L  H  Q  K  V  P  W  H  S  S  Q  V  *  S  V  K  T  E  S  Q  D  E  V  S  S  V  H   F2
       F  Q  R  K  L  A  G  V  D  *  L  Q  L  G  K  E  S  E  Q  T  L  H  M  L  G  S  F  F  I  F  D  E  S  C  D  N  I  K  V  I  Y  I  K  K  C  P  G  I  V  P  R  Y  N  Q  *  R  L  N  P  K  T  K  S  Q  V  F    F3
2401 GTTTCCAGAGGAAGCTTGCAGGAGTAGACTGACTGCAACTTGGAAAGGAGAGTGAACAAACACTTCACATGTTGGGCTCTTTCTTCATCTTTGATGAGTCCTGTGACAACATCAAGGTTATTTACATCAAAAAGTGCCCTGGCATAGTTCCCAGGTATAATCAGTGAAGACTGAATCCCAAGACGAAGTCTCAAGTGTTC 2600
     ----:----|----:----|----:----|----:----|----:----|----:----|----:----|----:----|----:----|----:----|----:----|----:----|----:----|----:----|----:----|----:----|----:----|----:----|----:----|----:----|
2401 CAAAGGTCTCCTTCGAACGTCCTCATCTGACTGACGTTGAACCTTTCCTCTCACTTGTTTGTGAAGTGTACAACCCGAGAAAGAAGTAGAAACTACTCAGGACACTGTTGTAGTTCCAATAAATGTAGTTTTTCACGGGACCGTATCAAGGGTCCATATTAGTCACTTCTGACTTAGGGTTCTGCTTCAGAGTTCACAAG 2600
      T  E  L  P  L  K  C  S  Y  V  S  Q  L  K  S  L  L  T  F  L  C  K  V  H  Q  A  R  E  E  D  K  I  L  G  T  V  V  D  L  N  N  V  D  F  L  A  R  A  Y  N  G  P  I  I  L  S  S  Q  I  G  L  R  L  R  L  H  E   F6
     Q  K  W  L  F  S  A  P  T  S  Q  S  C  S  P  F  S  L  S  C  V  S  *  M  N  P  E  K  K  M  K  S  S  D  Q  S  L  M  L  T  I  *  M  L  F  H  G  P  M  T  G  L  Y  L  *  H  L  S  F  G  L  V  F  D  *  T  N   F5
      N  G  S  S  A  Q  L  L  L  S  V  A  V  Q  F  P  S  H  V  F  V  E  C  T  P  S  K  R  *  R  Q  H  T  R  H  C  C  *  P  *  K  C  *  F  T  G  Q  C  L  E  W  T  Y  D  T  F  V  S  D  W  S  S  T  E  L  T    F4

      S  S  L  L  S  A  S  *  S  L  T  L  F  C  S  F  S  V  H  S  *  T  E  A  V  S  R  T  S  I  F  L  K  I  R  P  M  L  M  F  A  *  V  A  S  N  D  R  S  V  A  S  V  E  I  S  G  R  L  F  T  P  R  H  S  S  A   F1
       P  A  F  F  L  H  L  E  V  *  P  C  S  V  H  S  P  S  I  H  R  L  R  Q  S  P  V  P  V  S  S  *  K  F  A  P  C  *  C  L  H  E  L  H  L  M  I  A  L  W  H  Q  W  K  L  V  A  G  F  S  P  Q  G  I  L  Q    F2
     I  Q  P  S  F  C  I  L  K  F  D  L  V  L  F  I  L  R  P  F  I  D  *  G  S  L  P  Y  Q  Y  L  L  E  N  S  P  H  V  D  V  C  M  S  C  I  *  *  S  L  C  G  I  S  G  N  *  W  Q  A  F  H  P  K  A  F  F  S   F3
2601 ATCCAGCCTTCTTTCTGCATCTTGAAGTTTGACCTTGTTCTGTTCATTCTCCGTCCATTCATAGACTGAGGCAGTCTCCCGTACCAGTATCTTCTTGAAAATTCGCCCCATGTTGATGTTTGCATGAGTTGCATCTAATGATCGCTCTGTGGCATCAGTGGAAATTAGTGGCAGGCTTTTCACCCCAAGGCATTCTTCAG 2800
     ----:----|----:----|----:----|----:----|----:----|----:----|----:----|----:----|----:----|----:----|----:----|----:----|----:----|----:----|----:----|----:----|----:----|----:----|----:----|----:----|
2601 TAGGTCGGAAGAAAGACGTAGAACTTCAAACTGGAACAAGACAAGTAAGAGGCAGGTAAGTATCTGACTCCGTCAGAGGGCATGGTCATAGAAGAACTTTTAAGCGGGGTACAACTACAAACGTACTCAACGTAGATTACTAGCGAGACACCGTAGTCACCTTTAATCACCGTCCGAAAAGTGGGGTTCCGTAAGAAGTC 2800
       D  L  R  R  E  A  D  Q  L  K  V  K  N  Q  E  N  E  T  W  E  Y  V  S  A  T  E  R  V  L  I  K  K  F  I  R  G  M  N  I  N  A  H  T  A  D  L  S  R  E  T  A  D  T  S  I  L  P  L  S  K  V  G  L  C  E  E    F6
      M  W  G  E  K  Q  M  K  F  N  S  R  T  R  N  M  R  R  G  N  M  S  Q  P  L  R  G  Y  W  Y  R  R  S  F  E  G  W  T  S  T  Q  M  L  Q  M  *  H  D  S  Q  P  M  L  P  F  *  H  C  A  K  *  G  L  A  N  K  L   F5
     *  G  A  K  K  R  C  R  S  T  Q  G  Q  E  T  *  E  G  D  M  *  L  S  L  C  D  G  T  G  T  D  E  Q  F  N  A  G  H  Q  H  K  C  S  N  C  R  I  I  A  R  H  C  *  H  F  N  T  A  P  K  E  G  W  P  M  R  *    F4

      R  S  F  S  F  L  L  R  G  F  S  R  R  Y  Q  Y  W  L  T  V  S  E  F  V  L  H  L  L  K  E  P  R  S  H  W  Q  E  E  V  V  A  *  Q  S  V  Q  M  N  I  E  P  L  P  W  S  P  D  V  L  R  S  N  F  S  S  I    F1
     Q  D  H  F  H  F  Y  L  G  D  F  Q  D  D  T  S  T  G  *  L  S  P  N  L  F  Y  T  S  *  R  N  Q  G  H  I  G  R  K  K  L  L  H  D  K  A  Y  K  *  T  L  S  H  Y  P  G  A  L  M  Y  C  V  Q  I  F  H  Q  S   F2
      K  I  I  F  I  S  T  *  G  I  F  K  T  I  P  V  L  V  N  C  L  R  I  C  S  T  P  P  E  G  T  K  V  T  L  A  G  R  S  C  C  M  T  K  R  T  N  E  H  *  A  T  T  L  E  P  *  C  I  A  F  K  F  F  I  N  H   F3
2801 CAAGATCATTTTCATTTCTACTTAGGGGATTTTCAAGACGATACCAGTACTGGTTAACTGTCTCCGAATTTGTTCTACACCTCCTGAAGGAACCAAGGTCACATTGGCAGGAAGAAGTTGTTGCATGACAAAGCGTACAAATGAACATTGAGCCACTACCCTGGAGCCCTGATGTATGCGTTCAAATTTTTCATCAATC 3000
     ----:----|----:----|----:----|----:----|----:----|----:----|----:----|----:----|----:----|----:----|----:----|----:----|----:----|----:----|----:----|----:----|----:----|----:----|----:----|----:----|
2801 GTTCTAGTAAAAGTAAAGATGAATCCCCTAAAAGTTCTGCTATGGTCATGACCAATTGACAGAGGCTTAAACAAGATGTGGAGGACTTCCTTGGTTCCAGTGTAACCGTCCTTCTTCAACAACGTACTGTTTCGCATGTTTACTTGTAACTCGGTGATGGGACCTCGGGACTACATACGCAAGTTTAAAAAGTAGTTAG 3000
      A  L  D  N  E  N  R  S  L  P  N  E  L  R  Y  W  Y  Q  N  V  T  E  S  N  T  R  C  R  R  F  S  G  L  D  C  Q  C  S  S  T  T  A  H  C  L  T  C  I  F  M  S  G  S  G  Q  L  G  S  T  N  R  E  F  K  E  D  I    F6
       L  I  M  K  M  E  V  *  P  I  K  L  V  I  G  T  S  T  L  Q  R  R  I  Q  E  V  G  G  S  P  V  L  T  V  N  A  P  L  L  Q  Q  M  V  F  R  V  F  S  C  Q  A  V  V  R  S  G  Q  H  I  A  N  L  N  K  M  L    F5
      C  S  *  K  *  K  *  K  P  S  K  *  S  S  V  L  V  P  *  S  D  G  F  K  N  *  V  E  Q  L  F  W  P  *  M  P  L  F  F  N  N  C  S  L  A  Y  L  H  V  N  L  W  *  G  P  A  R  I  Y  Q  T  *  I  K  *  *  D   F4

     M  V  Y  Q  K  V  S  S  C  S  L  T  T  D  P  E  L  A  S  R  I  C  R  K  L  F  F  M  R  S  R  D  L  E  I  D  F  S  V  T  H  K  L  G  R  S  L  S  S  Q  F  A  T  S  T  G  R  V  H  S  T  E  L  G  L  L  S   F1
      W  Y  T  K  R  S  A  H  A  P  L  Q  L  T  Q  N  W  H  Q  E  S  A  E  S  C  F  S  *  D  L  V  T  *  K  L  T  S  V  L  H  T  N  L  A  D  H  F  R  H  S  L  P  Q  V  Q  G  V  C  I  R  L  N  W  D  Y  S  P   F2
       G  I  P  K  G  Q  L  M  L  P  Y  N  *  P  R  I  G  I  K  N  L  Q  K  V  V  F  H  E  I  S  *  P  R  N  *  L  Q  C  Y  T  Q  T  W  P  I  T  F  V  T  V  C  H  K  Y  R  A  C  A  F  D  *  I  G  I  T  L    F3
3001 ATGGTATACCAAAAGGTCAGCTCATGCTCCCTTACAACTGACCCAGAATTGGCATCAAGAATCTGCAGAAAGTTGTTTTTCATGAGATCTCGTGACCTAGAAATTGACTTCAGTGTTACACACAAACTTGGCCGATCACTTTCGTCACAGTTTGCCACAAGTACAGGGCGTGTGCATTCGACTGAATTGGGATTACTCTC 3200
     ----:----|----:----|----:----|----:----|----:----|----:----|----:----|----:----|----:----|----:----|----:----|----:----|----:----|----:----|----:----|----:----|----:----|----:----|----:----|----:----|
3001 TACCATATGGTTTTCCAGTCGAGTACGAGGGAATGTTGACTGGGTCTTAACCGTAGTTCTTAGACGTCTTTCAACAAAAAGTACTCTAGAGCACTGGATCTTTAACTGAAGTCACAATGTGTGTTTGAACCGGCTAGTGAAAGCAGTGTCAAACGGTGTTCATGTCCCGCACACGTAAGCTGACTTAACCCTAATGAGAG 3200
      M  T  Y  W  F  T  L  E  H  E  R  V  V  S  G  S  N  A  D  L  I  Q  L  F  N  N  K  M  L  D  R  S  R  S  I  S  K  L  T  V  C  L  S  P  R  D  S  E  D  C  N  A  V  L  V  P  R  T  C  E  V  S  N  P  N  S  E   F6
     *  P  I  G  F  P  *  S  M  S  G  *  L  Q  G  L  I  P  M  L  F  R  C  F  T  T  K  *  S  I  E  H  G  L  F  Q  S  *  H  *  V  C  V  Q  G  I  V  K  T  V  T  Q  W  L  Y  L  A  H  A  N  S  Q  I  P  I  V  R   F5
      H  Y  V  L  L  D  A  *  A  G  K  C  S  V  W  F  Q  C  *  S  D  A  S  L  Q  K  E  H  S  R  T  V  *  F  N  V  E  T  N  C  V  F  K  A  S  *  K  R  *  L  K  G  C  T  C  P  T  H  M  R  S  F  Q  S  *  E    F4

      R  N  N  T  W  S  T  P  F  T  L  A  V  H  N  R  T  V  K  E  Y  L  N  T  L  S  G  R  F  L  S  T  F  R  A  F  N  L  I  S  P  K  P  S  P  P  S  L  M  K  V  F  T  F  V  S  A  G  N  E  S  S  C  S  F  W  Q   F1
       G  I  T  H  G  Q  P  H  L  H  W  L  Y  I  T  E  Q  *  K  S  I  *  T  L  C  R  G  D  F  Y  P  P  F  E  L  L  T  *  Y  H  L  S  R  L  H  H  P  *  *  K  F  S  P  L  C  L  L  V  M  K  V  P  V  V  S  G    F2
     P  E  *  H  M  V  N  P  I  Y  I  G  C  T  *  Q  N  S  E  R  V  S  E  H  F  V  G  E  I  F  I  H  L  S  S  F  *  L  D  I  T  *  A  V  S  T  I  L  D  E  S  F  H  L  C  V  C  W  *  *  K  F  L  *  F  L  A   F3
3201 CCGGAATAACACATGGTCAACCCCATTTACATTGGCTGTACATAACAGAACAGTGAAAGAGTATCTGAACACTTTGTCGGGGAGATTTTTATCCACCTTTCGAGCTTTTAACTTGATATCACCTAAGCCGTCTCCACCATCCTTGATGAAAGTTTTCACCTTTGTGTCTGCTGGTAATGAAAGTTCCTGTAGTTTCTGGC 3400
     ----:----|----:----|----:----|----:----|----:----|----:----|----:----|----:----|----:----|----:----|----:----|----:----|----:----|----:----|----:----|----:----|----:----|----:----|----:----|----:----|
3201 GGCCTTATTGTGTACCAGTTGGGGTAAATGTAACCGACATGTATTGTCTTGTCACTTTCTCATAGACTTGTGAAACAGCCCCTCTAAAAATAGGTGGAAAGCTCGAAAATTGAACTATAGTGGATTCGGCAGAGGTGGTAGGAACTACTTTCAAAAGTGGAAACACAGACGACCATTACTTTCAAGGACATCAAAGACCG 3400
       R  F  L  V  H  D  V  G  N  V  N  A  T  C  L  L  V  T  F  S  Y  R  F  V  K  D  P  L  N  K  D  V  K  R  A  K  L  K  I  D  G  L  G  D  G  G  D  K  I  F  T  K  V  K  T  D  A  P  L  S  L  E  Q  L  K  Q    F6
      G  S  Y  C  M  T  L  G  M  *  M  P  Q  V  Y  C  F  L  S  L  T  D  S  C  K  T  P  S  I  K  I  W  R  E  L  K  *  S  S  I  V  *  A  T  E  V  M  R  S  S  L  K  *  R  Q  T  Q  Q  Y  H  F  N  R  Y  N  R  A   F5
     G  P  I  V  C  P  *  G  W  K  C  Q  S  Y  M  V  S  C  H  F  L  I  Q  V  S  Q  R  P  S  K  *  G  G  K  S  S  K  V  Q  Y  *  R  L  R  R  W  W  G  Q  H  F  N  E  G  K  H  R  S  T  I  F  T  G  T  T  E  P   F4

      I  S  S  S  K  S  I  R  D  F  A  T  A  D  T  *  S  N  S  N  P  Q  T  L  G  R  G  K  S  A  G  K  *  E  Y  S  P  M  M  S  V  G  S  H  S  A  F  V  F  R  D  C  S  A  R  T  L  S  D  S  E  S  T  N  L  K    F1
     K  Y  H  P  P  N  P  S  E  I  L  R  L  L  T  H  N  Q  T  Q  T  H  K  H  *  G  E  G  N  L  Q  E  N  E  N  I  H  P  *  C  Q  L  V  H  I  Q  P  L  F  S  E  T  V  L  P  E  L  C  L  I  L  S  Q  L  I  *  R   F2
      N  I  I  L  Q  I  H  Q  R  F  C  D  C  *  H  I  I  K  L  K  P  T  N  I  R  E  R  E  I  C  R  K  M  R  I  F  T  H  D  V  S  W  F  T  F  S  L  C  F  Q  R  L  F  C  Q  N  F  V  *  F  *  V  N  *  S  E  G   F3
3401 AAATATCATCCTCCAAATCCATCAGAGATTTTGCGACTGCTGACACATAATCAAACTCAAACCCACAAACATTAGGGAGAGGGAAATCTGCAGGAAAATGAGAATATTCACCCATGATGTCAGTTGGTTCACATTCAGCCTTTGTTTTCAGAGACTGTTCTGCCAGAACTTTGTCTGATTCTGAGTCAACTAATCTGAAG 3600
     ----:----|----:----|----:----|----:----|----:----|----:----|----:----|----:----|----:----|----:----|----:----|----:----|----:----|----:----|----:----|----:----|----:----|----:----|----:----|----:----|
3401 TTTATAGTAGGAGGTTTAGGTAGTCTCTAAAACGCTGACGACTGTGTATTAGTTTGAGTTTGGGTGTTTGTAATCCCTCTCCCTTTAGACGTCCTTTTACTCTTATAAGTGGGTACTACAGTCAACCAAGTGTAAGTCGGAAACAAAAGTCTCTGACAAGACGGTCTTGAAACAGACTAAGACTCAGTTGATTAGACTTC 3600
      C  I  D  D  E  L  D  M  L  S  K  A  V  A  S  V  Y  D  F  E  F  G  C  V  N  P  L  P  F  D  A  P  F  H  S  Y  E  G  M  I  D  T  P  E  C  E  A  K  T  K  L  S  Q  E  A  L  V  K  D  S  E  S  D  V  L  R  F   F6
       F  I  M  R  W  I  W  *  L  N  Q  S  Q  Q  C  M  I  L  S  L  G  V  F  M  L  S  L  S  I  Q  L  F  I  L  I  N  V  W  S  T  L  Q  N  V  N  L  R  Q  K  *  L  S  N  Q  W  F  K  T  Q  N  Q  T  L  *  D  S    F5
      L  Y  *  G  G  F  G  D  S  I  K  R  S  S  V  C  L  *  V  *  V  W  L  C  *  P  S  P  F  R  C  S  F  S  F  I  *  G  H  H  *  N  T  *  M  *  G  K  N  E  S  V  T  R  G  S  S  Q  R  I  R  L  *  S  I  Q  L   F4

     A  Q  K  P  G  R  Y  L  Y  S  I  A  V  T  C  F  G  G  W  R  R  G  K  P  I  *  A  F  *  T  L  *  S  F  L  Y  F  V  F  E  C  P  N  V  T  L  I  A  K  Q  S  S  G  V  I  C  L  N  S  S  V  S  F  Q  I  A  E   F1
      H  R  N  Q  A  D  T  C  I  Q  S  L  S  P  A  L  E  A  G  E  G  G  N  P  F  E  L  S  E  L  Y  R  A  S  C  T  S  F  L  N  V  P  M  S  L  S  *  L  S  S  P  L  G  S  F  V  *  I  H  Q  F  L  S  K  *  Q  K   F2
       T  E  T  R  Q  I  L  V  F  N  R  C  H  L  L  W  R  L  E  K  G  E  T  H  L  S  F  L  N  F  I  E  L  L  V  L  R  F  *  M  S  Q  C  H  S  H  S  *  A  V  L  W  G  H  L  F  K  F  I  S  F  F  P  N  S  R    F3
3601 GCACAGAAACCAGGCAGATACTTGTATTCAATCGCTGTCACCTGCTTTGGAGGCTGGAGAAGGGGGAAACCCATTTGAGCTTTCTGAACTTTATAGAGCTTCTTGTACTTCGTTTTTGAATGTCCCAATGTCACTCTCATAGCTAAGCAGTCCTCTGGGGTCATTTGTTTAAATTCATCAGTTTCTTTCCAAATAGCAGA 3800
     ----:----|----:----|----:----|----:----|----:----|----:----|----:----|----:----|----:----|----:----|----:----|----:----|----:----|----:----|----:----|----:----|----:----|----:----|----:----|----:----|
3601 CGTGTCTTTGGTCCGTCTATGAACATAAGTTAGCGACAGTGGACGAAACCTCCGACCTCTTCCCCCTTTGGGTAAACTCGAAAGACTTGAAATATCTCGAAGAACATGAAGCAAAAACTTACAGGGTTACAGTGAGAGTATCGATTCGTCAGGAGACCCCAGTAAACAAATTTAAGTAGTCAAAGAAAGGTTTATCGTCT 3800
      A  C  F  G  P  L  Y  K  Y  E  I  A  T  V  Q  K  P  P  Q  L  L  P  F  G  M  Q  A  K  Q  V  K  Y  L  K  K  Y  K  T  K  S  H  G  L  T  V  R  M  A  L  C  D  E  P  T  M  Q  K  F  E  D  T  E  K  W  I  A  S   F6
     P  V  S  V  L  C  I  S  T  N  L  R  Q  *  R  S  Q  L  S  S  P  F  S  V  W  K  L  K  R  F  K  I  S  S  R  T  S  R  K  Q  I  D  W  H  *  E  *  L  *  A  T  R  Q  P  *  K  N  L  N  M  L  K  K  G  F  L   F5
      C  L  F  W  A  S  V  Q  I  *  D  S  D  G  A  K  S  A  P  S  P  P  F  G  N  S  S  E  S  S  *  L  A  E  Q  V  E  N  K  F  T  G  I  D  S  E  Y  S  L  L  G  R  P  D  N  T  *  I  *  *  N  R  E  L  Y  C    F4

      T  L  F  A  I  *  N  F  *  L  S  L  S  F  G  L  S  K  K  N  R  T  S  T  S  S  S  I  D  S  L  Q  Y  V  S  I  E  S  I  R  G  F  K  R  T  R  F  T  Y  W  R  L  A  S  F  K  L  N  F  F  L  L  R  S  V  F  G   F1
       L  C  L  P  S  E  I  S  D  C  L  *  V  S  D  *  A  K  R  I  E  H  P  H  R  L  P  L  I  H  Y  S  M  F  Q  *  S  R  S  G  V  L  N  V  R  V  L  H  I  G  D  *  P  L  S  N  S  T  S  S  F  C  D  Q  F  L   F2
     N  F  V  C  H  L  K  F  L  I  V  S  E  F  R  T  E  Q  K  E  *  N  I  H  I  V  F  H  *  F  T  T  V  C  F  N  R  V  D  Q  G  F  *  T  Y  A  F  Y  I  L  A  I  S  L  F  Q  T  Q  L  L  P  S  A  I  S  F  W   F3
3801 AACTTTGTTTGCCATCTGAAATTTCTGATTGTCTCTGAGTTTCGGACTGAGCAAAAAGAATAGAACATCCACATCGTCTTCCATTGATTCACTACAGTATGTTTCAATAGAGTCGATCAGGGGTTTAAAACGTACGCGTTTTACATATTGGCGATTAGCCTCTTTCAAACTCAACTTCTTCCTTCTGCGATCAGTTTTTG 4000
     ----:----|----:----|----:----|----:----|----:----|----:----|----:----|----:----|----:----|----:----|----:----|----:----|----:----|----:----|----:----|----:----|----:----|----:----|----:----|----:----|
3801 TTGAAACAAACGGTAGACTTTAAAGACTAACAGAGACTCAAAGCCTGACTCGTTTTTCTTATCTTGTAGGTGTAGCAGAAGGTAACTAAGTGATGTCATACAAAGTTATCTCAGCTAGTCCCCAAAATTTGCATGCGCAAAATGTATAACCGCTAATCGGAGAAAGTTTGAGTTGAAGAAGGAAGACGCTAGTCAAAAAC 4000
      V  K  N  A  M  Q  F  K  Q  N  D  R  L  K  P  S  L  L  F  F  L  V  D  V  D  E  M  S  E  S  C  Y  T  E  I  S  D  I  L  P  K  L  R  V  R  K  V  Y  Q  R  N  A  E  K  L  S  L  K  K  R  R  R  D  T  K    F6
      F  K  T  Q  W  R  F  N  R  I  T  E  S  N  R  V  S  C  F  S  Y  F  M  W  M  T  K  W  Q  N  V  V  T  H  K  L  L  T  S  *  P  N  *  V  Y  A  N  *  M  N  A  I  L  R  K  *  V  *  S  R  G  E  A  I  L  K  Q   F5
     F  S  Q  K  G  D  S  I  E  S  Q  R  Q  T  E  S  Q  A  F  L  I  S  C  G  C  R  R  G  N  I  *  *  L  I  N  *  Y  L  R  D  P  T  K  F  T  R  T  K  C  I  P  S  *  G  R  E  F  E  V  E  E  K  Q  S  *  N  K    F4

      M  L  T  A  G  I  E  A  S  L  A  L  *  L  *  S  R  F  T  V  I  G  F  G  G  E  G  G  K  S  I  P  K  F  P  A  T  F  R  Q  K  Q  S  S  P  *  C  E  L  N  A  Y  V  S  R  F  F  D  S  N  F  S  F  K  R  S    F1
     A  C  *  L  Q  E  *  K  P  H  *  H  C  N  Y  D  Q  G  S  L  *  L  G  L  E  V  K  A  A  N  L  Y  P  S  F  L  Q  P  L  D  K  N  S  H  H  L  D  V  N  *  M  H  M  F  P  D  F  L  T  P  I  L  A  S  S  D  Q   F2
      H  V  N  C  R  N  R  S  L  T  S  I  V  T  M  I  K  V  H  C  N  W  V  W  R  *  R  R  Q  I  Y  T  Q  V  S  C  N  L  *  T  K  T  V  I  T  L  M  *  T  K  C  I  C  F  Q  I  F  *  L  Q  F  *  L  Q  A  I  K   F3
4001 GCATGTTAACTGCAGGAATAGAAGCCTCACTAGCATTGTAACTATGATCAAGGTTCACTGTAATTGGGTTTGGAGGTGAAGGCGGCAAATCTATACCCAAGTTTCCTGCAACCTTTAGACAAAAACAGTCATCACCTTGATGTGAACTAAATGCATATGTTTCCAGATTTTTTGACTCCAATTTTAGCTTCAAGCGATCA 4200
     ----:----|----:----|----:----|----:----|----:----|----:----|----:----|----:----|----:----|----:----|----:----|----:----|----:----|----:----|----:----|----:----|----:----|----:----|----:----|----:----|
4001 CGTACAATTGACGTCCTTATCTTCGGAGTGATCGTAACATTGATACTAGTTCCAAGTGACATTAACCCAAACCTCCACTTCCGCCGTTTAGATATGGGTTCAAAGGACGTTGGAAATCTGTTTTTGTCAGTAGTGGAACTACACTTGATTTACGTATACAAAGGTCTAAAAAACTGAGGTTAAAATCGAAGTTCGCTAGT 4200
      P  M  N  V  A  P  I  S  A  E  S  A  N  Y  S  H  D  L  N  V  T  I  P  N  P  P  S  P  P  L  D  I  G  L  N  G  A  V  K  L  C  F  C  D  D  G  Q  H  S  S  F  A  Y  T  E  L  N  K  S  E  L  K  L  K  L  R  D   F6
       C  T  L  Q  L  F  L  L  R  V  L  M  T  V  I  I  L  T  *  Q  L  Q  T  Q  L  H  L  R  C  I  *  V  W  T  E  Q  L  R  *  V  F  V  T  M  V  K  I  H  V  L  H  M  H  K  W  I  K  Q  S  W  N  *  S  *  A  I    F5
      A  H  *  S  C  S  Y  F  G  *  *  C  Q  L  *  S  *  P  E  S  Y  N  P  K  S  T  F  A  A  F  R  Y  G  L  K  R  C  G  K  S  L  F  L  *  *  R  S  T  F  *  I  C  I  N  G  S  K  K  V  G  I  K  A  E  L  S  *   F4

     S  R  T  R  H  L  L  H  N  V  L  G  K  T  D  E  L  S  F  S  T  S  V  P  K  *  S  L  K  T  S  T  Y  V  E  T  L  R  N  V  F  C  R  A  A  A  P  L  L  C  R  V  S  P  H  T  R  H  T  C  P  K  T  N  L  C  R   F1
      V  G  P  D  I  C  Y  T  T  F  W  E  R  L  M  N  F  R  F  L  R  Q  C  Q  S  E  A  *  K  H  P  R  M  L  K  H  C  G  T  S  F  V  E  L  L  L  H  Y  C  V  E  F  R  H  T  H  G  I  P  V  P  K  R  T  C  V  G   F2
       S  D  P  T  F  V  T  Q  R  F  G  K  D  *  *  T  F  V  F  Y  V  S  A  K  V  K  L  E  N  I  H  V  C  *  N  T  A  E  R  L  L  S  S  C  C  S  T  I  V  *  S  F  A  T  H  T  A  Y  L  S  Q  N  E  L  V    F3
4201 AGTCGGACCCGACATTTGTTACACAACGTTTTGGGAAAGACTGATGAACTTTCGTTTTCTACGTCAGTGCCAAAGTGAAGCTTGAAAACATCCACGTATGTTGAAACACTGCGGAACGTCTTTTGTCGAGCTGCTGCTCCACTATTGTGTAGAGTTTCGCCACACACACGGCATACCTGTCCCAAAACGAACTTGTGTAG 4400
     ----:----|----:----|----:----|----:----|----:----|----:----|----:----|----:----|----:----|----:----|----:----|----:----|----:----|----:----|----:----|----:----|----:----|----:----|----:----|----:----|
4201 TCAGCCTGGGCTGTAAACAATGTGTTGCAAAACCCTTTCTGACTACTTGAAAGCAAAAGATGCAGTCACGGTTTCACTTCGAACTTTTGTAGGTGCATACAACTTTGTGACGCCTTGCAGAAAACAGCTCGACGACGAGGTGATAACACATCTCAAAGCGGTGTGTGTGCCGTATGGACAGGGTTTTGCTTGAACACATC 4400
      L  R  V  R  C  K  N  C  L  T  K  P  F  V  S  S  E  N  E  V  D  T  G  F  H  L  K  F  V  D  V  Y  T  S  V  S  R  F  T  K  Q  R  A  A  A  G  S  N  H  L  T  E  G  C  V  R  C  V  Q  G  L  V  F  K  H  L   F6
     L  D  S  G  V  N  T  V  C  R  K  P  F  S  Q  H  V  K  T  K  *  T  L  A  L  T  F  S  S  F  M  W  T  H  Q  F  V  A  S  R  R  K  D  L  Q  Q  E  V  I  T  Y  L  K  A  V  C  V  A  Y  R  D  W  F  S  S  T  Y   F5
      T  P  G  S  M  Q  *  V  V  N  Q  S  L  S  I  F  K  R  K  R  R  *  H  W  L  S  A  Q  F  C  G  R  I  N  F  C  Q  P  V  D  K  T  S  S  S  S  W  *  Q  T  S  N  R  W  V  C  P  M  G  T  G  F  R  V  Q  T    F4

      S  S  M  S  V  L  K  *  K  K  T  I  T  I  *   F1
       L  P  C  L  C  *  N  E  K  K  P  L  Q  Y  X   F2
     V  F  H  V  C  A  E  M  K  K  N  H  Y  N  M  X   F3
4401 GTCTTCCATGTCTGTGCTGAAATGAAAAAAAACCATTACAATATGA 4446
     ----:----|----:----|----:----|----:----|----:-
4401 CAGAAGGTACAGACACGACTTTACTTTTTTTGGTAATGTTATACT 4446
      D  E  M  D  T  S  F  H  F  F  V  M  V  I  H   F6
      T  K  W  T  Q  A  S  I  F  F  F  W  *  L  I   F5
     P  R  G  H  R  H  Q  F  S  F  F  G  N  C  Y  S   F4
```

Notospermus geniculatus (Nge)

WGS: NMRB01002705.1

27001-32396
Notospermus geniculatus strain Ushimado scaffold2705, whole genome shotgun sequence

```
       S  H  K  D  G  P  V  P  S  G  C  P  *  S  C  L  P  D  M  W  *  *  T  P  S  *  S  S  K  A  T  E  E  V  L  *  G  E  R  G  S  *  N  H  *  S  S  L  R  Y  L  C  Q  G  *  R  *  T  P  V  S  R  L  C  L  Q  Q   F1
        A  T  R  M  D  Q  Y  H  R  D  V  L  D  H  V  C  R  I  C  G  D  E  L  Q  A  R  A  V  K  R  Q  K  K  F  Y  E  V  K  G  A  A  E  T  I  E  V  H  Y  G  I  S  V  R  D  E  D  E  H  Q  F  P  G  Y  A  C  N  R   F2
        P  Q  G  W  T  S  T  I  G  M  S  L  I  M  S  A  G  Y  V  V  M  N  S  K  L  E  Q  *  S  D  R  R  S  F  M  R  *  K  G  Q  L  K  P  L  K  F  I  T  V  S  L  S  G  M  K  M  N  T  S  F  Q  V  M  L  A  T    F3
   1 AGCCACAAGGATGGACCAGTACCATCGGGATGTCCTTGATCATGTCTGCCGGATATGTGGTGATGAACTCCAAGCTAGAGCAGTAAAGCGACAGAAGAAGTTTTATGAGGTGAAAGGGGCAGCTGAAACCATTGAAGTTCATTACGGTATCTCTGTCAGGGATGAAGATGAACACCAGTTTCCAGGTTATGCTTGCAACA 200
     ----:----|----:----|----:----|----:----|----:----|----:----|----:----|----:----|----:----|----:----|----:----|----:----|----:----|----:----|----:----|----:----|----:----|----:----|----:----|----:----|
   1 TCGGTGTTCCTACCTGGTCATGGTAGCCCTACAGGAACTAGTACAGACGGCCTATACACCACTACTTGAGGTTCGATCTCGTCATTTCGCTGTCTTCTTCAAAATACTCCACTTTCCCCGTCGACTTTGGTAACTTCAAGTAATGCCATAGAGACAGTCCCTACTTCTACTTGTGGTCAAAGGTCCAATACGAACGTTGT 200
      L  W  L  S  P  G  T  G  D  P  H  G  Q  D  H  R  G  S  I  H  H  H  V  G  L  *  L  L  L  A  V  S  S  T  K  H  P  S  L  P  L  Q  F  W  Q  L  E  N  R  Y  R  Q  *  P  H  L  H  V  G  T  E  L  N  H  K  C  C   F6
     X  G  C  P  H  V  L  V  M  P  I  D  K  I  M  D  A  P  Y  T  T  I  F  E  L  S  S  C  Y  L  S  L  L  L  K  I  L  H  F  P  C  S  F  G  N  F  N  M  V  T  D  R  D  P  I  F  I  F  V  L  K  W  T  I  S  A  V   F5
       A  V  L  I  S  W  Y  W  R  S  T  R  S  *  T  Q  R  I  H  P  S  S  S  W  A  L  A  T  F  R  C  F  F  N  *  S  T  F  P  A  A  S  V  M  S  T  *  *  P  I  E  T  L  S  S  S  S  C  W  N  G  P  *  A  Q  L    F4

       V  Q  K  *  A  *  Q  T  E  K  C  E  R  V  Q  K  L  N  C  V  S  V  Q  P  S  C  R  *  R  L  F  L  Y  W  C  W  F  *  T  W  A  G  S  A  S  G  A  S  P  C  R  S  C  D  Y  H  C  G  P  C  L  S  K  F  R  V  P   F1
        C  R  N  K  L  D  R  L  K  N  V  K  E  S  K  N  L  T  V  F  Q  F  S  P  H  A  D  E  D  C  F  C  I  G  V  G  S  K  L  G  L  A  V  P  Q  E  P  V  P  V  D  P  V  T  I  T  V  D  H  A  Y  Q  S  S  G  S    F2
     G  A  E  I  S  L  T  D  *  K  M  *  K  S  P  K  T  *  L  C  F  S  S  A  L  M  Q  M  K  T  V  F  V  L  V  L  V  L  N  L  G  W  Q  C  L  R  S  Q  S  L  *  I  L  *  L  S  L  W  T  M  P  I  K  V  Q  G  P   F3
 201 GGTGCAGAAATAAGCTTGACAGACTGAAAAATGTGAAAGAGTCCAAAAACTTAACTGTGTTTCAGTTCAGCCCTCATGCAGATGAAGACTGTTTTTGTATTGGTGTTGGTTCTAAACTTGGGCTGGCAGTGCCTCAGGAGCCAGTCCCTGTAGATCCTGTGACTATCACTGTGGACCATGCCTATCAAAGTTCAGGGTCC 400
     ----:----|----:----|----:----|----:----|----:----|----:----|----:----|----:----|----:----|----:----|----:----|----:----|----:----|----:----|----:----|----:----|----:----|----:----|----:----|----:----|
 201 CCACGTCTTTATTCGAACTGTCTGACTTTTTACACTTTCTCAGGTTTTTGAATTGACACAAAGTCAAGTCGGGAGTACGTCTACTTCTGACAAAAACATAACCACAACCAAGATTTGAACCCGACCGTCACGGAGTCCTCGGTCAGGGACATCTAGGACACTGATAGTGACACCTGGTACGGATAGTTTCAAGTCCCAGG 400
       T  C  F  Y  A  Q  C  V  S  F  H  S  L  T  W  F  S  L  Q  T  E  T  *  G  E  H  L  H  L  S  N  K  Y  Q  H  Q  N  *  V  Q  A  P  L  A  E  P  A  L  G  Q  L  D  Q  S  *  *  Q  P  G  H  R  D  F  N  L  T    F6
      P  A  S  I  L  K  V  S  Q  F  I  H  F  L  G  F  V  *  S  H  K  L  E  A  R  M  C  I  F  V  T  K  T  N  T  N  T  R  F  K  P  Q  C  H  R  L  L  W  D  R  Y  I  R  H  S  D  S  H  V  M  G  I  L  T  *  P  G   F5
     L  H  L  F  L  S  S  L  S  F  F  T  F  S  D  L  F  K  V  T  N  *  N  L  G  *  A  S  S  S  Q  K  Q  I  P  T  P  E  L  S  P  S  A  T  G  *  S  G  T  G  T  S  G  T  V  I  V  T  S  W  A  *  *  L  E  P  D    F4

       F  M  *  *  T  S  Y  *  A  S  K  K  N  T  L  K  S  G  K  P  S  V  C  *  T  P  E  T  Q  G  H  D  F  R  S  *  P  V  L  *  R  T  Q  G  G  Y  C  G  C  P  L  L  C  T  Y  S  K  T  V  G  H  R  T  *  A  G    F1
     P  S  C  S  K  P  V  I  E  P  R  K  K  I  P  L  N  Q  A  N  R  Q  Y  V  K  R  Q  R  L  K  G  M  I  S  G  V  D  Q  F  C  E  E  H  K  E  D  T  V  D  A  L  Y  F  V  L  I  Q  K  L  S  D  T  G  R  K  Q  E   F2
      L  H  V  V  N  Q  L  L  S  L  E  K  K  Y  P  *  I  R  Q  T  V  S  M  L  N  A  R  D  S  R  A  *  F  Q  E  L  T  S  F  V  K  N  T  R  R  I  L  W  M  P  F  T  L  Y  L  F  K  N  C  R  T  Q  D  V  S  R  K   F3
 401 CCTTCATGTAGTAAACCAGTTATTGAGCCTCGAAAAAAAATACCCTTAAATCAGGCAAACCGTCAGTATGTTAAACGCCAGAGACTCAAGGGCATGATTTCAGGAGTTGACCAGTTTTGTGAAGAACACAAGGAGGATACTGTGGATGCCCTTTACTTTGTACTTATTCAAAAACTGTCGGACACAGGACGTAAGCAGGA 600
     ----:----|----:----|----:----|----:----|----:----|----:----|----:----|----:----|----:----|----:----|----:----|----:----|----:----|----:----|----:----|----:----|----:----|----:----|----:----|----:----|
 401 GGAAGTACATCATTTGGTCAATAACTCGGAGCTTTTTTTTATGGGAATTTAGTCCGTTTGGCAGTCATACAATTTGCGGTCTCTGAGTTCCCGTACTAAAGTCCTCAACTGGTCAAAACACTTCTTGTGTTCCTCCTATGACACCTACGGGAAATGAAACATGAATAAGTTTTTGACAGCCTGTGTCCTGCATTCGTCCT 600
     G  K  M  Y  Y  V  L  *  Q  A  E  F  F  F  V  R  L  D  P  L  G  D  T  H  *  V  G  S  V  *  P  C  S  K  L  L  Q  G  T  K  H  L  V  C  P  P  Y  Q  P  H  G  K  S  Q  V  *  E  F  V  T  P  C  L  V  Y  A  P   F6
      R  *  T  T  F  W  N  N  L  R  S  F  F  Y  G  *  I  L  C  V  T  L  I  N  F  A  L  S  E  L  A  H  N  *  S  N  V  L  K  T  F  F  V  L  L  I  S  H  I  G  K  V  K  Y  K  N  L  F  Q  R  V  C  S  T  L  L   F5
      G  E  H  L  L  G  T  I  S  G  R  F  F  I  G  K  F  *  A  F  R  *  Y  T  L  R  W  L  S  L  P  M  I  E  P  T  S  W  N  Q  S  S  C  L  S  S  V  T  S  A  R  *  K  T  S  I  *  F  S  D  S  V  P  R  L  C  S   F4

     S  *  *  S  S  F  N  V  E  W  P  *  *  G  G  V  I  S  T  R  M  L  G  Y  A  C  K  E  S  Y  V  *  S  S  L  Q  A  D  L  L  W  Q  A  V  C  T  F  I  R  E  T  R  Y  F  *  E  K  L  G  I  S  I  E  K  P  C  I   F1
      A  D  R  V  R  S  M  W  N  G  R  D  E  V  E  L  S  V  P  E  C  L  G  M  R  V  R  S  L  M  S  K  A  H  Y  K  Q  T  Y  Y  G  K  P  F  V  H  L  L  E  K  P  G  I  S  K  K  N  L  V  F  R  S  K  N  H  V  L   F2
      L  I  E  F  V  Q  C  G  M  A  V  M  R  W  S  Y  Q  Y  Q  N  A  W  V  C  V  *  G  V  L  C  L  K  L  I  T  S  R  P  I  M  A  S  R  L  Y  I  Y  *  R  N  Q  V  F  L  R  K  T  W  Y  F  D  R  K  T  M  Y   F3
 601 AGCTGATAGAGTTCGTTCAATGTGGAATGGCCGTGATGAGGTGGAGTTATCAGTACCAGAATGCTTGGGTATGCGTGTAAGGAGTCTTATGTCTAAAGCTCATTACAAGCAGACCTATTATGGCAAGCCGTTTGTACATTTATTAGAGAAACCAGGTATTTCTAAGAAAAACTTGGTATTTCGATCGAAAAACCATGTAT 800
     ----:----|----:----|----:----|----:----|----:----|----:----|----:----|----:----|----:----|----:----|----:----|----:----|----:----|----:----|----:----|----:----|----:----|----:----|----:----|----:----|
 601 TCGACTATCTCAAGCAAGTTACACCTTACCGGCACTACTCCACCTCAATAGTCATGGTCTTACGAACCCATACGCACATTCCTCAGAATACAGATTTCGAGTAATGTTCGTCTGGATAATACCGTTCGGCAAACATGTAAATAATCTCTTTGGTCCATAAAGATTCTTTTTGAACCATAAAGCTAGCTTTTTGGTACATA 800
      L  Q  Y  L  E  N  L  T  S  H  G  H  H  P  P  T  I  L  V  L  I  S  P  Y  A  H  L  S  D  *  T  *  L  E  N  C  A  S  R  N  H  C  A  T  Q  V  N  I  L  S  V  L  Y  K  *  S  F  S  P  I  E  I  S  F  G  H  I   F6
     F  S  I  S  N  T  *  H  P  I  A  T  I  L  H  L  *  *  Y  W  F  A  Q  T  H  T  Y  P  T  K  H  R  F  S  M  V  L  L  G  I  I  A  L  R  K  Y  M  *  *  L  F  W  T  N  R  L  F  V  Q  Y  K  S  R  F  V  M  Y   F5
       A  S  L  T  R  E  I  H  F  P  R  S  S  T  S  N  D  T  G  S  H  K  P  I  R  T  L  L  R  I  D  L  A  *  *  L  C  V  *  *  P  L  G  N  T  C  K  N  S  F  G  P  I  E  L  F  F  K  T  N  R  D  F  F  W  T    F4

       T  P  K  N  M  V  L  R  R  V  T  W  Y  F  D  R  N  T  L  F  F  A  *  N  M  V  F  R  *  K  T  R  *  F  R  R  E  T  R  F  Y  G  R  I  L  I  K  P  G  N  S  T  E  K  H  G  F  S  R  I  T  *  F  *  A  R  K   F1
       R  R  K  T  W  F  Y  G  E  L  L  G  I  S  T  E  I  P  C  F  S  R  K  T  W  Y  F  D  E  K  L  G  N  S  G  E  K  Q  G  F  T  A  V  S  *  *  N  L  V  I  L  P  K  N  M  V  F  R  E  L  P  S  F  K  Q  E    F2
     Y  A  E  K  H  G  F  T  A  S  Y  L  V  F  R  Q  K  Y  L  V  F  R  V  K  H  G  I  S  M  K  N  *  V  I  Q  E  R  N  K  V  L  R  P  Y  L  D  K  T  W  *  F  Y  R  K  T  W  F  F  A  N  Y  L  V  L  S  K  K   F3
 801 TACGCCGAAAAACATGGTTTTACGGCGAGTTACTTGGTATTTCGACAGAAATACCTTGTTTTTCGCGTAAAACATGGTATTTCGATGAAAAACTAGGTAATTCAGGAGAGAAACAAGGTTTTACGGCCGTATCTTGATAAAACCTGGTAATTCTACCGAAAAACATGGTTTTTCGCGAATTACCTAGTTTTAAGCAAGAA 1000
     ----:----|----:----|----:----|----:----|----:----|----:----|----:----|----:----|----:----|----:----|----:----|----:----|----:----|----:----|----:----|----:----|----:----|----:----|----:----|----:----|
 801 ATGCGGCTTTTTGTACCAAAATGCCGCTCAATGAACCATAAAGCTGTCTTTATGGAACAAAAAGCGCATTTTGTACCATAAAGCTACTTTTTGATCCATTAAGTCCTCTCTTTGTTCCAAAATGCCGGCATAGAACTATTTTGGACCATTAAGATGGCTTTTTGTACCAAAAAGCGCTTAATGGATCAAAATTCGTTCTT 1000
       V  G  F  F  M  T  K  R  R  T  V  Q  Y  K  S  L  F  V  K  N  K  A  Y  F  M  T  N  R  H  F  V  L  Y  N  L  L  S  V  L  N  *  P  R  I  K  I  F  G  P  L  E  V  S  F  C  P  K  E  R  I  V  *  N  *  A  L    F6
       *  A  S  F  C  P  K  V  A  L  *  K  T  N  R  C  F  Y  R  T  K  R  T  F  C  P  I  E  I  F  F  *  T  I  *  S  L  F  L  T  K  R  G  Y  R  S  L  V  Q  Y  N  *  R  F  V  H  N  K  A  F  *  R  T  K  L  L  F   F5
     N  R  R  F  V  H  N  *  P  S  N  S  P  I  E  V  S  I  G  Q  K  E  R  L  V  H  Y  K  S  S  F  S  P  L  E  P  S  F  C  P  K  V  A  T  D  Q  Y  F  R  T  I  R  G  F  F  M  T  K  R  S  N  G  L  K  L  C  S   F4

       L  E  K  N  K  V  F  R  E  K  L  G  F  S  R  I  T  K  *  F  D  F  R  W  V  S  P  V  D  L  V  P  W  C  G  R  L  G  N  H  L  R  P  E  *  Q  K  L  S  S  K  T  S  *  G  P  F  L  L  Y  L  T  R  S  T  A    F1
     S  W  R  K  T  R  Y  F  E  R  N  L  V  F  R  E  L  P  S  N  S  T  F  A  G  F  P  Q  S  T  S  F  P  G  A  A  G  *  E  T  T  *  G  R  S  D  R  N  *  A  Q  K  L  H  R  A  L  F  C  C  I  *  R  G  Q  P  L   F2
      V  G  E  K  Q  G  I  S  R  E  T  W  F  F  E  N  Y  Q  V  I  R  L  S  L  G  F  P  S  R  P  R  S  L  V  R  Q  V  R  K  P  P  E  A  G  V  T  E  I  E  L  K  N  F  I  G  P  F  S  V  V  F  D  E  V  N  R  Y   F3
1001 AGTTGGAGAAAAACAAGGTATTTCGAGAGAAACTTGGTTTTTCGAGAATTACCAAGTAATTCGACTTTCGCTGGGTTTCCCCAGTCGACCTCGTTCCCTGGTGCGGCAGGTTAGGAAACCACCTGAGGCCGGAGTGACAGAAATTGAGCTCAAAAACTTCATAGGGCCCTTTTCTGTTGTATTTGACGAGGTCAACCGCT 1200
     ----:----|----:----|----:----|----:----|----:----|----:----|----:----|----:----|----:----|----:----|----:----|----:----|----:----|----:----|----:----|----:----|----:----|----:----|----:----|----:----|
1001 TCAACCTCTTTTTGTTCCATAAAGCTCTCTTTGAACCAAAAAGCTCTTAATGGTTCATTAAGCTGAAAGCGACCCAAAGGGGTCAGCTGGAGCAAGGGACCACGCCGTCCAATCCTTTGGTGGACTCCGGCCTCACTGTCTTTAACTCGAGTTTTTGAAGTATCCCGGGAAAAGACAACATAAACTGCTCCAGTTGGCGA 1200
     F  N  S  F  F  L  T  N  R  S  F  S  P  K  E  L  I  V  L  Y  N  S  K  R  Q  T  E  G  T  S  R  T  G  Q  H  P  L  N  P  F  W  R  L  G  S  H  C  F  N  L  E  F  V  E  Y  P  G  K  R  N  Y  K  V  L  D  V  A   F6
       T  P  S  F  C  P  I  E  L  S  V  Q  N  K  S  F  *  W  T  I  R  S  E  S  P  K  G  L  R  G  R  E  R  T  R  C  T  L  F  G  G  S  A  P  T  V  S  I  S  S  L  F  K  M  P  G  K  E  T  T  N  S  S  T  L  R    F5
      L  Q  L  F  V  L  Y  K  S  L  F  K  T  K  R  S  N  G  L  L  E  V  K  A  P  N  G  W  D  V  E  N  G  P  A  A  P  *  S  V  V  Q  P  R  L  S  L  F  Q  A  *  F  S  *  L  A  R  K  Q  Q  I  Q  R  P  *  G  S   F4

     T  V  I  G  L  H  K  H  D  K  A  R  Y  S  V  I  *  D  N  T  *  L  C  T  R  T  K  T  V  C  Y  S  S  Q  A  *  A  W  A  Y  I  H  V  D  R  *  A  N  I  L  F  K  D  A  A  A  T  T  R  S  *  L  N  V  G  L  W   F1
      L  *  *  A  Y  I  N  M  T  K  L  G  I  L  S  S  E  I  I  H  D  C  A  R  E  Q  K  R  S  A  I  A  H  R  P  R  P  G  P  T  Y  M  *  I  D  K  Q  T  Y  F  S  R  M  L  L  R  R  L  V  L  D  *  M  *  V  F  G   F2
      C  N  R  P  T  *  T  *  Q  S  *  V  F  C  H  L  R  *  Y  M  I  V  H  E  N  K  N  G  L  L  *  L  T  G  L  G  L  G  L  H  T  C  R  *  I  S  K  H  T  F  Q  G  C  C  C  D  D  S  F  L  I  E  C  R  S  L   F3
1201 ACTGTAATAGGCCTACATAAACATGACAAAGCTAGGTATTCTGTCATCTGAGATAATACATGATTGTGCACGAGAACAAAAACGGTCTGCTATAGCTCACAGGCCTAGGCCTGGGCCTACATACATGTAGATAGATAAGCAAACATACTTTTCAAGGATGCTGCTGCGACGACTCGTTCTTGATTGAATGTAGGTCTTTG 1400
     ----:----|----:----|----:----|----:----|----:----|----:----|----:----|----:----|----:----|----:----|----:----|----:----|----:----|----:----|----:----|----:----|----:----|----:----|----:----|----:----|
1201 TGACATTATCCGGATGTATTTGTACTGTTTCGATCCATAAGACAGTAGACTCTATTATGTACTAACACGTGCTCTTGTTTTTGCCAGACGATATCGAGTGTCCGGATCCGGACCCGGATGTATGTACATCTATCTATTCGTTTGTATGAAAAGTTCCTACGACGACGCTGCTGAGCAAGAACTAACTTACATCCAGAAAC 1400
      V  T  I  P  R  C  L  C  S  L  A  L  Y  E  T  M  Q  S  L  V  H  N  H  V  L  V  F  V  T  Q  *  L  E  C  A  *  A  Q  A  *  M  C  T  S  L  Y  A  F  M  S  K  L  S  A  A  A  V  V  R  E  Q  N  F  T  P  R  Q   F6
     *  Q  L  L  G  V  Y  V  H  C  L  *  T  N  Q  *  R  L  Y  Y  M  I  T  C  S  F  L  F  P  R  S  Y  S  V  P  R  P  R  P  R  C  V  H  L  Y  I  L  L  C  V  K  *  P  H  Q  Q  S  S  E  N  K  I  S  H  L  D  K   F5
       S  Y  Y  A  *  M  F  M  V  F  S  P  I  R  D  D  S  I  I  C  S  Q  A  R  S  C  F  R  D  A  I  A  *  L  G  L  G  P  G  V  Y  M  Y  I  S  L  C  V  Y  K  E  L  I  S  S  R  R  S  T  R  S  Q  I  Y  T  K    F4

       P  V  N  A  I  K  Q  K  M  R  V  W  M  S  W  M  A  C  I  A  A  E  D  V  T  P  T  *  Q  L  S  G  *  N  W  N  Y  L  I  M  C  V  S  R  F  V  S  F  V  S  L  F  Y  F  I  V  L  *  S  S  S  R  S  *  K  Q  D   F1
       Q  S  M  L  L  N  K  R  C  V  F  G  C  L  G  W  L  V  S  L  L  R  M  L  R  L  L  S  S  *  A  D  E  I  G  I  I  *  L  C  V  S  V  G  S  F  R  S  F  H  C  F  I  S  L  F  Y  N  H  P  V  V  V  E  N  K    F2
     A  S  Q  C  Y  *  T  K  D  A  C  L  D  V  L  D  G  L  Y  R  C  *  G  C  Y  A  Y  L  A  A  K  R  M  K  L  E  L  S  D  Y  V  C  Q  *  V  R  F  V  R  F  I  V  L  F  H  C  F  I  I  I  Q  S  *  L  K  T  R   F3
1401 GCCAGTCAATGCTATTAAACAAAAGATGCGTGTTTGGATGTCTTGGATGGCTTGTATCGCTGCTGAGGATGTTACGCCTACTTAGCAGCTAAGCGGATGAAATTGGAATTATCTGATTATGTGTGTCAGTAGGTTCGTTTCGTTCGTTTCATTGTTTTATTTCATTGTTTTATAATCATCCAGTCGTAGTTGAAAACAAG 1600
     ----:----|----:----|----:----|----:----|----:----|----:----|----:----|----:----|----:----|----:----|----:----|----:----|----:----|----:----|----:----|----:----|----:----|----:----|----:----|----:----|
1401 CGGTCAGTTACGATAATTTGTTTTCTACGCACAAACCTACAGAACCTACCGAACATAGCGACGACTCCTACAATGCGGATGAATCGTCGATTCGCCTACTTTAACCTTAATAGACTAATACACACAGTCATCCAAGCAAAGCAAGCAAAGTAACAAAATAAAGTAACAAAATATTAGTAGGTCAGCATCAACTTTTGTTC 1600
       G  T  L  A  I  L  C  F  I  R  T  Q  I  D  Q  I  A  Q  I  A  A  S  S  T  V  G  V  *  C  S  L  P  H  F  Q  F  *  R  I  I  H  T  L  L  N  T  E  N  T  E  N  N  *  K  M  T  K  Y  D  D  L  R  L  Q  F  C    F6
      A  L  *  H  *  *  V  F  S  A  H  K  S  T  K  S  P  K  Y  R  Q  Q  P  H  *  A  *  K  A  A  L  R  I  F  N  S  N  D  S  *  T  H  *  Y  T  R  K  T  R  K  M  T  K  N  *  Q  K  I  I  M  W  D  Y  N  F  V  L   F5
     P  W  D  I  S  N  F  L  L  H  T  N  P  H  R  P  H  S  T  D  S  S  L  I  N  R  R  S  L  L  *  A  S  S  I  P  I  I  Q  N  H  T  D  T  P  E  N  R  E  N  *  Q  K  I  E  N  N  *  L  *  G  T  T  T  S  F  L   F4

       Q  G  T  R  S  T  G  E  T  Q  R  K  S  N  Y  L  V  I  L  E  K  P  S  F  S  R  N  T  L  F  F  S  N  F  L  A  *  N  *  V  I  R  E  K  P  C  F  S  V  E  L  P  G  F  I  K  I  R  P  *  N  L  V  S  L  L   F1
     T  R  E  R  G  R  L  G  K  P  S  E  S  R  I  T  W  *  F  S  K  N  Q  V  S  L  E  I  P  C  F  S  P  T  F  L  L  K  T  R  *  F  A  K  N  H  V  F  R  *  N  Y  Q  V  L  S  R  Y  G  R  K  T  L  F  L  S  *   F2
      P  G  N  E  V  D  W  G  N  P  A  K  V  E  L  L  G  N  S  R  K  T  K  F  L  S  K  Y  L  V  F  L  Q  L  S  C  L  K  L  G  N  S  R  K  T  M  F  F  G  R  I  T  R  F  Y  Q  D  T  A  V  K  P  C  F  S  P  E   F3
1601 ACCAGGGAACGAGGTCGACTGGGGAAACCCAGCGAAAGTCGAATTACTTGGTAATTCTCGAAAAACCAAGTTTCTCTCGAAATACCTTGTTTTTCTCCAACTTTCTTGCTTAAAACTAGGTAATTCGCGAAAAACCATGTTTTTCGGTAGAATTACCAGGTTTTATCAAGATACGGCCGTAAAACCTTGTTTCTCTCCTG 1800
     ----:----|----:----|----:----|----:----|----:----|----:----|----:----|----:----|----:----|----:----|----:----|----:----|----:----|----:----|----:----|----:----|----:----|----:----|----:----|----:----|
1601 TGGTCCCTTGCTCCAGCTGACCCCTTTGGGTCGCTTTCAGCTTAATGAACCATTAAGAGCTTTTTGGTTCAAAGAGAGCTTTATGGAACAAAAAGAGGTTGAAAGAACGAATTTTGATCCATTAAGCGCTTTTTGGTACAAAAAGCCATCTTAATGGTCCAAAATAGTTCTATGCCGGCATTTTGGAACAAAGAGAGGAC 1800
     S  W  P  V  L  D  V  P  S  V  W  R  F  D  F  *  K  T  I  R  S  F  G  L  K  E  R  F  V  K  N  K  E  L  K  R  A  *  F  *  T  I  R  S  F  G  H  K  E  T  S  N  G  P  K  I  L  I  R  G  Y  F  R  T  E  R  R   F6
      G  P  F  S  T  S  Q  P  F  G  A  F  T  S  N  S  P  L  E  R  F  V  L  N  R  E  F  Y  R  T  K  R  W  S  E  Q  K  F  S  P  L  E  R  F  V  M  N  K  P  L  I  V  L  N  *  *  S  V  A  T  F  G  Q  K  E  G   F5
     V  L  S  R  P  R  S  P  F  G  L  S  L  R  I  V  Q  Y  N  E  F  F  W  T  E  R  S  I  G  Q  K  E  G  V  K  K  S  L  V  L  Y  N  A  F  F  W  T  K  R  Y  F  *  W  T  K  D  L  Y  P  R  L  V  K  N  R  E  Q   F4

     N  Y  L  V  L  H  R  N  T  M  F  Y  A  K  N  K  V  F  L  S  K  Y  Q  V  T  H  R  K  T  M  F  F  G  V  I  H  G  F  S  I  E  I  P  S  F  S  *  K  Y  L  V  S  L  I  N  V  Q  T  A  C  H  N  L  W  H  S  K   F1
      I  T  *  F  F  I  E  I  P  C  F  T  R  K  T  R  Y  F  C  R  N  T  K  *  L  T  V  K  P  C  F  S  A  *  Y  M  V  F  R  S  K  Y  Q  V  F  R  R  N  T  W  F  L  *  *  M  Y  K  R  L  A  I  T  Y  G  I  Q  R   F2
      L  P  S  S  S  S  K  Y  H  V  L  R  E  K  Q  G  I  S  V  E  I  P  S  N  S  P  *  N  H  V  F  R  R  N  T  W  F  F  D  R  N  T  K  F  F  V  E  I  P  G  F  F  N  K  C  T  N  G  L  P  *  P  M  A  F  K    F3
1801 AATTACCTAGTTCTTCATCGAAATACCATGTTTTACGCGAAAAACAAGGTATTTCTGTCGAAATACCAAGTAACTCACCGTAAAACCATGTTTTTCGGCGTAATACATGGTTTTTCGATCGAAATACCAAGTTTTTCGTAGAAATACCTGGTTTCTTTAATAAATGTACAAACGGCTTGCCATAACCTATGGCATTCAAA 2000
     ----:----|----:----|----:----|----:----|----:----|----:----|----:----|----:----|----:----|----:----|----:----|----:----|----:----|----:----|----:----|----:----|----:----|----:----|----:----|----:----|
1801 TTAATGGATCAAGAAGTAGCTTTATGGTACAAAATGCGCTTTTTGTTCCATAAAGACAGCTTTATGGTTCATTGAGTGGCATTTTGGTACAAAAAGCCGCATTATGTACCAAAAAGCTAGCTTTATGGTTCAAAAAGCATCTTTATGGACCAAAGAAATTATTTACATGTTTGCCGAACGGTATTGGATACCGTAAGTTT 2000
      F  *  R  T  R  *  R  F  V  M  N  *  A  F  F  L  T  N  R  D  F  Y  W  T  V  *  R  L  V  M  N  K  P  T  I  C  P  K  E  I  S  I  G  L  K  E  Y  F  Y  R  T  E  K  I  F  T  C  V  A  Q  W  L  R  H  C  E  F   F6
     S  N  G  L  E  E  D  F  Y  W  T  K  R  S  F  C  P  I  E  T  S  I  G  L  L  E  G  Y  F  W  T  K  R  R  L  V  H  N  K  S  R  F  V  L  N  K  T  S  I  G  P  K  K  L  L  H  V  F  P  K  G  Y  G  I  A  N  L   F5
       I  V  *  N  K  M  S  I  G  H  K  V  R  F  V  L  Y  K  Q  R  F  V  L  Y  S  V  T  F  G  H  K  E  A  Y  Y  M  T  K  R  D  F  Y  W  T  K  R  L  F  V  Q  N  R  *  Y  I  Y  L  R  S  A  M  V  *  P  M  *   F4

       R  *  D  L  C  Q  V  T  A  T  T  K  P  A  H  S  P  G  T  I  I  S  S  R  T  L  Q  V  Q  T  V  K  G  F  R  F  A  G  *  T  G  F  T  Q  C  T  R  Q  S  A  P  *  Y  H  E  G  L  S  F  I  P  C  *  L  S  I  T   F1
        D  K  T  S  A  K  S  L  Q  P  R  N  Q  L  T  V  L  E  P  S  F  L  P  G  H  C  K  Y  R  L  L  K  D  S  V  L  L  A  E  Q  D  L  L  S  A  Q  D  S  Q  P  L  D  I  M  R  D  Y  H  S  F  P  A  D  F  P  L   F2
     E  I  R  P  L  P  S  H  C  N  H  E  T  S  S  Q  S  W  N  H  H  F  F  Q  D  I  A  S  T  D  C  *  R  I  P  F  C  W  L  N  R  I  Y  S  V  H  K  T  V  S  P  L  I  S  *  G  T  I  I  H  S  L  L  T  F  H  Y   F3
2001 GAGATAAGACCTCTGCCAAGTCACTGCAACCACGAAACCAGCTCACAGTCCTGGAACCATCATTTCTTCCAGGACATTGCAAGTACAGACTGTTAAAGGATTCCGTTTTGCTGGCTGAACAGGATTTACTCAGTGCACAAGACAGTCAGCCCCTTGATATCATGAGGGACTATCATTCATTCCCTGCTGACTTTCCATTA 2200
     ----:----|----:----|----:----|----:----|----:----|----:----|----:----|----:----|----:----|----:----|----:----|----:----|----:----|----:----|----:----|----:----|----:----|----:----|----:----|----:----|
2001 CTCTATTCTGGAGACGGTTCAGTGACGTTGGTGCTTTGGTCGAGTGTCAGGACCTTGGTAGTAAAGAAGGTCCTGTAACGTTCATGTCTGACAATTTCCTAAGGCAAAACGACCGACTTGTCCTAAATGAGTCACGTGTTCTGTCAGTCGGGGAACTATAGTACTCCCTGATAGTAAGTAAGGGACGACTGAAAGGTAAT 2200
       L  Y  S  R  Q  W  T  V  A  V  V  F  G  A  *  L  G  P  V  M  M  E  E  L  V  N  C  T  C  V  T  L  P  N  R  K  A  P  Q  V  P  N  V  *  H  V  L  C  D  A  G  Q  Y  *  S  P  S  D  N  M  G  Q  Q  S  E  M    F6
      S  I  L  G  R  G  L  *  Q  L  W  S  V  L  E  C  D  Q  F  W  *  K  K  W  S  M  A  L  V  S  Q  *  L  I  G  N  Q  Q  S  F  L  I  *  E  T  C  L  V  T  L  G  K  I  D  H  P  V  I  M  *  E  R  S  V  K  W  *   F5
     L  S  L  V  E  A  L  D  S  C  G  R  F  W  S  V  T  R  S  G  D  N  R  G  P  C  Q  L  Y  L  S  N  F  S  E  T  K  S  A  S  C  S  K  S  L  A  C  S  L  *  G  R  S  I  M  L  S  *  *  E  N  G  A  S  K  G  N    F4

       K  C  N  G  S  *  T  *  L  H  F  C  C  C  K  N  A  S  R  N  R  Q  *  R  V  K  E  T  G  C  V  G  I  A  S  V  N  K  T  L  Y  C  H  Q  R  W  R  *  W  T  G  G  C  K  C  *  E  *  K  G  *  F  C  P  Y  *   F1
     P  N  V  M  G  V  E  L  D  Y  I  S  A  V  A  K  T  L  L  E  I  D  N  D  V  S  K  R  L  D  A  L  G  L  P  Q  S  T  K  L  C  T  V  I  K  D  G  G  D  G  L  G  D  V  N  V  K  S  R  K  A  D  F  A  L  T  D   F2
      Q  M  *  W  E  L  N  L  I  T  F  L  L  L  Q  K  R  F  *  K  *  T  M  T  C  Q  R  D  W  M  R  W  D  C  L  S  Q  Q  N  F  V  L  S  S  K  M  A  V  M  D  W  G  M  *  M  L  R  V  E  R  L  I  L  P  L  L  T   F3
2201 CCAAATGTAATGGGAGTTGAACTTGATTACATTTCTGCTGTTGCAAAAACGCTTCTAGAAATAGACAATGACGTGTCAAAGAGACTGGATGCGTTGGGATTGCCTCAGTCAACAAAACTTTGTACTGTCATCAAAGATGGCGGTGATGGACTGGGGGATGTAAATGTTAAGAGTAGAAAGGCTGATTTTGCCCTTACTGA 2400
     ----:----|----:----|----:----|----:----|----:----|----:----|----:----|----:----|----:----|----:----|----:----|----:----|----:----|----:----|----:----|----:----|----:----|----:----|----:----|----:----|
2201 GGTTTACATTACCCTCAACTTGAACTAATGTAAAGACGACAACGTTTTTGCGAAGATCTTTATCTGTTACTGCACAGTTTCTCTGACCTACGCAACCCTAACGGAGTCAGTTGTTTTGAAACATGACAGTAGTTTCTACCGCCACTACCTGACCCCCTACATTTACAATTCTCATCTTTCCGACTAAAACGGGAATGACT 2400
     V  L  H  L  P  L  Q  V  Q  N  C  K  Q  Q  Q  L  F  A  E  L  F  L  C  H  R  T  L  S  V  P  H  T  P  I  A  E  T  L  L  V  K  Y  Q  *  *  L  H  R  H  H  V  P  P  H  L  H  *  S  Y  F  P  Q  N  Q  G  *  Q   F6
      W  I  Y  H  S  N  F  K  I  V  N  R  S  N  C  F  R  K  *  F  Y  V  I  V  H  *  L  S  Q  I  R  Q  S  Q  R  L  *  C  F  K  T  S  D  D  F  I  A  T  I  S  Q  P  I  Y  I  N  L  T  S  L  S  I  K  G  K  S   F5
      G  F  T  I  P  T  S  S  S  *  M  E  A  T  A  F  V  S  R  S  I  S  L  S  T  D  F  L  S  S  A  N  P  N  G  *  D  V  F  S  Q  V  T  M  L  S  P  P  S  P  S  P  S  T  F  T  L  L  L  F  A  S  K  A  R  V  S   F4
```

```
      Q G F P I F F L C F A M C S K * C T W * K T Y H F Q R G I S Q L C * L Y S S G C G L * L * * E * Q A Q C G N N L V S C L * S Q K T D A  F1
       K V F R Y S F C V L Q C A A S D A H G E K H T I F K E E F P N S V D C T R P V V V C N C D E S D R P S V A I I L S H V S K A R K Q M Q  F2
       R F S D I L S V F C N V Q Q V M H M V K N I P F S K R N F P T L L T V L V R L W F V T V M R V T G P V W Q * S C L M S L K P E N R C   F3
2401 CAAGGTTTTCCGATATTCTTTCTGTGTTTTGCAATGTGCAGCAAGTGATGCACATGGTGAAAAACATACCATTTTCAAAGAGGAATTTCCCAACTCTGTTGACTGTACTCGTCCGGTTGTGGTTTGTAACTGTGATGAGAGTGACAGGCCCAGTGTGGCAATAATCTTGTCTCATGTCTCTAAAGCCAGAAAACAGATGC 2600
     ----:----|----:----|----:----|----:----|----:----|----:----|----:----|----:----|----:----|----:----|----:----|----:----|----:----|----:----|----:----|----:----|----:----|----:----|----:----|----:----|
2401 GTTCCAAAAGGCTATAAGAAAGACACAAAACGTTACACGTCGTTCACTACGTGTACCACTTTTTGTATGGTAAAAGTTTCTCCTTAAAGGGTTGAGACAACTGACATGAGCAGGCCAACACCAAACATTGACACTACTCTCACTGTCCGGGTCACACCGTTATTAGAACAGAGTACAGAGATTTCGGTCTTTTGTCTACG 2600
      C P K G I N K R H K A I H L L H H V H H F V Y W K * L P I E W S Q Q S Y E D P Q P K Y S H H S H C A W H P L L R T E H R * L W F V S A  F6
     V L N E S I R E T N Q L T C C T I C M T F F M G N E F L F K G V R N V T S T R N H N T V T I L T V P G T H C Y D Q R M D R F G S F L H  F5
       L T K R Y E K Q T K C H A A L S A C P S F C V M K L S S N G L E T S Q V R G T T T Q L Q S S L S L G L T A I I K D * T E L A L F C I   F4

       R G Y A S A F R P I F R Q C Y S R A * S V L L L H H G G R K I R K D F I R S A G Q W V K V P L H P L P C Y H C * L P G * C R I S S * G  F1
       G A T L Q L L D P S S G S V I R E H D L S F Y Y T M V D E K F E R I S S G L Q G S G S R F L C T L C H A T T A S C Q A D A G S H H R   F2
      K G L R F S F * T H L Q A V L F E S M I C P F T T P W W T K N S K G F H Q V C R A V G Q G S S A P S A M L P L L V A R L M Q D L I I G  F3
2601 AAGGGGCTACGCTTCAGCTTTTAGACCCATCTTCAGGCAGTGTTATTCGAGAGCATGATCTGTCCTTTTACTACACCATGGTGGACGAAAAATTCGAAAGGATTTCATCAGGTCTGCAGGGCAGTGGGTCAAGGTTCCTCTGCACCCTCTGCCATGCTACCACTGCTAGTTGCCAGGCTGATGCAGGATCTCATCATAGG 2800
     ----:----|----:----|----:----|----:----|----:----|----:----|----:----|----:----|----:----|----:----|----:----|----:----|----:----|----:----|----:----|----:----|----:----|----:----|----:----|----:----|
2601 TTCCCCGATGCGAAGTCGAAAATCTGGGTAGAAGTCCGTCACAATAAGCTCTCGTACTAGACAGGAAAATGATGTGGTACCACCTGCTTTTTAAGCTTTCCTAAAGTAGTCCAGACGTCCCGTCACCCAGTTCCAAGGAGACGTGGGAGACGGTACGATGGTGACGATCAACGGTCCGACTACGTCCTAGAGTAGTATCC 2800
       L P * A E A K L G M K L C H * E L A H D T R K S C W P P R F I R F S K M L D A P C H T L T G R C G R G H * W Q * N G P Q H L I E D Y   F6
      L P S R K L K * V W R * A T N N S L M I Q G K V V G H H V F F E F P N * * T Q L A T P * P E E A G E A M S G S S T A L S I C S R M M P  F5
     C P A V S * S K S G D E P L T I R S C S R D K * * V M T S S F N S L I E D P R C P L P D L N R Q V R Q W A V V A L Q W A S A P D * * L  F4

       S N S * R N S L I V * I S T * K S S K L G * R * T S Y T K Q R G E K F P I D F F K C K * A F S R C H T C Q Y Q Y W K G F E K G G C Q   F1
     V R T H E E T V S L Y K Y R L E N P Q N L G E D E L A I Q S K G V K S F P L I S S N A N E R S L D A T H A N I N I G R V L K K V V V R  F2
      F E L M K K Q S H C I N I D L K I L K T W V K M N * L Y K A K G * K V S H * F L Q M Q M S V L * M P H M P I S I L E G F * K R W L S G  F3
2801 GTTCGAACTCATGAAGAAACAGTCTCATTGTATAAATATCGACTTGAAAATCCTCAAAACTTGGGTGAAGATGAACTAGCTATACAAAGCAAAGGGGTGAAAAGTTTCCCATTGATTTCTTCAAATGCAAATGAGCGTTCTCTAGATGCCACACATGCCAATATCAATATTGGAAGGGTTTTGAAAAAGGTGGTTGTCAG 3000
     ----:----|----:----|----:----|----:----|----:----|----:----|----:----|----:----|----:----|----:----|----:----|----:----|----:----|----:----|----:----|----:----|----:----|----:----|----:----|----:----|
2801 CAAGCTTGAGTACTTCTTTGTCAGAGTAACATATTTATAGCTGAACTTTTAGGAGTTTTGAACCCACTTCTACTTGATCGATATGTTTCGTTTCCCCACTTTTCAAAGGGTAACTAAAGAAGTTTACGTTTACTCGCAAGAGATCTACGGTGTGTACGGTTATAGTTATAACCTTCCCAAAACTTTTTCCACCAACAGTC 3000
     P E F E H L F L R M T Y I D V Q F D E F S P H L H V L * V F C L P S F N G M S K K L H L H A N E L H W V H W Y * Y Q F P K S F P P Q *  F6
       N S S M F F C D * Q I F I S K F I R L V Q T F I F * S Y L A F P H F T E W Q N R * I C I L T R * I G C M G I D I N S P N Q F L H N D   F5
      T R V * S S V T E N Y L Y R S S F G * F K P S S S S A I C L L P T F L K G N I E E F A F S R E R S A V C A L I L I P L T K F F T T T L  F4

     G N C * N S * M G R K * * Q * N Q S S K C R E G V * C K N H E G N W A S T Q N D D G W K L C E G C L C C * K S W G C N Q F D Q K * * K  F1
       E I A E I H E W A E N D N N K T S L Q N A E K E F D A K I M K E I G L Q P K M M M A G N Y A R A V F A V E N L G V V T S L I K N D E R  F2
       K L L K F M N G Q K M I T I K P V F K M Q R R S L M Q K S * R K L G F N P K * * W L E I M R G L S L L L K I L G L * P V * S K M M K   F3
3001 GGAAATTGCTGAAATTCATGAATGGGCAGAAAATGATAACAATAAAACCAGTCTTCAAAATGCAGAGAAGGAGTTTGATGCAAAAATCATGAAGGAAATTGGGCTTCAACCCAAAATGATGATGGCTGGAAATTATGCGAGGGCTGTCTTTGCTGTTGAAAATCTTGGGGTTGTAACCAGTTTGATCAAAAATGATGAAA 3200
     ----:----|----:----|----:----|----:----|----:----|----:----|----:----|----:----|----:----|----:----|----:----|----:----|----:----|----:----|----:----|----:----|----:----|----:----|----:----|----:----|
3001 CCTTTAACGACTTTAAGTACTTACCCGTCTTTTACTATTGTTATTTTGGTCAGAAGTTTTACGTCTCTTCCTCAAACTACGTTTTTAGTACTTCCTTTAACCCGAAGTTGGGTTTTACTACTACCGACCTTTAATACGCTCCCGACAGAAACGACAACTTTTAGAACCCCAACATTGGTCAAACTAGTTTTTACTACTTT 3200
       P F Q Q F E H I P L F H Y C Y F W D E F H L S P T Q H L F * S P F Q A E V W F S S P Q F N H S P Q R Q Q Q F D Q P Q L W N S * F H H F  F6
     P F N S F N M F P C F I I V I F G T K L I C L L L K I C F D H L F N P K L G F H H H S S I I R P S D K S N F I K P N Y G T Q D F I I F  F5
       S I A S I * S H A S F S L L L V L R * F A S F S N S A F I M F S I P S * G L I I I A P F * A L A T K A T S F R P T T V L K I L F S S  F4

       K G A C L Y F V Q H F * * I A Q S L Q Q * H P F N F S S R * D C S I Q R K C P P V C L P S W G K L S I R S M V Q L Y S Q G R G A C R *  F1
       R E H V S T L F N I F N R L H R V Y S S N T P L T S V P D E T A A Y R E N A L Q F A F H L G E N F P Y V P W S N Y I H R V V E H V D   F2
     E G S M S L L C S T F L I D C T E F T A V T P L * L Q F Q M R L Q H T E K M P S S L P S I L G K T F H T F H G P I I F T G S W S M * M  F3
3201 GAAGGGAGCATGTCTCTACTTTGTTCAACATTTTTAATAGATTGCACAGAGTTTACAGCAGTAACACCCCTTTAACTTCAGTTCCAGATGAGACTGCAGCATACAGAGAAAATGCCCTCCAGTTTGCCTTCCATCTTGGGGAAAACTTTCCATACGTTCCATGGTCCAATTATATTCACAGGGTCGTGGAGCATGTAGAT 3400
     ----:----|----:----|----:----|----:----|----:----|----:----|----:----|----:----|----:----|----:----|----:----|----:----|----:----|----:----|----:----|----:----|----:----|----:----|----:----|----:----|
3201 CTTCCCTCGTACAGAGATGAAACAAGTTGTAAAAATTATCTAACGTGTCTCAAATGTCGTCATTGTGGGGAAATTGAAGTCAAGGTCTACTCTGACGTCGTATGTCTCTTTTACGGGAGGTCAAACGGAAGGTAGAACCCCTTTTGAAAGGTATGCAAGGTACCAGGTTAATATAAGTGTCCCAGCACCTCGTACATCTA 3400
       F P A H R * K T * C K * Y I A C L K C C Y C G K L K L E L H S Q L M C L F H G G T Q R G D Q P F S E M R E M T W N Y E C P R P A H L   F6
      S P L M D R S Q E V N K I S Q V S N V A T V G R * S * N W I L S C C V S F I G E L K G E M K P F V K W V N W P G I I N V P D H L M Y I  F5
     L L S C T E V K N L M K L L N C L T * L L L V G K V E T G S S V A A Y L S F A R W N A K W R P S F K G Y T G H D L * I * L T T S C T S  F4

       N I T * S I R C A K C W T S L K * G R * I R E * T V P L Y Q K V I E * A G * C F * P S R C P C V S L A V H K * S S A K T C S H * K G  F1
     E I L H D P S G V Q S V G L L S S E G G E S G N K L Y R F I R K S L S R Q D S A F D L R D A L V F H W L Y T S K V L Q K H A A T E K V  F2
      K Y Y M I H Q V C K V L D F S Q V R A V N P G I N C T A L S E S H * V G R I V L L T F E M P L C F I G C T Q V K F C K N M Q P L K R S  F3
3401 GAAATATTACATGATCCATCAGGTGTGCAAAGTGTTGGACTTCTCTCAAGTGAGGGCGGTGAATCCGGGAATAAACTGTACCGCTTTATCAGAAAGTCATTGAGTAGGCAGGATAGTGCTTTTGACCTTCGAGATGCCCTTGTGTTTCATTGGCTGTACACAAGTAAAGTTCTGCAAAAACATGCAGCCACTGAAAAGGT 3600
     ----:----|----:----|----:----|----:----|----:----|----:----|----:----|----:----|----:----|----:----|----:----|----:----|----:----|----:----|----:----|----:----|----:----|----:----|----:----|----:----|
3401 CTTTATAATGTACTAGGTAGTCCACACGTTTCACAACCTGAAGAGAGTTCACTCCCGCCACTTAGGCCCTTATTTGACATGGCGAAATAGTCTTTCAGTAACTCATCCGTCCTATCACGAAAACTGGAAGCTCTACGGGAACACAAAGTAACCGACATGTGTTCATTTCAAGACGTTTTTGTACGTCGGTGACTTTTCCA 3600
     H F I V H D M L H A F H Q V E R L H P R H I R S Y V T G S * * F T M S Y A P Y H K Q G E L H G Q T E N A T C L Y L E A F V H L W Q F P  F6
       F Y * M I W * T H L T N S K E * T L A T F G P I F Q V A K D S L * Q T P L I T S K V K S I G K H K M P Q V C T F N Q L F M C G S F L   F5
      S I N C S G D P T C L T P S R E L S P P S D P F L S Y R K I L F D N L L C S L A K S R R S A R T N * Q S Y V L L T R C F C A A V S F T  F4

     Q * D L Y N M W N G R S Q S A Y M R I * A R C S P R R * H D G R K L N N I H E V F K L N F T S S S L F L F A F G L F W P T V N T H K R  F1
       S K T C T I C G M A D H N R R T C A Y K Q D A A P E D D M M E E S * T I Y M K Y S N * I S L L L L C F F L P L V C F G L Q S T H T K D  F2
       V R L V Q Y V E W Q I T I G V H A H I S K M Q P Q K M T * W K K V K Q Y T * S I Q I E F H F F F S V S F C L W F V L A Y S Q H T Q K   F3
3601 CAGTAAGACTTGTACAATATGTGGAATGGCAGATCACAATCGGCGTACATGCGCATATAAGCAAGATGCAGCCCCAGAAGATGACATGATGGAAGAAAGTTAAACAATATACATGAAGTATTCAAATTGAATTTCACTTCTTCTTCTCTGTTTCTTTTTGCCTTTGGTTTGTTTGGCCTACAGTCAACACACACAAAAG 3800
     ----:----|----:----|----:----|----:----|----:----|----:----|----:----|----:----|----:----|----:----|----:----|----:----|----:----|----:----|----:----|----:----|----:----|----:----|----:----|----:----|
3601 GTCATTCTGAACATGTTATACACCTTACCGTCTAGTGTTAGCCGCATGTACGCGTATATTCGTTCTACGTCGGGGTCTTCTACTGTACTACCTTCTTTCAATTTGTTATATGTACTTCATAAGTTTAACTTAAAGTGAAGAAGAAGAGACAAAGAAAAACGGAAACCAAACAAAACCGGATGTCAGTTGTGTGTGTTTTC 3800
       * Y S K Y L I H F P L D C D A Y M R M Y A L H L G L L H C S P L F N F L I C S T N L N F K V E E E R N R K A K P K N Q G V T L V C L L  F6
     D T L S T C Y T S H C I V I P T C A C I L L I C G W F I V H H F F T L C Y V H L I * I S N * K K K E T E K Q R Q N T K A * L * C V C F  F5
       L L V Q V I H P I A S * L R R V H A Y L C S A A G S S S M I S S L * V I Y M F Y E F Q I E S R R R Q K K K G K T Q K P R C D V C V F  F4

       L G L * S P I F * T S T A * V I P F V T S L T L * R L D D F S W * K I V S N N A F I R L T P A W L K S L C C * C E K V L I G T T M K E  F1
       S V S D L P F F E P A Q H E * Y H L S Q V S H C S D S M I S P G E K L S V T M H L S D * H L P G S S L F V V N V K R C * L A P P * K   F2
     T R S L I S H F L N Q H S M S D T I C H K S H T V A T R * F L L V K N C Q * Q C I Y Q I D T C L A Q V S L L L M * K G A D W H H H E R  F3
3801 ACTCGGTCTCTGATCTCCCATTTTTTGAACCAGCACAGCATGAGTGATACCATTTGTCACAAGTCTCACACTGTAGCGACTCGATGATTTCTCCTGGTGAAAAATTGTCAGTAACAATGCATTTATCAGATTGACACCTGCCTGGCTCAAGTCTCTTTGTTGTTAATGTGAAAAGGTGCTGATTGGCACCACCATGAAAG 4000
     ----:----|----:----|----:----|----:----|----:----|----:----|----:----|----:----|----:----|----:----|----:----|----:----|----:----|----:----|----:----|----:----|----:----|----:----|----:----|----:----|
3801 TGAGCCAGAGACTAGAGGGTAAAAAACTTGGTCGTGTCGTACTCACTATGGTAAACAGTGTTCAGAGTGTGACATCGCTGAGCTACTAAAGAGGACCACTTTTTAACAGTCATTGTTACGTAAATAGTCTAACTGTGGACGGACCGAGTTCAGAGAAACAACAATTACACTTTTCCACGACTAACCGTGGTGGTACTTTC 4000
       S P R Q D G M K Q V L V A H T I G N T V L R V S Y R S S S K E Q H F I T L L L A N I L N V G A Q S L D R Q Q * H S F T S I P V V M F   F6
     V R D R I E W K K F W C L M L S V M Q * L D * V T A V R H N R R T F F Q * Y C H M * * I S V Q R A * T E K N N I H F P A S Q C W W S L  F5
     S E T E S R G N K S G A C C S H Y W K D C T E C Q L S E I I E G P S F N D T V I C K D S Q C R G P E L R K T T L T F L H Q N A G G H F  F4

       N * C W V H H N C C V T G H G K P R G N I L H L N * V G C K V Q N E V L S T W F L C W V L I V L L L V S T K H K Y P T F I K S S G S   F1
     K I S A G S I I T A V * Q A M A N P E G I S F I * I E L V A R S K T K Y C P P G S F A G C * L S C C W Y P P N T S T L L S S R A V E V  F2
      K L V L G P S * L L C D R P W Q T Q R E Y P S F E L S W L Q G P K R S I V H L V P L L G A D C P A A G I H Q T Q V P Y F H Q E Q W K C  F3
4001 AAAATTAGTGCTGGGTCCATCATAACTGCTGTGTGACAGGCCATGGCAAACCCAGAGGGAATATCCTTCATTTGAATTGAGTTGGTTGCAAGGTCCAAAACGAAGTATTGTCCACCTGGTTCCTTTGCTGGGTGCTGATTGTCCTGCTGCTGGTATCCACCAAACACAAGTACCCTACTTTCATCAAGAGCAGTGGAAGT 4200
     ----:----|----:----|----:----|----:----|----:----|----:----|----:----|----:----|----:----|----:----|----:----|----:----|----:----|----:----|----:----|----:----|----:----|----:----|----:----|----:----|
4001 TTTTAATCACGACCCAGGTAGTATTGACGACACACTGTCCGGTACCGTTTGGGTCTCCCTTATAGGAAGTAAACTTAACTCAACCAACGTTCCAGGTTTTGCTTCATAACAGGTGGACCAAGGAAACGACCCACGACTAACAGGACGACGACCATAGGTGGTTTGTGTTCATGGGATGAAAGTAGTTCTCGTCACCTTCA 4200
     S F * H Q T W * L Q Q T V P W P L G L P F I R * K F Q T P Q L T W F S T N D V Q N R Q Q T S I T R S S T D V L C L Y G V K M L L L P L  F6
       F N T S P G D Y S S H S L G H C V W L S Y G E N S N L Q N C P G F R L I T W R T G K S P A S Q G A A P I W W V C T G * K * * S C H F   F5
       F I L A P D M M V A T H C A M A F G S P I D K M Q I S N T A L D L V F Y Q G G P E K A P H Q N D Q Q Q Y G G F V L V R S E D L A T S T  F4

     V S * * Q G V L A K K I W H D * L * H C H R Y L A I N E F Q N S Q F Y L K N * L A L N C C P I C P W H L S * T P D I C Y Y F V S Q F C  F1
      C P D N K V S L P R K F G M I S C D T V T D T W P S M N S R T A N F T S R I D W R S I V A P F A P G I C L R P P T Y A T T L S L S S V  F2
      V L I T R C P C Q E N L A * L A V T L S Q I P G H Q * I P E Q P I L P Q E L T G A Q L L P H L P L A F V L D P R H M L L C L S V L   F3
4201 GTGTCCTGATAACAAGGTGTCCTTGCCAAGAAAATTTGGCATGATTAGCTGTGACACTGTCACAGATACCTGGCCATCAATGAATTCCAGAACAGCCAATTTTACCTCAAGAATTGACTGGCGCTCAATTGTTGCCCCATTTGCCCCTGGCATTTGTCTTAGACCCCCGACATATGCTACTACTTTGTCTCTCAGTTCTG 4400
     ----:----|----:----|----:----|----:----|----:----|----:----|----:----|----:----|----:----|----:----|----:----|----:----|----:----|----:----|----:----|----:----|----:----|----:----|----:----|----:----|
4201 CACAGGACTATTGTTCCACAGGAACGGTTCTTTTAAACCGTACTAATCGACACTGTGACAGTGTCTATGGACCGGTAGTTACTTAAGGTCTTGTCGGTTAAAATGGAGTTCTTAACTGACCGCGAGTTAACAACGGGGTAAACGGGGACCGTAAACAGAATCTGGGGGCTGTATACGATGATGAAACAGAGAGTCAAGAC 4400
      T D Q Y C P T R A L F I Q C S * S H C Q * L Y R A M L S N W F L W N * R L F Q S A S L Q Q G M Q G Q C K D * V G S M H * * K T E * N Q  F6
     H T R I V L H G Q W S F K A H N A T V S D C I G P W * H I G S C G I K G * S N V P A * N N G W K G R A N T K S G R C I S S S Q R E T R  F5
       H G S L L T D K G L F N P M I L Q S V T V S V Q G D I F E L V A L K V E L I S Q R E I T A G N A G P M Q R L G G V Y A V V K D R L E   F4

       S G V I C Y R V * L F * N H L K P F V L E * I N E V * I L V K C P M T L G S F L Y T T E * * C S Q Q * T I F M D P S Q C V G I S S L R  F1
       P V * Y A T G F N C S E T I * N H L Y W S K S M K Y R S L S N V P * P S G L F S T P P N S N A P S N E P F S W I P V S V W A Y L P S   F2
      F R C D M L P G L I V L K P F E T I C T G V N Q * S I D P C Q M S H D P R V F S L H H R I V M L P A M N H F H G S Q S V C G H I F P Q  F3
4401 TTCCGGTGTGATATGCTACCGGGTTTAATTGTTCTGAAACCATTTGAAACCATTTGTACTGGAGTAAATCAATGAAGTATAGATCCTTGTCAAATGTCCCATGACCCTCGGGTCTTTTCTCTACACCACCGAATAGTAATGCTCCCAGCAATGAACCATTTTCATGGATCCCAGTCAGTGTGTGGGCATATCTTCCCTCA 4600
     ----:----|----:----|----:----|----:----|----:----|----:----|----:----|----:----|----:----|----:----|----:----|----:----|----:----|----:----|----:----|----:----|----:----|----:----|----:----|----:----|
4401 AAGGCCACACTATACGATGGCCCAAATTAACAAGACTTTGGTAAACTTTGGTAAACATGACCTCATTTAGTTACTTCATATCTAGGAACAGTTTACAGGGTACTGGGAGCCCAGAAAAGAGATGTGGTGGCTTATCATTACGAGGGTCGTTACTTGGTAAAAGTACCTAGGGTCAGTCACACACCCGTATAGAAGGGAGT 4600
       E P T I H * R T * N N Q F W K F G N T S S Y I L S T Y I R T L H G M V R P D K R * V V S Y Y H E W C H V M K M S G L * H T P M D E R   F6
      N R H S I S G P K I T R F G N S V M Q V P T F * H L I S G Q * I D W S G R T K E R C W R I T I S G A I F W K * P D W D T H P C I K G *  F5
      T G T H Y A V P N L Q E S V M Q F W K Y Q L L D I F Y L D K D F T G H G E P R K E V G G F L L A G L L S G N E H I G T L T H A Y R G E  F4

       N I A H L L N C C A G P F G S I R A Y Q F G R I I L V S T K S F * D N Q I I V G L H S I * V H A P P N E D Q C F C C P C P S N S S S   F1
     G T S P I C L T V V Q V P S G L S E P T S L A E * F W S L P K V S K T T K S L W G S I R F R S M P P Q M K I N V F V A P V L A T P L A  F2
      E H R P S A * L L C R S L R V Y Q S L P V W Q N N S G L Y Q K F L R Q P N H C G A P F D L G P C P P K * R S M F L L P L S * Q L L * L  F3
4601 GGAACATCGCCCATCTGCTTAACTGTTGTGCAGGTCCCTTCGGGTCTATCAGAGCCTACCAGTTTGGCAGAATAATTCTGGTCTCTACCAAAAGTTTCTAAGACAACCAAATCATTGTGGGGCTCCATTCGATTTAGGTCCATGCCCCCCCAAATGAAGATCAATGTTTTTGTTGCCCCTGTCCTAGCAACTCCTCTAGC 4800
     ----:----|----:----|----:----|----:----|----:----|----:----|----:----|----:----|----:----|----:----|----:----|----:----|----:----|----:----|----:----|----:----|----:----|----:----|----:----|----:----|
4601 CCTTGTAGCGGGTAGACGAATTGACAACACGTCCAGGGAAGCCCAGATAGTCTCGGATGGTCAAACCGTCTTATTAAGACCAGAGATGGTTTTCAAAGATTCTGTTGGTTTAGTAACACCCCGAGGTAAGCTAAATCCAGGTACGGGGGGGTTTACTTCTAGTTACAAAAACAACGGGGACAGGATCGTTGAGGAGATCG 4800
     L F M A W R S L Q Q A P G K P D I L A * W N P L I I R T E V L L K * S L W I M T P S W E I * T W A G G F S S * H K Q Q G Q G L L E E L  F6
       S C R G D A * S N H L D R R T * * L R G T Q C F L E P R * W F N R L C G F * Q P A G N S K P G H G G L H L D I N K N G R D * C S R *  F5
       P V D G M Q K V T T C T G E P R D S G V L K A S Y N Q D R G F T E L V V L D N H P E M R N L D M G G W I F I L T K T A G T R A V G R A  F4

     S S P W S F I T S S G C I R T S S S M * * R P E N N L C T T A F F F G C I E N F H I I F L N M E G I I E D S S G Q C C Q W Q P G M H A  F1
      L L L G A S S R V L V V S E Q A A A C N K G R R T I C A P L H F F S D A L R T F T S F S * T W K E * L K I V P D N V V S G S L G C M P  F2
       F S L E L H H E F W L Y Q N K Q Q H V I K A G E Q S V H H C I F F R M H * E L S H H F P E H G R N N * R * F R T M L S V A A W D A C   F3
4801 TCTTCTCCTTGGAGCTTCATCACGAGTTCTGGTTGTATCAGAACAAGCAGCAGCATGTAATAAAGGCCGGAGAACAATCTGTGCACCACTGCATTTTTTTTCGGATGCATTGAGAACTTTCACATCATTTTCCTGAACATGGAAGGAATAATTGAAGATAGTTCCGGACAATGTTGTCAGTGGCAGCCTGGGATGCATGC 5000
     ----:----|----:----|----:----|----:----|----:----|----:----|----:----|----:----|----:----|----:----|----:----|----:----|----:----|----:----|----:----|----:----|----:----|----:----|----:----|----:----|
4801 AGAAGAGGAACCTCGAAGTAGTGCTCAAGACCAACATAGTCTTGTTCGTCGTCGTACATTATTTCCGGCCTCTTGTTAGACACGTGGTGACGTAAAAAAAAGCCTACGTAACTCTTGAAAGTGTAGTAAAAGGACTTGTACCTTCCTTATTAACTTCTATCAAGGCCTGTTACAACAGTCACCGTCGGACCCTACGTACG 5000
       E E G Q L K M V L E P Q I L V L L L M Y Y L G S F L R H V V A N K K P H M S F K * M M K R F M S P I I S S L E P C H Q * H C G P I C A  F6
     S K E K S S * * S N Q N Y * F L C C C T I F A P S C D T C W Q M K K R I C Q S S E C * K G S C P L F L Q L Y N R V I N D T A A Q S A H  F5
       R R R P A E D R T R T T D S C A A A H L L P R L V I Q A G S C K K E S A N L V K V D N E Q V H F S Y N F I T G S L T T L P L R P H M   F4
```

```
       T  K  H  R  H  S  Y  Y  F  T  I  N  H  I  P  F  V  E  K  V  *  E  C  W  C  *  C  F  S  S  C  N  R  F  H  R  P  N  Y  M  S  W  P  R  K  E  S  *  I  A  Y  I  T  T  H  C  F  F  S  S  L  P  A  L  L  Y  S   F1
        P  S  T  D  T  V  I  I  L  P  S  I  T  S  R  S  S  K  K  C  E  S  A  G  V  N  V  S  L  V  A  T  D  S  T  V  Q  I  I  C  P  G  P  E  K  R  A  K  L  P  I  S  P  L  T  A  F  L  V  V  F  R  R  F  F  I     F2
      H  Q  A  Q  T  Q  L  L  F  Y  H  Q  S  H  P  V  R  R  K  S  V  R  V  L  V  L  M  F  L  *  L  Q  Q  I  P  P  S  K  L  Y  V  L  A  Q  K  R  E  L  N  C  L  Y  H  H  S  L  L  F  *  *  S  S  G  A  S  L  *   F3
5001 CACCAAGCACAGACACAGTTATTATTTTACCATCAATCACATCCCGTTCGTCGAAAAAGTGTGAGAGTGCTGGTGTTAATGTTTCTCTAGTTGCAACAGATTCCACCGTCCAAATTATATGTCCTGGCCCAGAAAAGAGAGCTAAATTGCCTATATCACCACTCACTGCTTTTTTAGTAGTCTTCCGGCGCTTCTTTATA 5200
     ----:----|----:----|----:----|----:----|----:----|----:----|----:----|----:----|----:----|----:----|----:----|----:----|----:----|----:----|----:----|----:----|----:----|----:----|----:----|----:----|
5001 GTGGTTCGTGTCTGTGTCAATAATAAAATGGTAGTTAGTGTAGGGCAAGCAGCTTTTTCACACTCTCACGACCACAATTACAAAGAGATCAACGTTGTCTAAGGTGGCAGGTTTAATATACAGGACCGGGTCTTTTCTCTCGATTTAACGGATATAGTGGTGAGTGACGAAAAAATCATCAGAAGGCCGCGAAGAAATAT 5200
        V  L  C  L  C  L  *  *  K  V  M  L  *  M  G  N  T  S  F  T  H  S  H  Q  H  *  H  K  E  L  Q  L  L  N  W  R  G  F  *  I  D  Q  G  L  F  S  L  *  I  A  *  I  V  V  *  Q  K  K  L  L  R  G  A  S  R  *    F6
       W  W  A  C  V  C  N  N  N  *  W  *  D  C  G  T  R  R  F  L  T  L  T  S  T  N  I  N  R  *  N  C  C  I  G  G  D  L  N  Y  T  R  A  W  F  L  S  S  F  Q  R  Y  *  W  E  S  S  K  *  Y  D  E  P  A  E  K  Y   F5
      G  G  L  V  S  V  T  I  I  K  G  D  I  V  D  R  E  D  F  F  H  S  L  A  P  T  L  T  E  R  T  A  V  S  E  V  T  W  I  I  H  G  P  G  S  F  L  A  L  N  G  I  D  G  S  V  A  K  K  T  T  K  R  R  K  K  I    F4

        F  *  N  G  I  E  S  G  I  L  P  K  I  V  G  S  V  Y  F  C  H  H  S  C  H  L  R  P  S  A  A  I  Y  L  P  V  V  N  A  N  S  T  T  A  P  A  W  H  T  L  G  T  Y  V  Q  T  V  K  L  L  I  *  T  V  R     F1
      A  F  K  M  E  *  N  R  E  S  C  Q  K  S  *  V  L  F  T  S  A  I  I  H  V  I  Y  A  P  A  Q  P  F  I  Y  L  L  S  M  P  T  A  Q  L  R  L  R  G  T  L  S  A  P  M  C  R  R  S  N  Y  L  S  R  Q  Y  X     F2
       L  L  K  W  N  R  I  G  N  L  A  K  N  R  R  F  C  L  L  L  P  S  F  M  S  F  T  P  Q  R  S  H  L  F  T  C  C  Q  C  Q  Q  H  N  C  A  C  V  A  H  S  R  H  L  C  A  D  G  Q  T  T  Y  L  D  S  T      F3
5201 GCTTTTAAAATGGAATAGAATCGGGAATCTTGCCAAAAATCGTAGGTTCTGTTTACTTCTGCCATCATTCATGTCATTTACGCCCCAGCGCAGCCATTTATTTACCTGTTGTCAATGCCAACAGCACAACTGCGCCTGCGTGGCACACTCTCGGCACCTATGTGCAGACGGTCAAACTACTTATCTAGACAGTACG 5396
     ----:----|----:----|----:----|----:----|----:----|----:----|----:----|----:----|----:----|----:----|----:----|----:----|----:----|----:----|----:----|----:----|----:----|----:----|----:----|----:-
5201 CGAAAATTTTACCTTATCTTAGCCCTTAGAACGGTTTTTAGCATCCAAGACAAATGAAGACGGTAGTAAGTACAGTAAATGCGGGGTCGCGTCGGTAAATAAATGGACAACAGTTACGGTTGTCGTGTTGACGCGGACGCACCGTGTGAGAGCCGTGGATACACGTCTGCCAGTTTGATGAATAGATCTGTCATGC 5396
      L  K  *  F  P  I  S  D  P  I  K  G  F  I  T  P  E  T  *  K  Q  W  *  E  H  *  K  R  G  L  A  A  M  *  K  G  T  T  L  A  L  L  V  V  A  G  A  H  C  V  R  P  V  *  T  C  V  T  L  S  S  I  *  V  T  R     F6
        S  K  F  H  F  L  I  P  F  R  A  L  F  R  L  N  Q  K  S  R  G  D  N  M  D  N  V  G  W  R  L  W  K  N  V  Q  Q  *  H  W  C  C  L  Q  A  Q  T  A  C  E  R  C  R  H  A  S  P  *  V  V  *  R  S  L  V     F5
       A  K  L  I  S  Y  F  R  S  D  Q  W  F  D  Y  T  R  N  V  E  A  M  M  *  T  M  *  A  G  A  C  G  N  I  *  R  N  D  I  G  V  A  C  S  R  R  R  P  V  S  E  A  G  I  H  L  R  D  F  *  K  D  L  C  Y     F4
```

Matching transcriptomic data (TSA)

RAG1L:

```
>WGS: NMRB01002705.1:26000-31000
>TSA: GFRY01095552.1:beg-end
>TSA: GFRY01095553.1:beg-end
>TSA: GFRY01095554.1:beg-end
```

```
              1                                                                                                                                                                                                     200
NMRB01002705  TGACTGGCACAATCACAAACTCGGATTTCTGGACCACAATTGTGAGTGTGTACAAAAAATGTCCATGAAGTCACAACAGATGACACATGCCATGTCATAACACTTACTGTTGTTAGTACGTGAAGATACACTTCTTCCTGTCCTTCCTGCAAAATATACAAAAATCTGGATGAATGCTGCAGAAGTTTGATAATGATGCA
GFRY01095553
GFRY01095554
GFRY01095552
 Consensus    ........................................................................................................................................................................................................

             201                                                                                                                                                                                                     400
NMRB01002705 CAGCGAAACACGCAATAGGTAAATATCCTTGATGGCTGCGGCCATGATGCCAGTTTACCTGTATATCACGTGTACTTGAAGCAGAAGTGCGCAGAAGAGAGTGTGGCTCATGTCCCAAACCCCTGTCGGTCCCCTATGCAGGAAAGCTATGCGCACTGCTCGCAATCTGGTGTGCAAACAACAGAGCAACGAGGAAGTGA
GFRY01095553                                                                                                                                           CAGGAAAGCTATGCGCACTGCTCGCAATCTGGTGTGCAAACAACAGAGCAACGAGGAAGTGA
GFRY01095554                                                                                                                                           CAGGAAAGCTATGCGCACTGCTCGCAATCTGGTGTGCAAACAACAGAGCAACGAGGAAGTGA
GFRY01095552                                                                                                                                           CAGGAAAGCTATGCGCACTGCTCGCAATCTGGTGTGCAAACAACAGAGCAACGAGGAAGTGA
 Consensus   ..........................................................................................................................................CAGGAAAGCTATGCGCACTGCTCGCAATCTGGTGTGCAAACAACAGAGCAACGAGGAAGTGA

             401                                                                                                                                                                                                     600
NMRB01002705 AAACCCGATTGAAATCGATGCAATATAGCCGATTTTCTGCAGAATTATAAAGTTTGTTCGTTAGAATCTGGAGTACACTTGTAATTTGGATAGTCTGGATTAAAGGTAAGTTTACTAATACCCATTTACCGACAGCACTTTCTGACCTACGCAGTTGGAATGCGTGGATCTCACTGTTTACATTGGTGTATACAAGTCCA
GFRY01095553 AAACCCGATTGAAATCGATGCAATATAGCCGATTTTCTGCAGAATTATAAAGTTTGTTCGTTAGAATCTGGAGTACACTTGTAATTTGGATAGTCTGGATTAAAGGTA--------------------------------------------------------------------------------------------
GFRY01095554 AAACCCGATTGAAATCGATGCAATATAGCCGATTTTCTGCAGAATTATAAAGTTTGTTCGTTAGAATCTGGAGTACACTTGTAATTTGGATAGTCTGGATTAAAGGTA--------------------------------------------------------------------------------------------
GFRY01095552 AAACCCGATTGAAATCGATGCAATATAGCCGATTTTCTGCAGAATTATAAAGTTTGTTCGTTAGAATCTGGAGTACACTTGTAATTTGGATAGTCTGGATTAAAGGTA--------------------------------------------------------------------------------------------
 Consensus   AAACCCGATTGAAATCGATGCAATATAGCCGATTTTCTGCAGAATTATAAAGTTTGTTCGTTAGAATCTGGAGTACACTTGTAATTTGGATAGTCTGGATTAAAGGTA............................................................................................

             601                                                                                                                                                                                                     800
NMRB01002705 TAGCTATTGCCGAAAGAAAAATGCTGTCGGAGGTTTTTCACACGATGTCAGGCTATTTTTAGTAAAATGTCTGGTAAACCAGAACTATTTGATGGTACTAGGTGGAAATTTGGCAGTGTTTTGGCATTGATTCATCATCTGATCAGCTGATCACGCAGTTTCTTAGAATCTTCTGCATCAGCCATGTCAGCATTAGGCC
GFRY01095553 --------------------------------------------------------------------------------------------------------------------------------------------------------------------------------------------------------
GFRY01095554 --------------------------------------------------------------------------------------------------------------------------------------------------------------------------------------------------------
GFRY01095552 --------------------------------------------------------------------------------------------------------------------------------------------------------------------------------------------------------
 Consensus   ........................................................................................................................................................................................................

             801                                                                                                                                                                                                    1000
NMRB01002705 TATTGATTTCTAGGATAAGAGGAACGAAAGGAGAATGGTACTTCTCCAATGTTTCAACATCAGCTGATTTCTTTAGCAGAAGCATCGTTTTTCACTGATATGTTACTTTCTCAGTTGTTCCAGAGCCCTGAGAATAATAACTCTGAATTTCAAGTATTATTTGCAATCTAGGCCTCATTGTGTTGCTTGTTTTTCAGGTA
GFRY01095553 --------------------------------------------------------------------------------------------------------------------------------------------------------------------------------------------------------
GFRY01095554 --------------------------------------------------------------------------------------------------------------------------------------------------------------------------------------------------------
GFRY01095552 --------------------------------------------------------------------------------------------------------------------------------------------------------------------------------------------------------
 Consensus   ........................................................................................................................................................................................................

             1001                                                                                                                                                                                                   1200
NMRB01002705 CAGCCACAAGGATGGACCAGTACCATCGGGATGTCCTTGATCATGTCTGCCGGATATGTGGTGATGAACTCCAAGCTAGAGCAGTAAAGCGACAGAAGAAGTTTTATGAGGTGAAAGGGGCAGCTGAAACCATTGAAGTTCATTACGGTATCTCTGTCAGGGATGAAGATGAACACCAGTTTCCAGGTTATGCTTGCAAC
GFRY01095553 CAGCCACAAGGATGGACCAGTACCATCGGGATGTCCTTGATCATGTCTGCCGGATATGTGGTGATGAACTCCAAGCTAGAGCAGTAAAGCGACAGAAGAAGTTTTATGAGGTGAAAGGGGCAGCTGAAACCATTGAAGTTCATTACGGTATCTCTGTCAGGGATGAAGATGAACACCAGTTTCCAGGTTATGCTTGCAAC
GFRY01095554 CAGCCACAAGGATGGACCAGTACCATCGGGATGTCCTTGATCATGTCTGCCGGATATGTGGTGATGAACTCCAAGCTAGAGCAGTAAAGCGACAGAAGAAGTTTTATGAGGTGAAAGGGGCAGCTGAAACCATTGAAGTTCATTACGGTATCTCTGTCAGGGATGAAGATGAACACCAGTTTCCAGGTTATGCTTGCAAC
GFRY01095552 CAGCCACAAGGATGGACCAGTACCATCGGGATGTCCTTGATCATGTCTGCCGGATATGTGGTGATGAACTCCAAGCTAGAGCAGTAAAGCGACAGAAGAAGTTTTATGAGGTGAAAGGGGCAGCTGAAACCATTGAAGTTCATTACGGTATCTCTGTCAGGGATGAAGATGAACACCAGTTTCCAGGTTATGCTTGCAAC
 Consensus   CAGCCACAAGGATGGACCAGTACCATCGGGATGTCCTTGATCATGTCTGCCGGATATGTGGTGATGAACTCCAAGCTAGAGCAGTAAAGCGACAGAAGAAGTTTTATGAGGTGAAAGGGGCAGCTGAAACCATTGAAGTTCATTACGGTATCTCTGTCAGGGATGAAGATGAACACCAGTTTCCAGGTTATGCTTGCAAC

             1201                                                                                                                                                                                                   1400
NMRB01002705 AGGTGCAGAAATAAGCTTGACAGACTGAAAAATGTGAAGAGTCCAAAAACTTAACTGTGTTTCAGTTCAGCCCTCATGCAGATGAAGACTGTTTTTGTATTGGTGTTGGTTCTAAACTTGGGCTGGCAGTGCCTCAGGAGCCAGTCCCTGTAGATCCTGTGACTATCACTGTGGACCATGCCTATCAAAGTTCAGGGTC
GFRY01095553 AGGTGCAGAAATAAGCTTGACAGACTGAAAAATGTGA--GAGTCCAAAAACTTAACTGTGTTTCAGTTCAGCCCTCATGCAGATGAAGACTGTTTTTGTATTGGTGTTGGTTCTAAACTTGGGCTGGCAGTGCCTCAGGAGCCAGTCCCTGTAGATCCTGTGACTATCACTGTGGACCATGCCTATCAAAGTTCAGGGTC
GFRY01095554 AGGTGCAGAAATAAGCTTGACAGACTGAAAAATGTGA--GAGTCCAAAAACTTAACTGTGTTTCAGTTCAGCCCTCATGCAGATGAAGACTGTTTTTGTATTGGTGTTGGTTCTAAACTTGGGCTGGCAGTGCCTCAGGAGCCAGTCCCTGTAGATCCTGTGACTATCACTGTGGACCATGCCTATCAAAGTTCAGGGTC
GFRY01095552 AGGTGCAGAAATAAGCTTGACAGACTGAAAAATGTGA--GAGTCCAAAAACTTAACTGTGTTTCAGTTCAGCCCTCATGCAGATGAAGACTGTTTTTGTATTGGTGTTGGTTCTAAACTTGGGCTGGCAGTGCCTCAGGAGCCAGTCCCTGTAGATCCTGTGACTATCACTGTGGACCATGCCTATCAAAGTTCAGGGTC
 Consensus   AGGTGCAGAAATAAGCTTGACAGACTGAAAAATGTGA..GAGTCCAAAAACTTAACTGTGTTTCAGTTCAGCCCTCATGCAGATGAAGACTGTTTTTGTATTGGTGTTGGTTCTAAACTTGGGCTGGCAGTGCCTCAGGAGCCAGTCCCTGTAGATCCTGTGACTATCACTGTGGACCATGCCTATCAAAGTTCAGGGTC

             1401                                                                                                                                                                                                   1600
NMRB01002705 CCCTTCATGTAGTAAACCAGTTATTGAGCCTCGAAAAAAAATACCCTTAAATCAGGCAAACCGTCAGTATGTTAAACGCCAGAGACTCAAGGGCATGATTTCAGGAGTTGACCAGTTTTGTGAAGAACACAAGGAGGATACTGTGGATGCCCTTTACTTTGTACTTATTCAAAAACTGTCGGACACAGGACGTAAGCAGG
GFRY01095553 CCCTTCATGTAGTAAACCAGTTATTGAGCCTCGAAAAAAAATACCCTTAAATCAGGCAAACCGTCAGTATGTTAAACGCCAGAGACTCAAGGGCATGATTTCAGGAGTTGACCAGTTTTGTGAAGAACACAAGGAGGATACTGTGGATGCCCTTTACTTTGTACTTATTCAAAAACTGTCGGACACAGGACGTAAGCAGG
GFRY01095554 CCCTTCATGTAGTAAACCAGTTATTGAGCCTCGAAAAAAAATACCCTTAAATCAGGCAAACCGTCAGTATGTTAAACGCCAGAGACTCAAGGGCATGATTTCAGGAGTTGACCAGTTTTGTGAAGAACACAAGGAGGATACTGTGGATGCCCTTTACTTTGTACTTATTCAAAAACTGTCGGACACAGGACGTAAGCAGG
GFRY01095552 CCCTTCATGTAGTAAACCAGTTATTGAGCCTCGAAAAAAAATACCCTTAAATCAGGCAAACCGTCAGTATGTTAAACGCCAGAGACTCAAGGGCATGATTTCAGGAGTTGACCAGTTTTGTGAAGAACACAAGGAGGATACTGTGGATGCCCTTTACTTTGTACTTATTCAAAAACTGTCGGACACAGGACGTAAGCAGG
 Consensus   CCCTTCATGTAGTAAACCAGTTATTGAGCCTCGAAAAAAAATACCCTTAAATCAGGCAAACCGTCAGTATGTTAAACGCCAGAGACTCAAGGGCATGATTTCAGGAGTTGACCAGTTTTGTGAAGAACACAAGGAGGATACTGTGGATGCCCTTTACTTTGTACTTATTCAAAAACTGTCGGACACAGGACGTAAGCAGG

             1601                                                                                                                                                                                                   1800
NMRB01002705 AAGCTGATAGAGTTCGTTCAATGTGGAATGGCCGTGATGAGGTGGAGTTATCAGTACCAGAATGCTTGGGTATGCGTGTAAGGAGTCTTATGTCTAAAGCTCATTACAAGCAGACCTATTATGGCAAGCCGTTTGTACATTTATTAGAGAAACCAGGTATTTCTAAGAAAAACTTGGTATTTCGATCGAAAAACCATGTA
GFRY01095553 AAGCTGATAGAGTTCGTTCAATGTGGAATGGCCGTGATGAGGTGGAGTTATCAGTACCAGAATGCTTGGGTATGCGTGTAAGGAGTCTTATGTCTAAAGCTCATTACAAGCAGACCTAT---------------------------------------------------------------------------------
GFRY01095554 AAGCTGATAGAGTTCGTTCAATGTGGAATGGCCGTGATGAGG--------------------------------------------------------------------------------------------------------------------------------------------------------------
GFRY01095552 AAGCTGATAGAGTTCGTTCAATGTGGAATGGCCGTGATGAGGTGGAGTTATCAGTACCAGAATGCTTGGGTATGCGTGTAAGGAGTCTTATGTCTAAAGCTCATTACAAGCAGACCTAT---------------------------------------------------------------------------------
 Consensus   AAGCTGATAGAGTTCGTTCAATGTGGAATGGCCGTGATGAGGtggagttatcagtaccagaatgcttgggtatgcgtgtaaggagtcttatgtctaaagctcattacaagcagacctat.................................................................................

             1801                                                                                                                                                                                                   2000
NMRB01002705 TTACGCCGAAAAACATGGTTTTACGGCGAGTTACTTGGTATTTCGACAGAAATACCTTGTTTTTCGCGTAAAACATGGTATTTCGATGAAAAACTAGGTAATTCAGGAGAGAAACAAGGTTTTACGGCCGTATCTTGATAAAACCTGGTAATTCTACCGAAAAACATGGTTTTTCGCGAATTACCTAGTTTTAAGCAAGA
GFRY01095553 --------------------------------------------------------------------------------------------------------------------------------------------------------------------------------------------------------
GFRY01095554 --------------------------------------------------------------------------------------------------------------------------------------------------------------------------------------------------------
GFRY01095552 --------------------------------------------------------------------------------------------------------------------------------------------------------------------------------------------------------
 Consensus   ........................................................................................................................................................................................................

             2001                                                                                                                                                                                                   2200
NMRB01002705 AAGTTGGAGAAAAACAAGGTATTTCGAGAGAAACTTGGTTTTTCGAGAATTACCAAGTAATTCGACTTTCGCTGGGTTTCCCCAGTCGACCTCGTTCCCTGGTGCGGCAGGTTAGGAAACCACCTGAGGCCGGAGTGACAGAAATTGAGCTCAAAAACTTCATAGGGCCCTTTTCTGTTGTATTTGACGAGGTCAACCGC
GFRY01095553 --------------------------------------------------------------------------------------------------------------------------------------------------------------------------------------------------------
GFRY01095554 --------------------------------------------------------------------------------------------------------------------------------------------------------------------------------------------------------
GFRY01095552 --------------------------------------------------------------------------------------------------------------------------------------------------------------------------------------------------------
 Consensus   ........................................................................................................................................................................................................

             2201                                                                                                                                                                                                   2400
NMRB01002705 TACTGTAATAGGCCTACATAAACATGACAAAGCTAGGTATTCTGTCATCTGAGATAATACATGATTGTGCACGAGAACAAAAACGGTCTGCTATAGCTCACAGGCCTAGGCCTGGGCCTACATACATGTAGATAGATAAGCAAACATACTTTTCAAGGATGCTGCTGCGACGACTCGTTCTTGATTGAATGTAGGTCTTT
GFRY01095553 --------------------------------------------------------------------------------------------------------------------------------------------------------------------------------------------------------
GFRY01095554 --------------------------------------------------------------------------------------------------------------------------------------------------------------------------------------------------------
GFRY01095552 --------------------------------------------------------------------------------------------------------------------------------------------------------------------------------------------------------
 Consensus   ........................................................................................................................................................................................................

             2401                                                                                                                                                                                                   2600
NMRB01002705 GGCCAGTCAATGCTATTAAACAAAAGATGCGTGTTTGGATGTCTTGGATGGCTTGTATCGCTGCTGAGGATGTTACGCCTACTTAGCAGCTAAGCGGATGAAATTGGAATTATCTGATTATGTGTGTCAGTAGGTTCGTTTCGTTCGTTTCATTGTTTTATTTCATTGTTTTATAATCATCCAGTCGTAGTTGAAAACAA
GFRY01095553 --------------------------------------------------------------------------------------------------------------------------------------------------------------------------------------------------------
GFRY01095554 --------------------------------------------------------------------------------------------------------------------------------------------------------------------------------------------------------
GFRY01095552 --------------------------------------------------------------------------------------------------------------------------------------------------------------------------------------------------------
 Consensus   ........................................................................................................................................................................................................

             2601                                                                                                                                                                                                   2800
NMRB01002705 GACCAGGGAACGAGGTCGACTGGGGAAACCCAGCGAAAGTCGAATTACTTGGTAATTCTCGAAAAACCAAGTTTCTCTCGAAATACCTTGTTTTTCTCCAACTTTCTTGCTTAAAACTAGGTAATTCGCGAAAAACCATGTTTTTCGGTAGAATTACCAGGTTTTATCAAGATACGGCCGTAAAACCTTGTTTCTCTCCT
GFRY01095553 --------------------------------------------------------------------------------------------------------------------------------------------------------------------------------------------------------
GFRY01095554 --------------------------------------------------------------------------------------------------------------------------------------------------------------------------------------------------------
GFRY01095552 --------------------------------------------------------------------------------------------------------------------------------------------------------------------------------------------------------
 Consensus   ........................................................................................................................................................................................................

             2801                                                                                                                                                                                                   3000
NMRB01002705 GAATTACCTAGTTCTTCATCGAAATACCATGTTTTACGCGAAAAACAAGGTATTTCTGTCGAAATACCAAGTAACTCACCGTAAAACCATGTTTTTCGGCGTAATACATGGTTTTTCGATCGAAATACCAAGTTTTTCGTAGAAATACCTGGTTTCTTTAATAAATGTACAAACGGCTTGCCATAACCTATGGCATTCAA
GFRY01095553 -----------------------------------------------------------------------------------------------------------------------------------------------------------------------------------------------GGCATTCAA
GFRY01095554 --------------------------------------------------------------------------------------------------------------------------------------------------------------------------------------------------------
GFRY01095552 -----------------------------------------------------------------------------------------------------------------------------------------------------------------------------------------------GGCATTCAA
 Consensus   ...............................................................................................................................................................................................ggcattcaa

             3001                                                                                                                                                                                                   3200
NMRB01002705 AGAGATAAGACCTCTGCCAAGTCACTGCAACCACGAAACCAGCTCACAGTCCTGGAACCATCATTTCTTCCAGGACATTGCAAGTACAGACTGTTAAAGGATTCCGTTTTGCTGGCTGAACAGGATTTACTCAGTGCACAAGACAGTCAGCCCCTTGATATCATGAGGGACTATCATTCATTCCCTGCTGACTTTCCATT
GFRY01095553 AGAGATAAGACCTCTGCCAAGTCACTGCAACCACCAAACCAGCTCACAGTCCTGGAACCATCATTTCTTCCAGGACATTGCAAGTACAGACTGTTAAAGGATTCCGTTTTGCTGGCTGAACAGGATTTACTCAGTGCACAAGACAGTCAGCCCCTTGATATCATGAGGGACTATCATTCATTCCCTGCTGACTTTCCATT
```

```
GFRY01095554 --------------------------------------------------------------------------ACATTGCAAGTACAGACTGTTAAAGGATTCCGTTTTGCTGGCTGAACAGGATTTACTCAGTGCACAAGACAGTCAGCCCCTTGATATCATGAGGGACTATCATTCATTCCCTGCTGACTTTCCATT
GFRY01095552 AGAGATAAGACCTCTGCCAAGTCACTGCAACCACCAAACCAGCTCACAGTCCTGGAACCATCATTTCTTCCAGGACATTGCAAGTACAGACTGTTAAAGGATTCCGTTTTGCTGGCTGAACAGGATTTACTCAGTGCACAAGACAGTCAGCCCCTTGATATCATGAGGGACTATCATTCATTCCCTGCTGACTTTCCATT
 Consensus   agagataagacctctgccaagtcactgcaaccaccaaaccagctcacagtcctggaaccatcatttcttccaggACATTGCAAGTACAGACTGTTAAAGGATTCCGTTTTGCTGGCTGAACAGGATTTACTCAGTGCACAAGACAGTCAGCCCCTTGATATCATGAGGGACTATCATTCATTCCCTGCTGACTTTCCATT

             3201                                                                                                                                                                                                       3400
NMRB01002705 ACCAAATGTAATGGGAGTTGAACTTGATTACATTTCTGCTGTTGCAAAAACGCTTCTAGAAATAGACAATGACGTGTCAAAGAGACTGGATGCGTTGGGATTGCCTCAGTCAACAAAACTTTGTACTGTCATCAAAGATGGCGGTGATGGACTGGGGGATGTAAATGTTAAGAGTAGAAAGGCTGATTTTGCCCTTACTG
GFRY01095553 ACCAAATGTAATGGGAGTTGAACTTGATTACATTTCTGCTGTTGCAAAAACGCTTCTAGAAATAGACAATGACGTGTCAAAGAGACTGGATGCGTTGGGATTGCCTCAGTCAACAAAACTTTGTACTGTCATCAAAGATGGCGGTGATGGACTGGGGGATGTAAATGTTAAGAGTAGAAAGGCTGATTTTGCCCTTACTG
GFRY01095554 ACCAAATGTAATGGGAGTTGAACTTGATTACATTTCTGCTGTTGCAAAAACGCTTCTAGAAATAGACAATGACGTGTCAAAGAGACTGGATGCGTTGGGATTGCCTCAGTCAACAAAACTTTGTACTGTCATCAAAGATGGCGGTGATGGACTGGGGGATGTAAATGTTAAGAGTAGAAAGGCTGATTTTGCCCTTACTG
GFRY01095552 ACCAAATGTAATGGGAGTTGAACTTGATTACATTTCTGCTGTTGCAAAAACGCTTCTAGAAATAGACAATGACGTGTCAAAGAGACTGGATGCGTTGGGATTGCCTCAGTCAACAAAACTTTGTACTGTCATCAAAGATGGCGGTGATGGACTGGGGGATGTAAATGTTAAGAGTAGAAAGGCTGATTTTGCCCTTACTG
 Consensus   ACCAAATGTAATGGGAGTTGAACTTGATTACATTTCTGCTGTTGCAAAAACGCTTCTAGAAATAGACAATGACGTGTCAAAGAGACTGGATGCGTTGGGATTGCCTCAGTCAACAAAACTTTGTACTGTCATCAAAGATGGCGGTGATGGACTGGGGGATGTAAATGTTAAGAGTAGAAAGGCTGATTTTGCCCTTACTG

             3401                                                                                                                                                                                                       3600
NMRB01002705 ACAAGGTTTTCCGATATTCTTTCTGTGTTTTGCAATGTGCAGCAAGTGATGCACATGGTGAAAAACATACCATTTTCAAAGAGGAATTTCCCAACTCTGTTGACTGTACTCGTCCGGTTGTGGTTTGTAACTGTGATGAGAGTGACAGGCCCAGTGTGGCAATAATCTTGTCTCATGTCTCTAAAGCCAGAAAACAGATG
GFRY01095553 ACAAAGTTTTCCGATATTCTTTCTGTGTTTTGCAATGTGCAGCAAGTGATGCACATGGTGAAAAACATACCATTTTCAAAGAGGAATTTCCCAACTCTGTTGACTGTACTCGTCCGGTTGTGGTTTGTAACTGTGATGAGAGTGACAGGCCCAGTGTGGCAATAATCTTGTCTCATGTCTCTAAAGCCAGAAAACAGATG
GFRY01095554 ACAAAGTTTTCCGATATTCTTTCTGTGTTTTGCAATGTGCAGCAAGTGATGCACATGGTGAAAAACATACCATTTTCAAAGAGGAATTTCCCAACTCTGTTGACTGTACTCGTCCGGTTGTGGTTTGTAACTGTGATGAGAGTGACAGGCCCAGTGTGGCAATAATCTTGTCTCATGTCTCTAAAGCCAGAAAACAGATG
GFRY01095552 ACAAAGTTTTCCGATATTCTTTCTGTGTTTTGCAATGTGCAGCAAGTGATGCACATGGTGAAAAACATACCATTTTCAAAGAGGAATTTCCCAACTCTGTTGACTGTACTCGTCCGGTTGTGGTTTGTAACTGTGATGAGAGTGACAGGCCCAGTGTGGCAATAATCTTGTCTCATGTCTCTAAAGCCAGAAAACAGATG
 Consensus   ACAAaGTTTTCCGATATTCTTTCTGTGTTTTGCAATGTGCAGCAAGTGATGCACATGGTGAAAAACATACCATTTTCAAAGAGGAATTTCCCAACTCTGTTGACTGTACTCGTCCGGTTGTGGTTTGTAACTGTGATGAGAGTGACAGGCCCAGTGTGGCAATAATCTTGTCTCATGTCTCTAAAGCCAGAAAACAGATG

             3601                                                                                                                                                                                                       3800
NMRB01002705 CAAGGGGCTACGCTTCAGCTTTTAGACCCATCTTCAGGCAGTGTTATTCGAGAGCATGATCTGTCCTTTTACTACACCATGGTGGACGAAAAATTCGAAAGGATTTCATCAGGTCTGCAGGGCAGTGGGTCAAGGTTCCTCTGCACCCTCTGCCATGCTACCACTGCTAGTTGCCAGGCTGATGCAGGATCTCATCATAG
GFRY01095553 CAAGGGGCTACGCTTCAGCTTTTAGACCCATCTTCAGGCAGTGTTATTCGAGAGCATGATCTGTCCTTTTACTACACCATGGTGGACGAAAAATTCGAAAGGATTTCATCAGGTCTGCAGGGCAGTGGGTCAAGGTTCCTCTGCACCCTCTGCCATGCTACCACTGCTAGTTGCCAGGCTGATGTAGGATCTCATCATAG
GFRY01095554 CAAGGGGCTACGCTTCAGCTTTTAGACCCATCTTCAGGCAGTGTTATTCGAGAGCATGATCTGTCCTTTTACTACACCATGGTGGACGAAAAATTCGAAAGGATTTCATCAGGTCTGCAGGGCAGTGGGTCAAGGTTCCTCTGCACCCTCTGCCATGCTACCACTGCTAGTTGCCAGGCTGATGTAGGATCTCATCATAG
GFRY01095552 CAAGGGGCTACGCTTCAGCTTTTAGACCCATCTTCAGGCAGTGTTATTCGAGAGCATGATCTGTCCTTTTACTACACCATGGTGGACGAAAAATTCGAAAGGATTTCATCAGGTCTGCAGGGCAGTGGGTCAAGGTTCCTCTGCACCCTCTGCCATGCTACCACTGCTAGTTGCCAGGCTGATGTAGGATCTCATCATAG
 Consensus   CAAGGGGCTACGCTTCAGCTTTTAGACCCATCTTCAGGCAGTGTTATTCGAGAGCATGATCTGTCCTTTTACTACACCATGGTGGACGAAAAATTCGAAAGGATTTCATCAGGTCTGCAGGGCAGTGGGTCAAGGTTCCTCTGCACCCTCTGCCATGCTACCACTGCTAGTTGCCAGGCTGATGtAGGATCTCATCATAG

             3801                                                                                                                                                                                                       4000
NMRB01002705 GGTTCGAACTCATGAAGAAACAGTCTCATTGTATAAATATCGACTTGAAAATCCTCAAAACTTGGGTGAAGATGAACTAGCTATACAAAGCAAAGGGGTGAAAAGTTTCCCATTGATTTCTTCAAATGCAAATGAGCGTTCTCTAGATGCCACACATGCCAATATCAATATTGGAAGGGTTTTGAAAAAGGTGGTTGTCA
GFRY01095553 GGTTCGAACTCATGAAGAAACAGTCTCATTGTATAAATATCGACTTGAAAATCCTCAAAACTTGGGTGAAGATGAACTAGCTATACAAAGCAAAGAGGTGAAAAGTTTCCCATTGATTTCTTCAAATGCAAATGAGCGTTCTCTAGATGCCACACATGCCAATATCAATATTGGAAGGGTTTTGAAAAAGGTGGTTGTCA
GFRY01095554 GGTTCGAACTCATGAAGAAACAGTCTCATTGTATAAATATCGACTTGAAAATCCTCAAAACTTGGGTGAAGATGAACTAGCTATACAAAGCAAAGAGGTGAAAAGTTTCCCATTGATTTCTTCAAATGCAAATGAGCGTTCTCTAGATGCCACACATGCCAATATCAATATTGGAAGGGTTTTGAAAAAGGTGGTTGTCA
GFRY01095552 GGTTCGAACTCATGAAGAAACAGTCTCATTGTATAAATATCGACTTGAAAATCCTCAAAACTTGGGTGAAGATGAACTAGCTATACAAAGCAAAG---------------------------------------------------------------------------------------------------------
 Consensus   GGTTCGAACTCATGAAGAAACAGTCTCATTGTATAAATATCGACTTGAAAATCCTCAAAACTTGGGTGAAGATGAACTAGCTATACAAAGCAAAGaggtgaaaagtttcccattgatttcttcaaatgcaaatgagcgttctctagatgccacacatgccaatatcaatattggaagggttttgaaaaaggtggttgtca

             4001                                                                                                                                                                                                       4200
NMRB01002705 GGGAAATTGCTGAAATTCATGAATGGGCAGAAAATGATAACAATAAAACCAGTCTTCAAAATGCAGAGAAGGAGTTTGATGCAAAAATCATGAAGGAAATTGGGCTTCAACCCAAAATGATGATGGCTGGAAATTATGCGAGGGCTGTCTTTGCTGTTGAAAATCTTGGGGTTGTAACCAGTTTGATCAAAAATGATGAA
GFRY01095553 GGGAAATTGCTGAAATTCATGAATGGGCAGAAAATGATAACAATAAAACCAGTCTTCAAAATGCAGAGAAGGAGTTTGATGCAAAAATCATGAAGGAAATTGGGCTTCAACCCAAAATGATGATGCCTGGAAATTATGCGAGGGCTGTCTTTGCTGTTGAAAATCTTGGGGTTGTAACCAGTTTGATCAAAAATGATGAA
GFRY01095554 GGGAAATTGCTGAAATTCATGAATGGGCAGAAAATGATAACAATAAAACCAGTCTTCAAAATGCAGAGAAGGAGTTTGATGCAAAAATCATGAAGGAAATTGGGCTTCAACCCAAAATGATGATGCCTGGAAATTATGCGAGGGCTGTCTTTGCTGTTGAAAATCTTGGGGTTGTAACCAGTTTGATCAAAAATGATGAA
GFRY01095552 --------------------------------------------------------------------------------------------------------------------------------------------------------------------------------------------------------
 Consensus   gggaaattgctgaaattcatgaatgggcagaaaatgataacaataaaaccagtcttcaaaatgcagagaaggagtttgatgcaaaaatcatgaaggaaattgggcttcaacccaaaatgatgatgcctggaaattatgcgagggctgtctttgctgttgaaaatcttggggttgtaaccagtttgatcaaaaatgatgaa

             4201                                                                                                                                                                                                       4400
NMRB01002705 AGAAGGGAGCATGTCTCTACTTTGTTCAACATTTTTAATAGATTGCACAGAGTTTACAGCAGTAACACCCCTTTAACTTCAGTTCCAGATGAGACTGCAGCATACAGAGAAAATGCCCTCCAGTTTGCCTTCCATCTTGGGGAAAACTTTCCATACGTTCCATGGTCCAATTATATTCACAGGGTCGTGGAGCATGTAGA
GFRY01095553 AGAAGGGAGCATGTCTCTACTTTGTTCAACATTTTTAATAGATTGCACAGAGTTTACAGCAGTAACACCCCTTTAACTTCAGTTCCAGATGAGACTGCAGCATACAGAGAAAATGCCCTCCAGTTTGCCTTCCATCTTGGGGAAAACTTTCCATACGTTCCATGGTCCAATTATATTCACAGGGTCGTGGAGCATGTAGA
GFRY01095554 AGAAGGGAGCATGTCTCTACTTTGTTCAACATTTTTAATAGATTGCACAGAGTTTACAGCAGTAACACCCCTTTAACTTCAGTTCCAGATGAGACTGCAGCATACAGAGAAAATGCCCTCCAGTTTGCCTTCCATCTTGGGGAAAACTTTCCATACGTTCCATGGTCCAATTATATTCACAGGGTCGTGGAGCATGTAGA
GFRY01095552 --------------------------------------------------------------------------------AGTTCCAGATGAGACTGCAGCATACAGAGAAAATGCCCTCCAGTTTGCCTTCCATCTTGGGGAAAACTTTCCATACGTTCCATGGTCCAATTATATTCACAGGGTCGTGGAGCATGTAGA
 Consensus   agaagggagcatgtctctactttgttcaacatttttaatagattgcacagagtttacagcagtaacacccctttaacttcAGTTCCAGATGAGACTGCAGCATACAGAGAAAATGCCCTCCAGTTTGCCTTCCATCTTGGGGAAAACTTTCCATACGTTCCATGGTCCAATTATATTCACAGGGTCGTGGAGCATGTAGA

             4401                                                                                                                                                                                                       4600
NMRB01002705 TGAAATATTACATGATCCATCAGGTGTGCAAAGTGTTGGACTTCTCTCAAGTGAGGGCGGTGAATCCGGGAATAAACTGTACCGCTTTATCAGAAAGTCATTGAGTAGGCAGGATAGTGCTTTTGACCTTCGAGATGCCCTTGTGTTTCATTGGCTGTACACAAGTAAAGTTCTGCAAAAACATGCAGCCACTGAAAAGG
GFRY01095553 TGAAATATTACATGATCCATCAGGTGTGCAAAGTGTTGGACTTCTCTCAAGTGAGGGCGGTGAATCCGGGAATAAACTGTACCGCTTTATCAGAAAGTCATTGAGTAGGCAGGATAGTGCTTTTGACCTTCGAGATGCCCTTGTGTTTCATTGGCTGTACACAAGTAAAGTTCTGCAAAAACATGCAGCCACTGAAAAGG
GFRY01095554 TGAAATATTACATGATCCATCAGGTGTGCAAAGTGTTGGACTTCTCTCAAGTGAGGGCGGTGAATCCGGGAATAAACTGTACCGCTTTATCAGAAAGTCATTGAGTAGGCAGGATAGTGCTTTTGACCTTCGAGATGCCCTTGTGTTTCATTGGCTGTACACAAGTAAAGTTCTGCAAAAACATGCAGCCACTGAAAAGG
GFRY01095552 TGAAATATTACATGATCCATCAGGTGTGCAAAGTGTTGGACTTCTCTCAAGTGAGGGCGGTGAATCCGGGAATAAACTGTACCGCTTTATCAGAAAGTCATTGAGTAGGCAGGATAGTGCTTTTGACCTTCGAGATGCCCTTGTGTTTCATTGGCTGTACACAAGTAAAGTTCTGCAAAAACATGCAGCCACTGAAAAGG
 Consensus   TGAAATATTACATGATCCATCAGGTGTGCAAAGTGTTGGACTTCTCTCAAGTGAGGGCGGTGAATCCGGGAATAAACTGTACCGCTTTATCAGAAAGTCATTGAGTAGGCAGGATAGTGCTTTTGACCTTCGAGATGCCCTTGTGTTTCATTGGCTGTACACAAGTAAAGTTCTGCAAAAACATGCAGCCACTGAAAAGG

             4601                                                                                                                                                                                                       4800
NMRB01002705 TCAGTAAGACTTGTACAATATGTGGAATGGCAGATCACAATCGGCGTACATGCGCATATAAGCAAGATGCAGCCCCAGAAGATGACATGATGGAAGAAAGTTAAACAATATACATGAAGTATTCAAATTGAATTTCACTTCTTCTTCTCTGTTTCTTTTTGCCTTTGGTTTGTTTTGGCCTACAGTCAACACACACAAAA
GFRY01095553 TCAGTAAGACTTGTACAATATGTGGAATGGCAGATCACAATCGGCGTACATGCGCATATAAGCAAGATGCAGCCCCAGAAGATGACATGATGGAAGAAAGTTAAACAATATACATGAAGTATTCAAATTGAAAAAAA
GFRY01095554 TCAGTAAGACTTGTACAATATGTGGAATGGCAGATCACAATCGGCGTACATGCGCATATAAGCAAGATGCAGCCCCAGAAGATGACATGATGGAAGAAAGTTAAACAATATACATGAAGTATTCAAATTGAAAAAAA
GFRY01095552 TCAGTAAGACTTGTACAATATGTGGAATGGCAGATCACAATCGGCGTACATGCGCATATAAGCAAGATGCAGCCCCAGAAGATGACATGATGGAAGAAAGTTAAACAATATACATGAAGTATTCAAATTGAAAAAAA
 Consensus   TCAGTAAGACTTGTACAATATGTGGAATGGCAGATCACAATCGGCGTACATGCGCATATAAGCAAGATGCAGCCCCAGAAGATGACATGATGGAAGAAAGTTAAACAATATACATGAAGTATTCAAATTGAAAaaaaA.................................................................

             4801                                                                                                                                                                                                       5000
NMRB01002705 GACTCGGTCTCTGATCTCCCATTTTTTGAACCAGCACAGCATGAGTGATACCATTTGTCACAAGTCTCACACTGTAGCGACTCGATGATTTCTCCTGGTGAAAAATTGTCAGTAACAATGCATTTATCAGATTGACACCTGCCTGGCTCAAGTCTCTTTGTTGTTAATGTGAAAAGGTGCTGATTGGCACCACCATGAAA
GFRY01095553
GFRY01095554
GFRY01095552
 Consensus   ........................................................................................................................................................................................................
```

>TSA: GFRY01095552.1:beg-end

5'3' Frame 3

GKLCALLAIWCANNRATRK-KPD-NRCNIADFLQNYKVCSLESGVHL-FG-SGLKVQPQGWTSTIGMSLIMSAGYVVMNSKLEQ-SDRRSFMR-KGQLKPLKFITVSLSGMKMNTSFQVMLATGAEISLTD-KM-ESKNLTVFQFSPHADEDCFCIGVGSKLGLAVPQEPVPVDPVTITVDHAYQSSGSPSCSKPVIEPRKKIPLNQANRQYVKRQRLK
GMISGVDQFCEEHKEDTVDALYFVLIQKLSDTGRKQEADRVRSMWNGRDEVELSVPECLGMRVRSLMSKAHYKQTYGIQRDKTSAKSLQPPNQLTVLEPSFLPGHCKYRLLKDSVLLAEQDLLSAQDSQPLDIMRDYHSFPADFPLPNVMGVELDYISAVAKTLLEIDNDVSKRLDALGLPQSTKLCTVIKDGGDGLGDVNVKSRKADFALTDKVFRYS
FCVLQCAASDAHGEKHTIFKEEFPNSVDCTRPVVVCNCDESDRPSVAIILSHVSKARKQMQGATLQLLDPSSGSVIREHDLSFYYTMVDEKFERISSGLQGSGSRFLCTLCHATTASCQADVGSHHRVRTHEETVSLYKYRLENPQNLGEDELAIQSKEFQMRLQHTEKMPSSLPSILGKTFHTFHGPIIFTGSWSM-MKYYMIHQVCKVLDFSQVRAV
NPGINCTALSESH-VGRIVLLTFEMPLCFIGCTQVKFCKNMQPLKRSVRLVQYVEWQITIGVHAHISKMQPQKMT-WKKVKQYT-SIQIEK

>TSA: GFRY01095553.1:beg-end

5'3' Frame 3

GKLCALLAIWCANNRATRK-KPD-NRCNIADFLQNYKVCSLESGVHL-FG-SGLKVQPQGWTSTIGMSLIMSAGYVVMNSKLEQ-SDRRSFMR-KGQLKPLKFITVSLSGMKMNTSFQVMLATGAEISLTD-KM-ESKNLTVFQFSPHADEDCFCIGVGSKLGLAVPQEPVPVDPVTITVDHAYQSSGSPSCSKPVIEPRKKIPLNQANRQYVKRQRLK
GMISGVDQFCEEHKEDTVDALYFVLIQKLSDTGRKQEADRVRSMWNGRDEVELSVPECLGMRVRSLMSKAHYKQTYGIQRDKTSAKSLQPPNQLTVLEPSFLPGHCKYRLLKDSVLLAEQDLLSAQDSQPLDIMRDYHSFPADFPLPNVMGVELDYISAVAKTLLEIDNDVSKRLDALGLPQSTKLCTVIKDGGDGLGDVNVKSRKADFALTDKVFRYS
FCVLQCAASDAHGEKHTIFKEEFPNSVDCTRPVVVCNCDESDRPSVAIILSHVSKARKQMQGATLQLLDPSSGSVIREHDLSFYYTMVDEKFERISSGLQGSGSRFLCTLCHATTASCQADVGSHHRVRTHEETVSLYKYRLENPQNLGEDELAIQSKEVKSFPLISSNANERSLDATHANINIGRVLKKVVVREIAEIHEWAENDNNKTSLQNAEKEF
DAKIMKEIGLQPKMMMPGNYARAVFAVENLGVVTSLIKNDERREHVSTLFNIFNRLHRVYSSNTPLTSVPDETAAYRENALQFAFHLGENFPYVPWSNYIHRVVEHVDEILHDPSGVQSVGLLSSEGGESGNKLYRFIRKSLSRQDSAFDLRDALVFHWLYTSKVLQKHAATEKVSKTCTICGMADHNRRTCAYKQDAAPEDDMMEES-TIYMKYSN-K
K

>TSA: GFRY01095554.1:beg-end

5'3' Frame 2

RKAMRTARNLVCKQQSNEEVKTRLKSMQYSRFSAEL-SLFVRIWSTLVIWIVWIKGTATRMDQYHRDVLDHVCRICGDELQARAVKRQKKFYEVKGAAETIEVHYGISVRDEDEHQFPGYACNRCRNKLDRLKNVRVQKLNCVSVQPSCR-RLFLYWCWF-TWAGSASGASPCRSCDYHCGPCLSKFRVPFM--TSY-ASKKNTLKSGKPSVC-TPETQ
GHDFRS-PVL-RTQGGYCGCPLLCTYSKTVGHRT-AGS--SSFNVEWP--GHCKYRLLKDSVLLAEQDLLSAQDSQPLDIMRDYHSFPADFPLPNVMGVELDYISAVAKTLLEIDNDVSKRLDALGLPQSTKLCTVIKDGGDGLGDVNVKSRKADFALTDKVFRYSFCVLQCAASDAHGEKHTIFKEEFPNSVDCTRPVVVCNCDESDRPSVAIILSHV
SKARKQMQGATLQLLDPSSGSVIREHDLSFYYTMVDEKFERISSGLQGSGSRFLCTLCHATTASCQADVGSHHRVRTHEETVSLYKYRLENPQNLGEDELAIQSKEVKSFPLISSNANERSLDATHANINIGRVLKKVVVREIAEIHEWAENDNNKTSLQNAEKEFDAKIMKEIGLQPKMMMPGNYARAVFAVENLGVVTSLIKNDERREHVSTLFNIF
NRLHRVYSSNTPLTSVPDETAAYRENALQFAFHLGENFPYVPWSNYIHRVVEHVDEILHDPSGVQSVGLLSSEGGESGNKLYRFIRKSLSRQDSAFDLRDALVFHWLYTSKVLQKHAATEKVSKTCTICGMADHNRRTCAYKQDAAPEDDMMEES-TIYMKYSN-KK

Notospermus geniculatus (Nge)
WGS: NMRB01002182.1

66356-69356
Notospermus geniculatus strain Ushimado scaffold2182, whole genome shotgun sequence

```
      N K K K Q R S E E S L L H I K S R I C N F M K R V Q I Q C F N T F Q I S D I I S F P C Q I N I Q N C M L Q L S K G L F I F I N * G I   F1
     G T K K N K E V K N L C C I S N Q G Y V I S * K E F K Y N V L I H F R F Q I L F H S L V K S T Y R I V C Y N C Q R G C S F S S I E E S   F2
      E Q K K T K K * R I F A A Y Q I K D M * F H E K S S N T M F * Y I S D F R Y Y F I P L S N Q H T E L Y V T T V K G A V H F H Q L R N L  F3
   1 GGAACAAAAAAAAACAAAGAAGTGAAGAATCTTTGCTGCATATCAAATCAAGGATATGTAATTTCATGAAAAGAGTTCAAATACAATGTTTTAATACATTTCAGATTTCAGATATTATTTCATTCCCTTGTCAAATCAACATACAGAATTGTATGTTACAACTGTCAAAGGGGCTGTTCATTTTCATCAATTGAGGAATC  200
     ----:----|----:----|----:----|----:----|----:----|----:----|----:----|----:----|----:----|----:----|----:----|----:----|----:----|----:----|----:----|----:----|----:----|----:----|----:----|----:----|
   1 CCTTGTTTTTTTTTGTTTCTTCACTTCTTAGAAACGACGTATAGTTTAGTTCCTATACATTAAAGTACTTTTCTCAAGTTTATGTTACAAAATTATGTAAAGTCTAAAGTCTATAATAAAGTAAGGGAACAGTTTAGTTGTATGTCTTAACATACAATGTTGACAGTTTCCCCGACAAGTAAAAGTAGTTAACTCCTTAG  200
     L F L F F C L L S S D K S C I L D L I H L K M F L T * I C H K L V N * I E S I I E N G Q * I L M C F Q I N C S D F P S N M K M L Q P I   F6
      S C F F V F F H L I K A A Y * I L S I Y N * S F L E F V I N * Y M E S K L Y * K M G K D F * C V S N Y T V V T L P A T * K * * N L F  F5
      P V F F F L S T F F R Q M D F * P Y T I E H F S N L Y L T K I C K L N * I N N * E R T L D V Y L I T H * L Q * L P Q E N E D I S S D  F4

      * G V I L E K M E K A M K L Q I A A I I L Y S T T N N P C N L Q V H S I F R F P K D H N S D S S S D E N E Q P L Y E A E D S * F C H F  F1
     E V * F L R K W R K L * S C K L Q R L F C T A Q Q I I P A I C K C T A F S A F P R I T T Q I P Q V M K M N S P F M K L K I L N F V I L   F2
      R C D S * E N G E S Y E A A N C S D Y S V Q H N K * S L Q F A S A Q H F P L S Q G S Q L R F L K * * K * T A P L * S * R F L I L S F  F3
 201 TGAGGTGTGATTCTTGAGAAAATGGAGAAAGCTATGAAGCTGCAAATTGCAGCGATTATTCTGTACAGCACAACAAATAATCCCTGCAATTTGCAAGTGCACAGCATTTTCCGCTTTCCCAAGGATCACAACTCAGATTCCTCAAGTGATGAAAATGAACAGCCCCTTTATGAAGCTGAAGATTCTTAATTTTGTCATTT  400
     ----:----|----:----|----:----|----:----|----:----|----:----|----:----|----:----|----:----|----:----|----:----|----:----|----:----|----:----|----:----|----:----|----:----|----:----|----:----|----:----|
 201 ACTCCACACTAAGAACTCTTTTACCTCTTTCGATACTTCGACGTTTAACGTCGCTAATAAGACATGTCGTGTTGTTTATTAGGGACGTTAAACGTTCACGTGTCGTAAAAGGCGAAAGGGTTCCTAGTGTTGAGTCTAAGGAGTTCACTACTTTTACTTGTCGGGGAAATACTTCGACTTCTAAGAATTAAAACAGTAAA  400
     Q P T I R S F I S F A I F S C I A A I I R Y L V V F L G Q L K C T C L M K R K G L S * L E S E E L S S F S C G R * S A S S E * N Q * K   F6
      R L H S E Q S F P S L * S A A F Q L S * E T C C L L Y D R C N A L A C C K G S E W P D C S L N R L H H F H V A G K H L Q L N K I K D N  F5
      S T H N K L F H L F S H L Q L N C R N N Q V A C C I I G A I Q L H V A N E A K G L I V V * I G * T I F I F L G K I F S F I R L K T M  F4

      M N F W S E F M S V P R E D A C H G * V F K L * N E * S V P H G C V L * I T R K N D P * * * H L V E Y P H S Q F I L I H A L T G T A V  F1
     * I F G V N L C R F P E K T H A M D E F L N Y K M S E V S L M D V F F R S H E K T I H D N N I W L N I L I H N S F * F T L S Q V R Q   F2
      Y E F L E * I Y V G S P R R R M P W M S F * I I K * V K C P S W M C S L D H T K K R S M I I T S G * I S S F T I H S D S R S H R Y G S  F3
 401 TATGAATTTTTGGAGTGAATTTATGTCGGTTCCCCGAGAAGACGCATGCCATGGATGAGTTTTTAAATTATAAAATGAGTGAAGTGTCCCTCATGGATGTGTTCTTTAGATCACACGAAAAAACGATCCATGATAATAACATCTGGTTGAATATCCTCATTCACAATTCATTCTGATTCACGCTCTCACAGGTACGGCAG  600
     ----:----|----:----|----:----|----:----|----:----|----:----|----:----|----:----|----:----|----:----|----:----|----:----|----:----|----:----|----:----|----:----|----:----|----:----|----:----|----:----|
 401 ATACTTAAAAACCTCACTTAAATACAGCCAAGGGGCTCTTCTGCGTACGGTACCTACTCAAAAATTTAATATTTTACTCACTTCACAGGGAGTACCTACACAAGAAATCTAGTGTGCTTTTTTGCTAGGTACTATTATTGTAGACCAACTTATAGGAGTAAGTGTTAAGTAAGACTAAGTGCGAGAGTGTCCATGCCGTC  600
     I F K Q L S N I D T G R S S A H W P H T K L N Y F S H L T G * P H T R * I V R F F S G H Y Y C R T S Y G * E C N M R I * A R V P V A   F6
      * S N K S H I * T P E G L R M G H I L K * I I F H T F H G E H I H E K S * V F F R D M I I V D P Q I D E N V I * E S E R E * L Y P L  F5
      K H I K P T F K H R N G S F V C A M S S N K F * I L S T D R M S T N K L D C S F V I W S L L M Q N F I R M * L E N Q N V S E C T R C  F4

      V A S L S T A S A I F W E F L S G S K F L Q G F A G I Q P L K Y K S I S * I T I T R C V L S T * S F A N K P I K F V S C F S T F T G  F1
     L W P A C P Q P V Q Y F G N F L V A A S F C K D L L V Y N H * N T R A S L R S Q S P D V S C R L K A L R I N R * S L F P A S P P S L E   F2
      C G Q L V H S Q C N I L G I S * W Q Q V F A R I C W Y T T T E I Q E H L L D H N H Q M C P V D L K L C E * T D K V C F L L L H L H W R  F3
 601 TTGTGGCCAGCTTGTCCACAGCCAGTGCAATATTTTGGGAATTTCTTAGTGGCAGCAAGTTTTTGCAAGGATTTGCTGGTATACAACCACTGAAATACAAGAGCATCTTCTTAGATCACAATCACCAGATGTGTCCTGTCGACTTAAAGCTTTGCGAATAAACCGATAAAGTTTGTTTCCTGCTTCTCCACCTTCACTGGA  800
     ----:----|----:----|----:----|----:----|----:----|----:----|----:----|----:----|----:----|----:----|----:----|----:----|----:----|----:----|----:----|----:----|----:----|----:----|----:----|----:----|
 601 AACACCGGTCGAACAGGTGTCGGTCACGTTATAAAACCCTTAAAGAATCACCGTCGTTCAAAAACGTTCCTAAACGACCATATGTTGGTGACTTTATGTTCTCGTAGAGAATCTAGTGTTAGTGGTCTACACAGGACAGCTGAATTTCGAAACGCTTATTTGGCTATTTCAAACAAAGGACGAAGAGGTGGAAGTGACCT  800
     T T A L K D V A L A I N Q S N R L P L L N K C P N A P I C G S F Y L L M E * I V I V L H T R D V * L K A F L G I F N T E Q K E V K V P   F6
      Q P W S T W L W H L I K P I E * H C C T K A L I Q Q Y V V V S I C S C R K S * L * W I H G T S K F S Q S Y V S L T Q K R S R W R * Q  F5
      N H G A Q G C G T C Y K P F K K T A A L K Q L S K S T Y L W Q F V L A D R L D C D G S T D Q R S L A K R I F R Y L K N G A E G G E S S  F4

      E * T H A L N T M G S P N N F I Y M L H N F V C V I * P V Y I R K M L T E T E S K L Q C M L F V G F S F N G * C P S W I F A R I Y C L  F1
     S K P T L * I P W G A R I I S S T C S T T L C V * F D Q F T * G K C S P R Q K A N C S A C S L * D S V S T G N V R V G F L Q E Y T A C   F2
      V N P R F E Y H G E P E * F H L H A P Q L C V C N L T S L H K E N A H R D R K Q T A V H A L C R I Q F Q R V M S E L D F C K N I L L  F3
 801 GAGTAAACCCACGCTTTGAATACCATGGGGAGCCCGAATAATTTCATCTACATGCTCCACAACTTTGTGTGTGTAATTTGACCAGTTTACATAAGGAAAATGCTCACCGAGACAGAAAGCAAACTGCAGTGCATGCTCTTTGTAGGATTCAGTTTCAACGGGTAATGTCCGAGTTGGATTTTTGCAAGAATATACTGCTT 1000
     ----:----|----:----|----:----|----:----|----:----|----:----|----:----|----:----|----:----|----:----|----:----|----:----|----:----|----:----|----:----|----:----|----:----|----:----|----:----|----:----|
 801 CTCATTTGGGTGCGAAACTTATGGTACCCCTCGGGCTTATTAAAGTAGATGTACGAGGTGTTGAAACACACACATTAAACTGGTCAAATGTATTCCTTTTACGAGTGGCTCTGTCTTTCGTTTGACGTCACGTACGAGAAACATCCTAAGTCAAAGTTGCCCATTACAGGCTCAACCTAAAAACGTTCTTATATGACGAA 1000
     S Y V W A K F V M P L G F L K M * M S W L K T H T I Q G T * M L F I S V S V S L L S C H M S K T P N L K L P Y H G L Q I K A L I Y Q K   F6
      L T F G R K S Y W P S G S Y N * R C A G C S Q T H L K V L K C L S F A * R S L F C V A T C A R Q L I * N * R T I D S N S K Q L F I S S  F5
      L L G V S Q I G H P A R I I E D V H E V V K H T Y N S W N V Y P F H E G L C F A F Q L A H E K Y S E T E V P L T R T P N K C S Y V A  F4

      H F A E K I E Q C C H M L F S F W V F D * C G D Y A N V F A I K Y R T R I I S W H H E F R L Q A K G F P Q M L I Q H L F S M L Q G S S  F1
     I L L K R L N N V A T C C F L S G S L I N A E T M P M F L R S N I V R A * F P G I M S L G C K P R V F L K C S S N I F S A C C K A V   F2
      A F C * K D * T M L P H V V F F L G L * L M R R L C Q C F C D Q I S Y A H N F L A S * V * A A S Q G F S S N A H P T S F Q H V A R Q F  F3
1001 GCATTTTGCTGAAAAGATTGAACAATGTTGCCACATGTTGTTTTCTTTCTGGGTCTTTGATTAATGCGGAGACTATGCCAATGTTTTTGCGATCAAATATCGTACGCGCATAATTTCCTGGCATCATGAGTTTAGGCTGCAAGCCAAGGGTTTTCCTCAAATGCTCATCCAACATCTTTTCAGCATGTTGCAAGGCAGTT 1200
     ----:----|----:----|----:----|----:----|----:----|----:----|----:----|----:----|----:----|----:----|----:----|----:----|----:----|----:----|----:----|----:----|----:----|----:----|----:----|----:----|
1001 CGTAAAACGACTTTTCTAACTTGTTACAACGGTGTACAACAAAAGAAAGACCCAGAAACTAATTACGCCTCTGATACGGTTACAAAAACGCTAGTTTATAGCATGCGCGTATTAAAGGACCGTAGTACTCAAATCCGACGTTCGGTTCCCAAAAGGAGTTTACGAGTAGGTTGTAGAAAAGTCGTACAACGTTCCGTCAA 1200
     C K A S F I S C H Q W M N N E K Q T K S * H P S * A L T K A I L Y R V R M I E Q C * S N L S C A L P K G * I S M W C R K L M N C P L   F6
      A N Q Q F S Q V I N G C T T K K R P R Q N I R L S H W H K Q S * I D Y A C L K R A D H T * A A L W P N E E F A * G V D K * C T A L C N  F5
      Q M K S F L N F L T A V H Q K R E P D K I L A S V I G I N K R D F I T R A Y N G P M M L K P Q L G L T K R L H E D L M K E A H Q L A T  F4

      I L L R L S P C I H L S S F P Y K N L L E N P S K I Y I G M R G I E * S L C C I R S N Q R N A I D A F A L L G K I T V I Q I V R V F  F1
     L F F S D S V H A Y T S A V S L T R T F L K T L P R F I L A C V E S S D L S V A S E V I K G M L L T P L H F W A R S L S F R L S G F   F2
      Y S S Q T Q S M H T P Q Q F P L Q E P S * K P F Q D L Y W H A W N R V I S L L H P K * S K E C Y * R L C T F G Q D H C H S D C Q G F Q  F3
1201 CTATTCTTCTCAGACTCAGTCCATGCATACACCTCAGCAGTTTCCCTTACAAGAACCTTCTTGAAAACCCTTCCAAGATTTATATTGGCATGCGTGGAATCGAGTGATCTCTCTGTTGCATCCGAAGTAATCAAAGGAATGCTATTGACGCCTTTGCACTTTTGGGCAAGATCACTGTCATTCAGATTGTCAGGGTTTTC 1400
     ----:----|----:----|----:----|----:----|----:----|----:----|----:----|----:----|----:----|----:----|----:----|----:----|----:----|----:----|----:----|----:----|----:----|----:----|----:----|----:----|
1201 GATAAGAAGAGTCTGAGTCAGGTACGTATGTGGAGTCGTCAAAGGGAATGTTCTTGGAAGAACTTTTGGGAAGGTTCTAAATATAACCGTACGCACCTTAGCTCACTAGAGAGACAACGTAGGCTTCATTAGTTTCCTTACGATAACTGCGGAAACGTGAAAACCCGTTCTAGTGACAGTAAGTCTAACAGTCCCAAAAG 1400
     E I R R L S L G H M C R L L K G * L F R R S F G E L I * I P M R P I S H D R Q Q M R L L * L F A I S A K A S K P L I V T M * I T L T K   F6
      * E E * V * D M C V G * C N G K C S G E Q F G K W S K Y Q C A H F R T I E R N C G F Y D F S H * Q R R Q V K P C S * Q * E S Q * P K  F5
      R N K E S E T W A Y V E A T E R V L V K K F V R G L N I N A H T S D L S R E T A D S T I L P I S N V G K C K Q A L D S D N L N D P N E  F4

      K A V L V E G N C F I I G P C P A K R T N I R L T T G * S S R T K C A K E T G P T A L K A R G C P L K F F I Y H A V D K M Q L M F P N  F1
     R R Y L * R E T V S S * V R V L L K E P T S G * Q L D E V A E Q S V Q R K L D P L P * R P E D V L S N F S S T M L * I K C S * C S L T   F2
      G G T C R G K L F H H R S V S C * K N Q H Q A D N W M K * Q N K V C K G N W T H C L E G Q R M S S Q I F H L P C C R * N A A D V P *  F3
1401 AAGGCGGTACTTGTAGAGGGAAACTGTTTCATCATAGGTCCGTGTCCTGCTAAAAGAACCAACATCAGGCTGACAACTGGATGAAGTAGCAGAACAAAGTGTGCAAAGGAAACTGGACCCACTGCCTTGAAGGCCAGAGGATGTCCTCTCAAATTTTTCATCTACCATGCTGTAGATAAAATGCAGCTGATGTTCCCTAA 1600
     ----:----|----:----|----:----|----:----|----:----|----:----|----:----|----:----|----:----|----:----|----:----|----:----|----:----|----:----|----:----|----:----|----:----|----:----|----:----|----:----|
1401 TTCCGCCATGAACATCTCCCTTTGACAAAGTAGTATCCAGGCACAGGACGATTTTCTTGGTTGTAGTCCGACTGTTGACCTACTTCATCGTCTTGTTTCACACGTTTCCTTTGACCTGGGTGACGGAACTTCCGGTCTCCTACAGGAGAGTTTAAAAAGTAGATGGTACGACATCTATTTTACGTCGACTACAAGGGATT 1600
     L A T S T S P F Q K M M P G H G A L L V L M L S V V P H L L L V F H A F S V P G V A K F A L P H G R L N K M * W A T S L I C S I N G L   F6
      * P P V Q L P F S N * * L D T D Q * F F W C * A S L Q I F Y C F L T H L F P F Q V W Q R S P W L I D E * I K * R G H Q L Y F A A S T G *  F5
      L R Y K Y L S V T E D Y T R T R S F S G V D P Q C S S S T A S C L T C L F S S G S G Q L G S S T R E F K E D V M S Y I F H L Q H E R  F4

      T R P R T I Q Q L Q C S T F H F F S F P * Q T E Q K C * D T G S V T L I T M T N N D G S S I I N R A C K F F P E Q D M L F T I N N G H  F1
     H A P E P S S N C S V A P F I F S L S L D K L N R S V E I L G L S L S S Q * P T M T G R V L S T E L A N S S L N R T C S L P S T M A   F2
      H T P Q N H P A T A V * H L S F F L F P L T N * T E V L R Y W V C H S H H N D Q Q * R V E Y Y Q P S L Q I L P * T G H A L Y H Q Q W P  F3
1601 CACACGCCCCAGAACCATCCAGCAACTGCAGTGTAGCACCTTTCATTTTTTCTCTTTCCCTTGACAAACTGAACAGAAGTGTTGAGATACTGGGTCTGTCACTCTCATCACAATGACCAACAATGACGGGTCGAGTATTATCAACCGAGCTTGCAAATTCTTCCCTGAACAGGACATGCTCTTTACCATCAACAATGGCC 1800
     ----:----|----:----|----:----|----:----|----:----|----:----|----:----|----:----|----:----|----:----|----:----|----:----|----:----|----:----|----:----|----:----|----:----|----:----|----:----|----:----|
1601 GTGTGCGGGGTCTTGGTAGGTCGTTGACGTCACATCGTGGAAAGTAAAAAAGAGAAAGGGAACTGTTTGACTTGTCTTCACAACTCTATGACCCAGACAGTGAGAGTAGTGTTACTGGTTGTTACTGCCCAGCTCATAATAGTTGGCTCGAACGTTTAAGAAGGGACTTGTCCTGTACGAGAAATGGTAGTTGTTACCGG 1800
     V R G L V M W C S C H L V K * K K E K G Q C V S C F H Q S V P D T V R M V I V L L S P D L I I L R A Q L N K G S C S M S K V M L L P   F6
      C V G W F W G A V A T Y C R E N K R K G K V F Q V S T N L Y Q T Q * E * * L S W C H R T S Y * * G L K C I R G Q V P C A R * W * C H G  F5
      V C A G S G D L L Q L T A G K M K E R E R S L S F L L T S I S P R D S E D C H G V I V P R T N D V S S A F E E R F L V H E K G D V I A  F4

      T R K T A K R I T E N F I C S R K V C F L H F H I N I S K T I T P I F Y D C F Y D C R * W * P K F T E * F * N C V I * L P K T F C N  F1
     T Q E R Q Q K E * R K T L S V R A K S A C I F T L T S P R P S P P S F M I V F T T V D D G N P S S L S D F E I V S S N S Q R H F A T   F2
      H K K D S K K N N G K L Y L F A Q S L L S A F S H * H L Q D H P H L L * L F L R L * M M V T Q V H * V I L K L C H L T P K D I L Q L  F3
1801 ACACAAGAAAGACAGCAAAAAGAATAACGGAAAACTTTATCTGTTCGCGCAAAGTCTGCTTCTGCATTTTCACATTAACATCTCCAAGACCATCACCCCCATCTTTTATGATTGTTTTTACGACTGTAGATGATGGTAACCCAAGTTCACTGAGTGATTTTGAAATTGTGTCATCTAACTCCCAAAGACATTTTGCAAC 2000
     ----:----|----:----|----:----|----:----|----:----|----:----|----:----|----:----|----:----|----:----|----:----|----:----|----:----|----:----|----:----|----:----|----:----|----:----|----:----|----:----|
1801 TGTGTTCTTTCTGTCGTTTTTCTTATTGCCTTTTGAAATAGACAAGCGCGTTTCAGACGAAAGACGTAAAAGTGTAATTGTAGAGGTTCTGGTAGTGGGGGTAGAAAATACTAACAAAAATGCTGACATCTACTACCATTGGGTTCAAGTGACTCACTAAAACTTTAACACAGTAGATTGAGGGTTTCTGTAAAACGTTG 2000
     W V L F V A F L I V S F K I Q E R L T Q K R C K * M L M E L V M V G M K * S Q K * S Q L H H Y G L N V S H N Q F Q T M * S G F V N Q L   F6
      C L F S L L F F L P F S * R N A C L R S E A N E C * C R W S W * G W R K H N N K R S Y I I T V W T * Q T I K F N H * R V G L S M K C  F5
      V C S L C C F S Y R F V K D T R A F D A K Q M K V N V D G L G D G G D K I I T K V V T S S P L G L E S L S K S I T D D L E W L C K A V  F4

      C * Y I A E L N P * Q I R * C K V F R K V F E A S H D V L G F A I F I * Q D I L L S N N F G I F T E F V C T V T G Q K V W F S C C Q L  F1
     A D I * L N S T P D R F G S A K S S G K F L K R L M M S L G S Q S L S D R I S C * A I T S E F L Q S L Y A Q * P G R K F G S A A V S *   F2
      L I Y S * T Q P L T D S V V Q S L Q E S F * S V S * C P W V R N L Y L T Y P A E Q * L R N F Y R Y V C M H S D R A E S L V Q L L S A  F3
2001 TGCTGATATATAGCTGAACTCAACCCCTGACAGATTCGGTAGTGCAAAGTCTTCAGGAAAGTTTTTGAAGCGTCTCATGATGTCCTTGGGTTCGCAATCTTTATCTGACAGGATATCTCCTGCTGAGCAATAACTTCGGAATTTTTACAGAGTTTGTATGCACAGTGACCGGGCAGAAAGTTTGGTTCAGCTGCTGTCAGCT 2200
     ----:----|----:----|----:----|----:----|----:----|----:----|----:----|----:----|----:----|----:----|----:----|----:----|----:----|----:----|----:----|----:----|----:----|----:----|----:----|----:----|
2001 ACGACTATATATCGACTTGAGTTGGGGACTGTCTAAGCCATCACGTTTCAGAAGTCCTTTCAAAAACTTCGCAGAGTACTACAGGAACCCAAGCGTTAGAAATAGACTGTCCTATAGGACGACTCGTTATTGAAGCCTTAAAAATGTCTCAAACATACGTGTCACTGGCCCGTCTTTCAAACCAAGTCGACGACAGTCGA 2200
     Q Q Y I A S S L G Q C I R Y H L T K L F T K S A D * S T R P N A I K I Q C S I R S L L L K P I K V S N T H V T V P C F T Q N L Q Q * S   F6
      S S I Y L Q V * G R V S E T T C L R * S L K Q L T E H H G Q T R L R * R V P Y G A S C Y S R F K * L T Q I C L S R A S L K T * S S D A  F5
      A S I Y S F E V G S L N P L A F D E P F N K F R R M I D K P E C D K D S L I D Q Q A I V E S N K C L K Y A C H G P L F N P E A A T L  F4

      M G R F E R I C T C F I T L D V I C V S V M N F R L E S S N T H C * A F L R R Q F H V I T L T F P Q * K Y F I S L S F P P D V N K F L  F1
     W G G L R E F A H V L S R W M L Y A C L * * T L D W R V R T R I A K H S S G D S S M S S P * L F H S E S T L S A S L F L P T S T S F   F2
      D G E V * E N L H M F Y H A G C Y M R V C N E L * I G E F E H A L L S I P Q E T V P C H H L D F S T V K V L Y Q P L F S S R R Q Q V F  F3
2201 GATGGGGAGGTTTGAGAGAATTTGCACATGTTTTATCACGCTGGATGTTATATGCGTGTCTGTAATGAACTTTAGATTGGAGAGTTCGAACACGCATTGCTAAGCATTCCTCAGGAGACAGTTCCATGTCATCACCTTGACTTTTCCACAGTGAAAGTACTTTATCAGCCTCTCTTTTCCTCCCGACGTCAACAAGTTTT 2400
     ----:----|----:----|----:----|----:----|----:----|----:----|----:----|----:----|----:----|----:----|----:----|----:----|----:----|----:----|----:----|----:----|----:----|----:----|----:----|----:----|
2201 CTACCCCTCCAAACTCTCTTAAACGTGTACAAAATAGTGCGACCTACAATATACGCACAGACATTACTTGAAATCTAACCTCTCAAGCTTGTGCGTAACGATTCGTAAGGAGTCCTCTGTCAAGGTACAGTAGTGGAACTGAAAAGGTGTCACTTTCATGAAATAGTCGGAGAGAAAAGGAGGGCTGCAGTTGTTCAAAA 2400
     I P L N S L I Q V H K I V S S T I H T D I F K L N S L E F V C Q * A N R L L C N W T M V K V K G C H F Y K I L R E K G G S T L L N   F6
      S P S T Q S F K C M N * * A P H * I R T Q L S S * I P S N S C A N R S L M G * S V T G H * * R S K E V T F T S * * G R K E E R R * C T K  F5
      Q H P P K L S N A C T K D R Q I N Y A H R Y H V K S Q L T R V R M A L C E E P S L E M D D G Q S K W L S L V K D A E R K R G V D V L K  F4
```

```
      N * N G I Q R V N K I L L F F F T E I V N R Q D K F L K A S L F N V M S S D L L Q R R L F P * F R C G A G A * W S I W S S R P Q V S    F1
     * I K M A Y S E S T R S S F F S S Q K L S T D R I S S L R R L C L M * C L V T C C N G G F F R D S D V G L V H D G V F G R P G P K * A   F2
     E L K W H T A S Q Q D P P F F L H R N C Q Q T G * V P * G V F V * C D V * * L A A T E A F S V I Q M W G W C M M E Y L V V Q A P S K H   F3
2401 TGAATTAAAATGGCATACAGCGAGTCAACAAGATCCTCCTTTTTTTCTTCACAGAAATTGTCAACAGACAGGATAAGTTCCTTAAGGCGTCTTTGTTTAATGTGATGTCTAGTGACTTGCTGCAACGGAGGCTTTTTCCGTGATTCAGATGTGGGGCTGGTGCATGATGGAGTATTTGGTCGTCCAGGCCCCAAGTAAGC 2600
     ----:----|----:----|----:----|----:----|----:----|----:----|----:----|----:----|----:----|----:----|----:----|----:----|----:----|----:----|----:----|----:----|----:----|----:----|----:----|----:----|
2401 ACTTAATTTTACCGTATGTCGCTCAGTTGTTCTAGGAGGAAAAAAAGAAGTGTCTTTAACAGTTGTCTGTCCTATTCAAGGAATTCCGCAGAAACAAATTACACTACAGATCACTGAACGACGTTGCCTCCGAAAAAGGCACTAAGTCTACACCCCGACCACGTACTACCTCATAAACCAGCAGGTCCGGGGTTCATTCG 2600
     K F * F P M C R T L L I R R K K K V S I T L L C S L N R L A D K N L T I D L S K S C R L S K G H N L H P A P A H H L I Q D D L G W T L   F6
      S N F H C V A L * C S G G K K R * L F Q * C V P Y T G * P T K T * H S T * H S A A V S A K E T I * I H P Q H M I S Y K T T W A G L L    F5
     Q I L I A Y L S D V L D E K K E E C F N D V S L I L E K L R R Q K I H H R T V Q Q L P P K K R S E S T P S T C S P T N P R G P G L Y A   F4


     M I S F H S N H * G L N V D G D G H V Y P * N L T L * G K N W G A V K P F P S E T F F I R R Q T L F R H L L H I C F G K I S S D S P A   F1
      * S A S T V T T E G S M L M E M G M S I P R T * P C E E K T G V L S N L S P Q K H S S S G D K L C F D T C C I S V S E R F L Q I L Q Q  F2
      D Q L P Q * P L R A Q C * W R W A C L S L E L D L V R K K L G C C Q T F P L R N I L H Q A T N S V S T L V A Y L F R K D F F R F S S   F3
2601 ATGATCAGCTTCCACAGTAACCACTGAGGGCTCAATGTTGATGGAGATGGGCATGTCTATCCCTAGAACTTGACCTTGTGAGGAAAAAACTGGGGTGCTGTCAAACCTTTCCCCTCAGAAACATTCTTCATCAGGCGACAAACTCTGTTTCGACACTTGTTGCATATCTGTTTCGGAAAGATTTCTTCAGATTCTCCAGC 2800
     ----:----|----:----|----:----|----:----|----:----|----:----|----:----|----:----|----:----|----:----|----:----|----:----|----:----|----:----|----:----|----:----|----:----|----:----|----:----|----:----|
2601 TACTAGTCGAAGGTGTCATTGGTGACTCCCGAGTTACAACTACCTCTACCCGTACAGATAGGGATCTTGAACTGGAACACTCCTTTTTTGACCCCACGACAGTTTGGAAAGGGGAGTCTTTGTAAGAAGTAGTCCGCTGTTTGAGACAAAGCTGTGAACAACGTATAGACAAAGCCTTTCTAAAGAAGTCTAAGAGGTCG 2800
      M I L K W L L W Q P S L T S P S P C T * G * F K V K H P F F Q P A T L G K G E S V N K M L R C V R N R C K N C I Q K P F I E E S E G A  F6
     C S * S G C Y G S L A * H Q H L H A H R D R S S S R T L F F S P H Q * V K G R L F M R * * A V F E T E V S T A Y R N R F S K K L N E L   F5
      H D A E V T V V S P E I N I S I P M D I G L V Q G Q S S F V P T S D F R E G * F C E E D P S L S Q K S V Q Q M D T E S L N R * I R W    F4


      T E I P * C S * I V S A A S E T S * K D F C R F T G V S A S L Q R R Q I F * R T W A * Y W S I F V A S S G W R Y Y Q Y L K K N T K S Q  F1
       Q R F H N A A E S C Q Q P Q K P H R K T S A A S L V S A L H C R D G K S F E G P G H D T G P F L * Q V V G G D I T S T * K K T Q K V   F2
     N R D S I M Q L N R V S S L R N L I E R L L P L H W C Q R F I A E T A N L L K D L G M I L V H F C S K * W V E I L P V P E K K H K K S   F3
2801 AACAGAGATTCCATAATGCAGCTGAATCGTGTCAGCAGCCTCAGAAACCTCATAGAAAGACTTCTGCCGCTTCACTGGTGTCAGCGCTTCATTGCAGAGACGGCAAATCTTTTGAAGGACCTGGGCATGATACTGGTCCATTTTTGTAGCAAGTAGTGGGTGGAGATATTACCAGTACCTGAAAAAAAACACAAAAAGTC 3000
     ----:----|----:----|----:----|----:----|----:----|----:----|----:----|----:----|----:----|----:----|----:----|----:----|----:----|----:----|----:----|----:----|----:----|----:----|----:----|----:----|
2801 TTGTCTCTAAGGTATTACGTCGACTTAGCACAGTCGTCGGAGTCTTTGGAGTATCTTTCTGAAGACGGCGAAGTGACCACAGTCGCGAAGTAACGTCTCTGCCGTTTAGAAAACTTCCTGGACCCGTACTATGACCAGGTAAAAACATCGTTCATCACCCACCTCTATAATGGTCATGGACTTTTTTTGTGTTTTTCAG 3000
      V S I G Y H L Q I T D A A E S V E Y F S K Q R K V P T L A E N C L R C I K Q L V Q A H Y Q D M K T A L L P H L Y * W Y R F F F V F L   F6
      L L S E M I C S F R T L L R L F R M S L S R G S * Q H * R K M A S V A F R K F S R P M I S T W K Q L L Y H T S I N G T G S F F C L F D  F5
     C C L N W L A A S D H * C G * F G * L F V E A A E S T D A S * Q L S P L D K S P G P C S V P G N K Y C T T P P S I V L V Q F F V C F T   F4
```

Notospermus geniculatus (Nge)

WGS: NMRB01005477.1

4848-9190
Notospermus geniculatus strain Ushimado scaffold5477, whole genome shotgun sequence

```
   Y H T D V L K R L C R I C G G F L C P G I A P R Q K N F A S V L R F S R T I L L H Y G I S V E E V T Q F F P C M F A T S A K H S C I A  F1
   T T L M S * N A C A A F V E A F C A Q G L L L G K R T L L L F * D F L E P Y F F I M V F L L K K * R S F S L A C L Q Q V Q N T A V S L  F2
   P H * C P E T P V P H L W R L F V P R D C S S A K E L C F C F E I F S N H T S S L W Y F C * R S D A V F P L H V C N K C K T Q L Y R  F3
1 TACCACACTGATGTCCTGAAACGCCTGTGCCGCATTTGTGGAGGCTTTTTGTGCCCAGGGATTGCTCCTCGGCAAAAGAACTTTGCTTCTGTTTTGAGATTTTCTCGAACCATACTTCTTCATTATGGTATTTCTGTTGAAGAAGTGACGCAGTTTTTCCCTTGCATGTTTGCAACAAGTGCAAAACACAGCTGTATCGC 200
  ----:----|----:----|----:----|----:----|----:----|----:----|----:----|----:----|----:----|----:----|----:----|----:----|----:----|----:----|----:----|----:----|----:----|----:----|----:----|----:----|
1 ATGGTGTGACTACAGGACTTTGCGGACACGGCGTAAACACCTCCGAAAAACACGGGTCCCTAACGAGGAGCCGTTTTCTTGAAACGAAGACAAAACTCTAAAAGAGCTTGGTATGAAGAAGTAATACCATAAAGACAACTTCTTCACTGCGTCAAAAAGGGAACGTACAAACGTTGTTCACGTTTTGTGTCGACATAGCG 200
  X W V S T R F R R H R M Q P P K K H G P I A G R C F F K A E T K L N E R V M S R * * P I E T S S T V C N K G Q M N A V L A F C L Q I A  F6
  X G C Q H G S V G T G C K H L S K T G L S Q E E A F S S Q K Q K S I K E F W V E E N H Y K Q Q L L S A T K G K C T Q L L H L V C S Y R  F5
   V V S I D Q F A Q A A N T S A K Q A W P N S R P L L V K S R N Q S K R S G Y K K M I T N R N F F H R L K E R A H K C C T C F V A T D  F4

   W I Q K M S H R T E V Q N I S P F I M I R T G F V L L G K E M G I P I P H T E P M E V V I D E E D V D E M D L L D H S Y S S V L A T D  F1
   G F K R * A T E Q K C K I F P P S * * S G L V L C Y L A K K W G F L F L T L S Q W K W * L T K R M W M K W T C W I I A T P V F L Q Q  F2
   L D S K D E P Q N R S A K Y F P L H N D Q D W F C V T W Q R N G D S Y S S H * A N G S G D * R R G C G * N G L V G S * L L Q C S C N R  F3
201 TTGGATTCAAAAGATGAGCCACAGAACAGAAGTGCAAAATATTTCCCCCTTCATAATGATCAGGACTGGTTTTGTGTTACTTGGCAAAGAAATGGGGATTCCTATTCCTCACACTGAGCCAATGGAAGTGGTGATTGACGAAGAGGATGTGGATGAAATGGACTTGTTGGATCATAGCTACTCCAGTGTTCTTGCAACAG 400
  ----:----|----:----|----:----|----:----|----:----|----:----|----:----|----:----|----:----|----:----|----:----|----:----|----:----|----:----|----:----|----:----|----:----|----:----|----:----|----:----|
201 AACCTAAGTTTTCTACTCGGTGTCTTGTCTTCACGTTTATAAAGGGGGAAGTATTACTAGTCCTGACCAAAACACAATGAACCGTTTCTTTACCCCTAAGGATAAGGAGTGTGACTCGGTTACCTTCACCACTAACTGCTTCTCCTACACCTACTTTACCTGAACAACCTAGTATCGATGAGGTCACAAGAACGTTGTC 400
   Q I * F I L W L V S T C F I E G K M I I L V P K T N S P L S I P I G I G * V S G I S T T I S S S S T S S I S K N S * L * E L T R A V  F6
  K S E F S S G C F L L A F Y K G R * L S * S Q N Q T V Q C L F P S E * E E C Q A L P L P S Q R L P H P H F P S T P D Y S S W H E Q L L  F5
  S P N L L H A V S C F H L I N G G E Y H D P S T K H * K A F F H P N R N R V S L W H F H H N V F L I H I F H V Q Q I M A V G T N K C C  F4

   N S P V T D Q L S S A T K D S R T K K S L K D V N R R H I K H Y R L K E L F S H V S Q F C E D R L E S P V D V A Y C L L I Q N L I D  F1
  I I L * L I S * V L Q Q R T V A Q K N H * R M * T A V T S S I I A * K N F S L M C H S S V R T D L K V L L M L P T V C * F K I S * T  F2
   * F S C D * S A E F C N K G Q S H K K I T E G C E P P S H Q A L S P K R T F L S C V T V L * G Q T * K S C * C C L L F V D S K S H R L  F3
401 ATAATTCTCCTGTGACTGATCAGCTGAGTTCTGCAACAAAGGACAGTCGCACAAAAAAATCACTGAAGGATGTGAACCGCCGTCACATCAAGCATTATCGCCTAAAAGAACTTTTCTCTCATGTGTCACAGTTCTGTGAGGACAGACTTGAAAGTCCTGTTGATGTTGCCTACTGTTTGTTGATTCAAAATCTCATAGAC 600
  ----:----|----:----|----:----|----:----|----:----|----:----|----:----|----:----|----:----|----:----|----:----|----:----|----:----|----:----|----:----|----:----|----:----|----:----|----:----|----:----|
401 TATTAAGAGGACACTGACTAGTCGACTCAAGACGTTGTTTCCTGTCAGCGTGTTTTTTTAGTGACTTCCTACACTTGGCGGCAGTGTAGTTCGTAATAGCGGATTTTCTTGAAAAGAGAGTACACAGTGTCAAGACACTCCTGTCTGAACTTTCAGGACAACTACAACGGATGACAAACAACTAAGTTTTAGAGTATCTG 600
   S L E G T V S * S L E A V F S L R V F F D S F S T F R R * M L C * R R F S S K E * T D C N Q S S L S S L G T S T A * Q K N I * F R M S  F6
   Y N E Q S Q D A S N Q L L P C D C L F I V S P H S G G D C * A N D G L L V K R E H T V T R H P C V Q F D Q Q H Q R S N T S E F D * L  F5
  I I R R H S I L Q T R C C L V T A C F F * Q L I H V A T V D L M I A * F F K E R M H * L E T L V S K F T R N I N G V T Q Q N L I E Y V  F4

   S G R K K E A D R I M K M W K T D D D D T N L S V E E C L A M R V R S L Q S K C H Y K Q M Y S I H R D K T S S N S L Q P L K Q L S A I  F1
   L A G R K R Q T E L * K C G K P M T T T L I F L L K S V * L C V L G P S S Q S V I I N R C I Q F I E I R Q V Q T L C S L * N S F Q Q *  F2
   W P E E R G R Q N Y E N V E N R * R R H * S F C * R V F S Y A C * V P P V K V S L * T D V F N S * R * D K F K L F A A F K T A F S N  F3
601 TCTGGCCGGAAGAAAGAGGCAGACAGAATTATGAAAATGTGGAAAACCGATGACGACGACACTAATCTTTCTGTTGAAGAGTGTTTAGCTATGCGTGTTAGGTCCCTCCAGTCAAAGTGTCATTATAAACAGATGTATTCAATTCATAGAGATAAGACAAGTTCAAACTCTTTGCAGCCTTTAAAACAGCTTTCAGCAAT 800
  ----:----|----:----|----:----|----:----|----:----|----:----|----:----|----:----|----:----|----:----|----:----|----:----|----:----|----:----|----:----|----:----|----:----|----:----|----:----|----:----|
601 AGACCGGCCTTCTTTCTCCGTCTGTCTTAATACTTTTACACCTTTTGGCTACTGCTGCTGTGATTAGAAAGACAACTTCTCACAAATCGATACGCACAATCCAGGGAGGTCAGTTTCACAGTAATATTTGTCTACATAAGTTAAGTATCTCTATTCTGTTCAAGTTTGAGAAACGTCGGAAATTTTGTCGAAAGTCGTTA 800
   E P R F F S A S L I I F I H F V S S S S V L R E T S S H K A I R T L D R W D F H * * L C I Y E I * L S L V L E F E K C G K F C S E A I  F6
  S Q G S S L P L C F * S F T S F R H R R C * D K Q Q L T N L * A H * T G G T L T D N Y V S T N L E Y L Y S L N L S K A A K L V A K L L  F5
   R A P L F L C V S N H F H P F G I V V V S I K R N F L T * S H T N P G E L * L T M I F L H I * N M S I L C T * V R Q L R * F L K * C  F4

   E Y S F F P G Q S A Y Q L S K M E L C F V R N A F L Q K M A V N Q L I S W I T F R T F H V I S P V Q M F R G * N S I M C L L L Q N H C  F1
   N T R F S P A R V H I S C Q K W N C A L * E M H F F K R W L * T N * Y H G S L S E L S M * F P P S K C F G G R I Q L C V C Y C K I T  F2
   R I L V F P R P E C I S V V K N G T V L C E K C I S S K D G C E P T D I M D H F Q N F P C D F P R P N V S G V E F N Y V S A I A K S L  F3
801 AGAATACTCGTTTTTCCCCGGCCAGAGTGCATATCAGTTGTCAAAAATGGAACTGTGCTTTGTGAGAAATGCATTTCTTCAAAAGATGGCTGTGAACCAACTGATATCATGGATCACTTTCAGAACTTTCCATGTGATTTCCCCCGTCCAAATGTTTCGGGGGTAGAATTCAATTATGTGTCTGCTATTGCAAAATCACT 1000
  ----:----|----:----|----:----|----:----|----:----|----:----|----:----|----:----|----:----|----:----|----:----|----:----|----:----|----:----|----:----|----:----|----:----|----:----|----:----|----:----|
801 TCTTATGAGCAAAAAGGGGCCGGTCTCACGTATAGTCAACAGTTTTTACCTTGACACGAAACACTCTTTACGTAAAGAAGTTTTCTACCGACACTTGGTTGACTATAGTACCTAGTGAAAGTCTTGAAAGGTACACTAAAGGGGGCAGGTTTACAAAGCCCCCATCTTAAGTTAATACACAGACGATAACGTTTTAGTGA 1000
   S Y E N K G P W L A Y * N D F I S S H K T L F A N R * F I A T F W S I D H I V K L V K W T I E G T W I N R P Y F E I I H R S N C F *  F6
  L I S T K G R G S H M D T T L F P V T S Q S F H M E E F S P Q S G V S I M S * K * F K G H S K G R G F T E P T S N L * T D A I A F D S  F5
  Y F V R K E G A L T C I L Q * F H F Q A K H S I C K K L L H S H V L Q Y * P D S E S S E M H N G G D L H K P P L I * N H T Q * Q L I V  F4

   L N L T L S F V I G * K N * V * R L I H H S T R S L R I A L A T L T T * M T T T W Q I G L K V * R V C P L L N Q M P L T V P L I P H  F1
  A * T * P Y H L * * V E R T R S N A * Y T T L H D H * G S P W Q P * Q L E * Q Q L G R * D * R C E E Y A P Y * I R C H * P F P * F H T  F2
   L E L D P I I C N R L K E L G L T L D T P L Y T I I K D L R G N P D N L N D N N L A D R T K G V K S M P L I K S D A T D R S L D S T H  F3
1001 GCTTGAACTTGACCCTATCATTTGTAATAGGTTGAAAGAACTAGGTCTAACGCTTGATACACCACTCTACACGATCATTAAGGATCGCCTTGGCAACCCTGACAACTTGAATGACAACAACTTGGCAGATAGGACTAAAGGTGTGAAGAGTATGCCCCTTATTAAATCAGATGCCACTGACCGTTCCCTTGATTCCACAC 1200
  ----:----|----:----|----:----|----:----|----:----|----:----|----:----|----:----|----:----|----:----|----:----|----:----|----:----|----:----|----:----|----:----|----:----|----:----|----:----|----:----|
1001 CGAACTTGAACTGGGATAGTAAACATTATCCAACTTTCTTGATCCAGATTGCGAACTATGTGGTGAGATGTGCTAGTAATTCCTAGCGGAACCGTTGGGACTGTTGAACTTACTGTTGTTGAACCGTCTATCCTGATTTCCACACTTCTCATACGGGGAATAATTTAGTCTACGGTGACTGGCAAGGGAACTAAGGTGTG 1200
  Q K F K V R D N T I P Q F F * T * R K I C E V R D N L I A K A V R V V Q I V V V Q C I P S F T H L T H G K N F * I G S V T G K I G C  F6
   S S S S G I M Q L L N F S S P R V S S V G S * V I M L S R R P L G S L K F S L L K A S L V L P T F L I G R I L D S A V S R E R S E V  F5
  A Q V Q G * * K Y Y T S L V L D L A Q Y V V R C S * * P D G Q Q C G Q C S S H C C S P L Y S * L H S S Y A G * * I L H W Q G N G Q N W V  F4

   M Q I S I * A G F S R R Y L Y G R L L K F M N G L K Q I Q T S P G Y S M Q R S A W M K C L G K N L A C N L T * * C L A T M Q G N Y L S  F1
   C K Y Q S R Q G F Q E G T C T G D C S S S * M D * N R F K Q V Q V T A C R E V L G * S V * G K T W L A I S L N D A W Q L C K A T I * A  F2
   A N I N L G R V F K K V L V R E T A Q V H E W T E T D S N K S R L Q H A E K C L D E V F R E K L G L Q S H L M M P G N Y A R Q L F K  F3
1201 ATGCAAATATCAATCTAGGCAGGGTTTTCAAGAAGGTACTTGTACGGGAGACTGCTCAAGTTCATGAATGGACTGAAACAGATTCAAACAAGTCCAGGTTACAGCATGCAGAGAAGTGCTTGGATGAAGTGTTTAGGGAAAAACTTGGCTTGCAATCTCACTTAATGATGCCTGGCAACTATGCAAGGCAACTATTTAAG 1400
  ----:----|----:----|----:----|----:----|----:----|----:----|----:----|----:----|----:----|----:----|----:----|----:----|----:----|----:----|----:----|----:----|----:----|----:----|----:----|----:----|
1201 TACGTTTATAGTTAGATCCGTCCCAAAAGTTCTTCCATGAACATGCCCTCTGACGAGTTCAAGTACTTACCTGACTTTGTCTAAGTTTGTTCAGGTCCAATGTCGTACGTCTCTTCACGAACCTACTTCACAAATCCCTTTTTGAACCGAACGTTAGAGTGAATTACTACGGACCGTTGATACGTTCCGTTGATAAATTC 1400
   M C I D I * A P N E L L Y K Y P L S S L N M F P S F C I * V L G P * L M C L L A Q I F H K P F F K A Q L R V * H H R A V I C P L * K L  F6
  C A F I L R P L T K L F T S T R S V A * T * S H V S V S E F L D L N C C A S F H K S S T N L S F S P K C D * K I I G P L * A L C S N L  F5
   H L Y * D L C P K * S P V Q V P S Q E L E H I S Q F L N L C T W T V A H L S T S P H L T * P F V Q S A I E S L S A Q C S H L A V I *  F4

   Q Q T M L L S H P S S L M K R E D I M C S I S S H C F L K C S Q F T P A N L P E S N S L S L L P T T G K Y L C N L L S P W V N I F P T  F1
   S K P C Y Y H I P H H * * R E K I S C A Q Y P L T V F * N A V S L L Q Q I S Q R A I P * A Y C Q L Q G N I S A I C F H H G * T F S L  F2
  P A N H A I T S L I T D E E R R Y H V L N I L S L F S K M Q S V Y S S K S P R E Q F P E L T A N Y R E I S L Q F A F T M G E H F P Y  F3
1401 CCAGCAAACCATGCTATTATCACATCCCTCATCACTGATGAAGAGAGAAGATATCATGTGCTCAATATCCTCTCACTGTTTTCTAAAATGCAGTCAGTTTACTCCAGCAAATCTCCCAGAGAGCAATTCCCTGAGCTTACTGCCAACTACAGGGAAATATCTCTGCAATTTGCTTTCACCATGGGTGAACATTTTCCCTA 1600
  ----:----|----:----|----:----|----:----|----:----|----:----|----:----|----:----|----:----|----:----|----:----|----:----|----:----|----:----|----:----|----:----|----:----|----:----|----:----|----:----|
1401 GGTCGTTTGGTACGATAATAGTGTAGGGAGTAGTGACTACTTCTCTCTTCTATAGTACACGAGTTATAGGAGAGTGACAAAAGATTTTACGTCAGTCAAATGAGGTCGTTTAGAGGGTCTCTCGTTAAGGGACTCGAATGACGGTTGATGTCCCTTTATAGAGACGTTAAACGAAAGTGGTACCCACTTGTAAAAGGGAT 1600
   W C V M S N D C G E D S I F L S S I M H E I D E * Q K R F H L * N V G A F R G S L L E R L K S G V V P F Y R Q L K S E G H T F M K G  F6
   G A F W A I I V D R M V S S S L L Y * T S L I R E S N E L I C D T * E L L D G L S C N G S S V A L * L S I D R C N A K V M P S C K G *  F5
  A L L G H * * * M G * * Q H L S F I D H A * Y G R V T K * F A T L K S W C I E W L A I G Q A * Q W S C P F I E A I Q K * W P H V N E R  F4

   F H G P T T C T R * L S M C L R L L R T T N C Q L A V L D C C P V R A E R V V I S S I G I S G N I S L A P T V L A T * G T H L C F I  F1
  H S M V Q L H A Q G D * A C A * D Y * G R P T V S W L C W I A V Q * G R R E W * * A L S A Y Q E T S L S L R Q C W R L K G R T C V S L  F2
   I P W S N Y M H K V I E H V P E I I E D D Q L S V G C V G L L S S E G G E S G N K L Y R H I R K H L S R S D S A G D L R D A L V F H W  F3
1601 CATTCCATGGTCCAACTACATGCACAAGGTGATTGAGCATGTGCCTGAGATTATTGAGGACGACCAACTGTCAGTTGGCTGTGTTGGATTGCTGTCCAGTGAGGGCGGAGAGAGTGGTAATAAGCTCTATCGGCATATCAGGAAACATCTCTCTCGCTCCGACAGTGCTGGCGACTTAAGGGACGCACTTGTGTTTCATT 1800
  ----:----|----:----|----:----|----:----|----:----|----:----|----:----|----:----|----:----|----:----|----:----|----:----|----:----|----:----|----:----|----:----|----:----|----:----|----:----|----:----|
1601 GTAAGGTACCAGGTTGATGTACGTGTTCCACTAACTCGTACACGGACTCTAATAACTCCTGCTGGTTGACAGTCAACCGACACAACCTAACGACAGGTCACTCCCGCCTCTCTCACCATTATTCGAGATAGCCGTATAGTCCTTTGTAGAGAGAGCGAGGCTGTCACGACCGCTGAATTCCCTGCGTGAACACAAAGTAA 1800
   V N W P G V V H V L H N L M H R L N N L V V L Q * N A T N S G Q T L A S L T T I L E I P M D P F M E R A G V T S A V * P V C K H K M  F6
   M G H D L * M C L T I S C T G S I I S S W S D T P Q T P N S D L S P P S L P L L S * R C I L F C R E R E S L A P S K L S A S T N *  F5
  C E M T W S C A C P S Q A H A Q S * Q P R G V T L Q S H Q I A T W H P R L S H Y Y A R D A Y * S V D R E S R C H Q R S L P R V Q T E N  F4

  G F T L V K R F K I L Q P Q Q K S L S T V Q C V V R V A T T N V P V F M T P T Q F R P N A I L L V K I V Y K T K N G L Q I S Q I F D P  F1
   A L H * * K D S K S C S H N K N H S A L Y N V W * E W P Q Q T F L C L * P Q H S L D Q T P S C W * R L Y I K L K T V S K F R K F S T P  F2
   L Y T S K K I Q N L A A T T K T I T Q H C T M C G E S G H N K R S C V Y D P N T V * T K R H L V G K D C I * N * K R S P N F A N F R P  F3
1801 GGCTTTACACTAGTAAAAAGATTCAAAATCTTGCAGCCACAACAAAAATCACTCAGCACTGTACAATGTGTGGTGAGAGTGGCCACAACAAACGTTCCTGTGTTTATGACCCCAACACAGTTTAGACCAAACGCCATCTTGTTGGTAAAGATTGTATATAAAACTAAAAACGGTCTCCAAATTTCGCAAATTTTCGACCC 2000
  ----:----|----:----|----:----|----:----|----:----|----:----|----:----|----:----|----:----|----:----|----:----|----:----|----:----|----:----|----:----|----:----|----:----|----:----|----:----|----:----|
1801 CCGAAATGTGATCATTTTTCTAAGTTTTAGAACGTCGGTGTTGTTTTTAGTGAGTCGTGACATGTTACACACCACTCTCACCGGTGTTGTTTGCAAGGACACAAATACTGGGGTTGTGTCAAATCTGGTTTGCGGTAGAACAACCATTTCTAACATATATTTTGATTTTTGCCAGAGGTTTAAAGCGTTTAAAAGCTGGG 2000
   P K V S T F L N L I K C G C C F D S L V T C H T T L T A V V F T G T N I V G V C N L G F A M K N T F I T Y L V L F P R W I E C I K S G  F6
  Q S * V L L F I * F R A A V V F I V * C Q V I H P S L P W L L R E Q T * S G L V T * V L R W R T P L S Q I Y F * F R D G F K A F K R G  F5
   A K C * Y F S E F D Q L W L L F * E A S Y L T H H S H G C C V N R H K H G W C L K S W V G D Q Q Y L N Y I F S F V T E L N R L N E V  F4

   Q P G C I S S Y V Y T R D * F V S G Q L C L E T K * P E T Y I T E R L L Y T D E * R D G V F N T T H H V T S I P L R H S C R I Y Q C T  F1
   N R G V S Q A T S T R E T N L S R N V F V S R Q N N R R H I * R S V Y S T R M N D V T V F S T L R T M * R P F H Y V I H A V F I N A  F2
  P T G V Y L K L R L H E R L I C L G S T L S R D K I T G D I Y N G A S T L H G * M T * R C F Q H Y A P C D V H S I T S F M P Y L S M H  F3
2001 CCAACCGGGGTGTATCTCAAGCTACGTCTACACGAGAGACTAATTTGTCTCGGGTCAACTTTGTCTCGAGACAAAATAACCGGAGACATATATAACGGAGCGTCTACTCTACACGGATGAATGACGTGACGGTGTTTTCAACACTACGCACCATGTGACGTCCATTCCATTACGTCATTCATGCCGTATTTATCAATGCA 2200
  ----:----|----:----|----:----|----:----|----:----|----:----|----:----|----:----|----:----|----:----|----:----|----:----|----:----|----:----|----:----|----:----|----:----|----:----|----:----|----:----|
2001 GGTTGGCCCCACATAGAGTTCGATGCAGATGTGCTCTCTGATTAAACAGAGCCCAGTTGAAACAGAGCTCTGTTTTATTGGCCTCTGTATATATTGCCTCGCAGATGAGATGTGCCTACTTACTGCACTGCCACAAAAGTTGTGATGCGTGGTACACTGCAGGTAAGGTAATGCAGTAAGTACGGCATAAATAGTTACGT 2200
   W G P H I E L * T * V L S * N T E P * S Q R S V F Y G S V Y I V S R R S * V S S H R S P T K L V V C W T V D M G N R * E H R I * * H  F6
   G V P T Y R L S R R C S L I S Q R P D V K D R S L I I V P S M Y L P A D V R C P H I V H R H K * C * A G H S T W E M V D N M G Y K D I C  F5
  G L R P T D * A V D V R S V L K D R T L K T E L C F L R L C I Y R L T * E V R I F S T V T N E V S R V M H R G N W * T M * A T N I L A  F4

   H * * L R N Q Y R S L S R D R Q A I A S T R Q L S R D N F V A G Y L N P P T L L G S S * P E T K L S R D D L A S G S F * * E T T V K  F1
   H I D N Y G T N I G L C P E T D R R S R L H D N C L E T T L S R V I L T P L P Y W G Q V N P R Q S C L G T T * L Q A L S N K R L L * R  F2
   T L I T T E P I * Y F R P Q T G D R V Y T T T V S R Q L C R G L S * P P Y P T G V K L T R D K V V S G R L S F R L F L I R D Y C K D  F3
2201 CACATTGATAACTACGGAACCAATATAGGTCTTTGTCCCGAGACAGACAGGCGATCGCGTCTACACGACAACTGTCTCGAGACAACTTTGTCGCGGGTTATCTTAACCCCCCTACCCTACTGGGGTCAAGTTAACCCGAGACAAAGTTGTCTCGGGACGACTTAGCTTCAGGCTCTTTCTAATAGAGACTACTGTAAAG 2400
  ----:----|----:----|----:----|----:----|----:----|----:----|----:----|----:----|----:----|----:----|----:----|----:----|----:----|----:----|----:----|----:----|----:----|----:----|----:----|----:----|
2201 GTGTAACTATTGATGCCTTGGTTATATCCAGAAACAGGGCTCTGTCTGTCCGCTAGCGCAGATGTGCTGTTGACAGAGCTCTGTTGAAACAGCGCCCAATAGAATTGGGGGGATGGGATGACCCCAGTTCAATTGGGCTCTGTTTCAACAGAGCCCTGCTGAATCGAAGTCCGAGAAAGATTATCTCTGATGACATTTC 2400
  V C Q Y S R F W Y L D K D R S L C A I A D V R C S D R S L K T A P * R L G G V R S P D L * G S V F N D R S S K A E P E K * Y S V V T F  F6
   V N I V V S G I Y T K T G L C V P S R T * V V V T E L C S Q R P N D * G G * G V P T L N V R S L T T E P R S L K L S K R I L S * Q L  F5
  C M S L * P V L I P R Q G S V S L R D R R C S L Q R S V V K D R T I K V G R G * Q P * T L G L C L Q R P V V * S * A R E L L L S S Y L  F4
```

```
     T Q K I V K D C T H L L F I F T * A T F I S E I A H L T K K L * L H S I L * D D * V S C A K I R F * C F V N T E R N A I T F S Q I T P   F1
      L K R L * K I V H T F Y S F L H K Q L L F R R L H I S Q K N F D Y T Q Y C E M T R C P V P K * G F N V L L T L K E M Q L P F L K * L L  F2
      S K D C K R L Y T P F I H F Y I S N F Y F G D C T S H K K T L T T L N T V R * L G V L C Q N K V L M F C * H * K K C N Y L F S N N S   F3
2401 ACTCAAAAGATTGTAAAAGATTGTACACACCTTTTATTCATTTTTACATAAGCAACTTTTATTTCGGAGATTGCACATCTCACAAAAAAACTTTGACTACACTCAATACTGTGAGATGACTAGGTGTCCTGTGCCAAAATAAGGTTTTAATGTTTTGTTAACACTGAAAGAAATGCAATTACCTTTTCTCAAATAACTCC 2600
     ----:----|----:----|----:----|----:----|----:----|----:----|----:----|----:----|----:----|----:----|----:----|----:----|----:----|----:----|----:----|----:----|----:----|----:----|----:----|----:----|
2401 TGAGTTTTCTAACATTTTCTAACATGTGTGGAAAATAAGTAAAAATGTATTCGTTGAAAATAAAGCCTCTAACGTGTAGAGTGTTTTTTTGAAACTGATGTGAGTTATGACACTCTACTGATCCACAGGACACGGTTTTATTCCAAAATTACAAAACAATTGTGACTTTCTTTACGTTAATGGAAAAGAGTTTATTGAGG 2600
      V * F I T F S Q V C R K N M K V Y A V K I E S I A C R V F F S Q S C E I S H S S * T D Q A L I L N * H K T L V S L F A I V K E * I V G  F6
     S E F S Q L L N Y V G K I * K * M L L K * K P S Q V D * L F V K V V S L V T L H S P T R H W F L T K I N Q * C Q F F H L * R K E F L E   F5
      S L L N Y F I T C V K * E N K C L C S K N R L N C M E C F F K S * V * Y Q S I V L H G T G F Y P K L T K N V S F S I C N G K R L Y S   F4

      G I S G G A K N W H T S L S G H L Y S V P L L F R I Q R F F F F R P P V L G R Q S E Q V N E M S S I L P C A S P R P L Q Q R W N H L L  F1
       A Y R V A Q K T G T Q V Y L A I Y I V F L F C L E F S V F F S F D L Q S * V G S Q N K * M K C P L S C H V H R Q D H C S K G G T T C   F2
     W H I G W R K K L A H K F I W P S I * C S S F V * N S A F F F L S T S S L R S A V R T S E * N V L Y P A M C I A K T T A A K V E P L A   F3
2601 TGGCATATCGGGTGGCGCAAAAAACTGGCACACAAGTTTATCTGGCCATCTATATAGTGTTCCTCTTTTGTTTAGAATTCAGCGTTTTTTTTCTTTCGACCTCCAGTCTTAGGTCGGCAGTCAGAACAAGTGAATGAAATGTCCTCTATCCTGCCATGTGCATCGCCAAGACCACTGCAGCAAAGGTGGAACCACTTGC 2800
     ----:----|----:----|----:----|----:----|----:----|----:----|----:----|----:----|----:----|----:----|----:----|----:----|----:----|----:----|----:----|----:----|----:----|----:----|----:----|----:----|
2601 ACCGTATAGCCCACCGCGTTTTTTGACCGTGTGTTCAAATAGACCGGTAGATATATCACAAGGAGAAAACAAATCTTAAGTCGCAAAAAAAAGAAAGCTGGAGGTCAGAATCCAGCCGTCAGTCTTGTTCACTTACTTTACAGGAGATAGGACGGTACACGTAGCGGTTCTGGTGACGTCGTTTCCACCTTGGTGAACG 2800
      P M D P P A F F Q C V L K D P W R Y L T G R K N L I * R K K K K R G G T K P R C D S C T F S I D E I R G H A D G L G S C C L H F W K  F6
      Q C I P H R L F S A C L N I Q G D I Y H E E K T * F E A N K K R E V E L R L D A T L V L S H F T R * G A M H M A L V V A A F T S G S A  F5
     R A Y R T A C F V P V C T * R A M * I T N R K Q K S N L T K K E K S R W D * T P L * F L H I F H G R D Q W T C R W S W Q L L P P V V Q   F4

      H G P S Q T R E C M V S P G L K W F F T M Q S L A S Q T P G S I L F V V R T N K C C K A P P W K N T A S G L N K R D V * L A I S N P   F1
     Y M D L H R Q E N A W F L Q G * S G F L Q C S L W L H K H L D P S F L W * G Q T N V V K L H H G K I Q H Q D * T K G M Y D * P F Q T R   F2
      T W T F T D K R M H G F S R V E V V F Y N A V F G F T N T W I H P F C G E D K Q M L * S S T M E K Y S I R T K Q K G C M T S H F K P G  F3
2801 TACATGGACCTTCACAGACAAGAGAATGCATGGTTTCTCCAGGGTTGAAGTGGTTTTTTACAATGCAGTCTTTGGCTTCACAAACACCTGGATCCATCCTTTTTGTGGTGAGGACAAACAAATGTTGTAAAGCTCCACCATGGAAAAATACAGCATCAGGACTAAACAAAAGGGATGTATGACTAGCCATTTCAAACCCG 3000
     ----:----|----:----|----:----|----:----|----:----|----:----|----:----|----:----|----:----|----:----|----:----|----:----|----:----|----:----|----:----|----:----|----:----|----:----|----:----|----:----|
2801 ATGTACCTGGAAGTGTCTGTTCTCTTACGTACCAAAGAGGTCCCAACTTCACCAAAAAATGTTACGTCAGAAACCGAAGTGTTTGTGGACCTAGGTAGGAAAAACACCACTCCTGTTTGTTTACAACATTTCGAGGTGGTACCTTTTTATGTCGTAGTCCTGATTTGTTTTCCCTACATACTGATCGGTAAAGTTTGGGC 3000
      S C P G E C V L S H M T E G P N F H N K V I C D K A E C V G P D M R K T T L V F L H Q L A G G H F F V A D P S F L L S T H S A M E F G  F6
       V H V K V S L L I C P K E L T S T T K * L A T K P K V F V Q I W G K Q P S S L C I N Y L E V M S F Y L M L V L C F P H I V L W K L G   F5
      * M S R * L C S F A H N R W P Q L P K K C H L R Q S * L C R S G D K K H H P C V F T T F S W W P F I C C * S * V F P I Y S * G N * V R  F4

     D G A C F S V I D S V S V F T R K W C P P G L S S * * P P Y T S S L F E P R * F V V C P E I N T G S S V A P T R L S A L N E K S G F L   F1
      M V P A S R * L I V C R Y S L G N G A H R V Y H L D S R R T Q A A C L N Q G N L W Y V L K S I P V V L * H Q L V F P H * M K S Q V F S  F2
      W C L L L G D * * C V G I H * E M V P T G S I I L I A A V H K Q L V * T K V I C G M S * N Q Y R * F C S T N S S F R I K * K V R F S   F3
3001 GATGGTGCCTGCTTCTCGGTGATTGATAGTGTGTCGGTATTCACTAGGAAATGGTGCCCACCGGGTCTATCATCTTGATAGCCGCCGTACACAAGCAGCTTGTTTGAACCAAGGTAATTTGTGGTATGTCCTGAAATCAATACCGGTAGTTCTGTAGCACCAACTCGTCTTTCCGCATTAAATGAAAAGTCAGGTTTTCT 3200
     ----:----|----:----|----:----|----:----|----:----|----:----|----:----|----:----|----:----|----:----|----:----|----:----|----:----|----:----|----:----|----:----|----:----|----:----|----:----|----:----|
3001 CTACCACGGACGAAGAGCCACTAACTATCACACAGCCATAAGTGATCCTTTACCACGGGTGGCCCAGATAGTAGAACTATCGGCGGCATGTGTTCGTCGAACAAACTTGGTTCCATTAAACACCATACAGGACTTTAGTTATGGCCATCAAGACATCGTGGTTGAGCAGAAAGGCGTAATTTACTTTTCAGTCCAAAAGA 3200
       S P A Q K E T I S L T D T N V L F H H G G P R D D Q Y G G Y V L L K N S G L Y N T T H G S I L V P L E T A G V R R E A N F S F D P K R F6
     P H H R S R P S Q Y H T P I * * S I T G V P D I M K I A A T C L C S T Q V L T I Q P I D Q F * Y R Y N Q L V L E D K R M L H F T L N E   F5
      I T G A E R H N I T H R Y E S P F P A W R T * * R S L R R V C A A Q K F W P L K H Y T R F D I G T T R Y C W S T K G C * I F L * T K   F4

      T V T T T C P L I S T S R C T I V I S C I D C L L T P L S S W V R P P I K T M V P S L S V V S V A V * * A S G F R S K R L V H L * F S  F1
      Q L L Q P A H * Y Q H Q D V P L * Y P V * T V S * H H Y H P G * D H Q * R R W C H H Y Q * F L * Q Y D R L V V L D L K D W S I C N F   F2
     H S Y Y N L P T D I N I K M Y H C D I L Y R L S P N T I I I L G E T T N K D D G A I T I S S F C S S M I G * W F * I * K I G P F V I F   F3
3201 CACAGTTACTACAACCTGCCCACTGATATCAACATCAAGATGTACCATTGTGATATCCTGTATAGACTGTCTCCTAACACCATTATCATCCTGGGTGAGACCACCAATAAAGACGATGGTGCCATCACTATCAGTAGTTTCTGTAGCAGTATGATAGGCTAGTGGTTTTAGATCTAAAAGATTGGTCCATTTGTAATTTT 3400
     ----:----|----:----|----:----|----:----|----:----|----:----|----:----|----:----|----:----|----:----|----:----|----:----|----:----|----:----|----:----|----:----|----:----|----:----|----:----|----:----|
3201 GTGTCAATGATGTTGGACGGGTGACTATAGTTGTAGTTCTACATGGTAACACTATAGGACATATCTGACAGAGGATTGTGGTAATAGTAGGACCCACTCTGGTGGTTATTTCTGCTACCACGGTAGTGATAGTCATCAAAGACATCGTCATACTATCCGATCACCAAAATCTAGATTTTCTAACCAGGTAAACATTAAAA 3400
       V T V V V Q G S I D V D L H V M T I D Q I S Q R R V G N D D Q T L G G I F V I T G D S D T T E T A T H Y A L P K L D L L N T W K Y N  F6
      * L * * L R G V S I L M L I Y W Q S I R Y L S D G L V M I M R P S V V L L S S P A M V I L L K Q L L I I P * H N * I * F I P G N T I K  F5
     E C N S C G A W Q Y * C * S T G N H Y G T Y V T E * C W * * G P H S W W Y L R H H W * * * Y N R Y C Y S L S T T K S R F S Q D M Q L K   F4

      K V R K Y N L P S L V L S R P P S D E D C T P P Y I T M P I R R L S L C T L L Q C G H T W P L E C H H S D P * F E P Y N P D P Y H R    F1
     P K * E N I T Y H L L F C H D H H Q M K T A L H H T L Q C P S D D C L Y V P C Y S V G I P G L W N V T I L I H N L S L I I L I P T I G  F2
      Q S E K I * L T I S C F V T T T I R * R L H S T I H Y N A H Q T T V F M Y P V T V W A Y L A S G M S P F * S I I * A L * S * S L P * V F3
3401 CCAAAGTGAGAAAATATAACTTACCATCTCTTGTTTTGTCACGACCACCATCAGATGAAGACTGCACTCCACCATACATTACAATGCCCATCAGACGACTGTCTTTATGTACCCTGTTACAGTGTGGGCATACCTGGCCTCTGGAATGTCACCATTCTGATCCATAATTTGAGCCTTATAATCCTGATCCCTACCATAGG 3600
     ----:----|----:----|----:----|----:----|----:----|----:----|----:----|----:----|----:----|----:----|----:----|----:----|----:----|----:----|----:----|----:----|----:----|----:----|----:----|----:----|
3401 GGTTTCACTCTTTTATATTGAATGGTAGAGAACAAAACAGTGCTGGTGGTAGTCTACTTCTGACGTGAGGTGGTATGTAATGTTACGGGTAGTCTGCTGACAGAAATACATGGGACAATGTCACACCCGTATGGACCGGAGACCTTACAGTGGTAAGACTAGGTATTAAACTCGGAATATTAGGACTAGGGATGGTATCC 3600
      E L T L F Y L K G D R T K D R G G D S S S Q V G G Y M V I G M L R S D K H V R N C H P C V Q G R S H * W E S G Y N S G * L G S G * W L  F6
       W L S F I Y S V M E Q K T V V V M L H L S C E V M C * L A W * V V T K I Y G T V T H A Y R A E P I D G N Q D M I Q A K Y D Q D R G Y   F5
      G F H S F I V * W R K N Q * S W W * I F V A S W W V N C H G D S S Q R * T G Q * L T P M G P R Q F T V M R I W L K L R I I R I G V M P  F4

     Y A T L S G H C E A K W C W G P F P P K Y K Q * F W S R Q H L H G P D D G Q Q S Q F P L A H Q Q S * K L L Q N T E M A S N I F G H L *   F1
      T Q H C Q V I V R Q N G V G V H S P P N I N S S S G P G S T S M G P T T G S S P S F L S H I N S L E N C C R I Q K W P Q T Y L G T C R  F2
      R N T V R S L * G K M V L G S I P P Q I * T V V L V P A A P P W A R R R A A V P V S S R T S T V L K T A A E Y R N G L K H I W A P V   F3
3601 TACGCAACACTGTCAGGTCATTGTGAGGCAAAATGGTGTTGGGGTCCATTCCCCCCCAAATATAAACAGTAGTTCTGGTCCCGGCAGCACCTCCATGGGCCCGACGACGGGCAGCAGTCCCAGTTTCCTCTCGCACATCAACAGTCTTGAAAACTGCTGCAGAATACAGAAATGGCCTCAAACATATTTGGGCACCTGTA 3800
     ----:----|----:----|----:----|----:----|----:----|----:----|----:----|----:----|----:----|----:----|----:----|----:----|----:----|----:----|----:----|----:----|----:----|----:----|----:----|----:----|
3601 ATGCGTTGTGACAGTCCAGTAACACTCCGTTTTACCACAACCCCAGGTAAGGGGGGGTTTATATTTGTCATCAAGACCAGGGCCGTCGTGGAGGTACCCGGGCTGCTGCCCGTCGTCAGGGTCAAAGGAGAGCGTGTAGTTGTCAGAACTTTTGACGACGTCTTATGTCTTTACCGGAGTTTGTATAAACCCGTGGACAT 3800
      Y A V S D P * Q S A F H H Q P G N G G L Y L C Y N Q D R C C R W P G S S P C C D W N G R A C * C D Q F S S C F V S I A E F M N P C R Y F6
      T R L V T L D N H P L I T N P D M G G W I Y V T T R T G A A G G H A R R R A A T G T E E R V D V T K F V A A S Y L F P R L C I Q A G T F5
       V C C Q * T M T L C F P T P T W E G G F I F L L E P G P L V E M P G V V P L L G L K R E C M L L R S F Q Q L I C F H G * V Y K P V Q   F4

      I S F * H I Q D M N F V L L H V K S V V Y Y C S G L C C Q Q K S T M H S T K S R N R N K F T I D V I I V I E I H S T K C R C Y R R T V  F1
      F L S D T F R T * I S F C C M * K V * F I I V P A C A V S K S R P C I P P R A E T V T S S P S M L S L L L K Y I A R S A G V T A V P   F2
     D F F L T H S G H E F R F A A C E K C S L L L F R P V L S A K V D H A F H Q E Q K P * Q V H H R C Y H C Y * N T * H E V P V L P P Y R   F3
3801 GATTTCTTTCTGACACATTCAGGACATGAATTTCGTTTTGCTGCATGTGAAAAGTGTAGTTTATTATTGTTCCGGCCTGTGCTGTCAGCAAAAGTCGACCATGCATTCCACCAAGAGCAGAAACCGTAACAAGTTCACCATCGATGTTATCATTGTTATTGAAATACATAGCACGAAGTGCCGGTGTTACCGCCGTACCG 4000
     ----:----|----:----|----:----|----:----|----:----|----:----|----:----|----:----|----:----|----:----|----:----|----:----|----:----|----:----|----:----|----:----|----:----|----:----|----:----|----:----|
3801 CTAAAGAAAGACTGTGTAAGTCCTGTACTTAAAGCAAAACGACGTACACTTTTCACATCAAATAATAACAAGGCCGGACACGACAGTCGTTTTCAGCTGGTACGTAAGGTGGTTCTCGTCTTTGGCATTGTTCAAGTGGTAGCTACAATAGTAACAATAACTTTATGTATCGTGCTTCACGGCCACAATGGCGGCATGGC 4000
       I E K Q C M * S M F K T K S C T F L T T * * Q E P R H Q * C F D V M C E V L L L F R L L N V M S T I M T I S I C L V F H R H * R R V   F6
      S K K R V C E P C S N R K A A H S F H L K N N N R G T S D A F T S W A N W W S C F G Y C T * W R H * * Q * Q F V Y C S T G T N G G Y R  F5
     L N R E S V N L V H I E N Q Q M H F T Y N I I T G A Q A T L L L R G H M G G L A S V T V L E G D I N D N N N F Y M A R L A P T V A T G   F4

      G F G Q P Q Q M A L A R K * C H V M C I V H Q * L L C S F S A C F Y C F S N R K E A R T V K K I C R V R H V H D V M L C T D Y Q * Q  F1
     S D L V S H N K W P L P E N N A M * C A S S I N D F F V V F R R V F I A F L I E K K R E L S K R S A A S A M F M M * C C V Q I T N D N   F2
     R I W S A T T N G P C Q K I M P C D V H R P S M T S L * F F G V F L L L F * S K R S E N C Q K D L P R P P C S * C D V V Y R L P M T T   F3
4001 TCGGATTTGGTCAGCCACAACAAATGGCCCTTGCCAGAAAATAATGCCATGTGATGTGCATCGTCCATCAATGACTTCTTTGTAGTTTTTCGGCGTGTTTTTATTGCTTTTCTAATCGAAAAGAAGCGAGAACTGTCAAAAAGATCTGCCGCGTCCGCCATGTTCATGATGTGATGTTGTGTACAGATTACCAATGACAA 4200
     ----:----|----:----|----:----|----:----|----:----|----:----|----:----|----:----|----:----|----:----|----:----|----:----|----:----|----:----|----:----|----:----|----:----|----:----|----:----|----:----|
4001 AGCCTAAACCAGTCGGTGTTGTTTACCGGGAACGGTCTTTTATTACGGTACACTACACGTAGCAGGTAGTTACTGAAGAAACATCAAAAAGCCGCACAAAAATAACGAAAAGATTAGCTTTTCTTCGCTCTTGACAGTTTTTCTAGACGGCGCAGGCGGTACAAGTACTACACTACAACACATGTCTAATGGTTACTGTT 4200
     T P N P * G C C I A R A L F Y H W T I H M T W * H S R Q L K E A H K * Q K E L R F S A L V T L F I Q R T R W T * S T I N H V S * W H C   F6
      R I Q D A V V F P G Q W F I I G H S T C R G D I V E K Y N K P T N K N S K * D F L L S F Q * F S R G R G G H E H H S T T Y L N G I V   F5
      D S K T L W L L H G K G S F L A M H H A D D M L S K K T T K R R T K I A K R I S F F R S S D F L D A A D A M N M I H H Q T C I V L S L  F4

     R H N C A C A V L W R * H T S I Y E L S * C S V R M Q E L F D R R C V Q R F R C A H H I A S R R                                           F1
      G T T A R A Q Y C G A D T L R S T N * A D A Q C A C R S Y S I G A V Y K G F G V R T T S H R A X                                          F2
      A Q L R V R S T V A L T H F D L R T K L M L S A H A G V I R * A L C T K V S V C A P H R I A P                                            F3
4201 CGGCACAACTGCGCGTGCGCAGTACTGTGGCGCTGACACACTTCGATCTACGAACTAAGCTGATGCTCAGTGCGCATGCAGGAGTTATTCGATAGGCGCTGTGTACAAAGGTTTCGGTGTGCGCACCACATCGCATCGCGCCG 4343
     ----:----|----:----|----:----|----:----|----:----|----:----|----:----|----:----|----:----|----:----|----:----|----:----|----:----|----:----|---
4201 GCCGTGTTGACGCGCACGCGTCATGACACCGCGACTGTGTGAAGCTAGATGCTTGATTCGACTACGAGTCACGCGTACGTCCTCAATAAGCTATCCGCGACACATGTTTCCAAAGCCACACGCGTGGTGTAGCGTAGCGCGGC 4343
      R C L Q A H A T S H R Q C V E I * S S L Q H E T R M C S N N S L R Q T C L N R H A C W M A D R R                                          F6
     V A C S R T R L V T A S V C K S R R V L S I S L A C A P T I R Y A S H V F T E T H A G C R M A G                                           F5
      P V V A R A C Y Q P A S V S R D V F * A S A * H A H L L * E I P A T Y L P K P T R V V D C R A                                            F4
```

Notospermus geniculatus (Nge)

WGS: NMRB01000732.1

224146-230304
Notospermus geniculatus strain Ushimado scaffold732, whole genome shotgun sequence

```
       L  I  H  N  G  T  A  Q  R  R  E  S  V  F  Q  A  S  S  *  R  F  Q  V  F  A  L  Q  K  L  Q  L  *  F  T  Q  G  D  S  E  K  G  W  T  G  V  A  P  *  R  I  L  P  R  R  R  T  L  Y  V  R  C  S  R  G  E  L  C   F1
       *  S  T  M  A  Q  L  S  E  E  R  V  F  S  R  L  P  L  D  D  F  K  F  L  P  F  K  S  Y  S  Y  D  S  R  K  V  T  R  K  R  A  G  L  E  L  L  H  D  E  Y  Y  P  A  E  G  H  C  M  S  A  V  Q  G  E  N  S  V   F2
        D  P  Q  W  H  S  S  A  K  R  E  C  F  P  G  F  L  L  T  I  S  S  F  C  P  S  K  V  T  V  M  I  H  A  R  *  L  G  K  G  L  D  W  S  C  S  M  T  N  I  T  P  Q  K  D  T  V  C  P  L  F  K  G  R  T  L    F3
   1 TTGATCCACAATGGCACAGCTCAGCGAAGAGAGAGTGTTTTCCAGGCTTCCTCTTGACGATTTCAAGTTTTTGCCCTTCAAAAGTTACAGTTATGATTCACGCAAGGTGACTCGGAAAAGGGCTGGACTGGAGTTGCTCCATGACGAATATTACCCCGCAGAAGGACACTGTATGTCCGCTGTTCAAGGGGAGAACTCTG 200
     ----:----|----:----|----:----|----:----|----:----|----:----|----:----|----:----|----:----|----:----|----:----|----:----|----:----|----:----|----:----|----:----|----:----|----:----|----:----|----:----|
   1 AACTAGGTGTTACCGTGTCGAGTCGCTTCTCTCTCACAAAAGGTCCGAAGGAGAACTGCTAAAGTTCAAAAACGGGAAGTTTTCAATGTCAATACTAAGTGCGTTCCACTGAGCCTTTTCCCGACCTGACCTCAACGAGGTACTGCTTATAATGGGGCGTCTTCCTGTGACATACAGGCGACAAGTTCCCCTCTTGAGAC 200
       X  I  W  L  P  V  A  *  R  L  S  L  T  K  W  A  E  E  Q  R  N  *  T  K  A  R  *  F  N  C  N  H  N  V  C  P  S  E  S  F  P  Q  V  P  T  A  G  H  R  I  N  G  R  L  L  V  S  Y  T  R  Q  E  L  P  S  S  Q   F6
      X  S  G  C  H  C  L  E  A  F  L  S  H  K  G  P  R  K  V  I  E  L  K  Q  G  E  F  T  V  I  I  *  A  L  H  S  P  F  P  S  S  Q  L  Q  E  M  V  F  I  V  G  C  F  S  V  T  H  G  S  N  L  P  L  V  R    F5
     Q  D  V  I  A  C  S  L  S  S  L  T  N  E  L  S  G  R  S  S  K  L  N  K  G  K  L  L  *  L  *  S  E  R  L  T  V  R  F  L  A  P  S  S  N  S  W  S  S  Y  *  G  A  S  P  C  Q  I  D  A  T  *  P  S  F  E    F4

       Y  C  V  L  F  W  W  C  P  T  E  R  *  G  N  L  E  Y  E  Q  Y  H  P  G  N  G  G  D  V  R  *  R  R  C  R  Y  S  H  S  *  N  P  Q  G  G  P  S  A  G  S  N  V  V  Q  E  C  N  I  *  Y  P  T  P  G  H  Y  S   F1
        I  V  Y  F  F  G  G  A  R  R  K  D  E  A  T  W  S  M  N  N  I  I  Q  E  M  V  V  T  Y  D  E  E  D  V  D  I  V  T  V  K  T  L  K  V  G  R  V  R  G  L  M  L  F  K  S  V  T  Y  N  I  R  L  Q  D  T  T    F2
      L  L  C  T  F  L  V  V  P  D  G  K  M  R  Q  L  G  V  *  T  I  S  S  R  K  W  W  *  R  T  M  K  K  M  S  I  *  S  Q  L  K  P  S  R  W  A  E  C  G  V  *  C  C  S  R  V  *  H  I  I  S  D  S  R  T  L  Q   F3
 201 TTATTGTGTACTTTTTTGGTGGTGCCCGACGGAAAGATGAGGCAACTTGGAGTATGAACAATATCATCCAGGAAATGGTGGTGACGTACGATGAAGAAGATGTCGATATAGTCACAGTTAAAACCCTCAAGGTGGGCCGAGTGCGGGGTCTAATGTTGTTCAAGAGTGTAACATATAATATCCGACTCCAGGACACTACA 400
     ----:----|----:----|----:----|----:----|----:----|----:----|----:----|----:----|----:----|----:----|----:----|----:----|----:----|----:----|----:----|----:----|----:----|----:----|----:----|----:----|
 201 AATAACACATGAAAAAACCACCACGGGCTGCCTTTCTACTCCGTTGAACCTCATACTTGTTATAGTAGGTCCTTTACCACCACTGCATGCTACTTCTTCTACAGCTATATCAGTGTCAATTTTGGGAGTTCCACCCGGCTCACGCCCCAGATTACAACAAGTTCTCACATTGTATATTATAGGCTGAGGTCCTGTGATGT 400
       *  Q  T  S  K  Q  H  H  G  V  S  L  H  P  L  K  S  Y  S  C  Y  *  G  P  F  P  P  S  T  R  H  L  L  H  R  Y  L  *  L  *  F  G  *  P  P  G  L  A  P  D  L  T  T  *  S  H  L  M  Y  Y  G  V  G  P  C  *   F6
      N  N  H  V  K  K  T  T  G  S  P  F  I  L  C  S  P  T  H  V  I  D  D  L  F  H  H  H  R  V  I  F  F  I  D  I  Y  D  C  N  F  G  E  L  H  A  S  H  P  T  *  H  Q  E  L  T  Y  C  I  I  D  S  E  L  V  S  C    F5
     T  I  T  Y  K  K  P  P  A  R  R  F  S  S  A  V  Q  L  I  F  L  I  M  W  S  I  T  T  V  Y  S  S  S  S  T  S  I  T  V  T  L  V  R  L  T  P  R  T  R  P  R  I  N  N  L  L  T  V  Y  L  I  R  S  W  S  V  V    F4

       S  E  *  Q  C  S  A  W  T  A  S  H  G  C  P  S  I  F  G  A  N  I  G  V  A  C  I  L  Q  F  L  V  K  R  K  H  V  K  N  V  F  I  W  Y  L  S  F  L  F  I  L  L  *  Y  R  Y  T  S  L  L  F  H  L  F  I  H   F1
      V  V  S  N  S  A  A  H  G  R  P  L  M  G  A  H  P  F  L  V  P  I  *  E  W  H  A  Y  S  N  S  L  *  K  E  N  M  S  K  M  Y  S  F  G  I  Y  L  F  Y  S  Y  C  Y  N  T  G  I  H  L  F  Y  F  I  S  S  F  T    F2
      *  *  V  T  V  Q  R  M  D  G  L  S  W  V  P  I  H  F  W  C  Q  Y  R  S  G  M  H  T  P  I  P  C  E  K  K  T  C  Q  K  C  I  H  L  V  F  I  F  F  I  H  I  V  I  I  P  V  Y  I  S  F  I  S  S  L  H  S  P   F3
 401 GTAGTGAGTAACAGTGCAGCGCATGGACGGCCTCTCATGGGTGCCCATCCATTTTTGGTGCCAATATAGGAGTGGCATGCATACTCCAATTCCTTGTGAAAAGAAAACATGTCAAAAATGTATTCATTTGGTATTTATCTTTTTTATTCATATTGTTATAATACCGGTATACATCTCTTTTATTTCATCTCTTCATTCAC 600
     ----:----|----:----|----:----|----:----|----:----|----:----|----:----|----:----|----:----|----:----|----:----|----:----|----:----|----:----|----:----|----:----|----:----|----:----|----:----|----:----|
 401 CATCACTCATTGTCACGTCGCGTACCTGCCGGAGAGTACCCACGGGTAGGTAAAAACCACGGTTATATCCTCACCGTACGTATGAGGTTAAGGAACACTTTTCTTTTGTACAGTTTTTACATAAGTAAACCATAAATAGAAAAAATAAGTATAACAATATTATGGCCATATGTAGAGAAAATAAAGTAGAGAAGTAAGTG 600
       L  L  S  Y  C  H  L  A  H  V  A  E  *  P  H  G  D  M  K  P  A  L  I  P  T  A  H  M  S  W  N  R  T  F  L  F  C  T  L  F  T  N  M  Q  Y  K  D  K  K  N  M  N  N  Y  Y  R  Y  V  D  R  K  N  *  R  K  M  *   F6
      Y  H  T  V  T  C  R  M  S  P  R  E  H  T  G  M  W  K  Q  H  W  Y  L  L  P  M  C  V  G  I  G  Q  S  F  F  V  H  *  F  H  I  *  K  T  N  I  K  K  I  *  I  T  I  I  G  T  Y  M  E  K  I  E  D  R  *  E    F5
     T  T  L  L  L  A  A  C  P  R  G  R  M  P  A  W  G  N  K  T  G  I  Y  S  H  C  A  Y  E  L  E  K  H  F  S  F  M  D  F  I  Y  E  N  P  I  *  R  K  *  E  Y  Q  *  L  V  P  I  C  R  K  *  K  M  E  E  N  V    F4

       L  I  I  Y  I  P  V  I  H  S  Y  S  Q  S  L  Y  R  V  A  I  R  L  F  I  D  I  S  F  N  H  S  R  I  H  L  F  Y  S  Y  V  Y  T  I  K  Q  S  I  S  I  *  I  H  L  A  V  R  Y  I  F  I  F  S  S  L  L  Q  W   F1
        L  L  S  T  Y  P  S  F  T  L  I  H  N  L  Y  T  G  W  Q  F  V  S  L  *  T  F  L  L  I  I  A  E  F  I  S  F  I  H  M  F  I  P  L  S  N  P  Y  L  F  K  F  I  L  R  S  A  I  S  L  Y  S  H  L  F  C  N  G    F2
      Y  Y  L  H  T  R  H  S  L  L  F  T  I  F  I  P  G  G  N  S  S  L  Y  R  H  F  F  *  S  *  Q  N  S  S  L  L  F  I  C  L  Y  H  *  A  I  H  I  Y  L  N  S  S  C  G  P  L  Y  L  Y  I  L  I  S  F  A  M   F3
 601 CTTATTATCTACATACCCGTCATTCACTCTTATTCACAATCTTTATACCGGGTGGCAATTCGTCTCTTTATAGACATTTCTTTTAATCATAGCAGAATTCATCTCTTTTATTCATATGTTTATACCATTAAGCAATCCATATCTATTTAAATTCATCTTGCGGTCCGCTATATCTTTATATTCTCATCTCTTTTGCAATG 800
     ----:----|----:----|----:----|----:----|----:----|----:----|----:----|----:----|----:----|----:----|----:----|----:----|----:----|----:----|----:----|----:----|----:----|----:----|----:----|----:----|
 601 GAATAATAGATGTATGGGCAGTAAGTGAGAATAAGTGTTAGAAATATGGCCCACCGTTAAGCAGAGAAATATCTGTAAAGAAAATTAGTATCGTCTTAAGTAGAGAAAATAAGTATACAAATATGGTAATTCGTTAGGTATAGATAAATTTAAGTAGAACGCCAGGCGATATAGAAATATAAGAGTAGAGAAAACGTTAC 800
       R  I  *  M  G  T  M  *  E  *  E  C  D  K  Y  R  T  A  I  R  R  K  I  S  M  E  K  L  *  L  L  I  *  R  K  *  E  Y  T  *  V  M  L  C  D  M  D  I  *  I  *  R  A  T  R  *  I  K  I  N  E  D  R  K  C  H   F6
      G  *  *  R  C  V  R  *  E  S  K  N  V  I  K  I  G  P  P  L  E  D  R  *  L  C  K  K  *  D  Y  C  F  E  D  R  K  N  M  H  K  Y  W  *  A  I  W  I  *  K  F  E  D  Q  P  G  S  Y  R  *  I  R  M  E  K  A  I    F5
     K  N  D  V  Y  G  D  N  V  R  I  *  L  R  *  V  P  H  C  N  T  E  K  Y  V  N  R  K  I  M  A  S  N  M  E  K  I  *  I  N  I  G  N  L  L  G  Y  R  N  L  N  M  K  R  D  A  I  D  K  Y  E  *  R  K  Q  L    F4

       K  E  Y  T  V  Q  N  H  N  F  *  L  T  G  V  S  Y  Y  D  G  A  A  S  V  V  R  R  R  P  S  S  S  S  S  S  V  V  V  V  V  V  V  R  *  Q  G  T  F  R  N  R  W  S  Y  *  S  E  T  W  Y  I  Y  T  P  R  S  D   F1
        K  S  T  Q  F  K  I  T  I  F  S  S  L  G  *  A  I  T  M  V  Q  R  P  S  S  V  V  V  R  R  R  R  R  P  S  S  S  S  S  S  L  V  S  R  A  R  F  V  T  A  G  A  I  D  L  K  L  G  T  Y  I  P  L  G  Q    F2
      E  R  V  H  S  S  K  S  Q  F  L  A  H  W  G  E  L  L  R  W  C  S  V  R  R  P  S  S  S  V  V  V  V  V  V  V  R  R  R  R  R  R  C  P  L  A  G  H  V  S  *  P  L  E  L  L  I  *  N  L  V  H  I  Y  P  *  V  R   F3
 801 GAAGAGTACACAGTTCAAAATCACAATTTTTAGCTCACTGGGGTGAGCTATTACGATGGTGCAGCGTCCGTCGTCCGTCGTCGTCCGTCGTCGTCGTCGTCGTCGTCCGTCGTCGTCGTCGTTGTCCGTTAGCAGGGCACGTTTCGTAACCGCTGGAGCTATTGATCTGAAACTTGGTACATATATACCCCTAGGTCAG 1000
     ----:----|----:----|----:----|----:----|----:----|----:----|----:----|----:----|----:----|----:----|----:----|----:----|----:----|----:----|----:----|----:----|----:----|----:----|----:----|----:----|
 801 CTTCTCATGTGTCAAGTTTTAGTGTTAAAAATCGAGTGACCCCACTCGATAATGCTACCACGTCGCAGGCAGCAGGCAGCAGCAGGCAGCAGCAGCAGCAGCAGCAGCAGGCAGCAGCAACAGGCAATCGTCCCGTGCAAAGCATTGGCGACCTCGATAACTAGACTTTGAACCATGTATATATGGGGATCCAGTC 1000
       F  S  Y  V  T  *  F  *  L  K  *  S  V  P  T  L  *  *  S  P  A  A  D  T  T  R  R  R  G  D  D  D  D  D  D  T  T  T  T  T  T  T  R  *  C  P  V  N  R  L  R  Q  L  *  Q  D  S  V  Q  Y  M  Y  V  G  L  D   F6
      S  L  T  C  L  E  F  D  C  N  K  A  *  Q  P  S  S  N  R  H  H  L  T  R  R  G  D  D  D  T  T  T  T  T  T  T  R  R  R  R  R  R  Q  G  N  A  P  C  T  E  Y  G  S  S  S  N  I  Q  F  K  T  C  I  Y  G  *  T  L    F5
     P  F  L  V  C  N  L  I  V  I  K  L  E  S  P  H  A  I  V  I  T  C  R  G  D  D  T  T  T  R  R  R  R  R  G  D  D  D  D  D  N  D  T  L  L  A  R  K  T  V  A  P  A  I  S  R  F  S  P  V  Y  I  G  R  P  *    F4

       D  P  R  H  Q  V  S  V  R  F  D  S  Q  F  G  H  Q  V  A  E  T  K  N  T  K  S  T  I  T  P  E  L  M  T  R  S  S  S  N  F  Y  H  R  C  S  *  *  G  Y  I  T  Y  I  T  G  F  *  F  D  L  L  F  K  V  T  E   F1
        M  T  H  D  T  K  F  R  S  D  S  T  L  S  L  A  T  R  W  Q  K  P  K  T  Q  K  V  Q  *  L  L  N  *  *  L  D  H  L  Q  I  F  I  I  G  V  A  N  K  D  T  *  H  I  *  R  V  F  D  L  T  Y  F  S  R  S  Q  R    F2
      *  P  T  T  P  S  G  F  P  I  R  L  S  V  W  P  P  G  G  R  N  Q  K  H  K  K  Y  N  N  S  *  T  N  D  *  I  I  F  K  F  L  S  *  V  *  L  I  R  I  H  N  I  Y  N  G  F  L  I  *  P  T  F  Q  G  H  R  G   F3
1001 ATGACCCACGACACCAAGTTTCGGTCCGATTCGACTCTCAGTTTGGCCACCAGGTGGCAGAAACCAAAAACACAAAAAGTACAATAACTCCTGAACTAATGACTAGATCATCTTCAAATTTTTATCATAGGTGTAGCTAATAAGGATACATAACATATATAACGGGTTTTTGATTTGACCTACTTTTCAAGGTCACAGAG 1200
     ----:----|----:----|----:----|----:----|----:----|----:----|----:----|----:----|----:----|----:----|----:----|----:----|----:----|----:----|----:----|----:----|----:----|----:----|----:----|----:----|
1001 TACTGGGTGCTGTGGTTCAAAGCCAGGCTAAGCTGAGAGTCAAACCGGTGGTCCACCGTCTTTGGTTTTTGTGTTTTTCATGTTATTGAGGACTTGATTACTGATCTAGTAGAAGTTTAAAAATAGTATCCACATCGATTATTCCTATGTATTGTATATATTGCCCAAAAACTAAACTGGATGAAAAGTTCCAGTGTCTC 1200
       S  S  G  R  C  W  T  E  T  R  N  S  E  *  N  P  W  W  T  A  S  V  L  F  V  F  L  V  I  V  G  S  S  I  V  L  D  D  E  F  K  *  L  H  L  *  Y  P  Y  M  V  Y  I  V  P  K  Q  N  S  R  S  K  L  T  V  S   F6
      H  G  V  V  G  L  K  P  G  I  R  S  E  T  G  S  G  P  P  L  F  W  F  C  L  F  Y  L  L  E  Q  V  L  S  *  I  M  K  L  N  K  D  Y  T  Y  S  I  L  I  C  L  M  Y  L  P  N  K  I  Q  G  V  K  *  P  *  L    F5
     I  V  W  S  V  L  N  R  D  S  E  V  R  L  K  A  V  L  H  C  F  G  F  V  C  F  T  C  Y  S  R  F  *  H  S  S  *  R  *  I  K  I  M  P  T  A  L  L  S  V  Y  C  I  Y  R  T  K  S  K  V  *  K  E  L  D  C  L    F4

       V  K  V  R  Q  K  *  R  T  C  H  S  L  S  L  F  H  R  A  T  S  Y  L  A  *  T  C  I  G  G  V  P  *  K  I  P  S  F  G  P  I  R  H  P  I  W  P  P  G  G  V  T  X  X  X  X  X  X  X  X  X  X  X  X  X  X   F1
        S  K  F  D  K  S  D  E  P  A  T  V  C  H  Y  F  T  E  Q  L  H  I  L  H  K  H  A  L  E  G  Y  L  K  K  Y  Q  V  S  A  R  F  D  I  Q  Y  G  R  Q  V  A  *  P  X  X  X  X  X  X  X  X  X  X  X  X  X  X    F2
      Q  S  S  T  K  V  T  N  L  P  Q  F  V  I  I  S  P  S  N  F  I  S  C  I  N  M  H  W  R  G  T  L  K  N  T  K  F  R  P  D  S  T  S  N  M  A  A  R  W  R  N  X  X  X  X  X  X  X  X  X  X  X  X  X  X  X   F3
1201 GTCAAAGTTCGACAAAAGTGACGAACCTGCCACAGTTTGTCATTATTTCACCGAGCAACTTCATATCTTGCATAAACATGCATTGGAGGGGTACCTTAAAAAATACCAAGTTTCGGCCCGATTCGACATCCAATATGGCCGCCAGGTGGCGTAACCNNNNNNNNNNNNNNNNNNNNNNNNNNNNNNNNNNNNNNNNNNNN 1400
     ----:----|----:----|----:----|----:----|----:----|----:----|----:----|----:----|----:----|----:----|----:----|----:----|----:----|----:----|----:----|----:----|----:----|----:----|----:----|----:----|
1201 CAGTTTCAAGCTGTTTTCACTGCTTGGACGGTGTCAAACAGTAATAAAGTGGCTCGTTGAAGTATAGAACGTATTTGTACGTAACCTCCCCATGGAATTTTTTATGGTTCAAAGCCGGGCTAAGCTGTAGGTTATACCGGCGGTCCACCGCATTGGNNNNNNNNNNNNNNNNNNNNNNNNNNNNNNNNNNNNNNNNNNNN 1400
       T  L  T  R  C  F  H  R  V  Q  W  L  K  D  N  N  *  R  A  V  E  Y  R  A  Y  V  H  M  P  P  T  G  *  F  I  G  L  K  P  G  I  R  C  G  I  H  G  G  P  P  T  V  X  X  X  X  X  X  X  X  X  X  X  X  X  X   F6
      P  *  L  E  V  F  T  V  F  R  G  C  N  T  M  I  E  G  L  L  K  M  D  Q  M  F  M  C  Q  L  P  V  K  F  F  V  L  N  R  G  S  E  V  D  L  I  A  A  L  H  R  L  X  X  X  X  X  X  X  X  X  X  X  X  X  X    F5
     D  F  N  S  L  L  S  S  G  A  V  T  Q  *  *  K  V  S  C  S  *  I  K  C  L  C  A  N  S  P  Y  R  L  F  Y  W  T  E  A  R  N  S  M  W  Y  P  R  W  T  A  Y  G  X  X  X  X  X  X  X  X  X  X  X  X  X     F4

       X  X  X  X  X  X  X  X  X  X  X  X  X  X  X  X  X  X  X  X  X  X  X  X  X  X  X  X  X  X  X  X  X  X  X  X  X  X  X  X  X  X  X  X  X  X  X  X  X  X  X  X  X  X  X  X  X  X  X  X  X  X  X  X  X  X   F1
        X  X  X  X  X  X  X  X  X  X  X  X  X  X  X  X  X  X  X  X  X  X  X  X  X  X  X  X  X  X  X  X  X  X  X  X  X  X  X  X  X  X  X  X  X  X  X  X  X  X  X  X  X  X  X  X  X  X  X  X  X  X  X  X  X    F2
      X  X  X  X  X  X  X  X  X  X  X  X  X  X  X  X  X  X  X  X  X  X  X  X  X  X  X  X  X  X  X  X  X  X  X  X  X  X  X  X  X  X  X  X  X  X  X  X  X  X  X  X  X  X  X  X  X  X  X  X  X  X  X  X  X  X   F3
1401 NNNNNNNNNNNNNNNNNNNNNNNNNNNNNNNNNNNNNNNNNNNNNNNNNNNNNNNNNNNNNNNNNNNNNNNNNNNNNNNNNNNNNNNNNNNNNNNNNNNNNNNNNNNNNNNNNNNNNNNNNNNNNNNNNNNNNNNNNNNNNNNNNNNNNNNNNNNNNNNNNNNNNNNNNNNNNNNNNNNNNNNNNNNNNNNNNNNNN 1600
     ----:----|----:----|----:----|----:----|----:----|----:----|----:----|----:----|----:----|----:----|----:----|----:----|----:----|----:----|----:----|----:----|----:----|----:----|----:----|----:----|
1401 NNNNNNNNNNNNNNNNNNNNNNNNNNNNNNNNNNNNNNNNNNNNNNNNNNNNNNNNNNNNNNNNNNNNNNNNNNNNNNNNNNNNNNNNNNNNNNNNNNNNNNNNNNNNNNNNNNNNNNNNNNNNNNNNNNNNNNNNNNNNNNNNNNNNNNNNNNNNNNNNNNNNNNNNNNNNNNNNNNNNNNNNNNNNNNNNNNNNNN 1600
       X  X  X  X  X  X  X  X  X  X  X  X  X  X  X  X  X  X  X  X  X  X  X  X  X  X  X  X  X  X  X  X  X  X  X  X  X  X  X  X  X  X  X  X  X  X  X  X  X  X  X  X  X  X  X  X  X  X  X  X  X  X  X  X  X  X   F6
      X  X  X  X  X  X  X  X  X  X  X  X  X  X  X  X  X  X  X  X  X  X  X  X  X  X  X  X  X  X  X  X  X  X  X  X  X  X  X  X  X  X  X  X  X  X  X  X  X  X  X  X  X  X  X  X  X  X  X  X  X  X  X  X  X  X    F5
     X  X  X  X  X  X  X  X  X  X  X  X  X  X  X  X  X  X  X  X  X  X  X  X  X  X  X  X  X  X  X  X  X  X  X  X  X  X  X  X  X  X  X  X  X  X  X  X  X  X  X  X  X  X  X  X  X  X  X  X  X  X  X  X  X  X    F4

       X  X  X  X  X  X  X  X  X  X  X  X  X  X  X  A  L  T  Q  S  A  P  M  C  Y  V  A  P  C  R  *  P  L  C  P  C  E  V  S  A  R  S  D  I  Q  Y  G  R  Q  V  A  *  L  E  N  H  Q  S  A  V  T  Q  E  R  L  D  P   F1
        X  X  X  X  X  X  X  X  X  X  X  X  X  X  X  M  P  *  P  K  A  L  Q  C  A  M  W  P  P  A  D  D  L  Y  V  H  V  K  F  R  P  D  R  T  S  N  M  A  A  R  W  R  N  S  K  I  I  K  V  L  *  L  R  N  D  L  T  Q    F2
      X  X  X  X  X  X  X  X  X  X  X  X  X  X  X  C  L  N  P  K  R  S  N  V  L  C  G  P  L  Q  M  T  F  M  S  M  *  S  F  G  P  I  G  H  P  I  W  P  P  G  G  V  T  R  K  S  S  K  C  C  N  S  G  T  T  *  P  K   F3
1601 NNNNNNNNNNNNNNNNNNNNNNNNNNNNNNNNNNNNNNNNNNNATGCCTTAACCCAAAGCGCTCCAATGTGCTATGTGGCCCCCTGCAGATGACCTTTATGTCCATGTGAAGTTTCGGCCCGATCGGACATCCAATATGGCCGCCAGGTGGCGTAACTCGAAAATCATCAAAGTGCTGTAACTCAGGACGACTTGACCCA 1800
     ----:----|----:----|----:----|----:----|----:----|----:----|----:----|----:----|----:----|----:----|----:----|----:----|----:----|----:----|----:----|----:----|----:----|----:----|----:----|----:----|
1601 NNNNNNNNNNNNNNNNNNNNNNNNNNNNNNNNNNNNNNNNNNNTACGGAATTGGGTTTCGCGAGGTTACACGATACACCGGGGGACGTCTACTGGAAATACAGGTACACTTCAAAGCCGGGCTAGCCTGTAGGTTATACCGGCGGTCCACCGCATTGAGCTTTTAGTAGTTTCACGACATTGAGTCCTTGCTGAACTGGGT 1800
       X  X  X  X  X  X  X  X  X  X  X  X  X  X  X  A  K  V  W  L  A  G  I  H  *  T  A  G  Q  L  H  G  K  H  G  H  S  T  E  A  R  D  S  M  W  Y  P  R  W  T  A  Y  S  S  F  *  L  A  T  V  *  S  R  S  S  G   F6
      X  X  X  X  X  X  X  X  X  X  X  X  X  X  X  H  R  L  G  F  R  E  L  T  S  H  P  G  R  C  I  V  K  I  D  M  H  L  K  P  G  I  P  C  G  I  H  G  G  P  P  T  V  R  F  D  D  F  H  Q  L  E  P  V  V  Q  G    F5
     X  X  X  X  X  X  X  X  X  X  X  X  X  X  G  *  G  L  A  S  W  H  A  I  H  G  G  A  S  S  R  *  T  W  T  F  N  R  G  S  R  V  D  L  I  A  A  L  H  R  L  E  F  I  M  L  T  S  Y  S  L  F  S  K  V  W    F4

       K  C  S  N  V  L  C  H  V  C  G  P  L  Q  M  T  F  M  C  M  *  S  C  S  P  I  G  H  L  I  W  P  P  G  G  I  T  Q  K  S  S  V  F  S  G  N  D  L  T  Q  I  A  H  N  *  Y  V  P  P  S  V  I  R  L  L  C  P   F1
        S  A  P  M  C  Y  V  M  Y  V  V  P  C  R  *  P  L  C  A  C  E  V  A  A  Q  S  D  I  *  Y  G  R  Q  V  A  *  P  K  N  H  Q  S  F  L  G  M  T  *  P  R  L  L  I  T  D  M  Y  L  P  L  S  L  D  Y  Y  V  Q    F2
      V  L  Q  C  A  M  S  C  M  W  S  P  A  D  D  L  Y  V  H  V  K  L  Q  P  N  R  T  S  N  M  A  A  R  W  H  N  P  K  I  I  S  L  F  W  E  *  L  D  P  D  C  S  *  L  I  C  T  S  L  C  H  *  T  I  M  S   F3
1801 AAGTGCTCCAATGTGCTATGTCATGTATGTGGTCCCCTGCAGATGACCTTTATGTGCATGTGAAGTTGCAGCCCAATCGGACATCTAATATGGCCGCCAGGTGGCATAACCCAAAAATCATCAGTCTTTTCTGGGAATGACTTGACCCAGATTGCTCATAACTGATATGTACCTCCCTCTGTCATTAGACTATTATGTCC 2000
     ----:----|----:----|----:----|----:----|----:----|----:----|----:----|----:----|----:----|----:----|----:----|----:----|----:----|----:----|----:----|----:----|----:----|----:----|----:----|----:----|
1801 TTCACGAGGTTACACGATACAGTACATACACCAGGGGACGTCTACTGGAAATACACGTACACTTCAACGTCGGGTTAGCCTGTAGATTATACCGGCGGTCCACCGTATTGGGTTTTTAGTAGTCAGAAAAGACCCTTACTGAACTGGGTCTAACGAGTATTGACTATACATGGAGGGAGACAGTAATCTGATAATACAGG 2000
       F  H  E  L  T  S  H  *  T  H  P  G  R  C  I  V  K  I  H  M  H  L  Q  L  G  I  P  C  R  I  H  G  G  P  P  M  V  W  F  D  D  T  K  E  P  F  S  K  V  W  I  A  *  L  Q  Y  T  G  G  E  T  M  L  S  N  H  G   F6
      L  T  S  W  H  A  I  D  H  I  H  D  G  A  S  S  R  *  T  C  T  F  N  C  G  L  R  V  D  L  I  A  A  L  H  C  L  G  F  I  M  L  R  K  Q  S  H  S  S  G  S  Q  E  Y  S  I  H  V  E  R  Q  *  *  V  I  I  D    F5
     L  A  G  I  H  *  T  M  Y  T  T  G  Q  L  H  G  K  H  A  H  S  T  A  A  W  D  S  M  *  Y  P  R  W  T  A  Y  G  L  F  *  *  D  K  R  P  I  V  Q  G  L  N  S  M  V  S  I  Y  R  G  R  D  N  S  *  *  T    F4

       T  Q  *  A  H  G  P  W  T  I  S  L  Y  I  C  H  C  Y  V  V  L  V  H  F  R  Q  L  V  L  K  S  L  V  C  N  Q  L  D  F  W  P  F  Q  I  I  N  A  S  P  I  S  S  S  G  V  V  S  I  *  L  I  *  K  R  R  M  S   F1
        P  S  E  H  M  V  H  G  P  F  H  Y  T  F  V  I  A  M  L  F  W  F  I  S  D  N  W  C  *  S  H  W  S  A  I  S  W  I  F  G  H  S  K  *  *  M  Q  A  R  F  R  H  L  G  W  F  Q  Y  D  S  F  E  N  G  E  *    F2
      N  P  V  S  T  W  S  M  D  H  F  I  I  H  L  S  L  L  C  C  S  G  S  F  Q  T  T  G  A  E  V  T  G  L  Q  S  A  G  F  L  A  I  P  N  N  K  C  K  P  D  F  V  I  W  G  G  F  N  M  T  H  L  K  T  E  N  E   F3
2001 AACCCAGTGAGCACATGGTCCATGGACCATTTCATTATACATTTGTCATTGCTATGTTGTTCTGGTTCATTTCAGACAACTGGTGCTGAAGTCACTGGTCTGCAATCAGCTGGATTTTTGGCCATTCCAAATAATAAATGCAAGCCCGATTTCGTCATCTGGGGTGGTTTCAATATGACTCATTTGAAAACGGAGAATGA 2200
     ----:----|----:----|----:----|----:----|----:----|----:----|----:----|----:----|----:----|----:----|----:----|----:----|----:----|----:----|----:----|----:----|----:----|----:----|----:----|----:----|
2001 TTGGGTCACTCGTGTACCAGGTACCTGGTAAAGTAATATGTAAACAGTAACGATACAACAAGACCAAGTAAAGTCTGTTGACCACGACTTCAGTGACCAGACGTTAGTCGACCTAAAAACCGGTAAGGTTTATTATTTACGTTCGGGCTAAAGCAGTAGACCCCACCAAAGTTATACTGAGTAAACTTTTGCCTCTTACT 2200
       V  W  H  A  C  P  G  H  V  M  E  N  Y  M  Q  *  Q  *  T  T  R  T  *  K  L  C  S  T  S  F  D  S  T  Q  L  *  S  S  K  Q  G  N  W  I  I  F  A  L  G  I  E  D  D  P  T  T  E  I  H  S  M  Q  F  R  L  I   F6
      L  G  T  L  V  H  D  M  S  W  K  M  I  C  K  D  N  S  H  Q  E  P  E  N  *  V  V  P  A  S  T  V  P  R  C  D  A  P  N  K  A  M  G  F  L  L  H  L  G  S  K  T  M  Q  P  P  K  L  I  V  *  K  F  V  S  F  S    F5
     W  G  L  S  C  M  T  W  P  G  N  *  *  V  N  T  M  A  I  N  N  Q  N  M  E  S  L  Q  H  Q  L  *  Q  D  A  I  L  Q  I  K  P  W  E  L  Y  Y  I  C  A  R  N  R  *  R  P  H  N  *  Y  S  E  N  S  F  P  S  H    F4

       S  C  I  *  D  T  *  K  K  S  R  S  T  T  V  Q  Y  S  R  T  Q  T  K  S  S  N  W  T  Q  M  F  I  S  L  G  K  S  L  Q  A  E  P  A  T  H  L  L  K  Y  P  A  P  D  W  L  R  C  L  V  V  W  R  *  D  S  G   F1
        A  L  A  F  K  I  P  E  K  R  A  E  A  P  L  Y  N  I  Q  G  P  K  R  K  V  Q  T  G  H  R  C  L  S  V  W  G  S  P  F  K  P  N  R  P  L  I  Y  S  N  I  R  H  Q  I  G  C  A  V  W  W  F  G  D  E  I  Q  G    F2
      L  L  H  L  R  Y  L  K  K  E  Q  K  H  H  C  T  I  F  K  D  P  N  E  K  F  K  L  D  T  D  V  Y  Q  S  G  E  V  P  S  S  R  T  G  H  S  F  T  Q  I  S  G  T  R  L  V  A  L  F  G  G  L  E  M  R  F  R  E   F3
2201 GCTCTTGCATTTAAGATACCTGAAAAAAGAGCAGAAGCACCACTGTACAATATTCAAGGACCCAAACGAAAAGTTCAAACTGGACACAGATGTTTATCAGTCTGGGGAAGTCCCTTCAAGCCGAACCGGCCACTCATTTACTCAAATATCCGGCACCAGATTGGTTGCGCTGTTTGGTGGTTTGGAGATGAGATTCAGGG 2400
     ----:----|----:----|----:----|----:----|----:----|----:----|----:----|----:----|----:----|----:----|----:----|----:----|----:----|----:----|----:----|----:----|----:----|----:----|----:----|----:----|
2201 CGAGAACGTAAATTCTATGGACTTTTTTCTCGTCTTCGTGGTGACATGTTATAATTCCTGGGTTTGCTTTTCAAGTTTGACCTGTGTCTACAAATAGTCAGACCCCTTCAGGGAAGTTCGGCTTGGCCGGTGAGTAAATGAGTTTATAGGCCGTGGTCTAACCAACGCGACAAACCACCAAACCTCTACTCTAAGTCCC 2400
       L  E  Q  M  *  S  V  Q  F  F  L  L  V  V  T  C  Y  E  L  V  W  V  F  L  E  F  Q  V  C  I  N  I  L  R  P  F  D  R  *  A  S  G  A  V  *  K  S  L  Y  G  A  G  S  Q  N  R  Q  K  T  T  Q  L  H  S  E  P   F6
      S  K  C  K  L  Y  R  F  F  S  C  F  C  W  Q  V  I  N  L  S  G  F  S  F  N  L  S  S  V  S  T  *  *  D  P  S  T  G  E  L  R  V  P  W  E  N  V  *  I  D  P  V  L  N  T  A  S  N  P  P  K  S  I  L  N  L    F5
     A  R  A  N  L  I  G  S  F  L  A  S  A  G  S  Y  L  I  *  P  G  L  R  F  T  *  V  P  C  L  H  K  D  T  Q  P  L  G  K  L  G  F  R  G  S  M  *  E  F  I  R  C  W  I  P  Q  A  T  Q  H  N  P  S  S  I  *  P    F4
```

```
S D L Q L Q H Q P L A H L R R P V K M L T S M F * I W Q K T N G Q S * K H L K S Q P G V T I Q V L S L K F R G D R Q L Q L L E G L S M F1
A I C S Y N T N P * P I Y A D L S R C S L L C F E F G R K Q M D K A E N T * N H S Q V L P F R Y F H * N S G A T D N C N C W R D * V * F2
R S A V T T P T L S P F T Q T C Q D A H F Y V L N L A E N K W T K L K T P E I T A R C Y H S G T F I E I Q G R Q T I A I V G G I E Y F3
2401 AGCGATCTGCAGTTACAACACCAACCCTTAGCCCATTTACGCAGACCTGTCAAGATGCTCACTTCTATGTTTTGAATTTGGCAGAAAACAAATGGACAAAGCTGAAAACACCTGAAATCACAGCCAGGTGTTACCATTCAGGTACTTTCATTGAAATTCAGGGGCGACAGACAATTGCAATTGTTGGAGGGATTGAGTAT 2600
----:----|----:----|----:----|----:----|----:----|----:----|----:----|----:----|----:----|----:----|----:----|----:----|----:----|----:----|----:----|----:----|----:----|----:----|----:----|----:----|
2401 TCGCTAGACGTCAATGTTGTGGTTGGGAATCGGGTAAATGCGTCTGGACAGTTCTACGAGTGAAGATACAAAACTTAAACCGTCTTTTGTTTACCTGTTTCGACTTTTGTGGACTTTAGTGTCGGTCCACAATGGTAAGTCCATGAAAGTAACTTTAAGTCCCCGCTGTCTGTTAACGTTAACAACCTCCCTAACTCATA 2600
L S R C N C C W G K A W K R L G T L I S V E I N Q I Q C F V F P C L Q F C R F D C G P T V M * T S E N F N L P S L C N C N N S P N L I F6
S R D A T V V G V R L G N V C V Q * S A * K * T K F K A S F L H V F S F V G S I V A L H * W E P V K M S I * P R C V I A I T P P I S Y F5
A I Q L * L V L G * G M * A S R D L H E S R H K S N P L F C I S L A S F V Q F * L W T N G N L Y K * Q F E P A V S L Q L Q Q L S Q T F4

M G V H L A K G F P W M K * S F S P F * I * T L W M L Q Y K I L S F P L D L T N V S Y P I T A V V N * K T T W * Y L E A F S S I Q W N F1
W E C T L P K V S P G * N N H S L L F R F R H S G C C N T K Y C H S P W T * Q M F L I L S Q L * * I R K L L G D I W R L S A V Y N G F2
D G S A P C Q R F P L D E I I I L S F L D L D T L D V A I Q N I V I P L G P D K C F L S Y H S C S K L E N Y L V I F G G F Q Q Y T M E F3
2601 GATGGGAGTGCACCTTGCCAAAGGTTTCCCCTGGATGAAATAATCATTCTCTCCTTTTTAGATTTAGACACTCTGGATGTTGCAATACAAAATATTGTCATTCCCCTTGGACCTGACAAATGTTTCTTATCCTATCACAGCTGTAGTAAATTAGAAAACTACTTGGTGATATTTGGAGGCTTTCAGCAGTATACAATGGA 2800
----:----|----:----|----:----|----:----|----:----|----:----|----:----|----:----|----:----|----:----|----:----|----:----|----:----|----:----|----:----|----:----|----:----|----:----|----:----|----:----|
2601 CTACCCTCACGTGGAACGGTTTCCAAAGGGGACCTACTTTATTAGTAAGAGAGGAAAAATCTAAATCTGTGAGACCTACAACGTTATGTTTTATAACAGTAAGGGGAACCTGGACTGTTTACAAAGAATAGGATAGTGTCGACATCATTTAATCTTTTGATGAACCACTATAAACCTCCGAAAGTCGTCATATGTTACCT 2800
I P T C R A L P K G Q I F Y D N E G K * I * V S Q I N C Y L I N D N G K S R V F T E * G I V A T T F * F V V Q H Y K S A K L L I C H F6
S P L A G Q W L N G R S S I I M R E K K S K S V R S T A I C F I T M G R P G S L H K K D * * I Q L L N S F * K T I N P P K * C Y V I S F5
H H S H V K G F T E G P H F L * E R R K L N L C E P H Q L V F Y Q * E G Q V Q C I N R I R D C S Y Y I L F S S P S I Q L S E A T Y L P F4

L R R L H Q V A H F T C L I * N * N N I R N Y L H L Q F M R Q Q V T V L L L L T I L * C F V E E Q K N N T Q C S L E S R * F L V L V F1
T * E D F T K L R T S P A * F R T K T T * E T I C T S S L C D S R S Q C C Y C * Q F F D V L W R N R K T I H N V H * K A V S S * C L * F2
L E K T S P S C A L H L L D L E L K Q H K K L S A P P V Y A T A G H S V V I V D N S L M F C G G T E K Q Y T M F T R K P L V P S A C D F3
2801 ACTTGAGAAGACTTCACCAAGTTGCGCACTTCACCTGCTTGATTTAGAACTAAAACAACATAAGAAACTATCTGCACCTCCAGTTTATGCGACAGCAGGTCACAGTGTTGTTATTGTTGACAATTCTTTGATGTTTTGTGGAGGAACAGAAAAACAATACACAATGTTCACTAGAAAGCCGTTAGTTCCTAGTGCTTGTG 3000
----:----|----:----|----:----|----:----|----:----|----:----|----:----|----:----|----:----|----:----|----:----|----:----|----:----|----:----|----:----|----:----|----:----|----:----|----:----|----:----|
2801 TGAACTCTTCTGAAGTGGTTCAACGCGTGAAGTGGACGAACTAAATCTTGATTTTGTTGTATTCTTTGATAGACGTGGAGGTCAAATACGCTGTCGTCCAGTGTCACAACAATAACAACTGTTAAGAAACTACAAAACACCTCCTTGTCTTTTTGTTATGTGTTACAAGTGATCTTTCGGCAATCAAGGATCACGAACAC 3000
F K L L S * W T A C K V Q K I * F * F L M L F * R C R W N I R C C T V T N N N N V I R Q H K T S S C F F L V C H E S S L R * N R T S T F6
S S F V E G L Q A S * R S S K S S F C C L F S D A G G T * A V A P * L T T I T S L E K I N Q P P V S F C Y V I N V L F G N T G L A Q F5
V Q S S K V L N R V E G A Q N L V L V V Y S V I Q V E L K H S L L D C H Q * Q Q C N K S T K H L F L F V I C L T * * F A T L E * H K H F4

I W G P T A T * * N H Q K C P Q F S G F N V K P N A I D G C T V F A * I * K R F Q K A N T F V K T V N E K H V R A E R S K L D N L Q N F1
F G V Q L P H S R I T R S V P N S V G S M * S Q M Q S M A A L F L H K S E K D S K R Q I H L S R L * T R N T Y G Q K E V N * T I C K T F2
L G S N C H I V E S P E V S P I Q W V Q C E A K C N R W L H C F C I N L K K I P K G K Y I C Q D C K R E T R T G R K K * I R Q S A K F3
3001 ATTTGGGGTCCAACTGCCACATAGTAGAATCACCAGAAGTGTCCCCAATTCAGTGGGTTCAATGTGAAGCCAAATGCAATCGATGGCTGCACTGTTTTTGCATAAATCTGAAAAAGATTCCAAAGGCAAATACATTTGTCAAGACTGTAAACGAGAAACACGTACGGGCAGAAAGAAGTAAATTAGACAATCTGCAAAA 3200
----:----|----:----|----:----|----:----|----:----|----:----|----:----|----:----|----:----|----:----|----:----|----:----|----:----|----:----|----:----|----:----|----:----|----:----|----:----|----:----|
3001 TAAACCCCAGGTTGACGGTGTATCATCTTAGTGGTCTTCACAGGGGTTAAGTCACCCAAGTTACACTTCGGTTTACGTTAGCTACCGACGTGACAAAAACGTATTTAGACTTTTTCTAAGGTTTCCGTTTATGTAAACAGTTCTGACATTTGCTCTTTGTGCATGCCCGTCTTTCTTCATTTAATCTGTTAGACGTTTT 3200
I Q P G V A V Y Y F * W F H G W N L P N L T F G F A I S P Q V T K A Y I Q F L N W F A F V N T L V T F S F C T R A S L L L N S L R C F F6
S K P D L Q W M T S D G S T D G I * H T * H S A L H L R H S C Q K Q M F R F F I G F P L Y M Q * S Q L R S V R V P L F F Y I L C D A F F5
N P T W S G C L L I V L L T G L E T P E I H L W I C D I A A S N K C L D S F S E L L C I C K D L S Y V L F V Y P C F S T F * V I Q L F4

* D Q N T H K L Q I K S K T Q R P Q G N A L * K S H H P F S S L L I H T G P M F P K P S L G * R C R * N W K N A * R I N D I V L K P Y F1
K I K I P I N Y K L K V K P K D H K G M H S E N L T I L S H L C * S I L D P C S R S R R * V N D V V K T G K M H S V L T T S F * N L F2
L R S K Y P * T T N * K * N P K T T R E C T L K I S P S F L I F A D P Y W T H V P E A V V R L T M S L K L E K C I A Y * R H R F K T L F3
3201 CTAAGATCAAAATACCCATAAACTACAAATTAAAAGTAAAACCCAAAGACCACAAGGGAATGCACTCTGAAAATCTCACCATCCTTTCTCATCTTTGCTGATCCATACTGGACCCATGTTCCCGAAGCCGTCGTTAGGTTAACGATGTCGTTAAAACTGGAAAAATGCATAGCGTATTAACGACATCGTTTTAAAACCTT 3400
----:----|----:----|----:----|----:----|----:----|----:----|----:----|----:----|----:----|----:----|----:----|----:----|----:----|----:----|----:----|----:----|----:----|----:----|----:----|----:----|
3201 GATTCTAGTTTTATGGGTATTTGATGTTTAATTTTCATTTTGGGTTTCTGGTGTTCCCTTACGTGAGACTTTTAGAGTGGTAGGAAAGAGTAGAAACGACTAGGTATGACCTGGGTACAAGGGCTTCGGCAGCAATCCAATTGCTACAGCAATTTTGACCTTTTTACGTATCGCATAATTGCTGTAGCAAAATTTTGGAA 3400
* S * F V W L S C I L L L V W L G C P F A S Q F D * W G K E D K S I W V P G M N G F G D N P * R H R * F Q F F A Y R I L S M T K F G F6
S L D F Y G Y V V F * F Y F G F V V L S H V R F I E G D K R M K A S G Y Q V W T G S A T T L N V I D N F S S F H M A Y * R C R K L V K F5
V L I L I G M F * L N F T F G L S W L P I C E S F R V M R E * R Q Q D M S S G H E R L R R * T L S T T L V P F I C L T N V V D N * F R F4

D D F G S M G P G W L V S V R P P V S I A K M R K D G R I F A * S * S G W P C C C W V I S F V V G G S V S S * G W G S * F L C T F S I K F1
M T T S G A W V L V G W S V R Q Y P * Q R * E R M D G F L H E V R V D G L V V A G S * V L * * G A L F L L R V G G L S F C V H S Q * K F2
* R L R E H G S W L V G Q S A S I H S K D E K G W T D F C M K L E W M A L L L G H K F C S R G L C F F L G L G V L V F V Y I L N K S F3
3401 ATGACGACTTCGGGAGCATGGGTCCTGGTTGGTTGGTCAGTCCGCCAGTATCCATAGCAAAGATGAGAAAGGATGGACGGATTTTTGCATGAAGTTAGAGTGGATGGCCTTGTTGTTGCTGGGTCATAAGTTTTGTAGTAGGGGGCTCTGTTTCTTCTTAGGGTTGGGGGTCTTAGTTTTTGTGTACATTCTCAATAAAA 3600
----:----|----:----|----:----|----:----|----:----|----:----|----:----|----:----|----:----|----:----|----:----|----:----|----:----|----:----|----:----|----:----|----:----|----:----|----:----|----:----|
3401 TACTGCTGAAGCCCTCGTACCCAGGACCAACCAACCAGTCAGGCGGTCATAGGTATCGTTTCTACTCTTTCCTACCTGCCTAAAAACGTACTTCAATCTCACCTACCGGAACAACAACGACCCAGTATTCAAAACATCATCCCCCGAGACAAAGAAGAATCCCAACCCCCAGAATCAAAAACACATGTAAGAGTTATTTT 3600
* S S K P L M P G P Q N T L G G T D M A F I L F S P R I K A H L * L P H G Q Q Q Q T M L K T T P P E T E E * P Q P D * N K H V N E I F F6
H R S R S C P D Q N T P * D A L I W L L S S F P H V S K Q M F N S H I A K N N S P * L N Q L L P S Q K K K P N P T K T K T Y M R L L F5
I V V E P A H T R T P Q D T R W Y G Y C L H S L I S P N K C S T L T S P R T T A P D Y T K Y Y P A R N R R L T P P R L K Q T C E * Y F F4

V I S C P I I C Q F L W L P G L E H K W I F C V Q L V C S C Y I C F * M V * L M K A F A V E D Y C V A N E A R G H H G D H Q M S S P S F1
L Y H V P L F V S F F G C P D L N T S G F F V S N * F A V A T F V F R W C N * * K P L Q L R T T V * P M K P E D I T E T I R C L P L P F2
Y I M S H Y L S V S L V A R T * T Q V D F L C P T S L Q L L H L F L D G V T D E S L C S * G L L C S Q * S Q R T S R R P S D V F P F F3
3601 GTTATATCATGTCCCATTATTTGTCAGTTTCTTTGGTTGCCCGGACTTGAACACAAGTGGATTTTTTGTGTCCAACTAGTTTGCAGTTGCTACATTTGTTTTTAGATGGTGTAACTGATGAAAGCCTTTGCAGTTGAGGACTACTGTGTAGCCAATGAAGCCAGAGGACATCACGGAGACCATCAGATGTCTTCCCCTTC 3800
----:----|----:----|----:----|----:----|----:----|----:----|----:----|----:----|----:----|----:----|----:----|----:----|----:----|----:----|----:----|----:----|----:----|----:----|----:----|----:----|
3601 CAATATAGTACAGGGTAATAAACAGTCAAAGAAACCAACGGGCCTGAACTTGTGTTCACCTAAAAAACACAGGTTGATCAAACGTCAACGATGTAAACAAAAATCTACCACATTGACTACTTTCGGAAACGTCAACTCCTGATGACACATCGGTTACTTCGGTCTCCTGTAGTGCCTCTGGTAGTCTACAGAAGGGGAAG 3800
T I D H G M I Q * N R Q N G P S S C L H I K Q T W S T Q L Q * M Q K * I T Y S I F A K A T S S * Q T A L S A L P C * P S W * I D E G E F6
L * I M D W * K D T E K T A R V Q V C T S K K H G V L K C N S C K N K S P T V S S L R Q L Q P S S H L W H L W L V D R L G D S T K G K F5
N Y * T G N N T L K K P Q G S K F V L P N K T D L * N A T A V N T K L H H L Q H F G K C N L V V T Y G I F G S S M V S V M L H R G R F4

C * D F S E S H G I S C Y P L R Y L H * T G R R L Y Q G P S Q S A P G H A Q * L Y V N S S A K V H S Q N A L S K S F P S S L L F L C T F1
A E I F F P N L T E Y L V T R F V T F T R Q G A D C T R A P L N Q L L D M L N D F M * I V R P R C I V K M H Y Q N L S H L H C F F V F2
L R F F R I S R N I L L P A S L P S L D R A P I V P G P L S I S S W T C S M T L C K * F G Q G A * S K C I I K I F P I F T A F S L Y F3
3801 CTGCTGAGATTTTTCCGAATCTCACGGAATATCTTGTTACCCGCTTCGTTACCTTCACTAGACAGGGCGCCGATTGTACCAGGGCCCCTCTCAATCAGCTCCTGGACATGCTCAATGACTTTATGTAAATAGTTCGGCCAAGGTGCATAGTCAAAATGCATTATCAAAATCTTTCCCATCTTCACTGCTTTTTCTTTGTA 4000
----:----|----:----|----:----|----:----|----:----|----:----|----:----|----:----|----:----|----:----|----:----|----:----|----:----|----:----|----:----|----:----|----:----|----:----|----:----|----:----|
3801 GACGACTCTAAAAAGGCTTAGAGTGCCTTATAGAACAATGGGCGAAGCAATGGAAGTGATCTGTCCCGCGGCTAACATGGTCCCGGGGAGAGTTAGTCGAGGACCTGTACGAGTTACTGAAATACATTTATCAAGCCGGTTCCACGTATCAGTTTTACGTAATAGTTTTAGAAAGGGTAGAAGTGACGAAAAAGAAACAT 4000
Q Q S K E S D * P I D Q * G S R * * V P R R N Y W P G E * D A G P C A * H S * T F L E A L T C L * F A N D F D K G D E S S K R Q F6
R S L N K R I E R F I K N G A E N G E S S L A G I T G P G R E I L E Q V H E I V K H L Y N P W P A Y D F H M I L I K G M K V A K E K Y F5
G A S I K G F R V S Y R T V R K T V K V L C P A S Q V L A G R L * S R S M S L S K I Y I T R G L H M T L I C * * F R E W R * Q K K K T F4

G Q P H L Q N L F L V P * P C R F Y A V H G K W Q E L Q P G T S S F L P M A L N A K L R S H F L Q E R * F W H N S P A Y R A V G * G F1
L A N L I S K I C F W C L S P V D F T Q F T E N G K N F S Q V P L P F F R W H * M Q N Y V R I F C K K D N S G I I P R H T E L W V K A F2
W P T S S P K S V F G A L A L * I L R S S R K M A R T S A R Y L F L S S D G T K C K I T F A F S A R K I I L A * F P G I Q S C G L R P F3
4001 CTGGCCAACCTCATCTCCAAAATCTGTTTTTGGTGCCTTAGCCCTGTAGATTTTACGCAGTTCACGGAAAATGGCAAGAACTTCAGCCAGGTACCTCTCCCTTTCTTCCGATGGCACTAAATGCAAAATTACGTTCGCATTTTCTGCAAGAAAGATAATTCTGGCATAATTCCCCGGCATACAGAGCTGTGGGTTAAGGC 4200
----:----|----:----|----:----|----:----|----:----|----:----|----:----|----:----|----:----|----:----|----:----|----:----|----:----|----:----|----:----|----:----|----:----|----:----|----:----|----:----|
4001 GACCGGTTGGAGTAGAGGTTTTAGACAAAAACCACGGAATCGGGACATCTAAAATGCGTCAAGTGCCTTTTACCGTTCTTGAAGTCGGTCCATGGAGAGGGAAAGAAGGCTACCGTGATTTACGTTTTAATGCAAGCGTAAAAGACGTTCTTTCTATTAAGACCGTATTAAGGGGCCGTATGTCTCGACACCCAATTCCG 4200
V P W G * R W F R N K T G * G Q L N * A T * P F H C S S * G P V E E K R G I A S F A F N R E C K R C S L Y N Q C L E G A Y L A T P * P F6
Q G V E D G F D T K P A K A R Y I K R L E R F I A L V E A L Y R K R E E S P V L H L I V N A N E A L F I I R A Y N G P M C L Q P N L F5
S A L R M E L I Q K Q H R L G T S K V C N V S F P L F K L W T G R G K K R H C * I C F * T R M K Q L F S L E P M I G R C V S S H T L A F4

L L F F L H V H Q T L P L H L Q S W T S H R L F F P I Y S P L K F H A Q * V F * K R W P S * Y L H A * R P V P Y * W H P M L R E G L I F1
Y C F F Y M F I K L C L C I F N H G L H I G C F F P F I H R * N F T H N E F S E K D G Q V D I C M R S V Q C L I D G I R C * E R G * F F2
T V F F T C S S N F A S A S S I M D F T S A V F S H L F T V E I S R T M S F L K K M A K L I S A C V A S S A L L M A S D V E R G A D F3
4201 CTACTGTTTTTTTTACATGTTCATCAAACTTTGCCTCTGCATCTTCAATCATGGACTTCACATCGGCTGTTTTTCCCATTTATTCACCGTTGAAATTTCACGCACAATGAGTTTTCTGAAAAAGATGGCCAAGTTGATATCTGCATGCGTAGCGTCCAGTGCCTTATTGATGGCATCCGATGTTGAGAGAGGGGCTGAT 4400
----:----|----:----|----:----|----:----|----:----|----:----|----:----|----:----|----:----|----:----|----:----|----:----|----:----|----:----|----:----|----:----|----:----|----:----|----:----|----:----|
4201 GATGACAAAAAAAATGTACAAGTAGTTTGAAACGGAGACGTAGAAGTTAGTACCTGAAGTGTAGCCGACAAAAAAGGGTAAATAAGTGGCAACTTTAAAGTGCGTGTTACTCAAAAGACTTTTTCTACCGGTTCAACTATAGACGTACGCATCGCAGGTCACGGAATAACTACCGTAGGCTACAACTCTCTCCCCGACTA 4400
R S N K K C T * * V K G R C R * D H V E C R S N K G M * E G N F N * A C H T K Q F L H G L Q Y R C A Y R G T G * Q H C G I N L S P S I F6
G V T K K V H E D F K A E A D E I M S K V D A T K E W K N V T S I E R V I L K R F F I A L N I D A H T A D L A K N I A D S T S L P A S F5
* Q K K * M N M L S Q R Q M K L * P S * M P Q K K G N I * R Q F K V C L S N E S F S P W T S I Q M R L T W H R I S P M R H Q S L P Q F4

S R L W Q V Y L I Q P C Y G S L D S F E * K I L P F S S F L R M Y E * C * K N P D T F L P * P V Q Q Y K G C K E M T T L S L E D L P R P F1
H A F G K F I * F S L A T V P W I R S N K F C H F P R F F V C T S D V E R T Q T R F C R S P F S S T K G A K K * R A L K T S L G F2
F T P L A S L S N S A L L R F P G F V R I N S A I F L V S S Y V R V M L K E P R H V F A V A R S A V Q R V Q R N D D P E P * R P P S A F3
4401 TTCACGCCTTTGGCAAGTTTATCTAATTCAGCCTTGCTACGGTTCCCTGGATTCGTTCGAATAAATTCTGCCATTTTCCTCGTTTCTTCGTATGTACGAGTGATGTTGAAAGAACCCAGACACGTTTTTGCCGTAGCCCGTTCAGCAGTACAAAGGGTGCAAAGAAATGACGACCCTGAGCCTTGAAGACCTCCCTCGGC 4600
----:----|----:----|----:----|----:----|----:----|----:----|----:----|----:----|----:----|----:----|----:----|----:----|----:----|----:----|----:----|----:----|----:----|----:----|----:----|----:----|
4401 AAGTGCGGAAACCGTTCAAATAGATTAAGTCGGAACGATGCCAAGGGACCTAAGCAAGCTTATTTAAGACGGTAAAAGGAGCAAAGAAGCATACATGCTCACTACAACTTTCTTGGGTCTGTGCAAAAACGGCATCGGGCAAGTCGTCATGTTTCCCACGTTTCTTTACTGCTGGGACTCGGAACTTCTGGAGGGAGCCG 4600
E R R Q C T * R I * G Q * P E R S E N S Y I R G N E E N R R I Y S H H Q F F G S V N K G Y G T * C Y L P H L S I V V R L R S S R G R F6
K V G K A L K D L E A K S R N G P N T R I F E A M K R T E E Y T R T I N F S G L C T K A T A R E A T C L T C L F S S G S G Q L G G E A F5
N * A K P L N I * N L R A V T G Q I R E F L N Q W K G R K K T H V L S T S L V W V R K Q R L G N L L V F P A F F H R G Q A K F V E R P F4

D Q I S H L S * S Y R K * A C A A S T Y R Y P K L * L S L C S I A * G P S P V Q * E A C T G H L L P V S S H L Q W L P G V G D L S G F1
P I K F L I Y H R V I E N E P V L P P H T G I Q N F D F H C V R L H K G P L L F N R K H A Q D T C C L * V L I S N G F Q E W A I C P D F2
R S N F S S I I E L * K M S L C C L H I P V S K T L T F I V F D C I R A L S C S I G S M H R T L V A C E F S S P M A S R S G R F V R T F3
4601 CCGATCAAATTTCTCATCTATCATAGAGTTATAGAAAATGAGCCTGTGCTGCCTCCACATACCGGTATCCAAAACTTTGACTTTCATTGTGTTCGATTGCATAAGGGCCCTCTCCTGTTCAATAGGAAGCATGCACAGGACACTTGTTGCCTGTGAGTTCTCATCTCCAATGGCTTCCAGGAGTGGGCGATTTGTCCGGA 4800
----:----|----:----|----:----|----:----|----:----|----:----|----:----|----:----|----:----|----:----|----:----|----:----|----:----|----:----|----:----|----:----|----:----|----:----|----:----|----:----|
4601 GGCTAGTTTAAAGAGTAGATAGTATCTCAATATCTTTTACTCGGACACGACGGAGGTGTATGGCCATAGGTTTTGAAACTGAAAGTAACACAAGCTAACGTATTCCCGGGAGAGGACAAGTTATCCTTCGTACGTGTCCTGTGAACAACGGACACTCAAGAGTAGAGGTTACCGAAGGTCCTCACCCGCTAAACAGGCCT 4800
G S * I E * R D Y L * L F H A Q Q A E V Y R Y G F S Q S E N H E I A Y P G E G T * Y S A H V P C K N G T L E * R W H S G P T P S K D P F6
R D F K E D I M S N Y F I L R H R W M G T D L V K V K M T N S Q M L A R E Q E I P L M C L V S T A Q S N E D G I A E L P R N T R F5
G I L N R M * * L T I S F S G T S G G C V P I W F K S K * Q T R N C L P G R R N L L F C A C S V Q Q R H T R M E L P K W S H A I Q G S F4

Q S W V Y P L Q K L S P V F H Q F Q F G I S K L R K Q N E R P Y Q V G T C Q R I L Y V R I H H P F R L L H L L L L C V L P D W D S L D F1
R V G F I L F K N C H Q F S I N F N L A F Q N C E S K T K G L I R * E P V R G F F M Y G Y I T H S V C S I F Y C C V Y Y Q T G I P W I F2
E L G L S S S K T V T S F P S I S I W H F K T A K A K R K A L S G R N L S E D S L C T D T S P I P S A P S F I V V C I T R L G F P G F3
4801 CAGAGTTGGGTTTATCCTCTTCAAAAACTGTCACCAGTTTTCCATCAATTTCAATTTGGCATTTCAAAACTGCGAAAGCAAAACGAAAGGCCTTATCAGGTAGGAACCTGTCAGAGGATTCTTTATGTACGGATACATCACCCATTCCGTCTGCTCCATCTTTTATTGTTGTGTGTATTACCAGACTGGGATTCCCTGGA 5000
----:----|----:----|----:----|----:----|----:----|----:----|----:----|----:----|----:----|----:----|----:----|----:----|----:----|----:----|----:----|----:----|----:----|----:----|----:----|----:----|
4801 GTCTCAACCCAAATAGGAGAAGTTTTTGACAGTGGTCAAAAGGTAGTTAAAGTTAAACCGTAAAGTTTTGACGCTTTCGTTTTGCTTTCCGGAATAGTCCATCCTTGGACAGTCTCCTAAGAAATACATGCCTATGTAGTGGGTAAGGCAGACGAGGTAGAAAATAACAACACACATAATGGTCTGACCCTAAGGGACCT 5000
C L Q T * G R * F S D G T K W * N * N P M E F S R F C F S L G * * T P V Q * L I R * T R I C * G N R R S W R K N N H T N G S Q S E R S F6
V S N P K D E E F V T V L K G D I E I Q C K L V A F A F R F A K D P L F R D S S E K H V S V D G M G D A G D K I T T H I V L S P N G P F5
L T P N I R K L F Q * W N E M L K L K A N * F Q S L L V F P R I L Y S G T L P N K I Y P Y M V W E T Q E M K * Q Q T Y * W V P I G Q F4
```

```
       L  V  Q  F  G  E  S  L  C  Y  *  R  I  K  F  H  Q  G  L  S  N  S  I  G  I  E  P  A  N  T  L  A  I  W  S  C  E  L  N  E  I  I  I  E  F  G  *  N  V  N  W  L  E  S  T  W  C  I  V  N  F  H  K  R  T  M  E   F1
      *  S  N  L  V  N  P  F  A  I  K  G  S  S  S  I  K  V  L  A  T  A  S  G  *  N  Q  R  T  P  L  Q  F  G  V  A  S  S  T  R  L  S  L  N  S  D  R  M  S  I  G  S  K  V  L  G  A  S  S  I  F  T  R  E  R  W    F2
     S  S  P  I  W  *  I  P  L  L  L  K  D  Q  V  P  S  R  S  *  Q  Q  H  R  D  R  T  S  E  H  P  C  N  L  E  L  R  A  Q  R  D  Y  H  *  I  R  I  E  C  Q  L  A  R  K  Y  L  V  H  R  Q  F  S  Q  E  N  D  G     F3
5001 TCTAGTCCAATTTGGTGAATCCCTTTGCTATTAAAGGATCAAGTTCCATCAAGGTCTTAGCAACAGCATCGGGATAGAACCAGCGAACACCCTTGCAATTTGGAGTTGCGAGCTCAACGAGATTATCATTGAATTCGGATAGAATGTCAATTGGCTCGAAAGTACTTGGTGCATCGTCAATTTTCACAAGAGAACGATGG 5200
     ----:----|----:----|----:----|----:----|----:----|----:----|----:----|----:----|----:----|----:----|----:----|----:----|----:----|----:----|----:----|----:----|----:----|----:----|----:----|----:----|
5001 AGATCAGGTTAAACCACTTAGGGAAACGATAATTTCCTAGTTCAAGGTAGTTCCAGAATCGTTGTCGTAGCCCTATCTTGGTCGCTTGTGGGAACGTTAAACCTCAACGCTCGAGTTGCTCTAATAGTAACTTAAGCCTATCTTACAGTTAACCGAGCTTTCATGAACCACGTAGCAGTTAAAAGTGTTCTCTTGCTACC 5200
      R  T  W  N  P  S  D  R  Q  *  *  L  I  L  N  W  *  P  R  L  L  L  M  P  I  S  G  A  F  V  R  A  I  Q  L  Q  S  S  L  S  I  I  M  S  N  P  Y  F  T  L  Q  S  S  L  V  Q  H  M  T  L  K  *  L  L  V  I    F6
       D  L  G  I  Q  H  I  G  K  S  N  F  S  *  T  G  D  L  D  *  C  C  C  R  S  L  V  L  S  C  G  Q  L  K  S  N  R  A  *  R  S  *  *  Q  I  R  I  S  H  *  N  A  R  F  Y  K  T  C  R  *  N  E  C  S  F  S  P   F5
     I  *  D  L  K  T  F  G  K  A  I  L  P  D  L  E  M  L  T  K  A  V  A  D  P  Y  F  W  R  V  G  K  C  N  P  T  A  L  E  V  L  N  D  N  F  E  S  L  I  D  I  P  E  F  T  S  P  A  D  D  I  K  V  L  S  R  H     F4

       T  G  L  F  L  L  D  V  K  R  I  S  S  C  R  W  H  E  M  K  L  H  L  V  Q  F  V  G  W  L  *  S  C  N  A  L  A  F  *  K  *  I  L  P  A  V  L  R  L  A  L  K  I  F  H  P  T  C  Q  T  L  F  Y  G  K  S    F1
     K  L  D  S  F  S  W  T  S  R  E  Y  L  A  A  A  G  M  K  *  N  S  I  S  S  S  L  L  G  G  C  K  A  V  M  P  L  L  F  K  N  E  Y  C  L  R  Y  C  D  L  L  *  R  F  S  T  R  L  A  R  H  S  S  T  G  R  V     F2
      N  W  T  L  S  L  G  R  Q  E  N  I  *  L  P  L  A  *  N  E  T  P  S  R  P  V  C  W  V  A  V  K  L  *  C  P  C  F  L  K  M  N  T  A  C  G  I  A  T  C  F  E  D  F  P  P  D  L  P  D  T  L  L  R  E  E  C    F3
5201 AAACTGGACTCTTTCTCTTGGACGTCAAGAGAATATCTAGCTGCCGCTGGCATGAAATGAAACTCCATCTCGTCCAGTTTGTTGGGTGGCTGTAAAGCTGTAATGCCCTTGCTTTTAAAAATGAATACTGCCTGCGGTATTGCGACTTGCTTTGAAGATTTTCCACCCGACTTGCCAGACACTCTTCTACGGGAAGAGT 5400
     ----:----|----:----|----:----|----:----|----:----|----:----|----:----|----:----|----:----|----:----|----:----|----:----|----:----|----:----|----:----|----:----|----:----|----:----|----:----|----:----|
5201 TTTGACCTGAGAAAGAGAACCTGCAGTTCTCTTATAGATCGACGGCGACCGTACTTTACTTTGAGGTAGAGCAGGTCAAACAACCCACCGACATTTCGACATTACGGGAACGAAAAATTTTTACTTATGACGGACGCCATAACGCTGAACGAAACTTCTAAAAGGTGGGCTGAACGGTCTGTGAGAAGATGCCCTTCTCA 5400
     S  V  P  S  K  R  K  S  T  L  L  I  D  L  Q  R  Q  C  S  I  F  S  W  R  T  W  N  T  P  H  S  Y  L  Q  L  A  R  A  K  *  F  H  I  S  G  A  T  N  R  S  A  K  F  I  K  W  G  V  Q  W  V  S  K  *  P  F  L     F6
      F  Q  V  R  E  R  P  R  *  S  F  I  *  S  G  S  A  H  F  S  V  G  D  R  G  T  Q  Q  T  A  T  F  S  Y  H  G  Q  K  K  F  I  F  V  A  Q  P  I  A  V  Q  K  S  S  K  G  G  S  K  G  S  V  R  R  R  S  S    F5
       F  S  S  E  K  E  Q  V  D  L  S  Y  R  A  A  A  P  M  F  H  F  E  M  E  D  L  K  N  P  P  Q  L  A  T  I  G  K  S  K  L  F  S  Y  Q  R  R  Y  Q  S  K  S  Q  L  N  E  V  R  S  A  L  C  E  E  V  P  L  T   F4

     V  H  F  F  Q  P  N  I  P  D  L  P  T  F  F  F  F  I  F  I  I  *  G  N  *  *  R  T  K  Q  H  I  D  F  I  F  F  V  F  F  T  K  F  I  Y  S  T  S  I  I  R  G  F  N  L  F  C  F  K  Y  F  G  A  A  K  L  K    F1
      S  I  F  S  N  Q  I  S  R  T  F  Q  L  F  S  F  S  F  L  S  Y  R  V  I  D  N  V  Q  N  S  T  S  I  L  F  S  L  F  S  S  Q  N  L  F  I  L  L  Q  L  L  G  A  S  I  S  F  V  L  N  I  L  E  R  P  S  S  S   F2
       P  F  F  P  T  K  Y  P  G  P  S  N  F  F  L  F  H  F  Y  H  I  G  *  L  I  T  Y  K  T  A  H  R  F  Y  F  L  C  F  L  H  K  I  Y  L  F  Y  F  N  Y  *  G  L  Q  S  L  L  F  *  I  F  W  S  G  Q  A  Q    F3
5401 GTCCATTTTTTCCAACCAAATATCCCGGACCTTCCAACTTTTTTCTTTTTCATTTTTATCATATAGGGTAATTGATAACGTACAAAACAGCACATCGATTTTATTTTCTTTGTTTTCTTCACAAAATTTATTTATTCTACTTCAATTATTAGGGGCTTCAATCTCTTTTGTTTTAAATATTTTGGAGCGGCCAAGCTCAA 5600
     ----:----|----:----|----:----|----:----|----:----|----:----|----:----|----:----|----:----|----:----|----:----|----:----|----:----|----:----|----:----|----:----|----:----|----:----|----:----|----:----|
5401 CAGGTAAAAAAGGTTGGTTTATAGGGCCTGGAAGGTTGAAAAAAGAAAAAGTAAAAATAGTATATCCCATTAACTATTGCATGTTTTGTCGTGTAGCTAAAATAAAAGAAACAAAAGAAGTGTTTTAAATAAATAAGATGAAGTTAATAATCCCCGAAGTTAGAGAAAACAAAATTTATAAAACCTCGCCGGTTCGAGTT 5600
      T  W  K  K  W  G  F  I  G  S  R  G  V  K  K  K  K  M  K  I  M  Y  P  L  Q  Y  R  V  F  C  C  M  S  K  I  K  K  T  K  K  V  F  N  I  *  E  V  E  I  I  L  P  K  L  R  K  Q  K  L  Y  K  P  A  A  L  S  L   F6
     H  G  N  K  G  V  L  Y  G  P  G  E  L  K  K  R  K  *  K  *  *  I  P  Y  N  I  V  Y  L  V  A  C  R  N  *  K  R  Q  K  R  *  L  I  *  K  N  *  K  L  *  *  P  S  *  D  R  K  N  *  I  N  Q  L  P  W  A  *    F5
       D  M  K  E  L  W  I  D  R  V  K  W  S  K  E  K  E  N  K  D  Y  L  T  I  S  L  T  C  F  L  V  D  I  K  N  E  K  N  E  E  C  F  K  N  I  R  S  *  N  N  P  A  E  I  E  K  T  K  F  I  K  S  R  G  L  E    F4

     C  S  F  L  E  L  L  G  L  F  L  F  F  G  V  L  G  K  F  R  P  T  C  R  S  A  V  G  V  V  T  T  D  G  S  D  G  K  V  M  S  S  A  A  A  A  E  V  M  S  S  A  A  A  A  E  V  I  T  W  P  *  N  S  S  R  A    F1
       A  L  F  W  S  F  *  A  F  F  S  F  L  V  S  W  G  N  L  D  L  P  V  V  R  L  L  G  L  *  P  L  M  D  L  M  A  K  *  C  P  Q  Q  L  L  Q  R  S  C  P  Q  Q  L  L  Q  R  S  S  H  G  H  E  I  H  H  G   F2
     V  L  F  F  G  A  F  R  P  F  S  L  F  W  C  L  G  E  I  *  T  Y  L  S  F  G  C  W  G  C  D  H  *  W  I  *  W  Q  S  D  V  L  S  S  C  C  R  G  H  V  L  S  S  C  C  R  G  H  H  M  A  M  K  F  I  T  G    F3
5601 GTGCTCTTTTTTGGAGCTTTTAGGCCTTTTTCTCTTTTTTGGTGTCTTGGGGAAATTTAGACCTACCTGTCGTTCGGCTGTTGGGGTTGTGACCACTGATGGATCTGATGGCAAAGTGATGTCCTCAGCAGCTGCTGCAGAGGTCATGTCCTCAGCAGCTGCTGCAGAGGTCATCACATGGCCATGAAATTCATCACGGG 5800
     ----:----|----:----|----:----|----:----|----:----|----:----|----:----|----:----|----:----|----:----|----:----|----:----|----:----|----:----|----:----|----:----|----:----|----:----|----:----|----:----|
5601 CACGAGAAAAAACCTCGAAAATCCGGAAAAAGAGAAAAAACCACAGAACCCCTTTAAATCTGGATGGACAGCAAGCCGACAACCCCAACACTGGTGACTACCTAGACTACCGTTTCACTACAGGAGTCGTCGACGACGTCTCCAGTACAGGAGTCGTCGACGACGTCTCCAGTAGTGTACCGGTACTTTAAGTAGTGCCC 5800
       H  E  K  K  S  S  K  P  R  K  R  K  K  P  T  K  P  F  N  L  G  V  Q  R  E  A  T  P  T  T  V  V  S  P  D  S  P  L  T  I  D  E  A  A  A  A  S  T  M  D  E  A  A  A  A  S  T  M  V  H  G  H  F  E  D  R   F6
     T  S  K  K  P  A  K  L  G  K  E  R  K  Q  H  R  P  S  I  *  V  *  R  D  N  P  Q  Q  P  Q  S  W  Q  H  I  Q  H  C  L  S  T  R  L  L  Q  Q  L  P  *  T  R  L  L  Q  Q  L  P  *  *  M  A  M  F  N  M  V  P    F5
     L  A  R  K  Q  L  K  *  A  K  K  E  K  K  T  D  Q  P  F  K  S  R  G  T  T  R  S  N  P  N  H  G  S  I  S  R  I  A  F  H  H  G  *  C  S  S  C  L  D  H  G  *  C  S  S  C  L  D  D  C  P  W  S  I  *  *  P    F4

       T  N  D  I  S  L  S  S  F  V  G  Q  R  P  P  A  G  A  Q  S  G  H  F  S  F  G  C  F  S  N  L  A  I  F  F  F  A  L  K  S  P  P  T  F  L  A  R  S  H  I  N  W  Q  D  L  H  S  F  F  G  K  T  C  L  S  S    F1
     Q  L  M  T  F  H  Y  L  R  L  W  G  S  G  L  P  Q  E  H  K  V  D  I  F  H  S  G  A  S  Q  I  W  Q  F  F  S  L  P  *  S  H  P  P  H  F  W  H  A  H  T  S  T  G  R  T  C  T  V  F  L  E  R  R  V  C  P  R     F2
      N  *  *  H  F  I  I  F  V  C  G  A  A  A  S  R  R  S  T  K  W  T  F  F  I  R  V  L  L  K  F  G  N  F  F  L  C  P  E  V  T  P  H  I  F  G  T  L  T  H  Q  L  A  G  L  A  Q  F  F  W  K  D  V  F  V  L  V   F3
5801 CAACTAATGACATTTCATTATCTTCGTTTGTGGGGCAGCGGCCTCCCGCAGGAGCACAAAGTGGACATTTTTCATTCGGGTGCTTCTCAAATTTGGCAATTTTTTTCTTTGCCCTGAAGTCACCCCCCACATTTTTGGCACGCTCACACATCAACTGGCAGGACTTGCACAGTTTTTTTGGAAAGACGTGTTTGTCCTCG 6000
     ----:----|----:----|----:----|----:----|----:----|----:----|----:----|----:----|----:----|----:----|----:----|----:----|----:----|----:----|----:----|----:----|----:----|----:----|----:----|----:----|
5801 GTTGATTACTGTAAAGTAATAGAAGCAAACACCCCGTCGCCGGAGGGCGTCCTCGTGTTTCACCTGTAAAAAGTAAGCCCACGAAGAGTTTAAACCGTTAAAAAAAGAAACGGGACTTCAGTGGGGGGTGTAAAAACCGTGCGAGTGTGTAGTTGACCGTCCTGAACGTGTCAAAAAAACCTTTCTGCACAAACAGGAGC 6000
       A  V  L  S  M  E  N  D  E  N  T  P  C  R  G  G  A  P  A  C  L  P  C  K  E  N  P  H  K  E  F  K  A  I  K  K  K  A  R  F  D  G  G  V  N  K  A  R  E  C  M  L  Q  C  S  K  C  L  K  K  P  F  V  H  K  D  E   F6
      L  *  H  C  K  M  I  K  T  Q  P  A  A  A  E  R  L  L  V  F  H  V  N  K  M  R  T  S  R  L  N  P  L  K  K  R  Q  G  S  T  V  G  W  M  K  P  V  S  V  C  *  S  A  P  S  A  C  N  K  Q  F  S  T  N  T  R    F5
       C  S  I  V  N  *  *  R  R  K  H  P  L  P  R  G  C  S  C  L  T  S  M  K  *  E  P  A  E  *  I  Q  C  N  K  E  K  G  Q  L  *  G  G  C  K  Q  C  A  *  V  D  V  P  L  V  Q  V  T  K  K  S  L  R  T  Q  G  R   F4

     F  M  T  S  T  E  *  S  C  C  R  I  C  W  *  T  S  T  E  N  C  R  K  S  S  R  G  R  R  L  P  H  T  L  Q  R  L  D  T  R  S  L  W  R  L  S  *  L  A  I  S  E  K                                                    F1
      S  *  R  Q  L  S  K  V  A  A  G  Y  V  G  K  R  Q  Q  K  T  V  G  S  H  H  G  G  D  V  Y  H  T  P  C  S  V  W  I  L  V  L  C  G  D  C  H  D  W  P  F  L  K  X                                                 F2
       H  D  V  N  *  V  K  L  L  Q  D  M  L  V  N  V  N  R  K  L  S  E  V  I  T  G  E  T  F  T  T  H  P  A  A  F  G  Y  S  F  F  V  E  T  V  M  T  G  H  F  *  K  X                                                F3
6001 TTCATGACGTCAACTGAGTAAAGTTGCTGCAGGATATGTTGGTAAACGTCAACAGAAAACTGTCGGAAGTCATCACGGGGGAGACGTTTACCACACACCCTGCAGCGTTTGGATACTCGTTCTTTGTGGAGACTGTCATGACTGGCCATTTCTGAAAAA 6159
     ----:----|----:----|----:----|----:----|----:----|----:----|----:----|----:----|----:----|----:----|----:----|----:----|----:----|----:----|----:----|----:----
6001 AAGTACTGCAGTTGACTCATTTCAACGACGTCCTATACAACCATTTGCAGTTGTCTTTTGACAGCCTTCAGTAGTGCCCCCTCTGCAAATGGTGTGTGGGACGTCGCAAACCTATGAGCAAGAAACACCTCTGACAGTACTGACCGGTAAAGACTTTTT 6159
       N  M  V  D  V  S  Y  L  Q  Q  L  I  H  Q  Y  V  D  V  S  F  Q  R  F  D  D  R  P  L  R  K  G  C  V  R  C  R  K  S  V  R  E  K  H  L  S  D  H  S  A  M  E  S  F                                               F6
     T  *  S  T  L  Q  T  F  N  S  C  S  I  N  T  F  T  L  L  F  S  D  S  T  M  V  P  S  V  N  V  V  C  G  A  A  N  P  Y  E  N  K  T  S  V  T  M  V  P  W  K  Q  F                                                  F5
       E  H  R  *  S  L  L  T  A  A  P  Y  T  P  L  R  *  C  F  V  T  P  L  *  *  P  P  S  T  *  W  V  G  Q  L  T  Q  I  S  T  R  Q  P  S  Q  *  S  Q  G  N  R  F  F                                                F4
```

Matching transcriptomic data (TSA)

RAG2L

TSA GFRY01056743.1 5UTR is ~3000 bp upstream…therefore it was not included in the alignment, and moved out

```
NMRB01000732.1        221725  CGCAGACGCCGGTTTCAACTGAATAAAAATCCTTCACAATCGACATAAATACACAATATTTGAGCTGTCTTTTATTAC  221802
TSA GFRY01056743.1    1       CGCAGACGCCGGTTTCAACTGAATAAAAATCCTTCACAATCGACATAAATACACAATATTTGAGCTGTCTTTTATTAC  78
```

>WGS NMRB01000732.1:224100-227324
>TSA GFRY01056743.1:79-end
>TSA GFRY01085303.1:beg-end

```
              1                                                                                                                                                                                                          200
NMRB01000732                                                                             ATAATGTAGAATAATATCCTTCATTAGTCATTGATAGGCTCTAGATTTGATCCACAATGGCACAGCTCAGCGAAGAGAGAGTGTTTTCCAGGCTTCCTCTTGACGATTTCAAGTTTTTGCCCTTCAAAAGTTACAGTTATGATTCACGC
GFRY01056743  ------------------------------------------------------------------------------------------------------------TCATTGATAGGCTCTAGATTTGATCCACAATGGCACAGCTCAGCGAAGAGAGAGTGTTTTCCAGGCTTCCTCTTGACGATTTCAAGTTTTTGCCCTTCAAAAGTTACAGTTATGATTCACGC
GFRY01085303
 Consensus    ............................................................................................................tcattgataggctctagatttgatccacaatggcacagctcagcgaagagagagtgttttccaggcttcctcttgacgatttcaagtttttgcccttcaaaagttacagttatgattcacgc

            201                                                                                                                                                                                                        400
NMRB01000732 AAGGTGACTCGGAAAAGGGCTGGACTGGAGTTGCTCCATGACGAATATTACCCCGCAGAAGGACACTGTATGTCCGCTGTTCAAGGGGAGAACTCTGTTATTGTGTACTTTTTTGGTGGTGCCCGACGGAAAGATGAGGCAACTTGGAGTATGAACAATATCATCCAGGAAATGGTGGTGACGTACGATGAAGAAGATGT
GFRY01056743 AAGGTGACTCGGAAAAGGGCTGGACTGGAGTTGCTCCATGACGAATATTACCCCGCAGAAGGACACTGTATGTCCGCTGTTCAAGGGGAGAACTCTGTTATTGTGTACTTTTTTGGTGGTGCCCGACGGAAAGATGAGGCAACTTGGAGTATGAACAATATCATCCAGGAAATGGTGGTGACGTACGATGAAGAAGATGT
GFRY01085303
 Consensus   aaggtgactcggaaaagggctggactggagttgctccatgacgaatattaccccgcagaaggacactgtatgtccgctgttcaaggggagaactctgttattgtgtactttttttggtggtgcccgacggaaagatgaggcaacttggagtatgaacaatatcatccaggaaatggtggtgacgtacgatgaagaagatgt

            401                                                                                                                                                                                                        600
NMRB01000732 CGATATAGTCACAGTTAAAACCCTCAAGGTGGGCCGAGTGCGGGGTCTAATGTTGTTCAAGAGTGTAACATATAATATCCGACTCCAGGACACTACAGTAGTGAGTAACAGTGCAGCGCATGGACGGCCTCTCATGGGTGCCCATCCATTTTTGGTGCCAATATAGGAGTGGCATGCATACTCCAATTCCTTGTGAAAAG
GFRY01056743 CGATATAGTCACAGTTAAAACCCTCAAG------------------------------------------------------------------------------------------------------------------------------------------------------------------------
GFRY01085303
 Consensus   cgatatagtcacagttaaaaccctcaag........................................................................................................................................................................

            601                                                                                                                                                                                                        800
NMRB01000732 AAAACATGTCAAAAATGTATTCATTTGGTATTTATCTTTTTTATTCATATTGTTATAATACCGGTATACATCTCTTTTATTTCATCTCTTCATTCACCTTATTATCTACATACCCGTCATTCACTCTTATTCACAATCTTTATACCGGGTGGCAATTCGTCTCTTTATAGACATTTCTTTTAATCATAGCAGAATTCATC
GFRY01056743 ----------------------------------------------------------------------------------------------------------------------------------------------------------------------------------------------------------------
GFRY01085303
 Consensus   ................................................................................................................................................................................................

            801                                                                                                                                                                                                       1000
NMRB01000732 TCTTTTATTCATATGTTTATACCATTAAGCAATCCATATCTATTTAAATTCATCTTGCGGTCCGCTATATCTTTATATTCTCATCTCTTTTGCAATGGAAAGAGTACACAGTTCAAAATCACAATTTTTAGCTCACTGGGGTGAGCTATTACGATGGTGCAGCGTCCGTCGTCCGTCGTCGTCCGTCGTCGTCGTCG
GFRY01056743 ----------------------------------------------------------------------------------------------------------------------------------------------------------------------------------------------------------------
GFRY01085303
 Consensus   ................................................................................................................................................................................................

            1001                                                                                                                                                                                                      1200
NMRB01000732 TCCGTCGTCGTCGTCGTCGTTGTCCGTTAGCAGGGCACGTTTCGTAACCGCTGGAGCTATTGATCTGAAACTTGGTACATATATACCCCTAGGTCAGATGACCCACGACACCAAGTTTCGGTCCGATTCGACTCTCAGTTTGGCCACCAGGTGGCAGAAACCAAAAACACAAAAAGTACAATAACTCCTGAACTAATGAC
GFRY01056743 ----------------------------------------------------------------------------------------------------------------------------------------------------------------------------------------------------------------
GFRY01085303
 Consensus   ................................................................................................................................................................................................

            1201                                                                                                                                                                                                      1400
NMRB01000732 TAGATCATCTTCAAATTTTTATCATAGGTGTAGCTAATAAGGATACATAACATATATAACGGGTTTTTGATTTGACCTACTTTTCAAGGTCACAGAGGTCAAAGTTCGACAAAAGTGACGAACCTGCCACAGTTTGTCATTATTTCACCGAGCAACTTCATATCTTGCATAAACATGCATTGGAGGGGTACCTTAAAAAA
GFRY01056743 ----------------------------------------------------------------------------------------------------------------------------------------------------------------------------------------------------------------
GFRY01085303
 Consensus   ................................................................................................................................................................................................

            1401                                                                                                                                                                                                      1600
NMRB01000732 TACCAAGTTTCGGCCCGATTCGACATCCAATATGGCCGCCAGGTGGCGTAACCNNNNNNNNNNNNNNNNNNNNNNNNNNNNNNNNNNNNNNNNNNNNNNNNNNNNNNNNNNNNNNNNNNNNNNNNNNNNNNNNNNNNNNNNNNNNNNNNNNNNNNNNNNNNNNNNNNNNNNNNNNNNNNNNNNNNNNNNNNNNNNNNNNNNNNNN
GFRY01056743 ----------------------------------------------------------------------------------------------------------------------------------------------------------------------------------------------------------------
GFRY01085303
 Consensus   ................................................................................................................................................................................................

            1601                                                                                                                                                                                                      1800
NMRB01000732 NNNNNNNNNNNNNNNNNNNNNNNNNNNNNNNNNNNNNNNNNNNNNNNNNNNNNNNNNNNNNNNNNNNNNNNNNNNNNNNNNNNNNNNNNNNNNNNNNNNNNNNNNNNNNNNNNNNNNNNNNNNNNNNNNNNNNNNNNNNNNNNNATGCCTTAACCCAAAGCGCTCCAATGTGCTATGTGGCCCCCTGCAGATGACCTTTATGTCC
GFRY01056743 ----------------------------------------------------------------------------------------------------------------------------------------------------------------------------------------------------------------
GFRY01085303
 Consensus   ................................................................................................................................................................................................

            1801                                                                                                                                                                                                      2000
NMRB01000732 ATGTGAAGTTTCGGCCCGATCGGACATCCAATATGGCCGCCAGGTGGCGTAACTCGAAAATCATCAAAGTGCTGTAACTCAGGAACGACTTGACCCAAAGTGCTCCAATGTGCTATGTCATGTATGTGGTCCCCTGCAGATGACCTTTATGTGCATGTGAAGTTGCAGCCCAATCGGACATCTAATATGGCCGCCAGGTG
```

```
GFRY01056743  --------------------------------------------------------------------------------------------------------------------------------------------------------------------------------------------------------
GFRY01085303
 Consensus    ........................................................................................................................................................................................................

              2001                                                                                                                                                                                                2200
NMRB01000732  GCATAACCCAAAAATCATCAGTCTTTTCTGGGAATGACTTGACCCAGATTGCTCATAACTGATATGTACCTCCCTCTGTCATTAGACTATTATGTCCAACCCAGTGAGCACATGGTCCATGGACCATTTCATTATACATTTGTCATTGCTATGTTGTTCTGGTTCATTTCAGACAACTGGTGCTGAAGTCACTGGTCTGC
GFRY01056743  ------------------------------------------------------------------------------------------------------------------------------------------------------------------------ACAACTGGTGCTGAAGTCACTGGTCTGC
GFRY01085303
 Consensus    ........................................................................................................................................................................acaactggtgctgaagtcactggtctgc

              2201                                                                                                                                                                                                2400
NMRB01000732  AATCAGCTGGATTTTTGGCCATTCCAAATAATAAATGCAAGCCCGATTTCGTCATCTGGGGTGGTTTCAATATGACTCATTTGAAAACGGAGAATGAGCTCTTGCATTTAAGATACCTGAAAAAAGAGCAGAAGCACCACTGTACAATATTCAAGGACCCAAACGAAAAGTTCAAACTGGACACAGATGTTTATCAGTCT
GFRY01056743  AATCAGCTGGATTTTTGGCCATTCCAAATAATAAATGCAAGCCCGATTTCGTCATCTGGGGTGGTTTCAATATGACTCATTTGAAAACGGAGAATGAGCTCTTGCATTTAAGATACCTGAAAAAAGAGCAGAAGCACCACTGTACAATATTCAAGGACCCAAACGAAAAGTTCAAACTGGACACAGATGTTTATCAGTCT
GFRY01085303
 Consensus    aatcagctggatttttggccattccaaataataaatgcaagcccgatttcgtcatctggggtggtttcaatatgactcatttgaaaacggagaatgagctcttgcatttaagatacctgaaaaaagagcagaagcaccactgtacaatattcaaggacccaaacgaaaagttcaaactggacacagatgtttatcagtct

              2401                                                                                                                                                                                                2600
NMRB01000732  GGGGAAGTCCCTTCAAGCCGAACCGGCCACTCATTTACTCAAATATCCGGCACCAGATTGGTTGCGCTGTTTGGTGGTTTGGAGATGAGATTCAGGGAGCGATCTGCAGTTACAACACCAACCCTTAGCCCATTTACGCAGACCTGTCAAGATGCTCACTTCTATGTTTTGAATTTGGCAGAAAACAAATGGACAAAGCT
GFRY01056743  GGGGAAGTCCCTTCAAGCCGAACCGGCCACTCATTTACTCAAATATCCGGCACCAGATTGGTTGCGCTGTTTGGTGGTTTGGAGATGAGATTCAGGGAGCGATCTGCAGTTACAACATCAACCCTTAGCCCATTTACGCAGACCTGTCAAGATGCTCACTTCTATGTTTTGAATTTGGCAGAAAACAAATGGACAAAGCT
GFRY01085303
 Consensus    ggggaagtcccttcaagccgaaccggccactcatttactcaaatatccggcaccagattggttgcgctgtttggtggtttggagatgagattcagggagcgatctgcagttacaaca.caacccttagcccatttacgcagacctgtcaagatgctcacttctatgttttgaatttggcagaaaacaaatggacaaagct

              2601                                                                                                                                                                                                2800
NMRB01000732  GAAAACACCTGAAATCACAGCCAGGTGTTACCATTCAGGTACTTTCATTGAAATTCAGGGGCGACAGACAATTGCAATTGTTGGAGGGATTGAGTATGATGGGAGTGCACCTTGCCAAAGGTTTCCCCTGGATGAAATAATCATTCTCTCCTTTTTAGATTTAGACACTCTGGATGTTGCAATACAAAATATTGTCATTC
GFRY01056743  GAAAACACCTGAAATCACAGCCAGGTGTTACCATTCAGGTACTTTCATTGAAATTCAGGGGCGACAGACAATTGCAATTGTTGGAGGGATTCAGTATGATGGGAGTGCACCTTGCCAAAGGTTTCCCCTGGATGAAATAATCATTCTCTCCTTTTTAGATTTAGACACTCTGGATGTTGCAATACAAAATATTGTCATTC
GFRY01085303
 Consensus    gaaaacacctgaaatcacagccaggtgttaccattcaggtactttcattgaaattcaggggcgacagacaattgcaattgttggagggatt.agtatgatgggagtgcaccttgccaaaggtttcccctggatgaaataatcattctctcctttttagatttagacactctggatgttgcaatacaaaatattgtcattc

              2801                                                                                                                                                                                                3000
NMRB01000732  CCCTTGGACCTGACAAATGTTTCTTATCCTATCACAGCTGTAGTAAATTAGAAAACTACTTGGTGATATTTGGAGGCTTTCAGCAGTATACAATGGAACTTGAGAAGACTTCACCAAGTTGCGCACTTCACCTGCTTGATTTAGAACTAAAACAACATAAGAAACTATCTGCACCTCCAGTTTATGCGACAGCAGGTCAC
GFRY01056743  CCCTTGGACCTGACAAATGTT
GFRY01085303                       TCTTATCCTATCACAGCTGCAGTAAATTAGAAAACTACTTGGTGATATTTGGAGGCTTTCAGCAGTATACAATGGAACTTGAGAAGACTTCACCAAGTTGCGCACTTCACCTGCTTGATTTAGAACTAAAACAACATAAGAAACTATCTGCACCTCCAGTTTATGCGACAGCAGGTCAC
 Consensus    cccttggacctgacaaatgtttcttatcctatcacagctg.agtaaattagaaaactacttggtgatatttggaggctttcagcagtatacaatggaacttgagaagacttcaccaagttgcgcacttcacctgcttgatttagaactaaaacaacataagaaactatctgcacctccagtttatgcgacagcaggtcac

              3001                                                                                                                                                                                                3200
NMRB01000732  AGTGTTGTTATTGTTGACAATTCTTTGATGTTTTGTGGAGGAACAGAAAAACAATACACAATGTTCACTAGAAAGCCGTTAGTTCCTAGTGCTTGTGATTTGGGGTCCAACTGCCACATAGTAGAATCACCAGAAGTGTCCCCAATTCAGTGGGTTCAATGTGAAGCCAAATGCAATCGATGGCTGCACTGTTTTTGCAT
GFRY01056743
GFRY01085303  AGTGTTGTTATTGTTGACAATTCTTTGATGTTTTGTGGAGGAACAGAAAAACAATACACAATGTTCACTAGAAAGCCGTTAGTTCCTAGTGCTTGTGATTTGGGGTCCAACTGCCACATAGTAGAATCACCAGAAGTGTCCCCAATTCAGTGGGTTCAATGTGAAGCCAAATGCAAGCGATGGCTGCACTGTTTTTGCAT
 Consensus    agtgttgttattgttgacaattctttgatgttttgtggaggaacagaaaaacaatacacaatgttcactagaaagccgttagttcctagtgcttgtgatttggggtccaactgccacatagtagaatcaccagaagtgtccccaattcagtgggttcaatgtgaagccaaatgcaa.cgatggctgcactgtttttgcat

              3201                                                                                                                                                                                                3400
NMRB01000732  AAATCTGAAAAAGATTCCAAAAGGCAAATACATTTGTCAAGACTGTAAACGAGAAACACGTACGGGCAGAAAGAAGTAAATTAGACAATCTGCAAAACTAAGATCAAAATACCCATAAACTACAAATTAAAAGTAAAACCCAAAGACCACAAGGGAATGCACTCTGAAAATCTCACCATCCTTTCTCATCTTTGCTGATC
GFRY01056743
GFRY01085303  AAATCTGAAAAAGATTCCAAAAGGCAAATACATTTGTCAAGACTGTAAACGAGAAACACGTACGGGCAGAAAGAAGTAAATTAGACAATCTACAAAACTAAGATCAAAATACCCATAAACTACAAATTAAAAGTAAAACCCAAAGACCACAAGGGAATGCACTCTGAAAATCTCACCATCCTTTCTCATCTTTGCTGATC
 Consensus    aaatctgaaaaagattccaaaaggcaaatacatttgtcaagactgtaaacgagaaacacgtacgggcagaaagaagtaaattagacaatct.caaaactaagatcaaaatacccataaactacaaattaaaagtaaaacccaaagaccacaagggaatgcactctgaaaatctcaccatcctttctcatctttgctgatc

              3401                                              3452
NMRB01000732  CATACTGGACCCATGTTCCCGAAGCCGTCGTTAGGTTAACGATGTCGTTAAA
GFRY01056743
GFRY01085303  CATACTGGACCCATGTTCCCG
 Consensus    catactggacccatgttcccg...............................
```

>TSA GFRY01056743.1

5'3' Frame 3

QTPVSTE-KSFTIDINTQYLSCLLLLIDRL-I-STMAQLSEERVFSRLPLDDFKFLPFKSYSYDSRKVTRKRAGLELLHDEYYPAEGHCMSAVQGENSVIVYFFGGARRKDEATWSMNNIIQEMVVTYDEEDVDIVTVKTLKTTGAEVTGLQSAGFLAIPNNKCKPDFVIWGGFNMTHLKTENELLHLRYLKKEQKHHCTIFKDPNEKFKLDTDVYQSG
EVPSSRTGHSFTQISGTRLVALFGGLEMRFRERSAVTTSTLSPFTQTCQDAHFYVLNLAENKWTKLKTPEITARCYHSGTFIEIQGRQTIAIVGGIQYDGSAPCQRFPLDEIIILSFLDLDTLDVAIQNIVIPLGPDKC

>TSA GFRY01085303.1

5'3' Frame 3

LSYHSCSKLENYLVIFGGFQQYTMELEKTSPSCALHLLDLELKQHKKLSAPPVYATAGHSVVIVDNSLMFCGGTEKQYTMFTRKPLVPSACDLGSNCHIVESPEVSPIQWVQCEAKCKRWLHCFCINLKKIPKGKYICQDCKRETRTGRKK-IRQSTKLRSKYP-TTN-K-NPKTTRECTLKISPSFLIFADPYWTHVP

Aurelia Aurita (Aau)

(TaxID: 6145; Cnidaria; Scyphozoa; Semaeostomeae; Ulmaridae; Aurelia)

WGS: REGM01000520.1

58480-63820
Aurelia aurita isolate ABS-J01 scaffold520, whole genome shotgun sequence

```
       A  S  *  C  K  Y  V  Y  R  G  N  K  G  D  M  V  C  R  C  K  *  *  R  *  R  R  P  I  K  A  Y  M  Q  S  M  *  N  E  D  Q  K  V  G  K  S  S  L  F  P  W  L  G  A  V  E  K  C  F  Q  F  Q  F  R  F  *  V  I    F1
        P  V  N  V  N  M  Y  I  E  E  I  K  E  I  W  C  V  D  V  N  S  E  D  E  D  V  Q  S  K  H  I  C  R  A  C  E  M  K  I  K  R  L  A  K  V  H  C  S  L  G  L  G  L  L  K  N  V  F  N  F  N  L  G  S  K  L  S   F2
        Q  L  M  *  I  C  I  *  R  K  *  R  R  Y  G  V  S  M  *  I  V  K  M  K  T  S  N  Q  S  I  Y  A  E  H  V  K  *  R  S  K  G  W  Q  K  F  I  V  P  L  A  W  G  C  *  K  M  F  S  I  S  I  *  V  L  S  Y    F3
     1 GCCAGTTAATGTAAATATGTATATAGAGGAAATAAAGGAGATATGGTGTGTCGATGTAAATAGTGAAGATGAAGACGTCCAATCAAAGCATATATGCAGAGCATGTGAAATGAAGATCAAAAGGTTGGCAAAAGTTCATTGTTCCCTTGGCTTGGGGCTGTTGAAAAATGTTTTCAATTTCAATTTAGGTTCTAAGTTAT 200
       ----:----|----:----|----:----|----:----|----:----|----:----|----:----|----:----|----:----|----:----|----:----|----:----|----:----|----:----|----:----|----:----|----:----|----:----|----:----|----:----|
     1 CGGTCAATTACATTTATACATATATCTCCTTTATTTCCTCTATACCACACAGCTACATTTATCACTTCTACTTCTGCAGGTTAGTTTCGTATATACGTCTCGTACACTTTACTTCTAGTTTTCCAACCGTTTTCAAGTAACAAGGGAACCGAACCCCGACAACTTTTTACAAAAGTTAAAGTTAAATCCAAGATTCAATA 200
        A  L  *  H  L  Y  T  Y  L  P  F  L  P  S  I  T  H  R  H  L  Y  H  L  H  L  R  G  I  L  A  Y  I  C  L  M  H  F  S  S  *  F  T  P  L  L  E  N  N  G  Q  S  P  A  T  S  F  H  K  *  N  *  N  L  N  *  T  I   F6
       X  W  N  I  Y  I  H  I  Y  L  F  Y  L  L  Y  P  T  D  I  Y  I  T  F  I  F  V  D  L  *  L  M  Y  A  S  C  T  F  H  L  D  F  P  Q  C  F  N  M  T  G  K  A  Q  P  Q  Q  F  I  N  E  I  E  I  *  T  R  L  *    F5
        G  T  L  T  F  I  Y  I  S  S  I  F  S  I  H  H  T  S  T  F  L  S  S  S  S  T  W  D  F  C  I  H  L  A  H  S  I  F  I  L  L  N  A  F  T  *  Q  E  R  P  K  P  S  N  F  F  T  K  L  K  L  K  P  E  L  N    F4

       S  S  C  K  V  C  L  F  Y  L  K  C  *  Y  I  *  P  T  C  C  F  D  S  L  S  R  *  S  K  A  V  L  L  I  I  Y  L  F  *  T  F  N  A  V  L  S  K  F  T  *  T  I  S  P  F  Q  Q  L  H  G  T  I  T  S  L  K  R    F1
        P  H  V  R  C  A  Y  F  I  *  S  V  D  T  S  N  L  L  V  A  L  I  V  *  A  D  D  Q  K  Q  F  Y  *  L  F  I  C  S  K  L  L  M  L  Y  L  V  N  S  L  E  P  S  P  L  F  S  S  Y  M  A  L  L  Q  V  S  K   F2
       L  L  M  *  G  V  P  I  L  F  E  V  L  I  H  L  T  Y  L  L  L  *  *  F  K  Q  M  I  K  S  S  F  I  N  Y  L  F  V  L  N  F  *  C  C  T  *  *  I  H  L  N  H  L  P  F  S  A  A  T  W  H  Y  Y  K  S  Q  K    F3
   201 CTCCTCATGTAAGGTGTGCCTATTTTATTTGAAGTGTTGATACATCTAACCTACTTGTTGCTTTGATAGTTTAAGCAGATGATCAAAAGCAGTTTTATTAATTATTTATTTGTTCTAAACTTTTAATGCTGTACTTAGTAAATTCACTTGAACCATCTCCCCTTTTCAGCAGCTACATGGCACTATTACAAGTCTCAAAA 400
       ----:----|----:----|----:----|----:----|----:----|----:----|----:----|----:----|----:----|----:----|----:----|----:----|----:----|----:----|----:----|----:----|----:----|----:----|----:----|----:----|
   201 GAGGAGTACATTCCACACGGATAAAATAAACTTCACAACTATGTAGATTGGATGAACAACGAAACTATCAAATTCGTCTACTAGTTTTCGTCAAAATAATTAATAAATAAACAAGATTTGAAAATTACGACATGAATCATTTAAGTGAACTTGGTAGAGGGGAAAAGTCGTCGATGTACCGTGATAATGTTCAGAGTTTT 400
        E  E  H  L  T  H  R  N  *  K  F  H  Q  Y  M  *  G  V  Q  Q  K  S  L  K  L  L  H  D  F  A  T  K  N  I  I  *  K  N  *  V  K  L  A  T  S  L  L  N  V  Q  V  M  E  G  K  *  C  S  C  P  V  I  V  L  R  L   F6
       R  R  M  Y  P  T  G  I  K  N  S  T  N  I  C  R  V  *  K  N  S  Q  Y  N  L  C  I  I  L  L  L  K  I  L  *  K  N  T  R  F  K  *  H  Q  V  *  Y  I  *  K  F  W  R  G  K  E  A  A  V  H  C  *  *  L  D  *  F    F5
       D  G  *  T  L  H  A  *  K  I  Q  L  T  S  V  D  L  R  S  T  A  K  I  T  *  A  S  S  *  F  C  N  *  *  N  N  I  Q  E  L  S  K  I  S  Y  K  T  F  E  S  S  G  D  G  R  K  L  L  *  M  A  S  N  C  T  E  F    F4

        V  V  *  T  D  L  Q  N  G  T  *  L  S  R  K  S  W  F  K  T  V  *  F  V  Y  Q  T  L  R  T  I  V  M  P  I  K  E  T  S  N  Y  K  T  I  F  I  S  L  G  *  R  G  Q  K  H  T  K  R  V  A  Q  I  L  Q  K  F   F1
       E  L  C  R  Q  I  Y  K  M  A  L  N  C  Q  E  K  V  G  L  K  L  F  N  L  F  I  K  H  *  E  P  L  L  C  Q  *  K  K  H  Q  I  I  K  Q  F  L  S  P  *  V  K  E  V  K  S  T  Q  K  G  S  H  K  S  S  K  N  F    F2
        S  C  V  D  R  F  T  K  W  H  L  I  V  K  K  K  L  V  *  N  C  L  I  C  L  S  N  T  K  N  H  C  Y  A  N  K  R  N  I  K  L  *  N  N  F  Y  L  L  R  L  K  R  S  K  A  H  K  K  G  R  T  N  P  P  K  I  L   F3
   401 GAGTTGTGTAGACAGATTTACAAAATGGCACTTAATTGTCAAGAAAAAGTTGGTTTAAAACTGTTTAATTTGTTTATCAAACACTAAGAACCATTGTTATGCCAATAAAAGAAACATCAAATTATAAAACAATTTTTATCTCCTTAGGTTAAAGAGGTCAAAAGCACACAAAAAGGGTCGCACAAATCCTCCAAAAATTT 600
       ----:----|----:----|----:----|----:----|----:----|----:----|----:----|----:----|----:----|----:----|----:----|----:----|----:----|----:----|----:----|----:----|----:----|----:----|----:----|----:----|
   401 CTCAACACATCTGTCTAAATGTTTTACCGTGAATTAACAGTTCTTTTTCAACCAAATTTTGACAAATTAAACAAATAGTTTGTGATTCTTGGTAACAATACGGTTATTTTCTTTGTAGTTTAATATTTTGTTAAAAATAGAGGAATCCAATTTCTCCAGTTTTCGTGTGTTTTTCCCAGCGTGTTTAGGAGGTTTTTAAA 600
       L  T  T  Y  V  S  K  C  F  P  V  *  N  D  L  F  L  Q  N  L  V  T  *  N  T  *  *  V  S  L  V  M  T  I  G  I  F  S  V  D  F  *  L  V  I  K  I  E  K  P  *  L  P  *  F  C  V  F  L  T  A  C  I  R  W  F  N    F6
        L  Q  T  S  L  N  V  F  H  C  K  I  T  L  F  F  N  T  *  F  Q  K  I  Q  K  D  F  V  L  F  W  Q  *  A  L  L  L  F  M  L  N  Y  F  L  K  *  R  R  L  N  F  L  D  F  A  C  L  F  P  R  V  F  G  G  F  I   F5
        S  N  H  L  C  I  *  L  I  A  S  L  Q  *  S  F  T  P  K  F  S  N  L  K  N  I  L  C  *  S  G  N  N  H  W  Y  F  F  C  *  I  I  F  C  N  K  D  G  *  T  L  S  T  L  L  V  C  F  P  D  C  L  D  E  L  F  K   F4

       *  M  L  F  H  K  L  L  T  I  V  Q  A  A  M  F  V  T  M  I  C  Q  E  E  V  H  G  R  L  M  W  H  N  L  H  H  L  L  H  Q  C  M  Q  R  R  S  C  L  V  V  P  L  F  *  K  R  K  N  S  S  P  Q  P  Q  D  R  G    F1
        E  C  C  S  T  S  C  S  Q  L  C  K  L  Q  C  L  *  P  *  F  A  K  K  K  S  T  E  G  *  C  G  T  T  S  I  T  F  C  I  N  A  C  K  E  G  V  V  W  *  F  L  F  F  E  R  E  R  T  P  H  L  N  P  K  T  G  D   F2
        N  A  V  P  Q  V  A  H  N  C  A  S  C  N  V  C  D  H  D  L  P  R  R  S  P  R  K  A  N  V  A  Q  P  P  S  P  F  A  S  M  H  A  K  K  E  L  F  G  S  S  S  F  L  K  E  K  E  L  L  T  S  T  P  R  Q  G   F3
   601 TGAATGCTGTTCCACAAGTTGCTCACAATTGTGCAAGCTGCAATGTTTGTGACCATGATTTGCCAAGAAGAAGTCCACGGAAGGCTAATGTGGCACAACCTCCATCACCTTTTGCATCAATGCATGCAAAGAAGGAGTTGTTTGGTAGTTCCTCTTTTTTGAAAGAGAAAGAACTCCTCACCTCAACCCCAAGACAGGGG 800
       ----:----|----:----|----:----|----:----|----:----|----:----|----:----|----:----|----:----|----:----|----:----|----:----|----:----|----:----|----:----|----:----|----:----|----:----|----:----|----:----|
   601 ACTTACGACAAGGTGTTCAACGAGTGTTAACACGTTCGACGTTACAAACACTGGTACTAAACGGTTCTTCTTCAGGTGCCTTCCGATTACACCGTGTTGGAGGTAGTGGAAAACGTAGTTACGTACGTTTCTTCCTCAACAAACCATCAAGGAGAAAAAACTTTCTCTTTCTTGAGGAGTGGAGTTGGGGTTCTGTCCCC 800
        Q  I  S  N  W  L  N  S  V  I  T  C  A  A  I  N  T  V  M  I  Q  W  S  S  T  W  P  L  S  I  H  C  L  R  W  *  R  K  C  *  H  M  C  L  L  L  Q  K  T  T  G  R  K  Q  F  L  F  F  E  E  G  *  G  W  S  L  P   F6
       K  F  A  T  G  C  T  A  *  L  Q  A  L  Q  L  T  Q  S  W  S  K  G  L  L  L  G  R  F  A  L  T  A  C  G  G  D  G  K  A  D  I  C  A  F  F  S  N  N  P  L  E  E  K  K  F  S  F  S  S  R  V  E  V  G  L  C  P    F5
        S  H  Q  E  V  L  Q  E  C  N  H  L  S  C  H  K  H  G  H  N  A  L  F  F  D  V  S  P  *  H  P  V  V  E  M  V  K  Q  M  L  A  H  L  S  P  T  T  Q  Y  N  R  K  K  S  L  S  L  V  G  *  R  L  G  L  V  P   F4

        *  K  H  H  L  V  *  T  L  V  I  K  A  Q  F  L  N  Q  V  N  P  A  R  Q  T  Q  E  D  Q  A  K  Q  R  Y  H  F  S  T  *  Q  T  K  K  D  A  K  K  I  D  F  P  Q  F  S  R  N  L  M  M  S  V  R  N  R  M  R  T   F1
        E  S  I  I  W  C  R  R  W  *  S  K  P  N  S  S  I  R  *  I  Q  Q  G  K  H  K  R  T  K  Q  S  K  D  T  T  F  R  H  S  R  P  K  R  M  Q  K  K  *  T  F  H  S  F  P  G  I  *  *  C  L  *  E  T  E  *  E   F2
        M  K  A  S  F  G  V  D  A  G  D  Q  S  P  I  P  Q  S  G  E  S  S  K  A  N  T  R  G  P  S  K  A  K  I  P  L  F  D  I  A  D  Q  K  G  C  K  K  N  R  L  S  T  V  F  Q  E  F  D  D  V  C  K  K  Q  N  E  N   F3
   801 ATGAAAGCATCATTTGGTGTAGACGCTGGTGATCAAAGCCCAATTCCTCAATCAGGTGAATCCAGCAAGGCAAACACAAGAGGACCAAGCAAAGCAAAGATACCACTTTTCGACATAGCAGACCAAAAAGGATGCAAAAAAAATAGACTTTCCACAGTTTTCCAGGAATTTGATGATGTCTGTAAGAAACAGAATGAGAA 1000
       ----:----|----:----|----:----|----:----|----:----|----:----|----:----|----:----|----:----|----:----|----:----|----:----|----:----|----:----|----:----|----:----|----:----|----:----|----:----|----:----|
   801 TACTTTCGTAGTAAACCACATCTGCGACCACTAGTTTCGGGTTAAGGAGTTAGTCCACTTAGGTCGTTCCGTTTGTGTTCTCCTGGTTCGTTTCGTTTCTATGGTGAAAAGCTGTATCGTCTGGTTTTTCCTACGTTTTTTTTATCTGAAAGGTGTCAAAAGGTCCTTAAACTACTACAGACATTCTTTGTCTTACTCTT 1000
        H  F  C  *  K  T  Y  V  S  T  I  L  A  W  N  R  L  *  T  F  G  A  L  C  V  C  S  S  W  A  F  C  L  Y  W  K  E  V  Y  C  V  L  F  S  A  F  F  I  S  K  G  C  N  E  L  F  K  I  I  D  T  L  F  L  I  L   F6
        I  F  A  D  N  P  T  S  A  P  S  *  L  G  I  G  *  D  P  S  D  L  L  A  F  V  L  P  G  L  L  A  F  I  G  S  K  S  M  A  S  W  F  P  H  L  F  F  L  S  E  V  T  K  W  S  N  S  S  T  Q  L  F  C  F  S  F   F5
       S  S  L  M  M  Q  H  L  R  Q  H  D  F  G  L  E  E  I  L  H  I  W  C  P  L  C  L  L  V  L  C  L  L  S  V  V  K  R  C  L  L  G  F  L  I  C  F  F  Y  V  K  W  L  K  G  P  I  Q  H  H  R  Y  S  V  S  H  S    F4

        K  *  M  F  F  F  S  C  *  G  M  K  *  G  K  T  V  M  C  R  E  Q  R  S  L  T  I  Y  G  S  G  Q  I  D  K  Q  L  *  L  L  I  N  A  *  H  F  E  *  T  H  Y  *  A  K  G  N  T  N  H  Y  M  A  S  L  S  K   F1
       Q  N  R  C  S  F  F  H  V  E  V  *  N  E  E  K  R  *  C  A  G  S  K  G  A  *  Q  F  M  E  A  V  R  *  T  N  S  F  N  C  *  S  M  L  S  T  S  S  E  H  I  T  E  Q  R  A  I  Q  I  I  I  W  L  L  *  A  K    F2
        K  I  D  V  L  F  F  M  L  R  Y  E  M  R  K  N  G  D  V  Q  G  A  K  E  L  D  N  L  W  K  R  S  D  R  Q  T  A  L  T  A  D  Q  C  L  A  L  R  V  N  T  L  L  S  K  G  Q  Y  K  S  L  Y  G  F  F  E  Q  N   F3
  1001 CAAAATAGATGTTCTTTTTTTCATGTTGAGGTATGAAATGAGGAAAAACGGTGATGTGCAGGGAGCAAAGGAGCTTGACAATTTATGGAAGCGGTCAGATAGACAAACAGCTTTAACTGCTGATCAATGCTTAGCACTTCGAGTGAACACATTACTGAGCAAAGGGCAATACAAATCATTATATGGCTTCTTTGAGCAAA 1200
       ----:----|----:----|----:----|----:----|----:----|----:----|----:----|----:----|----:----|----:----|----:----|----:----|----:----|----:----|----:----|----:----|----:----|----:----|----:----|----:----|
  1001 GTTTTATCTACAAGAAAAAAAGTACAACTCCATACTTTACTCCTTTTTGCCACTACACGTCCCTCGTTTCCTCGAACTGTTAAATACCTTCGCCAGTCTATCTGTTTGTCGAAATTGACGACTAGTTACGAATCGTGAAGCTCACTTGTGTAATGACTCGTTTCCCGTTATGTTTAGTAATATACCGAAGAAACTCGTTT 1200
       V  F  Y  I  N  K  K  E  H  Q  P  I  F  H  P  F  V  T  I  H  L  S  C  L  L  K  V  I  *  P  L  P  *  I  S  L  C  S  *  S  S  I  L  A  *  C  K  S  H  V  C  *  Q  A  F  P  L  V  F  *  *  I  A  E  K  L  L    F6
        L  I  S  T  R  K  K  M  N  L  Y  S  I  L  F  F  P  S  T  C  P  A  F  S  S  S  L  K  H  F  R  D  S  L  C  V  A  K  V  A  S  *  H  K  A  S  R  T  F  V  N  S  L  L  P  C  Y  L  D  N  Y  P  K  K  S  C   F5
        C  F  L  H  E  K  K  *  T  S  T  H  F  S  S  F  R  H  H  A  P  L  L  P  A  Q  C  N  I  S  A  T  L  Y  V  F  L  K  L  Q  Q  D  I  S  L  V  E  L  S  C  M  V  S  C  L  A  I  C  I  M  I  H  S  R  Q  A  F   F4

       M  L  K  N  Q  F  *  H  L  Q  V  D  W  T  K  L  K  H  H  T  C  L  A  K  Q  N  T  R  F  L  T  M  K  E  I  L  C  F  T  I  *  K  L  I  Q  N  Q  E  M  L  W  A  V  L  T  Q  *  M  M  E  Y  Q  R  Q  M  P  K    F1
        C  *  K  I  S  F  S  T  S  K  *  T  G  Q  N  *  S  I  I  L  A  W  P  N  R  I  Q  D  S  *  Q  *  R  K  Y  Y  V  S  P  F  K  N  *  S  K  T  K  K  C  F  G  Q  F  *  P  N  E  *  W  S  T  N  A  K  C  Q  R   F2
        V  E  K  S  V  L  A  P  P  S  R  L  D  K  T  E  A  S  Y  L  P  G  Q  T  E  Y  K  I  L  D  N  E  G  N  I  M  F  H  H  L  K  T  D  P  K  P  R  N  A  L  G  S  F  N  P  M  N  D  G  V  P  T  P  N  A  K   F3
  1201 ATGTTGAAAAATCAGTTTTAGCACCTCCAAGTAGACTGGACAAAACTGAAGCATCATACTTGCCTGGCCAAACAGAATACAAGATTCTTGACAATGAAGGAAATATTATGTTTCACCATTTAAAAACTGATCCAAAACCAAGAAATGCTTTGGGCAGTTTTAACCCAATGAATGATGGAGTACCAACGCCAAATGCCAAA 1400
       ----:----|----:----|----:----|----:----|----:----|----:----|----:----|----:----|----:----|----:----|----:----|----:----|----:----|----:----|----:----|----:----|----:----|----:----|----:----|----:----|
  1201 TACAACTTTTTAGTCAAAATCGTGGAGGTTCATCTGACCTGTTTTGACTTCGTAGTATGAACGGACCGGTTTGTCTTATGTTCTAAGAACTGTTACTTCCTTTATAATACAAAGTGGTAAATTTTTGACTAGGTTTTGGTTCTTTACGAAACCCGTCAAAATTGGGTTACTTACTACCTCATGGTTGCGGTTTACGGTTT 1400
        I  N  F  F  *  N  *  C  R  W  T  S  Q  V  F  S  F  C  *  V  Q  R  A  L  C  F  V  L  N  K  V  I  F  S  I  N  H  K  V  M  *  F  S  I  W  F  W  S  I  S  Q  A  T  K  V  W  H  I  I  S  Y  W  R  W  I  G  F   F6
       F  T  S  F  D  T  K  A  G  G  L  L  S  S  L  V  S  A  D  Y  K  G  P  W  V  S  Y  L  I  R  S  L  S  P  F  I  I  N  *  W  K  F  V  S  G  F  G  L  F  A  K  P  L  K  L  G  I  F  S  P  T  G  V  G  F  A  L    F5
        H  Q  F  I  L  K  L  V  E  L  Y  V  P  C  F  Q  L  M  M  S  A  Q  G  F  L  I  C  S  E  Q  C  H  L  F  Y  *  T  E  G  N  L  F  Q  D  L  V  L  F  H  K  P  C  N  *  G  L  S  H  H  L  V  L  A  L  H  W   F4

        E  S  D  G  A  M  L  M  Q  L  P  R  L  *  K  K  Q  I  A  I  L  M  Q  T  F  *  Q  T  N  P  A  M  I  A  K  F  I  L  L  S  K  M  V  L  M  V  W  E  R  C  R  Y  I  K  K  K  M  T  N  F  F  Q  I  R  H  C  V   F1
        S  Q  M  V  L  C  *  C  N  C  P  D  S  E  R  S  R  *  P  F  *  C  R  H  F  D  K  P  I  Q  Q  *  *  Q  N  L  F  C  Y  Q  R  W  C  *  W  F  G  R  G  V  D  I  *  R  K  R  *  Q  I  S  S  R  *  G  T  A   F2
       G  V  R  W  C  Y  A  D  A  I  A  Q  T  L  K  E  A  D  S  H  F  D  A  D  I  L  T  N  Q  S  S  N  D  S  K  I  Y  S  V  I  K  D  G  A  D  G  L  G  E  V  S  I  Y  K  E  K  D  D  K  F  L  P  D  K  A  L  R    F3
  1401 GGAGTCAGATGGTGCTATGCTGATGCAATTGCCCAGACTCTGAAAGAAGCAGATAGCCATTTTGATGCAGACATTTTGACAAACCAATCCAGCAATGATAGCAAAATTTATTCTGTTATCAAAGATGGTGCTGATGGTTTGGGAGAGGTGTCGATATATAAAGAAAAAGATGACAAATTTCTTCCAGATAAGGCACTGCG 1600
       ----:----|----:----|----:----|----:----|----:----|----:----|----:----|----:----|----:----|----:----|----:----|----:----|----:----|----:----|----:----|----:----|----:----|----:----|----:----|----:----|
  1401 CCTCAGTCTACCACGATACGACTACGTTAACGGGTCTGAGACTTTCTTCGTCTATCGGTAAAACTACGTCTGTAAAACTGTTTGGTTAGGTCGTTACTATCGTTTTAAATAAGACAATAGTTTCTACCACGACTACCAAACCCTCTCCACAGCTATATATTTCTTTTTCTACTGTTTAAAGAAGGTCTATTCCGTGACGC 1600
        S  D  S  P  A  I  S  I  C  N  G  L  S  Q  F  F  C  I  A  M  K  I  C  V  N  Q  C  V  L  G  A  I  I  A  F  N  I  R  N  D  F  I  T  S  I  T  Q  S  L  H  R  Y  I  F  F  F  I  V  F  K  K  W  I  L  C  Q   F6
        P  T  L  H  H  *  A  S  A  I  A  W  V  R  F  S  A  S  L  W  K  S  A  S  M  K  V  F  W  D  L  L  S  L  L  I  *  E  T  I  L  S  P  A  S  P  K  P  S  T  D  I  Y  L  S  F  S  S  L  N  R  G  S  L  A  S  R   F5
       L  L  *  I  T  S  H  Q  H  L  Q  G  S  E  S  L  L  L  Y  G  N  Q  H  L  C  K  S  L  G  I  W  C  H  Y  C  F  K  N  Q  *  *  L  H  H  Q  H  N  P  L  P  T  S  I  Y  L  F  L  H  C  I  E  E  L  Y  P  V  A    F4

        L  V  L  G  *  W  N  V  L  A  S  Q  M  A  M  K  I  C  Q  C  I  V  Q  K  T  P  T  Q  C  V  *  I  D  P  C  *  N  A  L  E  M  K  T  I  G  E  H  S  V  H  Y  *  S  Q  L  S  K  R  G  N  I  *  K  I  K  L   F1
       F  *  F  W  V  S  G  M  F  L  H  L  K  W  Q  *  K  F  V  S  V  L  C  R  K  P  Q  L  S  A  Y  K  S  T  L  V  R  M  H  W  R  *  K  Q  *  G  N  T  Q  C  I  I  K  A  N  *  A  R  E  G  I  F  K  R  *  N  Y    F2
       F  S  F  G  L  V  E  C  S  C  I  S  N  G  N  E  N  L  S  V  Y  C  A  E  N  P  N  S  V  R  I  N  R  P  L  L  E  C  I  G  D  E  N  N  R  G  T  L  S  A  L  L  K  P  I  E  Q  E  R  E  Y  L  K  D  K  T  I    F3
  1601 TTTTAGTTTTGGGTTAGTGGAATGTTCTTGCATCTCAAATGGCAATGAAAATTTGTCAGTGTATTGTGCAGAAAACCCCAACTCAGTGCGTATAAATCGACCCTTGTTAGAATGCATTGGAGATGAAAACAATAGGGGAACACTCAGTGCATTATTAAAGCCAATTGAGCAAGAGAGGGAATATTTAAAAGATAAAACTA 1800
       ----:----|----:----|----:----|----:----|----:----|----:----|----:----|----:----|----:----|----:----|----:----|----:----|----:----|----:----|----:----|----:----|----:----|----:----|----:----|----:----|
  1601 AAAATCAAAACCCAATCACCTTACAAGAACGTAGAGTTTACCGTTACTTTTAAACAGTCACATAACACGTCTTTTGGGGTTGAGTCACGCATATTTAGCTGGGAACAATCTTACGTAACCTCTACTTTTGTTATCCCCTTGTGAGTCACGTAATAATTTCGGTTAACTCGTTCTCTCCCTTATAAATTTTCTATTTTGAT 1800
       T  K  T  K  P  *  H  F  T  R  A  D  *  I  A  I  F  I  Q  *  H  I  T  C  F  V  G  V  *  H  T  Y  I  S  G  Q  *  F  A  N  S  I  F  V  I  P  S  C  E  T  C  *  *  L  W  N  L  L  L  P  F  I  *  F  I  F  S    F6
        K  L  K  P  N  T  S  H  E  Q  M  E  F  P  L  S  F  K  D  T  Y  Q  A  S  F  G  L  E  T  R  I  F  R  G  K  N  S  H  M  P  S  S  F  L  L  P  V  S  L  A  N  N  F  G  I  S  C  S  L  S  Y  K  F  S  L  V   F5
       N  *  N  Q  T  L  P  I  N  K  C  R  L  H  C  H  F  N  T  L  T  N  H  L  F  G  W  S  L  A  Y  L  D  V  R  T  L  I  C  Q  L  H  F  C  Y  P  F  V  *  H  M  I  L  A  L  Q  A  L  S  P  I  N  L  L  Y  F  *    F4

       S  V  *  R  F  L  R  G  I  G  E  N  I  K  L  D  F  S  I  P  *  L  M  K  N  M  T  A  Q  *  V  V  T  K  E  L  V  P  T  I  C  V  S  S  A  M  Q  L  R  V  I  A  K  K  G  W  G  L  S  R  L  K  G  K  F  Q  I    F1
        Q  C  E  G  F  *  G  A  L  E  R  T  *  S  *  I  F  Q  F  H  D  *  *  K  I  *  P  L  N  E  W  L  P  R  S  W  F  Q  L  Y  V  C  P  L  Q  C  N  S  E  *  L  Q  R  R  A  G  D  F  Q  D  *  K  E  S  F  R  *   F2
        S  V  K  V  S  E  G  H  W  R  E  H  K  V  R  F  F  N  S  M  I  D  E  K  Y  D  R  S  M  S  G  Y  Q  G  A  G  S  N  Y  M  C  V  L  C  N  A  T  Q  S  N  C  K  E  G  L  G  T  F  K  I  E  R  K  V  S  D   F3
  1801 TCAGTGTGAAGGTTTCTGAGGGGCATTGGAGAGAACATAAAGTTAGATTTTTCAATTCCATGATTGATGAAAAATATGACCGCTCAATGAGTGGTTACCAAGGAGCTGGTTCCAACTATATGTGTGTCCTCTGCAATGCAACTCAGAGTAATTGCAAAGAAGGGCTGGGGACTTTCAAGATTGAAAGGAAAGTTTCAGAT 2000
       ----:----|----:----|----:----|----:----|----:----|----:----|----:----|----:----|----:----|----:----|----:----|----:----|----:----|----:----|----:----|----:----|----:----|----:----|----:----|----:----|
  1801 AGTCACACTTCCAAAGACTCCCCGTAACCTCTCTTGTATTTCAATCTAAAAAGTTAAGGTACTAACTACTTTTTATACTGGCGAGTTACTCACCAATGGTTCCTCGACCAAGGTTGATATACACACAGGAGACGTTACGTTGAGTCTCATTAACGTTTCTTCCCGACCCCTGAAAGTTCTAACTTTCCTTTCAAAGTCTA 2000
        D  T  H  L  N  R  L  P  M  P  S  F  M  F  N  S  K  E  I  G  H  N  I  F  F  I  V  A  *  H  T  T  V  L  S  S  T  G  V  I  H  T  D  E  A  I  C  S  L  T  I  A  F  F  P  Q  P  S  E  L  N  F  P  F  N  *  I   F6
       I  L  T  F  T  E  S  P  C  Q  L  S  C  L  T  L  N  K  L  E  M  I  S  S  F  Y  S  R  E  I  L  P  *  W  P  A  P  E  L  *  I  H  T  R  Q  L  A  V  *  L  L  Q  L  S  P  S  P  V  K  L  I  S  L  F  T  E  S    F5
        *  H  S  P  K  Q  P  A  N  S  L  V  Y  L  *  I  K  *  N  W  S  Q  H  F  I  H  G  S  L  S  H  N  G  L  L  Q  N  W  S  Y  T  H  G  R  C  H  L  E  S  Y  N  C  L  L  A  P  S  K  *  S  Q  F  S  L  K  L   F4

        T  F  S  *  H  T  M  S  E  *  I  L  T  P  *  L  K  K  K  *  V  R  Q  Q  E  A  *  K  R  F  Q  *  A  C  Q  R  R  K  R  N  L  L  M  Q  L  I  Q  T  S  T  L  A  N  F  L  G  S  *  *  L  D  L  L  L  G  *  R   F1
        P  S  A  S  T  L  C  Q  N  K  S  *  L  L  N  *  K  K  N  E  *  D  S  K  R  R  E  N  D  S  N  K  H  V  R  D  E  R  E  T  Y  *  C  N  S  Y  R  H  Q  H  W  P  I  F  *  E  V  D  N  *  T  C  C  W  G  D   F2
       N  L  Q  L  A  H  Y  V  R  I  N  P  D  S  L  I  E  K  K  M  S  E  T  A  R  G  V  K  T  I  P  I  S  M  S  E  T  K  E  K  L  I  D  A  T  H  T  D  I  N  I  G  Q  F  F  R  K  L  I  I  R  L  V  A  G  V  T    F3
  2001 AACCTTCAGCTAGCACACTATGTCAGAATAAATCCTGACTCCTTAATTGAAAAAAAAATGAGTGAGACAGCAAGAGGCGTGAAAACGATTCCAATAAGCATGTCAGAGACGAAAGAGAAACTTATTGATGCAACTCATACAGACATCAACATTGGCCAATTTTTTAGGAAGTTGATAATTAGACTTGTTGCTGGGGTGAC 2200
       ----:----|----:----|----:----|----:----|----:----|----:----|----:----|----:----|----:----|----:----|----:----|----:----|----:----|----:----|----:----|----:----|----:----|----:----|----:----|----:----|
  2001 TTGGAAGTCGATCGTGTGATACAGTCTTATTTAGGACTGAGGAATTAACTTTTTTTTTACTCACTCTGTCGTTCTCCGCACTTTTGCTAAGGTTATTCGTACAGTCTCTGCTTTCTCTTTGAATAACTACGTTGAGTATGTCTGTAGTTGTAACCGGTTAAAAAATCCTTCAACTATTAATCTGAACAACGACCCCACTG 2200
        V  K  L  *  C  V  I  D  S  Y  I  R  V  G  *  N  F  F  F  H  T  L  C  C  S  A  H  F  R  N  W  Y  A  H  *  L  R  F  L  F  K  N  I  C  S  M  C  V  D  V  N  A  L  K  K  P  L  Q  Y  N  S  K  N  S  P  H   F6
        L  R  *  S  A  C  *  T  L  I  F  G  S  E  K  I  S  F  F  I  L  S  V  A  L  P  T  F  V  I  G  I  L  M  D  S  V  F  S  F  S  I  S  A  V  *  V  S  M  L  M  P  W  N  K  L  F  N  I  I  L  S  T  A  P  T  V   F5
       Y  G  E  A  L  V  S  H  *  F  L  D  Q  S  R  L  Q  F  F  F  S  H  S  L  L  L  R  S  F  S  E  L  L  C  T  L  S  S  L  S  V  *  Q  H  L  E  Y  L  C  *  C  Q  G  I  K  *  S  T  S  L  *  V  Q  Q  Q  P  S    F4

        N  G  S  Q  Q  K  T  S  Q  *  F  *  K  M  R  N  K  S  L  M  *  Q  *  R  I  L  W  G  L  I  H  Q  *  *  C  L  V  T  M  Q  G  S  C  L  T  K  K  M  R  Q  Q  L  F  I  *  S  V  M  K  E  *  R  R  V  *  L   F1
       A  M  G  V  N  K  R  H  H  N  D  F  K  K  C  G  T  K  V  *  C  D  N  E  E  S  C  G  D  *  S  I  N  D  D  A  W  *  L  C  K  A  V  V  *  P  K  K  *  G  S  N  C  L  F  N  Q  *  *  R  S  K  E  E  F  N  C    F2
        Q  W  E  S  T  K  D  I  T  M  I  L  K  N  A  E  Q  K  F  D  V  T  M  K  N  L  V  G  I  N  P  S  M  M  M  P  G  N  Y  A  R  Q  L  F  D  Q  K  N  E  A  A  I  V  Y  L  I  S  D  E  G  V  K  K  S  L  I  A   F3
```

```
2201 GCAATGGGAGTCAACAAAAGACATCACAATGATTTTAAAAAATGCGGAACAAAAGTTTGATGTGACAATGAAGAATCTTGTGGGGATTAATCCATCAATGATGATGCCTGGTAACTATGCAAGGCAGTTGTTTGACCAAAAAAATGAGGCAGCAATTGTTTATTTAATCAGTGATGAAGGAGTAAAGAAGAGTTTAATTG 2400
     ----:----|----:----|----:----|----:----|----:----|----:----|----:----|----:----|----:----|----:----|----:----|----:----|----:----|----:----|----:----|----:----|----:----|----:----|----:----|----:----|
2201 CGTTACCCTCAGTTGTTTTCTGTAGTGTTACTAAAATTTTTTACGCCTTGTTTTCAAACTACACTGTTACTTCTTAGAACACCCCTAATTAGGTAGTTACTACTACGGACCATTGATACGTTCCGTCAACAAACTGGTTTTTTTACTCCGTCGTTAACAAATAAATTAGTCACTACTTCCTCATTTCTTCTCAAATTAAC 2400
     R L P L * C F V D C H N * F I R F L L K I H C H L I K H P N I W * H H H R T V I C P L Q K V L F I L C C N N I * D T I F S Y L L T * N F6
      C H S D V F S M V I I K F F A S C F N S T V I F F R T P I L G D I I I G P L * A L C N N S W F F S A A I T * K I L S S P T F F L K I F5
      A I P T L L L C * L S K L F H P V F T Q H S L S S D Q P S * D M L S S A Q Y S H L A T T Q G F F H P L L Q K N L * H H L L L S S N L Q F4

     L F Y K N I G F Y D Q F I E Q Q N Q T K M T L K N T R Q Q L * R W A S F F V R I L V L P V G L I I Y T K L L N M S K K C W K I Q L D Q F1
      C S T K I * V S T I S L * S N K T R Q R * H * K I Q G N S C R D G P A S L * G F * F C P L A * L S T Q N Y * T C P R N V G R S S W T R F2
      V L Q K Y R F L R S V Y R A T K P D K D D I K K Y K A T A V E M G Q L L C K D F S F A R W P N Y L H K I I E H V Q E M L E D P V G P F3
2401 CTGTTCTACAAAAATATAGGTTTCTACGATCAGTTTATAGAGCAACAAAACCAGACAAAGATGACATTAAAAAATACAAGGCAACAGCTGTAGAGATGGGCCAGCTTCTTTGTAAGGATTTTAGTTTTGCCCGTTGGCCTAATTATCTACACAAAATTATTGAACATGTCCAAGAAATGTTGGAAGATCCAGTTGGACCA 2600
     ----:----|----:----|----:----|----:----|----:----|----:----|----:----|----:----|----:----|----:----|----:----|----:----|----:----|----:----|----:----|----:----|----:----|----:----|----:----|----:----|
2401 GACAAGATGTTTTTATATCCAAAGATGCTAGTCAAATATCTCGTTGTTTTGGTCTGTTTCTACTGTAATTTTTTATGTTCCGTTGTCGACATCTCTACCCGGTCGAAGAAACATTCCTAAAATCAAAACGGGCAACCGGATTAATAGATGTGTTTTAATAACTTGTACAGGTTCTTTACAACCTTCTAGGTCAACCTGGT 2600
     S N * L F I P K * S * N I S C C F W V F I V N F F V L C C S Y L H A L K K T L I K T K G T P R I I * V F N N F M D L F H Q F I W N S W F6
     A T R C F Y L N R R D T * L A V F G S L S S M L F Y L A V A T S I P W S R Q L S K L K A R Q G L * R C L I I S C T W S I N S S G T P G F5
      Q E V F I Y T E V I L K Y L L L V L C L H C * F I C P L L Q L S P G A E K Y P N * N Q G N A * N D V C F * Q V H G L F T P L D L Q V F4

      A L L V H S A V K G M R V V I S C S G Y S G T N F Q T E E I L I K V * R M F * S F T G F I L H S S * N C L Q K * I T E R T D A P N V Q F1
      L Y W C I Q Q * R E * G W * * V V Q A I Q E P I F K P R R F L * R S R G C S K A S L A L F F T Q V R T A C R S R S P K E Q M L Q M F F2
     G S I G A F S S E G N E G G N K L F R L F R N Q F S N R G D S Y K G L E D V L K L H W L Y S S L K L E L L A E V D H R K N R C S K C S F3
2601 GGCTCTATTGGTGCATTCAGCAGTGAAGGGAATGAGGGTGGTAATAAGTTGTTCAGGCTATTCAGGAACCAATTTTCAAACCGAGGAGATTCTTATAAAGGTCTAGAGGATGTTCTAAAGCTTCACTGGCTTTATTCTTCACTCAAGTTAGAACTGCTTGCAGAAGTAGATCACCGAAAGAACAGATGCTCCAAATGTTC 2800
     ----:----|----:----|----:----|----:----|----:----|----:----|----:----|----:----|----:----|----:----|----:----|----:----|----:----|----:----|----:----|----:----|----:----|----:----|----:----|----:----|
2601 CCGAGATAACCACGTAAGTCGTCACTTCCCTTACTCCCACCATTATTCAACAAGTCCGATAAGTCCTTGGTTAAAAGTTTGGCTCCTCTAAGAATATTTCCAGATCTCCTACAAGATTTCGAAGTGACCGAAATAAGAAGTGAGTTCAATCTTGACGAACGTCTTCATCTAGTGGCTTTCTTGTCTACGAGGTTTACAAG 2800
      A R N T C E A T F P I L T T I L Q E P * E P V L K * V S S I R I F T * L I N * L K V P K I R * E L * F Q K C F Y I V S L V S A G F T F6
      P E I P A N L L S P F S P P L L N N L S N L F W N E F R P S E * L P R S S T R F S * Q S * E E S L N S S S A S T S * R F F L H E L H E F5
     L S * Q H M * C H L S H P H Y Y T T * A I * S G I K L G L L N K Y L D L P H E L A E S A K N K V * T L V A Q L L L D G F S C I S W I N F4

      S * G T I * V L V K Q N K L F K I Y D * R A P V Q H K T F D V I D L I K Q I Q A P I L Q S V L Y R L L F I F Q F Y F E A T V Y N F C F1
     R A R A Q Y E F L S N K T N S L K F M T E E P P C N T K P L T * L T * * N K Y K P P F C N L F F I V F S L S F S S I L K L Q Y T I F V F2
      E L G H N M S S C Q T K Q T L * N L * L K S P R A T Q N L * R N * P N K T N T S P H S A I C S L S S S L Y L S V L F * S Y S I Q F L C F3
2801 AGAGCTAGGGCACAATATGAGTTCTTGTCAAACAAAACAAACTCTTTAAAATTTATGACTGAAGAGCCCCCGTGCAACACAAAACCTTTGACGTAATTGACCTAATAAAACAAATACAAGCCCCCATTCTGCAATCTGTTCTTTATCGTCTTCTCTTTATCTTTCAGTTCTATTTGAAGCTACAGTATACAATTTTTGT 3000
     ----:----|----:----|----:----|----:----|----:----|----:----|----:----|----:----|----:----|----:----|----:----|----:----|----:----|----:----|----:----|----:----|----:----|----:----|----:----|----:----|
2801 TCTCGATCCCGTGTTATACTCAAGAACAGTTTGTTTTGTTTGAGAAATTTTAAATACTGACTTCTCGGGGGCACGTTGTGTTTTGGAAACTGCATTAACTGGATTATTTTGTTTATGTTCGGGGGTAAGACGTTAGACAAGAAATAGCAGAAGAGAAATAGAAAGTCAAGATAAAACTTCGATGTCATATGTTAAAAACA 3000
     * L * P V I H T R T L C F L S K L I * S Q L A G T C C L V K S T I S R I F C I C A G M R C D T R * R R R K I K * N * K S A V T Y L K Q F6
      S S P C L I L E Q * V F C V R * F K H S F L G R A V C F R Q R L Q G L L V F V L G W E A I Q E K D D E R * R E T R N Q L * L I C N K F5
      L A L A C Y S N K D F L V F E K F N I V S S G G H L V F G K V Y N V * Y F L Y L G G N Q L R N K I T K E K D K L E I K F S C Y V I K T F4

     A I F E C N L L Q H Y D F R P Y C G K A L M G A F V C V C L I L A N H L * F F V L L E R G S F S Q K F * R I S L * F D S Q E L H C G I F1
      Q Y L N A I C C S I M I L D H I A E R L * W G L L F V F A * F W Q I I S N S L C Y L N G A L S V K N F R G F L Y D L T V K N C I V V L F2
      N I * M Q F V A A L * F * T I L R K G F N G G F C L C L L D F G K S S L I L C A T * T G L F Q S K I L E D F F M I * Q S R I A L W Y F3
3001 GCAATATTTGAATGCAATTTGTTGCAGCATTATGATTTTAGACCATATTGCGGAAAGGCTTTAATGGGGGCTTTTGTTTGTGTTTGCTTGATTTTGGCAAATCATCTCTAATTCTTTGTGCTACTTGAACGGGGCTCTTTCAGTCAAAAATTTTAGAGGATTTCTTTATGATTTGACAGTCAAGAATTGCATTGTGGTAT 3200
     ----:----|----:----|----:----|----:----|----:----|----:----|----:----|----:----|----:----|----:----|----:----|----:----|----:----|----:----|----:----|----:----|----:----|----:----|----:----|----:----|
3001 CGTTATAAACTTACGTTAAACAACGTCGTAATACTAAAATCTGGTATAACGCCTTTCCGAAATTACCCCCGAAAACAAACACAAACGAACTAAAACCGTTTAGTAGAGATTAAGAAACACGATGAACTTGCCCCGAGAAAGTCAGTTTTTAAAATCTCCTAAAGAAATACTAAACTGTCAGTTCTTAACGTAACACCATA 3200
      A I N S H L K N C C * S K L G Y Q P F A K I P A K T Q T Q K I K A F * R * N K T S S S R P E K L * F N * L I E K H N S L * S N C Q P I F6
     H L I Q I C N T A A N H N * V M N R F P K L P P K Q K H K S S K P L D D R I R Q A V Q V P S K * D F I K S S K K I I Q C D L I A N H Y F5
      C Y K F A I Q Q L M I I K S W I A S L S * H P S K N T N A Q N Q C I M E L E K H * K F P A R E T L F K L P N R * S K V T L F Q M T T F4

      V G X K L F R L F R N Q F S N R G D S Y K G L E D V L K L H W L Y S S L K L E L L A E V D H R K N R C S K C S E L G H N M S S C Q T K F1
      L V X S C S G Y S G T N F Q T E E I L I K V * R M F * S F T G F I L H S S * N C L Q K * I T E R T D A P N V Q S * G T I * V L V K Q F2
     C W X * V V Q A I Q E P I F K P R R F L * R S R G C S K A S L A L F F T Q V R T A C R S R S P K E Q M L Q M F R A R A Q Y E F L S N K F3
3201 TGTTGGTGNTAAGTTGTTCAGGCTATTCAGGAACCAATTTTCAAACCGAGGAGATTCTTATAAAGGTCTAGAGGATGTTCTAAAGCTTCACTGGCTTTATTCTTCACTCAAGTTAGAACTGCTTGCAGAAGTAGATCACCGAAAGAACAGATGCTCCAAATGTTCAGAGCTAGGGCACAATATGAGTTCTTGTCAAACAA 3400
     ----:----|----:----|----:----|----:----|----:----|----:----|----:----|----:----|----:----|----:----|----:----|----:----|----:----|----:----|----:----|----:----|----:----|----:----|----:----|----:----|
3201 ACAACCACNATTCAACAAGTCCGATAAGTCCTTGGTTAAAAGTTTGGCTCCTCTAAGAATATTTCCAGATCTCCTACAAGATTTCGAAGTGACCGAAATAAGAAGTGAGTTCAATCTTGACGAACGTCTTCATCTAGTGGCTTTCTTGTCTACGAGGTTTACAAGTCTCGATCCCGTGTTATACTCAAGAACAGTTTGTT 3400
      T P X L N N L S N L F W N E F R P S E * L P R S S T R F S * Q S * E E S L N S S S A S T S * R F F L H E L H E S S P C L I L E Q * V F6
      Q Q H X T T * A I * S G I K L G L L N K Y L D L P H E L A E S A K N K V * T L V A Q L L L D G F S C I S W I N L A L A C Y S N K D F L F5
     N N T X L Q E P * E P V L K * V S S I R I F T * L I N * L K V P K I R * E L * F Q K C F Y I V S L V S A G F T * L * P V I H T R T L C F4

      Q T L * N L * L K S P R A T Q N L * R N * P N K T N T S P H S A I C S L S S S L Y L S V L F * S Y S I Q F L C N I * M Q F V A A L * F1
     N K L F K I Y D * R A P V Q H K T F D V I D L I K Q I Q A P I L Q S V L Y R L L F I F Q F Y F E A T V Y N F C A I F E C N L L Q H Y D F2
      T N S L K F M T E E P P C N T K P L T * L T * * N K Y K P P F C N L F F I V F S L S F S S I L K L Q Y T I F V Q Y L N A I C C S I M I F3
3401 AACAAACTCTTTAAAATTTATGACTGAAGAGCCCCCGTGCAACACAAAACCTTTGACGTAATTGACCTAATAAAACAAATACAAGCCCCCATTCTGCAATCTGTTCTTTATCGTCTTCTCTTTATCTTTCAGTTCTATTTGAAGCTACAGTATACAATTTTTGTGCAATATTTGAATGCAATTTGTTGCAGCATTATGA 3600
     ----:----|----:----|----:----|----:----|----:----|----:----|----:----|----:----|----:----|----:----|----:----|----:----|----:----|----:----|----:----|----:----|----:----|----:----|----:----|----:----|
3401 TTGTTTGAGAAATTTTAAATACTGACTTCTCGGGGGCACGTTGTGTTTTGGAAACTGCATTAACTGGATTATTTTGTTTATGTTCGGGGGTAAGACGTTAGACAAGAAATAGCAGAAGAGAAATAGAAAGTCAAGATAAAACTTCGATGTCATATGTTAAAAACACGTTATAAACTTACGTTAAACAACGTCGTAATACT 3600
     F C V R * F K H S F L G R A V C F R Q R L Q G L L V F V L G W E A I Q E K D D E R * R E T R N Q L * L I C N K H L I Q I C N T A A N H F6
      V F E K F N I V S S G G H L V F G K V Y N V * Y F L Y L G G N Q L R N K I T K E K D K L E I K F S C Y V I K T C Y K F A I Q Q L M I F5
      F L S K L I * S Q L A G T C C L V K S T I S R I F C I C A G M R C D T R * R R R K I K * N * K S A V T Y L K Q A I N S H L K N C C * S F4

     F * T I L R K G F N G G F C L C L L D F G K S S L I L C A T * T G L F Q S K I L E D F F M I * Q S R I A L W Y C W * A Q S M P V D N G F1
      F R P Y C G K A L M G A F V C V C L I L A N H L * F F V L L E R G S F S Q K F * R I S L * F D S Q E L H C G I V G E P K A C R L T M G F2
      L D H I A E R L * W G L L F V F A * F W Q I I S N S L C Y L N G A L S V K N F R G F L Y D L T V K N C I V V L L V S P K H A G * Q W F3
3601 TTTTAGACCATATTGCGGAAAGGCTTTAATGGGGGCTTTTGTTTGTGTTTGCTTGATTTTGGCAAATCATCTCTAATTCTTTGTGCTACTTGAACGGGGCTCTTTCAGTCAAAAATTTTAGAGGATTTCTTTATGATTTGACAGTCAAGAATTGCATTGTGGTATTGTTGGTGAGCCCAAAGCATGCCGGTTGACAATGG 3800
     ----:----|----:----|----:----|----:----|----:----|----:----|----:----|----:----|----:----|----:----|----:----|----:----|----:----|----:----|----:----|----:----|----:----|----:----|----:----|----:----|
3601 AAAATCTGGTATAACGCCTTTCCGAAATTACCCCCGAAAACAAACACAAACGAACTAAAACCGTTTAGTAGAGATTAAGAAACACGATGAACTTGCCCCGAGAAAGTCAGTTTTTAAAATCTCCTAAAGAAATACTAAACTGTCAGTTCTTAACGTAACACCATAACAACCACTCGGGTTTCGTACGGCCAACTGTTACC 3800
      N * V M N R F P K L P P K Q K H K S S K P L D D R I R Q A V Q V P S K * D F I K S S K K I I Q C D L I A N H Y Q Q H A W L M G T S L P F6
     I K S W I A S L S * H P S K N T N A Q N Q C I M E L E K H * K F P A R E T L F K L P N R * S K V T L F Q M T T N N T L G F C A P Q C H F5
      K L G Y Q P F A K I P A K T Q T Q K I K A F * R * N K T S S S R P E K L * F N * L I E K H N S L * S N C Q P I T P S G L A H R N V I F4

      S L K K Y S F C K L C T F L S L S L W L L A V I T E K F S F W Q S F * L H A K L M K P S F A A A I T F N P R D R C * D F I I N D T F I F1
      L * R N I L F V N Y A R F F L * V F G F W Q S L Q K N F P F G R V F S C M Q N * * S H L L Q L P S H L I Q G I G V K T L S S M I H S F2
     V F E E I F F L * I M H V S F F E S L A S G S H Y R K I F L L A E F L V A C K I D E A I F C S C H H I * S K G * V L R L Y H Q * Y I H F3
3801 GTCTTTGAAGAAATATTCTTTTTGTAAATTATGCACGTTTCTTTCTTTGAGTCTTTGGCTTCTGGCAGTCATTACAGAAAAATTTTCCTTTTGGCAGAGTTTTTAGTTGCATGCAAAATTGATGAAGCCATCTTTTGCAGCTGCCATCACATTTAATCCAAGGGATAGGTGTTAAGACTTTATCATCAATGATACATTCA 4000
     ----:----|----:----|----:----|----:----|----:----|----:----|----:----|----:----|----:----|----:----|----:----|----:----|----:----|----:----|----:----|----:----|----:----|----:----|----:----|----:----|
3801 CAGAAACTTCTTTATAAGAAAAACATTTAATACGTGCAAAGAAAGAAACTCAGAAACCGAAGACCGTCAGTAATGTCTTTTTAAAAGGAAAACCGTCTCAAAAATCAACGTACGTTTTAACTACTTCGGTAGAAAACGTCGACGGTAGTGTAAATTAGGTTCCCTATCCACAATTCTGAAATAGTAGTTACTATGTAAGT 4000
      D K F F Y E K Q L N H V N R E K L R Q S R A T M V S F N E K Q C L K * N C A F N I F G D K A A A M V N L G L S L H * S K I M L S V N F6
      T K S S I N K K Y I I C T E K K S D K A E P L * * L F I K R K A S N K T A H L I S S A M K Q L Q W * M * D L P Y T N L S * * * H Y M * F5
     P R Q L F I R K T F * A R K K R Q T K P K Q C D N C F F K G K P L T K L Q M C F Q H L W R K C S G D C K I W P I P T L V K D D I I C E F4

      T K L T L I W F H C F L C K K T Q T L F C S T T Y Q K * V I I Y E * H G V A S S C K F L G G C N F Q K Q F * N C I N N V Q G C R W G F1
     S P S S H * S G F I A F F V K R L K L C F V P P H I K S E S S S M N D M E W P A V A N S W G A A T F K N N F E I V S I M Y K V A D G G F2
      H Q A H T D L V S L L L S L * K D S N F V L F H H I S K V S H H L * M T W S G Q Q L Q I L G G L Q L S K T I L K L Y Q * C T R L Q M G D F3
4001 TCACCAAGCTCACACTGATCTGGTTTCATTGCTTTCTTTGTAAAAAGACTCAAACTTTGTTTTGTTCCACCACATATCAAAAGTGAGTCATCATCTATGAATGACATGGAGTGGCCAGCAGTTGCAAATTCTTGGGGGGCTGCAACTTTCAAAAACAATTTTGAAATTGTATCAATAATGTACAAGGTTGCAGATGGGGG 4200
     ----:----|----:----|----:----|----:----|----:----|----:----|----:----|----:----|----:----|----:----|----:----|----:----|----:----|----:----|----:----|----:----|----:----|----:----|----:----|----:----|
4001 AGTGGTTCGAGTGTGACTAGACCAAAGTAACGAAAGAAACATTTTTCTGAGTTTGAAACAAAACAAGGTGGTGTATAGTTTTCACTCAGTAGTAGATACTTACTGTACCTCACCGGTCGTCAACGTTTAAGAACCCCCCGACGTTGAAAGTTTTTGTTAAAACTTTAACATAGTTATTACATGTTCCAACGTCTACCCCC 4200
     M V L S V S I Q N * Q K R Q L F V * V K N Q E V V Y * F H T M M * S H C P T A L L Q L N K P P Q L K * F C N Q F Q I L L T C P Q L H P F6
      * W A * V S R T E N S E K Y F S E F K T K N W W M D F T L * * R H I V H L P W C N C I R P P S C S E F V I K F N Y * Y H V L N C I P F5
      D G L E C Q D P K M A K K T F L S L S Q K T G G C I L L S D D D I F S M S H G A T A F E Q P A A V K L F L K S I T D I I Y L T A S P P F4

     M S I * N Q F G A L L V A T K N Q Q V R W M N R G N S S S M I * N E D I H I * V W K I K * R Y F N L V T I T K F Q A N S I F N R N P S F1
      C R F E T S S V L C W * P P K I S K * D G * I G E T A A A * F E M K I F T S K F G K S S E D I S I L S L S P S F K L T A S L I E I R L F2
      V D L K P V R C S A G S H Q K S A S E M D E S G K Q Q Q H D L K * R Y S H L S L E N Q V K I F Q S C H Y H Q V S S * Q H L * * K S V F3
4201 ATGTCGATTTGAAACCAGTTCGGTGCTCTGCTGGTAGCCACCAAAAATCAGCAAGTGAGATGGATGAATCGGGGAAACAGCAGCAGCATGATTTGAAATGAAGATATTCACATCTAAGTTTGGAAAATCAAGTGAAGATATTTCAATCTTGTCACTATCACCAAGTTTCAAGCTAACAGCATCTTTAATAGAAATCCGTC 4400
     ----:----|----:----|----:----|----:----|----:----|----:----|----:----|----:----|----:----|----:----|----:----|----:----|----:----|----:----|----:----|----:----|----:----|----:----|----:----|----:----|
4201 TACAGCTAAACTTTGGTCAAGCCACGAGACGACCATCGGTGGTTTTTAGTCGTTCACTCTACCTACTTAGCCCCTTTGTCGTCGTCGTACTAAACTTTACTTCTATAAGTGTAGATTCAAACCTTTTAGTTCACTTCTATAAAGTTAGAACAGTGATAGTGGTTCAAAGTTCGATTGTCGTAGAAATTATCTTTAGGCAG 4400
      I D I Q F W N P A R S T A V L F * C T L H I F R P F L L L M I Q F S S I * M * T Q F I L H L Y K L R T V I V L N * A L L M K L L F G D F6
     S T S K F G T R H E A P L W W F D A L S I S S D P F C C C C S K F H L Y E C R L K S F * T F I N * D Q * * * W T E L * C C R * Y F D T F5
      H R N S V L E T S Q Q Y G G F I L L H S P H I P S V A A A H N S I F I N V D L N P F D L S S I E I K D S D G L K L S V A D K I S I R F4

      C D F T I * K K C T S N Q * N F V I N Q S C S V K C P C C N S N I S I Q N A P L G Q Y F I L F L F Q A Q V V N H R V W V G * L K Y I * F1
      V I L P S E K N A P P I N K I L S S T K V A V * N V L V A T A T F L S K M L H L G N I S S S F F S K H K L * I T G S G W A S * N I F F2
     L * F Y H L K K M H L Q S I K F C H Q P K L Q C E M S L L Q Q Q H F Y P K C S T W A I F H P L S F P S T S C K S Q G L G G L V E I Y L F3
4401 TTGTGATTTTACCATCTGAAAAAAATGCACCTCCAATCAATAAAATTTTGTCATCAACCAAAGTTGCAGTGTGAAATGTCCTTGTTGCAACAGCAACATTTCTATCCAAAATGCTCCACTTGGGCAATATTTCATCCTCTTTCTTTTCCAAGCACAAGTTGTAAATCACAGGGTCTGGGTGGGCTAGTTGAAATATATTT 4600
     ----:----|----:----|----:----|----:----|----:----|----:----|----:----|----:----|----:----|----:----|----:----|----:----|----:----|----:----|----:----|----:----|----:----|----:----|----:----|----:----|
4401 AACACTAAAATGGTAGACTTTTTTTACGTGGAGGTTAGTTATTTTAAAACAGTAGTTGGTTTCAACGTCACACTTTACAGGAACAACGTTGTCGTTGTAAAGATAGGTTTTACGAGGTGAACCCGTTATAAAGTAGGAGAAAGAAAAGGTTCGTGTTCAACATTTAGTGTCCCAGACCCACCCGATCAACTTTATATAAA 4600
      Q S K V M Q F F H V E L * Y F K T M L W L Q L T F H G Q Q L L L M E I W F A G S P C Y K M R K R K W A C T T F * L T Q T P * N F Y I F6
      K H N * W R F F I C R W D I F N Q * * G F N C H S I D K N C C C C K * G F H E V Q A I N * G R E K G L V L Q L D C P R P P S T S I Y K F5
     R T I K G D S F F A G G I L L I K D D V L T A T H F T R T A V A V N R D L I S W K P L I E D E K K E L C L N Y I V P D P H A L Q F I N F4

      T I K R P I C Y I R H * Q S T H H D K S V S H L S E C V S K T S C W Y T S R L N C N I L K Q W I T E I N L S I L G T G K N F S V K L F1
     E Q S K D L F V T L G I D N P P T M I K A S P I * V S V C P K R A A G I L P D * T A T F L N N G L L K S I S A F W A L G K I L A L S F F2
     N N Q K T Y L L H * A L T I H P P * * K R L P F E * V C V Q N E L L V Y F Q I E L Q H S * T M D Y * N Q S Q H F G H W E K F * R * A F F3
4601 GAACAATCAAAAGACCTATTTGTTACATTAGGCATTGACAATCCACCCACCATGATAAAAGCGTCTCCCATTTGAGTGAGTGTGTGTCCAAAACGAGCTGCTGGTATACTTCCAGATTGAACTGCAACATTCTTAAACAATGGATTACTGAAATCAATCTCAGCATTTTGGGCACTGGGAAAAATTTTAGCGTTAAGCTT 4800
     ----:----|----:----|----:----|----:----|----:----|----:----|----:----|----:----|----:----|----:----|----:----|----:----|----:----|----:----|----:----|----:----|----:----|----:----|----:----|----:----|
4601 CTTGTTAGTTTTCTGGATAAACAATGTAATCCGTAACTGTTAGGTGGGTGGTACTATTTTCGCAGAGGGTAAACTCACTCACACACAGGTTTTGCTCGACGACCATATGAAGGTCTAACTTGACGTTGTAAGAATTTGTTACCTAATGACTTTAGTTAGAGTCGTAAAACCCGTGACCCTTTTTAAAATCGCAATTCGAA 4800
     Q V I L L G I Q * M L C Q C D V W W S L L T E W K L S H T D L V L Q Q Y V E L N F Q L M R L C H I V S I L R L M K P V P F F K L T L S F6
      F L * F V * K N C * A N V I W G G H Y F R R G N S H T H T W F S S S T Y K W I S S C C E * V I S * Q F * D * C K P C Q S F N * R * A F5
      S C D F S R N T V N P M S L G G V M I F A D G M Q T L T H G F R A A P I S G S Q V A V N K F L P N S F D I E A N Q A S P F I K A N L K F4

     S F V S F F A Y Q * N * I I Y W A Q L H Q V Q S T P N K N F C F F L F I H A G N K S S F * E C W * L G T R R F Y K S C I F Y K N V I L F1
      L L C P F L L T N K I K S S I G R N C I K S N P P Q I K I S V S S F S F M L V T K A A F E S A G N W E P D V F T N P A S S I K M S F S F2
      F C V L F C L P I K L N H L L G A T A S S P I H P K * K F L F L P F H S C W * Q K Q L L R V L V I G N Q T F L Q I L H L L * K C H S F3
```

```
                                                                                                                                    
4801 TCTTTTGTGTCCTTTTTTGCTTACCAATAAAATTAAATCATCTATTGGGCGCAACTGCATCAAGTCCAATCCACCCCAAATAAAAATTTCTGTTTCTTCCTTTTCATTCATGCTGGTAACAAAAGCAGCTTTTGAGAGTGCTGGTAATTGGGAACCAGACGTTTTTACAAATCCTGCATCTTCTATAAAAATGTCATTCT 5000
     ----:----|----:----|----:----|----:----|----:----|----:----|----:----|----:----|----:----|----:----|----:----|----:----|----:----|----:----|----:----|----:----|----:----|----:----|----:----|----:----|
4801 AGAAAACACAGGAAAAAACGAATGGTTATTTTAATTTAGTAGATAACCCGCGTTGACGTAGTTCAGGTTAGGTGGGGTTTATTTTTAAAGACAAAGAAGGAAAAGTAAGTACGACCATTGTTTTCGTCGAAAACTCTCACGACCATTAACCCTTGGTCTGCAAAAATGTTTAGGACGTAGAAGATATTTTTACAGTAAGA 5000
      E  K  T  D  K  K  A  *  W  Y  F  *  I  M  *  Q  A  C  S  C  *  T  W  D  V  G  F  L  F  K  Q  K  K  R  K  M  *  A  P  L  L  L  L  K  Q  S  H  Q  Y  N  P  V  L  R  K  *  L  D  Q  M  K  *  L  F  T  M  R   F6
     K  K  Q  T  R  K  Q  K  G  I  F  N  F  *  R  N  P  A  V  A  D  L  G  I  W  G  L  Y  F  N  R  N  R  G  K  *  E  H  Q  Y  C  F  C  S  K  L  T  S  T  I  P  F  W  V  N  K  C  I  R  C  R  R  Y  F  H  *  E    F5
       R  K  H  G  K  K  S  V  L  L  I  L  D  D  I  P  R  L  Q  M  L  D  L  G  G  W  I  F  I  E  T  E  E  K  E  N  M  S  T  V  F  A  A  K  S  L  A  P  L  Q  S  G  S  T  K  V  F  G  A  D  E  I  F  I  D  N    F4

      S  Y  F  I  C  I  I  L  E  T  R  G  K  A  P  S  G  S  C  *  I  L  C  S  A  K  I  K  T  P  Y  *  K  I  S  C  H  L  C  I  A  I  F  I  I  L  F  V  T  T  K  I  F  H  Q  K  H  *  M  P  F  W  R  I  E  F  I   F1
       V  T  S  Y  V  *  Y  W  R  L  E  A  K  L  Q  V  D  P  A  E  F  C  A  P  P  R  *  K  H  R  T  E  K  L  A  V  I  S  A  S  P  S  S  S  S  F  L  S  P  P  K  S  F  I  K  N  I  K  C  P  S  G  G  *  N  S    F2
     Q  L  L  H  M  Y  N  I  G  D  *  R  Q  S  S  K  W  I  L  L  N  F  V  L  R  Q  D  K  N  T  V  L  K  N  *  L  S  S  L  H  R  H  L  H  H  P  F  C  H  H  Q  N  L  S  S  K  T  L  N  A  L  L  E  D  R  I  H   F3
5001 CAGTTACTTCATATGTATAATATTGGAGACTAGAGGCAAAGCTCCAAGTGGATCCTGCTGAATTTTGTGCTCCGCCAAGATAAAAACACCGTACTGAAAAATTAGCTGTCATCTCTGCATCGCCATCTTCATCATCCTTTTTGTCACCACCAAAATCTTTCATCAAAAACATTAAATGCCCTTCTGGAGGATAGAATTCA 5200
     ----:----|----:----|----:----|----:----|----:----|----:----|----:----|----:----|----:----|----:----|----:----|----:----|----:----|----:----|----:----|----:----|----:----|----:----|----:----|----:----|
5001 GTCAATGAAGTATACATATTATAACCTCTGATCTCCGTTTCGAGGTTCACCTAGGACGACTTAAAACACGAGGCGGTTCTATTTTTGTGGCATGACTTTTTAATCGACAGTAGAGACGTAGCGGTAGAAGTAGTAGGAAAAACAGTGGTGGTTTTAGAAAGTAGTTTTTGTAATTTACGGGAAGACCTCCTATCTTAAGT 5200
       L  *  K  M  H  I  I  N  S  V  L  P  L  A  G  L  P  D  Q  Q  I  K  H  E  A  L  I  F  V  G  Y  Q  F  I  L  Q  *  R  Q  M  A  M  K  M  M  R  K  T  V  V  L  I  K  *  *  F  C  *  I  G  K  Q  L  I  S  N    F6
      *  N  S  *  I  Y  L  I  P  S  *  L  C  L  E  L  H  I  R  S  F  K  T  S  R  W  S  L  F  V  T  S  F  F  *  S  D  D  R  C  R  W  R  *  *  G  K  Q  *  W  W  F  R  E  D  F  V  N  F  A  R  R  S  S  L  I  *   F5
     E  T  V  E  Y  T  Y  Y  Q  L  S  S  A  F  S  W  T  S  G  A  S  N  Q  A  G  G  L  Y  F  C  R  V  S  F  N  A  T  M  E  A  D  G  D  E  D  D  K  K  D  G  G  F  D  K  M  L  F  M  L  H  G  E  P  P  Y  F  E   F4

       S  F  N  V  L  K  V  F  F  P  S  Q  F  P  C  I  F  T  P  F  F  K  R  N  E  F  E  I  F  N  I  Y  H  S  N  W  R  M  R  H  F  D  F  K  N  R  X                                                        F1
     F  L  S  M  S  S  R  C  F  F  L  V  S  F  L  V  S  S  L  L  S  L  K  E  M  N  L  K  S  S  T  S  I  T  A  T  G  E  C  A  I  L  I  L  K  I  A                                                          F2
      F  F  Q  C  P  Q  G  V  F  S  *  S  V  S  L  Y  L  H  S  F  L  *  K  K  *  I  *  N  L  Q  H  L  S  Q  Q  L  E  N  A  P  F  *  F  *  K  S  X                                                         F3
5201 TTTCTTTCAATGTCCTCAAGGTGTTTTTTCCTAGTCAGTTTCCTTGTATCTTCACTCCTTTCTTTAAAAGAAATGAATTTGAAATCTTCAACATCTATCACAGCAACTGGAGAATGCGCCATTTTGATTTTAAAAATCGCA 5341
     ----:----|----:----|----:----|----:----|----:----|----:----|----:----|----:----|----:----|----:----|----:----|----:----|----:----|----:----|-
5201 AAAGAAAGTTACAGGAGTTCCACAAAAAAGGATCAGTCAAAGGAACATAGAAGTGAGGAAAGAAATTTTCTTTACTTAAACTTTAGAAGTTGTAGATAGTGTCGTTGACCTCTTACGCGGTAAAACTAAAATTTTTAGCGT 5341
     M  E  K  L  T  R  L  T  N  K  G  L  *  N  G  Q  I  K  V  G  K  K  L  L  F  S  N  S  I  K  L  M  *  *  L  L  Q  L  I  R  W  K  S  K  L  F  R                                                          F6
       K  K  *  H  G  *  P  T  K  E  *  D  T  E  K  Y  R  *  E  K  R  *  F  F  H  I  Q  F  R  *  C  R  D  C  C  S  S  F  A  G  N  Q  N  *  F  D  C                                                        F5
      N  R  E  I  D  E  L  H  K  K  R  T  L  K  R  T  D  E  S  R  E  K  F  S  I  F  K  F  D  E  V  D  I  V  A  V  P  S  H  A  M  K  I  K  F  I  A                                                         F4
```

B.lanceolatum(Bla)

(TaxID: 7740; Deuterostomia; Chordata; Cephalochordata; Branchiostomidae)

WGS: FLLO01000298.1

150587-157000
Branchiostoma lanceolatum genome assembly, contig: Sc0000297, whole genome shotgun sequence

```
     D  R  T  V  N  R  H  G  S  E  E  T  R  P  L  R  G  P  E  E  R  S  R  A  E  G  E  E  T  E  T  A  T  D  A  A  T  T  S  A  A  R  L  V  V  G  G  G  H  G  Q  I  W  D  I  L  E  *  S  R  L  R  V  D  P  F  S   F1
      I  E  P  *  T  D  M  G  R  K  R  P  D  P  S  E  A  Q  K  K  G  P  G  R  K  A  R  K  Q  K  P  P  Q  M  P  R  Q  L  L  Q  Q  G  *  L  W  E  G  G  T  V  R  S  G  I  F  *  S  R  V  D  *  E  *  I  H  S  A   F2
       *  N  R  E  Q  T  W  V  G  R  D  Q  T  P  P  R  P  R  R  K  V  P  G  G  R  R  G  N  R  N  R  H  R  C  R  D  N  F  C  S  K  V  S  C  G  R  G  A  R  S  D  L  G  Y  F  R  V  E  *  T  K  S  R  S  I  Q   F3
   1 GATAGAACCGTGAACAGACATGGGTCGGAAGAGACCAGACCCCTCCGAGGCCCAGAAGAAAGGTCCCGGGCGGAAGGCGAGGAAACAGAAACCGCCACAGATGCCGCGACAACTTCTGCAGCAAGGTTAGTTGTGGGAGGGGGGCACGGTCAGATCTGGGATATTTTAGAGTAGAGTAGACTAGAGTAGATCCATTCAG 200
     ----:----|----:----|----:----|----:----|----:----|----:----|----:----|----:----|----:----|----:----|----:----|----:----|----:----|----:----|----:----|----:----|----:----|----:----|----:----|----:----|
   1 CTATCTTGGCACTTGTCTGTACCCAGCCTTCTCTGGTCTGGGGAGGCTCCGGGTCTTCTTTCCAGGGCCCGCCTTCCGCTCCTTTGTCTTTGGCGGTGTCTACGGCGCTGTTGAAGACGTCGTTCCAATCAACACCCTCCCCCCGTGCCAGTCTAGACCCTATAAAATCTCATCTCATCTGATTCTCATCTAGGTAAGTC 200
      S  L  V  T  F  L  C  P  D  S  S  V  L  G  R  R  P  G  S  S  L  D  R  A  S  P  S  S  V  S  V  A  V  S  A  A  V  V  E  A  A  L  N  T  T  P  P  P  C  P  *  I  Q  S  I  K  S  Y  L  L  S  L  T  S  G  N  L   F6
     X  Y  F  R  S  C  V  H  T  P  L  S  W  V  G  G  L  G  L  L  F  T  G  P  P  L  R  P  F  L  F  R  W  L  H  R  S  L  K  Q  L  L  T  L  Q  P  L  P  A  R  D  S  R  P  Y  K  L  T  S  Y  V  L  L  L  D  M  *   F5
       I  S  G  H  V  S  M  P  R  F  L  G  S  G  E  S  A  W  F  F  P  G  P  R  F  A  L  F  C  F  G  G  C  I  G  R  C  S  R  C  C  P  *  N  H  S  P  P  V  T  L  D  P  I  N  *  L  L  T  S  *  S  Y  I  W  E   F4

     K  V  G  K  F  L  P  V  D  T  A  H  P  I  A  A  I  Q  L  L  N  C  H  S  R  Y  K  S  Y  R  *  L  R  R  N  K  F  V  V  *  A  S  K  N  M  K  R  A  F  Y  S  *  Q  S  K  F  C  V  T  Q  V  T  W  H  T  F  T   F1
      R  *  E  N  F  F  Q  L  I  P  L  I  Q  L  L  P  S  N  Y  *  T  V  I  P  G  T  N  P  I  G  D  C  V  E  T  N  L  L  F  E  H  P  K  I  *  K  G  P  F  I  A  D  S  Q  N  F  V  *  P  R  *  P  G  I  H  L   F2
       Q  G  R  K  I  S  S  S  *  Y  R  S  S  N  C  C  H  P  I  T  E  L  S  F  Q  V  Q  I  L  *  V  I  A  *  K  Q  I  C  C  L  S  I  Q  K  Y  E  K  G  L  L  *  L  T  V  K  I  L  C  D  P  G  N  L  A  Y  I  Y   F3
 201 CAAGGTAGGAAAATTTCTTCCAGTTGATACCGCTCATCCAATTGCTGCCATCCAATTACTGAACTGTCATTCCAGGTACAAATCCTATAGGTGATTGCGTAGAAACAAATTTGTTGTTTGAGCATCCAAAAATATGAAAAGGGCCTTTTATAGCTGACAGTCAAAATTTTGTGTGACCCAGGTAACCTGGCATACATTTA 400
     ----:----|----:----|----:----|----:----|----:----|----:----|----:----|----:----|----:----|----:----|----:----|----:----|----:----|----:----|----:----|----:----|----:----|----:----|----:----|----:----|
 201 GTTCCATCCTTTTAAAGAAGGTCAACTATGGCGAGTAGGTTAACGACGGTAGGTTAATGACTTGACAGTAAGGTCCATGTTTAGGATATCCACTAACGCATCTTTGTTTAAACAACAAACTCGTAGGTTTTTATACTTTTCCCGGAAAATATCGACTGTCAGTTTTAAAACACACTGGGTCCATTGGACCGTATGTAAAT 400
      L  T  P  F  N  R  G  T  S  V  A  *  G  I  A  A  M  W  N  S  F  Q  *  E  L  Y  L  D  *  L  H  N  R  L  F  L  N  T  T  Q  A  D  L  F  I  F  L  A  K  *  L  Q  C  D  F  N  Q  T  V  W  T  V  Q  C  V  N   F6
     C  P  L  F  I  E  E  L  Q  Y  R  E  D  L  Q  Q  W  G  I  V  S  S  D  N  W  T  C  I  R  Y  T  I  A  Y  F  C  I  Q  Q  K  L  M  W  F  Y  S  F  P  R  K  Y  S  V  T  L  I  K  H  S  G  P  L  R  A  Y  M   F5
       A  L  Y  S  F  K  K  W  N  I  G  S  M  W  N  S  G  D  L  *  Q  V  T  M  G  P  V  F  G  I  P  S  Q  T  S  V  F  K  N  N  S  C  G  F  I  H  F  P  G  K  I  A  S  L  *  F  K  T  H  G  L  Y  G  P  M  C  K   F4

     N  Y  T  Y  R  K  L  C  L  E  I  L  M  E  S  *  H  S  F  K  A  H  F  *  Q  Y  C  F  L  F  Q  R  V  D  P  T  *  E  F  T  *  Y  L  L  *  T  V  R  H  L  E  S  R  N  A  *  W  E  I  I  D  L  A  Q  *  S   F1
      Q  I  I  H  I  E  S  Y  V  *  K  F  *  W  K  V  N  I  L  L  R  H  I  F  N  S  T  V  S  Y  F  R  G  W  I  P  H  R  N  S  R  N  T  C  Y  K  L  S  A  I  L  N  L  A  M  L  D  G  K  L  S  I  S  H  S  N  L   F2
       K  L  Y  I  *  K  V  M  F  R  N  F  D  G  K  L  T  F  F  *  G  T  F  L  T  V  L  F  L  I  S  E  G  G  S  H  I  G  I  H  V  I  P  A  I  N  C  P  P  S  *  I  S  Q  C  L  M  G  N  Y  R  S  R  T  V  I  S   F3
 401 CAAATTATACATATAGAAAGTTATGTTTAGAAATTTTGATGGAAAGTTAACATTCTTTTAAGGCACATTTTTAACAGTACTGTTTCTTATTTCAGAGGGTGGATCCCACATAGGAATTCACGTAATACCTGCTATAAACTGTCCGCCATCTTGAATCTCGCAATGCTTGATGGGAAATTATCGATCTCGCACAGTAATCT 600
     ----:----|----:----|----:----|----:----|----:----|----:----|----:----|----:----|----:----|----:----|----:----|----:----|----:----|----:----|----:----|----:----|----:----|----:----|----:----|----:----|
 401 GTTTAATATGTATATCTTTCAATACAAATCTTTAAAACTACCTTTCAATTGTAAGAAAATTCCGTGTAAAAATTGTCATGACAAAGAAATAAAGTCTCCCACCTAGGGTGTATCCTTAAGTGCATTATGGACGATATTTGACAGCGGTAGAACTTAGAGCGTTACGAACTACCCTTTAATAGCTAGAGCGTGTCATTAGA 600
      V  F  *  V  Y  L  F  N  H  K  S  I  K  I  S  L  *  C  E  K  L  A  C  K  *  C  Y  Q  K  K  N  *  L  T  S  G  V  Y  S  N  V  Y  Y  R  S  Y  V  T  R  W  R  S  D  R  L  A  Q  H  S  I  I  S  R  A  C  Y  D   F6
     L  N  Y  M  Y  F  T  I  N  L  F  K  S  P  F  N  V  N  K  *  P  V  N  K  V  T  S  N  R  I  E  S  P  P  D  W  M  P  I  *  T  I  G  A  I  F  Q  G  G  D  Q  I  E  C  H  K  I  P  F  *  R  D  R  V  T  I   F5
       C  I  I  C  I  S  L  *  T  *  F  N  Q  H  F  T  L  M  R  K  L  C  M  K  L  L  V  T  E  *  K  L  P  H  I  G  C  L  F  E  R  L  V  Q  *  L  S  D  A  M  K  F  R  A  I  S  S  P  F  N  D  I  E  C  L  L  R   F4

     R  E  I  S  V  A  R  P  K  Q  T  H  E  N  Y  S  W  *  T  W  Q  R  P  G  A  R  C  S  S  A  C  P  C  L  P  L  L  S  S  V  P  G  K  R  T  W  P  G  V  K  F  L  C  R  G  R  G  I  P  S  C  G  I  V  P  Q  G   F1
      A  R  F  L  S  R  D  Q  N  K  P  T  R  I  T  A  G  K  H  G  R  G  R  A  R  A  V  Q  V  L  A  P  A  Y  P  C  C  Q  V  C  P  E  K  E  R  G  R  G  S  S  S  C  A  G  G  G  A  F  P  P  V  G  S  C  P  R  A   F2
       R  D  F  C  R  E  T  K  T  N  P  R  E  L  Q  L  V  N  M  A  E  A  G  R  A  L  F  K  C  L  P  L  L  T  P  A  V  K  C  A  R  K  K  N  V  A  G  G  Q  V  P  V  P  G  E  G  H  S  L  L  W  D  R  A  P  G   F3
 601 CGCGAGATTTCTGTCGCGAGACCAAAACAAACCCACGAGAATTACAGCTGGTAAACATGGCAGAGGCCGGGCGCGCGCTGTTCAAGTGCTTGCCCCTGCTTACCCCTGCTGTCAAGTGTGCCCGGAAAAAGAACGTGGCCGGGGGTCAAGTTCCTGTGCCGGGGGAGGGGCATTCCCTCCTGTGGGATCGTGCCCCAGGG 800
     ----:----|----:----|----:----|----:----|----:----|----:----|----:----|----:----|----:----|----:----|----:----|----:----|----:----|----:----|----:----|----:----|----:----|----:----|----:----|----:----|
 601 GCGCTCTAAAGACAGCGCTCTGGTTTTGTTTGGGTGCTCTTAATGTCGACCATTTGTACCGTCTCCGGCCCGCGCGCGACAAGTTCACGAACGGGGACGAATGGGGACGACAGTTCACACGGGCCTTTTTCTTGCACCGGCCCCCAGTTCAAGGACACGGCCCCCTCCCCGTAAGGGAGGACACCCTAGCACGGGGTCCC 800
      R  S  I  E  T  A  L  G  F  C  V  W  S  F  *  L  Q  Y  V  H  C  L  G  P  A  R  Q  E  L  A  Q  G  Q  K  G  R  S  D  L  T  G  P  F  L  V  H  G  P  T  L  N  R  H  R  P  L  P  M  G  E  Q  P  I  T  G  W   F6
     E  R  S  K  Q  R  S  V  L  V  F  G  R  S  N  C  S  T  F  M  A  S  A  P  R  A  S  N  L  H  K  G  R  S  V  G  A  T  L  H  A  R  F  F  T  A  P  P  *  T  G  T  G  P  S  P  C  E  R  R  H  S  R  A  G  P   F5
       A  L  N  R  D  R  S  W  F  L  G  V  L  I  V  A  P  L  C  P  L  P  R  A  R  A  T  *  T  S  A  G  A  *  G  Q  Q  *  T  H  G  S  F  S  R  P  R  P  D  L  E  Q  A  P  P  P  A  N  G  G  T  P  D  H  G  L   F4

     L  T  P  M  T  T  P  A  A  T  Y  W  W  C  W  P  R  S  *  T  S  S  W  S  T  P  P  W  T  M  *  M  S  A  I  L  P  Y  V  Y  M  C  C  F  M  D  I  F  K  S  N  R  L  S  *  L  F  Q  F  D  S  L  C  S  I  G  Y   F1
      *  P  R  *  Q  H  Q  P  L  R  I  G  G  V  G  P  G  A  E  H  P  P  G  R  H  H  H  G  R  C  R  C  Q  Q  Y  C  R  M  Y  I  C  V  V  L  W  I  F  S  K  A  T  A  Y  L  S  S  F  S  L  I  L  Y  V  Q  *  A   F2
       L  D  P  D  D  N  T  S  R  Y  V  L  V  V  L  A  Q  E  L  N  I  L  L  V  D  T  T  M  D  D  V  D  V  S  N  I  A  V  C  I  Y  V  L  F  Y  G  Y  F  Q  K  Q  P  P  I  L  A  L  S  V  *  F  F  M  F  N  R  L   F3
 801 CTTGACCCCGATGACAACACCAGCCGCTACGTATTGGTGGTGTTGGCCCAGGAGCTGAACATCCTCCTGGTCGACACCACCATGGACGATGTAGATGTCAGCAATATTGCCGTATGTATATATGTGTTGTTTTATGGATATTTTCAAAAGCAACCGCCTATCTTAGCTCTTTCAGTTTGATTCTTTATGTTCAATAGGCT 1000
     ----:----|----:----|----:----|----:----|----:----|----:----|----:----|----:----|----:----|----:----|----:----|----:----|----:----|----:----|----:----|----:----|----:----|----:----|----:----|----:----|
 801 GAACTGGGGCTACTGTTGTGGTCGGCGATGCATAACCACCACAACCGGGTCCTCGACTTGTAGGAGGACCAGCTGTGGTGGTACCTGCTACATCTACAGTCGTTATAACGGCATACATATATACACAACAAAATACCTATAAAAGTTTTCGTTGGCGGATAGAATCGAGAAAGTCAAACTAAGAAATACAAGTTATCCGA 1000
      K  V  G  I  V  V  G  A  A  V  Y  Q  H  H  Q  G  L  L  Q  V  D  E  Q  D  V  G  G  H  V  I  Y  I  D  A  I  N  G  Y  T  Y  I  H  Q  K  I  S  I  K  L  L  L  R  R  D  *  S  K  *  N  S  E  K  H  E  I  P   F6
     S  S  G  S  S  L  V  L  R  *  T  N  T  T  N  A  W  S  S  F  M  R  R  T  S  V  V  M  S  S  T  S  T  L  L  I  A  T  H  I  Y  T  N  N  *  P  Y  K  *  F  C  G  G  I  K  A  R  E  T  Q  N  K  I  N  L  L  S   F5
       A  Q  G  R  H  C  C  W  G  S  R  I  P  P  T  P  G  P  A  S  C  G  G  P  R  C  W  W  P  R  H  L  H  *  C  Y  Q  R  I  Y  I  H  T  T  K  H  I  N  E  F  A  V  A  *  R  L  E  K  L  K  I  R  *  T  *  Y  A   F4

     E  R  N  M  H  C  T  R  N  E  *  K  D  *  T  N  V  D  H  V  N  K  *  H  V  M  T  C  D  T  C  S  Y  I  S  L  S  T  L  N  C  I  V  N  T  R  N  F  I  A  P  R  H  E  Q  K  K  V  I  C  N  C  H  I  I  I   F1
      M  K  G  I  C  I  V  Q  E  M  N  E  K  I  R  Q  M  L  I  M  *  T  N  D  M  S  *  H  V  T  L  V  V  I  F  L  Y  L  H  *  I  V  L  L  I  P  E  T  L  *  P  Q  G  T  N  K  R  K  *  Y  A  I  A  I  L  L  L   F2
       *  K  E  Y  A  L  Y  K  K  *  M  K  R  L  D  K  C  *  S  C  K  Q  M  T  C  H  D  M  *  H  L  *  L  Y  F  F  I  Y  I  E  L  Y  C  *  Y  Q  K  L  Y  S  P  K  A  R  T  K  E  S  N  M  Q  L  P  Y  Y  Y  *   F3
1001 ATGAAAGGAATATGCATTGTACAAGAAATGAATGAAAAGATTAGACAAATGTTGATCATGTAAACAAATGACATGTCATGACATGTGACACTTGTAGTTATATTTCTTTATCTACATTGAATTGTATTGTTAATACCAGAAACTTTATAGCCCCAAGGCACGAACAAAAGAAAGTAATATGCAATTGCCATATTATTATT 1200
     ----:----|----:----|----:----|----:----|----:----|----:----|----:----|----:----|----:----|----:----|----:----|----:----|----:----|----:----|----:----|----:----|----:----|----:----|----:----|----:----|
1001 TACTTTCCTTATACGTAACATGTTCTTTACTTACTTTTCTAATCTGTTTACAACTAGTACATTTGTTTACTGTACAGTACTGTACACTGTGAACATCAATATAAAGAAATAGATGTAACTTAACATAACAATTATGGTCTTTGAAATATCGGGGTTCCGTGCTTGTTTTCTTTCATTATACGTTAACGGTATAATAATAA 1200
      *  S  L  F  I  C  Q  V  L  F  S  H  F  S  *  V  F  T  S  *  T  F  L  H  C  T  M  V  H  S  V  Q  L  *  I  E  K  D  V  N  F  Q  I  T  L  V  L  F  K  I  A  G  L  C  S  C  F  F  T  I  H  L  Q  W  I  I  I   F6
     H  F  S  Y  A  N  Y  L  F  H  I  F  L  N  S  L  H  Q  D  H  L  C  I  V  H  *  S  M  H  C  K  Y  N  Y  K  K  I  *  M  S  N  Y  Q  *  Y  W  F  S  *  L  G  L  A  R  V  F  S  L  L  I  C  N  G  Y  *  *   F5
       I  F  P  I  H  M  T  C  S  I  F  S  F  I  L  C  I  N  I  M  Y  V  F  S  M  D  H  C  T  V  S  T  T  I  N  R  *  R  C  Q  I  T  N  N  I  G  S  V  K  Y  G  W  P  V  F  L  F  Y  Y  A  I  A  M  N  N  N   F4

     N  T  G  S  E  K  Y  F  F  I  V  T  V  H  F  I  D  *  S  L  L  K  N  C  I  F  K  C  Q  S  L  M  L  T  V  L  H  L  I  L  S  V  C  K  L  I  L  V  E  *  F  N  K  I  I  L  N  L  S  D  *  F  *  *  M  V  S   F1
      T  R  A  P  R  S  I  S  L  S  S  L  F  T  L  L  T  E  A  S  *  R  I  V  F  L  N  V  K  V  *  C  *  Q  F  Y  I  L  S  Y  L  Y  V  N  *  S  W  L  S  D  L  I  K  S  F  *  I  F  L  T  S  F  D  E  W  F  R   F2
       H  G  L  R  E  V  F  L  Y  R  H  C  S  L  Y  *  L  K  P  P  K  E  L  Y  F  *  M  S  K  F  N  V  N  S  F  T  S  Y  P  I  C  M  *  I  D  L  G  *  V  I  *  *  N  H  F  E  S  F  *  L  V  L  M  N  G  F   F3
1201 AACACGGGCTCCGAGAAGTATTTCTTTATCGTCACTGTTCACTTTATTGACTGAAGCCTCCTAAAGAATTGTATTTTTAAATGTCAAAGTTTAATGTTAACAGTTTTACATCTTATCCTATCTGTATGTAAATTGATCTTGGTTGAGTGATTTAATAAAATCATTTTGAATCTTTCTGACTAGTTTTGATGAATGGTTTC 1400
     ----:----|----:----|----:----|----:----|----:----|----:----|----:----|----:----|----:----|----:----|----:----|----:----|----:----|----:----|----:----|----:----|----:----|----:----|----:----|----:----|
1201 TTGTGCCCGAGGCTCTTCATAAAGAAATAGCAGTGACAAGTGAAATAACTGACTTCGGAGGATTTCTTAACATAAAAATTTACAGTTTCAAATTACAATTGTCAAAATGTAGAATAGGATAGACATACATTTAACTAGAACCAACTCACTAAATTATTTTAGTAAAACTTAGAAAGACTGATCAAAACTACTTACCAAAG 1400
      L  V  P  E  S  F  Y  K  K  I  T  V  T  *  K  I  S  Q  L  R  R  F  F  Q  I  K  L  H  *  L  K  I  N  V  T  K  C  R  I  R  D  T  H  L  N  I  K  T  S  H  N  L  L  I  M  K  F  R  E  S  *  N  Q  H  I  T  E   F6
     *  C  P  S  R  S  T  N  R  *  R  *  Q  E  S  *  Q  S  F  G  G  L  S  N  Y  K  *  I  D  F  N  L  T  L  L  K  L  V  D  *  G  I  Q  I  Y  I  S  R  P  Q  T  I  *  Y  F  *  K  S  D  K  Q  S  T  K  I  F  P  K   F5
       V  R  A  G  L  L  I  E  K  D  D  S  N  V  K  N  V  S  A  E  *  L  I  T  N  K  F  T  L  T  *  H  *  C  N  *  M  K  D  *  R  Y  T  F  Q  D  Q  N  L  S  K  I  F  D  N  Q  I  K  R  V  L  K  S  S  H  N   F4

     T  L  S  I  R  F  A  S  K  S  *  S  I  G  Q  *  I  S  *  G  E  H  K  R  P  S  T  A  T  G  R  H  R  C  V  G  A  S  R  A  S  S  I  N  T  A  R  S  G  I  Q  S  F  L  Q  E  R  H  L  L  G  R  P  G  Y  R  T   F1
      L  *  V  *  G  L  H  Q  N  P  K  V  *  V  N  E  S  L  K  V  N  T  R  G  L  R  Q  L  R  G  V  T  G  V  W  G  P  A  E  P  V  P  S  T  R  R  G  A  V  S  R  A  F  S  R  N  A  I  Y  W  G  G  L  D  T  E   F2
       D  F  K  Y  K  V  C  I  K  I  L  K  Y  R  S  M  N  L  L  R  *  T  Q  E  A  F  D  S  Y  G  A  S  P  V  C  G  G  Q  Q  S  Q  F  H  Q  H  G  E  E  R  Y  P  E  L  S  P  G  T  P  S  T  G  E  A  W  I  P  N   F3
1401 GACTTTAAGTATAAGGTTTGCATCAAAATCCTAAAGTATAGGTCAATGAATCTCTTAAGGTGAACACAAGAGGCCTTCGACAGCTACGGGGCGTCACCGGTGTGTGGGGGCCAGCAGAGCCAGTTCCATCAACACGGCGAGGAGCGGTATCCAGAGCTTTCTCCAGGAACGCCATCTACTGGGGAGGCCTGGATACCGAA 1600
     ----:----|----:----|----:----|----:----|----:----|----:----|----:----|----:----|----:----|----:----|----:----|----:----|----:----|----:----|----:----|----:----|----:----|----:----|----:----|----:----|
1401 CTGAAATTCATATTCCAAACGTAGTTTTAGGATTTCATATCCAGTTACTTAGAGAATTCCACTTGTGTTCTCCGGAAGCTGTCGATGCCCCGCAGTGGCCACACACCCCCGGTCGTCTCGGTCAAGGTAGTTGTGCCGCTCCTCGCCATAGGTCTCGAAAGAGGTCCTTGCGGTAGATGACCCCTCCGGACCTATGGCTT 1600
      V  K  L  I  L  N  A  D  F  D  *  L  I  P  *  H  I  E  *  P  S  C  L  L  G  E  V  A  V  P  R  *  R  H  T  P  A  L  L  A  L  E  M  L  V  A  L  L  P  I  W  L  K  R  W  S  R  W  R  S  P  L  G  P  Y  R   F6
     S  K  L  Y  L  T  Q  M  L  I  R  F  Y  L  D  I  F  R  K  L  H  V  C  S  A  K  S  L  *  P  A  D  G  T  H  P  P  W  C  L  W  N  W  *  C  P  S  S  R  Y  G  S  S  E  G  P  V  G  D  V  P  S  A  Q  I  G  F   F5
       R  S  *  T  Y  P  K  C  *  F  G  L  T  Y  T  L  S  D  R  L  T  F  V  L  P  R  R  C  S  R  P  T  V  P  T  H  P  G  A  S  G  T  G  D  V  R  R  P  A  T  D  L  A  K  E  L  F  A  M  *  Q  P  P  R  S  V  S   F4

     H  V  P  H  R  *  A  G  *  G  A  S  E  C  R  W  Q  V  N  Q  G  Q  R  P  R  R  V  L  P  S  V  P  N  T  Q  H  F  F  F  F  N  T  R  H  *  G  D  A  A  G  N  T  T  Y  P  S  H  W  T  H  P  C  L  C  L  W   F1
      H  M  C  L  T  D  E  L  V  K  V  H  L  S  V  G  G  K  S  T  K  A  N  V  H  V  V  F  S  P  Q  S  P  T  P  S  T  S  S  S  S  T  P  A  T  E  E  M  Q  Q  G  T  P  P  T  P  R  T  G  H  T  L  V  S  V  S  G   F2
       T  C  A  S  P  M  S  W  L  R  C  I  *  V  S  V  A  S  Q  P  R  P  T  S  T  S  C  S  P  L  S  P  Q  H  P  A  L  L  L  L  Q  H  P  P  L  R  R  C  S  R  E  H  H  L  P  L  A  L  D  T  P  L  S  L  S  L  G   F3
1601 CACATGTGCCTCACCGATGAGCTGGTTAAGGTGCATCTGAGTGTCGGTGGCAAGTCAACCAAGGCCAACGTCCACGTCGTGTTCTCCCCTCAGTCCCCAACACCCAGCACTTCTTCTTCTTCAACACCCGCCACTGAGGAGATGCAGCAGGGAACACCACCTACCCCTCGCACTGGACACACCCTTGTCTCTGTCTCTGG 1800
     ----:----|----:----|----:----|----:----|----:----|----:----|----:----|----:----|----:----|----:----|----:----|----:----|----:----|----:----|----:----|----:----|----:----|----:----|----:----|----:----|
1601 GTGTACACGGAGTGGCTACTCGACCAATTCCACGTAGACTCACAGCCACCGTTCAGTTGGTTCCGGTTGGCAGGTGCAGCACAAGAGGGGAGTCAGGGGTTGTGGGTCGTGAAGAAGAAGAAGTTGTGGGCGGTGACTCCTCTACGTCGTCCCTTGTGGTGGATGGGGAGCGTGACCTGTGTGGGAACAGAGACAGAGACC 1800
      V  C  T  G  *  R  H  A  P  *  P  A  D  S  H  R  H  C  T  L  W  P  W  R  G  R  R  T  R  G  E  T  G  L  V  W  C  K  K  K  K  L  V  R  W  Q  P  S  A  A  P  F  V  V  *  G  E  C  Q  V  C  G  Q  R  Q  R  Q   F6
     V  H  A  E  G  I  L  Q  N  L  H  M  Q  T  D  T  A  L  *  G  L  G  V  D  V  D  H  E  G  R  L  G  W  C  G  A  S  R  R  R  *  C  G  G  S  L  L  H  L  L  S  C  W  R  G  R  A  S  S  V  G  K  D  R  D  R   F5
       C  M  H  R  V  S  S  T  L  T  C  R  L  T  P  P  L  D  V  L  A  L  T  W  T  T  N  E  G  *  D  G  V  G  L  V  E  E  E  E  V  G  A  V  S  S  I  C  C  P  V  G  G  V  G  R  V  P  C  V  R  T  E  T  E  P   F4

     D  T  C  H  P  F  W  W  L  R  D  A  P  E  T  G  A  V  C  V  L  C  T  D  D  K  G  W  R  L  L  Q  P  P  P  S  I  Q  H  L  D  D  A  A  S  P  Q  S  D  P  P  C  I  S  L  G  C  F  C  G  E  L  I  *  A  C  G   F1
      T  L  A  I  L  F  G  G  L  E  M  P  Q  R  Q  G  R  F  V  S  F  A  Q  T  T  K  D  G  G  F  Y  S  L  H  L  P  S  N  T  W  T  T  L  P  L  P  K  V  T  P  R  A  Y  H  S  A  A  F  V  E  S  S  S  E  L  V  V   F2
       H  L  P  S  F  L  V  A  *  R  C  P  R  D  R  G  G  L  C  P  L  H  R  R  Q  R  M  E  A  F  T  A  S  T  F  H  P  T  P  G  R  R  C  L  S  P  K  *  P  P  V  H  I  T  R  L  L  L  W  R  A  H  L  S  L  W   F3
1801 GACACTTGCCATCCTTTTTGGTGGCTTAGAGATGCCCCAGAGACAGGGGCGGTTTGTGTCCTTTGCACAGACGACAAAGGATGGAGGCTTTTACAGCCTCCACCTTCCATCCAACACCTGGACGACGCTGCCTCTCCCCAAAGTGACCCCCCGTGCATATCACTCGGCTGCTTTTGTGGAGAGCTCATCTGAGCTTGTGG 2000
     ----:----|----:----|----:----|----:----|----:----|----:----|----:----|----:----|----:----|----:----|----:----|----:----|----:----|----:----|----:----|----:----|----:----|----:----|----:----|----:----|
1801 CTGTGAACGGTAGGAAAAACCACCGAATCTCTACGGGGTCTCTGTCCCCGCCAAACACAGGAAACGTGTCTGCTGTTTCCTACCTCCGAAAATGTCGGAGGTGGAAGGTAGGTTGTGGACCTGCTGCGACGGAGAGGGGTTTCACTGGGGGGCACGTATAGTGAGCCGACGAAAACACCTCTCGAGTAGACTCGAACACC 2000
      S  V  Q  W  G  K  Q  H  S  L  S  A  G  S  V  P  A  T  Q  T  R  Q  V  S  S  L  P  H  L  S  K  C  G  G  G  E  M  W  C  R  S  S  A  A  E  G  W  L  S  G  G  H  M  D  S  P  K  Q  P  S  S  M  Q  A  Q  P   F6
     P  C  K  G  D  K  K  T  A  *  L  H  G  L  S  L  P  P  K  H  G  K  C  L  R  C  L  I  S  A  K  V  A  E  V  K  W  G  V  G  P  R  R  Q  R  E  G  F  H  G  G  T  C  I  V  R  S  S  K  H  L  A  *  R  L  K  H   F5
       V  S  A  M  R  K  P  P  K  S  I  G  W  L  C  P  R  N  T  D  K  A  C  V  V  F  S  P  P  K  *  L  R  W  R  G  D  L  V  Q  V  V  S  G  R  G  L  T  V  G  R  A  Y  *  E  A  A  K  T  S  L  E  D  S  S  T   F4

     S  G  G  G  F  I  Q  W  D  R  T  L  *  K  D  I  Y  *  G  G  P  L  C  *  D  K  *  G  G  V  Y  N  E  R  G  *  H  T  G  F  T  G  D  R  E  L  P  V  Q  P  L  L  Q  H  L  G  Q  Q  S  A  A  I  W  G  V  W  T   F1
      V  G  G  V  S  Y  S  G  T  E  P  S  E  R  I  S  I  R  E  V  L  C  V  K  I  S  E  G  G  Y  T  M  R  E  V  N  I  Q  G  L  P  E  T  G  S  Y  L  S  S  L  S  C  S  I  L  D  S  K  V  L  L  F  G  G  Y  G   F2
       *  W  G  G  F  H  T  V  G  Q  N  P  L  K  G  Y  L  L  G  R  S  S  V  L  R  *  V  R  G  G  I  Q  *  E  R  L  T  Y  R  V  Y  R  R  P  G  A  T  C  P  A  S  P  A  A  S  W  T  A  K  C  C  Y  L  G  G  M  D   F3
2001 TAGTGGGGGGGGTTTCATACAGTGGGACAGAACCCTCTGAAAGGATATCTATTAGGGAGGTCCTCTGTGTTAAGATAAGTGAGGGGGGGTATACAATGAGAGAGGTTAACATACAGGGTTTACCGGAGACCGGGAGCTACCTGTCCAGCCTCTCCTGCAGCATCTTGGACAGCAAAGTGCTGCTATTTGGGGGGTATGGA 2200
     ----:----|----:----|----:----|----:----|----:----|----:----|----:----|----:----|----:----|----:----|----:----|----:----|----:----|----:----|----:----|----:----|----:----|----:----|----:----|----:----|
2001 ATCACCCCCCCCAAAGTATGTCACCCTGTCTTGGGAGACTTTCCTATAGATAATCCCTCAGGAGACACAATTCTATTCACTCCCCCCCATATGTTACTCTCTCCAATTGTATGTCCCAAATGGCCTCTGGCCCTCGATGGACAGGTCGGAGAGGACGTCGTAGAACCTGTCGTTTCACGACGATAAACCCCCCATACCT 2200
      L  P  P  P  K  M  C  H  S  L  V  R  Q  F  S  I  *  *  P  P  G  R  H  *  S  L  H  P  P  T  Y  L  S  L  P  *  C  V  P  N  V  P  S  R  S  S  G  T  W  G  R  R  C  R  P  C  C  L  A  A  I  Q  P  T  H   F6
     Y  H  P  P  N  *  V  T  P  C  F  G  R  F  P  Y  R  N  P  L  D  E  T  N  L  Y  T  L  P  P  I  C  H  S  L  N  V  Y  L  T  *  R  L  G  P  A  V  Q  G  A  E  G  A  A  D  Q  V  A  F  H  Q  *  K  P  P  I  S   F5
       T  T  P  P  T  E  Y  L  P  V  S  G  E  S  L  I  D  I  L  S  T  R  Q  T  L  I  L  S  P  P  Y  V  I  L  S  T  L  M  C  P  K  G  S  V  P  L  *  R  D  L  R  E  Q  L  M  K  S  L  L  T  S  S  N  P  P  Y  P   F4

     G  *  P  P  S  I  W  *  T  L  H  Q  *  S  S  V  L  H  *  S  G  G  R  Q  G  N  G  S  T  N  S  R  S  H  G  Y  S  R  T  *  L  C  S  A  V  *  *  P  C  T  F  C  R  W  H  C  K  S  F  V  L  L  H  H  Q  G   F1
      Q  D  S  P  P  V  S  G  K  P  C  I  S  S  R  L  F  C  I  D  L  E  A  G  K  G  T  V  L  Q  T  P  G  H  M  A  T  A  G  H  S  S  A  A  L  S  D  D  L  V  L  F  V  G  G  T  A  K  A  L  S  C  Y  T  T  K  A   F2
       R  I  A  P  Q  Y  L  V  N  P  A  S  V  V  C  F  A  L  I  W  R  Q  A  R  E  R  F  Y  K  L  Q  V  T  V  W  L  Q  Q  D  I  A  L  Q  R  C  L  M  T  L  Y  L  F  *  V  A  L  Q  K  L  C  P  V  T  P  P  R  H   F3
2201 CAGGATAGCCCCCCAGTATCTGGTAAACCCTGCATCAGTAGTCGTCTGTTTTGCATTGATCTGGAGGCAGGCAAGGGAACGGTTCTACAAACTCCAGGTCACATGGCTACAGCAGGACATAGCTCTGCAGCGCTGTCTGATGACCTTGTACTTTTTGTAGGTGGCACTGCAAAAGCTTTGTCCTGTTACACCACCAAGGC 2400
     ----:----|----:----|----:----|----:----|----:----|----:----|----:----|----:----|----:----|----:----|----:----|----:----|----:----|----:----|----:----|----:----|----:----|----:----|----:----|----:----|
2201 GTCCTATCGGGGGGTCATAGACCATTTGGGACGTAGTCATCAGCAGACAAAACGTAACTAGACCTCCGTCCGTTCCCTTGCCAAGATGTTTGAGGTCCAGTGTACCGATGTCGTCCTGTATCGAGACGTCGCGACAGACTACTGGAACATGAAAAACATCCACCGTGACGTTTTCGAAACAGGACAATGTGGTGGTTCCG 2400
      V  P  Y  G  G  L  I  Q  Y  V  R  C  *  Y  D  D  T  K  C  Q  D  P  P  L  C  P  F  P  E  V  F  E  L  D  C  P  *  L  L  V  Y  S  Q  L  A  T  Q  H  G  Q  V  K  Q  L  H  C  Q  L  L  K  T  R  N  C  W  W  P   F6
     L  I  A  G  W  Y  R  T  F  G  A  D  T  T  T  Q  K  A  N  I  Q  L  C  A  L  S  R  N  *  L  S  W  T  V  H  S  C  C  S  M  A  R  C  R  Q  R  I  V  K  Y  K  K  Y  T  A  S  C  F  S  Q  G  T  V  G  G  L   F5
       C  S  L  G  G  T  D  P  L  G  Q  M  L  L  R  R  N  Q  M  S  R  S  A  P  L  P  V  T  R  C  V  G  P  *  M  A  V  A  P  C  L  E  A  A  S  D  S  S  R  T  S  K  T  P  P  V  A  F  A  K  D  Q  *  V  V  L  A   F4
```

T C A Q S L * P G E V H H C * H * G T F S H C L G P V * C L Q * M G P P T L C W P C I S P E R K L V L L Q V K * K E K K V K Q H N V I F1
L V P S P C D L E K C I I A S T E E L S P I A W V Q C D A C S K W V H Q H C A G L V S A P K G S W Y C C R S N R K K R K * S S T M S S F2
L C P V L V T W R S A S L L A L R N F L P L P G S S V M L A V N G S T N I V L A L Y Q P R K E A G T A A G Q I E R E E S E A A Q C H F3
2401 ACTTGTGCCCAGTCCTTGTGACCTGGAGAAGTGCATCATTGCTAGCACTGAGGAACTTTCTCCCATTGCCTGGGTCCAGTGTGATGCTTGCAGTAAATGGGTCCACCAACATTGTGCTGGCCTTGTATCAGCCCCGAAAGGAAGCTGGTACTGCTGCAGGTCAAATAGAAAGAGAAGAAAGTGAAGCAGCACAATGTCAT 2600
2401 TGAACACGGGTCAGGAACACTGGACCTCTTCACGTAGTAACGATCGTGACTCCTTGAAAGAGGGTAACGGACCCAGGTCACACTACGAACGTCATTTACCCAGGTGGTTGTAACACGACCGGAACATAGTCGGGGCTTTCCTTCGACCATGACGACGTCCAGTTTATCTTTCTCTTCTTTCACTTCGTCGTGTTACAGTA 2600
V Q A W D K H G P S T C * Q * C Q P V K E W Q R P G T H H K C Y I P G G V N H Q G Q I L G S L F S T S S C T L Y F S F F T F C C L T M F6
C K H G T R T V Q L L A D N S A S L F K R G N G P D L T I S A T F P D V L M T S A K Y * G R F S A P V A A P * I S L S S L S A A C H * F5
S T G L G Q S R S F H M M A L V S S S E G M A Q T W H S A Q L L H T W W C Q A P R T D A G F P L Q Y Q Q L D F L F L L F H L L V I D F4

M S P F I F L F H V H V I Y C Y L * R * R Q Q T * Y T Y K M F I N N K H G I Y K M F I N N S S K T Y F P I S N S R P V N L T L V H Q E F1
C H L L S F Y S M F M L F I V I Y K G K D N K H D I H T R C L * T I N M A Y T R C L * T I V V K H I S L Y P I V D L * I * H * C I R F2
H V T F Y L F I P C S C Y L L L S I K V K T T N M I Y I Q D V Y K Q * T W H I Q D V Y K Q * * * N I F P Y I Q * * T C K F D T S A S G F3
2601 CATGTCACCTTTTATCTTTTTATTCCATGTTCATGTTATTTATTGTTATCTATAAAGGTAAAGACAACAAACATGATATACATACAAGATGTTTATAAACAATAAACATGGCATATACAAGATGTTTATAAACAATAGTAGTAAAACATATTTCCCTATATCCAATAGTAGACCTGTAAATTTGACACTAGTGCATCAGG 2800
2601 GTACAGTGGAAAATAGAAAAATAAGGTACAAGTACAATAAATAACAATAGATATTTCCATTTCTGTTGTTTGTACTATATGTATGTTCTACAAATATTTGTTATTTGTACCGTATATGTTCTACAAATATTTGTTATCATCATTTTGTATAAAGGGATATAGGTTATCATCTGGACATTTAAACTGTGATCACGTAGTCC 2800
M D G K I K K N W T * T I * Q * R Y L Y L C C V H Y V Y L I N I F L L C P M Y L I N I F L L L L V Y K G I D L L L G T F K V S T C * F6
* T V K * R K I G H E H * K N N D I F T F V V F M I Y M C S T * L C Y V H C I C S T * L C Y Y F M N G * I W Y Y V Q L N S V L A D P F5
D H * R K D K * E M N M N N I T I * L P L S L L C S I C V L H K Y V I F M A Y V L H K Y V I T T F C I E R Y G I T S R Y I Q C * H M L F4

* R K R L I S G * P I K R * T F C T R P * R A S G T S D L K N * W D L * V M L H N * K N K D D I Y K M L I * Q * K * Y T Q D A Y Q E F1
N K E R G * Y Q V S Q L K G R R S V P G H D E L L V P L T L K I S G T C K * C Y I I K R I K M I Y T R C * F N N K S D I H K M L I K N F2
I K K E A D I R L A N * K V D V L Y Q A M T S F W Y L * P * K L V G P V S D A T * L K E * R * Y I Q D V N L T I K V I Y T R C L S R I F3
2801 AATAAAGAAAGAGGCTGATATCAGGTTAGCCAATTAAAAGGTAGACGTTCTGTACCAGGCCATGACGAGCTTCTGGTACCTCTGACCTTAAAAATTAGTGGGACCTGTAAGTGATGCTACATAATTAAAAGAATAAAGATGATATATACAAGATGTTTAATTTAACAATAAAAGTGATATACACAAGATGCTTATCAAGAA 3000
2801 TTATTTCTTTCTCCGACTATAGTCCAATCGGTTAATTTTCCATCTGCAAGACATGGTCCGGTACTGCTCGAAGACCATGGAGACTGGAATTTTTAATCACCCTGGACATTCACTACGATGTATTAATTTTCTTATTTCTACTATATATGTTCTACAATTAAATTGTTATTTTCACTATATGTGTTCTACGAATAGTTCTT 3000
S Y L F L S I D P * G I L L Y V N Q V L G H R A E P V E S R L F * H S R Y T I S C L * F F L S S I Y L I N I * C Y F H Y V C S A * * S F6
I F F S A S I L N A L * F T S T R Y W A M V L K Q Y R Q G * F N T P G T L S A V Y N F S Y L H Y I C S T L K V I F T I Y V L H K D L F5
F L S L P Q Y * T L W N F P L R E T G P W S S S R T G R V K F I L P V Q L H H * M I L L I F I I Y V L H * N L L L L S I C L I S I L F F4

* R * Y A Q D V Y V Q S I S V G K N K V M L R H I H L F W V H L Y K A P Q T Y I Q T Y I A A F T Y I E D T T S I R S N K N T K H I I S F1
K D D M H K M F M Y N L S V W A K I K L C * D I F T C F G Y I C T K H L K H T Y K H T * L H L P L I K L L V L G V I K I Q N I L F P F2
K M I C T R C L C T I Y Q C G Q K * S Y V E T Y S L V L G T S V Q S T S N I H T N I H S C I Y L Y * R Y * Y * E * * K Y K T Y Y F F3
3001 TAAAGATGATATGCACAAGATGTTTATGTACAATCTATCAGTGTGGGCAAAAATAAAGTTATGTTGAGACATATTCACTTGTTTTGGGTACATCTGTACAAAGCACCTCAAACATACATACAAACATACATAGCTGCATTTACCTATATTGAAGATACTACTAGTATTAGGAGTAATAAAAATACAAAACATATTATTTC 3200
3001 ATTTCTACTATACGTGTTCTACAAATACATGTTAGATAGTCACACCCGTTTTTATTTCAATACAACTCTGTATAAGTGAACAAAACCCATGTAGACATGTTTCGTGGAGTTTGTATGTATGTTTGTATGTATCGACGTAAATGGATATAACTTCTATGATGATCATAATCCTCATTATTTTTATGTTTTGTATAATAAAG 3200
Y L H Y A C S T * T C D I L T P L F L T I N L C I * K N Q T C R Y L A G * V Y M C V Y M A A N V * I S S V V L I L L L L F V F C I I E F6
I F I I H V L H K H V I * * H P C F Y L * T S V Y E S T K P V D T C L V E F M C V F M C L Q M * R Y Q L Y * * Y * S Y Y F Y L V Y * K F5
L S S I C L I N I Y L R D T H A F I F N H Q S M N V Q K P Y M Q V F C R L C V Y L C V Y S C K G I N F I S S T N P T I F I C F M N N F4

L Y P I V D L * I * H * C I R N E E R G * Y Q V S Q L K V R R S V P G H D Q L L A P L T L V G P V S D A T * L K E N N P A C F C H S N F1
Y I Q * * T C K F D T S A S G M K K E A D I R L A N * K L D V L Y Q A M T S F W H L * P * W D L * V M L H N * K K T T Q P V S V I Q F2
P I S N S R P V N L T L V H Q E * R K R L I S G * P I K S * T F C T R P * P A S G T S D L S G T C K * C Y I I K R K Q P S L F L S F K F3
3201 CCTATATCCAATAGTAGACCTGTAAATTTGACACTAGTGCATCAGGAATGAAGAAAGAGGCTGATATCAGGTTAGCCAATTAAAAGTTAGACGTTCTGTACCAGGCCATGACCAGCTTCTGGCACCTCTGACCTTAGTGGGACCTGTAAGTGATGCTACATAATTAAAAGAAAACAACCCAGCCTGTTTCTGTCATTCAA 3400
3201 GGATATAGGTTATCATCTGGACATTTAAACTGTGATCACGTAGTCCTTACTTCTTTCTCCGACTATAGTCCAATCGGTTAATTTTCAATCTGCAAGACATGGTCCGGTACTGGTCGAAGACCGTGGAGACTGGAATCACCCTGGACATTCACTACGATGTATTAATTTTCTTTTGTTGGGTCGGACAAAGACAGTAAGTT 3400
R Y G I T S R Y I Q C * H M L F S S L P Q Y * T L W N F T L R E T G P W S W S R A G R V K T P G T L S A V Y N F S F L G A Q K Q * E F6
G I D L L G T F K V S T C * S H L F L S I D P * G I L L * V N Q V L G H G A E P V E S R L P V Q L H H * M I L F C G L R N R D N L F5
G * I W Y Y V Q L N S V L A D P I F F S A S I L N A L * F N S T R Y W A M V L K Q C R Q G * H S R Y T I S C L * F F V V W G T E T M * F4

C W H N L A P L R T G C L R Q V L V N K M H Q G * Y * E L L A E * N S C L V P S T N R M S Q I F C S L V F A G S P V V A K S S A I T F1
T A G T T W H H L G Q V V * D R S * * I K C I K D N T E S C W L N R T H V L F P V Q T E C L K F F V A * F L Q V R L L W P N P P Q L P F2
L L A Q P G T T * D R L S E T G P S E * N A S R I I L R A V G * I E L M S C S Q Y K Q N V S N F L * L S F C R F A C C G Q I L R N Y H F3
3401 ACTGCTGGCACAACTTGGCACCACTTAGGACAGGTTGTCTGAGACAGGTCCTAGTGAATAAAATGCATCAAGGATAATACTGAGAGCTGTTGGCTGAATAGAACTCATGTCTTGTTCCCAGTACAAACAGAATGTCTCAAATTTTTTGTAGCTTAGTTTTTGCAGGTTCGCCTGTTGTGGCCAAATCCTCCGCAATTACC 3600
3401 TGACGACCGTGTTGAACCGTGGTGAATCCTGTCCAACAGACTCTGTCCAGGATCACTTATTTTACGTAGTTCCTATTATGACTCTCGACAACCGACTTATCTTGAGTACAGAACAAGGGTCATGTTTGTCTTACAGAGTTTAAAAAACATCGAATCAAAAACGTCCAAGCGGACAACACCGGTTTAGGAGGCGTTAATGG 3600
F Q Q C L R A G S L V P Q R L C T R T F L I C * P Y Y Q S S N A S Y F E H R T G L V F L I D * I K Q L K T K A P E G T T A L D E A I V F6
S S A C G P V V * S L N D S V P G L S Y F A D L I I S L A T P Q I S S M D Q E W Y L C F T E F K K Y S L K Q L N A Q Q P W I R R L * F5
V A P V V Q C W K P C T T Q S L D * H I F H M L S L V S L Q Q S F L V * T K N G T C V S H R L N K T A * N K C T R R N H G F G G C N G F4

T F V H I V S D R C L A L Q F F G T V Q P V N P Q D I P Q P I Q S V S F P R V T * S * L P K Q L V A C L I P L T * Q S S Y A T C T * R F1
H S Y T L * V T D A L L C S F L E L Y S Q * I H R T S L S P F K V F P F L E * P N L N C L N S L L P A S F P S L D K A P M L P A P E G F2
I R T H C E * Q M P C F A V F W N C T A S E S T G H P S A H S K C F L S * S D L I L T A * T A C C L P H S P H L T K L L C Y L H L K F3
3601 ACATTCGTACACATTGTGAGTGACAGATGCCTTGCTTTGCAGTTTTTTGGAACTGTACAGCCAGTGAATCCACAGGACATCCCTCAGCCCATTCAAAGTGTTTCCTTTCCTAGAGTGACCTAATCTTAACTGCCTAAACAGCTTGTTGCCTGCCTCATTCCCCTCACTTGACAAAGCTCCTATGCTACCTGCACCTGAAG 3800
3601 TGTAAGCATGTGTAACACTCACTGTCTACGGAACGAAACGTCAAAAAACCTTGACATGTCGGTCACTTAGGTGTCCTGTAGGGAGTCGGGTAAGTTTCACAAAGGAAAGGATCTCACTGGATTAGAATTGACGGATTTGTCGAACAACGGACGGAGTAAGGGGAGTGAACTGTTTCGAGGATACGATGGACGTGGACTTC 3800
V N T C M T L S L H R A K C N K P V T C G T F G C S M G * G M * L T E K G L T V * D * S G L C S T A Q R M G R V Q C L E * A V Q V Q L F6
W M R V C Q S H C I G Q K A T K Q F Q V A L S D V P C G E A W E F H K R E * L S R I K V A * V A Q Q R G * E G * K V F S R H * R C R F F5
C E Y V N H T V S A K S Q L K K S S Y L W H I W L V D R L G N L T N G K R S H G L R L Q R F L K N G A E N G E S S L A G I S G A G S F4

I L Y E L L Y M F S D L M E I I C A R S I W E I V S D H C D H F H C Y C F V L M N E F R E L F Q W P C A T V H L S Q G P E L A Q S F L F1
S S M S S C T C S V I L W R * F V Q E A Y G K L S L I I V T I S T A T V L Y * * T S S G S S F N G H V Q R Y T F L K V L N L L K A S F2
D P L * A L V H V Q * S Y G D N L C K K H M G N C L * S L * P F P L L F C T D E R V Q G G A L S M A M C N G T P F S R S * T C S K L P F3
3801 GATCCTCTATGAGCTCTTGTACATGTTCAGTGATCTTATGGAGATAATTTGTGCAAGAAGCATATGGGAAATTGTCTCTGATCATTGTGACCATTTCCACTGCTACTGTTTTGTACTGATGAACGAGTTCAGGGAGCTCTTTCAATGGCCATGTGCAACGGTACACCTTTCTCAAGGTCCTGAACTTGCTCAAAGCTTCC 4000
3801 CTAGGAGATACTCGAGAACATGTACAAGTCACTAGAATACCTCTATTAAACACGTTCTTCGTATACCCTTTAACAGAGACTAGTAACACTGGTAAAGGTGACGATGACAAAACATGACTACTTGCTCAAGTCCCTCGAGAAAGTTACCGGTACACGTTGCCATGTGGAAAGAGTTCCAGGACTTGAACGAGTTTCGAAGG 4000
I R * S S K Y M N L S R I S I I Q A L L M H S I T E S * Q S W K W Q * Q K T S I F S N L S S K * H G H A V T C R E * P G S S A * L K F6
S G R H A R T C T * H D * P S L K H L F C I P F Q R Q D N H G N G S S S N Q V S S R T * P A R E I A M H L P V G K E L D Q V Q E F S G F5
P D E I L E Q V H E T I K H L Y N T C S A Y P F N D R I M T V M E V A V T K Y Q H V L E P L E K L P W T C R Y V K R L T R F K S L A E F4

E Y C L F V C T G Y K T Y N M S S I I L I K * F S G I I A R H H Q C G I N T Y C S F Q V S V K L C F C L * D R I T N I C S F P P L R F1
L S T A C L S A L G T R L T T * A A L F S S N N S R A * L P G I I N A G L I P I V A F R C R S N C A S A C E I E S L T S A V F P H * E F2
* V L L V C L H W V Q D L Q H E Q H Y S H Q I I L G H N C Q A S S M R D * Y L L * L S G V G Q T V L L L V R * N H * H L Q F S P I E K F3
4001 TTGAGTACTGCTTGTTTGTCTGCACTGGGTACAAGACTTACAACATGAGCAGCATTATTCTCATCAAATAATTCTCGGGCATAATTGCCAGGCATCATCAATGCGGGATTAATACCTATTGTAGCTTTCAGGTGTCGGTCAAACTGTGCTTCTGCTTGTGAGATAGAATCACTAACATCTGCAGTTTTCCCCCATTGAGA 4200
4001 AACTCATGACGAACAAACAGACGTGACCCATGTTCTGAATGTTGTACTCGTCGTAATAAGAGTAGTTTATTAAGAGCCCGTATTAACGGTCCGTAGTAGTTACGCCCTAATTATGGATAACATCGAAAGTCCACAGCCAGTTTGACACGAAGACGAACACTCTATCTTAGTGATTGTAGACGTCAAAAGGGGGTAACTCT 4200
R S Y Q K N T Q V P Y L V * L M L L M I R M L Y N E P M I A L C * * H P I L V * Q L K * T D T L S H K Q K H S L I V L M Q L K G G N L F6
Q T S S T Q R C Q T C S K C C S C C * E * * I R P C L Q W A D D I R S * Y R N Y S E P T P * V T S R S T L Y F * * C R C N E G M S F5
K L V A Q K D A S P V L S V V H A A N N E D F L E R A Y N G P M M L A P N I G I T A K L H R D F Q A E A Q S I S D S V D A T K G W Q S F4

N P S N F S Y N Q R F E V A T H I Y I * V R C I Y G P F F R L T S Q H T L S L N T T A N I F K C D L G E I V R V * P S N V C S F F T L F1
T P A I S L T I N V L K * L P I F I S E C V A S M A L S L G S Q V S I L * A L T P Q Q T S L S A T S E R L S G F S L A M F A L S L H C F2
P Q Q F L L Q S T F * S S Y P Y L Y L S A L H L W P F L * A H K S A Y S E P * H H S K H L * V R P R R D C Q G L A * Q C L L F L Y I F3
4201 AACCCCAGCAATTTCTCTTACAATCAACGTTTTGAAGTAGCTACCCATATTTATATCTGAGTGCGTTGCATCTATGGCCCTTTCTTTAGGCTCACAAGTCAGCATACTCTGAGCCTTAACACCACAGCAAACATCTTTAAGTGCGACCTCGGAGAGATTGTCAGGGTTTAGCCTAGCAATGTTTGCTCTTTCTTTACATT 4400
4201 TTGGGGTCGTTAAAGAGAATGTTAGTTGCAAAACTTCATCGATGGGTATAAATATAGACTCACGCAACGTAGATACCGGGAAAGAAATCCGAGTGTTCAGTCGTATGAGACTCGGAATTGTGGTGTCGTTTGTAGAAATTCACGCTGGAGCCTCTCTAACAGTCCCAAATCGGATCGTTACAAACGAGAAAGAAATGTAA 4400
F G L L K E * L * R K S T A V W I * I Q T R Q M * R H G K K L S V L * C V R L R L V V A F M K L H S R P S I T L T * G L L T Q E K K V N F6
F G W C N R K C D V N Q L L * G Y K Y R L A N C R H G K R * A * L D A Y E S G * C W L L C R * T R G R L S Q * P K A * C H K S K R * M F5
V G A I E R V I L T K F Y S G M N I D S H T A D I A R E K P E C T L M S Q A K V G C C V D K L A V E S L N D P N L R A I N A R E K C F4

L K S P C D R V A S C L F K C F S S R C I T K C T E K F * T G T L Q A T P T S V P L F I Y H T I Y K G * V V * P L Y I I T H N N S H D F1
L R V L V T V * L P A F S N A S V L D A S Q S V Q R N F E P A P C K P P Q L R S H F S S I I L F T K V K * C D R F T L S P T I T L M F2
A * E S L * P C S F L P F Q M L Q F * M H H K V Y R E I L N R H P A S H P N F G P F T F H L S Y Y L Q R L S S V T A L H Y H P Q * L S * F3
4401 GCTTAAGAGTCCTTGTGACCGTGTAGCTTCCTGCCTTTTCAAATGCTTCAGTTCTAGATGCATCACAAAGTGTACAGAGAAATTTTGAACCGGCACCCTGCAAGCCACCCCAACTTCGGTCCCACTTTTCATCTATCATACTATTTACAAAGGTTAAGTAGTGTGACCGCTTTACATTATCACCCACAATAACTCTCATG 4600
4401 CGAATTCTCAGGAACACTGGCACATCGAAGGACGGAAAAGTTTACGAAGTCAAGATCTACGTAGTGTTTCACATGTCTCTTTAAAACTTGGCCGTGGGACGTTCGGTGGGGTTGAAGCCAGGGTGAAAAGTAGATAGTATGATAAATGTTTCCAATTCATCACACTGGCGAAATGTAATAGTGGGTGTTATTGAGAGTAC 4600
S L L G Q S R T A E Q R K L H K L E L H M V F H V S F N Q V P V R C A V G V E T G S K M * V I * L P * T T H G S * M I V W L L E * F6
A * S D K H G H L K R G K * I S * N * I C * L T Y L S I K F R C G A L W G L K P G V K * R D Y * K C L N L L T V A K C * * G C Y S E H F5
Q K L T R T V T Y S G A K E F A E T R S A D C L T C L F K S G A G Q L G G W S R D W K E D I M S N V F T L Y H S R K V N D G V I V R M F4

L V F H Y * S F L L N G * N T H T N S G F V I L I P Y R F K Q R S I A S Y R I R V I F S K N W N F F S I F L N G T F K N T K * K S * F1
I L S F I T S R S C S M G R I H T L T V D L S F S S P I A S S K G L L L L I E L G L S S V K T G T S S P S F S T E H L R T Q N E N L K F2
S C L S L L V V L A Q W V E Y T H * Q W I C H S H P L S L Q A K V Y C F L * N * G Y L Q * K L E L L L H L S Q R N I * E H K M K I L K F3
4601 ATCTTGTCTTTCATTACTAGTCGTTCTTGCTCAATGGGTAGAATACACACACTAACAGTGGATTTGTCATTCTCATCCCCTATCGCTTCAAGCAAAGGTCTATTGCTTCTTATAGAATTAGGGTTATCTTCAGTAAAAACTGGAACTCTTCTCCATCTTTCTCAACGGAACATTTAAGAACACAAATGAAAATCTTAA 4800
4601 TAGAACAGAAAGTAATGATCAGCAAGAACGAGTTACCCATCTTATGTGTGTGATTGTCACCTAAACAGTAAGAGTAGGGGATAGCGAAGTTCGTTTCCAGATAACGAAGAATATCTTAATCCCAATAGAAGTCATTTTTGACCTTGAAGAAGAGGTAGAAAGAGTTGCCTTGTAAATTCTTGTGTTTACTTTTAGAATT 4800
S R T K * * * D N K S L P Y F V C V L L P N T M R M G * R K L C L D I A E * L I L T I K L L F Q F K K E M K R L P V N L F V F H F D * F6
D Q R E N S T T R A * H T S Y V C * C H I Q * E * G R D S * A F T * Q K K Y F * P * R * Y F S S S R R W R E * R F M * S C L I F I K F5
I K D K M V L R E Q E I P L I C V S V T S K D N E D G I A E L L P R N S R I S N P N D E T F V P V E E G D K E V S C K L V C F S F R L F4

S F I R Q K T I T S L F V D G N I T H S I C A I F N N S M N H * R W I I W V Y A Q R L Q T R H Y H I I K F L Q C F C N S I T V G E C H F1
A L S G R K R S L A S L W T E T S P I P S A P S L I I V * I T K D G S F G S M P N D S R P D T I T S S S S N V F A T A S Q * G N V T F2
L Y P A E N D H * P L C G R K H H P F H L R H L * * * Y E S L K M D H L G L C P T T P D Q T L S H H Q V P P M F L Q Q H H S R G M S F3
4801 AGCTTTATCCGGCAGAAAACGATCACTAGCCTCTTTGTGGACGGAAACATCACCCATTCCATCTGCGCCATCTTTAATAATAGTATGAATCACTAAAGATGGATCATTTGGGTCTATGCCCAACGACTCCAGACCAGACACTATCACATCATCAAGTTCCTCCAATGTTTTTGCAACAGCATCACAGTAGGGGAATGTCA 5000
4801 TCGAAATAGGCCGTCTTTTGCTAGTGATCGGAGAAACACCTGCCTTTGTAGTGGGTAAGGTAGACGCGGTAGAAATTATTATCATACTTAGTGATTTCTACCTAGTAAACCCAGATACGGGTTGCTGAGGTCTGGTCTGTGATAGTGTAGTAGTTCAAGGAGGTTACAAAAACGTTGTCGTAGTGTCATCCCCTTACAGT 5000
L K I R C F V I V L R K T S P F M V W E M Q A M K L L L I F * L H I M Q T * A W R S W V L C * * M M L N R W H K Q L L M V T P S H * F6
F S * G A S F S * * G Q R P R F C * G N W R R W R * Y Y Y S D S F I S * K P R H G V V G S W V S D C * T G G I N K C C C * L L P I D F5
A K D P L F R D S A E K H V S V D G M G D A G D K I I T H I V L S P D N P D I G L S E L G S V I V D D L E E L T K A V A D C Y P F T F4

```
      A L C I W * R K V W N R D I E I L Y N T N R F K * I Q L T M D G T S S S T C F H W S V V I V I * V E V T L N * I S S C P W H V Q D F *  F1
       P F V F G D G K S G T E T L K S S T I L I G S S E S S L P W M V L V A A L V F T G V W * * * S E W R S P * T E Y L A V P G M Y R T S   F2
     R P L Y L V T E S L E Q R H * N P L Q Y * * V Q V N P A Y H G W Y * * Q H L F S L E C G N S D L S G G H L E L N I * L S L A C T G L L   F3
5001 CGCCCTTTGTATTTGGTGACGGAAAGTCTGGAACAGAGACATTGAAATCCTCTACAATACTAATAGGTTCAAGTGAATCCAGCTTACCATGGATGGTACTAGTAGCAGCACTTGTTTTCACTGGAGTGTGGTAATAGTGATCTGAGTGGAGGTCACCTTGAACTGAATATCTAGCTGTCCCTGGCATGTACAGGACTTCT 5200
     ----:----|----:----|----:----|----:----|----:----|----:----|----:----|----:----|----:----|----:----|----:----|----:----|----:----|----:----|----:----|----:----|----:----|----:----|----:----|----:----|
5001 GCGGGAAACATAAACCACTGCCTTTCAGACCTTGTCTCTGTAACTTTAGGAGATGTTATGATTATCCAAGTTCACTTAGGTCGAATGGTACCTACCATGATCATCGTCGTGAACAAAAGTGACCTCACACCATTATCACTAGACTCACCTCCAGTGGAACTTGACTTATAGATCGACAGGGACCGTACATGTCCTGAAGA 5200
       A R Q I Q H R F T Q F L S M S I R * L V L L N L H I W S V M S P V L L L V Q K * Q L T T I T I Q T S T V K F Q I D L Q G Q C T C S K   F6
      R G K Y K T V S L R S C L C Q F G R C Y * Y T * T F G A * W P H Y * Y C C K N E S S H P L L S R L P P * R S S F I * S D R A H V P S R  F5
     V G K T N P S P F D P V S V N F D E V I S I P E L S D L K G H I T S T A A S T K V P T H Y Y H D S H L D G Q V S Y R A T G P M Y L V E   F4

      G I Q F R G W R Q H M * I L A F H Q S I F F G I G I F C H * Q I Y P H C K T L F W C Q S G W F C T K P Q T L N C L S S V T I I T V I   F1
     K A F S S E G G D N T C K S L L F I K A Y S L A * E F F V I S K S T R I A R H S S G V N L V G F A P N H R L S T A S A L * R S L L S Y   F2
      R H S V Q R V E T T H V N P C F S S K H I L W H R N F L S L A N L P A L Q D T L L V S I W L V L H Q T T D S Q L P Q L C D D H Y C H T  F3
5201 AAGGCATTCAGTTCAGAGGGTGGAGACAACACATGTAAATCCTTGCTTTTCATCAAAGCATATTCTTTGGCATAGGAATTTTTTGTCATTAGCAAATCTACCCGCATTGCAAGACACTCTTCTGGTGTCAATCTGGTTGGTTTTGCACCAAACCACAGACTCTCAACTGCCTCAGCTCTGTGACGATCATTACTGTCATA 5400
     ----:----|----:----|----:----|----:----|----:----|----:----|----:----|----:----|----:----|----:----|----:----|----:----|----:----|----:----|----:----|----:----|----:----|----:----|----:----|----:----|
5201 TTCCGTAAGTCAAGTCTCCCACCTCTGTTGTGTACATTTAGGAACGAAAAGTAGTTTCGTATAAGAAACCGTATCCTTAAAAAACAGTAATCGTTTAGATGGGCGTAACGTTCTGTGAGAAGACCACAGTTAGACCAACCAAAACGTGGTTTGGTGTCTGAGAGTTGACGGAGTCGAGACACTGCTAGTAATGACAGTAT 5400
     * P M * N L P H L C C M Y I R A K * * L M N K P M P I K Q * * C I * G C Q L V S K Q H * D P Q N Q V L G C V R L Q R L E T V I M V T M  F6
      L C E T * L T S V V C T F G Q K E D F C I R Q C L F K K D N A F R G A N C S V R R T D I Q N T K C W V V S E * S G * S Q S S * * Q *   F5
      L A N L E S P P S L V H L D K S K M L A Y E K A Y S N K T M L L D V R M A L C E E P T L R T P K A G F W L S E V A E A R H R D N S D Y  F4

     Q V T S E C E E * H I F F R F I L F R A E A V D V V Q Q G F * P F V L Y I L P A N I V E C S F P Q T L P L Q I S R C C S L L P F I L T   F1
      R * L L S A K N S T S S F D S Y C S V Q K L L M L S N K G F N L L F F T Y C L P T S * S A A F L R L F H F R F P G V V V F F L S Y S H  F2
       G D F * V R R I A H L L S I H T V P C R S C * C C P T R V L T F C S L H T A C Q H R R V Q L S S D S S T S D F P V L * S S S F H T H   F3
5401 CAGGTGACTTCTGAGTGCGAAGAATAGCACATCTTCTTTCGATTCATACTGTTCCGTGCAGAAGCTGTTGATGTTGTCCAACAAGGGTTTTAACCTTTTGTTCTTTACATACTGCCTGCCAACATCGTAGAGTGCAGCTTTCCTCAGACTCTTCCACTTCAGATTTCCCGGTGTTGTAGTCTTCTTCCTTTCATACTCAC 5600
     ----:----|----:----|----:----|----:----|----:----|----:----|----:----|----:----|----:----|----:----|----:----|----:----|----:----|----:----|----:----|----:----|----:----|----:----|----:----|----:----|
5401 GTCCACTGAAGACTCACGCTTCTTATCGTGTAGAAGAAAGCTAAGTATGACAAGGCACGTCTTCGACAACTACAACAGGTTGTTCCCAAAATTGGAAAACAAGAAATGTATGACGGACGGTTGTAGCATCTCACGTCGAAAGGAGTCTGAGAAGGTGAAGTCTAAAGGGCCACAACATCAGAAGAAGGAAAGTATGAGTG 5600
      C T V E S H S S Y C M K K R N M S N R A S A T S T T W C P N * G K T R * M S G A L M T S H L K G * V R G S * I E R H Q L R R G K M S V  F6
     V P S K Q T R L I A C R R E I * V T G H L L Q Q H Q G V L T K V K Q E K C V A Q W C R L T C S E E S E E V E S K G T N Y D E E K * V *   F5
      L H S R L A F F L V D E K S E Y Q E T C F S N I N D L L P K L R K N K V Y Q R G V D Y L A A K R L S K W K L N G P T T T K K R E Y E   F4

      S T L M I L D V L H S I F H V A S L V R V Q T F D F Q C V K N I * Q V T * Q R I * V L C C H W R P A C W A T A R L F Q P I R D T L G A  F1
       L L S * S L M F F T V S S M L H P L F E Y R H L T F N V S K I F S K * P D K G F R S S V V I G D R H V G Q L P A F S N Q Y E I H L V   F2
     I Y S H D P * C S S Q Y L P C C I P C S S T D I * L S M C Q K Y L A S D L T K D L G P L L S L E T G M L G N C P P F P T N T R Y T W C   F3
5601 ATCTACTCTCATGATCCTTGATGTTCTTCACAGTATCTTCCATGTTGCATCCCTTGTTCGAGTACAGACATTTGACTTTCAATGTGTCAAAAATATTTAGCAAGTGACCTGACAAAGGATTTAGGTCCTCTGTTGTCATTGGAGACCGGCATGTTGGGCAACTGCCCGCCTTTTCCAACCAATACGAGATACACTTGGTG 5800
     ----:----|----:----|----:----|----:----|----:----|----:----|----:----|----:----|----:----|----:----|----:----|----:----|----:----|----:----|----:----|----:----|----:----|----:----|----:----|----:----|
5601 TAGATGAGAGTACTAGGAACTACAAGAAGTGTCATAGAAGGTACAACGTAGGGAACAAGCTCATGTCTGTAAACTGAAAGTTACACAGTTTTTATAAATCGTTCACTGGACTGTTTCCTAAATCCAGGAGACAACAGTAACCTCTGGCCGTACAACCCGTTGACGGGCGGAAAAGGTTGGTTATGCTCTATGTGAACCAC 5800
       D V R M I R S T R * L I K W T A D R T R T C V N S K * H T L F I * C T V Q C L I * T R Q Q * Q L G A H Q A V A R R K W G I R S V S P   F6
      M * E * S G Q H E E C Y R G H Q M G Q E L V S M Q S E I H * F Y K A L S R V F S K P G R N D N S V P M N P L Q G G K G V L V L Y V Q H  F5
     C R S E H D K I N K V T D E M N C G K N S Y L C K V K L T D F I N L L H G S L P N L D E T T M P S R C T P C S G A K E L W Y S I C K T   F4

       E N M I T T G D Q G Y F I W * S T A Y D T S I C T C N L L I D K T F N F D I Y N H S L I F G L N F F L F L F C L K * F S L V * S S F   F1
     Q K I * S Q L V I R G T S Y G N P R H M I Q V Y V L A T S * L T K R S I S I F T I T V L S S D * T S S S S S S V S N S F R L C E A V S   F2
      R K Y D H N W * S G V L H M V I H G I * Y K Y M Y L Q P P D * Q N V Q F R Y L Q S Q S Y L R T E L L P L P L L S Q I V F A C V K Q F R  F3
5801 CAGAAAATATGATCACAACTGGTGATCAGGGGTACTTCATATGGTAATCCACGGCATATGATACAAGTATATGTACTTGCAACCTCCTGATTGACAAAACGTTCAATTTCGATATTTACAATCACAGTCTTATCTTCGGACTGAACTTCTTCCTCTTCCTCTTCTGTCTCAAATAGTTTTCGCTTGTGTGAAGCAGTTTC 6000
     ----:----|----:----|----:----|----:----|----:----|----:----|----:----|----:----|----:----|----:----|----:----|----:----|----:----|----:----|----:----|----:----|----:----|----:----|----:----|----:----|
5801 GTCTTTTATACTAGTGTTGACCACTAGTCCCCATGAAGTATACCATTAGGTGCCGTATACTATGTTCATATACATGAACGTTGGAGGACTAACTGTTTTGCAAGTTAAAGCTATAAATGTTAGTGTCAGAATAGAAGCCTGACTTGAAGAAGGAGAAGGAGAAGACAGAGTTTATCAAAAGCGAACACACTTCGTCAAAG 6000
     A S F I I V V P S * P Y K M H Y D V A Y S V L I H V Q L R R I S L V N L K S I * L * L R I K P S F K K R K R K Q R L Y N E S T H L L K   F6
      L F Y S * L Q H D P T S * I T I W P M H Y L Y I Y K C G G S Q C F T * N R Y K C D C D * R R V S S R G R G R R D * I T K A Q T F C N   F5
      C F I H D C S T I L P V E Y P L G R C I I C T Y T S A V E Q N V F R E I E I N V I V T K D E S Q V E E E E E E T E F L K R K H S A T E  F4

     V A * S C T R T C * C T Y I H T T L C T Y V S P K Y W F S W L R S A S F S A A N A A V I F C V W L K D C H T L K G C S L T R F L E S F   F1
      W L E V A P E L V D A P T F T P L S V P T S V L N T G S V G L G R P R F L L Q M Q L S S S V C G * K T A T L * R D V L L L V F W S R S  F2
       G L K L H Q N L L M H L H S H H S L Y L R Q S * I L V Q L A * V G L V F C C K C S C H L L C V A E R L P H S E G M F S Y S F F G V V   F3
6001 GTGGCTTGAAGTTGCACCAGAACTTGTTGATGCACCTACATTCACACCACTCTCTGTACCTACGTCAGTCCTAAATACTGGTTCAGTTGGCTTAGGTCGGCCTCGTTTTCTGCTGCAAATGCAGCTGTCATCTTCTGTGTGTGGCTGAAAGACTGCCACACTCTGAAGGGATGTTCTCTTACTCGTTTTTGGAGTCGTT 6200
     ----:----|----:----|----:----|----:----|----:----|----:----|----:----|----:----|----:----|----:----|----:----|----:----|----:----|----:----|----:----|----:----|----:----|----:----|----:----|----:----|
6001 CACCGAACTTCAACGTGGTCTTGAACAACTACGTGGATGTAAGTGTGGTGAGAGACATGGATGCAGTCAGGATTTATGACCAAGTCAACCGAATCCAGCCGGAGCAAAAGACGACGTTTACGTCGACAGTAGAAGACACACACCGACTTTCTGACGGTGTGAGACTTCCCTACAAGAGAATGAGCAAAAAACCTCAGCAA 6200
      T A Q L Q V L V Q Q H V * M * V V R Q V * T L G L Y Q N L Q S L D A E N E A A F A A T M K Q T H S F S Q W V R F P H E R V R K K S D N  F6
     R P K F N C W F K N I C R C E C W E R Y R R * D * I S T * N A * T P R T K Q Q L H L Q * R R H T A S L S G C E S P I N E * E N K P T T   F5
      H S S T A G S S T S A G V N V G S E T G V D T R F V P E T P K P R G R K R S C I C S D D E T H P Q F V A V S Q L S T R K S T K Q L R    F4

      K L H T A L L T N G R W M D C T * I F L Y V D T K C F Q Y R I L E S F D R C F L * L W R N V S C T D T T Y V G N V F C V K Y T G H A D  F1
       S C I R H F * Q M E G G W T A L E S S C M L T L N V S S I A S L N L L T D V F C D F G G M F L A Q T R H T L A M Y S V * S I L D M L   F2
     Q A A Y G T S D K W K V D G L H L N L P V C * H * M F P V S H P * I F * Q M F S V T L E E C F L H R H D I R W Q C I L C E V Y W T C *   F3
6201 CAAGCTGCATACGGCACTTCTGACAAATGGAAGGTGGATGGACTGCACTTGAATCTTCCTGTATGTTGACACTAAATGTTTCCAGTATCGCATCCTTGAATCTTTTGACAGATGTTTTCTGTGACTTTGGAGGAATGTTTCTTGCACAGACACGACATACGTTGGCAATGTATTCTGTGTGAAGTATACTGGACATGCTG 6400
     ----:----|----:----|----:----|----:----|----:----|----:----|----:----|----:----|----:----|----:----|----:----|----:----|----:----|----:----|----:----|----:----|----:----|----:----|----:----|----:----|
6201 GTTCGACGTATGCCGTGAAGACTGTTTACCTTCCACCTACCTGACGTGAACTTAGAAGGACATACAACTGTGATTTACAAAGGTCATAGCGTAGGAACTTAGAAAACTGTCTACAAAAGACACTGAAACCTCCTTACAAAGAACGTGTCTGTGCTGTATGCAACCGTTACATAAGACACACTTCATATGACCTGTACGAC 6400
       L S C V A S R V F P L H I S Q V Q I K R Y T S V L H K W Y R M R S D K S L H K R H S Q L F T E Q V S V V Y T P L T N Q T F Y V P C A   F6
      * A A Y P V E S L H F T S P S C K F R G T H Q C * I N G T D C G Q I K Q C I N E T V K S S H K K C L C S M R Q C H I R H S T Y Q V H Q  F5
     E L Q M R C K Q C I S P P H V A S S D E Q I N V S F T E L I A D K F R K V S T K Q S K P P I N R A C V R C V N A I Y E T H L I S S M S   F4

       L L D I                                                                                                                             F1
     T F L T X                                                                                                                             F2
      P S * H X                                                                                                                            F3
6401 ACCTTCTTGACATA 6414
     ----:----|----
6401 TGGAAGAACTGTAT 6414
     S R R S M                                                                                                                             F6
       G E Q C                                                                                                                             F5
      V K K V Y                                                                                                                            F4
```

Hemicentrotus pulcherrimus (Hpu)
(TaxID: 7650; Deuterostomia; Echinodermata; Echinozoa)
WGS: BEXV01002133.1

4879-9079
Hemicentrotus pulcherrimus DNA, scaffold2135, whole genome shotgun sequence

```
       D  F  F  T  *  P  A  C  H  L  Q  P  *  T  H  F  C  F  A  Y  P  R  S  W  K  P  Q  S  S  T  G  K  L  Y  H  I  R  V  V  Y  V  A  R  M  *  R  T  R  K  A  C  L  P  R  R  S  M  R  S  *  F  L  A  F  M  V  L   F1
        I  F  S  L  D  Q  H  V  T  F  N  L  R  L  I  F  V  L  P  T  Q  G  H  G  N  R  R  A  P  Q  E  S  S  I  T  Y  V  S  F  M  W  L  V  C  E  E  Q  E  K  P  V  F  Q  G  E  V  *  G  A  D  S  *  R  L  W  Y  *   F2
         F  F  H  L  T  S  M  S  P  S  T  L  D  S  F  L  F  C  L  P  K  V  M  E  T  A  E  L  H  R  K  A  L  S  H  T  C  R  L  C  G  S  Y  V  K  N  K  K  S  L  S  S  K  E  K  Y  E  E  L  I  L  S  V  Y  G  I   F3
   1 GATTTTTTCACTTGACCAGCATGTCACCTTCAACCTTAGACTCATTTTTGTTTTGCCTACCCAAGGTCATGGAAACCGCAGAGCTCCACAGGAAAGCTCTATCACATACGTGTCGTTTATGTGGCTCGTATGTGAAGAACAAGAAAAGCCTGTCTTCCAAGGAGAAGTATGAGGAGCTGATTCTTAGCGTTTATGGTATT 200
     ----:----|----:----|----:----|----:----|----:----|----:----|----:----|----:----|----:----|----:----|----:----|----:----|----:----|----:----|----:----|----:----|----:----|----:----|----:----|----:----|
   1 CTAAAAAAGTGAACTGGTCGTACAGTGGAAGTTGGAATCTGAGTAAAAACAAAACGGATGGGTTCCAGTACCTTTGGCGTCTCGAGGTGTCCTTTCGAGATAGTGTATGCACAGCAAATACACCGAGCATACACTTCTTGTTCTTTTCGGACAGAAGGTTCCTCTTCATACTCCTCGACTAAGAATCGCAAATACCATAA 200
       S  K  K  V  Q  G  A  H  *  R  *  G  *  V  *  K  Q  K  A  *  G  L  D  H  F  G  C  L  E  V  P  F  S  *  *  M  R  T  T  *  T  A  R  I  H  L  V  L  F  A  Q  R  G  L  L  L  I  L  L  Q  N  K  A  N  I  T  N   F6
      X  N  K  *  K  V  L  M  D  G  E  V  K  S  E  N  K  N  Q  R  G  L  T  M  S  V  A  S  S  W  L  F  A  R  D  C  V  H  R  K  H  P  E  Y  T  F  F  L  F  L  R  D  E  L  S  F  Y  S  S  S  I  R  L  T  *  P  I    F5
        I  K  E  S  S  W  C  T  V  K  L  R  L  S  M  K  T  K  G  V  W  P  *  P  F  R  L  A  G  C  S  L  E  I  V  Y  T  D  N  I  H  S  T  H  S  S  C  S  F  G  T  K  W  P  S  T  H  P  A  S  E  *  R  K  H  Y     F4

       T  S  S  *  T  T  R  M  F  I  P  L  E  S  V  Y  H  A  G  M  N  M  T  T  *  Q  G  S  *  V  V  M  K  H  T  S  S  R  I  C  L  H  S  Q  *  Y  Q  K  G  K  Q  G  N  N  I  V  R  S  *  F  V  F  *  M  K  F  S   F1
        L  Q  A  R  R  R  G  C  S  S  P  *  N  L  C  I  M  Q  V  *  T  *  P  L  D  K  G  L  E  L  L  *  S  T  H  Q  A  G  F  V  Y  T  L  N  D  I  K  K  E  N  K  A  T  I  L  *  E  V  D  L  S  F  K  *  N  F   F2
       D  F  K  L  D  D  E  D  V  H  P  P  R  I  C  V  S  C  R  Y  E  H  D  H  L  T  R  V  L  S  C  Y  E  A  H  I  K  Q  D  L  F  T  L  S  M  I  S  K  R  K  T  R  Q  Q  Y  C  E  K  L  I  C  L  L  N  E  I  F   F3
 201 GACTTCAAGCTAGACGACGAGGATGTTCATCCCCCTAGAATCTGTGTATCATGCAGGTATGAACATGACCACTTGACAAGGGTCTTGAGTTGTTATGAAGCACACATCAAGCAGGATTTGTTTACACTCTCAATGATATCAAAAAGGAAAACAAGGCAACAATATTGTGAGAAGTTGATTTGTCTTTTAAATGAAATTTT 400
     ----:----|----:----|----:----|----:----|----:----|----:----|----:----|----:----|----:----|----:----|----:----|----:----|----:----|----:----|----:----|----:----|----:----|----:----|----:----|----:----|
 201 CTGAAGTTCGATCTGCTGCTCCTACAAGTAGGGGGATCTTAGACACATAGTACGTCCATACTTGTACTGGTGAACTGTTCCCAGAACTCAACAATACTTCGTGTGTAGTTCGTCCTAAACAAATGTGAGAGTTACTATAGTTTTTCCTTTTGTTCCGTTGTTATAACACTCTTCAACTAAACAGAAAATTTACTTTAAAA 400
        V  E  L  *  V  V  L  I  N  M  G  R  S  D  T  Y  *  A  P  I  F  M  V  V  Q  C  P  D  Q  T  T  I  F  C  V  D  L  L  I  Q  K  C  E  *  H  Y  *  F  P  F  C  P  L  L  I  T  L  L  Q  N  T  K  *  I  F  N   F6
       S  K  L  S  S  S  S  S  T  *  G  G  L  I  Q  T  D  H  L  Y  S  C  S  W  K  V  L  T  K  L  Q  *  S  A  C  M  L  C  S  K  N  V  S  E  I  I  D  F  L  F  V  L  C  C  Y  Q  S  F  N  I  Q  R  K  F  S  I  K   F5
      Q  S  *  A  L  R  R  P  H  E  D  G  *  F  R  H  I  M  C  T  H  V  H  G  S  S  L  P  R  S  N  N  H  L  V  C  *  A  P  N  T  *  V  R  L  S  I  L  F  S  F  L  A  V  I  N  H  S  T  S  K  D  K  L  H  F  K    F4

        L  L  *  H  S  H  S  V  R  P  E  Q  I  K  S  R  D  *  D  G  C  H  Q  C  L  D  L  K  C  S  E  H  P  Y  H  I  T  N  K  S  L  Q  Y  F  C  I  I  H  L  N  N  L  S  Q  I  S  *  *  K  I  K  L  Q  K  I  K    F1
      H  C  C  S  T  V  I  P  S  D  L  S  R  L  N  P  E  I  E  M  A  A  T  S  V  L  I  *  N  V  L  N  T  R  T  T  S  Q  T  S  L  C  S  T  F  V  S  F  T  *  I  I  *  V  K  F  H  D  E  R  *  S  Y  K  K  *  N   F2
       I  V  V  A  Q  S  F  R  Q  T  *  A  D  *  I  Q  R  L  R  W  L  P  P  V  S  *  F  E  M  F  *  T  P  V  P  H  H  K  Q  V  F  A  V  L  L  Y  H  S  L  K  *  F  K  S  N  F  M  M  K  D  K  V  T  K  N  K  T   F3
 401 CATTGTTGTAGCACAGTCATTCCGTCAGACCTGAGCAGATTAAATCCAGAGATTGAGATGGCTGCCACCAGTGTCTTGATTTGAAATGTTCTGAACACCCGTACCACATCACAAACAAGTCTTTGCAGTACTTTTGTATCATTCACTTAAATAATTTAAGTCAAATTTCATGATGAAAGATAAAGTTACAAAAAATAAAA 600
     ----:----|----:----|----:----|----:----|----:----|----:----|----:----|----:----|----:----|----:----|----:----|----:----|----:----|----:----|----:----|----:----|----:----|----:----|----:----|----:----|
 401 GTAACAACATCGTGTCAGTAAGGCAGTCTGGACTCGTCTAATTTAGGTCTCTAACTCTACCGACGGTGGTCACAGAACTAAACTTTACAAGACTTGTGGGCATGGTGTAGTGTTTGTTCAGAAACGTCATGAAAACATAGTAAGTGAATTTATTAAATTCAGTTTAAAGTACTACTTTCTATTTCAATGTTTTTTATTTT 600
      E  N  N  Y  C  L  *  E  T  L  G  S  C  I  L  D  L  S  Q  S  P  Q  W  W  H  R  S  K  F  H  E  S  C  G  Y  W  M  V  F  L  D  K  C  Y  K  Q  I  M  *  K  F  L  K  L  *  I  E  H  H  F  I  F  N  C  F  I  F   F6
       M  T  T  A  C  D  N  R  *  V  Q  A  S  *  I  W  L  N  L  H  S  G  G  T  D  Q  N  S  I  N  Q  V  G  T  G  C  *  L  C  T  K  A  T  S  K  Y  *  E  S  L  Y  N  L  D  F  K  M  I  F  S  L  T  V  F  F  L    F5
      *  Q  Q  L  V  T  M  G  D  S  R  L  L  N  F  G  S  I  S  I  A  A  V  L  T  K  I  Q  F  T  R  F  V  R  V  V  D  C  V  L  R  Q  L  V  K  T  D  N  V  *  I  I  *  T  L  N  *  S  S  L  Y  L  *  L  F  Y  F   F4

      H  A  V  Q  L  T  R  *  M  N  I  S  E  Y  K  L  T  G  L  Y  L  Y  L  M  S  K  F  N  F  S  S  L  F  S  Q  S  E  S  S  E  *  K  S  H  A  I  C  V  R  I  F  P  H  Y  R  H  F  V  K  T  G  T  I  Y  F  L  T    F1
       T  L  Y  N  L  Q  D  E  *  I  F  L  N  I  N  *  Q  G  Y  I  C  I  *  C  P  N  L  T  F  R  P  Y  F  H  K  V  K  V  V  N  E  N  R  M  Q  F  V  C  G  F  F  L  T  T  V  T  L  *  K  Q  E  Q  F  I  F  S  P   F2
        R  C  T  T  Y  K  M  N  E  Y  F  *  I  *  T  D  R  V  I  F  V  F  D  V  Q  I  *  L  F  V  P  I  F  T  K  *  K  *  *  M  K  I  A  C  N  L  C  A  D  F  S  S  L  P  S  L  C  E  N  R  N  N  L  F  S  H    F3
 601 CACGCTGTACAACTTACAAGATGAATGAATATTTCTGAATATAAACTGACAGGGTTATATTTGTATTTGATGTCCAAATTTAACTTTTCGTCCCTATTTTCACAAAGTGAAAGTAGTGAATGAAAATCGCATGCAATTTGTGTGCGGATTTTTCCTCACTACCGTCACTTTGTGAAAACAGGAACAATTTATTTTCTCAC 800
     ----:----|----:----|----:----|----:----|----:----|----:----|----:----|----:----|----:----|----:----|----:----|----:----|----:----|----:----|----:----|----:----|----:----|----:----|----:----|----:----|
 601 GTGCGACATGTTGAATGTTCTACTTACTTATAAAGACTTATATTTGACTGTCCCAATATAAACATAAACTACAGGTTTAAATTGAAAAGCAGGGATAAAAGTGTTTCACTTTCATCACTTACTTTTAGCGTACGTTAAACACACGCCTAAAAAGGAGTGATGGCAGTGAAACACTTTTGTCCTTGTTAAATAAAAGAGTG 800
       C  A  T  C  S  V  L  H  I  F  I  E  S  Y  L  S  V  P  N  Y  K  Y  K  I  D  L  N  L  K  E  D  R  N  E  C  L  S  L  L  S  H  F  D  C  A  I  Q  T  R  I  K  G  *  *  R  *  K  T  F  V  P  V  I  *  K  R  V   F6
      V  R  Q  V  V  *  L  I  F  S  Y  K  Q  I  Y  V  S  L  T  I  N  T  N  S  T  W  I  *  S  K  T  G  I  K  V  F  H  F  Y  H  I  F  I  A  H  L  K  H  A  S  K  E  E  S  G  D  S  Q  S  F  L  F  L  K  N  E  *   F5
       V  S  Y  L  K  C  S  S  H  I  N  R  F  I  F  Q  C  P  *  I  Q  I  Q  H  G  F  K  V  K  R  G  *  K  *  L  T  F  T  T  F  S  F  R  M  C  N  T  H  P  N  K  R  V  V  T  V  K  H  F  C  S  C  N  I  K  E     F4

      S  I  K  I  K  L  Y  L  S  H  L  F  I  D  Y  G  *  R  E  V  I  V  A  M  L  K  D  Q  P  T  Q  Q  V  A  R  L  L  P  I  S  L  P  T  Q  S  W  S  R  V  L  Y  V  R  Q  L  H  Q  P  N  G  R  L  *  E  Q  M  A   F1
       V  S  R  *  N  Y  I  C  L  I  C  L  *  T  M  D  D  E  K  *  *  S  Q  C  *  R  T  N  L  P  N  K  W  Q  D  S  C  Q  F  L  C  P  P  R  A  G  A  V  F  Y  M  *  G  N  Y  I  N  Q  T  E  G  C  R  N  R  W   F2
      Q  Y  Q  D  K  I  I  F  V  S  F  V  Y  R  L  W  M  T  R  S  D  S  R  N  A  E  G  P  T  Y  P  T  S  G  K  T  L  A  N  F  S  A  H  P  E  L  E  P  C  S  I  C  E  A  T  T  S  T  K  R  K  A  V  G  T  D  G   F3
 801 CAGTATCAAGATAAAATTATATTTGTCTCATTTGTTTATAGACTATGGATGACGAGAAGTGATAGTCGCAATGCTGAAGGACCAACCTACCCAACAAGTGGCAAGACTCTTGCCAATTTCTCTGCCCACCCAGAGCTGGAGCCGTGTTCTATATGTGAGGCAACTACATCAACCAAACGGAAGGCTGTAGGAACAGATGG 1000
     ----:----|----:----|----:----|----:----|----:----|----:----|----:----|----:----|----:----|----:----|----:----|----:----|----:----|----:----|----:----|----:----|----:----|----:----|----:----|----:----|
 801 GTCATAGTTCTATTTTAATATAAACAGAGTAAACAAATATCTGATACCTACTGCTCTTCACTATCAGCGTTACGACTTCCTGGTTGGATGGGTTGTTCACCGTTCTGAGAACGGTTAAAGAGACGGGTGGGTCTCGACCTCGGCACAAGATATACACTCCGTTGATGTAGTTGGTTTGCCTTCCGACATCCTTGTCTACC 1000
       L  I  L  I  F  N  Y  K  D  *  K  N  I  S  *  P  H  R  S  T  I  T  A  I  S  F  S  W  G  V  W  C  T  A  L  S  K  G  I  E  R  G  V  W  L  Q  L  R  T  R  Y  T  L  C  S  C  *  G  F  P  L  S  Y  S  C  I    F6
      W  Y  *  S  L  I  I  N  T  E  N  T  *  L  S  H  I  V  L  L  S  L  R  L  A  S  P  G  V  *  G  V  L  P  L  V  R  A  L  K  E  A  W  G  S  S  S  G  H  E  I  H  S  A  V  V  D  V  L  R  F  A  T  P  V  S  P   F5
      G  T  D  L  Y  F  *  I  Q  R  M  Q  K  Y  V  I  S  S  S  F  H  Y  D  C  H  Q  L  V  L  R  G  L  L  H  C  S  E  Q  W  N  R  Q  G  G  L  A  P  A  T  N  *  I  H  P  L  *  M  L  W  V  S  P  Q  L  F  L  H   F4

       F  L  P  Q  R  N  H  Q  L  S  F  L  D  Q  M  S  S  K  L  P  A  A  S  Q  P  L  Y  P  Q  L  P  Q  V  I  S  Q  S  N  Q  R  R  E  V  I  V  A  M  L  K  D  Q  P  T  I  C  V  L  Y  V  L  Q  L  R  *  P  K    F1
      P  S  S  P  K  E  T  I  S  C  R  F  W  T  R  *  A  A  S  F  L  Q  L  H  S  P  F  T  H  N  C  H  K  L  S  A  N  Q  T  K  D  E  K  *  *  S  Q  C  *  R  T  N  L  P  S  V  F  Y  M  C  C  N  Y  V  N  Q  R   F2
       L  P  P  P  K  K  P  S  A  V  V  S  G  P  D  E  Q  Q  A  S  C  S  F  T  A  P  L  P  T  T  A  T  S  Y  Q  P  I  K  P  K  T  R  S  D  S  R  N  A  E  G  P  T  Y  H  L  C  S  I  C  A  A  T  T  L  T  K  E   F3
1001 CCTTCCTCCCCCAAAGAAACCATCAGCTGTCGTTTCTGGACCAGATGAGCAGCAAGCTTCCTGCAGCTTCACAGCCCCTTTACCCACAACTGCCACAAGTTATCAGCCAATCAAACCAAAGACGAGAAGTGATAGTCGCAATGCTGAAGGACCAACCTACCATCTGTGTTCTATATGTGCTGCAACTACGTTAACCAAAG 1200
     ----:----|----:----|----:----|----:----|----:----|----:----|----:----|----:----|----:----|----:----|----:----|----:----|----:----|----:----|----:----|----:----|----:----|----:----|----:----|----:----|
1001 GGAAGGAGGGGGTTTCTTTGGTAGTCGACAGCAAAGACCTGGTCTACTCGTCGTTCGAAGGACGTCGAAGTGTCGGGGAAATGGGTGTTGACGGTGTTCAATAGTCGGTTAGTTTGGTTTCTGCTCTTCACTATCAGCGTTACGACTTCCTGGTTGGATGGTAGACACAAGATATACACGACGTTGATGCAATTGGTTTC 1200
      A  K  R  G  W  L  F  W  *  S  D  N  R  S  W  I  L  L  L  S  G  A  A  E  C  G  R  *  G  C  S  G  C  T  I  L  W  D  F  W  L  R  S  T  I  T  A  I  S  F  S  W  G  V  M  Q  T  R  Y  T  S  C  S  R  *  G  F   F6
       R  G  G  G  F  F  G  D  A  T  T  E  P  G  S  S  C  C  A  E  Q  L  K  V  A  G  K  G  V  V  A  V  L  *  *  G  I  L  G  F  V  L  L  S  L  R  L  A  S  P  G  V  *  W  R  H  E  I  H  A  A  V  V  N  V  L    F5
       G  E  E  G  L  S  V  M  L  Q  R  K  Q  V  L  H  A  A  L  K  R  C  S  *  L  G  K  V  W  L  Q  W  L  N  D  A  L  *  V  L  S  S  F  H  Y  D  C  H  Q  L  V  L  R  G  D  T  N  *  I  H  Q  L  *  T  L  W  L   F4

      R  R  L  *  E  Q  M  I  F  L  P  Q  R  Y  H  Q  L  Q  F  L  D  Q  M  N  R  *  L  S  V  V  S  Q  P  L  Y  P  Q  L  P  Q  P  L  Y  P  Q  L  P  Q  S  F  *  P  Q  L  P  Q  S  L  *  P  Q  P  P  Q  P  F  *   F1
       E  G  C  R  N  R  *  S  S  S  P  K  D  T  I  S  C  S  F  W  T  R  *  T  D  S  S  L  *  F  H  S  P  F  T  P  N  C  H  S  P  F  T  P  N  C  H  S  P  F  N  P  N  C  H  S  P  F  N  P  N  R  H  S  P  F  N   F2
       K  A  V  G  T  D  D  L  P  P  P  K  I  P  S  A  A  V  S  G  P  D  E  Q  I  A  L  C  S  F  T  A  P  L  P  P  T  A  T  A  P  L  P  P  T  A  T  V  L  L  T  P  T  A  T  V  P  L  T  P  T  A  T  A  L  L    F3
1201 AGAAGGCTGTAGGAACAGATGATCTTCCTCCCCCAAAGATACCATCAGCTGCAGTTTCTGGACCAGATGAACAGATAGCTCTCTGTAGTTTCACAGCCCCTTTACCCCCAACTGCCACAGCCCCTTTACCCCCAACTGCCACAGTCCTTTTAACCCCAACTGCCACAGTCCCTTTAACCCCAACCGCCACAGCCCTTTTA 1400
     ----:----|----:----|----:----|----:----|----:----|----:----|----:----|----:----|----:----|----:----|----:----|----:----|----:----|----:----|----:----|----:----|----:----|----:----|----:----|----:----|
1201 TCTTCCGACATCCTTGTCTACTAGAAGGAGGGGGTTTCTATGGTAGTCGACGTCAAAGACCTGGTCTACTTGTCTATCGAGAGACATCAAAGTGTCGGGGAAATGGGGGTTGACGGTGTCGGGGAAATGGGGGTTGACGGTGTCAGGAAAATTGGGGTTGACGGTGTCAGGGAAATTGGGGTTGGCGGTGTCGGGAAAAT 1400
       L  L  S  Y  S  C  I  I  K  R  G  W  L  Y  W  *  S  C  N  R  S  W  I  F  L  Y  S  E  T  T  E  C  G  R  *  G  W  S  G  C  G  R  *  G  W  S  G  C  D  K  *  G  W  S  G  C  D  R  *  G  W  G  G  C  G  K  *   F6
      S  F  A  T  P  V  S  S  R  G  G  G  F  I  G  D  A  A  T  E  P  G  S  S  C  I  A  R  Q  L  K  V  A  G  K  G  G  V  A  V  A  G  K  G  G  V  A  V  T  R  K  V  G  V  A  V  T  G  K  V  G  V  A  V  A  R  K    F5
       S  P  Q  L  F  L  H  D  E  E  G  L  S  V  M  L  Q  L  K  Q  V  L  H  V  S  L  E  R  Y  N  *  L  G  K  V  G  L  Q  W  L  G  K  V  G  L  Q  W  L  G  K  L  G  L  Q  W  L  G  K  L  G  L  R  W  L  G  K     F4

      S  *  L  P  Q  P  L  *  P  *  L  P  Q  P  L  *  P  K  L  P  Q  S  L  *  P  Q  L  P  Q  P  L  Y  P  Q  L  P  Q  S  L  *  P  Q  L  S  Q  P  L  *  P  Q  L  P  Q  P  L  *  P  Q  L  P  Q  P  L  *  P  Q  L   F1
       P  N  C  H  S  P  F  N  P  N  C  H  S  P  F  N  P  S  C  H  S  P  F  N  P  N  C  H  S  P  F  T  P  N  C  H  S  P  F  N  P  N  C  H  S  P  F  N  P  N  C  H  S  P  F  N  P  N  C  H  S  P  F  N  P  N    F2
       I  L  T  A  T  A  P  L  T  L  T  A  T  A  P  L  T  Q  A  A  T  V  P  L  T  P  T  A  T  A  P  L  P  P  T  A  T  V  P  L  T  P  T  V  T  A  P  L  T  P  T  A  T  A  P  L  T  P  T  A  T  A  P  L  T  P  T   F3
1401 ATCCTAACTGCCACAGCCCCTTTAACCCTAACTGCCACAGCCCCTTTAACCCAAGCTGCCACAGTCCCTTTAACCCCAACTGCCACAGCCCCTTTACCCCCAACTGCCACAGTCCCTTTAACCCCAACTGTCACAGCCCCTTTAACCCCAACTGCCACAGCCCCTTTAACCCCAACTGCCACAGCCCCTTTAACCCCAAC 1600
     ----:----|----:----|----:----|----:----|----:----|----:----|----:----|----:----|----:----|----:----|----:----|----:----|----:----|----:----|----:----|----:----|----:----|----:----|----:----|----:----|
1401 TAGGATTGACGGTGTCGGGGAAATTGGGATTGACGGTGTCGGGGAAATTGGGTTCGACGGTGTCAGGGAAATTGGGGTTGACGGTGTCGGGGAAATGGGGGTTGACGGTGTCAGGGAAATTGGGGTTGACAGTGTCGGGGAAATTGGGGTTGACGGTGTCGGGGAAATTGGGGTTGACGGTGTCGGGGAAATTGGGGTTG 1600
       D  *  S  G  C  G  R  *  G  *  S  G  C  G  R  *  G  L  S  G  C  D  R  *  G  W  S  G  C  G  R  *  G  W  S  G  C  D  R  *  G  W  S  D  C  G  R  *  G  W  S  G  C  G  R  *  G  W  S  G  C  G  R  *  G  W    F6
      I  R  V  A  V  A  G  K  V  R  V  A  V  A  G  K  V  W  A  A  V  T  G  K  V  G  V  A  V  A  G  K  G  G  V  A  V  T  G  K  V  G  V  T  V  A  G  K  V  G  V  A  V  A  G  K  V  G  V  A  V  A  G  K  V  G  V   F5
      L  G  L  Q  W  L  G  K  L  G  L  Q  W  L  G  K  L  G  L  Q  W  L  G  K  L  G  L  Q  W  L  G  K  V  G  L  Q  W  L  G  K  L  G  L  Q  *  L  G  K  L  G  L  Q  W  L  G  K  L  G  L  Q  W  L  G  K  L  G  L   F4

       P  Q  P  L  *  P  Q  L  P  Q  T  L  *  P  Q  L  P  Q  G  I  G  Q  S  *  P  R  T  G  L  I  S  H  V  L  Y  S  P  L  S  *  L  C  L  Q  G  S  P  L  S  E  Q  K  A  V  S  T  M  C  G  E  I  A  Q  R  T  E    F1
      C  H  S  P  F  N  P  N  C  H  R  P  F  N  P  N  C  H  K  V  S  A  N  R  D  Q  G  P  G  S  F  H  T  C  S  I  L  P  C  P  N  C  A  C  K  E  V  P  C  Q  S  K  R  Q  S  P  L  C  A  E  R  L  R  K  E  Q  S   F2
       A  T  A  P  L  T  P  T  A  T  D  P  L  T  P  T  A  T  R  Y  R  P  I  V  T  K  D  R  A  H  F  T  R  A  L  F  S  P  V  L  T  V  P  A  R  K  S  P  V  R  A  K  G  S  L  H  Y  V  R  R  D  C  A  K  N  R  A   F3
1601 TGCCACAGCCCCTTTAACCCCAACTGCCACAGACCCTTTAACCCCAACTGCCACAAGGTATCGGCCAATCGTGACCAAGGACCGGGCTCATTTCACACGTGCTCTATTCTCCCCTGTCCTAACTGTGCCTGCAAGGAAGTCCCCTGTCAGAGCAAAAGGCAGTCTCCACTATGTGCGGAGAGATTGCGCAAAGAACAGAG 1800
     ----:----|----:----|----:----|----:----|----:----|----:----|----:----|----:----|----:----|----:----|----:----|----:----|----:----|----:----|----:----|----:----|----:----|----:----|----:----|----:----|
1601 ACGGTGTCGGGGAAATTGGGGTTGACGGTGTCTGGGAAATTGGGGTTGACGGTGTTCCATAGCCGGTTAGCACTGGTTCCTGGCCCGAGTAAAGTGTGCACGAGATAAGAGGGGACAGGATTGACACGGACGTTCCTTCAGGGGACAGTCTCGTTTTCCGTCAGAGGTGATACACGCCTCTCTAACGCGTTTCTTGTCTC 1800
      S  G  C  G  R  *  G  W  S  G  C  V  R  *  G  W  S  G  C  P  I  P  W  D  H  G  L  V  P  S  M  E  C  T  S  *  E  G  R  D  *  S  H  R  C  P  L  G  R  D  S  C  F  A  T  E  V  I  H  P  S  I  A  C  L  V  S   F6
       A  V  A  G  K  V  G  V  A  V  S  G  K  V  G  V  A  V  L  Y  R  G  I  T  V  L  S  R  A  *  K  V  R  A  R  N  E  G  T  R  V  T  G  A  L  F  D  G  T  L  A  F  P  L  R  W  *  T  R  L  S  Q  A  F  F  L    F5
       Q  W  L  G  K  L  G  L  Q  W  L  G  K  L  G  L  Q  W  L  T  D  A  L  R  S  W  P  G  P  E  N  *  V  H  E  I  R  G  Q  G  L  Q  A  Q  L  S  T  G  Q  *  L  L  L  C  D  G  S  H  A  S  L  N  R  L  S  C  L   F4

      P  K  E  P  W  T  S  *  P  H  T  A  L  P  K  T  R  T  R  Q  T  S  G  S  S  D  F  T  T  G  *  G  M  K  K  M  S  E  Q  K  W  *  V  I  N  N  M  V  *  *  V  L  C  S  H  T  L  V  L  F  R  K  N  E  N  A  K   F1
       Q  R  S  P  G  L  H  D  L  T  Q  R  C  Q  K  R  G  R  D  R  P  L  V  L  R  T  S  Q  Q  V  E  E  *  K  R  *  A  S  K  N  G  K  *  L  T  I  W  F  D  E  F  S  V  V  I  R  *  S  Y  S  G  K  M  K  M  L  N   F2
       K  G  A  L  D  F  M  T  S  H  S  A  A  K  N  E  D  E  T  D  L  W  F  F  G  L  H  N  R  L  R  N  E  K  D  E  R  A  K  M  V  S  N  *  Q  Y  G  L  M  S  S  L  *  S  Y  A  S  L  I  Q  E  K  *  K  C  *   F3
1801 CCAAAGGAGCCCTGGACTTCATGACCTCACACAGCGCTGCCAAAAACGAGGACGAGACAGACCTCTGGTTCTTCGGACTTCACAACAGGTTGAGGAATGAAAAAGATGAGCGAGCAAAAATGGTAAGTAATTAACAATATGGTTTGATGAGTTCTCTGTAGTCATACGCTAGTCTTATTCAGGAAAAATGAAAATGCTAA 2000
     ----:----|----:----|----:----|----:----|----:----|----:----|----:----|----:----|----:----|----:----|----:----|----:----|----:----|----:----|----:----|----:----|----:----|----:----|----:----|----:----|
1801 GGTTTCCTCGGGACCTGAAGTACTGGAGTGTGTCGCGACGGTTTTTGCTCCTGCTCTGTCTGGAGACCAAGAAGCCTGAAGTGTTGTCCAACTCCTTACTTTTTCTACTCGCTCGTTTTTACCATTCATTAATTGTTATACCAAACTACTCAAGAGACATCAGTATGCGATCAGAATAAGTCCTTTTTACTTTTACGATT 2000
      G  F  S  G  Q  V  E  H  G  *  V  A  S  G  F  V  L  V  L  C  V  E  P  E  E  S  K  V  V  P  Q  P  I  F  F  I  L  S  C  F  H  Y  T  I  L  L  I  T  Q  H  T  R  Q  L  *  V  S  T  K  N  L  F  F  S  F  A  L   F6
      A  L  P  A  R  S  K  M  V  E  C  L  A  A  L  F  S  S  S  V  S  R  Q  N  K  P  S  *  L  L  N  L  F  S  F  S  S  R  A  F  I  T  L  L  *  C  Y  P  K  I  L  E  R  Y  D  Y  A  L  R  I  *  S  F  H  F  H  *   F5
       W  L  L  G  P  S  *  S  R  V  C  R  Q  W  F  R  P  R  S  L  G  R  T  R  R  V  E  C  C  T  S  S  H  F  L  H  A  L  L  F  P  L  Y  N  V  I  H  N  S  S  N  E  T  T  M  R  *  D  *  E  P  F  I  F  I  S    F4

      F  D  S  L  M  I  F  E  L  *  R  N  I  F  E  S  S  I  S  K  G  K  S  K  I  F  V  P  E  M  N  I  F  H  L  L  T  G  V  S  N  I  T  S  T  *  I  K  S  H  A  A  E  I  N  S  L  N  I  Y  V  L  H  V  Y  S  S   F1
       L  I  V  *  *  F  S  S  Y  E  E  T  F  L  N  Q  V  F  Q  R  A  S  P  R  Y  L  Y  L  R  *  I  F  F  I  F  *  L  G  S  Q  I  S  L  V  L  E  L  K  V  M  Q  L  R  *  I  P  *  I  Y  M  C  Y  M  Y  I  H    F2
      I  *  *  S  N  D  F  R  A  M  K  K  H  F  *  I  K  Y  F  K  G  Q  V  Q  D  I  C  T  *  D  E  Y  F  S  S  F  N  W  G  L  K  Y  H  *  Y  L  N  *  K  S  C  S  *  D  K  F  P  K  Y  I  C  A  T  C  I  F  I   F3
2001 ATTTGATAGTCTAATGATTTTCGAGCTATGAAGAAACATTTTTGAATCAAGTATTTCAAAGGGCAAGTCCAAGATATTTGTACCTGAGATGAATATTTTTCATCTTTTAACTGGGGTCTCAAATATCACTAGTACTTGAATTAAAAGTCATGCAGCTGAGATAAATTCCCTAAATATATATGTGCTACATGTATATTCAT 2200
     ----:----|----:----|----:----|----:----|----:----|----:----|----:----|----:----|----:----|----:----|----:----|----:----|----:----|----:----|----:----|----:----|----:----|----:----|----:----|----:----|
2001 TAAACTATCAGATTACTAAAAGCTCGATACTTCTTTGTAAAAACTTAGTTCATAAAGTTTCCCGTTCAGGTTCTATAAACATGGACTCTACTTATAAAAAGTAGAAAATTGACCCCAGAGTTTATAGTGATCATGAACTTAATTTTCAGTACGTCGACTCTATTTAAGGGATTTATATATACACGATGTACATATAAGTA 2200
       N  S  L  R  I  I  K  S  S  H  L  F  M  K  S  D  L  I  E  F  P  L  D  L  I  N  T  G  S  I  F  I  K  *  R  K  V  P  T  E  F  I  V  L  V  Q  I  L  L  *  A  A  S  I  F  E  R  F  I  Y  T  S  C  T  Y  E    F6
       I  Q  Y  D  L  S  K  R  A  I  F  F  C  K  Q  I  L  Y  K  L  P  C  T  W  S  I  Q  V  Q  S  S  Y  K  E  D  K  L  Q  P  R  L  Y  *  *  Y  K  F  *  F  D  H  L  Q  S  L  N  G  L  Y  I  H  A  V  H  I  N  M   F5
      F  K  I  T  *  H  N  E  L  *  S  S  V  N  K  F  *  T  N  *  L  A  L  G  L  Y  K  Y  R  L  H  I  N  K  M  K  *  S  P  D  *  I  D  S  T  S  S  N  F  T  M  C  S  L  Y  I  G  *  I  Y  I  H  *  M  Y  I  *   F4

       F  V  V  F  F  F  T  Y  L  G  N  G  A  L  D  R  K  E  E  Y  N  *  F  V  Y  R  *  L  P  G  D  A  R  G  D  S  L  H  Q  R  D  V  C  R  E  I  P  L  F  E  V  K  R  G  H  D  L  Q  T  T  *  S  A  H  Q  Q    F1
      L  L  L  Y  S  F  S  P  I  *  V  M  E  L  W  T  E  R  K  N  T  T  D  L  S  I  D  D  C  L  A  M  R  V  G  T  L  C  T  K  G  M  Y  A  E  K  Y  R  F  L  K  S  K  G  D  T  T  F  K  P  P  S  Q  L  T  N  R   F2
       F  C  C  I  L  F  H  L  F  R  *  W  S  F  G  Q  K  G  R  I  Q  L  I  C  L  *  M  T  A  W  R  C  A  W  G  L  F  A  P  K  G  C  M  Q  R  N  T  A  F  *  S  Q  K  G  T  R  P  S  N  H  L  V  S  S  P  T  G   F3
2201 CTTTGTTGTATTCTTTTTCACCTATTTAGGTAATGGAGCTTTGGACAGAAAGGAAGAATACAACTGATTTGTCTATAGATGACTGCCTGGCGATGCGCGTGGGGACTCTTTGCACCAAAGGGATGTATGCAGAGAAATACCGCTTTTTGAAGTCAAAAGGGGACACGACCTTCAAACCACCTAGTCAGCTCACCAACAG 2400
     ----:----|----:----|----:----|----:----|----:----|----:----|----:----|----:----|----:----|----:----|----:----|----:----|----:----|----:----|----:----|----:----|----:----|----:----|----:----|----:----|
2201 GAAAACAACATAAGAAAAAGTGGATAAATCCATTACCTCGAAACCTGTCTTTCCTTCTTATGTTGACTAAACAGATATCTACTGACGGACCGCTACGCGCACCCCTGAGAAACGTGGTTTCCCTACATACGTCTCTTTATGGCGAAAAACTTCAGTTTTCCCCTGTGCTGGAAGTTTGGTGGATCAGTCGAGTGGTTGTC 2400
      D  K  T  T  N  K  K  V  *  K  P  L  P  A  K  S  L  F  S  S  Y  L  Q  N  T  *  L  H  S  G  P  S  A  R  P  S  E  K  C  W  L  S  T  H  L  S  I  G  S  K  S  T  L  L  P  C  S  R  *  V  V  *  D  A  *  W  C   F6
       K  Q  Q  I  R  K  *  R  N  L  Y  H  L  K  P  C  F  P  L  I  C  S  I  Q  R  Y  I  V  A  Q  R  H  A  H  P  S  K  A  G  F  P  H  I  C  L  F  V  A  K  Q  L  *  F  P  V  R  G  E  F  W  R  T  L  E  G  V    F5
       R  K  N  Y  E  K  E  G  I  *  T  I  S  S  Q  V  S  L  F  F  V  V  S  K  D  I  S  S  Q  R  A  I  R  T  P  V  R  Q  V  L  P  I  Y  A  S  F  Y  R  K  K  F  D  F  P  S  V  V  K  L  G  G  L  *  S  V  L  L   F4
```

```
       G K L L H A W Q C E V R S D G W R E M C L S H S * E I T G R V * * Q F D V * A H P D Q R A Q Q T D * I C T A K L Y W R R M E L P R G S   F1
        E S Y Y M P G N V R F G L M D G G K C V Y H T P E K S L E E F D D N S M Y E P I R I N V R S K L T E F A L P N C I G V A W S Y P E A V  F2
        K A I T C L A M * G S V * W M E G N V S I T L L R N H W K S L M T I R C M S P S G S T C A A N * L N L H C Q T V L A S H G A T Q R Q    F3
2401 GGAAAGCTATTACATGCCTGGCAATGTGAGGTTCGGTCTGATGGATGGAGGGAAATGTGTCTATCACACTCCTGAGAAATCACTGGAAGAGTTTGATGACAATTCGATGTATGAGCCCATCCGGATCAACGTGCGCAGCAAACTGACTGAATTTGCACTGCCAAACTGTATTGGCGTCGCATGGAGCTACCCAGAGGCAG 2600
     ----:----|----:----|----:----|----:----|----:----|----:----|----:----|----:----|----:----|----:----|----:----|----:----|----:----|----:----|----:----|----:----|----:----|----:----|----:----|----:----|
2401 CCTTTCGATAATGTACGGACCGTTACACTCCAAGCCAGACTACCTACCTCCCTTTACACAGATAGTGTGAGGACTCTTTAGTGACCTTCTCAAACTACTGTTAAGCTACATACTCGGGTAGGCCTAGTTGCACGCGTCGTTTGACTGACTTAAACGTGACGGTTTGACATAACCGCAGCGTACCTCGATGGGTCTCCGTC 2600
       P F S N C A Q C H S T R D S P H L S I H R D C E Q S I V P L T Q H C N S T H A W G S * R A C C V S Q I Q V A L S Y Q R R M S S G L P L   F6
      P F A I V H R A I H P E T Q H I S P F T D I V S R L F * Q F L K I V I R H I L G D P D V H A A F Q S F K C Q W V T N A D C P A V W L C    F5
       S L * * M G P L T L N P R I S P P F H T * * V G S F D S S S N S S L E I Y S G M R I L T R L L S V S N A S G F Q I P T A H L * G S A     F4

       C K N P G R A R * K H P R R N A * S R P Q P * * P I H H H * H H I E R W G R W N G G D R S P Q D E V R Q I P A * Q G I S G F I C S L E   F1
        A K T L E E L D E N I R E G M L K V G L N P D D P S I I I D T T L K D G A D G M G E I A V H K M K S D K F L P D K A F R A S F V V L  F2
      L Q K P W K S * M K T S E K E C L K S A S T L M T H P S S L T P H * K M G Q M E W G R S Q S T R * S Q T N S C L T R H F G L H L * S *  F3
2601 TTGCAAAAACCCTGGAAGAGCTAGATGAAAACATCCGAGAAGGAATGCTTAAAGTCGGCCTCAACCCTGATGACCCATCCATCATCATTGACACCACATTGAAAGATGGGGCAGATGGAATGGGGGAGATCGCAGTCCACAAGATGAAGTCAGACAAATTCCTGCCTGACAAGGCATTTCGGGCTTCATTTGTAGTCTTG 2800
     ----:----|----:----|----:----|----:----|----:----|----:----|----:----|----:----|----:----|----:----|----:----|----:----|----:----|----:----|----:----|----:----|----:----|----:----|----:----|----:----|
2601 AACGTTTTTGGGACCTTCTCGATCTACTTTTGTAGGCTCTTCCTTACGAATTTCAGCCGGAGTTGGGACTACTGGGTAGGTAGTAGTAACTGTGGTGTAACTTTCTACCCCGTCTACCTTACCCCCTCTAGCGTCAGGTGTTCTACTTCAGTCTGTTTAAGGACGGACTGTTCCGTAAAGCCCGAAGTAAACATCAGAAC 2800
       Q L F G P L A L H F C G L L F A * L R G * G Q H G M W * * Q C W M S L H P L H F P P S R L G C S S T L C I G A Q C P M E P K M Q L R   F6
      N C F G Q F L * I F V D S F S H K F D A E V R I V W G D D N V G C Q F I P C I S H P L D C D V L H L * V F E Q R V L C K P S * K Y D Q    F5
      T A F V R S S S S S F M R S P I S L T P R L G S S G D M M M S V V N F S P A S P I P S I A T W L I F D S L N R G S L A N R A E N T T K    F4

       M * G Q A * * W N K G F G L Q G A Q A * L S N G * S A V I G G D W R R K F C I N L S R T N A Q D G E R T A N P P E Q Y H D N T C P H   F1
      K C E V K R D D G T R D L V F K E P K P N S V M V N R P L L E A I G D E N S A S T S A V L M R K M E K E R L I L Q N S I M T I H A R T   F2
       N V R S S V M M E Q G I W S S R S P S L T Q * W L I G R Y W R R L A T K I L H Q P Q P Y * C A R W R K N G * S S R T V S * Q Y M P A H  F3
2801 AAATGTGAGGTCAAGCGTGATGATGGAACAAGGGATTTGGTCTTCAAGGAGCCCAAGCCTAACTCAGTAATGGTTAATCGGCCGTTATTGGAGGCGATTGGCGACGAAAATTCTGCATCAACCTCAGCCGTACTAATGCGCAAGATGGAGAAAGAACGGCTAATCCTCCAGAACAGTATCATGACAATACATGCCCGCAC 3000
     ----:----|----:----|----:----|----:----|----:----|----:----|----:----|----:----|----:----|----:----|----:----|----:----|----:----|----:----|----:----|----:----|----:----|----:----|----:----|----:----|
2801 TTTACACTCCAGTTCGCACTACTACCTTGTTCCCTAAACCAGAAGTTCCTCGGGTTCGGATTGAGTCATTACCAATTAGCCGGCAATAACCTCCGCTAACCGCTGCTTTTAAGACGTAGTTGGAGTCGGCATGATTACGCGTTCTACCTCTTTCTTGCCGATTAGGAGGTCTTGTCATAGTACTGTTATGTACGGGCGTG 3000
      S I H P * A H H H F L P N P R * P A W A * S L L P * D A T I P P S Q R R F N Q M L R L R V L A C S P S L V A L G G S C Y * S L V H G C   F6
       F T L D L T I I S C P I Q D E L L G L R V * Y H N I P R * Q L R N A V F I R C * G * G Y * H A L H L F F P * D E L V T D H C Y M G A    F5
      F H S T L R S S P V L S K T K L S G L G L E T I T L R G N N S A I P S S F E A D V E A T S I R L I S F S R S I R W F L I M V I C A R V   F4

       T H * T T S T H H I Q * H D R * K A C T I I R G A T G K W V T L H M H A L P R Y K K I S K D T A R I I Q N * Q N T H R N T T N I N I H   F1
        H T R L H R L T I Y N S M I D E K L A R S S G G L Q G S G S H F I C T L C H A T K K S A K T Q L G S F K I D R T L T E T Q Q T S T Y I  F2
        T L D Y I D S P Y T I A * * M K S L H D H Q G G Y R E V G H T S Y A R S A T L Q K N Q Q R H S * D H S K L T E H S Q K H N K H Q H T    F3
3001 ACACACTAGACTACATCGACTCACCATATACAATAGCATGATAGATGAAAAGCTTGCACGATCATCAGGGGGGCTACAGGGAAGTGGGTCACACTTCATATGCACGCTCTGCCACGCTACAAAAAAATCAGCAAAGACACAGCTAGGATCATTCAAAATTGACAGAACACTCACAGAAACACAACAAACATCAACATACA 3200
     ----:----|----:----|----:----|----:----|----:----|----:----|----:----|----:----|----:----|----:----|----:----|----:----|----:----|----:----|----:----|----:----|----:----|----:----|----:----|----:----|
3001 TGTGTGATCTGATGTAGCTGAGTGGTATATGTTATCGTACTATCTACTTTTCGAACGTGCTAGTAGTCCCCCCGATGTCCCTTCACCCAGTGTGAAGTATACGTGCGAGACGGTGCGATGTTTTTTTAGTCGTTTCTGTGTCGATCCTAGTAAGTTTTAACTGTCTTGTGAGTGTCTTTGTGTTGTTTGTAGTTGTATGT 3200
       V C * V V D V * W I C Y C S L H F A Q V I M L P A V P F H T V S * I C A R G R * L F I L L S V A L I M * F Q C F V * L F V V F M L M C   F6
      C V S S * M S E G Y V I A H Y I F L K C S * * P P * L S T P * V E Y A R E A V S C F F * C L C L * S * E F N V S C E C F C L L C * C V    F5
       C V L S C R S V M Y L L M I S S F S A R D D P P S C P L P D C K M H V S Q W A V F F D A F V C S P D N L I S L V S V S V C C V D V Y    F4

       H N * S * Q S H T R * T G H G S W R R E T Q T A S H I R T K A A I D G C D P C * H Q S R S V L Q E D H C E G N S R C A * M G G L R K C   F1
        T T N P D N L T P D E L A T E A G G V K R K P L L T S E P K Q Q L M D A T H A D I S L G Q F F K K I I V R E I A G V H K W E A S E N  F2
       S Q L I L T I S H P M N W P R K L A A * N A N R F S H P N Q S S N * W M R P M L T S V S V S S S R R S L * G K * Q V C I N G R P P K M  F3
3201 TCACAACTAATCCTGACAATCTCACACCCGATGAACTGGCCACGGAAGCTGGCGGCGTGAAACGCAAACCGCTTCTCACATCCGAACCAAAGCAGCAATTGATGGATGCGACCCATGCTGACATCAGTCTCGGTCAGTTCTTCAAGAAGATCATTGTGAGGGAAATAGCAGGTGTGCATAAATGGGAGGCCTCCGAAAAT 3400
     ----:----|----:----|----:----|----:----|----:----|----:----|----:----|----:----|----:----|----:----|----:----|----:----|----:----|----:----|----:----|----:----|----:----|----:----|----:----|----:----|
3201 AGTGTTGATTAGGACTGTTAGAGTGTGGGCTACTTGACCGGTGCCTTCGACCGCCGCACTTTGCGTTTGGCGAAGAGTGTAGGCTTGGTTTCGTCGTTAACTACCTACGCTGGGTACGACTGTAGTCAGAGCCAGTCAAGAAGTTCTTCTAGTAACACTCCCTTTATCGTCCACACGTATTTACCCTCCGGAGGCTTTTA 3400
       * L * D Q C D * V R H V P W P L Q R R S V C V A E * M R V L A A I S P H S G H Q C * D R D T R * S S * Q S P F L L H A Y I P P R R F   F6
      D C S I R V I E C G I F Q G R F S A A H F A F R K E C G F W L L L Q H I R G M S V D T E T L E E L L D N H P F Y C T H M F P L G G F I   F5
      M V V L G S L R V G S S S A V S A P P T F R L G S R V D S G F C C N I S A V W A S M L R P * N K L F I M T L S I A P T C L H S A E S F   F4

       * A V H C R C R E T S R Y S R E R V T G Y S A K H H D A R K L C * G P L * R K E * G Y F L G T D * E * G K K G T T S L S F A A V S G   F1
      V K Q Y I V D A E R R L D I H V R E L L G T A P S I M M P G N Y A R A L F K E K N E D I F L E L I R N E E R K A L L R S V L Q Q F R A   F2
       L S S T L * M Q R D V S I F T * E S Y W V Q R Q A S * C Q E I M L G P S L K K R M R I F S W N * L G M R K E R H Y F A Q F C S S F G H  F3
3401 GTTAAGCAGTACATTGTAGATGCAGAGAGACGTCTCGATATTCACGTGAGAGAGTTACTGGGTACAGCGCCAAGCATCATGATGCCAGGAAATTATGCTAGGGCCCTCTTTAAAGAAAAGAATGAGGATATTTTCTTGGAACTGATTAGGAATGAGGAAAGAAAGGCACTACTTCGCTCAGTTTTGCAGCAGTTTCGGGC 3600
     ----:----|----:----|----:----|----:----|----:----|----:----|----:----|----:----|----:----|----:----|----:----|----:----|----:----|----:----|----:----|----:----|----:----|----:----|----:----|----:----|
3401 CAATTCGTCATGTAACATCTACGTCTCTCTGCAGAGCTATAAGTGCACTCTCTCAATGACCCATGTCGCGGTTCGTAGTACTACGGTCCTTTAATACGATCCCGGGAGAAATTTCTTTTCTTACTCCTATAAAAGAACCTTGACTAATCCTTACTCCTTTCTTTCCGTGATGAAGCGAGTCAAAACGTCGTCAAAGCCCG 3600
      H * A T C Q L H L S V D R Y E R S L T V P Y L A L C * S A L F N H * P G R * L F S H P Y K R P V S * S H P F F P V V E S L K A A T E P   F6
       N L L V N Y I C L S T E I N V H S L * Q T C R W A D H H W S I I S P G E K F F L I L I N E Q F Q N P I L F S L C * K A * N Q L L K P    F5
       T L C Y M T S A S L R R S I * T L S N S P V A G L M M I G P F * A L A R K L S F F S S I K K S S I L F S S L F A S S R E T K C C N R A   F4

      I E E G L S R A P T K Q E R G T G V * E E G C A D G K G A P R A L * I C V L A Q L P S Q D L G T H P R I D P L G G W A R K H W N L E W   F1
       L R K V Y R E H Q P N K K E V Q G F K K K A V Q M G R E L L E H F E Y V C W P N Y L H K I L E H T Q E S I L S E D G P G S I G I L S G  F2
       * G R S I E S T N Q T R K R Y R G L R R R L C R W E G S S * S T L N M C A G P T T F T R S W N T P K N R S S R R M G Q E A L E S * V   F3
3601 ATTGAGGAAGGTCTATCGAGAGCACCAACCAAACAAGAAAGAGGTACAGGGGTTTAAGAAGAAGGCTGTGCAGATGGGAAGGGAGCTCCTAGAGCACTTTGAATATGTGTGCTGGCCCAACTACCTTCACAAGATCTTGGAACACACCCAAGAATCGATCCTCTCGGAGGATGGGCCAGGAAGCATTGGAATCTTGAGTG 3800
     ----:----|----:----|----:----|----:----|----:----|----:----|----:----|----:----|----:----|----:----|----:----|----:----|----:----|----:----|----:----|----:----|----:----|----:----|----:----|----:----|
3601 TAACTCCTTCCAGATAGCTCTCGTGGTTGGTTTGTTCTTTCTCCATGTCCCCAAATTCTTCTTCCGACACGTCTACCCTTCCCTCGAGGATCTCGTGAAACTTATACACACGACCGGGTTGATGGAAGTGTTCTAGAACCTTGTGTGGGTTCTTAGCTAGGAGAGCCTCCTACCCGGTCCTTCGTAACCTTAGAACTCAC 3800
       M S S P R D L A G V L C S L P V P T * S S P Q A S P F P A G L A S Q I H T S A W S G E C S R P V C G L I S G R P P H A L F C Q F R S H   F6
      C Q P L D I S L V L W V L F L Y L P K L L L S H L H S P L E * L V K F I H A P G V V K V L D Q F V G L F R D E R L I P W S A N S D Q T    F5
       N L F T * R S C W G F L F S T C P N L F F A T C I P L S S R S C K S Y T H Q G L * R * L I K S C V W S D I R E S S P G P L M P I K L   F4

       * R Q R S G Q Q A F S Q A S E Q L L S * G R C V G W A K G H P V V S L A V Y K S E T S T T S R C N T A H V H V L Q M W C R R T Q H K V   F1
        E G S E A A N K L F R K L R N N F S R R G D V W D G L R D I L W F H W L Y T S P K L L R L R D V T R R T Y T C S R C G A E G H N I K  F2
      V K A A K R P T S F F A S F G T T S L V G E M C G M G * G T S C G F T G C I Q V R N F Y D F E M * H G A R T R A P D V V Q K D T T * S   F3
3801 GTGAAGGCAGCGAAGCGGCCAACAAGCTTTTTCGCAAGCTTCGGAACAACTTCTCTCGTAGGGGAGATGTGTGGGATGGGCTAAGGGACATCCTGTGGTTTCACTGGCTGTATACAAGTCCGAAACTTCTACGACTTCGAGATGTAACACGGCGCACGTACACGTGCTCCAGATGTGGTGCAGAAGGACACAACATAAAG 4000
     ----:----|----:----|----:----|----:----|----:----|----:----|----:----|----:----|----:----|----:----|----:----|----:----|----:----|----:----|----:----|----:----|----:----|----:----|----:----|----:----|
3801 CACTTCCGTCGCTTCGCCGGTTGTTCGAAAAAGCGTTCGAAGCCTTGTTGAAGAGAGCATCCCCTCTACACACCCTACCCGATTCCCTGTAGGACACCAAAGTGACCGACATATGTTCAGGCTTTGAAGATGCTGAAGCTCTACATTGTGCCGCGTGCATGTGCACGAGGTCTACACCACGTCTTCCTGTGTTGTATTTC 4000
        H L C R L P W C A K E C A E S C S R E Y P L H T P H A L P C G T T E S A T Y L D S V E V V E L H L V A C T C T S W I H H L L V C C L   F6
       T F A A F R G V L K K A L K P V V E R T P S I H P I P * P V D Q P K V P Q I C T R F K * S K S I Y C P A R V R A G S T T C F S V V Y L   F5
      P S P L S A A L L S K R L S R F L K E R L P S T H S P S L S M R H N * Q S Y V L G F S R R S R S T V R R V Y V H E L H P A S P C L M F   F4

        L * C E G N I K E C M K K * L F S N R F C L I P E F T I F N R S T D V S * R A C E K G I K Q Y M I Q F Y I V M I I I I F Y I V F I I    F1
      S C N V K A T * R N A * K N N S S P I D S V * F Q N L R F S I D L Q M S P S E L A K R A S S N I * F N F T L S * L L L S S T L C S S *   F2
       P V M * R Q H K G M H E K I T L L Q S I L F N S R I Y D F Q * I Y R C L L A S L R K G H Q A I Y D S I L H C H D Y Y Y L L H C V H H N  F3
4001 TCCTGTAATGTGAAGGCAACATAAAGGAATGCATGAAAAAATAACTCTTCTCCAATCGATTCTGTTTAATTCCAGAATTTACGATTTTCAATAGATCTACAGATGTCTCCTAGCGAGCTTGCGAAAAGGGCATCAAGCAATATATGATTCAATTTTACATTGTCATGATTATTATTATCTTCTACATTGTGTTCATCATA 4200
     ----:----|----:----|----:----|----:----|----:----|----:----|----:----|----:----|----:----|----:----|----:----|----:----|----:----|----:----|----:----|----:----|----:----|----:----|----:----|----:----|
4001 AGGACATTACACTTCCGTTGTATTTCCTTACGTACTTTTTTATTGAGAAGAGGTTAGCTAAGACAAATTAAGGTCTTAAATGCTAAAAGTTATCTAGATGTCTACAGAGGATCGCTCGAACGCTTTTCCCGTAGTTCGTTATATACTAAGTTAAAATGTAACAGTACTAATAATAATAGAAGATGTAACACAAGTAGTAT 4200
      T R Y H S P L M F S H M F F Y S K E L R N Q K I G S N V I K L L D V S T E * R A Q S F P M L C Y I I * N * M T M I I I I K * M T N M M   F6
       G T I H L C C L P I C S F I V R R W D I R N L E L I * S K * Y I * L H R R A L K R F P C * A I Y S E I K C Q * S * * * R R C Q T * *   F5
       D Q L T F A V Y L F A H F F L E E G I S E T * N W F K R N E I S R C I D G L S S A F L A D L L I H N L K V N D H N N N D E V N H E D Y  F4
```

Matching transcriptomic data (TSA)

```
>WGS: BEXV01002133.1:6400-9700
>TSA: IACU01059282.1:beg-end
>TSA: IACU01059285.1:beg-end
```

```
              1                                                                                                                                                                                                     200
BEXV01002133  CCCCAACTGTCACAGCCCCTTTAACCCCAACTGCCACAGCCCCTTTAACCCCAACTGCCACAGCCCCTTTAACCCCAACTGCCACAGCCCCTTTAACCCCAACTGCCACAGACCCTTTAACCCCAACTGCCACAAGGTATCGGCCAATCGTGACCAAGGACCGGGCTCATTTCACACGTGCTCTATTCTCCCCTGTCCTA
IACU01059282                                                                                          CCCTTTAACCCCAACTGCCACAGACCCTTTAACCCCAACTGCCACAAGGTATCGGCCAATCGTGACCAAGGACCGGGCTCATTTCACACGTGCTCTATTCTCCCCTGTCCTA
IACU01059285                                                                                                                                                                                   TTTCTCACGTGCTCTATTCTCCCCTGTCCTA
 Consensus    ........................................................................................ccctttaaccccaactgccacagaccctttaaccccaactgccacaaggtatcggccaatcgtgaccaaggaccgggctcaTTTCaCACGTGCTCTATTCTCCCCTGTCCTA

              201                                                                                                                                                                                                   400
BEXV01002133  ACTGTGCCTGCAAGGAAGTCCCCTGTCAGAGCAAAAGGCAGTCTCCACTATGTGCGGAGAGATTGCGCAAAGAACAGAGCCAAAGGAGCCCTGGACTTCATGACCTCACACAGCGCTGCCAAAAACGAGGACGAGACAGACCTCTGGTTCTTCGGACTTCACAACAGGTTGAGGAATGAAAAAGATGAGCGAGCAAAAAT
IACU01059282  ACTGTGCCTGCAAGGAAGTCCCCTGTCAGAGCAAAAGGCAGTCTCCACTATGTGCGGAGAGATTGCGCAAAGAACAGAGCCAAAGGAGCCCTGGACTTCATGACCTCACACAGCGCTGCCAAAAACGAGGACGAGACAGACCTCTGGTTCTTCGGACTTCACAACAGGTTGAGGAATGAAAAAGATGAGCGAGCAAAAAT
IACU01059285  ACTGTGCCTGCAAGGAAGTCCCCTGTCAGAGCAAAAGGCAGTCTCCACTATGTGCGGAGAGATTGCGCAAAGAACAGAGCCAAAGGAGCCCTGGACTTCATGACCTCACACAGCGCTGCCAAAAACGAGGACGAGACAGACCTCTGGTTCTTCGGACTTCACAACAGGTTGAGGAATGAAAAAGATGAGCGAGCAAAAAT
 Consensus    ACTGTGCCTGCAAGGAAGTCCCCTGTCAGAGCAAAAGGCAGTCTCCACTATGTGCGGAGAGATTGCGCAAAGAACAGAGCCAAAGGAGCCCTGGACTTCATGACCTCACACAGCGCTGCCAAAAACGAGGACGAGACAGACCTCTGGTTCTTCGGACTTCACAACAGGTTGAGGAATGAAAAAGATGAGCGAGCAAAAAT

              401                                                                                                                                                                                                   600
BEXV01002133  GGTAAGTAATTAACAATATGGTTTGATGAGTTCTCTGTAGTCATACGCTAGTCTTATTCAGGAAAAATGAAAATGCTAAATTTGATAGTCTAATGATTTTCGAGCTATGAAGAAACATTTTTGAATCAAGTATTTCAAAGGGCAAGTCCAAGATATTTGTACCTGAGATGAATATTTTTCATCTTTTAACTGGGGTCTCA
IACU01059282  GGTAA---------------------------------------------------------------------------------------------------------------------------------------------------------------------------------------------------
IACU01059285  GGTAA---------------------------------------------------------------------------------------------------------------------------------------------------------------------------------------------------
 Consensus    GGTAA...................................................................................................................................................................................................

              601                                                                                                                                                                                                   800
BEXV01002133  AATATCACTAGTACTTGAATTAAAAGTCATGCAGCTGAGATAAATTCCCTAAATATATATGTGCTACATGTATATTCATCTTTTGTTGTATTCTTTTTCACCTATTTAGGTAATGGAGCTTTGGACAGAAAGGAAGAATACAACTGATTTGTCTATAGATGACTGCCTGGCGATGCGCGTGGGGACTCTTTGCACCAAAG
IACU01059282  -----------------------------------------------------------------------------------------------------------------TGGAGCTTTGGACAGAAAGGAAGAATACAACTGATTTGTCTATAGATGACTGCCTGGCGATGCGCGTGGGGACTCTTTGCACCAAAG
IACU01059285  -----------------------------------------------------------------------------------------------------------------TGGAGCTTTGGACAGAAAGGAAGAATACAACTGATTTGTCTATAGATGACTGCCTGGCGATGCGCGTGGGGACTCTTTGCACCAAAG
 Consensus    .................................................................................................................TGGAGCTTTGGACAGAAAGGAAGAATACAACTGATTTGTCTATAGATGACTGCCTGGCGATGCGCGTGGGGACTCTTTGCACCAAAG

              801                                                                                                                                                                                                   1000
BEXV01002133  GGATGTATGCAGAGAAATACCGCTTTTTGAAGTCAAAAGGGGACACGACCTTCAAACCACCTAGTCAGCTCACCAACAGGGAAAGCTATTACATGCCTGGCAATGTGAGGTTCGGTCTGATGGATGGAGGGAAATGTGTCTATCACACTCCTGAGAAATCACTGGAAGAGTTTGATGACAATTCGATGTATGAGCCCATC
IACU01059282  GGATGTATGCAGAGAAATACCGCTTTTTGAAGTCAAAAGGGGACACGACCTTCAAACCACCTAGTCAGCTCACCAACAGGGAAAGCTATTACATGCCTGGCAATGTGAGGTTCGGTCTGATGGATGGAGGGAAATGTGTCTATCACACTCCTGAGAAATCACTGGAAGAGTTTGATGACAATTCGATGTATGAGCCCATC
IACU01059285  GGATGTATGCAGAGAAATACCGCTTTTTGAAGTCAAAAGGGGACACGACCTTCAAACCACCTAGTCAGCTCACCAACAGGGAAAGCTATTACATGCCTGGCAATGTGAGGTTCGGTCTGATGGATGGAGGGAAATGTGTCTATCACACTCCTGAGAAATCACTGGAAGAGTTTGATGACAATTCGATGTATGAGCCCATC
 Consensus    GGATGTATGCAGAGAAATACCGCTTTTTGAAGTCAAAAGGGGACACGACCTTCAAACCACCTAGTCAGCTCACCAACAGGGAAAGCTATTACATGCCTGGCAATGTGAGGTTCGGTCTGATGGATGGAGGGAAATGTGTCTATCACACTCCTGAGAAATCACTGGAAGAGTTTGATGACAATTCGATGTATGAGCCCATC

              1001                                                                                                                                                                                                  1200
BEXV01002133  CGGATCAACGTGCGCAGCAAACTGACTGAATTTGCACTGCCAAACTGTATTGGCGTCGCATGGAGCTACCCAGAGGCAGTTGCAAAAACCCTGGAAGAGCTAGATGAAAACATCCGAGAAGGAATGCTTAAAGTCGGCCTCAACCCTGATGACCCATCCATCATCATTGACACCACATTGAAAGATGGGGCAGATGGAAT
```

```
IACU01059282  CGGATCAACGTGCGCAGCAAACTGACTGAATTTGCACTGCCAAACTGTATTGGCGTCGCATGGAGCTACCCAGAGGCAGTTGCAAAAACCCTGGAAGAGCTAGATGAAAACATCCGAGAAGGAATGCTTAAAGTCGGCCTCAACCCTGATGACCCATCCATCATCATTGACACCACATTGAAAGATGGGGCAGATGGAAT
IACU01059285  CGGATCAACGTGCGCAGCAAACTGACTGAATTTGCACTGCCAAACTGTATTGGCGTCGCATGGAGCTACCCAGAGGCAGTTGCAAAAACCCTGGAAGAGCTAGATGAAAACATCCGAGAAGGAATGCTTAAAGTCGGCCTCAACCCTGATGACCCATCCATCATCATTGACACCACATTGAAAGATGGGGCAGATGGAAT
 Consensus    CGGATCAACGTGCGCAGCAAACTGACTGAATTTGCACTGCCAAACTGTATTGGCGTCGCATGGAGCTACCCAGAGGCAGTTGCAAAAACCCTGGAAGAGCTAGATGAAAACATCCGAGAAGGAATGCTTAAAGTCGGCCTCAACCCTGATGACCCATCCATCATCATTGACACCACATTGAAAGATGGGGCAGATGGAAT

              1201                                                                                                                                                                                                1400
BEXV01002133  GGGGGAGATCGCAGTCCACAAGATGAAGTCAGACAAATTCCTGCCTGACAAGGCATTTCGGGCTTCATTTGTAGTCTTGAAATGTGAGGTCAAGCGTGATGATGGAACAAGGGATTTGGTCTTCAAGGAGCCCAAGCCTAACTCAGTAATGGTTAATCGGCCGTTATTGGAGGCGATTGGCGACGAAAATTCTGCATCAA
IACU01059282  GGGGGAGATCGCAGTCCACAAGATGAAGTCAGACAAATTCCTGCCTGACAAGGCATTTCGGGCTTCATTTGTAGTCTTGAAATGTGAGGTCAAGCGTGATGATGGAACAAGGGATTTGGTCTTCAAGGAGCCCAAGCCTAACTCAGTAATGGTTAATCGGCCGTTATTGGAGGCGATTGGCGACGAAAATTCTGCATCAA
IACU01059285  GGGGGAGATCGCAGTCCACAAGATGAAGTCAGACAAATTCCTGCCTGACAAGGCATTTCGGGCTTCATTTGTAGTCTTGAAATGTGAGGTCAAGCGTGATGATGGAACAAGGGATTTGGTCTTCAAGGAGCCCAAGCCTAACTCAGTAATGGTTAATCGGCCGTTATTGGAGGCGATTGGCGACGAAAATTCTGCATCAA
 Consensus    GGGGGAGATCGCAGTCCACAAGATGAAGTCAGACAAATTCCTGCCTGACAAGGCATTTCGGGCTTCATTTGTAGTCTTGAAATGTGAGGTCAAGCGTGATGATGGAACAAGGGATTTGGTCTTCAAGGAGCCCAAGCCTAACTCAGTAATGGTTAATCGGCCGTTATTGGAGGCGATTGGCGACGAAAATTCTGCATCAA

              1401                                                                                                                                                                                                1600
BEXV01002133  CCTCAGCCGTACTAATGCGCAAGATGGAGAAAGAACGGCTAATCCTCCAGAACAGTATCATGACAATACATGCCCGCACACACACTAGACTACATCGACTCACCATATACAATAGCATGATAGATGAAAAGCTTGCACGATCATCAGGGGGGCTACAGGGAAGTGGGTCACACTTCATATGCACGCTCTGCCACGCTACA
IACU01059282  CCTCAGCCGTACTAATGCGCAAGATGGAGAAAGAACGGCTAATCCTCCAGAACAGTATCATGACAATACATGCCCGCACACACACTAGACTACATCGACTCACCATATACAATAGCATGATAGATGAAAAGCTTGCACGATCATCAGGGGGGCTACAGGGAAGTGGGTCACACTTCATATGCACGCTCTGCCACGCTACA
IACU01059285  CCTCAGCCGTACTAATGCGCAAGATGGAGAAAGAACGGCTAATCCTCCAGAACAGTATCATGACAATACATGCCCGCACACACACTAGACTACATCGACTCACCATATACAATAGCATGATAGATGAAAAGCTTGCACGATCATCAGGGGGGCTACAGGGAAGTGGGTCACACTTCATATGCACGCTCTGCCACGCTACA
 Consensus    CCTCAGCCGTACTAATGCGCAAGATGGAGAAAGAACGGCTAATCCTCCAGAACAGTATCATGACAATACATGCCCGCACACACACTAGACTACATCGACTCACCATATACAATAGCATGATAGATGAAAAGCTTGCACGATCATCAGGGGGGCTACAGGGAAGTGGGTCACACTTCATATGCACGCTCTGCCACGCTACA

              1601                                                                                                                                                                                                1800
BEXV01002133  AAAAAATCAGCAAAGACACAGCTAGGATCATTCAAAATTGACAGAACACTCACAGAAACACAACAAACATCAACATACATCACAACTAATCCTGACAATCTCACACCCGATGAACTGGCCACGGAAGCTGGCGGCGTGAAACGCAAACCGCTTCTCACATCCGAACCAAAGCAGCAATTGATGGATGCGACCCATGCTGA
IACU01059282  AAAACATCAGCAAAGACACAGCTAGGATCATTCAAAATTGACAGAACACTCACAGAAACACAACAAACATCAACATACATCACAACTAATCCTGACAATCTCACACCCGATGAACTGGCCACGGAAGCTGGCGGCGTGAAACGCAAACCGCTTCTCACATCCGAACCAAAGCAGCAATTGATGGATGCGACCCATGCTGA
IACU01059285  AAAACATCAGCAAAGACACAGCTAGGATCATTCAAAATTGACAGAACACTCACAGAAACACAACAAACATCAACATACATCACAACTAATCCTGACAATCTCACACCCGATGAACTGGCCACGGAAGCTGGCGGCGTGAAACGCAAACCGCTTCTCACATCCGAACCAAAGCAGCAATTGATGGATGCGACCCATGCTGA
 Consensus    AAAAcATCAGCAAAGACACAGCTAGGATCATTCAAAATTGACAGAACACTCACAGAAACACAACAAACATCAACATACATCACAACTAATCCTGACAATCTCACACCCGATGAACTGGCCACGGAAGCTGGCGGCGTGAAACGCAAACCGCTTCTCACATCCGAACCAAAGCAGCAATTGATGGATGCGACCCATGCTGA

              1801                                                                                                                                                                                                2000
BEXV01002133  CATCAGTCTCGGTCAGTTCTTCAAGAAGATCATTGTGAGGGAAATAGCAGGTGTGCATAAATGGGAGGCCTCCGAAAATGTTAAGCAGTACATTGTAGATGCAGAGAGACGTCTCGATATTCACGTGAGAGAGTTACTGGGTACAGCGCCAAGCATCATGATGCCAGGAAATTATGCTAGGGCCCTCTTTAAAGAAAAGA
IACU01059282  CATCAATCTCGGTCAGTTCTTCAAGAAGATCATTGTGAGGGAAATAGCAGGTGTGCATAAATGGGAGGCCTCCGAAAATGTTAAGCAGTACATTGTAGATGCAGAGAGACGTCTCGATATTCACGTGAGAGAGTTACTGGGTACAGCGCCAAGCCTCATGATGCCAGGAAATTATGCTAGGGCCCTCTTTAAAGAAAAGA
IACU01059285  CATCAATCTCGGTCAGTTCTTCAAGAAGATCATTGTGAGGGAAATAGCAGGTGTGCATAAATGGGAGGCCTCCGAAAATGTTAAGCAGTACATTGTAGATGCAGAGAGACGTCTCGATATTCACGTGAGAGAGTTACTGGGTACAGCGCCAAGCCTCATGATGCCAGGAAATTATGCTAGGGCCCTCTTTAAAGAAAAGA
 Consensus    CATCAaTCTCGGTCAGTTCTTCAAGAAGATCATTGTGAGGGAAATAGCAGGTGTGCATAAATGGGAGGCCTCCGAAAATGTTAAGCAGTACATTGTAGATGCAGAGAGACGTCTCGATATTCACGTGAGAGAGTTACTGGGTACAGCGCCAAGCcTCATGATGCCAGGAAATTATGCTAGGGCCCTCTTTAAAGAAAAGA

              2001                                                                                                                                                                                                2200
BEXV01002133  ATGAGGATATTTTCTTGGAACTGATTAGGAATGAGGAAAGAAAGGCACTACTTCGCTCAGTTTTGCAGCAGTTTCGGGCATTGAGGAAGGTCTATCGAGAGCACCAACCAAACAAGAAAGAGGTACAGGGGTTTAAGAAGAAGGCTGTGCAGATGGGAAGGGAGCTCCTAGAGCACTTTGAATATGTGTGCTGGCCCAAC
IACU01059282  ATGAGGATATTTTCTTGGAACTGATTAGGAATGAGGAAAGAAAGGCACTACTTCGCTCAGTTTTGCAGCAGTTTCGGGCATTGAGGAAGGTCTATCGAGAGCACCAACCAAACAAGAAAGAGGTACAGGGGTTTAAGAAGAAGGCTGTGCAGATGGGAAGGGAGCTCCTAGAGCACTTTGAATATGTGTGCTGGCCCAAC
IACU01059285  ATGAGGATATTTTCTTGGAACTGATTAGGAATGAGGAAAGAAAGGCACTACTTCGCTCAGTTTTGCAGCAGTTTCGGGCATTGAGGAAGGTCTATCGAGAGCACCAACCAAACAAGAAAGAGGTACAGGGGTTTAAGAAGAAGGCTGTGCAGATGGGAAGGGAGCTCCTAGAGCACTTTGAATATGTGTGCTGGCCCAAC
 Consensus    ATGAGGATATTTTCTTGGAACTGATTAGGAATGAGGAAAGAAAGGCACTACTTCGCTCAGTTTTGCAGCAGTTTCGGGCATTGAGGAAGGTCTATCGAGAGCACCAACCAAACAAGAAAGAGGTACAGGGGTTTAAGAAGAAGGCTGTGCAGATGGGAAGGGAGCTCCTAGAGCACTTTGAATATGTGTGCTGGCCCAAC

              2201                                                                                                                                                                                                2400
BEXV01002133  TACCTTCACAAGATCTTGGAACACACCCAAGAATCGATCCTCTCGGAGGATGGGCCAGGAAGCATTGGAATCTTGAGTGGTGAAGGCAGCGAAGCGGCCAACAAGCTTTTTCGCAAGCTTCGGAACAACTTCTCTCGTAGGGGAGATGTGTGGGATGGGCTAAGGGACATCCTGTGGTTTCACTGGCTGTATACAAGTCC
IACU01059282  TACCTTCACAAGATCTTGGAACACACCCAAGAATCGATCCTCTCGGAGGATGGGCCAGGAAGCATTGGAATCTTGAGTGGTGAAGGCAGCGAAGCGGCCAACAAGCTTTTTCGCAAGCTTCGGAACAACTTCTCTCGTAGGGGAGATGTGTGGGATGGGCTAAGGGACATCCTGTGGTTTCACTGGCTGTATACAAGTCC
IACU01059285  TACCTTCACAAGATCTTGGAACACACCCAAGAATCGATCCTCTCGGAGGATGGGCCAGGAAGCATTGGAATCTTGAGTGGTGAAGGCAGCGAAGCGGCCAACAAGCTTTTTCGCAAGCTTCGGAACAACTTCTCTCGTAGGGGAGATGTGTGGGATGGGCTAAGGGACATCCTGTGGTTTCACTGGCTGTATACAAGTCC
 Consensus    TACCTTCACAAGATCTTGGAACACACCCAAGAATCGATCCTCTCGGAGGATGGGCCAGGAAGCATTGGAATCTTGAGTGGTGAAGGCAGCGAAGCGGCCAACAAGCTTTTTCGCAAGCTTCGGAACAACTTCTCTCGTAGGGGAGATGTGTGGGATGGGCTAAGGGACATCCTGTGGTTTCACTGGCTGTATACAAGTCC

              2401                                                                                                                                                                                                2600
BEXV01002133  GAAACTTCTACGACTTCGAGATGTAACACGGCGCACGTACACGTGCTCCAGATGTGGTGCAGAAGGACACAACATAAAGTCCTGTAATGTGAAGGCAACATAAAGGAATGCATGAAAAAATAACTCTTCTCCAATCGATTCTGTTTAATTCCAGAATTTACGATTTTCAATAGATCTACAGATGTCTCCTAGCGAGCTTG
IACU01059282  GAAACTTCTACGACTTCGAGATGTAACACGGCGCACGTACACGTGCTCCAGATGTGGTGCAGAAGGACACAACATAAAGTCCTGTAATGTGAAGGCAACATAAAGGAATGCATGAAAAAATAACTCTTCTCCAATCGATTCTGTTTAATTCCAGAATTTACGATTTTCAATAGATCTACAGATGTCTCCTAGCGAGCTTG
IACU01059285  GAAACTTCTACGACTTCGAGATGTAACACGGCGCACGTACACGTGCTCCAGATGTGGTGCAGAAGGACACAACATAAAGTCCTGTAATGTGAAGGCAACATAAAGGAATGCATGAAAAAATAACTCTTCTCCAATCGATTCTGTTTAATTCCAGAATTTACGATTTTCAATAGATCTACAGATGTCTCCTAGCGAGCTTG
 Consensus    GAAACTTCTACGACTTCGAGATGTAACACGGCGCACGTACACGTGCTCCAGATGTGGTGCAGAAGGACACAACATAAAGTCCTGTAATGTGAAGGCAACATAAAGGAATGCATGAAAAAATAACTCTTCTCCAATCGATTCTGTTTAATTCCAGAATTTACGATTTTCAATAGATCTACAGATGTCTCCTAGCGAGCTTG

              2601                                                                                                                                                                                                2800
BEXV01002133  CGAAAAGGGCATCAAGCAATATATGATTCAATTTTACATTGTCATGATTATTATTATCTTCTACATTGTGTTCATCATAATTGCTACCGGTATTTTTGTGCGACATTTACCTTGCTGTTATCAAATAAATTGCTATTGATTACAGTTAAAGGAATAGTACAATCTGATTGAGAGGGCACTTTTTAGTGGAAAAAAATAAC
IACU01059282  CGAAAAGGGCATCAAGCAATATATGATTCAATTTTACATTGTCATGATTATTATTATCTTCTACATTGTGTTCATCATAATTGCTA------TTTTTGTGCGACATTTACCTTGCTGTTATCAAATAAATTGCTATTGATTACAGTTAAAGGAATAGTACAATCTGATTGAGAGGGCACTTTTTAGTGGAAAAAAATAAC
IACU01059285  CGAAAAGGGCATCAAGCAATATATGATTCAATTTTACATTGTCATGATTATTATTATCTTCTACATTGTGTTCATCATAATTGCTA------TTTTTGTGCGACATTTACCTTGCTGTTATCAAATAAATTGCTATTGATTACAGTTAAAGGAATAGTACAATCTGATTGAGAGGGCACTTTTTAGTGGAAAAAAATAAC
 Consensus    CGAAAAGGGCATCAAGCAATATATGATTCAATTTTACATTGTCATGATTATTATTATCTTCTACATTGTGTTCATCATAATTGCTA......TTTTTGTGCGACATTTACCTTGCTGTTATCAAATAAATTGCTATTGATTACAGTTAAAGGAATAGTACAATCTGATTGAGAGGGCACTTTTTAGTGGAAAAAAATAAC

              2801                                                                                                                                                                                                3000
BEXV01002133  CTTGATGTATGGGTTCGTTTGTAAACCTTATTCTGGCCTAGAATCAGCATTTTCAAATGTGTTTCAGGCCAATTTTGCATGATGATAAACACAAGCACATTTTCAGATATGTGTATATTGAAATGCTGAAAAAAAATTGCGTTTTGAATATGTGAGAATGAGCCAGAATCACATTTCTGAGAGATGATAAACGCAGATCT
IACU01059282  CTTGATGTATGGGTTCGTTTGTAAACCTTTTTCTGGCCTAGAATCAGCATTTTCAAATGTGTTTCAGGCCAATTTTGCATGATGATAAACACAAGCACATTTTCAGATATGTGTATATTGAAATGCTGAAAAAAAATTGCGTTTTGAATATGTGAGAATGAGCCAGAATCACATTTCTGAGAGATGATAAACGCAGATCT
IACU01059285  CTTGATGTATGGGTTCGTTTGTAAACCTTTTTCTGGCCTAGAATCAGCATTTTCAAATGTGTTTCAGGCCAATTTTGCATGATGATAAACACAAGCACATTTTCAGATATGTGTATATTGAAATGCTGAAAAAAAATTGCGTTTTGAATATGTGAGAATGAGCCAGAATCACATTTCTGAGAGATGATAAACGCAGATCT
 Consensus    CTTGATGTATGGGTTCGTTTGTAAACCTTtTTCTGGCCTAGAATCAGCATTTTCAAATGTGTTTCAGGCCAATTTTGCATGATGATAAACACAAGCACATTTTCAGATATGTGTATATTGAAATGCTGAAAAAAAATTGCGTTTTGAATATGTGAGAATGAGCCAGAATCACATTTCTGAGAGATGATAAACGCAGATCT

              3001                                                                                                                                                                                                3200
BEXV01002133  GTTTTGAAACATGTATATATATTTCGAACCTTCCCAGGCCAGTTTTCGGTTCGGAGAAATAGCCTGAGCCATGTGCCGAAGAATGTTTCAAGCGTGCCTTTGTCTTTGTCTATTGTTTATTGTTAACATACGTTGCGATAACGTGCTTCAACTATATTGACAAGTTATTGCTGTTTATTATCCTTTACCTTTACCTTTAA
IACU01059282  GTTTTGAAACATGTATATATATTTCGAACCTTCC-AGGCCAGTTTTCGGTTCGGAGAAATAGCCTGCGCCATGTGCCGAAGAATGTTTCAAGCGTGCCTTTGTCTTTGTCTATAGTTTATTGTTAACATAC
IACU01059285  GTTTTGAAACATGTATATATATTTCGAACCTTCC-AGGCCAGTTTTCGGTTCGGAGAAATAGCCTGCGCCATGTGCCGAAGAATGTTTCAAGCGTGCCTTTGTCTTTGTCTATAGTTTATTGTTAACATAC
 Consensus    GTTTTGAAACATGTATATATATTTCGAACCTTCC.AGGCCAGTTTTCGGTTCGGAGAAATAGCCTGcGCCATGTGCCGAAGAATGTTTCAAGCGTGCCTTTGTCTTTGTCTATaGTTTATTGTTAACATAC.....................................................................

              3201                                                                                                3301
BEXV01002133  TTACCATAAAATTATGTCTGTCAGTGAGAGTTCAAAGGCGAAAGGTTCAAACTGGGATGCGAATGAACTCAGGTGTTTGCTGCTCCTGTGGGCAGAACCAG
IACU01059282
IACU01059285
 Consensus    .....................................................................................................
```

>TSA: IACU01059282.1

5'3' Frame 2

PLTPTATDPLTPTATRYRPIVTKDRAHFTRALFSPVLTVPARKSPVRAKGSLHYVRRDCAKNRAKGALDFMTSHSAAKNEDETDLWFFGLHNRLRNEKDERAKMVMELWTERKNTTDLSIDDCLAMRVGTLCTKGMYAEKYRFLKSKGDTTFKPPSQLTNRESYYMPGNVRFGLMDGGKCVYHTPEKSLEEFDDNSMYEPIRINVRSKLTEFALPNCIG
VAWSYPEAVAKTLEELDENIREGMLKVGLNPDDPSIIIDTTLKDGADGMGEIAVHKMKSDKFLPDKAFRASFVVLKCEVKRDDGTRDLVFKEPKPNSVMVNRPLLEAIGDENSASTSAVLMRKMEKERLILQNSIMTIHARTHTRLHRLTIYNSMIDEKLARSSGGLQGSGSHFICTLCHATKTSAKTQLGSFKIDRTLTETQQTSTYITTNPDNLTPD
ELATEAGGVKRKPLLTSEPKQQLMDATHADINLGQFFKKIIVREIAGVHKWEASENVKQYIVDAERRLDIHVRELLGTAPSLMMPGNYARALFKEKNEDIFLELIRNEERKALLRSVLQQFRALRKVYREHQPNKKEVQGFKKKAVQMGRELLEHFEYVCWPNYLHKILEHTQESILSEDGPGSIGILSGEGSEAANKLFRKLRNNFSRRGDVWDGLRD
ILWFHWLYTSPKLLRLRDVTRRTYTCSRCGAEGHNIKSCNVKAT-RNA-KNNSSPIDSV-FQNLRFSIDLQMSPSELAKRASSNI-FNFTLS-LLLSSTLCSS-LLFLCDIYLAVIK-IAIDYS-RNSTI-LRGHFLVEKNNLDVWVRL-TFFWPRISIFKCVSGQFCMMINTSTFSDMCILKC-KKIAF-ICENEPESHF-EMINADLF-NMYIYFEP
SRPVFGSEK-PAPCAEECFKRAFVFVYSLLLTY

>TSA: IACU01059285.1

5'3' Frame 3

FSRALFSPVLTVPARKSPVRAKGSLHYVRRDCAKNRAKGALDFMTSHSAAKNEDETDLWFFGLHNRLRNEKDERAKMVMELWTERKNTTDLSIDDCLAMRVGTLCTKGMYAEKYRFLKSKGDTTFKPPSQLTNRESYYMPGNVRFGLMDGGKCVYHTPEKSLEEFDDNSMYEPIRINVRSKLTEFALPNCIGVAWSYPEAVAKTLEELDENIREGMLKV
GLNPDDPSIIIDTTLKDGADGMGEIAVHKMKSDKFLPDKAFRASFVVLKCEVKRDDGTRDLVFKEPKPNSVMVNRPLLEAIGDENSASTSAVLMRKMEKERLILQNSIMTIHARTHTRLHRLTIYNSMIDEKLARSSGGLQGSGSHFICTLCHATKTSAKTQLGSFKIDRTLTETQQTSTYITTNPDNLTPDELATEAGGVKRKPLLTSEPKQQLMDAT
HADINLGQFFKKIIVREIAGVHKWEASENVKQYIVDAERRLDIHVRELLGTAPSLMMPGNYARALFKEKNEDIFLELIRNEERKALLRSVLQQFRALRKVYREHQPNKKEVQGFKKKAVQMGRELLEHFEYVCWPNYLHKILEHTQESILSEDGPGSIGILSGEGSEAANKLFRKLRNNFSRRGDVWDGLRDILWFHWLYTSPKLLRLRDVTRRTYTCS
RCGAEGHNIKSCNVKAT-RNA-KNNSSPIDSV-FQNLRFSIDLQMSPSELAKRASSNI-FNFTLS-LLLSSTLCSS-LLFLCDIYLAVIK-IAIDYS-RNSTI-LRGHFLVEKNNLDVWVRL-TFFWPRISIFKCVSGQFCMMINTSTFSDMCILKC-KKIAF-ICENEPESHF-EMINADLF-NMYIYFEPSRPVFGSEK-PAPCAEECFKRAFVFVY
SLLLTY

Hemicentrotus pulcherrimus (Hpu)

WGS: BEXV01003119.1

21218-29856
Hemicentrotus pulcherrimus DNA, scaffold3118, whole genome shotgun sequence

```
      L  L  H  D  F  H  H  G  E  P  K  L  F  *  F  R  I  Y  S  I  T  S  G  *  G  K  *  K  E  S  H  T  Q  T  Q  S  F  R  D  G  I  R  R  T  F  P  F  *  G  A  L  F  Y  F  H  T  I  I  R  G  P  *  L  P  M  S  Y   F1
       C  F  M  T  F  T  M  A  N  L  N  Y  S  D  L  E  Y  I  P  L  Q  A  A  K  E  N  K  K  K  V  T  L  K  R  S  H  L  E  M  A  L  G  E  H  F  P  S  E  G  H  C  S  I  F  T  P  S  S  E  D  R  D  S  Q  C  R  I   F2
        A  S  *  L  S  P  W  R  T  *  I  I  L  I  *  N  I  F  H  Y  K  R  L  R  K  I  K  R  K  S  H  S  N  A  V  I  *  R  W  H  *  E  N  I  S  L  L  R  G  I  V  L  F  S  H  H  H  Q  R  T  V  T  P  N  V  V   F3
   1 TTGCTTCATGACTTTCACCATGGCGAACCTAAATTATTCTGATTTAGAATATATTCCATTACAAGCGGCTAAGGAAAATAAAAAGAAAGTCACACTCAAACGCAGTCATTTAGAGATGGCATTAGGAGAACATTTCCCTTCTGAGGGGCATTGTTCTATTTTCACACCATCATCAGAGGACCGTGACTCCCAATGTCGTA 200
     ----:----|----:----|----:----|----:----|----:----|----:----|----:----|----:----|----:----|----:----|----:----|----:----|----:----|----:----|----:----|----:----|----:----|----:----|----:----|----:----|
   1 AACGAAGTACTGAAAGTGGTACCGCTTGGATTTAATAAGACTAAATCTTATATAAGGTAATGTTCGCCGATTCCTTTTATTTTTCTTTCAGTGTGAGTTTGCGTCAGTAAATCTCTACCGTAATCCTCTTGTAAAGGGAAGACTCCCCGTAACAAGATAAAAGTGTGGTAGTAGTCTCCTGGCACTGAGGGTTACAGCAT 200
      X  S  *  S  K  *  W  P  S  G  L  N  N  Q  N  L  I  Y  E  M  V  L  P  *  P  F  Y  F  S  L  *  V  *  V  C  D  N  L  S  P  M  L  L  V  N  G  K  Q  P  A  N  N  *  K  *  V  M  M  L  P  G  H  S  G  I  D  Y   F6
     X  A  E  H  S  E  G  H  R  V  *  I  I  R  I  *  F  I  N  W  *  L  R  S  L  F  I  F  L  F  D  C  E  F  A  T  M  *  L  H  C  *  S  F  M  E  R  R  L  P  M  T  R  N  E  C  W  *  *  L  V  T  V  G  L  T  T   F5
       Q  K  M  V  K  V  M  A  F  R  F  *  E  S  K  S  Y  I  G  N  C  A  A  L  S  F  L  F  F  T  V  S  L  R  L  *  K  S  I  A  N  P  S  C  K  G  E  S  P  C  Q  E  I  K  V  G  D  D  S  S  R  S  E  W  H  R    F4

       S  H  I  W  R  G  K  I  E  R  G  E  D  L  A  G  W  G  K  H  N  R  V  P  S  K  S  *  *  G  *  R  L  Y  R  S  Q  L  V  Q  D  E  R  *  V  V  T  N  M  L  F  L  V  V  C  Y  F  C  *  A  I  I  Y  S  A  S  H   F1
        L  T  F  G  G  A  R  L  R  E  E  R  T  W  Q  D  G  A  N  I  T  E  F  L  L  K  A  D  E  D  D  V  Y  I  E  A  S  L  C  K  T  K  G  K  L  *  Q  T  C  F  S  L  S  F  V  I  F  V  R  L  L  Y  I  V  R  P    F2
      F  S  H  L  A  G  Q  D  *  E  R  R  G  P  G  R  M  G  Q  T  *  Q  S  S  F  *  K  L  M  R  M  T  S  I  *  K  P  A  C  A  R  R  K  V  S  C  D  K  H  A  F  P  C  R  L  L  F  L  L  G  Y  Y  I  *  C  V  P   F3
 201 TTCTCACATTTGGCGGGGCAAGATTGAGAGAGGAGAGGACCTGGCAGGATGGGGCAAACATAACAGAGTTCCTTCTAAAAGCTGATGAGGATGACGTCTATATAGAAGCCAGCTTGTGCAAGACGAAAGGTAAGTTGTGACAAACATGCTTTTCCTTGTCGTTTGTTATTTTTGTTAGGCTATTATATATAGTGCGTCCC 400
     ----:----|----:----|----:----|----:----|----:----|----:----|----:----|----:----|----:----|----:----|----:----|----:----|----:----|----:----|----:----|----:----|----:----|----:----|----:----|----:----|
 201 AAGAGTGTAAACCGCCCCGTTCTAACTCTCTCCTCTCCTGGACCGTCCTACCCCGTTTGTATTGTCTCAAGGAAGATTTTCGACTACTCCTACTGCAGATATATCTTCGGTCGAACACGTTCTGCTTTCCATTCAACACTGTTTGTACGAAAAGGAACAGCAAACAATAAAAACAATCCGATAATATATATCACGCAGGG 400
       E  *  M  Q  R  P  L  I  S  L  P  S  S  R  A  P  H  P  L  C  L  L  T  G  E  L  L  Q  H  P  H  R  R  Y  L  L  W  S  T  C  S  S  L  Y  T  T  T  V  F  M  S  K  R  T  T  Q  *  K  Q  *  A  I  I  Y  L  A  D    F6
      N  E  C  K  A  P  C  S  Q  S  L  L  P  G  P  L  I  P  C  V  Y  C  L  E  K  *  F  S  I  L  I  V  D  I  Y  F  G  A  Q  A  L  R  F  T  L  Q  S  L  C  A  K  G  Q  R  K  N  N  K  N  P  *  *  I  Y  H  T  G   F5
      I  R  V  N  P  P  A  L  N  L  S  S  L  V  Q  C  S  P  A  F  M  V  S  N  R  R  F  A  S  S  S  S  T  *  I  S  A  L  K  H  L  V  F  P  L  N  H  C  V  H  K  E  K  D  N  T  I  K  T  L  S  N  Y  I  T  R  G   F4

       N  K  K  K  R  N  P  E  L  S  M  F  L  S  *  L  D  F  N  F  K  E  K  I  T  F  L  E  S  S  F  S  A  L  S  *  L  *  H  L  I  Q  E  K  *  A  R  N  N  K  V  L  A  W  H  G  A  R  P  S  I  *  K  I  V  L    F1
      I  T  K  K  K  E  T  Q  N  Y  L  C  F  Y  H  N  L  I  S  I  L  K  R  K  S  H  F  *  R  V  H  F  L  P  F  H  N  Y  D  T  S  F  R  K  N  E  P  G  I  T  K  F  W  H  G  M  A  H  V  H  P  Y  K  K  *  F  Y   F2
       *  Q  K  K  K  K  P  R  I  I  Y  V  F  I  I  T  *  F  Q  F  *  R  E  N  H  I  S  R  E  F  I  F  C  P  F  I  I  M  T  P  H  S  G  K  M  S  Q  E  *  Q  S  S  G  M  A  W  R  T  S  I  H  I  K  N  S  F  T   F3
 401 ATAACAAAAAAAAAAGAAACCCAGAATTATCTATGTTTTTATCATAACTTGATTTCAATTTTAAAGAGAAAATCACATTTCTAGAGAGTTCATTTTCTGCCCTTTCATAATTATGACACCTCATTCAGGAAAAATGAGCCAGGAATAACAAAGTTCTGGCATGGCATGGCGCACGTCCATCCATATAAAAAATAGTTTTA 600
     ----:----|----:----|----:----|----:----|----:----|----:----|----:----|----:----|----:----|----:----|----:----|----:----|----:----|----:----|----:----|----:----|----:----|----:----|----:----|----:----|
 401 TATTGTTTTTTTTTTCTTTGGGTCTTAATAGATACAAAAATAGTATTGAACTAAAGTTAAAATTTCTCTTTTAGTGTAAAGATCTCTCAAGTAAAAGACGGGAAAGTATTAATACTGTGGAGTAAGTCCTTTTTACTCGGTCCTTATTGTTTCAAGACCGTACCGTACCGCGTGCAGGTAGGTATATTTTTTATCAAAAT 600
      W  L  L  F  F  L  F  G  S  N  D  I  N  K  D  Y  S  S  K  L  K  L  S  F  I  V  N  R  S  L  E  N  E  A  R  E  Y  N  H  C  R  M  *  S  F  H  A  L  F  L  L  T  R  A  H  C  P  A  R  G  D  M  Y  F  I  T  K   F6
       Y  C  F  F  F  F  G  L  I  I  *  T  K  I  M  V  Q  N  *  N  *  L  S  F  *  M  E  L  S  N  M  K  Q  G  K  M  I  I  V  G  *  E  P  F  I  L  W  S  Y  C  L  E  P  M  A  H  R  V  D  M  W  I  F  F  L  K    F5
      M  V  F  F  F  S  V  W  F  *  R  H  K  *  *  L  K  I  E  I  K  F  L  F  D  C  K  *  L  T  *  K  R  G  K  *  L  *  S  V  E  N  L  F  F  S  G  P  I  V  F  N  Q  C  P  M  A  C  T  W  G  Y  L  F  Y  N  *   F4

      L  G  F  D  Q  T  L  L  I  S  L  N  K  M  L  *  L  L  Y  N  *  L  D  *  K  T  T  S  Q  *  *  L  F  F  P  A  L  *  L  K  R  F  L  S  S  L  C  L  I  S  N  D  S  I  N  Y  Y  C  D  P  W  T  T  H  S  G  N   F1
       W  D  L  I  K  P  Y  *  F  L  *  I  K  C  Y  D  Y  F  T  I  N  L  I  K  R  R  H  P  N  D  N  F  F  F  L  R  Y  N  *  N  G  S  S  P  P  F  V  *  Y  L  T  T  V  L  T  I  I  A  I  R  G  Q  H  I  L  E  I   F2
       G  I  *  S  N  L  T  D  F  F  E  *  N  V  M  T  T  L  Q  L  T  *  L  K  D  D  I  P  M  I  T  F  F  S  C  V  I  T  K  T  V  P  L  L  P  L  F  N  I  *  R  Q  Y  *  L  L  R  S  V  D  N  T  F  W  K    F3
 601 CTGGGATTTGATCAAACCTTACTGATTTCTTTGAATAAAATGTTATGACTACTTTACAATTAACTTGATTAAAAGACGACATCCCAATGATAACTTTTTTTTCCTGCGTTATAACTAAAACGGTTCCTCTCCTCCCCTTTGTTTAATATCTAACGACAGTATTAACTATTATTGCGATCCGTGGACAACACATTCTGGAAA 800
     ----:----|----:----|----:----|----:----|----:----|----:----|----:----|----:----|----:----|----:----|----:----|----:----|----:----|----:----|----:----|----:----|----:----|----:----|----:----|----:----|
 601 GACCCTAAACTAGTTTGGAATGACTAAAGAAACTTATTTTACAATACTGATGAAATGTTAATTGAACTAATTTTCTGCTGTAGGGTTACTATTGAAAAAAAAGGACGCAATATTGATTTTGCCAAGGAGAGGAGGGAAACAAATTATAGATTGCTGTCATAATTGATAATAACGCTAGGCACCTGTTGTGTAAGACCTTT 800
       S  P  N  S  *  V  K  S  I  E  K  F  L  I  N  H  S  S  *  L  *  S  S  *  F  V  V  D  W  H  Y  S  K  K  G  A  N  Y  S  F  R  N  R  E  E  R  Q  K  I  D  L  S  L  I  L  *  *  Q  S  G  H  V  V  C  E  P  F   F6
      V  P  I  Q  D  F  R  V  S  K  K  S  Y  F  T  I  V  V  K  C  N  V  Q  N  F  S  S  M  G  I  I  V  K  K  E  Q  T  I  V  L  V  T  G  R  R  G  K  N  L  I  *  R  C  Y  *  S  N  N  R  D  T  S  L  V  N  Q  F   F5
       Q  S  K  I  L  G  *  Q  N  R  Q  I  F  H  *  S  *  K  V  I  L  K  I  L  L  R  C  G  L  S  L  K  K  K  R  R  *  L  *  F  P  E  E  G  G  K  T  *  Y  R  V  V  T  N  V  I  I  A  I  R  P  C  C  M  R  S    F4

       K  R  G  Q  Q  *  I  I  V  G  K  S  Y  G  *  *  L  M  N  I  L  Y  I  A  I  I  P  K  G  K  T  S  *  S  Q  L  V  T  L  Y  I  E  M  G  Q  L  T  Q  T  S  *  I  L  T  G  P  V  G  I  L  R  P  V  I  N  W  P   F1
       K  G  D  N  N  K  S  L  S  E  N  H  M  V  D  D  *  *  I  Y  C  I  *  L  L  S  Q  K  G  K  P  V  E  A  N  W  *  P  C  I  L  K  W  A  S  *  P  R  P  V  E  F  *  L  V  Q  *  E  F  S  D  Q  L  S  T  G    F2
      *  K  G  T  T  I  N  H  C  R  K  I  I  W  L  M  I  N  E  Y  T  V  Y  S  Y  Y  P  K  R  E  N  Q  L  K  P  T  G  N  P  V  Y  *  N  G  P  V  D  P  D  Q  L  N  S  N  W  S  S  R  N  S  Q  T  S  Y  Q  L  A   F3
 801 TAAAAGGGGACAACAATAAATCATTGTCGGAAAATCATATGGTTGATGATTAATGAATATACTGTATATAGCTATTATCCCAAAAGGGAAAACCAGTTGAAGCCAACTGGTAACCCTGTATATTGAAATGGGCCAGTTGACCCAGACCAGTTGAATTCTAACTGGTCCAGTAGGAATTCTCAGACCAGTTATCAACTGGC 1000
     ----:----|----:----|----:----|----:----|----:----|----:----|----:----|----:----|----:----|----:----|----:----|----:----|----:----|----:----|----:----|----:----|----:----|----:----|----:----|----:----|
 801 ATTTTCCCCTGTTGTTATTTAGTAACAGCCTTTTAGTATACCAACTACTAATTACTTATATGACATATATCGATAATAGGGTTTTCCCTTTTGGTCAACTTCGGTTGACCATTGGGACATATAACTTTACCCGGTCAACTGGGTCTGGTCAACTTAAGATTGACCAGGTCATCCTTAAGAGTCTGGTCAATAGTTGACCG 1000
       L  L  P  C  C  Y  I  M  T  P  F  D  Y  P  Q  H  N  I  F  I  S  Y  I  A  I  I  G  F  P  F  V  L  Q  L  W  S  T  V  R  Y  I  S  I  P  W  N  V  W  V  L  Q  I  R  V  P  G  T  P  I  R  L  G  T  I  L  Q    F6
      Y  F  P  V  V  I  F  *  Q  R  F  I  M  H  N  I  I  L  S  Y  V  T  Y  L  *  *  G  L  L  S  F  W  N  F  G  V  P  L  G  T  Y  Q  F  P  G  T  S  G  S  W  N  F  E  L  Q  D  L  L  F  E  *  V  L  *  *  S  A   F5
      I  F  P  S  L  L  L  D  N  D  S  F  *  I  T  S  S  *  H  I  Y  Q  I  Y  S  N  D  W  F  P  F  G  T  S  A  L  Q  Y  G  Q  I  N  F  H  A  L  Q  G  L  G  T  S  N  *  S  T  W  Y  S  N  E  S  W  N  D  V  P   F4

       N  G  N  M  P  T  D  Q  L  S  T  G  A  T  G  S  L  Q  T  D  L  Q  L  S  T  G  S  S  L  *  N  E  M  Q  S  H  Q  I  S  A  *  C  A  S  W  T  S  *  N  Q  L  E  P  V  E  F  S  W  F  H  F  E  S  N  C  F    F1
      Q  M  E  I  C  Q  Q  T  S  Y  Q  L  V  Q  L  E  V  Y  K  Q  T  Y  S  Y  Q  L  V  P  V  C  K  M  K  C  S  R  T  K  Y  P  L  D  V  H  H  G  P  V  R  I  N  W  N  Q  L  N  L  A  G  S  I  L  N  L  T  V  S   F2
       K  W  K  Y  A  N  R  P  V  I  N  W  C  N  W  K  F  T  N  R  P  T  V  I  N  W  F  Q  S  V  K  *  N  A  V  A  P  N  I  R  L  M  C  I  M  D  Q  L  E  S  T  G  T  S  *  I  *  L  V  P  F  *  I  *  L  F  P   F3
1001 CAAATGGAAATATGCCAACAGACCAGTTATCAACTGGTGCAACTGGAAGTTTACAAACAGACCTACAGTTATCAACTGGTTCCAGTCTGTAAAATGAAATGCAGTCGCACCAAATATCCGCTTGATGTGCATCATGGACCAGTTAGAATCAACTGGAACCAGTTGAATTTAGCTGGTTCCATTTTGAATCTAACTGTTTC 1200
     ----:----|----:----|----:----|----:----|----:----|----:----|----:----|----:----|----:----|----:----|----:----|----:----|----:----|----:----|----:----|----:----|----:----|----:----|----:----|----:----|
1001 GTTTACCTTTATACGGTTGTCTGGTCAATAGTTGACCACGTTGACCTTCAAATGTTTGTCTGGATGTCAATAGTTGACCAAGGTCAGACATTTTACTTTACGTCAGCGTGGTTTATAGGCGAACTACACGTAGTACCTGGTCAATCTTAGTTGACCTTGGTCAACTTAAATCGACCAAGGTAAAACTTAGATTGACAAAG 1200
      G  F  P  F  I  G  V  S  W  N  D  V  P  A  V  P  L  K  C  V  S  R  C  N  D  V  P  E  L  R  Y  F  S  I  C  D  C  W  I  D  A  Q  H  A  D  H  V  L  *  F  *  S  S  G  T  S  N  L  Q  N  W  K  S  D  L  Q  K   F6
       L  H  F  Y  A  L  L  G  T  I  L  Q  H  L  Q  F  N  V  F  L  G  V  T  I  L  Q  N  W  D  T  F  H  F  A  T  A  G  F  I  R  K  I  H  M  M  S  W  N  S  D  V  P  V  L  Q  I  *  S  T  G  N  Q  I  *  S  N    F5
       W  I  S  I  H  W  C  V  L  *  *  S  T  C  S  S  T  *  L  C  V  *  L  *  *  S  T  G  T  Q  L  I  F  H  L  R  V  L  Y  G  S  S  T  C  *  P  G  T  L  I  L  Q  F  W  N  F  K  A  P  E  M  K  F  R  V  T  E   F4

      Q  L  K  L  T  G  T  S  W  K  F  Q  Q  V  V  N  W  F  F  L  L  R  L  *  L  C  *  T  V  T  *  E  G  Q  *  F  T  Y  Q  Q  Y  Y  T  Y  L  N  M  I  K  A  D  R  H  Y  P  V  W  A  R  G  G  S  H  I  K  F  T   F1
       S  *  S  *  L  E  P  V  G  N  F  N  R  W  *  T  G  F  S  F  *  D  Y  D  C  V  K  L  S  H  E  R  V  S  N  L  P  I  N  N  T  I  L  T  *  I  *  *  K  L  T  G  I  I  Q  C  G  H  V  E  G  L  I  *  N  S  L   F2
       V  E  V  N  W  N  Q  L  E  I  S  T  G  G  K  L  V  F  P  S  E  I  M  T  V  L  N  C  H  M  R  G  S  V  I  Y  L  S  T  I  L  Y  L  P  K  Y  D  K  S  *  P  A  L  S  S  V  G  T  W  R  V  S  Y  K  I  H   F3
1201 CAGTTGAAGTTAACTGGAACCAGTTGGAAATTTCAACAGGTGGTAAACTGGTTTTTCCTTCTGAGATTATGACTGTGTTAAACTGTCACATGAGAGGGTCAGTAATTTACCTATCAACAATACTATACTTACCTAAATATGATAAAAGCTGACCGGCATTATCCAGTGTGGGCACGTGGAGGGTCTCATATAAAATTCAC 1400
     ----:----|----:----|----:----|----:----|----:----|----:----|----:----|----:----|----:----|----:----|----:----|----:----|----:----|----:----|----:----|----:----|----:----|----:----|----:----|----:----|
1201 GTCAACTTCAATTGACCTTGGTCAACCTTTAAAGTTGTCCACCATTTGACCAAAAAGGAAGACTCTAATACTGACACAATTTGACAGTGTACTCTCCCAGTCATTAAATGGATAGTTGTTATGATATGAATGGATTTATACTATTTTCGACTGGCCGTAATAGGTCACACCCGTGCACCTCCCAGAGTATATTTTAAGTG 1400
       W  N  F  N  V  P  V  L  Q  F  N  *  C  T  T  F  Q  N  K  R  R  L  N  H  S  H  *  V  T  V  H  S  P  *  Y  N  V  *  *  C  Y  *  V  *  R  F  I  I  F  A  S  R  C  *  G  T  H  A  R  P  P  D  *  I  F  N  V   F6
      G  T  S  T  L  Q  F  W  N  S  I  E  V  P  P  L  S  T  K  G  E  S  I  I  V  T  N  F  Q  *  M  L  P  D  T  I  *  R  D  V  I  S  Y  K  G  L  Y  S  L  L  Q  G  A  N  D  L  T  P  V  H  L  T  E  Y  L  I  *   F5
       L  Q  L  *  S  S  G  T  P  F  K  L  L  H  Y  V  P  K  E  K  Q  S  *  S  Q  T  L  S  D  C  S  L  T  L  L  K  G  I  L  L  V  I  S  V  *  I  H  Y  F  S  V  P  M  I  W  H  P  C  T  S  P  R  M  Y  F  E    F4

       C  L  K  L  S  F  F  N  K  K  *  K  I  N  T  F  K  V  G  R  R  S  V  N  Y  E  T  T  I  R  T  I  *  L  E  H  *  K  *  S  L  I  P  N  *  C  D  P  D  W  A  R  F  K  N  F  P  T  *  I  M  H  D  C  V  M  N   F1
        A  *  N  S  P  S  S  I  K  N  K  K  *  T  P  S  K  S  E  G  G  V  *  T  T  K  R  L  *  E  Q  Y  D  W  S  I  K  S  K  V  *  F  P  T  S  A  T  P  I  G  R  G  S  R  T  F  L  L  R  S  C  M  I  V  *  *   F2
      L  P  E  T  L  L  L  Q  *  K  I  K  N  K  H  L  Q  S  R  K  A  E  C  K  L  R  N  D  Y  K  N  N  M  I  G  A  L  K  V  K  F  D  S  Q  L  V  R  P  R  L  G  A  V  Q  E  L  S  Y  L  D  H  A  *  L  C  D  E   F3
1401 TTGCCTGAAACTCTCCTTCTTCAATAAAAAATAAAAAATAAACACCTTCAAAGTCGGAAGGCGGAGTGTAAACTACGAAACGACTATAAGAACAATATGATTGGAGCATTAAAAGTAAAGTTTGATTCCCAACTAGTGCGACCCCGATTGGGCGCGGTTCAAGAACTTTCCTACTTAGATCATGCATGATTGTGTGATGA 1600
     ----:----|----:----|----:----|----:----|----:----|----:----|----:----|----:----|----:----|----:----|----:----|----:----|----:----|----:----|----:----|----:----|----:----|----:----|----:----|----:----|
1401 AACGGACTTTGAGAGGAAGAAGTTATTTTTTATTTTTTATTTGTGGAAGTTTCAGCCTTCCGCCTCACATTTGATGCTTTGCTGATATTCTTGTTATACTAACCTCGTAATTTTCATTTCAAACTAAGGGTTGATCACGCTGGGGCTAACCCGCGCCAAGTTCTTGAAAGGATGAATCTAGTACGTACTAACACACTACT 1600
       Q  R  F  S  E  K  K  L  L  F  Y  F  I  F  V  K  L  T  P  L  R  L  T  F  *  S  V  V  I  L  V  I  H  N  S  C  *  F  Y  L  K  I  G  L  *  H  S  G  S  Q  A  R  N  L  F  K  G  V  *  I  M  C  S  Q  T  I    F6
       K  G  S  V  R  R  R  *  Y  F  I  F  F  L  C  R  *  L  R  F  A  S  H  L  S  R  F  S  *  L  F  L  I  I  P  A  N  F  T  F  N  S  E  W  S  T  R  G  R  N  P  A  T  *  S  S  E  *  K  S  *  A  H  N  H  S  S   F5
      S  A  Q  F  E  G  E  E  I  F  F  L  F  Y  V  G  E  F  D  S  P  P  T  Y  V  V  F  R  S  Y  S  C  Y  S  Q  L  M  L  L  L  T  Q  N  G  V  L  A  V  G  I  P  R  P  E  L  V  K  R  S  L  D  H  M  I  T  H  H   F4

       M  H  A  Q  S  I  F  Y  *  F  D  A  T  I  K  N  G  L  *  L  V  F  T  P  L  Q  H  S  L  R  Y  H  S  T  E  W  G  R  G  *  Y  L  S  S  S  V  A  Y  K  W  K  T  C  L  L  Y  F  T  *  I  Q  *  S  L  *  Q   F1
      T  C  M  R  N  Q  S  F  I  S  L  T  Q  Q  L  K  M  V  C  N  L  C  L  P  H  C  N  I  A  Y  V  T  I  L  L  N  G  G  G  G  D  I  Y  Q  A  V  L  H  I  N  G  K  L  V  C  F  I  S  H  K  Y  N  N  H  Y  S  N   F2
       H  A  C  A  I  N  L  L  L  V  *  R  N  N  *  K  W  F  V  T  C  V  Y  P  I  A  T  *  L  T  L  P  F  Y  *  M  G  E  G  V  I  F  I  K  Q  C  C  I  *  M  E  N  L  F  A  L  F  H  I  N  T  I  I  I  A  I   F3
1601 ACATGCATGCGCAATCAATCTTTTATTAGTTTGACGCAACAATTAAAAATGGTTTGTAACTTGTGTTTACCCCATTGCAACATAGCTTACGTTACCATTCTACTGAATGGGGGAGGGGGGTGATATTTATCAAGCAGTGTTGCATATAAATGGAAAACTTGTTTGCTTTATTTCACATAAATACAATAATCATTATAGCAA 1800
     ----:----|----:----|----:----|----:----|----:----|----:----|----:----|----:----|----:----|----:----|----:----|----:----|----:----|----:----|----:----|----:----|----:----|----:----|----:----|----:----|
1601 TGTACGTACGCGTTAGTTAGAAAATAATCAAACTGCGTTGTTAATTTTTACCAAACATTGAACACAAATGGGGTAACGTTGTATCGAATGCAATGGTAAGATGACTTACCCCCTCCCCCACTATAAATAGTTCGTCACAACGTATATTTACCTTTTGAACAAACGAAATAAAGTGTATTTATGTTATTAGTAATATCGTT 1800
      F  M  C  A  C  D  I  K  *  *  N  S  A  V  I  L  F  P  K  Y  S  T  N  V  G  N  C  C  L  K  R  *  W  E  V  S  H  P  L  P  H  Y  K  D  L  L  T  A  Y  L  H  F  V  Q  K  S  *  K  V  Y  I  C  Y  D  N  Y  C   F6
       C  A  H  A  I  L  R  K  N  T  Q  R  L  L  *  F  H  N  T  V  Q  T  *  G  M  A  V  Y  S  V  N  G  N  *  Q  I  P  S  P  T  I  N  I  L  C  H  Q  M  Y  I  S  F  K  N  A  K  N  *  M  F  V  I  I  M  I  A    F5
      V  H  M  R  L  *  D  K  I  L  K  V  C  C  N  F  I  T  Q  L  K  H  K  G  W  Q  L  M  A  *  T  V  M  R  S  F  P  P  P  P  P  S  I  *  *  A  T  N  C  I  F  P  F  S  T  Q  K  I  E  C  L  Y  L  L  *  *  L  L   F4

      Y  S  C  L  L  G  V  A  S  S  Y  R  I  K  N  F  Y  S  K  L  L  *  F  R  L  V  I  S  F  V  N  P  F  E  Q  N  F  M  R  I  *  I  Q  A  D  L  E  *  K  E  S  P  T  F  E  L  N  F  T  S  *  V  R  K  C  V  S   F1
       I  L  A  S  S  G  *  P  L  H  T  V  L  K  T  F  T  L  S  Y  C  D  L  G  *  *  L  V  L  L  I  R  S  S  R  T  S  C  V  F  E  Y  R  R  I  *  S  K  R  N  P  Q  L  L  S  *  I  S  L  L  E  S  G  N  V  *  V   F2
       F  L  P  P  R  G  S  L  F  I  P  Y  *  K  L  L  L  *  V  I  V  I  *  A  S  N  *  F  C  *  S  V  R  A  E  L  H  A  Y  L  N  T  G  G  F  R  V  K  G  I  P  N  F  *  A  K  F  H  F  L  S  Q  E  M  C  E    F3
1801 TATTCTTGCCTCCTCGGGGTAGCCTCTTCATACCGTATTAAAAACTTTTACTCTAAGTTATTGTGATTTAGGCTAGTAATTAGTTTTGTTAATCCGTTCGAGCAGAACTTCATGCGTATTTGAATACAGGCGGATTTAGAGTAAAAGGAATCCCCAACTTTTGAGCTAAATTTCACTTCTTGAGTCAGGAAATGTGTGAG 2000
     ----:----|----:----|----:----|----:----|----:----|----:----|----:----|----:----|----:----|----:----|----:----|----:----|----:----|----:----|----:----|----:----|----:----|----:----|----:----|----:----|
1801 ATAAGAACGGAGGAGCCCCATCGGAGAAGTATGGCATAATTTTTGAAAATGAGATTCAATAACACTAAATCCGATCATTAATCAAAACAATTAGGCAAGCTCGTCTTGAAGTACGCATAAACTTATGTCCGCCTAAATCTCATTTTCCTTAGGGGTTGAAAACTCGATTTAAAGTGAAGAACTCAGTCCTTTACACACTC 2000
       Y  E  Q  R  R  P  T  A  E  E  Y  R  I  L  F  K  *  E  L  N  N  H  N  L  S  T  I  L  K  T  L  G  N  S  C  F  K  M  R  I  Q  I  C  A  S  K  S  Y  F  S  D  G  V  K  S  S  F  K  V  E  Q  T  L  F  H  T  L   F6
      I  N  K  G  G  R  P  L  R  K  M  G  Y  *  F  S  K  S  *  T  I  T  I  *  A  L  L  *  N  Q  *  D  T  R  A  S  S  *  A  Y  K  F  V  P  P  N  L  T  F  P  I  G  L  K  Q  A  L  N  *  K  K  L  *  S  I  H  S   F5
       I  R  A  E  E  P  Y  G  R  *  V  T  N  F  V  K  V  R  L  *  Q  S  K  P  *  Y  N  T  K  N  I  R  E  L  L  V  E  H  T  N  S  Y  L  R  I  *  L  L  L  F  G  W  S  K  L  *  I  E  S  R  S  D  P  F  T  H    F4

       C  L  C  Q  T  Q  I  *  Q  T  E  M  P  I  N  Y  C  K  I  L  V  *  A  S  L  R  Y  G  M  L  H  V  V  M  C  V  M  K  L  I  R  C  H  V  C  R  K  H  S  K  *  L  F  *  F  N  L  S  *  M  V  R  Q  N  V  D  A   F1
        A  Y  V  K  P  R  F  N  K  Q  K  C  L  L  I  T  V  R  F  *  Y  R  P  P  Y  A  T  A  C  C  T  L  *  C  V  L  *  N  *  S  D  A  T  C  A  E  S  I  A  N  D  Y  F  D  L  I  *  V  K  W  S  V  K  M  L  T    F2
      L  P  M  S  N  P  D  L  T  N  R  N  A  Y  *  L  L  *  D  F  S  I  G  L  P  T  L  R  H  V  A  R  C  N  V  C  Y  E  I  D  P  M  P  R  V  Q  K  A  *  Q  M  I  I  L  I  *  F  E  L  N  G  P  S  K  C  *  R   F3
2001 TTGCCTATGTCAAACCCAGATTTAACAAACAGAAATGCCTATTAATTACTGTAAGATTTTAGTATAGGCCTCCCTACGCTACGGCATGTTGCACGTTGTAATGTGTGTTATGAAATTGATCCGATGCCACGTGTGCAGAAAGCATAGCAAATGATTATTTTGATTTAATTTGAGTTAAATGGTCCGTCAAAATGTTGACG 2200
     ----:----|----:----|----:----|----:----|----:----|----:----|----:----|----:----|----:----|----:----|----:----|----:----|----:----|----:----|----:----|----:----|----:----|----:----|----:----|----:----|
2001 AACGGATACAGTTTGGGTCTAAATTGTTTGTCTTTACGGATAATTAATGACATTCTAAAATCATATCCGGAGGGATGCGATGCCGTACAACGTGCAACATTACACACAATACTTTAACTAGGCTACGGTGCACACGTCTTTCGTATCGTTTACTAATAAAACTAAATTAAACTCAATTTACCAGGCAGTTTTACAACTGC 2200
       Q  R  H  *  V  W  I  *  C  V  S  I  G  I  L  *  Q  L  I  K  T  Y  A  E  R  R  *  P  M  N  C  T  T  I  H  T  I  F  N  I  R  H  W  T  H  L  F  C  L  L  H  N  N  Q  N  L  K  L  *  I  T  R  *  F  T  S    F6
       N  G  I  D  F  G  S  K  V  F  L  F  A  *  *  N  S  Y  S  K  L  I  P  R  G  V  S  R  C  T  A  R  Q  L  T  H  *  S  I  S  G  I  G  R  T  C  F  A  Y  C  I  I  I  K  I  *  N  S  N  F  P  G  D  F  H  Q  R   F5
      T  A  *  T  L  G  L  N  L  L  C  F  H  R  N  I  V  T  L  N  *  Y  L  G  G  *  A  V  A  H  Q  V  N  Y  H  T  N  H  F  Q  D  S  A  V  H  A  S  L  M  A  F  S  *  K  S  K  I  Q  T  L  H  D  T  L  I  N  V    F4

       K  C  V  M  H  G  N  P  I  S  D  S  H  I  R  P  M  T  H  H  S  R  G  R  *  A  R  C  A  S  Y  N  *  *  V  H  K  G  L  I  I  L  F  Q  Y  L  Y  *  V  D  T  P  P  T  T  P  F  F  Y  I  A  I  L  E  R  V    F1
      Q  S  A  S  C  M  V  T  R  Y  L  T  R  I  Y  D  Q  *  R  T  I  P  G  V  A  R  P  G  V  P  L  T  I  D  K  Y  I  K  V  L  *  S  C  F  N  I  F  I  R  S  T  H  P  R  P  R  P  S  S  I  L  Q  F  S  N  G  Y   F2
       K  V  R  H  A  W  *  P  D  I  *  L  A  Y  T  T  N  D  A  P  F  P  G  S  L  G  Q  V  C  L  L  Q  L  I  S  T  *  R  S  Y  N  L  V  S  I  S  L  L  G  R  H  T  P  D  H  A  L  L  L  Y  C  N  S  R  T  G  I   F3
2201 CAAAGTGCGTCATGCATGGTAACCCGATATCTGACTCGCATATACGACCAATGACGCACCATTCCCGGGGTCGCTAGGCCAGGTGTGCCTCTTACAATTGATAAGTACATAAAGGTCTTATAATCTTGTTTCAATATCTTTATTAGGTCGACACACCCCCGACCACGCCCTTCTTCTATATTGCAATTCTCGAACGGGTA 2400
     ----:----|----:----|----:----|----:----|----:----|----:----|----:----|----:----|----:----|----:----|----:----|----:----|----:----|----:----|----:----|----:----|----:----|----:----|----:----|----:----|
2201 GTTTCACGCAGTACGTACCATTGGGCTATAGACTGAGCGTATATGCTGGTTACTGCGTGGTAAGGGGCCCCAGCGATCCGGTCCACACGGAGAATGTTAACTATTCATGTATTTCCAGAATATTAGAACAAAGTTATAGAAATAATCCAGCTGTGTGGGGGCTGGTGCGGGAAGAAGATATAACGTTAAGAGCTTGCCCAT 2400
      A  F  H  T  M  C  P  L  G  I  D  S  E  C  I  R  G  I  V  C  W  E  R  P  R  *  A  L  H  A  E  *  L  Q  Y  T  C  L  P  R  I  I  K  N  *  Y  R  *  *  T  S  V  G  G  V  V  G  K  K  *  I  A  I  R  S  R  T   F6
       L  T  R  *  A  H  Y  G  S  I  Q  S  A  Y  V  V  L  S  A  G  N  G  P  D  S  P  W  T  H  R  K  C  N  I  L  V  Y  L  D  *  L  R  T  E  I  D  K  N  P  R  C  V  G  S  W  A  R  R  R  Y  Q  L  E  R  V  P    F5
       C  L  A  D  H  M  T  V  R  Y  R  V  R  M  Y  S  W  H  R  V  M  G  P  T  A  L  G  P  T  G  R  V  I  S  L  Y  M  F  T  K  Y  D  Q  K  L  I  K  I  L  D  V  C  G  R  G  R  G  E  E  I  N  C  N  E  F  P  Y   F4
```

```
      * L C S K T Q F T I F V L Q E V L F H L S M E Q R L W T S V N D R S Y G V V S I S R N I D A R M I * * F S R N * P F R E E E L R F F R   F1
      N Y V Q R R S L R S L C C R R Y S S T S P W S S G C G H R S T T D H M G W S R S L G I * M H G * F N D S P G I N P S E K R S S D F S D   F2
      I M F K D A V Y D L C V A G G T L P P L H G A A V V D I G Q R Q I I W G G L D L S E Y R C T D D L M I L Q E L T L P R R G A P I F Q   F3
2401 TAATTATGTTCAAAGACGCAGTTTACGATCTTTGTGTTGCAGGAGGTACTCTTCCACCTCTCCATGGAGCAGCGGTTGTGGACATCGGTCAACGACAGATCATATGGGGTGGTCTCGATCTCTCGGAATATAGATGCACGGATGATTTAATGATTCTCCAGGAATTAACCCTTCCGAGAAGAGGAGCTCCGATTTTTCAG 2600
     ----:----|----:----|----:----|----:----|----:----|----:----|----:----|----:----|----:----|----:----|----:----|----:----|----:----|----:----|----:----|----:----|----:----|----:----|----:----|----:----|
2401 ATTAATACAAGTTTCTGCGTCAAATGCTAGAAACACAACGTCCTCCATGAGAAGGTGGAGAGGTACCTCGTCGCCAACACCTGTAGCCAGTTGCTGTCTAGTATACCCCACCAGAGCTAGAGAGCCTTATATCTACGTGCCTACTAAATTACTAAGAGGTCCTTAATTGGGAAGGCTCTTCTCCTCGAGGCTAAAAAGTC 2600
      Y N H E F V C N V I K T N C S T S K W R E M S C R N H V D T L S L D Y P T T E I E R F I S A R I I * H N E L F * G K R S S S S R N K L   F6
      I I N L S A T * S R Q T A P P V R G G R W P A A T T S M P * R C I M H P P R S R E S Y L H V S S K I I R W S N V R G L L P A G I K *   F5
      L * T * L R L K R D K H Q L L Y E E V E G H L L P Q P C R D V V S * I P H D R D R P I Y I C P H N L S E G P I L G E S F L L E S K E   F4

      * H T F K L * N T K S L L G W V K T S H T K Q G L F L Q V * L R S I K W N Y I S H S * F R I I F P * S L T Q S P I S F S S S L T F L F   F1
      N I H S N S E T P S P F * A G * R H P T P N R A C S F R Y D S G A S N G T T S R I A S S E * Y F R D H L P S L Q Y L F L P L S H F S   F2
      I T Y I Q T L K H Q V P S R L G K D I P H Q T G P V P S G M T Q E H Q M E L H L A * L V Q N N I S V I T Y P V S N I F F F L S H I S L   F3
2601 ATAACATACATTCAAACTCTGAAACACCAAGTCCCTTCTAGGCTGGGTAAAGACATCCCACACCAAACAGGGCCTGTTCCTTCAGGTATGACTCAGGAGCATCAAATGGAACTACATCTCGCATAGCTAGTTCAGAATAATATTTCCGTGATCACTTACCCAGTCTCCAATATCTTTTTCTTCCTCTCTCACATTTCTCT 2800
     ----:----|----:----|----:----|----:----|----:----|----:----|----:----|----:----|----:----|----:----|----:----|----:----|----:----|----:----|----:----|----:----|----:----|----:----|----:----|----:----|
2601 TATTGTATGTAAGTTTGAGACTTTGTGGTTCAGGGAAGATCCGACCCATTTCTGTAGGGTGTGGTTTGTCCCGGACAAGGAAGTCCATACTGAGTCCTCGTAGTTTACCTTGATGTAGAGCGTATCGATCAAGTCTTATTATAAAGGCACTAGTGAATGGGTCAGAGGTTATAGAAAAAGAAGGAGAGAGTGTAAAGAGA 2800
      Y C V N L S Q F V L D R R P Q T F V D W V L C P R N R * T H S L L M L H F * M E C L * N L I I N G H D S V W D G I D K E E E R V N R   F6
      I V Y M * V R F C W T G E L S P L S M G C W V P G T G E P I V * S C * I S S C R A Y S T * F L I E T I V * G T E L I K K K R E * M E R   F5
      S L M C E F E S V G L G K * A P Y L C G V G F L A Q E K L Y S E P A D F P V V D R M A L E S Y Y K R S * K G L R W Y R K R G R E C K E   F4

      P P L S H F S F L V C I F L P H S F A I S F L P F F L D L F L Y S S S S K S N L T S I H E K V L H T S I * L C F * F R K E L F V F L   F1
      F L L S H I S L S S F V Y F S L T L S P S H F S H S F S T Y F F I L L P P K V T * H Q S M R K F C I L V Y N C V S D F E K N C L Y F F   F2
      S S S L T F L F P R L Y I S P S L F R H L I S P I L S R L I S L F F F L Q K * L D I N P * E S F A Y * Y I T V F L I S K R I V C I S L   F3
2801 TTCCTCCTCTCTCACATTTCTCTTTCCTCGTTTGTATATTTCTCCCTCACTCTTTCGCCATCTCATTTCTCCCATTCTTTCTCGACTTATTTCTTTATTCTTCTTCCTCCAAAAGTAACTTGACATCAATCCATGAGAAAGTTTTGCATACTAGTATATAACTGTGTTTCTGATTTCGAAAAGAATTGTTTGTATTTCTT 3000
     ----:----|----:----|----:----|----:----|----:----|----:----|----:----|----:----|----:----|----:----|----:----|----:----|----:----|----:----|----:----|----:----|----:----|----:----|----:----|----:----|
2801 AAGGAGGAGAGAGTGTAAAGAGAAAGGAGCAAACATATAAAGAGGGAGTGAGAAAGCGGTAGAGTAAAGAGGGTAAGAAAGAGCTGAATAAAGAAATAAGAAGAAGGAGGTTTTCATTGAACTGTAGTTAGGTACTCTTTCAAAACGTATGATCATATATTGACACAAAGACTAAAGCTTTTCTTAACAAACATAAAGAA 3000
      K G G R E C K E K R T Q I N R G * E K A M E N R G N K R S K N R * E E E E L L L K V D I W S F T K C V L I Y S H K Q N R F S N N T N R   F6
      E E E R V N R K G R K Y I E G E S K R W R M E G M R E R S I E K N K K R W F Y S S M L G H S L K A Y * Y I V T N R I E F L I T Q I E   F5
      K R R E * M E R E E N T Y K E R V R E G D * K E W E K E V * K K I R R G G F T V Q C * D M L F N Q M S T Y L Q T E S K S F F Q K Y K K   F4

      * T F T V I L Q T I Q P L G S K D F L R N T N S I F L K N Y I F I Y M C I H V H I K H I P Y M L H A K C T I M T M * N D R V T F * E Q   F1
      E L S L L Y Y K Q F S L W A A K I F * E I P I Q Y F * K I I Y L F I C V Y M Y I * N T F L T C C M Q N A Q L * Q C R M I E S R F E N K   F2
      N F H C Y I T N N S A F G Q Q R F S K K Y Q F N I F K K L Y I Y L Y V Y T C T Y K T H S L H V A C K M H N Y D N V E * * S H V L R T   F3
3001 TGAACTTTCACTGTTATATTACAAACAATTCAGCCTTTGGGCAGCAAAGATTTTCTAAGAAATACCAATTCAATATTTTTAAAAAATTATATATTTATTTATATGTGTATACATGTACATATAAAACACATTCCTTACATGTTGCATGCAAAATGCACAATTATGACAATGTAGAATGATAGAGTCACGTTTTGAGAACA 3200
     ----:----|----:----|----:----|----:----|----:----|----:----|----:----|----:----|----:----|----:----|----:----|----:----|----:----|----:----|----:----|----:----|----:----|----:----|----:----|----:----|
3001 ACTTGAAAGTGACAATATAATGTTTGTTAAGTCGGAAACCCGTCGTTTCTAAAAGATTCTTTATGGTTAAGTTATAAAAAATTTTTAATATATAAATAAATATACACATATGTACATGTATATTTTGTGTAAGGAATGTACAACGTACGTTTTACGTGTTAATACTGTTACATCTTACTATCTCAGTGCAAAACTCTTGT 3200
      Q V K V T I N C V I * G K P L L S K R L F V L E I N K F F * I N I * I H I C T C I F C M G * M N C A F H V I I V I Y F S L T V N Q S C   F6
      K F K * Q * I V F L E A K P C C L N E L F Y W N L I K L F N Y I * K Y T Y V H V Y L V C E K C T A H L I C L * S L T S H Y L * T K L V   F5
      S S E S N Y * L C N L R Q A A F I K * S I G I * Y K * F I I Y K N I H T Y M Y M Y F V N R V H Q M C F A C N H C H L I I S D R K S F   F4

      M Y T S L F Y L H I Y K F F Y F I D I H V T T I H L F L Q K E V A I L * Q C Y P V P T K L F Y L V A Y L W E H T K G T T T I H D F V K   F1
      C T L L Y S I Y I Y T N F S I L L T Y M * L Q S I C S Y R K K W P Y F D N A T R Y Q Q S S S I W W L I Y G N T Q R V L Q Q F T I L S   F2
      N V H F F I L F T Y I Q I F L F Y * H T C N Y N P F V L T E R S G H T L T M L P G T N K A L L F G G L S M G T H K G Y Y N N S R F C Q   F3
3201 AATGTACACTTCTTTATTCTATTTACATATATACAAATTTTTCTATTTTATTGACATACATGTAACTACAATCCATTTGTTCTTACAGAAAGAAGTGGCCATACTTTGACAATGCTACCCGGTACCAACAAAGCTCTTCTATTTGGTGGCTTATCTATGGGAACACACAAAGGGTACTACAACAATTCACGATTTTGTCA 3400
     ----:----|----:----|----:----|----:----|----:----|----:----|----:----|----:----|----:----|----:----|----:----|----:----|----:----|----:----|----:----|----:----|----:----|----:----|----:----|----:----|
3201 TTACATGTGAAGAAATAAGATAAATGTATATATGTTTAAAAAGATAAAATAACTGTATGTACATTGATGTTAGGTAAACAAGAATGTCTTTCTTCACCGGTATGAAACTGTTACGATGGGCCATGGTTGTTTCGAGAAGATAAACCACCGAATAGATACCCTTGTGTGTTTCCCATGATGTTGTTAAGTGCTAAAACAGT 3400
      I Y V E K N * K C I Y L N K * K I S M C T V V I W K N K C F S T A M S Q C H * G T G V F S K * K T A * R H S C V F P V V V I * S K T   F6
      F T C K K I R N V Y I C I K R N * Q C V H L * L G N T R V S L L P W V K V I S G P V L L A R R N P P K D I P V C L P Y * L L E R N Q *   F5
      L H V S R * E I * M Y V F K E I K N V Y M Y S C D M Q E * L F F H G Y K S L A V R Y W C L E E I Q H S I * P F V C L T S C C N V I K D   F4

      L V R M A D F T F W T Q T R T S G N T S R C P * F S Q G H T I P * Q * W R R T M S L S Q L * L E E L Y M K R L R Q P I V R P L M K L   F1
      N L * G W Q I L H F G H R H V R V A T H Q G A L D S A K G I P F R D S N G E G Q * V C R S F D W R S C I * R D C A N P S * G P * * N C   F2
      T C K D G R F Y I L D T D T Y E W Q H I K V P L I Q P R A Y H S V T V M E K D N E F V A A L I G G V V Y E E T A P T H R E A L N E I V   F3
3401 AACTTGTAAGGATGGCAGATTTTACATTTTGGACACAGACACGTACGAGTGGCAACACATCAAGGTGCCCTTGATTCAGCCAAGGGCATACCATTCCGTGACAGTAATGGAGAAGGACAATGAGTTTGTCGCAGCTTTGATTGGAGGAGTTGTATATGAAGAGACTGCGCCAACCCATCGTGAGGCCCTTAATGAAATTG 3600
     ----:----|----:----|----:----|----:----|----:----|----:----|----:----|----:----|----:----|----:----|----:----|----:----|----:----|----:----|----:----|----:----|----:----|----:----|----:----|----:----|
3401 TTGAACATTCCTACCGTCTAAAATGTAAAACCTGTGTCTGTGCATGCTCACCGTTGTGTAGTTCCACGGGAACTAAGTCGGTTCCCGTATGGTAAGGCACTGTCATTACCTCTTCCTGTTACTCAAACAGCGTCGAAACTAACCTCCTCAACATATACTTCTCTGACGCGGTTGGGTAGCACTCCGGGAATTACTTTAAC 3600
      L S T L I A S K V N Q V C V R V L P L V D L H G Q N L W P C V M G H C Y H L L V I L K D C S Q N S S N Y I F L S R W G M T L G K I F N   F6
      V Q L S P L N * M K S V S V Y S H C C M L T G K I * G L A Y W E T V T I S F S L S N T A A K I P P T T Y S S V A G V W R S A R L S I   F5
      F K Y P H C I K C K P C L C T R T A V C * P A R S E A L P M G N R S L L P S P C H T Q R L K S Q L L Q I H L S Q A L G D H P G * H F Q   F4

      S S * Q * I R K Y K T S V * K K F H F N Q A C Q L H T M F S C L L M Q P Q C T T M S L L L Q V E F R I K R K K * K R R V E K L H L L Y   F1
      R L N N R * G N T K L Q S E R S F T S T K H A N Y T Q C F L V F S C N H S A Q Q C H Y C C R W S S G S K E R N E R E E * K S F I Y C I   F2
      V L T I D K E I Q N F S L K E V S L Q P S M P T T H N V F L S S H A T T V H N N V I I V A G G V Q D Q K K E M K E K S R K A S S T V   F3
3601 TCGTCTTAACAATAGATAAGGAAATACAAAACTTCAGTCTGAAAGAAGTTTCACTTCAACCAAGCATGCCAACTACACACAATGTTTTCTTGTCTTCTCATGCAACCACAGTGCACAACAATGTCATTATTGTTGCAGGTGGAGTTCAGGATCAAAAGAAAGAAATGAAAGAGAAGAGTAGAAAAGCTTCATCTACTGTA 3800
     ----:----|----:----|----:----|----:----|----:----|----:----|----:----|----:----|----:----|----:----|----:----|----:----|----:----|----:----|----:----|----:----|----:----|----:----|----:----|----:----|
3601 AGCAGAATTGTTATCTATTCCTTTATGTTTTGAAGTCAGACTTTCTTCAAAGTGAAGTTGGTTCGTACGGTTGATGTGTGTTACAAAAGAACAGAAGAGTACGTTGGTGTCACGTGTTGTTACAGTAATAACAACGTCCACCTCAAGTCCTAGTTTTCTTTCTTTACTTTCTCTTCTCATCTTTTCGAAGTAGATGACAT 3800
      D D * C Y I L F Y L V E T Q F F N * K L W A H W S C V I N E Q R R M C G C H V V I D N N N C T S N L I L L F F H F L L T S F S * R S Y   F6
      T T K V I S L S I C F K L R F S T E S * G L M G V V C L T K K D E * A V V T C L L T M I T A P P T * S * F F S I F S F L L F A E D V T   F5
      R R L L L Y P F V F S * D S L L K V E V L C A L * V C H K R T K E H L W L A C C H * * Q Q L H L E P D F S L F S L S S Y F L K M * Q   F4

      T A S T * E Q K F L R P F L Q H V M Q A K H L Q L M D I L F I I W L V I L I T P F L Y L V A A Q G K F H Y * Q I D R S N P S H V I L N   F1
      Q H R L R S K S F * G L S C N M * C K R N I C N S W T F Y S S F G * * Y * * H L S C T W W Q L K A N F I T N R S I V R T R V M * F *   F2
      Y S I D L G A K V F E A F P A T C N A S E T F A T H G H S I H H L A S D T D N T F L V L G G S S R Q I S L L T D R S F E P E S C D S E   F3
3801 TACAGCATCGACTTAGGAGCAAAAGTTTTTGAGGCCTTTCCTGCAACATGTAATGCAAGCGAAACATTTGCAACTCATGGACATTCTATTCATCATTTGGCTAGTGATACTGATAACACCTTTCTTGTACTTGGTGGCAGCTCAAGGCAAATTTCATTACTAACAGATCGATCGTTCGAACCCGAGTCATGTGATTCTGA 4000
     ----:----|----:----|----:----|----:----|----:----|----:----|----:----|----:----|----:----|----:----|----:----|----:----|----:----|----:----|----:----|----:----|----:----|----:----|----:----|----:----|
3801 ATGTCGTAGCTGAATCCTCGTTTTCAAAAACTCCGGAAAGGACGTTGTACATTACGTTCGCTTTGTAAACGTTGAGTACCTGTAAGATAAGTAGTAAACCGATCACTATGACTATTGTGGAAAGAACATGAACCACCGTCGAGTTCCGTTTAAAGTAATGATTGTCTAGCTAGCAAGCTTGGGCTCAGTACACTAAGACT 4000
      V A D V * S C F N K L G K R C C T I C A F C K C S M S M R N M M Q S T I S I V G K K Y K T A A * P L N * * * C I S R E F G L * T I R   F6
      Y L M S K P A F T K S A K G A V H L A L S V N A V * P C E I * * K A L S V S L V K R T S P P L E L C I E N S V S R D N S G S D H S E S   F5
      I C C R S L L L L K Q P R E Q L M Y H L R F M Q L E H V N * E D N P * H Y Q Y C R E Q V Q H C S L A F K M V L L D I T R V R T M H N Q   F4

      H V P L H L L G T R * T L H G Y N A I E N A R N G F I Y T V * S * P E S Q K E I T I A K S A K N R L P V Y L L C A N Y Q T C N Q H T   F1
      T M Y H C I F W G Q G R P Y M D T M Q * K M P E M V S Y T L Y K A N Q N P R R R L P L Q K V Q K I D Y R Y T C Y V L I I K H V I S I H   F2
      P C T I A S S G D K V D P T W I Q C N R K C Q K W F H I H C I K L T R I P E G D Y H C K K C K K * T T G I L A M C * L S N M * S A Y T   F3
4001 ACCATGTACCATTGCATCTTCTGGGGACAAGGTAGACCCTACATGGATACAATGCAATAGAAAATGCCAGAAATGGTTTCATATACACTGTATAAAGCTAACCAGAATCCCAGAAGGAGATTACCATTGCAAAAAGTGCAAAAAATAGACTACCGGTATACTTGCTATGTGCTAATTATCAAACATGTAATCAGCATACA 4200
     ----:----|----:----|----:----|----:----|----:----|----:----|----:----|----:----|----:----|----:----|----:----|----:----|----:----|----:----|----:----|----:----|----:----|----:----|----:----|----:----|
4001 TGGTACATGGTAACGTAGAAGACCCCTGTTCCATCTGGGATGTACCTATGTTACGTTATCTTTTACGGTCTTTACCAAAGTATATGTGACATATTTCGATTGGTCTTAGGGTCTTCCTCTAATGGTAACGTTTTTCACGTTTTTTATCTGATGGCCATATGAACGATACACGATTAATAGTTTGTACATTAGTCGTATGT 4200
      F W T G N C R R P V L Y V R C P Y L A I S F A L F P K M Y V T Y L * G S D W F S I V M A F L A F F L S G T Y K S H A L * * V H L * C V   F6
      G H V M A D E P S L T S G V H I C H L L F H W F H N * I C Q I F S V L I G S P S * W Q L F H L F Y V V P I S A I H * N D F M Y D A Y   F5
      V M Y W Q M K Q P C P L G * M S V I C Y F I G S I T E Y V S Y L A L W F G L L L N G N C F T C F I S * R Y V Q * T S I I L C T I L M C   F4

      L M Y L K V I S C I H V C L K T L N H K Y W * V Y K V Q F T F S R K L N * K L C V T K N A R Y P I N * L S M F Q R * I W L I S * F S I   F1
      L C I L K S S L V Y M S V * K P * I I N T G K F T K Y N S H F L E N * I E S F V * R K M H D T Q L I N Y Q C S K D K F G L S V S S P S   F2
      Y V S * S H L L Y T C L F E N L E S * I L V S L Q S T I H I F * K I K L K A L C N E K C T I P N * L I I N V P K I N L A Y Q L V L H   F3
4201 CTTATGTATCTTAAAGTCATCTCTTGTATACATGTCTGTTTGAAAACCTTGAATCATAAATACTGGTAAGTTTACAAAGTACAATTCACATTTTCTAGAAAATTAAATTGAAAGCTTTGTGTAACGAAAAATGCACGATACCCAATTAATTAATTATCAATGTTCCAAAGATAAATTTGGCTTATCAGTTAGTTCTCCAT 4400
     ----:----|----:----|----:----|----:----|----:----|----:----|----:----|----:----|----:----|----:----|----:----|----:----|----:----|----:----|----:----|----:----|----:----|----:----|----:----|----:----|
4201 GAATACATAGAATTTCAGTAGAGAACATATGTACAGACAAACTTTTGGAACTTAGTATTTATGACCATTCAAATGTTTCATGTTAAGTGTAAAAGATCTTTTAATTTAACTTTCGAAACACATTGCTTTTTACGTGCTATGGGTTAATTAATTAATAGTTACAAGGTTTCTATTTAAACCGAATAGTCAATCAAGAGGTA 4400
      S I Y R L T M E Q I C T Q K F V K F * L Y Q Y T * L T C N V N E L F N F Q F S Q T V F F A R Y G I L * N D I N W L Y I Q S I L * N E M   F6
      V * T D * L * R K Y V H R N S F R S D Y I S T L K C L V I * M K * F I L N F A K H L S F H V I G L * N I I L T G F I F K A * * N T R W   F5
      K H I K F D D R T Y M D T Q F G Q I M F V P L N V F Y L E C K R S F * I S L K T Y R F I C S V W N I L * * H E L S L N P K D T L E G   F4

      L V D L T S K S I A F A * V V Y S L K G K Y D V L Q C S T E H K I K R I * S R S L I T G E Q Q F Y T Y T C T S E K R E R A Q * S K I I   F1
      * * I * P V R V S L L L K L S T V * K E N T M Y F N V L Q N T K * K E F N L V P * * Q V N N S F I L I H V P V R S V S E L S K V K *   F2
      P S R F N Q * E Y R F C L S C L Q F K R K I R C T S M F Y R T Q N K K N L I S F L N N R * T T V L Y L Y M Y Q * E A * A S S V K * N N   F3
4401 CCTAGTAGATTTAACCAGTAAGAGTATCGCTTTTGCTTAAGTTGTCTACAGTTTAAAAGGAAAATACGATGTACTTCAATGTTCTACAGAACACAAAATAAAAAGAATTTAATCTCGTTCCTTAATAACAGGTGAACAACAGTTTTATACTTATACATGTACCAGTGAGAAGCGTGAGCGAGCTCAGTAAAGTAAAATAA 4600
     ----:----|----:----|----:----|----:----|----:----|----:----|----:----|----:----|----:----|----:----|----:----|----:----|----:----|----:----|----:----|----:----|----:----|----:----|----:----|----:----|
4401 GGATCATCTAAATTGGTCATTCTCATAGCGAAAACGAATTCAACAGATGTCAAATTTTCCTTTTATGCTACATGAAGTTACAAGATGTCTTGTGTTTTATTTTTCTTAAATTAGAGCAAGGAATTATTGTCCACTTGTTGTCAAAATATGAATATGTACATGGTCACTCTTCGCACTCGCTCGAGTCATTTCATTTTATT 4600
      R T S K V L L L I A K A * T T * L K F P F Y S T S * H E V S C L I F L I * D R E K I V P S C C N * V * V H V L S F R S R A * Y L L I   F6
      G L L N L W Y S Y R K Q K L Q R C N L L F I R H V E I N * L V C F L F F K I E N R L L L H V V T K Y K Y M Y W H S A H A L E T F Y F L   F5
      D * Y I * G T L T D S K S L N D V T * F S F V I Y K L T R C F V F Y F S N L R T G * Y C T F L L K I S I C T G T L L T L S S L L T F Y   F4

      Y Q I H H S L K K K K K L E L K N L G R V C T I F K V L P F T C S G S I F I H * Y I T A M Y L T S N S T A E H T C I F I I T T N K I   F1
      F I R Y I I V * K K K K N * S * R T W E E C A Q F L K F Y L L H V Q D R Y S F I D I * L Q C I * L Q I V L L N I H V Y L L * Q L T R F   F2
      L S D T S * F K K K K K I R V K E P G K S V H N F * S F T F Y M F R I D I H S L I Y N C N V F D F K * Y C * T Y M Y I Y Y N N * Q D S   F3
4601 TTTATCAGATACATCATAGTTTAAAAAAAAAAAAAAAAAATTAGAGTTAAAGAACCTGGGAAGAGTGTGCACAATTTTTAAAGTTTTACCTTTTACATGTTCAGGATCGATATTCATTCATTGATATATAACTGCAATGTATTTGACTTCAAATAGTACTGCTGAACATACATGTATATTTATTATATAACAACTAACAAGATT 4800
     ----:----|----:----|----:----|----:----|----:----|----:----|----:----|----:----|----:----|----:----|----:----|----:----|----:----|----:----|----:----|----:----|----:----|----:----|----:----|----:----|
4601 AAATAGTCTATGTAGTATCAAATTTTTTTTTTTTTTTTTTAATCTCAATTTCTTGGACCCTTCTCACACGTGTTAAAAATTTCAAAATGGAAAATGTACAAGTCCTAGCTATAAGTAAGTAACTATATATTGACGTTACATAAACTGAAGTTTATCATGACGACTTGTATGTACATATAAATAATATTGTTGATTGTTCTAA 4800
      I * * I C * L K F F F F F N S N F F R P L T H V I K L T K G K V H E P D I N M * Q Y I V A I Y K V E F L V A S C V H I N I I V V L I   F6
      K D S V D Y N L F F F F I L T L S G P F L T C L K * L K V K * M N L I S I * E N I Y L Q L T N S K L Y Y Q Q V Y M Y I * * L L * C S   F5
      N I L Y M M T * F F F F F * L * L V Q S S H A C N K F N * R K C T * S R Y E N M S I Y S C H I Q S * I T S S F M C T Y K N Y C S V L N   F4

      P Q C K I L K K K N H R S S T S S L I L T F * K I E K V L A G F P N * I K T * I L * K V P I S I C I H I * T G N A K T K F K M S Q Q P   F1
      P S A R F * R K K I I G P A H L L L F * L S K K * K K F W L G F P I E * K H K S C E K Y L Y Q Y V F T F E L A M R K Q N S K C H S S P   F2
      P V Q D F E E K K S * V Q H I F S Y S N F L K N R K S S G W V S Q L N K N I N P V K S T Y I N M Y S H L N W Q C E N K I Q N V T A A   F3
4801 CCCCAGTGCAAGATTTTGAAGAAAAAAAATCATAGGTCCAGCACATCTTCTCTTATTCTAACTTTCTAAAAAATAGAAAAAGTTCTGGCTGGGTTTCCCAATTGAATAAAAACATAAATCCTGTGAAAAGTACCTATATCAATATGTATTCACATTTGAACTGGCAATGCGAAAACAAAATTCAAAATGTCACAGCAGCC 5000
     ----:----|----:----|----:----|----:----|----:----|----:----|----:----|----:----|----:----|----:----|----:----|----:----|----:----|----:----|----:----|----:----|----:----|----:----|----:----|----:----|
4801 GGGGTCACGTTCTAAAACTTCTTTTTTTTAGTATCCAGGTCGTGTAGAAGAGAATAAGATTGAAAGATTTTTTATCTTTTTCAAGACCGACCCAAAGGGTTAACTTATTTTTGTATTTAGGACACTTTTCATGGATATAGTTATACATAAGTGTAAACTTGACCGTTACGCTTTTGTTTTAAGTTTTACAGTGTCGTCGG 5000
      G W H L I K F F F F * L D L V D E R I R V K * F I S F T R A P N G L Q I F V Y I R H F T G I D I H I * M Q V P L A F V F N L I D C C G   F6
      E G T C S K S S F F D Y T W C M K E * E L K R F F L F L E P Q T E W N F L F M F G T F L V * I L I Y E C K F Q C H S F L I * F T V A A   F5
      G L A L N Q L F F I M P G A C R R K N * S E L F Y F F N Q S P K G I S Y F C L D Q S F Y R Y * Y T N V N S S A I R F C F E F H * L L   F4
```

```
     L F F L * C K * N C L K Q N * K I L R * E Y * F S K P E F N T L L H V H * Y M * N S F E E E K I Y L C C I A Y F * * E H C L * G * * H   F1
      Y F S C N V N K T A * N K T K K S C V E S I N L V N P S S I H Y C M Y I D T C R I L L K K K K Y I C A V L L T S N E N T A C K D N N    F2
     L I F L V M * I K L P E T K L K N L A L R V L I * * T R V Q Y I I A C T L I H V E F F * R R K N I F V L Y C L L L M R T L P V R I I T   F3
5001 CTTATTTTTCTTGTAATGTAAATAAAACTGCCTGAAACAAAACTAAAAAATCTTGCGTTGAGAGTATTAATTTAGTAAACCCGAGTTCAATACATTATTGCATGTACATTGATACATGTAGAATTCTTTTGAAGAAGAAAAAAATATATTTGTGCTGTATTGCTTACTTCTAATGAGAACACTGCCTGTAAGGATAATAAC 5200
     ----:----|----:----|----:----|----:----|----:----|----:----|----:----|----:----|----:----|----:----|----:----|----:----|----:----|----:----|----:----|----:----|----:----|----:----|----:----|----:----|
5001 GAATAAAAAGAACATTACATTTATTTTGACGGACTTTGTTTTGATTTTTTAGAACGCAACTCTCATAATTAAATCATTTGGGCTCAAGTTATGTAATAACGTACATGTAACTATGTACATCTTAAGAAAACTTCTTCTTTTTTTATATAAACACGACATAACGAATGAAGATTACTCTTGTGACGGACATTCCTATTATTG 5200
      K N K K Y H L Y F Q R F C F * F I K R Q S Y * N L L G S N L V N N C T C Q Y M Y F E K S S S F I Y K H Q I A * K * H S C Q R Y P Y Y   F6
     R I K R T I Y I F S G S V F S F F R A N L T N I * Y V R T * Y M I A H V N I C T S N K Q L L F F I N T S Y Q K S R I L V S G T L I I V  F5
     G * K E Q L T F L V A Q F L V L F D Q T S L I L K T F G L E I C * Q M Y M S V H L I R K F F F F Y I Q A T N S V E L S F V A Q L S L L   F4

      W * T S S T N N I V Y * Q I C Y K L P * S Y G E K N W S M N I S S I L R R R R A R A T P C V V P K S V T F * A M V T S S S F A A * S   F1
     I G R H L V Q I I L C I D K S V I S Y H E V M E K K I G Q * T F Q A S L E G E G P G Q L L A L C Q S P S H F E Q W * L R V V L L L N L  F2
     L V D I * Y K * Y C V L T N L L * V T M K L W R K K L V N E H F K H P * K E K G Q G N S L R C A K V R H I L S N G N F E * F C C L I F  F3
5201 ATTGGTAGACATCTAGTACAAATAATATTGTGTATTGACAAATCTGTTATAAGTTACCATGAAGTTATGGAGAAAAAAATTGGTCAATGAACATTTCAAGCATCCTTAGAAGGAGAAGGGCCAGGGCAACTCCTTGCGTTGTGCCAAAGTCCGTCACATTTTGAGCAATGGTAACTTCGAGTAGTTTTGCTGCTTAATCT 5400
     ----:----|----:----|----:----|----:----|----:----|----:----|----:----|----:----|----:----|----:----|----:----|----:----|----:----|----:----|----:----|----:----|----:----|----:----|----:----|----:----|
5201 TAACCATCTGTAGATCATGTTTATTATAACACATAACTGTTTAGACAATATTCAATGGTACTTCAATACCTCTTTTTTTAACCAGTTACTTGTAAAGTTCGTAGGAATCTTCCTCTTCCCGGTCCCGTTGAGGAACGCAACACGGTTTCAGGCAGTGTAAAACTCGTTACCATTGAAGCTCATCAAAACGACGAATTAGA 5400
     C Q Y V D L V F L I T Y Q C I Q * L N G H L * P S F F Q D I F M E L M R L L L L A L A V G Q T T G F D T V N Q A I T V E L L K A A * D  F6
      N T S M * Y L Y Y Q T N V F R N Y T V M F N H L F F N T L S C K L C G * F S F P W P L E K R Q A L T R * M K L L P L K S Y N Q Q K I   F5
      M P L C R T C I I N H I S L D T I L * W S T I S F F I P * H V N * A D K S P S P G P C S R A N H W L G D C K S C H Y S R T T K S S L R F4

     S N F R T G V E P V Y P Q D V T E I L C H I M L P S I M P T E I A K E L V G G F I S F S T Q P S N C L W S A W I L K D L L N M F Y D F   F1
      L I L G L V * S Q C T H R M S R R F C V T S C F L A * C R R R S R K S L L E A S F P S P L N P P T V C G P L G S S R I S * T C S M T L  F2
      * F * D W C R A S V P T G C H G D S V S H H A S * H N A D G D R E R A C W R L H F L L H S T L Q L S V V R L D P Q G S P E H V L * L   F3
5401 TCTAATTTTAGGACTGGTGTAGAGCCAGTGTACCCACAGGATGTCACGGAGATTCTGTGTCACATCATGCTTCCTAGCATAATGCCGACGGAGATCGCGAAAGAGCTTGTTGGAGGCTTCATTTCCTTCTCCACTCAACCCTCCAACTGTCTGTGGTCCGCTTGGATCCTCAAGGATCTCCTGAACATGTTCTATGACTT 5600
     ----:----|----:----|----:----|----:----|----:----|----:----|----:----|----:----|----:----|----:----|----:----|----:----|----:----|----:----|----:----|----:----|----:----|----:----|----:----|----:----|
5401 AGATTAAAATCCTGACCACATCTCGGTCACATGGGTGTCCTACAGTGCCTCTAAGACACAGTGTAGTACGAAGGATCGTATTACGGCTGCCTCTAGCGCTTTCTCGAACAACCTCCGAAGTAAAGGAAGAGGTGAGTTGGGAGGTTGACAGACACCAGGCGAACCTAGGAGTTCCTAGAGGACTTGTACAAGATACTGAA 5600
      E L K L V P T S G T Y G C S T V S I R H * M M S G L M I G V S I A F S S T P P K M E K E V * G E L Q R H D A Q I R L S R R F M N * S K  F6
     K * N * S Q H L A L T G V P H * P S E T D C * A E * C L A S P S R S L A Q Q L S * K R R W E V R W S D T T R K S G * P D G S C T R H S   F5
      R I K P S T Y L W H V W L I D R L N Q T V D H K R A Y H R R L D R F L K N S A E N G E G S L G G V T Q P G S P D E L I E Q V H E I V   F4

      V E I I R P G D I C K V S L Q Q V P H G D C I Y L V D L H L V N L G V V C S V Y G P E A R E Q D Q H F F Q P F T S F L I F D * L Q D N  F1
       W R * L G Q E T Y A K * V F N R F P M E T A F T L * T F T S S T L G W C A L Y T D R R R E N R T N T S F S L S R L S S S L T S C K T   F2
     C G D N * A R R H M Q S E S S T G S P W R L H L P C R P S P R Q P W G G V L C I R T G G E R T G P T L L S A F H V F P H L * L A A R Q  F3
5601 TGTGGAGATAATTAGGCCAGGAGACATATGCAAAGTGAGTCTTCAACAGGTTCCCCATGGAGACTGCATTTACCTTGTAGACCTTCACCTCGTCAACCTTGGGGTGGTGTGCTCTGTATACGGACCGGAGGCGAGAGAACAGGACCAACACTTCTTTCAGCCTTTCACGTCTTTCCTCATCTTTGACTAGCTGCAAGACA 5800
     ----:----|----:----|----:----|----:----|----:----|----:----|----:----|----:----|----:----|----:----|----:----|----:----|----:----|----:----|----:----|----:----|----:----|----:----|----:----|----:----|
5601 ACACCTCTATTAATCCGGTCCTCTGTATACGTTTCACTCAGAAGTTGTCCAAGGGGTACCTCTGACGTAAATGGAACATCTGGAAGTGGAGCAGTTGGAACCCCACCACACGAGACATATGCCTGGCCTCCGCTCTCTTGTCCTGGTTGTGAAGAAAGTCGGAAAGTGCAGAAAGGAGTAGAAACTGATCGACGTTCTGT 5800
       T S I I L G P S M H L T L R * C T G W P S Q M * R T S R * R T L R P T T H E T Y P G S A L S C S W C K K * G K V D K R M K S * S C S  F6
      Q P S L * A L L C I C L S D E V P E G H L S C K G Q L G E G R * G Q P P T S Q I R V P P S L V P G V S R E A K * T K G * R Q S A A L C  F5
     K H L Y N P W S V Y A F H T K L L N G M S V A N V K Y V K V E D V K P H H A R Y V S R L R S F L V L V E K L R E R R E E D K V L Q L V  F4

      V I V F Q G E Q C P R E I T R H H Q G G S * S K C R Q Q M I I K L F L C F L V A L L H I L S N F P C L D S G G L T Y D Q L L K K R A   F1
     M S L F F K A N N A R A K L P G I I K E G P S P S V D N K * S S N F F S A F S * L S F T S C R I S H V W I L A V S H M I S F L K N E P  F2
      C H C F S R R T M P A R N Y Q A S S R R V L V Q V * T T N D H Q T F S L L S R S S P S H L V E F P M S G F W R S H I * S A S * K T S Q F3
5801 ATGTCATTGTTTTTCAAGGCGAACAATGCCCGCGCGAAATTACCAGGCATCATCAAGGAGGGTCCTAGTCCAAGTGTAGACAACAAATGATCATCAAACTTTTTCTCTGCTTTCTCGTAGCTCTCCTTCACATCTTGTCGAATTTCCCATGTCTGGATTCTGGCGGTCTCACATATGATCAGCTTCTTAAAAAACGAGCC 6000
     ----:----|----:----|----:----|----:----|----:----|----:----|----:----|----:----|----:----|----:----|----:----|----:----|----:----|----:----|----:----|----:----|----:----|----:----|----:----|----:----|
5801 TACAGTAACAAAAAGTTCCGCTTGTTACGGGCGCGCTTTAATGGTCCGTAGTAGTTCCTCCCAGGATCAGGTTCACATCTGTTGTTTACTAGTAGTTTGAAAAAGAGACGAAAGAGCATCGAGAGGAAGTGTAGAACAGCTTAAAGGGTACAGACCTAAGACCGCCAGAGTGTATACTAGTCGAAGAATTTTTTGCTCGG 6000
     L T M T K * P S C H G R S I V L C * * P P D * D L H L C C I I M L S K R Q K R T A R R * M K D F K G H R S E P P R V Y S * S R L F R A  F6
      H * Q K E L R V I G A R F * W A D D L L T R T W T Y V V F S * * V K E R S E R L E G E C R T S N G M D P N Q R D * M H D A E * F V L   F5
      I D N N K L A F L A R A F N G P M M L S P G L G L T S L L H D D F K K E A K E Y S E K V D Q R I E W T Q I R A T E C I I L K K F F S G F4

     N V Y V R V G C I Y Q L L S R I * L Q D W T R F H T L * H L A K L G W R Q V I R V D E Y V V C Y G I R L L V C S G D P E T A * F R L S   F1
      M F M S A W V A S I N F S R G S D C R T G R D F T P C D I S P S W A G D R S S G L M S M * F A M A S V S S Y V L V I L K L P S S D L A  F2
      C L C P R G L H L S T S L A D L I A G L D E I S H L V T S R Q V G L E T G H Q G * * V C S L L W H P S P R M F W * S * N C L V P T *   F3
6001 AATGTTTATGTCCGCGTGGGTTGCATCTATCAACTTCTCTCGCGGATCTGATTGCAGGACTGGACGAGATTTCACACCTTGTGACATCTCGCCAAGTTGGGCTGGAGACAGGTCATCAGGGTTGATGAGTATGTAGTTTGCTATGGCATCCGTCTCCTCGTATGTTCTGGTGATCCTGAAACTGCCTAGTTCCGACTTAG 6200
     ----:----|----:----|----:----|----:----|----:----|----:----|----:----|----:----|----:----|----:----|----:----|----:----|----:----|----:----|----:----|----:----|----:----|----:----|----:----|----:----|
6001 TTACAAATACAGGCGCACCCAACGTAGATAGTTGAAGAGAGCGCCTAGACTAACGTCCTGACCTGCTCTAAAGTGTGGAACACTGTAGAGCGGTTCAACCCGACCTCTGTCCAGTAGTCCCAACTACTCATACATCAAACGATACCGTAGGCAGAGGAGCATACAAGACCACTAGGACTTTGACGGATCAAGGCTGAATC 6200
      L T * T R T P Q M * * S R E R I Q N C S Q V L N * V K H C R A L N P Q L C T M L T S S Y T T Q * P M R R R T H E P S G S V A * N R S L  F6
     W H K H G R P N C R D V E R A S R I A P S S S I E C R T V D R W T P S S V P * * P Q H T H L K S H C G D G R I N Q H D Q F Q R T G V *   F5
      I N I D A H T A D I L K E R P D S Q L V P R S K V G Q S M E G L Q A P S L D D P N I L I Y N A I A D T E E Y T R T I R F S G L E S K   F4

      R L S G S M A Q S T * E F R T T S L E A T I C P V K L L I N H G I V K P K I V P P P * I S N V D * K S T L S * C S P L A L Y P F Q E K  F1
       V S R V A W H K V H R N F E P L P W R P P S A L S S F S S I M E L * N R R L C L L H E F P M L T E S P L F R S A A L S H S I P S R R   F2
     P S L G * H G T K Y I G I S N H F P G G H H L P C Q A S H Q S W N C K T E D C A S S M N F Q C * L K V H S F V V Q P S R T L S L P G E   F3
6201 CCGTCTCTCGGGTAGCATGGCACAAAGTACATAGGAATTTCGAACCACTTCCCTGGAGGCCACCATCTGCCCTGTCAAGCTTCTCATCAATCATGGAATTGTAAAACCGAAGATTGTGCCTCCTCCATGAATTTCCAATGTTGACTGAAAGTCCACTCTTTCGTAGTGCAGCCCTCTCGCACTCTATCCCTTCCAGGAGA 6400
     ----:----|----:----|----:----|----:----|----:----|----:----|----:----|----:----|----:----|----:----|----:----|----:----|----:----|----:----|----:----|----:----|----:----|----:----|----:----|----:----|
6201 GGCAGAGAGCCCATCGTACCGTGTTTCATGTATCCTTAAAGCTTGGTGAAGGGACCTCCGGTGGTAGACGGGACAGTTCGAAGAGTAGTTAGTACCTTAACATTTTGGCTTCTAACACGGAGGAGGTACTTAAAGGTTACAACTGACTTTCAGGTGAGAAAGCATCACGTCGGGAGAGCGTGAGATAGGGAAGGTCCTCT 6400
       R R E P L M A C L V Y S N R V V E R S A V M Q G T L S R M L * P I T F G F I T G G G H I E L T S Q F D V R E Y H L G R A S * G K W S  F6
      G D R P Y C P V F Y M P I E F W K G P P W W R G Q * A E * * D H F Q L V S S Q A E E M F K W H Q S F T W E K T T C G E R V R D R G P S  F5
     A T E R T A H C L T C L F K S G S G Q L G G D A R D L K E D I M S N Y F R L N H R R W S N G I N V S L G S K R L A A R E C E I G E L L  F4

      N R * S A I V L V C N C L H Q R T G D K C G F R L I R F I N G Q P L V P K G N L T L D D R E C N P E G L V R E K A V S S L P E Y R D   F1
     R I D E A R L F S S A I A S T R G R V I N V D S G L S G S * T V N L S S P R V T S H L M T A N A T L K A L S G R R R S L A S L N T E M  F2
      E S M K R D C S R L Q L P P P E D G * * M W I Q A Y P V H K R S T S R P Q G * P H T * * P R M Q P * R P C Q G E G G L * P P * I Q R C F3
6401 AGAATCGATGAAGCGCGATTGTTCTCGTCTGCAATTGCCTCCACCAGAGGACGGGTGATAAATGTGGATTCAGGCTTATCCGGTTCATAAACGGTCAACCTCTCGTCCCCAAGGGTAACCTCACACTTGATGACCGCGAATGCAACCCTGAAGGCCTTGTCAGGGAGAAGGCGGTCTCTAGCCTCCCTGAATACAGAGAT 6600
     ----:----|----:----|----:----|----:----|----:----|----:----|----:----|----:----|----:----|----:----|----:----|----:----|----:----|----:----|----:----|----:----|----:----|----:----|----:----|----:----|
6401 TCTTAGCTACTTCGCGCTAACAAGAGCAGACGTTAACGGAGGTGGTCTCCTGCCCACTATTTACACCTAAGTCCGAATAGGCCAAGTATTTGCCAGTTGGAGAGCAGGGGTTCCCATTGGAGTGTGAACTACTGGCGCTTACGTTGGGACTTCCGGAACAGTCCCTCTTCCGCCAGAGATCGGAGGGACTTATGTCTCTA 6600
      F F R H L A I T R T Q L Q R W W L V P S L H P N L S I R N M F P * G R T G L P L R V S S S R S H L G S P R T L S F A T E L R G S Y L S  F6
       S D I F R S Q E R R C N G G G S S P H Y I H I * A * G T * L R D V E R G W P Y G * V Q H G R I C G Q L G Q * P S P P R * G G Q I C L  F5
      L I S S A R N N E D A I A E V L P R T I F T S E P K D P E Y V T L R E D G L T V E C K I V A F A V R F A K D P L L R D R A E R F V S I F4

     V P H S I C T V L D G G R Y F V V L F R I K S N F Q E S F C N I N V K F F Q S F C * C M P V R P C H S F E I W E R N L N Q R V M W R R   F1
      S P I P S A P S L M V V V I S * F S S G L S P T F R S P S A I S M S S S S R V F A N A C P Y G H A T P L R F G N G I S T K G L C G D V F2
      P P F H L H R P * W W S L F R S S L Q D * V Q L S G V L L Q Y Q C Q V L P E F L L M H A R T A M P L L * D L G T E S Q P K G Y V E T  F3
6601 GTCCCCCATTCCATCTGCACCGTCCTTGATGGTGGTCGTTATTTCGTAGTTCTCTTCAGGATTAAGTCCAACTTTCAGGAGTCCTTCTGCAATATCAATGTCAAGTTCTTCCAGAGTTTTTGCTAATGCATGCCCGTACGGCCATGCCACTCCTTTGAGATTTGGGAACGGAATCTCAACCAAAGGGTTATGTGGAGACG 6800
     ----:----|----:----|----:----|----:----|----:----|----:----|----:----|----:----|----:----|----:----|----:----|----:----|----:----|----:----|----:----|----:----|----:----|----:----|----:----|----:----|
6601 CAGGGGGTAAGGTAGACGTGGCAGGAACTACCACCAGCAATAAAGCATCAAGAGAAGTCCTAATTCAGGTTGAAAGTCCTCAGGAAGACGTTATAGTTACAGTTCAAGAAGGTCTCAAAAACGATTACGTACGGGCATGCCGGTACGGTGAGGAAACTCTAAACCCTTGCCTTAGAGTTGGTTTCCCAATACACCTCTGC 6800
       T G W E M Q V T R S P P R * K T T R K L I L D L K * S D K Q L I L T L N K W L K Q * H M G T R G H W E K S I Q S R F R L W L T I H L R F6
      H G G N W R C R G Q H H D N N R L E R * S * T W S E P T R R C Y * H * T R G S N K S I C A R V A M G S R Q S K P V S D * G F P * T S V  F5
       D G M G D A G D K I T T T I E Y N E E P N L G V K L L G E A I D I D L E E L T K A L A H G Y P W A V G K L N P F P I E V L P N H P S  F4

      Q V Q W L V R * T T I L H L P A F L D R R V V S V D C V P S H P K T N T A R H V * L L T L C K L V R G F Q Y V D A R A L E C I I L F F F1
       K S N G S Y D E P P Y S T C L P S L T G V W * V * T V S P P I R K R T L P G M Y D F S H S V S W * G G F N M S T P E L L S A S Y C S  F2
     S S P M A R T M N H H T P P A C L P * Q A C G E C R L C P L P S E N E H C Q A C M T S H T L * A G E G V S I C R R Q S S * V H H T V L  F3
6801 TCAAGTCCAATGGCTCGTACGATGAACCACCATACTCCACCTGCCTGCCTTCCTTGACAGGCGTGTGGTGAGTGTAGACTGTGTCCCCTCCCATCCGAAAACGAACACTGCCAGGCATGTATGACTTCTCACACTCTGTAAGCTGGTGAGGGGGTTTCAATATGTCGACGCCAGAGCTCTTGAGTGCATCATACTGTTCT 7000
     ----:----|----:----|----:----|----:----|----:----|----:----|----:----|----:----|----:----|----:----|----:----|----:----|----:----|----:----|----:----|----:----|----:----|----:----|----:----|----:----|
6801 AGTTCAGGTTACCGAGCATGCTACTTGGTGGTATGAGGTGGACGGACGGAAGGAACTGTCCGCACACCACTCACATCTGACACAGGGGAGGGTAGGCTTTTGCTTGTGACGGTCCGTACATACTGAAGAGTGTGAGACATTCGACCACTCCCCCAAAGTTATACAGCTGCGGTCTCGAGAACTCACGTAGTATGACAAGA 7000
       * T W H S T R H V V M S W R G A K R S L R T T L T S Q T G E W G F V F V A L C T H S R V S Q L S T L P N * Y T S A L A R S H M M S N  F6
      D L G I A R V I F W W V G G A Q R G Q C A H P S H L S H G R G D S F S C Q W A H I V E * V R Y A P S P T E I H R R W L E Q T C * V T R  F5
     T L D L P E Y S S G G Y E V Q R G E K V P T H H T Y V T D G G M R F R V S G P M Y S K E C E T L Q H P P K L I D V G S S K L A D Y Q E  F4

      V A S F R C H C G D S H G Q A V V N S Q Y N L L V I P C R H H L * K N R K D V S K L P Y L H I K R N N Q L S I F W * F F I T * V K F  F1
     L * R V F D V I V V T L M A K Q L S T V S T T F L S F H V D I T C R K I E K M * V N C H I C I S R G I I N Y Q S F G N F S L H K S N F  F2
      C S E F S M S L W * L S W P S S C Q Q S V Q P S C H S M S T S P V E K * K R C K * I A I S A Y Q E E * S I I N L L V I F H Y I S Q I S F3
7001 TTGTAGCGAGTTTTCGATGTCATTGTGGTGACTCTCATGGCCAAGCAGTTGTCAACAGTCAGTACAACCTTCTTGTCATTCCATGTCGACATCACCTGTAGAAAAATAGAAAAGATGTAAGTAAATTGCCATATCTGCATATCAAGAGGAATAATCAATTATCAATCTTTTGGTAATTTTTCATTACATAAGTCAAATTT 7200
     ----:----|----:----|----:----|----:----|----:----|----:----|----:----|----:----|----:----|----:----|----:----|----:----|----:----|----:----|----:----|----:----|----:----|----:----|----:----|----:----|
7001 AACATCGCTCAAAAGCTACAGTAACACCACTGAGAGTACCGGTTCGTCAACAGTTGTCAGTCATGTTGGAAGAACAGTAAGGTACAGCTGTAGTGGACATCTTTTTATCTTTTCTACATTCATTTAACGGTATAGACGTATAGTTCTCCTTATTAGTTAATAGTTAGAAAACCATTAAAAAGTAATGTATTCAGTTTAAA 7200
     K T A L K R H * Q P S E * P W A T T L L * Y L R R T M G H R C * R Y F F L F S T L L N G Y R C I L L F L * N D I K Q Y N K M V Y T L N  F6
      Q L S N E I D N H H S E H G L L Q * C D T C G E Q * E M D V D G T S F Y F L H L Y I A M D A Y * S S Y D I I L R K T I K * * M L * I   F5
      K Y R T K S T M T T V R M A L C N D V T L V V K K D N W T S M V Q L F I S F I Y T F Q W I Q M D L P I I L * * D K P L K E N C L D F K F4

     L N Y Y M * I I A I L P L K L I S L L S N K V M * S C Y N I C Q F H Q S K E F I * T I L C S P F * D * N L G * K Y E D S Y V L C Y I E   F1
      * I I T C R S L Q Y F H * N * F L S S A T K L C N L A T I Y A N F T K V K N L Y E Q Y S V H L F E I K T L V K N T K T H M C Y A I * R F2
      K L L H V D H C N T S I E I N F S P Q Q Q S Y V I L L Q Y M P I S P K * R I Y M N N T L F T F L R L K P W L K I R R L I C A M L Y R  F3
7201 CTAAATTATTACATGTAGATCATTGCAATACTTCCATTGAAATTAATTTCTCTCCTCAGCAACAAAGTTATGTAATCTTGCTACAATATATGCCAATTTCACCAAAGTAAAGAATTTATATGAACAATACTCTGTTCACCTTTTTGAGATTAAAACCTTGGTTAAAAATACGAAGACTCATATGTGCTATGCTATATAGA 7400
     ----:----|----:----|----:----|----:----|----:----|----:----|----:----|----:----|----:----|----:----|----:----|----:----|----:----|----:----|----:----|----:----|----:----|----:----|----:----|----:----|
7201 GATTTAATAATGTACATCTAGTAACGTTATGAAGGTAACTTTAATTAAAGAGAGGAGTCGTTGTTTCAATACATTAGAACGATGTTATATACGGTTAAAGTGGTTTCATTTCTTAAATATACTTGTTATGAGACAAGTGGAAAAACTCTAATTTTGGAACCAATTTTTATGCTTCTGAGTATACACGATACGATATATCT 7400
      R F * * M Y I M A I S G N F N I E R R L L L T I Y D Q * L I H W N * W L L S N I H V I S Q E G K Q S * F R P * F Y S S E Y T S H * I S F6
     E L N N C T S * Q L V E M S I L K E G * C C L * T I K S C Y I G I E G F Y L I * I F L V R N V K K L N F G Q N F I R L S M H A I S Y L  F5
      * I I V H L D N C Y K W Q F * N R E E A V F N H L R A V I Y A L K V L T F F K Y S C Y E T * R K S I L V K T L F V F V * I H * A I Y   F4

      I * R E F Q S A F I K S Q F T K H D R S * * L I T F H L S I V * V R G S D Q I N V Q * T S T F H P G I R Q L K C I I I S K * Y * T Y L F1
       F D E S F K V H S S N L N L Q N M I G V N N S S L F T S Q S F R * E D L I K * M Y S E P V L F I Q G L D N * S V L * Y Q N D I K H T   F2
     D L T R V S K C I H Q I S I Y K T * * E L I T H H F S P L N R L G K R I * S N K C T V N Q Y F S S R D * T I E V Y Y N I K M I L N I P   F3
7401 GATTTGACGAGAGTTTCAAAGTGCATTCATCAAATCTCAATTTACAAAACATGATAGGAGTTAATAACTCATCACTTTTCACCTCTCAATCGTTTAGGTAAGAGGATCTGATCAAATAAATGTACAGTGAACCAGTACTTTTCATCCAGGGATTAGACAATTGAAGTGTATTATAATATCAAAATGATATTAAACATACC 7600
     ----:----|----:----|----:----|----:----|----:----|----:----|----:----|----:----|----:----|----:----|----:----|----:----|----:----|----:----|----:----|----:----|----:----|----:----|----:----|----:----|
7401 CTAAACTGCTCTCAAAGTTTCACGTAAGTAGTTTAGAGTTAAATGTTTTGTACTATCCTCAATTATTGAGTAGTGAAAAGTGGAGAGTTAGCAAATCCATTCTCCTAGACTAGTTTATTTACATGTCACTTGGTCATGAAAAGTAGGTCCCTAATCTGTTAACTTCACATAATATTATAGTTTTACTATAATTTGTATGG 7600
       I Q R S N * L A N M L D * N V F C S L L * Y S M V K * R E I T * T L P D S * I F T C H V L V K * G P I L C N F H I I I D F H Y * V Y   F6
      S K V L T E F H M * * I E I * L V H Y S N I V * * K E G R L R K P L L I Q D F L H V T F W Y K E D L S * V I S T Y * L I L I I N F M G  F5
     L N S S L K L T C E D F R L K C F M I P T L L E D S K V E * D N L Y S S R I L Y I Y L S G T S K M W P N S L Q L T N Y Y * F S I L C V   F4
```

```
 F  T  S  W  V  I  A  I  L  Q  T  L  C  *  K  E  E  K  E  I  I  L  F  L  L  L  T  I  T  E  V  T  D  E  R  S  S  F  M  C  S  L  P  C  P  I  S  S  N  V  F  Q  T  A  L  C  I  P  D  F  L  E  A  S  S  W   F1
S  L  H  L  G  S  L  L  S  C  R  L  S  V  R  R  K  K  R  R  S  F  F  F  S  F  *  R  S  Q  K  S  L  M  N  E  A  A  S  C  V  L  F  L  A  Q  S  L  R  T  S  F  K  L  L  F  A  F  Q  T  F  L  K  L  V  P  G   F2
 L  Y  I  L  G  H  C  Y  L  A  D  S  L  L  E  G  R  K  G  D  H  S  F  S  P  S  D  D  H  R  S  H  *  *  T  K  Q  L  H  V  F  S  S  L  P  N  L  F  E  R  L  S  N  C  S  L  H  S  R  L  S  *  S  *  F  L  V   F3
7601 TCTTTACATCTTGGGTCATTGCTATCTTGCAGACTCTCTGTTAGAAGGAAGAAAAGGAGATCATTCTTTTTCTCCTTCTGACGATCACAGAAGTCACTGATGAACGAAGCAGCTTCATGTGTTCTCTTCCTTGCCCAATCTCTTCGAACGTCTTTCAAACTGCTCTTTGCATTCCAGACTTTCTTGAAGCTAGTTCCTGG 7800
     ----:----|----:----|----:----|----:----|----:----|----:----|----:----|----:----|----:----|----:----|----:----|----:----|----:----|----:----|----:----|----:----|----:----|----:----|----:----|----:----|
7601 AGAAATGTAGAACCCAGTAACGATAGAACGTCTGAGAGACAATCTTCCTTCTTTTCCTCTAGTAAGAAAAAGAGGAAGACTGCTAGTGTCTTCAGTGACTACTTGCTTCGTCGAAGTACACAAGAGAAGGAACGGGTTAGAGAAGCTTGCAGAAAGTTTGACGAGAAACGTAAGGTCTGAAAGAACTTCGATCAAGGACC 7800
R  K  V  D  Q  T  M  A  I  K  C  V  R  Q  *  F  S  S  F  S  I  M  R  K  R  R  R  V  I  V  S  T  V  S  S  R  L  L  K  M  H  E  R  G  Q  G  I  E  E  F  T  K  *  V  A  R  Q  M  G  S  K  R  S  A  L  E  Q   F6
 R  *  M  K  P  *  Q  *  R  A  S  E  R  N  S  P  L  F  P  S  *  E  K  E  G  E  S  S  *  L  L  *  Q  H  V  F  C  S  *  T  N  E  E  K  G  L  R  K  S  R  R  E  F  Q  E  K  C  E  L  S  E  Q  L  *  N  R   F5
  E  K  C  R  P  D  N  S  D  Q  L  S  E  T  L  L  F  F  L  L  D  N  K  K  E  K  Q  R  D  C  F  D  S  I  F  S  A  A  E  H  T  R  K  R  A  W  D  R  R  V  D  K  L  S  S  K  A  N  W  V  K  K  F  S  T  G  P   F4

S  R  I  F  F  G  K  Q  L  F  G  G  S  C  Y  C  R  P  *  H  W  G  G  *  D  R  W  G  W  R  W  R  R  R  L  E  L  W  Q  E  *  *  I  M  C  C  M  G  I  T  D  I  A  G  R  A  V  L  *  K  K  L  Q  M  G  E  F   F1
 P  G  F  F  S  A  N  S  S  L  V  A  V  A  T  V  G  P  N  T  G  V  V  E  T  D  G  D  G  G  G  G  G  G  L  N  S  G  R  S  D  E  L  C  A  A  W  A  S  Q  T  *  Q  E  G  L  C  S  E  K  S  C  K  W  A  S  F   F2
  Q  D  F  F  R  Q  T  A  L  W  W  Q  L  L  L  S  A  L  T  L  G  W  L  R  Q  M  G  M  E  V  E  A  E  A  *  T  L  A  G  V  M  N  Y  V  L  H  G  H  H  R  H  S  R  K  G  C  A  L  K  K  A  A  N  G  R  V   F3
7801 TCCAGGATTTTTTTCGGCAAACAGCTCTTTGGTGGCAGTTGCTACTGTCGGCCCTAACACTGGGGTGGTTGAGACAGATGGGGATGGAGGTGGAGGCGGAGGCTTGAACTCTGGCAGGAGTGATGAATTATGTGCTGCATGGGCATCACAGACATAGCAGGAAGGGCTGTGCTCTGAAAAAAGCTGCAAATGGGCGAGTT 8000
     ----:----|----:----|----:----|----:----|----:----|----:----|----:----|----:----|----:----|----:----|----:----|----:----|----:----|----:----|----:----|----:----|----:----|----:----|----:----|----:----|
7801 AGGTCCTAAAAAAAGCCGTTTGTCGAGAAACCACCGTCAACGATGACAGCCGGGATTGTGACCCCACCAACTCTGTCTACCCCTACCTCCACCTCCGCCTCCGAACTTGAGACCGTCCTCACTACTTAATACACGACGTACCCGTAGTGTCTGTATCGTCCTTCCCGACACGAGACTTTTTTCGACGTTTACCCGCTCAA 8000
 D  L  I  K  K  P  L  C  S  K  P  P  L  Q  *  Q  R  G  *  C  Q  P  P  Q  S  L  H  P  H  L  H  L  R  L  S  S  S  Q  C  S  H  H  I  I  H  Q  M  P  M  V  S  M  A  P  L  A  T  S  Q  F  F  S  C  I  P  S  N   F6
T  W  S  K  K  R  C  V  A  R  Q  H  C  N  S  S  D  A  R  V  S  P  H  N  L  C  I  P  I  S  T  S  A  S  A  Q  V  R  A  P  T  I  F  *  T  S  C  P  C  *  L  C  L  L  F  P  Q  A  R  F  F  A  A  F  P  R  T   F5
  G  P  N  K  E  A  F  L  E  K  T  A  T  A  V  T  P  G  L  V  P  T  T  S  V  S  P  S  P  P  P  P  P  P  K  F  E  P  L  L  S  S  N  H  A  A  H  A  D  C  V  Y  C  S  P  S  H  E  S  F  L  Q  L  H  A  L   F4

 P  L  V  L  L  S  L  L  H  G  L  V  G  S  M  Q  L  V  P  V  C  V  N  K  K  *  R  Q  N  N  V  C  F  N  V  Q  V  H  V  *  H  H  *  H  V  T  L  G  I  A  K  F  V  W  A  C  T  F  V  G  L  C  H  *  L  I  A   F1
  P  W  Y  C  F  P  C  C  M  A  W  *  D  R  C  S  W  C  L  C  A  *  I  K  N  K  D  K  I  M  F  V  L  M  Y  K  Y  M  Y  D  I  I  D  M  *  P  W  A  L  P  S  L  F  G  H  V  H  L  L  G  C  V  T  N  *  L   F2
S  L  G  T  A  F  P  A  A  W  L  G  R  I  D  A  V  G  A  C  V  R  E  *  K  I  K  T  K  *  C  L  F  *  C  T  S  T  C  M  T  S  L  T  C  N  P  G  H  C  Q  V  C  L  G  M  Y  I  C  W  A  V  S  L  I  N  C   F3
8001 TCCCTTGGTACTGCTTTCCCTGCTGCATGGCTTGGTAGGATCGATGCAGTTGGTGCCTGTGTGCGTGAATAAAAAATAAAGACAAAATAATGTTTGTTTTAATGTACAAGTACATGTATGACATCATTGACATGTAACCCTGGGCATTGCCAAGTTTGTTTGGGCATGTACATTTGTTGGGCTGTGTCACTAATTAATTG 8200
     ----:----|----:----|----:----|----:----|----:----|----:----|----:----|----:----|----:----|----:----|----:----|----:----|----:----|----:----|----:----|----:----|----:----|----:----|----:----|----:----|
8001 AGGGAACCATGACGAAAGGGACGACGTACCGAACCATCCTAGCTACGTCAACCACGGACACACGCACTTATTTTTTATTTCTGTTTTATTACAAACAAAATTACATGTTCATGTACATACTGTAGTAACTGTACATTGGGACCCGTAACGGTTCAAACAAACCCGTACATGTAAACAACCCGACACAGTGATTAATTAAC 8200
 G  K  T  S  S  E  R  S  C  P  K  T  P  D  I  C  N  T  G  T  H  T  F  L  F  Y  L  C  F  L  T  Q  K  L  T  C  T  C  T  H  C  *  Q  C  T  V  R  P  M  A  L  N  T  Q  A  H  V  N  T  P  S  H  *  *  N  I   F6
E  R  P  V  A  K  G  A  A  H  S  P  L  I  S  A  T  P  A  Q  T  R  S  Y  F  I  F  V  F  Y  H  K  N  *  H  V  L  V  H  I  V  D  N  V  H  L  G  P  C  Q  W  T  Q  K  P  M  Y  M  Q  Q  A  T  D  S  I  L  Q   F5
K  G  Q  Y  Q  K  G  Q  Q  M  A  Q  Y  S  R  H  L  Q  H  R  H  A  H  I  F  F  L  S  L  I  I  N  T  K  I  Y  L  Y  M  Y  S  M  M  S  M  Y  G  Q  A  N  G  L  K  N  P  C  T  C  K  N  P  Q  T  V  L  *  N   F4

 A  L  V  *  N  R  H  Q  H  P  S  T  Q  D  *  F  T  E  L  C  *  T  F  L  S  *  I  S  P  S  *  Y  T  K  N  V  L  F  I  R  *  S  F  I  G  V  C  L  L  L  Y  E  G  A  H  L  E  Y  Q  *  L  C  I  T  L  T   F1
L  H  *  C  K  T  D  T  N  I  P  L  R  K  I  N  L  L  N  F  A  R  L  S  S  H  E  S  V  Q  A  N  T  Q  K  M  S  Y  S  L  G  K  A  S  L  V  Y  A  C  Y  C  M  R  V  H  I  L  N  I  N  N  C  V  *  L  *  P   F2
 C  T  S  V  K  Q  T  P  T  S  L  Y  A  R  L  I  Y  *  T  L  L  D  F  P  L  M  N  Q  S  K  L  I  H  K  K  C  L  I  H  *  V  K  L  H  W  C  M  L  V  I  V  *  G  C  T  S  *  I  S  I  I  V  Y  N  F  D  Q   F3
8201 CTGCACTAGTGTAAAACAGACACCAACATCCCTCTACGCAAGATTAATTTACTGAACTTTGCTAGACTTTCCTCTCATGAATCAGTCCAAGCTAATACACAAAAAATGTCTTATTCATTAGGTAAAGCTTCATTGGTGTATGCTTGTTATTGTATGAGGGTGCACATCTTGAATATCAATAATTGTGTATAACTTTGACC 8400
     ----:----|----:----|----:----|----:----|----:----|----:----|----:----|----:----|----:----|----:----|----:----|----:----|----:----|----:----|----:----|----:----|----:----|----:----|----:----|----:----|
8201 GACGTGATCACATTTTGTCTGTGGTTGTAGGGAGATGCGTTCTAATTAAATGACTTGAAACGATCTGAAAGGAGAGTACTTAGTCAGGTTCGATTATGTGTTTTTTACAGAATAAGTAATCCATTTCGAAGTAACCACATACGAACAATAACATACTCCCACGTGTAGAACTTATAGTTATTAACACATATTGAAACTGG 8400
A  A  S  T  Y  F  L  C  W  C  G  E  V  C  S  *  N  V  S  S  Q  *  V  K  R  E  H  I  L  G  L  *  Y  V  F  F  T  K  N  M  L  Y  L  K  M  P  T  H  K  N  N  Y  S  P  A  C  R  S  Y  *  Y  N  H  I  V  K  V   F6
 Q  V  L  T  F  C  V  G  V  D  R  *  A  L  N  I  *  Q  V  K  S  S  K  G  R  M  F  *  D  L  S  I  C  L  F  H  R  I  *  *  T  F  S  *  Q  H  I  S  T  I  T  H  P  H  V  D  Q  I  D  I  I  T  Y  L  K  S   F5
  S  C  *  H  L  V  S  V  L  M  G  R  R  L  I  L  K  S  F  K  A  L  S  E  E  *  S  D  T  W  A  L  V  C  F  I  D  *  E  N  P  L  A  E  N  T  Y  A  Q  *  Q  I  L  T  C  M  K  F  I  L  L  Q  T  Y  S  Q  G   F4

N  K  A  M  C  T  A  S  V  E  T  H  L  H  L  L  H  M  I  E  G  *  I  L  S  P  C  S  S  S  K  L  M  *  *  M  A  N  N  S  A  L  D  L  S  N  L  D  L  F  L  E  E  L  T  T  S  P  H  I  L  H  I  I  P  R  A   F1
 T  K  L  C  V  L  Q  A  L  K  L  T  C  I  Y  Y  T  *  *  R  D  E  Y  Y  R  H  A  H  R  P  N  *  C  D  R  W  P  I  T  L  L  W  I  C  P  T  S  T  C  S  W  K  N  *  R  L  L  H  I  S  C  T  S  Y  R  E  L   F2
  Q  S  Y  V  V  Y  C  K  R  *  N  S  P  A  F  I  T  H  D  R  G  M  N  T  I  A  M  L  I  V  Q  I  D  V  I  D  G  Q  *  L  C  F  G  S  V  Q  P  R  P  V  L  G  R  T  D  D  F  S  T  Y  P  A  H  H  T  E  S   F3
8401 AACAAAGCTATGTGTACTGCAAGCGTTGAAACTCACCTGCATTTATTACACATGATAGAGGGATGAATACTATCGCCATGCTCATCGTCCAAATTGATGTGATAGATGGCCAATAACTCTGCTTTGGATCTGTCCAACCTCGACCTGTTCTTGGAAGAACTGACGACTTCTCCACATATCCTGCACATCATACCGAGAGC 8600
     ----:----|----:----|----:----|----:----|----:----|----:----|----:----|----:----|----:----|----:----|----:----|----:----|----:----|----:----|----:----|----:----|----:----|----:----|----:----|----:----|
8401 TTGTTTCGATACACATGACGTTCGCAACTTTGAGTGGACGTAAATAATGTGTACTATCTCCCTACTTATGATAGCGGTACGAGTAGCAGGTTTAACTACACTATCTACCGGTTATTGAGACGAAACCTAGACAGGTTGGAGCTGGACAAGAACCTTCTTGACTGCTGAAGAGGTGTATAGGACGTGTAGTATGGCTCTCG 8600
 L  L  A  I  H  V  A  L  T  S  V  *  R  C  K  N  C  M  I  S  P  H  I  S  D  G  H  E  D  D  L  N  I  H  Y  I  A  L  L  E  A  K  S  R  D  L  R  S  R  N  K  S  S  S  V  V  E  G  C  I  R  C  M  M  G  L  A   F6
W  C  L  *  T  Y  Q  L  R  Q  F  E  G  A  N  I  V  C  S  L  P  I  F  V  I  A  M  S  M  T  W  I  S  T  I  S  P  W  Y  S  Q  K  P  D  T  W  G  R  G  T  R  P  L  V  S  S  K  E  V  Y  G  A  C  *  V  S  L   F5
  V  F  S  H  T  S  C  A  N  F  S  V  Q  M  *  *  V  H  Y  L  S  S  Y  *  R  W  A  *  R  G  F  Q  H  S  L  H  G  I  V  R  S  Q  I  Q  G  V  E  V  Q  E  Q  F  F  Q  R  S  R  W  M  D  Q  V  D  Y  R  S   F4

 F  R  C  S  S  I  L  D  R  P  V  *  X   F1
  S  D  A  P  P  S  W  T  G  L  Y  K  X   F2
F  Q  M  L  L  H  L  G  Q  A  C  I  K   F3
8601 TTTCAGATGCTCCTCCATCTTGGACAGGCCTGTATAAAA 8639
     ----:----|----:----|----:----|----:----
8601 AAAGTCTACGAGGAGGTAGAACCTGTCCGGACATATTTT 8639
  K  L  H  E  E  M  K  S  L  G  T  Y  F   F6
 K  *  I  S  R  W  R  P  C  A  Q  I  F   F5
S  E  S  A  G  G  D  Q  V  P  R  Y  L   F4
```

Matching transcriptomic data (TSA)

RAG1L

>TSA: IACU01023786.1:beg-end

5'3' Frame 3

EKSYMPGSVRFRMGGDTVYTHHTPVKEGRQVEYGGSSYEPLDLTSPHNPLVEIPFPNLKGVAWPYGHALAKTLEELDIDIAEGLLKVGLNPEENYEITTTIKDGADGMGDISVFREARDRLLPDKAFRVAFAVIKCEVTLGDERLTVYEPDKPESTFITRPLVEAIADENNRASSILLLEGIECERAALRKSGLSVNIGNSWRRHNLRFYNSMIDEKLDRADGGLQGSGSKFLCTLCHATRETAKSELGSFRITRTYEETDAIANYILINPDDLSPAQLGEMSQGVKSRPVLQSDPREKLIDATHADINIGSFFKKLIICETARIQTWEIRQDVKESYEKAEKKFDDHLLSTLGLGPSLMMPGNFARALFALKNNDIVLQLVKDEERRERLKEVLVLFSRLRSVYRAHHPKVDEVKVYKVNAVSMGNLLKTHFAYVSWPNYLHKVIEHVQEILEDPSGPQTVGGLSGEGNEASNKLFRDLRRHYARKHDVTQNLRDILWVHWLYTSPKIRRLSSKTTRSYHCSKCDGLWHNARSCPGPSPSKDA-NVH-PIFFSITSW-LITDLSIHNIICTRCLPMLLSLQAVFSLEVSNTAQIYFFFFKRILHVSMYMQ-CIELGFTKLILSTQDFLVLFQAVLFTLQEK-GL

Query: **TSA: IACU01023786.1**
Subject **BEXV01003119.1** **Length: 48415** **Number of Matches: 3**

```
Score                  Expect   Identities       Gaps        Strand
3578 bits(1937)        0.0      1937/1937(100%)              0/1937(0%)    Plus/Minus

Query  1      GTGAGAAGTCATACATGCCTGGCAGTGTTCGTTTTCGGATGGGAGGGGACACAGTCTACA  60
              ||||||||||||||||||||||||||||||||||||||||||||||||||||||||||||
Sbjct  28149  GTGAGAAGTCATACATGCCTGGCAGTGTTCGTTTTCGGATGGGAGGGGACACAGTCTACA  28090

Query  61     CTCACCACACGCCTGTCAAGGAAGGCAGGCAGGTGGAGTATGGTGGTTCATCGTACGAGC  120
              ||||||||||||||||||||||||||||||||||||||||||||||||||||||||||||
Sbjct  28089  CTCACCACACGCCTGTCAAGGAAGGCAGGCAGGTGGAGTATGGTGGTTCATCGTACGAGC  28030

Query  121    CATTGGACTTGACGTCTCCACATAACCCTTTGGTTGAGATTCCGTTCCCAAATCTCAAAG  180
              ||||||||||||||||||||||||||||||||||||||||||||||||||||||||||||
Sbjct  28029  CATTGGACTTGACGTCTCCACATAACCCTTTGGTTGAGATTCCGTTCCCAAATCTCAAAG  27970

Query  181    GAGTGGCATGGCCGTACGGGCATGCATTAGCAAAAACTCTGGAAGAACTTGACATTGATA  240
              ||||||||||||||||||||||||||||||||||||||||||||||||||||||||||||
Sbjct  27969  GAGTGGCATGGCCGTACGGGCATGCATTAGCAAAAACTCTGGAAGAACTTGACATTGATA  27910

Query  241    TTGCAGAAGGACTCCTGAAAGTTGGACTTAATCCTGAAGAGAACTACGAAATAACGACCA  300
              ||||||||||||||||||||||||||||||||||||||||||||||||||||||||||||
Sbjct  27909  TTGCAGAAGGACTCCTGAAAGTTGGACTTAATCCTGAAGAGAACTACGAAATAACGACCA  27850

Query  301    CCATCAAGGACGGTGCAGATGGAATGGGGGACATCTCTGTATTCAGGGAGGCTAGAGACC  360
              ||||||||||||||||||||||||||||||||||||||||||||||||||||||||||||
Sbjct  27849  CCATCAAGGACGGTGCAGATGGAATGGGGGACATCTCTGTATTCAGGGAGGCTAGAGACC  27790

Query  361    GCCTTCTCCCTGACAAGGCCTTCAGGGTTGCATTCGCGGTCATCAAGTGTGAGGTTACCC  420
              ||||||||||||||||||||||||||||||||||||||||||||||||||||||||||||
Sbjct  27789  GCCTTCTCCCTGACAAGGCCTTCAGGGTTGCATTCGCGGTCATCAAGTGTGAGGTTACCC  27730

Query  421    TTGGGGACGAGAGGTTGACCGTTTATGAACCGGATAAGCCTGAATCCACATTTATCACCC  480
              ||||||||||||||||||||||||||||||||||||||||||||||||||||||||||||
Sbjct  27729  TTGGGGACGAGAGGTTGACCGTTTATGAACCGGATAAGCCTGAATCCACATTTATCACCC  27670

Query  481    GTCCTCTGGTGGAGGCAATTGCAGACGAGAACAATCGCGCTTCATCGATTCTTCTCCTGG  540
              ||||||||||||||||||||||||||||||||||||||||||||||||||||||||||||
Sbjct  27669  GTCCTCTGGTGGAGGCAATTGCAGACGAGAACAATCGCGCTTCATCGATTCTTCTCCTGG  27610

Query  541    AAGGGATAGAGTGCGAGAGGGCTGCACTACGAAAGAGTGGACTTTCAGTCAACATTGGAA  600
              ||||||||||||||||||||||||||||||||||||||||||||||||||||||||||||
Sbjct  27609  AAGGGATAGAGTGCGAGAGGGCTGCACTACGAAAGAGTGGACTTTCAGTCAACATTGGAA  27550

Query  601    ATTCATGGAGGAGGCACAATCTTCGGTTTTACAATTCCATGATTGATGAGAAGCTTGACA  660
              ||||||||||||||||||||||||||||||||||||||||||||||||||||||||||||
Sbjct  27549  ATTCATGGAGGAGGCACAATCTTCGGTTTTACAATTCCATGATTGATGAGAAGCTTGACA  27490

Query  661    GGGCAGATGGTGGCCTCCAGGGAAGTGGTTCGAAATTCCTATGTACTTTGTGCCATGCTA  720
              ||||||||||||||||||||||||||||||||||||||||||||||||||||||||||||
Sbjct  27489  GGGCAGATGGTGGCCTCCAGGGAAGTGGTTCGAAATTCCTATGTACTTTGTGCCATGCTA  27430

Query  721    CCCGAGAGACGGCTAAGTCGGAACTAGGCAGTTTCAGGATCACCAGAACATACGAGGAGA  780
              ||||||||||||||||||||||||||||||||||||||||||||||||||||||||||||
Sbjct  27429  CCCGAGAGACGGCTAAGTCGGAACTAGGCAGTTTCAGGATCACCAGAACATACGAGGAGA  27370

Query  781    CGGATGCCATAGCAAACTACATACTCATCAACCCTGATGACCTGTCTCCAGCCCAACTTG  840
              ||||||||||||||||||||||||||||||||||||||||||||||||||||||||||||
Sbjct  27369  CGGATGCCATAGCAAACTACATACTCATCAACCCTGATGACCTGTCTCCAGCCCAACTTG  27310
```

```
Query  841    GCGAGATGTCACAAGGTGTGAAATCTCGTCCAGTCCTGCAATCAGATCCGCGAGAGAAGT  900
              ||||||||||||||||||||||||||||||||||||||||||||||||||||||||||||
Sbjct  27309  GCGAGATGTCACAAGGTGTGAAATCTCGTCCAGTCCTGCAATCAGATCCGCGAGAGAAGT  27250

Query  901    TGATAGATGCAACCCACGCGGACATAAACATTGGCTCGTTTTTTAAGAAGCTGATCATAT  960
              ||||||||||||||||||||||||||||||||||||||||||||||||||||||||||||
Sbjct  27249  TGATAGATGCAACCCACGCGGACATAAACATTGGCTCGTTTTTTAAGAAGCTGATCATAT  27190

Query  961    GTGAGACCGCCAGAATCCAGACATGGGAAATTCGACAAGATGTGAAGGAGAGCTACGAGA  1020
              ||||||||||||||||||||||||||||||||||||||||||||||||||||||||||||
Sbjct  27189  GTGAGACCGCCAGAATCCAGACATGGGAAATTCGACAAGATGTGAAGGAGAGCTACGAGA  27130

Query  1021   AAGCAGAGAAAAAGTTTGATGATCATTTGTTGTCTACACTTGGACTAGGACCCTCCTTGA  1080
              ||||||||||||||||||||||||||||||||||||||||||||||||||||||||||||
Sbjct  27129  AAGCAGAGAAAAAGTTTGATGATCATTTGTTGTCTACACTTGGACTAGGACCCTCCTTGA  27070

Query  1081   TGATGCCTGGTAATTTCGCGCGGGCATTGTTCGCCTTGAAAAACAATGACATTGTCTTGC  1140
              ||||||||||||||||||||||||||||||||||||||||||||||||||||||||||||
Sbjct  27069  TGATGCCTGGTAATTTCGCGCGGGCATTGTTCGCCTTGAAAAACAATGACATTGTCTTGC  27010

Query  1141   AGCTAGTCAAAGATGAGGAAAGACGTGAAAGGCTGAAAGAAGTGTTGGTCCTGTTCTCTC  1200
              ||||||||||||||||||||||||||||||||||||||||||||||||||||||||||||
Sbjct  27009  AGCTAGTCAAAGATGAGGAAAGACGTGAAAGGCTGAAAGAAGTGTTGGTCCTGTTCTCTC  26950

Query  1201   GCCTCCGGTCCGTATACAGAGCACACCACCCCAAGGTTGACGAGGTGAAGGTCTACAAGG  1260
              ||||||||||||||||||||||||||||||||||||||||||||||||||||||||||||
Sbjct  26949  GCCTCCGGTCCGTATACAGAGCACACCACCCCAAGGTTGACGAGGTGAAGGTCTACAAGG  26890

Query  1261   TAAATGCAGTCTCCATGGGGAACCTGTTGAAGACTCACTTTGCATATGTCTCCTGGCCTA  1320
              ||||||||||||||||||||||||||||||||||||||||||||||||||||||||||||
Sbjct  26889  TAAATGCAGTCTCCATGGGGAACCTGTTGAAGACTCACTTTGCATATGTCTCCTGGCCTA  26830

Query  1321   ATTATCTCCACAAAGTCATAGAACATGTTCAGGAGATCCTTGAGGATCCAAGCGGACCAC  1380
              ||||||||||||||||||||||||||||||||||||||||||||||||||||||||||||
Sbjct  26829  ATTATCTCCACAAAGTCATAGAACATGTTCAGGAGATCCTTGAGGATCCAAGCGGACCAC  26770

Query  1381   AGACAGTTGGAGGGTTGAGTGGAGAAGGAAATGAAGCCTCCAACAAGCTCTTTCGCGATC  1440
              ||||||||||||||||||||||||||||||||||||||||||||||||||||||||||||
Sbjct  26769  AGACAGTTGGAGGGTTGAGTGGAGAAGGAAATGAAGCCTCCAACAAGCTCTTTCGCGATC  26710

Query  1441   TCCGTCGGCATTATGCTAGGAAGCATGATGTGACACAGAATCTCCGTGACATCCTGTGGG  1500
              ||||||||||||||||||||||||||||||||||||||||||||||||||||||||||||
Sbjct  26709  TCCGTCGGCATTATGCTAGGAAGCATGATGTGACACAGAATCTCCGTGACATCCTGTGGG  26650

Query  1501   TACACTGGCTCTACACCAGTCCTAAAATTAGAAGATTAAGCAGCAAAACTACTCGAAGTT  1560
              ||||||||||||||||||||||||||||||||||||||||||||||||||||||||||||
Sbjct  26649  TACACTGGCTCTACACCAGTCCTAAAATTAGAAGATTAAGCAGCAAAACTACTCGAAGTT  26590

Query  1561   ACCATTGCTCAAAATGTGACGGACTTTGGCACAACGCAAGGAGTTGCCCTGGCCCTTCTC  1620
              ||||||||||||||||||||||||||||||||||||||||||||||||||||||||||||
Sbjct  26589  ACCATTGCTCAAAATGTGACGGACTTTGGCACAACGCAAGGAGTTGCCCTGGCCCTTCTC  26530

Query  1621   CTTCTAAGGATGCTTGAAATGTTCATTGACCAAtttttttCTCCATAACTTCATGGTAAC  1680
              ||||||||||||||||||||||||||||||||||||||||||||||||||||||||||||
Sbjct  26529  CTTCTAAGGATGCTTGAAATGTTCATTGACCAATTTTTTTCTCCATAACTTCATGGTAAC  26470

Query  1681   TTATAACAGATTTGTCAATACACAATATTATTTGTACTAGATGTCTACCAATGTTATTAT  1740
              ||||||||||||||||||||||||||||||||||||||||||||||||||||||||||||
Sbjct  26469  TTATAACAGATTTGTCAATACACAATATTATTTGTACTAGATGTCTACCAATGTTATTAT  26410

Query  1741   CCTTACAGGCAGTGTTCTCATTAGAAGTAAGCAATACAGCACAAATATATTTTTTCTTCT  1800
              ||||||||||||||||||||||||||||||||||||||||||||||||||||||||||||
Sbjct  26409  CCTTACAGGCAGTGTTCTCATTAGAAGTAAGCAATACAGCACAAATATATTTTTTCTTCT  26350

Query  1801   TCAAAAGAATTCTACATGTATCAATGTACATGCAATAATGTATTGAACTCGGGTTTACTA  1860
              ||||||||||||||||||||||||||||||||||||||||||||||||||||||||||||
Sbjct  26349  TCAAAAGAATTCTACATGTATCAATGTACATGCAATAATGTATTGAACTCGGGTTTACTA  26290

Query  1861   AATTAATACTCTCAACGCAAGATTTTTTAGTTTTGTTTCAGGCAGTTTTATTTACATTAC  1920
              ||||||||||||||||||||||||||||||||||||||||||||||||||||||||||||
Sbjct  26289  AATTAATACTCTCAACGCAAGATTTTTTAGTTTTGTTTCAGGCAGTTTTATTTACATTAC  26230

Query  1921   AAGAAAAATAAGGGCTG  1937
              |||||||||||||||||
Sbjct  26229  AAGAAAAATAAGGGCTG  26213
```

RAG2L

>TSA: IACU01028708.1:beg-end

5'3' Frame 1

NSRTGIIMFKDAVYDLCVAGGTLPPLHGAAVVDIGQRQIIWGGLDLSEYRCTDDLMILQELTLPRKGAPIFQITYIQTLKHQVPSRLGKDIPHQTGPVPSERSGHTLTMLPGTNKALLFGGLSMGTHKGYYNNSRFCQTCKDGRFYILDTDTYEWQHIKVPLIQPRAYHSVTVMEKDNEFVAALIGGVVYEETAPTHREALNEIVVLTIDKEIQNFSLK
EVSLQPSMPTTHNVFLSSHATTVHNNVIIVAGGV

Query: **TSA: IACU01023786.1:beg-end**
Subject **BEXV01003119.1** **Length: 48415** **Number of Matches: 3**

Score	Expect	Identities	Gaps	Strand
854 bits(462)	0.0	468/471(99%)	0/471(0%)	Plus/Plus

```
Query  291    TGTTCCTTCAGAAAGAAGTGGCCATACTTTGACAATGCTACCCGGTACCAACAAAGCTCT  350
              ||||| | ||||||||||||||||||||||||||||||||||||||||||||||||||||
Sbjct  24495  TGTTCTTACAGAAAGAAGTGGCCATACTTTGACAATGCTACCCGGTACCAACAAAGCTCT  24554

Query  351    TCTATTTGGTGGCTTATCTATGGGAACACACAAAGGGTACTACAACAATTCACGATTTTG  410
              ||||||||||||||||||||||||||||||||||||||||||||||||||||||||||||
Sbjct  24555  TCTATTTGGTGGCTTATCTATGGGAACACACAAAGGGTACTACAACAATTCACGATTTTG  24614

Query  411    TCAAACTTGTAAGGATGGCAGATTTTACATTTTGGACACAGACACGTACGAGTGGCAACA  470
              ||||||||||||||||||||||||||||||||||||||||||||||||||||||||||||
Sbjct  24615  TCAAACTTGTAAGGATGGCAGATTTTACATTTTGGACACAGACACGTACGAGTGGCAACA  24674

Query  471    CATCAAGGTGCCCTTGATTCAGCCAAGGGCATACCATTCCGTGACAGTAATGGAGAAGGA  530
              ||||||||||||||||||||||||||||||||||||||||||||||||||||||||||||
Sbjct  24675  CATCAAGGTGCCCTTGATTCAGCCAAGGGCATACCATTCCGTGACAGTAATGGAGAAGGA  24734

Query  531    CAATGAGTTTGTCGCAGCTTTGATTGGAGGAGTTGTGTATGAAGAGACTGCGCCAACCCA  590
              |||||||||||||||||||||||||||||||||||| |||||||||||||||||||||||
Sbjct  24735  CAATGAGTTTGTCGCAGCTTTGATTGGAGGAGTTGTATATGAAGAGACTGCGCCAACCCA  24794

Query  591    TCGTGAGGCCCTTAATGAAATTGTCGTCTTAACAATAGATAAGGAAATACAAAACTTCAG  650
              ||||||||||||||||||||||||||||||||||||||||||||||||||||||||||||
Sbjct  24795  TCGTGAGGCCCTTAATGAAATTGTCGTCTTAACAATAGATAAGGAAATACAAAACTTCAG  24854

Query  651    TCTGAAAGAAGTTTCACTTCAACCAAGCATGCCAACTACACACAATGTTTTCTTGTCTTC  710
              ||||||||||||||||||||||||||||||||||||||||||||||||||||||||||||
Sbjct  24855  TCTGAAAGAAGTTTCACTTCAACCAAGCATGCCAACTACACACAATGTTTTCTTGTCTTC  24914

Query  711    TCATGCAACCACAGTGCACAACAATGTCATTATTGTTGCAGGTGGAGTTCA  761
              |||||||||||||||||||||||||||||||||||||||||||||||||||
Sbjct  24915  TCATGCAACCACAGTGCACAACAATGTCATTATTGTTGCAGGTGGAGTTCA  24965
```
