# Supplementary material for: Identification of RAG-like transposons in protostomes suggests their ancient bilaterian origin
Source: Mob DNA. 2020 May 6;11:17. doi: 10.1186/s13100-020-00214-y (PMC7204232; doi:10.1186/s13100-020-00214-y)
Supplement: Supplementary file 2 — Additional file 2: Figure S2. (a) Alignments of four groups of protostome RAGL transposon TIRs. Sequences of 5′-TIR and 3′-TIR (reverse complement) pairs are aligned from four groups: C. virginica (I), P. imbricata (II and III) and nemertean N. geniculatus (IV). Despite both being mollusks, the C. virginica and P. imbricata TIR sequences are very dissimilar both in sequence and length. None of the 4 groups contain an RSS nonamer-like region, however, the C. virginica 5′ and 3′ TIRs exhibit a length difference of 11 bp, reminiscent of the 11 bp difference in lengths of the spacers in the 12RSS and 23RSS that underlies the 12/23 rule of V(D) J recombination [3, 6]. Similarity color code: dark grey, fully conserved; light grey, partially conserved; white, no similarity. (b) Potential RAG-derived non-autonomous transposable elements in protostomes and deuterostomes. Summary of the potential RAG-derived non-autonomous elements identified in WGS database in organisms where the RAGL transposon was detected. The configuration of these elements, as well as TSD sequences (if present) are indicated. The e-values shown derive from blastn searches of the WGS project of each organism using previously detected RAGL TIR-containing margins (200 bp) as queries. [file 13100_2020_214_MOESM2_ESM.pdf]

a TIR groups

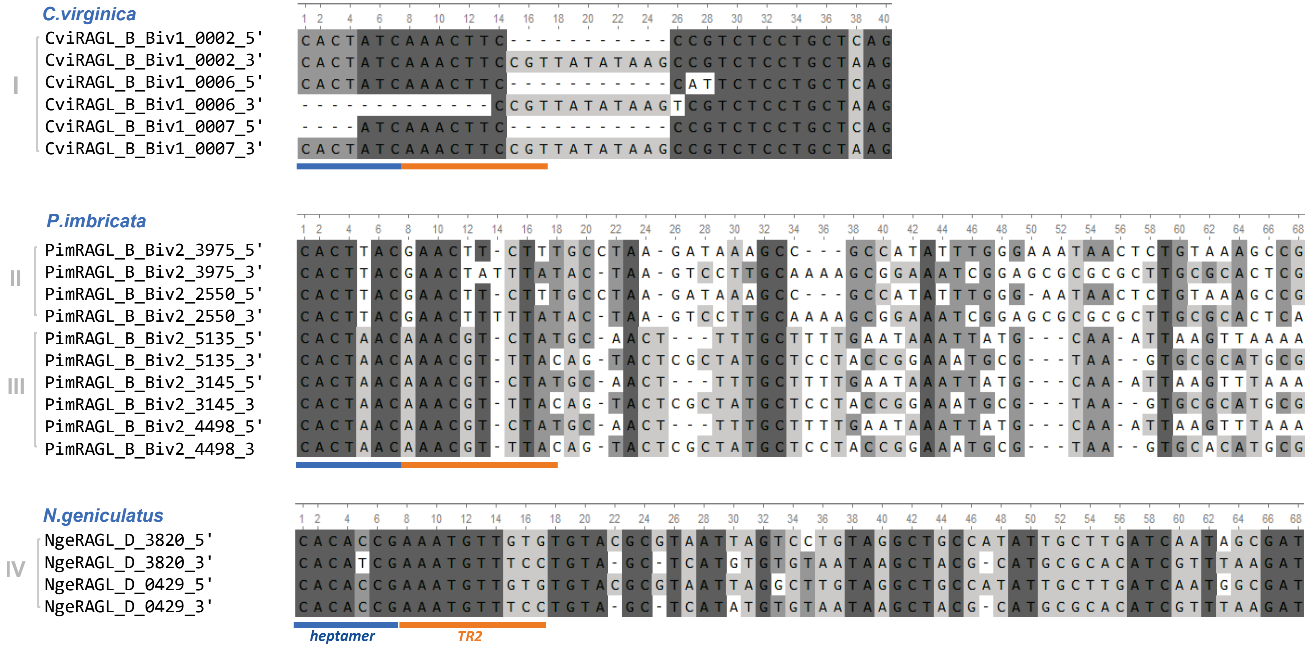

b Non-autonomous RAGL

Organism Group	Organism	WGS	Configuration	size(bp)	strand	start	end	TSD TIR-5'	TSD TIR-3'	e-val TIR5	e-val TIR3
Mollusca	C.viriginica	not found									
	C.gigas	not found									
	S.glomerata	not found									
	M.philippinarum	not found									
	B.platifrons	not found									
	P.imbricata	NIJJ01042937.1	TSD - TIR5 - TIR3 - TSD	1696	-	3359	1663	TACAG	TACAG	7.00E-59	5.00E-23
		NIJJ01019001.1	TSD - TIR5 - TIR3 - TSD	2169	-	7347	5178	CGACT	CGACG	5.00E-48	1.00E-18
		NIJJ01054255.1	TSD - TIR5 - TIR3 - TSD	1498	-	2915	1417	ACGCG	ACGCG	2.00E-59	6.00E-22
		NIJJ01018727.1	TIR5 - TIR3	2644	+	4723	7367	CACCT	CAGTT	2.00E-53	9.00E-20
		NIJJ01022200.1	TIR5 - TIR3	1684	-	1910	226	-	-	8.00E-52	3.00E-26
		NIJJ01068733.1	incTIR5-TIR3	2085	+	569	2654	-	-	3.00E-38	3.00E-26
Nemertea	N.geniculatus	not found									
Cephalochordata	B.belcheri	FQTN01000713.1	TSD - TIR5 - TIR3 - TSD	709	+	130294	131003	TAGGG	TAGGG	1.00E-87	1.00E-106
	B.lanceolatum	FLLO01000007.1	TSD - TIR5 - TIR3 - TSD	2246	-	2451556	2449310	CTACA	CTACA	1.00E-35	8.00E-51
		FLLO01000013.1	TSD - TIR5 - TIR3 - TSD	2198	+	490675	492873	TACCG	TACCG	8.00E-32	3.00E-50
		FLLO01000276.1	TSD - TIR5 - TIR3 - TSD	2192	+	63032	65224	CGACA	CGACA	6.00E-33	3.00E-49
		FLLO01000222.1	TSD - TIR5 - TIR3 - TSD	2209	+	381571	383780	CACAG	CACAG	2.00E-32	3.00E-49
		FLLO01000011.1	TSD - TIR5 - TIR3 - TSD	1813	+	4114656	4116469	CGGCG	CGGCG	2.00E-34	3.00E-49
		FLLO01000025.1	TSD - TIR5 - TIR3 - TSD	2223	-	992417	990194	CGACG	CAACG	2.00E-33	8.00E-51
		FLLO01010018.1	TSD - TIR5 - TIR3 - TSD	2354	-	3032	678	GTCCG	GTCCG	6.00E-33	1.00E-47
		FLLO01002344.1	TIR5 - TIR3	2191	+	680	2871	TGCCG	NNNNN	3.00E-30	7.00E-45
		FLLO01000463.1	TSD - TIR5 - TIR3 - TSD	2182	+	121209	123391	CAGGG	CAGGG	4.00E-35	1.00E-42
		FLLO01000338.1	incTIR5 - incTIR3	2047	+	70351	72398	-	-	1.00E-28	2.00E-38
	B.floridae	ABEP02041477.1	TSD - TIR5 - TIR3 - TSD	1904	-	4815	2911	AGATG	AGATG	1.00E-17	5.00E-47
		ABEP02015499.1	TSD - TIR5 - TIR3 - TSD	1919	-	16917	14998	CGACG	CGACG	1.00E-17	3.00E-49
		ABEP02002901.1	incTIR5 - incTIR3	1750	+	583	2333	-	-	2.00E-15	6.00E-40
Echinodermata	P.miniata	not found									
Hemichordata	P.fava	not found									
